# A global mapping of research on the relationship between oral health and nutritional status

**DOI:** 10.1038/s41405-026-00469-2

**Published:** 2026-07-23

**Authors:** Yuh-Shan Ho, Anastasios Grigoriadis, Abhishek Kumar, Nikolaos Christidis

**Affiliations:** 1 CT HO Trend, Songshan Dist., Taipei City, Taiwan; 2 Division of Oral Rehabilitation, Department of Dental Medicine, Karolinska Institutet, Huddinge, Sweden; 3 Academic Center for Geriatric Dentistry, Stockholm, Sweden; 4 Department of Conservative Dentistry and Endodontics, Dr. D. Y. Patil Dental College & Hospital, Dr. D. Y. Patil Vidyapeeth (Deemed to be University), Pune, India

**Keywords:** Nutrition and diet in dentistry, Dentistry

## Abstract

**Background/Objective:**

Oral health and nutritional status are closely interlinked through biological, functional, and behavioural pathways, yet the global research landscape connecting these domains has not previously been systematically mapped. This study aimed to provide a comprehensive bibliometric assessment of worldwide research linking oral health and nutritional status, identify influential publications, and analyze thematic developments over time.

**Materials and methods:**

Data were retrieved from the Science Citation Index Expanded (SCI-EXPANDED) within the Web of Science Core Collection. Publications from 1991–2024 were identified using a structured search strategy incorporating primary and extended terminology. Document characteristics, citation performance, authorship patterns, and publication indicators (*TP*, *IP*, *CP*, *FP*, *RP*, and *SP*) were analyzed alongside citation indicators (*C*_year_, *TC*_year_, *CPP*_year_). Author keywords were examined across three balanced time periods to identify major research foci.

**Results:**

A total of 10,074 publications were identified, including 8747 articles. Global research activity increased steadily, with citation maturity typically reached after 13 years. The USA, UK, Japan, and China led in publication output, while international collaboration was associated with higher citation impact. The ten most-cited articles reflected epidemiological surveillance, methodological standardization, global oral disease burden, and clinical nutrition guidelines. Keyword analysis revealed five overarching subjects: epidemiology, nutrition-related determinants of oral function and disease, clinical and quality-of-life outcomes, intervention strategies, and vulnerable populations.

**Conclusions:**

This study provides the first global bibliometric overview of research linking oral health and nutritional status. Findings highlight an expanding, interdisciplinary field with growing methodological sophistication and public health relevance.

## Introduction

Oral diseases are some of the most widespread health problems, affecting more than 3.5 billion people globally [1]. Oral diseases can cause pain and infection that leads to tooth loss and, in severe cases, oral cancers, resulting in functional difficulties, including chewing and swallowing problems [2]. All of these factors can greatly reduce a person’s quality of life. Further, oral health is strongly associated with some of the major non-communicable diseases, such as diabetes, cardiovascular conditions [3], cognitive decline [4, 5], and malnutrition [6]. Therefore, improving or maintaining good oral health is essential because it preserves daily function, protects overall wellbeing, and reduces the risk of serious disease.

In recent years, research on oral infections as a source of systemic diseases has also received much attention. Specifically, there is growing evidence to suggest that oral health is not only associated with general health-related measurements but may also have a potential causative role [3]. For example, studies have highlighted that chronic, low-grade inflammation resulting from periodontal disease may exacerbate systemic inflammation, thereby contributing to the development of atherosclerosis and increasing the risk of cardiovascular disease [7, 8]. Further, several large-scale and longitudinal studies have shown that tooth loss and reduced masticatory (chewing) function are linked to lower cognitive scores and a higher risk of cognitive impairment or dementia [9–11]. The ability to eat moderately hard foods is shown to be associated with the ability to perform day-to-day cognitive and intellectual activities [5]. Moreover, it has been suggested that mastication plays a fundamental mechanical and physiological role in preparing food for swallowing and supporting the function of the gastrointestinal tract [6]. By breaking down food into smaller, manageable particles and mixing it with saliva, chewing makes the swallowing process safer and more efficient [6]. It also stimulates digestive activity in the stomach and intestines, helping the body process nutrients more effectively.

Dental status and efficient chewing are closely linked to nutritional status because they influence food choices, meal enjoyment, and overall dietary intake [12]. When chewing ability is reduced, individuals may prefer softer, less nutritious foods, which can negatively affect their general health [12, 13]. This shift in dietary behaviour illustrates how impaired oral function can cascade into broader physiological consequences, underscoring the tightly interwoven relationship between oral and systemic health. Together, these findings emphasize the complex and potentially “bidirectional” interactions between oral health and general health.

As evidence linking chewing, swallowing, gastrointestinal function, and nutritional outcomes continues to grow, it is increasingly drawn from diverse disciplines such as dentistry, neurology, nutrition, and gastroenterology. This has created a research landscape that is both extensive and fragmented, making it difficult to obtain a comprehensive overview of how knowledge in this area has evolved. Consequently, an important knowledge gap remains regarding the overall structure, development, and thematic evolution of research linking oral health and nutritional status. A comprehensive bibliometric analysis is therefore warranted. The scientific output in this field has expanded rapidly in both volume and complexity, and a structured analysis can help clarify its evolution. By mapping publication trends, identifying influential authors and institutions, and highlighting dominant research themes, a bibliometric approach provides a coherent overview of the field. It also exposes gaps where further investigation is needed, offering a strong foundation for future studies and guiding researchers toward the most meaningful and impactful directions. Previous bibliometric studies in this field have generally been based on smaller datasets or more restricted publication subsets and have primarily focused on descriptive publication trends [14, 15]. In contrast, the present study provides, to our knowledge, the first large-scale global bibliometric mapping of research linking oral health and nutritional status across more than 10,000 publications. By integrating multiple publication and citation indicators with longitudinal thematic mapping, the study further proposes a structured conceptual framework comprising five overarching research domains linking oral health, nutrition, systemic disease, and public health perspectives.

Therefore, the aim of the present study was to comprehensively map the global research landscape linking oral health and nutritional status using established bibliometric methods. Specifically, we assessed publication and citation performance, identified influential publications, and examined thematic research trends over time. The study design was informed by established bibliometric and science-mapping approaches [16, 17].

## Materials and methods

### Data source and search strategy

The data for this study were obtained from the Web of Science Core Collection (WoSCC), the online version of the Science Citation Index Expanded (SCI-EXPANDED), with updates as of 21 November 2025. The SCI-EXPANDED indexes 9440 journals across 178 Web of Science categories. A structured search strategy using predefined oral health- and nutrition-related terms was applied to retrieve relevant publications. Quotation marks and Boolean operators (“OR”, “AND”) were used to identify records containing at least one search term in the title, abstract, or author keywords [18].

This bibliometric study was conducted in accordance with the BIBLIO checklist (Supplementary material 1), which outlines reporting recommendations for bibliometric studies in the biomedical literature [19]. Established bibliometric approaches were applied to support transparency and reproducibility.

### Search term design and keyword expansion

To improve retrieval sensitivity, both primary and extended terminology related to oral health and nutritional status were included in the search strategy. The complete search strategy is presented in Supplementary material 2.

### Document types and article selection

Original research articles were selected for detailed bibliometric analyses. Because Web of Science document types are not mutually exclusive, cumulative percentages may exceed 100%.

**Fig. 1:**
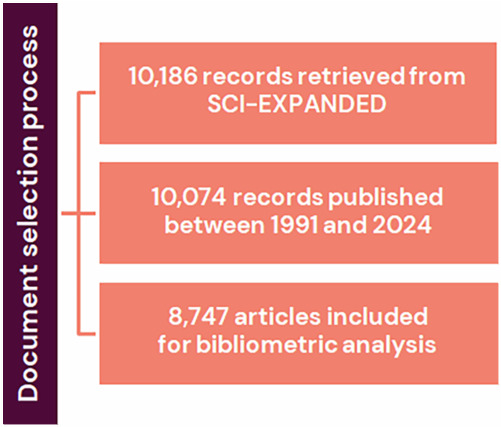
Document retrieval and study selection process. Flowchart of document retrieval and article selection process for the bibliometric analysis of oral health and nutritional status research.

Complete records, including annual citation counts, were exported to Microsoft 365 Excel for further analysis as previously described [20]. The final cleaned dataset is presented as Table S3. Journal impact factors (*IF*_2024_) were sourced from the 2024 Journal Citation Reports (JCR) edition. The document selection process is shown in Fig. 1.

### Data extraction

Complete records and annual citation counts from SCI-EXPANDED were downloaded into Microsoft Excel 365 and manually examined [20]. Bibliometric data were cleaned, standardized, and analyzed in Microsoft Excel 365 using established bibliometric procedures and manual verification methods.

A total of 10,186 documents were retrieved in the SCI-EXPANDED, including 10,074 documents (99% of the 10,186 documents) published between 1991 and 2024 and 112 documents published in 2025. Data standardization procedures were applied to improve consistency and reduce indexing-related inconsistencies.

### Authorship and affiliation identification

In WoSCC, the “reprint author” corresponds to the author responsible for correspondence. For clarity, the term “corresponding author” is used here [21]. The first and corresponding authors are typically considered the primary contributors to research output [22]. At the institutional level, the corresponding-author’s affiliation is typically used to denote the primary institutional origin or primary institutional affiliation of the research [23]. For documents with a single author, institution, or country, that entity was treated as both the first and corresponding author or affiliation [24]. Cases with multiple corresponding authors included all listed contributors. Affiliation names and geographic classifications were manually standardized to improve consistency across institutions and countries.

### Citation indicators and publication indicators for countries and institutions

The *CPP*_2024_ metric, representing the average number of citations per publication in a specific year, was analyzed alongside annual publication counts (*TP*). These indicators were used to evaluate temporal citation patterns and publication activity across the study period. Citation-based bibliometric indicators commonly used in research performance analyses were applied, including *C*_year_, *TC*_year_, and *CPP*_year_ [16, 23]. *TC*_year_ reflects the total number of citations received from the publication year until the end of the most recent year (2024 in this study; denoted as *TC*_2024_). Because citation counts in the WoSCC are continuously updated, *TC*_year_ was adopted as the primary citation measure to minimize temporal bias and provide a more stable and comprehensive assessment of impact, consistent with the recommendations. *CPP*_year_ shows the average number of citations per publication and is calculated as *CPP*_2024_ = *TC*_2024_/*TP*, where *TP* denotes the total number of publications [25].

Citation metrics can be applied across multiple dimensions, including total and annual publication output, as well as distributions by document type, language, Web of Science category, journal, country, institution, and individual articles. To provide a more comprehensive assessment of research performance, six publication indicators were proposed in 2014 to evaluate the productivity of countries and institutions. These indicators comprise total publications (*TP*), single-country or single-institution publications (*IP*; *IP*_C_ or *IP*_*I*_), internationally or inter-institutionally collaborative publications (*CP*; *CP*_C_ or *CP*_*I*_), first-author publications (*FP*), corresponding-author publications (*RP*), and single-author publications (*SP*). For cross-country comparisons, these six publication indicators were applied alongside their corresponding citation indicators (*CPP*_2024_) to assess research impact. Additionally, the six *CPP*_2024_ citation indicators aligned with these publication metrics were used to evaluate the influence of publications across document types, journals, countries, and institutions. Moreover, six citation indicators (*CPP*_2024_) corresponding to these publication indicators were used to evaluate the impact of publications on document types, journals, countries, and institutions.

To examine document type characteristics within a specific research field, two fundamental metrics have been proposed: the average number of citations per publication (*CPP*_year_) and the average number of authors per publication (*APP*) [26]. The analyses were based exclusively on standardized bibliographic metadata and bibliometric indicators available for all publications indexed in the Web of Science Core Collection, thereby ensuring consistent comparisons across the dataset.

## Results

### Characteristics of document types and languages

From 1991 to 2024, a total of 10,074 publications related to oral health and nutritional status were indexed in the SCI-EXPANDED database, encompassing 14 distinct document types (Table 1). Among these, 8747 were categorized as articles, representing 87% of all publications, with an average of 5.9 authors per publication.

**Table 1:** Citations and authors according to the document type.

Document type	*TP*	%	TP*	*AU*	*APP*	*TC*_2024_	*CPP*_2024_
Article	8747	87	8742	51,797	5.9	236,374	27
Review	1059	11	1059	4982	4.7	58,507	55
Proceedings paper	278	2.8	278	1292	4.6	15,862	57
Meeting abstract	147	1.5	146	678	4.6	23	0.16
Editorial material	79	0.78	78	290	3.7	1,262	16
Early access	28	0.28	28	160	5.7	169	6.0
Letter	21	0.21	21	83	4.0	95	4.5
Book chapter	13	0.13	13	42	3.2	875	67
Note	11	0.11	10	31	3.1	253	23
Correction	6	0.060	6	30	5.0	2	0.33
News item	3	0.030	2	2	1.0	0	0
Retracted publication	2	0.020	2	20	10	28	14
Data paper	1	0.010	1	9	9.0	3	3.0
Reprint	1	0.010	1	2	2.0	2	2.0

*TP* total number of publications; TP* total number of publications with author information in the SCI-EXPANDED database, % percentage of articles in all articles, *AU* total number of authors, *APP* average number of authors per publication, *TC*_2024_ total number of citations from WoSCC since publication year until the end of 2024, *CPP*_2024_ average number of citations per publication (*TC*_2024_/*TP*).

“Book chapters” (13 documents) demonstrated the highest *CPP*_2024_, averaging 67 citations per publication (totalling 875 citations). Review articles exhibited a *CPP*_2024_ that was 2.0 times higher than that of research articles.

Among the 10,074 publications addressing oral health in relation to nutritional status, seven were identified as classic works, each having accrued 1000 or more citations (*TC*_2024_ ≥ 1000) from the Web of Science Core Collection [27]. These classics comprised four original articles and three review papers. The most frequently cited publication was the review “Epidemiology and causes of preterm birth” [28]. It achieved a *TC*_2024_ of 5399 citations and a *C*_2024_ of 406 citations, making it the most influential publication in 2024. The second classic review, “Fluoride in drinking water: A review on the status and stress effects” [29], accumulated a *TC*_2024_ of 1,060 citations.

In oral health and nutritional status research, 10,074 articles were published in 15 languages. English was the dominant language, accounting for 8,454 articles (97% of all articles), followed by Spanish (103 articles), German (73), Portuguese (45), and French (41). Other publication languages included Russian (6 articles), Hungarian (5), Korean (5), Turkish (4), Polish (3), Czech (2), Japanese (2), and one article each in Chinese, Esperanto, and Italian. Additionally, one article published in *Chirurgia* had an unspecified language.

Non-English publications had lower citation impact (*CPP*_2024_ = 6.7) and lower authorship counts (*APP* = 4.7) than English-language publications (*CPP*_2024_ = 28; *APP* = 6.0). All 411 highly cited articles (*TC*_2024_ ≥ 100) were published in English.

### Characteristics of publication outputs

**Fig. 2:**
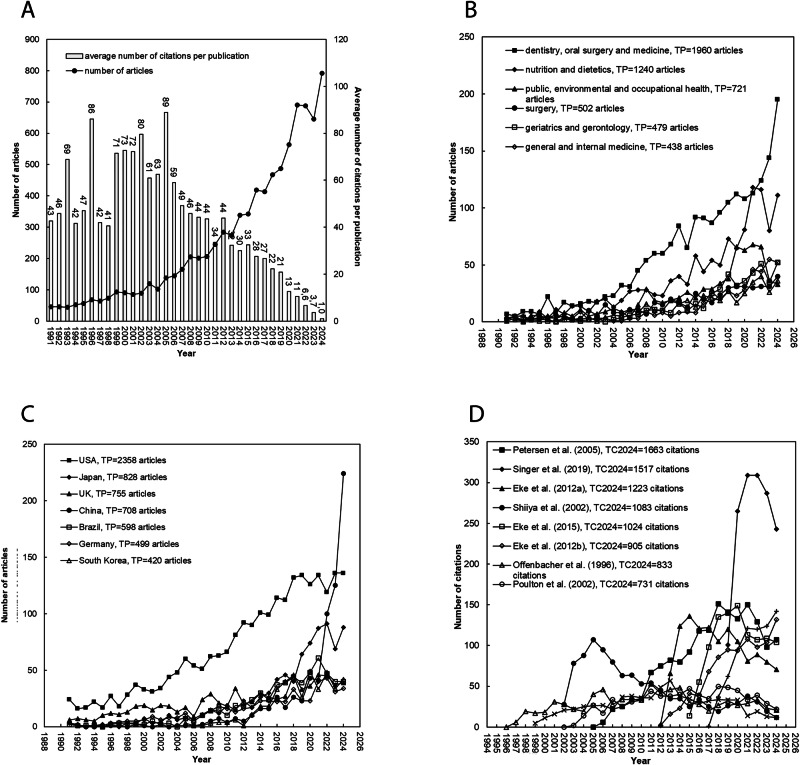
Bibliometric overview of research on oral health and nutritional status. (**A**) Annual number of publications and mean citations per article. (**B**) Development trends of the seven most productive Web of Science categories with at least 400 publications. (**C**) Development trends of the seven most productive countries with at least 400 publications. (**D**) Citation histories of the ten most frequently cited articles.

As shown in Fig. 2A, articles typically reached citation maturity around 13 years after publication. For example, 792 articles published in 2024 received a *TC*_2024_ of 764 citations, resulting in a *CPP*_2024_ of 1.0 citations per publication. In contrast, 138 articles published in 2004 accumulated a *TC*_2024_ of 12,270 citations, producing a *CPP*_2024_ of 89 citations per publication.

Publication output increased across three periods: 1991–2003, 2004–2014, and 2015–2024. Temporary stagnation and decline in publication output were observed during the COVID-19 pandemic period.

### Web of Science categories and journals in SCI-EXPANDED

From 1991 to 2024, oral health and nutritional status-related research was published in 1856 journals spanning 131 Web of Science categories within SCI-EXPANDED. The top 10 most productive categories are summarized in Table 2. A total of 3200 articles, representing 37% of all 8747 articles, were published within the two most productive categories. The category “dentistry, oral surgery and medicine” was the most productive, contributing 1960 articles (22%), followed by “nutrition and dietetics” with 1240 articles (14%).

**Table 2:** The top 10 most productive Web of Science categories.

Web of Science category	*TP* (%)	No. *J*	*APP*	*CPP*_2024_
Dentistry, oral surgery and medicine	1960 (22)	91	5.5	32
Nutrition and dietetics	1240 (14)	86	6.0	28
Public, environmental and occupational health	721 (8.2)	209	5.6	29
Surgery	502 (5.7)	213	6.9	23
Geriatrics and gerontology	479 (5.5)	56	6.3	26
General and internal medicine	438 (5.0)	162	6.2	18
Pediatrics	438 (5.0)	129	5.7	25
Otorhinolaryngology	301 (3.4)	43	6.1	30
Clinical neurology	276 (3.2)	212	7.4	29
Multidisciplinary sciences	265 (3.0)	73	6.7	19

*TP* total number of publications, *No. J* number of journals in a category in 2023, *APP* average number of authors per publication, *CPP*_2024_ average number of citations per publication (*TC*_2024_/*TP*).

Figure 2B illustrates the publication trends of the seven most productive Web of Science categories with more than 400 articles. The categories “dentistry, oral surgery and medicine” and “nutrition and dietetics” dominated the field throughout the study period.

Among the top 10 Web of Science categories listed in Table 2, “dentistry, oral surgery and medicine” recorded the highest *CPP*_2024_, with an average of 32 citations per publication. The “clinical neurology” category demonstrated the highest average number of authors per publication (*APP*), with 7.4 authors across 276 articles.

Table 3 presents the 10 most productive journals publishing oral health and nutritional status-related research, together with *IF*_2024_, *CPP*_2024_, and *APP* values. The *Journal of Periodontology* was the most productive journal, contributing 201 articles (2.3% of all articles), followed by *BMC Oral Health* and *Nutrients*. Among the journals listed in Table 3, the *Journal of Dental Research* demonstrated the highest *CPP*_2024_, with an average of 77 citations per publication. *Nutrients* showed the highest *APP* (7.4 authors per publication), whereas the *Journal of Public Health Dentistry* had the lowest *APP* (4.6 authors per publication). *The Lancet* (*IF*_2024_ = 88.5) and the *New England Journal of Medicine* (*IF*_2024_ = 78.5) represented the journals with the highest impact factors publishing articles related to oral health and nutritional status.

**Table 3:** The top 10 most productive journals.

Journal	*TP* (%)	*IF*_2024_	*APP*	*CPP*_2024_	Web of Science category
Journal of Periodontology	201 (2.3)	3.8	5.8	65	Dentistry, oral surgery and medicine
BMC Oral Health	183 (2.1)	3.1	6.1	12	Dentistry, oral surgery and medicine
Nutrients	181 (2.1)	5	7.4	12	Nutrition and dietetics
Journal of Clinical Periodontology	167 (1.9)	6.8	6.7	46	Dentistry, oral surgery and medicine
International Journal of Environmental Research and Public Health	135 (1.5)	^a^4.614	6.1	11	Environmental sciences Public, environmental and occupational health
PLoS One	119 (1.4)	2.6	7.2	20	Multidisciplinary sciences
Dysphagia	117 (1.3)	3	5.7	39	Otorhinolaryngology
Community Dentistry and Oral Epidemiology	115 (1.3)	2.1	4.9	45	Dentistry, oral surgery and medicine Public, environmental and occupational health
Journal of Dental Research	98 (1.1)	5.9	6.4	77	Dentistry, oral surgery and medicine
Journal of Public Health Dentistry	97 (1.1)	1.5	4.6	27	Dentistry, oral surgery and medicine Public, environmental and occupational health

*TP* total number of articles; %: percentage of articles in all articles, *IF*_2024_ journal impact factor in 2024. *APP* average number of authors per publication, *CPP*_2024_. average number of per publication (*TC*_2024_/*TP*).

^a^journal impact factor in 2021 (*IF*_2021_).

### Publication performances: countries and institutions

Within the SCI-EXPANDED database, 23 out of 8747 oral health and nutritional status-related articles (0.26%) lacked affiliation information. The remaining 8724 articles were authored by researchers affiliated with institutions in 139 countries. Of these, 6873 (79%) were single-country publications originating from 89 countries, whereas 1851 articles (21%) involved international collaboration among 137 countries.

Table 4 and Fig. 2C summarize the top 10 most productive countries, each contributing more than 370 publications. This group comprised four European countries, three Asian countries, two from the Americas, and one from Oceania. South Africa was the leading contributor from Africa, with 49 publications.

**Table 4:** Top 10 productive countries with *TP* > 370 articles.

Country	*TP*	*TP* (*n* = 8724)		*IP*_C_ (*n* = 6873)		*CP*_C_ (*n *= 1851)		*FP* (*n* = 8724)		*RP* (*n* = 8707)		*SP* (*n* = 314)	
		*R* (%)	*CPP*_2024_	*R* (%)	*CPP*_2024_	*R* (%)	*CPP*_2024_	*R* (%)	*CPP*_2024_	*R* (%)	*CPP*_2024_	*R* (%)	*CPP*_2024_
USA	2358	1 (27)	41	1 (23)	43	1 (42)	38	1 (22)	43	1 (22)	42	1 (33)	40
Japan	828	2 (9.5)	22	2 (10)	21	11 (6.8)	29	2 (8.9)	21	2 (9.0)	21	3 (4.8)	20
UK	755	3 (8.7)	47	5 (5.3)	48	2 (21)	46	5 (5.8)	47	5 (5.9)	47	2 (16)	38
China	708	4 (8.1)	12	3 (7.9)	7.8	8 (9.0)	24	3 (7.5)	10	3 (7.5)	11	11 (1.6)	21
Brazil	598	5 (6.9)	19	4 (6.2)	16	7 (9.3)	26	4 (6.1)	17	4 (6.1)	17	8 (2.9)	12
Germany	499	6 (5.7)	32	7 (4.2)	19	3 (12)	50	7 (4.4)	22	7 (4.5)	22	3 (4.8)	14
South Korea	420	7 (4.8)	15	6 (5.2)	13	18 (3.2)	23	6 (4.6)	14	6 (4.6)	14	6 (4.1)	7.8
Spain	398	8 (4.6)	33	8 (3.7)	22	10 (7.7)	51	8 (3.7)	24	8 (3.7)	24	11 (1.6)	5.6
Italy	386	9 (4.4)	30	9 (3.5)	22	9 (7.9)	43	9 (3.5)	24	9 (3.6)	22	15 (1.3)	55
Australia	371	10 (4.3)	30	11 (2.7)	28	4 (10)	32	11 (3.1)	27	10 (3.2)	27	7 (3.2)	56

*TP* number of total articles, *TP R* (%) rank and percentage of total articles, *IP*_C_ *R* (%) rank and percentage of single-country articles in all single-country articles, *CP*_C_ *R* (%) rank and percentage of internationally collaborative articles in all internationally collaborative articles, *FP R* (%) rank and the percentage of first-author articles in all first-author articles, *RP R* (%) rank and the percentage of corresponding-author articles in all corresponding-author articles, *SP R* (%) rank and the percentage of single-author articles in all single-author articles, *CPP*_2024_ average number of citations per publication (*CPP*_2024_ = *TC*_2024_/*TP*).

The United States ranked first across all six publication indicators, including total publications (*TP* = 2358), independent publications (*IP* = 1963), single-country publications (*IP*_*C*_ = 1581), internationally collaborative publications (*CP*_*C*_ = 777), first-author publications (*FP* = 1888), corresponding-author publications (*RP* = 1896), and single-author publications (*SP* = 103).

Among the top 10 most productive countries, the United Kingdom demonstrated the highest citation impact for several publication indicators, whereas Spain showed the highest *CPP*_2024_ for internationally collaborative publications. Australia demonstrated the highest *CPP*_2024_ for single-author publications.

Of the 8724 articles with affiliation information, 2745 (31%) were published by single institutions, whereas 5979 articles (69%) involved inter-institutional collaboration. Table 5 presents the 11 most productive institutions in the field. Harvard University led in total publications and inter-institutionally collaborative publications, whereas the University of São Paulo ranked first in independent publications, first-author publications, and corresponding-author publications. The University of Michigan and the University of Leeds recorded the highest number of single-author publications. Among the top institutions, the University of North Carolina demonstrated the highest *CPP*_2024_ values across several publication indicators, whereas University College London showed the highest *CPP*_2024_ values for both single-institution and single-author publications.

**Table 5:** Top 11 most productive institutions with *TP* > 70 articles.

Institution	*TP*	*TP* (*n* = 8724)		*IP*_I_ (*n* = 2745)		*CP*_I_ (*n* = 5979)		*FP* (*n* = 8724)		*RP* (*n* = 8667)		*SP* (*n* = 314)	
		*R* (%)	*CPP*_2024_	*R* (%)	*CPP*_2024_	*R* (%)	*CPP*_2024_	*R* (%)	*CPP*_2024_	*R* (%)	*CPP*_2024_	*R* (%)	*CPP*_2024_
Harvard U	155	1 (1.8)	42	53 (0.26)	40	1 (2.5)	42	4 (0.57)	35	4 (0.61)	32	41 (0.32)	43
USP	137	2 (1.6)	24	1 (1.3)	17	3 (1.7)	27	1 (0.91)	26	1 (0.90)	26	10 (1.0)	15
UM	116	3 (1.3)	58	8 (0.55)	41	2 (1.7)	61	6 (0.46)	46	11 (0.44)	43	1 (1.9)	49
UCL	101	4 (1.2)	59	11 (0.44)	86	4 (1.5)	56	5 (0.53)	75	5 (0.55)	70	10 (1)	162
SNU	92	5 (1.1)	18	3 (0.80)	18	7 (1.2)	18	2 (0.76)	18	2 (0.77)	18	N/A	N/A
UNC	89	6 (1.0)	68	4 (0.77)	82	8 (1.1)	64	6 (0.46)	83	6 (0.48)	80	N/A	N/A
KCL	83	7 (1.0)	35	22 (0.36)	8	6 (1.2)	39	10 (0.44)	23	9 (0.45)	21	N/A	N/A
JHU	79	8 (0.91)	37	122 (0.15)	39	5 (1.3)	37	25 (0.30)	23	25 (0.31)	22	N/A	N/A
KI	77	9 (0.88)	44	11 (0.44)	59	9 (1.1)	41	14 (0.41)	48	13 (0.43)	48	N/A	N/A
U Sydney	74	10 (0.85)	37	22 (0.36)	37	10 (1.1)	37	18 (0.36)	38	18 (0.38)	34	41 (0.32)	120
TMDU	74	10 (0.85)	17	11 (0.44)	13	11 (1.0)	18	6 (0.46)	17	7 (0.47)	17	41 (0.32)	2.0

*TP* total number of articles, *TP R* (%) rank and percentage of total articles, *IP*_I_ *R* (%) rank and percentage of single-institution articles in all single-institution articles, *CP*_I_ *R* (%) rank and percentage of inter-institutionally collaborative articles in all inter-institutionally collaborative articles, *FP R* (%) rank and percentage of first-author articles in all first-author articles, *RP R* (%) rank and percentage of corresponding-author articles in all corresponding-author articles, *SP R* (%) rank and the percentage of single-author articles in all single-author articles, *CPP*_2024_ average number of citations per publication (*CPP*_2024_ = *TC*_2024_/*TP*), *N/A* not available, *Harvard U* Harvard University, USA, *USP* University of São Paulo, Brazil, *UM* University of Michigan, USA, *UCL* University College London, UK, *SNU* Seoul National University, South Korea, *UNC* University of North Carolina, USA, *Kings Coll London* King’s College London, UK, *JHU* Johns Hopkins University, USA, *KI* Karolinska Institutet, Sweden, *U Sydney* University of Sydney, Australia, *TMDU* Tokyo Medical and Dental University, Japan.

### Citation histories of the ten most frequently cited articles

The ten most frequently cited articles are summarized in Table 6 and Fig. 2D. These publications primarily addressed periodontal disease epidemiology, oral-systemic health associations, nutritional management, and global public health perspectives.

**Table 6:** Top ten most frequently cited oral health with nutritional status-related articles.

Rank (*TC*_2024_)	Rank (*C*_2024_)	Title	Country	Reference
1 (1663)	4 (107)	The global burden of oral diseases and risks to oral health	Switzerland	Petersen et al. (2005) [30]
2 (1517)	1 (243)	ESPEN guideline on clinical nutrition in the intensive care unit	Israel, Estonia, Switzerland, Canada, UK, Belgium, Austria, Germany, Spain, Netherlands, Poland	Singer et al. (2019) [31]
3 (1223)	9 (71)	Prevalence of periodontitis in adults in the United States: 2009 and 2010	USA	Eke et al. (2012a) [32]
4 (1083)	254 (12)	Plasma ghrelin levels in lean and obese humans and the effect of glucose on ghrelin secretion	Japan	Shiiya et al. (2002) [35]
5 (1024)	5 (104)	Update on prevalence of periodontitis in adults in the United States: NHANES 2009 to 2012	USA	Eke et al. (2015) [33]
6 (905)	3 (132)	Update of the case definitions for population-based surveillance of periodontitis	USA	Eke et al. (2012b) [34]
7 (833)	94 (21)	Periodontal infection as a possible risk factor for preterm low birth weight	USA	Offenbacher et al. (1996) [36]
8 (731)	87 (22)	Association between children’s experience of socioeconomic disadvantage and adult health: A life-course study	New Zealand, UK, USA, Canada	Poulton et al. (2002) [37]
9 (720)	254 (12)	Destructive periodontal disease in adults 30 years of age and older in the United States, 1988-1994	USA, Norway	Albandar et al. (1999) [38]
10 (702)	2 (142)	Impact of the global burden of periodontal diseases on health, nutrition and wellbeing of mankind: A call for global action	Italy, China, Germany, USA	Tonetti et al. (2017) [39]

*TC*_2024_ total number of citations from WoSCC since publication year to the end of 2024, *C*_2024_ number of citations of an article in 2024 only.

The most influential publications included “The global burden of oral diseases and risks to oral health” [30], “ESPEN guideline on clinical nutrition in the intensive care unit” [31], and several highly cited epidemiological studies based on NHANES data examining the prevalence and surveillance definitions of periodontitis in adults in the United States [32–34]. Highly cited studies also addressed metabolic regulation and obesity-related mechanisms, including investigations of plasma ghrelin levels in lean and obese individuals [35], as well as the relationships between periodontal disease, adverse pregnancy outcomes, socioeconomic disadvantage, and systemic health consequences [36–38]. Another influential publication, “Impact of the global burden of periodontal diseases on health, nutrition and wellbeing of mankind: A call for global action” [39], emphasized the broader implications of periodontal diseases for general health and nutrition.

Citation trajectories demonstrated sustained citation activity over time, particularly for publications addressing epidemiology, public health burden, and clinical nutrition guidelines.

### Research foci

Keyword-based bibliometric approaches have increasingly been used to identify thematic developments and emerging research trends across scientific disciplines [40, 41]. Based on these approaches, a comprehensive keyword bank was constructed to evaluate thematic developments in oral health and nutritional status research [41].

In the present study, a total of 8747 oral health and nutritional status-related articles published between 1991 and 2024 were analyzed. To assess temporal trends in research focus, these publications were divided into three sub-periods, each containing a comparable number of articles: 1991–2013 (2900 articles), 2014–2020 (3031 articles), and 2021–2024 (2816 articles). Excluding the predefined search terms, the 20 most frequently used author keywords across these articles were identified. Their frequency and distribution across the three time periods are summarized in Table 7, offering insight into the shifting priorities and emerging themes in oral health and nutritional status searches over the past three decades.

**Table 7:** Top 20 most frequently used author keywords during 1991–2024.

Author keywords	*TP*	91-24 *R* (%) *n* = 7584	91-13 *R* (%) *n* = 2327	14-20 *R* (%) *n *= 2638	21-24 *R* (%) *n* = 2619
Obesity	459	1 (6.1)	1 (5.7)	1 (7.4)	1 (5.0)
Epidemiology	307	2 (4.0)	2 (3.6)	2 (5.6)	3 (2.9)
Children	218	3 (2.9)	5 (2.4)	3 (3.6)	6 (2.6)
Deglutition disorders	184	4 (2.4)	3 (2.7)	4 (2.7)	9 (1.9)
Quality of life	173	5 (2.3)	8 (1.8)	6 (2.4)	5 (2.6)
Risk factors	169	6 (2.2)	4 (2.6)	5 (2.4)	10 (1.8)
Stroke	168	7 (2.2)	11 (1.6)	8 (2.2)	4 (2.7)
Elderly	145	8 (1.9)	6 (2.1)	6 (2.4)	20 (1.3)
Deglutition	135	9 (1.8)	6 (2.1)	9 (2.1)	24 (1.2)
Nhanes	127	10 (1.7)	27 (0.86)	20 (1.1)	2 (3.0)
Enteral nutrition	125	11 (1.6)	10 (1.6)	10 (1.9)	16 (1.4)
Head and neck cancer	118	12 (1.6)	14 (1.4)	12 (1.6)	11 (1.6)
Child	100	13 (1.3)	29 (0.82)	11 (1.7)	18 (1.3)
Oral hygiene	100	13 (1.3)	29 (0.82)	13 (1.4)	11 (1.6)
Sarcopenia	100	13 (1.3)	410 (0.13)	14 (1.4)	8 (2.3)
Older adults	99	16 (1.3)	55 (0.60)	33 (0.87)	7 (2.4)
Rehabilitation	97	17 (1.3)	25 (0.90)	17 (1.3)	13 (1.6)
Overweight	86	18 (1.1)	20 (1.0)	16 (1.3)	27 (1.1)
Saliva	85	19 (1.1)	41 (0.69)	15 (1.4)	21 (1.3)
Diabetes mellitus	81	20 (1.1)	23 (0.95)	22 (1.1)	24 (1.2)

*TP* number of articles, % percentage in each period, *R* rank in each period.

**Fig. 3:**
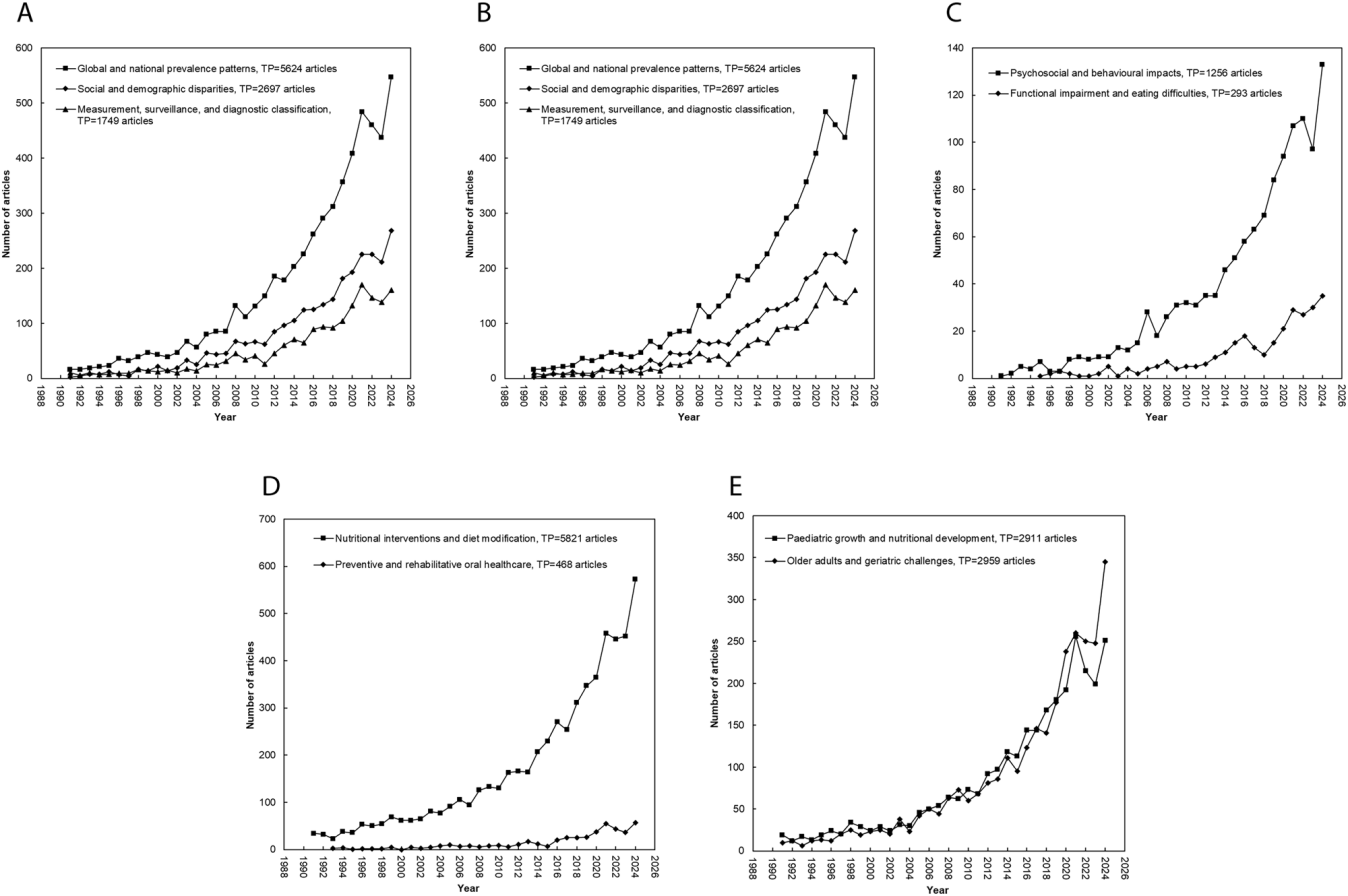
Development trends of major research themes. (**A**) Epidemiology and population burden. (**B**) Nutrition-related determinants of oral function and disease. (**C**) Clinical consequences and quality-of-life outcomes. (**D**) Intervention strategies and public health approaches. (**E**) Special populations and vulnerable groups.

We identified five overarching subjects in the research of the interconnection of nutrition and oral health (Fig. 3: (A) Epidemiology and population burden of oral–nutrition conditions; (B) Nutrition-related determinants of oral function and disease; (C) Clinical consequences and quality-of-life outcomes; (D) Intervention strategies and public health approaches; and (E) Special populations and vulnerable groups.

#### Subject 1: Epidemiology and population burden of oral-nutrition conditions

This subject focuses on the epidemiology and population burden of oral health and nutrition-related conditions. Research has primarily examined prevalence patterns, risk factors, socioeconomic disparities, and age-related vulnerabilities across different populations. Over time, studies increasingly integrated oral health within broader noncommunicable disease frameworks, highlighting the importance of surveillance and population-level monitoring for prevention and public health planning (Fig. 3A).

•*Topic 1:* Global and national prevalence patterns

 ∘*Supporting words:* epidemiology, children, adults, aged, cross-sectional study

•*Topic 2:* Social and demographic disparities

 ∘*Supporting words:* quality of life, life quality, risk factors, smoking, elderly

•*Topic 3:* Measurement, surveillance, and diagnostic classification

 ∘*Supporting words:* malnutrition, deglutition disorders, saliva, xerostomia

#### Subject 2: Nutrition-related determinants of oral function and disease

This subject examines the bidirectional relationship between nutritional status and oral health outcomes. Research has explored how obesity, malnutrition, dietary habits, and systemic conditions influence oral diseases and oral function, while impaired mastication and swallowing may affect nutritional intake. The findings reflect increasing recognition of oral health as both a determinant and consequence of nutritional status (Fig. 3B).

•*Topic 1:* Nutritional status as a determinant of oral health

 ∘*Supporting words:* obesity, underweight, body mass index, BMI, nutritional status

•*Topic 2:* Oral function as a determinant of nutrition

 ∘*Supporting words:* mastication, chewing efficiency, tooth loss, dysphagia

•*Topic 3:* Systemic conditions influencing oral–nutrition interactions

 ∘*Supporting words:* diabetes, diabetic, inflammation, systemic disease, frailty

#### Subject 3: Clinical consequences and quality-of-life outcomes

This subject addresses the clinical and functional consequences associated with impaired oral health and nutritional status. Research has emphasized dysphagia, reduced chewing ability, frailty, sarcopenia, and diminished quality of life, particularly among ageing and medically compromised populations. These studies highlight the broader functional and psychosocial impact of oral–nutrition interactions (Fig. 3C).

•*Topic 1:* Psychosocial and behavioural impacts

 ∘*Supporting words:* quality of life, oral health-related quality of life, elderly, care dependency

•*Topic 2:* Functional impairment and eating difficulties

 ∘*Supporting words:* mastication, chewing ability, swallowing difficulties, oral dysfunction

#### Subject 4: Intervention strategies and public health approaches

This subject focuses on interventions aimed at improving oral health and nutritional outcomes through preventive, rehabilitative, and multidisciplinary approaches. Research has evaluated dietary interventions, oral rehabilitation, enteral nutrition, preventive programs, and healthcare strategies designed to support overall health and quality of life (Fig. 3D).

•*Topic 1:* Nutritional interventions and diet modification

 ∘*Supporting words:* dietary intake, nutrition, feeding difficulties

•*Topic 2:* Preventive and rehabilitative oral healthcare

 ∘*Supporting words:* prosthodontics, denture use, rehabilitation, primary care

#### Subject 5: Special populations and vulnerable groups

This subject highlights research involving vulnerable populations, including older adults, children, stroke patients, individuals with head and neck cancer, and institutionalized populations. Studies have focused on the complex interactions between oral health, nutritional status, systemic disease, and functional decline within high-risk groups (Fig. 3E).

•*Topic 1:* Paediatric growth and nutritional development

 ∘*Supporting words:* children, growth, malnutrition, feeding disorders

•*Topic 2:* Older adults and geriatric challenges

 ∘*Supporting words:* older adults, frailty, dysphagia, xerostomia

## Discussion

The main findings of this bibliometric study were that research linking oral health and nutritional status has increased substantially over the past three decades, with growing interdisciplinary integration across dentistry, nutrition, medicine, geriatrics, and public health. International collaboration was associated with higher citation impact, while thematic analyses demonstrated a progressive shift from epidemiological surveillance toward broader clinical, functional, and policy-oriented research themes. Compared with previous bibliometric studies in this research area, the present analysis provides a broader and more comprehensive overview of the global literature by integrating multidimensional publication and citation indicators with thematic mapping across more than three decades of research output [14, 15]. Beyond describing publication trends, the proposed framework of five overarching research domains offers a structured interpretation of how oral health-nutrition research has evolved across epidemiological, clinical, functional, and public health dimensions [16, 17].

The higher citation impact observed for review articles is consistent with patterns reported in other dental research fields, including temporomandibular disorder and bruxism research [42, 43]. The most highly cited review article, *Epidemiology and causes of preterm birth*, was the first paper in a three-part series on preterm birth and outlines a broad range of determinants of both spontaneous and indicated preterm delivery [28]. The review highlights periodontal disease and low maternal body mass index as key maternal risk factors linking oral health and nutritional status to the etiological pathways of preterm birth [28].

The prolonged citation lifespan observed in the present study is consistent with citation patterns reported in other dental research fields, suggesting sustained long-term scholarly interest in clinically and epidemiologically relevant topics [43]. The steady increase in publication output, together with the dominance of the categories ‘dentistry, oral surgery and medicine’ and ‘nutrition and dietetics’, further highlights the growing interdisciplinary integration of oral health research with nutrition, medicine, geriatrics, and public health. This development likely reflects increasing recognition of the bidirectional relationships between oral conditions, systemic disease, ageing, and nutritional status [30, 44]. These bidirectional relationships may have important implications for overall health, functional independence, and quality of life, reinforcing the need for integrated approaches to the prevention and management of both oral and nutritional conditions [30, 31, 39].

International collaboration was associated with higher citation impact, suggesting that cross-national research networks may facilitate broader visibility and dissemination of research findings [45, 46]. Inter-institutional collaboration was also associated with higher citation impact, further supporting the importance of collaborative research environments in interdisciplinary oral health and nutrition research [45, 46].

The dominance of highly cited publications focusing on periodontal disease epidemiology, oral-systemic associations, and nutritional management reflects the increasing integration of oral health research within broader public health and medical frameworks [44]. Particularly influential were studies using NHANES data and internationally recognized periodontal surveillance definitions, highlighting the importance of standardized epidemiological approaches in shaping contemporary oral health research [32–34, 39].

The thematic analyses identified five major research domains linking oral health and nutritional status: epidemiology and population burden, nutrition-related determinants of oral disease and function, clinical and quality-of-life consequences, intervention strategies, and vulnerable populations. Together, these themes illustrate the increasingly interdisciplinary nature of the field and its integration with public health, geriatrics, chronic disease management, and patient-centred healthcare frameworks [30, 31, 39].

Beyond illustrating the growing research interest in oral-nutrition interactions, the thematic analyses identified a conceptual framework consistent with a bidirectional model in which oral dysfunction, mastication difficulties, dysphagia, and periodontal disease may negatively affect nutritional intake and systemic health, while nutritional imbalances, obesity, frailty, and chronic inflammatory conditions may contribute to deteriorating oral health outcomes [30, 31, 44]. The prominence of ageing-related themes, frailty, and quality-of-life outcomes further emphasizes the importance of oral health as an essential component of healthy ageing and overall wellbeing [30].

In addition, the increasing emphasis on preventive care, rehabilitation, and integrated intervention strategies suggests a gradual shift from isolated disease-oriented approaches toward broader multidisciplinary models that simultaneously address oral health, nutrition, and systemic health [31, 39].

### Study strengths

This study has several methodological strengths. By applying established bibliometric methods to a large dataset spanning more than three decades, it provides a systematic and transparent mapping of global research linking oral health and nutritional status. The use of SCI-EXPANDED as the data source, combined with rigorous data cleaning and multiple citation indicators (*TC*_2024_ and *CPP*_2024_), enhances the reliability of the findings and enables both long-term and contemporary impact assessments. The construction of a comprehensive keyword bank and the division of publications into three balanced time periods make it possible to identify clear thematic developments and emerging research areas. The classification into five overarching subjects supported by structured topic clusters offers a coherent framework for understanding the interdisciplinary nature of oral–nutrition research.

### Study limitations

However, several limitations should be acknowledged. Relying solely on SCI-EXPANDED may underrepresent regionally important or non-English publications, potentially biasing geographic and thematic distributions. Although extensive data treatment was implemented, residual indexing inconsistencies cannot be entirely ruled out. Citation-based metrics reflect research visibility rather than intrinsic quality and may favour fields with broader appeal, such as epidemiology and public health, over specialized clinical domains. Furthermore, keyword analyses depend on the terminology selected by authors, which can vary across disciplines and time periods despite the harmonization efforts achieved through the keyword bank.

Taken together, while these limitations are inherent to bibliometric approaches, the methodological rigor and comprehensive scope of this study provide a robust and informative overview of the research landscape at the intersection and interconnection of oral health and nutritional status.

### Future perspectives

**Fig. 4:**
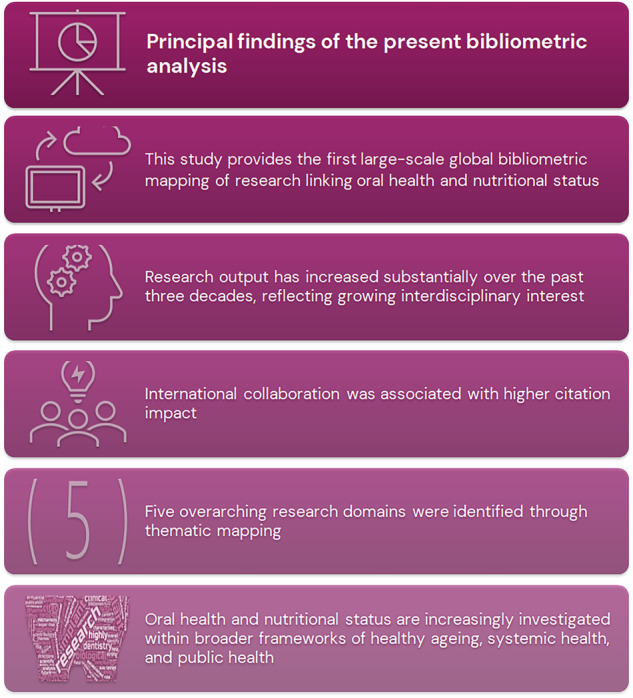
Summary of the principal findings of the present bibliometric analysis. The figure highlights the major findings of the study, including the global expansion of research linking oral health and nutritional status, increasing interdisciplinary interest, the positive association between international collaboration and citation impact, the identification of five overarching research domains, and the growing integration of oral health research within broader frameworks of healthy ageing, systemic health, and public health.

The present bibliometric analysis also highlights several important directions for future research. As the field continues to evolve, future research should further emphasize interdisciplinary studies that elucidate the biological, clinical, and public health dimensions of the relationship between oral health and nutritional status. The five overarching research domains identified in this study provide a useful conceptual framework for guiding future investigations and fostering interdisciplinary collaboration across dentistry, nutrition, geriatrics, and public health. Further international and interdisciplinary collaboration may facilitate more comprehensive investigations and strengthen the evidence base across diverse populations and healthcare settings. In addition, the growing prominence of ageing, frailty, quality of life, and vulnerable populations identified in the present analysis suggests that these areas warrant continued attention. Such efforts may ultimately support the development of more effective preventive strategies, clinical interventions, and public health policies aimed at improving both oral and general health outcomes. The principal findings of the present bibliometric analysis are summarized in Fig. 4.

## Conclusions

This bibliometric analysis provides the first comprehensive overview of global research linking oral health and nutritional status over more than three decades. By integrating multiple citation and publication indicators with keyword-based thematic mapping, the study reveals a steadily expanding and increasingly interdisciplinary field. Research has evolved from foundational epidemiological assessments toward more complex examinations of bidirectional oral–nutrition mechanisms, clinical and quality-of-life consequences, and public health and policy-oriented interventions. The identification of five overarching research subjects illustrates the maturation of the field and its growing alignment with broader non-communicable disease frameworks. While inherent limitations of bibliometric methods remain, the breadth and rigor of the present analysis offer a robust characterization of publication patterns, influential articles, and emerging thematic priorities. These findings may support future interdisciplinary research, preventive strategies, and policy development aimed at improving both oral and general health outcomes.

## Supplementary information

Supplementary material 1 - BIBLIO-Checklist

Supplementary material 2 - Search strategy

Supplementary material 3 - Full cleaned dataset

Supplementary material 4 - Institutional mergers.

## Data Availability

The datasets generated during and/or analysed during the current study are included in this published article and its supplementary information files.

## Generative artificial intelligence in manuscript preparation

No generative AI tools were used for data extraction, data analysis, or generation of scientific findings. Limited language assistance from ChatGPT was used during manuscript preparation. All intellectual content, interpretation, critical revision, and final approval of the manuscript were performed and verified by the authors.
