# Supplementary material for: A global mapping of research on the relationship between oral health and nutritional status
Source: BDJ Open. 2026 Jul 23;12:77. doi: 10.1038/s41405-026-00469-2 (PMC13396446; doi:10.1038/s41405-026-00469-2)
Supplement: Supplementary file 2 — Supplementary material 2 - Search strategy [file 41405_2026_469_MOESM2_ESM.pdf]

Supplementary material 2.

**Table S2.** Documentation of search strategy.
((TI=("periodontitis" OR "dysphagia" OR "oral health" OR "periodontal disease" OR "feeding behavior" OR "dental caries" OR "tooth loss" OR "periodontal diseases" OR "feeding behaviour" OR "xerostomia" OR "oral diseases" OR "dental prostheses" OR "swallowing function" OR "oral disease" OR "oral infection" OR "dental prosthesis" OR "gingival inflammation" OR "feeding behaviors" OR "edentulism" OR "oral infections" OR "masticatory performance" OR "swallowing dysfunction" OR "feeding behaviours" OR "chewing efficiency" OR "swallowing functions" OR "chewing function" OR "oral frailty" OR "teeth loss" OR "oral motor function" OR "swallowing dysfunctions" OR "periodontits" OR "dysphagias" OR "oral motor functions" OR "chewing functions" OR "periodontitits" OR "masticatory performances" OR "dental prosthes" OR "chewing efficiencies" OR "periodontites" OR "periodontital disease" OR "gingival inflammations" OR "dysphagial" OR "periodontitls" OR "periodontitiss" OR "periodontitisprone" OR "periodontital diseases" OR "oral healths" OR "dysphagiam" OR "diet texture modifications" OR "diet texture modification" OR "dental prosthess" OR "dental prosthesists" OR "Dental prosthesisAppendectomy" OR "chewing efficiencygroupintheAwere") OR AB=("periodontitis" OR "dysphagia" OR "oral health" OR "periodontal disease" OR "feeding behavior" OR "dental caries" OR "tooth loss" OR "periodontal diseases" OR "feeding behaviour" OR "xerostomia" OR "oral diseases" OR "dental prostheses" OR "swallowing function" OR "oral disease" OR "oral infection" OR "dental prosthesis" OR "gingival inflammation" OR "feeding behaviors" OR "edentulism" OR "oral infections" OR "masticatory performance" OR "swallowing dysfunction" OR "feeding behaviours" OR "chewing efficiency" OR "swallowing functions" OR "chewing function" OR "oral frailty" OR "teeth loss" OR "oral motor function" OR "swallowing dysfunctions" OR "periodontits" OR "dysphagias" OR "oral motor functions" OR "chewing functions" OR "periodontitits" OR "masticatory performances" OR "dental prosthes" OR "chewing efficiencies" OR "periodontites" OR "periodontital disease" OR "gingival inflammations" OR "dysphagial" OR "periodontitls" OR "periodontitiss" OR "periodontitisprone" OR "periodontital diseases" OR "oral healths" OR "dysphagiam" OR "diet texture modifications" OR "diet texture modification" OR "dental prosthess" OR "dental prosthesists" OR "Dental prosthesisAppendectomy" OR "chewing efficiencygroupintheAwere") OR AK=("periodontitis" OR "dysphagia" OR "oral health" OR "periodontal disease" OR "feeding behavior" OR "dental caries" OR "tooth loss" OR "periodontal diseases" OR "feeding behaviour" OR "xerostomia" OR "oral diseases" OR "dental prostheses" OR "swallowing function" OR "oral disease" OR "oral infection" OR "dental prosthesis" OR "gingival inflammation" OR "feeding behaviors" OR "edentulism" OR "oral infections" OR "masticatory performance" OR "swallowing dysfunction" OR "feeding behaviours" OR "chewing efficiency" OR "swallowing functions" OR "chewing function" OR "oral frailty" OR "teeth loss" OR "oral motor function" OR "swallowing dysfunctions" OR "periodontits" OR "dysphagias" OR "oral motor functions" OR "chewing functions" OR "periodontitits" OR "masticatory performances" OR "dental prosthes" OR "chewing efficiencies" OR "periodontites" OR "periodontital disease" OR "gingival inflammations" OR "dysphagial" OR "periodontitls" OR "periodontitiss" OR

"periodontitisprone" OR "periodontital diseases" OR "oral healths" OR "dysphagiam" OR "diet texture modifications" OR "diet texture modification" OR "dental prosthess" OR "dental prosthesists" OR "Dental prosthesisAppendectomy" OR "chewing efficiencygroupintheAwere")) AND (TI=("nutrition" OR "body mass index" OR "nutritional" OR "BMI" OR "malnutrition" OR "nutritional status" OR "dietary intake" OR "energy intake" OR "dietary habits" OR "dietary intakes" OR "nutritional deficiencies" OR "food selection" OR "energy intakes" OR "nutritional deficiency" OR "body mass indices" OR "body mass indexes" OR "nutrition risk" OR "dietary habit" OR "nutritionals" OR "food selections" OR "nutritions" OR "nutrition risks" OR "nutritional deficiences" OR "malnutritions" OR "nutritional deficiencies" OR "nutritional deficienciens") OR AB=("nutrition" OR "body mass index" OR "nutritional" OR "BMI" OR "malnutrition" OR "nutritional status" OR "dietary intake" OR "energy intake" OR "dietary habits" OR "dietary intakes" OR "nutritional deficiencies" OR "food selection" OR "energy intakes" OR "nutritional deficiency" OR "body mass indices" OR "body mass indexes" OR "nutrition risk" OR "dietary habit" OR "nutritionals" OR "food selections" OR "nutritions" OR "nutrition risks" OR "nutritional deficiences" OR "malnutritions" OR "nutritional deficiencies" OR "nutritional deficienciens") OR AK=("nutrition" OR "body mass index" OR "nutritional" OR "BMI" OR "malnutrition" OR "nutritional status" OR "dietary intake" OR "energy intake" OR "dietary habits" OR "dietary intakes" OR "nutritional deficiencies" OR "food selection" OR "energy intakes" OR "nutritional deficiency" OR "body mass indices" OR "body mass indexes" OR "nutrition risk" OR "dietary habit" OR "nutritionals" OR "food selections" OR "nutritions" OR "nutrition risks" OR "nutritional deficiences" OR "malnutritions" OR "nutritional deficiencies" OR "nutritional deficienciens"))) AND PY=1991-2024
