# Supplementary material for: A global mapping of research on the relationship between oral health and nutritional status
Source: BDJ Open. 2026 Jul 23;12:77. doi: 10.1038/s41405-026-00469-2 (PMC13396446; doi:10.1038/s41405-026-00469-2)
Supplement: Supplementary file 4 — Supplementary material 4 - Institutional mergers. [file 41405_2026_469_MOESM4_ESM.pdf]

Supplementary material 4.

**Table S4.** Summary of the institutional mergers.
• Mayo Clin & Mayo Fdn, USA, Mayo Clin Arizona, USA, Mayo Clin Coll Med Consultant, USA, Mayo Clin Florida, USA, Mayo Clin Minnesota, and Mayo Clin Jacksonville, USA were reclassified as **Mayo Clin, USA**.<br>• Harvard Med Sch, USA, Harvard TH Chan Sch Publ Hlth, USA, and Harvard Sch Dent Med, USA was reclassified as **Harvard Univ, USA**<br>• Sao Paulo State Univ, Brazil was reclassified as **Sao Paulo State Univ UNESP, Brazil**<br>• UCL Queen Sq Inst Neurol, the UK, UCL Inst Epidemiol & Hlth Care, the UK, UCL Eastman Dent Inst, the UK, UCL Eastman Dent Inst Oral Hlth Care Sci, the UK were reclassified as **UCL, the UK**<br>• Ctr Dis Control & Prevent CDC, USA and CDC, USA were reclassified as **Ctr Dis Control & Prevent, USA**<br>• INA PG, France was reclassified as **AgroParisTech, France**<br>• New York Univ, USA and NYU Coll Dent, USA, NYU Coll Nursing, USA; NYU Langone Hlth, USA; NYU Langone Orthoped Hosp, USA; and NYU Steinhardt, USA were reclassified as **NYU, USA**<br>• UAB Hosp Mataro, Spain and CIBERehd CSdM UAB, Spain were reclassified as **Hosp Mataro, Spain**<br>• Charite, Germany was reclassified as **Charite Univ Med Berlin, Germany**<br>• Karolinska Hosp, Sweden and Karolinska Univ Hosp Huddinge, Sweden were reclassified as **Karolinska Univ Hosp, Sweden**<br>• Acad Ctr Dent Amsterdam, Netherlands was reclassified as **Acad Ctr Dent Amsterdam ACTA, Netherlands**<br>• Dept Vet Affairs Med Ctr, USA was reclassified as **Vet Affairs Med Ctr, USA**<br>• Q3 Cleveland Clin, USA was reclassified as **Cleveland Clin, USA**<br>• Linkou Chang Gung Mem Hosp, Taiwan was reclassified as **Chang Gung Mem Hosp, Taiwan**<br>• Fed Univ Rio do Sul Grande UFRGS, Brazil was reclassified as **Univ Fed Rio Grande do Sul, Brazil**<br>• NIDCR, USA was reclassified as **Natl Inst Dent & Craniofacial Res, USA**<br>• Univ Med Greifswald, Germany was reclassified as **Univ Greifswald, Germany**<br>• Johns Hopkins Med, USA, Johns Hopkins Bayview Med Ctr, USA, Johns Hopkins Childrens Ctr, USA, Johns Hopkins Ctr Bariatr Surg, USA, Johns Hopkins Outpatient Ctr, USA, Sidney Kimmel Comprehens Canc Ctr Johns Hopkins, USA were reclassified as **Johns Hopkins Med Inst, USA**<br>• Johns Hopkins, USA, Johns Hopkins Sch Med, USA, Johns Hopkins Bloomberg Sch Publ Hlth, USA were reclassified as **Johns Hopkins Univ, USA**<br>• UFR Odontol, France was reclassified as **Univ Paris Diderot, France**<br>• Fed Univ Rio Grande do Sul UFRGS, Brazil was reclassified as **Univ Fed Rio Grande do Sul, Brazil**

- Univ 655 Colonia Santa Maria Ahuacatitlan, Mexico was reclassified as **Inst Nacl Salud Publ, Mexico**
- Fed Univ Pernambuco UFPE, Brazil was reclassified as **Univ Fed Pernambuco, Brazil**
- Loma Linda Univ Hlth, USA was reclassified as **Loma Linda Univ, USA**
- ICDDR B, Bangladesh was reclassified as **Int Ctr Diarrhoea Dis Res, Bangladesh**
- Ctr Invest & Rehabil Hereditary Ataxias CIRAH, Cuba was reclassified as **Ctr Invest & Rehabil Hereditary Ataxias, Cuba**
- Univ Tunis El Manar UTM, Tunisia was reclassified as **Univ Tunis El Manar, Tunisia**
- Educ Fdn Barretos UNIFEB, Brazil was reclassified as **Educ Fdn Barretos, Brazil**
- Univ Fed Uberlandia UFU, Brazil was reclassified as **Univ Fed Uberlandia, Brazil**
- Jawaharlal Inst Postgrad Med Educ & Res JIPMER, India was reclassified as **Jawaharlal Inst Postgrad Med Educ & Res, India**
- Univ Fed Pernambuco UFPE, Brazil was reclassified as **Univ Fed Pernambuco, Brazil**
- Natl Dent Res Inst Singapore, Singapore was reclassified as **Natl Dent Ctr Singapore, Singapore**
- Natl Univ Heart Ctr, Singapore was reclassified as **NUHS, Singapore**
- Univ Sao Paulos, Brazil was reclassified as **Univ Sao Paulo, Brazil**
- Univ Fed Pernambuco UFPE, Brazil was reclassified as **Univ Fed Pernambuco, Brazil**
- Catalan Inst Oncol, Spain was reclassified as **Catalan Inst Oncol ICO, Spain**
- CRESCA, Spain was reclassified as **Ctr Recerca Seguretat & Control Alimentari UPC CR, Spain**
- Ctr Integral San Prudencio, Spain was reclassified as **Ctr Integral Atenc Mayores San Prudencio, Spain**
- G dAnnunzio Univ Chieti Pescara, Italy was reclassified as **Univ G dAnnunzio, Italy**
- PGIMER, India and Post Grad Inst Med Educ & Res PGIMER, India were reclassified as **Post Grad Inst Med Educ & Res, India**
- UNMC Pediat Dent Residency, USA was reclassified as **Univ Nebraska Med Ctr, USA**
- UAB, USA was reclassified as **Univ Alabama Birmingham, USA**
- UFC, Brazil was reclassified as **Univ Fed Ceara, Brazil**
- LSU, USA was reclassified as **Louisiana State Univ, USA**
- USP, Brazil was reclassified as **Univ Sao Paulo, Brazil**
