# Supplementary material for: A global mapping of research on the relationship between oral health and nutritional status
Source: BDJ Open. 2026 Jul 23;12:77. doi: 10.1038/s41405-026-00469-2 (PMC13396446; doi:10.1038/s41405-026-00469-2)
Supplement: Supplementary file 3 — Supplementary material 3 - Full cleaned dataset [file 41405_2026_469_MOESM3_ESM.pdf]

Supplementary material 3 - *Part 1 - Words in title* . Full cleaned dataset used for analysis. Data cle
standardization were performed in Microsoft Excel 365 using commonly applied bibliometric data-p
procedures, including text standardization, duplicate handling, sorting/filtering functions, citation r
lookup utilities, and pivot table analyses

Words in title	TP	91-24 R (%)	91-13 R (%)	14-20 R (%)
oral	1188	1(13.581799474105408)	3 (10)	2 (14)
patients	1188	1(13.581799474105408)	2 (11)	1 (14)
health	1091	3(12.472847833542929)	4 (10)	3 (13)
feeding	849	4(9.706184977706641)	1 (12)	4 (10)
association	775	5(8.86018063336001)	16 (4.5)	6 (9.1)
children	769	6(8.791585686521094)	5 (8.8)	5 (10)
dysphagia	745	7(8.517205899165429)	11 (6.2)	8 (8.1)
dental	703	8(8.037041271293015)	7 (7.3)	7 (8.6)
nutritional	653	9(7.465416714302046)	8 (7.2)	9 (7.7)
adults	613	10(7.0081170687092715)	22 (3.6)	10 (7.5)
caries	601	11(6.87092717503144)	10 (6.6)	11 (7.5)
periodontitis	595	12(6.802332228192523)	17 (4.2)	15 (6.2)
nutrition	586	13(6.699439807934149)	12 (6)	12 (7.2)
status	574	14(6.562249914256317)	14 (5.3)	13 (7)
disease	548	15(6.265005144621012)	6 (7.6)	16 (5.9)
periodontal	541	16(6.184977706642277)	9 (7.1)	14 (6.3)
older	476	17(5.441865782554019)	32 (2.7)	19 (5.5)
risk	465	18(5.316108380016005)	20 (4)	18 (5.5)
intake	455	19(5.201783468617812)	15 (5)	17 (5.6)
factors	442	20(5.05316108380016)	25 (3.4)	20 (5.2)
food	436	21(4.984566136961243)	13 (5.3)	21 (4.9)
cross-sectional	415	22(4.744483823025037)	277 (0.45)	26 (4.1)
dietary	406	23(4.641591402766663)	19 (4.1)	23 (4.6)
cancer	394	24(4.50440150908883)	18 (4.1)	22 (4.7)
feeding	337	4(9.706184977706641)	1 (12)	4 (10)
obesity	321	25(3.6698296558820167)	21 (3.7)	25 (4.4)
care	313	26(3.5783697267634618)	34 (2.5)	27 (4)
patients	307	1(13.581799474105408)	2 (11)	1 (14)
survey	306	27(3.498342288784726)	54 (1.9)	24 (4.5)
diet	304	28(3.4754773065050877)	23 (3.6)	31 (3.4)
behavior	301	29(3.4411798330856294)	27 (3.1)	29 (3.7)
quality	296	30(3.3840173773865327)	32 (2.7)	30 (3.6)
oral	284	1(13.581799474105408)	3 (10)	2 (14)
health	282	3(12.472847833542929)	4 (10)	3 (13)
relationship	279	31(3.1896650280096037)	26 (3.3)	36 (3.1)
national	264	32(3.0181776609123125)	61 (1.8)	28 (4)
swallowing	262	33(2.995312678632674)	42 (2.1)	41 (2.8)

body	261	34(2.9838801874928547)	29 (2.8)	32 (3.3)
children	255	6(8.791585686521094)	5 (8.8)	5 (10)
elderly	248	35(2.835257802675203)	29 (2.8)	32 (3.3)
life	240	36(2.743797873556648)	65 (1.7)	37 (3.1)
clinical	239	37(2.7323653824168286)	46 (2.1)	41 (2.8)
prevalence	238	38(2.7209328912770094)	42 (2.1)	32 (3.3)
index	228	39(2.6066079798788158)	59 (1.8)	45 (2.6)
cohort	225	40(2.572310506459358)	131 (0.83)	38 (3)
impact	224	41(2.5608780153195383)	56 (1.9)	40 (2.9)
disease	220	15(6.265005144621012)	6 (7.6)	16 (5.9)
treatment	216	42(2.469418086200983)	28 (2.9)	43 (2.7)
dental	212	8(8.037041271293015)	7 (7.3)	7 (8.6)
eating	210	43(2.4008231393620667)	52 (2)	43 (2.7)
nutritional	208	9(7.465416714302046)	8 (7.2)	9 (7.7)
periodontal	206	16(6.184977706642277)	9 (7.1)	14 (6.3)
outcomes	202	44(2.309363210243512)	76 (1.4)	48 (2.4)
examination	200	45(2.2864982279638735)	94 (1.1)	35 (3.1)
weight	200	45(2.2864982279638735)	42 (2.1)	46 (2.5)
population	198	47(2.2636332456842347)	39 (2.2)	55 (2.2)
behaviour	192	48(2.1950382988453185)	24 (3.5)	72 (1.7)
tooth	192	48(2.1950382988453185)	38 (2.4)	57 (2.1)
caries	191	11(6.87092717503144)	10 (6.6)	11 (7.5)
esophageal	190	50(2.17217331656568)	39 (2.2)	50 (2.4)
levels	187	51(2.1378758431462215)	46 (2.1)	63 (2)
stroke	186	52(2.1264433520064023)	48 (2)	68 (1.9)
neck	184	53(2.1035783697267636)	50 (2)	57 (2.1)
malnutrition	183	54(2.092145878586944)	69 (1.6)	57 (2.1)
trial	183	54(2.092145878586944)	100 (1.1)	47 (2.5)
women	183	54(2.092145878586944)	36 (2.4)	68 (1.9)
gastrostomy	182	57(2.080713387447125)	31 (2.7)	57 (2.1)
mass	182	57(2.080713387447125)	65 (1.7)	48 (2.4)
head	181	59(2.0692808963073053)	50 (2)	53 (2.2)
dysphagia	180	7(8.517205899165429)	11 (6.2)	8 (8.1)
assessment	177	60(2.0235509317480282)	52 (2)	66 (1.9)
function	177	60(2.0235509317480282)	65 (1.7)	72 (1.7)
management	177	60(2.0235509317480282)	48 (2)	55 (2.2)
diabetes	175	63(2.000685949468389)	61 (1.8)	57 (2.1)
nutrition	175	13(6.699439807934149)	12 (6)	12 (7.2)
endoscopic	174	64(1.9892534583285697)	35 (2.5)	52 (2.2)
evaluation	174	64(1.9892534583285697)	83 (1.3)	62 (2.1)
childhood	173	66(1.9778209671887506)	54 (1.9)	64 (1.9)
adolescents	165	67(1.8863610380701956)	78 (1.3)	53 (2.2)
chronic	155	68(1.7720361266720017)	45 (2.1)	66 (1.9)

food	155	21(4.984566136961243)	13 (5.3)	21 (4.9)
status	154	14(6.562249914256317)	14 (5.3)	13 (7)
nhanes	152	69(1.7377386532525438)	183 (0.66)	100 (1.4)
intake	146	19(5.201783468617812)	15 (5)	17 (5.6)
protein	145	70(1.657711215273808)	68 (1.7)	86 (1.5)
consumption	143	71(1.6348462329941693)	86 (1.2)	51 (2.3)
korean	143	71(1.6348462329941693)	398 (0.31)	38 (3)
therapy	142	73(1.6234137418543502)	63 (1.7)	74 (1.7)
us	142	73(1.6234137418543502)	88 (1.2)	109 (1.3)
type	140	75(1.6005487595747114)	78 (1.3)	74 (1.7)
prospective	138	76(1.5776837772950727)	86 (1.2)	77 (1.6)
associations	136	77(1.554818795015434)	142 (0.79)	71 (1.8)
role	136	77(1.554818795015434)	56 (1.9)	130 (1)
energy	135	79(1.5433863038756146)	36 (2.4)	108 (1.3)
surgery	135	79(1.5433863038756146)	117 (0.9)	68 (1.9)
long-term	134	81(1.5319538127357952)	75 (1.4)	83 (1.6)
influence	132	82(1.5090888304561563)	56 (1.9)	89 (1.5)
japanese	132	82(1.5090888304561563)	94 (1.1)	64 (1.9)
association	131	5(8.86018063336001)	16 (4.5)	6 (9.1)
development	129	84(1.4747913570366982)	88 (1.2)	83 (1.6)
percutaneous	129	84(1.4747913570366982)	39 (2.2)	97 (1.4)
syndrome	126	86(1.44049388361724)	92 (1.2)	79 (1.6)
acute	125	87(1.4290613924774207)	85 (1.3)	83 (1.6)
oropharyngeal	125	87(1.4290613924774207)	76 (1.4)	102 (1.4)
gastric	124	89(1.4176289013376016)	63 (1.7)	102 (1.4)
young	124	89(1.4176289013376016)	72 (1.5)	92 (1.5)
periodontitis	123	12(6.802332228192523)	17 (4.2)	15 (6.2)
practices	122	91(1.3947639190579628)	106 (1)	78 (1.6)
habits	120	92(1.371898936778324)	106 (1)	112 (1.2)
cancer	119	24(4.50440150908883)	18 (4.1)	22 (4.7)
people	119	93(1.3604664456385047)	94 (1.1)	102 (1.4)
teeth	119	93(1.3604664456385047)	142 (0.79)	86 (1.5)
dietary	118	23(4.641591402766663)	19 (4.1)	23 (4.6)
experience	117	95(1.337601463358866)	69 (1.6)	102 (1.4)
severe	117	95(1.337601463358866)	78 (1.3)	89 (1.5)
risk	116	18(5.316108380016005)	20 (4)	18 (5.5)
age	112	97(1.2804390076597691)	94 (1.1)	89 (1.5)
case	112	97(1.2804390076597691)	98 (1.1)	112 (1.2)
growth	112	97(1.2804390076597691)	69 (1.6)	120 (1.1)
mortality	109	100(1.2461415342403108)	163 (0.72)	92 (1.5)
characteristics	108	101(1.2347090431004915)	142 (0.79)	92 (1.5)
obesity	108	25(3.6698296558820167)	21 (3.7)	25 (4.4)
serum	106	102(1.2118440608208527)	78 (1.3)	115 (1.2)

behaviors	105	103(1.2004115696810334)	142 (0.79)	79 (1.6)
activity	104	104(1.188979078541214)	124 (0.86)	102 (1.4)
adults	104	10(7.0081170687092715)	22 (3.6)	10 (7.5)
child	104	104(1.188979078541214)	92 (1.2)	74 (1.7)
community-dwelling	104	104(1.188979078541214)	206 (0.59)	100 (1.4)
diet	103	28(3.4754773065050877)	23 (3.6)	31 (3.4)
healthy	103	107(1.1775465874013946)	109 (0.93)	79 (1.6)
behaviour	102	48(2.1950382988453185)	24 (3.5)	72 (1.7)
functional	102	108(1.1661140962615753)	124 (0.86)	115 (1.2)
home	102	108(1.1661140962615753)	105 (1)	92 (1.5)
adult	101	110(1.154681605121756)	109 (0.93)	118 (1.2)
factors	100	20(5.05316108380016)	25 (3.4)	20 (5.2)
obese	100	111(1.1432491139819367)	102 (1)	102 (1.4)
physical	99	112(1.1318166228421174)	183 (0.66)	115 (1.2)
retrospective	99	112(1.1318166228421174)	301 (0.41)	134 (1)
salivary	98	114(1.120384131702298)	117 (0.9)	109 (1.3)
diseases	97	115(1.1089516405624786)	168 (0.69)	92 (1.5)
rehabilitation	96	116(1.0975191494226593)	168 (0.69)	126 (1.1)
tube	96	116(1.0975191494226593)	88 (1.2)	112 (1.2)
longitudinal	95	118(1.08608665828284)	124 (0.86)	109 (1.3)
model	95	118(1.08608665828284)	142 (0.79)	145 (0.89)
relationship	95	31(3.1896650280096037)	26 (3.3)	36 (3.1)
patterns	94	120(1.0746541671430205)	109 (0.93)	118 (1.2)
relation	93	121(1.0632216760032012)	78 (1.3)	128 (1.1)
individuals	92	122(1.0517891848633818)	362 (0.34)	97 (1.4)
intervention	92	122(1.0517891848633818)	142 (0.79)	139 (1)
korea	92	122(1.0517891848633818)	1516 (0.069)	86 (1.5)
laparoscopic	92	122(1.0517891848633818)	74 (1.5)	128 (1.1)
changes	91	126(1.0403566937235624)	124 (0.86)	120 (1.1)
behavior	90	29(3.4411798330856294)	27 (3.1)	29 (3.7)
metabolic	90	127(1.028924202583743)	183 (0.66)	79 (1.6)
enteral	88	128(1.0060592203041043)	98 (1.1)	120 (1.1)
diets	85	129(0.9717617468846461)	117 (0.9)	158 (0.82)
pilot	85	129(0.9717617468846461)	163 (0.72)	120 (1.1)
disorders	84	131(0.9603292557448267)	124 (0.86)	149 (0.86)
overweight	84	131(0.9603292557448267)	155 (0.76)	97 (1.4)
states	84	131(0.9603292557448267)	83 (1.3)	149 (0.86)
systemic	84	131(0.9603292557448267)	168 (0.69)	120 (1.1)
nutrient	83	135(0.9488967646050074)	88 (1.2)	126 (1.1)
treatment	83	42(2.469418086200983)	28 (2.9)	43 (2.7)
infants	81	136(0.9260317823253686)	100 (1.1)	134 (1)
maternal	81	136(0.9260317823253686)	117 (0.9)	120 (1.1)
body	80	34(2.9838801874928547)	29 (2.8)	32 (3.3)

elderly	80	35(2.835257802675203)	29 (2.8)	32 (3.3)
nursing	80	138(0.9145992911855493)	301 (0.41)	130 (1)
report	80	138(0.9145992911855493)	131 (0.83)	158 (0.82)
screening	79	140(0.90316680004573)	117 (0.9)	149 (0.86)
gastrostomy	78	57(2.080713387447125)	31 (2.7)	57 (2.1)
sclerosis	78	141(0.8917343089059107)	142 (0.79)	139 (1)
blood	77	142(0.8803018177660913)	131 (0.83)	205 (0.66)
older	77	17(5.441865782554019)	32 (2.7)	19 (5.5)
quality	77	30(3.3840173773865327)	32 (2.7)	30 (3.6)
frailty	75	143(0.8574368354864526)	1516 (0.069)	174 (0.76)
living	75	143(0.8574368354864526)	194 (0.62)	130 (1)
selection	75	143(0.8574368354864526)	59 (1.8)	228 (0.59)
vitamin	75	143(0.8574368354864526)	183 (0.66)	185 (0.73)
care	73	26(3.5783697267634618)	34 (2.5)	27 (4)
inflammation	73	147(0.8345718532068138)	155 (0.76)	217 (0.63)
endoscopic	72	64(1.9892534583285697)	35 (2.5)	52 (2.2)
symptoms	72	148(0.8231393620669945)	109 (0.93)	158 (0.82)
cardiovascular	71	149(0.8117068709271751)	168 (0.69)	149 (0.86)
hospital	71	149(0.8117068709271751)	256 (0.48)	158 (0.82)
conditions	70	151(0.8002743797873557)	155 (0.76)	149 (0.86)
energy	70	79(1.5433863038756146)	36 (2.4)	108 (1.3)
patient	70	151(0.8002743797873557)	109 (0.93)	205 (0.66)
support	70	151(0.8002743797873557)	256 (0.48)	166 (0.79)
women	70	54(2.092145878586944)	36 (2.4)	68 (1.9)
composition	69	154(0.7888418886475363)	218 (0.55)	141 (0.92)
control	69	154(0.7888418886475363)	131 (0.83)	185 (0.73)
does	69	154(0.7888418886475363)	102 (1)	166 (0.79)
rats	69	154(0.7888418886475363)	72 (1.5)	251 (0.56)
review	69	154(0.7888418886475363)	109 (0.93)	251 (0.56)
tooth	69	48(2.1950382988453185)	38 (2.4)	57 (2.1)
number	68	159(0.777409397507717)	520 (0.24)	130 (1)
program	68	159(0.777409397507717)	155 (0.76)	149 (0.86)
evidence	67	161(0.7659769063678976)	155 (0.76)	205 (0.66)
rural	66	162(0.7545444152280781)	124 (0.86)	149 (0.86)
approach	65	163(0.7431119240882588)	206 (0.59)	145 (0.89)
cervical	65	163(0.7431119240882588)	277 (0.45)	141 (0.92)
china	65	163(0.7431119240882588)	398 (0.31)	166 (0.79)
human	65	163(0.7431119240882588)	131 (0.83)	174 (0.76)
lateral	65	163(0.7431119240882588)	168 (0.69)	145 (0.89)
esophageal	64	50(2.17217331656568)	39 (2.2)	50 (2.4)
infection	64	168(0.7316794329484394)	183 (0.66)	145 (0.89)
percutaneous	64	84(1.4747913570366982)	39 (2.2)	97 (1.4)
population	64	47(2.2636332456842347)	39 (2.2)	55 (2.2)

predictors	64	168(0.7316794329484394)	168 (0.69)	251 (0.56)
preschool	64	168(0.7316794329484394)	131 (0.83)	194 (0.69)
residents	64	168(0.7316794329484394)	301 (0.41)	134 (1)
foods	63	172(0.72024694180862)	183 (0.66)	280 (0.49)
general	63	172(0.72024694180862)	163 (0.72)	205 (0.66)
infant	63	172(0.72024694180862)	131 (0.83)	217 (0.63)
pediatric	63	172(0.72024694180862)	218 (0.55)	185 (0.73)
primary	63	172(0.72024694180862)	117 (0.9)	217 (0.63)
school	63	172(0.72024694180862)	194 (0.62)	174 (0.76)
south	63	172(0.72024694180862)	142 (0.79)	134 (1)
aspiration	62	179(0.7088144506688008)	236 (0.52)	174 (0.76)
bone	62	179(0.7088144506688008)	142 (0.79)	228 (0.59)
complications	62	179(0.7088144506688008)	163 (0.72)	174 (0.76)
prevalence	62	38(2.7209328912770094)	42 (2.1)	32 (3.3)
swallowing	62	33(2.995312678632674)	42 (2.1)	41 (2.8)
undergoing	62	179(0.7088144506688008)	362 (0.34)	149 (0.86)
weight	62	45(2.2864982279638735)	42 (2.1)	46 (2.5)
case-control	61	183(0.6973819595289814)	256 (0.48)	158 (0.82)
chronic	61	68(1.7720361266720017)	45 (2.1)	66 (1.9)
gastrointestinal	61	183(0.6973819595289814)	131 (0.83)	158 (0.82)
observational	61	183(0.6973819595289814)	897 (0.14)	194 (0.69)
poor	61	183(0.6973819595289814)	362 (0.34)	141 (0.92)
sugar	61	183(0.6973819595289814)	218 (0.55)	134 (1)
clinical	60	37(2.7323653824168286)	46 (2.1)	41 (2.8)
dairy	60	188(0.685949468389162)	218 (0.55)	194 (0.69)
follow-up	60	188(0.685949468389162)	168 (0.69)	228 (0.59)
levels	60	51(2.1378758431462215)	46 (2.1)	63 (2)
protocol	60	188(0.685949468389162)	595 (0.21)	194 (0.69)
radiotherapy	60	188(0.685949468389162)	206 (0.59)	185 (0.73)
hygiene	59	192(0.6745169772493427)	329 (0.38)	158 (0.82)
implications	59	192(0.6745169772493427)	131 (0.83)	185 (0.73)
management	59	60(2.0235509317480282)	48 (2)	55 (2.2)
outcome	59	192(0.6745169772493427)	106 (1)	228 (0.59)
stroke	59	52(2.1264433520064023)	48 (2)	68 (1.9)
amyotrophic	58	195(0.6630844861095233)	194 (0.62)	174 (0.76)
brazilian	58	195(0.6630844861095233)	183 (0.66)	141 (0.92)
factor	58	195(0.6630844861095233)	194 (0.62)	194 (0.69)
head	58	59(2.0692808963073053)	50 (2)	53 (2.2)
masticatory	58	195(0.6630844861095233)	362 (0.34)	166 (0.79)
neck	58	53(2.1035783697267636)	50 (2)	57 (2.1)
pneumonia	58	195(0.6630844861095233)	329 (0.38)	185 (0.73)
sleep	58	195(0.6630844861095233)	277 (0.45)	158 (0.82)
social	58	195(0.6630844861095233)	131 (0.83)	280 (0.49)

assessment	57	60(2.0235509317480282)	52 (2)	66 (1.9)
community	57	202(0.6516519949697039)	236 (0.52)	166 (0.79)
eating	57	43(2.4008231393620667)	52 (2)	43 (2.7)
parameters	57	202(0.6516519949697039)	206 (0.59)	228 (0.59)
problems	57	202(0.6516519949697039)	131 (0.83)	194 (0.69)
subjects	57	202(0.6516519949697039)	109 (0.93)	251 (0.56)
system	57	202(0.6516519949697039)	168 (0.69)	228 (0.59)
covid-19	56	207(0.6402195038298846)	#SAKNAS!	1668 (0.066)
dysfunction	56	207(0.6402195038298846)	194 (0.62)	228 (0.59)
inflammatory	56	207(0.6402195038298846)	256 (0.48)	217 (0.63)
mellitus	56	207(0.6402195038298846)	163 (0.72)	217 (0.63)
students	56	207(0.6402195038298846)	277 (0.45)	264 (0.53)
tongue	56	207(0.6402195038298846)	398 (0.31)	174 (0.76)
versus	56	207(0.6402195038298846)	277 (0.45)	185 (0.73)
bypass	55	214(0.6287870126900652)	236 (0.52)	166 (0.79)
childhood	55	66(1.9778209671887506)	54 (1.9)	64 (1.9)
cognitive	55	214(0.6287870126900652)	329 (0.38)	174 (0.76)
survey	55	27(3.498342288784726)	54 (1.9)	24 (4.5)
survival	55	214(0.6287870126900652)	194 (0.62)	185 (0.73)
impact	54	41(2.5608780153195383)	56 (1.9)	40 (2.9)
influence	54	82(1.5090888304561563)	56 (1.9)	89 (1.5)
men	54	217(0.6173545215502457)	102 (1)	280 (0.49)
role	54	77(1.554818795015434)	56 (1.9)	130 (1)
prevention	53	218(0.6059220304104264)	183 (0.66)	361 (0.4)
chewing	52	219(0.594489539270607)	218 (0.55)	205 (0.66)
index	52	39(2.6066079798788158)	59 (1.8)	45 (2.6)
knowledge	52	219(0.594489539270607)	362 (0.34)	280 (0.49)
selection	52	143(0.8574368354864526)	59 (1.8)	228 (0.59)
carcinoma	51	221(0.5830570481307876)	236 (0.52)	280 (0.49)
determinants	51	221(0.5830570481307876)	256 (0.48)	174 (0.76)
diabetes	51	63(2.000685949468389)	61 (1.8)	57 (2.1)
exposure	51	221(0.5830570481307876)	206 (0.59)	280 (0.49)
national	51	32(3.0181776609123125)	61 (1.8)	28 (4)
cows	50	224(0.5716245569909684)	256 (0.48)	194 (0.69)
gastric	50	89(1.4176289013376016)	63 (1.7)	102 (1.4)
microbiota	50	224(0.5716245569909684)	595 (0.21)	251 (0.56)
placement	50	224(0.5716245569909684)	206 (0.59)	166 (0.79)
therapy	50	73(1.6234137418543502)	63 (1.7)	74 (1.7)
validation	50	224(0.5716245569909684)	362 (0.34)	217 (0.63)
acid	49	228(0.560192065851149)	236 (0.52)	264 (0.53)
dementia	49	228(0.560192065851149)	301 (0.41)	185 (0.73)
function	49	60(2.0235509317480282)	65 (1.7)	72 (1.7)
group	49	228(0.560192065851149)	155 (0.76)	333 (0.43)

life	49	36(2.743797873556648)	65 (1.7)	37 (3.1)
mass	49	57(2.080713387447125)	65 (1.7)	48 (2.4)
parental	49	228(0.560192065851149)	398 (0.31)	166 (0.79)
pressure	49	228(0.560192065851149)	362 (0.34)	280 (0.49)
appetite	48	233(0.5487595747113296)	142 (0.79)	361 (0.4)
birth	48	233(0.5487595747113296)	236 (0.52)	251 (0.56)
cerebral	48	233(0.5487595747113296)	256 (0.48)	251 (0.56)
heart	48	233(0.5487595747113296)	206 (0.59)	305 (0.46)
protein	48	70(1.657711215273808)	68 (1.7)	86 (1.5)
brazil	47	237(0.5373270835715103)	183 (0.66)	305 (0.46)
chinese	47	237(0.5373270835715103)	451 (0.28)	174 (0.76)
education	47	237(0.5373270835715103)	236 (0.52)	361 (0.4)
experience	47	95(1.337601463358866)	69 (1.6)	102 (1.4)
growth	47	97(1.2804390076597691)	69 (1.6)	120 (1.1)
malnutrition	47	54(2.092145878586944)	69 (1.6)	57 (2.1)
medical	47	237(0.5373270835715103)	256 (0.48)	305 (0.46)
novel	47	237(0.5373270835715103)	398 (0.31)	205 (0.66)
receptor	47	237(0.5373270835715103)	218 (0.55)	217 (0.63)
reflux	47	237(0.5373270835715103)	206 (0.59)	305 (0.46)
american	46	244(0.5258945924316909)	256 (0.48)	305 (0.46)
condition	46	244(0.5258945924316909)	329 (0.38)	251 (0.56)
indicators	46	244(0.5258945924316909)	301 (0.41)	264 (0.53)
score	46	244(0.5258945924316909)	2359 (0.034)	228 (0.59)
severity	46	244(0.5258945924316909)	218 (0.55)	461 (0.3)
according	45	249(0.5144621012918715)	520 (0.24)	205 (0.66)
dentition	45	249(0.5144621012918715)	236 (0.52)	280 (0.49)
foraging	45	249(0.5144621012918715)	109 (0.93)	333 (0.43)
geriatric	45	249(0.5144621012918715)	206 (0.59)	305 (0.46)
palsy	45	249(0.5144621012918715)	329 (0.38)	305 (0.46)
parkinson	45	249(0.5144621012918715)	301 (0.41)	264 (0.53)
regulation	45	249(0.5144621012918715)	117 (0.9)	361 (0.4)
bmi	44	256(0.5030296101520522)	362 (0.34)	251 (0.56)
muscle	44	256(0.5030296101520522)	1121 (0.1)	264 (0.53)
profile	44	256(0.5030296101520522)	398 (0.31)	280 (0.49)
rats	44	154(0.7888418886475363)	72 (1.5)	251 (0.56)
smoking	44	256(0.5030296101520522)	168 (0.69)	251 (0.56)
time	44	256(0.5030296101520522)	236 (0.52)	361 (0.4)
young	44	89(1.4176289013376016)	72 (1.5)	92 (1.5)
central	43	261(0.49159711901223274)	155 (0.76)	305 (0.46)
c-reactive	43	261(0.49159711901223274)	218 (0.55)	264 (0.53)
fat	43	261(0.49159711901223274)	218 (0.55)	228 (0.59)
gingival	43	261(0.49159711901223274)	218 (0.55)	228 (0.59)
laparoscopic	43	122(1.0517891848633818)	74 (1.5)	128 (1.1)

milk	43	261(0.49159711901223274)	329 (0.38)	305 (0.46)
saliva	43	261(0.49159711901223274)	398 (0.31)	228 (0.59)
state	43	261(0.49159711901223274)	194 (0.62)	264 (0.53)
anterior	42	268(0.4801646278724134)	520 (0.24)	228 (0.59)
behaviours	42	268(0.4801646278724134)	301 (0.41)	228 (0.59)
change	42	268(0.4801646278724134)	329 (0.38)	194 (0.69)
correlation	42	268(0.4801646278724134)	301 (0.41)	228 (0.59)
daily	42	268(0.4801646278724134)	451 (0.28)	228 (0.59)
frequency	42	268(0.4801646278724134)	236 (0.52)	264 (0.53)
leptin	42	268(0.4801646278724134)	142 (0.79)	280 (0.49)
lifestyle	42	268(0.4801646278724134)	595 (0.21)	228 (0.59)
plasma	42	268(0.4801646278724134)	168 (0.69)	305 (0.46)
sarcopenia	42	268(0.4801646278724134)	#SAKNAS!	361 (0.4)
species	42	268(0.4801646278724134)	194 (0.62)	280 (0.49)
surgical	42	268(0.4801646278724134)	206 (0.59)	305 (0.46)
water	42	268(0.4801646278724134)	301 (0.41)	228 (0.59)
brain	41	281(0.468732136732594)	301 (0.41)	228 (0.59)
cell	41	281(0.468732136732594)	398 (0.31)	305 (0.46)
japan	41	281(0.468732136732594)	451 (0.28)	217 (0.63)
long-term	41	81(1.5319538127357952)	75 (1.4)	83 (1.6)
mice	41	281(0.468732136732594)	329 (0.38)	205 (0.66)
multicenter	41	281(0.468732136732594)	897 (0.14)	280 (0.49)
nationwide	41	281(0.468732136732594)	1121 (0.1)	280 (0.49)
parents	41	281(0.468732136732594)	236 (0.52)	333 (0.43)
predictive	41	281(0.468732136732594)	398 (0.31)	305 (0.46)
rate	41	281(0.468732136732594)	168 (0.69)	280 (0.49)
responses	41	281(0.468732136732594)	194 (0.62)	361 (0.4)
socioeconomic	41	281(0.468732136732594)	362 (0.34)	149 (0.86)
stress	41	281(0.468732136732594)	717 (0.17)	174 (0.76)
ability	40	293(0.45729964559277464)	451 (0.28)	217 (0.63)
affect	40	293(0.45729964559277464)	398 (0.31)	217 (0.63)
assessing	40	293(0.45729964559277464)	520 (0.24)	264 (0.53)
comparative	40	293(0.45729964559277464)	398 (0.31)	361 (0.4)
male	40	293(0.45729964559277464)	256 (0.48)	228 (0.59)
metabolism	40	293(0.45729964559277464)	301 (0.41)	361 (0.4)
mothers	40	293(0.45729964559277464)	277 (0.45)	217 (0.63)
multiple	40	293(0.45729964559277464)	362 (0.34)	422 (0.33)
oropharyngeal	40	87(1.4290613924774207)	76 (1.4)	102 (1.4)
outcomes	40	44(2.309363210243512)	76 (1.4)	48 (2.4)
postoperative	40	293(0.45729964559277464)	595 (0.21)	333 (0.43)
preference	40	293(0.45729964559277464)	168 (0.69)	422 (0.33)
receiving	40	293(0.45729964559277464)	301 (0.41)	388 (0.36)
recovery	40	293(0.45729964559277464)	362 (0.34)	388 (0.36)

achalasia	39	305(0.4458671544529553)	277 (0.45)	205 (0.66)
adolescents	39	67(1.8863610380701956)	78 (1.3)	53 (2.2)
incidence	39	305(0.4458671544529553)	277 (0.45)	461 (0.3)
meal	39	305(0.4458671544529553)	520 (0.24)	251 (0.56)
relation	39	121(1.0632216760032012)	78 (1.3)	128 (1.1)
serum	39	102(1.2118440608208527)	78 (1.3)	115 (1.2)
severe	39	95(1.337601463358866)	78 (1.3)	89 (1.5)
training	39	305(0.4458671544529553)	595 (0.21)	264 (0.53)
type	39	75(1.6005487595747114)	78 (1.3)	74 (1.7)
disorder	38	309(0.43443466331313596)	717 (0.17)	194 (0.69)
drosophila	38	309(0.43443466331313596)	520 (0.24)	205 (0.66)
evaluation	38	64(1.9892534583285697)	83 (1.3)	62 (2.1)
fundoplication	38	309(0.43443466331313596)	256 (0.48)	361 (0.4)
markers	38	309(0.43443466331313596)	301 (0.41)	264 (0.53)
natural	38	309(0.43443466331313596)	301 (0.41)	228 (0.59)
needs	38	309(0.43443466331313596)	301 (0.41)	584 (0.23)
old	38	309(0.43443466331313596)	218 (0.55)	305 (0.46)
post-stroke	38	309(0.43443466331313596)	897 (0.14)	999 (0.13)
reconstruction	38	309(0.43443466331313596)	301 (0.41)	388 (0.36)
states	38	131(0.9603292557448267)	83 (1.3)	149 (0.86)
variation	38	309(0.43443466331313596)	168 (0.69)	280 (0.49)
acute	37	87(1.4290613924774207)	85 (1.3)	83 (1.6)
concentrations	37	319(0.4230021721733166)	256 (0.48)	228 (0.59)
expression	37	319(0.4230021721733166)	218 (0.55)	333 (0.43)
female	37	319(0.4230021721733166)	194 (0.62)	422 (0.33)
gastrectomy	37	319(0.4230021721733166)	897 (0.14)	228 (0.59)
gene	37	319(0.4230021721733166)	277 (0.45)	194 (0.69)
improve	37	319(0.4230021721733166)	236 (0.52)	361 (0.4)
pattern	37	319(0.4230021721733166)	362 (0.34)	305 (0.46)
preliminary	37	319(0.4230021721733166)	277 (0.45)	461 (0.3)
safety	37	319(0.4230021721733166)	520 (0.24)	205 (0.66)
sarcopenic	37	319(0.4230021721733166)	#SAKNAS!	280 (0.49)
southern	37	319(0.4230021721733166)	194 (0.62)	584 (0.23)
taste	37	319(0.4230021721733166)	362 (0.34)	264 (0.53)
aging	36	331(0.4115696810334972)	329 (0.38)	584 (0.23)
consumption	36	71(1.6348462329941693)	86 (1.2)	51 (2.3)
efficacy	36	331(0.4115696810334972)	595 (0.21)	333 (0.43)
family	36	331(0.4115696810334972)	277 (0.45)	526 (0.26)
gastroesophageal	36	331(0.4115696810334972)	236 (0.52)	422 (0.33)
obstructive	36	331(0.4115696810334972)	236 (0.52)	305 (0.46)
prospective	36	76(1.5776837772950727)	86 (1.2)	77 (1.6)
roux-en-y	36	331(0.4115696810334972)	362 (0.34)	280 (0.49)
schoolchildren	36	331(0.4115696810334972)	236 (0.52)	422 (0.33)

sea	36	331(0.4115696810334972)	142 (0.79)	584 (0.23)
stimulation	36	331(0.4115696810334972)	398 (0.31)	228 (0.59)
upper	36	331(0.4115696810334972)	301 (0.41)	305 (0.46)
urban	36	331(0.4115696810334972)	218 (0.55)	264 (0.53)
behavioral	35	342(0.40013718989367786)	329 (0.38)	280 (0.49)
content	35	342(0.40013718989367786)	277 (0.45)	388 (0.36)
development	35	84(1.4747913570366982)	88 (1.2)	83 (1.6)
fatty	35	342(0.40013718989367786)	277 (0.45)	461 (0.3)
guidelines	35	342(0.40013718989367786)	277 (0.45)	333 (0.43)
microbiome	35	342(0.40013718989367786)	1516 (0.069)	361 (0.4)
nutrient	35	135(0.9488967646050074)	88 (1.2)	126 (1.1)
postmenopausal	35	342(0.40013718989367786)	256 (0.48)	280 (0.49)
properties	35	342(0.40013718989367786)	1121 (0.1)	264 (0.53)
systematic	35	342(0.40013718989367786)	595 (0.21)	422 (0.33)
tube	35	116(1.0975191494226593)	88 (1.2)	112 (1.2)
us	35	73(1.6234137418543502)	88 (1.2)	109 (1.3)
affects	34	350(0.3887046987538585)	168 (0.69)	422 (0.33)
bacterial	34	350(0.3887046987538585)	520 (0.24)	264 (0.53)
child	34	104(1.188979078541214)	92 (1.2)	74 (1.7)
comprehensive	34	350(0.3887046987538585)	520 (0.24)	388 (0.36)
diagnosis	34	350(0.3887046987538585)	362 (0.34)	422 (0.33)
failure	34	350(0.3887046987538585)	520 (0.24)	361 (0.4)
intakes	34	350(0.3887046987538585)	362 (0.34)	194 (0.69)
mouth	34	350(0.3887046987538585)	362 (0.34)	461 (0.3)
predicting	34	350(0.3887046987538585)	717 (0.17)	280 (0.49)
pregnant	34	350(0.3887046987538585)	451 (0.28)	388 (0.36)
reduces	34	350(0.3887046987538585)	236 (0.52)	280 (0.49)
strategies	34	350(0.3887046987538585)	206 (0.59)	388 (0.36)
syndrome	34	86(1.44049388361724)	92 (1.2)	79 (1.6)
tool	34	350(0.3887046987538585)	520 (0.24)	205 (0.66)
age	33	97(1.2804390076597691)	94 (1.1)	89 (1.5)
chemoradiotherapy	33	362(0.37727220761403907)	277 (0.45)	305 (0.46)
chemotherapy	33	362(0.37727220761403907)	595 (0.21)	333 (0.43)
examination	33	45(2.2864982279638735)	94 (1.1)	35 (3.1)
exploring	33	362(0.37727220761403907)	520 (0.24)	584 (0.23)
gain	33	362(0.37727220761403907)	520 (0.24)	333 (0.43)
glucose	33	362(0.37727220761403907)	329 (0.38)	280 (0.49)
hypothalamic	33	362(0.37727220761403907)	183 (0.66)	422 (0.33)
impairment	33	362(0.37727220761403907)	595 (0.21)	333 (0.43)
japanese	33	82(1.5090888304561563)	94 (1.1)	64 (1.9)
larvae	33	362(0.37727220761403907)	124 (0.86)	584 (0.23)
months	33	362(0.37727220761403907)	451 (0.28)	388 (0.36)
palliative	33	362(0.37727220761403907)	277 (0.45)	388 (0.36)

people	33	93(1.3604664456385047)	94 (1.1)	102 (1.4)
pregnancy	33	362(0.37727220761403907)	218 (0.55)	694 (0.2)
qualitative	33	362(0.37727220761403907)	717 (0.17)	361 (0.4)
relationships	33	362(0.37727220761403907)	398 (0.31)	361 (0.4)
supplementation	33	362(0.37727220761403907)	451 (0.28)	388 (0.36)
value	33	362(0.37727220761403907)	362 (0.34)	305 (0.46)
2009-2014	32	377(0.3658397164742197)	#SAKNAS!	2548 (0.033)
area	32	377(0.3658397164742197)	451 (0.28)	526 (0.26)
case	32	97(1.2804390076597691)	98 (1.1)	112 (1.2)
common	32	377(0.3658397164742197)	520 (0.24)	333 (0.43)
current	32	377(0.3658397164742197)	329 (0.38)	333 (0.43)
density	32	377(0.3658397164742197)	256 (0.48)	461 (0.3)
enteral	32	128(1.0060592203041043)	98 (1.1)	120 (1.1)
fruit	32	377(0.3658397164742197)	256 (0.48)	461 (0.3)
long	32	377(0.3658397164742197)	595 (0.21)	264 (0.53)
methods	32	377(0.3658397164742197)	362 (0.34)	333 (0.43)
research	32	377(0.3658397164742197)	398 (0.31)	694 (0.2)
swallow	32	377(0.3658397164742197)	301 (0.41)	388 (0.36)
traits	32	377(0.3658397164742197)	717 (0.17)	526 (0.26)
anorexia	31	388(0.3544072253344004)	277 (0.45)	526 (0.26)
infants	31	136(0.9260317823253686)	100 (1.1)	134 (1)
measures	31	388(0.3544072253344004)	362 (0.34)	422 (0.33)
oesophageal	31	388(0.3544072253344004)	236 (0.52)	388 (0.36)
practice	31	388(0.3544072253344004)	398 (0.31)	333 (0.43)
questionnaire	31	388(0.3544072253344004)	398 (0.31)	584 (0.23)
repair	31	388(0.3544072253344004)	362 (0.34)	305 (0.46)
size	31	388(0.3544072253344004)	218 (0.55)	526 (0.26)
trial	31	54(2.092145878586944)	100 (1.1)	47 (2.5)
university	31	388(0.3544072253344004)	717 (0.17)	461 (0.3)
affecting	30	396(0.342974734194581)	595 (0.21)	584 (0.23)
alzheimer	30	396(0.342974734194581)	398 (0.31)	461 (0.3)
balance	30	396(0.342974734194581)	329 (0.38)	333 (0.43)
choice	30	396(0.342974734194581)	451 (0.28)	388 (0.36)
coronary	30	396(0.342974734194581)	236 (0.52)	461 (0.3)
does	30	154(0.7888418886475363)	102 (1)	166 (0.79)
duration	30	396(0.342974734194581)	717 (0.17)	361 (0.4)
dysphagic	30	396(0.342974734194581)	362 (0.34)	422 (0.33)
fluid	30	396(0.342974734194581)	362 (0.34)	333 (0.43)
fluoride	30	396(0.342974734194581)	218 (0.55)	584 (0.23)
genetic	30	396(0.342974734194581)	398 (0.31)	526 (0.26)
inpatients	30	396(0.342974734194581)	717 (0.17)	461 (0.3)
men	30	217(0.6173545215502457)	102 (1)	280 (0.49)
nasogastric	30	396(0.342974734194581)	236 (0.52)	584 (0.23)

obese	30	111(1.1432491139819367)	102 (1)	102 (1.4)
phase	30	396(0.342974734194581)	595 (0.21)	422 (0.33)
sleeve	30	396(0.342974734194581)	1121 (0.1)	280 (0.49)
against	29	410(0.33154224305476165)	520 (0.24)	388 (0.36)
bariatric	29	410(0.33154224305476165)	595 (0.21)	361 (0.4)
breastfeeding	29	410(0.33154224305476165)	520 (0.24)	526 (0.26)
edentulism	29	410(0.33154224305476165)	277 (0.45)	526 (0.26)
fish	29	410(0.33154224305476165)	236 (0.52)	526 (0.26)
hernia	29	410(0.33154224305476165)	451 (0.28)	388 (0.36)
home	29	108(1.1661140962615753)	105 (1)	92 (1.5)
hypertension	29	410(0.33154224305476165)	520 (0.24)	694 (0.2)
ischemic	29	410(0.33154224305476165)	717 (0.17)	526 (0.26)
low-income	29	410(0.33154224305476165)	329 (0.38)	361 (0.4)
muscular	29	410(0.33154224305476165)	451 (0.28)	816 (0.16)
peg	29	410(0.33154224305476165)	183 (0.66)	694 (0.2)
perception	29	410(0.33154224305476165)	897 (0.14)	461 (0.3)
preoperative	29	410(0.33154224305476165)	520 (0.24)	461 (0.3)
project	29	410(0.33154224305476165)	595 (0.21)	305 (0.46)
spanish	29	410(0.33154224305476165)	717 (0.17)	388 (0.36)
trends	29	410(0.33154224305476165)	897 (0.14)	333 (0.43)
xerostomia	29	410(0.33154224305476165)	595 (0.21)	305 (0.46)
activities	28	427(0.3201097519149423)	595 (0.21)	422 (0.33)
environmental	28	427(0.3201097519149423)	329 (0.38)	388 (0.36)
habits	28	92(1.371898936778324)	106 (1)	112 (1.2)
interaction	28	427(0.3201097519149423)	595 (0.21)	333 (0.43)
learning	28	427(0.3201097519149423)	520 (0.24)	816 (0.16)
myotomy	28	427(0.3201097519149423)	398 (0.31)	333 (0.43)
northern	28	427(0.3201097519149423)	595 (0.21)	361 (0.4)
outcome	28	192(0.6745169772493427)	106 (1)	228 (0.59)
practices	28	91(1.3947639190579628)	106 (1)	78 (1.6)
preferences	28	427(0.3201097519149423)	301 (0.41)	305 (0.46)
radiation	28	427(0.3201097519149423)	301 (0.41)	461 (0.3)
sensory	28	427(0.3201097519149423)	717 (0.17)	333 (0.43)
strength	28	427(0.3201097519149423)	2359 (0.034)	305 (0.46)
utilization	28	427(0.3201097519149423)	301 (0.41)	461 (0.3)
wild	28	427(0.3201097519149423)	256 (0.48)	584 (0.23)
acids	27	439(0.30867726077512286)	329 (0.38)	816 (0.16)
adult	27	110(1.154681605121756)	109 (0.93)	118 (1.2)
attitudes	27	439(0.30867726077512286)	451 (0.28)	584 (0.23)
burden	27	439(0.30867726077512286)	595 (0.21)	388 (0.36)
environment	27	439(0.30867726077512286)	451 (0.28)	422 (0.33)
feasibility	27	439(0.30867726077512286)	1121 (0.1)	280 (0.49)
foraging	27	249(0.5144621012918715)	109 (0.93)	333 (0.43)

ghrelin	27	439(0.30867726077512286)	168 (0.69)	816 (0.16)
healthy	27	107(1.1775465874013946)	109 (0.93)	79 (1.6)
history	27	439(0.30867726077512286)	520 (0.24)	388 (0.36)
improves	27	439(0.30867726077512286)	897 (0.14)	361 (0.4)
interventions	27	439(0.30867726077512286)	717 (0.17)	461 (0.3)
macronutrient	27	439(0.30867726077512286)	194 (0.62)	816 (0.16)
mediterranean	27	439(0.30867726077512286)	595 (0.21)	422 (0.33)
motor	27	439(0.30867726077512286)	256 (0.48)	461 (0.3)
patient	27	151(0.8002743797873557)	109 (0.93)	205 (0.66)
patterns	27	120(1.0746541671430205)	109 (0.93)	118 (1.2)
persons	27	439(0.30867726077512286)	595 (0.21)	461 (0.3)
pigs	27	439(0.30867726077512286)	301 (0.41)	694 (0.2)
possible	27	439(0.30867726077512286)	595 (0.21)	422 (0.33)
predict	27	439(0.30867726077512286)	362 (0.34)	361 (0.4)
resistance	27	439(0.30867726077512286)	717 (0.17)	251 (0.56)
restriction	27	439(0.30867726077512286)	277 (0.45)	388 (0.36)
review	27	154(0.7888418886475363)	109 (0.93)	251 (0.56)
services	27	439(0.30867726077512286)	362 (0.34)	526 (0.26)
stage	27	439(0.30867726077512286)	451 (0.28)	584 (0.23)
strategy	27	439(0.30867726077512286)	595 (0.21)	388 (0.36)
subjects	27	202(0.6516519949697039)	109 (0.93)	251 (0.56)
survivors	27	439(0.30867726077512286)	2359 (0.034)	251 (0.56)
symptoms	27	148(0.8231393620669945)	109 (0.93)	158 (0.82)
texture	27	439(0.30867726077512286)	1121 (0.1)	333 (0.43)
aspects	26	462(0.2972447696353035)	256 (0.48)	816 (0.16)
banding	26	462(0.2972447696353035)	155 (0.76)	999 (0.13)
caregivers	26	462(0.2972447696353035)	398 (0.31)	694 (0.2)
diets	26	129(0.9717617468846461)	117 (0.9)	158 (0.82)
efficiency	26	462(0.2972447696353035)	398 (0.31)	388 (0.36)
esophagectomy	26	462(0.2972447696353035)	520 (0.24)	333 (0.43)
exercise	26	462(0.2972447696353035)	451 (0.28)	526 (0.26)
investigation	26	462(0.2972447696353035)	897 (0.14)	816 (0.16)
maternal	26	136(0.9260317823253686)	117 (0.9)	120 (1.1)
permanent	26	462(0.2972447696353035)	301 (0.41)	526 (0.26)
plant	26	462(0.2972447696353035)	362 (0.34)	422 (0.33)
populations	26	462(0.2972447696353035)	329 (0.38)	461 (0.3)
prediction	26	462(0.2972447696353035)	451 (0.28)	816 (0.16)
predicts	26	462(0.2972447696353035)	717 (0.17)	584 (0.23)
primary	26	172(0.72024694180862)	117 (0.9)	217 (0.63)
rat	26	462(0.2972447696353035)	206 (0.59)	694 (0.2)
recommendations	26	462(0.2972447696353035)	451 (0.28)	422 (0.33)
regulation	26	249(0.5144621012918715)	117 (0.9)	361 (0.4)
representative	26	462(0.2972447696353035)	717 (0.17)	388 (0.36)

respiratory	26	462(0.2972447696353035)	595 (0.21)	526 (0.26)
salivary	26	114(1.120384131702298)	117 (0.9)	109 (1.3)
screening	26	140(0.90316680004573)	117 (0.9)	149 (0.86)
single	26	462(0.2972447696353035)	897 (0.14)	388 (0.36)
surgery	26	79(1.5433863038756146)	117 (0.9)	68 (1.9)
tissue	26	462(0.2972447696353035)	398 (0.31)	461 (0.3)
undernutrition	26	462(0.2972447696353035)	595 (0.21)	305 (0.46)
activity	25	104(1.188979078541214)	124 (0.86)	102 (1.4)
adiposity	25	481(0.2858122784954842)	329 (0.38)	461 (0.3)
adolescent	25	481(0.2858122784954842)	595 (0.21)	461 (0.3)
bacteria	25	481(0.2858122784954842)	398 (0.31)	333 (0.43)
black	25	481(0.2858122784954842)	277 (0.45)	694 (0.2)
changes	25	126(1.0403566937235624)	124 (0.86)	120 (1.1)
correlates	25	481(0.2858122784954842)	595 (0.21)	361 (0.4)
diabetic	25	481(0.2858122784954842)	595 (0.21)	388 (0.36)
difficulties	25	481(0.2858122784954842)	451 (0.28)	694 (0.2)
disorders	25	131(0.9603292557448267)	124 (0.86)	149 (0.86)
functional	25	108(1.1661140962615753)	124 (0.86)	115 (1.2)
hemodialysis	25	481(0.2858122784954842)	329 (0.38)	584 (0.23)
homes	25	481(0.2858122784954842)	717 (0.17)	388 (0.36)
injury	25	481(0.2858122784954842)	520 (0.24)	526 (0.26)
insights	25	481(0.2858122784954842)	2359 (0.034)	816 (0.16)
intensive	25	481(0.2858122784954842)	398 (0.31)	584 (0.23)
interactions	25	481(0.2858122784954842)	451 (0.28)	361 (0.4)
kidney	25	481(0.2858122784954842)	595 (0.21)	461 (0.3)
larvae	25	362(0.37727220761403907)	124 (0.86)	584 (0.23)
longitudinal	25	118(1.08608665828284)	124 (0.86)	109 (1.3)
method	25	481(0.2858122784954842)	398 (0.31)	694 (0.2)
rural	25	162(0.7545444152280781)	124 (0.86)	149 (0.86)
saudi	25	481(0.2858122784954842)	1516 (0.069)	461 (0.3)
series	25	481(0.2858122784954842)	897 (0.14)	461 (0.3)
sheep	25	481(0.2858122784954842)	451 (0.28)	305 (0.46)
toddlers	25	481(0.2858122784954842)	595 (0.21)	280 (0.49)
arthritis	24	500(0.2743797873556648)	595 (0.21)	461 (0.3)
blood	24	142(0.8803018177660913)	131 (0.83)	205 (0.66)
cohort	24	40(2.572310506459358)	131 (0.83)	38 (3)
control	24	154(0.7888418886475363)	131 (0.83)	185 (0.73)
effectiveness	24	500(0.2743797873556648)	520 (0.24)	461 (0.3)
european	24	500(0.2743797873556648)	218 (0.55)	694 (0.2)
feeding-behavior	24	500(0.2743797873556648)	142 (0.79)	2548 (0.033)
flow	24	500(0.2743797873556648)	329 (0.38)	461 (0.3)
free	24	500(0.2743797873556648)	451 (0.28)	388 (0.36)
gastrointestinal	24	183(0.6973819595289814)	131 (0.83)	158 (0.82)

host	24	500(0.2743797873556648)	277 (0.45)	584 (0.23)
human	24	163(0.7431119240882588)	131 (0.83)	174 (0.76)
implications	24	192(0.6745169772493427)	131 (0.83)	185 (0.73)
india	24	500(0.2743797873556648)	451 (0.28)	526 (0.26)
infant	24	172(0.72024694180862)	131 (0.83)	217 (0.63)
influencing	24	500(0.2743797873556648)	1516 (0.069)	694 (0.2)
insulin	24	500(0.2743797873556648)	398 (0.31)	388 (0.36)
nervosa	24	500(0.2743797873556648)	329 (0.38)	584 (0.23)
occlusal	24	500(0.2743797873556648)	1121 (0.1)	422 (0.33)
preschool	24	168(0.7316794329484394)	131 (0.83)	194 (0.69)
problems	24	202(0.6516519949697039)	131 (0.83)	194 (0.69)
psychological	24	500(0.2743797873556648)	717 (0.17)	422 (0.33)
public	24	500(0.2743797873556648)	717 (0.17)	422 (0.33)
pulmonary	24	500(0.2743797873556648)	362 (0.34)	584 (0.23)
report	24	138(0.9145992911855493)	131 (0.83)	158 (0.82)
short-term	24	500(0.2743797873556648)	398 (0.31)	526 (0.26)
social	24	195(0.6630844861095233)	131 (0.83)	280 (0.49)
appetite	23	233(0.5487595747113296)	142 (0.79)	361 (0.4)
associations	23	77(1.554818795015434)	142 (0.79)	71 (1.8)
atherosclerosis	23	516(0.26294729621584545)	256 (0.48)	999 (0.13)
attending	23	516(0.26294729621584545)	520 (0.24)	584 (0.23)
behaviors	23	103(1.2004115696810334)	142 (0.79)	79 (1.6)
bone	23	179(0.7088144506688008)	142 (0.79)	228 (0.59)
characteristics	23	101(1.2347090431004915)	142 (0.79)	92 (1.5)
denture	23	516(0.26294729621584545)	451 (0.28)	1233 (0.1)
design	23	516(0.26294729621584545)	595 (0.21)	305 (0.46)
esophagitis	23	516(0.26294729621584545)	595 (0.21)	461 (0.3)
feeding-behavior	23	500(0.2743797873556648)	142 (0.79)	2548 (0.033)
forest	23	516(0.26294729621584545)	897 (0.14)	333 (0.43)
gut	23	516(0.26294729621584545)	1121 (0.1)	461 (0.3)
hormone	23	516(0.26294729621584545)	362 (0.34)	422 (0.33)
humans	23	516(0.26294729621584545)	277 (0.45)	816 (0.16)
improvement	23	516(0.26294729621584545)	595 (0.21)	816 (0.16)
increases	23	516(0.26294729621584545)	595 (0.21)	388 (0.36)
intervention	23	122(1.0517891848633818)	142 (0.79)	139 (1)
leptin	23	268(0.4801646278724134)	142 (0.79)	280 (0.49)
malignant	23	516(0.26294729621584545)	398 (0.31)	584 (0.23)
model	23	118(1.08608665828284)	142 (0.79)	145 (0.89)
normal	23	516(0.26294729621584545)	451 (0.28)	461 (0.3)
pain	23	516(0.26294729621584545)	1121 (0.1)	333 (0.43)
pandemic	23	516(0.26294729621584545)	#SAKNAS!	2548 (0.033)
physiological	23	516(0.26294729621584545)	398 (0.31)	584 (0.23)
preterm	23	516(0.26294729621584545)	398 (0.31)	999 (0.13)

products	23	516(0.26294729621584545)	595 (0.21)	584 (0.23)
sclerosis	23	141(0.8917343089059107)	142 (0.79)	139 (1)
sea	23	331(0.4115696810334972)	142 (0.79)	584 (0.23)
south	23	172(0.72024694180862)	142 (0.79)	134 (1)
subjective	23	516(0.26294729621584545)	717 (0.17)	526 (0.26)
teeth	23	93(1.3604664456385047)	142 (0.79)	86 (1.5)
banding	22	462(0.2972447696353035)	155 (0.76)	999 (0.13)
behavioural	22	535(0.2515148050760261)	717 (0.17)	461 (0.3)
central	22	261(0.49159711901223274)	155 (0.76)	305 (0.46)
characterization	22	535(0.2515148050760261)	595 (0.21)	422 (0.33)
cluster	22	535(0.2515148050760261)	1121 (0.1)	388 (0.36)
complete	22	535(0.2515148050760261)	398 (0.31)	1668 (0.066)
concentration	22	535(0.2515148050760261)	329 (0.38)	584 (0.23)
conditions	22	151(0.8002743797873557)	155 (0.76)	149 (0.86)
disparities	22	535(0.2515148050760261)	398 (0.31)	461 (0.3)
ecology	22	535(0.2515148050760261)	301 (0.41)	1668 (0.066)
esophagus	22	535(0.2515148050760261)	256 (0.48)	999 (0.13)
evidence	22	161(0.7659769063678976)	155 (0.76)	205 (0.66)
group	22	228(0.560192065851149)	155 (0.76)	333 (0.43)
inflammation	22	147(0.8345718532068138)	155 (0.76)	217 (0.63)
late	22	535(0.2515148050760261)	398 (0.31)	526 (0.26)
lipid	22	535(0.2515148050760261)	897 (0.14)	422 (0.33)
locally	22	535(0.2515148050760261)	451 (0.28)	461 (0.3)
mechanisms	22	535(0.2515148050760261)	398 (0.31)	584 (0.23)
overweight	22	131(0.9603292557448267)	155 (0.76)	97 (1.4)
production	22	535(0.2515148050760261)	520 (0.24)	584 (0.23)
prognosis	22	535(0.2515148050760261)	1516 (0.069)	584 (0.23)
program	22	159(0.777409397507717)	155 (0.76)	149 (0.86)
promotion	22	535(0.2515148050760261)	717 (0.17)	422 (0.33)
secondary	22	535(0.2515148050760261)	717 (0.17)	694 (0.2)
setting	22	535(0.2515148050760261)	595 (0.21)	422 (0.33)
subgingival	22	535(0.2515148050760261)	1121 (0.1)	816 (0.16)
sugars	22	535(0.2515148050760261)	520 (0.24)	422 (0.33)
supplements	22	535(0.2515148050760261)	520 (0.24)	584 (0.23)
third	22	535(0.2515148050760261)	329 (0.38)	584 (0.23)
tubes	22	535(0.2515148050760261)	277 (0.45)	1233 (0.1)
variables	22	535(0.2515148050760261)	520 (0.24)	461 (0.3)
vegetable	22	535(0.2515148050760261)	897 (0.14)	461 (0.3)
adaptation	21	559(0.2400823139362067)	595 (0.21)	584 (0.23)
all-cause	21	559(0.2400823139362067)	1121 (0.1)	816 (0.16)
anthropometric	21	559(0.2400823139362067)	451 (0.28)	1668 (0.066)
atrophy	21	559(0.2400823139362067)	520 (0.24)	999 (0.13)
beverages	21	559(0.2400823139362067)	1121 (0.1)	361 (0.4)

complications	21	179(0.7088144506688008)	163 (0.72)	174 (0.76)
deficiency	21	559(0.2400823139362067)	398 (0.31)	816 (0.16)
dry	21	559(0.2400823139362067)	595 (0.21)	422 (0.33)
edentulous	21	559(0.2400823139362067)	451 (0.28)	526 (0.26)
elders	21	559(0.2400823139362067)	329 (0.38)	526 (0.26)
facilities	21	559(0.2400823139362067)	1516 (0.069)	694 (0.2)
fasting	21	559(0.2400823139362067)	451 (0.28)	461 (0.3)
fusion	21	559(0.2400823139362067)	1516 (0.069)	584 (0.23)
general	21	172(0.72024694180862)	163 (0.72)	205 (0.66)
gingivitis	21	559(0.2400823139362067)	398 (0.31)	584 (0.23)
ingestion	21	559(0.2400823139362067)	329 (0.38)	584 (0.23)
issues	21	559(0.2400823139362067)	256 (0.48)	999 (0.13)
larval	21	559(0.2400823139362067)	329 (0.38)	584 (0.23)
mellitus	21	207(0.6402195038298846)	163 (0.72)	217 (0.63)
mortality	21	100(1.2461415342403108)	163 (0.72)	92 (1.5)
mucositis	21	559(0.2400823139362067)	595 (0.21)	422 (0.33)
mutans	21	559(0.2400823139362067)	451 (0.28)	526 (0.26)
neurological	21	559(0.2400823139362067)	717 (0.17)	584 (0.23)
nutrients	21	559(0.2400823139362067)	329 (0.38)	584 (0.23)
paediatric	21	559(0.2400823139362067)	362 (0.34)	694 (0.2)
pharyngeal	21	559(0.2400823139362067)	398 (0.31)	816 (0.16)
pilot	21	129(0.9717617468846461)	163 (0.72)	120 (1.1)
plaque	21	559(0.2400823139362067)	520 (0.24)	694 (0.2)
prey	21	559(0.2400823139362067)	329 (0.38)	694 (0.2)
profiles	21	559(0.2400823139362067)	897 (0.14)	422 (0.33)
reduce	21	559(0.2400823139362067)	520 (0.24)	816 (0.16)
rheumatoid	21	559(0.2400823139362067)	595 (0.21)	461 (0.3)
schools	21	559(0.2400823139362067)	451 (0.28)	694 (0.2)
seasonal	21	559(0.2400823139362067)	451 (0.28)	422 (0.33)
sex	21	559(0.2400823139362067)	897 (0.14)	584 (0.23)
sociodemographic	21	559(0.2400823139362067)	717 (0.17)	526 (0.26)
sources	21	559(0.2400823139362067)	595 (0.21)	388 (0.36)
squamous	21	559(0.2400823139362067)	1121 (0.1)	584 (0.23)
tobacco	21	559(0.2400823139362067)	595 (0.21)	584 (0.23)
uk	21	559(0.2400823139362067)	1121 (0.1)	361 (0.4)
unit	21	559(0.2400823139362067)	301 (0.41)	999 (0.13)
adherence	20	595(0.22864982279638732)	#SAKNAS!	461 (0.3)
affects	20	350(0.3887046987538585)	168 (0.69)	422 (0.33)
alcohol	20	595(0.22864982279638732)	595 (0.21)	422 (0.33)
als	20	595(0.22864982279638732)	451 (0.28)	816 (0.16)
atresia	20	595(0.22864982279638732)	595 (0.21)	461 (0.3)
australian	20	595(0.22864982279638732)	1121 (0.1)	305 (0.46)
cardiovascular	20	149(0.8117068709271751)	168 (0.69)	149 (0.86)

cattle	20	595(0.22864982279638732)	717 (0.17)	694 (0.2)
complementary	20	595(0.22864982279638732)	1516 (0.069)	333 (0.43)
consequences	20	595(0.22864982279638732)	451 (0.28)	584 (0.23)
database	20	595(0.22864982279638732)	2359 (0.034)	816 (0.16)
depression	20	595(0.22864982279638732)	2359 (0.034)	461 (0.3)
digestive	20	595(0.22864982279638732)	1121 (0.1)	388 (0.36)
diseases	20	115(1.1089516405624786)	168 (0.69)	92 (1.5)
diversity	20	595(0.22864982279638732)	1121 (0.1)	694 (0.2)
drinking	20	595(0.22864982279638732)	451 (0.28)	816 (0.16)
eosinophilic	20	595(0.22864982279638732)	897 (0.14)	461 (0.3)
follow-up	20	188(0.685949468389162)	168 (0.69)	228 (0.59)
gestational	20	595(0.22864982279638732)	595 (0.21)	584 (0.23)
ghrelin	20	439(0.30867726077512286)	168 (0.69)	816 (0.16)
grazing	20	595(0.22864982279638732)	520 (0.24)	816 (0.16)
groups	20	595(0.22864982279638732)	520 (0.24)	694 (0.2)
herbivore	20	595(0.22864982279638732)	329 (0.38)	584 (0.23)
hiatal	20	595(0.22864982279638732)	1516 (0.069)	526 (0.26)
independent	20	595(0.22864982279638732)	897 (0.14)	526 (0.26)
influences	20	595(0.22864982279638732)	451 (0.28)	526 (0.26)
lateral	20	163(0.7431119240882588)	168 (0.69)	145 (0.89)
local	20	595(0.22864982279638732)	451 (0.28)	584 (0.23)
mineral	20	595(0.22864982279638732)	520 (0.24)	694 (0.2)
multidisciplinary	20	595(0.22864982279638732)	1516 (0.069)	461 (0.3)
nitrogen	20	595(0.22864982279638732)	897 (0.14)	526 (0.26)
perceptions	20	595(0.22864982279638732)	451 (0.28)	694 (0.2)
plasma	20	268(0.4801646278724134)	168 (0.69)	305 (0.46)
polymorphism	20	595(0.22864982279638732)	520 (0.24)	333 (0.43)
predictors	20	168(0.7316794329484394)	168 (0.69)	251 (0.56)
preference	20	293(0.45729964559277464)	168 (0.69)	422 (0.33)
prognostic	20	595(0.22864982279638732)	897 (0.14)	694 (0.2)
rate	20	281(0.468732136732594)	168 (0.69)	280 (0.49)
rehabilitation	20	116(1.0975191494226593)	168 (0.69)	126 (1.1)
scale	20	595(0.22864982279638732)	1121 (0.1)	694 (0.2)
smoking	20	256(0.5030296101520522)	168 (0.69)	251 (0.56)
stents	20	595(0.22864982279638732)	329 (0.38)	694 (0.2)
strictures	20	595(0.22864982279638732)	362 (0.34)	584 (0.23)
system	20	202(0.6516519949697039)	168 (0.69)	228 (0.59)
systemic	20	131(0.9603292557448267)	168 (0.69)	120 (1.1)
term	20	595(0.22864982279638732)	897 (0.14)	333 (0.43)
test	20	595(0.22864982279638732)	451 (0.28)	694 (0.2)
variation	20	309(0.43443466331313596)	168 (0.69)	280 (0.49)
2011-2014	19	628(0.21721733165656798)	#SAKNAS!	526 (0.26)
3d	19	628(0.21721733165656798)	#SAKNAS!	#SAKNAS!

african	19	628(0.21721733165656798)	301 (0.41)	816 (0.16)
amino	19	628(0.21721733165656798)	595 (0.21)	999 (0.13)
antioxidant	19	628(0.21721733165656798)	1121 (0.1)	694 (0.2)
apnea	19	628(0.21721733165656798)	595 (0.21)	461 (0.3)
availability	19	628(0.21721733165656798)	520 (0.24)	816 (0.16)
brazil	19	237(0.5373270835715103)	183 (0.66)	305 (0.46)
brazilian	19	195(0.6630844861095233)	183 (0.66)	141 (0.92)
calves	19	628(0.21721733165656798)	897 (0.14)	694 (0.2)
center	19	628(0.21721733165656798)	897 (0.14)	584 (0.23)
chemoradiation	19	628(0.21721733165656798)	329 (0.38)	816 (0.16)
city	19	628(0.21721733165656798)	717 (0.17)	461 (0.3)
communities	19	628(0.21721733165656798)	520 (0.24)	584 (0.23)
concurrent	19	628(0.21721733165656798)	451 (0.28)	816 (0.16)
context	19	628(0.21721733165656798)	595 (0.21)	422 (0.33)
digestibility	19	628(0.21721733165656798)	1516 (0.069)	816 (0.16)
epidemiological	19	628(0.21721733165656798)	1516 (0.069)	461 (0.3)
evaluating	19	628(0.21721733165656798)	1121 (0.1)	422 (0.33)
foods	19	172(0.72024694180862)	183 (0.66)	280 (0.49)
gingivalis	19	628(0.21721733165656798)	520 (0.24)	526 (0.26)
global	19	628(0.21721733165656798)	897 (0.14)	526 (0.26)
hypothalamic	19	362(0.37727220761403907)	183 (0.66)	422 (0.33)
identification	19	628(0.21721733165656798)	451 (0.28)	999 (0.13)
implant	19	628(0.21721733165656798)	595 (0.21)	584 (0.23)
infection	19	168(0.7316794329484394)	183 (0.66)	145 (0.89)
infections	19	628(0.21721733165656798)	362 (0.34)	694 (0.2)
lactating	19	628(0.21721733165656798)	451 (0.28)	694 (0.2)
lesions	19	628(0.21721733165656798)	717 (0.17)	526 (0.26)
link	19	628(0.21721733165656798)	897 (0.14)	584 (0.23)
mendelian	19	628(0.21721733165656798)	#SAKNAS!	2548 (0.033)
meta-analysis	19	628(0.21721733165656798)	2359 (0.034)	816 (0.16)
metabolic	19	127(1.028924202583743)	183 (0.66)	79 (1.6)
mexican	19	628(0.21721733165656798)	451 (0.28)	584 (0.23)
neuropeptide	19	628(0.21721733165656798)	277 (0.45)	2548 (0.033)
nhanes	19	69(1.7377386532525438)	183 (0.66)	100 (1.4)
parenting	19	628(0.21721733165656798)	897 (0.14)	526 (0.26)
peg	19	410(0.33154224305476165)	183 (0.66)	694 (0.2)
physical	19	112(1.1318166228421174)	183 (0.66)	115 (1.2)
physiology	19	628(0.21721733165656798)	398 (0.31)	584 (0.23)
porphyromonas	19	628(0.21721733165656798)	520 (0.24)	526 (0.26)
position	19	628(0.21721733165656798)	398 (0.31)	584 (0.23)
prevention	19	218(0.6059220304104264)	183 (0.66)	361 (0.4)
progression	19	628(0.21721733165656798)	362 (0.34)	816 (0.16)
rheological	19	628(0.21721733165656798)	897 (0.14)	999 (0.13)

service	19	628(0.21721733165656798)	451 (0.28)	694 (0.2)
single-center	19	628(0.21721733165656798)	1121 (0.1)	816 (0.16)
taiwan	19	628(0.21721733165656798)	520 (0.24)	694 (0.2)
temporal	19	628(0.21721733165656798)	520 (0.24)	694 (0.2)
therapeutic	19	628(0.21721733165656798)	451 (0.28)	1668 (0.066)
thickness	19	628(0.21721733165656798)	717 (0.17)	584 (0.23)
version	19	628(0.21721733165656798)	1121 (0.1)	584 (0.23)
vitamin	19	143(0.8574368354864526)	183 (0.66)	185 (0.73)
western	19	628(0.21721733165656798)	717 (0.17)	584 (0.23)
access	18	671(0.2057848405167486)	595 (0.21)	526 (0.26)
airway	18	671(0.2057848405167486)	717 (0.17)	461 (0.3)
alterations	18	671(0.2057848405167486)	595 (0.21)	694 (0.2)
amyotrophic	18	195(0.6630844861095233)	194 (0.62)	174 (0.76)
artery	18	671(0.2057848405167486)	595 (0.21)	694 (0.2)
artificial	18	671(0.2057848405167486)	717 (0.17)	999 (0.13)
biomarkers	18	671(0.2057848405167486)	#SAKNAS!	422 (0.33)
calcium	18	671(0.2057848405167486)	451 (0.28)	526 (0.26)
carbohydrate	18	671(0.2057848405167486)	717 (0.17)	584 (0.23)
cariogenic	18	671(0.2057848405167486)	717 (0.17)	694 (0.2)
cases	18	671(0.2057848405167486)	520 (0.24)	1668 (0.066)
cholesterol	18	671(0.2057848405167486)	520 (0.24)	999 (0.13)
costs	18	671(0.2057848405167486)	595 (0.21)	694 (0.2)
criteria	18	671(0.2057848405167486)	2359 (0.034)	694 (0.2)
distribution	18	671(0.2057848405167486)	329 (0.38)	999 (0.13)
dysfunction	18	207(0.6402195038298846)	194 (0.62)	228 (0.59)
factor	18	195(0.6630844861095233)	194 (0.62)	194 (0.69)
female	18	319(0.4230021721733166)	194 (0.62)	422 (0.33)
improving	18	671(0.2057848405167486)	717 (0.17)	694 (0.2)
individual	18	671(0.2057848405167486)	520 (0.24)	816 (0.16)
infarction	18	671(0.2057848405167486)	717 (0.17)	526 (0.26)
juvenile	18	671(0.2057848405167486)	595 (0.21)	584 (0.23)
knhanes	18	671(0.2057848405167486)	#SAKNAS!	461 (0.3)
leaf	18	671(0.2057848405167486)	717 (0.17)	461 (0.3)
living	18	143(0.8574368354864526)	194 (0.62)	130 (1)
macronutrient	18	439(0.30867726077512286)	194 (0.62)	816 (0.16)
magnetic	18	671(0.2057848405167486)	2359 (0.034)	816 (0.16)
marine	18	671(0.2057848405167486)	520 (0.24)	694 (0.2)
meals	18	671(0.2057848405167486)	717 (0.17)	694 (0.2)
measurement	18	671(0.2057848405167486)	897 (0.14)	584 (0.23)
mexico	18	671(0.2057848405167486)	717 (0.17)	526 (0.26)
nasopharyngeal	18	671(0.2057848405167486)	897 (0.14)	816 (0.16)
nissen	18	671(0.2057848405167486)	451 (0.28)	816 (0.16)
onset	18	671(0.2057848405167486)	717 (0.17)	694 (0.2)

pathogens	18	671(0.2057848405167486)	595 (0.21)	584 (0.23)
randomization	18	671(0.2057848405167486)	#SAKNAS!	2548 (0.033)
reproductive	18	671(0.2057848405167486)	451 (0.28)	1233 (0.1)
requiring	18	671(0.2057848405167486)	2359 (0.034)	584 (0.23)
resection	18	671(0.2057848405167486)	451 (0.28)	1233 (0.1)
responses	18	281(0.468732136732594)	194 (0.62)	361 (0.4)
school	18	172(0.72024694180862)	194 (0.62)	174 (0.76)
southern	18	319(0.4230021721733166)	194 (0.62)	584 (0.23)
species	18	268(0.4801646278724134)	194 (0.62)	280 (0.49)
spectrum	18	671(0.2057848405167486)	1516 (0.069)	388 (0.36)
spinal	18	671(0.2057848405167486)	717 (0.17)	1233 (0.1)
state	18	261(0.49159711901223274)	194 (0.62)	264 (0.53)
stent	18	671(0.2057848405167486)	398 (0.31)	584 (0.23)
survival	18	214(0.6287870126900652)	194 (0.62)	185 (0.73)
tract	18	671(0.2057848405167486)	717 (0.17)	461 (0.3)
validity	18	671(0.2057848405167486)	897 (0.14)	526 (0.26)
workers	18	671(0.2057848405167486)	595 (0.21)	816 (0.16)
action	17	710(0.19435234937692925)	451 (0.28)	816 (0.16)
administration	17	710(0.19435234937692925)	451 (0.28)	1233 (0.1)
advice	17	710(0.19435234937692925)	595 (0.21)	999 (0.13)
approach	17	163(0.7431119240882588)	206 (0.59)	145 (0.89)
autism	17	710(0.19435234937692925)	2359 (0.034)	361 (0.4)
biofilm	17	710(0.19435234937692925)	1121 (0.1)	694 (0.2)
cells	17	710(0.19435234937692925)	451 (0.28)	1233 (0.1)
community-dwelling	17	104(1.188979078541214)	206 (0.59)	100 (1.4)
developmental	17	710(0.19435234937692925)	717 (0.17)	526 (0.26)
evolution	17	710(0.19435234937692925)	717 (0.17)	584 (0.23)
exposure	17	221(0.5830570481307876)	206 (0.59)	280 (0.49)
formula	17	710(0.19435234937692925)	595 (0.21)	816 (0.16)
gender	17	710(0.19435234937692925)	897 (0.14)	816 (0.16)
geriatric	17	249(0.5144621012918715)	206 (0.59)	305 (0.46)
heart	17	233(0.5487595747113296)	206 (0.59)	305 (0.46)
high-fat	17	710(0.19435234937692925)	595 (0.21)	422 (0.33)
insecurity	17	710(0.19435234937692925)	2359 (0.034)	816 (0.16)
italian	17	710(0.19435234937692925)	2359 (0.034)	584 (0.23)
mediating	17	710(0.19435234937692925)	#SAKNAS!	1668 (0.066)
metal	17	710(0.19435234937692925)	451 (0.28)	999 (0.13)
oil	17	710(0.19435234937692925)	717 (0.17)	584 (0.23)
parameters	17	202(0.6516519949697039)	206 (0.59)	228 (0.59)
perspective	17	710(0.19435234937692925)	451 (0.28)	999 (0.13)
placement	17	224(0.5716245569909684)	206 (0.59)	166 (0.79)
posterior	17	710(0.19435234937692925)	2359 (0.034)	584 (0.23)
predictor	17	710(0.19435234937692925)	1516 (0.069)	388 (0.36)

preventive		17	710(0.19435234937692925)	717 (0.17)	584 (0.23)
radiotherapy		17	188(0.685949468389162)	206 (0.59)	185 (0.73)
rat		17	462(0.2972447696353035)	206 (0.59)	694 (0.2)
reflux		17	237(0.5373270835715103)	206 (0.59)	305 (0.46)
safe		17	710(0.19435234937692925)	520 (0.24)	816 (0.16)
sensitivity		17	710(0.19435234937692925)	1516 (0.069)	461 (0.3)
short		17	710(0.19435234937692925)	1121 (0.1)	816 (0.16)
spine		17	710(0.19435234937692925)	520 (0.24)	816 (0.16)
stenosis		17	710(0.19435234937692925)	717 (0.17)	584 (0.23)
strategies		17	350(0.3887046987538585)	206 (0.59)	388 (0.36)
structural		17	710(0.19435234937692925)	1121 (0.1)	584 (0.23)
structure		17	710(0.19435234937692925)	451 (0.28)	999 (0.13)
surgical		17	268(0.4801646278724134)	206 (0.59)	305 (0.46)
surveys		17	710(0.19435234937692925)	595 (0.21)	816 (0.16)
swedish		17	710(0.19435234937692925)	362 (0.34)	816 (0.16)
tertiary		17	710(0.19435234937692925)	#SAKNAS!	526 (0.26)
tropical		17	710(0.19435234937692925)	2359 (0.034)	422 (0.33)
volume		17	710(0.19435234937692925)	1516 (0.069)	584 (0.23)
white		17	710(0.19435234937692925)	520 (0.24)	694 (0.2)
	2009	16	743(0.18291985823710985)	2359 (0.034)	422 (0.33)
active		16	743(0.18291985823710985)	520 (0.24)	999 (0.13)
adjustable		16	743(0.18291985823710985)	301 (0.41)	999 (0.13)
alters		16	743(0.18291985823710985)	520 (0.24)	694 (0.2)
arabia		16	743(0.18291985823710985)	1516 (0.069)	816 (0.16)
areas		16	743(0.18291985823710985)	1121 (0.1)	461 (0.3)
benefits		16	743(0.18291985823710985)	897 (0.14)	526 (0.26)
beverage		16	743(0.18291985823710985)	2359 (0.034)	526 (0.26)
biological		16	743(0.18291985823710985)	1516 (0.069)	999 (0.13)
cancers		16	743(0.18291985823710985)	897 (0.14)	461 (0.3)
chewing		16	219(0.594489539270607)	218 (0.55)	205 (0.66)
composition		16	154(0.7888418886475363)	218 (0.55)	141 (0.92)
c-reactive		16	261(0.49159711901223274)	218 (0.55)	264 (0.53)
dairy		16	188(0.685949468389162)	218 (0.55)	194 (0.69)
deer		16	743(0.18291985823710985)	451 (0.28)	999 (0.13)
dental-caries		16	743(0.18291985823710985)	218 (0.55)	#SAKNAS!
dental-caries		16	743(0.18291985823710985)	218 (0.55)	#SAKNAS!
dystrophy		16	743(0.18291985823710985)	897 (0.14)	999 (0.13)
electrical		16	743(0.18291985823710985)	897 (0.14)	461 (0.3)
european		16	500(0.2743797873556648)	218 (0.55)	694 (0.2)
expression		16	319(0.4230021721733166)	218 (0.55)	333 (0.43)
fat		16	261(0.49159711901223274)	218 (0.55)	228 (0.59)
females		16	743(0.18291985823710985)	717 (0.17)	584 (0.23)
fiber		16	743(0.18291985823710985)	897 (0.14)	816 (0.16)

fluoride	16	396(0.342974734194581)	218 (0.55)	584 (0.23)
german	16	743(0.18291985823710985)	717 (0.17)	526 (0.26)
gingival	16	261(0.49159711901223274)	218 (0.55)	228 (0.59)
goats	16	743(0.18291985823710985)	451 (0.28)	1233 (0.1)
healthcare	16	743(0.18291985823710985)	2359 (0.034)	1233 (0.1)
hospitalization	16	743(0.18291985823710985)	1516 (0.069)	584 (0.23)
iron	16	743(0.18291985823710985)	717 (0.17)	816 (0.16)
lactation	16	743(0.18291985823710985)	717 (0.17)	461 (0.3)
liver	16	743(0.18291985823710985)	1121 (0.1)	584 (0.23)
machine	16	743(0.18291985823710985)	#SAKNAS!	1668 (0.066)
mediation	16	743(0.18291985823710985)	2359 (0.034)	1233 (0.1)
motility	16	743(0.18291985823710985)	520 (0.24)	1233 (0.1)
nationally	16	743(0.18291985823710985)	897 (0.14)	694 (0.2)
neurogenic	16	743(0.18291985823710985)	451 (0.28)	1233 (0.1)
nucleus	16	743(0.18291985823710985)	362 (0.34)	1233 (0.1)
old	16	309(0.43443466331313596)	218 (0.55)	305 (0.46)
parent	16	743(0.18291985823710985)	1516 (0.069)	584 (0.23)
pathway	16	743(0.18291985823710985)	1121 (0.1)	461 (0.3)
pediatric	16	172(0.72024694180862)	218 (0.55)	185 (0.73)
positive	16	743(0.18291985823710985)	1121 (0.1)	694 (0.2)
pregnancy	16	362(0.37727220761403907)	218 (0.55)	694 (0.2)
prophylactic	16	743(0.18291985823710985)	2359 (0.034)	422 (0.33)
receptor	16	237(0.5373270835715103)	218 (0.55)	217 (0.63)
recurrent	16	743(0.18291985823710985)	897 (0.14)	816 (0.16)
region	16	743(0.18291985823710985)	897 (0.14)	999 (0.13)
severity	16	244(0.5258945924316909)	218 (0.55)	461 (0.3)
signaling	16	743(0.18291985823710985)	1516 (0.069)	461 (0.3)
size	16	388(0.3544072253344004)	218 (0.55)	526 (0.26)
skeletal	16	743(0.18291985823710985)	897 (0.14)	526 (0.26)
soft	16	743(0.18291985823710985)	595 (0.21)	694 (0.2)
special	16	743(0.18291985823710985)	717 (0.17)	1668 (0.066)
stages	16	743(0.18291985823710985)	1121 (0.1)	584 (0.23)
successful	16	743(0.18291985823710985)	398 (0.31)	999 (0.13)
sugar	16	183(0.6973819595289814)	218 (0.55)	134 (1)
sugar-sweetened	16	743(0.18291985823710985)	2359 (0.034)	526 (0.26)
texture-modified	16	743(0.18291985823710985)	2359 (0.034)	1233 (0.1)
thai	16	743(0.18291985823710985)	717 (0.17)	526 (0.26)
urban	16	331(0.4115696810334972)	218 (0.55)	264 (0.53)
2010	15	787(0.1714873670972905)	1121 (0.1)	461 (0.3)
12-year-old	15	787(0.1714873670972905)	520 (0.24)	694 (0.2)
5-year	15	787(0.1714873670972905)	2359 (0.034)	526 (0.26)
acid	15	228(0.560192065851149)	236 (0.52)	264 (0.53)
across	15	787(0.1714873670972905)	1121 (0.1)	999 (0.13)

antibodies	15	787(0.1714873670972905)	1121 (0.1)	584 (0.23)
aphid	15	787(0.1714873670972905)	1121 (0.1)	816 (0.16)
aspiration	15	179(0.7088144506688008)	236 (0.52)	174 (0.76)
assess	15	787(0.1714873670972905)	897 (0.14)	584 (0.23)
birth	15	233(0.5487595747113296)	236 (0.52)	251 (0.56)
breast	15	787(0.1714873670972905)	1121 (0.1)	584 (0.23)
bypass	15	214(0.6287870126900652)	236 (0.52)	166 (0.79)
carcinoma	15	221(0.5830570481307876)	236 (0.52)	280 (0.49)
cardiometabolic	15	787(0.1714873670972905)	2359 (0.034)	694 (0.2)
causal	15	787(0.1714873670972905)	2359 (0.034)	2548 (0.033)
cause	15	787(0.1714873670972905)	717 (0.17)	1233 (0.1)
causes	15	787(0.1714873670972905)	595 (0.21)	816 (0.16)
classification	15	787(0.1714873670972905)	595 (0.21)	1668 (0.066)
community	15	202(0.6516519949697039)	236 (0.52)	166 (0.79)
comparing	15	787(0.1714873670972905)	520 (0.24)	999 (0.13)
coronary	15	396(0.342974734194581)	236 (0.52)	461 (0.3)
crevicular	15	787(0.1714873670972905)	520 (0.24)	694 (0.2)
dentists	15	787(0.1714873670972905)	595 (0.21)	816 (0.16)
dentition	15	249(0.5144621012918715)	236 (0.52)	280 (0.49)
discectomy	15	787(0.1714873670972905)	1516 (0.069)	816 (0.16)
eat	15	787(0.1714873670972905)	1516 (0.069)	816 (0.16)
education	15	237(0.5373270835715103)	236 (0.52)	361 (0.4)
essential	15	787(0.1714873670972905)	1121 (0.1)	1233 (0.1)
expenditure	15	787(0.1714873670972905)	595 (0.21)	1233 (0.1)
features	15	787(0.1714873670972905)	1516 (0.069)	816 (0.16)
fish	15	410(0.33154224305476165)	236 (0.52)	526 (0.26)
fitness	15	787(0.1714873670972905)	897 (0.14)	694 (0.2)
flap	15	787(0.1714873670972905)	595 (0.21)	2548 (0.033)
frequency	15	268(0.4801646278724134)	236 (0.52)	264 (0.53)
gastroesophageal	15	331(0.4115696810334972)	236 (0.52)	422 (0.33)
giant	15	787(0.1714873670972905)	1121 (0.1)	461 (0.3)
glycemic	15	787(0.1714873670972905)	1516 (0.069)	999 (0.13)
improve	15	319(0.4230021721733166)	236 (0.52)	361 (0.4)
indices	15	787(0.1714873670972905)	1516 (0.069)	816 (0.16)
infancy	15	787(0.1714873670972905)	717 (0.17)	694 (0.2)
international	15	787(0.1714873670972905)	897 (0.14)	1668 (0.066)
lead	15	787(0.1714873670972905)	595 (0.21)	584 (0.23)
maintenance	15	787(0.1714873670972905)	329 (0.38)	1668 (0.066)
medicine	15	787(0.1714873670972905)	1516 (0.069)	694 (0.2)
models	15	787(0.1714873670972905)	595 (0.21)	694 (0.2)
morbid	15	787(0.1714873670972905)	451 (0.28)	816 (0.16)
nasogastric	15	396(0.342974734194581)	236 (0.52)	584 (0.23)
north	15	787(0.1714873670972905)	398 (0.31)	1233 (0.1)

obstructive	15	331(0.4115696810334972)	236 (0.52)	305 (0.46)
oesophageal	15	388(0.3544072253344004)	236 (0.52)	388 (0.36)
outpatient	15	787(0.1714873670972905)	1516 (0.069)	694 (0.2)
parents	15	281(0.468732136732594)	236 (0.52)	333 (0.43)
poststroke	15	787(0.1714873670972905)	2359 (0.034)	999 (0.13)
presence	15	787(0.1714873670972905)	897 (0.14)	584 (0.23)
prevent	15	787(0.1714873670972905)	897 (0.14)	816 (0.16)
printing	15	787(0.1714873670972905)	#SAKNAS!	#SAKNAS!
procedure	15	787(0.1714873670972905)	595 (0.21)	694 (0.2)
propensity	15	787(0.1714873670972905)	2359 (0.034)	584 (0.23)
rates	15	787(0.1714873670972905)	520 (0.24)	816 (0.16)
reduces	15	350(0.3887046987538585)	236 (0.52)	280 (0.49)
schoolchildren	15	331(0.4115696810334972)	236 (0.52)	422 (0.33)
serotonin	15	787(0.1714873670972905)	301 (0.41)	1668 (0.066)
specific	15	787(0.1714873670972905)	398 (0.31)	2548 (0.033)
stricture	15	787(0.1714873670972905)	520 (0.24)	584 (0.23)
sweden	15	787(0.1714873670972905)	362 (0.34)	1668 (0.066)
systems	15	787(0.1714873670972905)	897 (0.14)	1668 (0.066)
time	15	256(0.5030296101520522)	236 (0.52)	361 (0.4)
toxicity	15	787(0.1714873670972905)	451 (0.28)	999 (0.13)
wic	15	787(0.1714873670972905)	520 (0.24)	1233 (0.1)
abdominal	14	836(0.16005487595747114)	717 (0.17)	1233 (0.1)
american	14	244(0.5258945924316909)	256 (0.48)	305 (0.46)
aspects	14	462(0.2972447696353035)	256 (0.48)	816 (0.16)
asthma	14	836(0.16005487595747114)	1121 (0.1)	694 (0.2)
atherosclerosis	14	516(0.26294729621584545)	256 (0.48)	999 (0.13)
breakfast	14	836(0.16005487595747114)	1121 (0.1)	816 (0.16)
capacity	14	836(0.16005487595747114)	1121 (0.1)	816 (0.16)
case-control	14	183(0.6973819595289814)	256 (0.48)	158 (0.82)
cavity	14	836(0.16005487595747114)	717 (0.17)	999 (0.13)
cerebral	14	233(0.5487595747113296)	256 (0.48)	251 (0.56)
challenge	14	836(0.16005487595747114)	1121 (0.1)	694 (0.2)
challenges	14	836(0.16005487595747114)	717 (0.17)	816 (0.16)
concentrations	14	319(0.4230021721733166)	256 (0.48)	228 (0.59)
considerations	14	836(0.16005487595747114)	717 (0.17)	816 (0.16)
continuous	14	836(0.16005487595747114)	717 (0.17)	694 (0.2)
contribution	14	836(0.16005487595747114)	398 (0.31)	1233 (0.1)
conventional	14	836(0.16005487595747114)	897 (0.14)	1668 (0.066)
cows	14	224(0.5716245569909684)	256 (0.48)	194 (0.69)
cricopharyngeal	14	836(0.16005487595747114)	717 (0.17)	816 (0.16)
deficiencies	14	836(0.16005487595747114)	398 (0.31)	1233 (0.1)
density	14	377(0.3658397164742197)	256 (0.48)	461 (0.3)
dentistry	14	836(0.16005487595747114)	520 (0.24)	1233 (0.1)

determinants	14	221(0.5830570481307876)	256 (0.48)	174 (0.76)
developing	14	836(0.16005487595747114)	717 (0.17)	816 (0.16)
differential	14	836(0.16005487595747114)	520 (0.24)	999 (0.13)
difficulty	14	836(0.16005487595747114)	2359 (0.034)	999 (0.13)
disabilities	14	836(0.16005487595747114)	897 (0.14)	816 (0.16)
effective	14	836(0.16005487595747114)	897 (0.14)	694 (0.2)
esophagus	14	535(0.2515148050760261)	256 (0.48)	999 (0.13)
ethiopia	14	836(0.16005487595747114)	2359 (0.034)	584 (0.23)
experiences	14	836(0.16005487595747114)	1516 (0.069)	999 (0.13)
families	14	836(0.16005487595747114)	595 (0.21)	999 (0.13)
force	14	836(0.16005487595747114)	1121 (0.1)	816 (0.16)
fruit	14	377(0.3658397164742197)	256 (0.48)	461 (0.3)
fundoplication	14	309(0.43443466331313596)	256 (0.48)	361 (0.4)
gland	14	836(0.16005487595747114)	717 (0.17)	816 (0.16)
hospital	14	149(0.8117068709271751)	256 (0.48)	158 (0.82)
impacts	14	836(0.16005487595747114)	1516 (0.069)	461 (0.3)
implementation	14	836(0.16005487595747114)	1516 (0.069)	694 (0.2)
independently	14	836(0.16005487595747114)	595 (0.21)	694 (0.2)
inflammatory	14	207(0.6402195038298846)	256 (0.48)	217 (0.63)
insect	14	836(0.16005487595747114)	398 (0.31)	1668 (0.066)
insurance	14	836(0.16005487595747114)	1121 (0.1)	584 (0.23)
issues	14	559(0.2400823139362067)	256 (0.48)	999 (0.13)
lung	14	836(0.16005487595747114)	1121 (0.1)	694 (0.2)
male	14	293(0.45729964559277464)	256 (0.48)	228 (0.59)
medical	14	237(0.5373270835715103)	256 (0.48)	305 (0.46)
metabolites	14	836(0.16005487595747114)	2359 (0.034)	1668 (0.066)
motor	14	439(0.30867726077512286)	256 (0.48)	461 (0.3)
neoadjuvant	14	836(0.16005487595747114)	595 (0.21)	694 (0.2)
neuromuscular	14	836(0.16005487595747114)	897 (0.14)	461 (0.3)
neurons	14	836(0.16005487595747114)	1516 (0.069)	584 (0.23)
obstruction	14	836(0.16005487595747114)	717 (0.17)	816 (0.16)
offspring	14	836(0.16005487595747114)	897 (0.14)	694 (0.2)
oxidative	14	836(0.16005487595747114)	2359 (0.034)	461 (0.3)
partial	14	836(0.16005487595747114)	520 (0.24)	1668 (0.066)
perioperative	14	836(0.16005487595747114)	1121 (0.1)	1668 (0.066)
polymorphisms	14	836(0.16005487595747114)	520 (0.24)	999 (0.13)
postmenopausal	14	342(0.40013718989367786)	256 (0.48)	280 (0.49)
prior	14	836(0.16005487595747114)	1121 (0.1)	816 (0.16)
professionals	14	836(0.16005487595747114)	717 (0.17)	2548 (0.033)
programme	14	836(0.16005487595747114)	717 (0.17)	816 (0.16)
quantitative	14	836(0.16005487595747114)	717 (0.17)	1233 (0.1)
reliability	14	836(0.16005487595747114)	897 (0.14)	694 (0.2)
renal	14	836(0.16005487595747114)	897 (0.14)	816 (0.16)

reward		14	836(0.16005487595747114)	897 (0.14)	461 (0.3)
root		14	836(0.16005487595747114)	1121 (0.1)	584 (0.23)
secretion		14	836(0.16005487595747114)	897 (0.14)	584 (0.23)
support		14	151(0.8002743797873557)	256 (0.48)	166 (0.79)
surface		14	836(0.16005487595747114)	897 (0.14)	999 (0.13)
surveillance		14	836(0.16005487595747114)	520 (0.24)	694 (0.2)
symptom		14	836(0.16005487595747114)	595 (0.21)	1668 (0.066)
toothbrushing		14	836(0.16005487595747114)	1121 (0.1)	584 (0.23)
toxin		14	836(0.16005487595747114)	451 (0.28)	816 (0.16)
traditional		14	836(0.16005487595747114)	1121 (0.1)	816 (0.16)
transplantation		14	836(0.16005487595747114)	1516 (0.069)	694 (0.2)
trophic		14	836(0.16005487595747114)	595 (0.21)	999 (0.13)
users		14	836(0.16005487595747114)	717 (0.17)	584 (0.23)
variability		14	836(0.16005487595747114)	717 (0.17)	694 (0.2)
wild		14	427(0.3201097519149423)	256 (0.48)	584 (0.23)
zinc		14	836(0.16005487595747114)	1121 (0.1)	1668 (0.066)
	2012	13	896(0.14862238481765175)	#SAKNAS!	361 (0.4)
	2018	13	896(0.14862238481765175)	#SAKNAS!	816 (0.16)
achalasia		13	305(0.4458671544529553)	277 (0.45)	205 (0.66)
africa		13	896(0.14862238481765175)	362 (0.34)	1668 (0.066)
animal		13	896(0.14862238481765175)	897 (0.14)	1668 (0.066)
anorexia		13	388(0.3544072253344004)	277 (0.45)	526 (0.26)
attachment		13	896(0.14862238481765175)	717 (0.17)	816 (0.16)
augmentation		13	896(0.14862238481765175)	2359 (0.034)	694 (0.2)
australia		13	896(0.14862238481765175)	717 (0.17)	1233 (0.1)
bangladesh		13	896(0.14862238481765175)	1121 (0.1)	816 (0.16)
black		13	481(0.2858122784954842)	277 (0.45)	694 (0.2)
cardiac		13	896(0.14862238481765175)	897 (0.14)	694 (0.2)
cervical		13	163(0.7431119240882588)	277 (0.45)	141 (0.92)
chemoradiotherapy		13	362(0.37727220761403907)	277 (0.45)	305 (0.46)
clinic		13	896(0.14862238481765175)	897 (0.14)	1233 (0.1)
complex		13	896(0.14862238481765175)	1516 (0.069)	999 (0.13)
congenital		13	896(0.14862238481765175)	1121 (0.1)	816 (0.16)
content		13	342(0.40013718989367786)	277 (0.45)	388 (0.36)
corn		13	896(0.14862238481765175)	2359 (0.034)	584 (0.23)
countries		13	896(0.14862238481765175)	595 (0.21)	999 (0.13)
crab		13	896(0.14862238481765175)	1516 (0.069)	526 (0.26)
cross-sectional		13	22(4.744483823025037)	277 (0.45)	26 (4.1)
death		13	896(0.14862238481765175)	520 (0.24)	1233 (0.1)
dentures		13	896(0.14862238481765175)	717 (0.17)	816 (0.16)
diagnostic		13	896(0.14862238481765175)	1516 (0.069)	999 (0.13)
drinks		13	896(0.14862238481765175)	717 (0.17)	816 (0.16)
edentulism		13	410(0.33154224305476165)	277 (0.45)	526 (0.26)

enamel	13 896(0.14862238481765175)	717 (0.17)	1668 (0.066)
epidemiology	13 896(0.14862238481765175)	595 (0.21)	999 (0.13)
epidermolysis	13 896(0.14862238481765175)	451 (0.28)	1233 (0.1)
events	13 896(0.14862238481765175)	595 (0.21)	694 (0.2)
examining	13 896(0.14862238481765175)	717 (0.17)	1233 (0.1)
family	13 331(0.4115696810334972)	277 (0.45)	526 (0.26)
fatty	13 342(0.40013718989367786)	277 (0.45)	461 (0.3)
functions	13 896(0.14862238481765175)	897 (0.14)	1233 (0.1)
gene	13 319(0.4230021721733166)	277 (0.45)	194 (0.69)
generalist	13 896(0.14862238481765175)	595 (0.21)	816 (0.16)
genes	13 896(0.14862238481765175)	897 (0.14)	999 (0.13)
germany	13 896(0.14862238481765175)	595 (0.21)	1233 (0.1)
guidelines	13 342(0.40013718989367786)	277 (0.45)	333 (0.43)
honey	13 896(0.14862238481765175)	717 (0.17)	584 (0.23)
host	13 500(0.2743797873556648)	277 (0.45)	584 (0.23)
humans	13 516(0.26294729621584545)	277 (0.45)	816 (0.16)
incidence	13 305(0.4458671544529553)	277 (0.45)	461 (0.3)
incident	13 896(0.14862238481765175)	1121 (0.1)	816 (0.16)
invasive	13 896(0.14862238481765175)	897 (0.14)	584 (0.23)
italy	13 896(0.14862238481765175)	897 (0.14)	694 (0.2)
laboratory	13 896(0.14862238481765175)	595 (0.21)	1233 (0.1)
laryngeal	13 896(0.14862238481765175)	595 (0.21)	1233 (0.1)
macaques	13 896(0.14862238481765175)	897 (0.14)	526 (0.26)
mandibular	13 896(0.14862238481765175)	897 (0.14)	1233 (0.1)
mastication	13 896(0.14862238481765175)	1121 (0.1)	694 (0.2)
measure	13 896(0.14862238481765175)	897 (0.14)	694 (0.2)
microbial	13 896(0.14862238481765175)	717 (0.17)	1233 (0.1)
modification	13 896(0.14862238481765175)	1121 (0.1)	816 (0.16)
monkeys	13 896(0.14862238481765175)	1121 (0.1)	526 (0.26)
mothers	13 293(0.45729964559277464)	277 (0.45)	217 (0.63)
neuropeptide	13 628(0.21721733165656798)	277 (0.45)	2548 (0.033)
newly	13 896(0.14862238481765175)	897 (0.14)	1233 (0.1)
palliative	13 362(0.37727220761403907)	277 (0.45)	388 (0.36)
paraesophageal	13 896(0.14862238481765175)	520 (0.24)	999 (0.13)
peripheral	13 896(0.14862238481765175)	897 (0.14)	584 (0.23)
plants	13 896(0.14862238481765175)	1121 (0.1)	526 (0.26)
preliminary	13 319(0.4230021721733166)	277 (0.45)	461 (0.3)
preschoolers	13 896(0.14862238481765175)	717 (0.17)	816 (0.16)
process	13 896(0.14862238481765175)	897 (0.14)	461 (0.3)
programs	13 896(0.14862238481765175)	2359 (0.034)	694 (0.2)
prosthesis	13 896(0.14862238481765175)	1121 (0.1)	999 (0.13)
receptors	13 896(0.14862238481765175)	1516 (0.069)	694 (0.2)
reduction	13 896(0.14862238481765175)	1121 (0.1)	694 (0.2)

replacement	13	896(0.14862238481765175)	717 (0.17)	694 (0.2)
restriction	13	439(0.30867726077512286)	277 (0.45)	388 (0.36)
risks	13	896(0.14862238481765175)	717 (0.17)	816 (0.16)
selective	13	896(0.14862238481765175)	520 (0.24)	816 (0.16)
self-expanding	13	896(0.14862238481765175)	451 (0.28)	999 (0.13)
simple	13	896(0.14862238481765175)	717 (0.17)	816 (0.16)
sleep	13	195(0.6630844861095233)	277 (0.45)	158 (0.82)
socio-economic	13	896(0.14862238481765175)	451 (0.28)	1233 (0.1)
spain	13	896(0.14862238481765175)	595 (0.21)	1233 (0.1)
speech	13	896(0.14862238481765175)	1121 (0.1)	1233 (0.1)
staff	13	896(0.14862238481765175)	897 (0.14)	584 (0.23)
streptococci	13	896(0.14862238481765175)	595 (0.21)	999 (0.13)
students	13	207(0.6402195038298846)	277 (0.45)	264 (0.53)
sucrose	13	896(0.14862238481765175)	897 (0.14)	526 (0.26)
temperature	13	896(0.14862238481765175)	1121 (0.1)	694 (0.2)
transition	13	896(0.14862238481765175)	717 (0.17)	816 (0.16)
tubes	13	535(0.2515148050760261)	277 (0.45)	1233 (0.1)
update	13	896(0.14862238481765175)	897 (0.14)	694 (0.2)
vascular	13	896(0.14862238481765175)	451 (0.28)	999 (0.13)
versus	13	207(0.6402195038298846)	277 (0.45)	185 (0.73)
vertical	13	896(0.14862238481765175)	595 (0.21)	1233 (0.1)
videofluoroscopic	13	896(0.14862238481765175)	1121 (0.1)	694 (0.2)
well-being	13	896(0.14862238481765175)	2359 (0.034)	694 (0.2)
whole	13	896(0.14862238481765175)	595 (0.21)	999 (0.13)
2013-2014	12	966(0.1371898936778324)	#SAKNAS!	816 (0.16)
acceptability	12	966(0.1371898936778324)	1516 (0.069)	816 (0.16)
adjustable	12	743(0.18291985823710985)	301 (0.41)	999 (0.13)
adverse	12	966(0.1371898936778324)	595 (0.21)	2548 (0.033)
african	12	628(0.21721733165656798)	301 (0.41)	816 (0.16)
albumin	12	966(0.1371898936778324)	717 (0.17)	999 (0.13)
alternative	12	966(0.1371898936778324)	897 (0.14)	1233 (0.1)
alveolar	12	966(0.1371898936778324)	897 (0.14)	999 (0.13)
banded	12	966(0.1371898936778324)	897 (0.14)	999 (0.13)
behaviours	12	268(0.4801646278724134)	301 (0.41)	228 (0.59)
brain	12	281(0.468732136732594)	301 (0.41)	228 (0.59)
bullosa	12	966(0.1371898936778324)	520 (0.24)	1233 (0.1)
circumference	12	966(0.1371898936778324)	897 (0.14)	999 (0.13)
commercial	12	966(0.1371898936778324)	1516 (0.069)	694 (0.2)
components	12	966(0.1371898936778324)	897 (0.14)	999 (0.13)
correlation	12	268(0.4801646278724134)	301 (0.41)	228 (0.59)
course	12	966(0.1371898936778324)	1516 (0.069)	999 (0.13)
decay	12	966(0.1371898936778324)	897 (0.14)	2548 (0.033)
dementia	12	228(0.560192065851149)	301 (0.41)	185 (0.73)

descriptive	12	966(0.1371898936778324)	1121 (0.1)	816 (0.16)
dilation	12	966(0.1371898936778324)	897 (0.14)	1233 (0.1)
diptera	12	966(0.1371898936778324)	398 (0.31)	1233 (0.1)
disability	12	966(0.1371898936778324)	1121 (0.1)	999 (0.13)
ecological	12	966(0.1371898936778324)	897 (0.14)	1233 (0.1)
ecology	12	535(0.2515148050760261)	301 (0.41)	1668 (0.066)
exploration	12	966(0.1371898936778324)	1121 (0.1)	1233 (0.1)
goat	12	966(0.1371898936778324)	595 (0.21)	1233 (0.1)
growing	12	966(0.1371898936778324)	595 (0.21)	1668 (0.066)
guideline	12	966(0.1371898936778324)	897 (0.14)	1233 (0.1)
hemiptera	12	966(0.1371898936778324)	1516 (0.069)	584 (0.23)
income	12	966(0.1371898936778324)	717 (0.17)	999 (0.13)
indicators	12	244(0.5258945924316909)	301 (0.41)	264 (0.53)
indigenous	12	966(0.1371898936778324)	2359 (0.034)	816 (0.16)
inequalities	12	966(0.1371898936778324)	1516 (0.069)	694 (0.2)
ingestive	12	966(0.1371898936778324)	897 (0.14)	1668 (0.066)
intestinal	12	966(0.1371898936778324)	595 (0.21)	2548 (0.033)
investigating	12	966(0.1371898936778324)	#SAKNAS!	694 (0.2)
liquid	12	966(0.1371898936778324)	897 (0.14)	1233 (0.1)
markers	12	309(0.43443466331313596)	301 (0.41)	264 (0.53)
mental	12	966(0.1371898936778324)	2359 (0.034)	999 (0.13)
metabolism	12	293(0.45729964559277464)	301 (0.41)	361 (0.4)
modeling	12	966(0.1371898936778324)	1516 (0.069)	999 (0.13)
modulation	12	966(0.1371898936778324)	897 (0.14)	816 (0.16)
morbidity	12	966(0.1371898936778324)	451 (0.28)	2548 (0.033)
morbidly	12	966(0.1371898936778324)	520 (0.24)	1668 (0.066)
myocardial	12	966(0.1371898936778324)	1121 (0.1)	584 (0.23)
natural	12	309(0.43443466331313596)	301 (0.41)	228 (0.59)
needs	12	309(0.43443466331313596)	301 (0.41)	584 (0.23)
nursing	12	138(0.9145992911855493)	301 (0.41)	130 (1)
occurrence	12	966(0.1371898936778324)	1516 (0.069)	816 (0.16)
olfactory	12	966(0.1371898936778324)	1121 (0.1)	584 (0.23)
overview	12	966(0.1371898936778324)	717 (0.17)	1233 (0.1)
palatability	12	966(0.1371898936778324)	897 (0.14)	694 (0.2)
palliation	12	966(0.1371898936778324)	595 (0.21)	816 (0.16)
parkinson	12	249(0.5144621012918715)	301 (0.41)	264 (0.53)
permanent	12	462(0.2972447696353035)	301 (0.41)	526 (0.26)
perspectives	12	966(0.1371898936778324)	897 (0.14)	694 (0.2)
ph	12	966(0.1371898936778324)	1121 (0.1)	816 (0.16)
pigs	12	439(0.30867726077512286)	301 (0.41)	694 (0.2)
preferences	12	427(0.3201097519149423)	301 (0.41)	305 (0.46)
preparation	12	966(0.1371898936778324)	1516 (0.069)	999 (0.13)
pre-school	12	966(0.1371898936778324)	717 (0.17)	999 (0.13)

prevalent	12	966(0.1371898936778324)	1516 (0.069)	694 (0.2)
promote	12	966(0.1371898936778324)	1121 (0.1)	816 (0.16)
protective	12	966(0.1371898936778324)	2359 (0.034)	816 (0.16)
radiation	12	427(0.3201097519149423)	301 (0.41)	461 (0.3)
ratio	12	966(0.1371898936778324)	1121 (0.1)	999 (0.13)
receiving	12	293(0.45729964559277464)	301 (0.41)	388 (0.36)
reconstruction	12	309(0.43443466331313596)	301 (0.41)	388 (0.36)
reducing	12	966(0.1371898936778324)	520 (0.24)	999 (0.13)
regulates	12	966(0.1371898936778324)	#SAKNAS!	816 (0.16)
residents	12	168(0.7316794329484394)	301 (0.41)	134 (1)
retrospective	12	112(1.1318166228421174)	301 (0.41)	134 (1)
selectivity	12	966(0.1371898936778324)	897 (0.14)	694 (0.2)
self-selection	12	966(0.1371898936778324)	451 (0.28)	999 (0.13)
serotonin	12	787(0.1714873670972905)	301 (0.41)	1668 (0.066)
significance	12	966(0.1371898936778324)	520 (0.24)	1233 (0.1)
spontaneous	12	966(0.1371898936778324)	520 (0.24)	999 (0.13)
stable	12	966(0.1371898936778324)	717 (0.17)	694 (0.2)
subacute	12	966(0.1371898936778324)	1516 (0.069)	816 (0.16)
swallow	12	377(0.3658397164742197)	301 (0.41)	388 (0.36)
tilapia	12	966(0.1371898936778324)	717 (0.17)	1233 (0.1)
turkey	12	966(0.1371898936778324)	897 (0.14)	1668 (0.066)
unit	12	559(0.2400823139362067)	301 (0.41)	999 (0.13)
upper	12	331(0.4115696810334972)	301 (0.41)	305 (0.46)
utilization	12	427(0.3201097519149423)	301 (0.41)	461 (0.3)
various	12	966(0.1371898936778324)	717 (0.17)	1668 (0.066)
virus	12	966(0.1371898936778324)	2359 (0.034)	999 (0.13)
water	12	268(0.4801646278724134)	301 (0.41)	228 (0.59)
acids	11	439(0.30867726077512286)	329 (0.38)	816 (0.16)
ad	11	1027(0.12575740253801304)	1121 (0.1)	1668 (0.066)
adiposity	11	481(0.2858122784954842)	329 (0.38)	461 (0.3)
admission	11	1027(0.12575740253801304)	1516 (0.069)	999 (0.13)
aging	11	331(0.4115696810334972)	329 (0.38)	584 (0.23)
algorithm	11	1027(0.12575740253801304)	595 (0.21)	1668 (0.066)
anesthesia	11	1027(0.12575740253801304)	1121 (0.1)	999 (0.13)
ant	11	1027(0.12575740253801304)	717 (0.17)	1233 (0.1)
antireflux	11	1027(0.12575740253801304)	717 (0.17)	816 (0.16)
arterial	11	1027(0.12575740253801304)	#SAKNAS!	999 (0.13)
asian	11	1027(0.12575740253801304)	2359 (0.034)	1233 (0.1)
balance	11	396(0.342974734194581)	329 (0.38)	333 (0.43)
barriers	11	1027(0.12575740253801304)	1121 (0.1)	1233 (0.1)
baseline	11	1027(0.12575740253801304)	897 (0.14)	816 (0.16)
beef	11	1027(0.12575740253801304)	1516 (0.069)	1668 (0.066)
beetle	11	1027(0.12575740253801304)	595 (0.21)	999 (0.13)

behavioral	11	342(0.40013718989367786)	329 (0.38)	280 (0.49)
beyond	11	1027(0.12575740253801304)	1121 (0.1)	999 (0.13)
birds	11	1027(0.12575740253801304)	398 (0.31)	1668 (0.066)
botulinum	11	1027(0.12575740253801304)	520 (0.24)	999 (0.13)
brown	11	1027(0.12575740253801304)	897 (0.14)	999 (0.13)
captive	11	1027(0.12575740253801304)	595 (0.21)	999 (0.13)
change	11	268(0.4801646278724134)	329 (0.38)	194 (0.69)
chemoradiation	11	628(0.21721733165656798)	329 (0.38)	816 (0.16)
cleaning	11	1027(0.12575740253801304)	#SAKNAS!	816 (0.16)
cognitive	11	214(0.6287870126900652)	329 (0.38)	174 (0.76)
complication	11	1027(0.12575740253801304)	717 (0.17)	1668 (0.066)
concentration	11	535(0.2515148050760261)	329 (0.38)	584 (0.23)
condition	11	244(0.5258945924316909)	329 (0.38)	251 (0.56)
containing	11	1027(0.12575740253801304)	717 (0.17)	1668 (0.066)
coverage	11	1027(0.12575740253801304)	2359 (0.034)	694 (0.2)
cross	11	1027(0.12575740253801304)	1121 (0.1)	1233 (0.1)
current	11	377(0.3658397164742197)	329 (0.38)	333 (0.43)
cytokines	11	1027(0.12575740253801304)	1516 (0.069)	999 (0.13)
decline	11	1027(0.12575740253801304)	#SAKNAS!	999 (0.13)
delivery	11	1027(0.12575740253801304)	897 (0.14)	2548 (0.033)
depressive	11	1027(0.12575740253801304)	1516 (0.069)	816 (0.16)
dietitians	11	1027(0.12575740253801304)	1516 (0.069)	999 (0.13)
digestion	11	1027(0.12575740253801304)	717 (0.17)	816 (0.16)
distribution	11	671(0.2057848405167486)	329 (0.38)	999 (0.13)
district	11	1027(0.12575740253801304)	1121 (0.1)	816 (0.16)
duchenne	11	1027(0.12575740253801304)	1121 (0.1)	1233 (0.1)
elders	11	559(0.2400823139362067)	329 (0.38)	526 (0.26)
elementary	11	1027(0.12575740253801304)	1121 (0.1)	816 (0.16)
environmental	11	427(0.3201097519149423)	329 (0.38)	388 (0.36)
excessive	11	1027(0.12575740253801304)	#SAKNAS!	461 (0.3)
exploratory	11	1027(0.12575740253801304)	1121 (0.1)	1233 (0.1)
flow	11	500(0.2743797873556648)	329 (0.38)	461 (0.3)
glucose	11	362(0.37727220761403907)	329 (0.38)	280 (0.49)
hay	11	1027(0.12575740253801304)	897 (0.14)	999 (0.13)
hba1c	11	1027(0.12575740253801304)	1121 (0.1)	999 (0.13)
height	11	1027(0.12575740253801304)	897 (0.14)	816 (0.16)
hemodialysis	11	481(0.2858122784954842)	329 (0.38)	584 (0.23)
hemoglobin	11	1027(0.12575740253801304)	717 (0.17)	1233 (0.1)
herbivore	11	595(0.22864982279638732)	329 (0.38)	584 (0.23)
hispanic	11	1027(0.12575740253801304)	1121 (0.1)	526 (0.26)
holstein	11	1027(0.12575740253801304)	#SAKNAS!	526 (0.26)
hygiene	11	192(0.6745169772493427)	329 (0.38)	158 (0.82)
indicator	11	1027(0.12575740253801304)	1516 (0.069)	584 (0.23)

indonesia	11	1027(0.12575740253801304)	2359 (0.034)	694 (0.2)
ingestion	11	559(0.2400823139362067)	329 (0.38)	584 (0.23)
interdental	11	1027(0.12575740253801304)	#SAKNAS!	999 (0.13)
involvement	11	1027(0.12575740253801304)	1121 (0.1)	816 (0.16)
joint	11	1027(0.12575740253801304)	1121 (0.1)	694 (0.2)
key	11	1027(0.12575740253801304)	595 (0.21)	2548 (0.033)
koreans	11	1027(0.12575740253801304)	1516 (0.069)	999 (0.13)
larval	11	559(0.2400823139362067)	329 (0.38)	584 (0.23)
load	11	1027(0.12575740253801304)	717 (0.17)	999 (0.13)
low-income	11	410(0.33154224305476165)	329 (0.38)	361 (0.4)
maintenance	11	787(0.1714873670972905)	329 (0.38)	1668 (0.066)
matter	11	1027(0.12575740253801304)	1516 (0.069)	816 (0.16)
mediates	11	1027(0.12575740253801304)	#SAKNAS!	999 (0.13)
melanogaster	11	1027(0.12575740253801304)	1121 (0.1)	999 (0.13)
mice	11	281(0.468732136732594)	329 (0.38)	205 (0.66)
milk	11	261(0.49159711901223274)	329 (0.38)	305 (0.46)
modulates	11	1027(0.12575740253801304)	897 (0.14)	999 (0.13)
molar	11	1027(0.12575740253801304)	1516 (0.069)	1233 (0.1)
mouse	11	1027(0.12575740253801304)	1516 (0.069)	1233 (0.1)
multicentre	11	1027(0.12575740253801304)	1516 (0.069)	694 (0.2)
neonatal	11	1027(0.12575740253801304)	595 (0.21)	1233 (0.1)
nervosa	11	500(0.2743797873556648)	329 (0.38)	584 (0.23)
neuronal	11	1027(0.12575740253801304)	398 (0.31)	1668 (0.066)
nutrients	11	559(0.2400823139362067)	329 (0.38)	584 (0.23)
optimal	11	1027(0.12575740253801304)	398 (0.31)	2548 (0.033)
orofacial	11	1027(0.12575740253801304)	1121 (0.1)	999 (0.13)
orthodontic	11	1027(0.12575740253801304)	1516 (0.069)	526 (0.26)
palsy	11	249(0.5144621012918715)	329 (0.38)	305 (0.46)
parenteral	11	1027(0.12575740253801304)	595 (0.21)	816 (0.16)
participants	11	1027(0.12575740253801304)	897 (0.14)	999 (0.13)
pea	11	1027(0.12575740253801304)	1516 (0.069)	#SAKNAS!
peptide	11	1027(0.12575740253801304)	717 (0.17)	1233 (0.1)
pneumonia	11	195(0.6630844861095233)	329 (0.38)	185 (0.73)
populations	11	462(0.2972447696353035)	329 (0.38)	461 (0.3)
postpartum	11	1027(0.12575740253801304)	#SAKNAS!	999 (0.13)
prey	11	559(0.2400823139362067)	329 (0.38)	694 (0.2)
processing	11	1027(0.12575740253801304)	1516 (0.069)	1233 (0.1)
provision	11	1027(0.12575740253801304)	897 (0.14)	1233 (0.1)
recent	11	1027(0.12575740253801304)	717 (0.17)	1233 (0.1)
remaining	11	1027(0.12575740253801304)	1516 (0.069)	816 (0.16)
resource	11	1027(0.12575740253801304)	595 (0.21)	1668 (0.066)
resources	11	1027(0.12575740253801304)	717 (0.17)	999 (0.13)
roles	11	1027(0.12575740253801304)	897 (0.14)	999 (0.13)

site		11	1027(0.12575740253801304)	1121 (0.1)	999 (0.13)
spatial		11	1027(0.12575740253801304)	717 (0.17)	1668 (0.066)
sphincter		11	1027(0.12575740253801304)	2359 (0.034)	1233 (0.1)
starch		11	1027(0.12575740253801304)	1121 (0.1)	694 (0.2)
stem		11	1027(0.12575740253801304)	1121 (0.1)	816 (0.16)
stents		11	595(0.22864982279638732)	329 (0.38)	694 (0.2)
streptococcus		11	1027(0.12575740253801304)	1121 (0.1)	1233 (0.1)
third		11	535(0.2515148050760261)	329 (0.38)	584 (0.23)
traumatic		11	1027(0.12575740253801304)	1516 (0.069)	816 (0.16)
treating		11	1027(0.12575740253801304)	717 (0.17)	1233 (0.1)
underweight		11	1027(0.12575740253801304)	897 (0.14)	584 (0.23)
usa		11	1027(0.12575740253801304)	1121 (0.1)	999 (0.13)
vitro		11	1027(0.12575740253801304)	1516 (0.069)	2548 (0.033)
weaning		11	1027(0.12575740253801304)	520 (0.24)	1233 (0.1)
why		11	1027(0.12575740253801304)	595 (0.21)	816 (0.16)
	2014	10	1111(0.11432491139819366)	#SAKNAS!	584 (0.23)
1999-2004		10	1111(0.11432491139819366)	897 (0.14)	816 (0.16)
1-year		10	1111(0.11432491139819366)	#SAKNAS!	999 (0.13)
25-hydroxyvitamin		10	1111(0.11432491139819366)	717 (0.17)	1233 (0.1)
abnormal		10	1111(0.11432491139819366)	897 (0.14)	1668 (0.066)
abnormalities		10	1111(0.11432491139819366)	595 (0.21)	2548 (0.033)
absorption		10	1111(0.11432491139819366)	717 (0.17)	816 (0.16)
acceptance		10	1111(0.11432491139819366)	1121 (0.1)	816 (0.16)
adolescence		10	1111(0.11432491139819366)	#SAKNAS!	1233 (0.1)
africa		10	896(0.14862238481765175)	362 (0.34)	1668 (0.066)
ageing		10	1111(0.11432491139819366)	1516 (0.069)	999 (0.13)
athletes		10	1111(0.11432491139819366)	1121 (0.1)	999 (0.13)
awareness		10	1111(0.11432491139819366)	1516 (0.069)	1668 (0.066)
baby		10	1111(0.11432491139819366)	#SAKNAS!	816 (0.16)
benign		10	1111(0.11432491139819366)	897 (0.14)	1233 (0.1)
biochemical		10	1111(0.11432491139819366)	717 (0.17)	1233 (0.1)
bmi		10	256(0.5030296101520522)	362 (0.34)	251 (0.56)
canadian		10	1111(0.11432491139819366)	1516 (0.069)	1668 (0.066)
chemical		10	1111(0.11432491139819366)	1121 (0.1)	816 (0.16)
circulating		10	1111(0.11432491139819366)	1516 (0.069)	1668 (0.066)
class		10	1111(0.11432491139819366)	1516 (0.069)	816 (0.16)
coffee		10	1111(0.11432491139819366)	#SAKNAS!	526 (0.26)
colonization		10	1111(0.11432491139819366)	897 (0.14)	816 (0.16)
component		10	1111(0.11432491139819366)	897 (0.14)	816 (0.16)
concentrate		10	1111(0.11432491139819366)	2359 (0.034)	816 (0.16)
count		10	1111(0.11432491139819366)	1516 (0.069)	694 (0.2)
de		10	1111(0.11432491139819366)	#SAKNAS!	584 (0.23)
defects		10	1111(0.11432491139819366)	595 (0.21)	2548 (0.033)

detection	10	1111(0.11432491139819366)	1516 (0.069)	584 (0.23)
diagnosis	10	350(0.3887046987538585)	362 (0.34)	422 (0.33)
diagnostics	10	1111(0.11432491139819366)	717 (0.17)	999 (0.13)
drink	10	1111(0.11432491139819366)	2359 (0.034)	584 (0.23)
dynamics	10	1111(0.11432491139819366)	1121 (0.1)	816 (0.16)
dysphagic	10	396(0.342974734194581)	362 (0.34)	422 (0.33)
educational	10	1111(0.11432491139819366)	1121 (0.1)	1233 (0.1)
emptying	10	1111(0.11432491139819366)	897 (0.14)	1233 (0.1)
erosion	10	1111(0.11432491139819366)	1121 (0.1)	999 (0.13)
estimating	10	1111(0.11432491139819366)	1516 (0.069)	584 (0.23)
feedlot	10	1111(0.11432491139819366)	2359 (0.034)	999 (0.13)
fibrosis	10	1111(0.11432491139819366)	1516 (0.069)	2548 (0.033)
field	10	1111(0.11432491139819366)	717 (0.17)	1233 (0.1)
finishing	10	1111(0.11432491139819366)	1516 (0.069)	#SAKNAS!
fluid	10	396(0.342974734194581)	362 (0.34)	333 (0.43)
form	10	1111(0.11432491139819366)	1121 (0.1)	1233 (0.1)
formation	10	1111(0.11432491139819366)	1121 (0.1)	999 (0.13)
gastroplasty	10	1111(0.11432491139819366)	897 (0.14)	1668 (0.066)
gerd	10	1111(0.11432491139819366)	1121 (0.1)	999 (0.13)
gum	10	1111(0.11432491139819366)	2359 (0.034)	816 (0.16)
habit	10	1111(0.11432491139819366)	1516 (0.069)	1233 (0.1)
helicobacter	10	1111(0.11432491139819366)	1121 (0.1)	999 (0.13)
hemorrhage	10	1111(0.11432491139819366)	1121 (0.1)	999 (0.13)
hormone	10	516(0.26294729621584545)	362 (0.34)	422 (0.33)
horses	10	1111(0.11432491139819366)	1121 (0.1)	694 (0.2)
household	10	1111(0.11432491139819366)	1516 (0.069)	1668 (0.066)
hypopharyngeal	10	1111(0.11432491139819366)	897 (0.14)	999 (0.13)
hypothalamus	10	1111(0.11432491139819366)	717 (0.17)	1668 (0.066)
ill	10	1111(0.11432491139819366)	1121 (0.1)	2548 (0.033)
immune	10	1111(0.11432491139819366)	1516 (0.069)	1668 (0.066)
indian	10	1111(0.11432491139819366)	595 (0.21)	1233 (0.1)
individuals	10	122(1.0517891848633818)	362 (0.34)	97 (1.4)
induction	10	1111(0.11432491139819366)	1121 (0.1)	1233 (0.1)
infections	10	628(0.21721733165656798)	362 (0.34)	694 (0.2)
inpatient	10	1111(0.11432491139819366)	1516 (0.069)	1668 (0.066)
insertion	10	1111(0.11432491139819366)	717 (0.17)	1233 (0.1)
intakes	10	350(0.3887046987538585)	362 (0.34)	194 (0.69)
intensity-modulated	10	1111(0.11432491139819366)	1121 (0.1)	1233 (0.1)
internal	10	1111(0.11432491139819366)	1516 (0.069)	816 (0.16)
iran	10	1111(0.11432491139819366)	595 (0.21)	1668 (0.066)
iv	10	1111(0.11432491139819366)	1516 (0.069)	816 (0.16)
knowledge	10	219(0.594489539270607)	362 (0.34)	280 (0.49)
length	10	1111(0.11432491139819366)	717 (0.17)	1668 (0.066)

liquids	10	1111(0.11432491139819366)	1516 (0.069)	694 (0.2)
literature	10	1111(0.11432491139819366)	595 (0.21)	2548 (0.033)
macaca	10	1111(0.11432491139819366)	1121 (0.1)	816 (0.16)
manifestations	10	1111(0.11432491139819366)	717 (0.17)	1233 (0.1)
manometry	10	1111(0.11432491139819366)	#SAKNAS!	816 (0.16)
masticatory	10	195(0.6630844861095233)	362 (0.34)	166 (0.79)
measures	10	388(0.3544072253344004)	362 (0.34)	422 (0.33)
measuring	10	1111(0.11432491139819366)	717 (0.17)	999 (0.13)
methods	10	377(0.3658397164742197)	362 (0.34)	333 (0.43)
microbiological	10	1111(0.11432491139819366)	897 (0.14)	1233 (0.1)
missing	10	1111(0.11432491139819366)	1516 (0.069)	1668 (0.066)
mixed-methods	10	1111(0.11432491139819366)	#SAKNAS!	526 (0.26)
modulate	10	1111(0.11432491139819366)	1121 (0.1)	999 (0.13)
morphology	10	1111(0.11432491139819366)	1121 (0.1)	999 (0.13)
mouth	10	350(0.3887046987538585)	362 (0.34)	461 (0.3)
multiple	10	293(0.45729964559277464)	362 (0.34)	422 (0.33)
muscles	10	1111(0.11432491139819366)	#SAKNAS!	999 (0.13)
necrosis	10	1111(0.11432491139819366)	717 (0.17)	1668 (0.066)
non-surgical	10	1111(0.11432491139819366)	2359 (0.034)	694 (0.2)
nucleus	10	743(0.18291985823710985)	362 (0.34)	1233 (0.1)
outpatients	10	1111(0.11432491139819366)	897 (0.14)	1233 (0.1)
paediatric	10	559(0.2400823139362067)	362 (0.34)	694 (0.2)
part	10	1111(0.11432491139819366)	1121 (0.1)	816 (0.16)
pathways	10	1111(0.11432491139819366)	1516 (0.069)	999 (0.13)
pattern	10	319(0.4230021721733166)	362 (0.34)	305 (0.46)
plant	10	462(0.2972447696353035)	362 (0.34)	422 (0.33)
poor	10	183(0.6973819595289814)	362 (0.34)	141 (0.92)
potentially	10	1111(0.11432491139819366)	2359 (0.034)	1233 (0.1)
predict	10	439(0.30867726077512286)	362 (0.34)	361 (0.4)
presentation	10	1111(0.11432491139819366)	897 (0.14)	999 (0.13)
pressure	10	228(0.560192065851149)	362 (0.34)	280 (0.49)
procedures	10	1111(0.11432491139819366)	717 (0.17)	2548 (0.033)
progression	10	628(0.21721733165656798)	362 (0.34)	816 (0.16)
promoting	10	1111(0.11432491139819366)	1516 (0.069)	1668 (0.066)
prostheses	10	1111(0.11432491139819366)	595 (0.21)	2548 (0.033)
proximal	10	1111(0.11432491139819366)	2359 (0.034)	999 (0.13)
pulmonary	10	500(0.2743797873556648)	362 (0.34)	584 (0.23)
pylori	10	1111(0.11432491139819366)	1121 (0.1)	999 (0.13)
rare	10	1111(0.11432491139819366)	595 (0.21)	1668 (0.066)
recipients	10	1111(0.11432491139819366)	1121 (0.1)	816 (0.16)
recovery	10	293(0.45729964559277464)	362 (0.34)	388 (0.36)
relative	10	1111(0.11432491139819366)	2359 (0.034)	999 (0.13)
repair	10	388(0.3544072253344004)	362 (0.34)	305 (0.46)

residential		10	1111(0.11432491139819366)	2359 (0.034)	694 (0.2)
roux-en-y		10	331(0.4115696810334972)	362 (0.34)	280 (0.49)
satisfaction		10	1111(0.11432491139819366)	897 (0.14)	999 (0.13)
school-aged		10	1111(0.11432491139819366)	2359 (0.034)	584 (0.23)
sectional		10	1111(0.11432491139819366)	1121 (0.1)	1668 (0.066)
self-efficacy		10	1111(0.11432491139819366)	1121 (0.1)	999 (0.13)
self-report		10	1111(0.11432491139819366)	897 (0.14)	1233 (0.1)
services		10	439(0.30867726077512286)	362 (0.34)	526 (0.26)
significant		10	1111(0.11432491139819366)	717 (0.17)	999 (0.13)
silage		10	1111(0.11432491139819366)	1516 (0.069)	694 (0.2)
society		10	1111(0.11432491139819366)	1516 (0.069)	999 (0.13)
socioeconomic		10	281(0.468732136732594)	362 (0.34)	149 (0.86)
strictures		10	595(0.22864982279638732)	362 (0.34)	584 (0.23)
success		10	1111(0.11432491139819366)	717 (0.17)	1233 (0.1)
supplement		10	1111(0.11432491139819366)	897 (0.14)	1233 (0.1)
sweden		10	787(0.1714873670972905)	362 (0.34)	1668 (0.066)
swedish		10	710(0.19435234937692925)	362 (0.34)	816 (0.16)
taste		10	319(0.4230021721733166)	362 (0.34)	264 (0.53)
transfer		10	1111(0.11432491139819366)	897 (0.14)	1233 (0.1)
treatments		10	1111(0.11432491139819366)	#SAKNAS!	816 (0.16)
tumor		10	1111(0.11432491139819366)	595 (0.21)	1668 (0.066)
turkish		10	1111(0.11432491139819366)	717 (0.17)	1233 (0.1)
undergoing		10	179(0.7088144506688008)	362 (0.34)	149 (0.86)
urinary		10	1111(0.11432491139819366)	2359 (0.034)	816 (0.16)
utility		10	1111(0.11432491139819366)	2359 (0.034)	816 (0.16)
validation		10	224(0.5716245569909684)	362 (0.34)	217 (0.63)
value		10	362(0.37727220761403907)	362 (0.34)	305 (0.46)
variations		10	1111(0.11432491139819366)	717 (0.17)	1668 (0.066)
variety		10	1111(0.11432491139819366)	#SAKNAS!	816 (0.16)
vegetables		10	1111(0.11432491139819366)	1516 (0.069)	999 (0.13)
	2015	9	1219(0.1028924202583743)	#SAKNAS!	584 (0.23)
	2019	9	1219(0.1028924202583743)	#SAKNAS!	2548 (0.033)
1988-1991		9	1219(0.1028924202583743)	398 (0.31)	#SAKNAS!
1988-1991		9	1219(0.1028924202583743)	398 (0.31)	#SAKNAS!
1988-1994		9	1219(0.1028924202583743)	398 (0.31)	#SAKNAS!
1988-1994		9	1219(0.1028924202583743)	398 (0.31)	#SAKNAS!
2008-2010		9	1219(0.1028924202583743)	2359 (0.034)	526 (0.26)
2011-2012		9	1219(0.1028924202583743)	#SAKNAS!	584 (0.23)
2013-2015		9	1219(0.1028924202583743)	#SAKNAS!	999 (0.13)
2016-2018		9	1219(0.1028924202583743)	#SAKNAS!	#SAKNAS!
adequacy		9	1219(0.1028924202583743)	897 (0.14)	1233 (0.1)
affect		9	293(0.45729964559277464)	398 (0.31)	217 (0.63)
agents		9	1219(0.1028924202583743)	717 (0.17)	1668 (0.066)

aggressive	9	1219(0.1028924202583743)	897 (0.14)	816 (0.16)
alzheimer	9	396(0.342974734194581)	398 (0.31)	461 (0.3)
appetitive	9	1219(0.1028924202583743)	#SAKNAS!	1668 (0.066)
approaches	9	1219(0.1028924202583743)	1121 (0.1)	999 (0.13)
atlantic	9	1219(0.1028924202583743)	717 (0.17)	999 (0.13)
audit	9	1219(0.1028924202583743)	595 (0.21)	1233 (0.1)
bacteria	9	481(0.2858122784954842)	398 (0.31)	333 (0.43)
beliefs	9	1219(0.1028924202583743)	717 (0.17)	1668 (0.066)
benefit	9	1219(0.1028924202583743)	1516 (0.069)	1233 (0.1)
birds	9	1027(0.12575740253801304)	398 (0.31)	1668 (0.066)
breathing	9	1219(0.1028924202583743)	1516 (0.069)	816 (0.16)
caregiver	9	1219(0.1028924202583743)	897 (0.14)	#SAKNAS!
caregivers	9	462(0.2972447696353035)	398 (0.31)	694 (0.2)
carotid	9	1219(0.1028924202583743)	717 (0.17)	1668 (0.066)
cell	9	281(0.468732136732594)	398 (0.31)	305 (0.46)
chickens	9	1219(0.1028924202583743)	717 (0.17)	1668 (0.066)
chilean	9	1219(0.1028924202583743)	1516 (0.069)	1233 (0.1)
china	9	163(0.7431119240882588)	398 (0.31)	166 (0.79)
cigarette	9	1219(0.1028924202583743)	595 (0.21)	1668 (0.066)
cleft	9	1219(0.1028924202583743)	1121 (0.1)	1668 (0.066)
clustering	9	1219(0.1028924202583743)	897 (0.14)	#SAKNAS!
comparative	9	293(0.45729964559277464)	398 (0.31)	361 (0.4)
compensatory	9	1219(0.1028924202583743)	520 (0.24)	1668 (0.066)
complete	9	535(0.2515148050760261)	398 (0.31)	1668 (0.066)
compounds	9	1219(0.1028924202583743)	2359 (0.034)	1668 (0.066)
consensus	9	1219(0.1028924202583743)	#SAKNAS!	816 (0.16)
consuming	9	1219(0.1028924202583743)	717 (0.17)	1668 (0.066)
contribution	9	836(0.16005487595747114)	398 (0.31)	1233 (0.1)
copepod	9	1219(0.1028924202583743)	398 (0.31)	#SAKNAS!
copepod	9	1219(0.1028924202583743)	398 (0.31)	#SAKNAS!
counseling	9	1219(0.1028924202583743)	897 (0.14)	816 (0.16)
critical	9	1219(0.1028924202583743)	1516 (0.069)	1233 (0.1)
culture	9	1219(0.1028924202583743)	451 (0.28)	#SAKNAS!
deciduous	9	1219(0.1028924202583743)	897 (0.14)	1233 (0.1)
decisions	9	1219(0.1028924202583743)	1516 (0.069)	694 (0.2)
deficiencies	9	836(0.16005487595747114)	398 (0.31)	1233 (0.1)
deficiency	9	559(0.2400823139362067)	398 (0.31)	816 (0.16)
dentate	9	1219(0.1028924202583743)	1516 (0.069)	999 (0.13)
dialysis	9	1219(0.1028924202583743)	897 (0.14)	1668 (0.066)
diptera	9	966(0.1371898936778324)	398 (0.31)	1233 (0.1)
direct	9	1219(0.1028924202583743)	897 (0.14)	1668 (0.066)
discharge	9	1219(0.1028924202583743)	2359 (0.034)	1233 (0.1)
disparities	9	535(0.2515148050760261)	398 (0.31)	461 (0.3)

drug	9	1219(0.1028924202583743)	717 (0.17)	1233 (0.1)
dyslipidemia	9	1219(0.1028924202583743)	897 (0.14)	1668 (0.066)
eastern	9	1219(0.1028924202583743)	1121 (0.1)	1233 (0.1)
efficiency	9	462(0.2972447696353035)	398 (0.31)	388 (0.36)
egg	9	1219(0.1028924202583743)	897 (0.14)	1233 (0.1)
equation	9	1219(0.1028924202583743)	2359 (0.034)	1233 (0.1)
estimates	9	1219(0.1028924202583743)	717 (0.17)	999 (0.13)
ethnic	9	1219(0.1028924202583743)	1121 (0.1)	1233 (0.1)
exclusive	9	1219(0.1028924202583743)	1121 (0.1)	2548 (0.033)
fecal	9	1219(0.1028924202583743)	#SAKNAS!	816 (0.16)
fermentation	9	1219(0.1028924202583743)	#SAKNAS!	2548 (0.033)
finland	9	1219(0.1028924202583743)	2359 (0.034)	999 (0.13)
forage	9	1219(0.1028924202583743)	1121 (0.1)	1233 (0.1)
framework	9	1219(0.1028924202583743)	897 (0.14)	2548 (0.033)
genetic	9	396(0.342974734194581)	398 (0.31)	526 (0.26)
gingivitis	9	559(0.2400823139362067)	398 (0.31)	584 (0.23)
gustatory	9	1219(0.1028924202583743)	1516 (0.069)	584 (0.23)
handgrip	9	1219(0.1028924202583743)	#SAKNAS!	1233 (0.1)
head-and-neck	9	1219(0.1028924202583743)	897 (0.14)	1668 (0.066)
heller	9	1219(0.1028924202583743)	1516 (0.069)	694 (0.2)
high-resolution	9	1219(0.1028924202583743)	#SAKNAS!	816 (0.16)
high-risk	9	1219(0.1028924202583743)	1121 (0.1)	1233 (0.1)
homeostasis	9	1219(0.1028924202583743)	595 (0.21)	1668 (0.066)
impairments	9	1219(0.1028924202583743)	1121 (0.1)	1233 (0.1)
inappropriate	9	1219(0.1028924202583743)	1121 (0.1)	1233 (0.1)
inequality	9	1219(0.1028924202583743)	#SAKNAS!	694 (0.2)
initiative	9	1219(0.1028924202583743)	2359 (0.034)	1668 (0.066)
insect	9	836(0.16005487595747114)	398 (0.31)	1668 (0.066)
insulin	9	500(0.2743797873556648)	398 (0.31)	388 (0.36)
intensive	9	481(0.2858122784954842)	398 (0.31)	584 (0.23)
island	9	1219(0.1028924202583743)	897 (0.14)	999 (0.13)
korean	9	71(1.6348462329941693)	398 (0.31)	38 (3)
lack	9	1219(0.1028924202583743)	1121 (0.1)	816 (0.16)
lactobacillus	9	1219(0.1028924202583743)	2359 (0.034)	999 (0.13)
lambs	9	1219(0.1028924202583743)	2359 (0.034)	694 (0.2)
language	9	1219(0.1028924202583743)	717 (0.17)	1668 (0.066)
laryngectomy	9	1219(0.1028924202583743)	897 (0.14)	999 (0.13)
late	9	535(0.2515148050760261)	398 (0.31)	526 (0.26)
libitum	9	1219(0.1028924202583743)	1121 (0.1)	1668 (0.066)
links	9	1219(0.1028924202583743)	1516 (0.069)	999 (0.13)
lipoprotein	9	1219(0.1028924202583743)	897 (0.14)	2548 (0.033)
live	9	1219(0.1028924202583743)	2359 (0.034)	1233 (0.1)
malignant	9	516(0.26294729621584545)	398 (0.31)	584 (0.23)

mechanism	9	1219(0.1028924202583743)	717 (0.17)	1668 (0.066)
mechanisms	9	535(0.2515148050760261)	398 (0.31)	584 (0.23)
methamphetamine	9	1219(0.1028924202583743)	1516 (0.069)	694 (0.2)
method	9	481(0.2858122784954842)	398 (0.31)	694 (0.2)
micronutrient	9	1219(0.1028924202583743)	897 (0.14)	2548 (0.033)
modifies	9	1219(0.1028924202583743)	1516 (0.069)	1233 (0.1)
mucosal	9	1219(0.1028924202583743)	717 (0.17)	1668 (0.066)
multivariate	9	1219(0.1028924202583743)	1121 (0.1)	816 (0.16)
myotomy	9	427(0.3201097519149423)	398 (0.31)	333 (0.43)
negatively	9	1219(0.1028924202583743)	#SAKNAS!	816 (0.16)
network	9	1219(0.1028924202583743)	897 (0.14)	2548 (0.033)
neuronal	9	1027(0.12575740253801304)	398 (0.31)	1668 (0.066)
nile	9	1219(0.1028924202583743)	897 (0.14)	1233 (0.1)
non-obese	9	1219(0.1028924202583743)	2359 (0.034)	694 (0.2)
north	9	787(0.1714873670972905)	398 (0.31)	1233 (0.1)
novel	9	237(0.5373270835715103)	398 (0.31)	205 (0.66)
objective	9	1219(0.1028924202583743)	1121 (0.1)	816 (0.16)
optimal	9	1027(0.12575740253801304)	398 (0.31)	2548 (0.033)
organic	9	1219(0.1028924202583743)	2359 (0.034)	2548 (0.033)
pakistan	9	1219(0.1028924202583743)	2359 (0.034)	1233 (0.1)
parental	9	228(0.560192065851149)	398 (0.31)	166 (0.79)
particle	9	1219(0.1028924202583743)	1121 (0.1)	1233 (0.1)
pathogen	9	1219(0.1028924202583743)	2359 (0.034)	816 (0.16)
pathology	9	1219(0.1028924202583743)	897 (0.14)	1233 (0.1)
pharyngeal	9	559(0.2400823139362067)	398 (0.31)	816 (0.16)
physiological	9	516(0.26294729621584545)	398 (0.31)	584 (0.23)
physiology	9	628(0.21721733165656798)	398 (0.31)	584 (0.23)
portuguese	9	1219(0.1028924202583743)	#SAKNAS!	2548 (0.033)
position	9	628(0.21721733165656798)	398 (0.31)	584 (0.23)
practice	9	388(0.3544072253344004)	398 (0.31)	333 (0.43)
predictive	9	281(0.468732136732594)	398 (0.31)	305 (0.46)
prenatal	9	1219(0.1028924202583743)	1121 (0.1)	1233 (0.1)
presenting	9	1219(0.1028924202583743)	1121 (0.1)	1233 (0.1)
preterm	9	516(0.26294729621584545)	398 (0.31)	999 (0.13)
profile	9	256(0.5030296101520522)	398 (0.31)	280 (0.49)
prosthetic	9	1219(0.1028924202583743)	897 (0.14)	1668 (0.066)
questionnaire	9	388(0.3544072253344004)	398 (0.31)	584 (0.23)
recurrence	9	1219(0.1028924202583743)	1516 (0.069)	694 (0.2)
regarding	9	1219(0.1028924202583743)	2359 (0.034)	999 (0.13)
relationships	9	362(0.37727220761403907)	398 (0.31)	361 (0.4)
removal	9	1219(0.1028924202583743)	717 (0.17)	2548 (0.033)
reproduction	9	1219(0.1028924202583743)	595 (0.21)	1233 (0.1)
research	9	377(0.3658397164742197)	398 (0.31)	694 (0.2)

river		9	1219(0.1028924202583743)	1121 (0.1)	1233 (0.1)
routine		9	1219(0.1028924202583743)	1516 (0.069)	999 (0.13)
rumen		9	1219(0.1028924202583743)	1121 (0.1)	1233 (0.1)
saliva		9	261(0.49159711901223274)	398 (0.31)	228 (0.59)
school-based		9	1219(0.1028924202583743)	2359 (0.034)	1233 (0.1)
short-term		9	500(0.2743797873556648)	398 (0.31)	526 (0.26)
shrimp		9	1219(0.1028924202583743)	717 (0.17)	1233 (0.1)
signals		9	1219(0.1028924202583743)	595 (0.21)	1668 (0.066)
smoke		9	1219(0.1028924202583743)	1121 (0.1)	1233 (0.1)
sodium		9	1219(0.1028924202583743)	2359 (0.034)	816 (0.16)
specific		9	787(0.1714873670972905)	398 (0.31)	2548 (0.033)
stent		9	671(0.2057848405167486)	398 (0.31)	584 (0.23)
stimulation		9	331(0.4115696810334972)	398 (0.31)	228 (0.59)
successful		9	743(0.18291985823710985)	398 (0.31)	999 (0.13)
sweet		9	1219(0.1028924202583743)	#SAKNAS!	999 (0.13)
technique		9	1219(0.1028924202583743)	1516 (0.069)	999 (0.13)
tests		9	1219(0.1028924202583743)	897 (0.14)	999 (0.13)
thickeners		9	1219(0.1028924202583743)	#SAKNAS!	816 (0.16)
thoracic		9	1219(0.1028924202583743)	1516 (0.069)	816 (0.16)
tissue		9	462(0.2972447696353035)	398 (0.31)	461 (0.3)
tongue		9	207(0.6402195038298846)	398 (0.31)	174 (0.76)
unresectable		9	1219(0.1028924202583743)	897 (0.14)	816 (0.16)
visits		9	1219(0.1028924202583743)	1121 (0.1)	999 (0.13)
voice		9	1219(0.1028924202583743)	1121 (0.1)	1668 (0.066)
wear		9	1219(0.1028924202583743)	1516 (0.069)	1668 (0.066)
wheat		9	1219(0.1028924202583743)	#SAKNAS!	999 (0.13)
zealand		9	1219(0.1028924202583743)	897 (0.14)	1668 (0.066)
	2011	8	1335(0.09145992911855493)	2359 (0.034)	694 (0.2)
ability		8	293(0.45729964559277464)	451 (0.28)	217 (0.63)
actinomycetemcomitans		8	1335(0.09145992911855493)	897 (0.14)	1233 (0.1)
action		8	710(0.19435234937692925)	451 (0.28)	816 (0.16)
additives		8	1335(0.09145992911855493)	2359 (0.034)	1668 (0.066)
administration		8	710(0.19435234937692925)	451 (0.28)	1233 (0.1)
agent		8	1335(0.09145992911855493)	1516 (0.069)	1233 (0.1)
aids		8	1335(0.09145992911855493)	451 (0.28)	#SAKNAS!
aids		8	1335(0.09145992911855493)	451 (0.28)	#SAKNAS!
als		8	595(0.22864982279638732)	451 (0.28)	816 (0.16)
anastomosis		8	1335(0.09145992911855493)	2359 (0.034)	999 (0.13)
anatomy		8	1335(0.09145992911855493)	897 (0.14)	1233 (0.1)
anemia		8	1335(0.09145992911855493)	897 (0.14)	999 (0.13)
anthropometric		8	559(0.2400823139362067)	451 (0.28)	1668 (0.066)
aphididae		8	1335(0.09145992911855493)	2359 (0.034)	999 (0.13)
area		8	377(0.3658397164742197)	451 (0.28)	526 (0.26)

attitudes	8	439(0.30867726077512286)	451 (0.28)	584 (0.23)
basic	8	1335(0.09145992911855493)	1516 (0.069)	2548 (0.033)
bass	8	1335(0.09145992911855493)	451 (0.28)	#SAKNAS!
bass	8	1335(0.09145992911855493)	451 (0.28)	#SAKNAS!
bedside	8	1335(0.09145992911855493)	1516 (0.069)	1668 (0.066)
bee	8	1335(0.09145992911855493)	897 (0.14)	1233 (0.1)
biology	8	1335(0.09145992911855493)	717 (0.17)	1668 (0.066)
bottle	8	1335(0.09145992911855493)	897 (0.14)	2548 (0.033)
bulbar	8	1335(0.09145992911855493)	#SAKNAS!	1668 (0.066)
calcium	8	671(0.2057848405167486)	451 (0.28)	526 (0.26)
carbohydrates	8	1335(0.09145992911855493)	897 (0.14)	2548 (0.033)
cells	8	710(0.19435234937692925)	451 (0.28)	1233 (0.1)
centers	8	1335(0.09145992911855493)	897 (0.14)	#SAKNAS!
child-feeding	8	1335(0.09145992911855493)	717 (0.17)	1668 (0.066)
chimpanzees	8	1335(0.09145992911855493)	1516 (0.069)	999 (0.13)
chinese	8	237(0.5373270835715103)	451 (0.28)	174 (0.76)
choice	8	396(0.342974734194581)	451 (0.28)	388 (0.36)
choices	8	1335(0.09145992911855493)	1516 (0.069)	999 (0.13)
coleoptera	8	1335(0.09145992911855493)	897 (0.14)	1233 (0.1)
college	8	1335(0.09145992911855493)	717 (0.17)	1233 (0.1)
communication	8	1335(0.09145992911855493)	897 (0.14)	1233 (0.1)
comorbidities	8	1335(0.09145992911855493)	1516 (0.069)	1233 (0.1)
concurrent	8	628(0.21721733165656798)	451 (0.28)	816 (0.16)
consequences	8	595(0.22864982279638732)	451 (0.28)	584 (0.23)
controls	8	1335(0.09145992911855493)	1121 (0.1)	816 (0.16)
convalescent	8	1335(0.09145992911855493)	2359 (0.034)	1233 (0.1)
counselling	8	1335(0.09145992911855493)	1121 (0.1)	1233 (0.1)
cues	8	1335(0.09145992911855493)	717 (0.17)	1668 (0.066)
cultural	8	1335(0.09145992911855493)	1516 (0.069)	694 (0.2)
culture	8	1219(0.1028924202583743)	451 (0.28)	#SAKNAS!
cycle	8	1335(0.09145992911855493)	897 (0.14)	999 (0.13)
daily	8	268(0.4801646278724134)	451 (0.28)	228 (0.59)
decrease	8	1335(0.09145992911855493)	1121 (0.1)	1668 (0.066)
deer	8	743(0.18291985823710985)	451 (0.28)	999 (0.13)
denture	8	516(0.26294729621584545)	451 (0.28)	1233 (0.1)
dependence	8	1335(0.09145992911855493)	897 (0.14)	1233 (0.1)
dependent	8	1335(0.09145992911855493)	#SAKNAS!	816 (0.16)
deprivation	8	1335(0.09145992911855493)	1121 (0.1)	1668 (0.066)
dietetic	8	1335(0.09145992911855493)	520 (0.24)	2548 (0.033)
difficulties	8	481(0.2858122784954842)	451 (0.28)	694 (0.2)
double	8	1335(0.09145992911855493)	1516 (0.069)	999 (0.13)
drinking	8	595(0.22864982279638732)	451 (0.28)	816 (0.16)
dynamic	8	1335(0.09145992911855493)	1121 (0.1)	1668 (0.066)

edentulous	8	559(0.2400823139362067)	451 (0.28)	526 (0.26)
emotional	8	1335(0.09145992911855493)	#SAKNAS!	816 (0.16)
environment	8	439(0.30867726077512286)	451 (0.28)	422 (0.33)
epidermolysis	8	896(0.14862238481765175)	451 (0.28)	1233 (0.1)
evaluate	8	1335(0.09145992911855493)	1121 (0.1)	2548 (0.033)
excess	8	1335(0.09145992911855493)	#SAKNAS!	816 (0.16)
exercise	8	462(0.2972447696353035)	451 (0.28)	526 (0.26)
expansion	8	1335(0.09145992911855493)	#SAKNAS!	1668 (0.066)
extract	8	1335(0.09145992911855493)	717 (0.17)	1233 (0.1)
fasting	8	559(0.2400823139362067)	451 (0.28)	461 (0.3)
fistula	8	1335(0.09145992911855493)	1516 (0.069)	999 (0.13)
frail	8	1335(0.09145992911855493)	2359 (0.034)	816 (0.16)
free	8	500(0.2743797873556648)	451 (0.28)	388 (0.36)
free-ranging	8	1335(0.09145992911855493)	897 (0.14)	1233 (0.1)
fruits	8	1335(0.09145992911855493)	2359 (0.034)	999 (0.13)
full	8	1335(0.09145992911855493)	#SAKNAS!	816 (0.16)
gastro-oesophageal	8	1335(0.09145992911855493)	717 (0.17)	1233 (0.1)
gestation	8	1335(0.09145992911855493)	897 (0.14)	1233 (0.1)
goats	8	743(0.18291985823710985)	451 (0.28)	1233 (0.1)
grade	8	1335(0.09145992911855493)	1516 (0.069)	1668 (0.066)
great	8	1335(0.09145992911855493)	717 (0.17)	1668 (0.066)
habitat	8	1335(0.09145992911855493)	897 (0.14)	999 (0.13)
hernia	8	410(0.33154224305476165)	451 (0.28)	388 (0.36)
high-density	8	1335(0.09145992911855493)	2359 (0.034)	#SAKNAS!
hiv	8	1335(0.09145992911855493)	717 (0.17)	2548 (0.033)
hormones	8	1335(0.09145992911855493)	1516 (0.069)	1233 (0.1)
hydration	8	1335(0.09145992911855493)	1121 (0.1)	1668 (0.066)
hymenoptera	8	1335(0.09145992911855493)	897 (0.14)	1668 (0.066)
hyperactivity	8	1335(0.09145992911855493)	1121 (0.1)	1233 (0.1)
hypofunction	8	1335(0.09145992911855493)	#SAKNAS!	2548 (0.033)
identification	8	628(0.21721733165656798)	451 (0.28)	999 (0.13)
identifying	8	1335(0.09145992911855493)	897 (0.14)	1668 (0.066)
image	8	1335(0.09145992911855493)	1121 (0.1)	999 (0.13)
immunity	8	1335(0.09145992911855493)	2359 (0.034)	999 (0.13)
immunoglobulin	8	1335(0.09145992911855493)	#SAKNAS!	584 (0.23)
india	8	500(0.2743797873556648)	451 (0.28)	526 (0.26)
indications	8	1335(0.09145992911855493)	1121 (0.1)	999 (0.13)
influences	8	595(0.22864982279638732)	451 (0.28)	526 (0.26)
information	8	1335(0.09145992911855493)	897 (0.14)	999 (0.13)
infusion	8	1335(0.09145992911855493)	1516 (0.069)	816 (0.16)
insects	8	1335(0.09145992911855493)	1516 (0.069)	816 (0.16)
integration	8	1335(0.09145992911855493)	595 (0.21)	1668 (0.066)
interactions	8	481(0.2858122784954842)	451 (0.28)	361 (0.4)

interleukin-6	8	1335(0.09145992911855493)	717 (0.17)	1668 (0.066)
interplay	8	1335(0.09145992911855493)	#SAKNAS!	1668 (0.066)
interprofessional	8	1335(0.09145992911855493)	2359 (0.034)	816 (0.16)
introduction	8	1335(0.09145992911855493)	2359 (0.034)	1233 (0.1)
items	8	1335(0.09145992911855493)	717 (0.17)	#SAKNAS!
japan	8	281(0.468732136732594)	451 (0.28)	217 (0.63)
jejunostomy	8	1335(0.09145992911855493)	717 (0.17)	1233 (0.1)
kingdom	8	1335(0.09145992911855493)	2359 (0.034)	999 (0.13)
lactating	8	628(0.21721733165656798)	451 (0.28)	694 (0.2)
lepidoptera	8	1335(0.09145992911855493)	595 (0.21)	1668 (0.066)
local	8	595(0.22864982279638732)	451 (0.28)	584 (0.23)
locally	8	535(0.2515148050760261)	451 (0.28)	461 (0.3)
longevity	8	1335(0.09145992911855493)	595 (0.21)	#SAKNAS!
males	8	1335(0.09145992911855493)	2359 (0.034)	816 (0.16)
malocclusion	8	1335(0.09145992911855493)	1516 (0.069)	1233 (0.1)
mealtime	8	1335(0.09145992911855493)	2359 (0.034)	816 (0.16)
mechanical	8	1335(0.09145992911855493)	2359 (0.034)	1233 (0.1)
metal	8	710(0.19435234937692925)	451 (0.28)	999 (0.13)
mexican	8	628(0.21721733165656798)	451 (0.28)	584 (0.23)
middle	8	1335(0.09145992911855493)	1121 (0.1)	1668 (0.066)
mild	8	1335(0.09145992911855493)	1516 (0.069)	1233 (0.1)
mobile	8	1335(0.09145992911855493)	2359 (0.034)	1233 (0.1)
moderate	8	1335(0.09145992911855493)	#SAKNAS!	584 (0.23)
monitoring	8	1335(0.09145992911855493)	2359 (0.034)	999 (0.13)
months	8	362(0.37727220761403907)	451 (0.28)	388 (0.36)
morbid	8	787(0.1714873670972905)	451 (0.28)	816 (0.16)
morbidity	8	966(0.1371898936778324)	451 (0.28)	2548 (0.033)
motivation	8	1335(0.09145992911855493)	2359 (0.034)	999 (0.13)
multilevel	8	1335(0.09145992911855493)	1516 (0.069)	999 (0.13)
muscular	8	410(0.33154224305476165)	451 (0.28)	816 (0.16)
mutans	8	559(0.2400823139362067)	451 (0.28)	526 (0.26)
neural	8	1335(0.09145992911855493)	#SAKNAS!	999 (0.13)
neurogenic	8	743(0.18291985823710985)	451 (0.28)	1233 (0.1)
neurologically	8	1335(0.09145992911855493)	1121 (0.1)	1233 (0.1)
nissen	8	671(0.2057848405167486)	451 (0.28)	816 (0.16)
nomogram	8	1335(0.09145992911855493)	#SAKNAS!	#SAKNAS!
normal	8	516(0.26294729621584545)	451 (0.28)	461 (0.3)
observation	8	1335(0.09145992911855493)	1516 (0.069)	2548 (0.033)
oncology	8	1335(0.09145992911855493)	717 (0.17)	1668 (0.066)
one-year	8	1335(0.09145992911855493)	1121 (0.1)	1233 (0.1)
osteoporosis	8	1335(0.09145992911855493)	897 (0.14)	2548 (0.033)
ovary	8	1335(0.09145992911855493)	1121 (0.1)	#SAKNAS!
per	8	1335(0.09145992911855493)	2359 (0.034)	1668 (0.066)

perceptions	8	595(0.22864982279638732)	451 (0.28)	694 (0.2)
persistent	8	1335(0.09145992911855493)	717 (0.17)	1668 (0.066)
perspective	8	710(0.19435234937692925)	451 (0.28)	999 (0.13)
plus	8	1335(0.09145992911855493)	2359 (0.034)	816 (0.16)
point	8	1335(0.09145992911855493)	1516 (0.069)	816 (0.16)
poland	8	1335(0.09145992911855493)	#SAKNAS!	1233 (0.1)
polycystic	8	1335(0.09145992911855493)	1121 (0.1)	#SAKNAS!
post	8	1335(0.09145992911855493)	1516 (0.069)	1233 (0.1)
practical	8	1335(0.09145992911855493)	1516 (0.069)	1233 (0.1)
predation	8	1335(0.09145992911855493)	717 (0.17)	1233 (0.1)
prediction	8	462(0.2972447696353035)	451 (0.28)	816 (0.16)
pregnant	8	350(0.3887046987538585)	451 (0.28)	388 (0.36)
preventing	8	1335(0.09145992911855493)	2359 (0.034)	816 (0.16)
radiological	8	1335(0.09145992911855493)	1121 (0.1)	1233 (0.1)
recommendations	8	462(0.2972447696353035)	451 (0.28)	422 (0.33)
registry	8	1335(0.09145992911855493)	#SAKNAS!	816 (0.16)
regression	8	1335(0.09145992911855493)	1121 (0.1)	999 (0.13)
relevance	8	1335(0.09145992911855493)	897 (0.14)	1233 (0.1)
reproductive	8	671(0.2057848405167486)	451 (0.28)	1233 (0.1)
resection	8	671(0.2057848405167486)	451 (0.28)	1233 (0.1)
restrictive	8	1335(0.09145992911855493)	1516 (0.069)	1233 (0.1)
schools	8	559(0.2400823139362067)	451 (0.28)	694 (0.2)
screen	8	1335(0.09145992911855493)	2359 (0.034)	2548 (0.033)
seasonal	8	559(0.2400823139362067)	451 (0.28)	422 (0.33)
self-expanding	8	896(0.14862238481765175)	451 (0.28)	999 (0.13)
self-selection	8	966(0.1371898936778324)	451 (0.28)	999 (0.13)
service	8	628(0.21721733165656798)	451 (0.28)	694 (0.2)
settings	8	1335(0.09145992911855493)	#SAKNAS!	584 (0.23)
sexual	8	1335(0.09145992911855493)	1121 (0.1)	999 (0.13)
shanghai	8	1335(0.09145992911855493)	1516 (0.069)	2548 (0.033)
shape	8	1335(0.09145992911855493)	1516 (0.069)	1668 (0.066)
sheep	8	481(0.2858122784954842)	451 (0.28)	305 (0.46)
socio-economic	8	896(0.14862238481765175)	451 (0.28)	1233 (0.1)
sows	8	1335(0.09145992911855493)	897 (0.14)	2548 (0.033)
speech-language	8	1335(0.09145992911855493)	2359 (0.034)	1668 (0.066)
stage	8	439(0.30867726077512286)	451 (0.28)	584 (0.23)
standard	8	1335(0.09145992911855493)	2359 (0.034)	999 (0.13)
structure	8	710(0.19435234937692925)	451 (0.28)	999 (0.13)
style	8	1335(0.09145992911855493)	1516 (0.069)	694 (0.2)
subsequent	8	1335(0.09145992911855493)	1516 (0.069)	1668 (0.066)
supplementation	8	362(0.37727220761403907)	451 (0.28)	388 (0.36)
supply	8	1335(0.09145992911855493)	717 (0.17)	1233 (0.1)
supportive	8	1335(0.09145992911855493)	#SAKNAS!	1233 (0.1)

suppresses	8	1335(0.09145992911855493)	2359 (0.034)	816 (0.16)
susceptibility	8	1335(0.09145992911855493)	1121 (0.1)	1668 (0.066)
sympatric	8	1335(0.09145992911855493)	595 (0.21)	#SAKNAS!
tanzania	8	1335(0.09145992911855493)	897 (0.14)	2548 (0.033)
tea	8	1335(0.09145992911855493)	#SAKNAS!	694 (0.2)
test	8	595(0.22864982279638732)	451 (0.28)	694 (0.2)
testing	8	1335(0.09145992911855493)	1121 (0.1)	816 (0.16)
thailand	8	1335(0.09145992911855493)	1121 (0.1)	1233 (0.1)
therapeutic	8	628(0.21721733165656798)	451 (0.28)	1668 (0.066)
thermal	8	1335(0.09145992911855493)	717 (0.17)	2548 (0.033)
tools	8	1335(0.09145992911855493)	2359 (0.034)	694 (0.2)
towards	8	1335(0.09145992911855493)	2359 (0.034)	#SAKNAS!
toxicity	8	787(0.1714873670972905)	451 (0.28)	999 (0.13)
toxin	8	836(0.16005487595747114)	451 (0.28)	816 (0.16)
transoral	8	1335(0.09145992911855493)	1516 (0.069)	1233 (0.1)
treat	8	1335(0.09145992911855493)	#SAKNAS!	694 (0.2)
triglyceride	8	1335(0.09145992911855493)	1516 (0.069)	1233 (0.1)
ultra-processed	8	1335(0.09145992911855493)	#SAKNAS!	1668 (0.066)
ultrasonography	8	1335(0.09145992911855493)	#SAKNAS!	999 (0.13)
ultrasound	8	1335(0.09145992911855493)	1516 (0.069)	1233 (0.1)
underwent	8	1335(0.09145992911855493)	1516 (0.069)	999 (0.13)
units	8	1335(0.09145992911855493)	1121 (0.1)	1668 (0.066)
unmet	8	1335(0.09145992911855493)	#SAKNAS!	1668 (0.066)
vascular	8	896(0.14862238481765175)	451 (0.28)	999 (0.13)
ventilation	8	1335(0.09145992911855493)	1121 (0.1)	1233 (0.1)
videofluoroscopy	8	1335(0.09145992911855493)	2359 (0.034)	816 (0.16)
visual	8	1335(0.09145992911855493)	2359 (0.034)	1668 (0.066)
ward	8	1335(0.09145992911855493)	2359 (0.034)	1233 (0.1)
wearing	8	1335(0.09145992911855493)	1516 (0.069)	#SAKNAS!
welfare	8	1335(0.09145992911855493)	2359 (0.034)	2548 (0.033)
working	8	1335(0.09145992911855493)	#SAKNAS!	1233 (0.1)
12-year-old	7	787(0.1714873670972905)	520 (0.24)	694 (0.2)
3-year-old	7	1485(0.08002743797873557)	1121 (0.1)	1668 (0.066)
5-year-old	7	1485(0.08002743797873557)	1121 (0.1)	1233 (0.1)
abundance	7	1485(0.08002743797873557)	1121 (0.1)	1668 (0.066)
according	7	249(0.5144621012918715)	520 (0.24)	205 (0.66)
accuracy	7	1485(0.08002743797873557)	1121 (0.1)	1233 (0.1)
active	7	743(0.18291985823710985)	520 (0.24)	999 (0.13)
adipose	7	1485(0.08002743797873557)	1516 (0.069)	1668 (0.066)
against	7	410(0.33154224305476165)	520 (0.24)	388 (0.36)
alters	7	743(0.18291985823710985)	520 (0.24)	694 (0.2)
americans	7	1485(0.08002743797873557)	717 (0.17)	2548 (0.033)
amongst	7	1485(0.08002743797873557)	1516 (0.069)	1233 (0.1)

amount	7	1485(0.08002743797873557)	#SAKNAS!	999 (0.13)
angle	7	1485(0.08002743797873557)	2359 (0.034)	1233 (0.1)
anterior	7	268(0.4801646278724134)	520 (0.24)	228 (0.59)
anthropometry	7	1485(0.08002743797873557)	1516 (0.069)	1233 (0.1)
antibody	7	1485(0.08002743797873557)	717 (0.17)	1668 (0.066)
anti-reflux	7	1485(0.08002743797873557)	#SAKNAS!	1233 (0.1)
anxiety	7	1485(0.08002743797873557)	2359 (0.034)	1233 (0.1)
aortic	7	1485(0.08002743797873557)	2359 (0.034)	1233 (0.1)
aphids	7	1485(0.08002743797873557)	1121 (0.1)	1668 (0.066)
apical	7	1485(0.08002743797873557)	#SAKNAS!	1233 (0.1)
assessing	7	293(0.45729964559277464)	520 (0.24)	264 (0.53)
atrophy	7	559(0.2400823139362067)	520 (0.24)	999 (0.13)
attending	7	516(0.26294729621584545)	520 (0.24)	584 (0.23)
availability	7	628(0.21721733165656798)	520 (0.24)	816 (0.16)
bacterial	7	350(0.3887046987538585)	520 (0.24)	264 (0.53)
balloon	7	1485(0.08002743797873557)	1121 (0.1)	1668 (0.066)
band	7	1485(0.08002743797873557)	1516 (0.069)	1233 (0.1)
bees	7	1485(0.08002743797873557)	#SAKNAS!	694 (0.2)
benthic	7	1485(0.08002743797873557)	1121 (0.1)	1668 (0.066)
bleeding	7	1485(0.08002743797873557)	1516 (0.069)	1233 (0.1)
bolus	7	1485(0.08002743797873557)	1121 (0.1)	2548 (0.033)
botulinum	7	1027(0.12575740253801304)	520 (0.24)	999 (0.13)
bowel	7	1485(0.08002743797873557)	2359 (0.034)	1668 (0.066)
breastfeeding	7	410(0.33154224305476165)	520 (0.24)	526 (0.26)
breast-feeding	7	1485(0.08002743797873557)	1121 (0.1)	1233 (0.1)
bulimia	7	1485(0.08002743797873557)	897 (0.14)	1668 (0.066)
bullosa	7	966(0.1371898936778324)	520 (0.24)	1233 (0.1)
cachexia	7	1485(0.08002743797873557)	2359 (0.034)	1668 (0.066)
caloric	7	1485(0.08002743797873557)	1121 (0.1)	1233 (0.1)
carbon	7	1485(0.08002743797873557)	2359 (0.034)	999 (0.13)
carcass	7	1485(0.08002743797873557)	2359 (0.034)	1668 (0.066)
cases	7	671(0.2057848405167486)	520 (0.24)	1668 (0.066)
cats	7	1485(0.08002743797873557)	1516 (0.069)	1668 (0.066)
chemistry	7	1485(0.08002743797873557)	1516 (0.069)	816 (0.16)
cholesterol	7	671(0.2057848405167486)	520 (0.24)	999 (0.13)
circadian	7	1485(0.08002743797873557)	595 (0.21)	2548 (0.033)
coastal	7	1485(0.08002743797873557)	1121 (0.1)	2548 (0.033)
colony	7	1485(0.08002743797873557)	1121 (0.1)	2548 (0.033)
combination	7	1485(0.08002743797873557)	1516 (0.069)	999 (0.13)
comfort	7	1485(0.08002743797873557)	2359 (0.034)	816 (0.16)
common	7	377(0.3658397164742197)	520 (0.24)	333 (0.43)
communities	7	628(0.21721733165656798)	520 (0.24)	584 (0.23)
comorbidity	7	1485(0.08002743797873557)	1516 (0.069)	1233 (0.1)

comparing	7	787(0.1714873670972905)	520 (0.24)	999 (0.13)
compensatory	7	1219(0.1028924202583743)	520 (0.24)	1668 (0.066)
compliance	7	1485(0.08002743797873557)	1121 (0.1)	1668 (0.066)
comprehensive	7	350(0.3887046987538585)	520 (0.24)	388 (0.36)
concept	7	1485(0.08002743797873557)	1516 (0.069)	816 (0.16)
contrasting	7	1485(0.08002743797873557)	1121 (0.1)	1233 (0.1)
cortex	7	1485(0.08002743797873557)	1516 (0.069)	1233 (0.1)
crevicular	7	787(0.1714873670972905)	520 (0.24)	694 (0.2)
crossbred	7	1485(0.08002743797873557)	#SAKNAS!	1233 (0.1)
cystic	7	1485(0.08002743797873557)	2359 (0.034)	#SAKNAS!
day	7	1485(0.08002743797873557)	897 (0.14)	1668 (0.066)
days	7	1485(0.08002743797873557)	2359 (0.034)	1668 (0.066)
death	7	896(0.14862238481765175)	520 (0.24)	1233 (0.1)
decreases	7	1485(0.08002743797873557)	1516 (0.069)	2548 (0.033)
definitive	7	1485(0.08002743797873557)	2359 (0.034)	1233 (0.1)
dentistry	7	836(0.16005487595747114)	520 (0.24)	1233 (0.1)
description	7	1485(0.08002743797873557)	1516 (0.069)	999 (0.13)
dietetic	7	1335(0.09145992911855493)	520 (0.24)	2548 (0.033)
differential	7	836(0.16005487595747114)	520 (0.24)	999 (0.13)
dilution	7	1485(0.08002743797873557)	595 (0.21)	2548 (0.033)
dissection	7	1485(0.08002743797873557)	1121 (0.1)	2548 (0.033)
distinct	7	1485(0.08002743797873557)	1516 (0.069)	1233 (0.1)
dose	7	1485(0.08002743797873557)	1516 (0.069)	1668 (0.066)
double-blind	7	1485(0.08002743797873557)	2359 (0.034)	1233 (0.1)
drosophila	7	309(0.43443466331313596)	520 (0.24)	205 (0.66)
dutch	7	1485(0.08002743797873557)	2359 (0.034)	1233 (0.1)
east	7	1485(0.08002743797873557)	1516 (0.069)	1668 (0.066)
effectiveness	7	500(0.2743797873556648)	520 (0.24)	461 (0.3)
elective	7	1485(0.08002743797873557)	1516 (0.069)	1668 (0.066)
elevation	7	1485(0.08002743797873557)	1121 (0.1)	1233 (0.1)
end-stage	7	1485(0.08002743797873557)	1516 (0.069)	1233 (0.1)
energetic	7	1485(0.08002743797873557)	1516 (0.069)	999 (0.13)
environments	7	1485(0.08002743797873557)	1516 (0.069)	1668 (0.066)
esophagectomy	7	462(0.2972447696353035)	520 (0.24)	333 (0.43)
europe	7	1485(0.08002743797873557)	#SAKNAS!	694 (0.2)
evolutionary	7	1485(0.08002743797873557)	897 (0.14)	1668 (0.066)
exploring	7	362(0.37727220761403907)	520 (0.24)	584 (0.23)
failure	7	350(0.3887046987538585)	520 (0.24)	361 (0.4)
fecundity	7	1485(0.08002743797873557)	1121 (0.1)	1668 (0.066)
fetal	7	1485(0.08002743797873557)	1516 (0.069)	999 (0.13)
fibre	7	1485(0.08002743797873557)	1121 (0.1)	1668 (0.066)
flexible	7	1485(0.08002743797873557)	897 (0.14)	#SAKNAS!
fourth	7	1485(0.08002743797873557)	897 (0.14)	1233 (0.1)

fracture	7	1485(0.08002743797873557)	2359 (0.034)	1233 (0.1)
fractures	7	1485(0.08002743797873557)	#SAKNAS!	1668 (0.066)
free-living	7	1485(0.08002743797873557)	1121 (0.1)	1668 (0.066)
french	7	1485(0.08002743797873557)	2359 (0.034)	999 (0.13)
gain	7	362(0.37727220761403907)	520 (0.24)	333 (0.43)
gingivalis	7	628(0.21721733165656798)	520 (0.24)	526 (0.26)
grazing	7	595(0.22864982279638732)	520 (0.24)	816 (0.16)
green	7	1485(0.08002743797873557)	1121 (0.1)	999 (0.13)
groups	7	595(0.22864982279638732)	520 (0.24)	694 (0.2)
having	7	1485(0.08002743797873557)	2359 (0.034)	1233 (0.1)
hip	7	1485(0.08002743797873557)	2359 (0.034)	1668 (0.066)
history	7	439(0.30867726077512286)	520 (0.24)	388 (0.36)
hong	7	1485(0.08002743797873557)	1516 (0.069)	816 (0.16)
hygienists	7	1485(0.08002743797873557)	2359 (0.034)	1233 (0.1)
hypertension	7	410(0.33154224305476165)	520 (0.24)	694 (0.2)
hyposalivation	7	1485(0.08002743797873557)	1516 (0.069)	999 (0.13)
identify	7	1485(0.08002743797873557)	1516 (0.069)	2548 (0.033)
idiopathic	7	1485(0.08002743797873557)	1516 (0.069)	1668 (0.066)
imaging	7	1485(0.08002743797873557)	1516 (0.069)	1233 (0.1)
independence	7	1485(0.08002743797873557)	#SAKNAS!	1668 (0.066)
indexes	7	1485(0.08002743797873557)	1516 (0.069)	2548 (0.033)
individual	7	671(0.2057848405167486)	520 (0.24)	816 (0.16)
injection	7	1485(0.08002743797873557)	1121 (0.1)	999 (0.13)
injury	7	481(0.2858122784954842)	520 (0.24)	526 (0.26)
intellectual	7	1485(0.08002743797873557)	2359 (0.034)	816 (0.16)
intensity	7	1485(0.08002743797873557)	2359 (0.034)	1668 (0.066)
interposition	7	1485(0.08002743797873557)	1121 (0.1)	999 (0.13)
inversely	7	1485(0.08002743797873557)	1516 (0.069)	816 (0.16)
iranian	7	1485(0.08002743797873557)	1516 (0.069)	1668 (0.066)
ireland	7	1485(0.08002743797873557)	2359 (0.034)	1233 (0.1)
islands	7	1485(0.08002743797873557)	1121 (0.1)	#SAKNAS!
junction	7	1485(0.08002743797873557)	1516 (0.069)	1233 (0.1)
kong	7	1485(0.08002743797873557)	1516 (0.069)	816 (0.16)
laser	7	1485(0.08002743797873557)	1516 (0.069)	999 (0.13)
laying	7	1485(0.08002743797873557)	2359 (0.034)	1233 (0.1)
learning	7	427(0.3201097519149423)	520 (0.24)	816 (0.16)
lesion	7	1485(0.08002743797873557)	2359 (0.034)	999 (0.13)
limitation	7	1485(0.08002743797873557)	2359 (0.034)	999 (0.13)
lip	7	1485(0.08002743797873557)	1516 (0.069)	2548 (0.033)
locust	7	1485(0.08002743797873557)	717 (0.17)	1668 (0.066)
maintain	7	1485(0.08002743797873557)	2359 (0.034)	1233 (0.1)
making	7	1485(0.08002743797873557)	#SAKNAS!	584 (0.23)
mammalian	7	1485(0.08002743797873557)	1516 (0.069)	1233 (0.1)

marginal	7	1485(0.08002743797873557)	1516 (0.069)	999 (0.13)
marine	7	671(0.2057848405167486)	520 (0.24)	694 (0.2)
matching	7	1485(0.08002743797873557)	#SAKNAS!	1233 (0.1)
matrix	7	1485(0.08002743797873557)	897 (0.14)	1668 (0.066)
maximum	7	1485(0.08002743797873557)	1516 (0.069)	2548 (0.033)
meal	7	305(0.4458671544529553)	520 (0.24)	251 (0.56)
mediate	7	1485(0.08002743797873557)	1516 (0.069)	1233 (0.1)
medication	7	1485(0.08002743797873557)	#SAKNAS!	2548 (0.033)
mineral	7	595(0.22864982279638732)	520 (0.24)	694 (0.2)
modelling	7	1485(0.08002743797873557)	2359 (0.034)	999 (0.13)
modifiable	7	1485(0.08002743797873557)	2359 (0.034)	2548 (0.033)
molecular	7	1485(0.08002743797873557)	2359 (0.034)	1233 (0.1)
morbidly	7	966(0.1371898936778324)	520 (0.24)	1668 (0.066)
motility	7	743(0.18291985823710985)	520 (0.24)	1233 (0.1)
multimorbidity	7	1485(0.08002743797873557)	#SAKNAS!	2548 (0.033)
mussel	7	1485(0.08002743797873557)	1121 (0.1)	1233 (0.1)
nasal	7	1485(0.08002743797873557)	1516 (0.069)	2548 (0.033)
nigeria	7	1485(0.08002743797873557)	717 (0.17)	2548 (0.033)
noninvasive	7	1485(0.08002743797873557)	717 (0.17)	1668 (0.066)
norway	7	1485(0.08002743797873557)	2359 (0.034)	1668 (0.066)
number	7	159(0.777409397507717)	520 (0.24)	130 (1)
nutritionally	7	1485(0.08002743797873557)	897 (0.14)	1668 (0.066)
nutritive	7	1485(0.08002743797873557)	897 (0.14)	1668 (0.066)
occupational	7	1485(0.08002743797873557)	#SAKNAS!	999 (0.13)
operation	7	1485(0.08002743797873557)	897 (0.14)	1668 (0.066)
opportunities	7	1485(0.08002743797873557)	1516 (0.069)	1668 (0.066)
orexin	7	1485(0.08002743797873557)	897 (0.14)	1233 (0.1)
overdentures	7	1485(0.08002743797873557)	717 (0.17)	#SAKNAS!
oxygen	7	1485(0.08002743797873557)	1516 (0.069)	1668 (0.066)
panel	7	1485(0.08002743797873557)	1121 (0.1)	2548 (0.033)
paraesophageal	7	896(0.14862238481765175)	520 (0.24)	999 (0.13)
partial	7	836(0.16005487595747114)	520 (0.24)	1668 (0.066)
participating	7	1485(0.08002743797873557)	2359 (0.034)	1233 (0.1)
perinatal	7	1485(0.08002743797873557)	1121 (0.1)	999 (0.13)
peroral	7	1485(0.08002743797873557)	#SAKNAS!	816 (0.16)
phenotype	7	1485(0.08002743797873557)	1121 (0.1)	2548 (0.033)
phenotypes	7	1485(0.08002743797873557)	#SAKNAS!	1668 (0.066)
plaque	7	559(0.2400823139362067)	520 (0.24)	694 (0.2)
pollen	7	1485(0.08002743797873557)	1121 (0.1)	1668 (0.066)
polymorphism	7	595(0.22864982279638732)	520 (0.24)	333 (0.43)
polymorphisms	7	836(0.16005487595747114)	520 (0.24)	999 (0.13)
porphyromonas	7	628(0.21721733165656798)	520 (0.24)	526 (0.26)
practitioners	7	1485(0.08002743797873557)	2359 (0.034)	1233 (0.1)

predator	7	1485(0.08002743797873557)	897 (0.14)	1668 (0.066)
predatory	7	1485(0.08002743797873557)	1516 (0.069)	1233 (0.1)
preoperative	7	410(0.33154224305476165)	520 (0.24)	461 (0.3)
prevents	7	1485(0.08002743797873557)	#SAKNAS!	1233 (0.1)
processes	7	1485(0.08002743797873557)	1121 (0.1)	1668 (0.066)
product	7	1485(0.08002743797873557)	1516 (0.069)	#SAKNAS!
production	7	535(0.2515148050760261)	520 (0.24)	584 (0.23)
professional	7	1485(0.08002743797873557)	#SAKNAS!	816 (0.16)
progress	7	1485(0.08002743797873557)	2359 (0.034)	2548 (0.033)
promotes	7	1485(0.08002743797873557)	1516 (0.069)	1233 (0.1)
prophylaxis	7	1485(0.08002743797873557)	1121 (0.1)	2548 (0.033)
providers	7	1485(0.08002743797873557)	2359 (0.034)	1233 (0.1)
providing	7	1485(0.08002743797873557)	2359 (0.034)	694 (0.2)
racial	7	1485(0.08002743797873557)	1516 (0.069)	1668 (0.066)
rates	7	787(0.1714873670972905)	520 (0.24)	816 (0.16)
rct	7	1485(0.08002743797873557)	#SAKNAS!	999 (0.13)
reduce	7	559(0.2400823139362067)	520 (0.24)	816 (0.16)
reducing	7	966(0.1371898936778324)	520 (0.24)	999 (0.13)
refeeding	7	1485(0.08002743797873557)	717 (0.17)	2548 (0.033)
regional	7	1485(0.08002743797873557)	1516 (0.069)	999 (0.13)
regions	7	1485(0.08002743797873557)	897 (0.14)	2548 (0.033)
regulate	7	1485(0.08002743797873557)	2359 (0.034)	1668 (0.066)
responsive	7	1485(0.08002743797873557)	2359 (0.034)	#SAKNAS!
retention	7	1485(0.08002743797873557)	897 (0.14)	1668 (0.066)
revision	7	1485(0.08002743797873557)	2359 (0.034)	1233 (0.1)
rice	7	1485(0.08002743797873557)	1516 (0.069)	#SAKNAS!
right	7	1485(0.08002743797873557)	1121 (0.1)	1668 (0.066)
safe	7	710(0.19435234937692925)	520 (0.24)	816 (0.16)
safety	7	319(0.4230021721733166)	520 (0.24)	205 (0.66)
satiety	7	1485(0.08002743797873557)	1516 (0.069)	1233 (0.1)
scaling	7	1485(0.08002743797873557)	#SAKNAS!	1233 (0.1)
school-age	7	1485(0.08002743797873557)	#SAKNAS!	1668 (0.066)
season	7	1485(0.08002743797873557)	1121 (0.1)	1233 (0.1)
sedentary	7	1485(0.08002743797873557)	2359 (0.034)	2548 (0.033)
selective	7	896(0.14862238481765175)	520 (0.24)	816 (0.16)
significance	7	966(0.1371898936778324)	520 (0.24)	1233 (0.1)
signs	7	1485(0.08002743797873557)	2359 (0.034)	999 (0.13)
simultaneous	7	1485(0.08002743797873557)	717 (0.17)	2548 (0.033)
six	7	1485(0.08002743797873557)	#SAKNAS!	1233 (0.1)
smokers	7	1485(0.08002743797873557)	#SAKNAS!	694 (0.2)
socio-demographic	7	1485(0.08002743797873557)	1516 (0.069)	999 (0.13)
solid	7	1485(0.08002743797873557)	2359 (0.034)	694 (0.2)
spine	7	710(0.19435234937692925)	520 (0.24)	816 (0.16)

spontaneous		7	966(0.1371898936778324)	520 (0.24)	999 (0.13)
strains		7	1485(0.08002743797873557)	595 (0.21)	#SAKNAS!
stricture		7	787(0.1714873670972905)	520 (0.24)	584 (0.23)
stunting		7	1485(0.08002743797873557)	1516 (0.069)	1668 (0.066)
sugars		7	535(0.2515148050760261)	520 (0.24)	422 (0.33)
supplemental		7	1485(0.08002743797873557)	1516 (0.069)	2548 (0.033)
supplementary		7	1485(0.08002743797873557)	2359 (0.034)	999 (0.13)
supplements		7	535(0.2515148050760261)	520 (0.24)	584 (0.23)
surveillance		7	836(0.16005487595747114)	520 (0.24)	694 (0.2)
syndromes		7	1485(0.08002743797873557)	2359 (0.034)	1668 (0.066)
taiwan		7	628(0.21721733165656798)	520 (0.24)	694 (0.2)
techniques		7	1485(0.08002743797873557)	1121 (0.1)	1233 (0.1)
teenagers		7	1485(0.08002743797873557)	897 (0.14)	2548 (0.033)
temporal		7	628(0.21721733165656798)	520 (0.24)	694 (0.2)
tephritidae		7	1485(0.08002743797873557)	520 (0.24)	#SAKNAS!
tephritidae		7	1485(0.08002743797873557)	520 (0.24)	#SAKNAS!
tolerance		7	1485(0.08002743797873557)	1516 (0.069)	1233 (0.1)
tool		7	350(0.3887046987538585)	520 (0.24)	205 (0.66)
toxins		7	1485(0.08002743797873557)	595 (0.21)	2548 (0.033)
trajectories		7	1485(0.08002743797873557)	2359 (0.034)	2548 (0.033)
transcranial		7	1485(0.08002743797873557)	#SAKNAS!	2548 (0.033)
trauma		7	1485(0.08002743797873557)	1516 (0.069)	#SAKNAS!
uganda		7	1485(0.08002743797873557)	1121 (0.1)	1668 (0.066)
underlying		7	1485(0.08002743797873557)	1121 (0.1)	1233 (0.1)
unhealthy		7	1485(0.08002743797873557)	#SAKNAS!	#SAKNAS!
unusual		7	1485(0.08002743797873557)	1516 (0.069)	1233 (0.1)
variables		7	535(0.2515148050760261)	520 (0.24)	461 (0.3)
vegetation		7	1485(0.08002743797873557)	#SAKNAS!	1233 (0.1)
veterans		7	1485(0.08002743797873557)	1121 (0.1)	1668 (0.066)
views		7	1485(0.08002743797873557)	2359 (0.034)	999 (0.13)
virulence		7	1485(0.08002743797873557)	897 (0.14)	1668 (0.066)
vivo		7	1485(0.08002743797873557)	1516 (0.069)	#SAKNAS!
voluntary		7	1485(0.08002743797873557)	1121 (0.1)	1668 (0.066)
waist		7	1485(0.08002743797873557)	897 (0.14)	2548 (0.033)
wards		7	1485(0.08002743797873557)	1516 (0.069)	1668 (0.066)
weaning		7	1027(0.12575740253801304)	520 (0.24)	1233 (0.1)
white		7	710(0.19435234937692925)	520 (0.24)	694 (0.2)
wic		7	787(0.1714873670972905)	520 (0.24)	1233 (0.1)
xanthan		7	1485(0.08002743797873557)	#SAKNAS!	1233 (0.1)
yeast		7	1485(0.08002743797873557)	1516 (0.069)	1668 (0.066)
younger		7	1485(0.08002743797873557)	1516 (0.069)	1233 (0.1)
	2013	6	1678(0.0685949468389162)	2359 (0.034)	1668 (0.066)
	2016	6	1678(0.0685949468389162)	#SAKNAS!	999 (0.13)

	45721	6	1678(0.0685949468389162)	1516 (0.069)	1233 (0.1)
3-year		6	1678(0.0685949468389162)	1516 (0.069)	1668 (0.066)
6-year		6	1678(0.0685949468389162)	2359 (0.034)	2548 (0.033)
abnormalities		6	1111(0.11432491139819366)	595 (0.21)	2548 (0.033)
academy		6	1678(0.0685949468389162)	897 (0.14)	2548 (0.033)
access		6	671(0.2057848405167486)	595 (0.21)	526 (0.26)
accumulation		6	1678(0.0685949468389162)	1516 (0.069)	1668 (0.066)
activation		6	1678(0.0685949468389162)	2359 (0.034)	1668 (0.066)
activities		6	427(0.3201097519149423)	595 (0.21)	422 (0.33)
adaptation		6	559(0.2400823139362067)	595 (0.21)	584 (0.23)
adaptive		6	1678(0.0685949468389162)	717 (0.17)	#SAKNAS!
adenocarcinoma		6	1678(0.0685949468389162)	1121 (0.1)	1233 (0.1)
adolescent		6	481(0.2858122784954842)	595 (0.21)	461 (0.3)
adulthood		6	1678(0.0685949468389162)	1516 (0.069)	1233 (0.1)
adverse		6	966(0.1371898936778324)	595 (0.21)	2548 (0.033)
advice		6	710(0.19435234937692925)	595 (0.21)	999 (0.13)
affecting		6	396(0.342974734194581)	595 (0.21)	584 (0.23)
alcohol		6	595(0.22864982279638732)	595 (0.21)	422 (0.33)
algal		6	1678(0.0685949468389162)	717 (0.17)	2548 (0.033)
algorithm		6	1027(0.12575740253801304)	595 (0.21)	1668 (0.066)
allergic		6	1678(0.0685949468389162)	1516 (0.069)	1668 (0.066)
alone		6	1678(0.0685949468389162)	2359 (0.034)	1233 (0.1)
alter		6	1678(0.0685949468389162)	1516 (0.069)	999 (0.13)
alterations		6	671(0.2057848405167486)	595 (0.21)	694 (0.2)
ambulatory		6	1678(0.0685949468389162)	717 (0.17)	#SAKNAS!
amino		6	628(0.21721733165656798)	595 (0.21)	999 (0.13)
apnea		6	628(0.21721733165656798)	595 (0.21)	461 (0.3)
artery		6	671(0.2057848405167486)	595 (0.21)	694 (0.2)
arthritis		6	500(0.2743797873556648)	595 (0.21)	461 (0.3)
assessments		6	1678(0.0685949468389162)	1121 (0.1)	1668 (0.066)
assimilation		6	1678(0.0685949468389162)	717 (0.17)	#SAKNAS!
associates		6	1678(0.0685949468389162)	#SAKNAS!	1668 (0.066)
assurance		6	1678(0.0685949468389162)	1121 (0.1)	1233 (0.1)
atresia		6	595(0.22864982279638732)	595 (0.21)	461 (0.3)
audit		6	1219(0.1028924202583743)	595 (0.21)	1233 (0.1)
bariatric		6	410(0.33154224305476165)	595 (0.21)	361 (0.4)
barium		6	1678(0.0685949468389162)	717 (0.17)	#SAKNAS!
beetle		6	1027(0.12575740253801304)	595 (0.21)	999 (0.13)
bidirectional		6	1678(0.0685949468389162)	2359 (0.034)	1668 (0.066)
biomarker		6	1678(0.0685949468389162)	1121 (0.1)	2548 (0.033)
bite		6	1678(0.0685949468389162)	2359 (0.034)	2548 (0.033)
born		6	1678(0.0685949468389162)	1121 (0.1)	1233 (0.1)
budget		6	1678(0.0685949468389162)	1121 (0.1)	1668 (0.066)

burden	6	439(0.30867726077512286)	595 (0.21)	388 (0.36)
burn	6	1678(0.0685949468389162)	1121 (0.1)	2548 (0.033)
cactus	6	1678(0.0685949468389162)	2359 (0.034)	1233 (0.1)
captive	6	1027(0.12575740253801304)	595 (0.21)	999 (0.13)
caterpillars	6	1678(0.0685949468389162)	897 (0.14)	2548 (0.033)
causes	6	787(0.1714873670972905)	595 (0.21)	816 (0.16)
centre	6	1678(0.0685949468389162)	2359 (0.034)	1233 (0.1)
cessation	6	1678(0.0685949468389162)	2359 (0.034)	999 (0.13)
characterization	6	535(0.2515148050760261)	595 (0.21)	422 (0.33)
chemotherapy	6	362(0.37727220761403907)	595 (0.21)	333 (0.43)
chile	6	1678(0.0685949468389162)	897 (0.14)	#SAKNAS!
cigarette	6	1219(0.1028924202583743)	595 (0.21)	1668 (0.066)
circadian	6	1485(0.08002743797873557)	595 (0.21)	2548 (0.033)
classification	6	787(0.1714873670972905)	595 (0.21)	1668 (0.066)
clinically	6	1678(0.0685949468389162)	2359 (0.034)	999 (0.13)
cognition	6	1678(0.0685949468389162)	#SAKNAS!	1668 (0.066)
colombia	6	1678(0.0685949468389162)	1516 (0.069)	1233 (0.1)
comparisons	6	1678(0.0685949468389162)	#SAKNAS!	1233 (0.1)
consecutive	6	1678(0.0685949468389162)	1516 (0.069)	1233 (0.1)
construct	6	1678(0.0685949468389162)	2359 (0.034)	1233 (0.1)
context	6	628(0.21721733165656798)	595 (0.21)	422 (0.33)
contribute	6	1678(0.0685949468389162)	1516 (0.069)	1668 (0.066)
conversion	6	1678(0.0685949468389162)	2359 (0.034)	1668 (0.066)
coronal	6	1678(0.0685949468389162)	1516 (0.069)	999 (0.13)
correlate	6	1678(0.0685949468389162)	1121 (0.1)	1668 (0.066)
correlates	6	481(0.2858122784954842)	595 (0.21)	361 (0.4)
correlations	6	1678(0.0685949468389162)	#SAKNAS!	1668 (0.066)
corrosive	6	1678(0.0685949468389162)	#SAKNAS!	999 (0.13)
cost	6	1678(0.0685949468389162)	1516 (0.069)	1668 (0.066)
costs	6	671(0.2057848405167486)	595 (0.21)	694 (0.2)
countries	6	896(0.14862238481765175)	595 (0.21)	999 (0.13)
country	6	1678(0.0685949468389162)	1121 (0.1)	1668 (0.066)
cross-cultural	6	1678(0.0685949468389162)	2359 (0.034)	1668 (0.066)
curative	6	1678(0.0685949468389162)	897 (0.14)	1668 (0.066)
cytokine	6	1678(0.0685949468389162)	1516 (0.069)	2548 (0.033)
damage	6	1678(0.0685949468389162)	897 (0.14)	2548 (0.033)
decision	6	1678(0.0685949468389162)	1516 (0.069)	2548 (0.033)
decision-making	6	1678(0.0685949468389162)	1121 (0.1)	1668 (0.066)
defects	6	1111(0.11432491139819366)	595 (0.21)	2548 (0.033)
deficit	6	1678(0.0685949468389162)	1121 (0.1)	1233 (0.1)
demographic	6	1678(0.0685949468389162)	1121 (0.1)	#SAKNAS!
dentist	6	1678(0.0685949468389162)	1121 (0.1)	1668 (0.066)
dentists	6	787(0.1714873670972905)	595 (0.21)	816 (0.16)

department	6	1678(0.0685949468389162)	2359 (0.034)	1233 (0.1)
design	6	516(0.26294729621584545)	595 (0.21)	305 (0.46)
device	6	1678(0.0685949468389162)	2359 (0.034)	1668 (0.066)
diabetic	6	481(0.2858122784954842)	595 (0.21)	388 (0.36)
difference	6	1678(0.0685949468389162)	#SAKNAS!	999 (0.13)
differentiation	6	1678(0.0685949468389162)	1121 (0.1)	1668 (0.066)
dilatation	6	1678(0.0685949468389162)	1121 (0.1)	1233 (0.1)
dilution	6	1485(0.08002743797873557)	595 (0.21)	2548 (0.033)
diurnal	6	1678(0.0685949468389162)	897 (0.14)	#SAKNAS!
diverse	6	1678(0.0685949468389162)	2359 (0.034)	1668 (0.066)
diverticulum	6	1678(0.0685949468389162)	1516 (0.069)	1233 (0.1)
dmft	6	1678(0.0685949468389162)	2359 (0.034)	#SAKNAS!
domestic	6	1678(0.0685949468389162)	897 (0.14)	#SAKNAS!
dopamine	6	1678(0.0685949468389162)	1516 (0.069)	1668 (0.066)
dry	6	559(0.2400823139362067)	595 (0.21)	422 (0.33)
dryness	6	1678(0.0685949468389162)	2359 (0.034)	1668 (0.066)
ecosystem	6	1678(0.0685949468389162)	1516 (0.069)	1233 (0.1)
efficacy	6	331(0.4115696810334972)	595 (0.21)	333 (0.43)
el	6	1678(0.0685949468389162)	#SAKNAS!	1233 (0.1)
emergency	6	1678(0.0685949468389162)	2359 (0.034)	1668 (0.066)
endoscopy	6	1678(0.0685949468389162)	1121 (0.1)	2548 (0.033)
enhancing	6	1678(0.0685949468389162)	#SAKNAS!	2548 (0.033)
epidemiology	6	896(0.14862238481765175)	595 (0.21)	999 (0.13)
erosive	6	1678(0.0685949468389162)	#SAKNAS!	1668 (0.066)
esophagitis	6	516(0.26294729621584545)	595 (0.21)	461 (0.3)
events	6	896(0.14862238481765175)	595 (0.21)	694 (0.2)
exercises	6	1678(0.0685949468389162)	2359 (0.034)	1233 (0.1)
expanding	6	1678(0.0685949468389162)	#SAKNAS!	1233 (0.1)
expenditure	6	787(0.1714873670972905)	595 (0.21)	1233 (0.1)
expert	6	1678(0.0685949468389162)	1516 (0.069)	999 (0.13)
extent	6	1678(0.0685949468389162)	897 (0.14)	1668 (0.066)
external	6	1678(0.0685949468389162)	2359 (0.034)	2548 (0.033)
extracts	6	1678(0.0685949468389162)	717 (0.17)	2548 (0.033)
familial	6	1678(0.0685949468389162)	1121 (0.1)	1233 (0.1)
families	6	836(0.16005487595747114)	595 (0.21)	999 (0.13)
finnish	6	1678(0.0685949468389162)	2359 (0.034)	1233 (0.1)
fixation	6	1678(0.0685949468389162)	#SAKNAS!	2548 (0.033)
flap	6	787(0.1714873670972905)	595 (0.21)	2548 (0.033)
flies	6	1678(0.0685949468389162)	1121 (0.1)	#SAKNAS!
fluorosis	6	1678(0.0685949468389162)	1516 (0.069)	1668 (0.066)
focus	6	1678(0.0685949468389162)	2359 (0.034)	1668 (0.066)
follow	6	1678(0.0685949468389162)	2359 (0.034)	816 (0.16)
formula	6	710(0.19435234937692925)	595 (0.21)	816 (0.16)

fresh	6	1678(0.0685949468389162)	1516 (0.069)	1668 (0.066)
gastrojejunostomy	6	1678(0.0685949468389162)	2359 (0.034)	1233 (0.1)
generalist	6	896(0.14862238481765175)	595 (0.21)	816 (0.16)
generation	6	1678(0.0685949468389162)	#SAKNAS!	2548 (0.033)
germany	6	896(0.14862238481765175)	595 (0.21)	1233 (0.1)
gestational	6	595(0.22864982279638732)	595 (0.21)	584 (0.23)
ghana	6	1678(0.0685949468389162)	717 (0.17)	2548 (0.033)
girls	6	1678(0.0685949468389162)	1516 (0.069)	1233 (0.1)
goat	6	966(0.1371898936778324)	595 (0.21)	1233 (0.1)
grain	6	1678(0.0685949468389162)	2359 (0.034)	1233 (0.1)
grass	6	1678(0.0685949468389162)	#SAKNAS!	999 (0.13)
greater	6	1678(0.0685949468389162)	1516 (0.069)	999 (0.13)
greek	6	1678(0.0685949468389162)	1121 (0.1)	2548 (0.033)
gross	6	1678(0.0685949468389162)	717 (0.17)	2548 (0.033)
growing	6	966(0.1371898936778324)	595 (0.21)	1668 (0.066)
guide	6	1678(0.0685949468389162)	1516 (0.069)	999 (0.13)
heat	6	1678(0.0685949468389162)	1121 (0.1)	1668 (0.066)
hematopoietic	6	1678(0.0685949468389162)	#SAKNAS!	816 (0.16)
hens	6	1678(0.0685949468389162)	2359 (0.034)	1233 (0.1)
herbivorous	6	1678(0.0685949468389162)	1121 (0.1)	1233 (0.1)
high-fat	6	710(0.19435234937692925)	595 (0.21)	422 (0.33)
hippocampal	6	1678(0.0685949468389162)	2359 (0.034)	1233 (0.1)
homeostasis	6	1219(0.1028924202583743)	595 (0.21)	1668 (0.066)
hospitals	6	1678(0.0685949468389162)	#SAKNAS!	1233 (0.1)
hosts	6	1678(0.0685949468389162)	897 (0.14)	#SAKNAS!
hunger	6	1678(0.0685949468389162)	1121 (0.1)	1668 (0.066)
hydrocolloids	6	1678(0.0685949468389162)	#SAKNAS!	#SAKNAS!
hyperglycemia	6	1678(0.0685949468389162)	2359 (0.034)	999 (0.13)
iddsi	6	1678(0.0685949468389162)	#SAKNAS!	2548 (0.033)
impairment	6	362(0.37727220761403907)	595 (0.21)	333 (0.43)
implant	6	628(0.21721733165656798)	595 (0.21)	584 (0.23)
improvement	6	516(0.26294729621584545)	595 (0.21)	816 (0.16)
inclusion	6	1678(0.0685949468389162)	#SAKNAS!	1233 (0.1)
increases	6	516(0.26294729621584545)	595 (0.21)	388 (0.36)
independently	6	836(0.16005487595747114)	595 (0.21)	694 (0.2)
indian	6	1111(0.11432491139819366)	595 (0.21)	1233 (0.1)
induces	6	1678(0.0685949468389162)	1516 (0.069)	999 (0.13)
inhibitor	6	1678(0.0685949468389162)	1121 (0.1)	1668 (0.066)
injuries	6	1678(0.0685949468389162)	1516 (0.069)	1668 (0.066)
innovative	6	1678(0.0685949468389162)	#SAKNAS!	1668 (0.066)
integration	6	1335(0.09145992911855493)	595 (0.21)	1668 (0.066)
interaction	6	427(0.3201097519149423)	595 (0.21)	333 (0.43)
intestinal	6	966(0.1371898936778324)	595 (0.21)	2548 (0.033)

intima-media	6 1678(0.0685949468389162)	1516 (0.069)	1668 (0.066)
intracerebral	6 1678(0.0685949468389162)	2359 (0.034)	1233 (0.1)
intrinsic	6 1678(0.0685949468389162)	1516 (0.069)	1668 (0.066)
inverse	6 1678(0.0685949468389162)	2359 (0.034)	1668 (0.066)
iran	6 1111(0.11432491139819366)	595 (0.21)	1668 (0.066)
juvenile	6 671(0.2057848405167486)	595 (0.21)	584 (0.23)
key	6 1027(0.12575740253801304)	595 (0.21)	2548 (0.033)
kidney	6 481(0.2858122784954842)	595 (0.21)	461 (0.3)
la	6 1678(0.0685949468389162)	1516 (0.069)	999 (0.13)
laboratory	6 896(0.14862238481765175)	595 (0.21)	1233 (0.1)
labrax	6 1678(0.0685949468389162)	595 (0.21)	#SAKNAS!
labrax	6 1678(0.0685949468389162)	595 (0.21)	#SAKNAS!
laryngeal	6 896(0.14862238481765175)	595 (0.21)	1233 (0.1)
larynx	6 1678(0.0685949468389162)	2359 (0.034)	1233 (0.1)
lead	6 787(0.1714873670972905)	595 (0.21)	584 (0.23)
lepidoptera	6 1335(0.09145992911855493)	595 (0.21)	1668 (0.066)
lifestyle	6 268(0.4801646278724134)	595 (0.21)	228 (0.59)
literacy	6 1678(0.0685949468389162)	1516 (0.069)	2548 (0.033)
literature	6 1111(0.11432491139819366)	595 (0.21)	2548 (0.033)
lobster	6 1678(0.0685949468389162)	2359 (0.034)	1233 (0.1)
long	6 377(0.3658397164742197)	595 (0.21)	264 (0.53)
longevity	6 1335(0.09145992911855493)	595 (0.21)	#SAKNAS!
lunch	6 1678(0.0685949468389162)	2359 (0.034)	1233 (0.1)
macronutrients	6 1678(0.0685949468389162)	1516 (0.069)	1668 (0.066)
manduca	6 1678(0.0685949468389162)	717 (0.17)	2548 (0.033)
mangrove	6 1678(0.0685949468389162)	1121 (0.1)	1233 (0.1)
mating	6 1678(0.0685949468389162)	1516 (0.069)	1668 (0.066)
meat	6 1678(0.0685949468389162)	2359 (0.034)	2548 (0.033)
mediterranean	6 439(0.30867726077512286)	595 (0.21)	422 (0.33)
methodology	6 1678(0.0685949468389162)	1516 (0.069)	1668 (0.066)
microbiota	6 224(0.5716245569909684)	595 (0.21)	251 (0.56)
microorganisms	6 1678(0.0685949468389162)	2359 (0.034)	1233 (0.1)
mixtures	6 1678(0.0685949468389162)	2359 (0.034)	2548 (0.033)
models	6 787(0.1714873670972905)	595 (0.21)	694 (0.2)
modifications	6 1678(0.0685949468389162)	717 (0.17)	#SAKNAS!
molars	6 1678(0.0685949468389162)	1121 (0.1)	1668 (0.066)
mountain	6 1678(0.0685949468389162)	2359 (0.034)	1233 (0.1)
mrna	6 1678(0.0685949468389162)	717 (0.17)	2548 (0.033)
mucositis	6 559(0.2400823139362067)	595 (0.21)	422 (0.33)
multi-center	6 1678(0.0685949468389162)	2359 (0.034)	1668 (0.066)
mussels	6 1678(0.0685949468389162)	717 (0.17)	#SAKNAS!
neoadjuvant	6 836(0.16005487595747114)	595 (0.21)	694 (0.2)
neonatal	6 1027(0.12575740253801304)	595 (0.21)	1233 (0.1)

night	6	1678(0.0685949468389162)	1516 (0.069)	1668 (0.066)
nonsurgical	6	1678(0.0685949468389162)	1516 (0.069)	1233 (0.1)
northern	6	427(0.3201097519149423)	595 (0.21)	361 (0.4)
nurses	6	1678(0.0685949468389162)	1121 (0.1)	1668 (0.066)
online	6	1678(0.0685949468389162)	2359 (0.034)	1233 (0.1)
opportunistic	6	1678(0.0685949468389162)	2359 (0.034)	1668 (0.066)
osteoarthritis	6	1678(0.0685949468389162)	2359 (0.034)	1233 (0.1)
outlet	6	1678(0.0685949468389162)	1121 (0.1)	1668 (0.066)
pairs	6	1678(0.0685949468389162)	2359 (0.034)	1668 (0.066)
palate	6	1678(0.0685949468389162)	897 (0.14)	#SAKNAS!
palliation	6	966(0.1371898936778324)	595 (0.21)	816 (0.16)
parenteral	6	1027(0.12575740253801304)	595 (0.21)	816 (0.16)
parotid	6	1678(0.0685949468389162)	1516 (0.069)	1233 (0.1)
participation	6	1678(0.0685949468389162)	1121 (0.1)	#SAKNAS!
pasture	6	1678(0.0685949468389162)	#SAKNAS!	1233 (0.1)
pathogens	6	671(0.2057848405167486)	595 (0.21)	584 (0.23)
paulo	6	1678(0.0685949468389162)	717 (0.17)	2548 (0.033)
perforation	6	1678(0.0685949468389162)	717 (0.17)	2548 (0.033)
persons	6	439(0.30867726077512286)	595 (0.21)	461 (0.3)
peru	6	1678(0.0685949468389162)	717 (0.17)	#SAKNAS!
phase	6	396(0.342974734194581)	595 (0.21)	422 (0.33)
plasticity	6	1678(0.0685949468389162)	1516 (0.069)	2548 (0.033)
possible	6	439(0.30867726077512286)	595 (0.21)	422 (0.33)
postoperative	6	293(0.45729964559277464)	595 (0.21)	333 (0.43)
potato	6	1678(0.0685949468389162)	1516 (0.069)	1668 (0.066)
powder	6	1678(0.0685949468389162)	#SAKNAS!	2548 (0.033)
prader-willi	6	1678(0.0685949468389162)	897 (0.14)	1668 (0.066)
preservation	6	1678(0.0685949468389162)	2359 (0.034)	1233 (0.1)
private	6	1678(0.0685949468389162)	2359 (0.034)	1668 (0.066)
procedure	6	787(0.1714873670972905)	595 (0.21)	694 (0.2)
products	6	516(0.26294729621584545)	595 (0.21)	584 (0.23)
project	6	410(0.33154224305476165)	595 (0.21)	305 (0.46)
prostheses	6	1111(0.11432491139819366)	595 (0.21)	2548 (0.033)
protocol	6	188(0.685949468389162)	595 (0.21)	194 (0.69)
psychiatric	6	1678(0.0685949468389162)	2359 (0.034)	1233 (0.1)
puerto	6	1678(0.0685949468389162)	1516 (0.069)	999 (0.13)
quantification	6	1678(0.0685949468389162)	2359 (0.034)	816 (0.16)
quantity	6	1678(0.0685949468389162)	897 (0.14)	2548 (0.033)
quasi-experimental	6	1678(0.0685949468389162)	#SAKNAS!	1233 (0.1)
radiologic	6	1678(0.0685949468389162)	1516 (0.069)	1668 (0.066)
rapid	6	1678(0.0685949468389162)	897 (0.14)	#SAKNAS!
rare	6	1111(0.11432491139819366)	595 (0.21)	1668 (0.066)
ration	6	1678(0.0685949468389162)	1516 (0.069)	1233 (0.1)

raw	6	1678(0.0685949468389162)	2359 (0.034)	#SAKNAS!
reactive	6	1678(0.0685949468389162)	2359 (0.034)	1668 (0.066)
refugee	6	1678(0.0685949468389162)	1516 (0.069)	1233 (0.1)
regulating	6	1678(0.0685949468389162)	1121 (0.1)	1668 (0.066)
reproduction	6	1219(0.1028924202583743)	595 (0.21)	1233 (0.1)
republic	6	1678(0.0685949468389162)	1516 (0.069)	1233 (0.1)
resource	6	1027(0.12575740253801304)	595 (0.21)	1668 (0.066)
respiratory	6	462(0.2972447696353035)	595 (0.21)	526 (0.26)
restoration	6	1678(0.0685949468389162)	717 (0.17)	#SAKNAS!
reveal	6	1678(0.0685949468389162)	#SAKNAS!	816 (0.16)
reveals	6	1678(0.0685949468389162)	#SAKNAS!	2548 (0.033)
rheumatoid	6	559(0.2400823139362067)	595 (0.21)	461 (0.3)
robotic	6	1678(0.0685949468389162)	#SAKNAS!	999 (0.13)
ruminal	6	1678(0.0685949468389162)	2359 (0.034)	1233 (0.1)
ruminants	6	1678(0.0685949468389162)	717 (0.17)	2548 (0.033)
salmon	6	1678(0.0685949468389162)	1121 (0.1)	1233 (0.1)
salvage	6	1678(0.0685949468389162)	#SAKNAS!	999 (0.13)
sao	6	1678(0.0685949468389162)	717 (0.17)	2548 (0.033)
scientific	6	1678(0.0685949468389162)	2359 (0.034)	1233 (0.1)
setting	6	535(0.2515148050760261)	595 (0.21)	422 (0.33)
sexta	6	1678(0.0685949468389162)	717 (0.17)	2548 (0.033)
signals	6	1219(0.1028924202583743)	595 (0.21)	1668 (0.066)
smell	6	1678(0.0685949468389162)	#SAKNAS!	1668 (0.066)
smile	6	1678(0.0685949468389162)	#SAKNAS!	1668 (0.066)
snack	6	1678(0.0685949468389162)	2359 (0.034)	1233 (0.1)
soft	6	743(0.18291985823710985)	595 (0.21)	694 (0.2)
source	6	1678(0.0685949468389162)	1121 (0.1)	1668 (0.066)
sources	6	559(0.2400823139362067)	595 (0.21)	388 (0.36)
southwest	6	1678(0.0685949468389162)	2359 (0.034)	999 (0.13)
spain	6	896(0.14862238481765175)	595 (0.21)	1233 (0.1)
starvation	6	1678(0.0685949468389162)	1121 (0.1)	1668 (0.066)
stay	6	1678(0.0685949468389162)	1516 (0.069)	1668 (0.066)
stomach	6	1678(0.0685949468389162)	1516 (0.069)	1233 (0.1)
strains	6	1485(0.08002743797873557)	595 (0.21)	#SAKNAS!
strategy	6	439(0.30867726077512286)	595 (0.21)	388 (0.36)
streptococci	6	896(0.14862238481765175)	595 (0.21)	999 (0.13)
styles	6	1678(0.0685949468389162)	1121 (0.1)	2548 (0.033)
surveys	6	710(0.19435234937692925)	595 (0.21)	816 (0.16)
suspension	6	1678(0.0685949468389162)	1121 (0.1)	#SAKNAS!
swallowing-related	6	1678(0.0685949468389162)	#SAKNAS!	2548 (0.033)
sympatric	6	1335(0.09145992911855493)	595 (0.21)	#SAKNAS!
symptom	6	836(0.16005487595747114)	595 (0.21)	1668 (0.066)
systematic	6	342(0.40013718989367786)	595 (0.21)	422 (0.33)

taiwanese	6	1678(0.0685949468389162)	2359 (0.034)	1233 (0.1)
team	6	1678(0.0685949468389162)	#SAKNAS!	1668 (0.066)
temporomandibular	6	1678(0.0685949468389162)	1516 (0.069)	1668 (0.066)
therapies	6	1678(0.0685949468389162)	1516 (0.069)	#SAKNAS!
thickening	6	1678(0.0685949468389162)	#SAKNAS!	2548 (0.033)
thyroid	6	1678(0.0685949468389162)	1516 (0.069)	1668 (0.066)
tobacco	6	559(0.2400823139362067)	595 (0.21)	584 (0.23)
toddlers	6	481(0.2858122784954842)	595 (0.21)	280 (0.49)
tool-10	6	1678(0.0685949468389162)	2359 (0.034)	1233 (0.1)
toward	6	1678(0.0685949468389162)	2359 (0.034)	1233 (0.1)
toxic	6	1678(0.0685949468389162)	897 (0.14)	2548 (0.033)
toxins	6	1485(0.08002743797873557)	595 (0.21)	2548 (0.033)
training	6	305(0.4458671544529553)	595 (0.21)	264 (0.53)
trajectory	6	1678(0.0685949468389162)	1516 (0.069)	2548 (0.033)
transporter	6	1678(0.0685949468389162)	1121 (0.1)	2548 (0.033)
trials	6	1678(0.0685949468389162)	1516 (0.069)	1233 (0.1)
trophic	6	836(0.16005487595747114)	595 (0.21)	999 (0.13)
trout	6	1678(0.0685949468389162)	897 (0.14)	#SAKNAS!
tumor	6	1111(0.11432491139819366)	595 (0.21)	1668 (0.066)
ulcers	6	1678(0.0685949468389162)	1516 (0.069)	1668 (0.066)
undernutrition	6	462(0.2972447696353035)	595 (0.21)	305 (0.46)
usefulness	6	1678(0.0685949468389162)	1516 (0.069)	2548 (0.033)
vertical	6	896(0.14862238481765175)	595 (0.21)	1233 (0.1)
video	6	1678(0.0685949468389162)	1121 (0.1)	2548 (0.033)
vietnam	6	1678(0.0685949468389162)	#SAKNAS!	999 (0.13)
visit	6	1678(0.0685949468389162)	1516 (0.069)	1668 (0.066)
wearers	6	1678(0.0685949468389162)	1121 (0.1)	#SAKNAS!
whole	6	896(0.14862238481765175)	595 (0.21)	999 (0.13)
why	6	1027(0.12575740253801304)	595 (0.21)	816 (0.16)
workers	6	671(0.2057848405167486)	595 (0.21)	816 (0.16)
workshop	6	1678(0.0685949468389162)	#SAKNAS!	999 (0.13)
world	6	1678(0.0685949468389162)	1516 (0.069)	1668 (0.066)
xerostomia	6	410(0.33154224305476165)	595 (0.21)	305 (0.46)
youth	6	1678(0.0685949468389162)	1516 (0.069)	1668 (0.066)
zenker	6	1678(0.0685949468389162)	1121 (0.1)	1668 (0.066)
2017	5	1894(0.05716245569909683)	#SAKNAS!	1668 (0.066)
2020	5	1894(0.05716245569909683)	#SAKNAS!	2548 (0.033)
45820	5	1894(0.05716245569909683)	#SAKNAS!	1668 (0.066)
&lt	5	1894(0.05716245569909683)	#SAKNAS!	1233 (0.1)
10-year	5	1894(0.05716245569909683)	#SAKNAS!	1668 (0.066)
2009-2010	5	1894(0.05716245569909683)	#SAKNAS!	999 (0.13)
2009-2012	5	1894(0.05716245569909683)	#SAKNAS!	1233 (0.1)
2010-2012	5	1894(0.05716245569909683)	#SAKNAS!	816 (0.16)

2015-2018	5	1894(0.0571624556990968З)	#SAKNAS!	#SAKNAS!
25-hydroxyvitamin	5	1111(0.11432491139819366)	717 (0.17)	1233 (0.1)
2-year	5	1894(0.05716245569909683)	1516 (0.069)	#SAKNAS!
abdominal	5	836(0.16005487595747114)	717 (0.17)	1233 (0.1)
aboriginal	5	1894(0.05716245569909683)	2359 (0.034)	1233 (0.1)
absorption	5	1111(0.11432491139819366)	717 (0.17)	816 (0.16)
abuse	5	1894(0.05716245569909683)	1121 (0.1)	1668 (0.066)
acyrthosiphon	5	1894(0.05716245569909683)	#SAKNAS!	#SAKNAS!
adaptive	5	1678(0.0685949468389162)	717 (0.17)	#SAKNAS!
adhd	5	1894(0.05716245569909683)	2359 (0.034)	1233 (0.1)
adiponectin	5	1894(0.05716245569909683)	2359 (0.034)	999 (0.13)
advances	5	1894(0.05716245569909683)	1516 (0.069)	2548 (0.033)
agents	5	1219(0.1028924202583743)	717 (0.17)	1668 (0.066)
ages	5	1894(0.05716245569909683)	#SAKNAS!	1233 (0.1)
aggregatibacter	5	1894(0.05716245569909683)	1516 (0.069)	1668 (0.066)
agrp	5	1894(0.05716245569909683)	1516 (0.069)	2548 (0.033)
airway	5	671(0.2057848405167486)	717 (0.17)	461 (0.3)
albumin	5	966(0.1371898936778324)	717 (0.17)	999 (0.13)
algal	5	1678(0.0685949468389162)	717 (0.17)	2548 (0.033)
alimentary	5	1894(0.05716245569909683)	1121 (0.1)	1668 (0.066)
allostatic	5	1894(0.05716245569909683)	1516 (0.069)	1668 (0.066)
ambulatory	5	1678(0.0685949468389162)	717 (0.17)	#SAKNAS!
america	5	1894(0.05716245569909683)	1516 (0.069)	2548 (0.033)
americans	5	1485(0.08002743797873557)	717 (0.17)	2548 (0.033)
animals	5	1894(0.05716245569909683)	1121 (0.1)	2548 (0.033)
ant	5	1027(0.12575740253801304)	717 (0.17)	1233 (0.1)
antibody	5	1485(0.08002743797873557)	717 (0.17)	1668 (0.066)
antimicrobial	5	1894(0.05716245569909683)	2359 (0.034)	2548 (0.033)
antireflux	5	1027(0.12575740253801304)	717 (0.17)	816 (0.16)
aphthous	5	1894(0.05716245569909683)	1516 (0.069)	1668 (0.066)
apparent	5	1894(0.05716245569909683)	2359 (0.034)	2548 (0.033)
applications	5	1894(0.05716245569909683)	#SAKNAS!	#SAKNAS!
appropriate	5	1894(0.05716245569909683)	1516 (0.069)	1668 (0.066)
aquaculture	5	1894(0.05716245569909683)	1516 (0.069)	1668 (0.066)
arctic	5	1894(0.05716245569909683)	1121 (0.1)	1668 (0.066)
article	5	1894(0.05716245569909683)	1516 (0.069)	1233 (0.1)
artificial	5	671(0.2057848405167486)	717 (0.17)	999 (0.13)
assimilation	5	1678(0.0685949468389162)	717 (0.17)	#SAKNAS!
atherogenic	5	1894(0.05716245569909683)	1121 (0.1)	2548 (0.033)
atlantic	5	1219(0.1028924202583743)	717 (0.17)	999 (0.13)
atrial	5	1894(0.05716245569909683)	#SAKNAS!	1668 (0.066)
attachment	5	896(0.14862238481765175)	717 (0.17)	816 (0.16)
attributes	5	1894(0.05716245569909683)	1516 (0.069)	1668 (0.066)

australia	5	896(0.14862238481765175)	717 (0.17)	1233 (0.1)
autologous	5	1894(0.05716245569909683)	1516 (0.069)	2548 (0.033)
automatic	5	1894(0.05716245569909683)	1516 (0.069)	2548 (0.033)
aversion	5	1894(0.05716245569909683)	1516 (0.069)	2548 (0.033)
axis	5	1894(0.05716245569909683)	#SAKNAS!	2548 (0.033)
balancing	5	1894(0.05716245569909683)	1121 (0.1)	1668 (0.066)
barium	5	1678(0.0685949468389162)	717 (0.17)	#SAKNAS!
base	5	1894(0.05716245569909683)	1121 (0.1)	2548 (0.033)
behavioural	5	535(0.2515148050760261)	717 (0.17)	461 (0.3)
beliefs	5	1219(0.1028924202583743)	717 (0.17)	1668 (0.066)
beneficial	5	1894(0.05716245569909683)	897 (0.14)	#SAKNAS!
binding	5	1894(0.05716245569909683)	717 (0.17)	#SAKNAS!
binding	5	1894(0.05716245569909683)	717 (0.17)	#SAKNAS!
biochemical	5	1111(0.11432491139819366)	717 (0.17)	1233 (0.1)
biology	5	1335(0.09145992911855493)	717 (0.17)	1668 (0.066)
boys	5	1894(0.05716245569909683)	#SAKNAS!	1668 (0.066)
brainstem	5	1894(0.05716245569909683)	1121 (0.1)	2548 (0.033)
bream	5	1894(0.05716245569909683)	717 (0.17)	#SAKNAS!
bream	5	1894(0.05716245569909683)	717 (0.17)	#SAKNAS!
brief	5	1894(0.05716245569909683)	2359 (0.034)	2548 (0.033)
britain	5	1894(0.05716245569909683)	1121 (0.1)	2548 (0.033)
broiler	5	1894(0.05716245569909683)	1516 (0.069)	2548 (0.033)
browsing	5	1894(0.05716245569909683)	1121 (0.1)	2548 (0.033)
butterfly	5	1894(0.05716245569909683)	1516 (0.069)	2548 (0.033)
calculus	5	1894(0.05716245569909683)	1516 (0.069)	#SAKNAS!
california	5	1894(0.05716245569909683)	#SAKNAS!	999 (0.13)
call	5	1894(0.05716245569909683)	2359 (0.034)	1233 (0.1)
cambodia	5	1894(0.05716245569909683)	#SAKNAS!	999 (0.13)
candida	5	1894(0.05716245569909683)	1516 (0.069)	#SAKNAS!
cannabinoid	5	1894(0.05716245569909683)	#SAKNAS!	1233 (0.1)
carbohydrate	5	671(0.2057848405167486)	717 (0.17)	584 (0.23)
cariogenic	5	671(0.2057848405167486)	717 (0.17)	694 (0.2)
carious	5	1894(0.05716245569909683)	1516 (0.069)	2548 (0.033)
carotid	5	1219(0.1028924202583743)	717 (0.17)	1668 (0.066)
cattle	5	595(0.22864982279638732)	717 (0.17)	694 (0.2)
cause	5	787(0.1714873670972905)	717 (0.17)	1233 (0.1)
cavity	5	836(0.16005487595747114)	717 (0.17)	999 (0.13)
challenges	5	836(0.16005487595747114)	717 (0.17)	816 (0.16)
checklist	5	1894(0.05716245569909683)	#SAKNAS!	2548 (0.033)
chemosensory	5	1894(0.05716245569909683)	2359 (0.034)	1233 (0.1)
chicken	5	1894(0.05716245569909683)	2359 (0.034)	1668 (0.066)
chickens	5	1219(0.1028924202583743)	717 (0.17)	1668 (0.066)
chief	5	1894(0.05716245569909683)	#SAKNAS!	#SAKNAS!

childbearing	5	1894(0.0571624556990968З)	1516 (0.069)	1668 (0.066)
child-feeding	5	1335(0.0914599291185549З)	717 (0.17)	1668 (0.066)
city	5	628(0.21721733165656798)	717 (0.17)	461 (0.3)
clients	5	1894(0.0571624556990968З)	1516 (0.069)	1233 (0.1)
cluster-randomized	5	1894(0.0571624556990968З)	2359 (0.034)	1233 (0.1)
college	5	1335(0.0914599291185549З)	717 (0.17)	1233 (0.1)
colon	5	1894(0.0571624556990968З)	1121 (0.1)	1668 (0.066)
colonies	5	1894(0.0571624556990968З)	2359 (0.034)	2548 (0.033)
colorectal	5	1894(0.0571624556990968З)	#SAKNAS!	1233 (0.1)
competition	5	1894(0.0571624556990968З)	1516 (0.069)	2548 (0.033)
complication	5	1027(0.12575740253801304)	717 (0.17)	1668 (0.066)
composite	5	1894(0.0571624556990968З)	2359 (0.034)	#SAKNAS!
concerns	5	1894(0.0571624556990968З)	#SAKNAS!	999 (0.13)
concomitant	5	1894(0.0571624556990968З)	2359 (0.034)	1668 (0.066)
connectivity	5	1894(0.0571624556990968З)	#SAKNAS!	1233 (0.1)
considerations	5	836(0.16005487595747114)	717 (0.17)	816 (0.16)
consistency	5	1894(0.0571624556990968З)	1516 (0.069)	1233 (0.1)
consuming	5	1219(0.1028924202583743)	717 (0.17)	1668 (0.066)
containing	5	1027(0.12575740253801304)	717 (0.17)	1668 (0.066)
continuous	5	836(0.16005487595747114)	717 (0.17)	694 (0.2)
coral	5	1894(0.0571624556990968З)	2359 (0.034)	999 (0.13)
cord	5	1894(0.0571624556990968З)	2359 (0.034)	2548 (0.033)
cost-effectiveness	5	1894(0.0571624556990968З)	#SAKNAS!	999 (0.13)
counts	5	1894(0.0571624556990968З)	1121 (0.1)	1668 (0.066)
cricopharyngeal	5	836(0.16005487595747114)	717 (0.17)	816 (0.16)
crp	5	1894(0.0571624556990968З)	1516 (0.069)	1668 (0.066)
cues	5	1335(0.0914599291185549З)	717 (0.17)	1668 (0.066)
decade	5	1894(0.0571624556990968З)	#SAKNAS!	2548 (0.033)
deep	5	1894(0.0571624556990968З)	#SAKNAS!	999 (0.13)
defense	5	1894(0.0571624556990968З)	2359 (0.034)	1233 (0.1)
deletion	5	1894(0.0571624556990968З)	#SAKNAS!	816 (0.16)
dentures	5	896(0.14862238481765175)	717 (0.17)	816 (0.16)
derivation	5	1894(0.0571624556990968З)	897 (0.14)	#SAKNAS!
desert	5	1894(0.0571624556990968З)	1121 (0.1)	1668 (0.066)
determining	5	1894(0.0571624556990968З)	2359 (0.034)	1233 (0.1)
developing	5	836(0.16005487595747114)	717 (0.17)	816 (0.16)
developmental	5	710(0.19435234937692925)	717 (0.17)	526 (0.26)
devices	5	1894(0.0571624556990968З)	#SAKNAS!	#SAKNAS!
diagnostics	5	1111(0.11432491139819366)	717 (0.17)	999 (0.13)
dicentrarchus	5	1894(0.0571624556990968З)	717 (0.17)	#SAKNAS!
dicentrarchus	5	1894(0.0571624556990968З)	717 (0.17)	#SAKNAS!
digestion	5	1027(0.12575740253801304)	717 (0.17)	816 (0.16)
discomfort	5	1894(0.0571624556990968З)	#SAKNAS!	1233 (0.1)

disorder	5	309(0.43443466331313596)	717 (0.17)	194 (0.69)
disturbance	5	1894(0.05716245569909683)	#SAKNAS!	#SAKNAS!
dogs	5	1894(0.05716245569909683)	1516 (0.069)	1233 (0.1)
dominance	5	1894(0.05716245569909683)	1121 (0.1)	1668 (0.066)
down	5	1894(0.05716245569909683)	#SAKNAS!	1233 (0.1)
drinks	5	896(0.14862238481765175)	717 (0.17)	816 (0.16)
drug	5	1219(0.1028924202583743)	717 (0.17)	1233 (0.1)
duration	5	396(0.342974734194581)	717 (0.17)	361 (0.4)
dwelling	5	1894(0.05716245569909683)	#SAKNAS!	1233 (0.1)
dysphagia-oriented	5	1894(0.05716245569909683)	2359 (0.034)	#SAKNAS!
dystrophic	5	1894(0.05716245569909683)	897 (0.14)	2548 (0.033)
eat-10	5	1894(0.05716245569909683)	2359 (0.034)	1668 (0.066)
economic	5	1894(0.05716245569909683)	#SAKNAS!	1668 (0.066)
edible	5	1894(0.05716245569909683)	#SAKNAS!	1668 (0.066)
egyptian	5	1894(0.05716245569909683)	2359 (0.034)	#SAKNAS!
electronic	5	1894(0.05716245569909683)	2359 (0.034)	1233 (0.1)
elite	5	1894(0.05716245569909683)	2359 (0.034)	1233 (0.1)
emerging	5	1894(0.05716245569909683)	1516 (0.069)	2548 (0.033)
enamel	5	896(0.14862238481765175)	717 (0.17)	1668 (0.066)
endovascular	5	1894(0.05716245569909683)	#SAKNAS!	1233 (0.1)
england	5	1894(0.05716245569909683)	1121 (0.1)	2548 (0.033)
enhances	5	1894(0.05716245569909683)	#SAKNAS!	1668 (0.066)
enzyme	5	1894(0.05716245569909683)	1516 (0.069)	1233 (0.1)
eruption	5	1894(0.05716245569909683)	897 (0.14)	2548 (0.033)
esophagogastric	5	1894(0.05716245569909683)	1516 (0.069)	#SAKNAS!
establishing	5	1894(0.05716245569909683)	2359 (0.034)	1668 (0.066)
estimate	5	1894(0.05716245569909683)	2359 (0.034)	2548 (0.033)
estimates	5	1219(0.1028924202583743)	717 (0.17)	999 (0.13)
estimation	5	1894(0.05716245569909683)	#SAKNAS!	1668 (0.066)
evolution	5	710(0.19435234937692925)	717 (0.17)	584 (0.23)
ewes	5	1894(0.05716245569909683)	2359 (0.034)	1668 (0.066)
examining	5	896(0.14862238481765175)	717 (0.17)	1233 (0.1)
excretion	5	1894(0.05716245569909683)	1516 (0.069)	1233 (0.1)
experiments	5	1894(0.05716245569909683)	897 (0.14)	2548 (0.033)
explorative	5	1894(0.05716245569909683)	2359 (0.034)	1668 (0.066)
extract	5	1335(0.09145992911855493)	717 (0.17)	1233 (0.1)
extracts	5	1678(0.0685949468389162)	717 (0.17)	2548 (0.033)
facial	5	1894(0.05716245569909683)	1516 (0.069)	2548 (0.033)
facility	5	1894(0.05716245569909683)	1516 (0.069)	#SAKNAS!
factor-帢	5	1894(0.05716245569909683)	717 (0.17)	#SAKNAS!
factor-帢	5	1894(0.05716245569909683)	717 (0.17)	#SAKNAS!
females	5	743(0.18291985823710985)	717 (0.17)	584 (0.23)
fiberoptic	5	1894(0.05716245569909683)	2359 (0.034)	2548 (0.033)

fibrillation	5	1894(0.0571624556990968З)	#SAKNAS!	1668 (0.066)
field	5	1111(0.11432491139819366)	717 (0.17)	1233 (0.1)
fluids	5	1894(0.05716245569909683)	1516 (0.069)	2548 (0.033)
fluoridation	5	1894(0.05716245569909683)	1516 (0.069)	1233 (0.1)
food-based	5	1894(0.05716245569909683)	1121 (0.1)	2548 (0.033)
fto	5	1894(0.05716245569909683)	1516 (0.069)	1233 (0.1)
fungal	5	1894(0.05716245569909683)	1516 (0.069)	#SAKNAS!
gastro-esophageal	5	1894(0.05716245569909683)	1516 (0.069)	2548 (0.033)
gastro-oesophageal	5	1335(0.09145992911855493)	717 (0.17)	1233 (0.1)
genetics	5	1894(0.05716245569909683)	1516 (0.069)	1668 (0.066)
genotype	5	1894(0.05716245569909683)	1121 (0.1)	#SAKNAS!
german	5	743(0.18291985823710985)	717 (0.17)	526 (0.26)
ghana	5	1678(0.0685949468389162)	717 (0.17)	2548 (0.033)
given	5	1894(0.05716245569909683)	897 (0.14)	2548 (0.033)
gland	5	836(0.16005487595747114)	717 (0.17)	816 (0.16)
glands	5	1894(0.05716245569909683)	2359 (0.034)	2548 (0.033)
glycaemic	5	1894(0.05716245569909683)	2359 (0.034)	1668 (0.066)
grasshopper	5	1894(0.05716245569909683)	897 (0.14)	2548 (0.033)
gravis	5	1894(0.05716245569909683)	1516 (0.069)	#SAKNAS!
great	5	1335(0.09145992911855493)	717 (0.17)	1668 (0.066)
gross	5	1678(0.0685949468389162)	717 (0.17)	2548 (0.033)
guangzhou	5	1894(0.05716245569909683)	#SAKNAS!	1233 (0.1)
hamburg	5	1894(0.05716245569909683)	#SAKNAS!	2548 (0.033)
health-promoting	5	1894(0.05716245569909683)	1121 (0.1)	2548 (0.033)
heavy	5	1894(0.05716245569909683)	2359 (0.034)	2548 (0.033)
heifers	5	1894(0.05716245569909683)	2359 (0.034)	1668 (0.066)
hemoglobin	5	1027(0.12575740253801304)	717 (0.17)	1233 (0.1)
herbivory	5	1894(0.05716245569909683)	#SAKNAS!	999 (0.13)
hernias	5	1894(0.05716245569909683)	1121 (0.1)	#SAKNAS!
high-sensitivity	5	1894(0.05716245569909683)	2359 (0.034)	1668 (0.066)
hiv	5	1335(0.09145992911855493)	717 (0.17)	2548 (0.033)
homes	5	481(0.2858122784954842)	717 (0.17)	388 (0.36)
honey	5	896(0.14862238481765175)	717 (0.17)	584 (0.23)
hours	5	1894(0.05716245569909683)	2359 (0.034)	2548 (0.033)
housing	5	1894(0.05716245569909683)	897 (0.14)	2548 (0.033)
huntington	5	1894(0.05716245569909683)	1121 (0.1)	2548 (0.033)
hybrid	5	1894(0.05716245569909683)	#SAKNAS!	1233 (0.1)
hypertensive	5	1894(0.05716245569909683)	#SAKNAS!	1668 (0.066)
hyperuricemia	5	1894(0.05716245569909683)	#SAKNAS!	1668 (0.066)
hypothalamus	5	1111(0.11432491139819366)	717 (0.17)	1668 (0.066)
hypothesis	5	1894(0.05716245569909683)	1121 (0.1)	1668 (0.066)
icu	5	1894(0.05716245569909683)	#SAKNAS!	1668 (0.066)
illness	5	1894(0.05716245569909683)	1516 (0.069)	2548 (0.033)

immigrant	5	1894(0.0571624556990968З)	1516 (0.069)	1668 (0.066)
implant-supported	5	1894(0.05716245569909683)	1516 (0.069)	#SAKNAS!
improving	5	671(0.2057848405167486)	717 (0.17)	694 (0.2)
imrt	5	1894(0.05716245569909683)	#SAKNAS!	999 (0.13)
income	5	966(0.1371898936778324)	717 (0.17)	999 (0.13)
individually	5	1894(0.05716245569909683)	1121 (0.1)	2548 (0.033)
industry	5	1894(0.05716245569909683)	#SAKNAS!	1668 (0.066)
infancy	5	787(0.1714873670972905)	717 (0.17)	694 (0.2)
infarction	5	671(0.2057848405167486)	717 (0.17)	526 (0.26)
inhibition	5	1894(0.05716245569909683)	1516 (0.069)	2548 (0.033)
inoperable	5	1894(0.05716245569909683)	1121 (0.1)	1668 (0.066)
inpatients	5	396(0.342974734194581)	717 (0.17)	461 (0.3)
insertion	5	1111(0.11432491139819366)	717 (0.17)	1233 (0.1)
institution	5	1894(0.05716245569909683)	#SAKNAS!	816 (0.16)
instrument	5	1894(0.05716245569909683)	1121 (0.1)	2548 (0.033)
integrating	5	1894(0.05716245569909683)	2359 (0.034)	1233 (0.1)
integrative	5	1894(0.05716245569909683)	2359 (0.034)	1668 (0.066)
interactive	5	1894(0.05716245569909683)	2359 (0.034)	1668 (0.066)
interleukin-6	5	1335(0.09145992911855493)	717 (0.17)	1668 (0.066)
intermittent	5	1894(0.05716245569909683)	1516 (0.069)	#SAKNAS!
interventional	5	1894(0.05716245569909683)	2359 (0.034)	#SAKNAS!
interventions	5	439(0.30867726077512286)	717 (0.17)	461 (0.3)
intolerance	5	1894(0.05716245569909683)	#SAKNAS!	1233 (0.1)
intrauterine	5	1894(0.05716245569909683)	717 (0.17)	#SAKNAS!
intrauterine	5	1894(0.05716245569909683)	717 (0.17)	#SAKNAS!
iron	5	743(0.18291985823710985)	717 (0.17)	816 (0.16)
ischaemic	5	1894(0.05716245569909683)	1516 (0.069)	1668 (0.066)
ischemic	5	410(0.33154224305476165)	717 (0.17)	526 (0.26)
isotopes	5	1894(0.05716245569909683)	897 (0.14)	2548 (0.033)
items	5	1335(0.09145992911855493)	717 (0.17)	#SAKNAS!
jejunostomy	5	1335(0.09145992911855493)	717 (0.17)	1233 (0.1)
kenya	5	1894(0.05716245569909683)	#SAKNAS!	1233 (0.1)
lactation	5	743(0.18291985823710985)	717 (0.17)	461 (0.3)
language	5	1219(0.1028924202583743)	717 (0.17)	1668 (0.066)
latinos	5	1894(0.05716245569909683)	#SAKNAS!	816 (0.16)
leads	5	1894(0.05716245569909683)	2359 (0.034)	1668 (0.066)
leaf	5	671(0.2057848405167486)	717 (0.17)	461 (0.3)
leaves	5	1894(0.05716245569909683)	1121 (0.1)	1668 (0.066)
lemur	5	1894(0.05716245569909683)	897 (0.14)	2548 (0.033)
lemurs	5	1894(0.05716245569909683)	717 (0.17)	#SAKNAS!
lemurs	5	1894(0.05716245569909683)	717 (0.17)	#SAKNAS!
length	5	1111(0.11432491139819366)	717 (0.17)	1668 (0.066)
lesions	5	628(0.21721733165656798)	717 (0.17)	526 (0.26)

light	5	1894(0.05716245569909683)	2359 (0.034)	2548 (0.033)
limb	5	1894(0.05716245569909683)	#SAKNAS!	2548 (0.033)
litter	5	1894(0.05716245569909683)	1121 (0.1)	2548 (0.033)
load	5	1027(0.12575740253801304)	717 (0.17)	999 (0.13)
lockdown	5	1894(0.05716245569909683)	#SAKNAS!	#SAKNAS!
locomotor	5	1894(0.05716245569909683)	897 (0.14)	#SAKNAS!
locust	5	1485(0.08002743797873557)	717 (0.17)	1668 (0.066)
main	5	1894(0.05716245569909683)	#SAKNAS!	1233 (0.1)
malignancies	5	1894(0.05716245569909683)	2359 (0.034)	1233 (0.1)
mandible	5	1894(0.05716245569909683)	2359 (0.034)	2548 (0.033)
manduca	5	1678(0.0685949468389162)	717 (0.17)	2548 (0.033)
manifestations	5	1111(0.11432491139819366)	717 (0.17)	1233 (0.1)
mapping	5	1894(0.05716245569909683)	#SAKNAS!	999 (0.13)
matters	5	1894(0.05716245569909683)	1516 (0.069)	1668 (0.066)
meals	5	671(0.2057848405167486)	717 (0.17)	694 (0.2)
measuring	5	1111(0.11432491139819366)	717 (0.17)	999 (0.13)
mechanism	5	1219(0.1028924202583743)	717 (0.17)	1668 (0.066)
mediators	5	1894(0.05716245569909683)	2359 (0.034)	1668 (0.066)
melatonin	5	1894(0.05716245569909683)	1516 (0.069)	2548 (0.033)
menopausal	5	1894(0.05716245569909683)	#SAKNAS!	1668 (0.066)
messages	5	1894(0.05716245569909683)	1516 (0.069)	1668 (0.066)
metals	5	1894(0.05716245569909683)	2359 (0.034)	1668 (0.066)
mexico	5	671(0.2057848405167486)	717 (0.17)	526 (0.26)
microbial	5	896(0.14862238481765175)	717 (0.17)	1233 (0.1)
migration	5	1894(0.05716245569909683)	1516 (0.069)	1668 (0.066)
military	5	1894(0.05716245569909683)	1516 (0.069)	#SAKNAS!
minerals	5	1894(0.05716245569909683)	#SAKNAS!	1668 (0.066)
minimally	5	1894(0.05716245569909683)	2359 (0.034)	1668 (0.066)
minimum	5	1894(0.05716245569909683)	2359 (0.034)	#SAKNAS!
mitochondrial	5	1894(0.05716245569909683)	#SAKNAS!	1668 (0.066)
mixing	5	1894(0.05716245569909683)	1121 (0.1)	#SAKNAS!
modifications	5	1678(0.0685949468389162)	717 (0.17)	#SAKNAS!
module	5	1894(0.05716245569909683)	2359 (0.034)	2548 (0.033)
movement	5	1894(0.05716245569909683)	1516 (0.069)	1668 (0.066)
mrna	5	1678(0.0685949468389162)	717 (0.17)	2548 (0.033)
mucosal	5	1219(0.1028924202583743)	717 (0.17)	1668 (0.066)
municipality	5	1894(0.05716245569909683)	#SAKNAS!	1233 (0.1)
mussels	5	1678(0.0685949468389162)	717 (0.17)	#SAKNAS!
myasthenia	5	1894(0.05716245569909683)	1516 (0.069)	#SAKNAS!
mytilus	5	1894(0.05716245569909683)	897 (0.14)	2548 (0.033)
nature	5	1894(0.05716245569909683)	1121 (0.1)	#SAKNAS!
necrosis	5	1111(0.11432491139819366)	717 (0.17)	1668 (0.066)
necrotizing	5	1894(0.05716245569909683)	897 (0.14)	#SAKNAS!

nellore	5	1894(0.05716245569909683)	#SAKNAS!	1668 (0.066)
neurologic	5	1894(0.05716245569909683)	897 (0.14)	#SAKNAS!
neurological	5	559(0.2400823139362067)	717 (0.17)	584 (0.23)
neurone	5	1894(0.05716245569909683)	1121 (0.1)	1668 (0.066)
newborns	5	1894(0.05716245569909683)	1516 (0.069)	1668 (0.066)
nigeria	5	1485(0.08002743797873557)	717 (0.17)	2548 (0.033)
nocturnal	5	1894(0.05716245569909683)	897 (0.14)	#SAKNAS!
noninvasive	5	1485(0.08002743797873557)	717 (0.17)	1668 (0.066)
normal-weight	5	1894(0.05716245569909683)	2359 (0.034)	1668 (0.066)
normative	5	1894(0.05716245569909683)	1121 (0.1)	2548 (0.033)
obstruction	5	836(0.16005487595747114)	717 (0.17)	816 (0.16)
ocean	5	1894(0.05716245569909683)	#SAKNAS!	2548 (0.033)
oesophagectomy	5	1894(0.05716245569909683)	1516 (0.069)	1668 (0.066)
oesophagus	5	1894(0.05716245569909683)	897 (0.14)	2548 (0.033)
office	5	1894(0.05716245569909683)	1121 (0.1)	1668 (0.066)
oil	5	710(0.19435234937692925)	717 (0.17)	584 (0.23)
oldest	5	1894(0.05716245569909683)	1121 (0.1)	2548 (0.033)
oncological	5	1894(0.05716245569909683)	1516 (0.069)	#SAKNAS!
oncology	5	1335(0.09145992911855493)	717 (0.17)	1668 (0.066)
onset	5	671(0.2057848405167486)	717 (0.17)	694 (0.2)
options	5	1894(0.05716245569909683)	717 (0.17)	#SAKNAS!
options	5	1894(0.05716245569909683)	717 (0.17)	#SAKNAS!
organization	5	1894(0.05716245569909683)	1516 (0.069)	2548 (0.033)
origin	5	1894(0.05716245569909683)	897 (0.14)	2548 (0.033)
overdentures	5	1485(0.08002743797873557)	717 (0.17)	#SAKNAS!
overview	5	966(0.1371898936778324)	717 (0.17)	1233 (0.1)
oxide	5	1894(0.05716245569909683)	2359 (0.034)	#SAKNAS!
pacific	5	1894(0.05716245569909683)	#SAKNAS!	999 (0.13)
paper	5	1894(0.05716245569909683)	2359 (0.034)	1233 (0.1)
parasitoid	5	1894(0.05716245569909683)	1121 (0.1)	1668 (0.066)
parts	5	1894(0.05716245569909683)	1121 (0.1)	2548 (0.033)
pathogenesis	5	1894(0.05716245569909683)	897 (0.14)	2548 (0.033)
pathogenic	5	1894(0.05716245569909683)	2359 (0.034)	1668 (0.066)
pathologists	5	1894(0.05716245569909683)	2359 (0.034)	2548 (0.033)
paulo	5	1678(0.0685949468389162)	717 (0.17)	2548 (0.033)
peptide	5	1027(0.12575740253801304)	717 (0.17)	1233 (0.1)
perforation	5	1678(0.0685949468389162)	717 (0.17)	2548 (0.033)
peritoneal	5	1894(0.05716245569909683)	2359 (0.034)	1668 (0.066)
persistent	5	1335(0.09145992911855493)	717 (0.17)	1668 (0.066)
personal	5	1894(0.05716245569909683)	1121 (0.1)	#SAKNAS!
peru	5	1678(0.0685949468389162)	717 (0.17)	#SAKNAS!
photodynamic	5	1894(0.05716245569909683)	1516 (0.069)	#SAKNAS!
plan	5	1894(0.05716245569909683)	#SAKNAS!	1233 (0.1)

plastic	5	1894(0.05716245569909683)	897 (0.14)	2548 (0.033)
policy	5	1894(0.05716245569909683)	1121 (0.1)	2548 (0.033)
polish	5	1894(0.05716245569909683)	2359 (0.034)	1668 (0.066)
polypharmacy	5	1894(0.05716245569909683)	#SAKNAS!	2548 (0.033)
postnatal	5	1894(0.05716245569909683)	897 (0.14)	2548 (0.033)
poverty	5	1894(0.05716245569909683)	1516 (0.069)	1233 (0.1)
pre	5	1894(0.05716245569909683)	#SAKNAS!	1668 (0.066)
predation	5	1335(0.09145992911855493)	717 (0.17)	1233 (0.1)
prediabetes	5	1894(0.05716245569909683)	#SAKNAS!	1233 (0.1)
predicting	5	350(0.3887046987538585)	717 (0.17)	280 (0.49)
predicts	5	462(0.2972447696353035)	717 (0.17)	584 (0.23)
pre-school	5	966(0.1371898936778324)	717 (0.17)	999 (0.13)
preschool-aged	5	1894(0.05716245569909683)	1516 (0.069)	2548 (0.033)
preschoolers	5	896(0.14862238481765175)	717 (0.17)	816 (0.16)
pretreatment	5	1894(0.05716245569909683)	2359 (0.034)	999 (0.13)
preventive	5	710(0.19435234937692925)	717 (0.17)	584 (0.23)
probiotics	5	1894(0.05716245569909683)	1516 (0.069)	2548 (0.033)
procedures	5	1111(0.11432491139819366)	717 (0.17)	2548 (0.033)
productivity	5	1894(0.05716245569909683)	#SAKNAS!	#SAKNAS!
professionals	5	836(0.16005487595747114)	717 (0.17)	2548 (0.033)
programme	5	836(0.16005487595747114)	717 (0.17)	816 (0.16)
progressive	5	1894(0.05716245569909683)	2359 (0.034)	2548 (0.033)
promotion	5	535(0.2515148050760261)	717 (0.17)	422 (0.33)
protein-energy	5	1894(0.05716245569909683)	1121 (0.1)	1668 (0.066)
proteins	5	1894(0.05716245569909683)	1516 (0.069)	1668 (0.066)
protocols	5	1894(0.05716245569909683)	1516 (0.069)	2548 (0.033)
psychological	5	500(0.2743797873556648)	717 (0.17)	422 (0.33)
psychosocial	5	1894(0.05716245569909683)	1516 (0.069)	1668 (0.066)
public	5	500(0.2743797873556648)	717 (0.17)	422 (0.33)
pureed	5	1894(0.05716245569909683)	#SAKNAS!	1668 (0.066)
qualitative	5	362(0.37727220761403907)	717 (0.17)	361 (0.4)
quantitative	5	836(0.16005487595747114)	717 (0.17)	1233 (0.1)
radiographic	5	1894(0.05716245569909683)	1516 (0.069)	1668 (0.066)
range	5	1894(0.05716245569909683)	1516 (0.069)	1668 (0.066)
rationale	5	1894(0.05716245569909683)	2359 (0.034)	999 (0.13)
real-world	5	1894(0.05716245569909683)	#SAKNAS!	2548 (0.033)
recent	5	1027(0.12575740253801304)	717 (0.17)	1233 (0.1)
refeeding	5	1485(0.08002743797873557)	717 (0.17)	2548 (0.033)
reflect	5	1894(0.05716245569909683)	1516 (0.069)	2548 (0.033)
refractory	5	1894(0.05716245569909683)	2359 (0.034)	1668 (0.066)
regain	5	1894(0.05716245569909683)	2359 (0.034)	1233 (0.1)
relates	5	1894(0.05716245569909683)	1121 (0.1)	#SAKNAS!
relations	5	1894(0.05716245569909683)	1516 (0.069)	1233 (0.1)

relevant	5	1894(0.0571624556990968З)	2359 (0.034)	2548 (0.033)
removable	5	1894(0.05716245569909683)	1516 (0.069)	2548 (0.033)
removal	5	1219(0.1028924202583743)	717 (0.17)	2548 (0.033)
repetitive	5	1894(0.05716245569909683)	#SAKNAS!	#SAKNAS!
replacement	5	896(0.14862238481765175)	717 (0.17)	694 (0.2)
replacer	5	1894(0.05716245569909683)	#SAKNAS!	2548 (0.033)
reports	5	1894(0.05716245569909683)	1516 (0.069)	2548 (0.033)
representative	5	462(0.2972447696353035)	717 (0.17)	388 (0.36)
residual	5	1894(0.05716245569909683)	1516 (0.069)	2548 (0.033)
resistance	5	439(0.30867726077512286)	717 (0.17)	251 (0.56)
resources	5	1027(0.12575740253801304)	717 (0.17)	999 (0.13)
responsiveness	5	1894(0.05716245569909683)	2359 (0.034)	1668 (0.066)
restoration	5	1678(0.0685949468389162)	717 (0.17)	#SAKNAS!
revisiting	5	1894(0.05716245569909683)	1121 (0.1)	1668 (0.066)
risk-factors	5	1894(0.05716245569909683)	717 (0.17)	#SAKNAS!
risk-factors	5	1894(0.05716245569909683)	717 (0.17)	#SAKNAS!
risks	5	896(0.14862238481765175)	717 (0.17)	816 (0.16)
ruminants	5	1678(0.0685949468389162)	717 (0.17)	2548 (0.033)
salt	5	1894(0.05716245569909683)	1516 (0.069)	1668 (0.066)
san	5	1894(0.05716245569909683)	1516 (0.069)	1233 (0.1)
sao	5	1678(0.0685949468389162)	717 (0.17)	2548 (0.033)
sars-cov-2	5	1894(0.05716245569909683)	#SAKNAS!	2548 (0.033)
science	5	1894(0.05716245569909683)	2359 (0.034)	1668 (0.066)
scores	5	1894(0.05716245569909683)	2359 (0.034)	1668 (0.066)
secondary	5	535(0.2515148050760261)	717 (0.17)	694 (0.2)
security	5	1894(0.05716245569909683)	#SAKNAS!	1668 (0.066)
self-expandable	5	1894(0.05716245569909683)	1516 (0.069)	1668 (0.066)
sensing	5	1894(0.05716245569909683)	#SAKNAS!	1233 (0.1)
sensory	5	427(0.3201097519149423)	717 (0.17)	333 (0.43)
sexta	5	1678(0.0685949468389162)	717 (0.17)	2548 (0.033)
sexually	5	1894(0.05716245569909683)	2359 (0.034)	1233 (0.1)
shift	5	1894(0.05716245569909683)	2359 (0.034)	1668 (0.066)
shrimp	5	1219(0.1028924202583743)	717 (0.17)	1233 (0.1)
signal	5	1894(0.05716245569909683)	1516 (0.069)	1233 (0.1)
significant	5	1111(0.11432491139819366)	717 (0.17)	999 (0.13)
significantly	5	1894(0.05716245569909683)	2359 (0.034)	2548 (0.033)
simple	5	896(0.14862238481765175)	717 (0.17)	816 (0.16)
simultaneous	5	1485(0.08002743797873557)	717 (0.17)	2548 (0.033)
sites	5	1894(0.05716245569909683)	1516 (0.069)	2548 (0.033)
skin	5	1894(0.05716245569909683)	2359 (0.034)	1668 (0.066)
snacks	5	1894(0.05716245569909683)	897 (0.14)	2548 (0.033)
sociodemographic	5	559(0.2400823139362067)	717 (0.17)	526 (0.26)
solutions	5	1894(0.05716245569909683)	#SAKNAS!	2548 (0.033)

soybean	5	1894(0.05716245569909683)	2359 (0.034)	#SAKNAS!
spanish	5	410(0.33154224305476165)	717 (0.17)	388 (0.36)
spatial	5	1027(0.12575740253801304)	717 (0.17)	1668 (0.066)
special	5	743(0.18291985823710985)	717 (0.17)	1668 (0.066)
spinal	5	671(0.2057848405167486)	717 (0.17)	1233 (0.1)
spiny	5	1894(0.05716245569909683)	1516 (0.069)	1668 (0.066)
spray	5	1894(0.05716245569909683)	#SAKNAS!	1233 (0.1)
stability	5	1894(0.05716245569909683)	#SAKNAS!	999 (0.13)
stable	5	966(0.1371898936778324)	717 (0.17)	694 (0.2)
steers	5	1894(0.05716245569909683)	#SAKNAS!	2548 (0.033)
stenosis	5	710(0.19435234937692925)	717 (0.17)	584 (0.23)
stenting	5	1894(0.05716245569909683)	1516 (0.069)	1668 (0.066)
stomatitis	5	1894(0.05716245569909683)	1516 (0.069)	2548 (0.033)
stress	5	281(0.468732136732594)	717 (0.17)	174 (0.76)
stroke-associated	5	1894(0.05716245569909683)	2359 (0.034)	#SAKNAS!
structures	5	1894(0.05716245569909683)	2359 (0.034)	1233 (0.1)
studying	5	1894(0.05716245569909683)	1121 (0.1)	#SAKNAS!
subjective	5	516(0.26294729621584545)	717 (0.17)	526 (0.26)
substitute	5	1894(0.05716245569909683)	1121 (0.1)	#SAKNAS!
success	5	1111(0.11432491139819366)	717 (0.17)	1233 (0.1)
supply	5	1335(0.09145992911855493)	717 (0.17)	1233 (0.1)
sustainable	5	1894(0.05716245569909683)	#SAKNAS!	2548 (0.033)
sweeteners	5	1894(0.05716245569909683)	1121 (0.1)	2548 (0.033)
targeting	5	1894(0.05716245569909683)	2359 (0.034)	1668 (0.066)
television	5	1894(0.05716245569909683)	1516 (0.069)	1233 (0.1)
thai	5	743(0.18291985823710985)	717 (0.17)	526 (0.26)
theory	5	1894(0.05716245569909683)	1121 (0.1)	2548 (0.033)
therapists	5	1894(0.05716245569909683)	#SAKNAS!	1668 (0.066)
thermal	5	1335(0.09145992911855493)	717 (0.17)	2548 (0.033)
thickener	5	1894(0.05716245569909683)	#SAKNAS!	1233 (0.1)
thickness	5	628(0.21721733165656798)	717 (0.17)	584 (0.23)
tilapia	5	966(0.1371898936778324)	717 (0.17)	1233 (0.1)
toddler	5	1894(0.05716245569909683)	1121 (0.1)	#SAKNAS!
toxicities	5	1894(0.05716245569909683)	#SAKNAS!	2548 (0.033)
trace	5	1894(0.05716245569909683)	1516 (0.069)	2548 (0.033)
tracheostomy	5	1894(0.05716245569909683)	#SAKNAS!	2548 (0.033)
tract	5	671(0.2057848405167486)	717 (0.17)	461 (0.3)
traits	5	377(0.3658397164742197)	717 (0.17)	526 (0.26)
transition	5	896(0.14862238481765175)	717 (0.17)	816 (0.16)
translation	5	1894(0.05716245569909683)	1516 (0.069)	2548 (0.033)
treating	5	1027(0.12575740253801304)	717 (0.17)	1233 (0.1)
tumors	5	1894(0.05716245569909683)	1121 (0.1)	#SAKNAS!
turkish	5	1111(0.11432491139819366)	717 (0.17)	1233 (0.1)

two-year	5	1894(0.05716245569909683)	#SAKNAS!	1668 (0.066)
ulcerative	5	1894(0.05716245569909683)	1121 (0.1)	2548 (0.033)
university	5	388(0.3544072253344004)	717 (0.17)	461 (0.3)
useful	5	1894(0.05716245569909683)	1121 (0.1)	1668 (0.066)
users	5	836(0.16005487595747114)	717 (0.17)	584 (0.23)
valid	5	1894(0.05716245569909683)	1516 (0.069)	1668 (0.066)
variability	5	836(0.16005487595747114)	717 (0.17)	694 (0.2)
variations	5	1111(0.11432491139819366)	717 (0.17)	1668 (0.066)
various	5	966(0.1371898936778324)	717 (0.17)	1668 (0.066)
view	5	1894(0.05716245569909683)	2359 (0.034)	999 (0.13)
visceral	5	1894(0.05716245569909683)	2359 (0.034)	2548 (0.033)
viscosity	5	1894(0.05716245569909683)	1516 (0.069)	1668 (0.066)
volatile	5	1894(0.05716245569909683)	2359 (0.034)	2548 (0.033)
weeks	5	1894(0.05716245569909683)	1516 (0.069)	2548 (0.033)
west	5	1894(0.05716245569909683)	1516 (0.069)	1668 (0.066)
western	5	628(0.21721733165656798)	717 (0.17)	584 (0.23)
whey	5	1894(0.05716245569909683)	#SAKNAS!	999 (0.13)
xxi	5	1894(0.05716245569909683)	#SAKNAS!	#SAKNAS!
you	5	1894(0.05716245569909683)	#SAKNAS!	1233 (0.1)
1999	4	2214(0.04572996455927746)	#SAKNAS!	1233 (0.1)
1999-2004	4	1111(0.11432491139819366)	897 (0.14)	816 (0.16)
2017-2020	4	2214(0.04572996455927746)	#SAKNAS!	#SAKNAS!
4-year	4	2214(0.04572996455927746)	#SAKNAS!	1233 (0.1)
abnormal	4	1111(0.11432491139819366)	897 (0.14)	1668 (0.066)
academy	4	1678(0.0685949468389162)	897 (0.14)	2548 (0.033)
act	4	2214(0.04572996455927746)	#SAKNAS!	1668 (0.066)
actinomycetemcomitans	4	1335(0.09145992911855493)	897 (0.14)	1233 (0.1)
actions	4	2214(0.04572996455927746)	2359 (0.034)	1233 (0.1)
acutely	4	2214(0.04572996455927746)	2359 (0.034)	2548 (0.033)
adaptations	4	2214(0.04572996455927746)	1516 (0.069)	1668 (0.066)
adequacy	4	1219(0.1028924202583743)	897 (0.14)	1233 (0.1)
adipokines	4	2214(0.04572996455927746)	1516 (0.069)	2548 (0.033)
advertisements	4	2214(0.04572996455927746)	2359 (0.034)	1233 (0.1)
agenda	4	2214(0.04572996455927746)	1516 (0.069)	1668 (0.066)
aggressive	4	1219(0.1028924202583743)	897 (0.14)	816 (0.16)
aid	4	2214(0.04572996455927746)	2359 (0.034)	2548 (0.033)
algae	4	2214(0.04572996455927746)	2359 (0.034)	2548 (0.033)
alteration	4	2214(0.04572996455927746)	1121 (0.1)	2548 (0.033)
altering	4	2214(0.04572996455927746)	2359 (0.034)	2548 (0.033)
alternative	4	966(0.1371898936778324)	897 (0.14)	1233 (0.1)
alveolar	4	966(0.1371898936778324)	897 (0.14)	999 (0.13)
anastomotic	4	2214(0.04572996455927746)	1516 (0.069)	1668 (0.066)
anatomy	4	1335(0.09145992911855493)	897 (0.14)	1233 (0.1)

anemia	4	1335(0.09145992911855493)	897 (0.14)	999 (0.13)
animal	4	896(0.14862238481765175)	897 (0.14)	1668 (0.066)
antarctic	4	2214(0.04572996455927746)	1516 (0.069)	2548 (0.033)
antioxidants	4	2214(0.04572996455927746)	1516 (0.069)	1668 (0.066)
apidae	4	2214(0.04572996455927746)	1516 (0.069)	2548 (0.033)
apis	4	2214(0.04572996455927746)	#SAKNAS!	1233 (0.1)
applicability	4	2214(0.04572996455927746)	#SAKNAS!	2548 (0.033)
arab	4	2214(0.04572996455927746)	1121 (0.1)	2548 (0.033)
arabian	4	2214(0.04572996455927746)	#SAKNAS!	2548 (0.033)
argentina	4	2214(0.04572996455927746)	2359 (0.034)	1233 (0.1)
assamese	4	2214(0.04572996455927746)	2359 (0.034)	1668 (0.066)
assess	4	787(0.1714873670972905)	897 (0.14)	584 (0.23)
asymptomatic	4	2214(0.04572996455927746)	2359 (0.034)	2548 (0.033)
ataxia	4	2214(0.04572996455927746)	1516 (0.069)	2548 (0.033)
attention	4	2214(0.04572996455927746)	1516 (0.069)	1668 (0.066)
attitude	4	2214(0.04572996455927746)	#SAKNAS!	2548 (0.033)
autistic	4	2214(0.04572996455927746)	2359 (0.034)	2548 (0.033)
avian	4	2214(0.04572996455927746)	1121 (0.1)	2548 (0.033)
avoidance	4	2214(0.04572996455927746)	#SAKNAS!	1233 (0.1)
back	4	2214(0.04572996455927746)	1121 (0.1)	2548 (0.033)
banded	4	966(0.1371898936778324)	897 (0.14)	999 (0.13)
baseline	4	1027(0.12575740253801304)	897 (0.14)	816 (0.16)
bat	4	2214(0.04572996455927746)	2359 (0.034)	1233 (0.1)
bee	4	1335(0.09145992911855493)	897 (0.14)	1233 (0.1)
beneficial	4	1894(0.05716245569909683)	897 (0.14)	#SAKNAS!
benefits	4	743(0.18291985823710985)	897 (0.14)	526 (0.26)
benign	4	1111(0.11432491139819366)	897 (0.14)	1233 (0.1)
bias	4	2214(0.04572996455927746)	#SAKNAS!	1233 (0.1)
bimaculatus	4	2214(0.04572996455927746)	#SAKNAS!	999 (0.13)
binge	4	2214(0.04572996455927746)	1516 (0.069)	1668 (0.066)
biobank	4	2214(0.04572996455927746)	#SAKNAS!	1668 (0.066)
biofilms	4	2214(0.04572996455927746)	2359 (0.034)	1233 (0.1)
bitter	4	2214(0.04572996455927746)	#SAKNAS!	1668 (0.066)
blenderized	4	2214(0.04572996455927746)	#SAKNAS!	1668 (0.066)
bombus	4	2214(0.04572996455927746)	2359 (0.034)	#SAKNAS!
border	4	2214(0.04572996455927746)	#SAKNAS!	1233 (0.1)
borer	4	2214(0.04572996455927746)	1516 (0.069)	1668 (0.066)
bottle	4	1335(0.09145992911855493)	897 (0.14)	2548 (0.033)
branched-chain	4	2214(0.04572996455927746)	#SAKNAS!	2548 (0.033)
british	4	2214(0.04572996455927746)	1121 (0.1)	#SAKNAS!
bronze	4	2214(0.04572996455927746)	#SAKNAS!	999 (0.13)
brown	4	1027(0.12575740253801304)	897 (0.14)	999 (0.13)
buffalo	4	2214(0.04572996455927746)	1516 (0.069)	2548 (0.033)

building	4	2214(0.04572996455927746)	2359 (0.034)	1233 (0.1)
bulimia	4	1485(0.08002743797873557)	897 (0.14)	1668 (0.066)
bulls	4	2214(0.04572996455927746)	1516 (0.069)	#SAKNAS!
burning	4	2214(0.04572996455927746)	2359 (0.034)	#SAKNAS!
cake	4	2214(0.04572996455927746)	#SAKNAS!	#SAKNAS!
calf	4	2214(0.04572996455927746)	#SAKNAS!	2548 (0.033)
calves	4	628(0.21721733165656798)	897 (0.14)	694 (0.2)
canada	4	2214(0.04572996455927746)	1516 (0.069)	2548 (0.033)
cancers	4	743(0.18291985823710985)	897 (0.14)	461 (0.3)
candidiasis	4	2214(0.04572996455927746)	1516 (0.069)	1668 (0.066)
canine	4	2214(0.04572996455927746)	2359 (0.034)	1668 (0.066)
carbohydrates	4	1335(0.09145992911855493)	897 (0.14)	2548 (0.033)
carcinomas	4	2214(0.04572996455927746)	1121 (0.1)	#SAKNAS!
cardiac	4	896(0.14862238481765175)	897 (0.14)	694 (0.2)
caregiver	4	1219(0.1028924202583743)	897 (0.14)	#SAKNAS!
cariogenicity	4	2214(0.04572996455927746)	2359 (0.034)	2548 (0.033)
caterpillar	4	2214(0.04572996455927746)	1121 (0.1)	2548 (0.033)
caterpillars	4	1678(0.0685949468389162)	897 (0.14)	2548 (0.033)
cause-specific	4	2214(0.04572996455927746)	#SAKNAS!	2548 (0.033)
causing	4	2214(0.04572996455927746)	#SAKNAS!	2548 (0.033)
caustic	4	2214(0.04572996455927746)	1516 (0.069)	1668 (0.066)
celiac	4	2214(0.04572996455927746)	2359 (0.034)	#SAKNAS!
center	4	628(0.21721733165656798)	897 (0.14)	584 (0.23)
centers	4	1335(0.09145992911855493)	897 (0.14)	#SAKNAS!
century	4	2214(0.04572996455927746)	1121 (0.1)	2548 (0.033)
cereal	4	2214(0.04572996455927746)	1516 (0.069)	1668 (0.066)
cerebrovascular	4	2214(0.04572996455927746)	897 (0.14)	#SAKNAS!
cerebrovascular	4	2214(0.04572996455927746)	897 (0.14)	#SAKNAS!
cervus	4	2214(0.04572996455927746)	897 (0.14)	#SAKNAS!
cervus	4	2214(0.04572996455927746)	897 (0.14)	#SAKNAS!
changing	4	2214(0.04572996455927746)	1516 (0.069)	#SAKNAS!
characterizing	4	2214(0.04572996455927746)	2359 (0.034)	1668 (0.066)
chemo	4	2214(0.04572996455927746)	2359 (0.034)	1233 (0.1)
chile	4	1678(0.0685949468389162)	897 (0.14)	#SAKNAS!
circuit	4	2214(0.04572996455927746)	#SAKNAS!	1668 (0.066)
circumference	4	966(0.1371898936778324)	897 (0.14)	999 (0.13)
cirrhosis	4	2214(0.04572996455927746)	#SAKNAS!	999 (0.13)
clinic	4	896(0.14862238481765175)	897 (0.14)	1233 (0.1)
clinics	4	2214(0.04572996455927746)	1121 (0.1)	#SAKNAS!
closely	4	2214(0.04572996455927746)	#SAKNAS!	2548 (0.033)
closure	4	2214(0.04572996455927746)	1121 (0.1)	#SAKNAS!
clustering	4	1219(0.1028924202583743)	897 (0.14)	#SAKNAS!
co2	4	2214(0.04572996455927746)	2359 (0.034)	1233 (0.1)

cocaine	4	2214(0.04572996455927746)	1516 (0.069)	2548 (0.033)
cohorts	4	2214(0.04572996455927746)	#SAKNAS!	1233 (0.1)
coleoptera	4	1335(0.09145992911855493)	897 (0.14)	1233 (0.1)
collective	4	2214(0.04572996455927746)	1516 (0.069)	2548 (0.033)
colonization	4	1111(0.11432491139819366)	897 (0.14)	816 (0.16)
committee	4	2214(0.04572996455927746)	1121 (0.1)	2548 (0.033)
communication	4	1335(0.09145992911855493)	897 (0.14)	1233 (0.1)
compensate	4	2214(0.04572996455927746)	#SAKNAS!	1233 (0.1)
competitive	4	2214(0.04572996455927746)	2359 (0.034)	2548 (0.033)
complaints	4	2214(0.04572996455927746)	2359 (0.034)	2548 (0.033)
complexity	4	2214(0.04572996455927746)	2359 (0.034)	2548 (0.033)
component	4	1111(0.11432491139819366)	897 (0.14)	816 (0.16)
components	4	966(0.1371898936778324)	897 (0.14)	999 (0.13)
concern	4	2214(0.04572996455927746)	#SAKNAS!	2548 (0.033)
conduit	4	2214(0.04572996455927746)	2359 (0.034)	1668 (0.066)
conservation	4	2214(0.04572996455927746)	2359 (0.034)	1668 (0.066)
constraints	4	2214(0.04572996455927746)	897 (0.14)	#SAKNAS!
constraints	4	2214(0.04572996455927746)	897 (0.14)	#SAKNAS!
consumer	4	2214(0.04572996455927746)	#SAKNAS!	1233 (0.1)
contents	4	2214(0.04572996455927746)	#SAKNAS!	1233 (0.1)
conventional	4	836(0.16005487595747114)	897 (0.14)	1668 (0.066)
copd	4	2214(0.04572996455927746)	2359 (0.034)	#SAKNAS!
copepods	4	2214(0.04572996455927746)	897 (0.14)	#SAKNAS!
copepods	4	2214(0.04572996455927746)	897 (0.14)	#SAKNAS!
coping	4	2214(0.04572996455927746)	2359 (0.034)	1668 (0.066)
cortical	4	2214(0.04572996455927746)	2359 (0.034)	1668 (0.066)
cortisol	4	2214(0.04572996455927746)	897 (0.14)	#SAKNAS!
cortisol	4	2214(0.04572996455927746)	897 (0.14)	#SAKNAS!
cotton	4	2214(0.04572996455927746)	#SAKNAS!	1668 (0.066)
cough	4	2214(0.04572996455927746)	#SAKNAS!	1233 (0.1)
counseling	4	1219(0.1028924202583743)	897 (0.14)	816 (0.16)
county	4	2214(0.04572996455927746)	2359 (0.034)	1233 (0.1)
cricket	4	2214(0.04572996455927746)	#SAKNAS!	999 (0.13)
critically	4	2214(0.04572996455927746)	#SAKNAS!	#SAKNAS!
crossover	4	2214(0.04572996455927746)	2359 (0.034)	2548 (0.033)
curative	4	1678(0.0685949468389162)	897 (0.14)	1668 (0.066)
curve	4	2214(0.04572996455927746)	1516 (0.069)	2548 (0.033)
cycle	4	1335(0.09145992911855493)	897 (0.14)	999 (0.13)
cyclic	4	2214(0.04572996455927746)	1516 (0.069)	2548 (0.033)
damage	4	1678(0.0685949468389162)	897 (0.14)	2548 (0.033)
danish	4	2214(0.04572996455927746)	2359 (0.034)	1233 (0.1)
daphnia	4	2214(0.04572996455927746)	1516 (0.069)	2548 (0.033)
day	4	1485(0.08002743797873557)	897 (0.14)	1668 (0.066)

decay	4	966(0.1371898936778324)	897 (0.14)	2548 (0.033)
deciduous	4	1219(0.1028924202583743)	897 (0.14)	1233 (0.1)
decompression	4	2214(0.04572996455927746)	#SAKNAS!	1668 (0.066)
defining	4	2214(0.04572996455927746)	1516 (0.069)	2548 (0.033)
degeneration	4	2214(0.04572996455927746)	2359 (0.034)	1668 (0.066)
degree	4	2214(0.04572996455927746)	2359 (0.034)	2548 (0.033)
delay	4	2214(0.04572996455927746)	1516 (0.069)	2548 (0.033)
delivery	4	1027(0.12575740253801304)	897 (0.14)	2548 (0.033)
dependence	4	1335(0.09145992911855493)	897 (0.14)	1233 (0.1)
derivation	4	1894(0.05716245569909683)	897 (0.14)	#SAKNAS!
dessert	4	2214(0.04572996455927746)	#SAKNAS!	2548 (0.033)
detect	4	2214(0.04572996455927746)	#SAKNAS!	2548 (0.033)
detecting	4	2214(0.04572996455927746)	#SAKNAS!	1233 (0.1)
determination	4	2214(0.04572996455927746)	1121 (0.1)	2548 (0.033)
diagnosing	4	2214(0.04572996455927746)	1516 (0.069)	2548 (0.033)
dialysis	4	1219(0.1028924202583743)	897 (0.14)	1668 (0.066)
dietetics	4	2214(0.04572996455927746)	1121 (0.1)	2548 (0.033)
diet-related	4	2214(0.04572996455927746)	#SAKNAS!	1668 (0.066)
differing	4	2214(0.04572996455927746)	1121 (0.1)	2548 (0.033)
dilation	4	966(0.1371898936778324)	897 (0.14)	1233 (0.1)
direct	4	1219(0.1028924202583743)	897 (0.14)	1668 (0.066)
disabilities	4	836(0.16005487595747114)	897 (0.14)	816 (0.16)
disaster	4	2214(0.04572996455927746)	#SAKNAS!	1233 (0.1)
discrimination	4	2214(0.04572996455927746)	1121 (0.1)	2548 (0.033)
disease-specific	4	2214(0.04572996455927746)	1516 (0.069)	#SAKNAS!
disparity	4	2214(0.04572996455927746)	2359 (0.034)	1668 (0.066)
distal	4	2214(0.04572996455927746)	2359 (0.034)	#SAKNAS!
disturbances	4	2214(0.04572996455927746)	1516 (0.069)	1668 (0.066)
diurnal	4	1678(0.0685949468389162)	897 (0.14)	#SAKNAS!
diverticulectomy	4	2214(0.04572996455927746)	1121 (0.1)	2548 (0.033)
diving	4	2214(0.04572996455927746)	1121 (0.1)	2548 (0.033)
doing	4	2214(0.04572996455927746)	2359 (0.034)	1668 (0.066)
domestic	4	1678(0.0685949468389162)	897 (0.14)	#SAKNAS!
dyslipidemia	4	1219(0.1028924202583743)	897 (0.14)	1668 (0.066)
dystrophic	4	1894(0.05716245569909683)	897 (0.14)	2548 (0.033)
dystrophy	4	743(0.18291985823710985)	897 (0.14)	999 (0.13)
ecological	4	966(0.1371898936778324)	897 (0.14)	1233 (0.1)
effective	4	836(0.16005487595747114)	897 (0.14)	694 (0.2)
efp	4	2214(0.04572996455927746)	#SAKNAS!	1668 (0.066)
egg	4	1219(0.1028924202583743)	897 (0.14)	1233 (0.1)
elaphus	4	2214(0.04572996455927746)	897 (0.14)	#SAKNAS!
elaphus	4	2214(0.04572996455927746)	897 (0.14)	#SAKNAS!
electrical	4	743(0.18291985823710985)	897 (0.14)	461 (0.3)

electromyographic	4	2214(0.04572996455927746)	#SAKNAS!	2548 (0.033)
electromyography	4	2214(0.04572996455927746)	1516 (0.069)	2548 (0.033)
element	4	2214(0.04572996455927746)	1121 (0.1)	#SAKNAS!
elephant	4	2214(0.04572996455927746)	1516 (0.069)	1668 (0.066)
emirates	4	2214(0.04572996455927746)	1121 (0.1)	2548 (0.033)
emission	4	2214(0.04572996455927746)	1516 (0.069)	#SAKNAS!
emptying	4	1111(0.11432491139819366)	897 (0.14)	1233 (0.1)
endocrine	4	2214(0.04572996455927746)	2359 (0.034)	1668 (0.066)
endodontic	4	2214(0.04572996455927746)	2359 (0.034)	1668 (0.066)
english	4	2214(0.04572996455927746)	1516 (0.069)	1668 (0.066)
ent	4	2214(0.04572996455927746)	1121 (0.1)	#SAKNAS!
eosinophilic	4	595(0.22864982279638732)	897 (0.14)	461 (0.3)
epidemiologic	4	2214(0.04572996455927746)	1516 (0.069)	1668 (0.066)
epigenetic	4	2214(0.04572996455927746)	#SAKNAS!	1233 (0.1)
eruption	4	1894(0.05716245569909683)	897 (0.14)	2548 (0.033)
esnupi	4	2214(0.04572996455927746)	#SAKNAS!	1233 (0.1)
espen	4	2214(0.04572996455927746)	#SAKNAS!	1668 (0.066)
etiology	4	2214(0.04572996455927746)	2359 (0.034)	1233 (0.1)
evolutionary	4	1485(0.08002743797873557)	897 (0.14)	1668 (0.066)
exacerbation	4	2214(0.04572996455927746)	2359 (0.034)	1668 (0.066)
existing	4	2214(0.04572996455927746)	#SAKNAS!	1668 (0.066)
expectations	4	2214(0.04572996455927746)	1516 (0.069)	1668 (0.066)
experiencing	4	2214(0.04572996455927746)	1121 (0.1)	#SAKNAS!
experiments	4	1894(0.05716245569909683)	897 (0.14)	2548 (0.033)
explain	4	2214(0.04572996455927746)	2359 (0.034)	2548 (0.033)
exposures	4	2214(0.04572996455927746)	#SAKNAS!	#SAKNAS!
extent	4	1678(0.0685949468389162)	897 (0.14)	1668 (0.066)
facilitators	4	2214(0.04572996455927746)	2359 (0.034)	#SAKNAS!
falls	4	2214(0.04572996455927746)	#SAKNAS!	2548 (0.033)
feeds	4	2214(0.04572996455927746)	1121 (0.1)	2548 (0.033)
fees	4	2214(0.04572996455927746)	2359 (0.034)	2548 (0.033)
fertilization	4	2214(0.04572996455927746)	2359 (0.034)	1668 (0.066)
fiber	4	743(0.18291985823710985)	897 (0.14)	816 (0.16)
fifth	4	2214(0.04572996455927746)	#SAKNAS!	1233 (0.1)
fitness	4	787(0.1714873670972905)	897 (0.14)	694 (0.2)
flavor	4	2214(0.04572996455927746)	1121 (0.1)	#SAKNAS!
flemish	4	2214(0.04572996455927746)	897 (0.14)	#SAKNAS!
flemish	4	2214(0.04572996455927746)	897 (0.14)	#SAKNAS!
flexible	4	1485(0.08002743797873557)	897 (0.14)	#SAKNAS!
flossing	4	2214(0.04572996455927746)	2359 (0.034)	1668 (0.066)
folate	4	2214(0.04572996455927746)	1516 (0.069)	#SAKNAS!
forest	4	516(0.26294729621584545)	897 (0.14)	333 (0.43)
formative	4	2214(0.04572996455927746)	#SAKNAS!	#SAKNAS!

forms	4	2214(0.04572996455927746)	1516 (0.069)	1668 (0.066)
formulas	4	2214(0.04572996455927746)	2359 (0.034)	2548 (0.033)
fourth	4	1485(0.08002743797873557)	897 (0.14)	1233 (0.1)
framework	4	1219(0.1028924202583743)	897 (0.14)	2548 (0.033)
free-ranging	4	1335(0.09145992911855493)	897 (0.14)	1233 (0.1)
freshwater	4	2214(0.04572996455927746)	2359 (0.034)	1233 (0.1)
fructose	4	2214(0.04572996455927746)	2359 (0.034)	1233 (0.1)
fully	4	2214(0.04572996455927746)	2359 (0.034)	1668 (0.066)
functioning	4	2214(0.04572996455927746)	#SAKNAS!	1233 (0.1)
functions	4	896(0.14862238481765175)	897 (0.14)	1233 (0.1)
gait	4	2214(0.04572996455927746)	#SAKNAS!	1668 (0.066)
galanin	4	2214(0.04572996455927746)	1121 (0.1)	2548 (0.033)
gastrectomy	4	319(0.4230021721733166)	897 (0.14)	228 (0.59)
gastroenterology	4	2214(0.04572996455927746)	2359 (0.034)	2548 (0.033)
gastro-intestinal	4	2214(0.04572996455927746)	1516 (0.069)	2548 (0.033)
gastroplasty	4	1111(0.11432491139819366)	897 (0.14)	1668 (0.066)
gastrostomies	4	2214(0.04572996455927746)	1121 (0.1)	2548 (0.033)
geese	4	2214(0.04572996455927746)	1121 (0.1)	#SAKNAS!
gel	4	2214(0.04572996455927746)	#SAKNAS!	1233 (0.1)
gels	4	2214(0.04572996455927746)	#SAKNAS!	2548 (0.033)
gender	4	710(0.19435234937692925)	897 (0.14)	816 (0.16)
genes	4	896(0.14862238481765175)	897 (0.14)	999 (0.13)
gerontological	4	2214(0.04572996455927746)	1121 (0.1)	#SAKNAS!
gestation	4	1335(0.09145992911855493)	897 (0.14)	1233 (0.1)
given	4	1894(0.05716245569909683)	897 (0.14)	2548 (0.033)
global	4	628(0.21721733165656798)	897 (0.14)	526 (0.26)
glossectomy	4	2214(0.04572996455927746)	#SAKNAS!	1233 (0.1)
glutamate	4	2214(0.04572996455927746)	2359 (0.034)	2548 (0.033)
glycation	4	2214(0.04572996455927746)	2359 (0.034)	1668 (0.066)
gorillas	4	2214(0.04572996455927746)	1516 (0.069)	2548 (0.033)
gradient	4	2214(0.04572996455927746)	897 (0.14)	#SAKNAS!
gradient	4	2214(0.04572996455927746)	897 (0.14)	#SAKNAS!
grasshopper	4	1894(0.05716245569909683)	897 (0.14)	2548 (0.033)
grasshoppers	4	2214(0.04572996455927746)	1516 (0.069)	#SAKNAS!
grip	4	2214(0.04572996455927746)	#SAKNAS!	1668 (0.066)
gryllus	4	2214(0.04572996455927746)	#SAKNAS!	999 (0.13)
guangxi	4	2214(0.04572996455927746)	#SAKNAS!	1233 (0.1)
guideline	4	966(0.1371898936778324)	897 (0.14)	1233 (0.1)
gum-based	4	2214(0.04572996455927746)	#SAKNAS!	1233 (0.1)
habitat	4	1335(0.09145992911855493)	897 (0.14)	999 (0.13)
hay	4	1027(0.12575740253801304)	897 (0.14)	999 (0.13)
head-and-neck	4	1219(0.1028924202583743)	897 (0.14)	1668 (0.066)
healing	4	2214(0.04572996455927746)	2359 (0.034)	1668 (0.066)

healthier	4	2214(0.04572996455927746)	2359 (0.034)	#SAKNAS!
height	4	1027(0.12575740253801304)	897 (0.14)	816 (0.16)
hepatic	4	2214(0.04572996455927746)	1516 (0.069)	#SAKNAS!
hepatology	4	2214(0.04572996455927746)	2359 (0.034)	2548 (0.033)
hereditary	4	2214(0.04572996455927746)	2359 (0.034)	2548 (0.033)
heterogeneity	4	2214(0.04572996455927746)	897 (0.14)	#SAKNAS!
heterogeneity	4	2214(0.04572996455927746)	897 (0.14)	#SAKNAS!
histological	4	2214(0.04572996455927746)	#SAKNAS!	1668 (0.066)
honeybee	4	2214(0.04572996455927746)	2359 (0.034)	1233 (0.1)
hormonal	4	2214(0.04572996455927746)	1121 (0.1)	#SAKNAS!
hosts	4	1678(0.0685949468389162)	897 (0.14)	#SAKNAS!
households	4	2214(0.04572996455927746)	#SAKNAS!	1668 (0.066)
housing	4	1894(0.05716245569909683)	897 (0.14)	2548 (0.033)
hpv	4	2214(0.04572996455927746)	2359 (0.034)	1233 (0.1)
hydrocarbons	4	2214(0.04572996455927746)	#SAKNAS!	2548 (0.033)
hymenoptera	4	1335(0.09145992911855493)	897 (0.14)	1668 (0.066)
hyoid	4	2214(0.04572996455927746)	1516 (0.069)	1668 (0.066)
hypopharyngeal	4	1111(0.11432491139819366)	897 (0.14)	999 (0.13)
hypoplasia	4	2214(0.04572996455927746)	1516 (0.069)	2548 (0.033)
identifying	4	1335(0.09145992911855493)	897 (0.14)	1668 (0.066)
immunological	4	2214(0.04572996455927746)	1516 (0.069)	2548 (0.033)
impedance	4	2214(0.04572996455927746)	#SAKNAS!	1668 (0.066)
improves	4	439(0.30867726077512286)	897 (0.14)	361 (0.4)
incremental	4	2214(0.04572996455927746)	1516 (0.069)	2548 (0.033)
independent	4	595(0.22864982279638732)	897 (0.14)	526 (0.26)
infant-feeding	4	2214(0.04572996455927746)	1516 (0.069)	1668 (0.066)
information	4	1335(0.09145992911855493)	897 (0.14)	999 (0.13)
ingestive	4	966(0.1371898936778324)	897 (0.14)	1668 (0.066)
ingredients	4	2214(0.04572996455927746)	2359 (0.034)	2548 (0.033)
in-hospital	4	2214(0.04572996455927746)	2359 (0.034)	#SAKNAS!
initial	4	2214(0.04572996455927746)	1121 (0.1)	#SAKNAS!
initiation	4	2214(0.04572996455927746)	2359 (0.034)	#SAKNAS!
institute	4	2214(0.04572996455927746)	#SAKNAS!	1668 (0.066)
insufficient	4	2214(0.04572996455927746)	1516 (0.069)	1668 (0.066)
interdisciplinary	4	2214(0.04572996455927746)	2359 (0.034)	1233 (0.1)
international	4	787(0.1714873670972905)	897 (0.14)	1668 (0.066)
interrelationship	4	2214(0.04572996455927746)	1516 (0.069)	2548 (0.033)
interview	4	2214(0.04572996455927746)	2359 (0.034)	#SAKNAS!
intraoral	4	2214(0.04572996455927746)	#SAKNAS!	1668 (0.066)
intubation	4	2214(0.04572996455927746)	2359 (0.034)	1668 (0.066)
invasion	4	2214(0.04572996455927746)	2359 (0.034)	1668 (0.066)
invasive	4	896(0.14862238481765175)	897 (0.14)	584 (0.23)
investigation	4	462(0.2972447696353035)	897 (0.14)	816 (0.16)

involving	4	2214(0.04572996455927746)	1516 (0.069)	1668 (0.066)
island	4	1219(0.1028924202583743)	897 (0.14)	999 (0.13)
isotope	4	2214(0.04572996455927746)	1516 (0.069)	2548 (0.033)
isotopes	4	1894(0.05716245569909683)	897 (0.14)	2548 (0.033)
israel	4	2214(0.04572996455927746)	2359 (0.034)	1668 (0.066)
italy	4	896(0.14862238481765175)	897 (0.14)	694 (0.2)
japonica	4	2214(0.04572996455927746)	1121 (0.1)	#SAKNAS!
jaw	4	2214(0.04572996455927746)	2359 (0.034)	2548 (0.033)
jejunal	4	2214(0.04572996455927746)	2359 (0.034)	1668 (0.066)
juice	4	2214(0.04572996455927746)	2359 (0.034)	1668 (0.066)
juveniles	4	2214(0.04572996455927746)	2359 (0.034)	1233 (0.1)
karachi	4	2214(0.04572996455927746)	#SAKNAS!	2548 (0.033)
kinase	4	2214(0.04572996455927746)	1121 (0.1)	#SAKNAS!
kindergarten	4	2214(0.04572996455927746)	#SAKNAS!	2548 (0.033)
knee	4	2214(0.04572996455927746)	#SAKNAS!	2548 (0.033)
labor	4	2214(0.04572996455927746)	1516 (0.069)	1668 (0.066)
landscape	4	2214(0.04572996455927746)	2359 (0.034)	2548 (0.033)
laryngectomy	4	1219(0.1028924202583743)	897 (0.14)	999 (0.13)
later	4	2214(0.04572996455927746)	2359 (0.034)	1233 (0.1)
latino	4	2214(0.04572996455927746)	2359 (0.034)	1233 (0.1)
leadership	4	2214(0.04572996455927746)	#SAKNAS!	1668 (0.066)
lean	4	2214(0.04572996455927746)	1121 (0.1)	2548 (0.033)
left	4	2214(0.04572996455927746)	1121 (0.1)	#SAKNAS!
lemur	4	1894(0.05716245569909683)	897 (0.14)	2548 (0.033)
lichen	4	2214(0.04572996455927746)	2359 (0.034)	1668 (0.066)
life-style	4	2214(0.04572996455927746)	1121 (0.1)	#SAKNAS!
lifestyles	4	2214(0.04572996455927746)	#SAKNAS!	1668 (0.066)
lifetime	4	2214(0.04572996455927746)	1516 (0.069)	#SAKNAS!
link	4	628(0.21721733165656798)	897 (0.14)	584 (0.23)
linking	4	2214(0.04572996455927746)	2359 (0.034)	2548 (0.033)
lipid	4	535(0.2515148050760261)	897 (0.14)	422 (0.33)
lipids	4	2214(0.04572996455927746)	2359 (0.034)	1233 (0.1)
lipoprotein	4	1219(0.1028924202583743)	897 (0.14)	2548 (0.033)
liquid	4	966(0.1371898936778324)	897 (0.14)	1233 (0.1)
location	4	2214(0.04572996455927746)	#SAKNAS!	2548 (0.033)
locomotor	4	1894(0.05716245569909683)	897 (0.14)	#SAKNAS!
long-evans	4	2214(0.04572996455927746)	897 (0.14)	#SAKNAS!
long-evans	4	2214(0.04572996455927746)	897 (0.14)	#SAKNAS!
macaques	4	896(0.14862238481765175)	897 (0.14)	526 (0.26)
madagascar	4	2214(0.04572996455927746)	1516 (0.069)	2548 (0.033)
magnesium	4	2214(0.04572996455927746)	1516 (0.069)	#SAKNAS!
maintaining	4	2214(0.04572996455927746)	2359 (0.034)	1668 (0.066)
maize	4	2214(0.04572996455927746)	1516 (0.069)	#SAKNAS!

malaysian	4	2214(0.04572996455927746)	2359 (0.034)	1668 (0.066)
managing	4	2214(0.04572996455927746)	1516 (0.069)	1668 (0.066)
mandibular	4	896(0.14862238481765175)	897 (0.14)	1233 (0.1)
manipulation	4	2214(0.04572996455927746)	1121 (0.1)	2548 (0.033)
manometric	4	2214(0.04572996455927746)	2359 (0.034)	2548 (0.033)
masseter	4	2214(0.04572996455927746)	#SAKNAS!	2548 (0.033)
material	4	2214(0.04572996455927746)	2359 (0.034)	1668 (0.066)
matrix	4	1485(0.08002743797873557)	897 (0.14)	1668 (0.066)
mealtimes	4	2214(0.04572996455927746)	#SAKNAS!	999 (0.13)
measure	4	896(0.14862238481765175)	897 (0.14)	694 (0.2)
measurement	4	671(0.2057848405167486)	897 (0.14)	584 (0.23)
medicaid	4	2214(0.04572996455927746)	2359 (0.034)	#SAKNAS!
medications	4	2214(0.04572996455927746)	#SAKNAS!	1668 (0.066)
medieval	4	2214(0.04572996455927746)	#SAKNAS!	1233 (0.1)
medium	4	2214(0.04572996455927746)	1121 (0.1)	#SAKNAS!
melanin-concentrating	4	2214(0.04572996455927746)	1121 (0.1)	2548 (0.033)
melanocortin	4	2214(0.04572996455927746)	1121 (0.1)	#SAKNAS!
mellifera	4	2214(0.04572996455927746)	#SAKNAS!	1233 (0.1)
memory	4	2214(0.04572996455927746)	#SAKNAS!	1233 (0.1)
metabolomic	4	2214(0.04572996455927746)	2359 (0.034)	#SAKNAS!
methane	4	2214(0.04572996455927746)	#SAKNAS!	#SAKNAS!
microbiological	4	1111(0.11432491139819366)	897 (0.14)	1233 (0.1)
micronutrient	4	1219(0.1028924202583743)	897 (0.14)	2548 (0.033)
micronutrients	4	2214(0.04572996455927746)	2359 (0.034)	2548 (0.033)
mining	4	2214(0.04572996455927746)	2359 (0.034)	1233 (0.1)
mixotrophic	4	2214(0.04572996455927746)	1516 (0.069)	2548 (0.033)
modulates	4	1027(0.12575740253801304)	897 (0.14)	999 (0.13)
modulation	4	966(0.1371898936778324)	897 (0.14)	816 (0.16)
moth	4	2214(0.04572996455927746)	897 (0.14)	#SAKNAS!
moth	4	2214(0.04572996455927746)	897 (0.14)	#SAKNAS!
multicenter	4	281(0.468732136732594)	897 (0.14)	280 (0.49)
multidimensional	4	2214(0.04572996455927746)	1121 (0.1)	#SAKNAS!
murine	4	2214(0.04572996455927746)	1516 (0.069)	2548 (0.033)
myositis	4	2214(0.04572996455927746)	#SAKNAS!	1668 (0.066)
mytilus	4	1894(0.05716245569909683)	897 (0.14)	2548 (0.033)
naive	4	2214(0.04572996455927746)	#SAKNAS!	999 (0.13)
nasopharyngeal	4	671(0.2057848405167486)	897 (0.14)	816 (0.16)
nationally	4	743(0.18291985823710985)	897 (0.14)	694 (0.2)
necrotizing	4	1894(0.05716245569909683)	897 (0.14)	#SAKNAS!
nectar	4	2214(0.04572996455927746)	#SAKNAS!	1233 (0.1)
negative	4	2214(0.04572996455927746)	2359 (0.034)	1668 (0.066)
neo-adjuvant	4	2214(0.04572996455927746)	2359 (0.034)	1233 (0.1)
neolithic	4	2214(0.04572996455927746)	2359 (0.034)	2548 (0.033)

neonates	4	2214(0.04572996455927746)	1516 (0.069)	2548 (0.033)
neophobia	4	2214(0.04572996455927746)	#SAKNAS!	1668 (0.066)
nerve	4	2214(0.04572996455927746)	#SAKNAS!	1668 (0.066)
nesfatin-1	4	2214(0.04572996455927746)	2359 (0.034)	1668 (0.066)
network	4	1219(0.1028924202583743)	897 (0.14)	2548 (0.033)
neurodegenerative	4	2214(0.04572996455927746)	2359 (0.034)	2548 (0.033)
neurologic	4	1894(0.05716245569909683)	897 (0.14)	#SAKNAS!
neuromuscular	4	836(0.16005487595747114)	897 (0.14)	461 (0.3)
neuron	4	2214(0.04572996455927746)	1516 (0.069)	1668 (0.066)
neurotrophic	4	2214(0.04572996455927746)	1121 (0.1)	#SAKNAS!
neutrophil	4	2214(0.04572996455927746)	2359 (0.034)	2548 (0.033)
newly	4	896(0.14862238481765175)	897 (0.14)	1233 (0.1)
nile	4	1219(0.1028924202583743)	897 (0.14)	1233 (0.1)
nitrogen	4	595(0.22864982279638732)	897 (0.14)	526 (0.26)
nocturnal	4	1894(0.05716245569909683)	897 (0.14)	#SAKNAS!
non-alcoholic	4	2214(0.04572996455927746)	2359 (0.034)	1668 (0.066)
noncommunicable	4	2214(0.04572996455927746)	2359 (0.034)	1668 (0.066)
non-smoking	4	2214(0.04572996455927746)	1516 (0.069)	1668 (0.066)
northeast	4	2214(0.04572996455927746)	1516 (0.069)	2548 (0.033)
northwest	4	2214(0.04572996455927746)	2359 (0.034)	1668 (0.066)
npy	4	2214(0.04572996455927746)	2359 (0.034)	2548 (0.033)
nuclear	4	2214(0.04572996455927746)	2359 (0.034)	2548 (0.033)
nutritionally	4	1485(0.08002743797873557)	897 (0.14)	1668 (0.066)
nutritionday	4	2214(0.04572996455927746)	2359 (0.034)	1233 (0.1)
nutritive	4	1485(0.08002743797873557)	897 (0.14)	1668 (0.066)
nymphs	4	2214(0.04572996455927746)	2359 (0.034)	1233 (0.1)
observational	4	183(0.6973819595289814)	897 (0.14)	194 (0.69)
occlusion	4	2214(0.04572996455927746)	#SAKNAS!	1668 (0.066)
oesophagus	4	1894(0.05716245569909683)	897 (0.14)	2548 (0.033)
offspring	4	836(0.16005487595747114)	897 (0.14)	694 (0.2)
ohrqol	4	2214(0.04572996455927746)	2359 (0.034)	#SAKNAS!
omega-3	4	2214(0.04572996455927746)	1516 (0.069)	#SAKNAS!
oncologic	4	2214(0.04572996455927746)	#SAKNAS!	1668 (0.066)
opening	4	2214(0.04572996455927746)	1516 (0.069)	2548 (0.033)
operation	4	1485(0.08002743797873557)	897 (0.14)	1668 (0.066)
operative	4	2214(0.04572996455927746)	2359 (0.034)	2548 (0.033)
optimizing	4	2214(0.04572996455927746)	1516 (0.069)	2548 (0.033)
oreochromis	4	2214(0.04572996455927746)	2359 (0.034)	1668 (0.066)
orexin	4	1485(0.08002743797873557)	897 (0.14)	1233 (0.1)
organ	4	2214(0.04572996455927746)	#SAKNAS!	2548 (0.033)
organisms	4	2214(0.04572996455927746)	1516 (0.069)	1668 (0.066)
origin	4	1894(0.05716245569909683)	897 (0.14)	2548 (0.033)
ornatus	4	2214(0.04572996455927746)	#SAKNAS!	1668 (0.066)

oro-pharyngeal	4	2214(0.04572996455927746)	2359 (0.034)	2548 (0.033)
oropharynx	4	2214(0.04572996455927746)	2359 (0.034)	1668 (0.066)
orthognathic	4	2214(0.04572996455927746)	2359 (0.034)	#SAKNAS!
osteoperio	4	2214(0.04572996455927746)	2359 (0.034)	2548 (0.033)
osteoporosis	4	1335(0.09145992911855493)	897 (0.14)	2548 (0.033)
osteoporotic	4	2214(0.04572996455927746)	1516 (0.069)	1668 (0.066)
outpatients	4	1111(0.11432491139819366)	897 (0.14)	1233 (0.1)
oviposition	4	2214(0.04572996455927746)	1121 (0.1)	2548 (0.033)
oxytocin	4	2214(0.04572996455927746)	1516 (0.069)	2548 (0.033)
palatability	4	966(0.1371898936778324)	897 (0.14)	694 (0.2)
palatable	4	2214(0.04572996455927746)	2359 (0.034)	1668 (0.066)
palate	4	1678(0.0685949468389162)	897 (0.14)	#SAKNAS!
pancreatic	4	2214(0.04572996455927746)	1121 (0.1)	2548 (0.033)
panulirus	4	2214(0.04572996455927746)	#SAKNAS!	1668 (0.066)
papillomavirus	4	2214(0.04572996455927746)	#SAKNAS!	999 (0.13)
parasitic	4	2214(0.04572996455927746)	2359 (0.034)	1233 (0.1)
parasitism	4	2214(0.04572996455927746)	1121 (0.1)	#SAKNAS!
parenting	4	628(0.21721733165656798)	897 (0.14)	526 (0.26)
parity	4	2214(0.04572996455927746)	1516 (0.069)	2548 (0.033)
partially	4	2214(0.04572996455927746)	#SAKNAS!	1233 (0.1)
partial-mouth	4	2214(0.04572996455927746)	#SAKNAS!	1233 (0.1)
participants	4	1027(0.12575740253801304)	897 (0.14)	999 (0.13)
participatory	4	2214(0.04572996455927746)	#SAKNAS!	1668 (0.066)
particular	4	2214(0.04572996455927746)	1516 (0.069)	2548 (0.033)
pastures	4	2214(0.04572996455927746)	#SAKNAS!	1668 (0.066)
pathogenesis	4	1894(0.05716245569909683)	897 (0.14)	2548 (0.033)
pathologies	4	2214(0.04572996455927746)	#SAKNAS!	1668 (0.066)
pathology	4	1219(0.1028924202583743)	897 (0.14)	1233 (0.1)
pathophysiology	4	2214(0.04572996455927746)	#SAKNAS!	1233 (0.1)
pediatrics	4	2214(0.04572996455927746)	2359 (0.034)	1668 (0.066)
pelleted	4	2214(0.04572996455927746)	1121 (0.1)	2548 (0.033)
penaeus	4	2214(0.04572996455927746)	1121 (0.1)	#SAKNAS!
penetration	4	2214(0.04572996455927746)	#SAKNAS!	1233 (0.1)
pepper	4	2214(0.04572996455927746)	1516 (0.069)	1668 (0.066)
perception	4	410(0.33154224305476165)	897 (0.14)	461 (0.3)
periodontitis-a	4	2214(0.04572996455927746)	#SAKNAS!	2548 (0.033)
periodontitis-associated	4	2214(0.04572996455927746)	2359 (0.034)	1668 (0.066)
peripheral	4	896(0.14862238481765175)	897 (0.14)	584 (0.23)
per-oral	4	2214(0.04572996455927746)	#SAKNAS!	999 (0.13)
perspectives	4	966(0.1371898936778324)	897 (0.14)	694 (0.2)
pest	4	2214(0.04572996455927746)	2359 (0.034)	1668 (0.066)
phases	4	2214(0.04572996455927746)	2359 (0.034)	#SAKNAS!
phenomenon	4	2214(0.04572996455927746)	2359 (0.034)	2548 (0.033)

phenotypic	4	2214(0.04572996455927746)	2359 (0.034)	1668 (0.066)
phloem	4	2214(0.04572996455927746)	1516 (0.069)	1668 (0.066)
physician	4	2214(0.04572996455927746)	2359 (0.034)	1233 (0.1)
physicians	4	2214(0.04572996455927746)	1516 (0.069)	1668 (0.066)
physiologic	4	2214(0.04572996455927746)	2359 (0.034)	2548 (0.033)
piglets	4	2214(0.04572996455927746)	#SAKNAS!	2548 (0.033)
pisum	4	2214(0.04572996455927746)	#SAKNAS!	#SAKNAS!
plain	4	2214(0.04572996455927746)	2359 (0.034)	1668 (0.066)
plastic	4	1894(0.05716245569909683)	897 (0.14)	2548 (0.033)
poisoning	4	2214(0.04572996455927746)	#SAKNAS!	1668 (0.066)
polysaccharide	4	2214(0.04572996455927746)	#SAKNAS!	#SAKNAS!
pompe	4	2214(0.04572996455927746)	2359 (0.034)	2548 (0.033)
positively	4	2214(0.04572996455927746)	#SAKNAS!	1668 (0.066)
post-acute	4	2214(0.04572996455927746)	#SAKNAS!	1233 (0.1)
postnatal	4	1894(0.05716245569909683)	897 (0.14)	2548 (0.033)
post-operative	4	2214(0.04572996455927746)	#SAKNAS!	999 (0.13)
post-stroke	4	309(0.43443466331313596)	897 (0.14)	999 (0.13)
prader-willi	4	1678(0.0685949468389162)	897 (0.14)	1668 (0.066)
precision	4	2214(0.04572996455927746)	2359 (0.034)	2548 (0.033)
predator	4	1485(0.08002743797873557)	897 (0.14)	1668 (0.066)
predators	4	2214(0.04572996455927746)	2359 (0.034)	1668 (0.066)
pre-eclampsia	4	2214(0.04572996455927746)	1121 (0.1)	2548 (0.033)
presbyphagia	4	2214(0.04572996455927746)	#SAKNAS!	1668 (0.066)
presence	4	787(0.1714873670972905)	897 (0.14)	584 (0.23)
presentation	4	1111(0.11432491139819366)	897 (0.14)	999 (0.13)
prevent	4	787(0.1714873670972905)	897 (0.14)	816 (0.16)
prevertebral	4	2214(0.04572996455927746)	2359 (0.034)	2548 (0.033)
previous	4	2214(0.04572996455927746)	2359 (0.034)	2548 (0.033)
previously	4	2214(0.04572996455927746)	1516 (0.069)	2548 (0.033)
primate	4	2214(0.04572996455927746)	#SAKNAS!	999 (0.13)
proactive	4	2214(0.04572996455927746)	2359 (0.034)	2548 (0.033)
problem	4	2214(0.04572996455927746)	2359 (0.034)	2548 (0.033)
process	4	896(0.14862238481765175)	897 (0.14)	461 (0.3)
profiles	4	559(0.2400823139362067)	897 (0.14)	422 (0.33)
prognostic	4	595(0.22864982279638732)	897 (0.14)	694 (0.2)
programmes	4	2214(0.04572996455927746)	897 (0.14)	#SAKNAS!
programmes	4	2214(0.04572996455927746)	897 (0.14)	#SAKNAS!
promoter	4	2214(0.04572996455927746)	1121 (0.1)	#SAKNAS!
proposal	4	2214(0.04572996455927746)	2359 (0.034)	2548 (0.033)
prosthetic	4	1219(0.1028924202583743)	897 (0.14)	1668 (0.066)
province	4	2214(0.04572996455927746)	#SAKNAS!	1233 (0.1)
provision	4	1027(0.12575740253801304)	897 (0.14)	1233 (0.1)
psoriasis	4	2214(0.04572996455927746)	2359 (0.034)	1668 (0.066)

pulse	4	2214(0.04572996455927746)	2359 (0.034)	2548 (0.033)
pupils	4	2214(0.04572996455927746)	1516 (0.069)	2548 (0.033)
quantity	4	1678(0.0685949468389162)	897 (0.14)	2548 (0.033)
race	4	2214(0.04572996455927746)	2359 (0.034)	1668 (0.066)
rapid	4	1678(0.0685949468389162)	897 (0.14)	#SAKNAS!
ratios	4	2214(0.04572996455927746)	2359 (0.034)	2548 (0.033)
real-time	4	2214(0.04572996455927746)	#SAKNAS!	1668 (0.066)
rearing	4	2214(0.04572996455927746)	2359 (0.034)	1668 (0.066)
receipt	4	2214(0.04572996455927746)	1516 (0.069)	1668 (0.066)
recession	4	2214(0.04572996455927746)	1516 (0.069)	2548 (0.033)
recording	4	2214(0.04572996455927746)	1516 (0.069)	2548 (0.033)
recurrent	4	743(0.18291985823710985)	897 (0.14)	816 (0.16)
reflex	4	2214(0.04572996455927746)	#SAKNAS!	1233 (0.1)
refugees	4	2214(0.04572996455927746)	#SAKNAS!	1668 (0.066)
region	4	743(0.18291985823710985)	897 (0.14)	999 (0.13)
regions	4	1485(0.08002743797873557)	897 (0.14)	2548 (0.033)
regular	4	2214(0.04572996455927746)	2359 (0.034)	1668 (0.066)
reinforcement	4	2214(0.04572996455927746)	1516 (0.069)	1668 (0.066)
relate	4	2214(0.04572996455927746)	1121 (0.1)	#SAKNAS!
relatives	4	2214(0.04572996455927746)	#SAKNAS!	1668 (0.066)
release	4	2214(0.04572996455927746)	1516 (0.069)	2548 (0.033)
relevance	4	1335(0.09145992911855493)	897 (0.14)	1233 (0.1)
reliability	4	836(0.16005487595747114)	897 (0.14)	694 (0.2)
renal	4	836(0.16005487595747114)	897 (0.14)	816 (0.16)
replacing	4	2214(0.04572996455927746)	2359 (0.034)	2548 (0.033)
reporting	4	2214(0.04572996455927746)	#SAKNAS!	#SAKNAS!
resident	4	2214(0.04572996455927746)	2359 (0.034)	1668 (0.066)
residing	4	2214(0.04572996455927746)	1516 (0.069)	#SAKNAS!
residue	4	2214(0.04572996455927746)	#SAKNAS!	999 (0.13)
resistin	4	2214(0.04572996455927746)	#SAKNAS!	999 (0.13)
resonance	4	2214(0.04572996455927746)	2359 (0.034)	2548 (0.033)
restorations	4	2214(0.04572996455927746)	1516 (0.069)	1668 (0.066)
retention	4	1485(0.08002743797873557)	897 (0.14)	1668 (0.066)
rethinking	4	2214(0.04572996455927746)	1121 (0.1)	#SAKNAS!
retinopathy	4	2214(0.04572996455927746)	#SAKNAS!	1233 (0.1)
reuteri	4	2214(0.04572996455927746)	#SAKNAS!	1233 (0.1)
reward	4	836(0.16005487595747114)	897 (0.14)	461 (0.3)
rheological	4	628(0.21721733165656798)	897 (0.14)	999 (0.13)
rheology	4	2214(0.04572996455927746)	1516 (0.069)	#SAKNAS!
rhythm	4	2214(0.04572996455927746)	1516 (0.069)	2548 (0.033)
rhythms	4	2214(0.04572996455927746)	2359 (0.034)	1668 (0.066)
rico	4	2214(0.04572996455927746)	1516 (0.069)	1668 (0.066)
riyadh	4	2214(0.04572996455927746)	2359 (0.034)	2548 (0.033)

rodent	4	2214(0.04572996455927746)	2359 (0.034)	1233 (0.1)
roles	4	1027(0.12575740253801304)	897 (0.14)	999 (0.13)
route	4	2214(0.04572996455927746)	1121 (0.1)	2548 (0.033)
salvador	4	2214(0.04572996455927746)	#SAKNAS!	1233 (0.1)
satisfaction	4	1111(0.11432491139819366)	897 (0.14)	999 (0.13)
scales	4	2214(0.04572996455927746)	2359 (0.034)	1233 (0.1)
schistocerca	4	2214(0.04572996455927746)	1121 (0.1)	2548 (0.033)
schizophrenia	4	2214(0.04572996455927746)	2359 (0.034)	2548 (0.033)
scoping	4	2214(0.04572996455927746)	#SAKNAS!	2548 (0.033)
sealants	4	2214(0.04572996455927746)	#SAKNAS!	1668 (0.066)
secondhand	4	2214(0.04572996455927746)	2359 (0.034)	2548 (0.033)
secretion	4	836(0.16005487595747114)	897 (0.14)	584 (0.23)
selectivity	4	966(0.1371898936778324)	897 (0.14)	694 (0.2)
self-care	4	2214(0.04572996455927746)	#SAKNAS!	1668 (0.066)
self-report	4	1111(0.11432491139819366)	897 (0.14)	1233 (0.1)
series	4	481(0.2858122784954842)	897 (0.14)	461 (0.3)
seston	4	2214(0.04572996455927746)	897 (0.14)	#SAKNAS!
seston	4	2214(0.04572996455927746)	897 (0.14)	#SAKNAS!
severely	4	2214(0.04572996455927746)	1516 (0.069)	#SAKNAS!
sex	4	559(0.2400823139362067)	897 (0.14)	584 (0.23)
shifts	4	2214(0.04572996455927746)	2359 (0.034)	2548 (0.033)
simplex	4	2214(0.04572996455927746)	1516 (0.069)	2548 (0.033)
simulation	4	2214(0.04572996455927746)	#SAKNAS!	1668 (0.066)
since	4	2214(0.04572996455927746)	2359 (0.034)	1233 (0.1)
single	4	462(0.2972447696353035)	897 (0.14)	388 (0.36)
single-level	4	2214(0.04572996455927746)	#SAKNAS!	1668 (0.066)
situation	4	2214(0.04572996455927746)	2359 (0.034)	1668 (0.066)
sjogren	4	2214(0.04572996455927746)	2359 (0.034)	1668 (0.066)
skeletal	4	743(0.18291985823710985)	897 (0.14)	526 (0.26)
skills	4	2214(0.04572996455927746)	2359 (0.034)	1233 (0.1)
skipping	4	2214(0.04572996455927746)	#SAKNAS!	2548 (0.033)
skull	4	2214(0.04572996455927746)	1516 (0.069)	#SAKNAS!
snacks	4	1894(0.05716245569909683)	897 (0.14)	2548 (0.033)
snail	4	2214(0.04572996455927746)	#SAKNAS!	1668 (0.066)
solitary	4	2214(0.04572996455927746)	1516 (0.069)	1668 (0.066)
sonic	4	2214(0.04572996455927746)	#SAKNAS!	1233 (0.1)
southeast	4	2214(0.04572996455927746)	2359 (0.034)	#SAKNAS!
sows	4	1335(0.09145992911855493)	897 (0.14)	2548 (0.033)
specificity	4	2214(0.04572996455927746)	2359 (0.034)	2548 (0.033)
spider	4	2214(0.04572996455927746)	1516 (0.069)	1668 (0.066)
sport	4	2214(0.04572996455927746)	2359 (0.034)	1233 (0.1)
spp	4	2214(0.04572996455927746)	1516 (0.069)	2548 (0.033)
spruce	4	2214(0.04572996455927746)	897 (0.14)	#SAKNAS!

spruce	4	2214(0.04572996455927746)	897 (0.14)	#SAKNAS!
sri	4	2214(0.04572996455927746)	1516 (0.069)	1668 (0.066)
staff	4	896(0.14862238481765175)	897 (0.14)	584 (0.23)
standardization	4	2214(0.04572996455927746)	#SAKNAS!	2548 (0.033)
statement	4	2214(0.04572996455927746)	2359 (0.034)	1233 (0.1)
storage	4	2214(0.04572996455927746)	2359 (0.034)	2548 (0.033)
straw	4	2214(0.04572996455927746)	1516 (0.069)	2548 (0.033)
strongly	4	2214(0.04572996455927746)	#SAKNAS!	1233 (0.1)
subclinical	4	2214(0.04572996455927746)	2359 (0.034)	1668 (0.066)
submandibular	4	2214(0.04572996455927746)	1121 (0.1)	#SAKNAS!
suboptimal	4	2214(0.04572996455927746)	#SAKNAS!	1668 (0.066)
substances	4	2214(0.04572996455927746)	2359 (0.034)	2548 (0.033)
subtypes	4	2214(0.04572996455927746)	2359 (0.034)	#SAKNAS!
sucrose	4	896(0.14862238481765175)	897 (0.14)	526 (0.26)
suitability	4	2214(0.04572996455927746)	1516 (0.069)	1668 (0.066)
summer	4	2214(0.04572996455927746)	2359 (0.034)	#SAKNAS!
supplement	4	1111(0.11432491139819366)	897 (0.14)	1233 (0.1)
suppression	4	2214(0.04572996455927746)	897 (0.14)	#SAKNAS!
suppression	4	2214(0.04572996455927746)	897 (0.14)	#SAKNAS!
surface	4	836(0.16005487595747114)	897 (0.14)	999 (0.13)
sweetened	4	2214(0.04572996455927746)	#SAKNAS!	999 (0.13)
swiss	4	2214(0.04572996455927746)	2359 (0.034)	2548 (0.033)
switch	4	2214(0.04572996455927746)	1516 (0.069)	2548 (0.033)
symptomatic	4	2214(0.04572996455927746)	2359 (0.034)	1668 (0.066)
systems	4	787(0.1714873670972905)	897 (0.14)	1668 (0.066)
tanzania	4	1335(0.09145992911855493)	897 (0.14)	2548 (0.033)
target	4	2214(0.04572996455927746)	#SAKNAS!	1668 (0.066)
tastes	4	2214(0.04572996455927746)	2359 (0.034)	1668 (0.066)
technical	4	2214(0.04572996455927746)	1121 (0.1)	2548 (0.033)
teenagers	4	1485(0.08002743797873557)	897 (0.14)	2548 (0.033)
temperate	4	2214(0.04572996455927746)	1121 (0.1)	2548 (0.033)
temporary	4	2214(0.04572996455927746)	2359 (0.034)	1668 (0.066)
term	4	595(0.22864982279638732)	897 (0.14)	333 (0.43)
terms	4	2214(0.04572996455927746)	2359 (0.034)	2548 (0.033)
terrestris	4	2214(0.04572996455927746)	2359 (0.034)	#SAKNAS!
tests	4	1219(0.1028924202583743)	897 (0.14)	999 (0.13)
theoretical	4	2214(0.04572996455927746)	1121 (0.1)	#SAKNAS!
three-dimensional	4	2214(0.04572996455927746)	#SAKNAS!	2548 (0.033)
toxic	4	1678(0.0685949468389162)	897 (0.14)	2548 (0.033)
tracheoesophageal	4	2214(0.04572996455927746)	2359 (0.034)	1668 (0.066)
transcript	4	2214(0.04572996455927746)	1516 (0.069)	2548 (0.033)
transcutaneous	4	2214(0.04572996455927746)	2359 (0.034)	1668 (0.066)
transfer	4	1111(0.11432491139819366)	897 (0.14)	1233 (0.1)

transnasal		4	2214(0.04572996455927746)	2359 (0.034)	1233 (0.1)
transplant		4	2214(0.04572996455927746)	1516 (0.069)	#SAKNAS!
trend		4	2214(0.04572996455927746)	#SAKNAS!	1668 (0.066)
trends		4	410(0.33154224305476165)	897 (0.14)	333 (0.43)
trismus		4	2214(0.04572996455927746)	2359 (0.034)	1668 (0.066)
trout		4	1678(0.0685949468389162)	897 (0.14)	#SAKNAS!
tube-fed		4	2214(0.04572996455927746)	1516 (0.069)	2548 (0.033)
tumour		4	2214(0.04572996455927746)	897 (0.14)	#SAKNAS!
tumour		4	2214(0.04572996455927746)	897 (0.14)	#SAKNAS!
turkey		4	966(0.1371898936778324)	897 (0.14)	1668 (0.066)
ultrasonic		4	2214(0.04572996455927746)	2359 (0.034)	2548 (0.033)
undergraduate		4	2214(0.04572996455927746)	2359 (0.034)	2548 (0.033)
understand		4	2214(0.04572996455927746)	1516 (0.069)	1668 (0.066)
underweight		4	1027(0.12575740253801304)	897 (0.14)	584 (0.23)
unresectable		4	1219(0.1028924202583743)	897 (0.14)	816 (0.16)
update		4	896(0.14862238481765175)	897 (0.14)	694 (0.2)
uric		4	2214(0.04572996455927746)	#SAKNAS!	#SAKNAS!
v		4	2214(0.04572996455927746)	#SAKNAS!	999 (0.13)
validity		4	671(0.2057848405167486)	897 (0.14)	526 (0.26)
variable		4	2214(0.04572996455927746)	1516 (0.069)	1668 (0.066)
variants		4	2214(0.04572996455927746)	2359 (0.034)	1668 (0.066)
varies		4	2214(0.04572996455927746)	2359 (0.034)	#SAKNAS!
vegetable		4	535(0.2515148050760261)	897 (0.14)	461 (0.3)
virulence		4	1485(0.08002743797873557)	897 (0.14)	1668 (0.066)
vitamins		4	2214(0.04572996455927746)	#SAKNAS!	2548 (0.033)
waist		4	1485(0.08002743797873557)	897 (0.14)	2548 (0.033)
wall		4	2214(0.04572996455927746)	2359 (0.034)	2548 (0.033)
wasting		4	2214(0.04572996455927746)	2359 (0.034)	2548 (0.033)
waters		4	2214(0.04572996455927746)	1516 (0.069)	2548 (0.033)
wave		4	2214(0.04572996455927746)	2359 (0.034)	1668 (0.066)
wintering		4	2214(0.04572996455927746)	1516 (0.069)	1668 (0.066)
woman		4	2214(0.04572996455927746)	#SAKNAS!	#SAKNAS!
yield		4	2214(0.04572996455927746)	#SAKNAS!	#SAKNAS!
zealand		4	1219(0.1028924202583743)	897 (0.14)	1668 (0.066)
	1758	3	2693(0.0342974734194581)	#SAKNAS!	#SAKNAS!
	1966	3	2693(0.0342974734194581)	#SAKNAS!	2548 (0.033)
	2010	3	787(0.1714873670972905)	1121 (0.1)	461 (0.3)
	2021	3	2693(0.0342974734194581)	#SAKNAS!	#SAKNAS!
10-year-old		3	2693(0.0342974734194581)	2359 (0.034)	2548 (0.033)
12-month		3	2693(0.0342974734194581)	#SAKNAS!	2548 (0.033)
12-to		3	2693(0.0342974734194581)	#SAKNAS!	1668 (0.066)
12-year-olds		3	2693(0.0342974734194581)	#SAKNAS!	1668 (0.066)
18-year-old		3	2693(0.0342974734194581)	2359 (0.034)	1668 (0.066)

1999-2002	3	2693(0.0342974734194581)	1121 (0.1)	#SAKNAS!
1999-2002	3	2693(0.0342974734194581)	1121 (0.1)	#SAKNAS!
2008-2009	3	2693(0.0342974734194581)	2359 (0.034)	2548 (0.033)
2011-2018	3	2693(0.0342974734194581)	#SAKNAS!	#SAKNAS!
2012-2013	3	2693(0.0342974734194581)	#SAKNAS!	1233 (0.1)
2012-2014	3	2693(0.0342974734194581)	#SAKNAS!	1233 (0.1)
2015-2016	3	2693(0.0342974734194581)	#SAKNAS!	2548 (0.033)
2017-2018	3	2693(0.0342974734194581)	#SAKNAS!	#SAKNAS!
21st	3	2693(0.0342974734194581)	1516 (0.069)	2548 (0.033)
2-to	3	2693(0.0342974734194581)	2359 (0.034)	2548 (0.033)
30-day	3	2693(0.0342974734194581)	#SAKNAS!	2548 (0.033)
3d-printed	3	2693(0.0342974734194581)	#SAKNAS!	#SAKNAS!
3-year-old	3	1485(0.08002743797873557)	1121 (0.1)	1668 (0.066)
5-to	3	2693(0.0342974734194581)	2359 (0.034)	2548 (0.033)
5-year-old	3	1485(0.08002743797873557)	1121 (0.1)	1233 (0.1)
6-to	3	2693(0.0342974734194581)	1516 (0.069)	2548 (0.033)
6-year-old	3	2693(0.0342974734194581)	2359 (0.034)	#SAKNAS!
7-year-old	3	2693(0.0342974734194581)	1516 (0.069)	2548 (0.033)
80-year-old	3	2693(0.0342974734194581)	1516 (0.069)	2548 (0.033)
absence	3	2693(0.0342974734194581)	2359 (0.034)	2548 (0.033)
abundance	3	1485(0.08002743797873557)	1121 (0.1)	1668 (0.066)
abundant	3	2693(0.0342974734194581)	1516 (0.069)	2548 (0.033)
abuse	3	1894(0.05716245569909683)	1121 (0.1)	1668 (0.066)
acceptance	3	1111(0.11432491139819366)	1121 (0.1)	816 (0.16)
accuracy	3	1485(0.08002743797873557)	1121 (0.1)	1233 (0.1)
acellular	3	2693(0.0342974734194581)	1516 (0.069)	2548 (0.033)
acetate	3	2693(0.0342974734194581)	2359 (0.034)	2548 (0.033)
achieving	3	2693(0.0342974734194581)	#SAKNAS!	1668 (0.066)
acidic	3	2693(0.0342974734194581)	#SAKNAS!	1668 (0.066)
acquisition	3	2693(0.0342974734194581)	#SAKNAS!	1668 (0.066)
across	3	787(0.1714873670972905)	1121 (0.1)	999 (0.13)
activates	3	2693(0.0342974734194581)	2359 (0.034)	2548 (0.033)
acupuncture	3	2693(0.0342974734194581)	#SAKNAS!	2548 (0.033)
ad	3	1027(0.12575740253801304)	1121 (0.1)	1668 (0.066)
additive	3	2693(0.0342974734194581)	2359 (0.034)	2548 (0.033)
addressing	3	2693(0.0342974734194581)	2359 (0.034)	2548 (0.033)
adenocarcinoma	3	1678(0.0685949468389162)	1121 (0.1)	1233 (0.1)
adipokine	3	2693(0.0342974734194581)	#SAKNAS!	1668 (0.066)
adjunct	3	2693(0.0342974734194581)	#SAKNAS!	2548 (0.033)
adjunctive	3	2693(0.0342974734194581)	#SAKNAS!	2548 (0.033)
adjuvant	3	2693(0.0342974734194581)	#SAKNAS!	2548 (0.033)
advanced-stage	3	2693(0.0342974734194581)	1516 (0.069)	#SAKNAS!
advancement	3	2693(0.0342974734194581)	1516 (0.069)	2548 (0.033)

advancing	3	2693(0.0342974734194581)	#SAKNAS!	#SAKNAS!
aedes	3	2693(0.0342974734194581)	2359 (0.034)	2548 (0.033)
aerodigestive	3	2693(0.0342974734194581)	#SAKNAS!	2548 (0.033)
agriculture	3	2693(0.0342974734194581)	#SAKNAS!	2548 (0.033)
alaska	3	2693(0.0342974734194581)	1121 (0.1)	#SAKNAS!
alaska	3	2693(0.0342974734194581)	1121 (0.1)	#SAKNAS!
alaskan	3	2693(0.0342974734194581)	#SAKNAS!	1233 (0.1)
albicans	3	2693(0.0342974734194581)	2359 (0.034)	#SAKNAS!
aleyrodidae	3	2693(0.0342974734194581)	1516 (0.069)	2548 (0.033)
alimentary	3	1894(0.0571624556990968З)	1121 (0.1)	1668 (0.066)
all-cause	3	559(0.2400823139362067)	1121 (0.1)	816 (0.16)
alteration	3	2214(0.04572996455927746)	1121 (0.1)	2548 (0.033)
alternate	3	2693(0.0342974734194581)	1516 (0.069)	#SAKNAS!
amazon	3	2693(0.0342974734194581)	2359 (0.034)	1668 (0.066)
amphetamine-regulated	3	2693(0.0342974734194581)	1516 (0.069)	#SAKNAS!
analytical	3	2693(0.0342974734194581)	2359 (0.034)	#SAKNAS!
anatomical	3	2693(0.0342974734194581)	2359 (0.034)	#SAKNAS!
anesthesia	3	1027(0.12575740253801304)	1121 (0.1)	999 (0.13)
animals	3	1894(0.05716245569909683)	1121 (0.1)	2548 (0.033)
antagonistic	3	2693(0.0342974734194581)	2359 (0.034)	#SAKNAS!
anthropogenic	3	2693(0.0342974734194581)	#SAKNAS!	1668 (0.066)
antibiotics	3	2693(0.0342974734194581)	1516 (0.069)	#SAKNAS!
antibodies	3	787(0.1714873670972905)	1121 (0.1)	584 (0.23)
anticholinergic	3	2693(0.0342974734194581)	2359 (0.034)	#SAKNAS!
anticipatory	3	2693(0.0342974734194581)	2359 (0.034)	2548 (0.033)
antigen	3	2693(0.0342974734194581)	2359 (0.034)	2548 (0.033)
anti-inflammatory	3	2693(0.0342974734194581)	#SAKNAS!	2548 (0.033)
antioxidant	3	628(0.21721733165656798)	1121 (0.1)	694 (0.2)
ants	3	2693(0.0342974734194581)	2359 (0.034)	#SAKNAS!
anura	3	2693(0.0342974734194581)	2359 (0.034)	2548 (0.033)
aphid	3	787(0.1714873670972905)	1121 (0.1)	816 (0.16)
aphids	3	1485(0.08002743797873557)	1121 (0.1)	1668 (0.066)
aphis	3	2693(0.0342974734194581)	#SAKNAS!	1668 (0.066)
apnea-hypopnea	3	2693(0.0342974734194581)	#SAKNAS!	1668 (0.066)
apple	3	2693(0.0342974734194581)	#SAKNAS!	1668 (0.066)
approaches	3	1219(0.1028924202583743)	1121 (0.1)	999 (0.13)
aquatic	3	2693(0.0342974734194581)	#SAKNAS!	1668 (0.066)
arab	3	2214(0.04572996455927746)	1121 (0.1)	2548 (0.033)
architecture	3	2693(0.0342974734194581)	2359 (0.034)	#SAKNAS!
arctic	3	1894(0.05716245569909683)	1121 (0.1)	1668 (0.066)
areas	3	743(0.18291985823710985)	1121 (0.1)	461 (0.3)
aric	3	2693(0.0342974734194581)	2359 (0.034)	2548 (0.033)
armigera	3	2693(0.0342974734194581)	#SAKNAS!	1668 (0.066)

ascia	3	2693(0.0342974734194581)	1121 (0.1)	#SAKNAS!
ascia	3	2693(0.0342974734194581)	1121 (0.1)	#SAKNAS!
assessments	3	1678(0.0685949468389162)	1121 (0.1)	1668 (0.066)
assistance	3	2693(0.0342974734194581)	2359 (0.034)	#SAKNAS!
assurance	3	1678(0.0685949468389162)	1121 (0.1)	1233 (0.1)
asthma	3	836(0.16005487595747114)	1121 (0.1)	694 (0.2)
asymmetric	3	2693(0.0342974734194581)	2359 (0.034)	1668 (0.066)
atherogenic	3	1894(0.05716245569909683)	1121 (0.1)	2548 (0.033)
atherosclerotic	3	2693(0.0342974734194581)	2359 (0.034)	#SAKNAS!
athletes	3	1111(0.11432491139819366)	1121 (0.1)	999 (0.13)
attenuates	3	2693(0.0342974734194581)	#SAKNAS!	2548 (0.033)
aurata	3	2693(0.0342974734194581)	1121 (0.1)	#SAKNAS!
aurata	3	2693(0.0342974734194581)	1121 (0.1)	#SAKNAS!
australian	3	595(0.22864982279638732)	1121 (0.1)	305 (0.46)
avian	3	2214(0.04572996455927746)	1121 (0.1)	2548 (0.033)
avoidant	3	2693(0.0342974734194581)	#SAKNAS!	#SAKNAS!
away-from-home	3	2693(0.0342974734194581)	2359 (0.034)	1668 (0.066)
bacillus	3	2693(0.0342974734194581)	1516 (0.069)	2548 (0.033)
back	3	2214(0.04572996455927746)	1121 (0.1)	2548 (0.033)
bad	3	2693(0.0342974734194581)	1121 (0.1)	#SAKNAS!
bad	3	2693(0.0342974734194581)	1121 (0.1)	#SAKNAS!
balancing	3	1894(0.05716245569909683)	1121 (0.1)	1668 (0.066)
balloon	3	1485(0.08002743797873557)	1121 (0.1)	1668 (0.066)
banana	3	2693(0.0342974734194581)	#SAKNAS!	1233 (0.1)
bangladesh	3	896(0.14862238481765175)	1121 (0.1)	816 (0.16)
bangladeshi	3	2693(0.0342974734194581)	1516 (0.069)	2548 (0.033)
barcelona	3	2693(0.0342974734194581)	2359 (0.034)	2548 (0.033)
bark	3	2693(0.0342974734194581)	1121 (0.1)	#SAKNAS!
bark	3	2693(0.0342974734194581)	1121 (0.1)	#SAKNAS!
barriers	3	1027(0.12575740253801304)	1121 (0.1)	1233 (0.1)
basal	3	2693(0.0342974734194581)	#SAKNAS!	1668 (0.066)
base	3	1894(0.05716245569909683)	1121 (0.1)	2548 (0.033)
bases	3	2693(0.0342974734194581)	2359 (0.034)	2548 (0.033)
bay	3	2693(0.0342974734194581)	1516 (0.069)	2548 (0.033)
bdnf	3	2693(0.0342974734194581)	1516 (0.069)	#SAKNAS!
beetles	3	2693(0.0342974734194581)	2359 (0.034)	#SAKNAS!
bemisia	3	2693(0.0342974734194581)	2359 (0.034)	1668 (0.066)
benthic	3	1485(0.08002743797873557)	1121 (0.1)	1668 (0.066)
beverages	3	559(0.2400823139362067)	1121 (0.1)	361 (0.4)
beyond	3	1027(0.12575740253801304)	1121 (0.1)	999 (0.13)
big	3	2693(0.0342974734194581)	#SAKNAS!	2548 (0.033)
biliopancreatic	3	2693(0.0342974734194581)	2359 (0.034)	#SAKNAS!
bioavailability	3	2693(0.0342974734194581)	#SAKNAS!	1668 (0.066)

biofeedback	3	2693(0.0342974734194581)	#SAKNAS!	1668 (0.066)
biofilm	3	710(0.19435234937692925)	1121 (0.1)	694 (0.2)
biomarker	3	1678(0.0685949468389162)	1121 (0.1)	2548 (0.033)
biomechanics	3	2693(0.0342974734194581)	2359 (0.034)	1668 (0.066)
bites	3	2693(0.0342974734194581)	#SAKNAS!	1668 (0.066)
bivalve	3	2693(0.0342974734194581)	2359 (0.034)	2548 (0.033)
bivalvia	3	2693(0.0342974734194581)	1121 (0.1)	#SAKNAS!
bivalvia	3	2693(0.0342974734194581)	1121 (0.1)	#SAKNAS!
bladder	3	2693(0.0342974734194581)	#SAKNAS!	1233 (0.1)
blends	3	2693(0.0342974734194581)	#SAKNAS!	2548 (0.033)
blind	3	2693(0.0342974734194581)	2359 (0.034)	2548 (0.033)
blue	3	2693(0.0342974734194581)	1516 (0.069)	#SAKNAS!
body-mass	3	2693(0.0342974734194581)	1516 (0.069)	2548 (0.033)
bolus	3	1485(0.08002743797873557)	1121 (0.1)	2548 (0.033)
bombyx	3	2693(0.0342974734194581)	2359 (0.034)	2548 (0.033)
born	3	1678(0.0685949468389162)	1121 (0.1)	1233 (0.1)
box	3	2693(0.0342974734194581)	2359 (0.034)	2548 (0.033)
brainstem	3	1894(0.05716245569909683)	1121 (0.1)	2548 (0.033)
brazilians	3	2693(0.0342974734194581)	1516 (0.069)	#SAKNAS!
breakfast	3	836(0.16005487595747114)	1121 (0.1)	816 (0.16)
breast	3	787(0.1714873670972905)	1121 (0.1)	584 (0.23)
breast-feeding	3	1485(0.08002743797873557)	1121 (0.1)	1233 (0.1)
breeding	3	2693(0.0342974734194581)	1516 (0.069)	2548 (0.033)
bridge	3	2693(0.0342974734194581)	#SAKNAS!	1668 (0.066)
britain	3	1894(0.05716245569909683)	1121 (0.1)	2548 (0.033)
british	3	2214(0.04572996455927746)	1121 (0.1)	#SAKNAS!
browsing	3	1894(0.05716245569909683)	1121 (0.1)	2548 (0.033)
buccal	3	2693(0.0342974734194581)	2359 (0.034)	1668 (0.066)
budget	3	1678(0.0685949468389162)	1121 (0.1)	1668 (0.066)
budworm	3	2693(0.0342974734194581)	1121 (0.1)	#SAKNAS!
budworm	3	2693(0.0342974734194581)	1121 (0.1)	#SAKNAS!
burn	3	1678(0.0685949468389162)	1121 (0.1)	2548 (0.033)
c1-2	3	2693(0.0342974734194581)	#SAKNAS!	2548 (0.033)
c57bl	3	2693(0.0342974734194581)	2359 (0.034)	2548 (0.033)
cadmium	3	2693(0.0342974734194581)	1121 (0.1)	#SAKNAS!
cadmium	3	2693(0.0342974734194581)	1121 (0.1)	#SAKNAS!
cafeteria	3	2693(0.0342974734194581)	2359 (0.034)	2548 (0.033)
calcification	3	2693(0.0342974734194581)	#SAKNAS!	#SAKNAS!
caloric	3	1485(0.08002743797873557)	1121 (0.1)	1233 (0.1)
calorie	3	2693(0.0342974734194581)	2359 (0.034)	1668 (0.066)
cambodian	3	2693(0.0342974734194581)	#SAKNAS!	#SAKNAS!
candy	3	2693(0.0342974734194581)	#SAKNAS!	1668 (0.066)
cane	3	2693(0.0342974734194581)	#SAKNAS!	1668 (0.066)

capability	3	2693(0.0342974734194581)	#SAKNAS!	#SAKNAS!
capacity	3	836(0.16005487595747114)	1121 (0.1)	816 (0.16)
cape	3	2693(0.0342974734194581)	2359 (0.034)	1668 (0.066)
carcinomas	3	2214(0.04572996455927746)	1121 (0.1)	#SAKNAS!
cardiorespiratory	3	2693(0.0342974734194581)	#SAKNAS!	1668 (0.066)
carers	3	2693(0.0342974734194581)	#SAKNAS!	1233 (0.1)
caries-related	3	2693(0.0342974734194581)	2359 (0.034)	2548 (0.033)
carolina	3	2693(0.0342974734194581)	1516 (0.069)	#SAKNAS!
carp	3	2693(0.0342974734194581)	#SAKNAS!	1668 (0.066)
cart	3	2693(0.0342974734194581)	1516 (0.069)	#SAKNAS!
casein	3	2693(0.0342974734194581)	#SAKNAS!	2548 (0.033)
category	3	2693(0.0342974734194581)	#SAKNAS!	2548 (0.033)
caterpillar	3	2214(0.04572996455927746)	1121 (0.1)	2548 (0.033)
cd	3	2693(0.0342974734194581)	1516 (0.069)	2548 (0.033)
century	3	2214(0.04572996455927746)	1121 (0.1)	2548 (0.033)
ceratitis-capitata	3	2693(0.0342974734194581)	1121 (0.1)	#SAKNAS!
ceratitis-capitata	3	2693(0.0342974734194581)	1121 (0.1)	#SAKNAS!
cerebrospinal	3	2693(0.0342974734194581)	2359 (0.034)	2548 (0.033)
chain	3	2693(0.0342974734194581)	#SAKNAS!	#SAKNAS!
challenge	3	836(0.16005487595747114)	1121 (0.1)	694 (0.2)
channel	3	2693(0.0342974734194581)	2359 (0.034)	2548 (0.033)
checkups	3	2693(0.0342974734194581)	#SAKNAS!	#SAKNAS!
chemical	3	1111(0.11432491139819366)	1121 (0.1)	816 (0.16)
chew	3	2693(0.0342974734194581)	2359 (0.034)	#SAKNAS!
cholecystokinin	3	2693(0.0342974734194581)	1121 (0.1)	#SAKNAS!
cholecystokinin	3	2693(0.0342974734194581)	1121 (0.1)	#SAKNAS!
choose	3	2693(0.0342974734194581)	1121 (0.1)	#SAKNAS!
choose	3	2693(0.0342974734194581)	1121 (0.1)	#SAKNAS!
chronotype	3	2693(0.0342974734194581)	#SAKNAS!	1668 (0.066)
circuits	3	2693(0.0342974734194581)	1516 (0.069)	2548 (0.033)
citrus	3	2693(0.0342974734194581)	#SAKNAS!	2548 (0.033)
cleft	3	1219(0.1028924202583743)	1121 (0.1)	1668 (0.066)
clinicopathological	3	2693(0.0342974734194581)	#SAKNAS!	2548 (0.033)
clinics	3	2214(0.04572996455927746)	1121 (0.1)	#SAKNAS!
closure	3	2214(0.04572996455927746)	1121 (0.1)	#SAKNAS!
cluster	3	535(0.2515148050760261)	1121 (0.1)	388 (0.36)
coastal	3	1485(0.08002743797873557)	1121 (0.1)	2548 (0.033)
cod	3	2693(0.0342974734194581)	2359 (0.034)	2548 (0.033)
collaboration	3	2693(0.0342974734194581)	2359 (0.034)	2548 (0.033)
collaborative	3	2693(0.0342974734194581)	#SAKNAS!	#SAKNAS!
colon	3	1894(0.05716245569909683)	1121 (0.1)	1668 (0.066)
colonic	3	2693(0.0342974734194581)	#SAKNAS!	1233 (0.1)
colony	3	1485(0.08002743797873557)	1121 (0.1)	2548 (0.033)

commensal	3	2693(0.0342974734194581)	#SAKNAS!	1668 (0.066)
committee	3	2214(0.04572996455927746)	1121 (0.1)	2548 (0.033)
community-living	3	2693(0.0342974734194581)	#SAKNAS!	2548 (0.033)
comorbid	3	2693(0.0342974734194581)	2359 (0.034)	#SAKNAS!
compliance	3	1485(0.08002743797873557)	1121 (0.1)	1668 (0.066)
complicating	3	2693(0.0342974734194581)	1516 (0.069)	#SAKNAS!
compression	3	2693(0.0342974734194581)	#SAKNAS!	1668 (0.066)
concerning	3	2693(0.0342974734194581)	#SAKNAS!	2548 (0.033)
congenital	3	896(0.14862238481765175)	1121 (0.1)	816 (0.16)
congo	3	2693(0.0342974734194581)	1516 (0.069)	#SAKNAS!
considering	3	2693(0.0342974734194581)	2359 (0.034)	2548 (0.033)
contemporary	3	2693(0.0342974734194581)	2359 (0.034)	1668 (0.066)
contrasting	3	1485(0.08002743797873557)	1121 (0.1)	1233 (0.1)
contributing	3	2693(0.0342974734194581)	2359 (0.034)	2548 (0.033)
contributions	3	2693(0.0342974734194581)	#SAKNAS!	2548 (0.033)
controlling	3	2693(0.0342974734194581)	2359 (0.034)	1668 (0.066)
controls	3	1335(0.09145992911855493)	1121 (0.1)	816 (0.16)
cope	3	2693(0.0342974734194581)	2359 (0.034)	2548 (0.033)
corals	3	2693(0.0342974734194581)	1516 (0.069)	2548 (0.033)
correlate	3	1678(0.0685949468389162)	1121 (0.1)	1668 (0.066)
corticosterone	3	2693(0.0342974734194581)	1121 (0.1)	#SAKNAS!
corticosterone	3	2693(0.0342974734194581)	1121 (0.1)	#SAKNAS!
counselling	3	1335(0.09145992911855493)	1121 (0.1)	1233 (0.1)
country	3	1678(0.0685949468389162)	1121 (0.1)	1668 (0.066)
counts	3	1894(0.05716245569909683)	1121 (0.1)	1668 (0.066)
croatian	3	2693(0.0342974734194581)	#SAKNAS!	1668 (0.066)
cross	3	1027(0.12575740253801304)	1121 (0.1)	1233 (0.1)
crude	3	2693(0.0342974734194581)	1516 (0.069)	2548 (0.033)
cruzi	3	2693(0.0342974734194581)	#SAKNAS!	1668 (0.066)
cu	3	2693(0.0342974734194581)	2359 (0.034)	2548 (0.033)
cucumber	3	2693(0.0342974734194581)	2359 (0.034)	1668 (0.066)
curriculum	3	2693(0.0342974734194581)	2359 (0.034)	#SAKNAS!
cycles	3	2693(0.0342974734194581)	2359 (0.034)	1668 (0.066)
d2	3	2693(0.0342974734194581)	2359 (0.034)	2548 (0.033)
d3	3	2693(0.0342974734194581)	1516 (0.069)	#SAKNAS!
decades	3	2693(0.0342974734194581)	#SAKNAS!	1668 (0.066)
decannulation	3	2693(0.0342974734194581)	#SAKNAS!	1668 (0.066)
decapoda	3	2693(0.0342974734194581)	1516 (0.069)	2548 (0.033)
decision-making	3	1678(0.0685949468389162)	1121 (0.1)	1668 (0.066)
decrease	3	1335(0.09145992911855493)	1121 (0.1)	1668 (0.066)
defect	3	2693(0.0342974734194581)	#SAKNAS!	1233 (0.1)
defenses	3	2693(0.0342974734194581)	2359 (0.034)	2548 (0.033)
deficit	3	1678(0.0685949468389162)	1121 (0.1)	1233 (0.1)

definition	3 2693(0.0342974734194581)	#SAKNAS!	1668 (0.066)
definitions	3 2693(0.0342974734194581)	1516 (0.069)	#SAKNAS!
deformity	3 2693(0.0342974734194581)	#SAKNAS!	1668 (0.066)
dehydration	3 2693(0.0342974734194581)	2359 (0.034)	#SAKNAS!
delirium	3 2693(0.0342974734194581)	#SAKNAS!	2548 (0.033)
delivering	3 2693(0.0342974734194581)	#SAKNAS!	#SAKNAS!
demographic	3 1678(0.0685949468389162)	1121 (0.1)	#SAKNAS!
dentin	3 2693(0.0342974734194581)	#SAKNAS!	2548 (0.033)
dentist	3 1678(0.0685949468389162)	1121 (0.1)	1668 (0.066)
dependency	3 2693(0.0342974734194581)	2359 (0.034)	2548 (0.033)
depletion	3 2693(0.0342974734194581)	1516 (0.069)	#SAKNAS!
deprivation	3 1335(0.09145992911855493)	1121 (0.1)	1668 (0.066)
depth	3 2693(0.0342974734194581)	#SAKNAS!	1668 (0.066)
descriptive	3 966(0.1371898936778324)	1121 (0.1)	816 (0.16)
desert	3 1894(0.05716245569909683)	1121 (0.1)	1668 (0.066)
designing	3 2693(0.0342974734194581)	#SAKNAS!	#SAKNAS!
destructive	3 2693(0.0342974734194581)	1516 (0.069)	#SAKNAS!
detergent	3 2693(0.0342974734194581)	#SAKNAS!	2548 (0.033)
deterioration	3 2693(0.0342974734194581)	2359 (0.034)	2548 (0.033)
determination	3 2214(0.04572996455927746)	1121 (0.1)	2548 (0.033)
develop	3 2693(0.0342974734194581)	1516 (0.069)	#SAKNAS!
diabetics	3 2693(0.0342974734194581)	2359 (0.034)	2548 (0.033)
diagnoses	3 2693(0.0342974734194581)	1516 (0.069)	2548 (0.033)
diarrhea	3 2693(0.0342974734194581)	2359 (0.034)	2548 (0.033)
diastolic	3 2693(0.0342974734194581)	2359 (0.034)	1668 (0.066)
dietetics	3 2214(0.04572996455927746)	1121 (0.1)	2548 (0.033)
dietitian	3 2693(0.0342974734194581)	#SAKNAS!	2548 (0.033)
differ	3 2693(0.0342974734194581)	2359 (0.034)	2548 (0.033)
differentiation	3 1678(0.0685949468389162)	1121 (0.1)	1668 (0.066)
differing	3 2214(0.04572996455927746)	1121 (0.1)	2548 (0.033)
difficult	3 2693(0.0342974734194581)	2359 (0.034)	2548 (0.033)
digestive	3 595(0.22864982279638732)	1121 (0.1)	388 (0.36)
digital	3 2693(0.0342974734194581)	#SAKNAS!	2548 (0.033)
dilatation	3 1678(0.0685949468389162)	1121 (0.1)	1233 (0.1)
dimensions	3 2693(0.0342974734194581)	#SAKNAS!	2548 (0.033)
dimorphic	3 2693(0.0342974734194581)	2359 (0.034)	1668 (0.066)
disability	3 966(0.1371898936778324)	1121 (0.1)	999 (0.13)
disc	3 2693(0.0342974734194581)	#SAKNAS!	2548 (0.033)
discrimination	3 2214(0.04572996455927746)	1121 (0.1)	2548 (0.033)
display	3 2693(0.0342974734194581)	2359 (0.034)	2548 (0.033)
disruption	3 2693(0.0342974734194581)	2359 (0.034)	#SAKNAS!
dissatisfaction	3 2693(0.0342974734194581)	2359 (0.034)	1668 (0.066)
dissection	3 1485(0.08002743797873557)	1121 (0.1)	2548 (0.033)

distress	3	2693(0.0342974734194581)	#SAKNAS!	2548 (0.033)
district	3	1027(0.12575740253801304)	1121 (0.1)	816 (0.16)
diversion	3	2693(0.0342974734194581)	2359 (0.034)	1668 (0.066)
diversity	3	595(0.22864982279638732)	1121 (0.1)	694 (0.2)
diverticulectomy	3	2214(0.04572996455927746)	1121 (0.1)	2548 (0.033)
diving	3	2214(0.04572996455927746)	1121 (0.1)	2548 (0.033)
dog	3	2693(0.0342974734194581)	#SAKNAS!	1668 (0.066)
dominance	3	1894(0.05716245569909683)	1121 (0.1)	1668 (0.066)
dopaminergic	3	2693(0.0342974734194581)	1121 (0.1)	#SAKNAS!
dopaminergic	3	2693(0.0342974734194581)	1121 (0.1)	#SAKNAS!
dosimetric	3	2693(0.0342974734194581)	2359 (0.034)	1668 (0.066)
drive	3	2693(0.0342974734194581)	2359 (0.034)	2548 (0.033)
drives	3	2693(0.0342974734194581)	#SAKNAS!	2548 (0.033)
drugs	3	2693(0.0342974734194581)	#SAKNAS!	#SAKNAS!
dsm	3	2693(0.0342974734194581)	#SAKNAS!	1668 (0.066)
duchenne	3	1027(0.12575740253801304)	1121 (0.1)	1233 (0.1)
dying	3	2693(0.0342974734194581)	2359 (0.034)	2548 (0.033)
dynamic	3	1335(0.09145992911855493)	1121 (0.1)	1668 (0.066)
dynamics	3	1111(0.11432491139819366)	1121 (0.1)	816 (0.16)
dysfunctions	3	2693(0.0342974734194581)	#SAKNAS!	2548 (0.033)
dysmotility	3	2693(0.0342974734194581)	2359 (0.034)	2548 (0.033)
dyspepsia	3	2693(0.0342974734194581)	#SAKNAS!	1233 (0.1)
early-stage	3	2693(0.0342974734194581)	#SAKNAS!	2548 (0.033)
earthworm	3	2693(0.0342974734194581)	#SAKNAS!	1233 (0.1)
eastern	3	1219(0.1028924202583743)	1121 (0.1)	1233 (0.1)
eaten	3	2693(0.0342974734194581)	1516 (0.069)	2548 (0.033)
edentulousness	3	2693(0.0342974734194581)	1516 (0.069)	2548 (0.033)
educational	3	1111(0.11432491139819366)	1121 (0.1)	1233 (0.1)
ehlers-danlos	3	2693(0.0342974734194581)	#SAKNAS!	1668 (0.066)
element	3	2214(0.04572996455927746)	1121 (0.1)	#SAKNAS!
elementary	3	1027(0.12575740253801304)	1121 (0.1)	816 (0.16)
elements	3	2693(0.0342974734194581)	2359 (0.034)	1668 (0.066)
elevation	3	1485(0.08002743797873557)	1121 (0.1)	1233 (0.1)
emirates	3	2214(0.04572996455927746)	1121 (0.1)	2548 (0.033)
employees	3	2693(0.0342974734194581)	#SAKNAS!	1668 (0.066)
employment	3	2693(0.0342974734194581)	#SAKNAS!	1233 (0.1)
emulsion	3	2693(0.0342974734194581)	#SAKNAS!	2548 (0.033)
encephalopathy	3	2693(0.0342974734194581)	1516 (0.069)	#SAKNAS!
endoscopy	3	1678(0.0685949468389162)	1121 (0.1)	2548 (0.033)
endothelial	3	2693(0.0342974734194581)	#SAKNAS!	2548 (0.033)
endotracheal	3	2693(0.0342974734194581)	2359 (0.034)	#SAKNAS!
energy-intake	3	2693(0.0342974734194581)	1121 (0.1)	#SAKNAS!
energy-intake	3	2693(0.0342974734194581)	1121 (0.1)	#SAKNAS!

engagement	3	2693(0.0342974734194581)	#SAKNAS!	#SAKNAS!
england	3	1894(0.0571624556990968З)	1121 (0.1)	2548 (0.033)
enjoyment	3	2693(0.0342974734194581)	#SAKNAS!	1668 (0.066)
ent	3	2214(0.04572996455927746)	1121 (0.1)	#SAKNAS!
entry	3	2693(0.0342974734194581)	#SAKNAS!	1668 (0.066)
erica	3	2693(0.0342974734194581)	#SAKNAS!	1668 (0.066)
erosion	3	1111(0.11432491139819366)	1121 (0.1)	999 (0.13)
essential	3	787(0.1714873670972905)	1121 (0.1)	1233 (0.1)
ethical	3	2693(0.0342974734194581)	1516 (0.069)	2548 (0.033)
ethics	3	2693(0.0342974734194581)	2359 (0.034)	2548 (0.033)
ethnic	3	1219(0.1028924202583743)	1121 (0.1)	1233 (0.1)
ethnicity	3	2693(0.0342974734194581)	2359 (0.034)	#SAKNAS!
ethylene	3	2693(0.0342974734194581)	#SAKNAS!	#SAKNAS!
eulemur	3	2693(0.0342974734194581)	1121 (0.1)	#SAKNAS!
eulemur	3	2693(0.0342974734194581)	1121 (0.1)	#SAKNAS!
evaluate	3	1335(0.09145992911855493)	1121 (0.1)	2548 (0.033)
evaluating	3	628(0.21721733165656798)	1121 (0.1)	422 (0.33)
example	3	2693(0.0342974734194581)	1121 (0.1)	#SAKNAS!
example	3	2693(0.0342974734194581)	1121 (0.1)	#SAKNAS!
exclusive	3	1219(0.1028924202583743)	1121 (0.1)	2548 (0.033)
experiencing	3	2214(0.04572996455927746)	1121 (0.1)	#SAKNAS!
experiment	3	2693(0.0342974734194581)	2359 (0.034)	2548 (0.033)
exploration	3	966(0.1371898936778324)	1121 (0.1)	1233 (0.1)
exploratory	3	1027(0.12575740253801304)	1121 (0.1)	1233 (0.1)
facilitates	3	2693(0.0342974734194581)	1516 (0.069)	2548 (0.033)
faecal	3	2693(0.0342974734194581)	1516 (0.069)	2548 (0.033)
familial	3	1678(0.0685949468389162)	1121 (0.1)	1233 (0.1)
farm	3	2693(0.0342974734194581)	1516 (0.069)	#SAKNAS!
farming	3	2693(0.0342974734194581)	2359 (0.034)	2548 (0.033)
fascicularis	3	2693(0.0342974734194581)	#SAKNAS!	2548 (0.033)
fatal	3	2693(0.0342974734194581)	1121 (0.1)	#SAKNAS!
fatal	3	2693(0.0342974734194581)	1121 (0.1)	#SAKNAS!
feasibility	3	439(0.30867726077512286)	1121 (0.1)	280 (0.49)
feasible	3	2693(0.0342974734194581)	2359 (0.034)	1668 (0.066)
fecundity	3	1485(0.08002743797873557)	1121 (0.1)	1668 (0.066)
feeder	3	2693(0.0342974734194581)	2359 (0.034)	2548 (0.033)
feeders	3	2693(0.0342974734194581)	1516 (0.069)	2548 (0.033)
feeds	3	2214(0.04572996455927746)	1121 (0.1)	2548 (0.033)
fibre	3	1485(0.08002743797873557)	1121 (0.1)	1668 (0.066)
fibrinogen	3	2693(0.0342974734194581)	1516 (0.069)	2548 (0.033)
filipino	3	2693(0.0342974734194581)	1121 (0.1)	#SAKNAS!
filipino	3	2693(0.0342974734194581)	1121 (0.1)	#SAKNAS!
fire	3	2693(0.0342974734194581)	1121 (0.1)	#SAKNAS!

fire	3	2693(0.0342974734194581)	1121 (0.1)	#SAKNAS!
five-year	3	2693(0.0342974734194581)	2359 (0.034)	#SAKNAS!
flaps	3	2693(0.0342974734194581)	1516 (0.069)	2548 (0.033)
flavonoids	3	2693(0.0342974734194581)	2359 (0.034)	#SAKNAS!
flavor	3	2214(0.04572996455927746)	1121 (0.1)	#SAKNAS!
flexibility	3	2693(0.0342974734194581)	1516 (0.069)	2548 (0.033)
flies	3	1678(0.0685949468389162)	1121 (0.1)	#SAKNAS!
flower	3	2693(0.0342974734194581)	2359 (0.034)	2548 (0.033)
foliage	3	2693(0.0342974734194581)	1516 (0.069)	#SAKNAS!
folic	3	2693(0.0342974734194581)	2359 (0.034)	#SAKNAS!
food-based	3	1894(0.0571624556990968З)	1121 (0.1)	2548 (0.033)
forage	3	1219(0.1028924202583743)	1121 (0.1)	1233 (0.1)
foragers	3	2693(0.0342974734194581)	2359 (0.034)	1668 (0.066)
force	3	836(0.16005487595747114)	1121 (0.1)	816 (0.16)
forces	3	2693(0.0342974734194581)	2359 (0.034)	#SAKNAS!
forebrain	3	2693(0.0342974734194581)	#SAKNAS!	1668 (0.066)
forests	3	2693(0.0342974734194581)	2359 (0.034)	2548 (0.033)
form	3	1111(0.11432491139819366)	1121 (0.1)	1233 (0.1)
format	3	2693(0.0342974734194581)	#SAKNAS!	1668 (0.066)
formation	3	1111(0.11432491139819366)	1121 (0.1)	999 (0.13)
formulations	3	2693(0.0342974734194581)	#SAKNAS!	2548 (0.033)
forsythia	3	2693(0.0342974734194581)	1516 (0.069)	#SAKNAS!
fragments	3	2693(0.0342974734194581)	#SAKNAS!	1233 (0.1)
free-choice	3	2693(0.0342974734194581)	2359 (0.034)	2548 (0.033)
free-living	3	1485(0.08002743797873557)	1121 (0.1)	1668 (0.066)
frequent	3	2693(0.0342974734194581)	2359 (0.034)	2548 (0.033)
frugivorous	3	2693(0.0342974734194581)	2359 (0.034)	2548 (0.033)
fungi	3	2693(0.0342974734194581)	2359 (0.034)	2548 (0.033)
further	3	2693(0.0342974734194581)	2359 (0.034)	2548 (0.033)
fuscata	3	2693(0.0342974734194581)	1516 (0.069)	2548 (0.033)
fusobacterium	3	2693(0.0342974734194581)	1516 (0.069)	2548 (0.033)
galanin	3	2214(0.04572996455927746)	1121 (0.1)	2548 (0.033)
gastrojejunal	3	2693(0.0342974734194581)	2359 (0.034)	2548 (0.033)
gastrostomies	3	2214(0.04572996455927746)	1121 (0.1)	2548 (0.033)
geese	3	2214(0.04572996455927746)	1121 (0.1)	#SAKNAS!
gelatin	3	2693(0.0342974734194581)	2359 (0.034)	#SAKNAS!
gelling	3	2693(0.0342974734194581)	#SAKNAS!	#SAKNAS!
gender-specific	3	2693(0.0342974734194581)	2359 (0.034)	#SAKNAS!
genotype	3	1894(0.05716245569909683)	1121 (0.1)	#SAKNAS!
genotypes	3	2693(0.0342974734194581)	#SAKNAS!	1668 (0.066)
genus	3	2693(0.0342974734194581)	1516 (0.069)	2548 (0.033)
geographic	3	2693(0.0342974734194581)	2359 (0.034)	1668 (0.066)
gerd	3	1111(0.11432491139819366)	1121 (0.1)	999 (0.13)

gerontological	3	2214(0.04572996455927746)	1121 (0.1)	#SAKNAS!
giant	3	787(0.1714873670972905)	1121 (0.1)	461 (0.3)
gigas	3	2693(0.0342974734194581)	1516 (0.069)	2548 (0.033)
gilthead	3	2693(0.0342974734194581)	1121 (0.1)	#SAKNAS!
gilthead	3	2693(0.0342974734194581)	1121 (0.1)	#SAKNAS!
glim	3	2693(0.0342974734194581)	#SAKNAS!	2548 (0.033)
glucocorticoid	3	2693(0.0342974734194581)	2359 (0.034)	1668 (0.066)
goals	3	2693(0.0342974734194581)	#SAKNAS!	1668 (0.066)
goldfish	3	2693(0.0342974734194581)	1516 (0.069)	2548 (0.033)
gorilla	3	2693(0.0342974734194581)	1516 (0.069)	2548 (0.033)
gossypii	3	2693(0.0342974734194581)	#SAKNAS!	1668 (0.066)
grains	3	2693(0.0342974734194581)	1516 (0.069)	#SAKNAS!
gray	3	2693(0.0342974734194581)	2359 (0.034)	2548 (0.033)
greek	3	1678(0.0685949468389162)	1121 (0.1)	2548 (0.033)
green	3	1485(0.08002743797873557)	1121 (0.1)	999 (0.13)
ground	3	2693(0.0342974734194581)	1516 (0.069)	#SAKNAS!
group-housed	3	2693(0.0342974734194581)	1121 (0.1)	#SAKNAS!
group-housed	3	2693(0.0342974734194581)	1121 (0.1)	#SAKNAS!
guidance	3	2693(0.0342974734194581)	2359 (0.034)	2548 (0.033)
guinea	3	2693(0.0342974734194581)	2359 (0.034)	2548 (0.033)
gulf	3	2693(0.0342974734194581)	2359 (0.034)	2548 (0.033)
gut	3	516(0.26294729621584545)	1121 (0.1)	461 (0.3)
habitats	3	2693(0.0342974734194581)	2359 (0.034)	#SAKNAS!
hand	3	2693(0.0342974734194581)	#SAKNAS!	2548 (0.033)
hba1c	3	1027(0.12575740253801304)	1121 (0.1)	999 (0.13)
health-care	3	2693(0.0342974734194581)	1121 (0.1)	#SAKNAS!
health-care	3	2693(0.0342974734194581)	1121 (0.1)	#SAKNAS!
healthful	3	2693(0.0342974734194581)	2359 (0.034)	1668 (0.066)
health-promoting	3	1894(0.05716245569909683)	1121 (0.1)	2548 (0.033)
hearing	3	2693(0.0342974734194581)	#SAKNAS!	#SAKNAS!
heat	3	1678(0.0685949468389162)	1121 (0.1)	1668 (0.066)
hedonic	3	2693(0.0342974734194581)	2359 (0.034)	2548 (0.033)
helical	3	2693(0.0342974734194581)	#SAKNAS!	2548 (0.033)
helicobacter	3	1111(0.11432491139819366)	1121 (0.1)	999 (0.13)
helicoverpa	3	2693(0.0342974734194581)	#SAKNAS!	1668 (0.066)
hemorrhage	3	1111(0.11432491139819366)	1121 (0.1)	999 (0.13)
hepatitis	3	2693(0.0342974734194581)	#SAKNAS!	2548 (0.033)
herbivores	3	2693(0.0342974734194581)	1516 (0.069)	#SAKNAS!
herbivorous	3	1678(0.0685949468389162)	1121 (0.1)	1233 (0.1)
hernias	3	1894(0.05716245569909683)	1121 (0.1)	#SAKNAS!
heterotrophic	3	2693(0.0342974734194581)	1516 (0.069)	2548 (0.033)
high-calorie	3	2693(0.0342974734194581)	1121 (0.1)	#SAKNAS!
high-calorie	3	2693(0.0342974734194581)	1121 (0.1)	#SAKNAS!

high-fiber	3	2693(0.0342974734194581)	1516 (0.069)	2548 (0.033)
high-risk	3	1219(0.1028924202583743)	1121 (0.1)	1233 (0.1)
hill	3	2693(0.0342974734194581)	#SAKNAS!	1233 (0.1)
hispanic	3	1027(0.12575740253801304)	1121 (0.1)	526 (0.26)
homeostatic	3	2693(0.0342974734194581)	2359 (0.034)	1668 (0.066)
homocysteine	3	2693(0.0342974734194581)	1516 (0.069)	#SAKNAS!
hormonal	3	2214(0.04572996455927746)	1121 (0.1)	#SAKNAS!
horse	3	2693(0.0342974734194581)	2359 (0.034)	2548 (0.033)
horses	3	1111(0.11432491139819366)	1121 (0.1)	694 (0.2)
hospitalisation	3	2693(0.0342974734194581)	#SAKNAS!	1668 (0.066)
hummingbirds	3	2693(0.0342974734194581)	1121 (0.1)	#SAKNAS!
hummingbirds	3	2693(0.0342974734194581)	1121 (0.1)	#SAKNAS!
hungarian	3	2693(0.0342974734194581)	2359 (0.034)	#SAKNAS!
hunger	3	1678(0.0685949468389162)	1121 (0.1)	1668 (0.066)
huntington	3	1894(0.05716245569909683)	1121 (0.1)	2548 (0.033)
hydration	3	1335(0.09145992911855493)	1121 (0.1)	1668 (0.066)
hyperactivity	3	1335(0.09145992911855493)	1121 (0.1)	1233 (0.1)
hyperphagia	3	2693(0.0342974734194581)	2359 (0.034)	2548 (0.033)
hypopharynx	3	2693(0.0342974734194581)	2359 (0.034)	2548 (0.033)
hypothesis	3	1894(0.05716245569909683)	1121 (0.1)	1668 (0.066)
il-6	3	2693(0.0342974734194581)	2359 (0.034)	1668 (0.066)
ill	3	1111(0.11432491139819366)	1121 (0.1)	2548 (0.033)
image	3	1335(0.09145992911855493)	1121 (0.1)	999 (0.13)
images	3	2693(0.0342974734194581)	#SAKNAS!	1668 (0.066)
imbalance	3	2693(0.0342974734194581)	2359 (0.034)	2548 (0.033)
immature	3	2693(0.0342974734194581)	#SAKNAS!	1668 (0.066)
immediate	3	2693(0.0342974734194581)	2359 (0.034)	#SAKNAS!
immune-inflammation	3	2693(0.0342974734194581)	#SAKNAS!	#SAKNAS!
immunodeficiency	3	2693(0.0342974734194581)	#SAKNAS!	#SAKNAS!
impairments	3	1219(0.1028924202583743)	1121 (0.1)	1233 (0.1)
implants	3	2693(0.0342974734194581)	#SAKNAS!	1668 (0.066)
implication	3	2693(0.0342974734194581)	2359 (0.034)	2548 (0.033)
improvements	3	2693(0.0342974734194581)	2359 (0.034)	#SAKNAS!
inappropriate	3	1219(0.1028924202583743)	1121 (0.1)	1233 (0.1)
incident	3	896(0.14862238481765175)	1121 (0.1)	816 (0.16)
incisor	3	2693(0.0342974734194581)	2359 (0.034)	2548 (0.033)
incorporating	3	2693(0.0342974734194581)	#SAKNAS!	1668 (0.066)
increment	3	2693(0.0342974734194581)	2359 (0.034)	1668 (0.066)
indicate	3	2693(0.0342974734194581)	1516 (0.069)	#SAKNAS!
indications	3	1335(0.09145992911855493)	1121 (0.1)	999 (0.13)
indirect	3	2693(0.0342974734194581)	#SAKNAS!	2548 (0.033)
individually	3	1894(0.05716245569909683)	1121 (0.1)	2548 (0.033)
induction	3	1111(0.11432491139819366)	1121 (0.1)	1233 (0.1)

infection-related	3	2693(0.0342974734194581)	#SAKNAS!	2548 (0.033)
inhabiting	3	2693(0.0342974734194581)	2359 (0.034)	1668 (0.066)
inhalation	3	2693(0.0342974734194581)	#SAKNAS!	2548 (0.033)
inhibiting	3	2693(0.0342974734194581)	#SAKNAS!	#SAKNAS!
inhibitor	3	1678(0.0685949468389162)	1121 (0.1)	1668 (0.066)
inhibits	3	2693(0.0342974734194581)	2359 (0.034)	2548 (0.033)
initial	3	2214(0.04572996455927746)	1121 (0.1)	#SAKNAS!
injection	3	1485(0.08002743797873557)	1121 (0.1)	999 (0.13)
inoperable	3	1894(0.0571624556990968З)	1121 (0.1)	1668 (0.066)
inorganic	3	2693(0.0342974734194581)	2359 (0.034)	#SAKNAS!
instrument	3	1894(0.0571624556990968З)	1121 (0.1)	2548 (0.033)
instrumental	3	2693(0.0342974734194581)	#SAKNAS!	1668 (0.066)
instrumentation	3	2693(0.0342974734194581)	2359 (0.034)	2548 (0.033)
insufficiency	3	2693(0.0342974734194581)	2359 (0.034)	2548 (0.033)
insurance	3	836(0.16005487595747114)	1121 (0.1)	584 (0.23)
intense	3	2693(0.0342974734194581)	1121 (0.1)	#SAKNAS!
intense	3	2693(0.0342974734194581)	1121 (0.1)	#SAKNAS!
intensity-modulated	3	1111(0.11432491139819366)	1121 (0.1)	1233 (0.1)
interdialytic	3	2693(0.0342974734194581)	#SAKNAS!	2548 (0.033)
interleukin	3	2693(0.0342974734194581)	2359 (0.034)	1668 (0.066)
interposition	3	1485(0.08002743797873557)	1121 (0.1)	999 (0.13)
intertidal	3	2693(0.0342974734194581)	1516 (0.069)	#SAKNAS!
intervertebral	3	2693(0.0342974734194581)	#SAKNAS!	2548 (0.033)
intestine	3	2693(0.0342974734194581)	1121 (0.1)	#SAKNAS!
intestine	3	2693(0.0342974734194581)	1121 (0.1)	#SAKNAS!
intramural	3	2693(0.0342974734194581)	1516 (0.069)	#SAKNAS!
intramuscular	3	2693(0.0342974734194581)	#SAKNAS!	2548 (0.033)
intuitive	3	2693(0.0342974734194581)	#SAKNAS!	1668 (0.066)
invest	3	2693(0.0342974734194581)	1516 (0.069)	2548 (0.033)
involvement	3	1027(0.12575740253801304)	1121 (0.1)	816 (0.16)
irradiation	3	2693(0.0342974734194581)	1516 (0.069)	2548 (0.033)
irregularity	3	2693(0.0342974734194581)	#SAKNAS!	1233 (0.1)
irritable	3	2693(0.0342974734194581)	#SAKNAS!	1668 (0.066)
islands	3	1485(0.08002743797873557)	1121 (0.1)	#SAKNAS!
isolate	3	2693(0.0342974734194581)	#SAKNAS!	2548 (0.033)
isotopic	3	2693(0.0342974734194581)	#SAKNAS!	1233 (0.1)
japonica	3	2214(0.04572996455927746)	1121 (0.1)	#SAKNAS!
java	3	2693(0.0342974734194581)	2359 (0.034)	2548 (0.033)
joint	3	1027(0.12575740253801304)	1121 (0.1)	694 (0.2)
jordan	3	2693(0.0342974734194581)	1121 (0.1)	#SAKNAS!
jordan	3	2693(0.0342974734194581)	1121 (0.1)	#SAKNAS!
juan	3	2693(0.0342974734194581)	1516 (0.069)	2548 (0.033)
kids	3	2693(0.0342974734194581)	2359 (0.034)	#SAKNAS!

kinase	3	2214(0.04572996455927746)	1121 (0.1)	#SAKNAS!
koges	3	2693(0.0342974734194581)	#SAKNAS!	1668 (0.066)
konjac	3	2693(0.0342974734194581)	#SAKNAS!	#SAKNAS!
krill	3	2693(0.0342974734194581)	2359 (0.034)	1668 (0.066)
kyoto-kameoka	3	2693(0.0342974734194581)	#SAKNAS!	1668 (0.066)
lack	3	1219(0.1028924202583743)	1121 (0.1)	816 (0.16)
lactose	3	2693(0.0342974734194581)	#SAKNAS!	2548 (0.033)
lake	3	2693(0.0342974734194581)	1516 (0.069)	#SAKNAS!
lanka	3	2693(0.0342974734194581)	2359 (0.034)	1668 (0.066)
large-scale	3	2693(0.0342974734194581)	#SAKNAS!	1233 (0.1)
laryngotracheal	3	2693(0.0342974734194581)	1516 (0.069)	#SAKNAS!
latent	3	2693(0.0342974734194581)	#SAKNAS!	#SAKNAS!
late-onset	3	2693(0.0342974734194581)	#SAKNAS!	1668 (0.066)
lean	3	2214(0.04572996455927746)	1121 (0.1)	2548 (0.033)
leaves	3	1894(0.05716245569909683)	1121 (0.1)	1668 (0.066)
left	3	2214(0.04572996455927746)	1121 (0.1)	#SAKNAS!
lens	3	2693(0.0342974734194581)	2359 (0.034)	#SAKNAS!
leukocyte	3	2693(0.0342974734194581)	#SAKNAS!	1668 (0.066)
libitum	3	1219(0.1028924202583743)	1121 (0.1)	1668 (0.066)
lifespan	3	2693(0.0342974734194581)	#SAKNAS!	1668 (0.066)
life-style	3	2214(0.04572996455927746)	1121 (0.1)	#SAKNAS!
ligament	3	2693(0.0342974734194581)	2359 (0.034)	#SAKNAS!
like	3	2693(0.0342974734194581)	2359 (0.034)	1668 (0.066)
linear	3	2693(0.0342974734194581)	2359 (0.034)	1668 (0.066)
lingual	3	2693(0.0342974734194581)	1121 (0.1)	#SAKNAS!
lingual	3	2693(0.0342974734194581)	1121 (0.1)	#SAKNAS!
linxian	3	2693(0.0342974734194581)	#SAKNAS!	1668 (0.066)
litopenaeus	3	2693(0.0342974734194581)	2359 (0.034)	1668 (0.066)
litter	3	1894(0.05716245569909683)	1121 (0.1)	2548 (0.033)
liver	3	743(0.18291985823710985)	1121 (0.1)	584 (0.23)
lizard	3	2693(0.0342974734194581)	1516 (0.069)	2548 (0.033)
locations	3	2693(0.0342974734194581)	2359 (0.034)	2548 (0.033)
low-level	3	2693(0.0342974734194581)	#SAKNAS!	1668 (0.066)
low-resource	3	2693(0.0342974734194581)	#SAKNAS!	1668 (0.066)
low-risk	3	2693(0.0342974734194581)	2359 (0.034)	1668 (0.066)
lung	3	836(0.16005487595747114)	1121 (0.1)	694 (0.2)
m3	3	2693(0.0342974734194581)	#SAKNAS!	1233 (0.1)
macaca	3	1111(0.11432491139819366)	1121 (0.1)	816 (0.16)
macrobrachium	3	2693(0.0342974734194581)	2359 (0.034)	1668 (0.066)
madrid	3	2693(0.0342974734194581)	2359 (0.034)	1668 (0.066)
malaysia	3	2693(0.0342974734194581)	#SAKNAS!	2548 (0.033)
mammal	3	2693(0.0342974734194581)	1516 (0.069)	2548 (0.033)
mangrove	3	1678(0.0685949468389162)	1121 (0.1)	1233 (0.1)

manipulation	3	2214(0.04572996455927746)	1121 (0.1)	2548 (0.033)
marina	3	2693(0.0342974734194581)	1516 (0.069)	2548 (0.033)
mastication	3	896(0.14862238481765175)	1121 (0.1)	694 (0.2)
materials	3	2693(0.0342974734194581)	2359 (0.034)	#SAKNAS!
mathematical	3	2693(0.0342974734194581)	2359 (0.034)	2548 (0.033)
maturation	3	2693(0.0342974734194581)	2359 (0.034)	1668 (0.066)
maximus	3	2693(0.0342974734194581)	2359 (0.034)	#SAKNAS!
media	3	2693(0.0342974734194581)	#SAKNAS!	2548 (0.033)
mediator	3	2693(0.0342974734194581)	#SAKNAS!	#SAKNAS!
medium	3	2214(0.04572996455927746)	1121 (0.1)	#SAKNAS!
medium-term	3	2693(0.0342974734194581)	#SAKNAS!	2548 (0.033)
meeting	3	2693(0.0342974734194581)	1121 (0.1)	#SAKNAS!
meeting	3	2693(0.0342974734194581)	1121 (0.1)	#SAKNAS!
melanin-concentrating	3	2214(0.04572996455927746)	1121 (0.1)	2548 (0.033)
melanocortin	3	2214(0.04572996455927746)	1121 (0.1)	#SAKNAS!
melanogaster	3	1027(0.12575740253801304)	1121 (0.1)	999 (0.13)
membrane	3	2693(0.0342974734194581)	#SAKNAS!	2548 (0.033)
menopause	3	2693(0.0342974734194581)	#SAKNAS!	1668 (0.066)
menstrual	3	2693(0.0342974734194581)	2359 (0.034)	1668 (0.066)
mentally	3	2693(0.0342974734194581)	1121 (0.1)	#SAKNAS!
mentally	3	2693(0.0342974734194581)	1121 (0.1)	#SAKNAS!
menu	3	2693(0.0342974734194581)	#SAKNAS!	1233 (0.1)
mesh	3	2693(0.0342974734194581)	1516 (0.069)	#SAKNAS!
microalgae	3	2693(0.0342974734194581)	2359 (0.034)	1668 (0.066)
microvascular	3	2693(0.0342974734194581)	1516 (0.069)	2548 (0.033)
middle	3	1335(0.09145992911855493)	1121 (0.1)	1668 (0.066)
migrant	3	2693(0.0342974734194581)	#SAKNAS!	1668 (0.066)
miniature	3	2693(0.0342974734194581)	1121 (0.1)	#SAKNAS!
miniature	3	2693(0.0342974734194581)	1121 (0.1)	#SAKNAS!
minimal	3	2693(0.0342974734194581)	2359 (0.034)	#SAKNAS!
miocene	3	2693(0.0342974734194581)	1516 (0.069)	2548 (0.033)
misclassification	3	2693(0.0342974734194581)	#SAKNAS!	1668 (0.066)
mixing	3	1894(0.05716245569909683)	1121 (0.1)	#SAKNAS!
mo	3	2693(0.0342974734194581)	2359 (0.034)	2548 (0.033)
mobility	3	2693(0.0342974734194581)	#SAKNAS!	2548 (0.033)
modality	3	2693(0.0342974734194581)	2359 (0.034)	1668 (0.066)
modification	3	896(0.14862238481765175)	1121 (0.1)	816 (0.16)
modify	3	2693(0.0342974734194581)	2359 (0.034)	2548 (0.033)
modifying	3	2693(0.0342974734194581)	2359 (0.034)	1668 (0.066)
modulate	3	1111(0.11432491139819366)	1121 (0.1)	999 (0.13)
molars	3	1678(0.0685949468389162)	1121 (0.1)	1668 (0.066)
monkey	3	2693(0.0342974734194581)	#SAKNAS!	1233 (0.1)
monkeys	3	896(0.14862238481765175)	1121 (0.1)	526 (0.26)

month	3	2693(0.0342974734194581)	#SAKNAS!	2548 (0.033)
monuste	3	2693(0.0342974734194581)	1121 (0.1)	#SAKNAS!
monuste	3	2693(0.0342974734194581)	1121 (0.1)	#SAKNAS!
moose	3	2693(0.0342974734194581)	1121 (0.1)	#SAKNAS!
moose	3	2693(0.0342974734194581)	1121 (0.1)	#SAKNAS!
mori	3	2693(0.0342974734194581)	2359 (0.034)	2548 (0.033)
morphological	3	2693(0.0342974734194581)	2359 (0.034)	1668 (0.066)
morphology	3	1111(0.11432491139819366)	1121 (0.1)	999 (0.13)
mosquitoes	3	2693(0.0342974734194581)	#SAKNAS!	1668 (0.066)
mother	3	2693(0.0342974734194581)	#SAKNAS!	2548 (0.033)
mouths	3	2693(0.0342974734194581)	2359 (0.034)	1668 (0.066)
multi-centre	3	2693(0.0342974734194581)	2359 (0.034)	1668 (0.066)
multicomponent	3	2693(0.0342974734194581)	#SAKNAS!	2548 (0.033)
multidimensional	3	2214(0.04572996455927746)	1121 (0.1)	#SAKNAS!
multi-professional	3	2693(0.0342974734194581)	#SAKNAS!	1668 (0.066)
multivariate	3	1219(0.1028924202583743)	1121 (0.1)	816 (0.16)
muscle	3	256(0.5030296101520522)	1121 (0.1)	264 (0.53)
mussel	3	1485(0.08002743797873557)	1121 (0.1)	1233 (0.1)
mutation	3	2693(0.0342974734194581)	2359 (0.034)	2548 (0.033)
mykiss	3	2693(0.0342974734194581)	1121 (0.1)	#SAKNAS!
mykiss	3	2693(0.0342974734194581)	1121 (0.1)	#SAKNAS!
myocardial	3	966(0.1371898936778324)	1121 (0.1)	584 (0.23)
myth	3	2693(0.0342974734194581)	2359 (0.034)	1668 (0.066)
myzus	3	2693(0.0342974734194581)	#SAKNAS!	1668 (0.066)
nadya-senpe	3	2693(0.0342974734194581)	#SAKNAS!	1668 (0.066)
narrative	3	2693(0.0342974734194581)	2359 (0.034)	#SAKNAS!
nation	3	2693(0.0342974734194581)	2359 (0.034)	1668 (0.066)
nationwide	3	281(0.468732136732594)	1121 (0.1)	280 (0.49)
native	3	2693(0.0342974734194581)	#SAKNAS!	1668 (0.066)
nature	3	1894(0.05716245569909683)	1121 (0.1)	#SAKNAS!
necessary	3	2693(0.0342974734194581)	2359 (0.034)	1668 (0.066)
necrotising	3	2693(0.0342974734194581)	2359 (0.034)	2548 (0.033)
neotropical	3	2693(0.0342974734194581)	#SAKNAS!	1668 (0.066)
neurodevelopmental	3	2693(0.0342974734194581)	2359 (0.034)	#SAKNAS!
neuroendocrine	3	2693(0.0342974734194581)	1516 (0.069)	#SAKNAS!
neurologically	3	1335(0.09145992911855493)	1121 (0.1)	1233 (0.1)
neurology	3	2693(0.0342974734194581)	#SAKNAS!	2548 (0.033)
neurone	3	1894(0.05716245569909683)	1121 (0.1)	1668 (0.066)
neurotrophic	3	2214(0.04572996455927746)	1121 (0.1)	#SAKNAS!
neutral	3	2693(0.0342974734194581)	#SAKNAS!	2548 (0.033)
nicu	3	2693(0.0342974734194581)	#SAKNAS!	2548 (0.033)
noctuidae	3	2693(0.0342974734194581)	2359 (0.034)	1668 (0.066)
noma	3	2693(0.0342974734194581)	#SAKNAS!	2548 (0.033)

nonalcoholic	3	2693(0.0342974734194581)	#SAKNAS!	2548 (0.033)
non-diabetic	3	2693(0.0342974734194581)	#SAKNAS!	1668 (0.066)
nondysphagic	3	2693(0.0342974734194581)	2359 (0.034)	#SAKNAS!
non-invasive	3	2693(0.0342974734194581)	2359 (0.034)	2548 (0.033)
nonnutritive	3	2693(0.0342974734194581)	1516 (0.069)	#SAKNAS!
non-smokers	3	2693(0.0342974734194581)	#SAKNAS!	2548 (0.033)
nordic	3	2693(0.0342974734194581)	#SAKNAS!	1668 (0.066)
normative	3	1894(0.0571624556990968З)	1121 (0.1)	2548 (0.033)
northeastern	3	2693(0.0342974734194581)	#SAKNAS!	1668 (0.066)
norwegian	3	2693(0.0342974734194581)	#SAKNAS!	1668 (0.066)
nucleatum	3	2693(0.0342974734194581)	1516 (0.069)	2548 (0.033)
nurse	3	2693(0.0342974734194581)	#SAKNAS!	1668 (0.066)
nurses	3	1678(0.0685949468389162)	1121 (0.1)	1668 (0.066)
nut	3	2693(0.0342974734194581)	2359 (0.034)	#SAKNAS!
objective	3	1219(0.1028924202583743)	1121 (0.1)	816 (0.16)
observations	3	2693(0.0342974734194581)	2359 (0.034)	1668 (0.066)
occlusal	3	500(0.2743797873556648)	1121 (0.1)	422 (0.33)
occurring	3	2693(0.0342974734194581)	2359 (0.034)	1668 (0.066)
octopus	3	2693(0.0342974734194581)	#SAKNAS!	#SAKNAS!
oculopharyngeal	3	2693(0.0342974734194581)	2359 (0.034)	#SAKNAS!
odontological	3	2693(0.0342974734194581)	2359 (0.034)	#SAKNAS!
oesophagitis	3	2693(0.0342974734194581)	#SAKNAS!	1668 (0.066)
office	3	1894(0.05716245569909683)	1121 (0.1)	1668 (0.066)
oldest	3	1894(0.05716245569909683)	1121 (0.1)	2548 (0.033)
olfactory	3	966(0.1371898936778324)	1121 (0.1)	584 (0.23)
oncorhynchus	3	2693(0.0342974734194581)	1121 (0.1)	#SAKNAS!
oncorhynchus	3	2693(0.0342974734194581)	1121 (0.1)	#SAKNAS!
one-stage	3	2693(0.0342974734194581)	1516 (0.069)	#SAKNAS!
one-year	3	1335(0.09145992911855493)	1121 (0.1)	1233 (0.1)
ontogenetic	3	2693(0.0342974734194581)	2359 (0.034)	2548 (0.033)
ontogeny	3	2693(0.0342974734194581)	1516 (0.069)	#SAKNAS!
opinion	3	2693(0.0342974734194581)	#SAKNAS!	2548 (0.033)
opinions	3	2693(0.0342974734194581)	2359 (0.034)	2548 (0.033)
opioid	3	2693(0.0342974734194581)	#SAKNAS!	2548 (0.033)
oral-health	3	2693(0.0342974734194581)	#SAKNAS!	#SAKNAS!
orally	3	2693(0.0342974734194581)	2359 (0.034)	2548 (0.033)
orange	3	2693(0.0342974734194581)	2359 (0.034)	2548 (0.033)
orca	3	2693(0.0342974734194581)	#SAKNAS!	1668 (0.066)
orchestoidea	3	2693(0.0342974734194581)	1516 (0.069)	2548 (0.033)
original	3	2693(0.0342974734194581)	#SAKNAS!	#SAKNAS!
origins	3	2693(0.0342974734194581)	1516 (0.069)	2548 (0.033)
orofacial	3	1027(0.12575740253801304)	1121 (0.1)	999 (0.13)
oscillation	3	2693(0.0342974734194581)	2359 (0.034)	2548 (0.033)

osteonecrosis	3	2693(0.0342974734194581)	2359 (0.034)	2548 (0.033)
osteophyte	3	2693(0.0342974734194581)	#SAKNAS!	1668 (0.066)
outbreak	3	2693(0.0342974734194581)	#SAKNAS!	#SAKNAS!
outlet	3	1678(0.0685949468389162)	1121 (0.1)	1668 (0.066)
ovary	3	1335(0.09145992911855493)	1121 (0.1)	#SAKNAS!
overlap	3	2693(0.0342974734194581)	2359 (0.034)	#SAKNAS!
oviposition	3	2214(0.04572996455927746)	1121 (0.1)	2548 (0.033)
pain	3	516(0.26294729621584545)	1121 (0.1)	333 (0.43)
pan	3	2693(0.0342974734194581)	#SAKNAS!	1233 (0.1)
pancreatic	3	2214(0.04572996455927746)	1121 (0.1)	2548 (0.033)
pandas	3	2693(0.0342974734194581)	2359 (0.034)	1668 (0.066)
panel	3	1485(0.08002743797873557)	1121 (0.1)	2548 (0.033)
paradox	3	2693(0.0342974734194581)	2359 (0.034)	2548 (0.033)
parasitism	3	2214(0.04572996455927746)	1121 (0.1)	#SAKNAS!
parasitoid	3	1894(0.05716245569909683)	1121 (0.1)	1668 (0.066)
paraventricular	3	2693(0.0342974734194581)	1121 (0.1)	#SAKNAS!
paraventricular	3	2693(0.0342974734194581)	1121 (0.1)	#SAKNAS!
part	3	1111(0.11432491139819366)	1121 (0.1)	816 (0.16)
participation	3	1678(0.0685949468389162)	1121 (0.1)	#SAKNAS!
particle	3	1219(0.1028924202583743)	1121 (0.1)	1233 (0.1)
partitioning	3	2693(0.0342974734194581)	1516 (0.069)	2548 (0.033)
partnership	3	2693(0.0342974734194581)	#SAKNAS!	2548 (0.033)
parts	3	1894(0.05716245569909683)	1121 (0.1)	2548 (0.033)
passerine	3	2693(0.0342974734194581)	1121 (0.1)	#SAKNAS!
passerine	3	2693(0.0342974734194581)	1121 (0.1)	#SAKNAS!
passive	3	2693(0.0342974734194581)	1121 (0.1)	#SAKNAS!
passive	3	2693(0.0342974734194581)	1121 (0.1)	#SAKNAS!
patagonia	3	2693(0.0342974734194581)	2359 (0.034)	2548 (0.033)
patch	3	2693(0.0342974734194581)	2359 (0.034)	1668 (0.066)
paternal	3	2693(0.0342974734194581)	#SAKNAS!	2548 (0.033)
path	3	2693(0.0342974734194581)	#SAKNAS!	1668 (0.066)
pathological	3	2693(0.0342974734194581)	#SAKNAS!	2548 (0.033)
pathway	3	743(0.18291985823710985)	1121 (0.1)	461 (0.3)
pcr	3	2693(0.0342974734194581)	#SAKNAS!	1233 (0.1)
peak	3	2693(0.0342974734194581)	#SAKNAS!	1668 (0.066)
pecking	3	2693(0.0342974734194581)	2359 (0.034)	1668 (0.066)
pelleted	3	2214(0.04572996455927746)	1121 (0.1)	2548 (0.033)
pelotas	3	2693(0.0342974734194581)	2359 (0.034)	2548 (0.033)
penaeus	3	2214(0.04572996455927746)	1121 (0.1)	#SAKNAS!
peptides	3	2693(0.0342974734194581)	1516 (0.069)	#SAKNAS!
percentage	3	2693(0.0342974734194581)	#SAKNAS!	1233 (0.1)
perch	3	2693(0.0342974734194581)	1516 (0.069)	#SAKNAS!
peri-implant	3	2693(0.0342974734194581)	#SAKNAS!	2548 (0.033)

perinatal	3	1485(0.08002743797873557)	1121 (0.1)	999 (0.13)
periodontal-disease	3	2693(0.0342974734194581)	1121 (0.1)	#SAKNAS!
periodontal-disease	3	2693(0.0342974734194581)	1121 (0.1)	#SAKNAS!
periodontally	3	2693(0.0342974734194581)	#SAKNAS!	#SAKNAS!
perioperative	3	836(0.16005487595747114)	1121 (0.1)	1668 (0.066)
peristalsis	3	2693(0.0342974734194581)	#SAKNAS!	1233 (0.1)
persicae	3	2693(0.0342974734194581)	#SAKNAS!	1668 (0.066)
person	3	2693(0.0342974734194581)	2359 (0.034)	2548 (0.033)
personal	3	1894(0.05716245569909683)	1121 (0.1)	#SAKNAS!
ph	3	966(0.1371898936778324)	1121 (0.1)	816 (0.16)
phenotype	3	1485(0.08002743797873557)	1121 (0.1)	2548 (0.033)
phenylketonuria	3	2693(0.0342974734194581)	1516 (0.069)	#SAKNAS!
philippines	3	2693(0.0342974734194581)	1516 (0.069)	#SAKNAS!
phosphorus	3	2693(0.0342974734194581)	#SAKNAS!	1668 (0.066)
physically	3	2693(0.0342974734194581)	2359 (0.034)	#SAKNAS!
physicochemical	3	2693(0.0342974734194581)	#SAKNAS!	#SAKNAS!
physico-chemical	3	2693(0.0342974734194581)	#SAKNAS!	#SAKNAS!
phytoplankton	3	2693(0.0342974734194581)	1516 (0.069)	#SAKNAS!
pieridae	3	2693(0.0342974734194581)	1121 (0.1)	#SAKNAS!
pieridae	3	2693(0.0342974734194581)	1121 (0.1)	#SAKNAS!
pituitary	3	2693(0.0342974734194581)	#SAKNAS!	1668 (0.066)
placental	3	2693(0.0342974734194581)	2359 (0.034)	#SAKNAS!
plants	3	896(0.14862238481765175)	1121 (0.1)	526 (0.26)
planus	3	2693(0.0342974734194581)	2359 (0.034)	2548 (0.033)
plate	3	2693(0.0342974734194581)	2359 (0.034)	2548 (0.033)
play	3	2693(0.0342974734194581)	2359 (0.034)	2548 (0.033)
plication	3	2693(0.0342974734194581)	1516 (0.069)	2548 (0.033)
plummer-vinson	3	2693(0.0342974734194581)	2359 (0.034)	2548 (0.033)
point-of-care	3	2693(0.0342974734194581)	#SAKNAS!	1668 (0.066)
policy	3	1894(0.05716245569909683)	1121 (0.1)	2548 (0.033)
pollen	3	1485(0.08002743797873557)	1121 (0.1)	1668 (0.066)
polycystic	3	1335(0.09145992911855493)	1121 (0.1)	#SAKNAS!
polyphagous	3	2693(0.0342974734194581)	1516 (0.069)	2548 (0.033)
positive	3	743(0.18291985823710985)	1121 (0.1)	694 (0.2)
post-extubation	3	2693(0.0342974734194581)	#SAKNAS!	2548 (0.033)
post-ingestive	3	2693(0.0342974734194581)	1516 (0.069)	#SAKNAS!
postprandial	3	2693(0.0342974734194581)	2359 (0.034)	#SAKNAS!
poultry	3	2693(0.0342974734194581)	2359 (0.034)	#SAKNAS!
power	3	2693(0.0342974734194581)	2359 (0.034)	#SAKNAS!
pre-diabetes	3	2693(0.0342974734194581)	#SAKNAS!	1668 (0.066)
pre-eclampsia	3	2214(0.04572996455927746)	1121 (0.1)	2548 (0.033)
prefrontal	3	2693(0.0342974734194581)	#SAKNAS!	2548 (0.033)
prehistoric	3	2693(0.0342974734194581)	1516 (0.069)	2548 (0.033)

prenatal	3	1219(0.1028924202583743)	1121 (0.1)	1233 (0.1)
prescription	3	2693(0.0342974734194581)	#SAKNAS!	2548 (0.033)
presentations	3	2693(0.0342974734194581)	#SAKNAS!	1668 (0.066)
presenting	3	1219(0.1028924202583743)	1121 (0.1)	1233 (0.1)
prior	3	836(0.16005487595747114)	1121 (0.1)	816 (0.16)
probing	3	2693(0.0342974734194581)	#SAKNAS!	1233 (0.1)
proceedings	3	2693(0.0342974734194581)	2359 (0.034)	1668 (0.066)
processes	3	1485(0.08002743797873557)	1121 (0.1)	1668 (0.066)
productive	3	2693(0.0342974734194581)	2359 (0.034)	1668 (0.066)
progenitor	3	2693(0.0342974734194581)	2359 (0.034)	2548 (0.033)
proinflammatory	3	2693(0.0342974734194581)	2359 (0.034)	2548 (0.033)
promote	3	966(0.1371898936778324)	1121 (0.1)	816 (0.16)
promoter	3	2214(0.04572996455927746)	1121 (0.1)	#SAKNAS!
proper	3	2693(0.0342974734194581)	2359 (0.034)	2548 (0.033)
properties	3	342(0.40013718989367786)	1121 (0.1)	264 (0.53)
prophylaxis	3	1485(0.08002743797873557)	1121 (0.1)	2548 (0.033)
propithecus	3	2693(0.0342974734194581)	2359 (0.034)	1668 (0.066)
prosthesis	3	896(0.14862238481765175)	1121 (0.1)	999 (0.13)
prosthodontic	3	2693(0.0342974734194581)	#SAKNAS!	1233 (0.1)
protection	3	2693(0.0342974734194581)	2359 (0.034)	1668 (0.066)
protein-energy	3	1894(0.05716245569909683)	1121 (0.1)	1668 (0.066)
proteomic	3	2693(0.0342974734194581)	#SAKNAS!	2548 (0.033)
provides	3	2693(0.0342974734194581)	1516 (0.069)	2548 (0.033)
provinces	3	2693(0.0342974734194581)	2359 (0.034)	#SAKNAS!
psychometric	3	2693(0.0342974734194581)	#SAKNAS!	1668 (0.066)
psyllid	3	2693(0.0342974734194581)	#SAKNAS!	2548 (0.033)
puree	3	2693(0.0342974734194581)	#SAKNAS!	#SAKNAS!
pylori	3	1111(0.11432491139819366)	1121 (0.1)	999 (0.13)
qatar	3	2693(0.0342974734194581)	#SAKNAS!	2548 (0.033)
quality-of-life	3	2693(0.0342974734194581)	1516 (0.069)	#SAKNAS!
quantifying	3	2693(0.0342974734194581)	1516 (0.069)	#SAKNAS!
quarantine	3	2693(0.0342974734194581)	#SAKNAS!	#SAKNAS!
r	3	2693(0.0342974734194581)	2359 (0.034)	2548 (0.033)
radiological	3	1335(0.09145992911855493)	1121 (0.1)	1233 (0.1)
rainbow	3	2693(0.0342974734194581)	1121 (0.1)	#SAKNAS!
rainbow	3	2693(0.0342974734194581)	1121 (0.1)	#SAKNAS!
ratio	3	966(0.1371898936778324)	1121 (0.1)	999 (0.13)
readiness	3	2693(0.0342974734194581)	2359 (0.034)	#SAKNAS!
real	3	2693(0.0342974734194581)	#SAKNAS!	#SAKNAS!
reality	3	2693(0.0342974734194581)	2359 (0.034)	1668 (0.066)
really	3	2693(0.0342974734194581)	1516 (0.069)	2548 (0.033)
reasons	3	2693(0.0342974734194581)	2359 (0.034)	2548 (0.033)
recessive	3	2693(0.0342974734194581)	1121 (0.1)	#SAKNAS!

recessive	3	2693(0.0342974734194581)	1121 (0.1)	#SAKNAS!
recipes	3	2693(0.0342974734194581)	2359 (0.034)	2548 (0.033)
recipients	3	1111(0.11432491139819366)	1121 (0.1)	816 (0.16)
recombinant	3	2693(0.0342974734194581)	1121 (0.1)	#SAKNAS!
recombinant	3	2693(0.0342974734194581)	1121 (0.1)	#SAKNAS!
record	3	2693(0.0342974734194581)	1121 (0.1)	#SAKNAS!
record	3	2693(0.0342974734194581)	1121 (0.1)	#SAKNAS!
recordings	3	2693(0.0342974734194581)	#SAKNAS!	2548 (0.033)
redo	3	2693(0.0342974734194581)	#SAKNAS!	1668 (0.066)
reduction	3	896(0.14862238481765175)	1121 (0.1)	694 (0.2)
referral	3	2693(0.0342974734194581)	2359 (0.034)	2548 (0.033)
reflections	3	2693(0.0342974734194581)	2359 (0.034)	2548 (0.033)
regression	3	1335(0.09145992911855493)	1121 (0.1)	999 (0.13)
regulating	3	1678(0.0685949468389162)	1121 (0.1)	1668 (0.066)
relate	3	2214(0.04572996455927746)	1121 (0.1)	#SAKNAS!
relates	3	1894(0.05716245569909683)	1121 (0.1)	#SAKNAS!
replace	3	2693(0.0342974734194581)	#SAKNAS!	2548 (0.033)
representations	3	2693(0.0342974734194581)	1516 (0.069)	2548 (0.033)
requirement	3	2693(0.0342974734194581)	2359 (0.034)	2548 (0.033)
restorative	3	2693(0.0342974734194581)	1516 (0.069)	2548 (0.033)
restricting	3	2693(0.0342974734194581)	#SAKNAS!	1668 (0.066)
result	3	2693(0.0342974734194581)	#SAKNAS!	2548 (0.033)
rethinking	3	2214(0.04572996455927746)	1121 (0.1)	#SAKNAS!
revisiting	3	1894(0.05716245569909683)	1121 (0.1)	1668 (0.066)
rhinitis	3	2693(0.0342974734194581)	#SAKNAS!	1668 (0.066)
rickets	3	2693(0.0342974734194581)	2359 (0.034)	1668 (0.066)
right	3	1485(0.08002743797873557)	1121 (0.1)	1668 (0.066)
ring-tailed	3	2693(0.0342974734194581)	1121 (0.1)	#SAKNAS!
ring-tailed	3	2693(0.0342974734194581)	1121 (0.1)	#SAKNAS!
river	3	1219(0.1028924202583743)	1121 (0.1)	1233 (0.1)
rock	3	2693(0.0342974734194581)	2359 (0.034)	2548 (0.033)
roma	3	2693(0.0342974734194581)	2359 (0.034)	2548 (0.033)
root	3	836(0.16005487595747114)	1121 (0.1)	584 (0.23)
roughage	3	2693(0.0342974734194581)	2359 (0.034)	1668 (0.066)
route	3	2214(0.04572996455927746)	1121 (0.1)	2548 (0.033)
rumen	3	1219(0.1028924202583743)	1121 (0.1)	1233 (0.1)
salmon	3	1678(0.0685949468389162)	1121 (0.1)	1233 (0.1)
salmonella	3	2693(0.0342974734194581)	2359 (0.034)	#SAKNAS!
samples	3	2693(0.0342974734194581)	#SAKNAS!	2548 (0.033)
sampling	3	2693(0.0342974734194581)	2359 (0.034)	2548 (0.033)
scale	3	595(0.22864982279638732)	1121 (0.1)	694 (0.2)
schistocerca	3	2214(0.04572996455927746)	1121 (0.1)	2548 (0.033)
schizophrenic	3	2693(0.0342974734194581)	#SAKNAS!	2548 (0.033)

scleroderma	3	2693(0.0342974734194581)	2359 (0.034)	2548 (0.033)
screenings	3	2693(0.0342974734194581)	#SAKNAS!	1668 (0.066)
sealant	3	2693(0.0342974734194581)	2359 (0.034)	2548 (0.033)
season	3	1485(0.08002743797873557)	1121 (0.1)	1233 (0.1)
seasons	3	2693(0.0342974734194581)	#SAKNAS!	2548 (0.033)
second	3	2693(0.0342974734194581)	#SAKNAS!	2548 (0.033)
sectional	3	1111(0.11432491139819366)	1121 (0.1)	1668 (0.066)
sediment	3	2693(0.0342974734194581)	1516 (0.069)	#SAKNAS!
seen	3	2693(0.0342974734194581)	1516 (0.069)	#SAKNAS!
segment	3	2693(0.0342974734194581)	2359 (0.034)	2548 (0.033)
segregation	3	2693(0.0342974734194581)	1516 (0.069)	#SAKNAS!
selectively	3	2693(0.0342974734194581)	1121 (0.1)	#SAKNAS!
selectively	3	2693(0.0342974734194581)	1121 (0.1)	#SAKNAS!
self-efficacy	3	1111(0.11432491139819366)	1121 (0.1)	999 (0.13)
self-feeding	3	2693(0.0342974734194581)	2359 (0.034)	1668 (0.066)
self-medication	3	2693(0.0342974734194581)	1516 (0.069)	2548 (0.033)
self-perception	3	2693(0.0342974734194581)	#SAKNAS!	2548 (0.033)
semi-intensive	3	2693(0.0342974734194581)	1516 (0.069)	#SAKNAS!
seniors	3	2693(0.0342974734194581)	#SAKNAS!	2548 (0.033)
separation	3	2693(0.0342974734194581)	1516 (0.069)	2548 (0.033)
sequelae	3	2693(0.0342974734194581)	2359 (0.034)	1668 (0.066)
sequencing	3	2693(0.0342974734194581)	#SAKNAS!	1668 (0.066)
sequential	3	2693(0.0342974734194581)	1516 (0.069)	2548 (0.033)
set	3	2693(0.0342974734194581)	2359 (0.034)	#SAKNAS!
seventh	3	2693(0.0342974734194581)	#SAKNAS!	#SAKNAS!
seven-year	3	2693(0.0342974734194581)	2359 (0.034)	2548 (0.033)
sexual	3	1335(0.09145992911855493)	1121 (0.1)	999 (0.13)
shelter	3	2693(0.0342974734194581)	1516 (0.069)	#SAKNAS!
short	3	710(0.19435234937692925)	1121 (0.1)	816 (0.16)
shortage	3	2693(0.0342974734194581)	2359 (0.034)	1668 (0.066)
sialorrhea	3	2693(0.0342974734194581)	2359 (0.034)	2548 (0.033)
side	3	2693(0.0342974734194581)	2359 (0.034)	1668 (0.066)
signalling	3	2693(0.0342974734194581)	1121 (0.1)	#SAKNAS!
signalling	3	2693(0.0342974734194581)	1121 (0.1)	#SAKNAS!
silent	3	2693(0.0342974734194581)	#SAKNAS!	2548 (0.033)
silkworm	3	2693(0.0342974734194581)	1516 (0.069)	#SAKNAS!
single-center	3	628(0.21721733165656798)	1121 (0.1)	816 (0.16)
single-centre	3	2693(0.0342974734194581)	#SAKNAS!	1668 (0.066)
site	3	1027(0.12575740253801304)	1121 (0.1)	999 (0.13)
sleeve	3	396(0.342974734194581)	1121 (0.1)	280 (0.49)
slug	3	2693(0.0342974734194581)	1121 (0.1)	#SAKNAS!
slug	3	2693(0.0342974734194581)	1121 (0.1)	#SAKNAS!
smoke	3	1219(0.1028924202583743)	1121 (0.1)	1233 (0.1)

snacking	3	2693(0.0342974734194581)	1121 (0.1)	#SAKNAS!
snacking	3	2693(0.0342974734194581)	1121 (0.1)	#SAKNAS!
socially	3	2693(0.0342974734194581)	1516 (0.069)	#SAKNAS!
soil	3	2693(0.0342974734194581)	2359 (0.034)	1668 (0.066)
solids	3	2693(0.0342974734194581)	#SAKNAS!	2548 (0.033)
solution	3	2693(0.0342974734194581)	2359 (0.034)	1668 (0.066)
sorghum	3	2693(0.0342974734194581)	#SAKNAS!	1233 (0.1)
source	3	1678(0.0685949468389162)	1121 (0.1)	1668 (0.066)
southeastern	3	2693(0.0342974734194581)	2359 (0.034)	1668 (0.066)
soy	3	2693(0.0342974734194581)	2359 (0.034)	2548 (0.033)
sp	3	2693(0.0342974734194581)	#SAKNAS!	1233 (0.1)
space	3	2693(0.0342974734194581)	#SAKNAS!	#SAKNAS!
sparus	3	2693(0.0342974734194581)	1121 (0.1)	#SAKNAS!
sparus	3	2693(0.0342974734194581)	1121 (0.1)	#SAKNAS!
specialist	3	2693(0.0342974734194581)	1121 (0.1)	#SAKNAS!
specialist	3	2693(0.0342974734194581)	1121 (0.1)	#SAKNAS!
specimens	3	2693(0.0342974734194581)	#SAKNAS!	1233 (0.1)
speech	3	896(0.14862238481765175)	1121 (0.1)	1233 (0.1)
spodoptera	3	2693(0.0342974734194581)	#SAKNAS!	1668 (0.066)
sports	3	2693(0.0342974734194581)	#SAKNAS!	1233 (0.1)
spring	3	2693(0.0342974734194581)	#SAKNAS!	1668 (0.066)
squamous	3	559(0.2400823139362067)	1121 (0.1)	584 (0.23)
squirrel	3	2693(0.0342974734194581)	2359 (0.034)	2548 (0.033)
stages	3	743(0.18291985823710985)	1121 (0.1)	584 (0.23)
standards	3	2693(0.0342974734194581)	1516 (0.069)	2548 (0.033)
starch	3	1027(0.12575740253801304)	1121 (0.1)	694 (0.2)
starvation	3	1678(0.0685949468389162)	1121 (0.1)	1668 (0.066)
stem	3	1027(0.12575740253801304)	1121 (0.1)	816 (0.16)
stepwise	3	2693(0.0342974734194581)	2359 (0.034)	#SAKNAS!
still	3	2693(0.0342974734194581)	2359 (0.034)	#SAKNAS!
stimulating	3	2693(0.0342974734194581)	2359 (0.034)	#SAKNAS!
stimuli	3	2693(0.0342974734194581)	2359 (0.034)	1668 (0.066)
stone	3	2693(0.0342974734194581)	#SAKNAS!	1233 (0.1)
strain	3	2693(0.0342974734194581)	2359 (0.034)	#SAKNAS!
stratification	3	2693(0.0342974734194581)	#SAKNAS!	1668 (0.066)
stream	3	2693(0.0342974734194581)	2359 (0.034)	2548 (0.033)
streams	3	2693(0.0342974734194581)	1516 (0.069)	#SAKNAS!
streptococcus	3	1027(0.12575740253801304)	1121 (0.1)	1233 (0.1)
structural	3	710(0.19435234937692925)	1121 (0.1)	584 (0.23)
student	3	2693(0.0342974734194581)	1516 (0.069)	2548 (0.033)
studying	3	1894(0.05716245569909683)	1121 (0.1)	#SAKNAS!
styles	3	1678(0.0685949468389162)	1121 (0.1)	2548 (0.033)
subgingival	3	535(0.2515148050760261)	1121 (0.1)	816 (0.16)

submandibular	3	2214(0.04572996455927746)	1121 (0.1)	#SAKNAS!
subpopulation	3	2693(0.0342974734194581)	#SAKNAS!	1668 (0.066)
substitute	3	1894(0.05716245569909683)	1121 (0.1)	#SAKNAS!
substituting	3	2693(0.0342974734194581)	2359 (0.034)	2548 (0.033)
substrate	3	2693(0.0342974734194581)	1516 (0.069)	2548 (0.033)
subtype	3	2693(0.0342974734194581)	1516 (0.069)	2548 (0.033)
suffering	3	2693(0.0342974734194581)	1516 (0.069)	2548 (0.033)
suitable	3	2693(0.0342974734194581)	#SAKNAS!	2548 (0.033)
summary	3	2693(0.0342974734194581)	1516 (0.069)	#SAKNAS!
supracricoid	3	2693(0.0342974734194581)	1516 (0.069)	2548 (0.033)
supragingival	3	2693(0.0342974734194581)	#SAKNAS!	2548 (0.033)
suprahyoid	3	2693(0.0342974734194581)	#SAKNAS!	2548 (0.033)
supranuclear	3	2693(0.0342974734194581)	#SAKNAS!	#SAKNAS!
surfaces	3	2693(0.0342974734194581)	2359 (0.034)	2548 (0.033)
survive	3	2693(0.0342974734194581)	#SAKNAS!	2548 (0.033)
susceptibility	3	1335(0.09145992911855493)	1121 (0.1)	1668 (0.066)
suspension	3	1678(0.0685949468389162)	1121 (0.1)	#SAKNAS!
sustainability	3	2693(0.0342974734194581)	#SAKNAS!	1233 (0.1)
swallowability	3	2693(0.0342974734194581)	#SAKNAS!	#SAKNAS!
swal-qol	3	2693(0.0342974734194581)	1516 (0.069)	#SAKNAS!
sward	3	2693(0.0342974734194581)	1516 (0.069)	#SAKNAS!
sweeteners	3	1894(0.05716245569909683)	1121 (0.1)	2548 (0.033)
swelling	3	2693(0.0342974734194581)	2359 (0.034)	2548 (0.033)
switching	3	2693(0.0342974734194581)	2359 (0.034)	#SAKNAS!
sydney	3	2693(0.0342974734194581)	2359 (0.034)	#SAKNAS!
synergistic	3	2693(0.0342974734194581)	#SAKNAS!	2548 (0.033)
syrian	3	2693(0.0342974734194581)	#SAKNAS!	2548 (0.033)
tabaci	3	2693(0.0342974734194581)	2359 (0.034)	1668 (0.066)
takashimadaira	3	2693(0.0342974734194581)	#SAKNAS!	1668 (0.066)
tamarins	3	2693(0.0342974734194581)	2359 (0.034)	#SAKNAS!
tannerella	3	2693(0.0342974734194581)	1516 (0.069)	#SAKNAS!
tannins	3	2693(0.0342974734194581)	1121 (0.1)	#SAKNAS!
tannins	3	2693(0.0342974734194581)	1121 (0.1)	#SAKNAS!
tap	3	2693(0.0342974734194581)	#SAKNAS!	1233 (0.1)
task	3	2693(0.0342974734194581)	1121 (0.1)	#SAKNAS!
task	3	2693(0.0342974734194581)	1121 (0.1)	#SAKNAS!
technical	3	2214(0.04572996455927746)	1121 (0.1)	2548 (0.033)
techniques	3	1485(0.08002743797873557)	1121 (0.1)	1233 (0.1)
temperament	3	2693(0.0342974734194581)	#SAKNAS!	1668 (0.066)
temperate	3	2214(0.04572996455927746)	1121 (0.1)	2548 (0.033)
temperature	3	896(0.14862238481765175)	1121 (0.1)	694 (0.2)
temperatures	3	2693(0.0342974734194581)	#SAKNAS!	1233 (0.1)
ten	3	2693(0.0342974734194581)	2359 (0.034)	#SAKNAS!

terminal	3	2693(0.0342974734194581)	2359 (0.034)	2548 (0.033)
terminology	3	2693(0.0342974734194581)	#SAKNAS!	2548 (0.033)
terrestrial	3	2693(0.0342974734194581)	2359 (0.034)	1668 (0.066)
testing	3	1335(0.09145992911855493)	1121 (0.1)	816 (0.16)
textural	3	2693(0.0342974734194581)	#SAKNAS!	#SAKNAS!
texture	3	439(0.30867726077512286)	1121 (0.1)	333 (0.43)
textures	3	2693(0.0342974734194581)	2359 (0.034)	1668 (0.066)
thailand	3	1335(0.09145992911855493)	1121 (0.1)	1233 (0.1)
them	3	2693(0.0342974734194581)	2359 (0.034)	#SAKNAS!
theoretical	3	2214(0.04572996455927746)	1121 (0.1)	#SAKNAS!
theory	3	1894(0.05716245569909683)	1121 (0.1)	2548 (0.033)
think	3	2693(0.0342974734194581)	#SAKNAS!	2548 (0.033)
thrive	3	2693(0.0342974734194581)	1121 (0.1)	#SAKNAS!
thrive	3	2693(0.0342974734194581)	1121 (0.1)	#SAKNAS!
thrombectomy	3	2693(0.0342974734194581)	#SAKNAS!	#SAKNAS!
thuringiensis	3	2693(0.0342974734194581)	1516 (0.069)	2548 (0.033)
thyroidectomy	3	2693(0.0342974734194581)	2359 (0.034)	#SAKNAS!
tnf-帢	3	2693(0.0342974734194581)	#SAKNAS!	2548 (0.033)
toddler	3	1894(0.05716245569909683)	1121 (0.1)	#SAKNAS!
toothbrushing	3	836(0.16005487595747114)	1121 (0.1)	584 (0.23)
tracheal	3	2693(0.0342974734194581)	2359 (0.034)	2548 (0.033)
traditional	3	836(0.16005487595747114)	1121 (0.1)	816 (0.16)
transcriptome	3	2693(0.0342974734194581)	#SAKNAS!	1668 (0.066)
transit	3	2693(0.0342974734194581)	#SAKNAS!	2548 (0.033)
transport	3	2693(0.0342974734194581)	2359 (0.034)	#SAKNAS!
transporter	3	1678(0.0685949468389162)	1121 (0.1)	2548 (0.033)
transtheoretical	3	2693(0.0342974734194581)	2359 (0.034)	2548 (0.033)
tribolium	3	2693(0.0342974734194581)	2359 (0.034)	1668 (0.066)
trichogramma	3	2693(0.0342974734194581)	2359 (0.034)	1668 (0.066)
triglycerides	3	2693(0.0342974734194581)	2359 (0.034)	#SAKNAS!
trimester	3	2693(0.0342974734194581)	#SAKNAS!	2548 (0.033)
troglodytes	3	2693(0.0342974734194581)	#SAKNAS!	1233 (0.1)
tsurugaya	3	2693(0.0342974734194581)	#SAKNAS!	1233 (0.1)
tuberculata	3	2693(0.0342974734194581)	1516 (0.069)	2548 (0.033)
tumors	3	1894(0.05716245569909683)	1121 (0.1)	#SAKNAS!
turnover	3	2693(0.0342974734194581)	1121 (0.1)	#SAKNAS!
turnover	3	2693(0.0342974734194581)	1121 (0.1)	#SAKNAS!
twin	3	2693(0.0342974734194581)	2359 (0.034)	2548 (0.033)
two-sample	3	2693(0.0342974734194581)	#SAKNAS!	#SAKNAS!
two-spotted	3	2693(0.0342974734194581)	#SAKNAS!	1233 (0.1)
uganda	3	1485(0.08002743797873557)	1121 (0.1)	1668 (0.066)
uk	3	559(0.2400823139362067)	1121 (0.1)	361 (0.4)
ulcerative	3	1894(0.05716245569909683)	1121 (0.1)	2548 (0.033)

underlying	3	1485(0.08002743797873557)	1121 (0.1)	1233 (0.1)
units	3	1335(0.09145992911855493)	1121 (0.1)	1668 (0.066)
usa	3	1027(0.12575740253801304)	1121 (0.1)	999 (0.13)
useful	3	1894(0.05716245569909683)	1121 (0.1)	1668 (0.066)
vagal	3	2693(0.0342974734194581)	1516 (0.069)	2548 (0.033)
vagotomy	3	2693(0.0342974734194581)	1121 (0.1)	#SAKNAS!
vagotomy	3	2693(0.0342974734194581)	1121 (0.1)	#SAKNAS!
validate	3	2693(0.0342974734194581)	2359 (0.034)	#SAKNAS!
vannamei	3	2693(0.0342974734194581)	2359 (0.034)	1668 (0.066)
variant	3	2693(0.0342974734194581)	2359 (0.034)	2548 (0.033)
varying	3	2693(0.0342974734194581)	2359 (0.034)	2548 (0.033)
ventilation	3	1335(0.09145992911855493)	1121 (0.1)	1233 (0.1)
ventricular	3	2693(0.0342974734194581)	1121 (0.1)	#SAKNAS!
ventricular	3	2693(0.0342974734194581)	1121 (0.1)	#SAKNAS!
ventromedial	3	2693(0.0342974734194581)	1516 (0.069)	#SAKNAS!
version	3	628(0.21721733165656798)	1121 (0.1)	584 (0.23)
veterans	3	1485(0.08002743797873557)	1121 (0.1)	1668 (0.066)
vi	3	2693(0.0342974734194581)	#SAKNAS!	1233 (0.1)
video	3	1678(0.0685949468389162)	1121 (0.1)	2548 (0.033)
videofluoroscopic	3	896(0.14862238481765175)	1121 (0.1)	694 (0.2)
viewing	3	2693(0.0342974734194581)	2359 (0.034)	1668 (0.066)
vii	3	2693(0.0342974734194581)	#SAKNAS!	#SAKNAS!
visits	3	1219(0.1028924202583743)	1121 (0.1)	999 (0.13)
vocal	3	2693(0.0342974734194581)	1516 (0.069)	2548 (0.033)
voice	3	1219(0.1028924202583743)	1121 (0.1)	1668 (0.066)
volume-viscosity	3	2693(0.0342974734194581)	1516 (0.069)	2548 (0.033)
voluntary	3	1485(0.08002743797873557)	1121 (0.1)	1668 (0.066)
volunteers	3	2693(0.0342974734194581)	1516 (0.069)	2548 (0.033)
wasp	3	2693(0.0342974734194581)	2359 (0.034)	2548 (0.033)
waste	3	2693(0.0342974734194581)	2359 (0.034)	#SAKNAS!
wearers	3	1678(0.0685949468389162)	1121 (0.1)	#SAKNAS!
week	3	2693(0.0342974734194581)	2359 (0.034)	1668 (0.066)
weevil	3	2693(0.0342974734194581)	1121 (0.1)	#SAKNAS!
weevil	3	2693(0.0342974734194581)	1121 (0.1)	#SAKNAS!
wet	3	2693(0.0342974734194581)	#SAKNAS!	1233 (0.1)
whales	3	2693(0.0342974734194581)	2359 (0.034)	#SAKNAS!
whelk	3	2693(0.0342974734194581)	1516 (0.069)	#SAKNAS!
whose	3	2693(0.0342974734194581)	1516 (0.069)	2548 (0.033)
will	3	2693(0.0342974734194581)	#SAKNAS!	1668 (0.066)
winter	3	2693(0.0342974734194581)	2359 (0.034)	#SAKNAS!
woolly	3	2693(0.0342974734194581)	1516 (0.069)	2548 (0.033)
worker	3	2693(0.0342974734194581)	1516 (0.069)	2548 (0.033)
working-age	3	2693(0.0342974734194581)	#SAKNAS!	#SAKNAS!

worsen	3	2693(0.0342974734194581)	2359 (0.034)	2548 (0.033)
x-ray	3	2693(0.0342974734194581)	2359 (0.034)	#SAKNAS!
years-results	3	2693(0.0342974734194581)	#SAKNAS!	1668 (0.066)
yogurt	3	2693(0.0342974734194581)	2359 (0.034)	1668 (0.066)
zebrafish	3	2693(0.0342974734194581)	#SAKNAS!	#SAKNAS!
zenker	3	1678(0.0685949468389162)	1121 (0.1)	1668 (0.066)
zimbabwe	3	2693(0.0342974734194581)	2359 (0.034)	#SAKNAS!
zinc	3	836(0.16005487595747114)	1121 (0.1)	1668 (0.066)
zn	3	2693(0.0342974734194581)	#SAKNAS!	1668 (0.066)
zooplankton	3	2693(0.0342974734194581)	1121 (0.1)	#SAKNAS!
zooplankton	3	2693(0.0342974734194581)	1121 (0.1)	#SAKNAS!
0,2	2	3437(0.0228649822796387З)	1516 (0.069)	#SAKNAS!
0,2	2	3437(0.02286498227963873)	1516 (0.069)	#SAKNAS!
2	2	3437(0.02286498227963873)	2359 (0.034)	#SAKNAS!
1924	2	3437(0.02286498227963873)	#SAKNAS!	1668 (0.066)
1995	2	3437(0.02286498227963873)	1516 (0.069)	#SAKNAS!
1995	2	3437(0.02286498227963873)	1516 (0.069)	#SAKNAS!
2001	2	3437(0.02286498227963873)	1516 (0.069)	#SAKNAS!
2001	2	3437(0.02286498227963873)	1516 (0.069)	#SAKNAS!
2002	2	3437(0.02286498227963873)	#SAKNAS!	#SAKNAS!
2030	2	3437(0.02286498227963873)	#SAKNAS!	#SAKNAS!
5289	2	3437(0.02286498227963873)	#SAKNAS!	2548 (0.033)
17938	2	3437(0.02286498227963873)	#SAKNAS!	2548 (0.033)
45693	2	3437(0.02286498227963873)	2359 (0.034)	#SAKNAS!
45721	2	1678(0.0685949468389162)	1516 (0.069)	1233 (0.1)
45722	2	3437(0.02286498227963873)	#SAKNAS!	2548 (0.033)
45942	2	3437(0.02286498227963873)	#SAKNAS!	2548 (0.033)
46005	2	3437(0.02286498227963873)	2359 (0.034)	2548 (0.033)
&gt	2	3437(0.02286498227963873)	#SAKNAS!	2548 (0.033)
10-item	2	3437(0.02286498227963873)	#SAKNAS!	1668 (0.066)
11-year	2	3437(0.02286498227963873)	2359 (0.034)	2548 (0.033)
13-to	2	3437(0.02286498227963873)	#SAKNAS!	1668 (0.066)
13-year-old	2	3437(0.02286498227963873)	1516 (0.069)	#SAKNAS!
13-year-old	2	3437(0.02286498227963873)	1516 (0.069)	#SAKNAS!
15-year	2	3437(0.02286498227963873)	#SAKNAS!	2548 (0.033)
1999-2012	2	3437(0.02286498227963873)	#SAKNAS!	1668 (0.066)
1-year-old	2	3437(0.02286498227963873)	1516 (0.069)	#SAKNAS!
1-year-old	2	3437(0.02286498227963873)	1516 (0.069)	#SAKNAS!
2.5-year-old	2	3437(0.02286498227963873)	1516 (0.069)	#SAKNAS!
2.5-year-old	2	3437(0.02286498227963873)	1516 (0.069)	#SAKNAS!
2003-2004	2	3437(0.02286498227963873)	1516 (0.069)	#SAKNAS!
2003-2004	2	3437(0.02286498227963873)	1516 (0.069)	#SAKNAS!
2005-2006	2	3437(0.02286498227963873)	#SAKNAS!	1668 (0.066)

2005-2016	2	3437(0.0228649822796387З)	#SAKNAS!	#SAKNAS!
2007-2008	2	3437(0.0228649822796387З)	2359 (0.034)	2548 (0.033)
2007-2015	2	3437(0.0228649822796387З)	#SAKNAS!	2548 (0.033)
2007-2018	2	3437(0.0228649822796387З)	#SAKNAS!	#SAKNAS!
2008-2014	2	3437(0.0228649822796387З)	#SAKNAS!	1668 (0.066)
2011-2016	2	3437(0.0228649822796387З)	#SAKNAS!	#SAKNAS!
2013-2018	2	3437(0.0228649822796387З)	#SAKNAS!	#SAKNAS!
2020-2021	2	3437(0.0228649822796387З)	#SAKNAS!	#SAKNAS!
20-year	2	3437(0.0228649822796387З)	#SAKNAS!	2548 (0.033)
21st	2	2693(0.0342974734194581)	1516 (0.069)	2548 (0.033)
24-month	2	3437(0.0228649822796387З)	#SAKNAS!	2548 (0.033)
2-ethylhexyl	2	3437(0.0228649822796387З)	#SAKNAS!	2548 (0.033)
2-year	2	1894(0.0571624556990968З)	1516 (0.069)	#SAKNAS!
30-year	2	3437(0.0228649822796387З)	2359 (0.034)	2548 (0.033)
35-	2	3437(0.0228649822796387З)	1516 (0.069)	#SAKNAS!
35-	2	3437(0.0228649822796387З)	1516 (0.069)	#SAKNAS!
3-5-year-old	2	3437(0.0228649822796387З)	#SAKNAS!	2548 (0.033)
3-month	2	3437(0.0228649822796387З)	#SAKNAS!	2548 (0.033)
3-to	2	3437(0.0228649822796387З)	#SAKNAS!	2548 (0.033)
3-year	2	1678(0.0685949468389162)	1516 (0.069)	1668 (0.066)
4-and	2	3437(0.0228649822796387З)	2359 (0.034)	2548 (0.033)
4th	2	3437(0.0228649822796387З)	#SAKNAS!	2548 (0.033)
4-year-old	2	3437(0.0228649822796387З)	2359 (0.034)	2548 (0.033)
50-	2	3437(0.0228649822796387З)	1516 (0.069)	#SAKNAS!
50-	2	3437(0.0228649822796387З)	1516 (0.069)	#SAKNAS!
55-84	2	3437(0.0228649822796387З)	2359 (0.034)	2548 (0.033)
5-fluorouracil	2	3437(0.0228649822796387З)	1516 (0.069)	#SAKNAS!
5-fluorouracil	2	3437(0.0228649822796387З)	1516 (0.069)	#SAKNAS!
60-70-year-old	2	3437(0.0228649822796387З)	1516 (0.069)	#SAKNAS!
60-70-year-old	2	3437(0.0228649822796387З)	1516 (0.069)	#SAKNAS!
6-and	2	3437(0.0228649822796387З)	2359 (0.034)	2548 (0.033)
6j	2	3437(0.0228649822796387З)	2359 (0.034)	#SAKNAS!
6-month	2	3437(0.0228649822796387З)	#SAKNAS!	1668 (0.066)
6-to	2	2693(0.0342974734194581)	1516 (0.069)	2548 (0.033)
70-year-old	2	3437(0.0228649822796387З)	#SAKNAS!	2548 (0.033)
7th	2	3437(0.0228649822796387З)	#SAKNAS!	#SAKNAS!
7-year-old	2	2693(0.0342974734194581)	1516 (0.069)	2548 (0.033)
7-year-olds	2	3437(0.0228649822796387З)	#SAKNAS!	#SAKNAS!
80-year-old	2	2693(0.0342974734194581)	1516 (0.069)	2548 (0.033)
8th	2	3437(0.0228649822796387З)	#SAKNAS!	#SAKNAS!
90-year-old	2	3437(0.0228649822796387З)	#SAKNAS!	#SAKNAS!
aap	2	3437(0.0228649822796387З)	#SAKNAS!	#SAKNAS!
abalone	2	3437(0.0228649822796387З)	2359 (0.034)	2548 (0.033)

ablation	2	3437(0.0228649822796387З)	#SAKNAS!	#SAKNAS!
able	2	3437(0.0228649822796387З)	#SAKNAS!	2548 (0.033)
absolute	2	3437(0.0228649822796387З)	1516 (0.069)	#SAKNAS!
absolute	2	3437(0.0228649822796387З)	1516 (0.069)	#SAKNAS!
abundant	2	2693(0.0342974734194581)	1516 (0.069)	2548 (0.033)
acceleration	2	3437(0.0228649822796387З)	#SAKNAS!	#SAKNAS!
acceptability	2	966(0.1371898936778324)	1516 (0.069)	816 (0.16)
accessible	2	3437(0.0228649822796387З)	2359 (0.034)	#SAKNAS!
accommodation	2	3437(0.0228649822796387З)	2359 (0.034)	#SAKNAS!
accra	2	3437(0.0228649822796387З)	1516 (0.069)	#SAKNAS!
accra	2	3437(0.0228649822796387З)	1516 (0.069)	#SAKNAS!
acculturation	2	3437(0.0228649822796387З)	#SAKNAS!	2548 (0.033)
accumbens	2	3437(0.0228649822796387З)	1516 (0.069)	#SAKNAS!
accumbens	2	3437(0.0228649822796387З)	1516 (0.069)	#SAKNAS!
accumulation	2	1678(0.0685949468389162)	1516 (0.069)	1668 (0.066)
acdf	2	3437(0.0228649822796387З)	#SAKNAS!	2548 (0.033)
acellular	2	2693(0.0342974734194581)	1516 (0.069)	2548 (0.033)
achieve	2	3437(0.0228649822796387З)	2359 (0.034)	#SAKNAS!
acidification	2	3437(0.0228649822796387З)	#SAKNAS!	2548 (0.033)
acidosis	2	3437(0.0228649822796387З)	2359 (0.034)	2548 (0.033)
actinobacillus	2	3437(0.0228649822796387З)	1516 (0.069)	#SAKNAS!
actinobacillus	2	3437(0.0228649822796387З)	1516 (0.069)	#SAKNAS!
activator	2	3437(0.0228649822796387З)	2359 (0.034)	#SAKNAS!
acylpeptidyl	2	3437(0.0228649822796387З)	#SAKNAS!	2548 (0.033)
adaptations	2	2214(0.04572996455927746)	1516 (0.069)	1668 (0.066)
addiction	2	3437(0.0228649822796387З)	#SAKNAS!	1668 (0.066)
adductor	2	3437(0.0228649822796387З)	#SAKNAS!	2548 (0.033)
adequate	2	3437(0.0228649822796387З)	2359 (0.034)	2548 (0.033)
adequately	2	3437(0.0228649822796387З)	2359 (0.034)	#SAKNAS!
adipokines	2	2214(0.04572996455927746)	1516 (0.069)	2548 (0.033)
adipose	2	1485(0.08002743797873557)	1516 (0.069)	1668 (0.066)
adjacent	2	3437(0.0228649822796387З)	2359 (0.034)	#SAKNAS!
adjusting	2	3437(0.0228649822796387З)	#SAKNAS!	2548 (0.033)
adjustment	2	3437(0.0228649822796387З)	#SAKNAS!	2548 (0.033)
ad-libitum	2	3437(0.0228649822796387З)	2359 (0.034)	2548 (0.033)
admission	2	1027(0.12575740253801304)	1516 (0.069)	999 (0.13)
admissions	2	3437(0.0228649822796387З)	#SAKNAS!	#SAKNAS!
adrenergic	2	3437(0.0228649822796387З)	#SAKNAS!	1668 (0.066)
adulthood	2	1678(0.0685949468389162)	1516 (0.069)	1233 (0.1)
advanced-stage	2	2693(0.0342974734194581)	1516 (0.069)	#SAKNAS!
advancement	2	2693(0.0342974734194581)	1516 (0.069)	2548 (0.033)
advances	2	1894(0.05716245569909683)	1516 (0.069)	2548 (0.033)
adversely	2	3437(0.0228649822796387З)	#SAKNAS!	2548 (0.033)

aegypti	2	3437(0.0228649822796387З)	2359 (0.034)	#SAKNAS!
affairs	2	3437(0.0228649822796387З)	1516 (0.069)	#SAKNAS!
affairs	2	3437(0.0228649822796387З)	1516 (0.069)	#SAKNAS!
african-american	2	3437(0.0228649822796387З)	2359 (0.034)	2548 (0.033)
ageing	2	1111(0.11432491139819366)	1516 (0.069)	999 (0.13)
agenda	2	2214(0.04572996455927746)	1516 (0.069)	1668 (0.066)
agent	2	1335(0.09145992911855493)	1516 (0.069)	1233 (0.1)
age-specific	2	3437(0.0228649822796387З)	#SAKNAS!	1668 (0.066)
aggregatibacter	2	1894(0.05716245569909683)	1516 (0.069)	1668 (0.066)
aggregation	2	3437(0.0228649822796387З)	2359 (0.034)	2548 (0.033)
agonist	2	3437(0.0228649822796387З)	2359 (0.034)	2548 (0.033)
agonistic	2	3437(0.0228649822796387З)	2359 (0.034)	#SAKNAS!
agreement	2	3437(0.0228649822796387З)	2359 (0.034)	2548 (0.033)
agrp	2	1894(0.05716245569909683)	1516 (0.069)	2548 (0.033)
airflow	2	3437(0.0228649822796387З)	#SAKNAS!	1668 (0.066)
ajman	2	3437(0.0228649822796387З)	1516 (0.069)	#SAKNAS!
ajman	2	3437(0.0228649822796387З)	1516 (0.069)	#SAKNAS!
albopictus	2	3437(0.0228649822796387З)	2359 (0.034)	2548 (0.033)
alces	2	3437(0.0228649822796387З)	1516 (0.069)	#SAKNAS!
alces	2	3437(0.0228649822796387З)	1516 (0.069)	#SAKNAS!
alert	2	3437(0.0228649822796387З)	#SAKNAS!	2548 (0.033)
aleyrodidae	2	2693(0.0342974734194581)	1516 (0.069)	2548 (0.033)
alfalfa	2	3437(0.0228649822796387З)	#SAKNAS!	1668 (0.066)
algeria	2	3437(0.0228649822796387З)	2359 (0.034)	#SAKNAS!
algerian	2	3437(0.0228649822796387З)	#SAKNAS!	2548 (0.033)
alginate	2	3437(0.0228649822796387З)	#SAKNAS!	2548 (0.033)
algorithms	2	3437(0.0228649822796387З)	#SAKNAS!	#SAKNAS!
alignment	2	3437(0.0228649822796387З)	#SAKNAS!	2548 (0.033)
alkaloid	2	3437(0.0228649822796387З)	1516 (0.069)	#SAKNAS!
alkaloid	2	3437(0.0228649822796387З)	1516 (0.069)	#SAKNAS!
allele	2	3437(0.0228649822796387З)	#SAKNAS!	1668 (0.066)
allergic	2	1678(0.0685949468389162)	1516 (0.069)	1668 (0.066)
alleviates	2	3437(0.0228649822796387З)	2359 (0.034)	#SAKNAS!
alleviation	2	3437(0.0228649822796387З)	#SAKNAS!	1668 (0.066)
allobates	2	3437(0.0228649822796387З)	2359 (0.034)	#SAKNAS!
allocation	2	3437(0.0228649822796387З)	2359 (0.034)	2548 (0.033)
allostatic	2	1894(0.05716245569909683)	1516 (0.069)	1668 (0.066)
alouatta	2	3437(0.0228649822796387З)	2359 (0.034)	2548 (0.033)
alpine	2	3437(0.0228649822796387З)	2359 (0.034)	#SAKNAS!
alter	2	1678(0.0685949468389162)	1516 (0.069)	999 (0.13)
alternate	2	2693(0.0342974734194581)	1516 (0.069)	#SAKNAS!
altitude	2	3437(0.0228649822796387З)	#SAKNAS!	1668 (0.066)
amazonia	2	3437(0.0228649822796387З)	#SAKNAS!	2548 (0.033)

ambient	2	3437(0.0228649822796387З)	1516 (0.069)	#SAKNAS!
ambient	2	3437(0.02286498227963873)	1516 (0.069)	#SAKNAS!
america	2	1894(0.05716245569909683)	1516 (0.069)	2548 (0.033)
americana	2	3437(0.02286498227963873)	1516 (0.069)	#SAKNAS!
americana	2	3437(0.02286498227963873)	1516 (0.069)	#SAKNAS!
ammp-8	2	3437(0.02286498227963873)	#SAKNAS!	2548 (0.033)
amongst	2	1485(0.08002743797873557)	1516 (0.069)	1233 (0.1)
amounts	2	3437(0.02286498227963873)	#SAKNAS!	#SAKNAS!
amphetamine-regulated	2	2693(0.0342974734194581)	1516 (0.069)	#SAKNAS!
amphipod	2	3437(0.02286498227963873)	2359 (0.034)	2548 (0.033)
amyloidosis	2	3437(0.02286498227963873)	#SAKNAS!	2548 (0.033)
amyotrophic-lateral-sclerosis	2	3437(0.02286498227963873)	1516 (0.069)	#SAKNAS!
amyotrophic-lateral-sclerosis	2	3437(0.02286498227963873)	1516 (0.069)	#SAKNAS!
anaerobic	2	3437(0.02286498227963873)	#SAKNAS!	2548 (0.033)
analgesic	2	3437(0.02286498227963873)	#SAKNAS!	2548 (0.033)
analyzing	2	3437(0.02286498227963873)	2359 (0.034)	2548 (0.033)
anastomotic	2	2214(0.04572996455927746)	1516 (0.069)	1668 (0.066)
anastrepha	2	3437(0.02286498227963873)	1516 (0.069)	#SAKNAS!
anastrepha	2	3437(0.02286498227963873)	1516 (0.069)	#SAKNAS!
ancient	2	3437(0.02286498227963873)	#SAKNAS!	#SAKNAS!
andean	2	3437(0.02286498227963873)	#SAKNAS!	2548 (0.033)
angina	2	3437(0.02286498227963873)	#SAKNAS!	1668 (0.066)
ankylosing	2	3437(0.02286498227963873)	2359 (0.034)	2548 (0.033)
anodal	2	3437(0.02286498227963873)	#SAKNAS!	1668 (0.066)
anopheles	2	3437(0.02286498227963873)	2359 (0.034)	2548 (0.033)
anorectic	2	3437(0.02286498227963873)	1516 (0.069)	#SAKNAS!
anorectic	2	3437(0.02286498227963873)	1516 (0.069)	#SAKNAS!
anorexic	2	3437(0.02286498227963873)	2359 (0.034)	2548 (0.033)
anorexigenic	2	3437(0.02286498227963873)	#SAKNAS!	#SAKNAS!
anril	2	3437(0.02286498227963873)	#SAKNAS!	1668 (0.066)
anser	2	3437(0.02286498227963873)	2359 (0.034)	#SAKNAS!
antagonist	2	3437(0.02286498227963873)	1516 (0.069)	#SAKNAS!
antagonist	2	3437(0.02286498227963873)	1516 (0.069)	#SAKNAS!
antarctic	2	2214(0.04572996455927746)	1516 (0.069)	2548 (0.033)
anthropometry	2	1485(0.08002743797873557)	1516 (0.069)	1233 (0.1)
antibacterial	2	3437(0.02286498227963873)	#SAKNAS!	2548 (0.033)
antibiotic	2	3437(0.02286498227963873)	#SAKNAS!	2548 (0.033)
antibiotics	2	2693(0.0342974734194581)	1516 (0.069)	#SAKNAS!
anticholinergics	2	3437(0.02286498227963873)	#SAKNAS!	1668 (0.066)
antidiabetic	2	3437(0.02286498227963873)	#SAKNAS!	1668 (0.066)
antioxidants	2	2214(0.04572996455927746)	1516 (0.069)	1668 (0.066)
antipsychotic	2	3437(0.02286498227963873)	2359 (0.034)	#SAKNAS!
apes	2	3437(0.02286498227963873)	2359 (0.034)	#SAKNAS!

aphthous	2	1894(0.05716245569909683)	1516 (0.069)	1668 (0.066)
apidae	2	2214(0.04572996455927746)	1516 (0.069)	2548 (0.033)
app	2	3437(0.02286498227963873)	#SAKNAS!	2548 (0.033)
appearance	2	3437(0.02286498227963873)	1516 (0.069)	#SAKNAS!
appearance	2	3437(0.02286498227963873)	1516 (0.069)	#SAKNAS!
appetite-regulating	2	3437(0.02286498227963873)	2359 (0.034)	2548 (0.033)
appetites	2	3437(0.02286498227963873)	1516 (0.069)	#SAKNAS!
appetites	2	3437(0.02286498227963873)	1516 (0.069)	#SAKNAS!
applying	2	3437(0.02286498227963873)	#SAKNAS!	2548 (0.033)
appropriate	2	1894(0.05716245569909683)	1516 (0.069)	1668 (0.066)
approximal	2	3437(0.02286498227963873)	1516 (0.069)	#SAKNAS!
approximal	2	3437(0.02286498227963873)	1516 (0.069)	#SAKNAS!
approximation	2	3437(0.02286498227963873)	#SAKNAS!	2548 (0.033)
aquaculture	2	1894(0.05716245569909683)	1516 (0.069)	1668 (0.066)
aqueous	2	3437(0.02286498227963873)	#SAKNAS!	2548 (0.033)
arabia	2	743(0.18291985823710985)	1516 (0.069)	816 (0.16)
araneae	2	3437(0.02286498227963873)	1516 (0.069)	#SAKNAS!
araneae	2	3437(0.02286498227963873)	1516 (0.069)	#SAKNAS!
arch	2	3437(0.02286498227963873)	#SAKNAS!	1668 (0.066)
arcuate	2	3437(0.02286498227963873)	#SAKNAS!	2548 (0.033)
argyrosomus	2	3437(0.02286498227963873)	#SAKNAS!	#SAKNAS!
arizona	2	3437(0.02286498227963873)	1516 (0.069)	#SAKNAS!
arizona	2	3437(0.02286498227963873)	1516 (0.069)	#SAKNAS!
arm	2	3437(0.02286498227963873)	2359 (0.034)	#SAKNAS!
aromatic	2	3437(0.02286498227963873)	#SAKNAS!	#SAKNAS!
aromobatidae	2	3437(0.02286498227963873)	2359 (0.034)	#SAKNAS!
arsenic	2	3437(0.02286498227963873)	#SAKNAS!	2548 (0.033)
artemia	2	3437(0.02286498227963873)	2359 (0.034)	2548 (0.033)
article	2	1894(0.05716245569909683)	1516 (0.069)	1233 (0.1)
ash	2	3437(0.02286498227963873)	1516 (0.069)	#SAKNAS!
ash	2	3437(0.02286498227963873)	1516 (0.069)	#SAKNAS!
asians	2	3437(0.02286498227963873)	2359 (0.034)	2548 (0.033)
aspect	2	3437(0.02286498227963873)	2359 (0.034)	#SAKNAS!
aspirin	2	3437(0.02286498227963873)	#SAKNAS!	1668 (0.066)
assay	2	3437(0.02286498227963873)	#SAKNAS!	1668 (0.066)
assignment	2	3437(0.02286498227963873)	2359 (0.034)	2548 (0.033)
asthmatics	2	3437(0.02286498227963873)	#SAKNAS!	#SAKNAS!
ataxia	2	2214(0.04572996455927746)	1516 (0.069)	2548 (0.033)
atcc	2	3437(0.02286498227963873)	#SAKNAS!	2548 (0.033)
atkinson	2	3437(0.02286498227963873)	1516 (0.069)	#SAKNAS!
atkinson	2	3437(0.02286498227963873)	1516 (0.069)	#SAKNAS!
atlas	2	3437(0.02286498227963873)	#SAKNAS!	2548 (0.033)
atmospheric	2	3437(0.02286498227963873)	2359 (0.034)	2548 (0.033)

atopic	2	3437(0.0228649822796387З)	#SAKNAS!	2548 (0.033)
atp	2	3437(0.0228649822796387З)	2359 (0.034)	2548 (0.033)
attainment	2	3437(0.0228649822796387З)	2359 (0.034)	2548 (0.033)
attendance	2	3437(0.0228649822796387З)	#SAKNAS!	1668 (0.066)
attention	2	2214(0.04572996455927746)	1516 (0.069)	1668 (0.066)
attenuation	2	3437(0.0228649822796387З)	2359 (0.034)	2548 (0.033)
attributes	2	1894(0.05716245569909683)	1516 (0.069)	1668 (0.066)
atypical	2	3437(0.0228649822796387З)	1516 (0.069)	#SAKNAS!
atypical	2	3437(0.0228649822796387З)	1516 (0.069)	#SAKNAS!
auckland	2	3437(0.0228649822796387З)	#SAKNAS!	1668 (0.066)
auratus	2	3437(0.0228649822796387З)	#SAKNAS!	1668 (0.066)
auscultation	2	3437(0.0228649822796387З)	#SAKNAS!	2548 (0.033)
australasian	2	3437(0.0228649822796387З)	#SAKNAS!	2548 (0.033)
australostichopus	2	3437(0.0228649822796387З)	2359 (0.034)	2548 (0.033)
austria	2	3437(0.0228649822796387З)	1516 (0.069)	#SAKNAS!
austria	2	3437(0.0228649822796387З)	1516 (0.069)	#SAKNAS!
authoritative	2	3437(0.0228649822796387З)	1516 (0.069)	#SAKNAS!
authoritative	2	3437(0.0228649822796387З)	1516 (0.069)	#SAKNAS!
autologous	2	1894(0.05716245569909683)	1516 (0.069)	2548 (0.033)
automatic	2	1894(0.05716245569909683)	1516 (0.069)	2548 (0.033)
auxiliary	2	3437(0.0228649822796387З)	#SAKNAS!	2548 (0.033)
avahi	2	3437(0.0228649822796387З)	1516 (0.069)	#SAKNAS!
avahi	2	3437(0.0228649822796387З)	1516 (0.069)	#SAKNAS!
avenues	2	3437(0.0228649822796387З)	1516 (0.069)	#SAKNAS!
avenues	2	3437(0.0228649822796387З)	1516 (0.069)	#SAKNAS!
aversion	2	1894(0.05716245569909683)	1516 (0.069)	2548 (0.033)
awaiting	2	3437(0.0228649822796387З)	#SAKNAS!	1668 (0.066)
awareness	2	1111(0.11432491139819366)	1516 (0.069)	1668 (0.066)
away	2	3437(0.0228649822796387З)	2359 (0.034)	#SAKNAS!
azar	2	3437(0.0228649822796387З)	#SAKNAS!	#SAKNAS!
bacillus	2	2693(0.0342974734194581)	1516 (0.069)	2548 (0.033)
background	2	3437(0.0228649822796387З)	2359 (0.034)	#SAKNAS!
balsam	2	3437(0.0228649822796387З)	1516 (0.069)	#SAKNAS!
balsam	2	3437(0.0228649822796387З)	1516 (0.069)	#SAKNAS!
band	2	1485(0.08002743797873557)	1516 (0.069)	1233 (0.1)
bands	2	3437(0.0228649822796387З)	2359 (0.034)	2548 (0.033)
bangladeshi	2	2693(0.0342974734194581)	1516 (0.069)	2548 (0.033)
bar	2	3437(0.0228649822796387З)	#SAKNAS!	1668 (0.066)
barley	2	3437(0.0228649822796387З)	#SAKNAS!	2548 (0.033)
barrett	2	3437(0.0228649822796387З)	1516 (0.069)	#SAKNAS!
barrett	2	3437(0.0228649822796387З)	1516 (0.069)	#SAKNAS!
barrier	2	3437(0.0228649822796387З)	2359 (0.034)	#SAKNAS!
based	2	3437(0.0228649822796387З)	#SAKNAS!	2548 (0.033)

basic	2	1335(0.09145992911855493)	1516 (0.069)	2548 (0.033)
basin	2	3437(0.02286498227963873)	#SAKNAS!	1668 (0.066)
bay	2	2693(0.0342974734194581)	1516 (0.069)	2548 (0.033)
bdnf	2	2693(0.0342974734194581)	1516 (0.069)	#SAKNAS!
beads	2	3437(0.02286498227963873)	#SAKNAS!	#SAKNAS!
bear	2	3437(0.02286498227963873)	1516 (0.069)	#SAKNAS!
bear	2	3437(0.02286498227963873)	1516 (0.069)	#SAKNAS!
bears	2	3437(0.02286498227963873)	2359 (0.034)	2548 (0.033)
bedside	2	1335(0.09145992911855493)	1516 (0.069)	1668 (0.066)
beef	2	1027(0.12575740253801304)	1516 (0.069)	1668 (0.066)
beginning	2	3437(0.02286498227963873)	#SAKNAS!	1668 (0.066)
behavioral-assessment	2	3437(0.02286498227963873)	1516 (0.069)	#SAKNAS!
behavioral-assessment	2	3437(0.02286498227963873)	1516 (0.069)	#SAKNAS!
belgium	2	3437(0.02286498227963873)	1516 (0.069)	#SAKNAS!
belgium	2	3437(0.02286498227963873)	1516 (0.069)	#SAKNAS!
benefit	2	1219(0.1028924202583743)	1516 (0.069)	1233 (0.1)
benthos	2	3437(0.02286498227963873)	1516 (0.069)	#SAKNAS!
benthos	2	3437(0.02286498227963873)	1516 (0.069)	#SAKNAS!
biases	2	3437(0.02286498227963873)	2359 (0.034)	2548 (0.033)
bibliometric	2	3437(0.02286498227963873)	#SAKNAS!	#SAKNAS!
bigel	2	3437(0.02286498227963873)	#SAKNAS!	#SAKNAS!
bilateral	2	3437(0.02286498227963873)	#SAKNAS!	2548 (0.033)
binge	2	2214(0.04572996455927746)	1516 (0.069)	1668 (0.066)
bioaccumulation	2	3437(0.02286498227963873)	1516 (0.069)	#SAKNAS!
bioaccumulation	2	3437(0.02286498227963873)	1516 (0.069)	#SAKNAS!
bioactive	2	3437(0.02286498227963873)	2359 (0.034)	2548 (0.033)
bioactivity	2	3437(0.02286498227963873)	2359 (0.034)	#SAKNAS!
biochemistry	2	3437(0.02286498227963873)	2359 (0.034)	2548 (0.033)
biocultural	2	3437(0.02286498227963873)	#SAKNAS!	2548 (0.033)
biodegradable	2	3437(0.02286498227963873)	2359 (0.034)	2548 (0.033)
biodiesel	2	3437(0.02286498227963873)	#SAKNAS!	2548 (0.033)
bioelectrical	2	3437(0.02286498227963873)	#SAKNAS!	#SAKNAS!
biologic	2	3437(0.02286498227963873)	2359 (0.034)	#SAKNAS!
biological	2	743(0.18291985823710985)	1516 (0.069)	999 (0.13)
biomass	2	3437(0.02286498227963873)	2359 (0.034)	2548 (0.033)
biopsies	2	3437(0.02286498227963873)	#SAKNAS!	2548 (0.033)
biopsy	2	3437(0.02286498227963873)	#SAKNAS!	#SAKNAS!
biting	2	3437(0.02286498227963873)	2359 (0.034)	#SAKNAS!
bleeding	2	1485(0.08002743797873557)	1516 (0.069)	1233 (0.1)
blue	2	2693(0.0342974734194581)	1516 (0.069)	#SAKNAS!
bmi-1	2	3437(0.02286498227963873)	2359 (0.034)	#SAKNAS!
bmi-for-age	2	3437(0.02286498227963873)	#SAKNAS!	#SAKNAS!
body-mass	2	2693(0.0342974734194581)	1516 (0.069)	2548 (0.033)

body-weight	2	3437(0.0228649822796387 3)	1516 (0.069)	#SAKNAS!
body-weight	2	3437(0.0228649822796387 3)	1516 (0.069)	#SAKNAS!
borer	2	2214(0.0457299645592774 6)	1516 (0.069)	1668 (0.066)
boron	2	3437(0.0228649822796387 3)	#SAKNAS!	#SAKNAS!
boundaries	2	3437(0.0228649822796387 3)	#SAKNAS!	1668 (0.066)
bovine	2	3437(0.0228649822796387 3)	2359 (0.034)	2548 (0.033)
bp	2	3437(0.0228649822796387 3)	2359 (0.034)	2548 (0.033)
brachionus	2	3437(0.0228649822796387 3)	#SAKNAS!	#SAKNAS!
braconidae	2	3437(0.0228649822796387 3)	2359 (0.034)	2548 (0.033)
brazilians	2	2693(0.0342974734194581)	1516 (0.069)	#SAKNAS!
breathing	2	1219(0.1028924202583743)	1516 (0.069)	816 (0.16)
breeding	2	2693(0.0342974734194581)	1516 (0.069)	2548 (0.033)
breeds	2	3437(0.0228649822796387 3)	2359 (0.034)	2548 (0.033)
bridging	2	3437(0.0228649822796387 3)	2359 (0.034)	#SAKNAS!
broiler	2	1894(0.0571624556990968 3)	1516 (0.069)	2548 (0.033)
broken	2	3437(0.0228649822796387 3)	2359 (0.034)	2548 (0.033)
brominated	2	3437(0.0228649822796387 3)	#SAKNAS!	#SAKNAS!
bronchial	2	3437(0.0228649822796387 3)	#SAKNAS!	2548 (0.033)
browser	2	3437(0.0228649822796387 3)	#SAKNAS!	1668 (0.066)
brush	2	3437(0.0228649822796387 3)	2359 (0.034)	2548 (0.033)
brushing	2	3437(0.0228649822796387 3)	#SAKNAS!	2548 (0.033)
buckthorn	2	3437(0.0228649822796387 3)	2359 (0.034)	#SAKNAS!
buffalo	2	2214(0.0457299645592774 6)	1516 (0.069)	2548 (0.033)
buffers	2	3437(0.0228649822796387 3)	#SAKNAS!	1668 (0.066)
bulls	2	2214(0.0457299645592774 6)	1516 (0.069)	#SAKNAS!
bumblebees	2	3437(0.0228649822796387 3)	2359 (0.034)	2548 (0.033)
burns	2	3437(0.0228649822796387 3)	#SAKNAS!	1668 (0.066)
butterfly	2	1894(0.0571624556990968 3)	1516 (0.069)	2548 (0.033)
butterflyfish	2	3437(0.0228649822796387 3)	2359 (0.034)	2548 (0.033)
byproducts	2	3437(0.0228649822796387 3)	#SAKNAS!	2548 (0.033)
by-products	2	3437(0.0228649822796387 3)	#SAKNAS!	2548 (0.033)
c2-c7	2	3437(0.0228649822796387 3)	2359 (0.034)	2548 (0.033)
cacopsylla	2	3437(0.0228649822796387 3)	#SAKNAS!	1668 (0.066)
calanoid	2	3437(0.0228649822796387 3)	1516 (0.069)	#SAKNAS!
calanoid	2	3437(0.0228649822796387 3)	1516 (0.069)	#SAKNAS!
calculus	2	1894(0.0571624556990968 3)	1516 (0.069)	#SAKNAS!
calonectris	2	3437(0.0228649822796387 3)	2359 (0.034)	2548 (0.033)
calories	2	3437(0.0228649822796387 3)	#SAKNAS!	2548 (0.033)
calotropis	2	3437(0.0228649822796387 3)	2359 (0.034)	2548 (0.033)
camel	2	3437(0.0228649822796387 3)	1516 (0.069)	#SAKNAS!
camel	2	3437(0.0228649822796387 3)	1516 (0.069)	#SAKNAS!
cameroon	2	3437(0.0228649822796387 3)	#SAKNAS!	#SAKNAS!
canada	2	2214(0.0457299645592774 6)	1516 (0.069)	2548 (0.033)

canadian	2	1111(0.11432491139819366)	1516 (0.069)	1668 (0.066)
canadians	2	3437(0.02286498227963873)	1516 (0.069)	#SAKNAS!
canadians	2	3437(0.02286498227963873)	1516 (0.069)	#SAKNAS!
canal	2	3437(0.02286498227963873)	#SAKNAS!	#SAKNAS!
cancer-a	2	3437(0.02286498227963873)	2359 (0.034)	#SAKNAS!
candida	2	1894(0.05716245569909683)	1516 (0.069)	#SAKNAS!
candidates	2	3437(0.02286498227963873)	#SAKNAS!	2548 (0.033)
candidatus	2	3437(0.02286498227963873)	#SAKNAS!	1668 (0.066)
candidiasis	2	2214(0.04572996455927746)	1516 (0.069)	1668 (0.066)
cannabis	2	3437(0.02286498227963873)	#SAKNAS!	2548 (0.033)
cannibalistic	2	3437(0.02286498227963873)	2359 (0.034)	2548 (0.033)
capitata	2	3437(0.02286498227963873)	1516 (0.069)	#SAKNAS!
capitata	2	3437(0.02286498227963873)	1516 (0.069)	#SAKNAS!
capra	2	3437(0.02286498227963873)	1516 (0.069)	#SAKNAS!
capra	2	3437(0.02286498227963873)	1516 (0.069)	#SAKNAS!
capsules	2	3437(0.02286498227963873)	1516 (0.069)	#SAKNAS!
capsules	2	3437(0.02286498227963873)	1516 (0.069)	#SAKNAS!
captivity	2	3437(0.02286498227963873)	2359 (0.034)	2548 (0.033)
capture	2	3437(0.02286498227963873)	2359 (0.034)	#SAKNAS!
carassius	2	3437(0.02286498227963873)	#SAKNAS!	1668 (0.066)
carbohydrate-rich	2	3437(0.02286498227963873)	2359 (0.034)	#SAKNAS!
cardia	2	3437(0.02286498227963873)	1516 (0.069)	#SAKNAS!
cardia	2	3437(0.02286498227963873)	1516 (0.069)	#SAKNAS!
caregiver-infant	2	3437(0.02286498227963873)	#SAKNAS!	2548 (0.033)
caries-a	2	3437(0.02286498227963873)	#SAKNAS!	#SAKNAS!
caring	2	3437(0.02286498227963873)	#SAKNAS!	2548 (0.033)
carious	2	1894(0.05716245569909683)	1516 (0.069)	2548 (0.033)
carnivora	2	3437(0.02286498227963873)	1516 (0.069)	#SAKNAS!
carnivora	2	3437(0.02286498227963873)	1516 (0.069)	#SAKNAS!
carnivore	2	3437(0.02286498227963873)	2359 (0.034)	2548 (0.033)
carnivory	2	3437(0.02286498227963873)	#SAKNAS!	2548 (0.033)
carolina	2	2693(0.0342974734194581)	1516 (0.069)	#SAKNAS!
cart	2	2693(0.0342974734194581)	1516 (0.069)	#SAKNAS!
cartilage	2	3437(0.02286498227963873)	#SAKNAS!	1668 (0.066)
case-matched	2	3437(0.02286498227963873)	2359 (0.034)	2548 (0.033)
case-series	2	3437(0.02286498227963873)	#SAKNAS!	#SAKNAS!
castaneum	2	3437(0.02286498227963873)	#SAKNAS!	1668 (0.066)
cat	2	3437(0.02286498227963873)	1516 (0.069)	#SAKNAS!
cat	2	3437(0.02286498227963873)	1516 (0.069)	#SAKNAS!
cataract	2	3437(0.02286498227963873)	1516 (0.069)	#SAKNAS!
cataract	2	3437(0.02286498227963873)	1516 (0.069)	#SAKNAS!
catfish	2	3437(0.02286498227963873)	2359 (0.034)	2548 (0.033)
catheter	2	3437(0.02286498227963873)	1516 (0.069)	#SAKNAS!

catheter	2	3437(0.0228649822796387З)	1516 (0.069)	#SAKNAS!
catla	2	3437(0.02286498227963873)	2359 (0.034)	2548 (0.033)
cats	2	1485(0.08002743797873557)	1516 (0.069)	1668 (0.066)
catta	2	3437(0.02286498227963873)	1516 (0.069)	#SAKNAS!
catta	2	3437(0.02286498227963873)	1516 (0.069)	#SAKNAS!
caustic	2	2214(0.04572996455927746)	1516 (0.069)	1668 (0.066)
cavia	2	3437(0.02286498227963873)	#SAKNAS!	2548 (0.033)
cd	2	2693(0.0342974734194581)	1516 (0.069)	2548 (0.033)
cellular	2	3437(0.02286498227963873)	2359 (0.034)	2548 (0.033)
cellulose	2	3437(0.02286498227963873)	2359 (0.034)	#SAKNAS!
centres	2	3437(0.02286498227963873)	1516 (0.069)	#SAKNAS!
centres	2	3437(0.02286498227963873)	1516 (0.069)	#SAKNAS!
cerastoderma	2	3437(0.02286498227963873)	1516 (0.069)	#SAKNAS!
cerastoderma	2	3437(0.02286498227963873)	1516 (0.069)	#SAKNAS!
ceratitis	2	3437(0.02286498227963873)	1516 (0.069)	#SAKNAS!
ceratitis	2	3437(0.02286498227963873)	1516 (0.069)	#SAKNAS!
cereal	2	2214(0.04572996455927746)	1516 (0.069)	1668 (0.066)
cerebellum	2	3437(0.02286498227963873)	2359 (0.034)	#SAKNAS!
cerebral-palsy	2	3437(0.02286498227963873)	1516 (0.069)	#SAKNAS!
cerebral-palsy	2	3437(0.02286498227963873)	1516 (0.069)	#SAKNAS!
cerevisiae	2	3437(0.02286498227963873)	#SAKNAS!	2548 (0.033)
certain	2	3437(0.02286498227963873)	#SAKNAS!	2548 (0.033)
chaetodon	2	3437(0.02286498227963873)	2359 (0.034)	2548 (0.033)
chagasic	2	3437(0.02286498227963873)	2359 (0.034)	2548 (0.033)
chairside	2	3437(0.02286498227963873)	2359 (0.034)	2548 (0.033)
chance	2	3437(0.02286498227963873)	2359 (0.034)	#SAKNAS!
changing	2	2214(0.04572996455927746)	1516 (0.069)	#SAKNAS!
characterisation	2	3437(0.02286498227963873)	#SAKNAS!	2548 (0.033)
characteristic	2	3437(0.02286498227963873)	2359 (0.034)	2548 (0.033)
charolais	2	3437(0.02286498227963873)	1516 (0.069)	#SAKNAS!
charolais	2	3437(0.02286498227963873)	1516 (0.069)	#SAKNAS!
chd	2	3437(0.02286498227963873)	2359 (0.034)	2548 (0.033)
cheap	2	3437(0.02286498227963873)	2359 (0.034)	#SAKNAS!
checkpoint	2	3437(0.02286498227963873)	#SAKNAS!	2548 (0.033)
chemerin	2	3437(0.02286498227963873)	#SAKNAS!	1668 (0.066)
chemical-composition	2	3437(0.02286498227963873)	1516 (0.069)	#SAKNAS!
chemical-composition	2	3437(0.02286498227963873)	1516 (0.069)	#SAKNAS!
chemically	2	3437(0.02286498227963873)	2359 (0.034)	2548 (0.033)
chemistry	2	1485(0.08002743797873557)	1516 (0.069)	816 (0.16)
chemo-radiotherapy	2	3437(0.02286498227963873)	#SAKNAS!	2548 (0.033)
chicks	2	3437(0.02286498227963873)	1516 (0.069)	#SAKNAS!
chicks	2	3437(0.02286498227963873)	1516 (0.069)	#SAKNAS!
childbearing	2	1894(0.05716245569909683)	1516 (0.069)	1668 (0.066)

childbirth	2	3437(0.0228649822796387З)	#SAKNAS!	1668 (0.066)
chilean	2	1219(0.1028924202583743)	1516 (0.069)	1233 (0.1)
chilonis	2	3437(0.0228649822796387З)	#SAKNAS!	1668 (0.066)
chimpanzee	2	3437(0.0228649822796387З)	2359 (0.034)	2548 (0.033)
chimpanzees	2	1335(0.09145992911855493)	1516 (0.069)	999 (0.13)
chinese-american	2	3437(0.0228649822796387З)	2359 (0.034)	#SAKNAS!
chlorhexidine	2	3437(0.0228649822796387З)	1516 (0.069)	#SAKNAS!
chlorhexidine	2	3437(0.0228649822796387З)	1516 (0.069)	#SAKNAS!
chloride	2	3437(0.0228649822796387З)	2359 (0.034)	#SAKNAS!
choices	2	1335(0.09145992911855493)	1516 (0.069)	999 (0.13)
chrysomelas	2	3437(0.0228649822796387З)	#SAKNAS!	2548 (0.033)
chrysophyte	2	3437(0.0228649822796387З)	2359 (0.034)	#SAKNAS!
circuits	2	2693(0.0342974734194581)	1516 (0.069)	2548 (0.033)
circulating	2	1111(0.11432491139819366)	1516 (0.069)	1668 (0.066)
circulation	2	3437(0.0228649822796387З)	#SAKNAS!	2548 (0.033)
cisplatin	2	3437(0.0228649822796387З)	2359 (0.034)	2548 (0.033)
citri	2	3437(0.0228649822796387З)	#SAKNAS!	2548 (0.033)
claim	2	3437(0.0228649822796387З)	#SAKNAS!	1668 (0.066)
clams	2	3437(0.0228649822796387З)	2359 (0.034)	#SAKNAS!
class	2	1111(0.11432491139819366)	1516 (0.069)	816 (0.16)
clavata	2	3437(0.0228649822796387З)	#SAKNAS!	2548 (0.033)
clefts	2	3437(0.0228649822796387З)	2359 (0.034)	#SAKNAS!
cleveland	2	3437(0.0228649822796387З)	2359 (0.034)	2548 (0.033)
clients	2	1894(0.0571624556990968З)	1516 (0.069)	1233 (0.1)
climate	2	3437(0.0228649822796387З)	#SAKNAS!	2548 (0.033)
clinician	2	3437(0.0228649822796387З)	#SAKNAS!	#SAKNAS!
clusters	2	3437(0.0228649822796387З)	#SAKNAS!	#SAKNAS!
cmi	2	3437(0.0228649822796387З)	#SAKNAS!	#SAKNAS!
cncm	2	3437(0.0228649822796387З)	#SAKNAS!	2548 (0.033)
cocaine	2	2214(0.04572996455927746)	1516 (0.069)	2548 (0.033)
coccinellidae	2	3437(0.0228649822796387З)	2359 (0.034)	2548 (0.033)
cockroach	2	3437(0.0228649822796387З)	2359 (0.034)	#SAKNAS!
coconut	2	3437(0.0228649822796387З)	#SAKNAS!	2548 (0.033)
coincidence	2	3437(0.0228649822796387З)	1516 (0.069)	#SAKNAS!
coincidence	2	3437(0.0228649822796387З)	1516 (0.069)	#SAKNAS!
coleomegilla	2	3437(0.0228649822796387З)	2359 (0.034)	2548 (0.033)
collective	2	2214(0.04572996455927746)	1516 (0.069)	2548 (0.033)
collis	2	3437(0.0228649822796387З)	#SAKNAS!	1668 (0.066)
colombia	2	1678(0.0685949468389162)	1516 (0.069)	1233 (0.1)
colombian	2	3437(0.0228649822796387З)	#SAKNAS!	#SAKNAS!
colorado	2	3437(0.0228649822796387З)	2359 (0.034)	2548 (0.033)
color-changeable	2	3437(0.0228649822796387З)	#SAKNAS!	2548 (0.033)
colossoma	2	3437(0.0228649822796387З)	#SAKNAS!	1668 (0.066)

com-b	2	3437(0.0228649822796387З)	#SAKNAS!	#SAKNAS!
combination	2	1485(0.08002743797873557)	1516 (0.069)	999 (0.13)
combinations	2	3437(0.02286498227963873)	#SAKNAS!	#SAKNAS!
come	2	3437(0.02286498227963873)	2359 (0.034)	2548 (0.033)
commercial	2	966(0.1371898936778324)	1516 (0.069)	694 (0.2)
communicating	2	3437(0.02286498227963873)	#SAKNAS!	2548 (0.033)
community-level	2	3437(0.02286498227963873)	#SAKNAS!	2548 (0.033)
comorbidities	2	1335(0.09145992911855493)	1516 (0.069)	1233 (0.1)
comorbidity	2	1485(0.08002743797873557)	1516 (0.069)	1233 (0.1)
companion	2	3437(0.02286498227963873)	2359 (0.034)	2548 (0.033)
comparable	2	3437(0.02286498227963873)	#SAKNAS!	2548 (0.033)
compare	2	3437(0.02286498227963873)	#SAKNAS!	2548 (0.033)
compensation	2	3437(0.02286498227963873)	1516 (0.069)	#SAKNAS!
compensation	2	3437(0.02286498227963873)	1516 (0.069)	#SAKNAS!
competing	2	3437(0.02286498227963873)	#SAKNAS!	2548 (0.033)
competition	2	1894(0.05716245569909683)	1516 (0.069)	2548 (0.033)
complementary	2	595(0.22864982279638732)	1516 (0.069)	333 (0.43)
completion	2	3437(0.02286498227963873)	2359 (0.034)	2548 (0.033)
complex	2	896(0.14862238481765175)	1516 (0.069)	999 (0.13)
complicating	2	2693(0.0342974734194581)	1516 (0.069)	#SAKNAS!
compositions	2	3437(0.02286498227963873)	2359 (0.034)	#SAKNAS!
computational	2	3437(0.02286498227963873)	2359 (0.034)	#SAKNAS!
computer	2	3437(0.02286498227963873)	#SAKNAS!	1668 (0.066)
concept	2	1485(0.08002743797873557)	1516 (0.069)	816 (0.16)
concepts	2	3437(0.02286498227963873)	2359 (0.034)	#SAKNAS!
concord	2	3437(0.02286498227963873)	#SAKNAS!	#SAKNAS!
conditional	2	3437(0.02286498227963873)	#SAKNAS!	2548 (0.033)
conditioning	2	3437(0.02286498227963873)	2359 (0.034)	2548 (0.033)
confectionery	2	3437(0.02286498227963873)	2359 (0.034)	2548 (0.033)
conference	2	3437(0.02286498227963873)	#SAKNAS!	1668 (0.066)
confirmatory	2	3437(0.02286498227963873)	2359 (0.034)	#SAKNAS!
congestive	2	3437(0.02286498227963873)	2359 (0.034)	2548 (0.033)
congo	2	2693(0.0342974734194581)	1516 (0.069)	#SAKNAS!
consecutive	2	1678(0.0685949468389162)	1516 (0.069)	1233 (0.1)
conservative	2	3437(0.02286498227963873)	#SAKNAS!	2548 (0.033)
consistency	2	1894(0.05716245569909683)	1516 (0.069)	1233 (0.1)
conspecific	2	3437(0.02286498227963873)	2359 (0.034)	2548 (0.033)
constituents	2	3437(0.02286498227963873)	2359 (0.034)	2548 (0.033)
constrictor	2	3437(0.02286498227963873)	#SAKNAS!	#SAKNAS!
consumers	2	3437(0.02286498227963873)	2359 (0.034)	2548 (0.033)
contaminant	2	3437(0.02286498227963873)	2359 (0.034)	2548 (0.033)
contaminants	2	3437(0.02286498227963873)	2359 (0.034)	2548 (0.033)
context-dependent	2	3437(0.02286498227963873)	#SAKNAS!	#SAKNAS!

continuing	2	3437(0.02286498227963873)	1516 (0.069)	#SAKNAS!
continuing	2	3437(0.02286498227963873)	1516 (0.069)	#SAKNAS!
continuously	2	3437(0.02286498227963873)	2359 (0.034)	#SAKNAS!
contraindication	2	3437(0.02286498227963873)	2359 (0.034)	2548 (0.033)
contribute	2	1678(0.0685949468389162)	1516 (0.069)	1668 (0.066)
contributes	2	3437(0.02286498227963873)	2359 (0.034)	#SAKNAS!
co-occurrence	2	3437(0.02286498227963873)	#SAKNAS!	#SAKNAS!
cooking	2	3437(0.02286498227963873)	#SAKNAS!	2548 (0.033)
cooperation	2	3437(0.02286498227963873)	2359 (0.034)	2548 (0.033)
coordination	2	3437(0.02286498227963873)	#SAKNAS!	1668 (0.066)
copper	2	3437(0.02286498227963873)	#SAKNAS!	1668 (0.066)
coprophagy	2	3437(0.02286498227963873)	2359 (0.034)	2548 (0.033)
corals	2	2693(0.0342974734194581)	1516 (0.069)	2548 (0.033)
cordatus	2	3437(0.02286498227963873)	#SAKNAS!	2548 (0.033)
core	2	3437(0.02286498227963873)	#SAKNAS!	2548 (0.033)
coronal	2	1678(0.0685949468389162)	1516 (0.069)	999 (0.13)
coronavirus	2	3437(0.02286498227963873)	#SAKNAS!	#SAKNAS!
correction	2	3437(0.02286498227963873)	2359 (0.034)	2548 (0.033)
cortex	2	1485(0.08002743797873557)	1516 (0.069)	1233 (0.1)
cost	2	1678(0.0685949468389162)	1516 (0.069)	1668 (0.066)
cotinine	2	3437(0.02286498227963873)	#SAKNAS!	2548 (0.033)
coturnix	2	3437(0.02286498227963873)	1516 (0.069)	#SAKNAS!
coturnix	2	3437(0.02286498227963873)	1516 (0.069)	#SAKNAS!
count	2	1111(0.11432491139819366)	1516 (0.069)	694 (0.2)
counterparts	2	3437(0.02286498227963873)	2359 (0.034)	2548 (0.033)
course	2	966(0.1371898936778324)	1516 (0.069)	999 (0.13)
cow	2	3437(0.02286498227963873)	1516 (0.069)	#SAKNAS!
cow	2	3437(0.02286498227963873)	1516 (0.069)	#SAKNAS!
cpd	2	3437(0.02286498227963873)	#SAKNAS!	1668 (0.066)
crab	2	896(0.14862238481765175)	1516 (0.069)	526 (0.26)
crabs	2	3437(0.02286498227963873)	1516 (0.069)	#SAKNAS!
crabs	2	3437(0.02286498227963873)	1516 (0.069)	#SAKNAS!
cranial	2	3437(0.02286498227963873)	2359 (0.034)	2548 (0.033)
crickets	2	3437(0.02286498227963873)	#SAKNAS!	2548 (0.033)
cricopharyngeus	2	3437(0.02286498227963873)	#SAKNAS!	#SAKNAS!
cristatus	2	3437(0.02286498227963873)	2359 (0.034)	#SAKNAS!
critical	2	1219(0.1028924202583743)	1516 (0.069)	1233 (0.1)
crop	2	3437(0.02286498227963873)	2359 (0.034)	2548 (0.033)
crowns	2	3437(0.02286498227963873)	#SAKNAS!	#SAKNAS!
crp	2	1894(0.05716245569909683)	1516 (0.069)	1668 (0.066)
crucial	2	3437(0.02286498227963873)	#SAKNAS!	1668 (0.066)
crude	2	2693(0.0342974734194581)	1516 (0.069)	2548 (0.033)
crural	2	3437(0.02286498227963873)	1516 (0.069)	#SAKNAS!

crural	2	3437(0.02286498227963873)	1516 (0.069)	#SAKNAS!
cruroplasty	2	3437(0.02286498227963873)	2359 (0.034)	#SAKNAS!
crustacea	2	3437(0.02286498227963873)	1516 (0.069)	#SAKNAS!
crustacea	2	3437(0.02286498227963873)	1516 (0.069)	#SAKNAS!
ctenomys	2	3437(0.02286498227963873)	2359 (0.034)	2548 (0.033)
cue	2	3437(0.02286498227963873)	2359 (0.034)	2548 (0.033)
culicidae	2	3437(0.02286498227963873)	2359 (0.034)	2548 (0.033)
culinary	2	3437(0.02286498227963873)	#SAKNAS!	2548 (0.033)
cultivars	2	3437(0.02286498227963873)	2359 (0.034)	#SAKNAS!
cultural	2	1335(0.09145992911855493)	1516 (0.069)	694 (0.2)
curricula	2	3437(0.02286498227963873)	2359 (0.034)	#SAKNAS!
curve	2	2214(0.04572996455927746)	1516 (0.069)	2548 (0.033)
cyclic	2	2214(0.04572996455927746)	1516 (0.069)	2548 (0.033)
cynodon	2	3437(0.02286498227963873)	#SAKNAS!	2548 (0.033)
cytokine	2	1678(0.0685949468389162)	1516 (0.069)	2548 (0.033)
cytokines	2	1027(0.12575740253801304)	1516 (0.069)	999 (0.13)
czech	2	3437(0.02286498227963873)	2359 (0.034)	2548 (0.033)
d3	2	2693(0.0342974734194581)	1516 (0.069)	#SAKNAS!
dactylon	2	3437(0.02286498227963873)	#SAKNAS!	2548 (0.033)
dalmatian	2	3437(0.02286498227963873)	#SAKNAS!	#SAKNAS!
danes	2	3437(0.02286498227963873)	2359 (0.034)	2548 (0.033)
danger	2	3437(0.02286498227963873)	#SAKNAS!	2548 (0.033)
daphnia	2	2214(0.04572996455927746)	1516 (0.069)	2548 (0.033)
databases	2	3437(0.02286498227963873)	1516 (0.069)	#SAKNAS!
databases	2	3437(0.02286498227963873)	1516 (0.069)	#SAKNAS!
dataset	2	3437(0.02286498227963873)	#SAKNAS!	#SAKNAS!
daycare	2	3437(0.02286498227963873)	2359 (0.034)	#SAKNAS!
day-case	2	3437(0.02286498227963873)	1516 (0.069)	#SAKNAS!
day-case	2	3437(0.02286498227963873)	1516 (0.069)	#SAKNAS!
daytime	2	3437(0.02286498227963873)	#SAKNAS!	2548 (0.033)
decapoda	2	2693(0.0342974734194581)	1516 (0.069)	2548 (0.033)
decide	2	3437(0.02286498227963873)	1516 (0.069)	#SAKNAS!
decide	2	3437(0.02286498227963873)	1516 (0.069)	#SAKNAS!
deciphering	2	3437(0.02286498227963873)	2359 (0.034)	#SAKNAS!
decision	2	1678(0.0685949468389162)	1516 (0.069)	2548 (0.033)
decisions	2	1219(0.1028924202583743)	1516 (0.069)	694 (0.2)
decomposition	2	3437(0.02286498227963873)	#SAKNAS!	#SAKNAS!
decorated	2	3437(0.02286498227963873)	#SAKNAS!	#SAKNAS!
decreases	2	1485(0.08002743797873557)	1516 (0.069)	2548 (0.033)
deep-sea	2	3437(0.02286498227963873)	2359 (0.034)	2548 (0.033)
defences	2	3437(0.02286498227963873)	2359 (0.034)	2548 (0.033)
deficient	2	3437(0.02286498227963873)	#SAKNAS!	#SAKNAS!
define	2	3437(0.02286498227963873)	#SAKNAS!	#SAKNAS!

defining	2	2214(0.04572996455927746)	1516 (0.069)	2548 (0.033)
definite	2	3437(0.02286498227963873)	1516 (0.069)	#SAKNAS!
definite	2	3437(0.02286498227963873)	1516 (0.069)	#SAKNAS!
definitions	2	2693(0.0342974734194581)	1516 (0.069)	#SAKNAS!
deglutition	2	3437(0.02286498227963873)	#SAKNAS!	2548 (0.033)
dehydrogenase	2	3437(0.02286498227963873)	#SAKNAS!	1668 (0.066)
delay	2	2214(0.04572996455927746)	1516 (0.069)	2548 (0.033)
delphi	2	3437(0.02286498227963873)	#SAKNAS!	2548 (0.033)
demand	2	3437(0.02286498227963873)	#SAKNAS!	1668 (0.066)
demand-feeding	2	3437(0.02286498227963873)	2359 (0.034)	2548 (0.033)
democratic	2	3437(0.02286498227963873)	#SAKNAS!	2548 (0.033)
demonstrate	2	3437(0.02286498227963873)	#SAKNAS!	2548 (0.033)
dentate	2	1219(0.1028924202583743)	1516 (0.069)	999 (0.13)
denticola	2	3437(0.02286498227963873)	2359 (0.034)	2548 (0.033)
deoxynivalenol	2	3437(0.02286498227963873)	2359 (0.034)	2548 (0.033)
departments	2	3437(0.02286498227963873)	#SAKNAS!	2548 (0.033)
depend	2	3437(0.02286498227963873)	2359 (0.034)	2548 (0.033)
depending	2	3437(0.02286498227963873)	#SAKNAS!	2548 (0.033)
depends	2	3437(0.02286498227963873)	1516 (0.069)	#SAKNAS!
depends	2	3437(0.02286498227963873)	1516 (0.069)	#SAKNAS!
depletion	2	2693(0.0342974734194581)	1516 (0.069)	#SAKNAS!
deposit	2	3437(0.02286498227963873)	2359 (0.034)	2548 (0.033)
depressive	2	1027(0.12575740253801304)	1516 (0.069)	816 (0.16)
dermal	2	3437(0.02286498227963873)	2359 (0.034)	2548 (0.033)
dermatitis	2	3437(0.02286498227963873)	#SAKNAS!	2548 (0.033)
dermatomyositis	2	3437(0.02286498227963873)	1516 (0.069)	#SAKNAS!
dermatomyositis	2	3437(0.02286498227963873)	1516 (0.069)	#SAKNAS!
desalivated	2	3437(0.02286498227963873)	1516 (0.069)	#SAKNAS!
desalivated	2	3437(0.02286498227963873)	1516 (0.069)	#SAKNAS!
description	2	1485(0.08002743797873557)	1516 (0.069)	999 (0.13)
desire	2	3437(0.02286498227963873)	#SAKNAS!	2548 (0.033)
destructive	2	2693(0.0342974734194581)	1516 (0.069)	#SAKNAS!
detection	2	1111(0.11432491139819366)	1516 (0.069)	584 (0.23)
determinant	2	3437(0.02286498227963873)	2359 (0.034)	2548 (0.033)
determines	2	3437(0.02286498227963873)	1516 (0.069)	#SAKNAS!
determines	2	3437(0.02286498227963873)	1516 (0.069)	#SAKNAS!
deterrent	2	3437(0.02286498227963873)	#SAKNAS!	1668 (0.066)
develop	2	2693(0.0342974734194581)	1516 (0.069)	#SAKNAS!
developments	2	3437(0.02286498227963873)	2359 (0.034)	#SAKNAS!
deviance	2	3437(0.02286498227963873)	#SAKNAS!	2548 (0.033)
d-fructose	2	3437(0.02286498227963873)	#SAKNAS!	1668 (0.066)
dha	2	3437(0.02286498227963873)	#SAKNAS!	1668 (0.066)
dhaka	2	3437(0.02286498227963873)	#SAKNAS!	2548 (0.033)

di	2	3437(0.0228649822796387З)	#SAKNAS!	1668 (0.066)
diagnoses	2	2693(0.0342974734194581)	1516 (0.069)	2548 (0.033)
diagnosing	2	2214(0.0457299645592774б)	1516 (0.069)	2548 (0.033)
diagnostic	2	896(0.14862238481765175)	1516 (0.069)	999 (0.13)
diaphragmatic	2	3437(0.0228649822796387З)	2359 (0.034)	#SAKNAS!
diaries	2	3437(0.0228649822796387З)	#SAKNAS!	1668 (0.066)
diatom	2	3437(0.0228649822796387З)	1516 (0.069)	#SAKNAS!
diatom	2	3437(0.0228649822796387З)	1516 (0.069)	#SAKNAS!
diatoms	2	3437(0.0228649822796387З)	1516 (0.069)	#SAKNAS!
diatoms	2	3437(0.0228649822796387З)	1516 (0.069)	#SAKNAS!
diego	2	3437(0.0228649822796387З)	#SAKNAS!	1668 (0.066)
diel	2	3437(0.0228649822796387З)	1516 (0.069)	#SAKNAS!
diel	2	3437(0.0228649822796387З)	1516 (0.069)	#SAKNAS!
dietary-intake	2	3437(0.0228649822796387З)	1516 (0.069)	#SAKNAS!
dietary-intake	2	3437(0.0228649822796387З)	1516 (0.069)	#SAKNAS!
dietitians	2	1027(0.12575740253801304)	1516 (0.069)	999 (0.13)
differentially	2	3437(0.0228649822796387З)	2359 (0.034)	2548 (0.033)
differently	2	3437(0.0228649822796387З)	2359 (0.034)	2548 (0.033)
digeorge	2	3437(0.0228649822796387З)	#SAKNAS!	1668 (0.066)
digestibility	2	628(0.21721733165656798)	1516 (0.069)	816 (0.16)
dimorphism	2	3437(0.0228649822796387З)	2359 (0.034)	2548 (0.033)
dinner	2	3437(0.0228649822796387З)	2359 (0.034)	2548 (0.033)
dinoflagellate	2	3437(0.0228649822796387З)	2359 (0.034)	2548 (0.033)
diomedea	2	3437(0.0228649822796387З)	1516 (0.069)	#SAKNAS!
diomedea	2	3437(0.0228649822796387З)	1516 (0.069)	#SAKNAS!
dioxide	2	3437(0.0228649822796387З)	#SAKNAS!	1668 (0.066)
diplodus	2	3437(0.0228649822796387З)	1516 (0.069)	#SAKNAS!
diplodus	2	3437(0.0228649822796387З)	1516 (0.069)	#SAKNAS!
direction	2	3437(0.0228649822796387З)	2359 (0.034)	2548 (0.033)
directly	2	3437(0.0228649822796387З)	#SAKNAS!	1668 (0.066)
disadvantage	2	3437(0.0228649822796387З)	2359 (0.034)	#SAKNAS!
discectomy	2	787(0.1714873670972905)	1516 (0.069)	816 (0.16)
discordant	2	3437(0.0228649822796387З)	1516 (0.069)	#SAKNAS!
discordant	2	3437(0.0228649822796387З)	1516 (0.069)	#SAKNAS!
discriminant	2	3437(0.0228649822796387З)	#SAKNAS!	1668 (0.066)
discriminate	2	3437(0.0228649822796387З)	#SAKNAS!	2548 (0.033)
discussion	2	3437(0.0228649822796387З)	1516 (0.069)	#SAKNAS!
discussion	2	3437(0.0228649822796387З)	1516 (0.069)	#SAKNAS!
disease-specific	2	2214(0.0457299645592774б)	1516 (0.069)	#SAKNAS!
displacement	2	3437(0.0228649822796387З)	#SAKNAS!	1668 (0.066)
displacements	2	3437(0.0228649822796387З)	2359 (0.034)	2548 (0.033)
distance	2	3437(0.0228649822796387З)	1516 (0.069)	#SAKNAS!
distance	2	3437(0.0228649822796387З)	1516 (0.069)	#SAKNAS!

distinct	2	1485(0.08002743797873557)	1516 (0.069)	1233 (0.1)
disturbances	2	2214(0.04572996455927746)	1516 (0.069)	1668 (0.066)
divergent	2	3437(0.02286498227963873)	#SAKNAS!	2548 (0.033)
diverticulum	2	1678(0.0685949468389162)	1516 (0.069)	1233 (0.1)
division	2	3437(0.02286498227963873)	1516 (0.069)	#SAKNAS!
division	2	3437(0.02286498227963873)	1516 (0.069)	#SAKNAS!
dna	2	3437(0.02286498227963873)	2359 (0.034)	2548 (0.033)
docosahexaenoic	2	3437(0.02286498227963873)	1516 (0.069)	#SAKNAS!
docosahexaenoic	2	3437(0.02286498227963873)	1516 (0.069)	#SAKNAS!
dogs	2	1894(0.05716245569909683)	1516 (0.069)	1233 (0.1)
dome	2	3437(0.02286498227963873)	#SAKNAS!	#SAKNAS!
dominant	2	3437(0.02286498227963873)	1516 (0.069)	#SAKNAS!
dominant	2	3437(0.02286498227963873)	1516 (0.069)	#SAKNAS!
dopamine	2	1678(0.0685949468389162)	1516 (0.069)	1668 (0.066)
dose	2	1485(0.08002743797873557)	1516 (0.069)	1668 (0.066)
dose-response	2	3437(0.02286498227963873)	#SAKNAS!	#SAKNAS!
double	2	1335(0.09145992911855493)	1516 (0.069)	999 (0.13)
dreissena	2	3437(0.02286498227963873)	2359 (0.034)	2548 (0.033)
drift-feeding	2	3437(0.02286498227963873)	#SAKNAS!	1668 (0.066)
driven	2	3437(0.02286498227963873)	#SAKNAS!	2548 (0.033)
drooling	2	3437(0.02286498227963873)	2359 (0.034)	2548 (0.033)
drosophilidae	2	3437(0.02286498227963873)	#SAKNAS!	1668 (0.066)
dual-energy	2	3437(0.02286498227963873)	2359 (0.034)	#SAKNAS!
dung	2	3437(0.02286498227963873)	#SAKNAS!	#SAKNAS!
duodenal	2	3437(0.02286498227963873)	2359 (0.034)	2548 (0.033)
duty	2	3437(0.02286498227963873)	#SAKNAS!	#SAKNAS!
dysarthria	2	3437(0.02286498227963873)	#SAKNAS!	1668 (0.066)
dysbiosis	2	3437(0.02286498227963873)	#SAKNAS!	#SAKNAS!
dyskinesia	2	3437(0.02286498227963873)	2359 (0.034)	2548 (0.033)
dysphagia-related	2	3437(0.02286498227963873)	#SAKNAS!	1668 (0.066)
dysphonia	2	3437(0.02286498227963873)	#SAKNAS!	1668 (0.066)
dysplasia	2	3437(0.02286498227963873)	2359 (0.034)	2548 (0.033)
dystonia	2	3437(0.02286498227963873)	2359 (0.034)	#SAKNAS!
dytiscidae	2	3437(0.02286498227963873)	2359 (0.034)	2548 (0.033)
dytiscus	2	3437(0.02286498227963873)	2359 (0.034)	2548 (0.033)
e2	2	3437(0.02286498227963873)	2359 (0.034)	#SAKNAS!
earlier	2	3437(0.02286498227963873)	2359 (0.034)	2548 (0.033)
earliest	2	3437(0.02286498227963873)	#SAKNAS!	1668 (0.066)
early-life	2	3437(0.02286498227963873)	2359 (0.034)	#SAKNAS!
early-phase	2	3437(0.02286498227963873)	#SAKNAS!	#SAKNAS!
east	2	1485(0.08002743797873557)	1516 (0.069)	1668 (0.066)
eat	2	787(0.1714873670972905)	1516 (0.069)	816 (0.16)
eaten	2	2693(0.0342974734194581)	1516 (0.069)	2548 (0.033)

ec	2	3437(0.0228649822796387З)	#SAKNAS!	1668 (0.066)
echinodermata	2	3437(0.0228649822796387З)	1516 (0.069)	#SAKNAS!
echinodermata	2	3437(0.0228649822796387З)	1516 (0.069)	#SAKNAS!
ecosystem	2	1678(0.0685949468389162)	1516 (0.069)	1233 (0.1)
ecosystems	2	3437(0.0228649822796387З)	1516 (0.069)	#SAKNAS!
ecosystems	2	3437(0.0228649822796387З)	1516 (0.069)	#SAKNAS!
ecuador	2	3437(0.0228649822796387З)	#SAKNAS!	2548 (0.033)
ecuadorian	2	3437(0.0228649822796387З)	#SAKNAS!	2548 (0.033)
e-delphi	2	3437(0.0228649822796387З)	#SAKNAS!	#SAKNAS!
edema	2	3437(0.0228649822796387З)	2359 (0.034)	#SAKNAS!
edentulousness	2	2693(0.0342974734194581)	1516 (0.069)	2548 (0.033)
edinburgh	2	3437(0.0228649822796387З)	2359 (0.034)	#SAKNAS!
edule	2	3437(0.0228649822796387З)	1516 (0.069)	#SAKNAS!
edule	2	3437(0.0228649822796387З)	1516 (0.069)	#SAKNAS!
edulis	2	3437(0.0228649822796387З)	1516 (0.069)	#SAKNAS!
edulis	2	3437(0.0228649822796387З)	1516 (0.069)	#SAKNAS!
egypt	2	3437(0.0228649822796387З)	#SAKNAS!	#SAKNAS!
elastase	2	3437(0.0228649822796387З)	2359 (0.034)	#SAKNAS!
elder	2	3437(0.0228649822796387З)	2359 (0.034)	#SAKNAS!
elder-friendly	2	3437(0.0228649822796387З)	#SAKNAS!	#SAKNAS!
elective	2	1485(0.08002743797873557)	1516 (0.069)	1668 (0.066)
electroencephalography	2	3437(0.0228649822796387З)	#SAKNAS!	#SAKNAS!
electromyography	2	2214(0.04572996455927746)	1516 (0.069)	2548 (0.033)
elephant	2	2214(0.04572996455927746)	1516 (0.069)	1668 (0.066)
eligible	2	3437(0.0228649822796387З)	#SAKNAS!	#SAKNAS!
eliminating	2	3437(0.0228649822796387З)	1516 (0.069)	#SAKNAS!
eliminating	2	3437(0.0228649822796387З)	1516 (0.069)	#SAKNAS!
elimination	2	3437(0.0228649822796387З)	2359 (0.034)	#SAKNAS!
emerald	2	3437(0.0228649822796387З)	1516 (0.069)	#SAKNAS!
emerald	2	3437(0.0228649822796387З)	1516 (0.069)	#SAKNAS!
emerging	2	1894(0.0571624556990968З)	1516 (0.069)	2548 (0.033)
emission	2	2214(0.04572996455927746)	1516 (0.069)	#SAKNAS!
emphasis	2	3437(0.0228649822796387З)	1516 (0.069)	#SAKNAS!
emphasis	2	3437(0.0228649822796387З)	1516 (0.069)	#SAKNAS!
empirical	2	3437(0.0228649822796387З)	#SAKNAS!	#SAKNAS!
encephalopathy	2	2693(0.0342974734194581)	1516 (0.069)	#SAKNAS!
endocrinological	2	3437(0.0228649822796387З)	#SAKNAS!	#SAKNAS!
end-of-life	2	3437(0.0228649822796387З)	2359 (0.034)	2548 (0.033)
endogeic	2	3437(0.0228649822796387З)	#SAKNAS!	1668 (0.066)
endogenous	2	3437(0.0228649822796387З)	1516 (0.069)	#SAKNAS!
endogenous	2	3437(0.0228649822796387З)	1516 (0.069)	#SAKNAS!
endoluminal	2	3437(0.0228649822796387З)	1516 (0.069)	#SAKNAS!
endoluminal	2	3437(0.0228649822796387З)	1516 (0.069)	#SAKNAS!

endoparasitoid	2	3437(0.0228649822796387 3)	2359 (0.034)	2548 (0.033)
endoscopically	2	3437(0.02286498227963873)	1516 (0.069)	#SAKNAS!
endoscopically	2	3437(0.02286498227963873)	1516 (0.069)	#SAKNAS!
end-stage	2	1485(0.08002743797873557)	1516 (0.069)	1233 (0.1)
endurance	2	3437(0.02286498227963873)	#SAKNAS!	1668 (0.066)
energetic	2	1485(0.08002743797873557)	1516 (0.069)	999 (0.13)
engaging	2	3437(0.02286498227963873)	2359 (0.034)	2548 (0.033)
english	2	2214(0.04572996455927746)	1516 (0.069)	1668 (0.066)
enrichment	2	3437(0.02286498227963873)	#SAKNAS!	1668 (0.066)
ensure	2	3437(0.02286498227963873)	#SAKNAS!	1668 (0.066)
enterica	2	3437(0.02286498227963873)	2359 (0.034)	#SAKNAS!
environmentally	2	3437(0.02286498227963873)	#SAKNAS!	#SAKNAS!
environments	2	1485(0.08002743797873557)	1516 (0.069)	1668 (0.066)
enzyme	2	1894(0.05716245569909683)	1516 (0.069)	1233 (0.1)
enzymes	2	3437(0.02286498227963873)	#SAKNAS!	1668 (0.066)
eoe	2	3437(0.02286498227963873)	#SAKNAS!	#SAKNAS!
eortc	2	3437(0.02286498227963873)	2359 (0.034)	2548 (0.033)
epidemic	2	3437(0.02286498227963873)	#SAKNAS!	#SAKNAS!
epidemiologic	2	2214(0.04572996455927746)	1516 (0.069)	1668 (0.066)
epidemiological	2	628(0.21721733165656798)	1516 (0.069)	461 (0.3)
epidermal	2	3437(0.02286498227963873)	2359 (0.034)	#SAKNAS!
epidermolysis-bullosa	2	3437(0.02286498227963873)	1516 (0.069)	#SAKNAS!
epidermolysis-bullosa	2	3437(0.02286498227963873)	1516 (0.069)	#SAKNAS!
epigenetics	2	3437(0.02286498227963873)	#SAKNAS!	#SAKNAS!
epiphrenic	2	3437(0.02286498227963873)	2359 (0.034)	2548 (0.033)
era	2	3437(0.02286498227963873)	2359 (0.034)	2548 (0.033)
erectus	2	3437(0.02286498227963873)	2359 (0.034)	2548 (0.033)
eriocheir	2	3437(0.02286498227963873)	#SAKNAS!	#SAKNAS!
erythrocyte	2	3437(0.02286498227963873)	2359 (0.034)	#SAKNAS!
esophagocoloplasty	2	3437(0.02286498227963873)	2359 (0.034)	2548 (0.033)
esophagogastric	2	1894(0.05716245569909683)	1516 (0.069)	#SAKNAS!
esophago-gastric	2	3437(0.02286498227963873)	1516 (0.069)	#SAKNAS!
esophago-gastric	2	3437(0.02286498227963873)	1516 (0.069)	#SAKNAS!
esrd	2	3437(0.02286498227963873)	1516 (0.069)	#SAKNAS!
esrd	2	3437(0.02286498227963873)	1516 (0.069)	#SAKNAS!
establishment	2	3437(0.02286498227963873)	#SAKNAS!	2548 (0.033)
estimating	2	1111(0.11432491139819366)	1516 (0.069)	584 (0.23)
estradiol	2	3437(0.02286498227963873)	2359 (0.034)	#SAKNAS!
estrogen	2	3437(0.02286498227963873)	1516 (0.069)	#SAKNAS!
estrogen	2	3437(0.02286498227963873)	1516 (0.069)	#SAKNAS!
ethical	2	2693(0.0342974734194581)	1516 (0.069)	2548 (0.033)
etv5	2	3437(0.02286498227963873)	2359 (0.034)	2548 (0.033)
eutrophic	2	3437(0.02286498227963873)	2359 (0.034)	#SAKNAS!

event	2	3437(0.0228649822796387З)	#SAKNAS!	2548 (0.033)
every	2	3437(0.0228649822796387З)	2359 (0.034)	#SAKNAS!
exacerbations	2	3437(0.0228649822796387З)	2359 (0.034)	#SAKNAS!
exchange	2	3437(0.0228649822796387З)	2359 (0.034)	#SAKNAS!
exclusion	2	3437(0.0228649822796387З)	#SAKNAS!	1668 (0.066)
excreta	2	3437(0.0228649822796387З)	#SAKNAS!	1668 (0.066)
excretion	2	1894(0.05716245569909683)	1516 (0.069)	1233 (0.1)
expectations	2	2214(0.04572996455927746)	1516 (0.069)	1668 (0.066)
expenditures	2	3437(0.0228649822796387З)	2359 (0.034)	2548 (0.033)
experiences	2	836(0.16005487595747114)	1516 (0.069)	999 (0.13)
expert	2	1678(0.0685949468389162)	1516 (0.069)	999 (0.13)
expiratory	2	3437(0.0228649822796387З)	#SAKNAS!	2548 (0.033)
explaining	2	3437(0.0228649822796387З)	2359 (0.034)	#SAKNAS!
exploitation	2	3437(0.0228649822796387З)	#SAKNAS!	2548 (0.033)
extension	2	3437(0.0228649822796387З)	#SAKNAS!	2548 (0.033)
extracellular	2	3437(0.0228649822796387З)	#SAKNAS!	1668 (0.066)
extraction	2	3437(0.0228649822796387З)	1516 (0.069)	#SAKNAS!
extraction	2	3437(0.0228649822796387З)	1516 (0.069)	#SAKNAS!
extreme	2	3437(0.0228649822796387З)	1516 (0.069)	#SAKNAS!
extreme	2	3437(0.0228649822796387З)	1516 (0.069)	#SAKNAS!
extubation	2	3437(0.0228649822796387З)	#SAKNAS!	#SAKNAS!
f1	2	3437(0.0228649822796387З)	#SAKNAS!	1668 (0.066)
fabricius	2	3437(0.0228649822796387З)	1516 (0.069)	#SAKNAS!
fabricius	2	3437(0.0228649822796387З)	1516 (0.069)	#SAKNAS!
facial	2	1894(0.05716245569909683)	1516 (0.069)	2548 (0.033)
facilitates	2	2693(0.0342974734194581)	1516 (0.069)	2548 (0.033)
facilitating	2	3437(0.0228649822796387З)	1516 (0.069)	#SAKNAS!
facilitating	2	3437(0.0228649822796387З)	1516 (0.069)	#SAKNAS!
facilities	2	559(0.2400823139362067)	1516 (0.069)	694 (0.2)
facility	2	1894(0.05716245569909683)	1516 (0.069)	#SAKNAS!
faecal	2	2693(0.0342974734194581)	1516 (0.069)	2548 (0.033)
faisal	2	3437(0.0228649822796387З)	#SAKNAS!	2548 (0.033)
fall	2	3437(0.0228649822796387З)	#SAKNAS!	2548 (0.033)
fallow	2	3437(0.0228649822796387З)	1516 (0.069)	#SAKNAS!
fallow	2	3437(0.0228649822796387З)	1516 (0.069)	#SAKNAS!
far	2	3437(0.0228649822796387З)	#SAKNAS!	2548 (0.033)
farm	2	2693(0.0342974734194581)	1516 (0.069)	#SAKNAS!
farmed	2	3437(0.0228649822796387З)	1516 (0.069)	#SAKNAS!
farmed	2	3437(0.0228649822796387З)	1516 (0.069)	#SAKNAS!
farmers	2	3437(0.0228649822796387З)	#SAKNAS!	2548 (0.033)
faster	2	3437(0.0228649822796387З)	#SAKNAS!	2548 (0.033)
fathers	2	3437(0.0228649822796387З)	#SAKNAS!	1668 (0.066)
fatigue	2	3437(0.0228649822796387З)	#SAKNAS!	#SAKNAS!

fattening	2	3437(0.0228649822796387З)	1516 (0.069)	#SAKNAS!
fattening	2	3437(0.02286498227963873)	1516 (0.069)	#SAKNAS!
favor	2	3437(0.02286498227963873)	2359 (0.034)	2548 (0.033)
favorable	2	3437(0.02286498227963873)	#SAKNAS!	2548 (0.033)
fear	2	3437(0.02286498227963873)	#SAKNAS!	2548 (0.033)
feature	2	3437(0.02286498227963873)	#SAKNAS!	2548 (0.033)
features	2	787(0.1714873670972905)	1516 (0.069)	816 (0.16)
feces	2	3437(0.02286498227963873)	1516 (0.069)	#SAKNAS!
feces	2	3437(0.02286498227963873)	1516 (0.069)	#SAKNAS!
feedback	2	3437(0.02286498227963873)	2359 (0.034)	2548 (0.033)
feeders	2	2693(0.0342974734194581)	1516 (0.069)	2548 (0.033)
feedings	2	3437(0.02286498227963873)	2359 (0.034)	2548 (0.033)
feeding-tube	2	3437(0.02286498227963873)	#SAKNAS!	#SAKNAS!
feline	2	3437(0.02286498227963873)	#SAKNAS!	#SAKNAS!
femoralis	2	3437(0.02286498227963873)	#SAKNAS!	#SAKNAS!
fenugreek	2	3437(0.02286498227963873)	1516 (0.069)	#SAKNAS!
fenugreek	2	3437(0.02286498227963873)	1516 (0.069)	#SAKNAS!
feral	2	3437(0.02286498227963873)	1516 (0.069)	#SAKNAS!
feral	2	3437(0.02286498227963873)	1516 (0.069)	#SAKNAS!
fertile	2	3437(0.02286498227963873)	#SAKNAS!	1668 (0.066)
fertility	2	3437(0.02286498227963873)	#SAKNAS!	1668 (0.066)
fetal	2	1485(0.08002743797873557)	1516 (0.069)	999 (0.13)
fewer	2	3437(0.02286498227963873)	#SAKNAS!	1668 (0.066)
fgf21	2	3437(0.02286498227963873)	#SAKNAS!	#SAKNAS!
fibrin	2	3437(0.02286498227963873)	2359 (0.034)	#SAKNAS!
fibrinogen	2	2693(0.0342974734194581)	1516 (0.069)	2548 (0.033)
fibromyalgia	2	3437(0.02286498227963873)	#SAKNAS!	#SAKNAS!
fibrosis	2	1111(0.11432491139819366)	1516 (0.069)	2548 (0.033)
filter-feeding	2	3437(0.02286498227963873)	1516 (0.069)	#SAKNAS!
filter-feeding	2	3437(0.02286498227963873)	1516 (0.069)	#SAKNAS!
finishing	2	1111(0.11432491139819366)	1516 (0.069)	#SAKNAS!
fir	2	3437(0.02286498227963873)	1516 (0.069)	#SAKNAS!
fir	2	3437(0.02286498227963873)	1516 (0.069)	#SAKNAS!
first-degree	2	3437(0.02286498227963873)	#SAKNAS!	1668 (0.066)
first-grade	2	3437(0.02286498227963873)	1516 (0.069)	#SAKNAS!
first-grade	2	3437(0.02286498227963873)	1516 (0.069)	#SAKNAS!
first-time	2	3437(0.02286498227963873)	2359 (0.034)	2548 (0.033)
fishes	2	3437(0.02286498227963873)	1516 (0.069)	#SAKNAS!
fishes	2	3437(0.02286498227963873)	1516 (0.069)	#SAKNAS!
fishmeal	2	3437(0.02286498227963873)	2359 (0.034)	#SAKNAS!
fistula	2	1335(0.09145992911855493)	1516 (0.069)	999 (0.13)
fit	2	3437(0.02286498227963873)	2359 (0.034)	#SAKNAS!
fits	2	3437(0.02286498227963873)	2359 (0.034)	2548 (0.033)

fl	2	3437(0.0228649822796387З)	#SAKNAS!	#SAKNAS!
flame	2	3437(0.0228649822796387З)	#SAKNAS!	#SAKNAS!
flaps	2	2693(0.0342974734194581)	1516 (0.069)	2548 (0.033)
flexibility	2	2693(0.0342974734194581)	1516 (0.069)	2548 (0.033)
flora	2	3437(0.0228649822796387З)	2359 (0.034)	#SAKNAS!
floral	2	3437(0.0228649822796387З)	1516 (0.069)	#SAKNAS!
floral	2	3437(0.0228649822796387З)	1516 (0.069)	#SAKNAS!
flour	2	3437(0.0228649822796387З)	2359 (0.034)	#SAKNAS!
flowering	2	3437(0.0228649822796387З)	#SAKNAS!	1668 (0.066)
fluctuations	2	3437(0.0228649822796387З)	1516 (0.069)	#SAKNAS!
fluctuations	2	3437(0.0228649822796387З)	1516 (0.069)	#SAKNAS!
fluids	2	1894(0.0571624556990968З)	1516 (0.069)	2548 (0.033)
fluoridation	2	1894(0.0571624556990968З)	1516 (0.069)	1233 (0.1)
fluoroscopic	2	3437(0.0228649822796387З)	2359 (0.034)	#SAKNAS!
fluoroscopically	2	3437(0.0228649822796387З)	1516 (0.069)	#SAKNAS!
fluoroscopically	2	3437(0.0228649822796387З)	1516 (0.069)	#SAKNAS!
fluoroscopy	2	3437(0.0228649822796387З)	2359 (0.034)	#SAKNAS!
fluorosis	2	1678(0.0685949468389162)	1516 (0.069)	1668 (0.066)
fluviatilis	2	3437(0.0228649822796387З)	1516 (0.069)	#SAKNAS!
fluviatilis	2	3437(0.0228649822796387З)	1516 (0.069)	#SAKNAS!
fly	2	3437(0.0228649822796387З)	#SAKNAS!	#SAKNAS!
focal	2	3437(0.0228649822796387З)	#SAKNAS!	2548 (0.033)
fodder	2	3437(0.0228649822796387З)	#SAKNAS!	2548 (0.033)
folate	2	2214(0.0457299645592774б)	1516 (0.069)	#SAKNAS!
fold	2	3437(0.0228649822796387З)	1516 (0.069)	#SAKNAS!
fold	2	3437(0.0228649822796387З)	1516 (0.069)	#SAKNAS!
foliage	2	2693(0.0342974734194581)	1516 (0.069)	#SAKNAS!
folivore	2	3437(0.0228649822796387З)	2359 (0.034)	2548 (0.033)
folivorous	2	3437(0.0228649822796387З)	2359 (0.034)	2548 (0.033)
food-consumption	2	3437(0.0228649822796387З)	1516 (0.069)	#SAKNAS!
food-consumption	2	3437(0.0228649822796387З)	1516 (0.069)	#SAKNAS!
food-deprivation	2	3437(0.0228649822796387З)	1516 (0.069)	#SAKNAS!
food-deprivation	2	3437(0.0228649822796387З)	1516 (0.069)	#SAKNAS!
forager	2	3437(0.0228649822796387З)	2359 (0.034)	2548 (0.033)
forceps	2	3437(0.0228649822796387З)	#SAKNAS!	#SAKNAS!
forearm	2	3437(0.0228649822796387З)	1516 (0.069)	#SAKNAS!
forearm	2	3437(0.0228649822796387З)	1516 (0.069)	#SAKNAS!
foregut	2	3437(0.0228649822796387З)	#SAKNAS!	1668 (0.066)
foreign	2	3437(0.0228649822796387З)	2359 (0.034)	2548 (0.033)
forgotten	2	3437(0.0228649822796387З)	#SAKNAS!	2548 (0.033)
forms	2	2214(0.0457299645592774б)	1516 (0.069)	1668 (0.066)
formulation	2	3437(0.0228649822796387З)	#SAKNAS!	#SAKNAS!
forsythia	2	2693(0.0342974734194581)	1516 (0.069)	#SAKNAS!

fossil	2	3437(0.0228649822796387З)	2359 (0.034)	2548 (0.033)
four-year-old	2	3437(0.0228649822796387З)	1516 (0.069)	#SAKNAS!
four-year-old	2	3437(0.0228649822796387З)	1516 (0.069)	#SAKNAS!
frailty	2	143(0.8574368354864526)	1516 (0.069)	174 (0.76)
france	2	3437(0.0228649822796387З)	1516 (0.069)	#SAKNAS!
france	2	3437(0.0228649822796387З)	1516 (0.069)	#SAKNAS!
fresh	2	1678(0.0685949468389162)	1516 (0.069)	1668 (0.066)
freshmen	2	3437(0.0228649822796387З)	2359 (0.034)	#SAKNAS!
frontiers	2	3437(0.0228649822796387З)	#SAKNAS!	#SAKNAS!
fto	2	1894(0.0571624556990968З)	1516 (0.069)	1233 (0.1)
fulvus	2	3437(0.0228649822796387З)	1516 (0.069)	#SAKNAS!
fulvus	2	3437(0.0228649822796387З)	1516 (0.069)	#SAKNAS!
fungal	2	1894(0.0571624556990968З)	1516 (0.069)	#SAKNAS!
fur	2	3437(0.0228649822796387З)	1516 (0.069)	#SAKNAS!
fur	2	3437(0.0228649822796387З)	1516 (0.069)	#SAKNAS!
fuscata	2	2693(0.0342974734194581)	1516 (0.069)	2548 (0.033)
fusion	2	559(0.2400823139362067)	1516 (0.069)	584 (0.23)
fusobacterium	2	2693(0.0342974734194581)	1516 (0.069)	2548 (0.033)
futures	2	3437(0.0228649822796387З)	#SAKNAS!	2548 (0.033)
gains	2	3437(0.0228649822796387З)	2359 (0.034)	#SAKNAS!
galapagos	2	3437(0.0228649822796387З)	2359 (0.034)	2548 (0.033)
gall-inducing	2	3437(0.0228649822796387З)	2359 (0.034)	2548 (0.033)
gaps	2	3437(0.0228649822796387З)	2359 (0.034)	#SAKNAS!
gastroduodenal	2	3437(0.0228649822796387З)	2359 (0.034)	2548 (0.033)
gastro-esophageal	2	1894(0.0571624556990968З)	1516 (0.069)	2548 (0.033)
gastro-intestinal	2	2214(0.04572996455927746)	1516 (0.069)	2548 (0.033)
gastrooesophageal	2	3437(0.0228649822796387З)	2359 (0.034)	#SAKNAS!
gastroparesis	2	3437(0.0228649822796387З)	#SAKNAS!	2548 (0.033)
gastropexy	2	3437(0.0228649822796387З)	2359 (0.034)	#SAKNAS!
gelatinous	2	3437(0.0228649822796387З)	2359 (0.034)	2548 (0.033)
gene-environment	2	3437(0.0228649822796387З)	#SAKNAS!	1668 (0.066)
generates	2	3437(0.0228649822796387З)	2359 (0.034)	2548 (0.033)
genetics	2	1894(0.0571624556990968З)	1516 (0.069)	1668 (0.066)
genome-wide	2	3437(0.0228649822796387З)	#SAKNAS!	2548 (0.033)
genus	2	2693(0.0342974734194581)	1516 (0.069)	2548 (0.033)
geometric	2	3437(0.0228649822796387З)	#SAKNAS!	2548 (0.033)
geometry	2	3437(0.0228649822796387З)	1516 (0.069)	#SAKNAS!
geometry	2	3437(0.0228649822796387З)	1516 (0.069)	#SAKNAS!
gestating	2	3437(0.0228649822796387З)	#SAKNAS!	#SAKNAS!
ghats	2	3437(0.0228649822796387З)	#SAKNAS!	#SAKNAS!
gianturco	2	3437(0.0228649822796387З)	1516 (0.069)	#SAKNAS!
gianturco	2	3437(0.0228649822796387З)	1516 (0.069)	#SAKNAS!
gift	2	3437(0.0228649822796387З)	#SAKNAS!	2548 (0.033)

gigas	2	2693(0.0342974734194581)	1516 (0.069)	2548 (0.033)
gingiva	2	3437(0.0228649822796387З)	1516 (0.069)	#SAKNAS!
gingiva	2	3437(0.0228649822796387З)	1516 (0.069)	#SAKNAS!
giraffe	2	3437(0.0228649822796387З)	2359 (0.034)	2548 (0.033)
girls	2	1678(0.0685949468389162)	1516 (0.069)	1233 (0.1)
gl	2	3437(0.0228649822796387З)	#SAKNAS!	2548 (0.033)
glossina-pallidipes	2	3437(0.0228649822796387З)	1516 (0.069)	#SAKNAS!
glossina-pallidipes	2	3437(0.0228649822796387З)	1516 (0.069)	#SAKNAS!
glucagon-like	2	3437(0.0228649822796387З)	#SAKNAS!	2548 (0.033)
glucomannan	2	3437(0.0228649822796387З)	#SAKNAS!	#SAKNAS!
glutamatergic	2	3437(0.0228649822796387З)	2359 (0.034)	2548 (0.033)
glutathione	2	3437(0.0228649822796387З)	1516 (0.069)	#SAKNAS!
glutathione	2	3437(0.0228649822796387З)	1516 (0.069)	#SAKNAS!
glycemic	2	787(0.1714873670972905)	1516 (0.069)	999 (0.13)
glycol	2	3437(0.0228649822796387З)	1516 (0.069)	#SAKNAS!
glycol	2	3437(0.0228649822796387З)	1516 (0.069)	#SAKNAS!
go	2	3437(0.0228649822796387З)	#SAKNAS!	2548 (0.033)
godart	2	3437(0.0228649822796387З)	1516 (0.069)	#SAKNAS!
godart	2	3437(0.0228649822796387З)	1516 (0.069)	#SAKNAS!
going	2	3437(0.0228649822796387З)	#SAKNAS!	2548 (0.033)
goldfish	2	2693(0.0342974734194581)	1516 (0.069)	2548 (0.033)
gorilla	2	2693(0.0342974734194581)	1516 (0.069)	2548 (0.033)
gorillas	2	2214(0.0457299645592774б)	1516 (0.069)	2548 (0.033)
gothenburg	2	3437(0.0228649822796387З)	2359 (0.034)	2548 (0.033)
government	2	3437(0.0228649822796387З)	2359 (0.034)	#SAKNAS!
grade	2	1335(0.0914599291185549З)	1516 (0.069)	1668 (0.066)
graft	2	3437(0.0228649822796387З)	#SAKNAS!	1668 (0.066)
graft-versus-host	2	3437(0.0228649822796387З)	1516 (0.069)	#SAKNAS!
graft-versus-host	2	3437(0.0228649822796387З)	1516 (0.069)	#SAKNAS!
grains	2	2693(0.0342974734194581)	1516 (0.069)	#SAKNAS!
granulocyte-macrophage	2	3437(0.0228649822796387З)	1516 (0.069)	#SAKNAS!
granulocyte-macrophage	2	3437(0.0228649822796387З)	1516 (0.069)	#SAKNAS!
graph	2	3437(0.0228649822796387З)	#SAKNAS!	1668 (0.066)
grasshoppers	2	2214(0.0457299645592774б)	1516 (0.069)	#SAKNAS!
gravis	2	1894(0.0571624556990968З)	1516 (0.069)	#SAKNAS!
greater	2	1678(0.0685949468389162)	1516 (0.069)	999 (0.13)
gregaria	2	3437(0.0228649822796387З)	2359 (0.034)	2548 (0.033)
greylag	2	3437(0.0228649822796387З)	1516 (0.069)	#SAKNAS!
greylag	2	3437(0.0228649822796387З)	1516 (0.069)	#SAKNAS!
grocery	2	3437(0.0228649822796387З)	1516 (0.069)	#SAKNAS!
grocery	2	3437(0.0228649822796387З)	1516 (0.069)	#SAKNAS!
ground	2	2693(0.0342974734194581)	1516 (0.069)	#SAKNAS!
grown	2	3437(0.0228649822796387З)	2359 (0.034)	#SAKNAS!

gugging	2	3437(0.0228649822796387З)	2359 (0.034)	#SAKNAS!
guide	2	1678(0.0685949468389162)	1516 (0.069)	999 (0.13)
guillain-barre	2	3437(0.0228649822796387З)	#SAKNAS!	2548 (0.033)
guilt	2	3437(0.0228649822796387З)	#SAKNAS!	2548 (0.033)
gullah	2	3437(0.0228649822796387З)	1516 (0.069)	#SAKNAS!
gullah	2	3437(0.0228649822796387З)	1516 (0.069)	#SAKNAS!
gum-chewing	2	3437(0.0228649822796387З)	#SAKNAS!	#SAKNAS!
gustatory	2	1219(0.1028924202583743)	1516 (0.069)	584 (0.23)
habit	2	1111(0.11432491139819366)	1516 (0.069)	1233 (0.1)
habitual	2	3437(0.0228649822796387З)	2359 (0.034)	#SAKNAS!
haematological	2	3437(0.0228649822796387З)	2359 (0.034)	2548 (0.033)
haemodialysis	2	3437(0.0228649822796387З)	#SAKNAS!	#SAKNAS!
haitian	2	3437(0.0228649822796387З)	1516 (0.069)	#SAKNAS!
haitian	2	3437(0.0228649822796387З)	1516 (0.069)	#SAKNAS!
half	2	3437(0.0228649822796387З)	1516 (0.069)	#SAKNAS!
half	2	3437(0.0228649822796387З)	1516 (0.069)	#SAKNAS!
haliotis	2	3437(0.0228649822796387З)	2359 (0.034)	2548 (0.033)
halitosis	2	3437(0.0228649822796387З)	#SAKNAS!	1668 (0.066)
han	2	3437(0.0228649822796387З)	#SAKNAS!	1668 (0.066)
hand-oral	2	3437(0.0228649822796387З)	1516 (0.069)	#SAKNAS!
hand-oral	2	3437(0.0228649822796387З)	1516 (0.069)	#SAKNAS!
hard	2	3437(0.0228649822796387З)	2359 (0.034)	2548 (0.033)
hardness	2	3437(0.0228649822796387З)	#SAKNAS!	2548 (0.033)
harmful	2	3437(0.0228649822796387З)	#SAKNAS!	1668 (0.066)
haw	2	3437(0.0228649822796387З)	#SAKNAS!	1668 (0.066)
heartburn	2	3437(0.0228649822796387З)	2359 (0.034)	#SAKNAS!
heating	2	3437(0.0228649822796387З)	#SAKNAS!	#SAKNAS!
heller	2	1219(0.1028924202583743)	1516 (0.069)	694 (0.2)
helsinki	2	3437(0.0228649822796387З)	#SAKNAS!	1668 (0.066)
hematoma	2	3437(0.0228649822796387З)	2359 (0.034)	#SAKNAS!
hemiptera	2	966(0.1371898936778324)	1516 (0.069)	584 (0.23)
hemolymph	2	3437(0.0228649822796387З)	2359 (0.034)	2548 (0.033)
hepatic	2	2214(0.04572996455927746)	1516 (0.069)	#SAKNAS!
herbal	2	3437(0.0228649822796387З)	#SAKNAS!	1668 (0.066)
herbivores	2	2693(0.0342974734194581)	1516 (0.069)	#SAKNAS!
hermetia	2	3437(0.0228649822796387З)	#SAKNAS!	#SAKNAS!
herpes	2	3437(0.0228649822796387З)	2359 (0.034)	#SAKNAS!
heterotrophic	2	2693(0.0342974734194581)	1516 (0.069)	2548 (0.033)
hexa	2	3437(0.0228649822796387З)	#SAKNAS!	1668 (0.066)
hiatal	2	595(0.22864982279638732)	1516 (0.069)	526 (0.26)
high-energy	2	3437(0.0228649822796387З)	#SAKNAS!	1668 (0.066)
high-fiber	2	2693(0.0342974734194581)	1516 (0.069)	2548 (0.033)
high-fibre	2	3437(0.0228649822796387З)	1516 (0.069)	#SAKNAS!

high-fibre	2	3437(0.0228649822796387З)	1516 (0.069)	#SAKNAS!
highlights	2	3437(0.02286498227963873)	2359 (0.034)	#SAKNAS!
high-pressure	2	3437(0.02286498227963873)	#SAKNAS!	#SAKNAS!
hinds	2	3437(0.02286498227963873)	1516 (0.069)	#SAKNAS!
hinds	2	3437(0.02286498227963873)	1516 (0.069)	#SAKNAS!
hircus	2	3437(0.02286498227963873)	1516 (0.069)	#SAKNAS!
hircus	2	3437(0.02286498227963873)	1516 (0.069)	#SAKNAS!
hisayama	2	3437(0.02286498227963873)	2359 (0.034)	2548 (0.033)
hispanics	2	3437(0.02286498227963873)	2359 (0.034)	2548 (0.033)
histology	2	3437(0.02286498227963873)	2359 (0.034)	2548 (0.033)
historical	2	3437(0.02286498227963873)	2359 (0.034)	2548 (0.033)
holstein-friesian	2	3437(0.02286498227963873)	#SAKNAS!	2548 (0.033)
homebound	2	3437(0.02286498227963873)	2359 (0.034)	2548 (0.033)
home-care	2	3437(0.02286498227963873)	#SAKNAS!	1668 (0.066)
hominin	2	3437(0.02286498227963873)	#SAKNAS!	2548 (0.033)
homocysteine	2	2693(0.0342974734194581)	1516 (0.069)	#SAKNAS!
homoptera	2	3437(0.02286498227963873)	1516 (0.069)	#SAKNAS!
homoptera	2	3437(0.02286498227963873)	1516 (0.069)	#SAKNAS!
honeydew	2	3437(0.02286498227963873)	1516 (0.069)	#SAKNAS!
honeydew	2	3437(0.02286498227963873)	1516 (0.069)	#SAKNAS!
hong	2	1485(0.08002743797873557)	1516 (0.069)	816 (0.16)
horizontal	2	3437(0.02286498227963873)	#SAKNAS!	2548 (0.033)
hormones	2	1335(0.09145992911855493)	1516 (0.069)	1233 (0.1)
hornworm	2	3437(0.02286498227963873)	2359 (0.034)	2548 (0.033)
hospital-associated	2	3437(0.02286498227963873)	#SAKNAS!	1668 (0.066)
hospitalization	2	743(0.18291985823710985)	1516 (0.069)	584 (0.23)
host-feeding	2	3437(0.02286498227963873)	1516 (0.069)	#SAKNAS!
host-feeding	2	3437(0.02286498227963873)	1516 (0.069)	#SAKNAS!
hotspots	2	3437(0.02286498227963873)	#SAKNAS!	#SAKNAS!
household	2	1111(0.11432491139819366)	1516 (0.069)	1668 (0.066)
howler	2	3437(0.02286498227963873)	#SAKNAS!	1668 (0.066)
huemul	2	3437(0.02286498227963873)	1516 (0.069)	#SAKNAS!
huemul	2	3437(0.02286498227963873)	1516 (0.069)	#SAKNAS!
humidity	2	3437(0.02286498227963873)	2359 (0.034)	#SAKNAS!
hunting	2	3437(0.02286498227963873)	2359 (0.034)	#SAKNAS!
hydrolysates	2	3437(0.02286498227963873)	#SAKNAS!	1668 (0.066)
hyoid	2	2214(0.04572996455927746)	1516 (0.069)	1668 (0.066)
hyperborea	2	3437(0.02286498227963873)	1516 (0.069)	#SAKNAS!
hyperborea	2	3437(0.02286498227963873)	1516 (0.069)	#SAKNAS!
hypercaloric	2	3437(0.02286498227963873)	2359 (0.034)	#SAKNAS!
hyperparathyroidism	2	3437(0.02286498227963873)	1516 (0.069)	#SAKNAS!
hyperparathyroidism	2	3437(0.02286498227963873)	1516 (0.069)	#SAKNAS!
hypertriglyceridemia	2	3437(0.02286498227963873)	2359 (0.034)	#SAKNAS!

hypoglossal	2	3437(0.0228649822796387З)	#SAKNAS!	2548 (0.033)
hypophosphatemia	2	3437(0.02286498227963873)	1516 (0.069)	#SAKNAS!
hypophosphatemia	2	3437(0.02286498227963873)	1516 (0.069)	#SAKNAS!
hypoplasia	2	2214(0.04572996455927746)	1516 (0.069)	2548 (0.033)
hyposalivation	2	1485(0.08002743797873557)	1516 (0.069)	999 (0.13)
hypothetical	2	3437(0.02286498227963873)	#SAKNAS!	2548 (0.033)
iatrogenic	2	3437(0.02286498227963873)	2359 (0.034)	2548 (0.033)
icdas	2	3437(0.02286498227963873)	#SAKNAS!	1668 (0.066)
identifies	2	3437(0.02286498227963873)	#SAKNAS!	2548 (0.033)
identify	2	1485(0.08002743797873557)	1516 (0.069)	2548 (0.033)
identities	2	3437(0.02286498227963873)	#SAKNAS!	1668 (0.066)
idiopathic	2	1485(0.08002743797873557)	1516 (0.069)	1668 (0.066)
igg	2	3437(0.02286498227963873)	2359 (0.034)	2548 (0.033)
ilembula	2	3437(0.02286498227963873)	#SAKNAS!	#SAKNAS!
illness	2	1894(0.05716245569909683)	1516 (0.069)	2548 (0.033)
illucens	2	3437(0.02286498227963873)	#SAKNAS!	#SAKNAS!
imaging	2	1485(0.08002743797873557)	1516 (0.069)	1233 (0.1)
immigrant	2	1894(0.05716245569909683)	1516 (0.069)	1668 (0.066)
immigration	2	3437(0.02286498227963873)	#SAKNAS!	2548 (0.033)
immune	2	1111(0.11432491139819366)	1516 (0.069)	1668 (0.066)
immunological	2	2214(0.04572996455927746)	1516 (0.069)	2548 (0.033)
impacting	2	3437(0.02286498227963873)	#SAKNAS!	2548 (0.033)
impacts	2	836(0.16005487595747114)	1516 (0.069)	461 (0.3)
impairs	2	3437(0.02286498227963873)	#SAKNAS!	1668 (0.066)
implant-based	2	3437(0.02286498227963873)	#SAKNAS!	2548 (0.033)
implant-supported	2	1894(0.05716245569909683)	1516 (0.069)	#SAKNAS!
implementation	2	836(0.16005487595747114)	1516 (0.069)	694 (0.2)
imprinting	2	3437(0.02286498227963873)	2359 (0.034)	#SAKNAS!
impulsivity	2	3437(0.02286498227963873)	#SAKNAS!	#SAKNAS!
inadequate	2	3437(0.02286498227963873)	2359 (0.034)	2548 (0.033)
incremental	2	2214(0.04572996455927746)	1516 (0.069)	2548 (0.033)
indexes	2	1485(0.08002743797873557)	1516 (0.069)	2548 (0.033)
indicate	2	2693(0.0342974734194581)	1516 (0.069)	#SAKNAS!
indicator	2	1027(0.12575740253801304)	1516 (0.069)	584 (0.23)
indices	2	787(0.1714873670972905)	1516 (0.069)	816 (0.16)
indirectly	2	3437(0.02286498227963873)	#SAKNAS!	1668 (0.066)
induces	2	1678(0.0685949468389162)	1516 (0.069)	999 (0.13)
ineffective	2	3437(0.02286498227963873)	#SAKNAS!	2548 (0.033)
inequalities	2	966(0.1371898936778324)	1516 (0.069)	694 (0.2)
infant-feeding	2	2214(0.04572996455927746)	1516 (0.069)	1668 (0.066)
infectious	2	3437(0.02286498227963873)	#SAKNAS!	#SAKNAS!
infestation	2	3437(0.02286498227963873)	#SAKNAS!	2548 (0.033)
influencing	2	500(0.2743797873556648)	1516 (0.069)	694 (0.2)

influential	2	3437(0.0228649822796387З)	#SAKNAS!	2548 (0.033)
informal	2	3437(0.0228649822796387З)	#SAKNAS!	#SAKNAS!
informing	2	3437(0.0228649822796387З)	#SAKNAS!	1668 (0.066)
infusion	2	1335(0.0914599291185549З)	1516 (0.069)	816 (0.16)
inhibit	2	3437(0.0228649822796387З)	2359 (0.034)	#SAKNAS!
inhibition	2	1894(0.0571624556990968З)	1516 (0.069)	2548 (0.033)
inhibitory	2	3437(0.0228649822796387З)	1516 (0.069)	#SAKNAS!
inhibitory	2	3437(0.0228649822796387З)	1516 (0.069)	#SAKNAS!
injections	2	3437(0.0228649822796387З)	2359 (0.034)	2548 (0.033)
injuries	2	1678(0.0685949468389162)	1516 (0.069)	1668 (0.066)
inks	2	3437(0.0228649822796387З)	#SAKNAS!	#SAKNAS!
innovation	2	3437(0.0228649822796387З)	#SAKNAS!	2548 (0.033)
inpatient	2	1111(0.114324911398193666)	1516 (0.069)	1668 (0.066)
insecticidal	2	3437(0.0228649822796387З)	2359 (0.034)	2548 (0.033)
insects	2	1335(0.0914599291185549З)	1516 (0.069)	816 (0.16)
insensitivity	2	3437(0.0228649822796387З)	1516 (0.069)	#SAKNAS!
insensitivity	2	3437(0.0228649822796387З)	1516 (0.069)	#SAKNAS!
institutional	2	3437(0.0228649822796387З)	2359 (0.034)	2548 (0.033)
instructions	2	3437(0.0228649822796387З)	#SAKNAS!	2548 (0.033)
insufficient	2	2214(0.0457299645592774б)	1516 (0.069)	1668 (0.066)
intact	2	3437(0.0228649822796387З)	2359 (0.034)	#SAKNAS!
intelligence-based	2	3437(0.0228649822796387З)	#SAKNAS!	#SAKNAS!
intensification	2	3437(0.0228649822796387З)	1516 (0.069)	#SAKNAS!
intensification	2	3437(0.0228649822796387З)	1516 (0.069)	#SAKNAS!
interact	2	3437(0.0228649822796387З)	#SAKNAS!	2548 (0.033)
interannual	2	3437(0.0228649822796387З)	2359 (0.034)	#SAKNAS!
interest	2	3437(0.0228649822796387З)	2359 (0.034)	2548 (0.033)
interferential	2	3437(0.0228649822796387З)	#SAKNAS!	1668 (0.066)
intermittent	2	1894(0.0571624556990968З)	1516 (0.069)	#SAKNAS!
internal	2	1111(0.114324911398193666)	1516 (0.069)	816 (0.16)
internet	2	3437(0.0228649822796387З)	2359 (0.034)	2548 (0.033)
interpretation	2	3437(0.0228649822796387З)	2359 (0.034)	#SAKNAS!
interrelation	2	3437(0.0228649822796387З)	2359 (0.034)	2548 (0.033)
interrelationship	2	2214(0.0457299645592774б)	1516 (0.069)	2548 (0.033)
intertidal	2	2693(0.0342974734194581)	1516 (0.069)	#SAKNAS!
interval	2	3437(0.0228649822796387З)	2359 (0.034)	#SAKNAS!
interviewing	2	3437(0.0228649822796387З)	2359 (0.034)	#SAKNAS!
interviews	2	3437(0.0228649822796387З)	#SAKNAS!	2548 (0.033)
intima-media	2	1678(0.0685949468389162)	1516 (0.069)	1668 (0.066)
intra-arterial	2	3437(0.0228649822796387З)	2359 (0.034)	2548 (0.033)
intracerebroventricular	2	3437(0.0228649822796387З)	2359 (0.034)	2548 (0.033)
intracranial	2	3437(0.0228649822796387З)	2359 (0.034)	#SAKNAS!
intractable	2	3437(0.0228649822796387З)	2359 (0.034)	2548 (0.033)

intramural	2	2693(0.0342974734194581)	1516 (0.069)	#SAKNAS!
intrasphincteric	2	3437(0.02286498227963873)	2359 (0.034)	2548 (0.033)
intrathoracic	2	3437(0.02286498227963873)	#SAKNAS!	1668 (0.066)
intra-thoracic	2	3437(0.02286498227963873)	2359 (0.034)	2548 (0.033)
intravenous	2	3437(0.02286498227963873)	#SAKNAS!	#SAKNAS!
intrinsic	2	1678(0.0685949468389162)	1516 (0.069)	1668 (0.066)
introduce	2	3437(0.02286498227963873)	#SAKNAS!	#SAKNAS!
inversely	2	1485(0.08002743797873557)	1516 (0.069)	816 (0.16)
invertebrate	2	3437(0.02286498227963873)	2359 (0.034)	2548 (0.033)
invest	2	2693(0.0342974734194581)	1516 (0.069)	2548 (0.033)
investigations	2	3437(0.02286498227963873)	#SAKNAS!	#SAKNAS!
invicta	2	3437(0.02286498227963873)	1516 (0.069)	#SAKNAS!
invicta	2	3437(0.02286498227963873)	1516 (0.069)	#SAKNAS!
involving	2	2214(0.04572996455927746)	1516 (0.069)	1668 (0.066)
ionic	2	3437(0.02286498227963873)	#SAKNAS!	2548 (0.033)
iranian	2	1485(0.08002743797873557)	1516 (0.069)	1668 (0.066)
irish	2	3437(0.02286498227963873)	2359 (0.034)	2548 (0.033)
irisin	2	3437(0.02286498227963873)	#SAKNAS!	2548 (0.033)
irradiation	2	2693(0.0342974734194581)	1516 (0.069)	2548 (0.033)
ischaemic	2	1894(0.05716245569909683)	1516 (0.069)	1668 (0.066)
isolates	2	3437(0.02286498227963873)	#SAKNAS!	2548 (0.033)
isolation	2	3437(0.02286498227963873)	#SAKNAS!	2548 (0.033)
isotope	2	2214(0.04572996455927746)	1516 (0.069)	2548 (0.033)
israeli	2	3437(0.02286498227963873)	2359 (0.034)	#SAKNAS!
iv	2	1111(0.11432491139819366)	1516 (0.069)	816 (0.16)
ivb	2	3437(0.02286498227963873)	2359 (0.034)	2548 (0.033)
jages	2	3437(0.02286498227963873)	#SAKNAS!	1668 (0.066)
jejunum	2	3437(0.02286498227963873)	2359 (0.034)	2548 (0.033)
jelly	2	3437(0.02286498227963873)	#SAKNAS!	#SAKNAS!
juan	2	2693(0.0342974734194581)	1516 (0.069)	2548 (0.033)
juices	2	3437(0.02286498227963873)	2359 (0.034)	#SAKNAS!
junction	2	1485(0.08002743797873557)	1516 (0.069)	1233 (0.1)
junk	2	3437(0.02286498227963873)	2359 (0.034)	2548 (0.033)
just	2	3437(0.02286498227963873)	#SAKNAS!	2548 (0.033)
karnataka	2	3437(0.02286498227963873)	2359 (0.034)	#SAKNAS!
kazakhstan	2	3437(0.02286498227963873)	#SAKNAS!	2548 (0.033)
kid	2	3437(0.02286498227963873)	2359 (0.034)	2548 (0.033)
killifish	2	3437(0.02286498227963873)	#SAKNAS!	2548 (0.033)
kinematic	2	3437(0.02286498227963873)	#SAKNAS!	2548 (0.033)
kinematics	2	3437(0.02286498227963873)	#SAKNAS!	#SAKNAS!
king	2	3437(0.02286498227963873)	2359 (0.034)	#SAKNAS!
know	2	3437(0.02286498227963873)	#SAKNAS!	2548 (0.033)
kong	2	1485(0.08002743797873557)	1516 (0.069)	816 (0.16)

korea	2	122(1.0517891848633818)	1516 (0.069)	86 (1.5)
koreans	2	1027(0.12575740253801304)	1516 (0.069)	999 (0.13)
kuchi-kara	2	3437(0.02286498227963873)	#SAKNAS!	1668 (0.066)
kwazulu-natal	2	3437(0.02286498227963873)	2359 (0.034)	2548 (0.033)
la	2	1678(0.0685949468389162)	1516 (0.069)	999 (0.13)
labeling	2	3437(0.02286498227963873)	#SAKNAS!	#SAKNAS!
labelling	2	3437(0.02286498227963873)	#SAKNAS!	1668 (0.066)
labels	2	3437(0.02286498227963873)	#SAKNAS!	1668 (0.066)
labial	2	3437(0.02286498227963873)	#SAKNAS!	2548 (0.033)
labor	2	2214(0.04572996455927746)	1516 (0.069)	1668 (0.066)
lacertid	2	3437(0.02286498227963873)	1516 (0.069)	#SAKNAS!
lacertid	2	3437(0.02286498227963873)	1516 (0.069)	#SAKNAS!
lactobacilli	2	3437(0.02286498227963873)	1516 (0.069)	#SAKNAS!
lactobacilli	2	3437(0.02286498227963873)	1516 (0.069)	#SAKNAS!
laevis	2	3437(0.02286498227963873)	2359 (0.034)	#SAKNAS!
lake	2	2693(0.0342974734194581)	1516 (0.069)	#SAKNAS!
lakes	2	3437(0.02286498227963873)	2359 (0.034)	2548 (0.033)
lam	2	3437(0.02286498227963873)	2359 (0.034)	2548 (0.033)
lao	2	3437(0.02286498227963873)	#SAKNAS!	1668 (0.066)
l-arginine	2	3437(0.02286498227963873)	2359 (0.034)	2548 (0.033)
laryngopharyngeal	2	3437(0.02286498227963873)	#SAKNAS!	#SAKNAS!
laryngotracheal	2	2693(0.0342974734194581)	1516 (0.069)	#SAKNAS!
laser	2	1485(0.08002743797873557)	1516 (0.069)	999 (0.13)
latinx	2	3437(0.02286498227963873)	#SAKNAS!	#SAKNAS!
leaflets	2	3437(0.02286498227963873)	2359 (0.034)	2548 (0.033)
leaks	2	3437(0.02286498227963873)	2359 (0.034)	#SAKNAS!
learning-based	2	3437(0.02286498227963873)	#SAKNAS!	#SAKNAS!
least	2	3437(0.02286498227963873)	2359 (0.034)	2548 (0.033)
lebanon	2	3437(0.02286498227963873)	#SAKNAS!	2548 (0.033)
legal	2	3437(0.02286498227963873)	#SAKNAS!	2548 (0.033)
leisure-time	2	3437(0.02286498227963873)	#SAKNAS!	#SAKNAS!
leontopithecus	2	3437(0.02286498227963873)	#SAKNAS!	2548 (0.033)
lessons	2	3437(0.02286498227963873)	#SAKNAS!	1668 (0.066)
libitum-fed	2	3437(0.02286498227963873)	#SAKNAS!	#SAKNAS!
libyan	2	3437(0.02286498227963873)	2359 (0.034)	#SAKNAS!
licury	2	3437(0.02286498227963873)	#SAKNAS!	#SAKNAS!
life-course	2	3437(0.02286498227963873)	1516 (0.069)	#SAKNAS!
life-course	2	3437(0.02286498227963873)	1516 (0.069)	#SAKNAS!
life-history	2	3437(0.02286498227963873)	2359 (0.034)	2548 (0.033)
life-threatening	2	3437(0.02286498227963873)	1516 (0.069)	#SAKNAS!
life-threatening	2	3437(0.02286498227963873)	1516 (0.069)	#SAKNAS!
lifetime	2	2214(0.04572996455927746)	1516 (0.069)	#SAKNAS!
lifting	2	3437(0.02286498227963873)	2359 (0.034)	2548 (0.033)

lightweight	2	3437(0.02286498227963873)	1516 (0.069)	#SAKNAS!
lightweight	2	3437(0.02286498227963873)	1516 (0.069)	#SAKNAS!
liking	2	3437(0.02286498227963873)	#SAKNAS!	1668 (0.066)
limestone	2	3437(0.02286498227963873)	#SAKNAS!	1668 (0.066)
limitations	2	3437(0.02286498227963873)	#SAKNAS!	#SAKNAS!
limiting	2	3437(0.02286498227963873)	2359 (0.034)	2548 (0.033)
limits	2	3437(0.02286498227963873)	2359 (0.034)	2548 (0.033)
limnephilidae	2	3437(0.02286498227963873)	2359 (0.034)	#SAKNAS!
limousin	2	3437(0.02286498227963873)	1516 (0.069)	#SAKNAS!
limousin	2	3437(0.02286498227963873)	1516 (0.069)	#SAKNAS!
line	2	3437(0.02286498227963873)	2359 (0.034)	#SAKNAS!
lines	2	3437(0.02286498227963873)	1516 (0.069)	#SAKNAS!
lines	2	3437(0.02286498227963873)	1516 (0.069)	#SAKNAS!
linkage	2	3437(0.02286498227963873)	2359 (0.034)	#SAKNAS!
links	2	1219(0.1028924202583743)	1516 (0.069)	999 (0.13)
linnaeus	2	3437(0.02286498227963873)	#SAKNAS!	#SAKNAS!
lion	2	3437(0.02286498227963873)	#SAKNAS!	2548 (0.033)
lip	2	1485(0.08002743797873557)	1516 (0.069)	2548 (0.033)
lipolysis	2	3437(0.02286498227963873)	1516 (0.069)	#SAKNAS!
lipolysis	2	3437(0.02286498227963873)	1516 (0.069)	#SAKNAS!
liquids	2	1111(0.11432491139819366)	1516 (0.069)	694 (0.2)
literacy	2	1678(0.0685949468389162)	1516 (0.069)	2548 (0.033)
lizard	2	2693(0.0342974734194581)	1516 (0.069)	2548 (0.033)
locus	2	3437(0.02286498227963873)	2359 (0.034)	#SAKNAS!
logistic	2	3437(0.02286498227963873)	2359 (0.034)	2548 (0.033)
longer	2	3437(0.02286498227963873)	#SAKNAS!	1668 (0.066)
long-gap	2	3437(0.02286498227963873)	#SAKNAS!	2548 (0.033)
longitudinal-study	2	3437(0.02286498227963873)	1516 (0.069)	#SAKNAS!
longitudinal-study	2	3437(0.02286498227963873)	1516 (0.069)	#SAKNAS!
long-tailed	2	3437(0.02286498227963873)	#SAKNAS!	2548 (0.033)
looped	2	3437(0.02286498227963873)	1516 (0.069)	#SAKNAS!
looped	2	3437(0.02286498227963873)	1516 (0.069)	#SAKNAS!
loose-housed	2	3437(0.02286498227963873)	1516 (0.069)	#SAKNAS!
loose-housed	2	3437(0.02286498227963873)	1516 (0.069)	#SAKNAS!
lost	2	3437(0.02286498227963873)	#SAKNAS!	#SAKNAS!
low-cost	2	3437(0.02286498227963873)	2359 (0.034)	2548 (0.033)
low-density	2	3437(0.02286498227963873)	2359 (0.034)	#SAKNAS!
lowland	2	3437(0.02286498227963873)	2359 (0.034)	2548 (0.033)
low-levels	2	3437(0.02286498227963873)	1516 (0.069)	#SAKNAS!
low-levels	2	3437(0.02286498227963873)	1516 (0.069)	#SAKNAS!
lusoria	2	3437(0.02286498227963873)	#SAKNAS!	2548 (0.033)
lymphedema	2	3437(0.02286498227963873)	#SAKNAS!	2548 (0.033)
lymphoma	2	3437(0.02286498227963873)	1516 (0.069)	#SAKNAS!

lymphoma	2	3437(0.0228649822796387)	1516 (0.069)	#SAKNAS!
macquart	2	3437(0.0228649822796387)	1516 (0.069)	#SAKNAS!
macquart	2	3437(0.0228649822796387)	1516 (0.069)	#SAKNAS!
macroalgal	2	3437(0.0228649822796387)	#SAKNAS!	1668 (0.066)
macronutrients	2	1678(0.0685949468389162)	1516 (0.069)	1668 (0.066)
macrophages	2	3437(0.0228649822796387)	#SAKNAS!	2548 (0.033)
macropomum	2	3437(0.0228649822796387)	#SAKNAS!	1668 (0.066)
macular	2	3437(0.0228649822796387)	#SAKNAS!	1668 (0.066)
maculata	2	3437(0.0228649822796387)	2359 (0.034)	2548 (0.033)
madagascar	2	2214(0.0457299645592774)	1516 (0.069)	2548 (0.033)
madinah	2	3437(0.0228649822796387)	#SAKNAS!	1668 (0.066)
magna	2	3437(0.0228649822796387)	#SAKNAS!	2548 (0.033)
magnesium	2	2214(0.0457299645592774)	1516 (0.069)	#SAKNAS!
maintains	2	3437(0.0228649822796387)	#SAKNAS!	#SAKNAS!
maize	2	2214(0.0457299645592774)	1516 (0.069)	#SAKNAS!
makes	2	3437(0.0228649822796387)	#SAKNAS!	2548 (0.033)
makkah	2	3437(0.0228649822796387)	#SAKNAS!	2548 (0.033)
malaria	2	3437(0.0228649822796387)	#SAKNAS!	1668 (0.066)
malawi	2	3437(0.0228649822796387)	#SAKNAS!	2548 (0.033)
malformations	2	3437(0.0228649822796387)	1516 (0.069)	#SAKNAS!
malformations	2	3437(0.0228649822796387)	1516 (0.069)	#SAKNAS!
malocclusion	2	1335(0.0914599291185549)	1516 (0.069)	1233 (0.1)
malocclusions	2	3437(0.0228649822796387)	2359 (0.034)	2548 (0.033)
mammal	2	2693(0.034297473419458)	1516 (0.069)	2548 (0.033)
mammalian	2	1485(0.0800274379787356)	1516 (0.069)	1233 (0.1)
mammals	2	3437(0.0228649822796387)	2359 (0.034)	2548 (0.033)
man	2	3437(0.0228649822796387)	1516 (0.069)	#SAKNAS!
man	2	3437(0.0228649822796387)	1516 (0.069)	#SAKNAS!
managing	2	2214(0.0457299645592774)	1516 (0.069)	1668 (0.066)
manner	2	3437(0.0228649822796387)	2359 (0.034)	2548 (0.033)
mannitol	2	3437(0.0228649822796387)	#SAKNAS!	1668 (0.066)
marburg	2	3437(0.0228649822796387)	2359 (0.034)	#SAKNAS!
mares	2	3437(0.0228649822796387)	2359 (0.034)	#SAKNAS!
marginal	2	1485(0.0800274379787356)	1516 (0.069)	999 (0.13)
marijuana	2	3437(0.0228649822796387)	#SAKNAS!	1668 (0.066)
marina	2	2693(0.034297473419458)	1516 (0.069)	2548 (0.033)
market	2	3437(0.0228649822796387)	2359 (0.034)	#SAKNAS!
marketing	2	3437(0.0228649822796387)	#SAKNAS!	2548 (0.033)
marmota	2	3437(0.0228649822796387)	2359 (0.034)	#SAKNAS!
marrow	2	3437(0.0228649822796387)	1516 (0.069)	#SAKNAS!
marrow	2	3437(0.0228649822796387)	1516 (0.069)	#SAKNAS!
marsupial	2	3437(0.0228649822796387)	2359 (0.034)	#SAKNAS!
masking	2	3437(0.0228649822796387)	2359 (0.034)	2548 (0.033)

massive	2	3437(0.0228649822796387З)	1516 (0.069)	#SAKNAS!
massive	2	3437(0.0228649822796387З)	1516 (0.069)	#SAKNAS!
maternal-child	2	3437(0.0228649822796387З)	2359 (0.034)	#SAKNAS!
mating	2	1678(0.0685949468389162)	1516 (0.069)	1668 (0.066)
matter	2	1027(0.12575740253801304)	1516 (0.069)	816 (0.16)
matters	2	1894(0.05716245569909683)	1516 (0.069)	1668 (0.066)
maxillectomy	2	3437(0.0228649822796387З)	#SAKNAS!	2548 (0.033)
maxillofacial	2	3437(0.0228649822796387З)	2359 (0.034)	#SAKNAS!
maximize	2	3437(0.0228649822796387З)	#SAKNAS!	2548 (0.033)
maximum	2	1485(0.08002743797873557)	1516 (0.069)	2548 (0.033)
mc4r	2	3437(0.0228649822796387З)	2359 (0.034)	#SAKNAS!
mealworm	2	3437(0.0228649822796387З)	#SAKNAS!	2548 (0.033)
mechanics	2	3437(0.0228649822796387З)	#SAKNAS!	1668 (0.066)
mechanistic	2	3437(0.0228649822796387З)	2359 (0.034)	2548 (0.033)
medial	2	3437(0.0228649822796387З)	#SAKNAS!	2548 (0.033)
mediate	2	1485(0.08002743797873557)	1516 (0.069)	1233 (0.1)
medicaid-eligible	2	3437(0.0228649822796387З)	1516 (0.069)	#SAKNAS!
medicaid-eligible	2	3437(0.0228649822796387З)	1516 (0.069)	#SAKNAS!
medically	2	3437(0.0228649822796387З)	1516 (0.069)	#SAKNAS!
medically	2	3437(0.0228649822796387З)	1516 (0.069)	#SAKNAS!
medicine	2	787(0.1714873670972905)	1516 (0.069)	694 (0.2)
megaesophagus	2	3437(0.0228649822796387З)	2359 (0.034)	2548 (0.033)
megalopa	2	3437(0.0228649822796387З)	#SAKNAS!	1668 (0.066)
melatonin	2	1894(0.05716245569909683)	1516 (0.069)	2548 (0.033)
mercury	2	3437(0.0228649822796387З)	1516 (0.069)	#SAKNAS!
mercury	2	3437(0.0228649822796387З)	1516 (0.069)	#SAKNAS!
mesh	2	2693(0.0342974734194581)	1516 (0.069)	#SAKNAS!
message	2	3437(0.0228649822796387З)	2359 (0.034)	2548 (0.033)
messages	2	1894(0.05716245569909683)	1516 (0.069)	1668 (0.066)
metabarcoding	2	3437(0.0228649822796387З)	#SAKNAS!	2548 (0.033)
metabolite	2	3437(0.0228649822796387З)	2359 (0.034)	2548 (0.033)
metabolizable	2	3437(0.0228649822796387З)	#SAKNAS!	#SAKNAS!
metallic	2	3437(0.0228649822796387З)	#SAKNAS!	1668 (0.066)
metalloproteinase-9	2	3437(0.0228649822796387З)	1516 (0.069)	#SAKNAS!
metalloproteinase-9	2	3437(0.0228649822796387З)	1516 (0.069)	#SAKNAS!
metamorphosis	2	3437(0.0228649822796387З)	#SAKNAS!	1668 (0.066)
metastatic	2	3437(0.0228649822796387З)	#SAKNAS!	1668 (0.066)
metformin	2	3437(0.0228649822796387З)	#SAKNAS!	2548 (0.033)
methadone	2	3437(0.0228649822796387З)	2359 (0.034)	2548 (0.033)
methamphetamine	2	1219(0.1028924202583743)	1516 (0.069)	694 (0.2)
methodological	2	3437(0.0228649822796387З)	#SAKNAS!	1668 (0.066)
methodology	2	1678(0.0685949468389162)	1516 (0.069)	1668 (0.066)
methylphenidate	2	3437(0.0228649822796387З)	2359 (0.034)	#SAKNAS!

mexican-american	2	3437(0.0228649822796387З)	1516 (0.069)	#SAKNAS!
mexican-american	2	3437(0.02286498227963873)	1516 (0.069)	#SAKNAS!
microbes	2	3437(0.02286498227963873)	#SAKNAS!	#SAKNAS!
microbiome	2	342(0.40013718989367786)	1516 (0.069)	361 (0.4)
microplastics	2	3437(0.02286498227963873)	#SAKNAS!	#SAKNAS!
microstructure	2	3437(0.02286498227963873)	#SAKNAS!	2548 (0.033)
microvascular	2	2693(0.0342974734194581)	1516 (0.069)	2548 (0.033)
middle-income	2	3437(0.02286498227963873)	2359 (0.034)	#SAKNAS!
midgut	2	3437(0.02286498227963873)	1516 (0.069)	#SAKNAS!
midgut	2	3437(0.02286498227963873)	1516 (0.069)	#SAKNAS!
might	2	3437(0.02286498227963873)	1516 (0.069)	#SAKNAS!
might	2	3437(0.02286498227963873)	1516 (0.069)	#SAKNAS!
migrants	2	3437(0.02286498227963873)	#SAKNAS!	2548 (0.033)
migration	2	1894(0.05716245569909683)	1516 (0.069)	1668 (0.066)
mild	2	1335(0.09145992911855493)	1516 (0.069)	1233 (0.1)
military	2	1894(0.05716245569909683)	1516 (0.069)	#SAKNAS!
milking	2	3437(0.02286498227963873)	2359 (0.034)	2548 (0.033)
mimicry	2	3437(0.02286498227963873)	#SAKNAS!	1668 (0.066)
mineralization	2	3437(0.02286498227963873)	#SAKNAS!	2548 (0.033)
minor	2	3437(0.02286498227963873)	2359 (0.034)	#SAKNAS!
miocene	2	2693(0.0342974734194581)	1516 (0.069)	2548 (0.033)
misperception	2	3437(0.02286498227963873)	#SAKNAS!	2548 (0.033)
missing	2	1111(0.11432491139819366)	1516 (0.069)	1668 (0.066)
misuse	2	3437(0.02286498227963873)	2359 (0.034)	#SAKNAS!
mitigates	2	3437(0.02286498227963873)	2359 (0.034)	2548 (0.033)
mitten	2	3437(0.02286498227963873)	#SAKNAS!	#SAKNAS!
mixotrophic	2	2214(0.04572996455927746)	1516 (0.069)	2548 (0.033)
modalities	2	3437(0.02286498227963873)	#SAKNAS!	2548 (0.033)
mode	2	3437(0.02286498227963873)	2359 (0.034)	#SAKNAS!
modeling	2	966(0.1371898936778324)	1516 (0.069)	999 (0.13)
moderating	2	3437(0.02286498227963873)	#SAKNAS!	2548 (0.033)
moderation	2	3437(0.02286498227963873)	2359 (0.034)	#SAKNAS!
modifier	2	3437(0.02286498227963873)	#SAKNAS!	2548 (0.033)
modifies	2	1219(0.1028924202583743)	1516 (0.069)	1233 (0.1)
modulating	2	3437(0.02286498227963873)	#SAKNAS!	2548 (0.033)
molar	2	1027(0.12575740253801304)	1516 (0.069)	1233 (0.1)
molitor	2	3437(0.02286498227963873)	#SAKNAS!	2548 (0.033)
mollis	2	3437(0.02286498227963873)	2359 (0.034)	2548 (0.033)
monensin	2	3437(0.02286498227963873)	#SAKNAS!	#SAKNAS!
monocentric	2	3437(0.02286498227963873)	#SAKNAS!	2548 (0.033)
monodon	2	3437(0.02286498227963873)	2359 (0.034)	#SAKNAS!
monoglyceride	2	3437(0.02286498227963873)	1516 (0.069)	#SAKNAS!
monoglyceride	2	3437(0.02286498227963873)	1516 (0.069)	#SAKNAS!

monozygotic	2	3437(0.02286498227963873)	1516 (0.069)	#SAKNAS!
monozygotic	2	3437(0.02286498227963873)	1516 (0.069)	#SAKNAS!
mood	2	3437(0.02286498227963873)	2359 (0.034)	2548 (0.033)
morphofunctional	2	3437(0.02286498227963873)	#SAKNAS!	#SAKNAS!
mother-child	2	3437(0.02286498227963873)	#SAKNAS!	2548 (0.033)
motherhood	2	3437(0.02286498227963873)	2359 (0.034)	2548 (0.033)
mother-young	2	3437(0.02286498227963873)	2359 (0.034)	2548 (0.033)
motion	2	3437(0.02286498227963873)	2359 (0.034)	#SAKNAS!
motivational	2	3437(0.02286498227963873)	2359 (0.034)	2548 (0.033)
mouse	2	1027(0.12575740253801304)	1516 (0.069)	1233 (0.1)
mousse	2	3437(0.02286498227963873)	#SAKNAS!	#SAKNAS!
mouthwash	2	3437(0.02286498227963873)	#SAKNAS!	2548 (0.033)
mouthwashes	2	3437(0.02286498227963873)	#SAKNAS!	2548 (0.033)
movement	2	1894(0.05716245569909683)	1516 (0.069)	1668 (0.066)
moving	2	3437(0.02286498227963873)	#SAKNAS!	#SAKNAS!
mucosa	2	3437(0.02286498227963873)	#SAKNAS!	2548 (0.033)
muller	2	3437(0.02286498227963873)	1516 (0.069)	#SAKNAS!
muller	2	3437(0.02286498227963873)	1516 (0.069)	#SAKNAS!
multicentre	2	1027(0.12575740253801304)	1516 (0.069)	694 (0.2)
multicentric	2	3437(0.02286498227963873)	2359 (0.034)	2548 (0.033)
multidisciplinary	2	595(0.22864982279638732)	1516 (0.069)	461 (0.3)
multigrain	2	3437(0.02286498227963873)	#SAKNAS!	#SAKNAS!
multi-institutional	2	3437(0.02286498227963873)	#SAKNAS!	2548 (0.033)
multilevel	2	1335(0.09145992911855493)	1516 (0.069)	999 (0.13)
multimodal	2	3437(0.02286498227963873)	#SAKNAS!	#SAKNAS!
multinational	2	3437(0.02286498227963873)	2359 (0.034)	2548 (0.033)
multi-omics	2	3437(0.02286498227963873)	#SAKNAS!	#SAKNAS!
multivariable	2	3437(0.02286498227963873)	#SAKNAS!	#SAKNAS!
mumbai	2	3437(0.02286498227963873)	#SAKNAS!	2548 (0.033)
munich	2	3437(0.02286498227963873)	#SAKNAS!	2548 (0.033)
murine	2	2214(0.04572996455927746)	1516 (0.069)	2548 (0.033)
musa	2	3437(0.02286498227963873)	#SAKNAS!	2548 (0.033)
musculoskeletal	2	3437(0.02286498227963873)	#SAKNAS!	2548 (0.033)
mustela	2	3437(0.02286498227963873)	1516 (0.069)	#SAKNAS!
mustela	2	3437(0.02286498227963873)	1516 (0.069)	#SAKNAS!
mutualistic	2	3437(0.02286498227963873)	2359 (0.034)	2548 (0.033)
my	2	3437(0.02286498227963873)	#SAKNAS!	1668 (0.066)
myasthenia	2	1894(0.05716245569909683)	1516 (0.069)	#SAKNAS!
mycotoxin	2	3437(0.02286498227963873)	#SAKNAS!	2548 (0.033)
myectomy	2	3437(0.02286498227963873)	#SAKNAS!	2548 (0.033)
myelopathy	2	3437(0.02286498227963873)	2359 (0.034)	#SAKNAS!
myeloperoxidase	2	3437(0.02286498227963873)	2359 (0.034)	#SAKNAS!
myths	2	3437(0.02286498227963873)	#SAKNAS!	2548 (0.033)

n-3	2	3437(0.0228649822796387З)	1516 (0.069)	#SAKNAS!
n-3	2	3437(0.02286498227963873)	1516 (0.069)	#SAKNAS!
nap	2	3437(0.02286498227963873)	#SAKNAS!	1668 (0.066)
nasal	2	1485(0.08002743797873557)	1516 (0.069)	2548 (0.033)
naturally	2	3437(0.02286498227963873)	2359 (0.034)	2548 (0.033)
nauplii	2	3437(0.02286498227963873)	2359 (0.034)	2548 (0.033)
ne	2	3437(0.02286498227963873)	1516 (0.069)	#SAKNAS!
ne	2	3437(0.02286498227963873)	1516 (0.069)	#SAKNAS!
nematodes	2	3437(0.02286498227963873)	2359 (0.034)	2548 (0.033)
neonates	2	2214(0.04572996455927746)	1516 (0.069)	2548 (0.033)
neoplasms	2	3437(0.02286498227963873)	2359 (0.034)	#SAKNAS!
nepal	2	3437(0.02286498227963873)	#SAKNAS!	1668 (0.066)
nepali	2	3437(0.02286498227963873)	2359 (0.034)	2548 (0.033)
nephropathy	2	3437(0.02286498227963873)	2359 (0.034)	#SAKNAS!
net	2	3437(0.02286498227963873)	1516 (0.069)	#SAKNAS!
net	2	3437(0.02286498227963873)	1516 (0.069)	#SAKNAS!
networks	2	3437(0.02286498227963873)	#SAKNAS!	#SAKNAS!
neurobiological	2	3437(0.02286498227963873)	2359 (0.034)	2548 (0.033)
neuroendocrine	2	2693(0.0342974734194581)	1516 (0.069)	#SAKNAS!
neuron	2	2214(0.04572996455927746)	1516 (0.069)	1668 (0.066)
neurons	2	836(0.16005487595747114)	1516 (0.069)	584 (0.23)
neuropeptide-y	2	3437(0.02286498227963873)	1516 (0.069)	#SAKNAS!
neuropeptide-y	2	3437(0.02286498227963873)	1516 (0.069)	#SAKNAS!
neurorehabilitation	2	3437(0.02286498227963873)	2359 (0.034)	#SAKNAS!
neurosecretory	2	3437(0.02286498227963873)	#SAKNAS!	2548 (0.033)
neurotensin	2	3437(0.02286498227963873)	1516 (0.069)	#SAKNAS!
neurotensin	2	3437(0.02286498227963873)	1516 (0.069)	#SAKNAS!
nevada	2	3437(0.02286498227963873)	1516 (0.069)	#SAKNAS!
nevada	2	3437(0.02286498227963873)	1516 (0.069)	#SAKNAS!
newborns	2	1894(0.05716245569909683)	1516 (0.069)	1668 (0.066)
next	2	3437(0.02286498227963873)	#SAKNAS!	1668 (0.066)
niger	2	3437(0.02286498227963873)	#SAKNAS!	1668 (0.066)
night	2	1678(0.0685949468389162)	1516 (0.069)	1668 (0.066)
nighttime	2	3437(0.02286498227963873)	#SAKNAS!	#SAKNAS!
niloticus	2	3437(0.02286498227963873)	2359 (0.034)	2548 (0.033)
nine-year	2	3437(0.02286498227963873)	2359 (0.034)	2548 (0.033)
noise	2	3437(0.02286498227963873)	#SAKNAS!	2548 (0.033)
non	2	3437(0.02286498227963873)	1516 (0.069)	#SAKNAS!
non	2	3437(0.02286498227963873)	1516 (0.069)	#SAKNAS!
non-hispanic	2	3437(0.02286498227963873)	2359 (0.034)	2548 (0.033)
non-indigenous	2	3437(0.02286498227963873)	#SAKNAS!	1668 (0.066)
non-linear	2	3437(0.02286498227963873)	2359 (0.034)	2548 (0.033)
nonnutritive	2	2693(0.0342974734194581)	1516 (0.069)	#SAKNAS!

nonobese	2	3437(0.0228649822796387З)	#SAKNAS!	2548 (0.033)
nonoral	2	3437(0.0228649822796387З)	1516 (0.069)	#SAKNAS!
nonoral	2	3437(0.0228649822796387З)	1516 (0.069)	#SAKNAS!
non-plaque-induced	2	3437(0.0228649822796387З)	#SAKNAS!	1668 (0.066)
nonpregnant	2	3437(0.0228649822796387З)	#SAKNAS!	1668 (0.066)
nonsmokers	2	3437(0.0228649822796387З)	2359 (0.034)	2548 (0.033)
non-smoking	2	2214(0.0457299645592774б)	1516 (0.069)	1668 (0.066)
nonsurgical	2	1678(0.0685949468389162)	1516 (0.069)	1233 (0.1)
nor	2	3437(0.0228649822796387З)	2359 (0.034)	2548 (0.033)
northeast	2	2214(0.0457299645592774б)	1516 (0.069)	2548 (0.033)
north-east	2	3437(0.0228649822796387З)	2359 (0.034)	#SAKNAS!
north-western	2	3437(0.0228649822796387З)	#SAKNAS!	2548 (0.033)
norvegicus	2	3437(0.0228649822796387З)	2359 (0.034)	2548 (0.033)
nosocomial	2	3437(0.0228649822796387З)	2359 (0.034)	#SAKNAS!
note	2	3437(0.0228649822796387З)	1516 (0.069)	#SAKNAS!
note	2	3437(0.0228649822796387З)	1516 (0.069)	#SAKNAS!
npffr2	2	3437(0.0228649822796387З)	2359 (0.034)	#SAKNAS!
nucleatum	2	2693(0.0342974734194581)	1516 (0.069)	2548 (0.033)
nuclei	2	3437(0.0228649822796387З)	2359 (0.034)	2548 (0.033)
nuculanidae	2	3437(0.0228649822796387З)	1516 (0.069)	#SAKNAS!
nuculanidae	2	3437(0.0228649822796387З)	1516 (0.069)	#SAKNAS!
nuptial	2	3437(0.0228649822796387З)	#SAKNAS!	2548 (0.033)
nurse-led	2	3437(0.0228649822796387З)	#SAKNAS!	2548 (0.033)
nursery	2	3437(0.0228649822796387З)	#SAKNAS!	1668 (0.066)
nutritional-status	2	3437(0.0228649822796387З)	1516 (0.069)	#SAKNAS!
nutritional-status	2	3437(0.0228649822796387З)	1516 (0.069)	#SAKNAS!
nutritional-value	2	3437(0.0228649822796387З)	1516 (0.069)	#SAKNAS!
nutritional-value	2	3437(0.0228649822796387З)	1516 (0.069)	#SAKNAS!
nutrition-related	2	3437(0.0228649822796387З)	#SAKNAS!	2548 (0.033)
nutritious	2	3437(0.0228649822796387З)	#SAKNAS!	#SAKNAS!
oak	2	3437(0.0228649822796387З)	2359 (0.034)	2548 (0.033)
obliqua	2	3437(0.0228649822796387З)	1516 (0.069)	#SAKNAS!
obliqua	2	3437(0.0228649822796387З)	1516 (0.069)	#SAKNAS!
observable	2	3437(0.0228649822796387З)	#SAKNAS!	2548 (0.033)
observation	2	1335(0.0914599291185549З)	1516 (0.069)	2548 (0.033)
obtain	2	3437(0.0228649822796387З)	#SAKNAS!	2548 (0.033)
o-c2	2	3437(0.0228649822796387З)	2359 (0.034)	2548 (0.033)
occluding	2	3437(0.0228649822796387З)	#SAKNAS!	2548 (0.033)
occurrence	2	966(0.1371898936778324)	1516 (0.069)	816 (0.16)
octogenarians	2	3437(0.0228649822796387З)	2359 (0.034)	2548 (0.033)
odontoid	2	3437(0.0228649822796387З)	#SAKNAS!	1668 (0.066)
oesophagectomy	2	1894(0.0571624556990968З)	1516 (0.069)	1668 (0.066)
oesophagogastric	2	3437(0.0228649822796387З)	2359 (0.034)	2548 (0.033)

offsets	2	3437(0.02286498227963873)	2359 (0.034)	2548 (0.033)
ohasama	2	3437(0.02286498227963873)	#SAKNAS!	2548 (0.033)
ohio	2	3437(0.02286498227963873)	1516 (0.069)	#SAKNAS!
ohio	2	3437(0.02286498227963873)	1516 (0.069)	#SAKNAS!
oleracea	2	3437(0.02286498227963873)	2359 (0.034)	#SAKNAS!
oligopeptidase	2	3437(0.02286498227963873)	#SAKNAS!	2548 (0.033)
omega-3	2	2214(0.04572996455927746)	1516 (0.069)	#SAKNAS!
oncological	2	1894(0.05716245569909683)	1516 (0.069)	#SAKNAS!
one-anastomosis	2	3437(0.02286498227963873)	#SAKNAS!	#SAKNAS!
one-stage	2	2693(0.0342974734194581)	1516 (0.069)	#SAKNAS!
ontogeny	2	2693(0.0342974734194581)	1516 (0.069)	#SAKNAS!
opening	2	2214(0.04572996455927746)	1516 (0.069)	2548 (0.033)
open-label	2	3437(0.02286498227963873)	#SAKNAS!	1668 (0.066)
opportunities	2	1485(0.08002743797873557)	1516 (0.069)	1668 (0.066)
opportunity	2	3437(0.02286498227963873)	2359 (0.034)	#SAKNAS!
optimization	2	3437(0.02286498227963873)	#SAKNAS!	#SAKNAS!
optimizing	2	2214(0.04572996455927746)	1516 (0.069)	2548 (0.033)
opuntia	2	3437(0.02286498227963873)	#SAKNAS!	1668 (0.066)
oral-health-related	2	3437(0.02286498227963873)	#SAKNAS!	2548 (0.033)
orchestoidea	2	2693(0.0342974734194581)	1516 (0.069)	2548 (0.033)
orexin-a	2	3437(0.02286498227963873)	2359 (0.034)	2548 (0.033)
organisms	2	2214(0.04572996455927746)	1516 (0.069)	1668 (0.066)
organization	2	1894(0.05716245569909683)	1516 (0.069)	2548 (0.033)
organoleptic	2	3437(0.02286498227963873)	2359 (0.034)	2548 (0.033)
organs	2	3437(0.02286498227963873)	2359 (0.034)	2548 (0.033)
origins	2	2693(0.0342974734194581)	1516 (0.069)	2548 (0.033)
orodispersible	2	3437(0.02286498227963873)	#SAKNAS!	2548 (0.033)
orosensory	2	3437(0.02286498227963873)	#SAKNAS!	1668 (0.066)
orseis	2	3437(0.02286498227963873)	1516 (0.069)	#SAKNAS!
orseis	2	3437(0.02286498227963873)	1516 (0.069)	#SAKNAS!
orthodontic	2	1027(0.12575740253801304)	1516 (0.069)	526 (0.26)
orthoptera	2	3437(0.02286498227963873)	2359 (0.034)	#SAKNAS!
osas	2	3437(0.02286498227963873)	2359 (0.034)	#SAKNAS!
oscillations	2	3437(0.02286498227963873)	2359 (0.034)	2548 (0.033)
oscillatory	2	3437(0.02286498227963873)	2359 (0.034)	#SAKNAS!
osteoporotic	2	2214(0.04572996455927746)	1516 (0.069)	1668 (0.066)
osteotomy	2	3437(0.02286498227963873)	#SAKNAS!	2548 (0.033)
otassha	2	3437(0.02286498227963873)	#SAKNAS!	#SAKNAS!
otolaryngologist	2	3437(0.02286498227963873)	#SAKNAS!	#SAKNAS!
otolaryngology	2	3437(0.02286498227963873)	#SAKNAS!	#SAKNAS!
otorhinolaryngological	2	3437(0.02286498227963873)	#SAKNAS!	1668 (0.066)
outdoor	2	3437(0.02286498227963873)	2359 (0.034)	2548 (0.033)
outpatient	2	787(0.1714873670972905)	1516 (0.069)	694 (0.2)

outside	2	3437(0.0228649822796387З)	#SAKNAS!	2548 (0.033)
overcoming	2	3437(0.02286498227963873)	#SAKNAS!	2548 (0.033)
overexpression	2	3437(0.02286498227963873)	2359 (0.034)	2548 (0.033)
overload	2	3437(0.02286498227963873)	#SAKNAS!	1668 (0.066)
overnutrition	2	3437(0.02286498227963873)	#SAKNAS!	1668 (0.066)
owner	2	3437(0.02286498227963873)	#SAKNAS!	1668 (0.066)
oxygen	2	1485(0.08002743797873557)	1516 (0.069)	1668 (0.066)
oxyrrhis	2	3437(0.02286498227963873)	1516 (0.069)	#SAKNAS!
oxyrrhis	2	3437(0.02286498227963873)	1516 (0.069)	#SAKNAS!
oxytocin	2	2214(0.04572996455927746)	1516 (0.069)	2548 (0.033)
package	2	3437(0.02286498227963873)	2359 (0.034)	#SAKNAS!
paclitaxel	2	3437(0.02286498227963873)	1516 (0.069)	#SAKNAS!
paclitaxel	2	3437(0.02286498227963873)	1516 (0.069)	#SAKNAS!
pair	2	3437(0.02286498227963873)	2359 (0.034)	#SAKNAS!
paleopathology	2	3437(0.02286498227963873)	#SAKNAS!	1668 (0.066)
palestine	2	3437(0.02286498227963873)	#SAKNAS!	2548 (0.033)
palestinian	2	3437(0.02286498227963873)	#SAKNAS!	#SAKNAS!
pancreatoduodenectomy	2	3437(0.02286498227963873)	2359 (0.034)	2548 (0.033)
papillae	2	3437(0.02286498227963873)	#SAKNAS!	2548 (0.033)
papillomavirus-positive	2	3437(0.02286498227963873)	#SAKNAS!	1668 (0.066)
paradigm	2	3437(0.02286498227963873)	2359 (0.034)	2548 (0.033)
paralarvae	2	3437(0.02286498227963873)	#SAKNAS!	#SAKNAS!
paralysis	2	3437(0.02286498227963873)	2359 (0.034)	#SAKNAS!
parana	2	3437(0.02286498227963873)	1516 (0.069)	#SAKNAS!
parana	2	3437(0.02286498227963873)	1516 (0.069)	#SAKNAS!
parasite	2	3437(0.02286498227963873)	2359 (0.034)	#SAKNAS!
parasitoids	2	3437(0.02286498227963873)	2359 (0.034)	#SAKNAS!
parathyroid	2	3437(0.02286498227963873)	2359 (0.034)	#SAKNAS!
parent	2	743(0.18291985823710985)	1516 (0.069)	584 (0.23)
parental-reported	2	3437(0.02286498227963873)	2359 (0.034)	#SAKNAS!
parent-child	2	3437(0.02286498227963873)	#SAKNAS!	1668 (0.066)
parenteral-nutrition	2	3437(0.02286498227963873)	1516 (0.069)	#SAKNAS!
parenteral-nutrition	2	3437(0.02286498227963873)	1516 (0.069)	#SAKNAS!
paris	2	3437(0.02286498227963873)	#SAKNAS!	#SAKNAS!
parity	2	2214(0.04572996455927746)	1516 (0.069)	2548 (0.033)
park	2	3437(0.02286498227963873)	1516 (0.069)	#SAKNAS!
park	2	3437(0.02286498227963873)	1516 (0.069)	#SAKNAS!
parotid	2	1678(0.0685949468389162)	1516 (0.069)	1233 (0.1)
participate	2	3437(0.02286498227963873)	1516 (0.069)	#SAKNAS!
participate	2	3437(0.02286498227963873)	1516 (0.069)	#SAKNAS!
particles	2	3437(0.02286498227963873)	1516 (0.069)	#SAKNAS!
particles	2	3437(0.02286498227963873)	1516 (0.069)	#SAKNAS!
particular	2	2214(0.04572996455927746)	1516 (0.069)	2548 (0.033)

partitioning	2	2693(0.0342974734194581)	1516 (0.069)	2548 (0.033)
patagonian	2	3437(0.0228649822796387З)	1516 (0.069)	#SAKNAS!
patagonian	2	3437(0.02286498227963873)	1516 (0.069)	#SAKNAS!
patelliferum	2	3437(0.02286498227963873)	1516 (0.069)	#SAKNAS!
patelliferum	2	3437(0.02286498227963873)	1516 (0.069)	#SAKNAS!
pathologist	2	3437(0.02286498227963873)	#SAKNAS!	2548 (0.033)
pathways	2	1111(0.11432491139819366)	1516 (0.069)	999 (0.13)
patient-generated	2	3437(0.02286498227963873)	2359 (0.034)	2548 (0.033)
pdr	2	3437(0.02286498227963873)	#SAKNAS!	1668 (0.066)
pea	2	1027(0.12575740253801304)	1516 (0.069)	#SAKNAS!
pear	2	3437(0.02286498227963873)	#SAKNAS!	#SAKNAS!
pectoralis	2	3437(0.02286498227963873)	1516 (0.069)	#SAKNAS!
pectoralis	2	3437(0.02286498227963873)	1516 (0.069)	#SAKNAS!
pectoris	2	3437(0.02286498227963873)	#SAKNAS!	1668 (0.066)
pediatrician	2	3437(0.02286498227963873)	2359 (0.034)	#SAKNAS!
peer	2	3437(0.02286498227963873)	#SAKNAS!	1668 (0.066)
pellets	2	3437(0.02286498227963873)	#SAKNAS!	2548 (0.033)
penetration-aspiration	2	3437(0.02286498227963873)	2359 (0.034)	2548 (0.033)
penguins	2	3437(0.02286498227963873)	2359 (0.034)	#SAKNAS!
peninsula	2	3437(0.02286498227963873)	2359 (0.034)	#SAKNAS!
pepper	2	2214(0.04572996455927746)	1516 (0.069)	1668 (0.066)
peptic	2	3437(0.02286498227963873)	2359 (0.034)	2548 (0.033)
peptide-1	2	3437(0.02286498227963873)	#SAKNAS!	2548 (0.033)
peptides	2	2693(0.0342974734194581)	1516 (0.069)	#SAKNAS!
perch	2	2693(0.0342974734194581)	1516 (0.069)	#SAKNAS!
perforator	2	3437(0.02286498227963873)	#SAKNAS!	#SAKNAS!
performances	2	3437(0.02286498227963873)	#SAKNAS!	2548 (0.033)
performing	2	3437(0.02286498227963873)	#SAKNAS!	#SAKNAS!
peri	2	3437(0.02286498227963873)	2359 (0.034)	2548 (0.033)
peri-implantitis	2	3437(0.02286498227963873)	#SAKNAS!	#SAKNAS!
periodontopathogenic	2	3437(0.02286498227963873)	#SAKNAS!	1668 (0.066)
peripherally	2	3437(0.02286498227963873)	#SAKNAS!	#SAKNAS!
periprosthetic	2	3437(0.02286498227963873)	#SAKNAS!	2548 (0.033)
persian	2	3437(0.02286498227963873)	#SAKNAS!	#SAKNAS!
persist	2	3437(0.02286498227963873)	2359 (0.034)	2548 (0.033)
persisting	2	3437(0.02286498227963873)	2359 (0.034)	2548 (0.033)
personality	2	3437(0.02286498227963873)	#SAKNAS!	2548 (0.033)
pet	2	3437(0.02286498227963873)	2359 (0.034)	2548 (0.033)
pg-sga	2	3437(0.02286498227963873)	2359 (0.034)	2548 (0.033)
phagocytosis	2	3437(0.02286498227963873)	#SAKNAS!	2548 (0.033)
phagostimulants	2	3437(0.02286498227963873)	1516 (0.069)	#SAKNAS!
phagostimulants	2	3437(0.02286498227963873)	1516 (0.069)	#SAKNAS!
pharmacovigilance	2	3437(0.02286498227963873)	#SAKNAS!	#SAKNAS!

pharynx	2	3437(0.0228649822796387)	2359 (0.034)	2548 (0.033)
phenolic	2	3437(0.0228649822796387)	1516 (0.069)	#SAKNAS!
phenolic	2	3437(0.0228649822796387)	1516 (0.069)	#SAKNAS!
phenology	2	3437(0.0228649822796387)	1516 (0.069)	#SAKNAS!
phenology	2	3437(0.0228649822796387)	1516 (0.069)	#SAKNAS!
phenome-wide	2	3437(0.0228649822796387)	#SAKNAS!	2548 (0.033)
phenotyping	2	3437(0.0228649822796387)	#SAKNAS!	2548 (0.033)
phenylketonuria	2	2693(0.0342974734194581)	1516 (0.069)	#SAKNAS!
pheromone	2	3437(0.0228649822796387)	2359 (0.034)	#SAKNAS!
philippines	2	2693(0.0342974734194581)	1516 (0.069)	#SAKNAS!
phloem	2	2214(0.0457299645592775)	1516 (0.069)	1668 (0.066)
phoniatric	2	3437(0.0228649822796387)	#SAKNAS!	#SAKNAS!
photodynamic	2	1894(0.0571624556990968)	1516 (0.069)	#SAKNAS!
photoperiod	2	3437(0.0228649822796387)	1516 (0.069)	#SAKNAS!
photoperiod	2	3437(0.0228649822796387)	1516 (0.069)	#SAKNAS!
phthalate	2	3437(0.0228649822796387)	#SAKNAS!	2548 (0.033)
physicians	2	2214(0.0457299645592775)	1516 (0.069)	1668 (0.066)
phytoplankton	2	2693(0.0342974734194581)	1516 (0.069)	#SAKNAS!
phytoplasma	2	3437(0.0228649822796387)	#SAKNAS!	1668 (0.066)
picea	2	3437(0.0228649822796387)	1516 (0.069)	#SAKNAS!
picea	2	3437(0.0228649822796387)	1516 (0.069)	#SAKNAS!
picky	2	3437(0.0228649822796387)	#SAKNAS!	2548 (0.033)
pigra	2	3437(0.0228649822796387)	2359 (0.034)	2548 (0.033)
pine	2	3437(0.0228649822796387)	1516 (0.069)	#SAKNAS!
pine	2	3437(0.0228649822796387)	1516 (0.069)	#SAKNAS!
plant-insect	2	3437(0.0228649822796387)	#SAKNAS!	#SAKNAS!
plasminogen	2	3437(0.0228649822796387)	2359 (0.034)	#SAKNAS!
plasticity	2	1678(0.0685949468389162)	1516 (0.069)	2548 (0.033)
platelet	2	3437(0.0228649822796387)	#SAKNAS!	2548 (0.033)
plating	2	3437(0.0228649822796387)	#SAKNAS!	2548 (0.033)
platinum	2	3437(0.0228649822796387)	1516 (0.069)	#SAKNAS!
platinum	2	3437(0.0228649822796387)	1516 (0.069)	#SAKNAS!
plays	2	3437(0.0228649822796387)	#SAKNAS!	2548 (0.033)
plication	2	2693(0.0342974734194581)	1516 (0.069)	2548 (0.033)
pneumatic	2	3437(0.0228649822796387)	2359 (0.034)	#SAKNAS!
pocket	2	3437(0.0228649822796387)	#SAKNAS!	2548 (0.033)
pocketing	2	3437(0.0228649822796387)	#SAKNAS!	2548 (0.033)
pockets	2	3437(0.0228649822796387)	2359 (0.034)	2548 (0.033)
poem	2	3437(0.0228649822796387)	2359 (0.034)	#SAKNAS!
point	2	1335(0.0914599291185549)	1516 (0.069)	816 (0.16)
polyaromatic	2	3437(0.0228649822796387)	#SAKNAS!	2548 (0.033)
polycyclic	2	3437(0.0228649822796387)	#SAKNAS!	#SAKNAS!
polyethylene	2	3437(0.0228649822796387)	1516 (0.069)	#SAKNAS!

polyethylene	2	3437(0.02286498227963873)	1516 (0.069)	#SAKNAS!
polyfluoroalkyl	2	3437(0.02286498227963873)	#SAKNAS!	2548 (0.033)
polymorpha	2	3437(0.02286498227963873)	2359 (0.034)	2548 (0.033)
polyphagous	2	2693(0.0342974734194581)	1516 (0.069)	2548 (0.033)
polyurethane	2	3437(0.02286498227963873)	#SAKNAS!	2548 (0.033)
pomc	2	3437(0.02286498227963873)	#SAKNAS!	1668 (0.066)
pond	2	3437(0.02286498227963873)	2359 (0.034)	#SAKNAS!
pontine	2	3437(0.02286498227963873)	1516 (0.069)	#SAKNAS!
pontine	2	3437(0.02286498227963873)	1516 (0.069)	#SAKNAS!
poorer	2	3437(0.02286498227963873)	2359 (0.034)	2548 (0.033)
popular	2	3437(0.02286498227963873)	#SAKNAS!	2548 (0.033)
porcellus	2	3437(0.02286498227963873)	#SAKNAS!	2548 (0.033)
porridge	2	3437(0.02286498227963873)	#SAKNAS!	#SAKNAS!
portable	2	3437(0.02286498227963873)	2359 (0.034)	#SAKNAS!
portion	2	3437(0.02286498227963873)	2359 (0.034)	2548 (0.033)
porto	2	3437(0.02286498227963873)	1516 (0.069)	#SAKNAS!
porto	2	3437(0.02286498227963873)	1516 (0.069)	#SAKNAS!
portugal	2	3437(0.02286498227963873)	2359 (0.034)	#SAKNAS!
positions	2	3437(0.02286498227963873)	#SAKNAS!	1668 (0.066)
positron	2	3437(0.02286498227963873)	1516 (0.069)	#SAKNAS!
positron	2	3437(0.02286498227963873)	1516 (0.069)	#SAKNAS!
post	2	1335(0.09145992911855493)	1516 (0.069)	1233 (0.1)
post-covid-19	2	3437(0.02286498227963873)	#SAKNAS!	#SAKNAS!
poster	2	3437(0.02286498227963873)	#SAKNAS!	2548 (0.033)
post-esophagectomy	2	3437(0.02286498227963873)	#SAKNAS!	2548 (0.033)
post-ingestive	2	2693(0.0342974734194581)	1516 (0.069)	#SAKNAS!
post-intensive	2	3437(0.02286498227963873)	#SAKNAS!	#SAKNAS!
postlaryngectomy	2	3437(0.02286498227963873)	1516 (0.069)	#SAKNAS!
postlaryngectomy	2	3437(0.02286498227963873)	1516 (0.069)	#SAKNAS!
post-menopausal	2	3437(0.02286498227963873)	#SAKNAS!	2548 (0.033)
post-radiotherapy	2	3437(0.02286498227963873)	#SAKNAS!	2548 (0.033)
posttreatment	2	3437(0.02286498227963873)	#SAKNAS!	2548 (0.033)
postural	2	3437(0.02286498227963873)	#SAKNAS!	2548 (0.033)
post-weaning	2	3437(0.02286498227963873)	#SAKNAS!	2548 (0.033)
potassium	2	3437(0.02286498227963873)	#SAKNAS!	2548 (0.033)
potato	2	1678(0.0685949468389162)	1516 (0.069)	1668 (0.066)
pouch	2	3437(0.02286498227963873)	2359 (0.034)	2548 (0.033)
pouches	2	3437(0.02286498227963873)	#SAKNAS!	2548 (0.033)
poverty	2	1894(0.05716245569909683)	1516 (0.069)	1233 (0.1)
practical	2	1335(0.09145992911855493)	1516 (0.069)	1233 (0.1)
practice-based	2	3437(0.02286498227963873)	2359 (0.034)	#SAKNAS!
pragmatic	2	3437(0.02286498227963873)	#SAKNAS!	1668 (0.066)
prairie	2	3437(0.02286498227963873)	#SAKNAS!	1668 (0.066)

pre-	2	3437(0.0228649822796387З)	1516 (0.069)	#SAKNAS!
pre-	2	3437(0.0228649822796387З)	1516 (0.069)	#SAKNAS!
prebiotic	2	3437(0.0228649822796387З)	#SAKNAS!	2548 (0.033)
predatory	2	1485(0.08002743797873557)	1516 (0.069)	1233 (0.1)
predictor	2	710(0.19435234937692925)	1516 (0.069)	388 (0.36)
predyces簣	2	3437(0.0228649822796387З)	2359 (0.034)	2548 (0.033)
preeclampsia	2	3437(0.0228649822796387З)	2359 (0.034)	#SAKNAS!
pre-existing	2	3437(0.0228649822796387З)	2359 (0.034)	#SAKNAS!
pregnancies	2	3437(0.0228649822796387З)	#SAKNAS!	2548 (0.033)
prehistoric	2	2693(0.0342974734194581)	1516 (0.069)	2548 (0.033)
premature	2	3437(0.0228649822796387З)	#SAKNAS!	1668 (0.066)
prematurity	2	3437(0.0228649822796387З)	2359 (0.034)	#SAKNAS!
pre-operative	2	3437(0.0228649822796387З)	#SAKNAS!	#SAKNAS!
preparation	2	966(0.1371898936778324)	1516 (0.069)	999 (0.13)
pre-pregnancy	2	3437(0.0228649822796387З)	#SAKNAS!	#SAKNAS!
presbytis	2	3437(0.0228649822796387З)	#SAKNAS!	2548 (0.033)
preschool-aged	2	1894(0.05716245569909683)	1516 (0.069)	2548 (0.033)
preschool-children	2	3437(0.0228649822796387З)	2359 (0.034)	#SAKNAS!
preserves	2	3437(0.0228649822796387З)	#SAKNAS!	1668 (0.066)
pressures	2	3437(0.0228649822796387З)	#SAKNAS!	1668 (0.066)
prevalent	2	966(0.1371898936778324)	1516 (0.069)	694 (0.2)
preventative	2	3437(0.0228649822796387З)	2359 (0.034)	#SAKNAS!
previously	2	2214(0.04572996455927746)	1516 (0.069)	2548 (0.033)
prevotella	2	3437(0.0228649822796387З)	2359 (0.034)	2548 (0.033)
probabilistic	2	3437(0.0228649822796387З)	#SAKNAS!	2548 (0.033)
probable	2	3437(0.0228649822796387З)	#SAKNAS!	1668 (0.066)
probiotic	2	3437(0.0228649822796387З)	#SAKNAS!	2548 (0.033)
probiotics	2	1894(0.05716245569909683)	1516 (0.069)	2548 (0.033)
procera	2	3437(0.0228649822796387З)	2359 (0.034)	2548 (0.033)
processing	2	1027(0.12575740253801304)	1516 (0.069)	1233 (0.1)
produce	2	3437(0.0228649822796387З)	2359 (0.034)	#SAKNAS!
producer	2	3437(0.0228649822796387З)	1516 (0.069)	#SAKNAS!
producer	2	3437(0.0228649822796387З)	1516 (0.069)	#SAKNAS!
produces	2	3437(0.0228649822796387З)	2359 (0.034)	2548 (0.033)
product	2	1485(0.08002743797873557)	1516 (0.069)	#SAKNAS!
professors	2	3437(0.0228649822796387З)	#SAKNAS!	1668 (0.066)
profitability	2	3437(0.0228649822796387З)	2359 (0.034)	#SAKNAS!
profunda	2	3437(0.0228649822796387З)	#SAKNAS!	#SAKNAS!
prognosis	2	535(0.2515148050760261)	1516 (0.069)	584 (0.23)
programming	2	3437(0.0228649822796387З)	1516 (0.069)	#SAKNAS!
programming	2	3437(0.0228649822796387З)	1516 (0.069)	#SAKNAS!
pro-inflammatory	2	3437(0.0228649822796387З)	2359 (0.034)	2548 (0.033)
projections	2	3437(0.0228649822796387З)	#SAKNAS!	1668 (0.066)

promotes	2	1485(0.08002743797873557)	1516 (0.069)	1233 (0.1)
promoting	2	1111(0.11432491139819366)	1516 (0.069)	1668 (0.066)
proopiomelanocortin	2	3437(0.02286498227963873)	#SAKNAS!	2548 (0.033)
propionate	2	3437(0.02286498227963873)	2359 (0.034)	2548 (0.033)
propionic	2	3437(0.02286498227963873)	#SAKNAS!	1668 (0.066)
propofol	2	3437(0.02286498227963873)	#SAKNAS!	2548 (0.033)
propolis	2	3437(0.02286498227963873)	2359 (0.034)	2548 (0.033)
proportion	2	3437(0.02286498227963873)	2359 (0.034)	#SAKNAS!
prostaglandin	2	3437(0.02286498227963873)	2359 (0.034)	#SAKNAS!
protease	2	3437(0.02286498227963873)	2359 (0.034)	2548 (0.033)
protect	2	3437(0.02286498227963873)	#SAKNAS!	1668 (0.066)
protecting	2	3437(0.02286498227963873)	#SAKNAS!	1668 (0.066)
proteins	2	1894(0.05716245569909683)	1516 (0.069)	1668 (0.066)
proteome	2	3437(0.02286498227963873)	2359 (0.034)	#SAKNAS!
protocols	2	1894(0.05716245569909683)	1516 (0.069)	2548 (0.033)
provides	2	2693(0.0342974734194581)	1516 (0.069)	2548 (0.033)
proximate	2	3437(0.02286498227963873)	#SAKNAS!	2548 (0.033)
proximity	2	3437(0.02286498227963873)	2359 (0.034)	#SAKNAS!
proxy	2	3437(0.02286498227963873)	#SAKNAS!	#SAKNAS!
pruni	2	3437(0.02286498227963873)	#SAKNAS!	1668 (0.066)
prymnesium	2	3437(0.02286498227963873)	1516 (0.069)	#SAKNAS!
prymnesium	2	3437(0.02286498227963873)	1516 (0.069)	#SAKNAS!
pseudomonas	2	3437(0.02286498227963873)	2359 (0.034)	#SAKNAS!
psychopathology	2	3437(0.02286498227963873)	2359 (0.034)	#SAKNAS!
psychosis	2	3437(0.02286498227963873)	2359 (0.034)	#SAKNAS!
psychosocial	2	1894(0.05716245569909683)	1516 (0.069)	1668 (0.066)
pta	2	3437(0.02286498227963873)	#SAKNAS!	2548 (0.033)
puberty	2	3437(0.02286498227963873)	2359 (0.034)	2548 (0.033)
puerto	2	1678(0.0685949468389162)	1516 (0.069)	999 (0.13)
puntazzo	2	3437(0.02286498227963873)	1516 (0.069)	#SAKNAS!
puntazzo	2	3437(0.02286498227963873)	1516 (0.069)	#SAKNAS!
pupils	2	2214(0.04572996455927746)	1516 (0.069)	2548 (0.033)
purchasing	2	3437(0.02286498227963873)	1516 (0.069)	#SAKNAS!
purchasing	2	3437(0.02286498227963873)	1516 (0.069)	#SAKNAS!
purposes	2	3437(0.02286498227963873)	2359 (0.034)	#SAKNAS!
pursuant	2	3437(0.02286498227963873)	#SAKNAS!	1668 (0.066)
putative	2	3437(0.02286498227963873)	#SAKNAS!	1668 (0.066)
putting	2	3437(0.02286498227963873)	1516 (0.069)	#SAKNAS!
putting	2	3437(0.02286498227963873)	1516 (0.069)	#SAKNAS!
pycnogenol簣	2	3437(0.02286498227963873)	#SAKNAS!	#SAKNAS!
pyramid	2	3437(0.02286498227963873)	2359 (0.034)	2548 (0.033)
pyy	2	3437(0.02286498227963873)	#SAKNAS!	2548 (0.033)
quadriceps	2	3437(0.02286498227963873)	#SAKNAS!	2548 (0.033)

quail	2	3437(0.0228649822796387З)	1516 (0.069)	#SAKNAS!
quail	2	3437(0.0228649822796387З)	1516 (0.069)	#SAKNAS!
quality-of-life	2	2693(0.0342974734194581)	1516 (0.069)	#SAKNAS!
quantifying	2	2693(0.0342974734194581)	1516 (0.069)	#SAKNAS!
quebec	2	3437(0.0228649822796387З)	2359 (0.034)	2548 (0.033)
questionnaires	2	3437(0.0228649822796387З)	#SAKNAS!	2548 (0.033)
quick	2	3437(0.0228649822796387З)	2359 (0.034)	2548 (0.033)
rabbit	2	3437(0.0228649822796387З)	2359 (0.034)	2548 (0.033)
rabbits	2	3437(0.0228649822796387З)	1516 (0.069)	#SAKNAS!
rabbits	2	3437(0.0228649822796387З)	1516 (0.069)	#SAKNAS!
races	2	3437(0.0228649822796387З)	2359 (0.034)	#SAKNAS!
racial	2	1485(0.08002743797873557)	1516 (0.069)	1668 (0.066)
radial	2	3437(0.0228649822796387З)	1516 (0.069)	#SAKNAS!
radial	2	3437(0.0228649822796387З)	1516 (0.069)	#SAKNAS!
radical	2	3437(0.0228649822796387З)	2359 (0.034)	#SAKNAS!
radiographic	2	1894(0.0571624556990968З)	1516 (0.069)	1668 (0.066)
radiographically	2	3437(0.0228649822796387З)	#SAKNAS!	2548 (0.033)
radiologic	2	1678(0.0685949468389162)	1516 (0.069)	1668 (0.066)
ramadan	2	3437(0.0228649822796387З)	1516 (0.069)	#SAKNAS!
ramadan	2	3437(0.0228649822796387З)	1516 (0.069)	#SAKNAS!
random	2	3437(0.0228649822796387З)	2359 (0.034)	2548 (0.033)
range	2	1894(0.0571624556990968З)	1516 (0.069)	1668 (0.066)
rangelands	2	3437(0.0228649822796387З)	#SAKNAS!	2548 (0.033)
ranges	2	3437(0.0228649822796387З)	1516 (0.069)	#SAKNAS!
ranges	2	3437(0.0228649822796387З)	1516 (0.069)	#SAKNAS!
rank	2	3437(0.0228649822796387З)	2359 (0.034)	2548 (0.033)
ration	2	1678(0.0685949468389162)	1516 (0.069)	1233 (0.1)
rations	2	3437(0.0228649822796387З)	2359 (0.034)	#SAKNAS!
really	2	2693(0.0342974734194581)	1516 (0.069)	2548 (0.033)
reassessment	2	3437(0.0228649822796387З)	#SAKNAS!	2548 (0.033)
receipt	2	2214(0.04572996455927746)	1516 (0.069)	1668 (0.066)
receptors	2	896(0.14862238481765175)	1516 (0.069)	694 (0.2)
recession	2	2214(0.04572996455927746)	1516 (0.069)	2548 (0.033)
recognition	2	3437(0.0228649822796387З)	#SAKNAS!	#SAKNAS!
recommendation	2	3437(0.0228649822796387З)	#SAKNAS!	2548 (0.033)
recommending	2	3437(0.0228649822796387З)	#SAKNAS!	2548 (0.033)
recording	2	2214(0.04572996455927746)	1516 (0.069)	2548 (0.033)
records	2	3437(0.0228649822796387З)	#SAKNAS!	2548 (0.033)
records-based	2	3437(0.0228649822796387З)	#SAKNAS!	2548 (0.033)
recreational	2	3437(0.0228649822796387З)	#SAKNAS!	2548 (0.033)
recurrence	2	1219(0.1028924202583743)	1516 (0.069)	694 (0.2)
redox	2	3437(0.0228649822796387З)	2359 (0.034)	#SAKNAS!
reef	2	3437(0.0228649822796387З)	#SAKNAS!	2548 (0.033)

reference	2	3437(0.0228649822796387З)	2359 (0.034)	#SAKNAS!
reflect	2	1894(0.0571624556990968З)	1516 (0.069)	2548 (0.033)
reflection	2	3437(0.0228649822796387З)	2359 (0.034)	2548 (0.033)
refugee	2	1678(0.0685949468389162)	1516 (0.069)	1233 (0.1)
regimen	2	3437(0.0228649822796387З)	#SAKNAS!	#SAKNAS!
regimes	2	3437(0.0228649822796387З)	1516 (0.069)	#SAKNAS!
regimes	2	3437(0.0228649822796387З)	1516 (0.069)	#SAKNAS!
regional	2	1485(0.08002743797873557)	1516 (0.069)	999 (0.13)
register	2	3437(0.0228649822796387З)	#SAKNAS!	#SAKNAS!
regius	2	3437(0.0228649822796387З)	#SAKNAS!	#SAKNAS!
regulations	2	3437(0.0228649822796387З)	2359 (0.034)	#SAKNAS!
regulator	2	3437(0.0228649822796387З)	2359 (0.034)	#SAKNAS!
regurgitation	2	3437(0.0228649822796387З)	2359 (0.034)	#SAKNAS!
reinforcement	2	2214(0.0457299645592774б)	1516 (0.069)	1668 (0.066)
relations	2	1894(0.0571624556990968З)	1516 (0.069)	1233 (0.1)
release	2	2214(0.0457299645592774б)	1516 (0.069)	2548 (0.033)
relief	2	3437(0.0228649822796387З)	#SAKNAS!	2548 (0.033)
remaining	2	1027(0.12575740253801304)	1516 (0.069)	816 (0.16)
remains	2	3437(0.0228649822796387З)	2359 (0.034)	#SAKNAS!
remote	2	3437(0.0228649822796387З)	#SAKNAS!	1668 (0.066)
removable	2	1894(0.0571624556990968З)	1516 (0.069)	2548 (0.033)
repeat	2	3437(0.0228649822796387З)	#SAKNAS!	2548 (0.033)
reports	2	1894(0.0571624556990968З)	1516 (0.069)	2548 (0.033)
representations	2	2693(0.0342974734194581)	1516 (0.069)	2548 (0.033)
republic	2	1678(0.0685949468389162)	1516 (0.069)	1233 (0.1)
require	2	3437(0.0228649822796387З)	2359 (0.034)	2548 (0.033)
requirements	2	3437(0.0228649822796387З)	2359 (0.034)	2548 (0.033)
resectable	2	3437(0.0228649822796387З)	2359 (0.034)	2548 (0.033)
reserve	2	3437(0.0228649822796387З)	2359 (0.034)	#SAKNAS!
residence	2	3437(0.0228649822796387З)	2359 (0.034)	#SAKNAS!
residing	2	2214(0.0457299645592774б)	1516 (0.069)	#SAKNAS!
residual	2	1894(0.0571624556990968З)	1516 (0.069)	2548 (0.033)
residues	2	3437(0.0228649822796387З)	2359 (0.034)	#SAKNAS!
resistant	2	3437(0.0228649822796387З)	2359 (0.034)	2548 (0.033)
resolution	2	3437(0.0228649822796387З)	#SAKNAS!	2548 (0.033)
resorption	2	3437(0.0228649822796387З)	2359 (0.034)	#SAKNAS!
respiration	2	3437(0.0228649822796387З)	2359 (0.034)	2548 (0.033)
responders	2	3437(0.0228649822796387З)	2359 (0.034)	#SAKNAS!
rest	2	3437(0.0228649822796387З)	2359 (0.034)	2548 (0.033)
resting	2	3437(0.0228649822796387З)	#SAKNAS!	1668 (0.066)
restorations	2	2214(0.0457299645592774б)	1516 (0.069)	1668 (0.066)
restorative	2	2693(0.0342974734194581)	1516 (0.069)	2548 (0.033)
restores	2	3437(0.0228649822796387З)	#SAKNAS!	2548 (0.033)

restrictive	2	1335(0.09145992911855493)	1516 (0.069)	1233 (0.1)
resumption	2	3437(0.02286498227963873)	2359 (0.034)	2548 (0.033)
retardants	2	3437(0.02286498227963873)	#SAKNAS!	#SAKNAS!
retinol	2	3437(0.02286498227963873)	#SAKNAS!	#SAKNAS!
retirement	2	3437(0.02286498227963873)	1516 (0.069)	#SAKNAS!
retirement	2	3437(0.02286498227963873)	1516 (0.069)	#SAKNAS!
retort	2	3437(0.02286498227963873)	#SAKNAS!	#SAKNAS!
retrosternal	2	3437(0.02286498227963873)	1516 (0.069)	#SAKNAS!
retrosternal	2	3437(0.02286498227963873)	1516 (0.069)	#SAKNAS!
return	2	3437(0.02286498227963873)	2359 (0.034)	2548 (0.033)
revisional	2	3437(0.02286498227963873)	2359 (0.034)	#SAKNAS!
rewarding	2	3437(0.02286498227963873)	#SAKNAS!	1668 (0.066)
rewards	2	3437(0.02286498227963873)	2359 (0.034)	2548 (0.033)
rheology	2	2214(0.04572996455927746)	1516 (0.069)	#SAKNAS!
rhesus	2	3437(0.02286498227963873)	2359 (0.034)	2548 (0.033)
rhesus-monkeys	2	3437(0.02286498227963873)	1516 (0.069)	#SAKNAS!
rhesus-monkeys	2	3437(0.02286498227963873)	1516 (0.069)	#SAKNAS!
rheumatic	2	3437(0.02286498227963873)	2359 (0.034)	2548 (0.033)
rhinella	2	3437(0.02286498227963873)	#SAKNAS!	2548 (0.033)
rhinoceroses	2	3437(0.02286498227963873)	2359 (0.034)	2548 (0.033)
rhinopithecus	2	3437(0.02286498227963873)	#SAKNAS!	1668 (0.066)
rhythm	2	2214(0.04572996455927746)	1516 (0.069)	2548 (0.033)
rican	2	3437(0.02286498227963873)	#SAKNAS!	1668 (0.066)
rice	2	1485(0.08002743797873557)	1516 (0.069)	#SAKNAS!
rich	2	3437(0.02286498227963873)	#SAKNAS!	1668 (0.066)
rico	2	2214(0.04572996455927746)	1516 (0.069)	1668 (0.066)
ring	2	3437(0.02286498227963873)	#SAKNAS!	2548 (0.033)
rings	2	3437(0.02286498227963873)	1516 (0.069)	#SAKNAS!
rings	2	3437(0.02286498227963873)	1516 (0.069)	#SAKNAS!
risky	2	3437(0.02286498227963873)	2359 (0.034)	#SAKNAS!
rna	2	3437(0.02286498227963873)	#SAKNAS!	2548 (0.033)
robot	2	3437(0.02286498227963873)	#SAKNAS!	2548 (0.033)
rodents	2	3437(0.02286498227963873)	#SAKNAS!	1668 (0.066)
roe	2	3437(0.02286498227963873)	2359 (0.034)	#SAKNAS!
roman	2	3437(0.02286498227963873)	2359 (0.034)	2548 (0.033)
rossetti	2	3437(0.02286498227963873)	#SAKNAS!	1668 (0.066)
rotationally	2	3437(0.02286498227963873)	#SAKNAS!	1668 (0.066)
routine	2	1219(0.1028924202583743)	1516 (0.069)	999 (0.13)
roux	2	3437(0.02286498227963873)	#SAKNAS!	1668 (0.066)
rtog	2	3437(0.02286498227963873)	2359 (0.034)	2548 (0.033)
rules	2	3437(0.02286498227963873)	#SAKNAS!	2548 (0.033)
ruminant	2	3437(0.02286498227963873)	2359 (0.034)	2548 (0.033)
rumination	2	3437(0.02286498227963873)	#SAKNAS!	#SAKNAS!

sacc	2	3437(0.02286498227963873)	#SAKNAS!	1668 (0.066)
saccharomyces	2	3437(0.02286498227963873)	#SAKNAS!	2548 (0.033)
salmo	2	3437(0.02286498227963873)	2359 (0.034)	#SAKNAS!
salt	2	1894(0.05716245569909683)	1516 (0.069)	1668 (0.066)
salts	2	3437(0.02286498227963873)	2359 (0.034)	#SAKNAS!
san	2	1894(0.05716245569909683)	1516 (0.069)	1233 (0.1)
sara	2	3437(0.02286498227963873)	2359 (0.034)	2548 (0.033)
sardinian	2	3437(0.02286498227963873)	#SAKNAS!	#SAKNAS!
satiation	2	3437(0.02286498227963873)	#SAKNAS!	1668 (0.066)
satiety	2	1485(0.08002743797873557)	1516 (0.069)	1233 (0.1)
saudi	2	481(0.2858122784954842)	1516 (0.069)	461 (0.3)
scallop	2	3437(0.02286498227963873)	2359 (0.034)	2548 (0.033)
scavenger	2	3437(0.02286498227963873)	1516 (0.069)	#SAKNAS!
scavenger	2	3437(0.02286498227963873)	1516 (0.069)	#SAKNAS!
schatzki	2	3437(0.02286498227963873)	1516 (0.069)	#SAKNAS!
schatzki	2	3437(0.02286498227963873)	1516 (0.069)	#SAKNAS!
schweinfurthii	2	3437(0.02286498227963873)	#SAKNAS!	1668 (0.066)
sciences	2	3437(0.02286498227963873)	#SAKNAS!	2548 (0.033)
scoring	2	3437(0.02286498227963873)	2359 (0.034)	2548 (0.033)
scottish	2	3437(0.02286498227963873)	1516 (0.069)	#SAKNAS!
scottish	2	3437(0.02286498227963873)	1516 (0.069)	#SAKNAS!
seabream	2	3437(0.02286498227963873)	1516 (0.069)	#SAKNAS!
seabream	2	3437(0.02286498227963873)	1516 (0.069)	#SAKNAS!
seagrass	2	3437(0.02286498227963873)	#SAKNAS!	#SAKNAS!
sealing	2	3437(0.02286498227963873)	#SAKNAS!	2548 (0.033)
search	2	3437(0.02286498227963873)	#SAKNAS!	#SAKNAS!
seaweed	2	3437(0.02286498227963873)	2359 (0.034)	#SAKNAS!
second-generation	2	3437(0.02286498227963873)	1516 (0.069)	#SAKNAS!
second-generation	2	3437(0.02286498227963873)	1516 (0.069)	#SAKNAS!
sedation	2	3437(0.02286498227963873)	#SAKNAS!	#SAKNAS!
sediment	2	2693(0.0342974734194581)	1516 (0.069)	#SAKNAS!
seeds	2	3437(0.02286498227963873)	#SAKNAS!	1668 (0.066)
seeking	2	3437(0.02286498227963873)	2359 (0.034)	2548 (0.033)
seen	2	2693(0.0342974734194581)	1516 (0.069)	#SAKNAS!
segmentation	2	3437(0.02286498227963873)	2359 (0.034)	2548 (0.033)
segregation	2	2693(0.0342974734194581)	1516 (0.069)	#SAKNAS!
select	2	3437(0.02286498227963873)	#SAKNAS!	2548 (0.033)
self-assessment	2	3437(0.02286498227963873)	#SAKNAS!	#SAKNAS!
self-expandable	2	1894(0.05716245569909683)	1516 (0.069)	1668 (0.066)
self-feeders	2	3437(0.02286498227963873)	1516 (0.069)	#SAKNAS!
self-feeders	2	3437(0.02286498227963873)	1516 (0.069)	#SAKNAS!
self-medication	2	2693(0.0342974734194581)	1516 (0.069)	2548 (0.033)
self-regulation	2	3437(0.02286498227963873)	2359 (0.034)	2548 (0.033)

semiarid	2	3437(0.0228649822796387З)	2359 (0.034)	#SAKNAS!
semi-arid	2	3437(0.0228649822796387З)	2359 (0.034)	#SAKNAS!
semi-intensive	2	2693(0.0342974734194581)	1516 (0.069)	#SAKNAS!
semi-solid	2	3437(0.0228649822796387З)	#SAKNAS!	2548 (0.033)
semisynthetic	2	3437(0.0228649822796387З)	2359 (0.034)	#SAKNAS!
senegalensis	2	3437(0.0228649822796387З)	1516 (0.069)	#SAKNAS!
senegalensis	2	3437(0.0228649822796387З)	1516 (0.069)	#SAKNAS!
senegalese	2	3437(0.0228649822796387З)	2359 (0.034)	2548 (0.033)
senescence	2	3437(0.0228649822796387З)	#SAKNAS!	2548 (0.033)
senior	2	3437(0.0228649822796387З)	#SAKNAS!	2548 (0.033)
sensitivity	2	710(0.19435234937692925)	1516 (0.069)	461 (0.3)
sensor	2	3437(0.0228649822796387З)	2359 (0.034)	2548 (0.033)
sensors	2	3437(0.0228649822796387З)	#SAKNAS!	#SAKNAS!
separation	2	2693(0.0342974734194581)	1516 (0.069)	2548 (0.033)
sequential	2	2693(0.0342974734194581)	1516 (0.069)	2548 (0.033)
serbian	2	3437(0.0228649822796387З)	#SAKNAS!	1668 (0.066)
serotonergic	2	3437(0.0228649822796387З)	2359 (0.034)	#SAKNAS!
serving	2	3437(0.0228649822796387З)	#SAKNAS!	#SAKNAS!
sesarmidae	2	3437(0.0228649822796387З)	2359 (0.034)	2548 (0.033)
severely	2	2214(0.04572996455927746)	1516 (0.069)	#SAKNAS!
shanghai	2	1335(0.09145992911855493)	1516 (0.069)	2548 (0.033)
shape	2	1335(0.09145992911855493)	1516 (0.069)	1668 (0.066)
shaping	2	3437(0.0228649822796387З)	2359 (0.034)	#SAKNAS!
shark	2	3437(0.0228649822796387З)	#SAKNAS!	2548 (0.033)
sharpi	2	3437(0.0228649822796387З)	2359 (0.034)	2548 (0.033)
sharpsnout	2	3437(0.0228649822796387З)	1516 (0.069)	#SAKNAS!
sharpsnout	2	3437(0.0228649822796387З)	1516 (0.069)	#SAKNAS!
shear	2	3437(0.0228649822796387З)	#SAKNAS!	2548 (0.033)
shearwater	2	3437(0.0228649822796387З)	2359 (0.034)	2548 (0.033)
shellfish	2	3437(0.0228649822796387З)	2359 (0.034)	2548 (0.033)
shelter	2	2693(0.0342974734194581)	1516 (0.069)	#SAKNAS!
shika	2	3437(0.0228649822796387З)	#SAKNAS!	#SAKNAS!
short-chain	2	3437(0.0228649822796387З)	2359 (0.034)	#SAKNAS!
shorter	2	3437(0.0228649822796387З)	2359 (0.034)	#SAKNAS!
shrimps	2	3437(0.0228649822796387З)	#SAKNAS!	1668 (0.066)
siberian	2	3437(0.0228649822796387З)	#SAKNAS!	2548 (0.033)
sibling	2	3437(0.0228649822796387З)	#SAKNAS!	1668 (0.066)
siblings	2	3437(0.0228649822796387З)	1516 (0.069)	#SAKNAS!
siblings	2	3437(0.0228649822796387З)	1516 (0.069)	#SAKNAS!
sicca	2	3437(0.0228649822796387З)	#SAKNAS!	2548 (0.033)
signal	2	1894(0.05716245569909683)	1516 (0.069)	1233 (0.1)
signaling	2	743(0.18291985823710985)	1516 (0.069)	461 (0.3)
signature	2	3437(0.0228649822796387З)	#SAKNAS!	#SAKNAS!

silage	2	1111(0.11432491139819366)	1516 (0.069)	694 (0.2)
silicone	2	3437(0.02286498227963873)	1516 (0.069)	#SAKNAS!
silicone	2	3437(0.02286498227963873)	1516 (0.069)	#SAKNAS!
silkworm	2	2693(0.0342974734194581)	1516 (0.069)	#SAKNAS!
simplex	2	2214(0.04572996455927746)	1516 (0.069)	2548 (0.033)
sinensis	2	3437(0.02286498227963873)	#SAKNAS!	#SAKNAS!
singapore	2	3437(0.02286498227963873)	#SAKNAS!	2548 (0.033)
single-arm	2	3437(0.02286498227963873)	#SAKNAS!	#SAKNAS!
single-blind	2	3437(0.02286498227963873)	#SAKNAS!	2548 (0.033)
single-institution	2	3437(0.02286498227963873)	#SAKNAS!	#SAKNAS!
single-session	2	3437(0.02286498227963873)	1516 (0.069)	#SAKNAS!
single-session	2	3437(0.02286498227963873)	1516 (0.069)	#SAKNAS!
sites	2	1894(0.05716245569909683)	1516 (0.069)	2548 (0.033)
sitobion	2	3437(0.02286498227963873)	#SAKNAS!	2548 (0.033)
sixth	2	3437(0.02286498227963873)	#SAKNAS!	2548 (0.033)
sixth-instar	2	3437(0.02286498227963873)	1516 (0.069)	#SAKNAS!
sixth-instar	2	3437(0.02286498227963873)	1516 (0.069)	#SAKNAS!
sizes	2	3437(0.02286498227963873)	#SAKNAS!	2548 (0.033)
skeletons	2	3437(0.02286498227963873)	1516 (0.069)	#SAKNAS!
skeletons	2	3437(0.02286498227963873)	1516 (0.069)	#SAKNAS!
skill	2	3437(0.02286498227963873)	#SAKNAS!	2548 (0.033)
skull	2	2214(0.04572996455927746)	1516 (0.069)	#SAKNAS!
sleepiness	2	3437(0.02286498227963873)	#SAKNAS!	2548 (0.033)
slow-growing	2	3437(0.02286498227963873)	1516 (0.069)	#SAKNAS!
slow-growing	2	3437(0.02286498227963873)	1516 (0.069)	#SAKNAS!
snub-nosed	2	3437(0.02286498227963873)	#SAKNAS!	1668 (0.066)
socially	2	2693(0.0342974734194581)	1516 (0.069)	#SAKNAS!
societies	2	3437(0.02286498227963873)	2359 (0.034)	#SAKNAS!
society	2	1111(0.11432491139819366)	1516 (0.069)	999 (0.13)
socio-behavioral	2	3437(0.02286498227963873)	1516 (0.069)	#SAKNAS!
socio-behavioral	2	3437(0.02286498227963873)	1516 (0.069)	#SAKNAS!
socio-demographic	2	1485(0.08002743797873557)	1516 (0.069)	999 (0.13)
soda	2	3437(0.02286498227963873)	1516 (0.069)	#SAKNAS!
soda	2	3437(0.02286498227963873)	1516 (0.069)	#SAKNAS!
soldier	2	3437(0.02286498227963873)	#SAKNAS!	#SAKNAS!
solea	2	3437(0.02286498227963873)	1516 (0.069)	#SAKNAS!
solea	2	3437(0.02286498227963873)	1516 (0.069)	#SAKNAS!
solenopsis	2	3437(0.02286498227963873)	1516 (0.069)	#SAKNAS!
solenopsis	2	3437(0.02286498227963873)	1516 (0.069)	#SAKNAS!
solitary	2	2214(0.04572996455927746)	1516 (0.069)	1668 (0.066)
soluble	2	3437(0.02286498227963873)	#SAKNAS!	1668 (0.066)
somatic	2	3437(0.02286498227963873)	#SAKNAS!	2548 (0.033)
sorting	2	3437(0.02286498227963873)	#SAKNAS!	1668 (0.066)

sow	2	3437(0.02286498227963873)	2359 (0.034)	#SAKNAS!
spasm	2	3437(0.02286498227963873)	#SAKNAS!	#SAKNAS!
spastic	2	3437(0.02286498227963873)	#SAKNAS!	1668 (0.066)
spatiotemporal	2	3437(0.02286498227963873)	#SAKNAS!	2548 (0.033)
specialization	2	3437(0.02286498227963873)	2359 (0.034)	2548 (0.033)
spectrometry	2	3437(0.02286498227963873)	#SAKNAS!	2548 (0.033)
spectrum	2	671(0.2057848405167486)	1516 (0.069)	388 (0.36)
spider	2	2214(0.04572996455927746)	1516 (0.069)	1668 (0.066)
spineless	2	3437(0.02286498227963873)	#SAKNAS!	1668 (0.066)
spinocerebellar	2	3437(0.02286498227963873)	2359 (0.034)	#SAKNAS!
spiny	2	1894(0.05716245569909683)	1516 (0.069)	1668 (0.066)
spondylitis	2	3437(0.02286498227963873)	2359 (0.034)	2548 (0.033)
spondylosis	2	3437(0.02286498227963873)	#SAKNAS!	1668 (0.066)
spondylotic	2	3437(0.02286498227963873)	2359 (0.034)	#SAKNAS!
sporadic	2	3437(0.02286498227963873)	#SAKNAS!	2548 (0.033)
spp	2	2214(0.04572996455927746)	1516 (0.069)	2548 (0.033)
spread	2	3437(0.02286498227963873)	2359 (0.034)	2548 (0.033)
squirrels	2	3437(0.02286498227963873)	2359 (0.034)	#SAKNAS!
sri	2	2214(0.04572996455927746)	1516 (0.069)	1668 (0.066)
st	2	3437(0.02286498227963873)	2359 (0.034)	2548 (0.033)
standards	2	2693(0.0342974734194581)	1516 (0.069)	2548 (0.033)
standing	2	3437(0.02286498227963873)	2359 (0.034)	#SAKNAS!
star	2	3437(0.02286498227963873)	1516 (0.069)	#SAKNAS!
star	2	3437(0.02286498227963873)	1516 (0.069)	#SAKNAS!
starchy	2	3437(0.02286498227963873)	#SAKNAS!	2548 (0.033)
start	2	3437(0.02286498227963873)	#SAKNAS!	2548 (0.033)
statistical	2	3437(0.02286498227963873)	#SAKNAS!	#SAKNAS!
statuses	2	3437(0.02286498227963873)	#SAKNAS!	2548 (0.033)
stay	2	1678(0.0685949468389162)	1516 (0.069)	1668 (0.066)
steady	2	3437(0.02286498227963873)	#SAKNAS!	1668 (0.066)
stenting	2	1894(0.05716245569909683)	1516 (0.069)	1668 (0.066)
stiffness	2	3437(0.02286498227963873)	#SAKNAS!	2548 (0.033)
stimulates	2	3437(0.02286498227963873)	2359 (0.034)	2548 (0.033)
stomach	2	1678(0.0685949468389162)	1516 (0.069)	1233 (0.1)
stomatitis	2	1894(0.05716245569909683)	1516 (0.069)	2548 (0.033)
strata	2	3437(0.02286498227963873)	#SAKNAS!	2548 (0.033)
straw	2	2214(0.04572996455927746)	1516 (0.069)	2548 (0.033)
streams	2	2693(0.0342974734194581)	1516 (0.069)	#SAKNAS!
striatum	2	3437(0.02286498227963873)	#SAKNAS!	1668 (0.066)
striatus	2	3437(0.02286498227963873)	#SAKNAS!	1668 (0.066)
stricta	2	3437(0.02286498227963873)	#SAKNAS!	1668 (0.066)
strombus	2	3437(0.02286498227963873)	1516 (0.069)	#SAKNAS!
strombus	2	3437(0.02286498227963873)	1516 (0.069)	#SAKNAS!

student	2	2693(0.0342974734194581)	1516 (0.069)	2548 (0.033)
stunting	2	1485(0.08002743797873557)	1516 (0.069)	1668 (0.066)
style	2	1335(0.09145992911855493)	1516 (0.069)	694 (0.2)
subacute	2	966(0.1371898936778324)	1516 (0.069)	816 (0.16)
sub-acute	2	3437(0.02286498227963873)	#SAKNAS!	2548 (0.033)
subarachnoid	2	3437(0.02286498227963873)	2359 (0.034)	#SAKNAS!
sub-clinical	2	3437(0.02286498227963873)	1516 (0.069)	#SAKNAS!
sub-clinical	2	3437(0.02286498227963873)	1516 (0.069)	#SAKNAS!
submucosal	2	3437(0.02286498227963873)	2359 (0.034)	#SAKNAS!
subsequent	2	1335(0.09145992911855493)	1516 (0.069)	1668 (0.066)
substance	2	3437(0.02286498227963873)	2359 (0.034)	2548 (0.033)
substitutes	2	3437(0.02286498227963873)	#SAKNAS!	2548 (0.033)
substrate	2	2693(0.0342974734194581)	1516 (0.069)	2548 (0.033)
subtilis	2	3437(0.02286498227963873)	1516 (0.069)	#SAKNAS!
subtilis	2	3437(0.02286498227963873)	1516 (0.069)	#SAKNAS!
subtotal	2	3437(0.02286498227963873)	#SAKNAS!	#SAKNAS!
subtropical	2	3437(0.02286498227963873)	#SAKNAS!	1668 (0.066)
subtype	2	2693(0.0342974734194581)	1516 (0.069)	2548 (0.033)
suburban	2	3437(0.02286498227963873)	2359 (0.034)	2548 (0.033)
sucking	2	3437(0.02286498227963873)	#SAKNAS!	1668 (0.066)
sudan	2	3437(0.02286498227963873)	#SAKNAS!	2548 (0.033)
suffering	2	2693(0.0342974734194581)	1516 (0.069)	2548 (0.033)
sugar-free	2	3437(0.02286498227963873)	#SAKNAS!	1668 (0.066)
sugary	2	3437(0.02286498227963873)	#SAKNAS!	#SAKNAS!
suitability	2	2214(0.04572996455927746)	1516 (0.069)	1668 (0.066)
summary	2	2693(0.0342974734194581)	1516 (0.069)	#SAKNAS!
superselective	2	3437(0.02286498227963873)	2359 (0.034)	2548 (0.033)
supplemental	2	1485(0.08002743797873557)	1516 (0.069)	2548 (0.033)
supporting	2	3437(0.02286498227963873)	#SAKNAS!	#SAKNAS!
supports	2	3437(0.02286498227963873)	#SAKNAS!	2548 (0.033)
suppressive	2	3437(0.02286498227963873)	1516 (0.069)	#SAKNAS!
suppressive	2	3437(0.02286498227963873)	1516 (0.069)	#SAKNAS!
supracricoid	2	2693(0.0342974734194581)	1516 (0.069)	2548 (0.033)
supraglottic	2	3437(0.02286498227963873)	2359 (0.034)	#SAKNAS!
surgically	2	3437(0.02286498227963873)	2359 (0.034)	2548 (0.033)
survey-based	2	3437(0.02286498227963873)	#SAKNAS!	2548 (0.033)
surviving	2	3437(0.02286498227963873)	2359 (0.034)	2548 (0.033)
survivorship	2	3437(0.02286498227963873)	#SAKNAS!	1668 (0.066)
suspension-feeding	2	3437(0.02286498227963873)	2359 (0.034)	2548 (0.033)
suzukii	2	3437(0.02286498227963873)	#SAKNAS!	1668 (0.066)
swal-qol	2	2693(0.0342974734194581)	1516 (0.069)	#SAKNAS!
sward	2	2693(0.0342974734194581)	1516 (0.069)	#SAKNAS!
sweets	2	3437(0.02286498227963873)	2359 (0.034)	2548 (0.033)

swine	2	3437(0.0228649822796387З)	2359 (0.034)	#SAKNAS!
switch	2	2214(0.04572996455927746)	1516 (0.069)	2548 (0.033)
symbionts	2	3437(0.02286498227963873)	2359 (0.034)	2548 (0.033)
synaptic	2	3437(0.02286498227963873)	2359 (0.034)	2548 (0.033)
synchronous	2	3437(0.02286498227963873)	2359 (0.034)	2548 (0.033)
synthetic	2	3437(0.02286498227963873)	2359 (0.034)	2548 (0.033)
taberu	2	3437(0.02286498227963873)	#SAKNAS!	1668 (0.066)
tactile	2	3437(0.02286498227963873)	1516 (0.069)	#SAKNAS!
tactile	2	3437(0.02286498227963873)	1516 (0.069)	#SAKNAS!
take	2	3437(0.02286498227963873)	#SAKNAS!	2548 (0.033)
taking	2	3437(0.02286498227963873)	#SAKNAS!	2548 (0.033)
talk	2	3437(0.02286498227963873)	#SAKNAS!	2548 (0.033)
talking	2	3437(0.02286498227963873)	#SAKNAS!	1668 (0.066)
tambaqui	2	3437(0.02286498227963873)	#SAKNAS!	1668 (0.066)
tannerella	2	2693(0.0342974734194581)	1516 (0.069)	#SAKNAS!
tannin	2	3437(0.02286498227963873)	2359 (0.034)	2548 (0.033)
tannin-rich	2	3437(0.02286498227963873)	2359 (0.034)	2548 (0.033)
tanzanian	2	3437(0.02286498227963873)	1516 (0.069)	#SAKNAS!
tanzanian	2	3437(0.02286498227963873)	1516 (0.069)	#SAKNAS!
tasteless	2	3437(0.02286498227963873)	#SAKNAS!	2548 (0.033)
taurine	2	3437(0.02286498227963873)	2359 (0.034)	2548 (0.033)
taxes	2	3437(0.02286498227963873)	#SAKNAS!	2548 (0.033)
t-cell	2	3437(0.02286498227963873)	2359 (0.034)	#SAKNAS!
tdcs	2	3437(0.02286498227963873)	#SAKNAS!	2548 (0.033)
teaching	2	3437(0.02286498227963873)	#SAKNAS!	#SAKNAS!
teams	2	3437(0.02286498227963873)	2359 (0.034)	#SAKNAS!
technique	2	1219(0.1028924202583743)	1516 (0.069)	999 (0.13)
tegmental	2	3437(0.02286498227963873)	#SAKNAS!	2548 (0.033)
telangiectasia	2	3437(0.02286498227963873)	2359 (0.034)	2548 (0.033)
television	2	1894(0.05716245569909683)	1516 (0.069)	1233 (0.1)
temporomandibular	2	1678(0.0685949468389162)	1516 (0.069)	1668 (0.066)
tenebrio	2	3437(0.02286498227963873)	#SAKNAS!	2548 (0.033)
tension	2	3437(0.02286498227963873)	1516 (0.069)	#SAKNAS!
tension	2	3437(0.02286498227963873)	1516 (0.069)	#SAKNAS!
test-retest	2	3437(0.02286498227963873)	2359 (0.034)	2548 (0.033)
tetanus	2	3437(0.02286498227963873)	#SAKNAS!	1668 (0.066)
tetricus	2	3437(0.02286498227963873)	#SAKNAS!	#SAKNAS!
therapies	2	1678(0.0685949468389162)	1516 (0.069)	#SAKNAS!
thick	2	3437(0.02286498227963873)	2359 (0.034)	#SAKNAS!
thin	2	3437(0.02286498227963873)	2359 (0.034)	#SAKNAS!
thinness	2	3437(0.02286498227963873)	#SAKNAS!	1668 (0.066)
thiocyanate	2	3437(0.02286498227963873)	#SAKNAS!	2548 (0.033)
thixotropy	2	3437(0.02286498227963873)	2359 (0.034)	2548 (0.033)

thoracic	2	1219(0.1028924202583743)	1516 (0.069)	816 (0.16)
thoracoscopic	2	3437(0.02286498227963873)	#SAKNAS!	1668 (0.066)
threat	2	3437(0.02286498227963873)	#SAKNAS!	#SAKNAS!
threshold	2	3437(0.02286498227963873)	2359 (0.034)	2548 (0.033)
thuringiensis	2	2693(0.0342974734194581)	1516 (0.069)	2548 (0.033)
thymus	2	3437(0.02286498227963873)	2359 (0.034)	#SAKNAS!
thyroid	2	1678(0.0685949468389162)	1516 (0.069)	1668 (0.066)
tibetan	2	3437(0.02286498227963873)	#SAKNAS!	1668 (0.066)
tissues	2	3437(0.02286498227963873)	#SAKNAS!	1668 (0.066)
titicaca	2	3437(0.02286498227963873)	#SAKNAS!	2548 (0.033)
toddlerhood	2	3437(0.02286498227963873)	#SAKNAS!	2548 (0.033)
tokyo	2	3437(0.02286498227963873)	2359 (0.034)	2548 (0.033)
tolerable	2	3437(0.02286498227963873)	#SAKNAS!	2548 (0.033)
tolerance	2	1485(0.08002743797873557)	1516 (0.069)	1233 (0.1)
tomato	2	3437(0.02286498227963873)	1516 (0.069)	#SAKNAS!
tomato	2	3437(0.02286498227963873)	1516 (0.069)	#SAKNAS!
tomography	2	3437(0.02286498227963873)	1516 (0.069)	#SAKNAS!
tomography	2	3437(0.02286498227963873)	1516 (0.069)	#SAKNAS!
tomorrow	2	3437(0.02286498227963873)	2359 (0.034)	2548 (0.033)
topical	2	3437(0.02286498227963873)	1516 (0.069)	#SAKNAS!
topical	2	3437(0.02286498227963873)	1516 (0.069)	#SAKNAS!
trace	2	1894(0.05716245569909683)	1516 (0.069)	2548 (0.033)
tracheo-oesophageal	2	3437(0.02286498227963873)	#SAKNAS!	2548 (0.033)
tracheotomy	2	3437(0.02286498227963873)	2359 (0.034)	#SAKNAS!
tracking	2	3437(0.02286498227963873)	1516 (0.069)	#SAKNAS!
tracking	2	3437(0.02286498227963873)	1516 (0.069)	#SAKNAS!
traction	2	3437(0.02286498227963873)	2359 (0.034)	#SAKNAS!
traditions	2	3437(0.02286498227963873)	#SAKNAS!	1668 (0.066)
train	2	3437(0.02286498227963873)	#SAKNAS!	2548 (0.033)
trajectory	2	1678(0.0685949468389162)	1516 (0.069)	2548 (0.033)
transcript	2	2214(0.04572996455927746)	1516 (0.069)	2548 (0.033)
transcription	2	3437(0.02286498227963873)	2359 (0.034)	#SAKNAS!
transcriptional	2	3437(0.02286498227963873)	2359 (0.034)	#SAKNAS!
transcriptomic	2	3437(0.02286498227963873)	#SAKNAS!	#SAKNAS!
transgastric	2	3437(0.02286498227963873)	#SAKNAS!	2548 (0.033)
transgenerational	2	3437(0.02286498227963873)	#SAKNAS!	#SAKNAS!
transhiatal	2	3437(0.02286498227963873)	1516 (0.069)	#SAKNAS!
transhiatal	2	3437(0.02286498227963873)	1516 (0.069)	#SAKNAS!
translating	2	3437(0.02286498227963873)	2359 (0.034)	2548 (0.033)
translation	2	1894(0.05716245569909683)	1516 (0.069)	2548 (0.033)
translational	2	3437(0.02286498227963873)	#SAKNAS!	2548 (0.033)
translocation	2	3437(0.02286498227963873)	#SAKNAS!	1668 (0.066)
transmission	2	3437(0.02286498227963873)	#SAKNAS!	1668 (0.066)

transoral	2	1335(0.09145992911855493)	1516 (0.069)	1233 (0.1)
transplant	2	2214(0.04572996455927746)	1516 (0.069)	#SAKNAS!
transplantation	2	836(0.16005487595747114)	1516 (0.069)	694 (0.2)
trauma	2	1485(0.08002743797873557)	1516 (0.069)	#SAKNAS!
traumatic	2	1027(0.12575740253801304)	1516 (0.069)	816 (0.16)
tree	2	3437(0.02286498227963873)	2359 (0.034)	2548 (0.033)
trees	2	3437(0.02286498227963873)	2359 (0.034)	#SAKNAS!
trefoil	2	3437(0.02286498227963873)	2359 (0.034)	#SAKNAS!
treponema	2	3437(0.02286498227963873)	2359 (0.034)	2548 (0.033)
trials	2	1678(0.0685949468389162)	1516 (0.069)	1233 (0.1)
trichoptera	2	3437(0.02286498227963873)	2359 (0.034)	#SAKNAS!
triglyceride	2	1335(0.09145992911855493)	1516 (0.069)	1233 (0.1)
trinidad	2	3437(0.02286498227963873)	2359 (0.034)	2548 (0.033)
triple	2	3437(0.02286498227963873)	2359 (0.034)	2548 (0.033)
tromso	2	3437(0.02286498227963873)	#SAKNAS!	#SAKNAS!
trypanosoma	2	3437(0.02286498227963873)	#SAKNAS!	2548 (0.033)
tube-enteral-fed	2	3437(0.02286498227963873)	1516 (0.069)	#SAKNAS!
tube-enteral-fed	2	3437(0.02286498227963873)	1516 (0.069)	#SAKNAS!
tube-fed	2	2214(0.04572996455927746)	1516 (0.069)	2548 (0.033)
tuberculata	2	2693(0.0342974734194581)	1516 (0.069)	2548 (0.033)
tumours	2	3437(0.02286498227963873)	2359 (0.034)	2548 (0.033)
tunisia	2	3437(0.02286498227963873)	2359 (0.034)	2548 (0.033)
tv	2	3437(0.02286498227963873)	2359 (0.034)	2548 (0.033)
twice	2	3437(0.02286498227963873)	2359 (0.034)	2548 (0.033)
twins	2	3437(0.02286498227963873)	2359 (0.034)	2548 (0.033)
typhimurium	2	3437(0.02286498227963873)	2359 (0.034)	#SAKNAS!
typical	2	3437(0.02286498227963873)	2359 (0.034)	2548 (0.033)
tyrosine	2	3437(0.02286498227963873)	#SAKNAS!	#SAKNAS!
u.s	2	3437(0.02286498227963873)	2359 (0.034)	2548 (0.033)
ulcer	2	3437(0.02286498227963873)	#SAKNAS!	1668 (0.066)
ulceration	2	3437(0.02286498227963873)	2359 (0.034)	2548 (0.033)
ulcers	2	1678(0.0685949468389162)	1516 (0.069)	1668 (0.066)
ultradian	2	3437(0.02286498227963873)	2359 (0.034)	#SAKNAS!
ultra-endurance	2	3437(0.02286498227963873)	#SAKNAS!	#SAKNAS!
ultrasonographic	2	3437(0.02286498227963873)	#SAKNAS!	2548 (0.033)
ultrasound	2	1335(0.09145992911855493)	1516 (0.069)	1233 (0.1)
under-five	2	3437(0.02286498227963873)	#SAKNAS!	#SAKNAS!
undergone	2	3437(0.02286498227963873)	#SAKNAS!	#SAKNAS!
underpinning	2	3437(0.02286498227963873)	2359 (0.034)	2548 (0.033)
understand	2	2214(0.04572996455927746)	1516 (0.069)	1668 (0.066)
underwent	2	1335(0.09145992911855493)	1516 (0.069)	999 (0.13)
unintentional	2	3437(0.02286498227963873)	2359 (0.034)	2548 (0.033)
unpredictable	2	3437(0.02286498227963873)	2359 (0.034)	2548 (0.033)

unsuccessful	2	3437(0.0228649822796387)	#SAKNAS!	#SAKNAS!
unusual	2	1485(0.0800274379787355)	1516 (0.069)	1233 (0.1)
upwelling	2	3437(0.0228649822796387)	#SAKNAS!	2548 (0.033)
usage	2	3437(0.0228649822796387)	1516 (0.069)	#SAKNAS!
usage	2	3437(0.0228649822796387)	1516 (0.069)	#SAKNAS!
usefulness	2	1678(0.0685949468389162)	1516 (0.069)	2548 (0.033)
us-mexican	2	3437(0.0228649822796387)	#SAKNAS!	1668 (0.066)
usual	2	3437(0.0228649822796387)	2359 (0.034)	#SAKNAS!
utilisation	2	3437(0.0228649822796387)	2359 (0.034)	#SAKNAS!
utilizing	2	3437(0.0228649822796387)	#SAKNAS!	#SAKNAS!
vaccination	2	3437(0.0228649822796387)	#SAKNAS!	2548 (0.033)
vagal	2	2693(0.0342974734194581)	1516 (0.069)	2548 (0.033)
vaginal	2	3437(0.0228649822796387)	2359 (0.034)	2548 (0.033)
valid	2	1894(0.0571624556990968)	1516 (0.069)	1668 (0.066)
valve	2	3437(0.0228649822796387)	2359 (0.034)	#SAKNAS!
variable	2	2214(0.0457299645592775)	1516 (0.069)	1668 (0.066)
vegan	2	3437(0.0228649822796387)	#SAKNAS!	1668 (0.066)
vegetable-exposure	2	3437(0.0228649822796387)	#SAKNAS!	2548 (0.033)
vegetables	2	1111(0.114324911398194)	1516 (0.069)	999 (0.13)
vegetarians	2	3437(0.0228649822796387)	2359 (0.034)	#SAKNAS!
ventilatory	2	3437(0.0228649822796387)	2359 (0.034)	2548 (0.033)
ventral	2	3437(0.0228649822796387)	#SAKNAS!	2548 (0.033)
ventromedial	2	2693(0.0342974734194581)	1516 (0.069)	#SAKNAS!
verreauxi	2	3437(0.0228649822796387)	#SAKNAS!	1668 (0.066)
vertebrate	2	3437(0.0228649822796387)	#SAKNAS!	1668 (0.066)
viable	2	3437(0.0228649822796387)	2359 (0.034)	#SAKNAS!
victims	2	3437(0.0228649822796387)	2359 (0.034)	2548 (0.033)
videofluorography	2	3437(0.0228649822796387)	2359 (0.034)	2548 (0.033)
vietnamese	2	3437(0.0228649822796387)	#SAKNAS!	2548 (0.033)
viscosity	2	1894(0.0571624556990968)	1516 (0.069)	1668 (0.066)
vision	2	3437(0.0228649822796387)	#SAKNAS!	#SAKNAS!
visit	2	1678(0.0685949468389162)	1516 (0.069)	1668 (0.066)
visiting	2	3437(0.0228649822796387)	#SAKNAS!	#SAKNAS!
visitors	2	3437(0.0228649822796387)	2359 (0.034)	#SAKNAS!
vitro	2	1027(0.125757402538013)	1516 (0.069)	2548 (0.033)
vivo	2	1485(0.0800274379787355)	1516 (0.069)	#SAKNAS!
vocal	2	2693(0.0342974734194581)	1516 (0.069)	2548 (0.033)
volume	2	710(0.194352349376929)	1516 (0.069)	584 (0.23)
volumetric	2	3437(0.0228649822796387)	#SAKNAS!	1668 (0.066)
volume-viscosity	2	2693(0.0342974734194581)	1516 (0.069)	2548 (0.033)
volunteers	2	2693(0.0342974734194581)	1516 (0.069)	2548 (0.033)
volvulus	2	3437(0.0228649822796387)	2359 (0.034)	2548 (0.033)
vulgaris	2	3437(0.0228649822796387)	#SAKNAS!	2548 (0.033)

vulnerable	2	3437(0.0228649822796387)	2359 (0.034)	2548 (0.033)
wales	2	3437(0.0228649822796387)	1516 (0.069)	#SAKNAS!
wales	2	3437(0.0228649822796387)	1516 (0.069)	#SAKNAS!
walking	2	3437(0.0228649822796387)	#SAKNAS!	2548 (0.033)
wards	2	1485(0.0800274379787355)	1516 (0.069)	1668 (0.066)
warming	2	3437(0.0228649822796387)	#SAKNAS!	#SAKNAS!
warning	2	3437(0.0228649822796387)	#SAKNAS!	2548 (0.033)
waters	2	2214(0.0457299645592774)	1516 (0.069)	2548 (0.033)
waves	2	3437(0.0228649822796387)	#SAKNAS!	#SAKNAS!
ways	2	3437(0.0228649822796387)	2359 (0.034)	2548 (0.033)
weakness	2	3437(0.0228649822796387)	2359 (0.034)	2548 (0.033)
wear	2	1219(0.102892420258374)	1516 (0.069)	1668 (0.066)
wearable	2	3437(0.0228649822796387)	#SAKNAS!	#SAKNAS!
wearing	2	1335(0.0914599291185549)	1516 (0.069)	#SAKNAS!
weather	2	3437(0.0228649822796387)	1516 (0.069)	#SAKNAS!
weather	2	3437(0.0228649822796387)	1516 (0.069)	#SAKNAS!
web	2	3437(0.0228649822796387)	1516 (0.069)	#SAKNAS!
web	2	3437(0.0228649822796387)	1516 (0.069)	#SAKNAS!
weekly	2	3437(0.0228649822796387)	2359 (0.034)	2548 (0.033)
weeks	2	1894(0.0571624556990968)	1516 (0.069)	2548 (0.033)
wehncke	2	3437(0.0228649822796387)	2359 (0.034)	2548 (0.033)
weighing	2	3437(0.0228649822796387)	2359 (0.034)	2548 (0.033)
weight-loss	2	3437(0.0228649822796387)	1516 (0.069)	#SAKNAS!
weight-loss	2	3437(0.0228649822796387)	1516 (0.069)	#SAKNAS!
wellbeing	2	3437(0.0228649822796387)	#SAKNAS!	1668 (0.066)
wellness	2	3437(0.0228649822796387)	#SAKNAS!	2548 (0.033)
west	2	1894(0.0571624556990968)	1516 (0.069)	1668 (0.066)
whelk	2	2693(0.034297473419458)	1516 (0.069)	#SAKNAS!
whose	2	2693(0.034297473419458)	1516 (0.069)	2548 (0.033)
willingness	2	3437(0.0228649822796387)	#SAKNAS!	2548 (0.033)
wilson	2	3437(0.0228649822796387)	#SAKNAS!	2548 (0.033)
wintering	2	2214(0.0457299645592774)	1516 (0.069)	1668 (0.066)
wisdom	2	3437(0.0228649822796387)	2359 (0.034)	2548 (0.033)
wistar	2	3437(0.0228649822796387)	#SAKNAS!	2548 (0.033)
wolves	2	3437(0.0228649822796387)	2359 (0.034)	#SAKNAS!
woody	2	3437(0.0228649822796387)	2359 (0.034)	#SAKNAS!
woolly	2	2693(0.034297473419458)	1516 (0.069)	2548 (0.033)
worker	2	2693(0.034297473419458)	1516 (0.069)	2548 (0.033)
workplace	2	3437(0.0228649822796387)	#SAKNAS!	2548 (0.033)
workshops	2	3437(0.0228649822796387)	#SAKNAS!	1668 (0.066)
world	2	1678(0.068594946838916)	1516 (0.069)	1668 (0.066)
worries	2	3437(0.0228649822796387)	#SAKNAS!	#SAKNAS!
worse	2	3437(0.0228649822796387)	2359 (0.034)	2548 (0.033)

worsens		2	3437(0.02286498227963873)	#SAKNAS!	2548 (0.033)
xenopus		2	3437(0.02286498227963873)	2359 (0.034)	#SAKNAS!
xylem		2	3437(0.02286498227963873)	#SAKNAS!	1668 (0.066)
yakui		2	3437(0.02286498227963873)	#SAKNAS!	1668 (0.066)
yamagata		2	3437(0.02286498227963873)	#SAKNAS!	2548 (0.033)
year-old		2	3437(0.02286498227963873)	#SAKNAS!	2548 (0.033)
yeast		2	1485(0.08002743797873557)	1516 (0.069)	1668 (0.066)
yeasts		2	3437(0.02286498227963873)	2359 (0.034)	2548 (0.033)
yesterday		2	3437(0.02286498227963873)	2359 (0.034)	2548 (0.033)
yoldia		2	3437(0.02286498227963873)	1516 (0.069)	#SAKNAS!
yoldia		2	3437(0.02286498227963873)	1516 (0.069)	#SAKNAS!
younger		2	1485(0.08002743797873557)	1516 (0.069)	1233 (0.1)
your		2	3437(0.02286498227963873)	2359 (0.034)	2548 (0.033)
youth		2	1678(0.0685949468389162)	1516 (0.069)	1668 (0.066)
youths		2	3437(0.02286498227963873)	1516 (0.069)	#SAKNAS!
youths		2	3437(0.02286498227963873)	1516 (0.069)	#SAKNAS!
yunnan		2	3437(0.02286498227963873)	#SAKNAS!	2548 (0.033)
yy		2	3437(0.02286498227963873)	2359 (0.034)	2548 (0.033)
zebra		2	3437(0.02286498227963873)	2359 (0.034)	2548 (0.033)
zebu		2	3437(0.02286498227963873)	#SAKNAS!	1668 (0.066)
zero-p		2	3437(0.02286498227963873)	#SAKNAS!	2548 (0.033)
zika		2	3437(0.02286498227963873)	#SAKNAS!	#SAKNAS!
zones		2	3437(0.02286498227963873)	#SAKNAS!	2548 (0.033)
zoo		2	3437(0.02286498227963873)	#SAKNAS!	2548 (0.033)
z-stents		2	3437(0.02286498227963873)	1516 (0.069)	#SAKNAS!
z-stents		2	3437(0.02286498227963873)	1516 (0.069)	#SAKNAS!
	0,9	1	5110(0.011432491139819366)	#SAKNAS!	2548 (0.033)
	1	1	5110(0.011432491139819366)	2359 (0.034)	#SAKNAS!
	1	1	5110(0.011432491139819366)	2359 (0.034)	#SAKNAS!
	2	1	3437(0.02286498227963873)	2359 (0.034)	#SAKNAS!
	2	1	3437(0.02286498227963873)	2359 (0.034)	#SAKNAS!
	3	1	5110(0.011432491139819366)	#SAKNAS!	2548 (0.033)
	264,7	1	5110(0.011432491139819366)	#SAKNAS!	#SAKNAS!
	1505	1	5110(0.011432491139819366)	#SAKNAS!	#SAKNAS!
	1512	1	5110(0.011432491139819366)	#SAKNAS!	#SAKNAS!
	1790	1	5110(0.011432491139819366)	#SAKNAS!	#SAKNAS!
	1794	1	5110(0.011432491139819366)	2359 (0.034)	#SAKNAS!
	1794	1	5110(0.011432491139819366)	2359 (0.034)	#SAKNAS!
	1828	1	5110(0.011432491139819366)	#SAKNAS!	#SAKNAS!
	1833	1	5110(0.011432491139819366)	#SAKNAS!	#SAKNAS!
	1837	1	5110(0.011432491139819366)	2359 (0.034)	#SAKNAS!
	1837	1	5110(0.011432491139819366)	2359 (0.034)	#SAKNAS!
	1841	1	5110(0.011432491139819366)	#SAKNAS!	#SAKNAS!

1849	1	5110(0.011432491139819366)	#SAKNAS!	2548 (0.033)
1931	1	5110(0.011432491139819366)	#SAKNAS!	2548 (0.033)
1945	1	5110(0.011432491139819366)	2359 (0.034)	#SAKNAS!
1945	1	5110(0.011432491139819366)	2359 (0.034)	#SAKNAS!
1956	1	5110(0.011432491139819366)	2359 (0.034)	#SAKNAS!
1956	1	5110(0.011432491139819366)	2359 (0.034)	#SAKNAS!
1969	1	5110(0.011432491139819366)	2359 (0.034)	#SAKNAS!
1969	1	5110(0.011432491139819366)	2359 (0.034)	#SAKNAS!
1971	1	5110(0.011432491139819366)	#SAKNAS!	2548 (0.033)
1982	1	5110(0.011432491139819366)	#SAKNAS!	2548 (0.033)
1988	1	5110(0.011432491139819366)	#SAKNAS!	2548 (0.033)
1990	1	5110(0.011432491139819366)	#SAKNAS!	#SAKNAS!
1991	1	5110(0.011432491139819366)	2359 (0.034)	#SAKNAS!
1991	1	5110(0.011432491139819366)	2359 (0.034)	#SAKNAS!
1994	1	5110(0.011432491139819366)	2359 (0.034)	#SAKNAS!
1994	1	5110(0.011432491139819366)	2359 (0.034)	#SAKNAS!
1998	1	5110(0.011432491139819366)	2359 (0.034)	#SAKNAS!
1998	1	5110(0.011432491139819366)	2359 (0.034)	#SAKNAS!
2009	1	743(0.18291985823710985)	2359 (0.034)	422 (0.33)
2011	1	1335(0.09145992911855493)	2359 (0.034)	694 (0.2)
2013	1	1678(0.0685949468389162)	2359 (0.034)	1668 (0.066)
2023	1	5110(0.011432491139819366)	#SAKNAS!	#SAKNAS!
2024	1	5110(0.011432491139819366)	#SAKNAS!	#SAKNAS!
2071	1	5110(0.011432491139819366)	2359 (0.034)	#SAKNAS!
2071	1	5110(0.011432491139819366)	2359 (0.034)	#SAKNAS!
2131	1	5110(0.011432491139819366)	2359 (0.034)	#SAKNAS!
2131	1	5110(0.011432491139819366)	2359 (0.034)	#SAKNAS!
2200	1	5110(0.011432491139819366)	#SAKNAS!	2548 (0.033)
3063	1	5110(0.011432491139819366)	#SAKNAS!	2548 (0.033)
3111	1	5110(0.011432491139819366)	#SAKNAS!	2548 (0.033)
4061	1	5110(0.011432491139819366)	#SAKNAS!	2548 (0.033)
4716	1	5110(0.011432491139819366)	#SAKNAS!	2548 (0.033)
5943	1	5110(0.011432491139819366)	#SAKNAS!	#SAKNAS!
7589	1	5110(0.011432491139819366)	#SAKNAS!	2548 (0.033)
8020	1	5110(0.011432491139819366)	2359 (0.034)	#SAKNAS!
8020	1	5110(0.011432491139819366)	2359 (0.034)	#SAKNAS!
12174	1	5110(0.011432491139819366)	#SAKNAS!	2548 (0.033)
13998	1	5110(0.011432491139819366)	#SAKNAS!	2548 (0.033)
18476	1	5110(0.011432491139819366)	2359 (0.034)	#SAKNAS!
18476	1	5110(0.011432491139819366)	2359 (0.034)	#SAKNAS!
45660	1	5110(0.011432491139819366)	#SAKNAS!	#SAKNAS!
45667	1	5110(0.011432491139819366)	#SAKNAS!	2548 (0.033)
45674	1	5110(0.011432491139819366)	2359 (0.034)	#SAKNAS!

45674	1	5110(0.011432491139819366)	2359 (0.034)	#SAKNAS!
45676	1	5110(0.011432491139819366)	#SAKNAS!	2548 (0.033)
45692	1	5110(0.011432491139819366)	#SAKNAS!	2548 (0.033)
45693	1	3437(0.02286498227963873)	2359 (0.034)	#SAKNAS!
45694	1	5110(0.011432491139819366)	2359 (0.034)	#SAKNAS!
45694	1	5110(0.011432491139819366)	2359 (0.034)	#SAKNAS!
45717	1	5110(0.011432491139819366)	#SAKNAS!	#SAKNAS!
45727	1	5110(0.011432491139819366)	#SAKNAS!	2548 (0.033)
45752	1	5110(0.011432491139819366)	#SAKNAS!	2548 (0.033)
45753	1	5110(0.011432491139819366)	2359 (0.034)	#SAKNAS!
45753	1	5110(0.011432491139819366)	2359 (0.034)	#SAKNAS!
45783	1	5110(0.011432491139819366)	#SAKNAS!	2548 (0.033)
45791	1	5110(0.011432491139819366)	#SAKNAS!	2548 (0.033)
45796	1	5110(0.011432491139819366)	#SAKNAS!	2548 (0.033)
45817	1	5110(0.011432491139819366)	#SAKNAS!	2548 (0.033)
45847	1	5110(0.011432491139819366)	#SAKNAS!	2548 (0.033)
45848	1	5110(0.011432491139819366)	#SAKNAS!	2548 (0.033)
45852	1	5110(0.011432491139819366)	2359 (0.034)	#SAKNAS!
45852	1	5110(0.011432491139819366)	2359 (0.034)	#SAKNAS!
45883	1	5110(0.011432491139819366)	#SAKNAS!	#SAKNAS!
45910	1	5110(0.011432491139819366)	2359 (0.034)	#SAKNAS!
45910	1	5110(0.011432491139819366)	2359 (0.034)	#SAKNAS!
45911	1	5110(0.011432491139819366)	#SAKNAS!	2548 (0.033)
45923	1	5110(0.011432491139819366)	#SAKNAS!	#SAKNAS!
45974	1	5110(0.011432491139819366)	2359 (0.034)	#SAKNAS!
45974	1	5110(0.011432491139819366)	2359 (0.034)	#SAKNAS!
46005	1	3437(0.02286498227963873)	2359 (0.034)	2548 (0.033)
46010	1	5110(0.011432491139819366)	2359 (0.034)	#SAKNAS!
46010	1	5110(0.011432491139819366)	2359 (0.034)	#SAKNAS!
!goggin	1	5110(0.011432491139819366)	2359 (0.034)	#SAKNAS!
!goggin	1	5110(0.011432491139819366)	2359 (0.034)	#SAKNAS!
!kimble	1	5110(0.011432491139819366)	2359 (0.034)	#SAKNAS!
!kimble	1	5110(0.011432491139819366)	2359 (0.034)	#SAKNAS!
!patel	1	5110(0.011432491139819366)	2359 (0.034)	#SAKNAS!
!patel	1	5110(0.011432491139819366)	2359 (0.034)	#SAKNAS!
!roseveare	1	5110(0.011432491139819366)	2359 (0.034)	#SAKNAS!
!roseveare	1	5110(0.011432491139819366)	2359 (0.034)	#SAKNAS!
!shepherd	1	5110(0.011432491139819366)	2359 (0.034)	#SAKNAS!
!shepherd	1	5110(0.011432491139819366)	2359 (0.034)	#SAKNAS!
!simmonds	1	5110(0.011432491139819366)	2359 (0.034)	#SAKNAS!
!simmonds	1	5110(0.011432491139819366)	2359 (0.034)	#SAKNAS!
?□p63	1	5110(0.011432491139819366)	2359 (0.034)	#SAKNAS!
?□p63	1	5110(0.011432491139819366)	2359 (0.034)	#SAKNAS!

?□80	1	5110(0.011432491139819366)	2359 (0.034)	#SAKNAS!
?□80	1	5110(0.011432491139819366)	2359 (0.034)	#SAKNAS!
0.5-year-old	1	5110(0.011432491139819366)	2359 (0.034)	#SAKNAS!
0.5-year-old	1	5110(0.011432491139819366)	2359 (0.034)	#SAKNAS!
0-2	1	5110(0.011432491139819366)	#SAKNAS!	#SAKNAS!
0-5	1	5110(0.011432491139819366)	#SAKNAS!	#SAKNAS!
0-5-month-old	1	5110(0.011432491139819366)	#SAKNAS!	#SAKNAS!
1.5-4.5	1	5110(0.011432491139819366)	2359 (0.034)	#SAKNAS!
1.5-4.5	1	5110(0.011432491139819366)	2359 (0.034)	#SAKNAS!
10-species	1	5110(0.011432491139819366)	2359 (0.034)	#SAKNAS!
10-species	1	5110(0.011432491139819366)	2359 (0.034)	#SAKNAS!
10-year-old	1	2693(0.0342974734194581)	2359 (0.034)	2548 (0.033)
10-y-old	1	5110(0.011432491139819366)	2359 (0.034)	#SAKNAS!
10-y-old	1	5110(0.011432491139819366)	2359 (0.034)	#SAKNAS!
11c	1	5110(0.011432491139819366)	2359 (0.034)	#SAKNAS!
11c	1	5110(0.011432491139819366)	2359 (0.034)	#SAKNAS!
11-year	1	3437(0.02286498227963873)	2359 (0.034)	2548 (0.033)
11-year-old	1	5110(0.011432491139819366)	2359 (0.034)	#SAKNAS!
11-year-old	1	5110(0.011432491139819366)	2359 (0.034)	#SAKNAS!
11-years	1	5110(0.011432491139819366)	#SAKNAS!	2548 (0.033)
11帣-hydroxysteroid	1	5110(0.011432491139819366)	#SAKNAS!	2548 (0.033)
12th	1	5110(0.011432491139819366)	#SAKNAS!	#SAKNAS!
12-week	1	5110(0.011432491139819366)	#SAKNAS!	#SAKNAS!
12-year	1	5110(0.011432491139819366)	#SAKNAS!	2548 (0.033)
13-year	1	5110(0.011432491139819366)	#SAKNAS!	#SAKNAS!
1438g	1	5110(0.011432491139819366)	2359 (0.034)	#SAKNAS!
1438g	1	5110(0.011432491139819366)	2359 (0.034)	#SAKNAS!
14-d	1	5110(0.011432491139819366)	#SAKNAS!	2548 (0.033)
14-year-old	1	5110(0.011432491139819366)	#SAKNAS!	#SAKNAS!
15-cm	1	5110(0.011432491139819366)	#SAKNAS!	2548 (0.033)
15-year-old	1	5110(0.011432491139819366)	#SAKNAS!	2548 (0.033)
15-year-olds	1	5110(0.011432491139819366)	#SAKNAS!	2548 (0.033)
16-year-olds	1	5110(0.011432491139819366)	#SAKNAS!	2548 (0.033)
17-47	1	5110(0.011432491139819366)	2359 (0.034)	#SAKNAS!
17-47	1	5110(0.011432491139819366)	2359 (0.034)	#SAKNAS!
17-year	1	5110(0.011432491139819366)	#SAKNAS!	#SAKNAS!
17-year-old	1	5110(0.011432491139819366)	#SAKNAS!	2548 (0.033)
18-39	1	5110(0.011432491139819366)	#SAKNAS!	#SAKNAS!
18-74	1	5110(0.011432491139819366)	2359 (0.034)	#SAKNAS!
18-74	1	5110(0.011432491139819366)	2359 (0.034)	#SAKNAS!
18-month	1	5110(0.011432491139819366)	#SAKNAS!	2548 (0.033)
18-year-old	1	2693(0.0342974734194581)	2359 (0.034)	1668 (0.066)
18-year-olds	1	5110(0.011432491139819366)	#SAKNAS!	2548 (0.033)

1958-1970	1	5110(0.011432491139819366)	2359 (0.034)	#SAKNAS!
1958-1970	1	5110(0.011432491139819366)	2359 (0.034)	#SAKNAS!
1989-1995	1	5110(0.011432491139819366)	2359 (0.034)	#SAKNAS!
1989-1995	1	5110(0.011432491139819366)	2359 (0.034)	#SAKNAS!
1989-2001	1	5110(0.011432491139819366)	2359 (0.034)	#SAKNAS!
1989-2001	1	5110(0.011432491139819366)	2359 (0.034)	#SAKNAS!
1999-2010	1	5110(0.011432491139819366)	#SAKNAS!	2548 (0.033)
1999-2014	1	5110(0.011432491139819366)	#SAKNAS!	2548 (0.033)
1999-2020	1	5110(0.011432491139819366)	#SAKNAS!	#SAKNAS!
19-year-old	1	5110(0.011432491139819366)	#SAKNAS!	2548 (0.033)
19-years-olds	1	5110(0.011432491139819366)	2359 (0.034)	#SAKNAS!
19-years-olds	1	5110(0.011432491139819366)	2359 (0.034)	#SAKNAS!
1-day-old	1	5110(0.011432491139819366)	2359 (0.034)	#SAKNAS!
1-day-old	1	5110(0.011432491139819366)	2359 (0.034)	#SAKNAS!
1-deoxynojirimycin	1	5110(0.011432491139819366)	#SAKNAS!	2548 (0.033)
1h-nmr	1	5110(0.011432491139819366)	#SAKNAS!	#SAKNAS!
1-y	1	5110(0.011432491139819366)	#SAKNAS!	#SAKNAS!
1-year-olds	1	5110(0.011432491139819366)	#SAKNAS!	2548 (0.033)
2.5-year	1	5110(0.011432491139819366)	#SAKNAS!	2548 (0.033)
2000-2014	1	5110(0.011432491139819366)	#SAKNAS!	2548 (0.033)
2001-2002	1	5110(0.011432491139819366)	2359 (0.034)	#SAKNAS!
2001-2002	1	5110(0.011432491139819366)	2359 (0.034)	#SAKNAS!
2001-2004	1	5110(0.011432491139819366)	#SAKNAS!	#SAKNAS!
2002-2015	1	5110(0.011432491139819366)	#SAKNAS!	2548 (0.033)
2003-04	1	5110(0.011432491139819366)	2359 (0.034)	#SAKNAS!
2003-04	1	5110(0.011432491139819366)	2359 (0.034)	#SAKNAS!
2003-2012	1	5110(0.011432491139819366)	#SAKNAS!	2548 (0.033)
2003-2013	1	5110(0.011432491139819366)	#SAKNAS!	2548 (0.033)
2003-2016	1	5110(0.011432491139819366)	#SAKNAS!	2548 (0.033)
2003-2018	1	5110(0.011432491139819366)	#SAKNAS!	#SAKNAS!
2005-08	1	5110(0.011432491139819366)	2359 (0.034)	#SAKNAS!
2005-08	1	5110(0.011432491139819366)	2359 (0.034)	#SAKNAS!
2005-2008	1	5110(0.011432491139819366)	#SAKNAS!	2548 (0.033)
2005-2010	1	5110(0.011432491139819366)	#SAKNAS!	2548 (0.033)
2005-2018	1	5110(0.011432491139819366)	#SAKNAS!	#SAKNAS!
2005-2020	1	5110(0.011432491139819366)	#SAKNAS!	#SAKNAS!
2007-2008	1	3437(0.02286498227963873)	2359 (0.034)	2548 (0.033)
2007-2014	1	5110(0.011432491139819366)	#SAKNAS!	2548 (0.033)
2007-2021	1	5110(0.011432491139819366)	#SAKNAS!	#SAKNAS!
2008-2009	1	2693(0.0342974734194581)	2359 (0.034)	2548 (0.033)
2008-2010	1	1219(0.1028924202583743)	2359 (0.034)	526 (0.26)
2008-2015	1	5110(0.011432491139819366)	#SAKNAS!	2548 (0.033)
2009-2016	1	5110(0.011432491139819366)	#SAKNAS!	#SAKNAS!

2009-2018	1	5110(0.011432491139819366)	#SAKNAS!	#SAKNAS!
2011-16	1	5110(0.011432491139819366)	#SAKNAS!	#SAKNAS!
2011-2020	1	5110(0.011432491139819366)	#SAKNAS!	#SAKNAS!
2012-2015	1	5110(0.011432491139819366)	#SAKNAS!	#SAKNAS!
2012-2018	1	5110(0.011432491139819366)	#SAKNAS!	#SAKNAS!
2012-2020	1	5110(0.011432491139819366)	#SAKNAS!	#SAKNAS!
2013-2016	1	5110(0.011432491139819366)	#SAKNAS!	#SAKNAS!
2014-2015	1	5110(0.011432491139819366)	#SAKNAS!	#SAKNAS!
2015-2020	1	5110(0.011432491139819366)	#SAKNAS!	#SAKNAS!
2016-2019	1	5110(0.011432491139819366)	#SAKNAS!	#SAKNAS!
2018-2028	1	5110(0.011432491139819366)	#SAKNAS!	2548 (0.033)
2019-2021	1	5110(0.011432491139819366)	#SAKNAS!	#SAKNAS!
2021-2022	1	5110(0.011432491139819366)	#SAKNAS!	#SAKNAS!
20-79	1	5110(0.011432491139819366)	2359 (0.034)	#SAKNAS!
20-79	1	5110(0.011432491139819366)	2359 (0.034)	#SAKNAS!
21-64	1	5110(0.011432491139819366)	#SAKNAS!	2548 (0.033)
21-mm	1	5110(0.011432491139819366)	2359 (0.034)	#SAKNAS!
21-mm	1	5110(0.011432491139819366)	2359 (0.034)	#SAKNAS!
22q11	1	5110(0.011432491139819366)	#SAKNAS!	2548 (0.033)
22q11.2	1	5110(0.011432491139819366)	#SAKNAS!	2548 (0.033)
23-hour	1	5110(0.011432491139819366)	#SAKNAS!	2548 (0.033)
24-h	1	5110(0.011432491139819366)	#SAKNAS!	2548 (0.033)
24-month-old	1	5110(0.011432491139819366)	2359 (0.034)	#SAKNAS!
24-month-old	1	5110(0.011432491139819366)	2359 (0.034)	#SAKNAS!
25-44	1	5110(0.011432491139819366)	#SAKNAS!	#SAKNAS!
25-79	1	5110(0.011432491139819366)	#SAKNAS!	2548 (0.033)
25-year	1	5110(0.011432491139819366)	2359 (0.034)	#SAKNAS!
25-year	1	5110(0.011432491139819366)	2359 (0.034)	#SAKNAS!
27-year	1	5110(0.011432491139819366)	#SAKNAS!	#SAKNAS!
2a	1	5110(0.011432491139819366)	2359 (0.034)	#SAKNAS!
2a	1	5110(0.011432491139819366)	2359 (0.034)	#SAKNAS!
2-arachidonoylglycerol	1	5110(0.011432491139819366)	#SAKNAS!	2548 (0.033)
2h	1	5110(0.011432491139819366)	#SAKNAS!	2548 (0.033)
2nd	1	5110(0.011432491139819366)	#SAKNAS!	2548 (0.033)
2-to	1	2693(0.0342974734194581)	2359 (0.034)	2548 (0.033)
30-42	1	5110(0.011432491139819366)	2359 (0.034)	#SAKNAS!
30-42	1	5110(0.011432491139819366)	2359 (0.034)	#SAKNAS!
30-and	1	5110(0.011432491139819366)	#SAKNAS!	#SAKNAS!
30-year	1	3437(0.02286498227963873)	2359 (0.034)	2548 (0.033)
3-4-year-old	1	5110(0.011432491139819366)	2359 (0.034)	#SAKNAS!
3-4-year-old	1	5110(0.011432491139819366)	2359 (0.034)	#SAKNAS!
35-5	1	5110(0.011432491139819366)	#SAKNAS!	2548 (0.033)
35-54	1	5110(0.011432491139819366)	#SAKNAS!	2548 (0.033)

35-79	1	5110(0.011432491139819366)	#SAKNAS!	2548 (0.033)
36-month-old	1	5110(0.011432491139819366)	#SAKNAS!	2548 (0.033)
37-year	1	5110(0.011432491139819366)	#SAKNAS!	2548 (0.033)
3-and	1	5110(0.011432491139819366)	#SAKNAS!	2548 (0.033)
3dct	1	5110(0.011432491139819366)	#SAKNAS!	#SAKNAS!
3-dimensional	1	5110(0.011432491139819366)	#SAKNAS!	2548 (0.033)
3d-printable	1	5110(0.011432491139819366)	#SAKNAS!	#SAKNAS!
3-level	1	5110(0.011432491139819366)	#SAKNAS!	#SAKNAS!
3-week	1	5110(0.011432491139819366)	2359 (0.034)	#SAKNAS!
3-week	1	5110(0.011432491139819366)	2359 (0.034)	#SAKNAS!
3-weekly	1	5110(0.011432491139819366)	#SAKNAS!	#SAKNAS!
3xtg-ad	1	5110(0.011432491139819366)	#SAKNAS!	2548 (0.033)
4??-tetrabromodiphenyl	1	5110(0.011432491139819366)	#SAKNAS!	#SAKNAS!
40years	1	5110(0.011432491139819366)	#SAKNAS!	2548 (0.033)
4-24-month-old	1	5110(0.011432491139819366)	2359 (0.034)	#SAKNAS!
4-24-month-old	1	5110(0.011432491139819366)	2359 (0.034)	#SAKNAS!
44-year-old	1	5110(0.011432491139819366)	2359 (0.034)	#SAKNAS!
44-year-old	1	5110(0.011432491139819366)	2359 (0.034)	#SAKNAS!
45-80	1	5110(0.011432491139819366)	#SAKNAS!	#SAKNAS!
472-patient	1	5110(0.011432491139819366)	#SAKNAS!	2548 (0.033)
4-and	1	3437(0.02286498227963873)	2359 (0.034)	2548 (0.033)
4-diaminobutane	1	5110(0.011432491139819366)	2359 (0.034)	#SAKNAS!
4-diaminobutane	1	5110(0.011432491139819366)	2359 (0.034)	#SAKNAS!
4-month-old	1	5110(0.011432491139819366)	2359 (0.034)	#SAKNAS!
4-month-old	1	5110(0.011432491139819366)	2359 (0.034)	#SAKNAS!
4th-instar	1	5110(0.011432491139819366)	2359 (0.034)	#SAKNAS!
4th-instar	1	5110(0.011432491139819366)	2359 (0.034)	#SAKNAS!
4-week	1	5110(0.011432491139819366)	#SAKNAS!	2548 (0.033)
4-year-old	1	3437(0.02286498227963873)	2359 (0.034)	2548 (0.033)
50-to	1	5110(0.011432491139819366)	#SAKNAS!	#SAKNAS!
5-11years	1	5110(0.011432491139819366)	#SAKNAS!	2548 (0.033)
55-79	1	5110(0.011432491139819366)	#SAKNAS!	2548 (0.033)
55-84	1	3437(0.02286498227963873)	2359 (0.034)	2548 (0.033)
5-7-year-old	1	5110(0.011432491139819366)	2359 (0.034)	#SAKNAS!
5-7-year-old	1	5110(0.011432491139819366)	2359 (0.034)	#SAKNAS!
5-ag	1	5110(0.011432491139819366)	#SAKNAS!	#SAKNAS!
5-dimethyl-4-hydroxy-3	1	5110(0.011432491139819366)	#SAKNAS!	2548 (0.033)
5-fluorouracil-based	1	5110(0.011432491139819366)	2359 (0.034)	#SAKNAS!
5-fluorouracil-based	1	5110(0.011432491139819366)	2359 (0.034)	#SAKNAS!
5-fu	1	5110(0.011432491139819366)	2359 (0.034)	#SAKNAS!
5-fu	1	5110(0.011432491139819366)	2359 (0.034)	#SAKNAS!
5-ht1a	1	5110(0.011432491139819366)	2359 (0.034)	#SAKNAS!
5-ht1a	1	5110(0.011432491139819366)	2359 (0.034)	#SAKNAS!

5-ht1b	1	5110(0.011432491139819366)	2359 (0.034)	#SAKNAS!
5-ht1b	1	5110(0.011432491139819366)	2359 (0.034)	#SAKNAS!
5-ht6	1	5110(0.011432491139819366)	#SAKNAS!	2548 (0.033)
5httlpr	1	5110(0.011432491139819366)	2359 (0.034)	#SAKNAS!
5httlpr	1	5110(0.011432491139819366)	2359 (0.034)	#SAKNAS!
5-hydroxyindoleacetic	1	5110(0.011432491139819366)	2359 (0.034)	#SAKNAS!
5-hydroxyindoleacetic	1	5110(0.011432491139819366)	2359 (0.034)	#SAKNAS!
5-item	1	5110(0.011432491139819366)	#SAKNAS!	#SAKNAS!
5-to	1	2693(0.0342974734194581)	2359 (0.034)	2548 (0.033)
5-y	1	5110(0.011432491139819366)	2359 (0.034)	#SAKNAS!
5-y	1	5110(0.011432491139819366)	2359 (0.034)	#SAKNAS!
5-year	1	787(0.1714873670972905)	2359 (0.034)	526 (0.26)
6.5-y-old	1	5110(0.011432491139819366)	2359 (0.034)	#SAKNAS!
6.5-y-old	1	5110(0.011432491139819366)	2359 (0.034)	#SAKNAS!
60years	1	5110(0.011432491139819366)	2359 (0.034)	#SAKNAS!
60years	1	5110(0.011432491139819366)	2359 (0.034)	#SAKNAS!
6-12-year-old	1	5110(0.011432491139819366)	#SAKNAS!	#SAKNAS!
6-24-month-old	1	5110(0.011432491139819366)	2359 (0.034)	#SAKNAS!
6-24-month-old	1	5110(0.011432491139819366)	2359 (0.034)	#SAKNAS!
6-36-month-old	1	5110(0.011432491139819366)	#SAKNAS!	2548 (0.033)
6-36months	1	5110(0.011432491139819366)	#SAKNAS!	2548 (0.033)
65-	1	5110(0.011432491139819366)	2359 (0.034)	#SAKNAS!
65-	1	5110(0.011432491139819366)	2359 (0.034)	#SAKNAS!
65+age	1	5110(0.011432491139819366)	#SAKNAS!	#SAKNAS!
653-7	1	5110(0.011432491139819366)	2359 (0.034)	#SAKNAS!
653-7	1	5110(0.011432491139819366)	2359 (0.034)	#SAKNAS!
65-74-year-old	1	5110(0.011432491139819366)	2359 (0.034)	#SAKNAS!
65-74-year-old	1	5110(0.011432491139819366)	2359 (0.034)	#SAKNAS!
6700-5600	1	5110(0.011432491139819366)	2359 (0.034)	#SAKNAS!
6700-5600	1	5110(0.011432491139819366)	2359 (0.034)	#SAKNAS!
6-7-year-old	1	5110(0.011432491139819366)	2359 (0.034)	#SAKNAS!
6-7-year-old	1	5110(0.011432491139819366)	2359 (0.034)	#SAKNAS!
6-8months	1	5110(0.011432491139819366)	#SAKNAS!	2548 (0.033)
68-year-old	1	5110(0.011432491139819366)	2359 (0.034)	#SAKNAS!
68-year-old	1	5110(0.011432491139819366)	2359 (0.034)	#SAKNAS!
6-and	1	3437(0.02286498227963873)	2359 (0.034)	2548 (0.033)
6j	1	3437(0.02286498227963873)	2359 (0.034)	#SAKNAS!
6-month-old	1	5110(0.011432491139819366)	#SAKNAS!	#SAKNAS!
6-months	1	5110(0.011432491139819366)	#SAKNAS!	2548 (0.033)
6-n-propylthiouracil	1	5110(0.011432491139819366)	2359 (0.034)	#SAKNAS!
6-n-propylthiouracil	1	5110(0.011432491139819366)	2359 (0.034)	#SAKNAS!
6th-3rd	1	5110(0.011432491139819366)	2359 (0.034)	#SAKNAS!
6th-3rd	1	5110(0.011432491139819366)	2359 (0.034)	#SAKNAS!

6th-instar	1	5110(0.011432491139819366)	2359 (0.034)	#SAKNAS!
6th-instar	1	5110(0.011432491139819366)	2359 (0.034)	#SAKNAS!
6-week	1	5110(0.011432491139819366)	#SAKNAS!	#SAKNAS!
6-year	1	1678(0.0685949468389162)	2359 (0.034)	2548 (0.033)
6-year-old	1	2693(0.0342974734194581)	2359 (0.034)	#SAKNAS!
6-year-olds	1	5110(0.011432491139819366)	#SAKNAS!	#SAKNAS!
75-year-old	1	5110(0.011432491139819366)	2359 (0.034)	#SAKNAS!
75-year-old	1	5110(0.011432491139819366)	2359 (0.034)	#SAKNAS!
75-year-olds	1	5110(0.011432491139819366)	2359 (0.034)	#SAKNAS!
75-year-olds	1	5110(0.011432491139819366)	2359 (0.034)	#SAKNAS!
79-year-old	1	5110(0.011432491139819366)	#SAKNAS!	2548 (0.033)
7-9-year-old	1	5110(0.011432491139819366)	#SAKNAS!	#SAKNAS!
80-year-olds	1	5110(0.011432491139819366)	#SAKNAS!	#SAKNAS!
82-to	1	5110(0.011432491139819366)	#SAKNAS!	2548 (0.033)
84-year-old	1	5110(0.011432491139819366)	#SAKNAS!	2548 (0.033)
8-tetrachlorodibenzo-p-dioxin	1	5110(0.011432491139819366)	2359 (0.034)	#SAKNAS!
8-tetrachlorodibenzo-p-dioxin	1	5110(0.011432491139819366)	2359 (0.034)	#SAKNAS!
8-week	1	5110(0.011432491139819366)	2359 (0.034)	#SAKNAS!
8-week	1	5110(0.011432491139819366)	2359 (0.034)	#SAKNAS!
90-day	1	5110(0.011432491139819366)	#SAKNAS!	#SAKNAS!
97-09	1	5110(0.011432491139819366)	2359 (0.034)	#SAKNAS!
97-09	1	5110(0.011432491139819366)	2359 (0.034)	#SAKNAS!
9-and	1	5110(0.011432491139819366)	#SAKNAS!	#SAKNAS!
9-month	1	5110(0.011432491139819366)	#SAKNAS!	2548 (0.033)
9p	1	5110(0.011432491139819366)	2359 (0.034)	#SAKNAS!
9p	1	5110(0.011432491139819366)	2359 (0.034)	#SAKNAS!
9-year	1	5110(0.011432491139819366)	#SAKNAS!	#SAKNAS!
9-year-old	1	5110(0.011432491139819366)	#SAKNAS!	2548 (0.033)
a-1	1	5110(0.011432491139819366)	2359 (0.034)	#SAKNAS!
a-1	1	5110(0.011432491139819366)	2359 (0.034)	#SAKNAS!
a10	1	5110(0.011432491139819366)	#SAKNAS!	#SAKNAS!
a1c	1	5110(0.011432491139819366)	#SAKNAS!	#SAKNAS!
a3669g	1	5110(0.011432491139819366)	#SAKNAS!	2548 (0.033)
a4	1	5110(0.011432491139819366)	#SAKNAS!	2548 (0.033)
aafp	1	5110(0.011432491139819366)	#SAKNAS!	#SAKNAS!
aaha	1	5110(0.011432491139819366)	#SAKNAS!	#SAKNAS!
aalge	1	5110(0.011432491139819366)	2359 (0.034)	#SAKNAS!
aalge	1	5110(0.011432491139819366)	2359 (0.034)	#SAKNAS!
aav-mediated	1	5110(0.011432491139819366)	#SAKNAS!	2548 (0.033)
abadia	1	5110(0.011432491139819366)	#SAKNAS!	#SAKNAS!
abalone	1	3437(0.02286498227963873)	2359 (0.034)	2548 (0.033)
abatus	1	5110(0.011432491139819366)	#SAKNAS!	#SAKNAS!
abdominis	1	5110(0.011432491139819366)	2359 (0.034)	#SAKNAS!

abdominis	1	5110(0.011432491139819366)	2359 (0.034)	#SAKNAS!
abdulrahman	1	5110(0.011432491139819366)	#SAKNAS!	2548 (0.033)
abeparvovec	1	5110(0.011432491139819366)	#SAKNAS!	#SAKNAS!
aberrant	1	5110(0.011432491139819366)	#SAKNAS!	#SAKNAS!
abha	1	5110(0.011432491139819366)	2359 (0.034)	#SAKNAS!
abha	1	5110(0.011432491139819366)	2359 (0.034)	#SAKNAS!
abies	1	5110(0.011432491139819366)	2359 (0.034)	#SAKNAS!
abies	1	5110(0.011432491139819366)	2359 (0.034)	#SAKNAS!
abilities	1	5110(0.011432491139819366)	2359 (0.034)	#SAKNAS!
abilities	1	5110(0.011432491139819366)	2359 (0.034)	#SAKNAS!
abiotic	1	5110(0.011432491139819366)	#SAKNAS!	2548 (0.033)
aboriginal	1	1894(0.05716245569909683)	2359 (0.034)	1233 (0.1)
aborigine	1	5110(0.011432491139819366)	2359 (0.034)	#SAKNAS!
aborigine	1	5110(0.011432491139819366)	2359 (0.034)	#SAKNAS!
about-child	1	5110(0.011432491139819366)	#SAKNAS!	#SAKNAS!
abrupt	1	5110(0.011432491139819366)	#SAKNAS!	2548 (0.033)
abscesses	1	5110(0.011432491139819366)	#SAKNAS!	2548 (0.033)
abscisic	1	5110(0.011432491139819366)	#SAKNAS!	2548 (0.033)
absence	1	2693(0.0342974734194581)	2359 (0.034)	2548 (0.033)
absent	1	5110(0.011432491139819366)	2359 (0.034)	#SAKNAS!
absent	1	5110(0.011432491139819366)	2359 (0.034)	#SAKNAS!
absi	1	5110(0.011432491139819366)	#SAKNAS!	#SAKNAS!
absorbable	1	5110(0.011432491139819366)	#SAKNAS!	#SAKNAS!
absorptiometry	1	5110(0.011432491139819366)	2359 (0.034)	#SAKNAS!
absorptiometry	1	5110(0.011432491139819366)	2359 (0.034)	#SAKNAS!
absorptive	1	5110(0.011432491139819366)	2359 (0.034)	#SAKNAS!
absorptive	1	5110(0.011432491139819366)	2359 (0.034)	#SAKNAS!
abuse-deterrent	1	5110(0.011432491139819366)	#SAKNAS!	2548 (0.033)
academic	1	5110(0.011432491139819366)	#SAKNAS!	#SAKNAS!
accelerates	1	5110(0.011432491139819366)	#SAKNAS!	2548 (0.033)
accelerometry	1	5110(0.011432491139819366)	2359 (0.034)	#SAKNAS!
accelerometry	1	5110(0.011432491139819366)	2359 (0.034)	#SAKNAS!
accent>	1	5110(0.011432491139819366)	#SAKNAS!	#SAKNAS!
accessible	1	3437(0.02286498227963873)	2359 (0.034)	#SAKNAS!
accessing	1	5110(0.011432491139819366)	#SAKNAS!	2548 (0.033)
accessions	1	5110(0.011432491139819366)	#SAKNAS!	#SAKNAS!
access-time	1	5110(0.011432491139819366)	#SAKNAS!	#SAKNAS!
accident	1	5110(0.011432491139819366)	2359 (0.034)	#SAKNAS!
accident	1	5110(0.011432491139819366)	2359 (0.034)	#SAKNAS!
accidental	1	5110(0.011432491139819366)	#SAKNAS!	2548 (0.033)
accidents	1	5110(0.011432491139819366)	2359 (0.034)	#SAKNAS!
accidents	1	5110(0.011432491139819366)	2359 (0.034)	#SAKNAS!
accommodation	1	3437(0.02286498227963873)	2359 (0.034)	#SAKNAS!

account	1	5110(0.011432491139819366)	#SAKNAS!	#SAKNAS!
accountable	1	5110(0.011432491139819366)	#SAKNAS!	2548 (0.033)
accumulate	1	5110(0.011432491139819366)	2359 (0.034)	#SAKNAS!
accumulate	1	5110(0.011432491139819366)	2359 (0.034)	#SAKNAS!
acetate	1	2693(0.0342974734194581)	2359 (0.034)	2548 (0.033)
acetylcholine	1	5110(0.011432491139819366)	#SAKNAS!	#SAKNAS!
achieve	1	3437(0.02286498227963873)	2359 (0.034)	#SAKNAS!
achievers	1	5110(0.011432491139819366)	2359 (0.034)	#SAKNAS!
achievers	1	5110(0.011432491139819366)	2359 (0.034)	#SAKNAS!
acholi	1	5110(0.011432491139819366)	#SAKNAS!	2548 (0.033)
acid-based	1	5110(0.011432491139819366)	2359 (0.034)	#SAKNAS!
acid-based	1	5110(0.011432491139819366)	2359 (0.034)	#SAKNAS!
acid-deprived	1	5110(0.011432491139819366)	#SAKNAS!	2548 (0.033)
acid-fortified	1	5110(0.011432491139819366)	#SAKNAS!	#SAKNAS!
acid-imbalanced	1	5110(0.011432491139819366)	2359 (0.034)	#SAKNAS!
acid-imbalanced	1	5110(0.011432491139819366)	2359 (0.034)	#SAKNAS!
acidity	1	5110(0.011432491139819366)	#SAKNAS!	2548 (0.033)
acidophilus	1	5110(0.011432491139819366)	#SAKNAS!	#SAKNAS!
acidosis	1	3437(0.02286498227963873)	2359 (0.034)	2548 (0.033)
aciduria	1	5110(0.011432491139819366)	2359 (0.034)	#SAKNAS!
aciduria	1	5110(0.011432491139819366)	2359 (0.034)	#SAKNAS!
acinar	1	5110(0.011432491139819366)	2359 (0.034)	#SAKNAS!
acinar	1	5110(0.011432491139819366)	2359 (0.034)	#SAKNAS!
acorn	1	5110(0.011432491139819366)	#SAKNAS!	2548 (0.033)
acoustic	1	5110(0.011432491139819366)	#SAKNAS!	#SAKNAS!
acpas	1	5110(0.011432491139819366)	#SAKNAS!	2548 (0.033)
acquires	1	5110(0.011432491139819366)	2359 (0.034)	#SAKNAS!
acquires	1	5110(0.011432491139819366)	2359 (0.034)	#SAKNAS!
acrididae	1	5110(0.011432491139819366)	2359 (0.034)	#SAKNAS!
acrididae	1	5110(0.011432491139819366)	2359 (0.034)	#SAKNAS!
acromegaly	1	5110(0.011432491139819366)	#SAKNAS!	#SAKNAS!
actinobacillus-actinomycetemco	1	5110(0.011432491139819366)	2359 (0.034)	#SAKNAS!
actinobacillus-actinomycetemco	1	5110(0.011432491139819366)	2359 (0.034)	#SAKNAS!
actinopterygii	1	5110(0.011432491139819366)	2359 (0.034)	#SAKNAS!
actinopterygii	1	5110(0.011432491139819366)	2359 (0.034)	#SAKNAS!
actions	1	2214(0.04572996455927746)	2359 (0.034)	1233 (0.1)
activates	1	2693(0.0342974734194581)	2359 (0.034)	2548 (0.033)
activating	1	5110(0.011432491139819366)	2359 (0.034)	#SAKNAS!
activating	1	5110(0.011432491139819366)	2359 (0.034)	#SAKNAS!
activation	1	1678(0.0685949468389162)	2359 (0.034)	1668 (0.066)
activator	1	3437(0.02286498227963873)	2359 (0.034)	#SAKNAS!
actors	1	5110(0.011432491139819366)	#SAKNAS!	2548 (0.033)
acts	1	5110(0.011432491139819366)	#SAKNAS!	#SAKNAS!

actual	1	5110(0.011432491139819366)	#SAKNAS!	2548 (0.033)
actualisation	1	5110(0.011432491139819366)	#SAKNAS!	2548 (0.033)
actuator	1	5110(0.011432491139819366)	#SAKNAS!	2548 (0.033)
aculeatus	1	5110(0.011432491139819366)	2359 (0.034)	#SAKNAS!
aculeatus	1	5110(0.011432491139819366)	2359 (0.034)	#SAKNAS!
acute-care	1	5110(0.011432491139819366)	#SAKNAS!	2548 (0.033)
acute-leukemia	1	5110(0.011432491139819366)	2359 (0.034)	#SAKNAS!
acute-leukemia	1	5110(0.011432491139819366)	2359 (0.034)	#SAKNAS!
acutely	1	2214(0.04572996455927746)	2359 (0.034)	2548 (0.033)
acute-phase	1	5110(0.011432491139819366)	2359 (0.034)	#SAKNAS!
acute-phase	1	5110(0.011432491139819366)	2359 (0.034)	#SAKNAS!
acute-stroke	1	5110(0.011432491139819366)	2359 (0.034)	#SAKNAS!
acute-stroke	1	5110(0.011432491139819366)	2359 (0.034)	#SAKNAS!
adapt	1	5110(0.011432491139819366)	#SAKNAS!	2548 (0.033)
adapta瘺瓊o	1	5110(0.011432491139819366)	#SAKNAS!	#SAKNAS!
adapting	1	5110(0.011432491139819366)	#SAKNAS!	2548 (0.033)
adaptively	1	5110(0.011432491139819366)	#SAKNAS!	#SAKNAS!
adcabc	1	5110(0.011432491139819366)	#SAKNAS!	#SAKNAS!
additive	1	2693(0.0342974734194581)	2359 (0.034)	2548 (0.033)
additives	1	1335(0.09145992911855493)	2359 (0.034)	1668 (0.066)
address	1	5110(0.011432491139819366)	#SAKNAS!	2548 (0.033)
addressing	1	2693(0.0342974734194581)	2359 (0.034)	2548 (0.033)
adduction	1	5110(0.011432491139819366)	2359 (0.034)	#SAKNAS!
adduction	1	5110(0.011432491139819366)	2359 (0.034)	#SAKNAS!
a-deficient	1	5110(0.011432491139819366)	2359 (0.034)	#SAKNAS!
a-deficient	1	5110(0.011432491139819366)	2359 (0.034)	#SAKNAS!
adelie	1	5110(0.011432491139819366)	#SAKNAS!	#SAKNAS!
adeno-associated	1	5110(0.011432491139819366)	2359 (0.034)	#SAKNAS!
adeno-associated	1	5110(0.011432491139819366)	2359 (0.034)	#SAKNAS!
adenoma	1	5110(0.011432491139819366)	#SAKNAS!	2548 (0.033)
adenotonsillar	1	5110(0.011432491139819366)	#SAKNAS!	#SAKNAS!
adenylate	1	5110(0.011432491139819366)	#SAKNAS!	#SAKNAS!
adequate	1	3437(0.02286498227963873)	2359 (0.034)	2548 (0.033)
adequately	1	3437(0.02286498227963873)	2359 (0.034)	#SAKNAS!
adhd	1	1894(0.05716245569909683)	2359 (0.034)	1233 (0.1)
adhesive	1	5110(0.011432491139819366)	#SAKNAS!	2548 (0.033)
adipocyte	1	5110(0.011432491139819366)	2359 (0.034)	#SAKNAS!
adipocyte	1	5110(0.011432491139819366)	2359 (0.034)	#SAKNAS!
adipocytes	1	5110(0.011432491139819366)	#SAKNAS!	2548 (0.033)
adiponectin	1	1894(0.05716245569909683)	2359 (0.034)	999 (0.13)
adipoq-rs1501299	1	5110(0.011432491139819366)	#SAKNAS!	2548 (0.033)
adipostat	1	5110(0.011432491139819366)	2359 (0.034)	#SAKNAS!
adipostat	1	5110(0.011432491139819366)	2359 (0.034)	#SAKNAS!

adipsin	1	5110(0.011432491139819366)	#SAKNAS!	#SAKNAS!
adjacent	1	3437(0.02286498227963873)	2359 (0.034)	#SAKNAS!
adjustments	1	5110(0.011432491139819366)	#SAKNAS!	2548 (0.033)
adjusts	1	5110(0.011432491139819366)	#SAKNAS!	#SAKNAS!
adl	1	5110(0.011432491139819366)	#SAKNAS!	2548 (0.033)
ad-libitum	1	3437(0.02286498227963873)	2359 (0.034)	2548 (0.033)
adls	1	5110(0.011432491139819366)	#SAKNAS!	2548 (0.033)
administer	1	5110(0.011432491139819366)	#SAKNAS!	#SAKNAS!
adolescents-a	1	5110(0.011432491139819366)	#SAKNAS!	#SAKNAS!
adolescentsresults	1	5110(0.011432491139819366)	#SAKNAS!	2548 (0.033)
adrenal	1	5110(0.011432491139819366)	#SAKNAS!	#SAKNAS!
adrenomedullin	1	5110(0.011432491139819366)	#SAKNAS!	#SAKNAS!
adropin	1	5110(0.011432491139819366)	#SAKNAS!	2548 (0.033)
adsorbents	1	5110(0.011432491139819366)	#SAKNAS!	#SAKNAS!
adult-onset	1	5110(0.011432491139819366)	#SAKNAS!	#SAKNAS!
adults-a	1	5110(0.011432491139819366)	#SAKNAS!	#SAKNAS!
adults-evaluation	1	5110(0.011432491139819366)	#SAKNAS!	2548 (0.033)
advance	1	5110(0.011432491139819366)	#SAKNAS!	#SAKNAS!
advantages	1	5110(0.011432491139819366)	2359 (0.034)	#SAKNAS!
advantages	1	5110(0.011432491139819366)	2359 (0.034)	#SAKNAS!
advent	1	5110(0.011432491139819366)	2359 (0.034)	#SAKNAS!
advent	1	5110(0.011432491139819366)	2359 (0.034)	#SAKNAS!
adverse-effects	1	5110(0.011432491139819366)	2359 (0.034)	#SAKNAS!
adverse-effects	1	5110(0.011432491139819366)	2359 (0.034)	#SAKNAS!
advertisements	1	2214(0.04572996455927746)	2359 (0.034)	1233 (0.1)
advertising	1	5110(0.011432491139819366)	#SAKNAS!	2548 (0.033)
advisability	1	5110(0.011432491139819366)	#SAKNAS!	#SAKNAS!
advise	1	5110(0.011432491139819366)	2359 (0.034)	#SAKNAS!
advise	1	5110(0.011432491139819366)	2359 (0.034)	#SAKNAS!
advocating	1	5110(0.011432491139819366)	#SAKNAS!	2548 (0.033)
aedes	1	2693(0.0342974734194581)	2359 (0.034)	2548 (0.033)
aegean	1	5110(0.011432491139819366)	#SAKNAS!	#SAKNAS!
aegypti	1	3437(0.02286498227963873)	2359 (0.034)	#SAKNAS!
aerobic	1	5110(0.011432491139819366)	#SAKNAS!	#SAKNAS!
aeruginosa	1	5110(0.011432491139819366)	#SAKNAS!	#SAKNAS!
aetiology	1	5110(0.011432491139819366)	2359 (0.034)	#SAKNAS!
aetiology	1	5110(0.011432491139819366)	2359 (0.034)	#SAKNAS!
afferents	1	5110(0.011432491139819366)	#SAKNAS!	2548 (0.033)
affinis	1	5110(0.011432491139819366)	2359 (0.034)	#SAKNAS!
affinis	1	5110(0.011432491139819366)	2359 (0.034)	#SAKNAS!
affluent	1	5110(0.011432491139819366)	#SAKNAS!	2548 (0.033)
afford	1	5110(0.011432491139819366)	#SAKNAS!	#SAKNAS!
affordability	1	5110(0.011432491139819366)	#SAKNAS!	2548 (0.033)

affordable	1	5110(0.011432491139819366)	#SAKNAS!	#SAKNAS!
afghan	1	5110(0.011432491139819366)	#SAKNAS!	#SAKNAS!
afghanistan	1	5110(0.011432491139819366)	#SAKNAS!	2548 (0.033)
african-american	1	3437(0.02286498227963873)	2359 (0.034)	2548 (0.033)
african-americans	1	5110(0.011432491139819366)	2359 (0.034)	#SAKNAS!
african-americans	1	5110(0.011432491139819366)	2359 (0.034)	#SAKNAS!
agaricus	1	5110(0.011432491139819366)	#SAKNAS!	#SAKNAS!
age-at-death	1	5110(0.011432491139819366)	2359 (0.034)	#SAKNAS!
age-at-death	1	5110(0.011432491139819366)	2359 (0.034)	#SAKNAS!
aged-care	1	5110(0.011432491139819366)	#SAKNAS!	#SAKNAS!
aged-children	1	5110(0.011432491139819366)	#SAKNAS!	#SAKNAS!
age-dependent	1	5110(0.011432491139819366)	2359 (0.034)	#SAKNAS!
age-dependent	1	5110(0.011432491139819366)	2359 (0.034)	#SAKNAS!
age-friendly	1	5110(0.011432491139819366)	#SAKNAS!	#SAKNAS!
age-groups	1	5110(0.011432491139819366)	2359 (0.034)	#SAKNAS!
age-groups	1	5110(0.011432491139819366)	2359 (0.034)	#SAKNAS!
age-independent	1	5110(0.011432491139819366)	2359 (0.034)	#SAKNAS!
age-independent	1	5110(0.011432491139819366)	2359 (0.034)	#SAKNAS!
age-period-cohort	1	5110(0.011432491139819366)	#SAKNAS!	2548 (0.033)
age-when	1	5110(0.011432491139819366)	#SAKNAS!	#SAKNAS!
aggravate	1	5110(0.011432491139819366)	#SAKNAS!	2548 (0.033)
aggravates	1	5110(0.011432491139819366)	#SAKNAS!	2548 (0.033)
aggravation	1	5110(0.011432491139819366)	2359 (0.034)	#SAKNAS!
aggravation	1	5110(0.011432491139819366)	2359 (0.034)	#SAKNAS!
aggregation	1	3437(0.02286498227963873)	2359 (0.034)	2548 (0.033)
aging-a	1	5110(0.011432491139819366)	#SAKNAS!	2548 (0.033)
agonist	1	3437(0.02286498227963873)	2359 (0.034)	2548 (0.033)
agonistic	1	3437(0.02286498227963873)	2359 (0.034)	#SAKNAS!
agonists	1	5110(0.011432491139819366)	#SAKNAS!	2548 (0.033)
agouti	1	5110(0.011432491139819366)	2359 (0.034)	#SAKNAS!
agouti	1	5110(0.011432491139819366)	2359 (0.034)	#SAKNAS!
agouti-related	1	5110(0.011432491139819366)	#SAKNAS!	2548 (0.033)
agreement	1	3437(0.02286498227963873)	2359 (0.034)	2548 (0.033)
agrestis	1	5110(0.011432491139819366)	2359 (0.034)	#SAKNAS!
agrestis	1	5110(0.011432491139819366)	2359 (0.034)	#SAKNAS!
agribusiness	1	5110(0.011432491139819366)	#SAKNAS!	#SAKNAS!
agro-ecological	1	5110(0.011432491139819366)	2359 (0.034)	#SAKNAS!
agro-ecological	1	5110(0.011432491139819366)	2359 (0.034)	#SAKNAS!
a-hei	1	5110(0.011432491139819366)	#SAKNAS!	#SAKNAS!
ahli	1	5110(0.011432491139819366)	2359 (0.034)	#SAKNAS!
ahli	1	5110(0.011432491139819366)	2359 (0.034)	#SAKNAS!
ai	1	5110(0.011432491139819366)	2359 (0.034)	#SAKNAS!
ai	1	5110(0.011432491139819366)	2359 (0.034)	#SAKNAS!

aid	1	2214(0.04572996455927746)	2359 (0.034)	2548 (0.033)
aileu	1	5110(0.011432491139819366)	#SAKNAS!	2548 (0.033)
ailuropoda	1	5110(0.011432491139819366)	2359 (0.034)	#SAKNAS!
ailuropoda	1	5110(0.011432491139819366)	2359 (0.034)	#SAKNAS!
aiming	1	5110(0.011432491139819366)	#SAKNAS!	2548 (0.033)
aiptasia	1	5110(0.011432491139819366)	#SAKNAS!	#SAKNAS!
ait	1	5110(0.011432491139819366)	2359 (0.034)	#SAKNAS!
ait	1	5110(0.011432491139819366)	2359 (0.034)	#SAKNAS!
al	1	5110(0.011432491139819366)	#SAKNAS!	#SAKNAS!
alagille	1	5110(0.011432491139819366)	2359 (0.034)	#SAKNAS!
alagille	1	5110(0.011432491139819366)	2359 (0.034)	#SAKNAS!
al-ahsa	1	5110(0.011432491139819366)	#SAKNAS!	#SAKNAS!
alarm	1	5110(0.011432491139819366)	2359 (0.034)	#SAKNAS!
alarm	1	5110(0.011432491139819366)	2359 (0.034)	#SAKNAS!
albanian	1	5110(0.011432491139819366)	#SAKNAS!	#SAKNAS!
albatrosses	1	5110(0.011432491139819366)	2359 (0.034)	#SAKNAS!
albatrosses	1	5110(0.011432491139819366)	2359 (0.034)	#SAKNAS!
alberta	1	5110(0.011432491139819366)	#SAKNAS!	#SAKNAS!
albicans	1	2693(0.0342974734194581)	2359 (0.034)	#SAKNAS!
albocinereus	1	5110(0.011432491139819366)	2359 (0.034)	#SAKNAS!
albocinereus	1	5110(0.011432491139819366)	2359 (0.034)	#SAKNAS!
albopictus	1	3437(0.02286498227963873)	2359 (0.034)	2548 (0.033)
albuminuria	1	5110(0.011432491139819366)	#SAKNAS!	2548 (0.033)
alcohol-dependent	1	5110(0.011432491139819366)	#SAKNAS!	2548 (0.033)
alcoholic	1	5110(0.011432491139819366)	#SAKNAS!	#SAKNAS!
aldehyde	1	5110(0.011432491139819366)	#SAKNAS!	#SAKNAS!
aldolase	1	5110(0.011432491139819366)	2359 (0.034)	#SAKNAS!
aldolase	1	5110(0.011432491139819366)	2359 (0.034)	#SAKNAS!
alectoris	1	5110(0.011432491139819366)	#SAKNAS!	#SAKNAS!
alegranza	1	5110(0.011432491139819366)	2359 (0.034)	#SAKNAS!
alegranza	1	5110(0.011432491139819366)	2359 (0.034)	#SAKNAS!
alegre	1	5110(0.011432491139819366)	2359 (0.034)	#SAKNAS!
alegre	1	5110(0.011432491139819366)	2359 (0.034)	#SAKNAS!
alendronate	1	5110(0.011432491139819366)	2359 (0.034)	#SAKNAS!
alendronate	1	5110(0.011432491139819366)	2359 (0.034)	#SAKNAS!
alerts	1	5110(0.011432491139819366)	#SAKNAS!	2548 (0.033)
alessandro	1	5110(0.011432491139819366)	#SAKNAS!	#SAKNAS!
alexandri	1	5110(0.011432491139819366)	#SAKNAS!	2548 (0.033)
alfalfa-pellet	1	5110(0.011432491139819366)	2359 (0.034)	#SAKNAS!
alfalfa-pellet	1	5110(0.011432491139819366)	2359 (0.034)	#SAKNAS!
alfalfa-ryegrass	1	5110(0.011432491139819366)	#SAKNAS!	#SAKNAS!
algae	1	2214(0.04572996455927746)	2359 (0.034)	2548 (0.033)
algae-herbivore	1	5110(0.011432491139819366)	#SAKNAS!	#SAKNAS!

algeria	1	3437(0.0228649822796387З)	2359 (0.034)	#SAKNAS!
alginates	1	5110(0.011432491139819366)	2359 (0.034)	#SAKNAS!
alginates	1	5110(0.011432491139819366)	2359 (0.034)	#SAKNAS!
algirus	1	5110(0.011432491139819366)	#SAKNAS!	2548 (0.033)
algivore	1	5110(0.011432491139819366)	2359 (0.034)	#SAKNAS!
algivore	1	5110(0.011432491139819366)	2359 (0.034)	#SAKNAS!
aliado	1	5110(0.011432491139819366)	#SAKNAS!	#SAKNAS!
alien	1	5110(0.011432491139819366)	2359 (0.034)	#SAKNAS!
alien	1	5110(0.011432491139819366)	2359 (0.034)	#SAKNAS!
alike	1	5110(0.011432491139819366)	2359 (0.034)	#SAKNAS!
alike	1	5110(0.011432491139819366)	2359 (0.034)	#SAKNAS!
alimentation	1	5110(0.011432491139819366)	#SAKNAS!	2548 (0.033)
alkaline-phosphatases	1	5110(0.011432491139819366)	2359 (0.034)	#SAKNAS!
alkaline-phosphatases	1	5110(0.011432491139819366)	2359 (0.034)	#SAKNAS!
alkaloids	1	5110(0.011432491139819366)	2359 (0.034)	#SAKNAS!
alkaloids	1	5110(0.011432491139819366)	2359 (0.034)	#SAKNAS!
alkyl	1	5110(0.011432491139819366)	#SAKNAS!	2548 (0.033)
allelic	1	5110(0.011432491139819366)	#SAKNAS!	2548 (0.033)
allelochemical	1	5110(0.011432491139819366)	2359 (0.034)	#SAKNAS!
allelochemical	1	5110(0.011432491139819366)	2359 (0.034)	#SAKNAS!
allelochemicals	1	5110(0.011432491139819366)	2359 (0.034)	#SAKNAS!
allelochemicals	1	5110(0.011432491139819366)	2359 (0.034)	#SAKNAS!
allergies	1	5110(0.011432491139819366)	#SAKNAS!	#SAKNAS!
allergy	1	5110(0.011432491139819366)	2359 (0.034)	#SAKNAS!
allergy	1	5110(0.011432491139819366)	2359 (0.034)	#SAKNAS!
alleviate	1	5110(0.011432491139819366)	#SAKNAS!	#SAKNAS!
alleviates	1	3437(0.0228649822796387З)	2359 (0.034)	#SAKNAS!
alleviating	1	5110(0.011432491139819366)	#SAKNAS!	2548 (0.033)
alliance	1	5110(0.011432491139819366)	#SAKNAS!	#SAKNAS!
allobates	1	3437(0.0228649822796387З)	2359 (0.034)	#SAKNAS!
allocation	1	3437(0.0228649822796387З)	2359 (0.034)	2548 (0.033)
allogeneic	1	5110(0.011432491139819366)	2359 (0.034)	#SAKNAS!
allogeneic	1	5110(0.011432491139819366)	2359 (0.034)	#SAKNAS!
allosucking	1	5110(0.011432491139819366)	2359 (0.034)	#SAKNAS!
allosucking	1	5110(0.011432491139819366)	2359 (0.034)	#SAKNAS!
allowance	1	5110(0.011432491139819366)	#SAKNAS!	2548 (0.033)
alluvial	1	5110(0.011432491139819366)	2359 (0.034)	#SAKNAS!
alluvial	1	5110(0.011432491139819366)	2359 (0.034)	#SAKNAS!
almada-seixal	1	5110(0.011432491139819366)	#SAKNAS!	2548 (0.033)
almonds	1	5110(0.011432491139819366)	2359 (0.034)	#SAKNAS!
almonds	1	5110(0.011432491139819366)	2359 (0.034)	#SAKNAS!
alone	1	1678(0.0685949468389162)	2359 (0.034)	1233 (0.1)
alosa	1	5110(0.011432491139819366)	#SAKNAS!	2548 (0.033)

alouatta	1	3437(0.02286498227963873)	2359 (0.034)	2548 (0.033)
alpelisib-related	1	5110(0.011432491139819366)	#SAKNAS!	#SAKNAS!
alpha-amylase	1	5110(0.011432491139819366)	2359 (0.034)	#SAKNAS!
alpha-amylase	1	5110(0.011432491139819366)	2359 (0.034)	#SAKNAS!
alpha-ketoglutarate	1	5110(0.011432491139819366)	2359 (0.034)	#SAKNAS!
alpha-ketoglutarate	1	5110(0.011432491139819366)	2359 (0.034)	#SAKNAS!
alpha-melanocyte-stimulating	1	5110(0.011432491139819366)	#SAKNAS!	2548 (0.033)
alpha-tocopherol	1	5110(0.011432491139819366)	#SAKNAS!	#SAKNAS!
alpine	1	3437(0.02286498227963873)	2359 (0.034)	#SAKNAS!
alpinus	1	5110(0.011432491139819366)	2359 (0.034)	#SAKNAS!
alpinus	1	5110(0.011432491139819366)	2359 (0.034)	#SAKNAS!
alspac	1	5110(0.011432491139819366)	2359 (0.034)	#SAKNAS!
alspac	1	5110(0.011432491139819366)	2359 (0.034)	#SAKNAS!
altering	1	2214(0.04572996455927746)	2359 (0.034)	2548 (0.033)
alternation	1	5110(0.011432491139819366)	#SAKNAS!	2548 (0.033)
alumni	1	5110(0.011432491139819366)	#SAKNAS!	#SAKNAS!
amarali	1	5110(0.011432491139819366)	#SAKNAS!	#SAKNAS!
amazon	1	2693(0.0342974734194581)	2359 (0.034)	1668 (0.066)
amazona	1	5110(0.011432491139819366)	2359 (0.034)	#SAKNAS!
amazona	1	5110(0.011432491139819366)	2359 (0.034)	#SAKNAS!
amazonas	1	5110(0.011432491139819366)	#SAKNAS!	#SAKNAS!
amazons	1	5110(0.011432491139819366)	2359 (0.034)	#SAKNAS!
amazons	1	5110(0.011432491139819366)	2359 (0.034)	#SAKNAS!
ambidirectional	1	5110(0.011432491139819366)	#SAKNAS!	#SAKNAS!
ambulation	1	5110(0.011432491139819366)	#SAKNAS!	#SAKNAS!
ambystomatid	1	5110(0.011432491139819366)	#SAKNAS!	#SAKNAS!
ameerega	1	5110(0.011432491139819366)	#SAKNAS!	#SAKNAS!
amel	1	5110(0.011432491139819366)	#SAKNAS!	2548 (0.033)
ameliorate	1	5110(0.011432491139819366)	#SAKNAS!	#SAKNAS!
ameliorates	1	5110(0.011432491139819366)	#SAKNAS!	2548 (0.033)
amenorrhea	1	5110(0.011432491139819366)	2359 (0.034)	#SAKNAS!
amenorrhea	1	5110(0.011432491139819366)	2359 (0.034)	#SAKNAS!
amid	1	5110(0.011432491139819366)	#SAKNAS!	#SAKNAS!
amifostine	1	5110(0.011432491139819366)	2359 (0.034)	#SAKNAS!
amifostine	1	5110(0.011432491139819366)	2359 (0.034)	#SAKNAS!
amino-acid	1	5110(0.011432491139819366)	2359 (0.034)	#SAKNAS!
amino-acid	1	5110(0.011432491139819366)	2359 (0.034)	#SAKNAS!
aminopeptidase	1	5110(0.011432491139819366)	2359 (0.034)	#SAKNAS!
aminopeptidase	1	5110(0.011432491139819366)	2359 (0.034)	#SAKNAS!
ammonia	1	5110(0.011432491139819366)	2359 (0.034)	#SAKNAS!
ammonia	1	5110(0.011432491139819366)	2359 (0.034)	#SAKNAS!
amp	1	5110(0.011432491139819366)	#SAKNAS!	2548 (0.033)
amparafaravola	1	5110(0.011432491139819366)	#SAKNAS!	#SAKNAS!

amphioctopus	1	5110(0.011432491139819366)	#SAKNAS!	#SAKNAS!
amphipod	1	3437(0.02286498227963873)	2359 (0.034)	2548 (0.033)
amphiura	1	5110(0.011432491139819366)	2359 (0.034)	#SAKNAS!
amphiura	1	5110(0.011432491139819366)	2359 (0.034)	#SAKNAS!
ampk	1	5110(0.011432491139819366)	#SAKNAS!	#SAKNAS!
ampk-mtor	1	5110(0.011432491139819366)	#SAKNAS!	#SAKNAS!
amsan	1	5110(0.011432491139819366)	#SAKNAS!	#SAKNAS!
amur	1	5110(0.011432491139819366)	#SAKNAS!	#SAKNAS!
amygdalar	1	5110(0.011432491139819366)	2359 (0.034)	#SAKNAS!
amygdalar	1	5110(0.011432491139819366)	2359 (0.034)	#SAKNAS!
anabas	1	5110(0.011432491139819366)	#SAKNAS!	#SAKNAS!
anacardiaceae	1	5110(0.011432491139819366)	2359 (0.034)	#SAKNAS!
anacardiaceae	1	5110(0.011432491139819366)	2359 (0.034)	#SAKNAS!
anaemia	1	5110(0.011432491139819366)	#SAKNAS!	#SAKNAS!
anaesthesia	1	5110(0.011432491139819366)	#SAKNAS!	2548 (0.033)
anahita	1	5110(0.011432491139819366)	2359 (0.034)	#SAKNAS!
anahita	1	5110(0.011432491139819366)	2359 (0.034)	#SAKNAS!
anal	1	5110(0.011432491139819366)	#SAKNAS!	#SAKNAS!
anality	1	5110(0.011432491139819366)	2359 (0.034)	#SAKNAS!
anality	1	5110(0.011432491139819366)	2359 (0.034)	#SAKNAS!
analysing	1	5110(0.011432491139819366)	#SAKNAS!	2548 (0.033)
analysis???	1	5110(0.011432491139819366)	#SAKNAS!	#SAKNAS!
analytic	1	5110(0.011432491139819366)	#SAKNAS!	#SAKNAS!
analytical	1	2693(0.0342974734194581)	2359 (0.034)	#SAKNAS!
analytics	1	5110(0.011432491139819366)	#SAKNAS!	#SAKNAS!
analyze	1	5110(0.011432491139819366)	#SAKNAS!	#SAKNAS!
analyzes	1	5110(0.011432491139819366)	#SAKNAS!	2548 (0.033)
analyzing	1	3437(0.02286498227963873)	2359 (0.034)	2548 (0.033)
anaplerosis	1	5110(0.011432491139819366)	#SAKNAS!	2548 (0.033)
anastomosis	1	1335(0.09145992911855493)	2359 (0.034)	999 (0.13)
anatinus	1	5110(0.011432491139819366)	2359 (0.034)	#SAKNAS!
anatinus	1	5110(0.011432491139819366)	2359 (0.034)	#SAKNAS!
anatolia	1	5110(0.011432491139819366)	#SAKNAS!	#SAKNAS!
anatomical	1	2693(0.0342974734194581)	2359 (0.034)	#SAKNAS!
anchor-based	1	5110(0.011432491139819366)	#SAKNAS!	#SAKNAS!
ancillary	1	5110(0.011432491139819366)	2359 (0.034)	#SAKNAS!
ancillary	1	5110(0.011432491139819366)	2359 (0.034)	#SAKNAS!
and-9	1	5110(0.011432491139819366)	2359 (0.034)	#SAKNAS!
and-9	1	5110(0.011432491139819366)	2359 (0.034)	#SAKNAS!
anderson	1	5110(0.011432491139819366)	#SAKNAS!	#SAKNAS!
andes	1	5110(0.011432491139819366)	2359 (0.034)	#SAKNAS!
andes	1	5110(0.011432491139819366)	2359 (0.034)	#SAKNAS!
andrei	1	5110(0.011432491139819366)	#SAKNAS!	2548 (0.033)

anelosimus	1	5110(0.011432491139819366)	2359 (0.034)	#SAKNAS!
anelosimus	1	5110(0.011432491139819366)	2359 (0.034)	#SAKNAS!
anesthetic	1	5110(0.011432491139819366)	2359 (0.034)	#SAKNAS!
anesthetic	1	5110(0.011432491139819366)	2359 (0.034)	#SAKNAS!
aneurogeriatric	1	5110(0.011432491139819366)	#SAKNAS!	2548 (0.033)
aneurysm	1	5110(0.011432491139819366)	#SAKNAS!	2548 (0.033)
anganwadi	1	5110(0.011432491139819366)	2359 (0.034)	#SAKNAS!
anganwadi	1	5110(0.011432491139819366)	2359 (0.034)	#SAKNAS!
angeles	1	5110(0.011432491139819366)	#SAKNAS!	2548 (0.033)
angiography	1	5110(0.011432491139819366)	#SAKNAS!	2548 (0.033)
angioplasty	1	5110(0.011432491139819366)	2359 (0.034)	#SAKNAS!
angioplasty	1	5110(0.011432491139819366)	2359 (0.034)	#SAKNAS!
angle	1	1485(0.08002743797873557)	2359 (0.034)	1233 (0.1)
anguilla	1	5110(0.011432491139819366)	2359 (0.034)	#SAKNAS!
anguilla	1	5110(0.011432491139819366)	2359 (0.034)	#SAKNAS!
angular	1	5110(0.011432491139819366)	2359 (0.034)	#SAKNAS!
angular	1	5110(0.011432491139819366)	2359 (0.034)	#SAKNAS!
anhidrosis	1	5110(0.011432491139819366)	2359 (0.034)	#SAKNAS!
anhidrosis	1	5110(0.011432491139819366)	2359 (0.034)	#SAKNAS!
anhydrobiosis	1	5110(0.011432491139819366)	#SAKNAS!	#SAKNAS!
anhydrobiotic	1	5110(0.011432491139819366)	#SAKNAS!	#SAKNAS!
anin	1	5110(0.011432491139819366)	#SAKNAS!	2548 (0.033)
anisotropic	1	5110(0.011432491139819366)	2359 (0.034)	#SAKNAS!
anisotropic	1	5110(0.011432491139819366)	2359 (0.034)	#SAKNAS!
anka	1	5110(0.011432491139819366)	#SAKNAS!	#SAKNAS!
ankylosing	1	3437(0.02286498227963873)	2359 (0.034)	2548 (0.033)
ann	1	5110(0.011432491139819366)	#SAKNAS!	#SAKNAS!
anna	1	5110(0.011432491139819366)	#SAKNAS!	#SAKNAS!
annamensis	1	5110(0.011432491139819366)	#SAKNAS!	2548 (0.033)
annelida	1	5110(0.011432491139819366)	2359 (0.034)	#SAKNAS!
annelida	1	5110(0.011432491139819366)	2359 (0.034)	#SAKNAS!
annual	1	5110(0.011432491139819366)	2359 (0.034)	#SAKNAS!
annual	1	5110(0.011432491139819366)	2359 (0.034)	#SAKNAS!
anomalies	1	5110(0.011432491139819366)	#SAKNAS!	#SAKNAS!
anomaloglossus	1	5110(0.011432491139819366)	2359 (0.034)	#SAKNAS!
anomaloglossus	1	5110(0.011432491139819366)	2359 (0.034)	#SAKNAS!
anomura	1	5110(0.011432491139819366)	2359 (0.034)	#SAKNAS!
anomura	1	5110(0.011432491139819366)	2359 (0.034)	#SAKNAS!
anopheles	1	3437(0.02286498227963873)	2359 (0.034)	2548 (0.033)
anorexia-cachexia	1	5110(0.011432491139819366)	#SAKNAS!	2548 (0.033)
anorexia-nervosa	1	5110(0.011432491139819366)	2359 (0.034)	#SAKNAS!
anorexia-nervosa	1	5110(0.011432491139819366)	2359 (0.034)	#SAKNAS!
anorexic	1	3437(0.02286498227963873)	2359 (0.034)	2548 (0.033)

another	1	5110(0.011432491139819366)	#SAKNAS!	2548 (0.033)
anrs	1	5110(0.011432491139819366)	#SAKNAS!	2548 (0.033)
anser	1	3437(0.02286498227963873)	2359 (0.034)	#SAKNAS!
anser-anser	1	5110(0.011432491139819366)	2359 (0.034)	#SAKNAS!
anser-anser	1	5110(0.011432491139819366)	2359 (0.034)	#SAKNAS!
antacid	1	5110(0.011432491139819366)	#SAKNAS!	#SAKNAS!
antagonistic	1	2693(0.0342974734194581)	2359 (0.034)	#SAKNAS!
antagonists	1	5110(0.011432491139819366)	2359 (0.034)	#SAKNAS!
antagonists	1	5110(0.011432491139819366)	2359 (0.034)	#SAKNAS!
ant-eating	1	5110(0.011432491139819366)	2359 (0.034)	#SAKNAS!
ant-eating	1	5110(0.011432491139819366)	2359 (0.034)	#SAKNAS!
antegrade-retrograde	1	5110(0.011432491139819366)	#SAKNAS!	#SAKNAS!
antenatal	1	5110(0.011432491139819366)	#SAKNAS!	#SAKNAS!
antennal	1	5110(0.011432491139819366)	#SAKNAS!	2548 (0.033)
anterolateral	1	5110(0.011432491139819366)	#SAKNAS!	#SAKNAS!
antheraea	1	5110(0.011432491139819366)	2359 (0.034)	#SAKNAS!
antheraea	1	5110(0.011432491139819366)	2359 (0.034)	#SAKNAS!
anthranilic	1	5110(0.011432491139819366)	#SAKNAS!	#SAKNAS!
anthropological	1	5110(0.011432491139819366)	#SAKNAS!	#SAKNAS!
anthropometrics	1	5110(0.011432491139819366)	#SAKNAS!	#SAKNAS!
anti-apolipoprotein	1	5110(0.011432491139819366)	2359 (0.034)	#SAKNAS!
anti-apolipoprotein	1	5110(0.011432491139819366)	2359 (0.034)	#SAKNAS!
anti-aspiration	1	5110(0.011432491139819366)	#SAKNAS!	#SAKNAS!
anti-asthmatic	1	5110(0.011432491139819366)	#SAKNAS!	#SAKNAS!
antibody-associated	1	5110(0.011432491139819366)	#SAKNAS!	#SAKNAS!
anticancer	1	5110(0.011432491139819366)	#SAKNAS!	#SAKNAS!
anticaries	1	5110(0.011432491139819366)	#SAKNAS!	2548 (0.033)
anticariogenesis	1	5110(0.011432491139819366)	2359 (0.034)	#SAKNAS!
anticariogenesis	1	5110(0.011432491139819366)	2359 (0.034)	#SAKNAS!
anticarsia-gemmatalis	1	5110(0.011432491139819366)	2359 (0.034)	#SAKNAS!
anticarsia-gemmatalis	1	5110(0.011432491139819366)	2359 (0.034)	#SAKNAS!
anticholinergic	1	2693(0.0342974734194581)	2359 (0.034)	#SAKNAS!
anticipatory	1	2693(0.0342974734194581)	2359 (0.034)	2548 (0.033)
anti-defoliate	1	5110(0.011432491139819366)	2359 (0.034)	#SAKNAS!
anti-defoliate	1	5110(0.011432491139819366)	2359 (0.034)	#SAKNAS!
antifeedant	1	5110(0.011432491139819366)	#SAKNAS!	2548 (0.033)
antigen	1	2693(0.0342974734194581)	2359 (0.034)	2548 (0.033)
antigens	1	5110(0.011432491139819366)	2359 (0.034)	#SAKNAS!
antigens	1	5110(0.011432491139819366)	2359 (0.034)	#SAKNAS!
anti-infectious	1	5110(0.011432491139819366)	2359 (0.034)	#SAKNAS!
anti-infectious	1	5110(0.011432491139819366)	2359 (0.034)	#SAKNAS!
antimicrobial	1	1894(0.05716245569909683)	2359 (0.034)	2548 (0.033)
antimuscarinic	1	5110(0.011432491139819366)	#SAKNAS!	#SAKNAS!

anti-nutritional	1	5110(0.011432491139819366)	2359 (0.034)	#SAKNAS!
anti-nutritional	1	5110(0.011432491139819366)	2359 (0.034)	#SAKNAS!
anti-nxp2	1	5110(0.011432491139819366)	#SAKNAS!	#SAKNAS!
antioxidant-rich	1	5110(0.011432491139819366)	#SAKNAS!	2548 (0.033)
antiplaque	1	5110(0.011432491139819366)	2359 (0.034)	#SAKNAS!
antiplaque	1	5110(0.011432491139819366)	2359 (0.034)	#SAKNAS!
anti-predator	1	5110(0.011432491139819366)	2359 (0.034)	#SAKNAS!
anti-predator	1	5110(0.011432491139819366)	2359 (0.034)	#SAKNAS!
antipsychotic	1	3437(0.02286498227963873)	2359 (0.034)	#SAKNAS!
antipsychotics	1	5110(0.011432491139819366)	2359 (0.034)	#SAKNAS!
antipsychotics	1	5110(0.011432491139819366)	2359 (0.034)	#SAKNAS!
antiretroviral-experienced	1	5110(0.011432491139819366)	#SAKNAS!	2548 (0.033)
antisecretory	1	5110(0.011432491139819366)	2359 (0.034)	#SAKNAS!
antisecretory	1	5110(0.011432491139819366)	2359 (0.034)	#SAKNAS!
antiseptic	1	5110(0.011432491139819366)	#SAKNAS!	2548 (0.033)
antixenosis	1	5110(0.011432491139819366)	#SAKNAS!	#SAKNAS!
antral	1	5110(0.011432491139819366)	2359 (0.034)	#SAKNAS!
antral	1	5110(0.011432491139819366)	2359 (0.034)	#SAKNAS!
ants	1	2693(0.0342974734194581)	2359 (0.034)	#SAKNAS!
anura	1	2693(0.0342974734194581)	2359 (0.034)	2548 (0.033)
anxiety	1	1485(0.08002743797873557)	2359 (0.034)	1233 (0.1)
aorta	1	5110(0.011432491139819366)	#SAKNAS!	2548 (0.033)
aortic	1	1485(0.08002743797873557)	2359 (0.034)	1233 (0.1)
aortobronchial	1	5110(0.011432491139819366)	#SAKNAS!	2548 (0.033)
aortoesophageal	1	5110(0.011432491139819366)	#SAKNAS!	2548 (0.033)
aospine	1	5110(0.011432491139819366)	2359 (0.034)	#SAKNAS!
aospine	1	5110(0.011432491139819366)	2359 (0.034)	#SAKNAS!
aotearoa	1	5110(0.011432491139819366)	#SAKNAS!	#SAKNAS!
apama	1	5110(0.011432491139819366)	2359 (0.034)	#SAKNAS!
apama	1	5110(0.011432491139819366)	2359 (0.034)	#SAKNAS!
apart	1	5110(0.011432491139819366)	2359 (0.034)	#SAKNAS!
apart	1	5110(0.011432491139819366)	2359 (0.034)	#SAKNAS!
apatinib	1	5110(0.011432491139819366)	#SAKNAS!	#SAKNAS!
ape	1	5110(0.011432491139819366)	#SAKNAS!	2548 (0.033)
apella	1	5110(0.011432491139819366)	2359 (0.034)	#SAKNAS!
apella	1	5110(0.011432491139819366)	2359 (0.034)	#SAKNAS!
apert	1	5110(0.011432491139819366)	2359 (0.034)	#SAKNAS!
apert	1	5110(0.011432491139819366)	2359 (0.034)	#SAKNAS!
apes	1	3437(0.02286498227963873)	2359 (0.034)	#SAKNAS!
apex	1	5110(0.011432491139819366)	#SAKNAS!	#SAKNAS!
aphagia	1	5110(0.011432491139819366)	2359 (0.034)	#SAKNAS!
aphagia	1	5110(0.011432491139819366)	2359 (0.034)	#SAKNAS!
aphalaridae	1	5110(0.011432491139819366)	#SAKNAS!	2548 (0.033)

aphididae	1	1335(0.09145992911855493)	2359 (0.034)	999 (0.13)
aphidius	1	5110(0.011432491139819366)	2359 (0.034)	#SAKNAS!
aphidius	1	5110(0.011432491139819366)	2359 (0.034)	#SAKNAS!
aphytis-lingnanensis	1	5110(0.011432491139819366)	2359 (0.034)	#SAKNAS!
aphytis-lingnanensis	1	5110(0.011432491139819366)	2359 (0.034)	#SAKNAS!
apiaries	1	5110(0.011432491139819366)	#SAKNAS!	2548 (0.033)
aplodactylus-punctatus	1	5110(0.011432491139819366)	2359 (0.034)	#SAKNAS!
aplodactylus-punctatus	1	5110(0.011432491139819366)	2359 (0.034)	#SAKNAS!
apnoea	1	5110(0.011432491139819366)	#SAKNAS!	2548 (0.033)
apo	1	5110(0.011432491139819366)	2359 (0.034)	#SAKNAS!
apo	1	5110(0.011432491139819366)	2359 (0.034)	#SAKNAS!
apoa	1	5110(0.011432491139819366)	#SAKNAS!	#SAKNAS!
apollonia	1	5110(0.011432491139819366)	#SAKNAS!	2548 (0.033)
appalachian	1	5110(0.011432491139819366)	#SAKNAS!	2548 (0.033)
apparent	1	1894(0.05716245569909683)	2359 (0.034)	2548 (0.033)
appendages	1	5110(0.011432491139819366)	#SAKNAS!	#SAKNAS!
appetite-regulating	1	3437(0.02286498227963873)	2359 (0.034)	2548 (0.033)
apples	1	5110(0.011432491139819366)	#SAKNAS!	2548 (0.033)
appliances	1	5110(0.011432491139819366)	#SAKNAS!	2548 (0.033)
application-based	1	5110(0.011432491139819366)	#SAKNAS!	#SAKNAS!
appraisal	1	5110(0.011432491139819366)	#SAKNAS!	2548 (0.033)
appreciation	1	5110(0.011432491139819366)	#SAKNAS!	#SAKNAS!
apps	1	5110(0.011432491139819366)	#SAKNAS!	#SAKNAS!
aproaches	1	5110(0.011432491139819366)	#SAKNAS!	2548 (0.033)
apron	1	5110(0.011432491139819366)	#SAKNAS!	#SAKNAS!
apulia	1	5110(0.011432491139819366)	#SAKNAS!	#SAKNAS!
aquafeed	1	5110(0.011432491139819366)	#SAKNAS!	#SAKNAS!
aquaporin-3	1	5110(0.011432491139819366)	#SAKNAS!	#SAKNAS!
arabia-a	1	5110(0.011432491139819366)	#SAKNAS!	2548 (0.033)
arabidopsis	1	5110(0.011432491139819366)	2359 (0.034)	#SAKNAS!
arabidopsis	1	5110(0.011432491139819366)	2359 (0.034)	#SAKNAS!
arachidonic-acid	1	5110(0.011432491139819366)	2359 (0.034)	#SAKNAS!
arachidonic-acid	1	5110(0.011432491139819366)	2359 (0.034)	#SAKNAS!
arapaima	1	5110(0.011432491139819366)	#SAKNAS!	2548 (0.033)
aratus	1	5110(0.011432491139819366)	2359 (0.034)	#SAKNAS!
aratus	1	5110(0.011432491139819366)	2359 (0.034)	#SAKNAS!
arboreal	1	5110(0.011432491139819366)	#SAKNAS!	#SAKNAS!
arcage	1	5110(0.011432491139819366)	#SAKNAS!	2548 (0.033)
archaeological	1	5110(0.011432491139819366)	#SAKNAS!	2548 (0.033)
archipelago	1	5110(0.011432491139819366)	2359 (0.034)	#SAKNAS!
archipelago	1	5110(0.011432491139819366)	2359 (0.034)	#SAKNAS!
architecture	1	2693(0.0342974734194581)	2359 (0.034)	#SAKNAS!
arctica	1	5110(0.011432491139819366)	2359 (0.034)	#SAKNAS!

arctica	1 5110(0.011432491139819366)	2359 (0.034)	#SAKNAS!
arcticus	1 5110(0.011432491139819366)	#SAKNAS!	2548 (0.033)
arduino-based	1 5110(0.011432491139819366)	#SAKNAS!	#SAKNAS!
area-defined	1 5110(0.011432491139819366)	2359 (0.034)	#SAKNAS!
area-defined	1 5110(0.011432491139819366)	2359 (0.034)	#SAKNAS!
arena	1 5110(0.011432491139819366)	2359 (0.034)	#SAKNAS!
arena	1 5110(0.011432491139819366)	2359 (0.034)	#SAKNAS!
arfid	1 5110(0.011432491139819366)	#SAKNAS!	#SAKNAS!
argentina	1 2214(0.04572996455927746)	2359 (0.034)	1233 (0.1)
arginine	1 5110(0.011432491139819366)	#SAKNAS!	2548 (0.033)
argopecten	1 5110(0.011432491139819366)	#SAKNAS!	2548 (0.033)
aric	1 2693(0.0342974734194581)	2359 (0.034)	2548 (0.033)
arid	1 5110(0.011432491139819366)	2359 (0.034)	#SAKNAS!
arid	1 5110(0.011432491139819366)	2359 (0.034)	#SAKNAS!
arising	1 5110(0.011432491139819366)	2359 (0.034)	#SAKNAS!
arising	1 5110(0.011432491139819366)	2359 (0.034)	#SAKNAS!
arius	1 5110(0.011432491139819366)	2359 (0.034)	#SAKNAS!
arius	1 5110(0.011432491139819366)	2359 (0.034)	#SAKNAS!
arjuna	1 5110(0.011432491139819366)	2359 (0.034)	#SAKNAS!
arjuna	1 5110(0.011432491139819366)	2359 (0.034)	#SAKNAS!
arm	1 3437(0.02286498227963873)	2359 (0.034)	#SAKNAS!
armata	1 5110(0.011432491139819366)	#SAKNAS!	2548 (0.033)
aroma	1 5110(0.011432491139819366)	#SAKNAS!	2548 (0.033)
aromobatidae	1 3437(0.02286498227963873)	2359 (0.034)	#SAKNAS!
arrangement	1 5110(0.011432491139819366)	2359 (0.034)	#SAKNAS!
arrangement	1 5110(0.011432491139819366)	2359 (0.034)	#SAKNAS!
arrest	1 5110(0.011432491139819366)	2359 (0.034)	#SAKNAS!
arrest	1 5110(0.011432491139819366)	2359 (0.034)	#SAKNAS!
arresting	1 5110(0.011432491139819366)	#SAKNAS!	2548 (0.033)
arsanjan	1 5110(0.011432491139819366)	2359 (0.034)	#SAKNAS!
arsanjan	1 5110(0.011432491139819366)	2359 (0.034)	#SAKNAS!
artemia	1 3437(0.02286498227963873)	2359 (0.034)	2548 (0.033)
arteries	1 5110(0.011432491139819366)	2359 (0.034)	#SAKNAS!
arteries	1 5110(0.011432491139819366)	2359 (0.034)	#SAKNAS!
arthroplasty	1 5110(0.011432491139819366)	#SAKNAS!	#SAKNAS!
arytenoid	1 5110(0.011432491139819366)	2359 (0.034)	#SAKNAS!
arytenoid	1 5110(0.011432491139819366)	2359 (0.034)	#SAKNAS!
ascorbic	1 5110(0.011432491139819366)	2359 (0.034)	#SAKNAS!
ascorbic	1 5110(0.011432491139819366)	2359 (0.034)	#SAKNAS!
ascorbic-acid	1 5110(0.011432491139819366)	2359 (0.034)	#SAKNAS!
ascorbic-acid	1 5110(0.011432491139819366)	2359 (0.034)	#SAKNAS!
asd	1 5110(0.011432491139819366)	#SAKNAS!	2548 (0.033)
asgb	1 5110(0.011432491139819366)	2359 (0.034)	#SAKNAS!

asgb	1	5110(0.011432491139819366)	2359 (0.034)	#SAKNAS!
asian	1	1027(0.12575740253801304)	2359 (0.034)	1233 (0.1)
asians	1	3437(0.02286498227963873)	2359 (0.034)	2548 (0.033)
asiatic	1	5110(0.011432491139819366)	2359 (0.034)	#SAKNAS!
asiatic	1	5110(0.011432491139819366)	2359 (0.034)	#SAKNAS!
asiatica	1	5110(0.011432491139819366)	2359 (0.034)	#SAKNAS!
asiatica	1	5110(0.011432491139819366)	2359 (0.034)	#SAKNAS!
aspect	1	3437(0.02286498227963873)	2359 (0.034)	#SAKNAS!
aspen	1	5110(0.011432491139819366)	2359 (0.034)	#SAKNAS!
aspen	1	5110(0.011432491139819366)	2359 (0.034)	#SAKNAS!
aspergillus	1	5110(0.011432491139819366)	2359 (0.034)	#SAKNAS!
aspergillus	1	5110(0.011432491139819366)	2359 (0.034)	#SAKNAS!
asphaltic	1	5110(0.011432491139819366)	#SAKNAS!	2548 (0.033)
aspirational	1	5110(0.011432491139819366)	#SAKNAS!	#SAKNAS!
aspiration-related	1	5110(0.011432491139819366)	#SAKNAS!	#SAKNAS!
aspirations	1	5110(0.011432491139819366)	#SAKNAS!	#SAKNAS!
asprosin	1	5110(0.011432491139819366)	#SAKNAS!	#SAKNAS!
assamensis	1	5110(0.011432491139819366)	2359 (0.034)	#SAKNAS!
assamensis	1	5110(0.011432491139819366)	2359 (0.034)	#SAKNAS!
assamese	1	2214(0.04572996455927746)	2359 (0.034)	1668 (0.066)
assaying	1	5110(0.011432491139819366)	#SAKNAS!	2548 (0.033)
assemblage	1	5110(0.011432491139819366)	2359 (0.034)	#SAKNAS!
assemblage	1	5110(0.011432491139819366)	2359 (0.034)	#SAKNAS!
assertive	1	5110(0.011432491139819366)	2359 (0.034)	#SAKNAS!
assertive	1	5110(0.011432491139819366)	2359 (0.034)	#SAKNAS!
assessor-blinded	1	5110(0.011432491139819366)	#SAKNAS!	2548 (0.033)
assignment	1	3437(0.02286498227963873)	2359 (0.034)	2548 (0.033)
assistance	1	2693(0.0342974734194581)	2359 (0.034)	#SAKNAS!
assistant	1	5110(0.011432491139819366)	#SAKNAS!	2548 (0.033)
associate	1	5110(0.011432491139819366)	#SAKNAS!	2548 (0.033)
associative	1	5110(0.011432491139819366)	#SAKNAS!	2548 (0.033)
assortative	1	5110(0.011432491139819366)	2359 (0.034)	#SAKNAS!
assortative	1	5110(0.011432491139819366)	2359 (0.034)	#SAKNAS!
assuaging	1	5110(0.011432491139819366)	2359 (0.034)	#SAKNAS!
assuaging	1	5110(0.011432491139819366)	2359 (0.034)	#SAKNAS!
asteraceae	1	5110(0.011432491139819366)	2359 (0.034)	#SAKNAS!
asteraceae	1	5110(0.011432491139819366)	2359 (0.034)	#SAKNAS!
asteroidea	1	5110(0.011432491139819366)	2359 (0.034)	#SAKNAS!
asteroidea	1	5110(0.011432491139819366)	2359 (0.034)	#SAKNAS!
asthmatic	1	5110(0.011432491139819366)	#SAKNAS!	#SAKNAS!
astrangia	1	5110(0.011432491139819366)	#SAKNAS!	2548 (0.033)
astropecten-articulatus	1	5110(0.011432491139819366)	2359 (0.034)	#SAKNAS!
astropecten-articulatus	1	5110(0.011432491139819366)	2359 (0.034)	#SAKNAS!

asymmetric	1	2693(0.0342974734194581)	2359 (0.034)	1668 (0.066)
asymmetricum	1	5110(0.011432491139819366)	#SAKNAS!	2548 (0.033)
asymmetry	1	5110(0.011432491139819366)	2359 (0.034)	#SAKNAS!
asymmetry	1	5110(0.011432491139819366)	2359 (0.034)	#SAKNAS!
asymptomatic	1	2214(0.04572996455927746)	2359 (0.034)	2548 (0.033)
ataxia-a	1	5110(0.011432491139819366)	#SAKNAS!	2548 (0.033)
ataxias	1	5110(0.011432491139819366)	#SAKNAS!	#SAKNAS!
ataxia-telangiectasia	1	5110(0.011432491139819366)	2359 (0.034)	#SAKNAS!
ataxia-telangiectasia	1	5110(0.011432491139819366)	2359 (0.034)	#SAKNAS!
atelerix	1	5110(0.011432491139819366)	#SAKNAS!	2548 (0.033)
ateles	1	5110(0.011432491139819366)	2359 (0.034)	#SAKNAS!
ateles	1	5110(0.011432491139819366)	2359 (0.034)	#SAKNAS!
ater	1	5110(0.011432491139819366)	2359 (0.034)	#SAKNAS!
ater	1	5110(0.011432491139819366)	2359 (0.034)	#SAKNAS!
atherogenesis	1	5110(0.011432491139819366)	2359 (0.034)	#SAKNAS!
atherogenesis	1	5110(0.011432491139819366)	2359 (0.034)	#SAKNAS!
atheroprotective	1	5110(0.011432491139819366)	#SAKNAS!	2548 (0.033)
atherosclerotic	1	2693(0.0342974734194581)	2359 (0.034)	#SAKNAS!
athlete	1	5110(0.011432491139819366)	#SAKNAS!	#SAKNAS!
athletes-an	1	5110(0.011432491139819366)	#SAKNAS!	#SAKNAS!
athletic	1	5110(0.011432491139819366)	#SAKNAS!	2548 (0.033)
at-home	1	5110(0.011432491139819366)	#SAKNAS!	2548 (0.033)
athospital	1	5110(0.011432491139819366)	#SAKNAS!	2548 (0.033)
atmospheric	1	3437(0.02286498227963873)	2359 (0.034)	2548 (0.033)
atp	1	3437(0.02286498227963873)	2359 (0.034)	2548 (0.033)
atra	1	5110(0.011432491139819366)	#SAKNAS!	#SAKNAS!
atresia-tracheoesophageal	1	5110(0.011432491139819366)	#SAKNAS!	2548 (0.033)
atricapilla	1	5110(0.011432491139819366)	2359 (0.034)	#SAKNAS!
atricapilla	1	5110(0.011432491139819366)	2359 (0.034)	#SAKNAS!
atricauda	1	5110(0.011432491139819366)	#SAKNAS!	2548 (0.033)
at-risk	1	5110(0.011432491139819366)	#SAKNAS!	#SAKNAS!
atrophic-erosive	1	5110(0.011432491139819366)	#SAKNAS!	2548 (0.033)
attack	1	5110(0.011432491139819366)	2359 (0.034)	#SAKNAS!
attack	1	5110(0.011432491139819366)	2359 (0.034)	#SAKNAS!
attainable	1	5110(0.011432491139819366)	#SAKNAS!	#SAKNAS!
attainment	1	3437(0.02286498227963873)	2359 (0.034)	2548 (0.033)
attempts	1	5110(0.011432491139819366)	#SAKNAS!	2548 (0.033)
attenders	1	5110(0.011432491139819366)	2359 (0.034)	#SAKNAS!
attenders	1	5110(0.011432491139819366)	2359 (0.034)	#SAKNAS!
attention-deficit	1	5110(0.011432491139819366)	#SAKNAS!	#SAKNAS!
attenuate	1	5110(0.011432491139819366)	#SAKNAS!	#SAKNAS!
attenuation	1	3437(0.02286498227963873)	2359 (0.034)	2548 (0.033)
attract	1	5110(0.011432491139819366)	#SAKNAS!	2548 (0.033)

attraction	1	5110(0.011432491139819366)	#SAKNAS!	#SAKNAS!
attractiveness	1	5110(0.011432491139819366)	#SAKNAS!	2548 (0.033)
attributable	1	5110(0.011432491139819366)	2359 (0.034)	#SAKNAS!
attributable	1	5110(0.011432491139819366)	2359 (0.034)	#SAKNAS!
atyaephyra	1	5110(0.011432491139819366)	#SAKNAS!	2548 (0.033)
audiology	1	5110(0.011432491139819366)	#SAKNAS!	2548 (0.033)
audiovestibular	1	5110(0.011432491139819366)	#SAKNAS!	2548 (0.033)
auf	1	5110(0.011432491139819366)	2359 (0.034)	#SAKNAS!
auf	1	5110(0.011432491139819366)	2359 (0.034)	#SAKNAS!
augment	1	5110(0.011432491139819366)	#SAKNAS!	2548 (0.033)
augmentation	1	896(0.14862238481765175)	2359 (0.034)	694 (0.2)
august	1	5110(0.011432491139819366)	2359 (0.034)	#SAKNAS!
august	1	5110(0.011432491139819366)	2359 (0.034)	#SAKNAS!
aurelia-aurita	1	5110(0.011432491139819366)	2359 (0.034)	#SAKNAS!
aurelia-aurita	1	5110(0.011432491139819366)	2359 (0.034)	#SAKNAS!
australian-born	1	5110(0.011432491139819366)	#SAKNAS!	2548 (0.033)
australians	1	5110(0.011432491139819366)	#SAKNAS!	2548 (0.033)
australostichopus	1	3437(0.02286498227963873)	2359 (0.034)	2548 (0.033)
austriacus	1	5110(0.011432491139819366)	2359 (0.034)	#SAKNAS!
austriacus	1	5110(0.011432491139819366)	2359 (0.034)	#SAKNAS!
austrian	1	5110(0.011432491139819366)	2359 (0.034)	#SAKNAS!
austrian	1	5110(0.011432491139819366)	2359 (0.034)	#SAKNAS!
autism	1	710(0.19435234937692925)	2359 (0.034)	361 (0.4)
autistic	1	2214(0.04572996455927746)	2359 (0.034)	2548 (0.033)
autoantibodies	1	5110(0.011432491139819366)	2359 (0.034)	#SAKNAS!
autoantibodies	1	5110(0.011432491139819366)	2359 (0.034)	#SAKNAS!
autoantigens	1	5110(0.011432491139819366)	#SAKNAS!	2548 (0.033)
autograft	1	5110(0.011432491139819366)	#SAKNAS!	2548 (0.033)
autoimmune	1	5110(0.011432491139819366)	#SAKNAS!	#SAKNAS!
autonomic	1	5110(0.011432491139819366)	2359 (0.034)	#SAKNAS!
autonomic	1	5110(0.011432491139819366)	2359 (0.034)	#SAKNAS!
autonomous	1	5110(0.011432491139819366)	#SAKNAS!	2548 (0.033)
autonomy	1	5110(0.011432491139819366)	#SAKNAS!	2548 (0.033)
autophagy	1	5110(0.011432491139819366)	#SAKNAS!	#SAKNAS!
autopsy	1	5110(0.011432491139819366)	#SAKNAS!	#SAKNAS!
autotomization	1	5110(0.011432491139819366)	#SAKNAS!	2548 (0.033)
autumn	1	5110(0.011432491139819366)	2359 (0.034)	#SAKNAS!
autumn	1	5110(0.011432491139819366)	2359 (0.034)	#SAKNAS!
avenae	1	5110(0.011432491139819366)	#SAKNAS!	2548 (0.033)
average	1	5110(0.011432491139819366)	#SAKNAS!	#SAKNAS!
aversive	1	5110(0.011432491139819366)	#SAKNAS!	2548 (0.033)
avidity	1	5110(0.011432491139819366)	2359 (0.034)	#SAKNAS!
avidity	1	5110(0.011432491139819366)	2359 (0.034)	#SAKNAS!

aviz	1	5110(0.011432491139819366)	#SAKNAS!	#SAKNAS!
avoiding	1	5110(0.011432491139819366)	2359 (0.034)	#SAKNAS!
avoiding	1	5110(0.011432491139819366)	2359 (0.034)	#SAKNAS!
avon	1	5110(0.011432491139819366)	2359 (0.034)	#SAKNAS!
avon	1	5110(0.011432491139819366)	2359 (0.034)	#SAKNAS!
awakening	1	5110(0.011432491139819366)	2359 (0.034)	#SAKNAS!
awakening	1	5110(0.011432491139819366)	2359 (0.034)	#SAKNAS!
away	1	3437(0.02286498227963873)	2359 (0.034)	#SAKNAS!
away-from-home	1	2693(0.0342974734194581)	2359 (0.034)	1668 (0.066)
axenic	1	5110(0.011432491139819366)	2359 (0.034)	#SAKNAS!
axenic	1	5110(0.011432491139819366)	2359 (0.034)	#SAKNAS!
axis-boosted	1	5110(0.011432491139819366)	#SAKNAS!	#SAKNAS!
ayder	1	5110(0.011432491139819366)	#SAKNAS!	#SAKNAS!
azadirachtin	1	5110(0.011432491139819366)	#SAKNAS!	2548 (0.033)
azithromycin	1	5110(0.011432491139819366)	#SAKNAS!	2548 (0.033)
a瘍a穩	1	5110(0.011432491139819366)	#SAKNAS!	#SAKNAS!
b1	1	5110(0.011432491139819366)	#SAKNAS!	#SAKNAS!
b12	1	5110(0.011432491139819366)	#SAKNAS!	#SAKNAS!
ba	1	5110(0.011432491139819366)	2359 (0.034)	#SAKNAS!
ba	1	5110(0.011432491139819366)	2359 (0.034)	#SAKNAS!
babassu	1	5110(0.011432491139819366)	2359 (0.034)	#SAKNAS!
babassu	1	5110(0.011432491139819366)	2359 (0.034)	#SAKNAS!
babies	1	5110(0.011432491139819366)	#SAKNAS!	#SAKNAS!
baboon	1	5110(0.011432491139819366)	#SAKNAS!	2548 (0.033)
baby-friendly	1	5110(0.011432491139819366)	#SAKNAS!	#SAKNAS!
baby-led	1	5110(0.011432491139819366)	#SAKNAS!	2548 (0.033)
background	1	3437(0.02286498227963873)	2359 (0.034)	#SAKNAS!
backgrounds	1	5110(0.011432491139819366)	#SAKNAS!	#SAKNAS!
baclofen	1	5110(0.011432491139819366)	2359 (0.034)	#SAKNAS!
baclofen	1	5110(0.011432491139819366)	2359 (0.034)	#SAKNAS!
bacterial-feeding	1	5110(0.011432491139819366)	2359 (0.034)	#SAKNAS!
bacterial-feeding	1	5110(0.011432491139819366)	2359 (0.034)	#SAKNAS!
bacteriological	1	5110(0.011432491139819366)	2359 (0.034)	#SAKNAS!
bacteriological	1	5110(0.011432491139819366)	2359 (0.034)	#SAKNAS!
bacterium	1	5110(0.011432491139819366)	#SAKNAS!	#SAKNAS!
bacterivory	1	5110(0.011432491139819366)	2359 (0.034)	#SAKNAS!
bacterivory	1	5110(0.011432491139819366)	2359 (0.034)	#SAKNAS!
baculovirus-challenged	1	5110(0.011432491139819366)	#SAKNAS!	2548 (0.033)
bagasse	1	5110(0.011432491139819366)	#SAKNAS!	#SAKNAS!
bahia	1	5110(0.011432491139819366)	#SAKNAS!	2548 (0.033)
bahrain	1	5110(0.011432491139819366)	#SAKNAS!	#SAKNAS!
baladi	1	5110(0.011432491139819366)	#SAKNAS!	2548 (0.033)
balancan	1	5110(0.011432491139819366)	2359 (0.034)	#SAKNAS!

balancan	1	5110(0.011432491139819366)	2359 (0.034)	#SAKNAS!
balb	1	5110(0.011432491139819366)	#SAKNAS!	2548 (0.033)
ballabio	1	5110(0.011432491139819366)	#SAKNAS!	2548 (0.033)
ballet	1	5110(0.011432491139819366)	2359 (0.034)	#SAKNAS!
ballet	1	5110(0.011432491139819366)	2359 (0.034)	#SAKNAS!
balochistan	1	5110(0.011432491139819366)	#SAKNAS!	2548 (0.033)
baltimore	1	5110(0.011432491139819366)	2359 (0.034)	#SAKNAS!
baltimore	1	5110(0.011432491139819366)	2359 (0.034)	#SAKNAS!
bamboo	1	5110(0.011432491139819366)	2359 (0.034)	#SAKNAS!
bamboo	1	5110(0.011432491139819366)	2359 (0.034)	#SAKNAS!
bamboo-leaf-based	1	5110(0.011432491139819366)	#SAKNAS!	2548 (0.033)
band-c	1	5110(0.011432491139819366)	2359 (0.034)	#SAKNAS!
band-c	1	5110(0.011432491139819366)	2359 (0.034)	#SAKNAS!
bandings	1	5110(0.011432491139819366)	#SAKNAS!	2548 (0.033)
bands	1	3437(0.02286498227963873)	2359 (0.034)	2548 (0.033)
banks	1	5110(0.011432491139819366)	2359 (0.034)	#SAKNAS!
banks	1	5110(0.011432491139819366)	2359 (0.034)	#SAKNAS!
barbadensis	1	5110(0.011432491139819366)	2359 (0.034)	#SAKNAS!
barbadensis	1	5110(0.011432491139819366)	2359 (0.034)	#SAKNAS!
barbara	1	5110(0.011432491139819366)	#SAKNAS!	#SAKNAS!
barbatus	1	5110(0.011432491139819366)	2359 (0.034)	#SAKNAS!
barbatus	1	5110(0.011432491139819366)	2359 (0.034)	#SAKNAS!
barcelona	1	2693(0.0342974734194581)	2359 (0.034)	2548 (0.033)
barcoding	1	5110(0.011432491139819366)	#SAKNAS!	2548 (0.033)
bariloche	1	5110(0.011432491139819366)	#SAKNAS!	2548 (0.033)
barn	1	5110(0.011432491139819366)	#SAKNAS!	#SAKNAS!
barrel	1	5110(0.011432491139819366)	#SAKNAS!	2548 (0.033)
bar-retained	1	5110(0.011432491139819366)	#SAKNAS!	#SAKNAS!
barretts-esophagus	1	5110(0.011432491139819366)	2359 (0.034)	#SAKNAS!
barretts-esophagus	1	5110(0.011432491139819366)	2359 (0.034)	#SAKNAS!
barrier	1	3437(0.02286498227963873)	2359 (0.034)	#SAKNAS!
bars	1	5110(0.011432491139819366)	#SAKNAS!	#SAKNAS!
based-sample	1	5110(0.011432491139819366)	#SAKNAS!	#SAKNAS!
bases	1	2693(0.0342974734194581)	2359 (0.034)	2548 (0.033)
basilar	1	5110(0.011432491139819366)	#SAKNAS!	2548 (0.033)
bat	1	2214(0.04572996455927746)	2359 (0.034)	1233 (0.1)
batatas	1	5110(0.011432491139819366)	2359 (0.034)	#SAKNAS!
batatas	1	5110(0.011432491139819366)	2359 (0.034)	#SAKNAS!
bats	1	5110(0.011432491139819366)	#SAKNAS!	#SAKNAS!
bayesian	1	5110(0.011432491139819366)	#SAKNAS!	#SAKNAS!
bayley	1	5110(0.011432491139819366)	2359 (0.034)	#SAKNAS!
bayley	1	5110(0.011432491139819366)	2359 (0.034)	#SAKNAS!
bc	1	5110(0.011432491139819366)	2359 (0.034)	#SAKNAS!

bc	1	5110(0.011432491139819366)	2359 (0.034)	#SAKNAS!
bce-200	1	5110(0.011432491139819366)	#SAKNAS!	2548 (0.033)
bde-47	1	5110(0.011432491139819366)	#SAKNAS!	#SAKNAS!
bdelloid	1	5110(0.011432491139819366)	#SAKNAS!	#SAKNAS!
beak	1	5110(0.011432491139819366)	#SAKNAS!	2548 (0.033)
beam	1	5110(0.011432491139819366)	2359 (0.034)	#SAKNAS!
beam	1	5110(0.011432491139819366)	2359 (0.034)	#SAKNAS!
beam-conditioning	1	5110(0.011432491139819366)	2359 (0.034)	#SAKNAS!
beam-conditioning	1	5110(0.011432491139819366)	2359 (0.034)	#SAKNAS!
bean	1	5110(0.011432491139819366)	#SAKNAS!	2548 (0.033)
bean-based	1	5110(0.011432491139819366)	#SAKNAS!	#SAKNAS!
bears	1	3437(0.02286498227963873)	2359 (0.034)	2548 (0.033)
beaver	1	5110(0.011432491139819366)	#SAKNAS!	2548 (0.033)
became	1	5110(0.011432491139819366)	#SAKNAS!	#SAKNAS!
bedding	1	5110(0.011432491139819366)	2359 (0.034)	#SAKNAS!
bedding	1	5110(0.011432491139819366)	2359 (0.034)	#SAKNAS!
beet	1	5110(0.011432491139819366)	2359 (0.034)	#SAKNAS!
beet	1	5110(0.011432491139819366)	2359 (0.034)	#SAKNAS!
beetles	1	2693(0.0342974734194581)	2359 (0.034)	#SAKNAS!
beetroot	1	5110(0.011432491139819366)	#SAKNAS!	2548 (0.033)
beginnings	1	5110(0.011432491139819366)	2359 (0.034)	#SAKNAS!
beginnings	1	5110(0.011432491139819366)	2359 (0.034)	#SAKNAS!
begins	1	5110(0.011432491139819366)	#SAKNAS!	2548 (0.033)
behaviar	1	5110(0.011432491139819366)	2359 (0.034)	#SAKNAS!
behaviar	1	5110(0.011432491139819366)	2359 (0.034)	#SAKNAS!
behavior-change	1	5110(0.011432491139819366)	2359 (0.034)	#SAKNAS!
behavior-change	1	5110(0.011432491139819366)	2359 (0.034)	#SAKNAS!
behcet	1	5110(0.011432491139819366)	2359 (0.034)	#SAKNAS!
behcet	1	5110(0.011432491139819366)	2359 (0.034)	#SAKNAS!
beijing	1	5110(0.011432491139819366)	#SAKNAS!	#SAKNAS!
belarusian	1	5110(0.011432491139819366)	#SAKNAS!	#SAKNAS!
belgrade	1	5110(0.011432491139819366)	2359 (0.034)	#SAKNAS!
belgrade	1	5110(0.011432491139819366)	2359 (0.034)	#SAKNAS!
belief	1	5110(0.011432491139819366)	#SAKNAS!	#SAKNAS!
bell	1	5110(0.011432491139819366)	#SAKNAS!	2548 (0.033)
bellydent	1	5110(0.011432491139819366)	#SAKNAS!	#SAKNAS!
belowground	1	5110(0.011432491139819366)	#SAKNAS!	2548 (0.033)
belzebuth	1	5110(0.011432491139819366)	2359 (0.034)	#SAKNAS!
belzebuth	1	5110(0.011432491139819366)	2359 (0.034)	#SAKNAS!
bemisia	1	2693(0.0342974734194581)	2359 (0.034)	1668 (0.066)
bench	1	5110(0.011432491139819366)	#SAKNAS!	2548 (0.033)
benchmarking	1	5110(0.011432491139819366)	#SAKNAS!	#SAKNAS!
benchmarks	1	5110(0.011432491139819366)	#SAKNAS!	#SAKNAS!

bend	1	5110(0.011432491139819366)	2359 (0.034)	#SAKNAS!
bend	1	5110(0.011432491139819366)	2359 (0.034)	#SAKNAS!
beneficiaries	1	5110(0.011432491139819366)	#SAKNAS!	#SAKNAS!
benefit-risk	1	5110(0.011432491139819366)	2359 (0.034)	#SAKNAS!
benefit-risk	1	5110(0.011432491139819366)	2359 (0.034)	#SAKNAS!
benghazi	1	5110(0.011432491139819366)	#SAKNAS!	#SAKNAS!
benguela	1	5110(0.011432491139819366)	#SAKNAS!	2548 (0.033)
bennett	1	5110(0.011432491139819366)	#SAKNAS!	#SAKNAS!
benzoate	1	5110(0.011432491139819366)	#SAKNAS!	2548 (0.033)
bergamo	1	5110(0.011432491139819366)	2359 (0.034)	#SAKNAS!
bergamo	1	5110(0.011432491139819366)	2359 (0.034)	#SAKNAS!
bergenia	1	5110(0.011432491139819366)	2359 (0.034)	#SAKNAS!
bergenia	1	5110(0.011432491139819366)	2359 (0.034)	#SAKNAS!
beringei	1	5110(0.011432491139819366)	#SAKNAS!	2548 (0.033)
bermuda	1	5110(0.011432491139819366)	#SAKNAS!	2548 (0.033)
bernicla	1	5110(0.011432491139819366)	2359 (0.034)	#SAKNAS!
bernicla	1	5110(0.011432491139819366)	2359 (0.034)	#SAKNAS!
berries	1	5110(0.011432491139819366)	2359 (0.034)	#SAKNAS!
berries	1	5110(0.011432491139819366)	2359 (0.034)	#SAKNAS!
bet	1	5110(0.011432491139819366)	#SAKNAS!	2548 (0.033)
beta-3	1	5110(0.011432491139819366)	#SAKNAS!	2548 (0.033)
beta-band	1	5110(0.011432491139819366)	#SAKNAS!	#SAKNAS!
beta-blockers	1	5110(0.011432491139819366)	#SAKNAS!	2548 (0.033)
betel	1	5110(0.011432491139819366)	2359 (0.034)	#SAKNAS!
betel	1	5110(0.011432491139819366)	2359 (0.034)	#SAKNAS!
better-quality	1	5110(0.011432491139819366)	#SAKNAS!	2548 (0.033)
betweenaphis	1	5110(0.011432491139819366)	#SAKNAS!	2548 (0.033)
beverage	1	743(0.18291985823710985)	2359 (0.034)	526 (0.26)
beyond-brand	1	5110(0.011432491139819366)	2359 (0.034)	#SAKNAS!
beyond-brand	1	5110(0.011432491139819366)	2359 (0.034)	#SAKNAS!
bezoar	1	5110(0.011432491139819366)	#SAKNAS!	#SAKNAS!
bfrs	1	5110(0.011432491139819366)	#SAKNAS!	#SAKNAS!
bhopal	1	5110(0.011432491139819366)	#SAKNAS!	2548 (0.033)
bia-derived	1	5110(0.011432491139819366)	#SAKNAS!	#SAKNAS!
bialystok	1	5110(0.011432491139819366)	#SAKNAS!	#SAKNAS!
biases	1	3437(0.02286498227963873)	2359 (0.034)	2548 (0.033)
bicornis	1	5110(0.011432491139819366)	2359 (0.034)	#SAKNAS!
bicornis	1	5110(0.011432491139819366)	2359 (0.034)	#SAKNAS!
bictegravir	1	5110(0.011432491139819366)	#SAKNAS!	2548 (0.033)
bidirectional	1	1678(0.0685949468389162)	2359 (0.034)	1668 (0.066)
bifidobacteriaceae	1	5110(0.011432491139819366)	2359 (0.034)	#SAKNAS!
bifidobacteriaceae	1	5110(0.011432491139819366)	2359 (0.034)	#SAKNAS!
bifidobacterium	1	5110(0.011432491139819366)	#SAKNAS!	2548 (0.033)

bighorn	1	5110(0.011432491139819366)	2359 (0.034)	#SAKNAS!
bighorn	1	5110(0.011432491139819366)	2359 (0.034)	#SAKNAS!
bile	1	5110(0.011432491139819366)	#SAKNAS!	2548 (0.033)
bilineatus	1	5110(0.011432491139819366)	2359 (0.034)	#SAKNAS!
bilineatus	1	5110(0.011432491139819366)	2359 (0.034)	#SAKNAS!
biliopancreatic	1	2693(0.0342974734194581)	2359 (0.034)	#SAKNAS!
bin	1	5110(0.011432491139819366)	#SAKNAS!	2548 (0.033)
binds	1	5110(0.011432491139819366)	2359 (0.034)	#SAKNAS!
binds	1	5110(0.011432491139819366)	2359 (0.034)	#SAKNAS!
binge-like	1	5110(0.011432491139819366)	2359 (0.034)	#SAKNAS!
binge-like	1	5110(0.011432491139819366)	2359 (0.034)	#SAKNAS!
bioaccessibility	1	5110(0.011432491139819366)	#SAKNAS!	#SAKNAS!
bioactive	1	3437(0.02286498227963873)	2359 (0.034)	2548 (0.033)
bioactivity	1	3437(0.02286498227963873)	2359 (0.034)	#SAKNAS!
bioarchaeological	1	5110(0.011432491139819366)	#SAKNAS!	#SAKNAS!
bioarchaeology	1	5110(0.011432491139819366)	#SAKNAS!	2548 (0.033)
bioassay	1	5110(0.011432491139819366)	2359 (0.034)	#SAKNAS!
bioassay	1	5110(0.011432491139819366)	2359 (0.034)	#SAKNAS!
bioassays	1	5110(0.011432491139819366)	2359 (0.034)	#SAKNAS!
bioassays	1	5110(0.011432491139819366)	2359 (0.034)	#SAKNAS!
biochemistry	1	3437(0.02286498227963873)	2359 (0.034)	2548 (0.033)
biodegradable	1	3437(0.02286498227963873)	2359 (0.034)	2548 (0.033)
biodeposits	1	5110(0.011432491139819366)	#SAKNAS!	2548 (0.033)
biodiversity	1	5110(0.011432491139819366)	#SAKNAS!	#SAKNAS!
bioenergetic	1	5110(0.011432491139819366)	#SAKNAS!	#SAKNAS!
bioenterics	1	5110(0.011432491139819366)	2359 (0.034)	#SAKNAS!
bioenterics	1	5110(0.011432491139819366)	2359 (0.034)	#SAKNAS!
biofilm-demineralization	1	5110(0.011432491139819366)	#SAKNAS!	2548 (0.033)
biofilm-insights	1	5110(0.011432491139819366)	#SAKNAS!	2548 (0.033)
biofilms	1	2214(0.04572996455927746)	2359 (0.034)	1233 (0.1)
bio-functional	1	5110(0.011432491139819366)	#SAKNAS!	#SAKNAS!
biogeochemical	1	5110(0.011432491139819366)	#SAKNAS!	2548 (0.033)
biogeographical	1	5110(0.011432491139819366)	2359 (0.034)	#SAKNAS!
biogeographical	1	5110(0.011432491139819366)	2359 (0.034)	#SAKNAS!
biologic	1	3437(0.02286498227963873)	2359 (0.034)	#SAKNAS!
biologically	1	5110(0.011432491139819366)	2359 (0.034)	#SAKNAS!
biologically	1	5110(0.011432491139819366)	2359 (0.034)	#SAKNAS!
bio-markers	1	5110(0.011432491139819366)	2359 (0.034)	#SAKNAS!
bio-markers	1	5110(0.011432491139819366)	2359 (0.034)	#SAKNAS!
biomarkers-soluble	1	5110(0.011432491139819366)	#SAKNAS!	2548 (0.033)
biomass	1	3437(0.02286498227963873)	2359 (0.034)	2548 (0.033)
biomechanical	1	5110(0.011432491139819366)	#SAKNAS!	2548 (0.033)
biomechanics	1	2693(0.0342974734194581)	2359 (0.034)	1668 (0.066)

biometrics	1	5110(0.011432491139819366)	2359 (0.034)	#SAKNAS!
biometrics	1	5110(0.011432491139819366)	2359 (0.034)	#SAKNAS!
biomphalaria-glabrata	1	5110(0.011432491139819366)	2359 (0.034)	#SAKNAS!
biomphalaria-glabrata	1	5110(0.011432491139819366)	2359 (0.034)	#SAKNAS!
bionic	1	5110(0.011432491139819366)	2359 (0.034)	#SAKNAS!
bionic	1	5110(0.011432491139819366)	2359 (0.034)	#SAKNAS!
bioqualim	1	5110(0.011432491139819366)	#SAKNAS!	#SAKNAS!
biosignals	1	5110(0.011432491139819366)	#SAKNAS!	#SAKNAS!
biosocial	1	5110(0.011432491139819366)	#SAKNAS!	#SAKNAS!
biosynthesis	1	5110(0.011432491139819366)	#SAKNAS!	#SAKNAS!
biosynthetic	1	5110(0.011432491139819366)	#SAKNAS!	#SAKNAS!
biotransformation	1	5110(0.011432491139819366)	#SAKNAS!	2548 (0.033)
biotrophic	1	5110(0.011432491139819366)	#SAKNAS!	#SAKNAS!
biracial	1	5110(0.011432491139819366)	2359 (0.034)	#SAKNAS!
biracial	1	5110(0.011432491139819366)	2359 (0.034)	#SAKNAS!
bird	1	5110(0.011432491139819366)	2359 (0.034)	#SAKNAS!
bird	1	5110(0.011432491139819366)	2359 (0.034)	#SAKNAS!
birth-related	1	5110(0.011432491139819366)	#SAKNAS!	#SAKNAS!
birth-to-ten	1	5110(0.011432491139819366)	2359 (0.034)	#SAKNAS!
birth-to-ten	1	5110(0.011432491139819366)	2359 (0.034)	#SAKNAS!
birthweight	1	5110(0.011432491139819366)	2359 (0.034)	#SAKNAS!
birthweight	1	5110(0.011432491139819366)	2359 (0.034)	#SAKNAS!
biscuit	1	5110(0.011432491139819366)	#SAKNAS!	#SAKNAS!
bisphosphate	1	5110(0.011432491139819366)	2359 (0.034)	#SAKNAS!
bisphosphate	1	5110(0.011432491139819366)	2359 (0.034)	#SAKNAS!
bisphosphonate-related	1	5110(0.011432491139819366)	2359 (0.034)	#SAKNAS!
bisphosphonate-related	1	5110(0.011432491139819366)	2359 (0.034)	#SAKNAS!
bisulcus	1	5110(0.011432491139819366)	2359 (0.034)	#SAKNAS!
bisulcus	1	5110(0.011432491139819366)	2359 (0.034)	#SAKNAS!
bite	1	1678(0.0685949468389162)	2359 (0.034)	2548 (0.033)
bite-force	1	5110(0.011432491139819366)	#SAKNAS!	2548 (0.033)
biting	1	3437(0.02286498227963873)	2359 (0.034)	#SAKNAS!
bits	1	5110(0.011432491139819366)	#SAKNAS!	2548 (0.033)
bitter-sensitive	1	5110(0.011432491139819366)	#SAKNAS!	2548 (0.033)
bitwin	1	5110(0.011432491139819366)	#SAKNAS!	#SAKNAS!
bivalve	1	2693(0.0342974734194581)	2359 (0.034)	2548 (0.033)
bivalves	1	5110(0.011432491139819366)	#SAKNAS!	#SAKNAS!
biweekly	1	5110(0.011432491139819366)	#SAKNAS!	2548 (0.033)
black-backed	1	5110(0.011432491139819366)	2359 (0.034)	#SAKNAS!
black-backed	1	5110(0.011432491139819366)	2359 (0.034)	#SAKNAS!
blackbird	1	5110(0.011432491139819366)	2359 (0.034)	#SAKNAS!
blackbird	1	5110(0.011432491139819366)	2359 (0.034)	#SAKNAS!
blackcaps	1	5110(0.011432491139819366)	2359 (0.034)	#SAKNAS!

blackcaps	1	5110(0.011432491139819366)	2359 (0.034)	#SAKNAS!
black-children	1	5110(0.011432491139819366)	2359 (0.034)	#SAKNAS!
black-children	1	5110(0.011432491139819366)	2359 (0.034)	#SAKNAS!
blatta	1	5110(0.011432491139819366)	#SAKNAS!	#SAKNAS!
blattella	1	5110(0.011432491139819366)	2359 (0.034)	#SAKNAS!
blattella	1	5110(0.011432491139819366)	2359 (0.034)	#SAKNAS!
blattodea	1	5110(0.011432491139819366)	#SAKNAS!	#SAKNAS!
blazei	1	5110(0.011432491139819366)	#SAKNAS!	#SAKNAS!
blend	1	5110(0.011432491139819366)	#SAKNAS!	#SAKNAS!
blind	1	2693(0.0342974734194581)	2359 (0.034)	2548 (0.033)
blindness	1	5110(0.011432491139819366)	#SAKNAS!	2548 (0.033)
bloating	1	5110(0.011432491139819366)	#SAKNAS!	#SAKNAS!
bloch	1	5110(0.011432491139819366)	2359 (0.034)	#SAKNAS!
bloch	1	5110(0.011432491139819366)	2359 (0.034)	#SAKNAS!
block	1	5110(0.011432491139819366)	2359 (0.034)	#SAKNAS!
block	1	5110(0.011432491139819366)	2359 (0.034)	#SAKNAS!
bloodborne	1	5110(0.011432491139819366)	2359 (0.034)	#SAKNAS!
bloodborne	1	5110(0.011432491139819366)	2359 (0.034)	#SAKNAS!
bloodstream	1	5110(0.011432491139819366)	2359 (0.034)	#SAKNAS!
bloodstream	1	5110(0.011432491139819366)	2359 (0.034)	#SAKNAS!
blooming	1	5110(0.011432491139819366)	#SAKNAS!	#SAKNAS!
blowfly	1	5110(0.011432491139819366)	2359 (0.034)	#SAKNAS!
blowfly	1	5110(0.011432491139819366)	2359 (0.034)	#SAKNAS!
bluegill	1	5110(0.011432491139819366)	2359 (0.034)	#SAKNAS!
bluegill	1	5110(0.011432491139819366)	2359 (0.034)	#SAKNAS!
bmal1	1	5110(0.011432491139819366)	#SAKNAS!	#SAKNAS!
bmi-1	1	3437(0.02286498227963873)	2359 (0.034)	#SAKNAS!
bmi-modified	1	5110(0.011432491139819366)	#SAKNAS!	#SAKNAS!
bmorco	1	5110(0.011432491139819366)	#SAKNAS!	2548 (0.033)
boa	1	5110(0.011432491139819366)	#SAKNAS!	#SAKNAS!
board	1	5110(0.011432491139819366)	#SAKNAS!	2548 (0.033)
boarding	1	5110(0.011432491139819366)	2359 (0.034)	#SAKNAS!
boarding	1	5110(0.011432491139819366)	2359 (0.034)	#SAKNAS!
boars	1	5110(0.011432491139819366)	2359 (0.034)	#SAKNAS!
boars	1	5110(0.011432491139819366)	2359 (0.034)	#SAKNAS!
boccardia	1	5110(0.011432491139819366)	#SAKNAS!	2548 (0.033)
body-temperature	1	5110(0.011432491139819366)	2359 (0.034)	#SAKNAS!
body-temperature	1	5110(0.011432491139819366)	2359 (0.034)	#SAKNAS!
boer	1	5110(0.011432491139819366)	#SAKNAS!	#SAKNAS!
bog	1	5110(0.011432491139819366)	#SAKNAS!	#SAKNAS!
boisd	1	5110(0.011432491139819366)	#SAKNAS!	#SAKNAS!
bolbometopon	1	5110(0.011432491139819366)	#SAKNAS!	2548 (0.033)
bolster	1	5110(0.011432491139819366)	2359 (0.034)	#SAKNAS!

bolster	1	5110(0.011432491139819366)	2359 (0.034)	#SAKNAS!
bombus	1	2214(0.04572996455927746)	2359 (0.034)	#SAKNAS!
bombyx	1	2693(0.0342974734194581)	2359 (0.034)	2548 (0.033)
bone-density	1	5110(0.011432491139819366)	2359 (0.034)	#SAKNAS!
bone-density	1	5110(0.011432491139819366)	2359 (0.034)	#SAKNAS!
bone-mineral	1	5110(0.011432491139819366)	2359 (0.034)	#SAKNAS!
bone-mineral	1	5110(0.011432491139819366)	2359 (0.034)	#SAKNAS!
bonnaterre	1	5110(0.011432491139819366)	#SAKNAS!	#SAKNAS!
bonobo	1	5110(0.011432491139819366)	#SAKNAS!	2548 (0.033)
book	1	5110(0.011432491139819366)	#SAKNAS!	#SAKNAS!
booklet	1	5110(0.011432491139819366)	2359 (0.034)	#SAKNAS!
booklet	1	5110(0.011432491139819366)	2359 (0.034)	#SAKNAS!
boone	1	5110(0.011432491139819366)	#SAKNAS!	2548 (0.033)
boosting	1	5110(0.011432491139819366)	2359 (0.034)	#SAKNAS!
boosting	1	5110(0.011432491139819366)	2359 (0.034)	#SAKNAS!
borderline	1	5110(0.011432491139819366)	#SAKNAS!	2548 (0.033)
boreioglycaspis	1	5110(0.011432491139819366)	2359 (0.034)	#SAKNAS!
boreioglycaspis	1	5110(0.011432491139819366)	2359 (0.034)	#SAKNAS!
bornean	1	5110(0.011432491139819366)	#SAKNAS!	2548 (0.033)
boscii	1	5110(0.011432491139819366)	2359 (0.034)	#SAKNAS!
boscii	1	5110(0.011432491139819366)	2359 (0.034)	#SAKNAS!
bosnia	1	5110(0.011432491139819366)	2359 (0.034)	#SAKNAS!
bosnia	1	5110(0.011432491139819366)	2359 (0.034)	#SAKNAS!
bossou	1	5110(0.011432491139819366)	2359 (0.034)	#SAKNAS!
bossou	1	5110(0.011432491139819366)	2359 (0.034)	#SAKNAS!
boston	1	5110(0.011432491139819366)	2359 (0.034)	#SAKNAS!
boston	1	5110(0.011432491139819366)	2359 (0.034)	#SAKNAS!
bostwick	1	5110(0.011432491139819366)	#SAKNAS!	2548 (0.033)
botanical	1	5110(0.011432491139819366)	#SAKNAS!	2548 (0.033)
botswana	1	5110(0.011432491139819366)	2359 (0.034)	#SAKNAS!
botswana	1	5110(0.011432491139819366)	2359 (0.034)	#SAKNAS!
bottled	1	5110(0.011432491139819366)	#SAKNAS!	2548 (0.033)
bottle-feeding	1	5110(0.011432491139819366)	2359 (0.034)	#SAKNAS!
bottle-feeding	1	5110(0.011432491139819366)	2359 (0.034)	#SAKNAS!
bottom-up	1	5110(0.011432491139819366)	#SAKNAS!	#SAKNAS!
bougie	1	5110(0.011432491139819366)	#SAKNAS!	#SAKNAS!
boulardii	1	5110(0.011432491139819366)	#SAKNAS!	#SAKNAS!
boundary	1	5110(0.011432491139819366)	2359 (0.034)	#SAKNAS!
boundary	1	5110(0.011432491139819366)	2359 (0.034)	#SAKNAS!
bouquet	1	5110(0.011432491139819366)	#SAKNAS!	#SAKNAS!
bovine	1	3437(0.02286498227963873)	2359 (0.034)	2548 (0.033)
bowel	1	1485(0.08002743797873557)	2359 (0.034)	1668 (0.066)
box	1	2693(0.0342974734194581)	2359 (0.034)	2548 (0.033)

bp	1	3437(0.02286498227963873)	2359 (0.034)	2548 (0.033)
bpifa1	1	5110(0.011432491139819366)	#SAKNAS!	2548 (0.033)
braconidae	1	3437(0.02286498227963873)	2359 (0.034)	2548 (0.033)
brains	1	5110(0.011432491139819366)	2359 (0.034)	#SAKNAS!
brains	1	5110(0.011432491139819366)	2359 (0.034)	#SAKNAS!
brain-stem	1	5110(0.011432491139819366)	2359 (0.034)	#SAKNAS!
brain-stem	1	5110(0.011432491139819366)	2359 (0.034)	#SAKNAS!
brain-tumor	1	5110(0.011432491139819366)	2359 (0.034)	#SAKNAS!
brain-tumor	1	5110(0.011432491139819366)	2359 (0.034)	#SAKNAS!
bran	1	5110(0.011432491139819366)	#SAKNAS!	#SAKNAS!
brandt	1	5110(0.011432491139819366)	#SAKNAS!	#SAKNAS!
branta	1	5110(0.011432491139819366)	2359 (0.034)	#SAKNAS!
branta	1	5110(0.011432491139819366)	2359 (0.034)	#SAKNAS!
brasila	1	5110(0.011432491139819366)	2359 (0.034)	#SAKNAS!
brasila	1	5110(0.011432491139819366)	2359 (0.034)	#SAKNAS!
brasileira	1	5110(0.011432491139819366)	#SAKNAS!	#SAKNAS!
brassica	1	5110(0.011432491139819366)	#SAKNAS!	2548 (0.033)
brazilian-portuguese	1	5110(0.011432491139819366)	#SAKNAS!	#SAKNAS!
braziliansa	1	5110(0.011432491139819366)	#SAKNAS!	2548 (0.033)
brazzaville	1	5110(0.011432491139819366)	2359 (0.034)	#SAKNAS!
brazzaville	1	5110(0.011432491139819366)	2359 (0.034)	#SAKNAS!
brea	1	5110(0.011432491139819366)	#SAKNAS!	2548 (0.033)
breadth	1	5110(0.011432491139819366)	#SAKNAS!	2548 (0.033)
break	1	5110(0.011432491139819366)	#SAKNAS!	#SAKNAS!
breakfasts	1	5110(0.011432491139819366)	2359 (0.034)	#SAKNAS!
breakfasts	1	5110(0.011432491139819366)	2359 (0.034)	#SAKNAS!
breakfast-three	1	5110(0.011432491139819366)	#SAKNAS!	2548 (0.033)
breaks	1	5110(0.011432491139819366)	2359 (0.034)	#SAKNAS!
breaks	1	5110(0.011432491139819366)	2359 (0.034)	#SAKNAS!
breastfeeding-friendly	1	5110(0.011432491139819366)	2359 (0.034)	#SAKNAS!
breastfeeding-friendly	1	5110(0.011432491139819366)	2359 (0.034)	#SAKNAS!
breastmilk	1	5110(0.011432491139819366)	#SAKNAS!	2548 (0.033)
breath	1	5110(0.011432491139819366)	2359 (0.034)	#SAKNAS!
breath	1	5110(0.011432491139819366)	2359 (0.034)	#SAKNAS!
breathing-related	1	5110(0.011432491139819366)	#SAKNAS!	2548 (0.033)
breeds	1	3437(0.02286498227963873)	2359 (0.034)	2548 (0.033)
brent	1	5110(0.011432491139819366)	2359 (0.034)	#SAKNAS!
brent	1	5110(0.011432491139819366)	2359 (0.034)	#SAKNAS!
breviceps	1	5110(0.011432491139819366)	#SAKNAS!	2548 (0.033)
brevis	1	5110(0.011432491139819366)	2359 (0.034)	#SAKNAS!
brevis	1	5110(0.011432491139819366)	2359 (0.034)	#SAKNAS!
brick	1	5110(0.011432491139819366)	#SAKNAS!	2548 (0.033)
bridging	1	3437(0.02286498227963873)	2359 (0.034)	#SAKNAS!

bridle	1	5110(0.011432491139819366)	#SAKNAS!	#SAKNAS!
brief	1	1894(0.05716245569909683)	2359 (0.034)	2548 (0.033)
brief-motivational	1	5110(0.011432491139819366)	#SAKNAS!	#SAKNAS!
bright	1	5110(0.011432491139819366)	#SAKNAS!	#SAKNAS!
bring	1	5110(0.011432491139819366)	2359 (0.034)	#SAKNAS!
bring	1	5110(0.011432491139819366)	2359 (0.034)	#SAKNAS!
broad	1	5110(0.011432491139819366)	2359 (0.034)	#SAKNAS!
broad	1	5110(0.011432491139819366)	2359 (0.034)	#SAKNAS!
broadbent	1	5110(0.011432491139819366)	2359 (0.034)	#SAKNAS!
broadbent	1	5110(0.011432491139819366)	2359 (0.034)	#SAKNAS!
broadly	1	5110(0.011432491139819366)	#SAKNAS!	2548 (0.033)
broilers	1	5110(0.011432491139819366)	2359 (0.034)	#SAKNAS!
broilers	1	5110(0.011432491139819366)	2359 (0.034)	#SAKNAS!
broken	1	3437(0.02286498227963873)	2359 (0.034)	2548 (0.033)
brood	1	5110(0.011432491139819366)	2359 (0.034)	#SAKNAS!
brood	1	5110(0.011432491139819366)	2359 (0.034)	#SAKNAS!
broodnest	1	5110(0.011432491139819366)	2359 (0.034)	#SAKNAS!
broodnest	1	5110(0.011432491139819366)	2359 (0.034)	#SAKNAS!
brothers	1	5110(0.011432491139819366)	#SAKNAS!	#SAKNAS!
browse	1	5110(0.011432491139819366)	#SAKNAS!	#SAKNAS!
bruchid	1	5110(0.011432491139819366)	2359 (0.034)	#SAKNAS!
bruchid	1	5110(0.011432491139819366)	2359 (0.034)	#SAKNAS!
bruchidius	1	5110(0.011432491139819366)	2359 (0.034)	#SAKNAS!
bruchidius	1	5110(0.011432491139819366)	2359 (0.034)	#SAKNAS!
brush	1	3437(0.02286498227963873)	2359 (0.034)	2548 (0.033)
brushes	1	5110(0.011432491139819366)	#SAKNAS!	2548 (0.033)
brushtail	1	5110(0.011432491139819366)	2359 (0.034)	#SAKNAS!
brushtail	1	5110(0.011432491139819366)	2359 (0.034)	#SAKNAS!
brycon	1	5110(0.011432491139819366)	#SAKNAS!	2548 (0.033)
bsg	1	5110(0.011432491139819366)	#SAKNAS!	#SAKNAS!
bspghan	1	5110(0.011432491139819366)	#SAKNAS!	#SAKNAS!
bt	1	5110(0.011432491139819366)	#SAKNAS!	2548 (0.033)
btxa	1	5110(0.011432491139819366)	#SAKNAS!	2548 (0.033)
buccal	1	2693(0.0342974734194581)	2359 (0.034)	1668 (0.066)
buccinum-undatum	1	5110(0.011432491139819366)	2359 (0.034)	#SAKNAS!
buccinum-undatum	1	5110(0.011432491139819366)	2359 (0.034)	#SAKNAS!
buckthorn	1	3437(0.02286498227963873)	2359 (0.034)	#SAKNAS!
bud	1	5110(0.011432491139819366)	2359 (0.034)	#SAKNAS!
bud	1	5110(0.011432491139819366)	2359 (0.034)	#SAKNAS!
budesonide	1	5110(0.011432491139819366)	#SAKNAS!	2548 (0.033)
buds	1	5110(0.011432491139819366)	2359 (0.034)	#SAKNAS!
buds	1	5110(0.011432491139819366)	2359 (0.034)	#SAKNAS!
buffer	1	5110(0.011432491139819366)	#SAKNAS!	2548 (0.033)

buffet	1	5110(0.011432491139819366)	#SAKNAS!	2548 (0.033)
bufonids	1	5110(0.011432491139819366)	#SAKNAS!	#SAKNAS!
bug	1	5110(0.011432491139819366)	#SAKNAS!	#SAKNAS!
building	1	2214(0.04572996455927746)	2359 (0.034)	1233 (0.1)
builds	1	5110(0.011432491139819366)	2359 (0.034)	#SAKNAS!
builds	1	5110(0.011432491139819366)	2359 (0.034)	#SAKNAS!
bujumbura	1	5110(0.011432491139819366)	2359 (0.034)	#SAKNAS!
bujumbura	1	5110(0.011432491139819366)	2359 (0.034)	#SAKNAS!
bulb	1	5110(0.011432491139819366)	#SAKNAS!	2548 (0.033)
bulgaria	1	5110(0.011432491139819366)	2359 (0.034)	#SAKNAS!
bulgaria	1	5110(0.011432491139819366)	2359 (0.034)	#SAKNAS!
bulhosa	1	5110(0.011432491139819366)	2359 (0.034)	#SAKNAS!
bulhosa	1	5110(0.011432491139819366)	2359 (0.034)	#SAKNAS!
bulimia-nervosa	1	5110(0.011432491139819366)	2359 (0.034)	#SAKNAS!
bulimia-nervosa	1	5110(0.011432491139819366)	2359 (0.034)	#SAKNAS!
bulk	1	5110(0.011432491139819366)	2359 (0.034)	#SAKNAS!
bulk	1	5110(0.011432491139819366)	2359 (0.034)	#SAKNAS!
bull	1	5110(0.011432491139819366)	#SAKNAS!	2548 (0.033)
bullet	1	5110(0.011432491139819366)	#SAKNAS!	2548 (0.033)
bumble	1	5110(0.011432491139819366)	2359 (0.034)	#SAKNAS!
bumble	1	5110(0.011432491139819366)	2359 (0.034)	#SAKNAS!
bumblebees	1	3437(0.02286498227963873)	2359 (0.034)	2548 (0.033)
bumphead	1	5110(0.011432491139819366)	#SAKNAS!	2548 (0.033)
bundled	1	5110(0.011432491139819366)	#SAKNAS!	#SAKNAS!
burbank	1	5110(0.011432491139819366)	#SAKNAS!	2548 (0.033)
buren	1	5110(0.011432491139819366)	2359 (0.034)	#SAKNAS!
buren	1	5110(0.011432491139819366)	2359 (0.034)	#SAKNAS!
burial	1	5110(0.011432491139819366)	#SAKNAS!	#SAKNAS!
burkina	1	5110(0.011432491139819366)	#SAKNAS!	2548 (0.033)
burning	1	2214(0.04572996455927746)	2359 (0.034)	#SAKNAS!
burnout	1	5110(0.011432491139819366)	#SAKNAS!	#SAKNAS!
burnout-related	1	5110(0.011432491139819366)	#SAKNAS!	2548 (0.033)
burst	1	5110(0.011432491139819366)	#SAKNAS!	2548 (0.033)
burundi	1	5110(0.011432491139819366)	2359 (0.034)	#SAKNAS!
burundi	1	5110(0.011432491139819366)	2359 (0.034)	#SAKNAS!
bushcricket	1	5110(0.011432491139819366)	#SAKNAS!	2548 (0.033)
busting	1	5110(0.011432491139819366)	#SAKNAS!	2548 (0.033)
butterflyfish	1	3437(0.02286498227963873)	2359 (0.034)	2548 (0.033)
button	1	5110(0.011432491139819366)	#SAKNAS!	#SAKNAS!
butyrate	1	5110(0.011432491139819366)	#SAKNAS!	#SAKNAS!
bwindi	1	5110(0.011432491139819366)	2359 (0.034)	#SAKNAS!
bwindi	1	5110(0.011432491139819366)	2359 (0.034)	#SAKNAS!
bypasses	1	5110(0.011432491139819366)	#SAKNAS!	#SAKNAS!

by-product	1	5110(0.011432491139819366)	#SAKNAS!	#SAKNAS!
c1q	1	5110(0.011432491139819366)	#SAKNAS!	#SAKNAS!
c2-7	1	5110(0.011432491139819366)	#SAKNAS!	2548 (0.033)
c2-c7	1	3437(0.02286498227963873)	2359 (0.034)	2548 (0.033)
c-4	1	5110(0.011432491139819366)	2359 (0.034)	#SAKNAS!
c-4	1	5110(0.011432491139819366)	2359 (0.034)	#SAKNAS!
c57bl	1	2693(0.0342974734194581)	2359 (0.034)	2548 (0.033)
c57bl6	1	5110(0.011432491139819366)	#SAKNAS!	#SAKNAS!
c-634g	1	5110(0.011432491139819366)	#SAKNAS!	2548 (0.033)
ca	1	5110(0.011432491139819366)	#SAKNAS!	#SAKNAS!
cabbage	1	5110(0.011432491139819366)	#SAKNAS!	2548 (0.033)
cachexia	1	1485(0.08002743797873557)	2359 (0.034)	1668 (0.066)
cactus	1	1678(0.0685949468389162)	2359 (0.034)	1233 (0.1)
cadavers	1	5110(0.011432491139819366)	#SAKNAS!	2548 (0.033)
caddisflies	1	5110(0.011432491139819366)	#SAKNAS!	#SAKNAS!
cafe	1	5110(0.011432491139819366)	#SAKNAS!	2548 (0.033)
cafeteria	1	2693(0.0342974734194581)	2359 (0.034)	2548 (0.033)
cafeteria-style	1	5110(0.011432491139819366)	#SAKNAS!	2548 (0.033)
caffeine	1	5110(0.011432491139819366)	#SAKNAS!	#SAKNAS!
caffeine-mediated	1	5110(0.011432491139819366)	#SAKNAS!	2548 (0.033)
cage	1	5110(0.011432491139819366)	#SAKNAS!	#SAKNAS!
caged	1	5110(0.011432491139819366)	#SAKNAS!	2548 (0.033)
cage-plate	1	5110(0.011432491139819366)	#SAKNAS!	#SAKNAS!
calan	1	5110(0.011432491139819366)	2359 (0.034)	#SAKNAS!
calan	1	5110(0.011432491139819366)	2359 (0.034)	#SAKNAS!
calanus	1	5110(0.011432491139819366)	2359 (0.034)	#SAKNAS!
calanus	1	5110(0.011432491139819366)	2359 (0.034)	#SAKNAS!
calcium-phosphate	1	5110(0.011432491139819366)	#SAKNAS!	2548 (0.033)
calculating	1	5110(0.011432491139819366)	#SAKNAS!	#SAKNAS!
calculi	1	5110(0.011432491139819366)	#SAKNAS!	2548 (0.033)
cali	1	5110(0.011432491139819366)	2359 (0.034)	#SAKNAS!
cali	1	5110(0.011432491139819366)	2359 (0.034)	#SAKNAS!
calibration	1	5110(0.011432491139819366)	2359 (0.034)	#SAKNAS!
calibration	1	5110(0.011432491139819366)	2359 (0.034)	#SAKNAS!
caligus	1	5110(0.011432491139819366)	#SAKNAS!	2548 (0.033)
call	1	1894(0.0571624556990968З)	2359 (0.034)	1233 (0.1)
callithrix	1	5110(0.011432491139819366)	#SAKNAS!	#SAKNAS!
callitrichidae	1	5110(0.011432491139819366)	#SAKNAS!	2548 (0.033)
calls	1	5110(0.011432491139819366)	#SAKNAS!	#SAKNAS!
calonectris	1	3437(0.02286498227963873)	2359 (0.034)	2548 (0.033)
caloric-intake	1	5110(0.011432491139819366)	2359 (0.034)	#SAKNAS!
caloric-intake	1	5110(0.011432491139819366)	2359 (0.034)	#SAKNAS!
calorie	1	2693(0.0342974734194581)	2359 (0.034)	1668 (0.066)

calorie-dense	1	5110(0.011432491139819366)	#SAKNAS!	2548 (0.033)
calotropis	1	3437(0.02286498227963873)	2359 (0.034)	2548 (0.033)
calyptraeotheres	1	5110(0.011432491139819366)	#SAKNAS!	2548 (0.033)
camara	1	5110(0.011432491139819366)	#SAKNAS!	#SAKNAS!
cambodians	1	5110(0.011432491139819366)	#SAKNAS!	2548 (0.033)
cambridgeshire	1	5110(0.011432491139819366)	#SAKNAS!	2548 (0.033)
camcam	1	5110(0.011432491139819366)	#SAKNAS!	#SAKNAS!
camelopardalis	1	5110(0.011432491139819366)	#SAKNAS!	2548 (0.033)
camels	1	5110(0.011432491139819366)	#SAKNAS!	#SAKNAS!
campaign	1	5110(0.011432491139819366)	#SAKNAS!	2548 (0.033)
campaigns	1	5110(0.011432491139819366)	#SAKNAS!	#SAKNAS!
campinas	1	5110(0.011432491139819366)	2359 (0.034)	#SAKNAS!
campinas	1	5110(0.011432491139819366)	2359 (0.034)	#SAKNAS!
camrelizumab-containing	1	5110(0.011432491139819366)	#SAKNAS!	2548 (0.033)
canadensis	1	5110(0.011432491139819366)	2359 (0.034)	#SAKNAS!
canadensis	1	5110(0.011432491139819366)	2359 (0.034)	#SAKNAS!
canalized	1	5110(0.011432491139819366)	#SAKNAS!	2548 (0.033)
canaria	1	5110(0.011432491139819366)	2359 (0.034)	#SAKNAS!
canaria	1	5110(0.011432491139819366)	2359 (0.034)	#SAKNAS!
canarian	1	5110(0.011432491139819366)	2359 (0.034)	#SAKNAS!
canarian	1	5110(0.011432491139819366)	2359 (0.034)	#SAKNAS!
canary	1	5110(0.011432491139819366)	2359 (0.034)	#SAKNAS!
canary	1	5110(0.011432491139819366)	2359 (0.034)	#SAKNAS!
cancer-a	1	3437(0.02286498227963873)	2359 (0.034)	#SAKNAS!
cancer-free	1	5110(0.011432491139819366)	2359 (0.034)	#SAKNAS!
cancer-free	1	5110(0.011432491139819366)	2359 (0.034)	#SAKNAS!
cancerologia	1	5110(0.011432491139819366)	#SAKNAS!	2548 (0.033)
cancer-patients	1	5110(0.011432491139819366)	2359 (0.034)	#SAKNAS!
cancer-patients	1	5110(0.011432491139819366)	2359 (0.034)	#SAKNAS!
cancrum	1	5110(0.011432491139819366)	2359 (0.034)	#SAKNAS!
cancrum	1	5110(0.011432491139819366)	2359 (0.034)	#SAKNAS!
candidate!	1	5110(0.011432491139819366)	2359 (0.034)	#SAKNAS!
candidate!	1	5110(0.011432491139819366)	2359 (0.034)	#SAKNAS!
canescens	1	5110(0.011432491139819366)	2359 (0.034)	#SAKNAS!
canescens	1	5110(0.011432491139819366)	2359 (0.034)	#SAKNAS!
canidae	1	5110(0.011432491139819366)	2359 (0.034)	#SAKNAS!
canidae	1	5110(0.011432491139819366)	2359 (0.034)	#SAKNAS!
canine	1	2214(0.04572996455927746)	2359 (0.034)	1668 (0.066)
canis	1	5110(0.011432491139819366)	2359 (0.034)	#SAKNAS!
canis	1	5110(0.011432491139819366)	2359 (0.034)	#SAKNAS!
canna	1	5110(0.011432491139819366)	#SAKNAS!	2548 (0.033)
cannabinoid1	1	5110(0.011432491139819366)	2359 (0.034)	#SAKNAS!
cannabinoid1	1	5110(0.011432491139819366)	2359 (0.034)	#SAKNAS!

canned	1	5110(0.011432491139819366)	2359 (0.034)	#SAKNAS!
canned	1	5110(0.011432491139819366)	2359 (0.034)	#SAKNAS!
cannibalism	1	5110(0.011432491139819366)	2359 (0.034)	#SAKNAS!
cannibalism	1	5110(0.011432491139819366)	2359 (0.034)	#SAKNAS!
cannibalistic	1	3437(0.02286498227963873)	2359 (0.034)	2548 (0.033)
cannula	1	5110(0.011432491139819366)	#SAKNAS!	#SAKNAS!
cantabricus	1	5110(0.011432491139819366)	2359 (0.034)	#SAKNAS!
cantabricus	1	5110(0.011432491139819366)	2359 (0.034)	#SAKNAS!
capabilities	1	5110(0.011432491139819366)	#SAKNAS!	#SAKNAS!
capacities	1	5110(0.011432491139819366)	#SAKNAS!	#SAKNAS!
cape	1	2693(0.0342974734194581)	2359 (0.034)	1668 (0.066)
capella	1	5110(0.011432491139819366)	#SAKNAS!	#SAKNAS!
capensis	1	5110(0.011432491139819366)	#SAKNAS!	2548 (0.033)
capillary	1	5110(0.011432491139819366)	#SAKNAS!	2548 (0.033)
capnography	1	5110(0.011432491139819366)	#SAKNAS!	#SAKNAS!
capsaicin	1	5110(0.011432491139819366)	2359 (0.034)	#SAKNAS!
capsaicin	1	5110(0.011432491139819366)	2359 (0.034)	#SAKNAS!
captive-born	1	5110(0.011432491139819366)	#SAKNAS!	#SAKNAS!
captivity	1	3437(0.02286498227963873)	2359 (0.034)	2548 (0.033)
capture	1	3437(0.02286498227963873)	2359 (0.034)	#SAKNAS!
capuchin	1	5110(0.011432491139819366)	2359 (0.034)	#SAKNAS!
capuchin	1	5110(0.011432491139819366)	2359 (0.034)	#SAKNAS!
carabid	1	5110(0.011432491139819366)	#SAKNAS!	#SAKNAS!
carausius	1	5110(0.011432491139819366)	2359 (0.034)	#SAKNAS!
carausius	1	5110(0.011432491139819366)	2359 (0.034)	#SAKNAS!
carbidopa	1	5110(0.011432491139819366)	#SAKNAS!	2548 (0.033)
carbohydrate-rich	1	3437(0.02286498227963873)	2359 (0.034)	#SAKNAS!
carbon	1	1485(0.08002743797873557)	2359 (0.034)	999 (0.13)
carbonyl11c	1	5110(0.011432491139819366)	2359 (0.034)	#SAKNAS!
carbonyl11c	1	5110(0.011432491139819366)	2359 (0.034)	#SAKNAS!
carboplatin	1	5110(0.011432491139819366)	2359 (0.034)	#SAKNAS!
carboplatin	1	5110(0.011432491139819366)	2359 (0.034)	#SAKNAS!
carboxymethylated	1	5110(0.011432491139819366)	#SAKNAS!	#SAKNAS!
carboxymethylcellulose	1	5110(0.011432491139819366)	2359 (0.034)	#SAKNAS!
carboxymethylcellulose	1	5110(0.011432491139819366)	2359 (0.034)	#SAKNAS!
carcass	1	1485(0.08002743797873557)	2359 (0.034)	1668 (0.066)
carcharhinidae	1	5110(0.011432491139819366)	#SAKNAS!	2548 (0.033)
carcharhiniformes	1	5110(0.011432491139819366)	#SAKNAS!	2548 (0.033)
carcinoma-a	1	5110(0.011432491139819366)	#SAKNAS!	#SAKNAS!
carcinosarcoma	1	5110(0.011432491139819366)	2359 (0.034)	#SAKNAS!
carcinosarcoma	1	5110(0.011432491139819366)	2359 (0.034)	#SAKNAS!
carcinus	1	5110(0.011432491139819366)	#SAKNAS!	2548 (0.033)
cardiobacterium	1	5110(0.011432491139819366)	2359 (0.034)	#SAKNAS!

cardiobacterium	1	5110(0.011432491139819366)	2359 (0.034)	#SAKNAS!
cardiometabolic	1	787(0.1714873670972905)	2359 (0.034)	694 (0.2)
cardiomyotomy	1	5110(0.011432491139819366)	2359 (0.034)	#SAKNAS!
cardiomyotomy	1	5110(0.011432491139819366)	2359 (0.034)	#SAKNAS!
cardiovascular-high-risk	1	5110(0.011432491139819366)	#SAKNAS!	#SAKNAS!
cardui	1	5110(0.011432491139819366)	#SAKNAS!	2548 (0.033)
care-a	1	5110(0.011432491139819366)	#SAKNAS!	#SAKNAS!
care-dependent	1	5110(0.011432491139819366)	#SAKNAS!	2548 (0.033)
carefully	1	5110(0.011432491139819366)	#SAKNAS!	2548 (0.033)
caregiver-reported	1	5110(0.011432491139819366)	#SAKNAS!	2548 (0.033)
caregivers-intervention	1	5110(0.011432491139819366)	#SAKNAS!	#SAKNAS!
caregiving	1	5110(0.011432491139819366)	#SAKNAS!	2548 (0.033)
cares-hapin	1	5110(0.011432491139819366)	#SAKNAS!	#SAKNAS!
care-specific	1	5110(0.011432491139819366)	2359 (0.034)	#SAKNAS!
care-specific	1	5110(0.011432491139819366)	2359 (0.034)	#SAKNAS!
caretaking	1	5110(0.011432491139819366)	#SAKNAS!	2548 (0.033)
caretrack	1	5110(0.011432491139819366)	#SAKNAS!	#SAKNAS!
cargos	1	5110(0.011432491139819366)	#SAKNAS!	2548 (0.033)
caribbean	1	5110(0.011432491139819366)	#SAKNAS!	#SAKNAS!
caries-free	1	5110(0.011432491139819366)	#SAKNAS!	#SAKNAS!
caries-reducing	1	5110(0.011432491139819366)	2359 (0.034)	#SAKNAS!
caries-reducing	1	5110(0.011432491139819366)	2359 (0.034)	#SAKNAS!
caries-related	1	2693(0.0342974734194581)	2359 (0.034)	2548 (0.033)
caries-risk	1	5110(0.011432491139819366)	#SAKNAS!	2548 (0.033)
caries-suspected	1	5110(0.011432491139819366)	#SAKNAS!	#SAKNAS!
cariogenicity	1	2214(0.04572996455927746)	2359 (0.034)	2548 (0.033)
cariogram	1	5110(0.011432491139819366)	2359 (0.034)	#SAKNAS!
cariogram	1	5110(0.011432491139819366)	2359 (0.034)	#SAKNAS!
carnauba	1	5110(0.011432491139819366)	#SAKNAS!	#SAKNAS!
carnea	1	5110(0.011432491139819366)	2359 (0.034)	#SAKNAS!
carnea	1	5110(0.011432491139819366)	2359 (0.034)	#SAKNAS!
carnitine	1	5110(0.011432491139819366)	#SAKNAS!	#SAKNAS!
carnival	1	5110(0.011432491139819366)	2359 (0.034)	#SAKNAS!
carnival	1	5110(0.011432491139819366)	2359 (0.034)	#SAKNAS!
carnivore	1	3437(0.02286498227963873)	2359 (0.034)	2548 (0.033)
carnivores	1	5110(0.011432491139819366)	2359 (0.034)	#SAKNAS!
carnivores	1	5110(0.011432491139819366)	2359 (0.034)	#SAKNAS!
carnivorous	1	5110(0.011432491139819366)	#SAKNAS!	2548 (0.033)
carolitertii	1	5110(0.011432491139819366)	2359 (0.034)	#SAKNAS!
carolitertii	1	5110(0.011432491139819366)	2359 (0.034)	#SAKNAS!
carpio	1	5110(0.011432491139819366)	2359 (0.034)	#SAKNAS!
carpio	1	5110(0.011432491139819366)	2359 (0.034)	#SAKNAS!
carrier	1	5110(0.011432491139819366)	#SAKNAS!	#SAKNAS!

carrier-mediated	1	5110(0.011432491139819366)	2359 (0.034)	#SAKNAS!
carrier-mediated	1	5110(0.011432491139819366)	2359 (0.034)	#SAKNAS!
carriers	1	5110(0.011432491139819366)	#SAKNAS!	2548 (0.033)
carrying	1	5110(0.011432491139819366)	#SAKNAS!	2548 (0.033)
cascading	1	5110(0.011432491139819366)	#SAKNAS!	2548 (0.033)
caseinate	1	5110(0.011432491139819366)	#SAKNAS!	2548 (0.033)
case-match	1	5110(0.011432491139819366)	2359 (0.034)	#SAKNAS!
case-match	1	5110(0.011432491139819366)	2359 (0.034)	#SAKNAS!
case-matched	1	3437(0.02286498227963873)	2359 (0.034)	2548 (0.033)
caseous	1	5110(0.011432491139819366)	#SAKNAS!	#SAKNAS!
case-parent	1	5110(0.011432491139819366)	2359 (0.034)	#SAKNAS!
case-parent	1	5110(0.011432491139819366)	2359 (0.034)	#SAKNAS!
case-report	1	5110(0.011432491139819366)	2359 (0.034)	#SAKNAS!
case-report	1	5110(0.011432491139819366)	2359 (0.034)	#SAKNAS!
case-reports	1	5110(0.011432491139819366)	2359 (0.034)	#SAKNAS!
case-reports	1	5110(0.011432491139819366)	2359 (0.034)	#SAKNAS!
case-study	1	5110(0.011432491139819366)	#SAKNAS!	2548 (0.033)
cash	1	5110(0.011432491139819366)	#SAKNAS!	#SAKNAS!
cashew	1	5110(0.011432491139819366)	#SAKNAS!	#SAKNAS!
cassava	1	5110(0.011432491139819366)	#SAKNAS!	2548 (0.033)
caste	1	5110(0.011432491139819366)	2359 (0.034)	#SAKNAS!
caste	1	5110(0.011432491139819366)	2359 (0.034)	#SAKNAS!
castration	1	5110(0.011432491139819366)	#SAKNAS!	2548 (0.033)
casts	1	5110(0.011432491139819366)	#SAKNAS!	2548 (0.033)
catalonia	1	5110(0.011432491139819366)	#SAKNAS!	#SAKNAS!
catarrhine	1	5110(0.011432491139819366)	#SAKNAS!	2548 (0.033)
catastrophic	1	5110(0.011432491139819366)	#SAKNAS!	#SAKNAS!
catch-up	1	5110(0.011432491139819366)	2359 (0.034)	#SAKNAS!
catch-up	1	5110(0.011432491139819366)	2359 (0.034)	#SAKNAS!
catecholamines	1	5110(0.011432491139819366)	#SAKNAS!	2548 (0.033)
categorization	1	5110(0.011432491139819366)	#SAKNAS!	#SAKNAS!
categorize	1	5110(0.011432491139819366)	#SAKNAS!	2548 (0.033)
catering	1	5110(0.011432491139819366)	#SAKNAS!	#SAKNAS!
catfish	1	3437(0.02286498227963873)	2359 (0.034)	2548 (0.033)
catherines	1	5110(0.011432491139819366)	2359 (0.034)	#SAKNAS!
catherines	1	5110(0.011432491139819366)	2359 (0.034)	#SAKNAS!
catla	1	3437(0.02286498227963873)	2359 (0.034)	2548 (0.033)
catus	1	5110(0.011432491139819366)	2359 (0.034)	#SAKNAS!
catus	1	5110(0.011432491139819366)	2359 (0.034)	#SAKNAS!
cauca	1	5110(0.011432491139819366)	#SAKNAS!	#SAKNAS!
caucasian	1	5110(0.011432491139819366)	2359 (0.034)	#SAKNAS!
caucasian	1	5110(0.011432491139819366)	2359 (0.034)	#SAKNAS!
caudomedial	1	5110(0.011432491139819366)	#SAKNAS!	2548 (0.033)

caulophyllum	1	5110(0.011432491139819366)	#SAKNAS!	#SAKNAS!
causal	1	787(0.1714873670972905)	2359 (0.034)	2548 (0.033)
cavities	1	5110(0.011432491139819366)	#SAKNAS!	2548 (0.033)
cayo	1	5110(0.011432491139819366)	#SAKNAS!	2548 (0.033)
cb1	1	5110(0.011432491139819366)	#SAKNAS!	2548 (0.033)
cb1r-	1	5110(0.011432491139819366)	#SAKNAS!	2548 (0.033)
cbp-positive	1	5110(0.011432491139819366)	#SAKNAS!	#SAKNAS!
cchamide-1	1	5110(0.011432491139819366)	#SAKNAS!	#SAKNAS!
cchamide2-receptor	1	5110(0.011432491139819366)	#SAKNAS!	#SAKNAS!
cchs	1	5110(0.011432491139819366)	#SAKNAS!	#SAKNAS!
cci-779	1	5110(0.011432491139819366)	#SAKNAS!	2548 (0.033)
ccks	1	5110(0.011432491139819366)	#SAKNAS!	2548 (0.033)
cd4	1	5110(0.011432491139819366)	2359 (0.034)	#SAKNAS!
cd4	1	5110(0.011432491139819366)	2359 (0.034)	#SAKNAS!
cd4+cell	1	5110(0.011432491139819366)	#SAKNAS!	2548 (0.033)
cd8	1	5110(0.011432491139819366)	2359 (0.034)	#SAKNAS!
cd8	1	5110(0.011432491139819366)	2359 (0.034)	#SAKNAS!
cddp	1	5110(0.011432491139819366)	#SAKNAS!	#SAKNAS!
cdh	1	5110(0.011432491139819366)	#SAKNAS!	#SAKNAS!
ce	1	5110(0.011432491139819366)	#SAKNAS!	2548 (0.033)
ceara	1	5110(0.011432491139819366)	2359 (0.034)	#SAKNAS!
ceara	1	5110(0.011432491139819366)	2359 (0.034)	#SAKNAS!
cebus	1	5110(0.011432491139819366)	2359 (0.034)	#SAKNAS!
cebus	1	5110(0.011432491139819366)	2359 (0.034)	#SAKNAS!
cecum	1	5110(0.011432491139819366)	2359 (0.034)	#SAKNAS!
cecum	1	5110(0.011432491139819366)	2359 (0.034)	#SAKNAS!
cedrelone	1	5110(0.011432491139819366)	2359 (0.034)	#SAKNAS!
cedrelone	1	5110(0.011432491139819366)	2359 (0.034)	#SAKNAS!
celiac	1	2214(0.04572996455927746)	2359 (0.034)	#SAKNAS!
cell-mediated-immunity	1	5110(0.011432491139819366)	2359 (0.034)	#SAKNAS!
cell-mediated-immunity	1	5110(0.011432491139819366)	2359 (0.034)	#SAKNAS!
cellular	1	3437(0.02286498227963873)	2359 (0.034)	2548 (0.033)
cellulose	1	3437(0.02286498227963873)	2359 (0.034)	#SAKNAS!
cementoblast	1	5110(0.011432491139819366)	2359 (0.034)	#SAKNAS!
cementoblast	1	5110(0.011432491139819366)	2359 (0.034)	#SAKNAS!
cementum	1	5110(0.011432491139819366)	2359 (0.034)	#SAKNAS!
cementum	1	5110(0.011432491139819366)	2359 (0.034)	#SAKNAS!
center-delivered	1	5110(0.011432491139819366)	2359 (0.034)	#SAKNAS!
center-delivered	1	5110(0.011432491139819366)	2359 (0.034)	#SAKNAS!
center-reported	1	5110(0.011432491139819366)	#SAKNAS!	2548 (0.033)
centipede	1	5110(0.011432491139819366)	#SAKNAS!	2548 (0.033)
central-acting	1	5110(0.011432491139819366)	#SAKNAS!	2548 (0.033)
centre	1	1678(0.0685949468389162)	2359 (0.034)	1233 (0.1)

centurio	1	5110(0.011432491139819366)	#SAKNAS!	2548 (0.033)
cephalopods	1	5110(0.011432491139819366)	#SAKNAS!	2548 (0.033)
cephalus	1	5110(0.011432491139819366)	2359 (0.034)	#SAKNAS!
cephalus	1	5110(0.011432491139819366)	2359 (0.034)	#SAKNAS!
cerastoderma-edule	1	5110(0.011432491139819366)	2359 (0.034)	#SAKNAS!
cerastoderma-edule	1	5110(0.011432491139819366)	2359 (0.034)	#SAKNAS!
cereals	1	5110(0.011432491139819366)	#SAKNAS!	2548 (0.033)
cerebellar	1	5110(0.011432491139819366)	#SAKNAS!	2548 (0.033)
cerebellum	1	3437(0.02286498227963873)	2359 (0.034)	#SAKNAS!
cerebrospinal	1	2693(0.0342974734194581)	2359 (0.034)	2548 (0.033)
cerebrospinal-fluid	1	5110(0.011432491139819366)	2359 (0.034)	#SAKNAS!
cerebrospinal-fluid	1	5110(0.011432491139819366)	2359 (0.034)	#SAKNAS!
cergas	1	5110(0.011432491139819366)	#SAKNAS!	#SAKNAS!
cerrado	1	5110(0.011432491139819366)	#SAKNAS!	2548 (0.033)
cervid	1	5110(0.011432491139819366)	2359 (0.034)	#SAKNAS!
cervid	1	5110(0.011432491139819366)	2359 (0.034)	#SAKNAS!
cervidae	1	5110(0.011432491139819366)	2359 (0.034)	#SAKNAS!
cervidae	1	5110(0.011432491139819366)	2359 (0.034)	#SAKNAS!
cervids	1	5110(0.011432491139819366)	#SAKNAS!	2548 (0.033)
cessation	1	1678(0.0685949468389162)	2359 (0.034)	999 (0.13)
cestoda	1	5110(0.011432491139819366)	2359 (0.034)	#SAKNAS!
cestoda	1	5110(0.011432491139819366)	2359 (0.034)	#SAKNAS!
cfa	1	5110(0.011432491139819366)	2359 (0.034)	#SAKNAS!
cfa	1	5110(0.011432491139819366)	2359 (0.034)	#SAKNAS!
cfq	1	5110(0.011432491139819366)	2359 (0.034)	#SAKNAS!
cfq	1	5110(0.011432491139819366)	2359 (0.034)	#SAKNAS!
cga	1	5110(0.011432491139819366)	#SAKNAS!	#SAKNAS!
chabaudi	1	5110(0.011432491139819366)	2359 (0.034)	#SAKNAS!
chabaudi	1	5110(0.011432491139819366)	2359 (0.034)	#SAKNAS!
chaetica	1	5110(0.011432491139819366)	#SAKNAS!	2548 (0.033)
chaetodon	1	3437(0.02286498227963873)	2359 (0.034)	2548 (0.033)
chaff-based	1	5110(0.011432491139819366)	2359 (0.034)	#SAKNAS!
chaff-based	1	5110(0.011432491139819366)	2359 (0.034)	#SAKNAS!
chagasic	1	3437(0.02286498227963873)	2359 (0.034)	2548 (0.033)
chair	1	5110(0.011432491139819366)	#SAKNAS!	2548 (0.033)
chairside	1	3437(0.02286498227963873)	2359 (0.034)	2548 (0.033)
chair-side	1	5110(0.011432491139819366)	#SAKNAS!	2548 (0.033)
challenging	1	5110(0.011432491139819366)	#SAKNAS!	#SAKNAS!
chamber	1	5110(0.011432491139819366)	#SAKNAS!	#SAKNAS!
champ	1	5110(0.011432491139819366)	#SAKNAS!	#SAKNAS!
champlain	1	5110(0.011432491139819366)	2359 (0.034)	#SAKNAS!
champlain	1	5110(0.011432491139819366)	2359 (0.034)	#SAKNAS!
chance	1	3437(0.02286498227963873)	2359 (0.034)	#SAKNAS!

chances	1	5110(0.011432491139819366)	#SAKNAS!	2548 (0.033)
change-based	1	5110(0.011432491139819366)	2359 (0.034)	#SAKNAS!
change-based	1	5110(0.011432491139819366)	2359 (0.034)	#SAKNAS!
channel	1	2693(0.0342974734194581)	2359 (0.034)	2548 (0.033)
chaperone	1	5110(0.011432491139819366)	2359 (0.034)	#SAKNAS!
chaperone	1	5110(0.011432491139819366)	2359 (0.034)	#SAKNAS!
chapter	1	5110(0.011432491139819366)	#SAKNAS!	#SAKNAS!
characiforms	1	5110(0.011432491139819366)	2359 (0.034)	#SAKNAS!
characiforms	1	5110(0.011432491139819366)	2359 (0.034)	#SAKNAS!
character	1	5110(0.011432491139819366)	2359 (0.034)	#SAKNAS!
character	1	5110(0.011432491139819366)	2359 (0.034)	#SAKNAS!
characteristic	1	3437(0.02286498227963873)	2359 (0.034)	2548 (0.033)
characterizing	1	2214(0.04572996455927746)	2359 (0.034)	1668 (0.066)
chara-fragilis	1	5110(0.011432491139819366)	2359 (0.034)	#SAKNAS!
chara-fragilis	1	5110(0.011432491139819366)	2359 (0.034)	#SAKNAS!
charge	1	5110(0.011432491139819366)	#SAKNAS!	2548 (0.033)
charls	1	5110(0.011432491139819366)	#SAKNAS!	#SAKNAS!
charm	1	5110(0.011432491139819366)	#SAKNAS!	#SAKNAS!
charr	1	5110(0.011432491139819366)	2359 (0.034)	#SAKNAS!
charr	1	5110(0.011432491139819366)	2359 (0.034)	#SAKNAS!
chd	1	3437(0.02286498227963873)	2359 (0.034)	2548 (0.033)
cheap	1	3437(0.02286498227963873)	2359 (0.034)	#SAKNAS!
cheating	1	5110(0.011432491139819366)	#SAKNAS!	2548 (0.033)
check-all-that-apply	1	5110(0.011432491139819366)	#SAKNAS!	2548 (0.033)
checklista穢	1	5110(0.011432491139819366)	2359 (0.034)	#SAKNAS!
checklista穢	1	5110(0.011432491139819366)	2359 (0.034)	#SAKNAS!
checks	1	5110(0.011432491139819366)	#SAKNAS!	2548 (0.033)
check-up	1	5110(0.011432491139819366)	#SAKNAS!	#SAKNAS!
cheek	1	5110(0.011432491139819366)	2359 (0.034)	#SAKNAS!
cheek	1	5110(0.011432491139819366)	2359 (0.034)	#SAKNAS!
cheese	1	5110(0.011432491139819366)	2359 (0.034)	#SAKNAS!
cheese	1	5110(0.011432491139819366)	2359 (0.034)	#SAKNAS!
cheffia	1	5110(0.011432491139819366)	#SAKNAS!	#SAKNAS!
cheilitis	1	5110(0.011432491139819366)	2359 (0.034)	#SAKNAS!
cheilitis	1	5110(0.011432491139819366)	2359 (0.034)	#SAKNAS!
chemically	1	3437(0.02286498227963873)	2359 (0.034)	2548 (0.033)
chemo	1	2214(0.04572996455927746)	2359 (0.034)	1233 (0.1)
chemo-attract	1	5110(0.011432491139819366)	#SAKNAS!	#SAKNAS!
chemoboost	1	5110(0.011432491139819366)	2359 (0.034)	#SAKNAS!
chemoboost	1	5110(0.011432491139819366)	2359 (0.034)	#SAKNAS!
chemoembolization	1	5110(0.011432491139819366)	#SAKNAS!	#SAKNAS!
chemoirradiation	1	5110(0.011432491139819366)	2359 (0.034)	#SAKNAS!
chemoirradiation	1	5110(0.011432491139819366)	2359 (0.034)	#SAKNAS!

chemo-radiation	1	5110(0.011432491139819366)	2359 (0.034)	#SAKNAS!
chemo-radiation	1	5110(0.011432491139819366)	2359 (0.034)	#SAKNAS!
chemoradiation-induced	1	5110(0.011432491139819366)	#SAKNAS!	2548 (0.033)
chemosensory	1	1894(0.05716245569909683)	2359 (0.034)	1233 (0.1)
chemotherapeutic	1	5110(0.011432491139819366)	#SAKNAS!	2548 (0.033)
chemotherapy-based	1	5110(0.011432491139819366)	#SAKNAS!	2548 (0.033)
chest	1	5110(0.011432491139819366)	2359 (0.034)	#SAKNAS!
chest	1	5110(0.011432491139819366)	2359 (0.034)	#SAKNAS!
chew	1	2693(0.0342974734194581)	2359 (0.034)	#SAKNAS!
chews	1	5110(0.011432491139819366)	#SAKNAS!	2548 (0.033)
chiari	1	5110(0.011432491139819366)	2359 (0.034)	#SAKNAS!
chiari	1	5110(0.011432491139819366)	2359 (0.034)	#SAKNAS!
chiasmocleis	1	5110(0.011432491139819366)	#SAKNAS!	2548 (0.033)
chick	1	5110(0.011432491139819366)	#SAKNAS!	2548 (0.033)
chicken	1	1894(0.05716245569909683)	2359 (0.034)	1668 (0.066)
chickpea-mealworm	1	5110(0.011432491139819366)	#SAKNAS!	#SAKNAS!
chicory	1	5110(0.011432491139819366)	#SAKNAS!	2548 (0.033)
child-	1	5110(0.011432491139819366)	#SAKNAS!	#SAKNAS!
childcare	1	5110(0.011432491139819366)	#SAKNAS!	#SAKNAS!
child-oidp	1	5110(0.011432491139819366)	#SAKNAS!	#SAKNAS!
child-oral	1	5110(0.011432491139819366)	#SAKNAS!	2548 (0.033)
childrearing	1	5110(0.011432491139819366)	#SAKNAS!	2548 (0.033)
child-related	1	5110(0.011432491139819366)	#SAKNAS!	#SAKNAS!
children-extent	1	5110(0.011432491139819366)	2359 (0.034)	#SAKNAS!
children-extent	1	5110(0.011432491139819366)	2359 (0.034)	#SAKNAS!
children-sediento	1	5110(0.011432491139819366)	#SAKNAS!	#SAKNAS!
children-the	1	5110(0.011432491139819366)	#SAKNAS!	2548 (0.033)
child-youth	1	5110(0.011432491139819366)	#SAKNAS!	2548 (0.033)
chilensis	1	5110(0.011432491139819366)	2359 (0.034)	#SAKNAS!
chilensis	1	5110(0.011432491139819366)	2359 (0.034)	#SAKNAS!
chimeric	1	5110(0.011432491139819366)	#SAKNAS!	#SAKNAS!
chimpanzee	1	3437(0.02286498227963873)	2359 (0.034)	2548 (0.033)
chinese-american	1	3437(0.02286498227963873)	2359 (0.034)	#SAKNAS!
chinese-born	1	5110(0.011432491139819366)	#SAKNAS!	2548 (0.033)
chinook	1	5110(0.011432491139819366)	#SAKNAS!	2548 (0.033)
chipping	1	5110(0.011432491139819366)	#SAKNAS!	#SAKNAS!
chirixalus	1	5110(0.011432491139819366)	2359 (0.034)	#SAKNAS!
chirixalus	1	5110(0.011432491139819366)	2359 (0.034)	#SAKNAS!
chiroderma	1	5110(0.011432491139819366)	#SAKNAS!	2548 (0.033)
chironomids	1	5110(0.011432491139819366)	#SAKNAS!	2548 (0.033)
chiroptera	1	5110(0.011432491139819366)	#SAKNAS!	2548 (0.033)
chirp	1	5110(0.011432491139819366)	#SAKNAS!	#SAKNAS!
chlamydia	1	5110(0.011432491139819366)	#SAKNAS!	#SAKNAS!

chlamydomonas	1 5110(0.011432491139819366)	2359 (0.034)	#SAKNAS!
chlamydomonas	1 5110(0.011432491139819366)	2359 (0.034)	#SAKNAS!
chlorella	1 5110(0.011432491139819366)	#SAKNAS!	#SAKNAS!
chloride	1 3437(0.02286498227963873)	2359 (0.034)	#SAKNAS!
choanoflagellate	1 5110(0.011432491139819366)	2359 (0.034)	#SAKNAS!
choanoflagellate	1 5110(0.011432491139819366)	2359 (0.034)	#SAKNAS!
cholecystectomy	1 5110(0.011432491139819366)	2359 (0.034)	#SAKNAS!
cholecystectomy	1 5110(0.011432491139819366)	2359 (0.034)	#SAKNAS!
cholecystokinin-like	1 5110(0.011432491139819366)	#SAKNAS!	#SAKNAS!
cholesterol-rich	1 5110(0.011432491139819366)	#SAKNAS!	2548 (0.033)
chongqing	1 5110(0.011432491139819366)	#SAKNAS!	2548 (0.033)
choosy	1 5110(0.011432491139819366)	#SAKNAS!	2548 (0.033)
choristoneura	1 5110(0.011432491139819366)	2359 (0.034)	#SAKNAS!
choristoneura	1 5110(0.011432491139819366)	2359 (0.034)	#SAKNAS!
chorthippus	1 5110(0.011432491139819366)	#SAKNAS!	2548 (0.033)
chow	1 5110(0.011432491139819366)	#SAKNAS!	#SAKNAS!
chrna5	1 5110(0.011432491139819366)	#SAKNAS!	2548 (0.033)
chromatography	1 5110(0.011432491139819366)	#SAKNAS!	#SAKNAS!
chronic-alcoholism	1 5110(0.011432491139819366)	2359 (0.034)	#SAKNAS!
chronic-alcoholism	1 5110(0.011432491139819366)	2359 (0.034)	#SAKNAS!
chronically	1 5110(0.011432491139819366)	2359 (0.034)	#SAKNAS!
chronically	1 5110(0.011432491139819366)	2359 (0.034)	#SAKNAS!
chronological	1 5110(0.011432491139819366)	#SAKNAS!	2548 (0.033)
chronologies	1 5110(0.011432491139819366)	#SAKNAS!	#SAKNAS!
chrononutrition	1 5110(0.011432491139819366)	#SAKNAS!	#SAKNAS!
chrono-nutrition	1 5110(0.011432491139819366)	#SAKNAS!	2548 (0.033)
chrysoperla	1 5110(0.011432491139819366)	2359 (0.034)	#SAKNAS!
chrysoperla	1 5110(0.011432491139819366)	2359 (0.034)	#SAKNAS!
chrysophyte	1 3437(0.02286498227963873)	2359 (0.034)	#SAKNAS!
chrysothamnus	1 5110(0.011432491139819366)	2359 (0.034)	#SAKNAS!
chrysothamnus	1 5110(0.011432491139819366)	2359 (0.034)	#SAKNAS!
chuatsi	1 5110(0.011432491139819366)	#SAKNAS!	#SAKNAS!
church	1 5110(0.011432491139819366)	2359 (0.034)	#SAKNAS!
church	1 5110(0.011432491139819366)	2359 (0.034)	#SAKNAS!
chylothorax	1 5110(0.011432491139819366)	#SAKNAS!	2548 (0.033)
chymotrypsin-like	1 5110(0.011432491139819366)	#SAKNAS!	2548 (0.033)
cichlidae	1 5110(0.011432491139819366)	#SAKNAS!	2548 (0.033)
cichlids	1 5110(0.011432491139819366)	2359 (0.034)	#SAKNAS!
cichlids	1 5110(0.011432491139819366)	2359 (0.034)	#SAKNAS!
ciliate	1 5110(0.011432491139819366)	2359 (0.034)	#SAKNAS!
ciliate	1 5110(0.011432491139819366)	2359 (0.034)	#SAKNAS!
ciliatum	1 5110(0.011432491139819366)	#SAKNAS!	2548 (0.033)
circuitry	1 5110(0.011432491139819366)	#SAKNAS!	2548 (0.033)

circular	1	5110(0.011432491139819366)	2359 (0.034)	#SAKNAS!
circular	1	5110(0.011432491139819366)	2359 (0.034)	#SAKNAS!
circumferential	1	5110(0.011432491139819366)	2359 (0.034)	#SAKNAS!
circumferential	1	5110(0.011432491139819366)	2359 (0.034)	#SAKNAS!
circumvent	1	5110(0.011432491139819366)	2359 (0.034)	#SAKNAS!
circumvent	1	5110(0.011432491139819366)	2359 (0.034)	#SAKNAS!
circumventricular	1	5110(0.011432491139819366)	2359 (0.034)	#SAKNAS!
circumventricular	1	5110(0.011432491139819366)	2359 (0.034)	#SAKNAS!
cirrhatus	1	5110(0.011432491139819366)	#SAKNAS!	2548 (0.033)
cirrhifer	1	5110(0.011432491139819366)	#SAKNAS!	#SAKNAS!
cirrhinus	1	5110(0.011432491139819366)	2359 (0.034)	#SAKNAS!
cirrhinus	1	5110(0.011432491139819366)	2359 (0.034)	#SAKNAS!
cirt	1	5110(0.011432491139819366)	#SAKNAS!	2548 (0.033)
cisplatin	1	3437(0.02286498227963873)	2359 (0.034)	2548 (0.033)
cities	1	5110(0.011432491139819366)	#SAKNAS!	2548 (0.033)
citizen	1	5110(0.011432491139819366)	#SAKNAS!	2548 (0.033)
citizens	1	5110(0.011432491139819366)	#SAKNAS!	2548 (0.033)
citric	1	5110(0.011432491139819366)	#SAKNAS!	2548 (0.033)
citricidaon	1	5110(0.011432491139819366)	#SAKNAS!	2548 (0.033)
city-iraq	1	5110(0.011432491139819366)	#SAKNAS!	#SAKNAS!
civilian	1	5110(0.011432491139819366)	#SAKNAS!	2548 (0.033)
civilization	1	5110(0.011432491139819366)	2359 (0.034)	#SAKNAS!
civilization	1	5110(0.011432491139819366)	2359 (0.034)	#SAKNAS!
ckd	1	5110(0.011432491139819366)	2359 (0.034)	#SAKNAS!
ckd	1	5110(0.011432491139819366)	2359 (0.034)	#SAKNAS!
cladoceran	1	5110(0.011432491139819366)	2359 (0.034)	#SAKNAS!
cladoceran	1	5110(0.011432491139819366)	2359 (0.034)	#SAKNAS!
cladodes	1	5110(0.011432491139819366)	#SAKNAS!	2548 (0.033)
claims	1	5110(0.011432491139819366)	#SAKNAS!	#SAKNAS!
clams	1	3437(0.02286498227963873)	2359 (0.034)	#SAKNAS!
clarity	1	5110(0.011432491139819366)	2359 (0.034)	#SAKNAS!
clarity	1	5110(0.011432491139819366)	2359 (0.034)	#SAKNAS!
clarki	1	5110(0.011432491139819366)	2359 (0.034)	#SAKNAS!
clarki	1	5110(0.011432491139819366)	2359 (0.034)	#SAKNAS!
class-based	1	5110(0.011432491139819366)	2359 (0.034)	#SAKNAS!
class-based	1	5110(0.011432491139819366)	2359 (0.034)	#SAKNAS!
classic	1	5110(0.011432491139819366)	2359 (0.034)	#SAKNAS!
classic	1	5110(0.011432491139819366)	2359 (0.034)	#SAKNAS!
classical	1	5110(0.011432491139819366)	#SAKNAS!	#SAKNAS!
classifier	1	5110(0.011432491139819366)	#SAKNAS!	#SAKNAS!
clay	1	5110(0.011432491139819366)	2359 (0.034)	#SAKNAS!
clay	1	5110(0.011432491139819366)	2359 (0.034)	#SAKNAS!
clear	1	5110(0.011432491139819366)	#SAKNAS!	2548 (0.033)

clearance	1	5110(0.011432491139819366)	2359 (0.034)	#SAKNAS!
clearance	1	5110(0.011432491139819366)	2359 (0.034)	#SAKNAS!
clear-water	1	5110(0.011432491139819366)	#SAKNAS!	2548 (0.033)
clear簣	1	5110(0.011432491139819366)	#SAKNAS!	2548 (0.033)
clefts	1	3437(0.02286498227963873)	2359 (0.034)	#SAKNAS!
clem	1	5110(0.011432491139819366)	2359 (0.034)	#SAKNAS!
clem	1	5110(0.011432491139819366)	2359 (0.034)	#SAKNAS!
cleveland	1	3437(0.02286498227963873)	2359 (0.034)	2548 (0.033)
climatic	1	5110(0.011432491139819366)	2359 (0.034)	#SAKNAS!
climatic	1	5110(0.011432491139819366)	2359 (0.034)	#SAKNAS!
climbing	1	5110(0.011432491139819366)	#SAKNAS!	#SAKNAS!
clinical-administrative	1	5110(0.011432491139819366)	#SAKNAS!	2548 (0.033)
clinical-geriatric	1	5110(0.011432491139819366)	2359 (0.034)	#SAKNAS!
clinical-geriatric	1	5110(0.011432491139819366)	2359 (0.034)	#SAKNAS!
clinically	1	1678(0.0685949468389162)	2359 (0.034)	999 (0.13)
clinico-biochemical	1	5110(0.011432491139819366)	#SAKNAS!	#SAKNAS!
clipping	1	5110(0.011432491139819366)	2359 (0.034)	#SAKNAS!
clipping	1	5110(0.011432491139819366)	2359 (0.034)	#SAKNAS!
clm	1	5110(0.011432491139819366)	#SAKNAS!	2548 (0.033)
clock	1	5110(0.011432491139819366)	#SAKNAS!	2548 (0.033)
cloi-d-00160r2	1	5110(0.011432491139819366)	2359 (0.034)	#SAKNAS!
cloi-d-00160r2	1	5110(0.011432491139819366)	2359 (0.034)	#SAKNAS!
clonal	1	5110(0.011432491139819366)	#SAKNAS!	#SAKNAS!
clonazepam	1	5110(0.011432491139819366)	#SAKNAS!	#SAKNAS!
cloning	1	5110(0.011432491139819366)	2359 (0.034)	#SAKNAS!
cloning	1	5110(0.011432491139819366)	2359 (0.034)	#SAKNAS!
closed-loop	1	5110(0.011432491139819366)	#SAKNAS!	2548 (0.033)
closing	1	5110(0.011432491139819366)	#SAKNAS!	2548 (0.033)
clotrimazole	1	5110(0.011432491139819366)	#SAKNAS!	#SAKNAS!
clover	1	5110(0.011432491139819366)	2359 (0.034)	#SAKNAS!
clover	1	5110(0.011432491139819366)	2359 (0.034)	#SAKNAS!
clozapine	1	5110(0.011432491139819366)	#SAKNAS!	#SAKNAS!
clozapine-treated	1	5110(0.011432491139819366)	#SAKNAS!	#SAKNAS!
clsa	1	5110(0.011432491139819366)	#SAKNAS!	#SAKNAS!
cluj-napoca	1	5110(0.011432491139819366)	#SAKNAS!	2548 (0.033)
clupeiform	1	5110(0.011432491139819366)	2359 (0.034)	#SAKNAS!
clupeiform	1	5110(0.011432491139819366)	2359 (0.034)	#SAKNAS!
cluster-randomised	1	5110(0.011432491139819366)	#SAKNAS!	2548 (0.033)
cluster-randomized	1	1894(0.05716245569909683)	2359 (0.034)	1233 (0.1)
cnn	1	5110(0.011432491139819366)	#SAKNAS!	2548 (0.033)
cnr1	1	5110(0.011432491139819366)	#SAKNAS!	2548 (0.033)
cns	1	5110(0.011432491139819366)	#SAKNAS!	#SAKNAS!
cntf	1	5110(0.011432491139819366)	2359 (0.034)	#SAKNAS!

cntf	1	5110(0.011432491139819366)	2359 (0.034)	#SAKNAS!
co2	1	2214(0.04572996455927746)	2359 (0.034)	1233 (0.1)
co2concentration	1	5110(0.011432491139819366)	#SAKNAS!	2548 (0.033)
coadministration	1	5110(0.011432491139819366)	2359 (0.034)	#SAKNAS!
coadministration	1	5110(0.011432491139819366)	2359 (0.034)	#SAKNAS!
coarse	1	5110(0.011432491139819366)	2359 (0.034)	#SAKNAS!
coarse	1	5110(0.011432491139819366)	2359 (0.034)	#SAKNAS!
coccidial	1	5110(0.011432491139819366)	2359 (0.034)	#SAKNAS!
coccidial	1	5110(0.011432491139819366)	2359 (0.034)	#SAKNAS!
coccidiosis	1	5110(0.011432491139819366)	2359 (0.034)	#SAKNAS!
coccidiosis	1	5110(0.011432491139819366)	2359 (0.034)	#SAKNAS!
coccinellidae	1	3437(0.02286498227963873)	2359 (0.034)	2548 (0.033)
coccoides	1	5110(0.011432491139819366)	2359 (0.034)	#SAKNAS!
coccoides	1	5110(0.011432491139819366)	2359 (0.034)	#SAKNAS!
cockle	1	5110(0.011432491139819366)	2359 (0.034)	#SAKNAS!
cockle	1	5110(0.011432491139819366)	2359 (0.034)	#SAKNAS!
cockles	1	5110(0.011432491139819366)	2359 (0.034)	#SAKNAS!
cockles	1	5110(0.011432491139819366)	2359 (0.034)	#SAKNAS!
cockroach	1	3437(0.02286498227963873)	2359 (0.034)	#SAKNAS!
cockroach-specific	1	5110(0.011432491139819366)	#SAKNAS!	2548 (0.033)
cocoa	1	5110(0.011432491139819366)	#SAKNAS!	#SAKNAS!
co-crystallized	1	5110(0.011432491139819366)	2359 (0.034)	#SAKNAS!
co-crystallized	1	5110(0.011432491139819366)	2359 (0.034)	#SAKNAS!
cod	1	2693(0.0342974734194581)	2359 (0.034)	2548 (0.033)
co-design	1	5110(0.011432491139819366)	#SAKNAS!	#SAKNAS!
codesigned	1	5110(0.011432491139819366)	#SAKNAS!	#SAKNAS!
coding	1	5110(0.011432491139819366)	#SAKNAS!	2548 (0.033)
coefficient	1	5110(0.011432491139819366)	#SAKNAS!	#SAKNAS!
coefficients	1	5110(0.011432491139819366)	2359 (0.034)	#SAKNAS!
coefficients	1	5110(0.011432491139819366)	2359 (0.034)	#SAKNAS!
coeliac	1	5110(0.011432491139819366)	#SAKNAS!	2548 (0.033)
coenzyme	1	5110(0.011432491139819366)	#SAKNAS!	2548 (0.033)
coevolution	1	5110(0.011432491139819366)	#SAKNAS!	#SAKNAS!
coexistence	1	5110(0.011432491139819366)	#SAKNAS!	#SAKNAS!
co-existence	1	5110(0.011432491139819366)	#SAKNAS!	#SAKNAS!
coexisting	1	5110(0.011432491139819366)	#SAKNAS!	#SAKNAS!
cofactors	1	5110(0.011432491139819366)	#SAKNAS!	2548 (0.033)
cognate	1	5110(0.011432491139819366)	#SAKNAS!	#SAKNAS!
cognitively	1	5110(0.011432491139819366)	2359 (0.034)	#SAKNAS!
cognitively	1	5110(0.011432491139819366)	2359 (0.034)	#SAKNAS!
coherence	1	5110(0.011432491139819366)	#SAKNAS!	2548 (0.033)
cohesion	1	5110(0.011432491139819366)	2359 (0.034)	#SAKNAS!
cohesion	1	5110(0.011432491139819366)	2359 (0.034)	#SAKNAS!

coho	1	5110(0.011432491139819366)	#SAKNAS!	2548 (0.033)
c-oidp	1	5110(0.011432491139819366)	#SAKNAS!	2548 (0.033)
coincident	1	5110(0.011432491139819366)	2359 (0.034)	#SAKNAS!
coincident	1	5110(0.011432491139819366)	2359 (0.034)	#SAKNAS!
coix	1	5110(0.011432491139819366)	#SAKNAS!	2548 (0.033)
cola	1	5110(0.011432491139819366)	2359 (0.034)	#SAKNAS!
cola	1	5110(0.011432491139819366)	2359 (0.034)	#SAKNAS!
cold	1	5110(0.011432491139819366)	#SAKNAS!	2548 (0.033)
coldent	1	5110(0.011432491139819366)	#SAKNAS!	#SAKNAS!
cold-stressed	1	5110(0.011432491139819366)	#SAKNAS!	#SAKNAS!
cold-water	1	5110(0.011432491139819366)	#SAKNAS!	2548 (0.033)
coleomegilla	1	3437(0.02286498227963873)	2359 (0.034)	2548 (0.033)
coleopteran	1	5110(0.011432491139819366)	2359 (0.034)	#SAKNAS!
coleopteran	1	5110(0.011432491139819366)	2359 (0.034)	#SAKNAS!
colitis	1	5110(0.011432491139819366)	#SAKNAS!	2548 (0.033)
colius	1	5110(0.011432491139819366)	#SAKNAS!	2548 (0.033)
collaboration	1	2693(0.0342974734194581)	2359 (0.034)	2548 (0.033)
collagen	1	5110(0.011432491139819366)	#SAKNAS!	#SAKNAS!
collapsibility	1	5110(0.011432491139819366)	2359 (0.034)	#SAKNAS!
collapsibility	1	5110(0.011432491139819366)	2359 (0.034)	#SAKNAS!
collar	1	5110(0.011432491139819366)	2359 (0.034)	#SAKNAS!
collar	1	5110(0.011432491139819366)	2359 (0.034)	#SAKNAS!
collection	1	5110(0.011432491139819366)	#SAKNAS!	2548 (0.033)
collector-inductively	1	5110(0.011432491139819366)	#SAKNAS!	2548 (0.033)
collembola	1	5110(0.011432491139819366)	2359 (0.034)	#SAKNAS!
collembola	1	5110(0.011432491139819366)	2359 (0.034)	#SAKNAS!
collembolan	1	5110(0.011432491139819366)	#SAKNAS!	#SAKNAS!
collider	1	5110(0.011432491139819366)	#SAKNAS!	2548 (0.033)
collier	1	5110(0.011432491139819366)	2359 (0.034)	#SAKNAS!
collier	1	5110(0.011432491139819366)	2359 (0.034)	#SAKNAS!
collins	1	5110(0.011432491139819366)	#SAKNAS!	#SAKNAS!
collis-nissen	1	5110(0.011432491139819366)	#SAKNAS!	2548 (0.033)
co-localized	1	5110(0.011432491139819366)	#SAKNAS!	#SAKNAS!
colocalizes	1	5110(0.011432491139819366)	#SAKNAS!	#SAKNAS!
colonies	1	1894(0.05716245569909683)	2359 (0.034)	2548 (0.033)
colony-stimulating	1	5110(0.011432491139819366)	2359 (0.034)	#SAKNAS!
colony-stimulating	1	5110(0.011432491139819366)	2359 (0.034)	#SAKNAS!
colorado	1	3437(0.02286498227963873)	2359 (0.034)	2548 (0.033)
colorful	1	5110(0.011432491139819366)	#SAKNAS!	#SAKNAS!
colour-contrast	1	5110(0.011432491139819366)	#SAKNAS!	#SAKNAS!
colourimetric	1	5110(0.011432491139819366)	#SAKNAS!	2548 (0.033)
columbia	1	5110(0.011432491139819366)	2359 (0.034)	#SAKNAS!
columbia	1	5110(0.011432491139819366)	2359 (0.034)	#SAKNAS!

combined-modality	1	5110(0.011432491139819366)	2359 (0.034)	#SAKNAS!
combined-modality	1	5110(0.011432491139819366)	2359 (0.034)	#SAKNAS!
come	1	3437(0.02286498227963873)	2359 (0.034)	2548 (0.033)
comfort	1	1485(0.08002743797873557)	2359 (0.034)	816 (0.16)
comfortability	1	5110(0.011432491139819366)	#SAKNAS!	2548 (0.033)
comfortable	1	5110(0.011432491139819366)	#SAKNAS!	2548 (0.033)
comfort-focused	1	5110(0.011432491139819366)	#SAKNAS!	#SAKNAS!
co-mimics	1	5110(0.011432491139819366)	#SAKNAS!	#SAKNAS!
commercially	1	5110(0.011432491139819366)	#SAKNAS!	2548 (0.033)
commercially-prepared	1	5110(0.011432491139819366)	#SAKNAS!	2548 (0.033)
commercials	1	5110(0.011432491139819366)	2359 (0.034)	#SAKNAS!
commercials	1	5110(0.011432491139819366)	2359 (0.034)	#SAKNAS!
commission	1	5110(0.011432491139819366)	#SAKNAS!	2548 (0.033)
commoner	1	5110(0.011432491139819366)	2359 (0.034)	#SAKNAS!
commoner	1	5110(0.011432491139819366)	2359 (0.034)	#SAKNAS!
commonly	1	5110(0.011432491139819366)	#SAKNAS!	2548 (0.033)
communal	1	5110(0.011432491139819366)	2359 (0.034)	#SAKNAS!
communal	1	5110(0.011432491139819366)	2359 (0.034)	#SAKNAS!
communicable	1	5110(0.011432491139819366)	#SAKNAS!	2548 (0.033)
community-hospital	1	5110(0.011432491139819366)	2359 (0.034)	#SAKNAS!
community-hospital	1	5110(0.011432491139819366)	2359 (0.034)	#SAKNAS!
community-supported	1	5110(0.011432491139819366)	#SAKNAS!	#SAKNAS!
comorbid	1	2693(0.0342974734194581)	2359 (0.034)	#SAKNAS!
co-morbidities	1	5110(0.011432491139819366)	2359 (0.034)	#SAKNAS!
co-morbidities	1	5110(0.011432491139819366)	2359 (0.034)	#SAKNAS!
companion	1	3437(0.02286498227963873)	2359 (0.034)	2548 (0.033)
companions	1	5110(0.011432491139819366)	#SAKNAS!	#SAKNAS!
company	1	5110(0.011432491139819366)	#SAKNAS!	#SAKNAS!
compelling	1	5110(0.011432491139819366)	#SAKNAS!	2548 (0.033)
compensates	1	5110(0.011432491139819366)	2359 (0.034)	#SAKNAS!
compensates	1	5110(0.011432491139819366)	2359 (0.034)	#SAKNAS!
compensating	1	5110(0.011432491139819366)	2359 (0.034)	#SAKNAS!
compensating	1	5110(0.011432491139819366)	2359 (0.034)	#SAKNAS!
competency	1	5110(0.011432491139819366)	#SAKNAS!	2548 (0.033)
competent	1	5110(0.011432491139819366)	#SAKNAS!	2548 (0.033)
competitive	1	2214(0.04572996455927746)	2359 (0.034)	2548 (0.033)
competitively	1	5110(0.011432491139819366)	2359 (0.034)	#SAKNAS!
competitively	1	5110(0.011432491139819366)	2359 (0.034)	#SAKNAS!
competitors	1	5110(0.011432491139819366)	#SAKNAS!	#SAKNAS!
complaining	1	5110(0.011432491139819366)	#SAKNAS!	2548 (0.033)
complaints	1	2214(0.04572996455927746)	2359 (0.034)	2548 (0.033)
completely	1	5110(0.011432491139819366)	#SAKNAS!	#SAKNAS!
completing	1	5110(0.011432491139819366)	2359 (0.034)	#SAKNAS!

completing	1	5110(0.011432491139819366)	2359 (0.034)	#SAKNAS!
completion	1	3437(0.02286498227963873)	2359 (0.034)	2548 (0.033)
complexing	1	5110(0.011432491139819366)	2359 (0.034)	#SAKNAS!
complexing	1	5110(0.011432491139819366)	2359 (0.034)	#SAKNAS!
complexity	1	2214(0.04572996455927746)	2359 (0.034)	2548 (0.033)
compliant	1	5110(0.011432491139819366)	#SAKNAS!	#SAKNAS!
complimentary	1	5110(0.011432491139819366)	#SAKNAS!	2548 (0.033)
component-specific	1	5110(0.011432491139819366)	#SAKNAS!	2548 (0.033)
composite	1	1894(0.05716245569909683)	2359 (0.034)	#SAKNAS!
composites	1	5110(0.011432491139819366)	#SAKNAS!	#SAKNAS!
composites-based	1	5110(0.011432491139819366)	#SAKNAS!	#SAKNAS!
compositions	1	3437(0.02286498227963873)	2359 (0.034)	#SAKNAS!
compounds	1	1219(0.1028924202583743)	2359 (0.034)	1668 (0.066)
comprising	1	5110(0.011432491139819366)	#SAKNAS!	#SAKNAS!
compromise	1	5110(0.011432491139819366)	#SAKNAS!	2548 (0.033)
computational	1	3437(0.02286498227963873)	2359 (0.034)	#SAKNAS!
comunitaria	1	5110(0.011432491139819366)	#SAKNAS!	2548 (0.033)
concentrate	1	1111(0.11432491139819366)	2359 (0.034)	816 (0.16)
concepcion	1	5110(0.011432491139819366)	2359 (0.034)	#SAKNAS!
concepcion	1	5110(0.011432491139819366)	2359 (0.034)	#SAKNAS!
conception	1	5110(0.011432491139819366)	2359 (0.034)	#SAKNAS!
conception	1	5110(0.011432491139819366)	2359 (0.034)	#SAKNAS!
concepts	1	3437(0.02286498227963873)	2359 (0.034)	#SAKNAS!
conceptual	1	5110(0.011432491139819366)	#SAKNAS!	2548 (0.033)
conch	1	5110(0.011432491139819366)	2359 (0.034)	#SAKNAS!
conch	1	5110(0.011432491139819366)	2359 (0.034)	#SAKNAS!
concomitant	1	1894(0.05716245569909683)	2359 (0.034)	1668 (0.066)
concomitants	1	5110(0.011432491139819366)	2359 (0.034)	#SAKNAS!
concomitants	1	5110(0.011432491139819366)	2359 (0.034)	#SAKNAS!
concordance	1	5110(0.011432491139819366)	#SAKNAS!	#SAKNAS!
concordant	1	5110(0.011432491139819366)	#SAKNAS!	2548 (0.033)
concurrently	1	5110(0.011432491139819366)	#SAKNAS!	#SAKNAS!
conditioning	1	3437(0.02286498227963873)	2359 (0.034)	2548 (0.033)
conductance	1	5110(0.011432491139819366)	2359 (0.034)	#SAKNAS!
conductance	1	5110(0.011432491139819366)	2359 (0.034)	#SAKNAS!
conductive	1	5110(0.011432491139819366)	#SAKNAS!	#SAKNAS!
conduit	1	2214(0.04572996455927746)	2359 (0.034)	1668 (0.066)
confectioneries	1	5110(0.011432491139819366)	#SAKNAS!	#SAKNAS!
confectionery	1	3437(0.02286498227963873)	2359 (0.034)	2548 (0.033)
configurations	1	5110(0.011432491139819366)	#SAKNAS!	#SAKNAS!
confirm	1	5110(0.011432491139819366)	#SAKNAS!	2548 (0.033)
confirmatory	1	3437(0.02286498227963873)	2359 (0.034)	#SAKNAS!
confirming	1	5110(0.011432491139819366)	#SAKNAS!	2548 (0.033)

confirms	1	5110(0.011432491139819366)	#SAKNAS!	#SAKNAS!
conflict	1	5110(0.011432491139819366)	#SAKNAS!	#SAKNAS!
conform	1	5110(0.011432491139819366)	#SAKNAS!	2548 (0.033)
confounders	1	5110(0.011432491139819366)	#SAKNAS!	2548 (0.033)
confusum	1	5110(0.011432491139819366)	2359 (0.034)	#SAKNAS!
confusum	1	5110(0.011432491139819366)	2359 (0.034)	#SAKNAS!
congestive	1	3437(0.02286498227963873)	2359 (0.034)	2548 (0.033)
congregate	1	5110(0.011432491139819366)	#SAKNAS!	#SAKNAS!
congress	1	5110(0.011432491139819366)	#SAKNAS!	#SAKNAS!
conifers	1	5110(0.011432491139819366)	#SAKNAS!	2548 (0.033)
conjunction	1	5110(0.011432491139819366)	#SAKNAS!	#SAKNAS!
connection	1	5110(0.011432491139819366)	#SAKNAS!	2548 (0.033)
connections	1	5110(0.011432491139819366)	#SAKNAS!	#SAKNAS!
connective	1	5110(0.011432491139819366)	2359 (0.034)	#SAKNAS!
connective	1	5110(0.011432491139819366)	2359 (0.034)	#SAKNAS!
connexin	1	5110(0.011432491139819366)	#SAKNAS!	2548 (0.033)
conotrachelus	1	5110(0.011432491139819366)	2359 (0.034)	#SAKNAS!
conotrachelus	1	5110(0.011432491139819366)	2359 (0.034)	#SAKNAS!
consciousness	1	5110(0.011432491139819366)	#SAKNAS!	#SAKNAS!
conscripts	1	5110(0.011432491139819366)	#SAKNAS!	2548 (0.033)
conservation	1	2214(0.04572996455927746)	2359 (0.034)	1668 (0.066)
consideration	1	5110(0.011432491139819366)	2359 (0.034)	#SAKNAS!
consideration	1	5110(0.011432491139819366)	2359 (0.034)	#SAKNAS!
considering	1	2693(0.0342974734194581)	2359 (0.034)	2548 (0.033)
consistencies	1	5110(0.011432491139819366)	#SAKNAS!	2548 (0.033)
consistent	1	5110(0.011432491139819366)	2359 (0.034)	#SAKNAS!
consistent	1	5110(0.011432491139819366)	2359 (0.034)	#SAKNAS!
consistometer	1	5110(0.011432491139819366)	#SAKNAS!	2548 (0.033)
consortium	1	5110(0.011432491139819366)	#SAKNAS!	2548 (0.033)
conspecific	1	3437(0.02286498227963873)	2359 (0.034)	2548 (0.033)
constellatus	1	5110(0.011432491139819366)	#SAKNAS!	#SAKNAS!
constipation	1	5110(0.011432491139819366)	#SAKNAS!	2548 (0.033)
constituent	1	5110(0.011432491139819366)	#SAKNAS!	2548 (0.033)
constituents	1	3437(0.02286498227963873)	2359 (0.034)	2548 (0.033)
constitution	1	5110(0.011432491139819366)	#SAKNAS!	#SAKNAS!
constitutional	1	5110(0.011432491139819366)	#SAKNAS!	2548 (0.033)
constitutionally	1	5110(0.011432491139819366)	2359 (0.034)	#SAKNAS!
constitutionally	1	5110(0.011432491139819366)	2359 (0.034)	#SAKNAS!
constraining	1	5110(0.011432491139819366)	#SAKNAS!	#SAKNAS!
constrains	1	5110(0.011432491139819366)	#SAKNAS!	2548 (0.033)
constraint	1	5110(0.011432491139819366)	2359 (0.034)	#SAKNAS!
constraint	1	5110(0.011432491139819366)	2359 (0.034)	#SAKNAS!
constriction	1	5110(0.011432491139819366)	2359 (0.034)	#SAKNAS!

constriction	1	5110(0.011432491139819366)	2359 (0.034)	#SAKNAS!
constrictotermes	1	5110(0.011432491139819366)	#SAKNAS!	#SAKNAS!
construct	1	1678(0.0685949468389162)	2359 (0.034)	1233 (0.1)
construction	1	5110(0.011432491139819366)	#SAKNAS!	#SAKNAS!
consult	1	5110(0.011432491139819366)	#SAKNAS!	2548 (0.033)
consultants	1	5110(0.011432491139819366)	2359 (0.034)	#SAKNAS!
consultants	1	5110(0.011432491139819366)	2359 (0.034)	#SAKNAS!
consultation	1	5110(0.011432491139819366)	2359 (0.034)	#SAKNAS!
consultation	1	5110(0.011432491139819366)	2359 (0.034)	#SAKNAS!
consumer-demand	1	5110(0.011432491139819366)	#SAKNAS!	2548 (0.033)
consumers	1	3437(0.02286498227963873)	2359 (0.034)	2548 (0.033)
contact	1	5110(0.011432491139819366)	#SAKNAS!	2548 (0.033)
contacts	1	5110(0.011432491139819366)	#SAKNAS!	2548 (0.033)
contains	1	5110(0.011432491139819366)	2359 (0.034)	#SAKNAS!
contains	1	5110(0.011432491139819366)	2359 (0.034)	#SAKNAS!
contaminant	1	3437(0.02286498227963873)	2359 (0.034)	2548 (0.033)
contaminants	1	3437(0.02286498227963873)	2359 (0.034)	2548 (0.033)
contemporary	1	2693(0.0342974734194581)	2359 (0.034)	1668 (0.066)
contest	1	5110(0.011432491139819366)	#SAKNAS!	#SAKNAS!
contextual	1	5110(0.011432491139819366)	2359 (0.034)	#SAKNAS!
contextual	1	5110(0.011432491139819366)	2359 (0.034)	#SAKNAS!
continent	1	5110(0.011432491139819366)	#SAKNAS!	2548 (0.033)
contingent	1	5110(0.011432491139819366)	#SAKNAS!	#SAKNAS!
continuously	1	3437(0.02286498227963873)	2359 (0.034)	#SAKNAS!
contortus	1	5110(0.011432491139819366)	#SAKNAS!	2548 (0.033)
contra	1	5110(0.011432491139819366)	#SAKNAS!	2548 (0.033)
contraceptives	1	5110(0.011432491139819366)	2359 (0.034)	#SAKNAS!
contraceptives	1	5110(0.011432491139819366)	2359 (0.034)	#SAKNAS!
contract	1	5110(0.011432491139819366)	#SAKNAS!	2548 (0.033)
contraindication	1	3437(0.02286498227963873)	2359 (0.034)	2548 (0.033)
contralateral	1	5110(0.011432491139819366)	#SAKNAS!	#SAKNAS!
contrast	1	5110(0.011432491139819366)	2359 (0.034)	#SAKNAS!
contrast	1	5110(0.011432491139819366)	2359 (0.034)	#SAKNAS!
contributes	1	3437(0.02286498227963873)	2359 (0.034)	#SAKNAS!
contributing	1	2693(0.0342974734194581)	2359 (0.034)	2548 (0.033)
contributor	1	5110(0.011432491139819366)	#SAKNAS!	#SAKNAS!
controlling	1	2693(0.0342974734194581)	2359 (0.034)	1668 (0.066)
conut	1	5110(0.011432491139819366)	#SAKNAS!	#SAKNAS!
convalescence	1	5110(0.011432491139819366)	2359 (0.034)	#SAKNAS!
convalescence	1	5110(0.011432491139819366)	2359 (0.034)	#SAKNAS!
convalescent	1	1335(0.09145992911855493)	2359 (0.034)	1233 (0.1)
convergent	1	5110(0.011432491139819366)	#SAKNAS!	#SAKNAS!
conversation	1	5110(0.011432491139819366)	#SAKNAS!	#SAKNAS!

conversations	1	5110(0.011432491139819366)	#SAKNAS!	#SAKNAS!
conversion	1	1678(0.0685949468389162)	2359 (0.034)	1668 (0.066)
conversional	1	5110(0.011432491139819366)	#SAKNAS!	#SAKNAS!
conversions	1	5110(0.011432491139819366)	#SAKNAS!	#SAKNAS!
co-occurring	1	5110(0.011432491139819366)	#SAKNAS!	#SAKNAS!
cooperation	1	3437(0.02286498227963873)	2359 (0.034)	2548 (0.033)
coordinate	1	5110(0.011432491139819366)	#SAKNAS!	2548 (0.033)
coots	1	5110(0.011432491139819366)	#SAKNAS!	2548 (0.033)
copd	1	2214(0.04572996455927746)	2359 (0.034)	#SAKNAS!
cope	1	2693(0.0342974734194581)	2359 (0.034)	2548 (0.033)
copenhagen	1	5110(0.011432491139819366)	2359 (0.034)	#SAKNAS!
copenhagen	1	5110(0.011432491139819366)	2359 (0.034)	#SAKNAS!
coping	1	2214(0.04572996455927746)	2359 (0.034)	1668 (0.066)
coprophagic	1	5110(0.011432491139819366)	2359 (0.034)	#SAKNAS!
coprophagic	1	5110(0.011432491139819366)	2359 (0.034)	#SAKNAS!
coprophagy	1	3437(0.02286498227963873)	2359 (0.034)	2548 (0.033)
copula	1	5110(0.011432491139819366)	#SAKNAS!	#SAKNAS!
coral	1	1894(0.05716245569909683)	2359 (0.034)	999 (0.13)
corallivorous	1	5110(0.011432491139819366)	2359 (0.034)	#SAKNAS!
corallivorous	1	5110(0.011432491139819366)	2359 (0.034)	#SAKNAS!
cord	1	1894(0.05716245569909683)	2359 (0.034)	2548 (0.033)
co-receptor	1	5110(0.011432491139819366)	#SAKNAS!	2548 (0.033)
coriacea	1	5110(0.011432491139819366)	2359 (0.034)	#SAKNAS!
coriacea	1	5110(0.011432491139819366)	2359 (0.034)	#SAKNAS!
corn	1	896(0.14862238481765175)	2359 (0.034)	584 (0.23)
cornelia	1	5110(0.011432491139819366)	#SAKNAS!	#SAKNAS!
cornerstone	1	5110(0.011432491139819366)	#SAKNAS!	2548 (0.033)
corn-silage	1	5110(0.011432491139819366)	2359 (0.034)	#SAKNAS!
corn-silage	1	5110(0.011432491139819366)	2359 (0.034)	#SAKNAS!
corn-soy	1	5110(0.011432491139819366)	#SAKNAS!	2548 (0.033)
coronally	1	5110(0.011432491139819366)	2359 (0.034)	#SAKNAS!
coronally	1	5110(0.011432491139819366)	2359 (0.034)	#SAKNAS!
coronata	1	5110(0.011432491139819366)	#SAKNAS!	#SAKNAS!
coronatus	1	5110(0.011432491139819366)	2359 (0.034)	#SAKNAS!
coronatus	1	5110(0.011432491139819366)	2359 (0.034)	#SAKNAS!
corps	1	5110(0.011432491139819366)	#SAKNAS!	#SAKNAS!
correcting	1	5110(0.011432491139819366)	#SAKNAS!	#SAKNAS!
correction	1	3437(0.02286498227963873)	2359 (0.034)	2548 (0.033)
correlating	1	5110(0.011432491139819366)	#SAKNAS!	2548 (0.033)
correlational	1	5110(0.011432491139819366)	#SAKNAS!	#SAKNAS!
corresponding	1	5110(0.011432491139819366)	#SAKNAS!	2548 (0.033)
corriedale	1	5110(0.011432491139819366)	#SAKNAS!	2548 (0.033)
corrosive-induced	1	5110(0.011432491139819366)	2359 (0.034)	#SAKNAS!

corrosive-induced	1	5110(0.011432491139819366)	2359 (0.034)	#SAKNAS!
cortical	1	2214(0.04572996455927746)	2359 (0.034)	1668 (0.066)
corticotropin-releasing	1	5110(0.011432491139819366)	2359 (0.034)	#SAKNAS!
corticotropin-releasing	1	5110(0.011432491139819366)	2359 (0.034)	#SAKNAS!
cory	1	5110(0.011432491139819366)	2359 (0.034)	#SAKNAS!
cory	1	5110(0.011432491139819366)	2359 (0.034)	#SAKNAS!
coryphaena	1	5110(0.011432491139819366)	#SAKNAS!	2548 (0.033)
cosmioides	1	5110(0.011432491139819366)	#SAKNAS!	2548 (0.033)
costa	1	5110(0.011432491139819366)	#SAKNAS!	2548 (0.033)
cost-offset	1	5110(0.011432491139819366)	#SAKNAS!	#SAKNAS!
cost-of-living	1	5110(0.011432491139819366)	#SAKNAS!	#SAKNAS!
coton	1	5110(0.011432491139819366)	#SAKNAS!	#SAKNAS!
counsellors	1	5110(0.011432491139819366)	#SAKNAS!	2548 (0.033)
counselors	1	5110(0.011432491139819366)	#SAKNAS!	#SAKNAS!
counterparts	1	3437(0.02286498227963873)	2359 (0.034)	2548 (0.033)
counties	1	5110(0.011432491139819366)	2359 (0.034)	#SAKNAS!
counties	1	5110(0.011432491139819366)	2359 (0.034)	#SAKNAS!
counting	1	5110(0.011432491139819366)	#SAKNAS!	2548 (0.033)
county	1	2214(0.04572996455927746)	2359 (0.034)	1233 (0.1)
county-level	1	5110(0.011432491139819366)	#SAKNAS!	2548 (0.033)
courses	1	5110(0.011432491139819366)	2359 (0.034)	#SAKNAS!
courses	1	5110(0.011432491139819366)	2359 (0.034)	#SAKNAS!
courtship	1	5110(0.011432491139819366)	2359 (0.034)	#SAKNAS!
courtship	1	5110(0.011432491139819366)	2359 (0.034)	#SAKNAS!
coverage	1	1027(0.12575740253801304)	2359 (0.034)	694 (0.2)
covid-19-associated	1	5110(0.011432491139819366)	#SAKNAS!	#SAKNAS!
covid-19-induced	1	5110(0.011432491139819366)	#SAKNAS!	#SAKNAS!
coyoacan	1	5110(0.011432491139819366)	#SAKNAS!	#SAKNAS!
cpeo	1	5110(0.011432491139819366)	#SAKNAS!	2548 (0.033)
c-peptide	1	5110(0.011432491139819366)	#SAKNAS!	#SAKNAS!
cpi	1	5110(0.011432491139819366)	#SAKNAS!	#SAKNAS!
cpne7	1	5110(0.011432491139819366)	#SAKNAS!	#SAKNAS!
cpoe	1	5110(0.011432491139819366)	#SAKNAS!	2548 (0.033)
crack	1	5110(0.011432491139819366)	#SAKNAS!	#SAKNAS!
cranberries	1	5110(0.011432491139819366)	#SAKNAS!	2548 (0.033)
cranial	1	3437(0.02286498227963873)	2359 (0.034)	2548 (0.033)
craniocerebral	1	5110(0.011432491139819366)	#SAKNAS!	#SAKNAS!
craniofacial	1	5110(0.011432491139819366)	#SAKNAS!	#SAKNAS!
crassifolia	1	5110(0.011432491139819366)	2359 (0.034)	#SAKNAS!
crassifolia	1	5110(0.011432491139819366)	2359 (0.034)	#SAKNAS!
craving	1	5110(0.011432491139819366)	#SAKNAS!	2548 (0.033)
cravings	1	5110(0.011432491139819366)	#SAKNAS!	#SAKNAS!
crayfish	1	5110(0.011432491139819366)	2359 (0.034)	#SAKNAS!

crayfish	1	5110(0.011432491139819366)	2359 (0.034)	#SAKNAS!
creating	1	5110(0.011432491139819366)	2359 (0.034)	#SAKNAS!
creating	1	5110(0.011432491139819366)	2359 (0.034)	#SAKNAS!
creatinine	1	5110(0.011432491139819366)	#SAKNAS!	#SAKNAS!
creation	1	5110(0.011432491139819366)	#SAKNAS!	2548 (0.033)
creativity	1	5110(0.011432491139819366)	#SAKNAS!	#SAKNAS!
cree	1	5110(0.011432491139819366)	#SAKNAS!	2548 (0.033)
crenata	1	5110(0.011432491139819366)	2359 (0.034)	#SAKNAS!
crenata	1	5110(0.011432491139819366)	2359 (0.034)	#SAKNAS!
crested	1	5110(0.011432491139819366)	#SAKNAS!	2548 (0.033)
crf	1	5110(0.011432491139819366)	2359 (0.034)	#SAKNAS!
crf	1	5110(0.011432491139819366)	2359 (0.034)	#SAKNAS!
cribroelphidium	1	5110(0.011432491139819366)	#SAKNAS!	#SAKNAS!
cricohyoidoepiglottopexy	1	5110(0.011432491139819366)	2359 (0.034)	#SAKNAS!
cricohyoidoepiglottopexy	1	5110(0.011432491139819366)	2359 (0.034)	#SAKNAS!
crisis	1	5110(0.011432491139819366)	#SAKNAS!	#SAKNAS!
crisponi	1	5110(0.011432491139819366)	#SAKNAS!	#SAKNAS!
crispula	1	5110(0.011432491139819366)	2359 (0.034)	#SAKNAS!
crispula	1	5110(0.011432491139819366)	2359 (0.034)	#SAKNAS!
cristatus	1	3437(0.02286498227963873)	2359 (0.034)	#SAKNAS!
criteria	1	671(0.2057848405167486)	2359 (0.034)	694 (0.2)
croatia	1	5110(0.011432491139819366)	2359 (0.034)	#SAKNAS!
croatia	1	5110(0.011432491139819366)	2359 (0.034)	#SAKNAS!
crohn	1	5110(0.011432491139819366)	#SAKNAS!	2548 (0.033)
crop	1	3437(0.02286498227963873)	2359 (0.034)	2548 (0.033)
crops	1	5110(0.011432491139819366)	#SAKNAS!	2548 (0.033)
crosnieri	1	5110(0.011432491139819366)	#SAKNAS!	2548 (0.033)
crossbreeding	1	5110(0.011432491139819366)	#SAKNAS!	2548 (0.033)
cross-country	1	5110(0.011432491139819366)	#SAKNAS!	2548 (0.033)
cross-cultural	1	1678(0.0685949468389162)	2359 (0.034)	1668 (0.066)
crosse	1	5110(0.011432491139819366)	#SAKNAS!	2548 (0.033)
cross-examining	1	5110(0.011432491139819366)	#SAKNAS!	#SAKNAS!
cross-intestinal	1	5110(0.011432491139819366)	2359 (0.034)	#SAKNAS!
cross-intestinal	1	5110(0.011432491139819366)	2359 (0.034)	#SAKNAS!
crossover	1	2214(0.04572996455927746)	2359 (0.034)	2548 (0.033)
crosssectional	1	5110(0.011432491139819366)	#SAKNAS!	#SAKNAS!
cross-sucking	1	5110(0.011432491139819366)	2359 (0.034)	#SAKNAS!
cross-sucking	1	5110(0.011432491139819366)	2359 (0.034)	#SAKNAS!
cross-trait	1	5110(0.011432491139819366)	#SAKNAS!	#SAKNAS!
cruroplasty	1	3437(0.02286498227963873)	2359 (0.034)	#SAKNAS!
crushing	1	5110(0.011432491139819366)	#SAKNAS!	2548 (0.033)
cryotherapy	1	5110(0.011432491139819366)	#SAKNAS!	2548 (0.033)
crystalline	1	5110(0.011432491139819366)	#SAKNAS!	#SAKNAS!

csn1s1	1	5110(0.011432491139819366)	2359 (0.034)	#SAKNAS!
csn1s1	1	5110(0.011432491139819366)	2359 (0.034)	#SAKNAS!
ctenomys	1	3437(0.02286498227963873)	2359 (0.034)	2548 (0.033)
ctenopharyngodon	1	5110(0.011432491139819366)	#SAKNAS!	2548 (0.033)
c-terminal	1	5110(0.011432491139819366)	#SAKNAS!	2548 (0.033)
ct-guided	1	5110(0.011432491139819366)	2359 (0.034)	#SAKNAS!
ct-guided	1	5110(0.011432491139819366)	2359 (0.034)	#SAKNAS!
cu	1	2693(0.0342974734194581)	2359 (0.034)	2548 (0.033)
cuba	1	5110(0.011432491139819366)	2359 (0.034)	#SAKNAS!
cuba	1	5110(0.011432491139819366)	2359 (0.034)	#SAKNAS!
cucumaria	1	5110(0.011432491139819366)	#SAKNAS!	2548 (0.033)
cucumber	1	2693(0.0342974734194581)	2359 (0.034)	1668 (0.066)
cue	1	3437(0.02286498227963873)	2359 (0.034)	2548 (0.033)
culebras	1	5110(0.011432491139819366)	#SAKNAS!	#SAKNAS!
culex	1	5110(0.011432491139819366)	#SAKNAS!	2548 (0.033)
culicidae	1	3437(0.02286498227963873)	2359 (0.034)	2548 (0.033)
cull	1	5110(0.011432491139819366)	#SAKNAS!	#SAKNAS!
culpaeus	1	5110(0.011432491139819366)	2359 (0.034)	#SAKNAS!
culpaeus	1	5110(0.011432491139819366)	2359 (0.034)	#SAKNAS!
culprits	1	5110(0.011432491139819366)	#SAKNAS!	2548 (0.033)
cultivars	1	3437(0.02286498227963873)	2359 (0.034)	#SAKNAS!
culturally	1	5110(0.011432491139819366)	#SAKNAS!	#SAKNAS!
culture-independent	1	5110(0.011432491139819366)	2359 (0.034)	#SAKNAS!
culture-independent	1	5110(0.011432491139819366)	2359 (0.034)	#SAKNAS!
cuminum	1	5110(0.011432491139819366)	#SAKNAS!	#SAKNAS!
cumulative	1	5110(0.011432491139819366)	2359 (0.034)	#SAKNAS!
cumulative	1	5110(0.011432491139819366)	2359 (0.034)	#SAKNAS!
cuniculus	1	5110(0.011432491139819366)	2359 (0.034)	#SAKNAS!
cuniculus	1	5110(0.011432491139819366)	2359 (0.034)	#SAKNAS!
curculionidae	1	5110(0.011432491139819366)	2359 (0.034)	#SAKNAS!
curculionidae	1	5110(0.011432491139819366)	2359 (0.034)	#SAKNAS!
curcumin	1	5110(0.011432491139819366)	#SAKNAS!	#SAKNAS!
curdlan	1	5110(0.011432491139819366)	#SAKNAS!	#SAKNAS!
cure	1	5110(0.011432491139819366)	#SAKNAS!	2548 (0.033)
currently	1	5110(0.011432491139819366)	#SAKNAS!	#SAKNAS!
currents	1	5110(0.011432491139819366)	2359 (0.034)	#SAKNAS!
currents	1	5110(0.011432491139819366)	2359 (0.034)	#SAKNAS!
curricula	1	3437(0.02286498227963873)	2359 (0.034)	#SAKNAS!
curricular	1	5110(0.011432491139819366)	#SAKNAS!	2548 (0.033)
curriculum	1	2693(0.0342974734194581)	2359 (0.034)	#SAKNAS!
curry	1	5110(0.011432491139819366)	#SAKNAS!	#SAKNAS!
curtipennis	1	5110(0.011432491139819366)	#SAKNAS!	2548 (0.033)
curves	1	5110(0.011432491139819366)	2359 (0.034)	#SAKNAS!

curves	1	5110(0.011432491139819366)	2359 (0.034)	#SAKNAS!
customised	1	5110(0.011432491139819366)	#SAKNAS!	2548 (0.033)
customization	1	5110(0.011432491139819366)	#SAKNAS!	#SAKNAS!
cutoff	1	5110(0.011432491139819366)	#SAKNAS!	2548 (0.033)
cut-off	1	5110(0.011432491139819366)	#SAKNAS!	#SAKNAS!
cuts	1	5110(0.011432491139819366)	2359 (0.034)	#SAKNAS!
cuts	1	5110(0.011432491139819366)	2359 (0.034)	#SAKNAS!
cuttlefish	1	5110(0.011432491139819366)	2359 (0.034)	#SAKNAS!
cuttlefish	1	5110(0.011432491139819366)	2359 (0.034)	#SAKNAS!
cutworm	1	5110(0.011432491139819366)	2359 (0.034)	#SAKNAS!
cutworm	1	5110(0.011432491139819366)	2359 (0.034)	#SAKNAS!
cyanobacteria	1	5110(0.011432491139819366)	2359 (0.034)	#SAKNAS!
cyanobacteria	1	5110(0.011432491139819366)	2359 (0.034)	#SAKNAS!
cyanobacterial	1	5110(0.011432491139819366)	#SAKNAS!	2548 (0.033)
cyanogen	1	5110(0.011432491139819366)	2359 (0.034)	#SAKNAS!
cyanogen	1	5110(0.011432491139819366)	2359 (0.034)	#SAKNAS!
cyclase-activating	1	5110(0.011432491139819366)	#SAKNAS!	#SAKNAS!
cycles	1	2693(0.0342974734194581)	2359 (0.034)	1668 (0.066)
cyclosporine	1	5110(0.011432491139819366)	2359 (0.034)	#SAKNAS!
cyclosporine	1	5110(0.011432491139819366)	2359 (0.034)	#SAKNAS!
cyminum	1	5110(0.011432491139819366)	#SAKNAS!	#SAKNAS!
cynomolgus	1	5110(0.011432491139819366)	#SAKNAS!	#SAKNAS!
cyphergaster	1	5110(0.011432491139819366)	#SAKNAS!	#SAKNAS!
cypridopsis-vidua	1	5110(0.011432491139819366)	2359 (0.034)	#SAKNAS!
cypridopsis-vidua	1	5110(0.011432491139819366)	2359 (0.034)	#SAKNAS!
cyprinidae	1	5110(0.011432491139819366)	2359 (0.034)	#SAKNAS!
cyprinidae	1	5110(0.011432491139819366)	2359 (0.034)	#SAKNAS!
cypriniformes	1	5110(0.011432491139819366)	2359 (0.034)	#SAKNAS!
cypriniformes	1	5110(0.011432491139819366)	2359 (0.034)	#SAKNAS!
cyprinus	1	5110(0.011432491139819366)	2359 (0.034)	#SAKNAS!
cyprinus	1	5110(0.011432491139819366)	2359 (0.034)	#SAKNAS!
cyrtonema	1	5110(0.011432491139819366)	#SAKNAS!	#SAKNAS!
cystatin	1	5110(0.011432491139819366)	#SAKNAS!	#SAKNAS!
cystic	1	1485(0.08002743797873557)	2359 (0.034)	#SAKNAS!
cystic-fibrosis	1	5110(0.011432491139819366)	2359 (0.034)	#SAKNAS!
cystic-fibrosis	1	5110(0.011432491139819366)	2359 (0.034)	#SAKNAS!
cystinosis	1	5110(0.011432491139819366)	2359 (0.034)	#SAKNAS!
cystinosis	1	5110(0.011432491139819366)	2359 (0.034)	#SAKNAS!
cytokinin-mediated	1	5110(0.011432491139819366)	2359 (0.034)	#SAKNAS!
cytokinin-mediated	1	5110(0.011432491139819366)	2359 (0.034)	#SAKNAS!
cytomegalovirus	1	5110(0.011432491139819366)	#SAKNAS!	#SAKNAS!
czech	1	3437(0.02286498227963873)	2359 (0.034)	2548 (0.033)
d2	1	2693(0.0342974734194581)	2359 (0.034)	2548 (0.033)

da	1	5110(0.011432491139819366)	#SAKNAS!	#SAKNAS!
dairy-products	1	5110(0.011432491139819366)	2359 (0.034)	#SAKNAS!
dairy-products	1	5110(0.011432491139819366)	2359 (0.034)	#SAKNAS!
damages	1	5110(0.011432491139819366)	#SAKNAS!	#SAKNAS!
dams	1	5110(0.011432491139819366)	#SAKNAS!	2548 (0.033)
damselfishes	1	5110(0.011432491139819366)	#SAKNAS!	2548 (0.033)
danazol	1	5110(0.011432491139819366)	2359 (0.034)	#SAKNAS!
danazol	1	5110(0.011432491139819366)	2359 (0.034)	#SAKNAS!
dancers	1	5110(0.011432491139819366)	2359 (0.034)	#SAKNAS!
dancers	1	5110(0.011432491139819366)	2359 (0.034)	#SAKNAS!
danes	1	3437(0.02286498227963873)	2359 (0.034)	2548 (0.033)
danio	1	5110(0.011432491139819366)	2359 (0.034)	#SAKNAS!
danio	1	5110(0.011432491139819366)	2359 (0.034)	#SAKNAS!
danish	1	2214(0.04572996455927746)	2359 (0.034)	1233 (0.1)
daphnia-ambigua	1	5110(0.011432491139819366)	2359 (0.034)	#SAKNAS!
daphnia-ambigua	1	5110(0.011432491139819366)	2359 (0.034)	#SAKNAS!
dar	1	5110(0.011432491139819366)	2359 (0.034)	#SAKNAS!
dar	1	5110(0.011432491139819366)	2359 (0.034)	#SAKNAS!
darkness	1	5110(0.011432491139819366)	#SAKNAS!	#SAKNAS!
darwini	1	5110(0.011432491139819366)	2359 (0.034)	#SAKNAS!
darwini	1	5110(0.011432491139819366)	2359 (0.034)	#SAKNAS!
dash	1	5110(0.011432491139819366)	#SAKNAS!	2548 (0.033)
dashtestan	1	5110(0.011432491139819366)	2359 (0.034)	#SAKNAS!
dashtestan	1	5110(0.011432491139819366)	2359 (0.034)	#SAKNAS!
dassie-rat	1	5110(0.011432491139819366)	2359 (0.034)	#SAKNAS!
dassie-rat	1	5110(0.011432491139819366)	2359 (0.034)	#SAKNAS!
data2010	1	5110(0.011432491139819366)	#SAKNAS!	2548 (0.033)
database	1	595(0.22864982279638732)	2359 (0.034)	816 (0.16)
data-base	1	5110(0.011432491139819366)	2359 (0.034)	#SAKNAS!
data-base	1	5110(0.011432491139819366)	2359 (0.034)	#SAKNAS!
data-driven	1	5110(0.011432491139819366)	#SAKNAS!	#SAKNAS!
date	1	5110(0.011432491139819366)	#SAKNAS!	#SAKNAS!
daubentonia-madagascariensis	1	5110(0.011432491139819366)	2359 (0.034)	#SAKNAS!
daubentonia-madagascariensis	1	5110(0.011432491139819366)	2359 (0.034)	#SAKNAS!
daughters	1	5110(0.011432491139819366)	2359 (0.034)	#SAKNAS!
daughters	1	5110(0.011432491139819366)	2359 (0.034)	#SAKNAS!
davidi	1	5110(0.011432491139819366)	#SAKNAS!	2548 (0.033)
dawley-national	1	5110(0.011432491139819366)	#SAKNAS!	#SAKNAS!
daycare	1	3437(0.02286498227963873)	2359 (0.034)	#SAKNAS!
day-care	1	5110(0.011432491139819366)	2359 (0.034)	#SAKNAS!
day-care	1	5110(0.011432491139819366)	2359 (0.034)	#SAKNAS!
daylong	1	5110(0.011432491139819366)	#SAKNAS!	#SAKNAS!
days	1	1485(0.08002743797873557)	2359 (0.034)	1668 (0.066)

daytime-only	1	5110(0.011432491139819366)	#SAKNAS!	2548 (0.033)
d-binding	1	5110(0.011432491139819366)	#SAKNAS!	2548 (0.033)
dds	1	5110(0.011432491139819366)	#SAKNAS!	#SAKNAS!
debris	1	5110(0.011432491139819366)	#SAKNAS!	2548 (0.033)
decarboxylase	1	5110(0.011432491139819366)	2359 (0.034)	#SAKNAS!
decarboxylase	1	5110(0.011432491139819366)	2359 (0.034)	#SAKNAS!
deceleration	1	5110(0.011432491139819366)	2359 (0.034)	#SAKNAS!
deceleration	1	5110(0.011432491139819366)	2359 (0.034)	#SAKNAS!
deciphering	1	3437(0.02286498227963873)	2359 (0.034)	#SAKNAS!
decision-makers	1	5110(0.011432491139819366)	2359 (0.034)	#SAKNAS!
decision-makers	1	5110(0.011432491139819366)	2359 (0.034)	#SAKNAS!
decussatus	1	5110(0.011432491139819366)	2359 (0.034)	#SAKNAS!
decussatus	1	5110(0.011432491139819366)	2359 (0.034)	#SAKNAS!
deep-sea	1	3437(0.02286498227963873)	2359 (0.034)	2548 (0.033)
de-escalated	1	5110(0.011432491139819366)	#SAKNAS!	2548 (0.033)
de-escalation	1	5110(0.011432491139819366)	#SAKNAS!	2548 (0.033)
defb1	1	5110(0.011432491139819366)	#SAKNAS!	#SAKNAS!
defecation	1	5110(0.011432491139819366)	2359 (0.034)	#SAKNAS!
defecation	1	5110(0.011432491139819366)	2359 (0.034)	#SAKNAS!
defence	1	5110(0.011432491139819366)	2359 (0.034)	#SAKNAS!
defence	1	5110(0.011432491139819366)	2359 (0.034)	#SAKNAS!
defences	1	3437(0.02286498227963873)	2359 (0.034)	2548 (0.033)
defense	1	1894(0.05716245569909683)	2359 (0.034)	1233 (0.1)
defenses	1	2693(0.0342974734194581)	2359 (0.034)	2548 (0.033)
defensive	1	5110(0.011432491139819366)	#SAKNAS!	2548 (0.033)
definable	1	5110(0.011432491139819366)	2359 (0.034)	#SAKNAS!
definable	1	5110(0.011432491139819366)	2359 (0.034)	#SAKNAS!
definitive	1	1485(0.08002743797873557)	2359 (0.034)	1233 (0.1)
deformities	1	5110(0.011432491139819366)	2359 (0.034)	#SAKNAS!
deformities	1	5110(0.011432491139819366)	2359 (0.034)	#SAKNAS!
defrosted	1	5110(0.011432491139819366)	#SAKNAS!	#SAKNAS!
degeneration	1	2214(0.04572996455927746)	2359 (0.034)	1668 (0.066)
degenerative	1	5110(0.011432491139819366)	2359 (0.034)	#SAKNAS!
degenerative	1	5110(0.011432491139819366)	2359 (0.034)	#SAKNAS!
deglutitive	1	5110(0.011432491139819366)	#SAKNAS!	#SAKNAS!
degree	1	2214(0.04572996455927746)	2359 (0.034)	2548 (0.033)
dehydration	1	2693(0.0342974734194581)	2359 (0.034)	#SAKNAS!
delavayi	1	5110(0.011432491139819366)	#SAKNAS!	#SAKNAS!
delays	1	5110(0.011432491139819366)	#SAKNAS!	#SAKNAS!
deletions	1	5110(0.011432491139819366)	#SAKNAS!	2548 (0.033)
delhi	1	5110(0.011432491139819366)	#SAKNAS!	2548 (0.033)
deliberations	1	5110(0.011432491139819366)	#SAKNAS!	2548 (0.033)
delta	1	5110(0.011432491139819366)	2359 (0.034)	#SAKNAS!

delta	1	5110(0.011432491139819366)	2359 (0.034)	#SAKNAS!
demand-feeding	1	3437(0.02286498227963873)	2359 (0.034)	2548 (0.033)
demeester	1	5110(0.011432491139819366)	#SAKNAS!	#SAKNAS!
dementia-an	1	5110(0.011432491139819366)	#SAKNAS!	#SAKNAS!
demographics	1	5110(0.011432491139819366)	2359 (0.034)	#SAKNAS!
demographics	1	5110(0.011432491139819366)	2359 (0.034)	#SAKNAS!
demonstrates	1	5110(0.011432491139819366)	#SAKNAS!	2548 (0.033)
demonstrating	1	5110(0.011432491139819366)	#SAKNAS!	2548 (0.033)
demyelination	1	5110(0.011432491139819366)	#SAKNAS!	#SAKNAS!
dendrobatidae	1	5110(0.011432491139819366)	#SAKNAS!	#SAKNAS!
denervation	1	5110(0.011432491139819366)	#SAKNAS!	#SAKNAS!
dengue	1	5110(0.011432491139819366)	#SAKNAS!	#SAKNAS!
denies	1	5110(0.011432491139819366)	2359 (0.034)	#SAKNAS!
denies	1	5110(0.011432491139819366)	2359 (0.034)	#SAKNAS!
denosumab-related	1	5110(0.011432491139819366)	#SAKNAS!	2548 (0.033)
dense	1	5110(0.011432491139819366)	2359 (0.034)	#SAKNAS!
dense	1	5110(0.011432491139819366)	2359 (0.034)	#SAKNAS!
densities	1	5110(0.011432491139819366)	2359 (0.034)	#SAKNAS!
densities	1	5110(0.011432491139819366)	2359 (0.034)	#SAKNAS!
density-dependent	1	5110(0.011432491139819366)	#SAKNAS!	2548 (0.033)
dentaisez	1	5110(0.011432491139819366)	#SAKNAS!	#SAKNAS!
dental-health	1	5110(0.011432491139819366)	2359 (0.034)	#SAKNAS!
dental-health	1	5110(0.011432491139819366)	2359 (0.034)	#SAKNAS!
dentally	1	5110(0.011432491139819366)	#SAKNAS!	2548 (0.033)
denticola	1	3437(0.02286498227963873)	2359 (0.034)	2548 (0.033)
dentisits	1	5110(0.011432491139819366)	#SAKNAS!	#SAKNAS!
dento-alveolar	1	5110(0.011432491139819366)	2359 (0.034)	#SAKNAS!
dento-alveolar	1	5110(0.011432491139819366)	2359 (0.034)	#SAKNAS!
dentognathic	1	5110(0.011432491139819366)	#SAKNAS!	2548 (0.033)
denture-wearing	1	5110(0.011432491139819366)	#SAKNAS!	2548 (0.033)
deoxynivalenol	1	3437(0.02286498227963873)	2359 (0.034)	2548 (0.033)
department	1	1678(0.0685949468389162)	2359 (0.034)	1233 (0.1)
depend	1	3437(0.02286498227963873)	2359 (0.034)	2548 (0.033)
dependency	1	2693(0.0342974734194581)	2359 (0.034)	2548 (0.033)
deployment	1	5110(0.011432491139819366)	#SAKNAS!	2548 (0.033)
deposit	1	3437(0.02286498227963873)	2359 (0.034)	2548 (0.033)
deposit-feeder	1	5110(0.011432491139819366)	2359 (0.034)	#SAKNAS!
deposit-feeder	1	5110(0.011432491139819366)	2359 (0.034)	#SAKNAS!
deposition	1	5110(0.011432491139819366)	#SAKNAS!	2548 (0.033)
depot	1	5110(0.011432491139819366)	2359 (0.034)	#SAKNAS!
depot	1	5110(0.011432491139819366)	2359 (0.034)	#SAKNAS!
depress	1	5110(0.011432491139819366)	2359 (0.034)	#SAKNAS!
depress	1	5110(0.011432491139819366)	2359 (0.034)	#SAKNAS!

depression	1 595(0.22864982279638732)	2359 (0.034)	461 (0.3)
der	1 5110(0.011432491139819366)	2359 (0.034)	#SAKNAS!
der	1 5110(0.011432491139819366)	2359 (0.034)	#SAKNAS!
derivatives	1 5110(0.011432491139819366)	2359 (0.034)	#SAKNAS!
derivatives	1 5110(0.011432491139819366)	2359 (0.034)	#SAKNAS!
dermal	1 3437(0.02286498227963873)	2359 (0.034)	2548 (0.033)
dermis	1 5110(0.011432491139819366)	2359 (0.034)	#SAKNAS!
dermis	1 5110(0.011432491139819366)	2359 (0.034)	#SAKNAS!
deroceras	1 5110(0.011432491139819366)	2359 (0.034)	#SAKNAS!
deroceras	1 5110(0.011432491139819366)	2359 (0.034)	#SAKNAS!
des	1 5110(0.011432491139819366)	2359 (0.034)	#SAKNAS!
des	1 5110(0.011432491139819366)	2359 (0.034)	#SAKNAS!
descending	1 5110(0.011432491139819366)	#SAKNAS!	2548 (0.033)
describing	1 5110(0.011432491139819366)	#SAKNAS!	2548 (0.033)
desipramine	1 5110(0.011432491139819366)	2359 (0.034)	#SAKNAS!
desipramine	1 5110(0.011432491139819366)	2359 (0.034)	#SAKNAS!
desmarest	1 5110(0.011432491139819366)	2359 (0.034)	#SAKNAS!
desmarest	1 5110(0.011432491139819366)	2359 (0.034)	#SAKNAS!
desmarestii	1 5110(0.011432491139819366)	#SAKNAS!	2548 (0.033)
despite	1 5110(0.011432491139819366)	#SAKNAS!	2548 (0.033)
desserts	1 5110(0.011432491139819366)	2359 (0.034)	#SAKNAS!
desserts	1 5110(0.011432491139819366)	2359 (0.034)	#SAKNAS!
destination	1 5110(0.011432491139819366)	#SAKNAS!	#SAKNAS!
destiny	1 5110(0.011432491139819366)	#SAKNAS!	2548 (0.033)
destruction	1 5110(0.011432491139819366)	2359 (0.034)	#SAKNAS!
destruction	1 5110(0.011432491139819366)	2359 (0.034)	#SAKNAS!
desynchronization	1 5110(0.011432491139819366)	#SAKNAS!	#SAKNAS!
detention	1 5110(0.011432491139819366)	#SAKNAS!	#SAKNAS!
deteriorating	1 5110(0.011432491139819366)	#SAKNAS!	#SAKNAS!
deterioration	1 2693(0.0342974734194581)	2359 (0.034)	2548 (0.033)
determinant	1 3437(0.02286498227963873)	2359 (0.034)	2548 (0.033)
determining	1 1894(0.05716245569909683)	2359 (0.034)	1233 (0.1)
deters	1 5110(0.011432491139819366)	#SAKNAS!	2548 (0.033)
detoxification	1 5110(0.011432491139819366)	2359 (0.034)	#SAKNAS!
detoxification	1 5110(0.011432491139819366)	2359 (0.034)	#SAKNAS!
detritivores	1 5110(0.011432491139819366)	#SAKNAS!	#SAKNAS!
detritivorous	1 5110(0.011432491139819366)	#SAKNAS!	#SAKNAS!
deuterium	1 5110(0.011432491139819366)	2359 (0.034)	#SAKNAS!
deuterium	1 5110(0.011432491139819366)	2359 (0.034)	#SAKNAS!
deutetrabenazine	1 5110(0.011432491139819366)	#SAKNAS!	2548 (0.033)
deutsche	1 5110(0.011432491139819366)	#SAKNAS!	2548 (0.033)
devaluation	1 5110(0.011432491139819366)	2359 (0.034)	#SAKNAS!
devaluation	1 5110(0.011432491139819366)	2359 (0.034)	#SAKNAS!

developmentally	1	5110(0.011432491139819366)	2359 (0.034)	#SAKNAS!
developmentally	1	5110(0.011432491139819366)	2359 (0.034)	#SAKNAS!
developments	1	3437(0.02286498227963873)	2359 (0.034)	#SAKNAS!
development-third	1	5110(0.011432491139819366)	2359 (0.034)	#SAKNAS!
development-third	1	5110(0.011432491139819366)	2359 (0.034)	#SAKNAS!
deviate	1	5110(0.011432491139819366)	2359 (0.034)	#SAKNAS!
deviate	1	5110(0.011432491139819366)	2359 (0.034)	#SAKNAS!
device	1	1678(0.0685949468389162)	2359 (0.034)	1668 (0.066)
device-assisted	1	5110(0.011432491139819366)	#SAKNAS!	#SAKNAS!
device-facilitated	1	5110(0.011432491139819366)	#SAKNAS!	2548 (0.033)
dexamethasone	1	5110(0.011432491139819366)	#SAKNAS!	#SAKNAS!
dexamethasone-treatment	1	5110(0.011432491139819366)	#SAKNAS!	2548 (0.033)
d-galactose	1	5110(0.011432491139819366)	#SAKNAS!	#SAKNAS!
dgem	1	5110(0.011432491139819366)	2359 (0.034)	#SAKNAS!
dgem	1	5110(0.011432491139819366)	2359 (0.034)	#SAKNAS!
dgg	1	5110(0.011432491139819366)	2359 (0.034)	#SAKNAS!
dgg	1	5110(0.011432491139819366)	2359 (0.034)	#SAKNAS!
dgkj	1	5110(0.011432491139819366)	#SAKNAS!	2548 (0.033)
diabetes-oral	1	5110(0.011432491139819366)	#SAKNAS!	#SAKNAS!
diabetes-periodontitis	1	5110(0.011432491139819366)	#SAKNAS!	#SAKNAS!
diabetes-specific	1	5110(0.011432491139819366)	#SAKNAS!	#SAKNAS!
diabetic-friendly	1	5110(0.011432491139819366)	#SAKNAS!	#SAKNAS!
diabetics	1	2693(0.0342974734194581)	2359 (0.034)	2548 (0.033)
diabetologists	1	5110(0.011432491139819366)	#SAKNAS!	#SAKNAS!
diabetology	1	5110(0.011432491139819366)	#SAKNAS!	#SAKNAS!
diabrotica	1	5110(0.011432491139819366)	2359 (0.034)	#SAKNAS!
diabrotica	1	5110(0.011432491139819366)	2359 (0.034)	#SAKNAS!
diadema	1	5110(0.011432491139819366)	2359 (0.034)	#SAKNAS!
diadema	1	5110(0.011432491139819366)	2359 (0.034)	#SAKNAS!
diagnose	1	5110(0.011432491139819366)	#SAKNAS!	#SAKNAS!
dialium	1	5110(0.011432491139819366)	#SAKNAS!	2548 (0.033)
diamides	1	5110(0.011432491139819366)	#SAKNAS!	#SAKNAS!
diamondback	1	5110(0.011432491139819366)	2359 (0.034)	#SAKNAS!
diamondback	1	5110(0.011432491139819366)	2359 (0.034)	#SAKNAS!
diaphorima	1	5110(0.011432491139819366)	#SAKNAS!	#SAKNAS!
diaphorina	1	5110(0.011432491139819366)	#SAKNAS!	2548 (0.033)
diaphragmatic	1	3437(0.02286498227963873)	2359 (0.034)	#SAKNAS!
diarrhea	1	2693(0.0342974734194581)	2359 (0.034)	2548 (0.033)
diastolic	1	2693(0.0342974734194581)	2359 (0.034)	1668 (0.066)
diazepam-binding	1	5110(0.011432491139819366)	#SAKNAS!	#SAKNAS!
dicentrarcus	1	5110(0.011432491139819366)	2359 (0.034)	#SAKNAS!
dicentrarcus	1	5110(0.011432491139819366)	2359 (0.034)	#SAKNAS!
diceros	1	5110(0.011432491139819366)	2359 (0.034)	#SAKNAS!

diceros	1	5110(0.011432491139819366)	2359 (0.034)	#SAKNAS!
dictate	1	5110(0.011432491139819366)	#SAKNAS!	2548 (0.033)
didelphis	1	5110(0.011432491139819366)	#SAKNAS!	#SAKNAS!
didn	1	5110(0.011432491139819366)	#SAKNAS!	2548 (0.033)
die	1	5110(0.011432491139819366)	2359 (0.034)	#SAKNAS!
die	1	5110(0.011432491139819366)	2359 (0.034)	#SAKNAS!
diencephalon	1	5110(0.011432491139819366)	2359 (0.034)	#SAKNAS!
diencephalon	1	5110(0.011432491139819366)	2359 (0.034)	#SAKNAS!
dietary-induced	1	5110(0.011432491139819366)	2359 (0.034)	#SAKNAS!
dietary-induced	1	5110(0.011432491139819366)	2359 (0.034)	#SAKNAS!
diet-borne	1	5110(0.011432491139819366)	#SAKNAS!	2548 (0.033)
diet-diaries	1	5110(0.011432491139819366)	#SAKNAS!	2548 (0.033)
diet-microbiota-host	1	5110(0.011432491139819366)	#SAKNAS!	2548 (0.033)
differ	1	2693(0.0342974734194581)	2359 (0.034)	2548 (0.033)
differentially	1	3437(0.02286498227963873)	2359 (0.034)	2548 (0.033)
differentiates	1	5110(0.011432491139819366)	#SAKNAS!	2548 (0.033)
differentiating	1	5110(0.011432491139819366)	#SAKNAS!	2548 (0.033)
differently	1	3437(0.02286498227963873)	2359 (0.034)	2548 (0.033)
different-sized	1	5110(0.011432491139819366)	#SAKNAS!	2548 (0.033)
difficult	1	2693(0.0342974734194581)	2359 (0.034)	2548 (0.033)
difficulty	1	836(0.16005487595747114)	2359 (0.034)	999 (0.13)
diffuse	1	5110(0.011432491139819366)	2359 (0.034)	#SAKNAS!
diffuse	1	5110(0.011432491139819366)	2359 (0.034)	#SAKNAS!
digastric	1	5110(0.011432491139819366)	#SAKNAS!	#SAKNAS!
digest	1	5110(0.011432491139819366)	#SAKNAS!	#SAKNAS!
digest-fees	1	5110(0.011432491139819366)	#SAKNAS!	#SAKNAS!
digluconate	1	5110(0.011432491139819366)	2359 (0.034)	#SAKNAS!
digluconate	1	5110(0.011432491139819366)	2359 (0.034)	#SAKNAS!
dihydroxyflavone	1	5110(0.011432491139819366)	#SAKNAS!	#SAKNAS!
dilations	1	5110(0.011432491139819366)	#SAKNAS!	#SAKNAS!
dilemma	1	5110(0.011432491139819366)	#SAKNAS!	#SAKNAS!
dilutions	1	5110(0.011432491139819366)	2359 (0.034)	#SAKNAS!
dilutions	1	5110(0.011432491139819366)	2359 (0.034)	#SAKNAS!
dimension	1	5110(0.011432491139819366)	#SAKNAS!	#SAKNAS!
dimethylarginine	1	5110(0.011432491139819366)	2359 (0.034)	#SAKNAS!
dimethylarginine	1	5110(0.011432491139819366)	2359 (0.034)	#SAKNAS!
diminishes	1	5110(0.011432491139819366)	#SAKNAS!	#SAKNAS!
dimorphic	1	2693(0.0342974734194581)	2359 (0.034)	1668 (0.066)
dimorphism	1	3437(0.02286498227963873)	2359 (0.034)	2548 (0.033)
dining	1	5110(0.011432491139819366)	#SAKNAS!	#SAKNAS!
dinner	1	3437(0.02286498227963873)	2359 (0.034)	2548 (0.033)
dinoflagellate	1	3437(0.02286498227963873)	2359 (0.034)	2548 (0.033)
dinoflagellates	1	5110(0.011432491139819366)	#SAKNAS!	2548 (0.033)

dinophyceae	1	5110(0.011432491139819366)	#SAKNAS!	2548 (0.033)
diode	1	5110(0.011432491139819366)	#SAKNAS!	2548 (0.033)
dioscorea	1	5110(0.011432491139819366)	#SAKNAS!	#SAKNAS!
dioxin	1	5110(0.011432491139819366)	#SAKNAS!	2548 (0.033)
dip	1	5110(0.011432491139819366)	#SAKNAS!	2548 (0.033)
diphenyl	1	5110(0.011432491139819366)	#SAKNAS!	#SAKNAS!
diploid	1	5110(0.011432491139819366)	2359 (0.034)	#SAKNAS!
diploid	1	5110(0.011432491139819366)	2359 (0.034)	#SAKNAS!
diplopoda	1	5110(0.011432491139819366)	#SAKNAS!	#SAKNAS!
dipping	1	5110(0.011432491139819366)	#SAKNAS!	#SAKNAS!
direction	1	3437(0.02286498227963873)	2359 (0.034)	2548 (0.033)
directions	1	5110(0.011432491139819366)	2359 (0.034)	#SAKNAS!
directions	1	5110(0.011432491139819366)	2359 (0.034)	#SAKNAS!
directive	1	5110(0.011432491139819366)	2359 (0.034)	#SAKNAS!
directive	1	5110(0.011432491139819366)	2359 (0.034)	#SAKNAS!
directives	1	5110(0.011432491139819366)	#SAKNAS!	#SAKNAS!
disabled-child	1	5110(0.011432491139819366)	2359 (0.034)	#SAKNAS!
disabled-child	1	5110(0.011432491139819366)	2359 (0.034)	#SAKNAS!
disadvantage	1	3437(0.02286498227963873)	2359 (0.034)	#SAKNAS!
disappearance	1	5110(0.011432491139819366)	2359 (0.034)	#SAKNAS!
disappearance	1	5110(0.011432491139819366)	2359 (0.034)	#SAKNAS!
disastrous	1	5110(0.011432491139819366)	#SAKNAS!	2548 (0.033)
discards	1	5110(0.011432491139819366)	#SAKNAS!	2548 (0.033)
discharge	1	1219(0.1028924202583743)	2359 (0.034)	1233 (0.1)
disclosing	1	5110(0.011432491139819366)	#SAKNAS!	2548 (0.033)
disconnection-based	1	5110(0.011432491139819366)	#SAKNAS!	#SAKNAS!
discontinuity	1	5110(0.011432491139819366)	#SAKNAS!	2548 (0.033)
discrete	1	5110(0.011432491139819366)	2359 (0.034)	#SAKNAS!
discrete	1	5110(0.011432491139819366)	2359 (0.034)	#SAKNAS!
discriminates	1	5110(0.011432491139819366)	#SAKNAS!	2548 (0.033)
discus	1	5110(0.011432491139819366)	2359 (0.034)	#SAKNAS!
discus	1	5110(0.011432491139819366)	2359 (0.034)	#SAKNAS!
discussing	1	5110(0.011432491139819366)	#SAKNAS!	#SAKNAS!
disease-modifying	1	5110(0.011432491139819366)	#SAKNAS!	#SAKNAS!
disease-resistant	1	5110(0.011432491139819366)	2359 (0.034)	#SAKNAS!
disease-resistant	1	5110(0.011432491139819366)	2359 (0.034)	#SAKNAS!
disease-united	1	5110(0.011432491139819366)	#SAKNAS!	2548 (0.033)
disentangling	1	5110(0.011432491139819366)	2359 (0.034)	#SAKNAS!
disentangling	1	5110(0.011432491139819366)	2359 (0.034)	#SAKNAS!
disinhibition	1	5110(0.011432491139819366)	#SAKNAS!	#SAKNAS!
disjunct	1	5110(0.011432491139819366)	#SAKNAS!	#SAKNAS!
diskectomy	1	5110(0.011432491139819366)	#SAKNAS!	#SAKNAS!
disks	1	5110(0.011432491139819366)	#SAKNAS!	2548 (0.033)

dislodgement	1	5110(0.011432491139819366)	#SAKNAS!	#SAKNAS!
dismutase	1	5110(0.011432491139819366)	#SAKNAS!	#SAKNAS!
disorder-a	1	5110(0.011432491139819366)	#SAKNAS!	#SAKNAS!
disparity	1	2214(0.04572996455927746)	2359 (0.034)	1668 (0.066)
dispersal	1	5110(0.011432491139819366)	#SAKNAS!	2548 (0.033)
dispersions	1	5110(0.011432491139819366)	#SAKNAS!	2548 (0.033)
displacements	1	3437(0.02286498227963873)	2359 (0.034)	2548 (0.033)
display	1	2693(0.0342974734194581)	2359 (0.034)	2548 (0.033)
disruption	1	2693(0.0342974734194581)	2359 (0.034)	#SAKNAS!
disruptions	1	5110(0.011432491139819366)	#SAKNAS!	#SAKNAS!
disruptive	1	5110(0.011432491139819366)	#SAKNAS!	2548 (0.033)
disrupts	1	5110(0.011432491139819366)	2359 (0.034)	#SAKNAS!
disrupts	1	5110(0.011432491139819366)	2359 (0.034)	#SAKNAS!
dissatisfaction	1	2693(0.0342974734194581)	2359 (0.034)	1668 (0.066)
disseminating	1	5110(0.011432491139819366)	#SAKNAS!	2548 (0.033)
dissociable	1	5110(0.011432491139819366)	#SAKNAS!	#SAKNAS!
dissociation	1	5110(0.011432491139819366)	#SAKNAS!	2548 (0.033)
dissostichus	1	5110(0.011432491139819366)	#SAKNAS!	#SAKNAS!
distal	1	2214(0.04572996455927746)	2359 (0.034)	#SAKNAS!
distance-based	1	5110(0.011432491139819366)	#SAKNAS!	#SAKNAS!
distinctive	1	5110(0.011432491139819366)	#SAKNAS!	2548 (0.033)
distinguishing	1	5110(0.011432491139819366)	#SAKNAS!	2548 (0.033)
distraction	1	5110(0.011432491139819366)	#SAKNAS!	#SAKNAS!
distributional	1	5110(0.011432491139819366)	#SAKNAS!	#SAKNAS!
districts	1	5110(0.011432491139819366)	#SAKNAS!	#SAKNAS!
disturbing	1	5110(0.011432491139819366)	#SAKNAS!	#SAKNAS!
disturbs	1	5110(0.011432491139819366)	#SAKNAS!	#SAKNAS!
dit-coombe	1	5110(0.011432491139819366)	#SAKNAS!	#SAKNAS!
diterpenes	1	5110(0.011432491139819366)	2359 (0.034)	#SAKNAS!
diterpenes	1	5110(0.011432491139819366)	2359 (0.034)	#SAKNAS!
divergence	1	5110(0.011432491139819366)	2359 (0.034)	#SAKNAS!
divergence	1	5110(0.011432491139819366)	2359 (0.034)	#SAKNAS!
diverse	1	1678(0.0685949468389162)	2359 (0.034)	1668 (0.066)
diversion	1	2693(0.0342974734194581)	2359 (0.034)	1668 (0.066)
diverticula	1	5110(0.011432491139819366)	2359 (0.034)	#SAKNAS!
diverticula	1	5110(0.011432491139819366)	2359 (0.034)	#SAKNAS!
diverticulitis	1	5110(0.011432491139819366)	2359 (0.034)	#SAKNAS!
diverticulitis	1	5110(0.011432491139819366)	2359 (0.034)	#SAKNAS!
dmanisi	1	5110(0.011432491139819366)	#SAKNAS!	2548 (0.033)
dmf	1	5110(0.011432491139819366)	#SAKNAS!	#SAKNAS!
dmft	1	1678(0.0685949468389162)	2359 (0.034)	#SAKNAS!
dmhf	1	5110(0.011432491139819366)	#SAKNAS!	2548 (0.033)
dms	1	5110(0.011432491139819366)	#SAKNAS!	2548 (0.033)

dna	1	3437(0.0228649822796387З)	2359 (0.034)	2548 (0.033)
dnqp	1	5110(0.011432491139819366)	#SAKNAS!	2548 (0.033)
docetaxel	1	5110(0.011432491139819366)	#SAKNAS!	2548 (0.033)
docetaxel-cisplatin-fluorouracil	1	5110(0.011432491139819366)	#SAKNAS!	#SAKNAS!
doctors	1	5110(0.011432491139819366)	#SAKNAS!	#SAKNAS!
document	1	5110(0.011432491139819366)	#SAKNAS!	2548 (0.033)
documents	1	5110(0.011432491139819366)	#SAKNAS!	2548 (0.033)
doesn	1	5110(0.011432491139819366)	#SAKNAS!	2548 (0.033)
dog-human	1	5110(0.011432491139819366)	#SAKNAS!	#SAKNAS!
doing	1	2214(0.04572996455927746)	2359 (0.034)	1668 (0.066)
domesticus	1	5110(0.011432491139819366)	#SAKNAS!	#SAKNAS!
dominance-related	1	5110(0.011432491139819366)	2359 (0.034)	#SAKNAS!
dominance-related	1	5110(0.011432491139819366)	2359 (0.034)	#SAKNAS!
dominican	1	5110(0.011432491139819366)	#SAKNAS!	#SAKNAS!
don	1	5110(0.011432491139819366)	2359 (0.034)	#SAKNAS!
don	1	5110(0.011432491139819366)	2359 (0.034)	#SAKNAS!
door-to-oral	1	5110(0.011432491139819366)	#SAKNAS!	#SAKNAS!
dopamine-4	1	5110(0.011432491139819366)	#SAKNAS!	2548 (0.033)
dopamine-neuropeptide	1	5110(0.011432491139819366)	#SAKNAS!	#SAKNAS!
doped	1	5110(0.011432491139819366)	#SAKNAS!	#SAKNAS!
dor	1	5110(0.011432491139819366)	2359 (0.034)	#SAKNAS!
dor	1	5110(0.011432491139819366)	2359 (0.034)	#SAKNAS!
dorsal	1	5110(0.011432491139819366)	2359 (0.034)	#SAKNAS!
dorsal	1	5110(0.011432491139819366)	2359 (0.034)	#SAKNAS!
dorsalis	1	5110(0.011432491139819366)	2359 (0.034)	#SAKNAS!
dorsalis	1	5110(0.011432491139819366)	2359 (0.034)	#SAKNAS!
dorset	1	5110(0.011432491139819366)	2359 (0.034)	#SAKNAS!
dorset	1	5110(0.011432491139819366)	2359 (0.034)	#SAKNAS!
doryteuthis	1	5110(0.011432491139819366)	#SAKNAS!	#SAKNAS!
dose-addition	1	5110(0.011432491139819366)	2359 (0.034)	#SAKNAS!
dose-addition	1	5110(0.011432491139819366)	2359 (0.034)	#SAKNAS!
dose-dependent	1	5110(0.011432491139819366)	#SAKNAS!	#SAKNAS!
doses	1	5110(0.011432491139819366)	#SAKNAS!	#SAKNAS!
dose-volume	1	5110(0.011432491139819366)	#SAKNAS!	#SAKNAS!
dosimetric	1	2693(0.0342974734194581)	2359 (0.034)	1668 (0.066)
double-blind	1	1485(0.08002743797873557)	2359 (0.034)	1233 (0.1)
doubly	1	5110(0.011432491139819366)	#SAKNAS!	#SAKNAS!
doves	1	5110(0.011432491139819366)	2359 (0.034)	#SAKNAS!
doves	1	5110(0.011432491139819366)	2359 (0.034)	#SAKNAS!
dowling	1	5110(0.011432491139819366)	2359 (0.034)	#SAKNAS!
dowling	1	5110(0.011432491139819366)	2359 (0.034)	#SAKNAS!
dr	1	5110(0.011432491139819366)	2359 (0.034)	#SAKNAS!
dr	1	5110(0.011432491139819366)	2359 (0.034)	#SAKNAS!

dramatic	1	5110(0.011432491139819366)	#SAKNAS!	2548 (0.033)
dramatically	1	5110(0.011432491139819366)	2359 (0.034)	#SAKNAS!
dramatically	1	5110(0.011432491139819366)	2359 (0.034)	#SAKNAS!
draught	1	5110(0.011432491139819366)	2359 (0.034)	#SAKNAS!
draught	1	5110(0.011432491139819366)	2359 (0.034)	#SAKNAS!
drd4	1	5110(0.011432491139819366)	#SAKNAS!	2548 (0.033)
dreissena	1	3437(0.02286498227963873)	2359 (0.034)	2548 (0.033)
drift	1	5110(0.011432491139819366)	#SAKNAS!	#SAKNAS!
drink	1	1111(0.11432491139819366)	2359 (0.034)	584 (0.23)
drive	1	2693(0.0342974734194581)	2359 (0.034)	2548 (0.033)
drivers	1	5110(0.011432491139819366)	#SAKNAS!	#SAKNAS!
driving	1	5110(0.011432491139819366)	#SAKNAS!	#SAKNAS!
droebachiensis	1	5110(0.011432491139819366)	2359 (0.034)	#SAKNAS!
droebachiensis	1	5110(0.011432491139819366)	2359 (0.034)	#SAKNAS!
drooling	1	3437(0.02286498227963873)	2359 (0.034)	2548 (0.033)
dropout	1	5110(0.011432491139819366)	#SAKNAS!	2548 (0.033)
drosophila-melanogaster	1	5110(0.011432491139819366)	2359 (0.034)	#SAKNAS!
drosophila-melanogaster	1	5110(0.011432491139819366)	2359 (0.034)	#SAKNAS!
drosophila-serido	1	5110(0.011432491139819366)	2359 (0.034)	#SAKNAS!
drosophila-serido	1	5110(0.011432491139819366)	2359 (0.034)	#SAKNAS!
drought	1	5110(0.011432491139819366)	#SAKNAS!	2548 (0.033)
dryness	1	1678(0.0685949468389162)	2359 (0.034)	1668 (0.066)
dryopithecine	1	5110(0.011432491139819366)	#SAKNAS!	2548 (0.033)
ds	1	5110(0.011432491139819366)	#SAKNAS!	#SAKNAS!
dsa篢	1	5110(0.011432491139819366)	#SAKNAS!	#SAKNAS!
dswal-qol	1	5110(0.011432491139819366)	#SAKNAS!	2548 (0.033)
dual	1	5110(0.011432491139819366)	#SAKNAS!	2548 (0.033)
dual-axis	1	5110(0.011432491139819366)	2359 (0.034)	#SAKNAS!
dual-axis	1	5110(0.011432491139819366)	2359 (0.034)	#SAKNAS!
dual-energy	1	3437(0.02286498227963873)	2359 (0.034)	#SAKNAS!
dual-tasks	1	5110(0.011432491139819366)	#SAKNAS!	2548 (0.033)
duck	1	5110(0.011432491139819366)	#SAKNAS!	2548 (0.033)
ducks	1	5110(0.011432491139819366)	2359 (0.034)	#SAKNAS!
ducks	1	5110(0.011432491139819366)	2359 (0.034)	#SAKNAS!
duke	1	5110(0.011432491139819366)	#SAKNAS!	#SAKNAS!
duodena	1	5110(0.011432491139819366)	2359 (0.034)	#SAKNAS!
duodena	1	5110(0.011432491139819366)	2359 (0.034)	#SAKNAS!
duodenal	1	3437(0.02286498227963873)	2359 (0.034)	2548 (0.033)
duodenoileal	1	5110(0.011432491139819366)	#SAKNAS!	2548 (0.033)
durability	1	5110(0.011432491139819366)	#SAKNAS!	2548 (0.033)
durum	1	5110(0.011432491139819366)	#SAKNAS!	#SAKNAS!
durvalumab	1	5110(0.011432491139819366)	#SAKNAS!	#SAKNAS!
dutch	1	1485(0.08002743797873557)	2359 (0.034)	1233 (0.1)

dwindles	1	5110(0.011432491139819366)	2359 (0.034)	#SAKNAS!
dwindles	1	5110(0.011432491139819366)	2359 (0.034)	#SAKNAS!
dying	1	2693(0.0342974734194581)	2359 (0.034)	2548 (0.033)
dymus	1	5110(0.011432491139819366)	#SAKNAS!	#SAKNAS!
dymus-hr	1	5110(0.011432491139819366)	#SAKNAS!	#SAKNAS!
dynamically	1	5110(0.011432491139819366)	#SAKNAS!	#SAKNAS!
dynamicity	1	5110(0.011432491139819366)	#SAKNAS!	#SAKNAS!
dynamo	1	5110(0.011432491139819366)	#SAKNAS!	2548 (0.033)
dynorphin	1	5110(0.011432491139819366)	#SAKNAS!	2548 (0.033)
dysautonomia	1	5110(0.011432491139819366)	2359 (0.034)	#SAKNAS!
dysautonomia	1	5110(0.011432491139819366)	2359 (0.034)	#SAKNAS!
dysgnathic	1	5110(0.011432491139819366)	2359 (0.034)	#SAKNAS!
dysgnathic	1	5110(0.011432491139819366)	2359 (0.034)	#SAKNAS!
dyskinesia	1	3437(0.02286498227963873)	2359 (0.034)	2548 (0.033)
dysmotility	1	2693(0.0342974734194581)	2359 (0.034)	2548 (0.033)
dysphagia-a	1	5110(0.011432491139819366)	#SAKNAS!	#SAKNAS!
dysphagia-friendly	1	5110(0.011432491139819366)	#SAKNAS!	2548 (0.033)
dysphagia-oriented	1	1894(0.05716245569909683)	2359 (0.034)	#SAKNAS!
dysphagia-specific	1	5110(0.011432491139819366)	#SAKNAS!	#SAKNAS!
dysphaging	1	5110(0.011432491139819366)	#SAKNAS!	#SAKNAS!
dysphagy	1	5110(0.011432491139819366)	#SAKNAS!	2548 (0.033)
dysplasia	1	3437(0.02286498227963873)	2359 (0.034)	2548 (0.033)
dyspnea	1	5110(0.011432491139819366)	#SAKNAS!	2548 (0.033)
dysregulation	1	5110(0.011432491139819366)	#SAKNAS!	#SAKNAS!
dystonia	1	3437(0.02286498227963873)	2359 (0.034)	#SAKNAS!
dystrophica	1	5110(0.011432491139819366)	#SAKNAS!	2548 (0.033)
dytiscidae	1	3437(0.02286498227963873)	2359 (0.034)	2548 (0.033)
dytiscus	1	3437(0.02286498227963873)	2359 (0.034)	2548 (0.033)
e.v	1	5110(0.011432491139819366)	#SAKNAS!	2548 (0.033)
e2	1	3437(0.02286498227963873)	2359 (0.034)	#SAKNAS!
eagle	1	5110(0.011432491139819366)	#SAKNAS!	2548 (0.033)
earlier	1	3437(0.02286498227963873)	2359 (0.034)	2548 (0.033)
early-life	1	3437(0.02286498227963873)	2359 (0.034)	#SAKNAS!
early-onset	1	5110(0.011432491139819366)	2359 (0.034)	#SAKNAS!
early-onset	1	5110(0.011432491139819366)	2359 (0.034)	#SAKNAS!
early-to-advanced	1	5110(0.011432491139819366)	#SAKNAS!	2548 (0.033)
earthquake	1	5110(0.011432491139819366)	#SAKNAS!	2548 (0.033)
ease	1	5110(0.011432491139819366)	#SAKNAS!	#SAKNAS!
easo-defined	1	5110(0.011432491139819366)	#SAKNAS!	#SAKNAS!
easy	1	5110(0.011432491139819366)	2359 (0.034)	#SAKNAS!
easy	1	5110(0.011432491139819366)	2359 (0.034)	#SAKNAS!
eat-10	1	1894(0.05716245569909683)	2359 (0.034)	1668 (0.066)
eat-10spa	1	5110(0.011432491139819366)	#SAKNAS!	2548 (0.033)

eat-ability	1 5110(0.011432491139819366)	#SAKNAS!	2548 (0.033)
eating-related	1 5110(0.011432491139819366)	#SAKNAS!	#SAKNAS!
eat-lancet	1 5110(0.011432491139819366)	#SAKNAS!	#SAKNAS!
ecc	1 5110(0.011432491139819366)	#SAKNAS!	#SAKNAS!
echium	1 5110(0.011432491139819366)	#SAKNAS!	2548 (0.033)
echocardiographic	1 5110(0.011432491139819366)	#SAKNAS!	2548 (0.033)
echocardiography	1 5110(0.011432491139819366)	#SAKNAS!	2548 (0.033)
echogenicity	1 5110(0.011432491139819366)	#SAKNAS!	#SAKNAS!
e-cigarettes	1 5110(0.011432491139819366)	#SAKNAS!	#SAKNAS!
eckardt	1 5110(0.011432491139819366)	#SAKNAS!	2548 (0.033)
ecohistological	1 5110(0.011432491139819366)	2359 (0.034)	#SAKNAS!
ecohistological	1 5110(0.011432491139819366)	2359 (0.034)	#SAKNAS!
ecomorphology	1 5110(0.011432491139819366)	#SAKNAS!	#SAKNAS!
economically	1 5110(0.011432491139819366)	#SAKNAS!	2548 (0.033)
economy	1 5110(0.011432491139819366)	#SAKNAS!	2548 (0.033)
ecopal	1 5110(0.011432491139819366)	#SAKNAS!	#SAKNAS!
ecophysiological	1 5110(0.011432491139819366)	#SAKNAS!	2548 (0.033)
ectoparasitoid	1 5110(0.011432491139819366)	2359 (0.034)	#SAKNAS!
ectoparasitoid	1 5110(0.011432491139819366)	2359 (0.034)	#SAKNAS!
edelphi	1 5110(0.011432491139819366)	#SAKNAS!	#SAKNAS!
edelstein	1 5110(0.011432491139819366)	2359 (0.034)	#SAKNAS!
edelstein	1 5110(0.011432491139819366)	2359 (0.034)	#SAKNAS!
edema	1 3437(0.02286498227963873)	2359 (0.034)	#SAKNAS!
edematous	1 5110(0.011432491139819366)	2359 (0.034)	#SAKNAS!
edematous	1 5110(0.011432491139819366)	2359 (0.034)	#SAKNAS!
edentate	1 5110(0.011432491139819366)	#SAKNAS!	#SAKNAS!
ediacaran	1 5110(0.011432491139819366)	#SAKNAS!	#SAKNAS!
edinburgh	1 3437(0.02286498227963873)	2359 (0.034)	#SAKNAS!
edition	1 5110(0.011432491139819366)	2359 (0.034)	#SAKNAS!
edition	1 5110(0.011432491139819366)	2359 (0.034)	#SAKNAS!
educate	1 5110(0.011432491139819366)	2359 (0.034)	#SAKNAS!
educate	1 5110(0.011432491139819366)	2359 (0.034)	#SAKNAS!
educators	1 5110(0.011432491139819366)	#SAKNAS!	#SAKNAS!
edwards	1 5110(0.011432491139819366)	2359 (0.034)	#SAKNAS!
edwards	1 5110(0.011432491139819366)	2359 (0.034)	#SAKNAS!
edwardsi	1 5110(0.011432491139819366)	2359 (0.034)	#SAKNAS!
edwardsi	1 5110(0.011432491139819366)	2359 (0.034)	#SAKNAS!
eel	1 5110(0.011432491139819366)	2359 (0.034)	#SAKNAS!
eel	1 5110(0.011432491139819366)	2359 (0.034)	#SAKNAS!
effectively	1 5110(0.011432491139819366)	#SAKNAS!	2548 (0.033)
efferents	1 5110(0.011432491139819366)	#SAKNAS!	#SAKNAS!
efficiencies	1 5110(0.011432491139819366)	#SAKNAS!	2548 (0.033)
efficient	1 5110(0.011432491139819366)	2359 (0.034)	#SAKNAS!

efficient	1	5110(0.011432491139819366)	2359 (0.034)	#SAKNAS!
efflux	1	5110(0.011432491139819366)	2359 (0.034)	#SAKNAS!
efflux	1	5110(0.011432491139819366)	2359 (0.034)	#SAKNAS!
effort	1	5110(0.011432491139819366)	#SAKNAS!	2548 (0.033)
efforts	1	5110(0.011432491139819366)	2359 (0.034)	#SAKNAS!
efforts	1	5110(0.011432491139819366)	2359 (0.034)	#SAKNAS!
egcg	1	5110(0.011432491139819366)	2359 (0.034)	#SAKNAS!
egcg	1	5110(0.011432491139819366)	2359 (0.034)	#SAKNAS!
egestion	1	5110(0.011432491139819366)	2359 (0.034)	#SAKNAS!
egestion	1	5110(0.011432491139819366)	2359 (0.034)	#SAKNAS!
eggs	1	5110(0.011432491139819366)	2359 (0.034)	#SAKNAS!
eggs	1	5110(0.011432491139819366)	2359 (0.034)	#SAKNAS!
egjoo	1	5110(0.011432491139819366)	#SAKNAS!	#SAKNAS!
egrets	1	5110(0.011432491139819366)	2359 (0.034)	#SAKNAS!
egrets	1	5110(0.011432491139819366)	2359 (0.034)	#SAKNAS!
egyptian	1	1894(0.05716245569909683)	2359 (0.034)	#SAKNAS!
ehealth	1	5110(0.011432491139819366)	#SAKNAS!	2548 (0.033)
ehlers-dantos	1	5110(0.011432491139819366)	2359 (0.034)	#SAKNAS!
ehlers-dantos	1	5110(0.011432491139819366)	2359 (0.034)	#SAKNAS!
eica-essd-eugms	1	5110(0.011432491139819366)	#SAKNAS!	2548 (0.033)
eichner	1	5110(0.011432491139819366)	#SAKNAS!	#SAKNAS!
eicosapentaenoic	1	5110(0.011432491139819366)	2359 (0.034)	#SAKNAS!
eicosapentaenoic	1	5110(0.011432491139819366)	2359 (0.034)	#SAKNAS!
eiffingeri	1	5110(0.011432491139819366)	2359 (0.034)	#SAKNAS!
eiffingeri	1	5110(0.011432491139819366)	2359 (0.034)	#SAKNAS!
eight	1	5110(0.011432491139819366)	#SAKNAS!	2548 (0.033)
einflufl	1	5110(0.011432491139819366)	2359 (0.034)	#SAKNAS!
einflufl	1	5110(0.011432491139819366)	2359 (0.034)	#SAKNAS!
eisenia	1	5110(0.011432491139819366)	#SAKNAS!	2548 (0.033)
ejection	1	5110(0.011432491139819366)	#SAKNAS!	2548 (0.033)
elaidic	1	5110(0.011432491139819366)	#SAKNAS!	#SAKNAS!
elastase	1	3437(0.02286498227963873)	2359 (0.034)	#SAKNAS!
elasticity	1	5110(0.011432491139819366)	#SAKNAS!	2548 (0.033)
elastography	1	5110(0.011432491139819366)	#SAKNAS!	#SAKNAS!
elbasvir	1	5110(0.011432491139819366)	#SAKNAS!	2548 (0.033)
elder	1	3437(0.02286498227963873)	2359 (0.034)	#SAKNAS!
e-learning	1	5110(0.011432491139819366)	#SAKNAS!	2548 (0.033)
electroacupuncture	1	5110(0.011432491139819366)	#SAKNAS!	#SAKNAS!
electrocardiographic	1	5110(0.011432491139819366)	2359 (0.034)	#SAKNAS!
electrocardiographic	1	5110(0.011432491139819366)	2359 (0.034)	#SAKNAS!
electrocautery	1	5110(0.011432491139819366)	2359 (0.034)	#SAKNAS!
electrocautery	1	5110(0.011432491139819366)	2359 (0.034)	#SAKNAS!
electrode	1	5110(0.011432491139819366)	#SAKNAS!	2548 (0.033)

electrodiagnostic	1	5110(0.011432491139819366)	#SAKNAS!	#SAKNAS!
electrolytes	1	5110(0.011432491139819366)	#SAKNAS!	#SAKNAS!
electromagnetic	1	5110(0.011432491139819366)	2359 (0.034)	#SAKNAS!
electromagnetic	1	5110(0.011432491139819366)	2359 (0.034)	#SAKNAS!
electronic	1	1894(0.05716245569909683)	2359 (0.034)	1233 (0.1)
electronics	1	5110(0.011432491139819366)	#SAKNAS!	#SAKNAS!
electrophysiological	1	5110(0.011432491139819366)	#SAKNAS!	#SAKNAS!
elefante	1	5110(0.011432491139819366)	#SAKNAS!	2548 (0.033)
elegans	1	5110(0.011432491139819366)	#SAKNAS!	2548 (0.033)
elegansnematodes	1	5110(0.011432491139819366)	#SAKNAS!	2548 (0.033)
elemental	1	5110(0.011432491139819366)	2359 (0.034)	#SAKNAS!
elemental	1	5110(0.011432491139819366)	2359 (0.034)	#SAKNAS!
element-binding	1	5110(0.011432491139819366)	#SAKNAS!	2548 (0.033)
elements	1	2693(0.0342974734194581)	2359 (0.034)	1668 (0.066)
elephants	1	5110(0.011432491139819366)	#SAKNAS!	2548 (0.033)
elephas	1	5110(0.011432491139819366)	#SAKNAS!	#SAKNAS!
elevational	1	5110(0.011432491139819366)	2359 (0.034)	#SAKNAS!
elevational	1	5110(0.011432491139819366)	2359 (0.034)	#SAKNAS!
eleven	1	5110(0.011432491139819366)	2359 (0.034)	#SAKNAS!
eleven	1	5110(0.011432491139819366)	2359 (0.034)	#SAKNAS!
elicit	1	5110(0.011432491139819366)	#SAKNAS!	#SAKNAS!
eligibility	1	5110(0.011432491139819366)	#SAKNAS!	#SAKNAS!
elimination	1	3437(0.02286498227963873)	2359 (0.034)	#SAKNAS!
elite	1	1894(0.05716245569909683)	2359 (0.034)	1233 (0.1)
elongation	1	5110(0.011432491139819366)	#SAKNAS!	#SAKNAS!
elongational	1	5110(0.011432491139819366)	#SAKNAS!	2548 (0.033)
elsa-brasil	1	5110(0.011432491139819366)	#SAKNAS!	#SAKNAS!
elucidate	1	5110(0.011432491139819366)	#SAKNAS!	#SAKNAS!
elucidating	1	5110(0.011432491139819366)	#SAKNAS!	#SAKNAS!
elysia	1	5110(0.011432491139819366)	2359 (0.034)	#SAKNAS!
elysia	1	5110(0.011432491139819366)	2359 (0.034)	#SAKNAS!
embedding	1	5110(0.011432491139819366)	#SAKNAS!	#SAKNAS!
embeds	1	5110(0.011432491139819366)	#SAKNAS!	2548 (0.033)
emblica	1	5110(0.011432491139819366)	#SAKNAS!	2548 (0.033)
emended	1	5110(0.011432491139819366)	2359 (0.034)	#SAKNAS!
emended	1	5110(0.011432491139819366)	2359 (0.034)	#SAKNAS!
emergencies	1	5110(0.011432491139819366)	#SAKNAS!	2548 (0.033)
emergency	1	1678(0.0685949468389162)	2359 (0.034)	1668 (0.066)
emery-dreifuss	1	5110(0.011432491139819366)	#SAKNAS!	#SAKNAS!
emissions	1	5110(0.011432491139819366)	#SAKNAS!	#SAKNAS!
emitted	1	5110(0.011432491139819366)	#SAKNAS!	#SAKNAS!
emotion	1	5110(0.011432491139819366)	#SAKNAS!	2548 (0.033)
emperor	1	5110(0.011432491139819366)	#SAKNAS!	2548 (0.033)

emphasizing	1	5110(0.011432491139819366)	2359 (0.034)	#SAKNAS!
emphasizing	1	5110(0.011432491139819366)	2359 (0.034)	#SAKNAS!
empirically	1	5110(0.011432491139819366)	#SAKNAS!	#SAKNAS!
empyema	1	5110(0.011432491139819366)	#SAKNAS!	#SAKNAS!
emulgels	1	5110(0.011432491139819366)	#SAKNAS!	#SAKNAS!
en	1	5110(0.011432491139819366)	#SAKNAS!	2548 (0.033)
enablers	1	5110(0.011432491139819366)	#SAKNAS!	#SAKNAS!
enables	1	5110(0.011432491139819366)	#SAKNAS!	#SAKNAS!
encephalomyopathy	1	5110(0.011432491139819366)	#SAKNAS!	#SAKNAS!
enchytraeid	1	5110(0.011432491139819366)	2359 (0.034)	#SAKNAS!
enchytraeid	1	5110(0.011432491139819366)	2359 (0.034)	#SAKNAS!
enchytraeus	1	5110(0.011432491139819366)	2359 (0.034)	#SAKNAS!
enchytraeus	1	5110(0.011432491139819366)	2359 (0.034)	#SAKNAS!
encounter	1	5110(0.011432491139819366)	2359 (0.034)	#SAKNAS!
encounter	1	5110(0.011432491139819366)	2359 (0.034)	#SAKNAS!
encouragement	1	5110(0.011432491139819366)	#SAKNAS!	2548 (0.033)
encuesta	1	5110(0.011432491139819366)	#SAKNAS!	2548 (0.033)
endarterectomy	1	5110(0.011432491139819366)	2359 (0.034)	#SAKNAS!
endarterectomy	1	5110(0.011432491139819366)	2359 (0.034)	#SAKNAS!
endemic	1	5110(0.011432491139819366)	2359 (0.034)	#SAKNAS!
endemic	1	5110(0.011432491139819366)	2359 (0.034)	#SAKNAS!
endocannabinoids	1	5110(0.011432491139819366)	#SAKNAS!	#SAKNAS!
endocrine	1	2214(0.04572996455927746)	2359 (0.034)	1668 (0.066)
endocycling	1	5110(0.011432491139819366)	#SAKNAS!	2548 (0.033)
endodontic	1	2214(0.04572996455927746)	2359 (0.034)	1668 (0.066)
end-of-life	1	3437(0.02286498227963873)	2359 (0.034)	2548 (0.033)
endoflip	1	5110(0.011432491139819366)	#SAKNAS!	#SAKNAS!
endografting	1	5110(0.011432491139819366)	#SAKNAS!	2548 (0.033)
endoleak	1	5110(0.011432491139819366)	#SAKNAS!	2548 (0.033)
endometriosis	1	5110(0.011432491139819366)	2359 (0.034)	#SAKNAS!
endometriosis	1	5110(0.011432491139819366)	2359 (0.034)	#SAKNAS!
endonasal	1	5110(0.011432491139819366)	#SAKNAS!	2548 (0.033)
endoparasite	1	5110(0.011432491139819366)	#SAKNAS!	#SAKNAS!
endoparasitoid	1	3437(0.02286498227963873)	2359 (0.034)	2548 (0.033)
endoplasmic	1	5110(0.011432491139819366)	#SAKNAS!	2548 (0.033)
endoprostheses	1	5110(0.011432491139819366)	#SAKNAS!	#SAKNAS!
endoreplication	1	5110(0.011432491139819366)	#SAKNAS!	2548 (0.033)
endoscope	1	5110(0.011432491139819366)	2359 (0.034)	#SAKNAS!
endoscope	1	5110(0.011432491139819366)	2359 (0.034)	#SAKNAS!
endosteal	1	5110(0.011432491139819366)	#SAKNAS!	2548 (0.033)
endotracheal	1	2693(0.0342974734194581)	2359 (0.034)	#SAKNAS!
endpoints	1	5110(0.011432491139819366)	#SAKNAS!	2548 (0.033)
endproducts	1	5110(0.011432491139819366)	#SAKNAS!	2548 (0.033)

end-products	1 5110(0.011432491139819366)	2359 (0.034)	#SAKNAS!
end-products	1 5110(0.011432491139819366)	2359 (0.034)	#SAKNAS!
energetical	1 5110(0.011432491139819366)	2359 (0.034)	#SAKNAS!
energetical	1 5110(0.011432491139819366)	2359 (0.034)	#SAKNAS!
energetics	1 5110(0.011432491139819366)	2359 (0.034)	#SAKNAS!
energetics	1 5110(0.011432491139819366)	2359 (0.034)	#SAKNAS!
energy-cost	1 5110(0.011432491139819366)	2359 (0.034)	#SAKNAS!
energy-cost	1 5110(0.011432491139819366)	2359 (0.034)	#SAKNAS!
energy-dense	1 5110(0.011432491139819366)	2359 (0.034)	#SAKNAS!
energy-dense	1 5110(0.011432491139819366)	2359 (0.034)	#SAKNAS!
energy-efficient	1 5110(0.011432491139819366)	2359 (0.034)	#SAKNAS!
energy-efficient	1 5110(0.011432491139819366)	2359 (0.034)	#SAKNAS!
engage	1 5110(0.011432491139819366)	#SAKNAS!	2548 (0.033)
engaging	1 3437(0.02286498227963873)	2359 (0.034)	2548 (0.033)
enge	1 5110(0.011432491139819366)	#SAKNAS!	#SAKNAS!
engineering	1 5110(0.011432491139819366)	2359 (0.034)	#SAKNAS!
engineering	1 5110(0.011432491139819366)	2359 (0.034)	#SAKNAS!
enhance	1 5110(0.011432491139819366)	#SAKNAS!	2548 (0.033)
enhancement	1 5110(0.011432491139819366)	#SAKNAS!	#SAKNAS!
enhydra	1 5110(0.011432491139819366)	2359 (0.034)	#SAKNAS!
enhydra	1 5110(0.011432491139819366)	2359 (0.034)	#SAKNAS!
enlargement	1 5110(0.011432491139819366)	2359 (0.034)	#SAKNAS!
enlargement	1 5110(0.011432491139819366)	2359 (0.034)	#SAKNAS!
enrichments	1 5110(0.011432491139819366)	2359 (0.034)	#SAKNAS!
enrichments	1 5110(0.011432491139819366)	2359 (0.034)	#SAKNAS!
enrollment	1 5110(0.011432491139819366)	#SAKNAS!	#SAKNAS!
ensifera	1 5110(0.011432491139819366)	#SAKNAS!	#SAKNAS!
ensiled	1 5110(0.011432491139819366)	2359 (0.034)	#SAKNAS!
ensiled	1 5110(0.011432491139819366)	2359 (0.034)	#SAKNAS!
enso	1 5110(0.011432491139819366)	#SAKNAS!	#SAKNAS!
enteric	1 5110(0.011432491139819366)	#SAKNAS!	#SAKNAS!
enterica	1 3437(0.02286498227963873)	2359 (0.034)	#SAKNAS!
enterocolitis	1 5110(0.011432491139819366)	#SAKNAS!	#SAKNAS!
enteroendocrine	1 5110(0.011432491139819366)	#SAKNAS!	#SAKNAS!
enterosalivary	1 5110(0.011432491139819366)	#SAKNAS!	2548 (0.033)
enterostomy	1 5110(0.011432491139819366)	#SAKNAS!	#SAKNAS!
entropy	1 5110(0.011432491139819366)	#SAKNAS!	#SAKNAS!
entropy-balanced	1 5110(0.011432491139819366)	#SAKNAS!	#SAKNAS!
entwicklung	1 5110(0.011432491139819366)	2359 (0.034)	#SAKNAS!
entwicklung	1 5110(0.011432491139819366)	2359 (0.034)	#SAKNAS!
environmental-conditions	1 5110(0.011432491139819366)	2359 (0.034)	#SAKNAS!
environmental-conditions	1 5110(0.011432491139819366)	2359 (0.034)	#SAKNAS!
en-y	1 5110(0.011432491139819366)	#SAKNAS!	2548 (0.033)

enzymatic	1	5110(0.011432491139819366)	#SAKNAS!	#SAKNAS!
eortc	1	3437(0.02286498227963873)	2359 (0.034)	2548 (0.033)
eortc-qlq-h&n35	1	5110(0.011432491139819366)	#SAKNAS!	#SAKNAS!
eosinophilia	1	5110(0.011432491139819366)	#SAKNAS!	#SAKNAS!
eotaxin	1	5110(0.011432491139819366)	#SAKNAS!	2548 (0.033)
epa	1	5110(0.011432491139819366)	#SAKNAS!	2548 (0.033)
epg	1	5110(0.011432491139819366)	#SAKNAS!	2548 (0.033)
epibiotic	1	5110(0.011432491139819366)	#SAKNAS!	2548 (0.033)
epicardial	1	5110(0.011432491139819366)	#SAKNAS!	2548 (0.033)
epic-potsdam	1	5110(0.011432491139819366)	#SAKNAS!	2548 (0.033)
epidermal	1	3437(0.02286498227963873)	2359 (0.034)	#SAKNAS!
epiglottic	1	5110(0.011432491139819366)	#SAKNAS!	#SAKNAS!
epilepsy	1	5110(0.011432491139819366)	#SAKNAS!	#SAKNAS!
epilithic	1	5110(0.011432491139819366)	#SAKNAS!	2548 (0.033)
epimastigotes	1	5110(0.011432491139819366)	#SAKNAS!	#SAKNAS!
epipap	1	5110(0.011432491139819366)	2359 (0.034)	#SAKNAS!
epipap	1	5110(0.011432491139819366)	2359 (0.034)	#SAKNAS!
epiphrenic	1	3437(0.02286498227963873)	2359 (0.034)	2548 (0.033)
episodes	1	5110(0.011432491139819366)	#SAKNAS!	2548 (0.033)
episodic	1	5110(0.011432491139819366)	#SAKNAS!	2548 (0.033)
epithelial	1	5110(0.011432491139819366)	#SAKNAS!	2548 (0.033)
epithelium	1	5110(0.011432491139819366)	2359 (0.034)	#SAKNAS!
epithelium	1	5110(0.011432491139819366)	2359 (0.034)	#SAKNAS!
epos	1	5110(0.011432491139819366)	#SAKNAS!	#SAKNAS!
epstein-barr	1	5110(0.011432491139819366)	#SAKNAS!	#SAKNAS!
eptatretus	1	5110(0.011432491139819366)	#SAKNAS!	2548 (0.033)
equal	1	5110(0.011432491139819366)	#SAKNAS!	#SAKNAS!
equation	1	1219(0.1028924202583743)	2359 (0.034)	1233 (0.1)
equicaloric	1	5110(0.011432491139819366)	#SAKNAS!	2548 (0.033)
equids	1	5110(0.011432491139819366)	#SAKNAS!	2548 (0.033)
equine	1	5110(0.011432491139819366)	2359 (0.034)	#SAKNAS!
equine	1	5110(0.011432491139819366)	2359 (0.034)	#SAKNAS!
era	1	3437(0.02286498227963873)	2359 (0.034)	2548 (0.033)
eras	1	5110(0.011432491139819366)	#SAKNAS!	#SAKNAS!
erato	1	5110(0.011432491139819366)	2359 (0.034)	#SAKNAS!
erato	1	5110(0.011432491139819366)	2359 (0.034)	#SAKNAS!
erbb4	1	5110(0.011432491139819366)	#SAKNAS!	#SAKNAS!
erectile	1	5110(0.011432491139819366)	2359 (0.034)	#SAKNAS!
erectile	1	5110(0.011432491139819366)	2359 (0.034)	#SAKNAS!
erectus	1	3437(0.02286498227963873)	2359 (0.034)	2548 (0.033)
eretmochelys	1	5110(0.011432491139819366)	2359 (0.034)	#SAKNAS!
eretmochelys	1	5110(0.011432491139819366)	2359 (0.034)	#SAKNAS!
ergogenic	1	5110(0.011432491139819366)	#SAKNAS!	2548 (0.033)

eridania	1	5110(0.011432491139819366)	#SAKNAS!	2548 (0.033)
erosions	1	5110(0.011432491139819366)	2359 (0.034)	#SAKNAS!
erosions	1	5110(0.011432491139819366)	2359 (0.034)	#SAKNAS!
error	1	5110(0.011432491139819366)	#SAKNAS!	2548 (0.033)
errors	1	5110(0.011432491139819366)	#SAKNAS!	2548 (0.033)
erupted	1	5110(0.011432491139819366)	#SAKNAS!	#SAKNAS!
ervi	1	5110(0.011432491139819366)	2359 (0.034)	#SAKNAS!
ervi	1	5110(0.011432491139819366)	2359 (0.034)	#SAKNAS!
erythritoland	1	5110(0.011432491139819366)	#SAKNAS!	2548 (0.033)
erythrocyte	1	3437(0.02286498227963873)	2359 (0.034)	#SAKNAS!
es	1	5110(0.011432491139819366)	2359 (0.034)	#SAKNAS!
es	1	5110(0.011432491139819366)	2359 (0.034)	#SAKNAS!
escape	1	5110(0.011432491139819366)	#SAKNAS!	2548 (0.033)
esian	1	5110(0.011432491139819366)	#SAKNAS!	2548 (0.033)
esophageal-carcinoma	1	5110(0.011432491139819366)	2359 (0.034)	#SAKNAS!
esophageal-carcinoma	1	5110(0.011432491139819366)	2359 (0.034)	#SAKNAS!
esophageal-perforation	1	5110(0.011432491139819366)	2359 (0.034)	#SAKNAS!
esophageal-perforation	1	5110(0.011432491139819366)	2359 (0.034)	#SAKNAS!
esophagocardial	1	5110(0.011432491139819366)	2359 (0.034)	#SAKNAS!
esophagocardial	1	5110(0.011432491139819366)	2359 (0.034)	#SAKNAS!
esophagocoloplasty	1	3437(0.02286498227963873)	2359 (0.034)	2548 (0.033)
esophagojejunal	1	5110(0.011432491139819366)	2359 (0.034)	#SAKNAS!
esophagojejunal	1	5110(0.011432491139819366)	2359 (0.034)	#SAKNAS!
esophagojejunostomy	1	5110(0.011432491139819366)	#SAKNAS!	2548 (0.033)
esophagomyotomy	1	5110(0.011432491139819366)	2359 (0.034)	#SAKNAS!
esophagomyotomy	1	5110(0.011432491139819366)	2359 (0.034)	#SAKNAS!
esophagoplasty	1	5110(0.011432491139819366)	#SAKNAS!	2548 (0.033)
esophagoscope	1	5110(0.011432491139819366)	#SAKNAS!	2548 (0.033)
esophagoscopic	1	5110(0.011432491139819366)	2359 (0.034)	#SAKNAS!
esophagoscopic	1	5110(0.011432491139819366)	2359 (0.034)	#SAKNAS!
esophagoscopy	1	5110(0.011432491139819366)	#SAKNAS!	2548 (0.033)
esophagostomy	1	5110(0.011432491139819366)	#SAKNAS!	#SAKNAS!
esophagram	1	5110(0.011432491139819366)	#SAKNAS!	2548 (0.033)
esox	1	5110(0.011432491139819366)	#SAKNAS!	2548 (0.033)
espanola	1	5110(0.011432491139819366)	#SAKNAS!	2548 (0.033)
espghan-naspghan	1	5110(0.011432491139819366)	#SAKNAS!	2548 (0.033)
establishing	1	1894(0.05716245569909683)	2359 (0.034)	1668 (0.066)
establishments	1	5110(0.011432491139819366)	2359 (0.034)	#SAKNAS!
establishments	1	5110(0.011432491139819366)	2359 (0.034)	#SAKNAS!
esteem	1	5110(0.011432491139819366)	#SAKNAS!	#SAKNAS!
ester	1	5110(0.011432491139819366)	2359 (0.034)	#SAKNAS!
ester	1	5110(0.011432491139819366)	2359 (0.034)	#SAKNAS!
esterase	1	5110(0.011432491139819366)	#SAKNAS!	2548 (0.033)

estimate	1	1894(0.0571624556990968 3)	2359 (0.034)	2548 (0.033)
estimators	1	5110(0.011432491139819366)	#SAKNAS!	#SAKNAS!
estonia	1	5110(0.011432491139819366)	2359 (0.034)	#SAKNAS!
estonia	1	5110(0.011432491139819366)	2359 (0.034)	#SAKNAS!
estradiol	1	3437(0.02286498227963873)	2359 (0.034)	#SAKNAS!
estuarine	1	5110(0.011432491139819366)	2359 (0.034)	#SAKNAS!
estuarine	1	5110(0.011432491139819366)	2359 (0.034)	#SAKNAS!
estuary	1	5110(0.011432491139819366)	#SAKNAS!	2548 (0.033)
et-22	1	5110(0.011432491139819366)	#SAKNAS!	#SAKNAS!
ethanol	1	5110(0.011432491139819366)	#SAKNAS!	2548 (0.033)
ether	1	5110(0.011432491139819366)	#SAKNAS!	#SAKNAS!
ethics	1	2693(0.0342974734194581)	2359 (0.034)	2548 (0.033)
ethinyl	1	5110(0.011432491139819366)	#SAKNAS!	#SAKNAS!
ethiopia	1	836(0.16005487595747114)	2359 (0.034)	584 (0.23)
ethiopian	1	5110(0.011432491139819366)	2359 (0.034)	#SAKNAS!
ethiopian	1	5110(0.011432491139819366)	2359 (0.034)	#SAKNAS!
ethnically	1	5110(0.011432491139819366)	#SAKNAS!	#SAKNAS!
ethnicity	1	2693(0.0342974734194581)	2359 (0.034)	#SAKNAS!
ethnicity-based	1	5110(0.011432491139819366)	#SAKNAS!	#SAKNAS!
ethnodentistry	1	5110(0.011432491139819366)	#SAKNAS!	#SAKNAS!
ethylicin	1	5110(0.011432491139819366)	#SAKNAS!	#SAKNAS!
etiology	1	2214(0.04572996455927746)	2359 (0.034)	1233 (0.1)
etruscan	1	5110(0.011432491139819366)	2359 (0.034)	#SAKNAS!
etruscan	1	5110(0.011432491139819366)	2359 (0.034)	#SAKNAS!
etv5	1	3437(0.02286498227963873)	2359 (0.034)	2548 (0.033)
eucalypt-feeding	1	5110(0.011432491139819366)	2359 (0.034)	#SAKNAS!
eucalypt-feeding	1	5110(0.011432491139819366)	2359 (0.034)	#SAKNAS!
eucalyptus	1	5110(0.011432491139819366)	#SAKNAS!	2548 (0.033)
eugenii	1	5110(0.011432491139819366)	2359 (0.034)	#SAKNAS!
eugenii	1	5110(0.011432491139819366)	2359 (0.034)	#SAKNAS!
eulophus	1	5110(0.011432491139819366)	2359 (0.034)	#SAKNAS!
eulophus	1	5110(0.011432491139819366)	2359 (0.034)	#SAKNAS!
euproctidis	1	5110(0.011432491139819366)	2359 (0.034)	#SAKNAS!
euproctidis	1	5110(0.011432491139819366)	2359 (0.034)	#SAKNAS!
eur	1	5110(0.011432491139819366)	2359 (0.034)	#SAKNAS!
eur	1	5110(0.011432491139819366)	2359 (0.034)	#SAKNAS!
eurasian	1	5110(0.011432491139819366)	2359 (0.034)	#SAKNAS!
eurasian	1	5110(0.011432491139819366)	2359 (0.034)	#SAKNAS!
euryhaline	1	5110(0.011432491139819366)	#SAKNAS!	#SAKNAS!
eurytemora	1	5110(0.011432491139819366)	2359 (0.034)	#SAKNAS!
eurytemora	1	5110(0.011432491139819366)	2359 (0.034)	#SAKNAS!
eustachian	1	5110(0.011432491139819366)	#SAKNAS!	#SAKNAS!
euterpe	1	5110(0.011432491139819366)	#SAKNAS!	#SAKNAS!

euterpina-acutifrons	1	5110(0.011432491139819366)	2359 (0.034)	#SAKNAS!
euterpina-acutifrons	1	5110(0.011432491139819366)	2359 (0.034)	#SAKNAS!
eutrophic	1	3437(0.02286498227963873)	2359 (0.034)	#SAKNAS!
evacuation	1	5110(0.011432491139819366)	#SAKNAS!	#SAKNAS!
eva-jf	1	5110(0.011432491139819366)	#SAKNAS!	#SAKNAS!
ever	1	5110(0.011432491139819366)	#SAKNAS!	2548 (0.033)
every	1	3437(0.02286498227963873)	2359 (0.034)	#SAKNAS!
everyday	1	5110(0.011432491139819366)	#SAKNAS!	2548 (0.033)
evident	1	5110(0.011432491139819366)	#SAKNAS!	#SAKNAS!
ewes	1	1894(0.05716245569909683)	2359 (0.034)	1668 (0.066)
exacerbates	1	5110(0.011432491139819366)	#SAKNAS!	2548 (0.033)
exacerbation	1	2214(0.04572996455927746)	2359 (0.034)	1668 (0.066)
exacerbations	1	3437(0.02286498227963873)	2359 (0.034)	#SAKNAS!
examinations	1	5110(0.011432491139819366)	#SAKNAS!	2548 (0.033)
examinee	1	5110(0.011432491139819366)	#SAKNAS!	2548 (0.033)
examiner	1	5110(0.011432491139819366)	2359 (0.034)	#SAKNAS!
examiner	1	5110(0.011432491139819366)	2359 (0.034)	#SAKNAS!
excellent	1	5110(0.011432491139819366)	#SAKNAS!	2548 (0.033)
exception	1	5110(0.011432491139819366)	#SAKNAS!	#SAKNAS!
excesses	1	5110(0.011432491139819366)	2359 (0.034)	#SAKNAS!
excesses	1	5110(0.011432491139819366)	2359 (0.034)	#SAKNAS!
exchange	1	3437(0.02286498227963873)	2359 (0.034)	#SAKNAS!
excise	1	5110(0.011432491139819366)	2359 (0.034)	#SAKNAS!
excise	1	5110(0.011432491139819366)	2359 (0.034)	#SAKNAS!
excitability	1	5110(0.011432491139819366)	2359 (0.034)	#SAKNAS!
excitability	1	5110(0.011432491139819366)	2359 (0.034)	#SAKNAS!
excitation	1	5110(0.011432491139819366)	#SAKNAS!	2548 (0.033)
excitatory	1	5110(0.011432491139819366)	#SAKNAS!	2548 (0.033)
exclusively	1	5110(0.011432491139819366)	2359 (0.034)	#SAKNAS!
exclusively	1	5110(0.011432491139819366)	2359 (0.034)	#SAKNAS!
exercises	1	1678(0.0685949468389162)	2359 (0.034)	1233 (0.1)
exert	1	5110(0.011432491139819366)	#SAKNAS!	2548 (0.033)
exfoliation	1	5110(0.011432491139819366)	#SAKNAS!	2548 (0.033)
exhibit	1	5110(0.011432491139819366)	#SAKNAS!	2548 (0.033)
exhibits	1	5110(0.011432491139819366)	#SAKNAS!	#SAKNAS!
existence	1	5110(0.011432491139819366)	#SAKNAS!	#SAKNAS!
exocrine	1	5110(0.011432491139819366)	#SAKNAS!	2548 (0.033)
exogenous	1	5110(0.011432491139819366)	#SAKNAS!	2548 (0.033)
exome	1	5110(0.011432491139819366)	#SAKNAS!	2548 (0.033)
exopeptidase	1	5110(0.011432491139819366)	#SAKNAS!	2548 (0.033)
exophiala	1	5110(0.011432491139819366)	2359 (0.034)	#SAKNAS!
exophiala	1	5110(0.011432491139819366)	2359 (0.034)	#SAKNAS!
exophthalmus	1	5110(0.011432491139819366)	2359 (0.034)	#SAKNAS!

exophthalmus	1	5110(0.011432491139819366)	2359 (0.034)	#SAKNAS!
exosomes	1	5110(0.011432491139819366)	#SAKNAS!	2548 (0.033)
expandable	1	5110(0.011432491139819366)	2359 (0.034)	#SAKNAS!
expandable	1	5110(0.011432491139819366)	2359 (0.034)	#SAKNAS!
expectancy	1	5110(0.011432491139819366)	#SAKNAS!	2548 (0.033)
expenditures	1	3437(0.02286498227963873)	2359 (0.034)	2548 (0.033)
expenses	1	5110(0.011432491139819366)	#SAKNAS!	2548 (0.033)
experiment	1	2693(0.0342974734194581)	2359 (0.034)	2548 (0.033)
experimentally	1	5110(0.011432491139819366)	#SAKNAS!	#SAKNAS!
experimental-study	1	5110(0.011432491139819366)	2359 (0.034)	#SAKNAS!
experimental-study	1	5110(0.011432491139819366)	2359 (0.034)	#SAKNAS!
experts	1	5110(0.011432491139819366)	2359 (0.034)	#SAKNAS!
experts	1	5110(0.011432491139819366)	2359 (0.034)	#SAKNAS!
explain	1	2214(0.04572996455927746)	2359 (0.034)	2548 (0.033)
explainable	1	5110(0.011432491139819366)	#SAKNAS!	#SAKNAS!
explaining	1	3437(0.02286498227963873)	2359 (0.034)	#SAKNAS!
explanations	1	5110(0.011432491139819366)	2359 (0.034)	#SAKNAS!
explanations	1	5110(0.011432491139819366)	2359 (0.034)	#SAKNAS!
explantation	1	5110(0.011432491139819366)	#SAKNAS!	2548 (0.033)
explantion	1	5110(0.011432491139819366)	2359 (0.034)	#SAKNAS!
explantion	1	5110(0.011432491139819366)	2359 (0.034)	#SAKNAS!
exploit	1	5110(0.011432491139819366)	#SAKNAS!	2548 (0.033)
explorative	1	1894(0.05716245569909683)	2359 (0.034)	1668 (0.066)
explore	1	5110(0.011432491139819366)	#SAKNAS!	2548 (0.033)
expressions	1	5110(0.011432491139819366)	#SAKNAS!	2548 (0.033)
ex-q	1	5110(0.011432491139819366)	#SAKNAS!	2548 (0.033)
extant	1	5110(0.011432491139819366)	#SAKNAS!	2548 (0.033)
extended-care	1	5110(0.011432491139819366)	2359 (0.034)	#SAKNAS!
extended-care	1	5110(0.011432491139819366)	2359 (0.034)	#SAKNAS!
extended-release	1	5110(0.011432491139819366)	#SAKNAS!	2548 (0.033)
extends	1	5110(0.011432491139819366)	#SAKNAS!	2548 (0.033)
extensive	1	5110(0.011432491139819366)	2359 (0.034)	#SAKNAS!
extensive	1	5110(0.011432491139819366)	2359 (0.034)	#SAKNAS!
external	1	1678(0.0685949468389162)	2359 (0.034)	2548 (0.033)
extracorporeal	1	5110(0.011432491139819366)	#SAKNAS!	#SAKNAS!
extractions	1	5110(0.011432491139819366)	#SAKNAS!	#SAKNAS!
extraordinary	1	5110(0.011432491139819366)	2359 (0.034)	#SAKNAS!
extraordinary	1	5110(0.011432491139819366)	2359 (0.034)	#SAKNAS!
extrapontine	1	5110(0.011432491139819366)	2359 (0.034)	#SAKNAS!
extrapontine	1	5110(0.011432491139819366)	2359 (0.034)	#SAKNAS!
extremely	1	5110(0.011432491139819366)	#SAKNAS!	#SAKNAS!
extremis	1	5110(0.011432491139819366)	#SAKNAS!	2548 (0.033)
extrinsic	1	5110(0.011432491139819366)	2359 (0.034)	#SAKNAS!

extrinsic	1	5110(0.011432491139819366)	2359 (0.034)	#SAKNAS!
extrused	1	5110(0.011432491139819366)	#SAKNAS!	2548 (0.033)
ex-vivo	1	5110(0.011432491139819366)	#SAKNAS!	#SAKNAS!
eye	1	5110(0.011432491139819366)	2359 (0.034)	#SAKNAS!
eye	1	5110(0.011432491139819366)	2359 (0.034)	#SAKNAS!
fabrication	1	5110(0.011432491139819366)	#SAKNAS!	2548 (0.033)
face	1	5110(0.011432491139819366)	#SAKNAS!	2548 (0.033)
face-to-face	1	5110(0.011432491139819366)	#SAKNAS!	#SAKNAS!
facilitation	1	5110(0.011432491139819366)	#SAKNAS!	#SAKNAS!
facilitators	1	2214(0.04572996455927746)	2359 (0.034)	#SAKNAS!
facilitatory	1	5110(0.011432491139819366)	#SAKNAS!	#SAKNAS!
factor-alpha	1	5110(0.011432491139819366)	#SAKNAS!	2548 (0.033)
factorial	1	5110(0.011432491139819366)	#SAKNAS!	#SAKNAS!
factors-a	1	5110(0.011432491139819366)	#SAKNAS!	#SAKNAS!
factory	1	5110(0.011432491139819366)	2359 (0.034)	#SAKNAS!
factory	1	5110(0.011432491139819366)	2359 (0.034)	#SAKNAS!
fadoi	1	5110(0.011432491139819366)	#SAKNAS!	#SAKNAS!
fads	1	5110(0.011432491139819366)	2359 (0.034)	#SAKNAS!
fads	1	5110(0.011432491139819366)	2359 (0.034)	#SAKNAS!
faecal-ft-nirs	1	5110(0.011432491139819366)	#SAKNAS!	2548 (0.033)
faeces	1	5110(0.011432491139819366)	#SAKNAS!	2548 (0.033)
faers	1	5110(0.011432491139819366)	#SAKNAS!	#SAKNAS!
fail	1	5110(0.011432491139819366)	2359 (0.034)	#SAKNAS!
fail	1	5110(0.011432491139819366)	2359 (0.034)	#SAKNAS!
fails	1	5110(0.011432491139819366)	#SAKNAS!	2548 (0.033)
failure-results	1	5110(0.011432491139819366)	#SAKNAS!	#SAKNAS!
failures	1	5110(0.011432491139819366)	#SAKNAS!	2548 (0.033)
failure-to-thrive	1	5110(0.011432491139819366)	2359 (0.034)	#SAKNAS!
failure-to-thrive	1	5110(0.011432491139819366)	2359 (0.034)	#SAKNAS!
faim2	1	5110(0.011432491139819366)	2359 (0.034)	#SAKNAS!
faim2	1	5110(0.011432491139819366)	2359 (0.034)	#SAKNAS!
fairule	1	5110(0.011432491139819366)	2359 (0.034)	#SAKNAS!
fairule	1	5110(0.011432491139819366)	2359 (0.034)	#SAKNAS!
faisalabad	1	5110(0.011432491139819366)	#SAKNAS!	#SAKNAS!
falcon	1	5110(0.011432491139819366)	#SAKNAS!	#SAKNAS!
faltering	1	5110(0.011432491139819366)	2359 (0.034)	#SAKNAS!
faltering	1	5110(0.011432491139819366)	2359 (0.034)	#SAKNAS!
familiar	1	5110(0.011432491139819366)	2359 (0.034)	#SAKNAS!
familiar	1	5110(0.011432491139819366)	2359 (0.034)	#SAKNAS!
familiaris	1	5110(0.011432491139819366)	2359 (0.034)	#SAKNAS!
familiaris	1	5110(0.011432491139819366)	2359 (0.034)	#SAKNAS!
familiarity	1	5110(0.011432491139819366)	#SAKNAS!	2548 (0.033)
family-	1	5110(0.011432491139819366)	#SAKNAS!	#SAKNAS!

family-centered	1	5110(0.011432491139819366)	#SAKNAS!	2548 (0.033)
family-style	1	5110(0.011432491139819366)	#SAKNAS!	#SAKNAS!
fangsiao	1	5110(0.011432491139819366)	#SAKNAS!	#SAKNAS!
farming	1	2693(0.0342974734194581)	2359 (0.034)	2548 (0.033)
farmland	1	5110(0.011432491139819366)	#SAKNAS!	2548 (0.033)
farnese	1	5110(0.011432491139819366)	#SAKNAS!	#SAKNAS!
farrowing	1	5110(0.011432491139819366)	#SAKNAS!	#SAKNAS!
fars	1	5110(0.011432491139819366)	#SAKNAS!	2548 (0.033)
fars-iran	1	5110(0.011432491139819366)	#SAKNAS!	#SAKNAS!
fascia	1	5110(0.011432491139819366)	#SAKNAS!	#SAKNAS!
fasciitis	1	5110(0.011432491139819366)	2359 (0.034)	#SAKNAS!
fasciitis	1	5110(0.011432491139819366)	2359 (0.034)	#SAKNAS!
faso	1	5110(0.011432491139819366)	#SAKNAS!	2548 (0.033)
fast-changing	1	5110(0.011432491139819366)	#SAKNAS!	2548 (0.033)
fasting-induced	1	5110(0.011432491139819366)	#SAKNAS!	2548 (0.033)
fate	1	5110(0.011432491139819366)	#SAKNAS!	2548 (0.033)
fat-free	1	5110(0.011432491139819366)	#SAKNAS!	2548 (0.033)
fatty-acids	1	5110(0.011432491139819366)	2359 (0.034)	#SAKNAS!
fatty-acids	1	5110(0.011432491139819366)	2359 (0.034)	#SAKNAS!
favor	1	3437(0.02286498227963873)	2359 (0.034)	2548 (0.033)
favorite	1	5110(0.011432491139819366)	#SAKNAS!	#SAKNAS!
fawns	1	5110(0.011432491139819366)	2359 (0.034)	#SAKNAS!
fawns	1	5110(0.011432491139819366)	2359 (0.034)	#SAKNAS!
fctors	1	5110(0.011432491139819366)	#SAKNAS!	#SAKNAS!
fda	1	5110(0.011432491139819366)	#SAKNAS!	#SAKNAS!
feasible	1	2693(0.0342974734194581)	2359 (0.034)	1668 (0.066)
feather	1	5110(0.011432491139819366)	#SAKNAS!	2548 (0.033)
feathers	1	5110(0.011432491139819366)	2359 (0.034)	#SAKNAS!
feathers	1	5110(0.011432491139819366)	2359 (0.034)	#SAKNAS!
federal	1	5110(0.011432491139819366)	2359 (0.034)	#SAKNAS!
federal	1	5110(0.011432491139819366)	2359 (0.034)	#SAKNAS!
feedback	1	3437(0.02286498227963873)	2359 (0.034)	2548 (0.033)
feed-deprivation	1	5110(0.011432491139819366)	#SAKNAS!	2548 (0.033)
feed-driven	1	5110(0.011432491139819366)	2359 (0.034)	#SAKNAS!
feed-driven	1	5110(0.011432491139819366)	2359 (0.034)	#SAKNAS!
feeder	1	2693(0.0342974734194581)	2359 (0.034)	2548 (0.033)
feeding-an	1	5110(0.011432491139819366)	#SAKNAS!	#SAKNAS!
feeding-habits	1	5110(0.011432491139819366)	2359 (0.034)	#SAKNAS!
feeding-habits	1	5110(0.011432491139819366)	2359 (0.034)	#SAKNAS!
feeding-response	1	5110(0.011432491139819366)	#SAKNAS!	#SAKNAS!
feedings	1	3437(0.02286498227963873)	2359 (0.034)	2548 (0.033)
feeding-shifts	1	5110(0.011432491139819366)	#SAKNAS!	2548 (0.033)
feedlot	1	1111(0.11432491139819366)	2359 (0.034)	999 (0.13)

feedstuffs	1	5110(0.011432491139819366)	#SAKNAS!	2548 (0.033)
feeling	1	5110(0.011432491139819366)	#SAKNAS!	2548 (0.033)
feelings	1	5110(0.011432491139819366)	2359 (0.034)	#SAKNAS!
feelings	1	5110(0.011432491139819366)	2359 (0.034)	#SAKNAS!
fees	1	2214(0.04572996455927746)	2359 (0.034)	2548 (0.033)
feira	1	5110(0.011432491139819366)	#SAKNAS!	2548 (0.033)
felis	1	5110(0.011432491139819366)	2359 (0.034)	#SAKNAS!
felis	1	5110(0.011432491139819366)	2359 (0.034)	#SAKNAS!
femoral	1	5110(0.011432491139819366)	#SAKNAS!	#SAKNAS!
fenfluramine	1	5110(0.011432491139819366)	2359 (0.034)	#SAKNAS!
fenfluramine	1	5110(0.011432491139819366)	2359 (0.034)	#SAKNAS!
fenland	1	5110(0.011432491139819366)	#SAKNAS!	2548 (0.033)
fenoxycarb	1	5110(0.011432491139819366)	2359 (0.034)	#SAKNAS!
fenoxycarb	1	5110(0.011432491139819366)	2359 (0.034)	#SAKNAS!
fentanyl	1	5110(0.011432491139819366)	#SAKNAS!	2548 (0.033)
ferritin	1	5110(0.011432491139819366)	#SAKNAS!	2548 (0.033)
fertiliser	1	5110(0.011432491139819366)	#SAKNAS!	#SAKNAS!
fertilitat	1	5110(0.011432491139819366)	2359 (0.034)	#SAKNAS!
fertilitat	1	5110(0.011432491139819366)	2359 (0.034)	#SAKNAS!
fertilization	1	2214(0.04572996455927746)	2359 (0.034)	1668 (0.066)
fescue	1	5110(0.011432491139819366)	2359 (0.034)	#SAKNAS!
fescue	1	5110(0.011432491139819366)	2359 (0.034)	#SAKNAS!
fever	1	5110(0.011432491139819366)	#SAKNAS!	2548 (0.033)
fi	1	5110(0.011432491139819366)	#SAKNAS!	#SAKNAS!
fiberoptic	1	1894(0.05716245569909683)	2359 (0.034)	2548 (0.033)
fibers	1	5110(0.011432491139819366)	#SAKNAS!	#SAKNAS!
fibre-optic	1	5110(0.011432491139819366)	#SAKNAS!	2548 (0.033)
fibrillary	1	5110(0.011432491139819366)	#SAKNAS!	#SAKNAS!
fibrin	1	3437(0.02286498227963873)	2359 (0.034)	#SAKNAS!
fibroblast	1	5110(0.011432491139819366)	#SAKNAS!	#SAKNAS!
fibroblasts	1	5110(0.011432491139819366)	#SAKNAS!	2548 (0.033)
fibrolytic	1	5110(0.011432491139819366)	#SAKNAS!	#SAKNAS!
fibrosis-related	1	5110(0.011432491139819366)	#SAKNAS!	#SAKNAS!
fibula	1	5110(0.011432491139819366)	#SAKNAS!	#SAKNAS!
fiddler-crabs	1	5110(0.011432491139819366)	2359 (0.034)	#SAKNAS!
fiddler-crabs	1	5110(0.011432491139819366)	2359 (0.034)	#SAKNAS!
fidelity	1	5110(0.011432491139819366)	2359 (0.034)	#SAKNAS!
fidelity	1	5110(0.011432491139819366)	2359 (0.034)	#SAKNAS!
fields	1	5110(0.011432491139819366)	2359 (0.034)	#SAKNAS!
fields	1	5110(0.011432491139819366)	2359 (0.034)	#SAKNAS!
fifteen-year-old	1	5110(0.011432491139819366)	#SAKNAS!	2548 (0.033)
fifth-instar	1	5110(0.011432491139819366)	#SAKNAS!	2548 (0.033)
fight	1	5110(0.011432491139819366)	#SAKNAS!	2548 (0.033)

fiji	1	5110(0.011432491139819366)	#SAKNAS!	#SAKNAS!
filefish	1	5110(0.011432491139819366)	#SAKNAS!	#SAKNAS!
filiformis	1	5110(0.011432491139819366)	2359 (0.034)	#SAKNAS!
filiformis	1	5110(0.011432491139819366)	2359 (0.034)	#SAKNAS!
fill	1	5110(0.011432491139819366)	2359 (0.034)	#SAKNAS!
fill	1	5110(0.011432491139819366)	2359 (0.034)	#SAKNAS!
films	1	5110(0.011432491139819366)	#SAKNAS!	#SAKNAS!
final	1	5110(0.011432491139819366)	#SAKNAS!	#SAKNAS!
financial	1	5110(0.011432491139819366)	#SAKNAS!	#SAKNAS!
fine-mapping	1	5110(0.011432491139819366)	#SAKNAS!	2548 (0.033)
finger	1	5110(0.011432491139819366)	#SAKNAS!	#SAKNAS!
fingerprint	1	5110(0.011432491139819366)	#SAKNAS!	2548 (0.033)
finland	1	1219(0.1028924202583743)	2359 (0.034)	999 (0.13)
finnish	1	1678(0.0685949468389162)	2359 (0.034)	1233 (0.1)
finoral	1	5110(0.011432491139819366)	#SAKNAS!	#SAKNAS!
finut	1	5110(0.011432491139819366)	#SAKNAS!	2548 (0.033)
fire-induced	1	5110(0.011432491139819366)	2359 (0.034)	#SAKNAS!
fire-induced	1	5110(0.011432491139819366)	2359 (0.034)	#SAKNAS!
firmicutes	1	5110(0.011432491139819366)	#SAKNAS!	#SAKNAS!
firmus	1	5110(0.011432491139819366)	#SAKNAS!	2548 (0.033)
first-ever	1	5110(0.011432491139819366)	#SAKNAS!	2548 (0.033)
first-line	1	5110(0.011432491139819366)	#SAKNAS!	2548 (0.033)
first-time	1	3437(0.02286498227963873)	2359 (0.034)	2548 (0.033)
first-wave	1	5110(0.011432491139819366)	#SAKNAS!	#SAKNAS!
first-year	1	5110(0.011432491139819366)	#SAKNAS!	2548 (0.033)
fisher-hunter-gatherers	1	5110(0.011432491139819366)	#SAKNAS!	2548 (0.033)
fishery	1	5110(0.011432491139819366)	#SAKNAS!	2548 (0.033)
fishing	1	5110(0.011432491139819366)	#SAKNAS!	#SAKNAS!
fishmeal	1	3437(0.02286498227963873)	2359 (0.034)	#SAKNAS!
fishmeal-free	1	5110(0.011432491139819366)	#SAKNAS!	#SAKNAS!
fissure	1	5110(0.011432491139819366)	#SAKNAS!	2548 (0.033)
fistulas	1	5110(0.011432491139819366)	#SAKNAS!	#SAKNAS!
fit	1	3437(0.02286498227963873)	2359 (0.034)	#SAKNAS!
fit4treatment	1	5110(0.011432491139819366)	#SAKNAS!	#SAKNAS!
fits	1	3437(0.02286498227963873)	2359 (0.034)	2548 (0.033)
fitting	1	5110(0.011432491139819366)	2359 (0.034)	#SAKNAS!
fitting	1	5110(0.011432491139819366)	2359 (0.034)	#SAKNAS!
five-item	1	5110(0.011432491139819366)	#SAKNAS!	#SAKNAS!
five-year	1	2693(0.0342974734194581)	2359 (0.034)	#SAKNAS!
five-year-old	1	5110(0.011432491139819366)	2359 (0.034)	#SAKNAS!
five-year-old	1	5110(0.011432491139819366)	2359 (0.034)	#SAKNAS!
fjord	1	5110(0.011432491139819366)	#SAKNAS!	2548 (0.033)
flaccid	1	5110(0.011432491139819366)	#SAKNAS!	#SAKNAS!

flagellate	1	5110(0.011432491139819366)	2359 (0.034)	#SAKNAS!
flagellate	1	5110(0.011432491139819366)	2359 (0.034)	#SAKNAS!
flagellates	1	5110(0.011432491139819366)	2359 (0.034)	#SAKNAS!
flagellates	1	5110(0.011432491139819366)	2359 (0.034)	#SAKNAS!
flags	1	5110(0.011432491139819366)	#SAKNAS!	2548 (0.033)
flamingo	1	5110(0.011432491139819366)	#SAKNAS!	#SAKNAS!
flat	1	5110(0.011432491139819366)	#SAKNAS!	#SAKNAS!
flatfish	1	5110(0.011432491139819366)	2359 (0.034)	#SAKNAS!
flatfish	1	5110(0.011432491139819366)	2359 (0.034)	#SAKNAS!
flavescens	1	5110(0.011432491139819366)	#SAKNAS!	2548 (0.033)
flavonoid	1	5110(0.011432491139819366)	#SAKNAS!	2548 (0.033)
flavonoids	1	2693(0.0342974734194581)	2359 (0.034)	#SAKNAS!
flavored	1	5110(0.011432491139819366)	#SAKNAS!	#SAKNAS!
flavour	1	5110(0.011432491139819366)	#SAKNAS!	2548 (0.033)
flesh	1	5110(0.011432491139819366)	#SAKNAS!	2548 (0.033)
flipbook	1	5110(0.011432491139819366)	#SAKNAS!	2548 (0.033)
floc	1	5110(0.011432491139819366)	2359 (0.034)	#SAKNAS!
floc	1	5110(0.011432491139819366)	2359 (0.034)	#SAKNAS!
flood	1	5110(0.011432491139819366)	#SAKNAS!	#SAKNAS!
floppy	1	5110(0.011432491139819366)	2359 (0.034)	#SAKNAS!
floppy	1	5110(0.011432491139819366)	2359 (0.034)	#SAKNAS!
flora	1	3437(0.02286498227963873)	2359 (0.034)	#SAKNAS!
florence	1	5110(0.011432491139819366)	#SAKNAS!	2548 (0.033)
floss	1	5110(0.011432491139819366)	#SAKNAS!	2548 (0.033)
flossing	1	2214(0.04572996455927746)	2359 (0.034)	1668 (0.066)
flour	1	3437(0.02286498227963873)	2359 (0.034)	#SAKNAS!
flowchart	1	5110(0.011432491139819366)	#SAKNAS!	#SAKNAS!
flower	1	2693(0.0342974734194581)	2359 (0.034)	2548 (0.033)
flower-visiting	1	5110(0.011432491139819366)	2359 (0.034)	#SAKNAS!
flower-visiting	1	5110(0.011432491139819366)	2359 (0.034)	#SAKNAS!
fluctuate	1	5110(0.011432491139819366)	2359 (0.034)	#SAKNAS!
fluctuate	1	5110(0.011432491139819366)	2359 (0.034)	#SAKNAS!
fluctuating	1	5110(0.011432491139819366)	2359 (0.034)	#SAKNAS!
fluctuating	1	5110(0.011432491139819366)	2359 (0.034)	#SAKNAS!
flume	1	5110(0.011432491139819366)	2359 (0.034)	#SAKNAS!
flume	1	5110(0.011432491139819366)	2359 (0.034)	#SAKNAS!
fluorescens	1	5110(0.011432491139819366)	2359 (0.034)	#SAKNAS!
fluorescens	1	5110(0.011432491139819366)	2359 (0.034)	#SAKNAS!
fluorides	1	5110(0.011432491139819366)	#SAKNAS!	#SAKNAS!
fluoriferous	1	5110(0.011432491139819366)	2359 (0.034)	#SAKNAS!
fluoriferous	1	5110(0.011432491139819366)	2359 (0.034)	#SAKNAS!
fluoroscopic	1	3437(0.02286498227963873)	2359 (0.034)	#SAKNAS!
fluoroscopic-guided	1	5110(0.011432491139819366)	#SAKNAS!	2548 (0.033)

fluoroscopy	1	3437(0.02286498227963873)	2359 (0.034)	#SAKNAS!
fluorouracil	1	5110(0.011432491139819366)	#SAKNAS!	2548 (0.033)
flushes-a	1	5110(0.011432491139819366)	#SAKNAS!	#SAKNAS!
flying	1	5110(0.011432491139819366)	#SAKNAS!	#SAKNAS!
foals	1	5110(0.011432491139819366)	#SAKNAS!	2548 (0.033)
foam	1	5110(0.011432491139819366)	#SAKNAS!	#SAKNAS!
foams	1	5110(0.011432491139819366)	#SAKNAS!	#SAKNAS!
focus	1	1678(0.0685949468389162)	2359 (0.034)	1668 (0.066)
focusing	1	5110(0.011432491139819366)	2359 (0.034)	#SAKNAS!
focusing	1	5110(0.011432491139819366)	2359 (0.034)	#SAKNAS!
fois	1	5110(0.011432491139819366)	#SAKNAS!	2548 (0.033)
fois-it	1	5110(0.011432491139819366)	#SAKNAS!	#SAKNAS!
foliar	1	5110(0.011432491139819366)	#SAKNAS!	#SAKNAS!
folic	1	2693(0.0342974734194581)	2359 (0.034)	#SAKNAS!
folivore	1	3437(0.02286498227963873)	2359 (0.034)	2548 (0.033)
folivorous	1	3437(0.02286498227963873)	2359 (0.034)	2548 (0.033)
folk	1	5110(0.011432491139819366)	#SAKNAS!	2548 (0.033)
follow	1	1678(0.0685949468389162)	2359 (0.034)	816 (0.16)
follows	1	5110(0.011432491139819366)	#SAKNAS!	2548 (0.033)
follow-ups	1	5110(0.011432491139819366)	#SAKNAS!	2548 (0.033)
food4toddlers	1	5110(0.011432491139819366)	#SAKNAS!	2548 (0.033)
food-carbohydrate	1	5110(0.011432491139819366)	2359 (0.034)	#SAKNAS!
food-carbohydrate	1	5110(0.011432491139819366)	2359 (0.034)	#SAKNAS!
food-industry	1	5110(0.011432491139819366)	2359 (0.034)	#SAKNAS!
food-industry	1	5110(0.011432491139819366)	2359 (0.034)	#SAKNAS!
food-intake	1	5110(0.011432491139819366)	2359 (0.034)	#SAKNAS!
food-intake	1	5110(0.011432491139819366)	2359 (0.034)	#SAKNAS!
food-restricted	1	5110(0.011432491139819366)	2359 (0.034)	#SAKNAS!
food-restricted	1	5110(0.011432491139819366)	2359 (0.034)	#SAKNAS!
foodscape	1	5110(0.011432491139819366)	#SAKNAS!	2548 (0.033)
food-web	1	5110(0.011432491139819366)	2359 (0.034)	#SAKNAS!
food-web	1	5110(0.011432491139819366)	2359 (0.034)	#SAKNAS!
footballers	1	5110(0.011432491139819366)	#SAKNAS!	#SAKNAS!
foothills	1	5110(0.011432491139819366)	#SAKNAS!	#SAKNAS!
footprint	1	5110(0.011432491139819366)	2359 (0.034)	#SAKNAS!
footprint	1	5110(0.011432491139819366)	2359 (0.034)	#SAKNAS!
fora	1	5110(0.011432491139819366)	#SAKNAS!	2548 (0.033)
forage-based	1	5110(0.011432491139819366)	2359 (0.034)	#SAKNAS!
forage-based	1	5110(0.011432491139819366)	2359 (0.034)	#SAKNAS!
forager	1	3437(0.02286498227963873)	2359 (0.034)	2548 (0.033)
forager-farmers	1	5110(0.011432491139819366)	#SAKNAS!	#SAKNAS!
foragers	1	2693(0.0342974734194581)	2359 (0.034)	1668 (0.066)
foramen	1	5110(0.011432491139819366)	#SAKNAS!	2548 (0.033)

foraminatus	1	5110(0.011432491139819366)	#SAKNAS!	#SAKNAS!
foraminifera	1	5110(0.011432491139819366)	#SAKNAS!	#SAKNAS!
forces	1	2693(0.0342974734194581)	2359 (0.034)	#SAKNAS!
foreign	1	3437(0.02286498227963873)	2359 (0.034)	2548 (0.033)
forest-dwelling	1	5110(0.011432491139819366)	2359 (0.034)	#SAKNAS!
forest-dwelling	1	5110(0.011432491139819366)	2359 (0.034)	#SAKNAS!
forestiers	1	5110(0.011432491139819366)	2359 (0.034)	#SAKNAS!
forestiers	1	5110(0.011432491139819366)	2359 (0.034)	#SAKNAS!
forests	1	2693(0.0342974734194581)	2359 (0.034)	2548 (0.033)
forest-savanna	1	5110(0.011432491139819366)	#SAKNAS!	#SAKNAS!
forget	1	5110(0.011432491139819366)	2359 (0.034)	#SAKNAS!
forget	1	5110(0.011432491139819366)	2359 (0.034)	#SAKNAS!
formal	1	5110(0.011432491139819366)	#SAKNAS!	#SAKNAS!
formats	1	5110(0.011432491139819366)	#SAKNAS!	2548 (0.033)
former	1	5110(0.011432491139819366)	#SAKNAS!	2548 (0.033)
formicidae	1	5110(0.011432491139819366)	#SAKNAS!	#SAKNAS!
formosan	1	5110(0.011432491139819366)	2359 (0.034)	#SAKNAS!
formosan	1	5110(0.011432491139819366)	2359 (0.034)	#SAKNAS!
formulae	1	5110(0.011432491139819366)	#SAKNAS!	#SAKNAS!
formula-fed	1	5110(0.011432491139819366)	2359 (0.034)	#SAKNAS!
formula-fed	1	5110(0.011432491139819366)	2359 (0.034)	#SAKNAS!
formula-feeding	1	5110(0.011432491139819366)	2359 (0.034)	#SAKNAS!
formula-feeding	1	5110(0.011432491139819366)	2359 (0.034)	#SAKNAS!
formulas	1	2214(0.04572996455927746)	2359 (0.034)	2548 (0.033)
formulating	1	5110(0.011432491139819366)	#SAKNAS!	#SAKNAS!
forpus	1	5110(0.011432491139819366)	2359 (0.034)	#SAKNAS!
forpus	1	5110(0.011432491139819366)	2359 (0.034)	#SAKNAS!
fortification	1	5110(0.011432491139819366)	#SAKNAS!	#SAKNAS!
fortifying	1	5110(0.011432491139819366)	2359 (0.034)	#SAKNAS!
fortifying	1	5110(0.011432491139819366)	2359 (0.034)	#SAKNAS!
forty-year	1	5110(0.011432491139819366)	#SAKNAS!	2548 (0.033)
forward	1	5110(0.011432491139819366)	#SAKNAS!	#SAKNAS!
fos-cells	1	5110(0.011432491139819366)	#SAKNAS!	2548 (0.033)
fossa	1	5110(0.011432491139819366)	#SAKNAS!	#SAKNAS!
fossil	1	3437(0.02286498227963873)	2359 (0.034)	2548 (0.033)
foster	1	5110(0.011432491139819366)	#SAKNAS!	2548 (0.033)
foundation	1	5110(0.011432491139819366)	2359 (0.034)	#SAKNAS!
foundation	1	5110(0.011432491139819366)	2359 (0.034)	#SAKNAS!
four-to-six	1	5110(0.011432491139819366)	#SAKNAS!	2548 (0.033)
fowls	1	5110(0.011432491139819366)	2359 (0.034)	#SAKNAS!
fowls	1	5110(0.011432491139819366)	2359 (0.034)	#SAKNAS!
fq	1	5110(0.011432491139819366)	#SAKNAS!	#SAKNAS!
fractal	1	5110(0.011432491139819366)	#SAKNAS!	#SAKNAS!

fraction	1	5110(0.011432491139819366)	#SAKNAS!	2548 (0.033)
fractional	1	5110(0.011432491139819366)	2359 (0.034)	#SAKNAS!
fractional	1	5110(0.011432491139819366)	2359 (0.034)	#SAKNAS!
fractions	1	5110(0.011432491139819366)	2359 (0.034)	#SAKNAS!
fractions	1	5110(0.011432491139819366)	2359 (0.034)	#SAKNAS!
fracture	1	1485(0.08002743797873557)	2359 (0.034)	1233 (0.1)
fragiless	1	5110(0.011432491139819366)	#SAKNAS!	#SAKNAS!
fragment	1	5110(0.011432491139819366)	2359 (0.034)	#SAKNAS!
fragment	1	5110(0.011432491139819366)	2359 (0.034)	#SAKNAS!
fragmentation	1	5110(0.011432491139819366)	2359 (0.034)	#SAKNAS!
fragmentation	1	5110(0.011432491139819366)	2359 (0.034)	#SAKNAS!
frail	1	1335(0.09145992911855493)	2359 (0.034)	816 (0.16)
franc	1	5110(0.011432491139819366)	2359 (0.034)	#SAKNAS!
franc	1	5110(0.011432491139819366)	2359 (0.034)	#SAKNAS!
franciscan	1	5110(0.011432491139819366)	#SAKNAS!	2548 (0.033)
fratercula	1	5110(0.011432491139819366)	2359 (0.034)	#SAKNAS!
fratercula	1	5110(0.011432491139819366)	2359 (0.034)	#SAKNAS!
frax	1	5110(0.011432491139819366)	#SAKNAS!	2548 (0.033)
fraxinus	1	5110(0.011432491139819366)	2359 (0.034)	#SAKNAS!
fraxinus	1	5110(0.011432491139819366)	2359 (0.034)	#SAKNAS!
free-choice	1	2693(0.0342974734194581)	2359 (0.034)	2548 (0.033)
free-end	1	5110(0.011432491139819366)	#SAKNAS!	2548 (0.033)
free-flap	1	5110(0.011432491139819366)	#SAKNAS!	#SAKNAS!
free-range	1	5110(0.011432491139819366)	#SAKNAS!	2548 (0.033)
freezing	1	5110(0.011432491139819366)	#SAKNAS!	#SAKNAS!
french	1	1485(0.08002743797873557)	2359 (0.034)	999 (0.13)
frequencies	1	5110(0.011432491139819366)	2359 (0.034)	#SAKNAS!
frequencies	1	5110(0.011432491139819366)	2359 (0.034)	#SAKNAS!
frequency-dependent	1	5110(0.011432491139819366)	2359 (0.034)	#SAKNAS!
frequency-dependent	1	5110(0.011432491139819366)	2359 (0.034)	#SAKNAS!
frequent	1	2693(0.0342974734194581)	2359 (0.034)	2548 (0.033)
frequently	1	5110(0.011432491139819366)	2359 (0.034)	#SAKNAS!
frequently	1	5110(0.011432491139819366)	2359 (0.034)	#SAKNAS!
freshly	1	5110(0.011432491139819366)	#SAKNAS!	#SAKNAS!
freshman	1	5110(0.011432491139819366)	2359 (0.034)	#SAKNAS!
freshman	1	5110(0.011432491139819366)	2359 (0.034)	#SAKNAS!
freshmen	1	3437(0.02286498227963873)	2359 (0.034)	#SAKNAS!
freshwater	1	2214(0.04572996455927746)	2359 (0.034)	1233 (0.1)
friderici	1	5110(0.011432491139819366)	2359 (0.034)	#SAKNAS!
friderici	1	5110(0.011432491139819366)	2359 (0.034)	#SAKNAS!
frondosa	1	5110(0.011432491139819366)	#SAKNAS!	2548 (0.033)
frontier	1	5110(0.011432491139819366)	#SAKNAS!	2548 (0.033)
front-of-pack	1	5110(0.011432491139819366)	#SAKNAS!	#SAKNAS!

front-of-package	1	5110(0.011432491139819366)	#SAKNAS!	#SAKNAS!
frozen	1	5110(0.011432491139819366)	#SAKNAS!	#SAKNAS!
fructose	1	2214(0.04572996455927746)	2359 (0.034)	1233 (0.1)
fructosyltransferase	1	5110(0.011432491139819366)	2359 (0.034)	#SAKNAS!
fructosyltransferase	1	5110(0.011432491139819366)	2359 (0.034)	#SAKNAS!
frugiperda	1	5110(0.011432491139819366)	#SAKNAS!	2548 (0.033)
frugivore	1	5110(0.011432491139819366)	#SAKNAS!	2548 (0.033)
frugivores	1	5110(0.011432491139819366)	#SAKNAS!	2548 (0.033)
frugivorous	1	2693(0.0342974734194581)	2359 (0.034)	2548 (0.033)
frugivory	1	5110(0.011432491139819366)	2359 (0.034)	#SAKNAS!
frugivory	1	5110(0.011432491139819366)	2359 (0.034)	#SAKNAS!
fruiting	1	5110(0.011432491139819366)	2359 (0.034)	#SAKNAS!
fruiting	1	5110(0.011432491139819366)	2359 (0.034)	#SAKNAS!
fruits	1	1335(0.09145992911855493)	2359 (0.034)	999 (0.13)
frustrations	1	5110(0.011432491139819366)	#SAKNAS!	#SAKNAS!
fry	1	5110(0.011432491139819366)	2359 (0.034)	#SAKNAS!
fry	1	5110(0.011432491139819366)	2359 (0.034)	#SAKNAS!
fsmps	1	5110(0.011432491139819366)	#SAKNAS!	#SAKNAS!
fucata	1	5110(0.011432491139819366)	#SAKNAS!	2548 (0.033)
fuel	1	5110(0.011432491139819366)	2359 (0.034)	#SAKNAS!
fuel	1	5110(0.011432491139819366)	2359 (0.034)	#SAKNAS!
fueling	1	5110(0.011432491139819366)	#SAKNAS!	#SAKNAS!
fuels	1	5110(0.011432491139819366)	#SAKNAS!	2548 (0.033)
fukui	1	5110(0.011432491139819366)	#SAKNAS!	#SAKNAS!
full-day	1	5110(0.011432491139819366)	#SAKNAS!	#SAKNAS!
full-thickness	1	5110(0.011432491139819366)	#SAKNAS!	2548 (0.033)
fully	1	2214(0.04572996455927746)	2359 (0.034)	1668 (0.066)
fumiferana	1	5110(0.011432491139819366)	2359 (0.034)	#SAKNAS!
fumiferana	1	5110(0.011432491139819366)	2359 (0.034)	#SAKNAS!
function-driven	1	5110(0.011432491139819366)	#SAKNAS!	2548 (0.033)
fundamentals	1	5110(0.011432491139819366)	2359 (0.034)	#SAKNAS!
fundamentals	1	5110(0.011432491139819366)	2359 (0.034)	#SAKNAS!
fundic	1	5110(0.011432491139819366)	#SAKNAS!	#SAKNAS!
fundus	1	5110(0.011432491139819366)	2359 (0.034)	#SAKNAS!
fundus	1	5110(0.011432491139819366)	2359 (0.034)	#SAKNAS!
funfte	1	5110(0.011432491139819366)	#SAKNAS!	2548 (0.033)
fungi	1	2693(0.0342974734194581)	2359 (0.034)	2548 (0.033)
funnels	1	5110(0.011432491139819366)	#SAKNAS!	2548 (0.033)
furanone	1	5110(0.011432491139819366)	#SAKNAS!	2548 (0.033)
furnarius	1	5110(0.011432491139819366)	2359 (0.034)	#SAKNAS!
furnarius	1	5110(0.011432491139819366)	2359 (0.034)	#SAKNAS!
further	1	2693(0.0342974734194581)	2359 (0.034)	2548 (0.033)
fussy	1	5110(0.011432491139819366)	#SAKNAS!	2548 (0.033)

g1359a	1 5110(0.011432491139819366)	#SAKNAS!	2548 (0.033)
gaba	1 5110(0.011432491139819366)	#SAKNAS!	#SAKNAS!
gabab-induced	1 5110(0.011432491139819366)	2359 (0.034)	#SAKNAS!
gabab-induced	1 5110(0.011432491139819366)	2359 (0.034)	#SAKNAS!
gad2	1 5110(0.011432491139819366)	2359 (0.034)	#SAKNAS!
gad2	1 5110(0.011432491139819366)	2359 (0.034)	#SAKNAS!
gadus	1 5110(0.011432491139819366)	#SAKNAS!	2548 (0.033)
gadus-morhua	1 5110(0.011432491139819366)	2359 (0.034)	#SAKNAS!
gadus-morhua	1 5110(0.011432491139819366)	2359 (0.034)	#SAKNAS!
gahi	1 5110(0.011432491139819366)	#SAKNAS!	#SAKNAS!
gaining	1 5110(0.011432491139819366)	#SAKNAS!	2548 (0.033)
gains	1 3437(0.02286498227963873)	2359 (0.034)	#SAKNAS!
galactose	1 5110(0.011432491139819366)	#SAKNAS!	#SAKNAS!
galapagos	1 3437(0.02286498227963873)	2359 (0.034)	2548 (0.033)
galicia	1 5110(0.011432491139819366)	2359 (0.034)	#SAKNAS!
galicia	1 5110(0.011432491139819366)	2359 (0.034)	#SAKNAS!
galician	1 5110(0.011432491139819366)	#SAKNAS!	2548 (0.033)
gallinarum	1 5110(0.011432491139819366)	2359 (0.034)	#SAKNAS!
gallinarum	1 5110(0.011432491139819366)	2359 (0.034)	#SAKNAS!
gall-inducing	1 3437(0.02286498227963873)	2359 (0.034)	2548 (0.033)
galloprovincialis	1 5110(0.011432491139819366)	#SAKNAS!	2548 (0.033)
gallstones	1 5110(0.011432491139819366)	#SAKNAS!	#SAKNAS!
game	1 5110(0.011432491139819366)	#SAKNAS!	2548 (0.033)
gamma	1 5110(0.011432491139819366)	#SAKNAS!	2548 (0.033)
gammarus	1 5110(0.011432491139819366)	2359 (0.034)	#SAKNAS!
gammarus	1 5110(0.011432491139819366)	2359 (0.034)	#SAKNAS!
ganglia	1 5110(0.011432491139819366)	#SAKNAS!	#SAKNAS!
gannets	1 5110(0.011432491139819366)	#SAKNAS!	2548 (0.033)
ganzi	1 5110(0.011432491139819366)	#SAKNAS!	2548 (0.033)
gaoligong	1 5110(0.011432491139819366)	#SAKNAS!	2548 (0.033)
gap	1 5110(0.011432491139819366)	#SAKNAS!	#SAKNAS!
gaps	1 3437(0.02286498227963873)	2359 (0.034)	#SAKNAS!
garden	1 5110(0.011432491139819366)	#SAKNAS!	2548 (0.033)
gardening	1 5110(0.011432491139819366)	#SAKNAS!	2548 (0.033)
garment	1 5110(0.011432491139819366)	2359 (0.034)	#SAKNAS!
garment	1 5110(0.011432491139819366)	2359 (0.034)	#SAKNAS!
garthi	1 5110(0.011432491139819366)	#SAKNAS!	2548 (0.033)
gasseri	1 5110(0.011432491139819366)	#SAKNAS!	#SAKNAS!
gasterosteus	1 5110(0.011432491139819366)	2359 (0.034)	#SAKNAS!
gasterosteus	1 5110(0.011432491139819366)	2359 (0.034)	#SAKNAS!
gastric-emptying	1 5110(0.011432491139819366)	2359 (0.034)	#SAKNAS!
gastric-emptying	1 5110(0.011432491139819366)	2359 (0.034)	#SAKNAS!
gastric-tube	1 5110(0.011432491139819366)	2359 (0.034)	#SAKNAS!

gastric-tube	1	5110(0.011432491139819366)	2359 (0.034)	#SAKNAS!
gastritis	1	5110(0.011432491139819366)	#SAKNAS!	2548 (0.033)
gastroduodenal	1	3437(0.02286498227963873)	2359 (0.034)	2548 (0.033)
gastroenteric	1	5110(0.011432491139819366)	#SAKNAS!	2548 (0.033)
gastroenterol	1	5110(0.011432491139819366)	2359 (0.034)	#SAKNAS!
gastroenterol	1	5110(0.011432491139819366)	2359 (0.034)	#SAKNAS!
gastroenterological	1	5110(0.011432491139819366)	2359 (0.034)	#SAKNAS!
gastroenterological	1	5110(0.011432491139819366)	2359 (0.034)	#SAKNAS!
gastroenterologist	1	5110(0.011432491139819366)	2359 (0.034)	#SAKNAS!
gastroenterologist	1	5110(0.011432491139819366)	2359 (0.034)	#SAKNAS!
gastroenterology	1	2214(0.04572996455927746)	2359 (0.034)	2548 (0.033)
gastrojejunal	1	2693(0.0342974734194581)	2359 (0.034)	2548 (0.033)
gastrojejunostomy	1	1678(0.0685949468389162)	2359 (0.034)	1233 (0.1)
gastronomic	1	5110(0.011432491139819366)	#SAKNAS!	#SAKNAS!
gastronomy	1	5110(0.011432491139819366)	#SAKNAS!	#SAKNAS!
gastrooesophageal	1	3437(0.02286498227963873)	2359 (0.034)	#SAKNAS!
gastropexy	1	3437(0.02286498227963873)	2359 (0.034)	#SAKNAS!
gastrophysics	1	5110(0.011432491139819366)	#SAKNAS!	2548 (0.033)
gastropod	1	5110(0.011432491139819366)	2359 (0.034)	#SAKNAS!
gastropod	1	5110(0.011432491139819366)	2359 (0.034)	#SAKNAS!
gastropoda	1	5110(0.011432491139819366)	2359 (0.034)	#SAKNAS!
gastropoda	1	5110(0.011432491139819366)	2359 (0.034)	#SAKNAS!
gastrotomy	1	5110(0.011432491139819366)	#SAKNAS!	#SAKNAS!
gastrula	1	5110(0.011432491139819366)	#SAKNAS!	#SAKNAS!
gatherings	1	5110(0.011432491139819366)	#SAKNAS!	2548 (0.033)
gauge-raised	1	5110(0.011432491139819366)	#SAKNAS!	#SAKNAS!
gcn1	1	5110(0.011432491139819366)	2359 (0.034)	#SAKNAS!
gcn1	1	5110(0.011432491139819366)	2359 (0.034)	#SAKNAS!
gcn2	1	5110(0.011432491139819366)	2359 (0.034)	#SAKNAS!
gcn2	1	5110(0.011432491139819366)	2359 (0.034)	#SAKNAS!
gej	1	5110(0.011432491139819366)	#SAKNAS!	#SAKNAS!
gelatin	1	2693(0.0342974734194581)	2359 (0.034)	#SAKNAS!
gelatine	1	5110(0.011432491139819366)	2359 (0.034)	#SAKNAS!
gelatine	1	5110(0.011432491139819366)	2359 (0.034)	#SAKNAS!
gelatinous	1	3437(0.02286498227963873)	2359 (0.034)	2548 (0.033)
gel-based	1	5110(0.011432491139819366)	#SAKNAS!	#SAKNAS!
gel-biopolymer	1	5110(0.011432491139819366)	#SAKNAS!	#SAKNAS!
gel-forming	1	5110(0.011432491139819366)	2359 (0.034)	#SAKNAS!
gel-forming	1	5110(0.011432491139819366)	2359 (0.034)	#SAKNAS!
gelukspan	1	5110(0.011432491139819366)	2359 (0.034)	#SAKNAS!
gelukspan	1	5110(0.011432491139819366)	2359 (0.034)	#SAKNAS!
gender-dependent	1	5110(0.011432491139819366)	2359 (0.034)	#SAKNAS!
gender-dependent	1	5110(0.011432491139819366)	2359 (0.034)	#SAKNAS!

genders	1	5110(0.011432491139819366)	#SAKNAS!	2548 (0.033)
gender-specific	1	2693(0.0342974734194581)	2359 (0.034)	#SAKNAS!
gene-expression	1	5110(0.011432491139819366)	2359 (0.034)	#SAKNAS!
gene-expression	1	5110(0.011432491139819366)	2359 (0.034)	#SAKNAS!
gene-lifestyle	1	5110(0.011432491139819366)	#SAKNAS!	2548 (0.033)
general-hospital	1	5110(0.011432491139819366)	2359 (0.034)	#SAKNAS!
general-hospital	1	5110(0.011432491139819366)	2359 (0.034)	#SAKNAS!
generalis	1	5110(0.011432491139819366)	#SAKNAS!	2548 (0.033)
generalists	1	5110(0.011432491139819366)	2359 (0.034)	#SAKNAS!
generalists	1	5110(0.011432491139819366)	2359 (0.034)	#SAKNAS!
generalization	1	5110(0.011432491139819366)	#SAKNAS!	2548 (0.033)
general-population	1	5110(0.011432491139819366)	#SAKNAS!	#SAKNAS!
generate	1	5110(0.011432491139819366)	2359 (0.034)	#SAKNAS!
generate	1	5110(0.011432491139819366)	2359 (0.034)	#SAKNAS!
generates	1	3437(0.02286498227963873)	2359 (0.034)	2548 (0.033)
genesis	1	5110(0.011432491139819366)	2359 (0.034)	#SAKNAS!
genesis	1	5110(0.011432491139819366)	2359 (0.034)	#SAKNAS!
genioglossus	1	5110(0.011432491139819366)	2359 (0.034)	#SAKNAS!
genioglossus	1	5110(0.011432491139819366)	2359 (0.034)	#SAKNAS!
geniohyoid	1	5110(0.011432491139819366)	#SAKNAS!	#SAKNAS!
genistein	1	5110(0.011432491139819366)	2359 (0.034)	#SAKNAS!
genistein	1	5110(0.011432491139819366)	2359 (0.034)	#SAKNAS!
genome	1	5110(0.011432491139819366)	#SAKNAS!	2548 (0.033)
genomic	1	5110(0.011432491139819366)	#SAKNAS!	2548 (0.033)
genotype-by-sex-by-diet	1	5110(0.011432491139819366)	#SAKNAS!	2548 (0.033)
genotypic	1	5110(0.011432491139819366)	#SAKNAS!	2548 (0.033)
gentilis	1	5110(0.011432491139819366)	2359 (0.034)	#SAKNAS!
gentilis	1	5110(0.011432491139819366)	2359 (0.034)	#SAKNAS!
geochemistry	1	5110(0.011432491139819366)	2359 (0.034)	#SAKNAS!
geochemistry	1	5110(0.011432491139819366)	2359 (0.034)	#SAKNAS!
geographic	1	2693(0.0342974734194581)	2359 (0.034)	1668 (0.066)
geographical	1	5110(0.011432491139819366)	2359 (0.034)	#SAKNAS!
geographical	1	5110(0.011432491139819366)	2359 (0.034)	#SAKNAS!
geometrical	1	5110(0.011432491139819366)	2359 (0.034)	#SAKNAS!
geometrical	1	5110(0.011432491139819366)	2359 (0.034)	#SAKNAS!
george	1	5110(0.011432491139819366)	2359 (0.034)	#SAKNAS!
george	1	5110(0.011432491139819366)	2359 (0.034)	#SAKNAS!
georgia	1	5110(0.011432491139819366)	2359 (0.034)	#SAKNAS!
georgia	1	5110(0.011432491139819366)	2359 (0.034)	#SAKNAS!
geranium	1	5110(0.011432491139819366)	#SAKNAS!	#SAKNAS!
geriatric-neurologic	1	5110(0.011432491139819366)	2359 (0.034)	#SAKNAS!
geriatric-neurologic	1	5110(0.011432491139819366)	2359 (0.034)	#SAKNAS!
geriatric-patients	1	5110(0.011432491139819366)	2359 (0.034)	#SAKNAS!

geriatric-patients	1	5110(0.011432491139819366)	2359 (0.034)	#SAKNAS!
geriatrics	1	5110(0.011432491139819366)	#SAKNAS!	2548 (0.033)
germanica	1	5110(0.011432491139819366)	2359 (0.034)	#SAKNAS!
germanica	1	5110(0.011432491139819366)	2359 (0.034)	#SAKNAS!
german-speaking	1	5110(0.011432491139819366)	#SAKNAS!	#SAKNAS!
germany-the	1	5110(0.011432491139819366)	#SAKNAS!	#SAKNAS!
germinated-brown-rice-based	1	5110(0.011432491139819366)	#SAKNAS!	#SAKNAS!
gestations	1	5110(0.011432491139819366)	#SAKNAS!	#SAKNAS!
getting	1	5110(0.011432491139819366)	#SAKNAS!	2548 (0.033)
g-formula	1	5110(0.011432491139819366)	#SAKNAS!	2548 (0.033)
gg	1	5110(0.011432491139819366)	#SAKNAS!	2548 (0.033)
ghrelin-induced	1	5110(0.011432491139819366)	2359 (0.034)	#SAKNAS!
ghrelin-induced	1	5110(0.011432491139819366)	2359 (0.034)	#SAKNAS!
gibbon	1	5110(0.011432491139819366)	#SAKNAS!	2548 (0.033)
gibel	1	5110(0.011432491139819366)	#SAKNAS!	2548 (0.033)
gibelio	1	5110(0.011432491139819366)	#SAKNAS!	2548 (0.033)
gibelion	1	5110(0.011432491139819366)	#SAKNAS!	2548 (0.033)
gilts	1	5110(0.011432491139819366)	2359 (0.034)	#SAKNAS!
gilts	1	5110(0.011432491139819366)	2359 (0.034)	#SAKNAS!
gingipains	1	5110(0.011432491139819366)	#SAKNAS!	#SAKNAS!
giniplus	1	5110(0.011432491139819366)	2359 (0.034)	#SAKNAS!
giniplus	1	5110(0.011432491139819366)	2359 (0.034)	#SAKNAS!
ginseng	1	5110(0.011432491139819366)	#SAKNAS!	#SAKNAS!
giraffa	1	5110(0.011432491139819366)	#SAKNAS!	2548 (0.033)
giraffa-camelopardalis	1	5110(0.011432491139819366)	2359 (0.034)	#SAKNAS!
giraffa-camelopardalis	1	5110(0.011432491139819366)	2359 (0.034)	#SAKNAS!
giraffe	1	3437(0.02286498227963873)	2359 (0.034)	2548 (0.033)
girl	1	5110(0.011432491139819366)	#SAKNAS!	2548 (0.033)
girolando	1	5110(0.011432491139819366)	#SAKNAS!	#SAKNAS!
give	1	5110(0.011432491139819366)	#SAKNAS!	2548 (0.033)
givers	1	5110(0.011432491139819366)	2359 (0.034)	#SAKNAS!
givers	1	5110(0.011432491139819366)	2359 (0.034)	#SAKNAS!
giving	1	5110(0.011432491139819366)	#SAKNAS!	2548 (0.033)
gla	1	5110(0.011432491139819366)	2359 (0.034)	#SAKNAS!
gla	1	5110(0.011432491139819366)	2359 (0.034)	#SAKNAS!
glacier	1	5110(0.011432491139819366)	#SAKNAS!	#SAKNAS!
gladii	1	5110(0.011432491139819366)	#SAKNAS!	#SAKNAS!
gladius	1	5110(0.011432491139819366)	#SAKNAS!	#SAKNAS!
glands	1	1894(0.05716245569909683)	2359 (0.034)	2548 (0.033)
glial	1	5110(0.011432491139819366)	#SAKNAS!	#SAKNAS!
glide	1	5110(0.011432491139819366)	#SAKNAS!	2548 (0.033)
glider	1	5110(0.011432491139819366)	#SAKNAS!	2548 (0.033)
gliders	1	5110(0.011432491139819366)	#SAKNAS!	2548 (0.033)

gliptins	1	5110(0.011432491139819366)	#SAKNAS!	2548 (0.033)
globus	1	5110(0.011432491139819366)	#SAKNAS!	2548 (0.033)
glomerida	1	5110(0.011432491139819366)	#SAKNAS!	#SAKNAS!
glomeridae	1	5110(0.011432491139819366)	#SAKNAS!	#SAKNAS!
glomeris	1	5110(0.011432491139819366)	#SAKNAS!	#SAKNAS!
glottic	1	5110(0.011432491139819366)	2359 (0.034)	#SAKNAS!
glottic	1	5110(0.011432491139819366)	2359 (0.034)	#SAKNAS!
glp1	1	5110(0.011432491139819366)	#SAKNAS!	#SAKNAS!
glp-1	1	5110(0.011432491139819366)	#SAKNAS!	#SAKNAS!
glucocorticoid	1	2693(0.0342974734194581)	2359 (0.034)	1668 (0.066)
glucocorticoids	1	5110(0.011432491139819366)	#SAKNAS!	#SAKNAS!
glucomannan-mediated	1	5110(0.011432491139819366)	#SAKNAS!	#SAKNAS!
glucomannans	1	5110(0.011432491139819366)	#SAKNAS!	2548 (0.033)
glucosides	1	5110(0.011432491139819366)	2359 (0.034)	#SAKNAS!
glucosides	1	5110(0.011432491139819366)	2359 (0.034)	#SAKNAS!
glucosinolate	1	5110(0.011432491139819366)	2359 (0.034)	#SAKNAS!
glucosinolate	1	5110(0.011432491139819366)	2359 (0.034)	#SAKNAS!
glue	1	5110(0.011432491139819366)	2359 (0.034)	#SAKNAS!
glue	1	5110(0.011432491139819366)	2359 (0.034)	#SAKNAS!
glut2	1	5110(0.011432491139819366)	#SAKNAS!	#SAKNAS!
glutamate	1	2214(0.04572996455927746)	2359 (0.034)	2548 (0.033)
glutamatergic	1	3437(0.02286498227963873)	2359 (0.034)	2548 (0.033)
glutamine	1	5110(0.011432491139819366)	#SAKNAS!	2548 (0.033)
glutaric	1	5110(0.011432491139819366)	2359 (0.034)	#SAKNAS!
glutaric	1	5110(0.011432491139819366)	2359 (0.034)	#SAKNAS!
gluten	1	5110(0.011432491139819366)	#SAKNAS!	2548 (0.033)
gluten-free	1	5110(0.011432491139819366)	#SAKNAS!	#SAKNAS!
glycaemic	1	1894(0.05716245569909683)	2359 (0.034)	1668 (0.066)
glycation	1	2214(0.04572996455927746)	2359 (0.034)	1668 (0.066)
glycemia	1	5110(0.011432491139819366)	#SAKNAS!	#SAKNAS!
glycemia-sensitive	1	5110(0.011432491139819366)	2359 (0.034)	#SAKNAS!
glycemia-sensitive	1	5110(0.011432491139819366)	2359 (0.034)	#SAKNAS!
glycogen	1	5110(0.011432491139819366)	#SAKNAS!	2548 (0.033)
glycohemoglobin	1	5110(0.011432491139819366)	2359 (0.034)	#SAKNAS!
glycohemoglobin	1	5110(0.011432491139819366)	2359 (0.034)	#SAKNAS!
glycolytic	1	5110(0.011432491139819366)	#SAKNAS!	2548 (0.033)
gmelin	1	5110(0.011432491139819366)	2359 (0.034)	#SAKNAS!
gmelin	1	5110(0.011432491139819366)	2359 (0.034)	#SAKNAS!
gnotobiotic	1	5110(0.011432491139819366)	#SAKNAS!	#SAKNAS!
gnri	1	5110(0.011432491139819366)	#SAKNAS!	#SAKNAS!
gntr	1	5110(0.011432491139819366)	#SAKNAS!	#SAKNAS!
goal-directed	1	5110(0.011432491139819366)	#SAKNAS!	2548 (0.033)
goats*	1	743(0.18291985823710985)	451 (0.28)	1233 (0.1)

goblet	1	5110(0.011432491139819366)	#SAKNAS!	#SAKNAS!
gohai	1	5110(0.011432491139819366)	#SAKNAS!	#SAKNAS!
goiter	1	5110(0.011432491139819366)	#SAKNAS!	#SAKNAS!
goiters	1	5110(0.011432491139819366)	2359 (0.034)	#SAKNAS!
goiters	1	5110(0.011432491139819366)	2359 (0.034)	#SAKNAS!
gojjam	1	5110(0.011432491139819366)	#SAKNAS!	2548 (0.033)
gokceada	1	5110(0.011432491139819366)	#SAKNAS!	#SAKNAS!
golden	1	5110(0.011432491139819366)	#SAKNAS!	2548 (0.033)
golden-headed	1	5110(0.011432491139819366)	#SAKNAS!	#SAKNAS!
gompertz-makeham	1	5110(0.011432491139819366)	2359 (0.034)	#SAKNAS!
gompertz-makeham	1	5110(0.011432491139819366)	2359 (0.034)	#SAKNAS!
gorge	1	5110(0.011432491139819366)	2359 (0.034)	#SAKNAS!
gorge	1	5110(0.011432491139819366)	2359 (0.034)	#SAKNAS!
goteborg	1	5110(0.011432491139819366)	2359 (0.034)	#SAKNAS!
goteborg	1	5110(0.011432491139819366)	2359 (0.034)	#SAKNAS!
gothenburg	1	3437(0.02286498227963873)	2359 (0.034)	2548 (0.033)
government	1	3437(0.02286498227963873)	2359 (0.034)	#SAKNAS!
governmental	1	5110(0.011432491139819366)	#SAKNAS!	2548 (0.033)
gower	1	5110(0.011432491139819366)	#SAKNAS!	#SAKNAS!
gpr120	1	5110(0.011432491139819366)	#SAKNAS!	2548 (0.033)
gps	1	5110(0.011432491139819366)	2359 (0.034)	#SAKNAS!
gps	1	5110(0.011432491139819366)	2359 (0.034)	#SAKNAS!
gradients	1	5110(0.011432491139819366)	#SAKNAS!	2548 (0.033)
grafts	1	5110(0.011432491139819366)	2359 (0.034)	#SAKNAS!
grafts	1	5110(0.011432491139819366)	2359 (0.034)	#SAKNAS!
grain	1	1678(0.0685949468389162)	2359 (0.034)	1233 (0.1)
grain-pellet	1	5110(0.011432491139819366)	2359 (0.034)	#SAKNAS!
grain-pellet	1	5110(0.011432491139819366)	2359 (0.034)	#SAKNAS!
gramine	1	5110(0.011432491139819366)	2359 (0.034)	#SAKNAS!
gramine	1	5110(0.011432491139819366)	2359 (0.034)	#SAKNAS!
graminivorous	1	5110(0.011432491139819366)	2359 (0.034)	#SAKNAS!
graminivorous	1	5110(0.011432491139819366)	2359 (0.034)	#SAKNAS!
gran	1	5110(0.011432491139819366)	2359 (0.034)	#SAKNAS!
gran	1	5110(0.011432491139819366)	2359 (0.034)	#SAKNAS!
granarius	1	5110(0.011432491139819366)	#SAKNAS!	#SAKNAS!
grande	1	5110(0.011432491139819366)	2359 (0.034)	#SAKNAS!
grande	1	5110(0.011432491139819366)	2359 (0.034)	#SAKNAS!
grandmothers	1	5110(0.011432491139819366)	#SAKNAS!	2548 (0.033)
grandparents	1	5110(0.011432491139819366)	#SAKNAS!	2548 (0.033)
granivorous	1	5110(0.011432491139819366)	#SAKNAS!	2548 (0.033)
granular	1	5110(0.011432491139819366)	2359 (0.034)	#SAKNAS!
granular	1	5110(0.011432491139819366)	2359 (0.034)	#SAKNAS!
granulicatella	1	5110(0.011432491139819366)	#SAKNAS!	2548 (0.033)

granuloma	1	5110(0.011432491139819366)	#SAKNAS!	2548 (0.033)
granulomatosis	1	5110(0.011432491139819366)	2359 (0.034)	#SAKNAS!
granulomatosis	1	5110(0.011432491139819366)	2359 (0.034)	#SAKNAS!
grape	1	5110(0.011432491139819366)	2359 (0.034)	#SAKNAS!
grape	1	5110(0.011432491139819366)	2359 (0.034)	#SAKNAS!
grapes	1	5110(0.011432491139819366)	#SAKNAS!	#SAKNAS!
grapevine	1	5110(0.011432491139819366)	2359 (0.034)	#SAKNAS!
grapevine	1	5110(0.011432491139819366)	2359 (0.034)	#SAKNAS!
graphical	1	5110(0.011432491139819366)	#SAKNAS!	#SAKNAS!
grassland	1	5110(0.011432491139819366)	2359 (0.034)	#SAKNAS!
grassland	1	5110(0.011432491139819366)	2359 (0.034)	#SAKNAS!
grasslands	1	5110(0.011432491139819366)	2359 (0.034)	#SAKNAS!
grasslands	1	5110(0.011432491139819366)	2359 (0.034)	#SAKNAS!
grass-silage	1	5110(0.011432491139819366)	2359 (0.034)	#SAKNAS!
grass-silage	1	5110(0.011432491139819366)	2359 (0.034)	#SAKNAS!
gravo	1	5110(0.011432491139819366)	#SAKNAS!	#SAKNAS!
gray	1	2693(0.0342974734194581)	2359 (0.034)	2548 (0.033)
grayling	1	5110(0.011432491139819366)	#SAKNAS!	2548 (0.033)
graz	1	5110(0.011432491139819366)	2359 (0.034)	#SAKNAS!
graz	1	5110(0.011432491139819366)	2359 (0.034)	#SAKNAS!
grazed	1	5110(0.011432491139819366)	#SAKNAS!	2548 (0.033)
grazoprevir	1	5110(0.011432491139819366)	#SAKNAS!	2548 (0.033)
greece	1	5110(0.011432491139819366)	#SAKNAS!	#SAKNAS!
greenhouse	1	5110(0.011432491139819366)	#SAKNAS!	2548 (0.033)
greenlip	1	5110(0.011432491139819366)	#SAKNAS!	2548 (0.033)
green-lipped	1	5110(0.011432491139819366)	2359 (0.034)	#SAKNAS!
green-lipped	1	5110(0.011432491139819366)	2359 (0.034)	#SAKNAS!
green-morph	1	5110(0.011432491139819366)	#SAKNAS!	#SAKNAS!
gregaria	1	3437(0.02286498227963873)	2359 (0.034)	2548 (0.033)
gregarious	1	5110(0.011432491139819366)	2359 (0.034)	#SAKNAS!
gregarious	1	5110(0.011432491139819366)	2359 (0.034)	#SAKNAS!
grey	1	5110(0.011432491139819366)	2359 (0.034)	#SAKNAS!
grey	1	5110(0.011432491139819366)	2359 (0.034)	#SAKNAS!
grizzled	1	5110(0.011432491139819366)	#SAKNAS!	#SAKNAS!
grooming	1	5110(0.011432491139819366)	2359 (0.034)	#SAKNAS!
grooming	1	5110(0.011432491139819366)	2359 (0.034)	#SAKNAS!
groundwaters	1	5110(0.011432491139819366)	2359 (0.034)	#SAKNAS!
groundwaters	1	5110(0.011432491139819366)	2359 (0.034)	#SAKNAS!
grouse	1	5110(0.011432491139819366)	2359 (0.034)	#SAKNAS!
grouse	1	5110(0.011432491139819366)	2359 (0.034)	#SAKNAS!
grow	1	5110(0.011432491139819366)	#SAKNAS!	#SAKNAS!
grow2gether	1	5110(0.011432491139819366)	#SAKNAS!	2548 (0.033)
grower-finisher	1	5110(0.011432491139819366)	#SAKNAS!	#SAKNAS!

growers	1	5110(0.011432491139819366)	2359 (0.034)	#SAKNAS!
growers	1	5110(0.011432491139819366)	2359 (0.034)	#SAKNAS!
growing-finishing	1	5110(0.011432491139819366)	#SAKNAS!	2548 (0.033)
grown	1	3437(0.02286498227963873)	2359 (0.034)	#SAKNAS!
growth-development	1	5110(0.011432491139819366)	#SAKNAS!	#SAKNAS!
growth-restricted	1	5110(0.011432491139819366)	#SAKNAS!	2548 (0.033)
guam	1	5110(0.011432491139819366)	#SAKNAS!	#SAKNAS!
guanacos	1	5110(0.011432491139819366)	2359 (0.034)	#SAKNAS!
guanacos	1	5110(0.011432491139819366)	2359 (0.034)	#SAKNAS!
guangdong	1	5110(0.011432491139819366)	#SAKNAS!	2548 (0.033)
guanicoe	1	5110(0.011432491139819366)	2359 (0.034)	#SAKNAS!
guanicoe	1	5110(0.011432491139819366)	2359 (0.034)	#SAKNAS!
guanidine	1	5110(0.011432491139819366)	#SAKNAS!	#SAKNAS!
guaraquecaba	1	5110(0.011432491139819366)	2359 (0.034)	#SAKNAS!
guaraquecaba	1	5110(0.011432491139819366)	2359 (0.034)	#SAKNAS!
guardians	1	5110(0.011432491139819366)	#SAKNAS!	#SAKNAS!
guarding	1	5110(0.011432491139819366)	#SAKNAS!	#SAKNAS!
gugging	1	3437(0.02286498227963873)	2359 (0.034)	#SAKNAS!
guidance	1	2693(0.0342974734194581)	2359 (0.034)	2548 (0.033)
guideline-based	1	5110(0.011432491139819366)	#SAKNAS!	#SAKNAS!
guides	1	5110(0.011432491139819366)	#SAKNAS!	2548 (0.033)
guilan	1	5110(0.011432491139819366)	#SAKNAS!	#SAKNAS!
guillemot	1	5110(0.011432491139819366)	2359 (0.034)	#SAKNAS!
guillemot	1	5110(0.011432491139819366)	2359 (0.034)	#SAKNAS!
guinea	1	2693(0.0342974734194581)	2359 (0.034)	2548 (0.033)
guinea-pig	1	5110(0.011432491139819366)	2359 (0.034)	#SAKNAS!
guinea-pig	1	5110(0.011432491139819366)	2359 (0.034)	#SAKNAS!
gulf	1	2693(0.0342974734194581)	2359 (0.034)	2548 (0.033)
gum	1	1111(0.11432491139819366)	2359 (0.034)	816 (0.16)
gum-biopolymers	1	5110(0.011432491139819366)	#SAKNAS!	#SAKNAS!
gum-modified	1	5110(0.011432491139819366)	#SAKNAS!	#SAKNAS!
gunshot	1	5110(0.011432491139819366)	#SAKNAS!	#SAKNAS!
gustation	1	5110(0.011432491139819366)	#SAKNAS!	2548 (0.033)
gut-derived	1	5110(0.011432491139819366)	#SAKNAS!	2548 (0.033)
guts	1	5110(0.011432491139819366)	#SAKNAS!	#SAKNAS!
gvhd	1	5110(0.011432491139819366)	#SAKNAS!	2548 (0.033)
gymnodiniales	1	5110(0.011432491139819366)	#SAKNAS!	2548 (0.033)
gynecologic	1	5110(0.011432491139819366)	#SAKNAS!	2548 (0.033)
gynes	1	5110(0.011432491139819366)	#SAKNAS!	#SAKNAS!
gypsy-moth	1	5110(0.011432491139819366)	2359 (0.034)	#SAKNAS!
gypsy-moth	1	5110(0.011432491139819366)	2359 (0.034)	#SAKNAS!
gyrus	1	5110(0.011432491139819366)	2359 (0.034)	#SAKNAS!
gyrus	1	5110(0.011432491139819366)	2359 (0.034)	#SAKNAS!

h2s	1	5110(0.011432491139819366)	#SAKNAS!	#SAKNAS!
h70	1	5110(0.011432491139819366)	2359 (0.034)	#SAKNAS!
h70	1	5110(0.011432491139819366)	2359 (0.034)	#SAKNAS!
ha	1	5110(0.011432491139819366)	2359 (0.034)	#SAKNAS!
ha	1	5110(0.011432491139819366)	2359 (0.034)	#SAKNAS!
h-abc	1	5110(0.011432491139819366)	#SAKNAS!	#SAKNAS!
habitats	1	2693(0.0342974734194581)	2359 (0.034)	#SAKNAS!
habitual	1	3437(0.02286498227963873)	2359 (0.034)	#SAKNAS!
haematological	1	3437(0.02286498227963873)	2359 (0.034)	2548 (0.033)
haematopus	1	5110(0.011432491139819366)	2359 (0.034)	#SAKNAS!
haematopus	1	5110(0.011432491139819366)	2359 (0.034)	#SAKNAS!
haemolymph	1	5110(0.011432491139819366)	2359 (0.034)	#SAKNAS!
haemolymph	1	5110(0.011432491139819366)	2359 (0.034)	#SAKNAS!
haemonchus	1	5110(0.011432491139819366)	#SAKNAS!	2548 (0.033)
haemophilia	1	5110(0.011432491139819366)	#SAKNAS!	2548 (0.033)
haemorrhage	1	5110(0.011432491139819366)	#SAKNAS!	2548 (0.033)
hague	1	5110(0.011432491139819366)	2359 (0.034)	#SAKNAS!
hague	1	5110(0.011432491139819366)	2359 (0.034)	#SAKNAS!
hake	1	5110(0.011432491139819366)	#SAKNAS!	2548 (0.033)
half-day	1	5110(0.011432491139819366)	#SAKNAS!	#SAKNAS!
half-solidification	1	5110(0.011432491139819366)	2359 (0.034)	#SAKNAS!
half-solidification	1	5110(0.011432491139819366)	2359 (0.034)	#SAKNAS!
haliday	1	5110(0.011432491139819366)	2359 (0.034)	#SAKNAS!
haliday	1	5110(0.011432491139819366)	2359 (0.034)	#SAKNAS!
haliotis	1	3437(0.02286498227963873)	2359 (0.034)	2548 (0.033)
halo	1	5110(0.011432491139819366)	#SAKNAS!	#SAKNAS!
halo-vest	1	5110(0.011432491139819366)	#SAKNAS!	2548 (0.033)
hamilton	1	5110(0.011432491139819366)	2359 (0.034)	#SAKNAS!
hamilton	1	5110(0.011432491139819366)	2359 (0.034)	#SAKNAS!
hamsters	1	5110(0.011432491139819366)	#SAKNAS!	2548 (0.033)
handfeeding	1	5110(0.011432491139819366)	#SAKNAS!	2548 (0.033)
handle	1	5110(0.011432491139819366)	2359 (0.034)	#SAKNAS!
handle	1	5110(0.011432491139819366)	2359 (0.034)	#SAKNAS!
handling	1	5110(0.011432491139819366)	#SAKNAS!	#SAKNAS!
hands	1	5110(0.011432491139819366)	#SAKNAS!	#SAKNAS!
hannai	1	5110(0.011432491139819366)	2359 (0.034)	#SAKNAS!
hannai	1	5110(0.011432491139819366)	2359 (0.034)	#SAKNAS!
hap1	1	5110(0.011432491139819366)	2359 (0.034)	#SAKNAS!
hap1	1	5110(0.011432491139819366)	2359 (0.034)	#SAKNAS!
haplochromis	1	5110(0.011432491139819366)	2359 (0.034)	#SAKNAS!
haplochromis	1	5110(0.011432491139819366)	2359 (0.034)	#SAKNAS!
happiness	1	5110(0.011432491139819366)	#SAKNAS!	#SAKNAS!
haptophyceae	1	5110(0.011432491139819366)	2359 (0.034)	#SAKNAS!

haptophyceae	1	5110(0.011432491139819366)	2359 (0.034)	#SAKNAS!
harassment	1	5110(0.011432491139819366)	#SAKNAS!	2548 (0.033)
harboring	1	5110(0.011432491139819366)	#SAKNAS!	2548 (0.033)
hard	1	3437(0.02286498227963873)	2359 (0.034)	2548 (0.033)
hardship	1	5110(0.011432491139819366)	#SAKNAS!	#SAKNAS!
hargeisa	1	5110(0.011432491139819366)	#SAKNAS!	2548 (0.033)
harlequin	1	5110(0.011432491139819366)	2359 (0.034)	#SAKNAS!
harlequin	1	5110(0.011432491139819366)	2359 (0.034)	#SAKNAS!
harm	1	5110(0.011432491139819366)	#SAKNAS!	#SAKNAS!
harsh	1	5110(0.011432491139819366)	2359 (0.034)	#SAKNAS!
harsh	1	5110(0.011432491139819366)	2359 (0.034)	#SAKNAS!
harvest	1	5110(0.011432491139819366)	2359 (0.034)	#SAKNAS!
harvest	1	5110(0.011432491139819366)	2359 (0.034)	#SAKNAS!
harvester	1	5110(0.011432491139819366)	#SAKNAS!	#SAKNAS!
hasankeyf	1	5110(0.011432491139819366)	#SAKNAS!	#SAKNAS!
hashimoto	1	5110(0.011432491139819366)	#SAKNAS!	2548 (0.033)
hashish	1	5110(0.011432491139819366)	#SAKNAS!	2548 (0.033)
hausa	1	5110(0.011432491139819366)	#SAKNAS!	#SAKNAS!
having	1	1485(0.08002743797873557)	2359 (0.034)	1233 (0.1)
hawkmoth	1	5110(0.011432491139819366)	2359 (0.034)	#SAKNAS!
hawkmoth	1	5110(0.011432491139819366)	2359 (0.034)	#SAKNAS!
hawksbill	1	5110(0.011432491139819366)	2359 (0.034)	#SAKNAS!
hawksbill	1	5110(0.011432491139819366)	2359 (0.034)	#SAKNAS!
hays	1	5110(0.011432491139819366)	#SAKNAS!	2548 (0.033)
hay-to-grain	1	5110(0.011432491139819366)	#SAKNAS!	2548 (0.033)
hchs	1	5110(0.011432491139819366)	#SAKNAS!	2548 (0.033)
hdl	1	5110(0.011432491139819366)	#SAKNAS!	2548 (0.033)
hdl-c	1	5110(0.011432491139819366)	#SAKNAS!	#SAKNAS!
hdl-cholesterol	1	5110(0.011432491139819366)	#SAKNAS!	#SAKNAS!
he	1	5110(0.011432491139819366)	#SAKNAS!	2548 (0.033)
he4	1	5110(0.011432491139819366)	#SAKNAS!	#SAKNAS!
headed	1	5110(0.011432491139819366)	#SAKNAS!	2548 (0.033)
healing	1	2214(0.04572996455927746)	2359 (0.034)	1668 (0.066)
healthcare	1	743(0.18291985823710985)	2359 (0.034)	1233 (0.1)
health-enhancing	1	5110(0.011432491139819366)	2359 (0.034)	#SAKNAS!
health-enhancing	1	5110(0.011432491139819366)	2359 (0.034)	#SAKNAS!
healthful	1	2693(0.0342974734194581)	2359 (0.034)	1668 (0.066)
healthier	1	2214(0.04572996455927746)	2359 (0.034)	#SAKNAS!
health-problems	1	5110(0.011432491139819366)	2359 (0.034)	#SAKNAS!
health-problems	1	5110(0.011432491139819366)	2359 (0.034)	#SAKNAS!
healthy-eater	1	5110(0.011432491139819366)	#SAKNAS!	2548 (0.033)
heartburn	1	3437(0.02286498227963873)	2359 (0.034)	#SAKNAS!
heat-shock	1	5110(0.011432491139819366)	2359 (0.034)	#SAKNAS!

heat-shock	1	5110(0.011432491139819366)	2359 (0.034)	#SAKNAS!
heavy	1	1894(0.05716245569909683)	2359 (0.034)	2548 (0.033)
hedgehog	1	5110(0.011432491139819366)	#SAKNAS!	2548 (0.033)
hedonic	1	2693(0.0342974734194581)	2359 (0.034)	2548 (0.033)
heifer	1	5110(0.011432491139819366)	#SAKNAS!	#SAKNAS!
heifers	1	1894(0.05716245569909683)	2359 (0.034)	1668 (0.066)
helgolandicus	1	5110(0.011432491139819366)	2359 (0.034)	#SAKNAS!
helgolandicus	1	5110(0.011432491139819366)	2359 (0.034)	#SAKNAS!
helicobacter?ylori	1	5110(0.011432491139819366)	#SAKNAS!	#SAKNAS!
heliconius	1	5110(0.011432491139819366)	2359 (0.034)	#SAKNAS!
heliconius	1	5110(0.011432491139819366)	2359 (0.034)	#SAKNAS!
heliothis-zea	1	5110(0.011432491139819366)	2359 (0.034)	#SAKNAS!
heliothis-zea	1	5110(0.011432491139819366)	2359 (0.034)	#SAKNAS!
helium-neon	1	5110(0.011432491139819366)	2359 (0.034)	#SAKNAS!
helium-neon	1	5110(0.011432491139819366)	2359 (0.034)	#SAKNAS!
heller-dor	1	5110(0.011432491139819366)	2359 (0.034)	#SAKNAS!
heller-dor	1	5110(0.011432491139819366)	2359 (0.034)	#SAKNAS!
help	1	5110(0.011432491139819366)	#SAKNAS!	2548 (0.033)
helping	1	5110(0.011432491139819366)	2359 (0.034)	#SAKNAS!
helping	1	5110(0.011432491139819366)	2359 (0.034)	#SAKNAS!
helps	1	5110(0.011432491139819366)	#SAKNAS!	#SAKNAS!
hemagglutinins	1	5110(0.011432491139819366)	#SAKNAS!	2548 (0.033)
hematological	1	5110(0.011432491139819366)	2359 (0.034)	#SAKNAS!
hematological	1	5110(0.011432491139819366)	2359 (0.034)	#SAKNAS!
hematoma	1	3437(0.02286498227963873)	2359 (0.034)	#SAKNAS!
heme-peroxidase	1	5110(0.011432491139819366)	#SAKNAS!	2548 (0.033)
hemiglossectomy	1	5110(0.011432491139819366)	#SAKNAS!	2548 (0.033)
hemionus	1	5110(0.011432491139819366)	2359 (0.034)	#SAKNAS!
hemionus	1	5110(0.011432491139819366)	2359 (0.034)	#SAKNAS!
hemiparetic	1	5110(0.011432491139819366)	#SAKNAS!	2548 (0.033)
hemochromatosis	1	5110(0.011432491139819366)	#SAKNAS!	#SAKNAS!
hemocyte	1	5110(0.011432491139819366)	#SAKNAS!	2548 (0.033)
hemogram	1	5110(0.011432491139819366)	#SAKNAS!	#SAKNAS!
hemolymph	1	3437(0.02286498227963873)	2359 (0.034)	2548 (0.033)
hemorrhagic	1	5110(0.011432491139819366)	2359 (0.034)	#SAKNAS!
hemorrhagic	1	5110(0.011432491139819366)	2359 (0.034)	#SAKNAS!
hemp	1	5110(0.011432491139819366)	#SAKNAS!	2548 (0.033)
hens	1	1678(0.0685949468389162)	2359 (0.034)	1233 (0.1)
hepatocellular	1	5110(0.011432491139819366)	2359 (0.034)	#SAKNAS!
hepatocellular	1	5110(0.011432491139819366)	2359 (0.034)	#SAKNAS!
hepatocyte	1	5110(0.011432491139819366)	2359 (0.034)	#SAKNAS!
hepatocyte	1	5110(0.011432491139819366)	2359 (0.034)	#SAKNAS!
hepatol	1	5110(0.011432491139819366)	2359 (0.034)	#SAKNAS!

hepatol	1	5110(0.011432491139819366)	2359 (0.034)	#SAKNAS!
hepatology	1	2214(0.04572996455927746)	2359 (0.034)	2548 (0.033)
herat	1	5110(0.011432491139819366)	#SAKNAS!	2548 (0.033)
herb	1	5110(0.011432491139819366)	#SAKNAS!	2548 (0.033)
herbaceous	1	5110(0.011432491139819366)	2359 (0.034)	#SAKNAS!
herbaceous	1	5110(0.011432491139819366)	2359 (0.034)	#SAKNAS!
herbage	1	5110(0.011432491139819366)	2359 (0.034)	#SAKNAS!
herbage	1	5110(0.011432491139819366)	2359 (0.034)	#SAKNAS!
herbs	1	5110(0.011432491139819366)	#SAKNAS!	2548 (0.033)
herders	1	5110(0.011432491139819366)	#SAKNAS!	2548 (0.033)
hereditary	1	2214(0.04572996455927746)	2359 (0.034)	2548 (0.033)
herniae	1	5110(0.011432491139819366)	#SAKNAS!	2548 (0.033)
hernia-related	1	5110(0.011432491139819366)	#SAKNAS!	2548 (0.033)
herpes	1	3437(0.02286498227963873)	2359 (0.034)	#SAKNAS!
herpesviridae	1	5110(0.011432491139819366)	2359 (0.034)	#SAKNAS!
herpesviridae	1	5110(0.011432491139819366)	2359 (0.034)	#SAKNAS!
herring	1	5110(0.011432491139819366)	#SAKNAS!	2548 (0.033)
herring-feeding	1	5110(0.011432491139819366)	#SAKNAS!	#SAKNAS!
herzegovina	1	5110(0.011432491139819366)	2359 (0.034)	#SAKNAS!
herzegovina	1	5110(0.011432491139819366)	2359 (0.034)	#SAKNAS!
heterogeneous	1	5110(0.011432491139819366)	#SAKNAS!	#SAKNAS!
heterozygous	1	5110(0.011432491139819366)	2359 (0.034)	#SAKNAS!
heterozygous	1	5110(0.011432491139819366)	2359 (0.034)	#SAKNAS!
hexacoral	1	5110(0.011432491139819366)	#SAKNAS!	#SAKNAS!
hexasticha	1	5110(0.011432491139819366)	#SAKNAS!	#SAKNAS!
hf	1	5110(0.011432491139819366)	#SAKNAS!	#SAKNAS!
hg	1	5110(0.011432491139819366)	#SAKNAS!	2548 (0.033)
hiatoplasty	1	5110(0.011432491139819366)	2359 (0.034)	#SAKNAS!
hiatoplasty	1	5110(0.011432491139819366)	2359 (0.034)	#SAKNAS!
hiatus	1	5110(0.011432491139819366)	2359 (0.034)	#SAKNAS!
hiatus	1	5110(0.011432491139819366)	2359 (0.034)	#SAKNAS!
hidden	1	5110(0.011432491139819366)	#SAKNAS!	2548 (0.033)
hidradenitis	1	5110(0.011432491139819366)	#SAKNAS!	#SAKNAS!
hierarchical	1	5110(0.011432491139819366)	#SAKNAS!	#SAKNAS!
hierarchy	1	5110(0.011432491139819366)	2359 (0.034)	#SAKNAS!
hierarchy	1	5110(0.011432491139819366)	2359 (0.034)	#SAKNAS!
high-caloric	1	5110(0.011432491139819366)	2359 (0.034)	#SAKNAS!
high-caloric	1	5110(0.011432491139819366)	2359 (0.034)	#SAKNAS!
high-carotid	1	5110(0.011432491139819366)	#SAKNAS!	#SAKNAS!
high-concentrate	1	5110(0.011432491139819366)	#SAKNAS!	#SAKNAS!
high-density	1	1335(0.09145992911855493)	2359 (0.034)	#SAKNAS!
high-dose	1	5110(0.011432491139819366)	2359 (0.034)	#SAKNAS!
high-dose	1	5110(0.011432491139819366)	2359 (0.034)	#SAKNAS!

higher-level	1	5110(0.011432491139819366)	#SAKNAS!	#SAKNAS!
high-fat-diet-induced	1	5110(0.011432491139819366)	#SAKNAS!	2548 (0.033)
high-frequency	1	5110(0.011432491139819366)	#SAKNAS!	#SAKNAS!
high-grade	1	5110(0.011432491139819366)	#SAKNAS!	2548 (0.033)
high-incidence	1	5110(0.011432491139819366)	#SAKNAS!	#SAKNAS!
high-income	1	5110(0.011432491139819366)	#SAKNAS!	#SAKNAS!
high-intensity	1	5110(0.011432491139819366)	2359 (0.034)	#SAKNAS!
high-intensity	1	5110(0.011432491139819366)	2359 (0.034)	#SAKNAS!
highlight	1	5110(0.011432491139819366)	#SAKNAS!	2548 (0.033)
highlights	1	3437(0.02286498227963873)	2359 (0.034)	#SAKNAS!
high-phase	1	5110(0.011432491139819366)	#SAKNAS!	#SAKNAS!
high-protein	1	5110(0.011432491139819366)	#SAKNAS!	#SAKNAS!
high-quality	1	5110(0.011432491139819366)	2359 (0.034)	#SAKNAS!
high-quality	1	5110(0.011432491139819366)	2359 (0.034)	#SAKNAS!
high-sensitivity	1	1894(0.05716245569909683)	2359 (0.034)	1668 (0.066)
hindbrain	1	5110(0.011432491139819366)	#SAKNAS!	#SAKNAS!
hinders	1	5110(0.011432491139819366)	#SAKNAS!	2548 (0.033)
hindi	1	5110(0.011432491139819366)	#SAKNAS!	2548 (0.033)
hint-8	1	5110(0.011432491139819366)	#SAKNAS!	#SAKNAS!
hip	1	1485(0.08002743797873557)	2359 (0.034)	1668 (0.066)
hippocamelus	1	5110(0.011432491139819366)	2359 (0.034)	#SAKNAS!
hippocamelus	1	5110(0.011432491139819366)	2359 (0.034)	#SAKNAS!
hippocampal	1	1678(0.0685949468389162)	2359 (0.034)	1233 (0.1)
hippocampus	1	5110(0.011432491139819366)	#SAKNAS!	2548 (0.033)
hippocampus-dependent	1	5110(0.011432491139819366)	#SAKNAS!	2548 (0.033)
hippodamia	1	5110(0.011432491139819366)	#SAKNAS!	2548 (0.033)
hippophae	1	5110(0.011432491139819366)	#SAKNAS!	#SAKNAS!
hippurus	1	5110(0.011432491139819366)	#SAKNAS!	2548 (0.033)
hisayama	1	3437(0.02286498227963873)	2359 (0.034)	2548 (0.033)
hispanics	1	3437(0.02286498227963873)	2359 (0.034)	2548 (0.033)
histamine	1	5110(0.011432491139819366)	2359 (0.034)	#SAKNAS!
histamine	1	5110(0.011432491139819366)	2359 (0.034)	#SAKNAS!
histogram	1	5110(0.011432491139819366)	#SAKNAS!	#SAKNAS!
histology	1	3437(0.02286498227963873)	2359 (0.034)	2548 (0.033)
histomorphometry	1	5110(0.011432491139819366)	2359 (0.034)	#SAKNAS!
histomorphometry	1	5110(0.011432491139819366)	2359 (0.034)	#SAKNAS!
histopathological	1	5110(0.011432491139819366)	#SAKNAS!	#SAKNAS!
historical	1	3437(0.02286498227963873)	2359 (0.034)	2548 (0.033)
hitachi	1	5110(0.011432491139819366)	#SAKNAS!	#SAKNAS!
hive	1	5110(0.011432491139819366)	#SAKNAS!	#SAKNAS!
hiv-positive	1	5110(0.011432491139819366)	2359 (0.034)	#SAKNAS!
hiv-positive	1	5110(0.011432491139819366)	2359 (0.034)	#SAKNAS!
hmgcr	1	5110(0.011432491139819366)	#SAKNAS!	#SAKNAS!

hoarseness	1	5110(0.011432491139819366)	#SAKNAS!	#SAKNAS!
hodgkin	1	5110(0.011432491139819366)	2359 (0.034)	#SAKNAS!
hodgkin	1	5110(0.011432491139819366)	2359 (0.034)	#SAKNAS!
holarctic	1	5110(0.011432491139819366)	2359 (0.034)	#SAKNAS!
holarctic	1	5110(0.011432491139819366)	2359 (0.034)	#SAKNAS!
hold	1	5110(0.011432491139819366)	#SAKNAS!	#SAKNAS!
holes	1	5110(0.011432491139819366)	#SAKNAS!	2548 (0.033)
holidays	1	5110(0.011432491139819366)	#SAKNAS!	#SAKNAS!
holistic	1	5110(0.011432491139819366)	#SAKNAS!	#SAKNAS!
holobenthic	1	5110(0.011432491139819366)	#SAKNAS!	#SAKNAS!
holstein-zebu	1	5110(0.011432491139819366)	#SAKNAS!	2548 (0.033)
homa	1	5110(0.011432491139819366)	#SAKNAS!	#SAKNAS!
homebound	1	3437(0.02286498227963873)	2359 (0.034)	2548 (0.033)
homecare	1	5110(0.011432491139819366)	#SAKNAS!	2548 (0.033)
home-delivered	1	5110(0.011432491139819366)	#SAKNAS!	#SAKNAS!
home-dwelling	1	5110(0.011432491139819366)	#SAKNAS!	2548 (0.033)
homeless	1	5110(0.011432491139819366)	2359 (0.034)	#SAKNAS!
homeless	1	5110(0.011432491139819366)	2359 (0.034)	#SAKNAS!
homeostatic	1	2693(0.0342974734194581)	2359 (0.034)	1668 (0.066)
home-prepared	1	5110(0.011432491139819366)	#SAKNAS!	2548 (0.033)
homes-incidence	1	5110(0.011432491139819366)	#SAKNAS!	2548 (0.033)
homes-results	1	5110(0.011432491139819366)	#SAKNAS!	2548 (0.033)
homes-the	1	5110(0.011432491139819366)	#SAKNAS!	2548 (0.033)
home-visit	1	5110(0.011432491139819366)	#SAKNAS!	#SAKNAS!
homim	1	5110(0.011432491139819366)	#SAKNAS!	2548 (0.033)
hominids	1	5110(0.011432491139819366)	#SAKNAS!	2548 (0.033)
homo	1	5110(0.011432491139819366)	2359 (0.034)	#SAKNAS!
homo	1	5110(0.011432491139819366)	2359 (0.034)	#SAKNAS!
homocystein	1	5110(0.011432491139819366)	#SAKNAS!	#SAKNAS!
homo-exopolysaccharide	1	5110(0.011432491139819366)	#SAKNAS!	2548 (0.033)
homogeneity	1	5110(0.011432491139819366)	2359 (0.034)	#SAKNAS!
homogeneity	1	5110(0.011432491139819366)	2359 (0.034)	#SAKNAS!
homogenization	1	5110(0.011432491139819366)	#SAKNAS!	#SAKNAS!
honeybee	1	2214(0.04572996455927746)	2359 (0.034)	1233 (0.1)
honeyed	1	5110(0.011432491139819366)	#SAKNAS!	#SAKNAS!
hood	1	5110(0.011432491139819366)	2359 (0.034)	#SAKNAS!
hood	1	5110(0.011432491139819366)	2359 (0.034)	#SAKNAS!
hook	1	5110(0.011432491139819366)	2359 (0.034)	#SAKNAS!
hook	1	5110(0.011432491139819366)	2359 (0.034)	#SAKNAS!
hope	1	5110(0.011432491139819366)	2359 (0.034)	#SAKNAS!
hope	1	5110(0.011432491139819366)	2359 (0.034)	#SAKNAS!
horizons	1	5110(0.011432491139819366)	#SAKNAS!	#SAKNAS!
hornworm	1	3437(0.02286498227963873)	2359 (0.034)	2548 (0.033)

horribilis	1	5110(0.011432491139819366)	#SAKNAS!	#SAKNAS!
horse	1	2693(0.0342974734194581)	2359 (0.034)	2548 (0.033)
horseshoe-crab	1	5110(0.011432491139819366)	2359 (0.034)	#SAKNAS!
horseshoe-crab	1	5110(0.011432491139819366)	2359 (0.034)	#SAKNAS!
hospital-acquired	1	5110(0.011432491139819366)	#SAKNAS!	#SAKNAS!
hospitalizations	1	5110(0.011432491139819366)	#SAKNAS!	2548 (0.033)
hospiton	1	5110(0.011432491139819366)	#SAKNAS!	2548 (0.033)
host-environment	1	5110(0.011432491139819366)	#SAKNAS!	#SAKNAS!
host-locate	1	5110(0.011432491139819366)	#SAKNAS!	2548 (0.033)
host-plant	1	5110(0.011432491139819366)	2359 (0.034)	#SAKNAS!
host-plant	1	5110(0.011432491139819366)	2359 (0.034)	#SAKNAS!
host-seeking	1	5110(0.011432491139819366)	2359 (0.034)	#SAKNAS!
host-seeking	1	5110(0.011432491139819366)	2359 (0.034)	#SAKNAS!
hours	1	1894(0.05716245569909683)	2359 (0.034)	2548 (0.033)
house	1	5110(0.011432491139819366)	#SAKNAS!	2548 (0.033)
housebound	1	5110(0.011432491139819366)	2359 (0.034)	#SAKNAS!
housebound	1	5110(0.011432491139819366)	2359 (0.034)	#SAKNAS!
hoyuk	1	5110(0.011432491139819366)	#SAKNAS!	#SAKNAS!
hpp	1	5110(0.011432491139819366)	#SAKNAS!	#SAKNAS!
hpv	1	2214(0.04572996455927746)	2359 (0.034)	1233 (0.1)
hrept	1	5110(0.011432491139819366)	#SAKNAS!	2548 (0.033)
hscrp	1	5110(0.011432491139819366)	#SAKNAS!	#SAKNAS!
hs-crp	1	5110(0.011432491139819366)	2359 (0.034)	#SAKNAS!
hs-crp	1	5110(0.011432491139819366)	2359 (0.034)	#SAKNAS!
htr1b	1	5110(0.011432491139819366)	2359 (0.034)	#SAKNAS!
htr1b	1	5110(0.011432491139819366)	2359 (0.034)	#SAKNAS!
hua	1	5110(0.011432491139819366)	#SAKNAS!	#SAKNAS!
hualien	1	5110(0.011432491139819366)	2359 (0.034)	#SAKNAS!
hualien	1	5110(0.011432491139819366)	2359 (0.034)	#SAKNAS!
hudson	1	5110(0.011432491139819366)	#SAKNAS!	2548 (0.033)
huge	1	5110(0.011432491139819366)	#SAKNAS!	2548 (0.033)
huizhou	1	5110(0.011432491139819366)	#SAKNAS!	#SAKNAS!
human-influenced	1	5110(0.011432491139819366)	2359 (0.034)	#SAKNAS!
human-influenced	1	5110(0.011432491139819366)	2359 (0.034)	#SAKNAS!
humanitarian	1	5110(0.011432491139819366)	#SAKNAS!	2548 (0.033)
human-modified	1	5110(0.011432491139819366)	#SAKNAS!	#SAKNAS!
human-natural	1	5110(0.011432491139819366)	#SAKNAS!	2548 (0.033)
human-occupied	1	5110(0.011432491139819366)	#SAKNAS!	2548 (0.033)
humidity	1	3437(0.02286498227963873)	2359 (0.034)	#SAKNAS!
humpback	1	5110(0.011432491139819366)	2359 (0.034)	#SAKNAS!
humpback	1	5110(0.011432491139819366)	2359 (0.034)	#SAKNAS!
hungarian	1	2693(0.0342974734194581)	2359 (0.034)	#SAKNAS!
hungary	1	5110(0.011432491139819366)	2359 (0.034)	#SAKNAS!

hungary	1	5110(0.011432491139819366)	2359 (0.034)	#SAKNAS!
hunter-gatherer	1	5110(0.011432491139819366)	#SAKNAS!	#SAKNAS!
hunting	1	3437(0.02286498227963873)	2359 (0.034)	#SAKNAS!
huntingtin-associated	1	5110(0.011432491139819366)	2359 (0.034)	#SAKNAS!
huntingtin-associated	1	5110(0.011432491139819366)	2359 (0.034)	#SAKNAS!
huso	1	5110(0.011432491139819366)	2359 (0.034)	#SAKNAS!
huso	1	5110(0.011432491139819366)	2359 (0.034)	#SAKNAS!
hyaluronate	1	5110(0.011432491139819366)	#SAKNAS!	#SAKNAS!
hybrids	1	5110(0.011432491139819366)	#SAKNAS!	#SAKNAS!
hydrobia	1	5110(0.011432491139819366)	2359 (0.034)	#SAKNAS!
hydrobia	1	5110(0.011432491139819366)	2359 (0.034)	#SAKNAS!
hydrochloride	1	5110(0.011432491139819366)	2359 (0.034)	#SAKNAS!
hydrochloride	1	5110(0.011432491139819366)	2359 (0.034)	#SAKNAS!
hydrocorals	1	5110(0.011432491139819366)	#SAKNAS!	2548 (0.033)
hydrogel	1	5110(0.011432491139819366)	#SAKNAS!	2548 (0.033)
hydrogel-based	1	5110(0.011432491139819366)	#SAKNAS!	#SAKNAS!
hydrophilic	1	5110(0.011432491139819366)	#SAKNAS!	#SAKNAS!
hydrophobic	1	5110(0.011432491139819366)	#SAKNAS!	#SAKNAS!
hydroxide	1	5110(0.011432491139819366)	#SAKNAS!	2548 (0.033)
hydroxyapatite	1	5110(0.011432491139819366)	2359 (0.034)	#SAKNAS!
hydroxyapatite	1	5110(0.011432491139819366)	2359 (0.034)	#SAKNAS!
hydroxycitrate	1	5110(0.011432491139819366)	2359 (0.034)	#SAKNAS!
hydroxycitrate	1	5110(0.011432491139819366)	2359 (0.034)	#SAKNAS!
hygienic-dietary	1	5110(0.011432491139819366)	#SAKNAS!	#SAKNAS!
hygienist	1	5110(0.011432491139819366)	#SAKNAS!	#SAKNAS!
hygienists	1	1485(0.08002743797873557)	2359 (0.034)	1233 (0.1)
hylastes	1	5110(0.011432491139819366)	2359 (0.034)	#SAKNAS!
hylastes	1	5110(0.011432491139819366)	2359 (0.034)	#SAKNAS!
hymenolepis-diminuta	1	5110(0.011432491139819366)	2359 (0.034)	#SAKNAS!
hymenolepis-diminuta	1	5110(0.011432491139819366)	2359 (0.034)	#SAKNAS!
hymenopteran	1	5110(0.011432491139819366)	2359 (0.034)	#SAKNAS!
hymenopteran	1	5110(0.011432491139819366)	2359 (0.034)	#SAKNAS!
hyoid-complex	1	5110(0.011432491139819366)	#SAKNAS!	2548 (0.033)
hyoid-larynx	1	5110(0.011432491139819366)	#SAKNAS!	2548 (0.033)
hyolaryngeal	1	5110(0.011432491139819366)	#SAKNAS!	2548 (0.033)
hyperandrogenism	1	5110(0.011432491139819366)	2359 (0.034)	#SAKNAS!
hyperandrogenism	1	5110(0.011432491139819366)	2359 (0.034)	#SAKNAS!
hypercaloric	1	3437(0.02286498227963873)	2359 (0.034)	#SAKNAS!
hypercholesterolemia	1	5110(0.011432491139819366)	2359 (0.034)	#SAKNAS!
hypercholesterolemia	1	5110(0.011432491139819366)	2359 (0.034)	#SAKNAS!
hypercholesterolemic	1	5110(0.011432491139819366)	#SAKNAS!	2548 (0.033)
hyperglycemia	1	1678(0.0685949468389162)	2359 (0.034)	999 (0.13)
hyperhomocysteinemia	1	5110(0.011432491139819366)	#SAKNAS!	#SAKNAS!

hypericum	1	5110(0.011432491139819366)	#SAKNAS!	2548 (0.033)
hyperintensity	1	5110(0.011432491139819366)	#SAKNAS!	#SAKNAS!
hyperleptinemia	1	5110(0.011432491139819366)	2359 (0.034)	#SAKNAS!
hyperleptinemia	1	5110(0.011432491139819366)	2359 (0.034)	#SAKNAS!
hyperlipemia	1	5110(0.011432491139819366)	2359 (0.034)	#SAKNAS!
hyperlipemia	1	5110(0.011432491139819366)	2359 (0.034)	#SAKNAS!
hyperlipidemia	1	5110(0.011432491139819366)	#SAKNAS!	2548 (0.033)
hypermobility	1	5110(0.011432491139819366)	#SAKNAS!	2548 (0.033)
hypernatremia	1	5110(0.011432491139819366)	#SAKNAS!	2548 (0.033)
hyperostosis	1	5110(0.011432491139819366)	2359 (0.034)	#SAKNAS!
hyperostosis	1	5110(0.011432491139819366)	2359 (0.034)	#SAKNAS!
hyperparasitoid	1	5110(0.011432491139819366)	2359 (0.034)	#SAKNAS!
hyperparasitoid	1	5110(0.011432491139819366)	2359 (0.034)	#SAKNAS!
hyperphagia	1	2693(0.0342974734194581)	2359 (0.034)	2548 (0.033)
hyperprolactinemia	1	5110(0.011432491139819366)	#SAKNAS!	2548 (0.033)
hypersensitivity	1	5110(0.011432491139819366)	#SAKNAS!	2548 (0.033)
hypertense	1	5110(0.011432491139819366)	#SAKNAS!	#SAKNAS!
hypertension-associated	1	5110(0.011432491139819366)	#SAKNAS!	2548 (0.033)
hypertensives	1	5110(0.011432491139819366)	2359 (0.034)	#SAKNAS!
hypertensives	1	5110(0.011432491139819366)	2359 (0.034)	#SAKNAS!
hypertriglyceridemia	1	3437(0.02286498227963873)	2359 (0.034)	#SAKNAS!
hypertrophy	1	5110(0.011432491139819366)	#SAKNAS!	#SAKNAS!
hypnodiet	1	5110(0.011432491139819366)	#SAKNAS!	#SAKNAS!
hypnosis	1	5110(0.011432491139819366)	#SAKNAS!	#SAKNAS!
hypoalbuminemia	1	5110(0.011432491139819366)	#SAKNAS!	2548 (0.033)
hypoallergenic	1	5110(0.011432491139819366)	#SAKNAS!	#SAKNAS!
hypocretin-1	1	5110(0.011432491139819366)	2359 (0.034)	#SAKNAS!
hypocretin-1	1	5110(0.011432491139819366)	2359 (0.034)	#SAKNAS!
hypoglycemia	1	5110(0.011432491139819366)	#SAKNAS!	#SAKNAS!
hypoglycemic	1	5110(0.011432491139819366)	#SAKNAS!	#SAKNAS!
hypokalemia	1	5110(0.011432491139819366)	#SAKNAS!	#SAKNAS!
hypomineralisation	1	5110(0.011432491139819366)	#SAKNAS!	#SAKNAS!
hypomineralized	1	5110(0.011432491139819366)	#SAKNAS!	#SAKNAS!
hypomyelination	1	5110(0.011432491139819366)	#SAKNAS!	#SAKNAS!
hypophagia	1	5110(0.011432491139819366)	2359 (0.034)	#SAKNAS!
hypophagia	1	5110(0.011432491139819366)	2359 (0.034)	#SAKNAS!
hypopharynx	1	2693(0.0342974734194581)	2359 (0.034)	2548 (0.033)
hypophosphatemic	1	5110(0.011432491139819366)	2359 (0.034)	#SAKNAS!
hypophosphatemic	1	5110(0.011432491139819366)	2359 (0.034)	#SAKNAS!
hypothalamus-pituitary	1	5110(0.011432491139819366)	#SAKNAS!	#SAKNAS!
hypotheses	1	5110(0.011432491139819366)	#SAKNAS!	2548 (0.033)
hypouricemic	1	5110(0.011432491139819366)	#SAKNAS!	#SAKNAS!
hysterophorus	1	5110(0.011432491139819366)	2359 (0.034)	#SAKNAS!

hysterophorus	1	5110(0.011432491139819366)	2359 (0.034)	#SAKNAS!
hystricognathi	1	5110(0.011432491139819366)	2359 (0.034)	#SAKNAS!
hystricognathi	1	5110(0.011432491139819366)	2359 (0.034)	#SAKNAS!
i-1079	1	5110(0.011432491139819366)	#SAKNAS!	#SAKNAS!
i-3856	1	5110(0.011432491139819366)	#SAKNAS!	2548 (0.033)
iana	1	5110(0.011432491139819366)	2359 (0.034)	#SAKNAS!
iana	1	5110(0.011432491139819366)	2359 (0.034)	#SAKNAS!
iasi	1	5110(0.011432491139819366)	#SAKNAS!	2548 (0.033)
iatrogenic	1	3437(0.02286498227963873)	2359 (0.034)	2548 (0.033)
ib	1	5110(0.011432491139819366)	#SAKNAS!	2548 (0.033)
ibadan	1	5110(0.011432491139819366)	2359 (0.034)	#SAKNAS!
ibadan	1	5110(0.011432491139819366)	2359 (0.034)	#SAKNAS!
ibex	1	5110(0.011432491139819366)	#SAKNAS!	2548 (0.033)
ice-cream	1	5110(0.011432491139819366)	2359 (0.034)	#SAKNAS!
ice-cream	1	5110(0.011432491139819366)	2359 (0.034)	#SAKNAS!
icelandic	1	5110(0.011432491139819366)	2359 (0.034)	#SAKNAS!
icelandic	1	5110(0.011432491139819366)	2359 (0.034)	#SAKNAS!
iceman	1	5110(0.011432491139819366)	#SAKNAS!	#SAKNAS!
icm	1	5110(0.011432491139819366)	#SAKNAS!	2548 (0.033)
id	1	5110(0.011432491139819366)	#SAKNAS!	#SAKNAS!
idaho	1	5110(0.011432491139819366)	2359 (0.034)	#SAKNAS!
idaho	1	5110(0.011432491139819366)	2359 (0.034)	#SAKNAS!
ideation	1	5110(0.011432491139819366)	#SAKNAS!	2548 (0.033)
idefics	1	5110(0.011432491139819366)	#SAKNAS!	2548 (0.033)
idella	1	5110(0.011432491139819366)	#SAKNAS!	2548 (0.033)
idf	1	5110(0.011432491139819366)	#SAKNAS!	2548 (0.033)
iga	1	5110(0.011432491139819366)	2359 (0.034)	#SAKNAS!
iga	1	5110(0.011432491139819366)	2359 (0.034)	#SAKNAS!
igf	1	5110(0.011432491139819366)	#SAKNAS!	2548 (0.033)
igf1	1	5110(0.011432491139819366)	2359 (0.034)	#SAKNAS!
igf1	1	5110(0.011432491139819366)	2359 (0.034)	#SAKNAS!
igf-1	1	5110(0.011432491139819366)	2359 (0.034)	#SAKNAS!
igf-1	1	5110(0.011432491139819366)	2359 (0.034)	#SAKNAS!
igfbp-2	1	5110(0.011432491139819366)	2359 (0.034)	#SAKNAS!
igfbp-2	1	5110(0.011432491139819366)	2359 (0.034)	#SAKNAS!
igf-i	1	5110(0.011432491139819366)	2359 (0.034)	#SAKNAS!
igf-i	1	5110(0.011432491139819366)	2359 (0.034)	#SAKNAS!
igf-ii	1	5110(0.011432491139819366)	2359 (0.034)	#SAKNAS!
igf-ii	1	5110(0.011432491139819366)	2359 (0.034)	#SAKNAS!
igg	1	3437(0.02286498227963873)	2359 (0.034)	2548 (0.033)
igr	1	5110(0.011432491139819366)	2359 (0.034)	#SAKNAS!
igr	1	5110(0.011432491139819366)	2359 (0.034)	#SAKNAS!
igy	1	5110(0.011432491139819366)	#SAKNAS!	2548 (0.033)

iii-iv	1	5110(0.011432491139819366)	2359 (0.034)	#SAKNAS!
iii-iv	1	5110(0.011432491139819366)	2359 (0.034)	#SAKNAS!
iii-phase	1	5110(0.011432491139819366)	2359 (0.034)	#SAKNAS!
iii-phase	1	5110(0.011432491139819366)	2359 (0.034)	#SAKNAS!
il-10	1	5110(0.011432491139819366)	#SAKNAS!	2548 (0.033)
il10-1082	1	5110(0.011432491139819366)	2359 (0.034)	#SAKNAS!
il10-1082	1	5110(0.011432491139819366)	2359 (0.034)	#SAKNAS!
il6	1	5110(0.011432491139819366)	#SAKNAS!	2548 (0.033)
il-6	1	2693(0.0342974734194581)	2359 (0.034)	1668 (0.066)
il-6-174	1	5110(0.011432491139819366)	2359 (0.034)	#SAKNAS!
il-6-174	1	5110(0.011432491139819366)	2359 (0.034)	#SAKNAS!
il-8	1	5110(0.011432491139819366)	#SAKNAS!	2548 (0.033)
ileal	1	5110(0.011432491139819366)	#SAKNAS!	#SAKNAS!
illicit	1	5110(0.011432491139819366)	#SAKNAS!	#SAKNAS!
illuminate	1	5110(0.011432491139819366)	#SAKNAS!	2548 (0.033)
imam	1	5110(0.011432491139819366)	#SAKNAS!	2548 (0.033)
imbalance	1	2693(0.0342974734194581)	2359 (0.034)	2548 (0.033)
imbricata	1	5110(0.011432491139819366)	2359 (0.034)	#SAKNAS!
imbricata	1	5110(0.011432491139819366)	2359 (0.034)	#SAKNAS!
imidazole-containing	1	5110(0.011432491139819366)	2359 (0.034)	#SAKNAS!
imidazole-containing	1	5110(0.011432491139819366)	2359 (0.034)	#SAKNAS!
immediate	1	2693(0.0342974734194581)	2359 (0.034)	#SAKNAS!
immediately	1	5110(0.011432491139819366)	#SAKNAS!	2548 (0.033)
immigrants	1	5110(0.011432491139819366)	#SAKNAS!	2548 (0.033)
immobility	1	5110(0.011432491139819366)	#SAKNAS!	2548 (0.033)
immortalization	1	5110(0.011432491139819366)	2359 (0.034)	#SAKNAS!
immortalization	1	5110(0.011432491139819366)	2359 (0.034)	#SAKNAS!
immunity	1	1335(0.09145992911855493)	2359 (0.034)	999 (0.13)
immunization	1	5110(0.011432491139819366)	#SAKNAS!	2548 (0.033)
immuno	1	5110(0.011432491139819366)	#SAKNAS!	2548 (0.033)
immunoglobulins	1	5110(0.011432491139819366)	#SAKNAS!	#SAKNAS!
immunohistochemical	1	5110(0.011432491139819366)	2359 (0.034)	#SAKNAS!
immunohistochemical	1	5110(0.011432491139819366)	2359 (0.034)	#SAKNAS!
immunoinflammatory	1	5110(0.011432491139819366)	#SAKNAS!	#SAKNAS!
immunologic	1	5110(0.011432491139819366)	#SAKNAS!	2548 (0.033)
immunometabolic	1	5110(0.011432491139819366)	#SAKNAS!	#SAKNAS!
immunopathogeneses	1	5110(0.011432491139819366)	2359 (0.034)	#SAKNAS!
immunopathogeneses	1	5110(0.011432491139819366)	2359 (0.034)	#SAKNAS!
immunotest	1	5110(0.011432491139819366)	#SAKNAS!	2548 (0.033)
immunotherapy-related	1	5110(0.011432491139819366)	#SAKNAS!	#SAKNAS!
impacto	1	5110(0.011432491139819366)	#SAKNAS!	#SAKNAS!
impairing	1	5110(0.011432491139819366)	#SAKNAS!	#SAKNAS!
impairment-mbsimp	1	5110(0.011432491139819366)	2359 (0.034)	#SAKNAS!

impairment-mbsimp	1	5110(0.011432491139819366)	2359 (0.034)	#SAKNAS!
impenetrable	1	5110(0.011432491139819366)	2359 (0.034)	#SAKNAS!
impenetrable	1	5110(0.011432491139819366)	2359 (0.034)	#SAKNAS!
implant-assisted	1	5110(0.011432491139819366)	2359 (0.034)	#SAKNAS!
implant-assisted	1	5110(0.011432491139819366)	2359 (0.034)	#SAKNAS!
implantation	1	5110(0.011432491139819366)	#SAKNAS!	2548 (0.033)
implantology	1	5110(0.011432491139819366)	#SAKNAS!	#SAKNAS!
implementing	1	5110(0.011432491139819366)	2359 (0.034)	#SAKNAS!
implementing	1	5110(0.011432491139819366)	2359 (0.034)	#SAKNAS!
implication	1	2693(0.0342974734194581)	2359 (0.034)	2548 (0.033)
import	1	5110(0.011432491139819366)	#SAKNAS!	#SAKNAS!
imprinting	1	3437(0.02286498227963873)	2359 (0.034)	#SAKNAS!
improvements	1	2693(0.0342974734194581)	2359 (0.034)	#SAKNAS!
impulse	1	5110(0.011432491139819366)	#SAKNAS!	2548 (0.033)
imrt-sib	1	5110(0.011432491139819366)	#SAKNAS!	2548 (0.033)
imu	1	5110(0.011432491139819366)	#SAKNAS!	#SAKNAS!
inability	1	5110(0.011432491139819366)	#SAKNAS!	#SAKNAS!
inaccurate	1	5110(0.011432491139819366)	#SAKNAS!	2548 (0.033)
inactivation	1	5110(0.011432491139819366)	2359 (0.034)	#SAKNAS!
inactivation	1	5110(0.011432491139819366)	2359 (0.034)	#SAKNAS!
inactive	1	5110(0.011432491139819366)	2359 (0.034)	#SAKNAS!
inactive	1	5110(0.011432491139819366)	2359 (0.034)	#SAKNAS!
inactivity	1	5110(0.011432491139819366)	#SAKNAS!	#SAKNAS!
inadequacies	1	5110(0.011432491139819366)	#SAKNAS!	2548 (0.033)
inadequate	1	3437(0.02286498227963873)	2359 (0.034)	2548 (0.033)
inanopheles	1	5110(0.011432491139819366)	#SAKNAS!	2548 (0.033)
inattention	1	5110(0.011432491139819366)	2359 (0.034)	#SAKNAS!
inattention	1	5110(0.011432491139819366)	2359 (0.034)	#SAKNAS!
inauris	1	5110(0.011432491139819366)	2359 (0.034)	#SAKNAS!
inauris	1	5110(0.011432491139819366)	2359 (0.034)	#SAKNAS!
incerta	1	5110(0.011432491139819366)	#SAKNAS!	2548 (0.033)
incidences	1	5110(0.011432491139819366)	#SAKNAS!	2548 (0.033)
incidental	1	5110(0.011432491139819366)	#SAKNAS!	#SAKNAS!
incidentaloma	1	5110(0.011432491139819366)	#SAKNAS!	#SAKNAS!
incilius	1	5110(0.011432491139819366)	#SAKNAS!	#SAKNAS!
incision	1	5110(0.011432491139819366)	#SAKNAS!	2548 (0.033)
incisor	1	2693(0.0342974734194581)	2359 (0.034)	2548 (0.033)
include	1	5110(0.011432491139819366)	#SAKNAS!	2548 (0.033)
includes	1	5110(0.011432491139819366)	2359 (0.034)	#SAKNAS!
includes	1	5110(0.011432491139819366)	2359 (0.034)	#SAKNAS!
income-disparities	1	5110(0.011432491139819366)	#SAKNAS!	2548 (0.033)
income-related	1	5110(0.011432491139819366)	#SAKNAS!	2548 (0.033)
incontinence	1	5110(0.011432491139819366)	#SAKNAS!	#SAKNAS!

incorporation	1	5110(0.011432491139819366)	2359 (0.034)	#SAKNAS!
incorporation	1	5110(0.011432491139819366)	2359 (0.034)	#SAKNAS!
increment	1	2693(0.0342974734194581)	2359 (0.034)	1668 (0.066)
incretin	1	5110(0.011432491139819366)	#SAKNAS!	#SAKNAS!
incubation	1	5110(0.011432491139819366)	2359 (0.034)	#SAKNAS!
incubation	1	5110(0.011432491139819366)	2359 (0.034)	#SAKNAS!
incurable	1	5110(0.011432491139819366)	#SAKNAS!	2548 (0.033)
independent-living	1	5110(0.011432491139819366)	2359 (0.034)	#SAKNAS!
independent-living	1	5110(0.011432491139819366)	2359 (0.034)	#SAKNAS!
index-2005	1	5110(0.011432491139819366)	#SAKNAS!	2548 (0.033)
index-a	1	5110(0.011432491139819366)	#SAKNAS!	#SAKNAS!
index-for-age	1	5110(0.011432491139819366)	2359 (0.034)	#SAKNAS!
index-for-age	1	5110(0.011432491139819366)	2359 (0.034)	#SAKNAS!
index-related	1	5110(0.011432491139819366)	#SAKNAS!	#SAKNAS!
indians	1	5110(0.011432491139819366)	2359 (0.034)	#SAKNAS!
indians	1	5110(0.011432491139819366)	2359 (0.034)	#SAKNAS!
indication	1	5110(0.011432491139819366)	#SAKNAS!	2548 (0.033)
indication-dependent	1	5110(0.011432491139819366)	#SAKNAS!	2548 (0.033)
indicus	1	5110(0.011432491139819366)	#SAKNAS!	#SAKNAS!
indies	1	5110(0.011432491139819366)	#SAKNAS!	2548 (0.033)
indigenous	1	966(0.1371898936778324)	2359 (0.034)	816 (0.16)
indispensable	1	5110(0.011432491139819366)	#SAKNAS!	2548 (0.033)
individuality	1	5110(0.011432491139819366)	#SAKNAS!	2548 (0.033)
indolent	1	5110(0.011432491139819366)	2359 (0.034)	#SAKNAS!
indolent	1	5110(0.011432491139819366)	2359 (0.034)	#SAKNAS!
indomethacin	1	5110(0.011432491139819366)	#SAKNAS!	#SAKNAS!
indonesia	1	1027(0.12575740253801304)	2359 (0.034)	694 (0.2)
indonesian	1	5110(0.011432491139819366)	2359 (0.034)	#SAKNAS!
indonesian	1	5110(0.011432491139819366)	2359 (0.034)	#SAKNAS!
indoor-fed	1	5110(0.011432491139819366)	#SAKNAS!	#SAKNAS!
indoors	1	5110(0.011432491139819366)	#SAKNAS!	#SAKNAS!
indo-pacific	1	5110(0.011432491139819366)	#SAKNAS!	#SAKNAS!
indri	1	5110(0.011432491139819366)	2359 (0.034)	#SAKNAS!
indri	1	5110(0.011432491139819366)	2359 (0.034)	#SAKNAS!
induce	1	5110(0.011432491139819366)	#SAKNAS!	#SAKNAS!
inducer	1	5110(0.011432491139819366)	#SAKNAS!	2548 (0.033)
inducing	1	5110(0.011432491139819366)	#SAKNAS!	2548 (0.033)
industrial	1	5110(0.011432491139819366)	#SAKNAS!	2548 (0.033)
industrial-populations	1	5110(0.011432491139819366)	2359 (0.034)	#SAKNAS!
industrial-populations	1	5110(0.011432491139819366)	2359 (0.034)	#SAKNAS!
in-dwelling	1	5110(0.011432491139819366)	#SAKNAS!	#SAKNAS!
inedible	1	5110(0.011432491139819366)	#SAKNAS!	2548 (0.033)
inequitable-a	1	5110(0.011432491139819366)	#SAKNAS!	#SAKNAS!

inequities	1	5110(0.011432491139819366)	#SAKNAS!	#SAKNAS!
inerme	1	5110(0.011432491139819366)	#SAKNAS!	2548 (0.033)
inertial	1	5110(0.011432491139819366)	#SAKNAS!	#SAKNAS!
ines	1	5110(0.011432491139819366)	2359 (0.034)	#SAKNAS!
ines	1	5110(0.011432491139819366)	2359 (0.034)	#SAKNAS!
inexpensive	1	5110(0.011432491139819366)	2359 (0.034)	#SAKNAS!
inexpensive	1	5110(0.011432491139819366)	2359 (0.034)	#SAKNAS!
infantile	1	5110(0.011432491139819366)	2359 (0.034)	#SAKNAS!
infantile	1	5110(0.011432491139819366)	2359 (0.034)	#SAKNAS!
infant-oriented	1	5110(0.011432491139819366)	2359 (0.034)	#SAKNAS!
infant-oriented	1	5110(0.011432491139819366)	2359 (0.034)	#SAKNAS!
infarct	1	5110(0.011432491139819366)	#SAKNAS!	#SAKNAS!
infective	1	5110(0.011432491139819366)	#SAKNAS!	2548 (0.033)
in-feed	1	5110(0.011432491139819366)	2359 (0.034)	#SAKNAS!
in-feed	1	5110(0.011432491139819366)	2359 (0.034)	#SAKNAS!
inference	1	5110(0.011432491139819366)	#SAKNAS!	#SAKNAS!
inferior	1	5110(0.011432491139819366)	#SAKNAS!	2548 (0.033)
inferring	1	5110(0.011432491139819366)	2359 (0.034)	#SAKNAS!
inferring	1	5110(0.011432491139819366)	2359 (0.034)	#SAKNAS!
infertility	1	5110(0.011432491139819366)	#SAKNAS!	2548 (0.033)
infiltrants	1	5110(0.011432491139819366)	#SAKNAS!	#SAKNAS!
infiltration	1	5110(0.011432491139819366)	#SAKNAS!	2548 (0.033)
infirmary	1	5110(0.011432491139819366)	2359 (0.034)	#SAKNAS!
infirmary	1	5110(0.011432491139819366)	2359 (0.034)	#SAKNAS!
inflammaging	1	5110(0.011432491139819366)	#SAKNAS!	#SAKNAS!
inflammasome	1	5110(0.011432491139819366)	#SAKNAS!	#SAKNAS!
infliximab	1	5110(0.011432491139819366)	#SAKNAS!	2548 (0.033)
influencers	1	5110(0.011432491139819366)	#SAKNAS!	2548 (0.033)
influenza	1	5110(0.011432491139819366)	#SAKNAS!	#SAKNAS!
influx	1	5110(0.011432491139819366)	2359 (0.034)	#SAKNAS!
influx	1	5110(0.011432491139819366)	2359 (0.034)	#SAKNAS!
informatics	1	5110(0.011432491139819366)	#SAKNAS!	2548 (0.033)
information-motivation-behavio	1	5110(0.011432491139819366)	#SAKNAS!	2548 (0.033)
informative	1	5110(0.011432491139819366)	2359 (0.034)	#SAKNAS!
informative	1	5110(0.011432491139819366)	2359 (0.034)	#SAKNAS!
infusional	1	5110(0.011432491139819366)	2359 (0.034)	#SAKNAS!
infusional	1	5110(0.011432491139819366)	2359 (0.034)	#SAKNAS!
ingestion-related	1	5110(0.011432491139819366)	2359 (0.034)	#SAKNAS!
ingestion-related	1	5110(0.011432491139819366)	2359 (0.034)	#SAKNAS!
ingredient-based	1	5110(0.011432491139819366)	#SAKNAS!	2548 (0.033)
ingredients	1	2214(0.04572996455927746)	2359 (0.034)	2548 (0.033)
inhabitant	1	5110(0.011432491139819366)	#SAKNAS!	#SAKNAS!
inhabitants	1	5110(0.011432491139819366)	#SAKNAS!	2548 (0.033)

inhabiting	1	2693(0.0342974734194581)	2359 (0.034)	1668 (0.066)
inhibit	1	3437(0.0228649822796387З)	2359 (0.034)	#SAKNAS!
inhibitor-1	1	5110(0.011432491139819366)	2359 (0.034)	#SAKNAS!
inhibitor-1	1	5110(0.011432491139819366)	2359 (0.034)	#SAKNAS!
inhibitors	1	5110(0.011432491139819366)	#SAKNAS!	#SAKNAS!
inhibits	1	2693(0.0342974734194581)	2359 (0.034)	2548 (0.033)
in-hospital	1	2214(0.04572996455927746)	2359 (0.034)	#SAKNAS!
ini-0602	1	5110(0.011432491139819366)	#SAKNAS!	2548 (0.033)
initiate	1	5110(0.011432491139819366)	#SAKNAS!	2548 (0.033)
initiation	1	2214(0.04572996455927746)	2359 (0.034)	#SAKNAS!
initiative	1	1219(0.1028924202583743)	2359 (0.034)	1668 (0.066)
injecting	1	5110(0.011432491139819366)	2359 (0.034)	#SAKNAS!
injecting	1	5110(0.011432491139819366)	2359 (0.034)	#SAKNAS!
injections	1	3437(0.02286498227963873)	2359 (0.034)	2548 (0.033)
inmates	1	5110(0.011432491139819366)	#SAKNAS!	#SAKNAS!
innate	1	5110(0.011432491139819366)	2359 (0.034)	#SAKNAS!
innate	1	5110(0.011432491139819366)	2359 (0.034)	#SAKNAS!
inner-city-dwelling	1	5110(0.011432491139819366)	2359 (0.034)	#SAKNAS!
inner-city-dwelling	1	5110(0.011432491139819366)	2359 (0.034)	#SAKNAS!
innervation	1	5110(0.011432491139819366)	#SAKNAS!	2548 (0.033)
inoculation	1	5110(0.011432491139819366)	#SAKNAS!	#SAKNAS!
inoculum	1	5110(0.011432491139819366)	#SAKNAS!	2548 (0.033)
inorganic	1	2693(0.0342974734194581)	2359 (0.034)	#SAKNAS!
in-patients	1	5110(0.011432491139819366)	#SAKNAS!	2548 (0.033)
insect-dwelling	1	5110(0.011432491139819366)	#SAKNAS!	#SAKNAS!
insecticidal	1	3437(0.02286498227963873)	2359 (0.034)	2548 (0.033)
insectivore	1	5110(0.011432491139819366)	#SAKNAS!	2548 (0.033)
insecure	1	5110(0.011432491139819366)	#SAKNAS!	#SAKNAS!
insecurity	1	710(0.19435234937692925)	2359 (0.034)	816 (0.16)
insemap	1	5110(0.011432491139819366)	#SAKNAS!	#SAKNAS!
insensitive	1	5110(0.011432491139819366)	#SAKNAS!	#SAKNAS!
insight	1	5110(0.011432491139819366)	#SAKNAS!	#SAKNAS!
insights	1	481(0.2858122784954842)	2359 (0.034)	816 (0.16)
insomnia	1	5110(0.011432491139819366)	#SAKNAS!	2548 (0.033)
inspirations	1	5110(0.011432491139819366)	#SAKNAS!	#SAKNAS!
instability	1	5110(0.011432491139819366)	#SAKNAS!	2548 (0.033)
instantaneous	1	5110(0.011432491139819366)	#SAKNAS!	#SAKNAS!
institutes	1	5110(0.011432491139819366)	#SAKNAS!	2548 (0.033)
institutional	1	3437(0.02286498227963873)	2359 (0.034)	2548 (0.033)
institutions	1	5110(0.011432491139819366)	2359 (0.034)	#SAKNAS!
institutions	1	5110(0.011432491139819366)	2359 (0.034)	#SAKNAS!
instituto	1	5110(0.011432491139819366)	#SAKNAS!	2548 (0.033)
in-store	1	5110(0.011432491139819366)	#SAKNAS!	2548 (0.033)

instream	1	5110(0.011432491139819366)	#SAKNAS!	#SAKNAS!
instreptococcus	1	5110(0.011432491139819366)	#SAKNAS!	2548 (0.033)
instruction	1	5110(0.011432491139819366)	#SAKNAS!	2548 (0.033)
instrumentation	1	2693(0.0342974734194581)	2359 (0.034)	2548 (0.033)
instrumentation-a	1	5110(0.011432491139819366)	#SAKNAS!	2548 (0.033)
instruments	1	5110(0.011432491139819366)	#SAKNAS!	#SAKNAS!
insufficiency	1	2693(0.0342974734194581)	2359 (0.034)	2548 (0.033)
insulation	1	5110(0.011432491139819366)	2359 (0.034)	#SAKNAS!
insulation	1	5110(0.011432491139819366)	2359 (0.034)	#SAKNAS!
insulin-like	1	5110(0.011432491139819366)	#SAKNAS!	#SAKNAS!
intact	1	3437(0.02286498227963873)	2359 (0.034)	#SAKNAS!
integral	1	5110(0.011432491139819366)	#SAKNAS!	2548 (0.033)
integrate	1	5110(0.011432491139819366)	#SAKNAS!	#SAKNAS!
integrating	1	1894(0.05716245569909683)	2359 (0.034)	1233 (0.1)
integrative	1	1894(0.05716245569909683)	2359 (0.034)	1668 (0.066)
integrator	1	5110(0.011432491139819366)	2359 (0.034)	#SAKNAS!
integrator	1	5110(0.011432491139819366)	2359 (0.034)	#SAKNAS!
intellectual	1	1485(0.08002743797873557)	2359 (0.034)	816 (0.16)
intellectually	1	5110(0.011432491139819366)	#SAKNAS!	2548 (0.033)
intelligence	1	5110(0.011432491139819366)	#SAKNAS!	#SAKNAS!
intensity	1	1485(0.08002743797873557)	2359 (0.034)	1668 (0.066)
interactive	1	1894(0.05716245569909683)	2359 (0.034)	1668 (0.066)
interactivity	1	5110(0.011432491139819366)	#SAKNAS!	#SAKNAS!
interannual	1	3437(0.02286498227963873)	2359 (0.034)	#SAKNAS!
inter-arch	1	5110(0.011432491139819366)	#SAKNAS!	2548 (0.033)
interbody	1	5110(0.011432491139819366)	#SAKNAS!	#SAKNAS!
intercollegiate	1	5110(0.011432491139819366)	#SAKNAS!	2548 (0.033)
interconnectedness	1	5110(0.011432491139819366)	#SAKNAS!	#SAKNAS!
inter-dental	1	5110(0.011432491139819366)	#SAKNAS!	2548 (0.033)
interdepartmental	1	5110(0.011432491139819366)	2359 (0.034)	#SAKNAS!
interdepartmental	1	5110(0.011432491139819366)	2359 (0.034)	#SAKNAS!
interdependency	1	5110(0.011432491139819366)	2359 (0.034)	#SAKNAS!
interdependency	1	5110(0.011432491139819366)	2359 (0.034)	#SAKNAS!
interdisciplinary	1	2214(0.04572996455927746)	2359 (0.034)	1233 (0.1)
interest	1	3437(0.02286498227963873)	2359 (0.034)	2548 (0.033)
interface	1	5110(0.011432491139819366)	2359 (0.034)	#SAKNAS!
interface	1	5110(0.011432491139819366)	2359 (0.034)	#SAKNAS!
interface-feeding	1	5110(0.011432491139819366)	2359 (0.034)	#SAKNAS!
interface-feeding	1	5110(0.011432491139819366)	2359 (0.034)	#SAKNAS!
interfere	1	5110(0.011432491139819366)	#SAKNAS!	2548 (0.033)
interim	1	5110(0.011432491139819366)	2359 (0.034)	#SAKNAS!
interim	1	5110(0.011432491139819366)	2359 (0.034)	#SAKNAS!
inter-individual	1	5110(0.011432491139819366)	#SAKNAS!	2548 (0.033)

interleukin	1	2693(0.0342974734194581)	2359 (0.034)	1668 (0.066)
interleukin-1	1	5110(0.011432491139819366)	#SAKNAS!	2548 (0.033)
interleukin-10	1	5110(0.011432491139819366)	2359 (0.034)	#SAKNAS!
interleukin-10	1	5110(0.011432491139819366)	2359 (0.034)	#SAKNAS!
interleukin-17	1	5110(0.011432491139819366)	2359 (0.034)	#SAKNAS!
interleukin-17	1	5110(0.011432491139819366)	2359 (0.034)	#SAKNAS!
interleukin-1桊	1	5110(0.011432491139819366)	2359 (0.034)	#SAKNAS!
interleukin-1桊	1	5110(0.011432491139819366)	2359 (0.034)	#SAKNAS!
intermedia	1	5110(0.011432491139819366)	#SAKNAS!	2548 (0.033)
intermediate	1	5110(0.011432491139819366)	2359 (0.034)	#SAKNAS!
intermediate	1	5110(0.011432491139819366)	2359 (0.034)	#SAKNAS!
internet	1	3437(0.02286498227963873)	2359 (0.034)	2548 (0.033)
inter-patch	1	5110(0.011432491139819366)	2359 (0.034)	#SAKNAS!
inter-patch	1	5110(0.011432491139819366)	2359 (0.034)	#SAKNAS!
interpopulation	1	5110(0.011432491139819366)	#SAKNAS!	#SAKNAS!
interpretation	1	3437(0.02286498227963873)	2359 (0.034)	#SAKNAS!
interpretive	1	5110(0.011432491139819366)	#SAKNAS!	2548 (0.033)
interprofessional	1	1335(0.09145992911855493)	2359 (0.034)	816 (0.16)
inter-professional	1	5110(0.011432491139819366)	#SAKNAS!	#SAKNAS!
interrai	1	5110(0.011432491139819366)	#SAKNAS!	2548 (0.033)
interrater	1	5110(0.011432491139819366)	#SAKNAS!	#SAKNAS!
inter-rater	1	5110(0.011432491139819366)	2359 (0.034)	#SAKNAS!
inter-rater	1	5110(0.011432491139819366)	2359 (0.034)	#SAKNAS!
interrelation	1	3437(0.02286498227963873)	2359 (0.034)	2548 (0.033)
interrogate	1	5110(0.011432491139819366)	#SAKNAS!	#SAKNAS!
intersections	1	5110(0.011432491139819366)	#SAKNAS!	#SAKNAS!
interspecific	1	5110(0.011432491139819366)	2359 (0.034)	#SAKNAS!
interspecific	1	5110(0.011432491139819366)	2359 (0.034)	#SAKNAS!
intersucking	1	5110(0.011432491139819366)	2359 (0.034)	#SAKNAS!
intersucking	1	5110(0.011432491139819366)	2359 (0.034)	#SAKNAS!
interval	1	3437(0.02286498227963873)	2359 (0.034)	#SAKNAS!
intervene	1	5110(0.011432491139819366)	#SAKNAS!	2548 (0.033)
interventional	1	1894(0.05716245569909683)	2359 (0.034)	#SAKNAS!
interview	1	2214(0.04572996455927746)	2359 (0.034)	#SAKNAS!
interviewing	1	3437(0.02286498227963873)	2359 (0.034)	#SAKNAS!
intestinal-transplant	1	5110(0.011432491139819366)	#SAKNAS!	2548 (0.033)
intestines	1	5110(0.011432491139819366)	2359 (0.034)	#SAKNAS!
intestines	1	5110(0.011432491139819366)	2359 (0.034)	#SAKNAS!
intoxicated	1	5110(0.011432491139819366)	#SAKNAS!	#SAKNAS!
intoxication	1	5110(0.011432491139819366)	2359 (0.034)	#SAKNAS!
intoxication	1	5110(0.011432491139819366)	2359 (0.034)	#SAKNAS!
intra	1	5110(0.011432491139819366)	#SAKNAS!	#SAKNAS!
intra-arterial	1	3437(0.02286498227963873)	2359 (0.034)	2548 (0.033)

intracellular	1	5110(0.011432491139819366)	#SAKNAS!	#SAKNAS!
intracerebral	1	1678(0.0685949468389162)	2359 (0.034)	1233 (0.1)
intracerebroventricular	1	3437(0.02286498227963873)	2359 (0.034)	2548 (0.033)
intracranial	1	3437(0.02286498227963873)	2359 (0.034)	#SAKNAS!
intractable	1	3437(0.02286498227963873)	2359 (0.034)	2548 (0.033)
intragastric	1	5110(0.011432491139819366)	2359 (0.034)	#SAKNAS!
intragastric	1	5110(0.011432491139819366)	2359 (0.034)	#SAKNAS!
intranasal	1	5110(0.011432491139819366)	#SAKNAS!	2548 (0.033)
intraoperative	1	5110(0.011432491139819366)	#SAKNAS!	#SAKNAS!
intraperitoneal	1	5110(0.011432491139819366)	#SAKNAS!	2548 (0.033)
intra-plant	1	5110(0.011432491139819366)	2359 (0.034)	#SAKNAS!
intra-plant	1	5110(0.011432491139819366)	2359 (0.034)	#SAKNAS!
intraruminal	1	5110(0.011432491139819366)	2359 (0.034)	#SAKNAS!
intraruminal	1	5110(0.011432491139819366)	2359 (0.034)	#SAKNAS!
intra-sexual	1	5110(0.011432491139819366)	#SAKNAS!	2548 (0.033)
intraspecies	1	5110(0.011432491139819366)	2359 (0.034)	#SAKNAS!
intraspecies	1	5110(0.011432491139819366)	2359 (0.034)	#SAKNAS!
intraspecific	1	5110(0.011432491139819366)	2359 (0.034)	#SAKNAS!
intraspecific	1	5110(0.011432491139819366)	2359 (0.034)	#SAKNAS!
intrasphincteric	1	3437(0.02286498227963873)	2359 (0.034)	2548 (0.033)
intrathecal	1	5110(0.011432491139819366)	2359 (0.034)	#SAKNAS!
intrathecal	1	5110(0.011432491139819366)	2359 (0.034)	#SAKNAS!
intra-thoracic	1	3437(0.02286498227963873)	2359 (0.034)	2548 (0.033)
introduction	1	1335(0.09145992911855493)	2359 (0.034)	1233 (0.1)
intrusive	1	5110(0.011432491139819366)	2359 (0.034)	#SAKNAS!
intrusive	1	5110(0.011432491139819366)	2359 (0.034)	#SAKNAS!
intubation	1	2214(0.04572996455927746)	2359 (0.034)	1668 (0.066)
inuit	1	5110(0.011432491139819366)	2359 (0.034)	#SAKNAS!
inuit	1	5110(0.011432491139819366)	2359 (0.034)	#SAKNAS!
inulin	1	5110(0.011432491139819366)	#SAKNAS!	#SAKNAS!
invagination	1	5110(0.011432491139819366)	#SAKNAS!	2548 (0.033)
invasion	1	2214(0.04572996455927746)	2359 (0.034)	1668 (0.066)
inventory	1	5110(0.011432491139819366)	#SAKNAS!	#SAKNAS!
inverse	1	1678(0.0685949468389162)	2359 (0.034)	1668 (0.066)
inversion	1	5110(0.011432491139819366)	#SAKNAS!	#SAKNAS!
invertebrate	1	3437(0.02286498227963873)	2359 (0.034)	2548 (0.033)
invertebrates	1	5110(0.011432491139819366)	#SAKNAS!	#SAKNAS!
investigator-masked	1	5110(0.011432491139819366)	#SAKNAS!	2548 (0.033)
involuntary	1	5110(0.011432491139819366)	2359 (0.034)	#SAKNAS!
involuntary	1	5110(0.011432491139819366)	2359 (0.034)	#SAKNAS!
iodine	1	5110(0.011432491139819366)	#SAKNAS!	#SAKNAS!
iodontitis	1	5110(0.011432491139819366)	#SAKNAS!	#SAKNAS!
ion	1	5110(0.011432491139819366)	#SAKNAS!	2548 (0.033)

ionian	1	5110(0.011432491139819366)	#SAKNAS!	2548 (0.033)
ionophore	1	5110(0.011432491139819366)	#SAKNAS!	2548 (0.033)
iowa	1	5110(0.011432491139819366)	#SAKNAS!	#SAKNAS!
ipomoea	1	5110(0.011432491139819366)	2359 (0.034)	#SAKNAS!
ipomoea	1	5110(0.011432491139819366)	2359 (0.034)	#SAKNAS!
iq	1	5110(0.011432491139819366)	#SAKNAS!	2548 (0.033)
iraqi	1	5110(0.011432491139819366)	#SAKNAS!	#SAKNAS!
ireland	1	1485(0.08002743797873557)	2359 (0.034)	1233 (0.1)
irish	1	3437(0.02286498227963873)	2359 (0.034)	2548 (0.033)
iron-based	1	5110(0.011432491139819366)	#SAKNAS!	#SAKNAS!
iron-sucrose	1	5110(0.011432491139819366)	2359 (0.034)	#SAKNAS!
iron-sucrose	1	5110(0.011432491139819366)	2359 (0.034)	#SAKNAS!
irony	1	5110(0.011432491139819366)	#SAKNAS!	2548 (0.033)
irradians	1	5110(0.011432491139819366)	#SAKNAS!	2548 (0.033)
irregular	1	5110(0.011432491139819366)	2359 (0.034)	#SAKNAS!
irregular	1	5110(0.011432491139819366)	2359 (0.034)	#SAKNAS!
irrelevant	1	5110(0.011432491139819366)	#SAKNAS!	2548 (0.033)
irresectable	1	5110(0.011432491139819366)	2359 (0.034)	#SAKNAS!
irresectable	1	5110(0.011432491139819366)	2359 (0.034)	#SAKNAS!
irrespective	1	5110(0.011432491139819366)	#SAKNAS!	2548 (0.033)
ischemia	1	5110(0.011432491139819366)	2359 (0.034)	#SAKNAS!
ischemia	1	5110(0.011432491139819366)	2359 (0.034)	#SAKNAS!
isde	1	5110(0.011432491139819366)	#SAKNAS!	2548 (0.033)
isk	1	5110(0.011432491139819366)	#SAKNAS!	#SAKNAS!
islander	1	5110(0.011432491139819366)	#SAKNAS!	#SAKNAS!
isocaloric	1	5110(0.011432491139819366)	2359 (0.034)	#SAKNAS!
isocaloric	1	5110(0.011432491139819366)	2359 (0.034)	#SAKNAS!
isoenergetically	1	5110(0.011432491139819366)	2359 (0.034)	#SAKNAS!
isoenergetically	1	5110(0.011432491139819366)	2359 (0.034)	#SAKNAS!
isoflavone	1	5110(0.011432491139819366)	2359 (0.034)	#SAKNAS!
isoflavone	1	5110(0.011432491139819366)	2359 (0.034)	#SAKNAS!
isoforms	1	5110(0.011432491139819366)	2359 (0.034)	#SAKNAS!
isoforms	1	5110(0.011432491139819366)	2359 (0.034)	#SAKNAS!
isolate-based	1	5110(0.011432491139819366)	#SAKNAS!	#SAKNAS!
isometric	1	5110(0.011432491139819366)	#SAKNAS!	2548 (0.033)
isoperistaltic	1	5110(0.011432491139819366)	2359 (0.034)	#SAKNAS!
isoperistaltic	1	5110(0.011432491139819366)	2359 (0.034)	#SAKNAS!
isoptera	1	5110(0.011432491139819366)	2359 (0.034)	#SAKNAS!
isoptera	1	5110(0.011432491139819366)	2359 (0.034)	#SAKNAS!
ispghan	1	5110(0.011432491139819366)	#SAKNAS!	#SAKNAS!
israel	1	2214(0.04572996455927746)	2359 (0.034)	1668 (0.066)
israeli	1	3437(0.02286498227963873)	2359 (0.034)	#SAKNAS!
issuance	1	5110(0.011432491139819366)	2359 (0.034)	#SAKNAS!

issuance	1	5110(0.011432491139819366)	2359 (0.034)	#SAKNAS!
italian	1	710(0.19435234937692925)	2359 (0.034)	584 (0.23)
itsm	1	5110(0.011432491139819366)	#SAKNAS!	2548 (0.033)
iva	1	5110(0.011432491139819366)	#SAKNAS!	2548 (0.033)
ivb	1	3437(0.02286498227963873)	2359 (0.034)	2548 (0.033)
ivig	1	5110(0.011432491139819366)	#SAKNAS!	#SAKNAS!
ivor	1	5110(0.011432491139819366)	#SAKNAS!	2548 (0.033)
iwate-kenco	1	5110(0.011432491139819366)	#SAKNAS!	2548 (0.033)
ix	1	5110(0.011432491139819366)	2359 (0.034)	#SAKNAS!
ix	1	5110(0.011432491139819366)	2359 (0.034)	#SAKNAS!
iztapalapa	1	5110(0.011432491139819366)	#SAKNAS!	2548 (0.033)
jabaquara	1	5110(0.011432491139819366)	2359 (0.034)	#SAKNAS!
jabaquara	1	5110(0.011432491139819366)	2359 (0.034)	#SAKNAS!
jablonski-momeni	1	5110(0.011432491139819366)	2359 (0.034)	#SAKNAS!
jablonski-momeni	1	5110(0.011432491139819366)	2359 (0.034)	#SAKNAS!
jacchus	1	5110(0.011432491139819366)	#SAKNAS!	#SAKNAS!
jackal	1	5110(0.011432491139819366)	2359 (0.034)	#SAKNAS!
jackal	1	5110(0.011432491139819366)	2359 (0.034)	#SAKNAS!
jacobaea	1	5110(0.011432491139819366)	#SAKNAS!	#SAKNAS!
jakarta	1	5110(0.011432491139819366)	#SAKNAS!	2548 (0.033)
jalisco	1	5110(0.011432491139819366)	#SAKNAS!	2548 (0.033)
jaschnov	1	5110(0.011432491139819366)	2359 (0.034)	#SAKNAS!
jaschnov	1	5110(0.011432491139819366)	2359 (0.034)	#SAKNAS!
jaschnovia	1	5110(0.011432491139819366)	2359 (0.034)	#SAKNAS!
jaschnovia	1	5110(0.011432491139819366)	2359 (0.034)	#SAKNAS!
jasmonic	1	5110(0.011432491139819366)	#SAKNAS!	2548 (0.033)
java	1	2693(0.0342974734194581)	2359 (0.034)	2548 (0.033)
jaw	1	2214(0.04572996455927746)	2359 (0.034)	2548 (0.033)
jaw-opening	1	5110(0.011432491139819366)	#SAKNAS!	2548 (0.033)
jcog0706s1	1	5110(0.011432491139819366)	#SAKNAS!	2548 (0.033)
jdm	1	5110(0.011432491139819366)	#SAKNAS!	#SAKNAS!
jeddah	1	5110(0.011432491139819366)	#SAKNAS!	2548 (0.033)
jejunal	1	2214(0.04572996455927746)	2359 (0.034)	1668 (0.066)
jejunum	1	3437(0.02286498227963873)	2359 (0.034)	2548 (0.033)
jekelianus	1	5110(0.011432491139819366)	2359 (0.034)	#SAKNAS!
jekelianus	1	5110(0.011432491139819366)	2359 (0.034)	#SAKNAS!
jellyfish	1	5110(0.011432491139819366)	#SAKNAS!	2548 (0.033)
jelskii	1	5110(0.011432491139819366)	#SAKNAS!	2548 (0.033)
jesus	1	5110(0.011432491139819366)	#SAKNAS!	#SAKNAS!
jet	1	5110(0.011432491139819366)	#SAKNAS!	#SAKNAS!
jiangxi	1	5110(0.011432491139819366)	#SAKNAS!	2548 (0.033)
jinzhou	1	5110(0.011432491139819366)	#SAKNAS!	#SAKNAS!
jk2	1	5110(0.011432491139819366)	2359 (0.034)	#SAKNAS!

jk2	1	5110(0.011432491139819366)	2359 (0.034)	#SAKNAS!
job	1	5110(0.011432491139819366)	#SAKNAS!	#SAKNAS!
john	1	5110(0.011432491139819366)	#SAKNAS!	2548 (0.033)
johor	1	5110(0.011432491139819366)	#SAKNAS!	#SAKNAS!
jointly	1	5110(0.011432491139819366)	#SAKNAS!	2548 (0.033)
jomon	1	5110(0.011432491139819366)	2359 (0.034)	#SAKNAS!
jomon	1	5110(0.011432491139819366)	2359 (0.034)	#SAKNAS!
jordanian	1	5110(0.011432491139819366)	2359 (0.034)	#SAKNAS!
jordanian	1	5110(0.011432491139819366)	2359 (0.034)	#SAKNAS!
jouf	1	5110(0.011432491139819366)	#SAKNAS!	#SAKNAS!
journey	1	5110(0.011432491139819366)	#SAKNAS!	#SAKNAS!
j-shine	1	5110(0.011432491139819366)	#SAKNAS!	2548 (0.033)
jubail	1	5110(0.011432491139819366)	2359 (0.034)	#SAKNAS!
jubail	1	5110(0.011432491139819366)	2359 (0.034)	#SAKNAS!
jugular	1	5110(0.011432491139819366)	#SAKNAS!	2548 (0.033)
juice	1	2214(0.04572996455927746)	2359 (0.034)	1668 (0.066)
juices	1	3437(0.02286498227963873)	2359 (0.034)	#SAKNAS!
jump	1	5110(0.011432491139819366)	#SAKNAS!	2548 (0.033)
junior	1	5110(0.011432491139819366)	#SAKNAS!	2548 (0.033)
junk	1	3437(0.02286498227963873)	2359 (0.034)	2548 (0.033)
juveniles	1	2214(0.04572996455927746)	2359 (0.034)	1233 (0.1)
k12	1	5110(0.011432491139819366)	#SAKNAS!	#SAKNAS!
k2	1	5110(0.011432491139819366)	#SAKNAS!	2548 (0.033)
kabarole	1	5110(0.011432491139819366)	2359 (0.034)	#SAKNAS!
kabarole	1	5110(0.011432491139819366)	2359 (0.034)	#SAKNAS!
kabini	1	5110(0.011432491139819366)	#SAKNAS!	2548 (0.033)
kamakura	1	5110(0.011432491139819366)	#SAKNAS!	2548 (0.033)
kanapoi	1	5110(0.011432491139819366)	#SAKNAS!	2548 (0.033)
kangaroos	1	5110(0.011432491139819366)	#SAKNAS!	2548 (0.033)
kanglaite	1	5110(0.011432491139819366)	#SAKNAS!	#SAKNAS!
kappa	1	5110(0.011432491139819366)	#SAKNAS!	#SAKNAS!
karayaka	1	5110(0.011432491139819366)	2359 (0.034)	#SAKNAS!
karayaka	1	5110(0.011432491139819366)	2359 (0.034)	#SAKNAS!
karenina	1	5110(0.011432491139819366)	#SAKNAS!	#SAKNAS!
karnataka	1	3437(0.02286498227963873)	2359 (0.034)	#SAKNAS!
karst	1	5110(0.011432491139819366)	#SAKNAS!	2548 (0.033)
kashiwa	1	5110(0.011432491139819366)	#SAKNAS!	#SAKNAS!
kaveri	1	5110(0.011432491139819366)	#SAKNAS!	2548 (0.033)
kavrepalanchowk	1	5110(0.011432491139819366)	#SAKNAS!	2548 (0.033)
kctd15	1	5110(0.011432491139819366)	#SAKNAS!	2548 (0.033)
kda	1	5110(0.011432491139819366)	2359 (0.034)	#SAKNAS!
kda	1	5110(0.011432491139819366)	2359 (0.034)	#SAKNAS!
kelps	1	5110(0.011432491139819366)	#SAKNAS!	#SAKNAS!

kennedy	1	5110(0.011432491139819366)	#SAKNAS!	2548 (0.033)
keratinocyte	1	5110(0.011432491139819366)	#SAKNAS!	2548 (0.033)
keratinocytes	1	5110(0.011432491139819366)	#SAKNAS!	#SAKNAS!
kercadr	1	5110(0.011432491139819366)	2359 (0.034)	#SAKNAS!
kercadr	1	5110(0.011432491139819366)	2359 (0.034)	#SAKNAS!
kerguelen	1	5110(0.011432491139819366)	#SAKNAS!	#SAKNAS!
kerman	1	5110(0.011432491139819366)	2359 (0.034)	#SAKNAS!
kerman	1	5110(0.011432491139819366)	2359 (0.034)	#SAKNAS!
kernel	1	5110(0.011432491139819366)	#SAKNAS!	#SAKNAS!
ketogenic	1	5110(0.011432491139819366)	#SAKNAS!	#SAKNAS!
ketosis	1	5110(0.011432491139819366)	2359 (0.034)	#SAKNAS!
ketosis	1	5110(0.011432491139819366)	2359 (0.034)	#SAKNAS!
keystone	1	5110(0.011432491139819366)	2359 (0.034)	#SAKNAS!
keystone	1	5110(0.011432491139819366)	2359 (0.034)	#SAKNAS!
kg	1	5110(0.011432491139819366)	2359 (0.034)	#SAKNAS!
kg	1	5110(0.011432491139819366)	2359 (0.034)	#SAKNAS!
kgp	1	5110(0.011432491139819366)	2359 (0.034)	#SAKNAS!
kgp	1	5110(0.011432491139819366)	2359 (0.034)	#SAKNAS!
khartooran	1	5110(0.011432491139819366)	#SAKNAS!	2548 (0.033)
khunlook	1	5110(0.011432491139819366)	#SAKNAS!	2548 (0.033)
kibale	1	5110(0.011432491139819366)	2359 (0.034)	#SAKNAS!
kibale	1	5110(0.011432491139819366)	2359 (0.034)	#SAKNAS!
kid	1	3437(0.02286498227963873)	2359 (0.034)	2548 (0.033)
kids	1	2693(0.0342974734194581)	2359 (0.034)	#SAKNAS!
kihon	1	5110(0.011432491139819366)	#SAKNAS!	2548 (0.033)
killer	1	5110(0.011432491139819366)	#SAKNAS!	#SAKNAS!
kimberley	1	5110(0.011432491139819366)	#SAKNAS!	2548 (0.033)
kind	1	5110(0.011432491139819366)	#SAKNAS!	2548 (0.033)
kindergarten-based	1	5110(0.011432491139819366)	#SAKNAS!	2548 (0.033)
kinesio	1	5110(0.011432491139819366)	#SAKNAS!	#SAKNAS!
kinetics	1	5110(0.011432491139819366)	2359 (0.034)	#SAKNAS!
kinetics	1	5110(0.011432491139819366)	2359 (0.034)	#SAKNAS!
kinetoplastid	1	5110(0.011432491139819366)	2359 (0.034)	#SAKNAS!
kinetoplastid	1	5110(0.011432491139819366)	2359 (0.034)	#SAKNAS!
king	1	3437(0.02286498227963873)	2359 (0.034)	#SAKNAS!
kingdom	1	1335(0.09145992911855493)	2359 (0.034)	999 (0.13)
kinshasa	1	5110(0.011432491139819366)	2359 (0.034)	#SAKNAS!
kinshasa	1	5110(0.011432491139819366)	2359 (0.034)	#SAKNAS!
kisarawe	1	5110(0.011432491139819366)	#SAKNAS!	#SAKNAS!
kisspeptin	1	5110(0.011432491139819366)	#SAKNAS!	2548 (0.033)
kiwifruit	1	5110(0.011432491139819366)	#SAKNAS!	2548 (0.033)
klaus	1	5110(0.011432491139819366)	2359 (0.034)	#SAKNAS!
klaus	1	5110(0.011432491139819366)	2359 (0.034)	#SAKNAS!

kleptoplasts	1	5110(0.011432491139819366)	2359 (0.034)	#SAKNAS!
kleptoplasts	1	5110(0.011432491139819366)	2359 (0.034)	#SAKNAS!
kmc	1	5110(0.011432491139819366)	#SAKNAS!	#SAKNAS!
knanes	1	5110(0.011432491139819366)	#SAKNAS!	#SAKNAS!
knife	1	5110(0.011432491139819366)	#SAKNAS!	2548 (0.033)
knockdown	1	5110(0.011432491139819366)	#SAKNAS!	2548 (0.033)
knockout	1	5110(0.011432491139819366)	#SAKNAS!	#SAKNAS!
knowledge-a	1	5110(0.011432491139819366)	#SAKNAS!	#SAKNAS!
ko	1	5110(0.011432491139819366)	2359 (0.034)	#SAKNAS!
ko	1	5110(0.011432491139819366)	2359 (0.034)	#SAKNAS!
koala	1	5110(0.011432491139819366)	2359 (0.034)	#SAKNAS!
koala	1	5110(0.011432491139819366)	2359 (0.034)	#SAKNAS!
koalas	1	5110(0.011432491139819366)	#SAKNAS!	2548 (0.033)
koehler	1	5110(0.011432491139819366)	2359 (0.034)	#SAKNAS!
koehler	1	5110(0.011432491139819366)	2359 (0.034)	#SAKNAS!
koji	1	5110(0.011432491139819366)	#SAKNAS!	#SAKNAS!
konig	1	5110(0.011432491139819366)	#SAKNAS!	2548 (0.033)
konik	1	5110(0.011432491139819366)	#SAKNAS!	#SAKNAS!
koranic	1	5110(0.011432491139819366)	2359 (0.034)	#SAKNAS!
koranic	1	5110(0.011432491139819366)	2359 (0.034)	#SAKNAS!
krill	1	2693(0.0342974734194581)	2359 (0.034)	1668 (0.066)
kruger-national-park	1	5110(0.011432491139819366)	2359 (0.034)	#SAKNAS!
kruger-national-park	1	5110(0.011432491139819366)	2359 (0.034)	#SAKNAS!
kryptolebias	1	5110(0.011432491139819366)	#SAKNAS!	2548 (0.033)
kuala	1	5110(0.011432491139819366)	#SAKNAS!	2548 (0.033)
kudzu	1	5110(0.011432491139819366)	#SAKNAS!	2548 (0.033)
kumbh	1	5110(0.011432491139819366)	#SAKNAS!	2548 (0.033)
kunstfutter	1	5110(0.011432491139819366)	2359 (0.034)	#SAKNAS!
kunstfutter	1	5110(0.011432491139819366)	2359 (0.034)	#SAKNAS!
kuwait	1	5110(0.011432491139819366)	#SAKNAS!	2548 (0.033)
kuwaiti	1	5110(0.011432491139819366)	#SAKNAS!	#SAKNAS!
kwazulu	1	5110(0.011432491139819366)	2359 (0.034)	#SAKNAS!
kwazulu	1	5110(0.011432491139819366)	2359 (0.034)	#SAKNAS!
kwazulu-natal	1	3437(0.02286498227963873)	2359 (0.034)	2548 (0.033)
label	1	5110(0.011432491139819366)	#SAKNAS!	2548 (0.033)
label-free	1	5110(0.011432491139819366)	#SAKNAS!	#SAKNAS!
labeo	1	5110(0.011432491139819366)	2359 (0.034)	#SAKNAS!
labeo	1	5110(0.011432491139819366)	2359 (0.034)	#SAKNAS!
laborers	1	5110(0.011432491139819366)	2359 (0.034)	#SAKNAS!
laborers	1	5110(0.011432491139819366)	2359 (0.034)	#SAKNAS!
labrid	1	5110(0.011432491139819366)	2359 (0.034)	#SAKNAS!
labrid	1	5110(0.011432491139819366)	2359 (0.034)	#SAKNAS!
lacanobia	1	5110(0.011432491139819366)	2359 (0.034)	#SAKNAS!

lacanobia	1	5110(0.011432491139819366)	2359 (0.034)	#SAKNAS!
laceration	1	5110(0.011432491139819366)	2359 (0.034)	#SAKNAS!
laceration	1	5110(0.011432491139819366)	2359 (0.034)	#SAKNAS!
lachesis	1	5110(0.011432491139819366)	#SAKNAS!	#SAKNAS!
lactate	1	5110(0.011432491139819366)	#SAKNAS!	2548 (0.033)
lactational	1	5110(0.011432491139819366)	2359 (0.034)	#SAKNAS!
lactational	1	5110(0.011432491139819366)	2359 (0.034)	#SAKNAS!
lactic	1	5110(0.011432491139819366)	#SAKNAS!	2548 (0.033)
lactobacillus	1	1219(0.1028924202583743)	2359 (0.034)	999 (0.13)
lactoferrin-luteolin	1	5110(0.011432491139819366)	#SAKNAS!	#SAKNAS!
ladakh	1	5110(0.011432491139819366)	#SAKNAS!	#SAKNAS!
laevigata	1	5110(0.011432491139819366)	#SAKNAS!	2548 (0.033)
laevis	1	3437(0.02286498227963873)	2359 (0.034)	#SAKNAS!
lagothricha	1	5110(0.011432491139819366)	#SAKNAS!	2548 (0.033)
lagothrix	1	5110(0.011432491139819366)	#SAKNAS!	2548 (0.033)
laizhou	1	5110(0.011432491139819366)	#SAKNAS!	2548 (0.033)
lakes	1	3437(0.02286498227963873)	2359 (0.034)	2548 (0.033)
lam	1	3437(0.02286498227963873)	2359 (0.034)	2548 (0.033)
lama	1	5110(0.011432491139819366)	2359 (0.034)	#SAKNAS!
lama	1	5110(0.011432491139819366)	2359 (0.034)	#SAKNAS!
lamb	1	5110(0.011432491139819366)	#SAKNAS!	#SAKNAS!
lambs	1	1219(0.1028924202583743)	2359 (0.034)	694 (0.2)
laminoplasty	1	5110(0.011432491139819366)	#SAKNAS!	#SAKNAS!
landscape	1	2214(0.04572996455927746)	2359 (0.034)	2548 (0.033)
landscapes	1	5110(0.011432491139819366)	#SAKNAS!	2548 (0.033)
landscape-scale	1	5110(0.011432491139819366)	2359 (0.034)	#SAKNAS!
landscape-scale	1	5110(0.011432491139819366)	2359 (0.034)	#SAKNAS!
lange	1	5110(0.011432491139819366)	#SAKNAS!	#SAKNAS!
langur	1	5110(0.011432491139819366)	#SAKNAS!	#SAKNAS!
langurs	1	5110(0.011432491139819366)	#SAKNAS!	2548 (0.033)
lanka	1	2693(0.0342974734194581)	2359 (0.034)	1668 (0.066)
lankan	1	5110(0.011432491139819366)	2359 (0.034)	#SAKNAS!
lankan	1	5110(0.011432491139819366)	2359 (0.034)	#SAKNAS!
lantana	1	5110(0.011432491139819366)	#SAKNAS!	#SAKNAS!
laos	1	5110(0.011432491139819366)	#SAKNAS!	#SAKNAS!
laparoendoscopic	1	5110(0.011432491139819366)	2359 (0.034)	#SAKNAS!
laparoendoscopic	1	5110(0.011432491139819366)	2359 (0.034)	#SAKNAS!
laparo-endoscopic	1	5110(0.011432491139819366)	#SAKNAS!	2548 (0.033)
laparogastroscopy-a	1	5110(0.011432491139819366)	#SAKNAS!	#SAKNAS!
large-amplitude	1	5110(0.011432491139819366)	#SAKNAS!	#SAKNAS!
l-arginine	1	3437(0.02286498227963873)	2359 (0.034)	2548 (0.033)
larvatus	1	5110(0.011432491139819366)	#SAKNAS!	2548 (0.033)
larviculture	1	5110(0.011432491139819366)	#SAKNAS!	#SAKNAS!

laryngomalacia	1 5110(0.011432491139819366)	#SAKNAS!	2548 (0.033)
laryngopharyngectomy	1 5110(0.011432491139819366)	#SAKNAS!	2548 (0.033)
laryngoscopy-based	1 5110(0.011432491139819366)	#SAKNAS!	#SAKNAS!
larynx	1 1678(0.0685949468389162)	2359 (0.034)	1233 (0.1)
lasbela	1 5110(0.011432491139819366)	#SAKNAS!	2548 (0.033)
laser-assisted	1 5110(0.011432491139819366)	#SAKNAS!	2548 (0.033)
lasius	1 5110(0.011432491139819366)	#SAKNAS!	2548 (0.033)
lata	1 5110(0.011432491139819366)	#SAKNAS!	#SAKNAS!
latch	1 5110(0.011432491139819366)	#SAKNAS!	2548 (0.033)
latency	1 5110(0.011432491139819366)	#SAKNAS!	2548 (0.033)
later	1 2214(0.04572996455927746)	2359 (0.034)	1233 (0.1)
laterality	1 5110(0.011432491139819366)	2359 (0.034)	#SAKNAS!
laterality	1 5110(0.011432491139819366)	2359 (0.034)	#SAKNAS!
latest	1 5110(0.011432491139819366)	#SAKNAS!	#SAKNAS!
late-stage	1 5110(0.011432491139819366)	#SAKNAS!	#SAKNAS!
latex	1 5110(0.011432491139819366)	2359 (0.034)	#SAKNAS!
latex	1 5110(0.011432491139819366)	2359 (0.034)	#SAKNAS!
latin	1 5110(0.011432491139819366)	#SAKNAS!	#SAKNAS!
latino	1 2214(0.04572996455927746)	2359 (0.034)	1233 (0.1)
laxative	1 5110(0.011432491139819366)	2359 (0.034)	#SAKNAS!
laxative	1 5110(0.011432491139819366)	2359 (0.034)	#SAKNAS!
layer	1 5110(0.011432491139819366)	2359 (0.034)	#SAKNAS!
layer	1 5110(0.011432491139819366)	2359 (0.034)	#SAKNAS!
layers	1 5110(0.011432491139819366)	2359 (0.034)	#SAKNAS!
layers	1 5110(0.011432491139819366)	2359 (0.034)	#SAKNAS!
laying	1 1485(0.08002743797873557)	2359 (0.034)	1233 (0.1)
l-carnitine	1 5110(0.011432491139819366)	#SAKNAS!	#SAKNAS!
lcdc	1 5110(0.011432491139819366)	#SAKNAS!	2548 (0.033)
l-cell	1 5110(0.011432491139819366)	#SAKNAS!	2548 (0.033)
l-citrulline-fed	1 5110(0.011432491139819366)	#SAKNAS!	#SAKNAS!
leached	1 5110(0.011432491139819366)	#SAKNAS!	#SAKNAS!
leads	1 1894(0.05716245569909683)	2359 (0.034)	1668 (0.066)
leaf-beetle	1 5110(0.011432491139819366)	2359 (0.034)	#SAKNAS!
leaf-beetle	1 5110(0.011432491139819366)	2359 (0.034)	#SAKNAS!
leaf-eared	1 5110(0.011432491139819366)	2359 (0.034)	#SAKNAS!
leaf-eared	1 5110(0.011432491139819366)	2359 (0.034)	#SAKNAS!
leafhopper	1 5110(0.011432491139819366)	2359 (0.034)	#SAKNAS!
leafhopper	1 5110(0.011432491139819366)	2359 (0.034)	#SAKNAS!
leaflets	1 3437(0.02286498227963873)	2359 (0.034)	2548 (0.033)
leaf-litter	1 5110(0.011432491139819366)	#SAKNAS!	2548 (0.033)
leafminer	1 5110(0.011432491139819366)	2359 (0.034)	#SAKNAS!
leafminer	1 5110(0.011432491139819366)	2359 (0.034)	#SAKNAS!
leafy	1 5110(0.011432491139819366)	2359 (0.034)	#SAKNAS!

leafy	1	5110(0.011432491139819366)	2359 (0.034)	#SAKNAS!
leak	1	5110(0.011432491139819366)	2359 (0.034)	#SAKNAS!
leak	1	5110(0.011432491139819366)	2359 (0.034)	#SAKNAS!
leakage	1	5110(0.011432491139819366)	#SAKNAS!	2548 (0.033)
leaks	1	3437(0.02286498227963873)	2359 (0.034)	#SAKNAS!
leap2	1	5110(0.011432491139819366)	#SAKNAS!	#SAKNAS!
least	1	3437(0.02286498227963873)	2359 (0.034)	2548 (0.033)
lebanese	1	5110(0.011432491139819366)	#SAKNAS!	2548 (0.033)
lectures	1	5110(0.011432491139819366)	#SAKNAS!	2548 (0.033)
lee	1	5110(0.011432491139819366)	#SAKNAS!	#SAKNAS!
left-behind	1	5110(0.011432491139819366)	#SAKNAS!	2548 (0.033)
leg	1	5110(0.011432491139819366)	#SAKNAS!	2548 (0.033)
legislation	1	5110(0.011432491139819366)	#SAKNAS!	2548 (0.033)
legumes	1	5110(0.011432491139819366)	#SAKNAS!	2548 (0.033)
leishmaniasis	1	5110(0.011432491139819366)	2359 (0.034)	#SAKNAS!
leishmaniasis	1	5110(0.011432491139819366)	2359 (0.034)	#SAKNAS!
leisure	1	5110(0.011432491139819366)	#SAKNAS!	2548 (0.033)
lemonade	1	5110(0.011432491139819366)	2359 (0.034)	#SAKNAS!
lemonade	1	5110(0.011432491139819366)	2359 (0.034)	#SAKNAS!
lengthens	1	5110(0.011432491139819366)	#SAKNAS!	#SAKNAS!
length-weight	1	5110(0.011432491139819366)	#SAKNAS!	2548 (0.033)
leniusculus	1	5110(0.011432491139819366)	2359 (0.034)	#SAKNAS!
leniusculus	1	5110(0.011432491139819366)	2359 (0.034)	#SAKNAS!
lennox-gastaut	1	5110(0.011432491139819366)	#SAKNAS!	#SAKNAS!
lens	1	2693(0.0342974734194581)	2359 (0.034)	#SAKNAS!
lep	1	5110(0.011432491139819366)	#SAKNAS!	2548 (0.033)
lepeophtheirus	1	5110(0.011432491139819366)	#SAKNAS!	2548 (0.033)
lepidopteran	1	5110(0.011432491139819366)	2359 (0.034)	#SAKNAS!
lepidopteran	1	5110(0.011432491139819366)	2359 (0.034)	#SAKNAS!
lepidorhombus	1	5110(0.011432491139819366)	2359 (0.034)	#SAKNAS!
lepidorhombus	1	5110(0.011432491139819366)	2359 (0.034)	#SAKNAS!
lepilemur	1	5110(0.011432491139819366)	2359 (0.034)	#SAKNAS!
lepilemur	1	5110(0.011432491139819366)	2359 (0.034)	#SAKNAS!
leporinus	1	5110(0.011432491139819366)	2359 (0.034)	#SAKNAS!
leporinus	1	5110(0.011432491139819366)	2359 (0.034)	#SAKNAS!
lepr-rs1137100	1	5110(0.011432491139819366)	#SAKNAS!	2548 (0.033)
leptin-deficient	1	5110(0.011432491139819366)	#SAKNAS!	2548 (0.033)
lerp-forming	1	5110(0.011432491139819366)	#SAKNAS!	2548 (0.033)
lesion	1	1485(0.08002743797873557)	2359 (0.034)	999 (0.13)
lessepsian	1	5110(0.011432491139819366)	#SAKNAS!	#SAKNAS!
lesser	1	5110(0.011432491139819366)	#SAKNAS!	2548 (0.033)
lesser-known	1	5110(0.011432491139819366)	#SAKNAS!	#SAKNAS!
lesson	1	5110(0.011432491139819366)	#SAKNAS!	#SAKNAS!

lethocerus	1	5110(0.011432491139819366)	#SAKNAS!	#SAKNAS!
leucaena	1	5110(0.011432491139819366)	#SAKNAS!	#SAKNAS!
leucocytes	1	5110(0.011432491139819366)	2359 (0.034)	#SAKNAS!
leucocytes	1	5110(0.011432491139819366)	2359 (0.034)	#SAKNAS!
leucomelas	1	5110(0.011432491139819366)	#SAKNAS!	2548 (0.033)
leuconostoc	1	5110(0.011432491139819366)	#SAKNAS!	#SAKNAS!
leukemia	1	5110(0.011432491139819366)	#SAKNAS!	#SAKNAS!
leukocytes	1	5110(0.011432491139819366)	#SAKNAS!	2548 (0.033)
leukocytosis	1	5110(0.011432491139819366)	#SAKNAS!	2548 (0.033)
leukoencephalomalacia	1	5110(0.011432491139819366)	#SAKNAS!	2548 (0.033)
leukopenia	1	5110(0.011432491139819366)	2359 (0.034)	#SAKNAS!
leukopenia	1	5110(0.011432491139819366)	2359 (0.034)	#SAKNAS!
leukoplakia	1	5110(0.011432491139819366)	2359 (0.034)	#SAKNAS!
leukoplakia	1	5110(0.011432491139819366)	2359 (0.034)	#SAKNAS!
leukotriene	1	5110(0.011432491139819366)	2359 (0.034)	#SAKNAS!
leukotriene	1	5110(0.011432491139819366)	2359 (0.034)	#SAKNAS!
levi	1	5110(0.011432491139819366)	2359 (0.034)	#SAKNAS!
levi	1	5110(0.011432491139819366)	2359 (0.034)	#SAKNAS!
levodopa	1	5110(0.011432491139819366)	#SAKNAS!	2548 (0.033)
levy	1	5110(0.011432491139819366)	#SAKNAS!	#SAKNAS!
lewis	1	5110(0.011432491139819366)	#SAKNAS!	2548 (0.033)
lewisii	1	5110(0.011432491139819366)	2359 (0.034)	#SAKNAS!
lewisii	1	5110(0.011432491139819366)	2359 (0.034)	#SAKNAS!
l-glu	1	5110(0.011432491139819366)	#SAKNAS!	#SAKNAS!
liberation	1	5110(0.011432491139819366)	#SAKNAS!	#SAKNAS!
libyan	1	3437(0.02286498227963873)	2359 (0.034)	#SAKNAS!
lice	1	5110(0.011432491139819366)	#SAKNAS!	2548 (0.033)
lichen	1	2214(0.04572996455927746)	2359 (0.034)	1668 (0.066)
licuri	1	5110(0.011432491139819366)	#SAKNAS!	#SAKNAS!
lid	1	5110(0.011432491139819366)	2359 (0.034)	#SAKNAS!
lid	1	5110(0.011432491139819366)	2359 (0.034)	#SAKNAS!
lidocaine	1	5110(0.011432491139819366)	#SAKNAS!	#SAKNAS!
life-conditions	1	5110(0.011432491139819366)	#SAKNAS!	#SAKNAS!
lifecourse	1	5110(0.011432491139819366)	#SAKNAS!	2548 (0.033)
life-history	1	3437(0.02286498227963873)	2359 (0.034)	2548 (0.033)
lifelong	1	5110(0.011432491139819366)	#SAKNAS!	2548 (0.033)
lift	1	5110(0.011432491139819366)	#SAKNAS!	2548 (0.033)
lifting	1	3437(0.02286498227963873)	2359 (0.034)	2548 (0.033)
ligament	1	2693(0.0342974734194581)	2359 (0.034)	#SAKNAS!
ligand	1	5110(0.011432491139819366)	#SAKNAS!	2548 (0.033)
ligation	1	5110(0.011432491139819366)	2359 (0.034)	#SAKNAS!
ligation	1	5110(0.011432491139819366)	2359 (0.034)	#SAKNAS!
ligature-induced	1	5110(0.011432491139819366)	#SAKNAS!	#SAKNAS!

light	1	1894(0.0571624556990968З)	2359 (0.034)	2548 (0.033)
lighting	1	5110(0.011432491139819366)	2359 (0.034)	#SAKNAS!
lighting	1	5110(0.011432491139819366)	2359 (0.034)	#SAKNAS!
lignosulfonate	1	5110(0.011432491139819366)	#SAKNAS!	2548 (0.033)
like	1	2693(0.0342974734194581)	2359 (0.034)	1668 (0.066)
limit	1	5110(0.011432491139819366)	#SAKNAS!	#SAKNAS!
limitation	1	1485(0.08002743797873557)	2359 (0.034)	999 (0.13)
limiting	1	3437(0.02286498227963873)	2359 (0.034)	2548 (0.033)
limits	1	3437(0.02286498227963873)	2359 (0.034)	2548 (0.033)
limnephilidae	1	3437(0.02286498227963873)	2359 (0.034)	#SAKNAS!
limonoid	1	5110(0.011432491139819366)	2359 (0.034)	#SAKNAS!
limonoid	1	5110(0.011432491139819366)	2359 (0.034)	#SAKNAS!
limpets	1	5110(0.011432491139819366)	#SAKNAS!	2548 (0.033)
line	1	3437(0.02286498227963873)	2359 (0.034)	#SAKNAS!
lineage	1	5110(0.011432491139819366)	2359 (0.034)	#SAKNAS!
lineage	1	5110(0.011432491139819366)	2359 (0.034)	#SAKNAS!
linear	1	2693(0.0342974734194581)	2359 (0.034)	1668 (0.066)
lineatus	1	5110(0.011432491139819366)	#SAKNAS!	#SAKNAS!
lineolaris	1	5110(0.011432491139819366)	#SAKNAS!	#SAKNAS!
lingonberry	1	5110(0.011432491139819366)	#SAKNAS!	#SAKNAS!
linguistic-cultural	1	5110(0.011432491139819366)	#SAKNAS!	2548 (0.033)
lining	1	5110(0.011432491139819366)	2359 (0.034)	#SAKNAS!
lining	1	5110(0.011432491139819366)	2359 (0.034)	#SAKNAS!
linkage	1	3437(0.02286498227963873)	2359 (0.034)	#SAKNAS!
linking	1	2214(0.04572996455927746)	2359 (0.034)	2548 (0.033)
linoleic	1	5110(0.011432491139819366)	#SAKNAS!	#SAKNAS!
lint	1	5110(0.011432491139819366)	#SAKNAS!	#SAKNAS!
linx	1	5110(0.011432491139819366)	#SAKNAS!	#SAKNAS!
linx篢	1	5110(0.011432491139819366)	#SAKNAS!	2548 (0.033)
lion-tailed	1	5110(0.011432491139819366)	2359 (0.034)	#SAKNAS!
lion-tailed	1	5110(0.011432491139819366)	2359 (0.034)	#SAKNAS!
lipase	1	5110(0.011432491139819366)	#SAKNAS!	2548 (0.033)
lipid-lowering	1	5110(0.011432491139819366)	#SAKNAS!	#SAKNAS!
lipids	1	2214(0.04572996455927746)	2359 (0.034)	1233 (0.1)
lipin	1	5110(0.011432491139819366)	#SAKNAS!	2548 (0.033)
lipocalin	1	5110(0.011432491139819366)	#SAKNAS!	#SAKNAS!
lipopolysaccharide	1	5110(0.011432491139819366)	#SAKNAS!	2548 (0.033)
lipoxin	1	5110(0.011432491139819366)	#SAKNAS!	2548 (0.033)
liquid-loaded	1	5110(0.011432491139819366)	#SAKNAS!	#SAKNAS!
lisaplus	1	5110(0.011432491139819366)	2359 (0.034)	#SAKNAS!
lisaplus	1	5110(0.011432491139819366)	2359 (0.034)	#SAKNAS!
lithodidae	1	5110(0.011432491139819366)	2359 (0.034)	#SAKNAS!
lithodidae	1	5110(0.011432491139819366)	2359 (0.034)	#SAKNAS!

lithuania	1	5110(0.011432491139819366)	2359 (0.034)	#SAKNAS!
lithuania	1	5110(0.011432491139819366)	2359 (0.034)	#SAKNAS!
litopenaeus	1	2693(0.0342974734194581)	2359 (0.034)	1668 (0.066)
littoralis	1	5110(0.011432491139819366)	#SAKNAS!	#SAKNAS!
littorea	1	5110(0.011432491139819366)	2359 (0.034)	#SAKNAS!
littorea	1	5110(0.011432491139819366)	2359 (0.034)	#SAKNAS!
littorina	1	5110(0.011432491139819366)	2359 (0.034)	#SAKNAS!
littorina	1	5110(0.011432491139819366)	2359 (0.034)	#SAKNAS!
live	1	1219(0.1028924202583743)	2359 (0.034)	1233 (0.1)
livelihood	1	5110(0.011432491139819366)	#SAKNAS!	#SAKNAS!
livelihoods	1	5110(0.011432491139819366)	#SAKNAS!	2548 (0.033)
live-piloting	1	5110(0.011432491139819366)	#SAKNAS!	2548 (0.033)
lives	1	5110(0.011432491139819366)	#SAKNAS!	#SAKNAS!
livestock	1	5110(0.011432491139819366)	#SAKNAS!	#SAKNAS!
lividus	1	5110(0.011432491139819366)	#SAKNAS!	#SAKNAS!
liviidae	1	5110(0.011432491139819366)	#SAKNAS!	2548 (0.033)
livingstone	1	5110(0.011432491139819366)	2359 (0.034)	#SAKNAS!
livingstone	1	5110(0.011432491139819366)	2359 (0.034)	#SAKNAS!
livingstonii	1	5110(0.011432491139819366)	2359 (0.034)	#SAKNAS!
livingstonii	1	5110(0.011432491139819366)	2359 (0.034)	#SAKNAS!
lizards	1	5110(0.011432491139819366)	2359 (0.034)	#SAKNAS!
lizards	1	5110(0.011432491139819366)	2359 (0.034)	#SAKNAS!
loading	1	5110(0.011432491139819366)	#SAKNAS!	#SAKNAS!
loads	1	5110(0.011432491139819366)	#SAKNAS!	#SAKNAS!
lobectomy	1	5110(0.011432491139819366)	2359 (0.034)	#SAKNAS!
lobectomy	1	5110(0.011432491139819366)	2359 (0.034)	#SAKNAS!
lobster	1	1678(0.0685949468389162)	2359 (0.034)	1233 (0.1)
localization	1	5110(0.011432491139819366)	#SAKNAS!	#SAKNAS!
locally-bred	1	5110(0.011432491139819366)	2359 (0.034)	#SAKNAS!
locally-bred	1	5110(0.011432491139819366)	2359 (0.034)	#SAKNAS!
locations	1	2693(0.0342974734194581)	2359 (0.034)	2548 (0.033)
lochs	1	5110(0.011432491139819366)	2359 (0.034)	#SAKNAS!
lochs	1	5110(0.011432491139819366)	2359 (0.034)	#SAKNAS!
locomotive	1	5110(0.011432491139819366)	#SAKNAS!	#SAKNAS!
locoregional	1	5110(0.011432491139819366)	#SAKNAS!	2548 (0.033)
locoregionally	1	5110(0.011432491139819366)	#SAKNAS!	2548 (0.033)
locus	1	3437(0.02286498227963873)	2359 (0.034)	#SAKNAS!
locusta	1	5110(0.011432491139819366)	2359 (0.034)	#SAKNAS!
locusta	1	5110(0.011432491139819366)	2359 (0.034)	#SAKNAS!
locusta-migratoria	1	5110(0.011432491139819366)	2359 (0.034)	#SAKNAS!
locusta-migratoria	1	5110(0.011432491139819366)	2359 (0.034)	#SAKNAS!
locusts	1	5110(0.011432491139819366)	2359 (0.034)	#SAKNAS!
locusts	1	5110(0.011432491139819366)	2359 (0.034)	#SAKNAS!

lodgepole	1	5110(0.011432491139819366)	2359 (0.034)	#SAKNAS!
lodgepole	1	5110(0.011432491139819366)	2359 (0.034)	#SAKNAS!
log	1	5110(0.011432491139819366)	#SAKNAS!	#SAKNAS!
loggers	1	5110(0.011432491139819366)	2359 (0.034)	#SAKNAS!
loggers	1	5110(0.011432491139819366)	2359 (0.034)	#SAKNAS!
logistic	1	3437(0.02286498227963873)	2359 (0.034)	2548 (0.033)
logistics	1	5110(0.011432491139819366)	2359 (0.034)	#SAKNAS!
logistics	1	5110(0.011432491139819366)	2359 (0.034)	#SAKNAS!
logopedic	1	5110(0.011432491139819366)	2359 (0.034)	#SAKNAS!
logopedic	1	5110(0.011432491139819366)	2359 (0.034)	#SAKNAS!
logoplex	1	5110(0.011432491139819366)	#SAKNAS!	#SAKNAS!
loire	1	5110(0.011432491139819366)	#SAKNAS!	2548 (0.033)
lolium	1	5110(0.011432491139819366)	#SAKNAS!	2548 (0.033)
lomako	1	5110(0.011432491139819366)	2359 (0.034)	#SAKNAS!
lomako	1	5110(0.011432491139819366)	2359 (0.034)	#SAKNAS!
lombardy	1	5110(0.011432491139819366)	#SAKNAS!	#SAKNAS!
london	1	5110(0.011432491139819366)	#SAKNAS!	2548 (0.033)
longitudinally	1	5110(0.011432491139819366)	2359 (0.034)	#SAKNAS!
longitudinally	1	5110(0.011432491139819366)	2359 (0.034)	#SAKNAS!
long-lasting	1	5110(0.011432491139819366)	#SAKNAS!	2548 (0.033)
longurio	1	5110(0.011432491139819366)	#SAKNAS!	2548 (0.033)
looking	1	5110(0.011432491139819366)	#SAKNAS!	#SAKNAS!
loop	1	5110(0.011432491139819366)	2359 (0.034)	#SAKNAS!
loop	1	5110(0.011432491139819366)	2359 (0.034)	#SAKNAS!
loop-gastrostomy	1	5110(0.011432491139819366)	2359 (0.034)	#SAKNAS!
loop-gastrostomy	1	5110(0.011432491139819366)	2359 (0.034)	#SAKNAS!
loose	1	5110(0.011432491139819366)	2359 (0.034)	#SAKNAS!
loose	1	5110(0.011432491139819366)	2359 (0.034)	#SAKNAS!
lophiosilurus	1	5110(0.011432491139819366)	#SAKNAS!	2548 (0.033)
lopholithodes	1	5110(0.011432491139819366)	#SAKNAS!	#SAKNAS!
los	1	5110(0.011432491139819366)	#SAKNAS!	2548 (0.033)
losing	1	5110(0.011432491139819366)	#SAKNAS!	#SAKNAS!
lovastatin	1	5110(0.011432491139819366)	2359 (0.034)	#SAKNAS!
lovastatin	1	5110(0.011432491139819366)	2359 (0.034)	#SAKNAS!
low-birth-weight	1	5110(0.011432491139819366)	2359 (0.034)	#SAKNAS!
low-birth-weight	1	5110(0.011432491139819366)	2359 (0.034)	#SAKNAS!
low-cost	1	3437(0.02286498227963873)	2359 (0.034)	2548 (0.033)
low-density	1	3437(0.02286498227963873)	2359 (0.034)	#SAKNAS!
low-dose	1	5110(0.011432491139819366)	#SAKNAS!	2548 (0.033)
lower-income	1	5110(0.011432491139819366)	#SAKNAS!	#SAKNAS!
low-fat	1	5110(0.011432491139819366)	#SAKNAS!	2548 (0.033)
low-fluoride	1	5110(0.011432491139819366)	2359 (0.034)	#SAKNAS!
low-fluoride	1	5110(0.011432491139819366)	2359 (0.034)	#SAKNAS!

low-grade	1	5110(0.011432491139819366)	#SAKNAS!	2548 (0.033)
lowland	1	3437(0.02286498227963873)	2359 (0.034)	2548 (0.033)
low-nutrient-dense	1	5110(0.011432491139819366)	2359 (0.034)	#SAKNAS!
low-nutrient-dense	1	5110(0.011432491139819366)	2359 (0.034)	#SAKNAS!
low-pressure	1	5110(0.011432491139819366)	2359 (0.034)	#SAKNAS!
low-pressure	1	5110(0.011432491139819366)	2359 (0.034)	#SAKNAS!
low-prevalence	1	5110(0.011432491139819366)	2359 (0.034)	#SAKNAS!
low-prevalence	1	5110(0.011432491139819366)	2359 (0.034)	#SAKNAS!
low-protein	1	5110(0.011432491139819366)	2359 (0.034)	#SAKNAS!
low-protein	1	5110(0.011432491139819366)	2359 (0.034)	#SAKNAS!
low-quality	1	5110(0.011432491139819366)	#SAKNAS!	#SAKNAS!
low-risk	1	2693(0.0342974734194581)	2359 (0.034)	1668 (0.066)
low-sodium	1	5110(0.011432491139819366)	#SAKNAS!	#SAKNAS!
low-weight	1	5110(0.011432491139819366)	#SAKNAS!	#SAKNAS!
low-yield	1	5110(0.011432491139819366)	#SAKNAS!	#SAKNAS!
lozenges	1	5110(0.011432491139819366)	#SAKNAS!	2548 (0.033)
lp	1	5110(0.011432491139819366)	2359 (0.034)	#SAKNAS!
lp	1	5110(0.011432491139819366)	2359 (0.034)	#SAKNAS!
lpxa4	1	5110(0.011432491139819366)	#SAKNAS!	#SAKNAS!
lpz	1	5110(0.011432491139819366)	#SAKNAS!	2548 (0.033)
lrgab-mediated	1	5110(0.011432491139819366)	#SAKNAS!	2548 (0.033)
lrygb	1	5110(0.011432491139819366)	#SAKNAS!	2548 (0.033)
ls-1	1	5110(0.011432491139819366)	#SAKNAS!	2548 (0.033)
l-tryptophan	1	5110(0.011432491139819366)	#SAKNAS!	#SAKNAS!
lubricant	1	5110(0.011432491139819366)	#SAKNAS!	#SAKNAS!
lubricating	1	5110(0.011432491139819366)	#SAKNAS!	#SAKNAS!
lucerne	1	5110(0.011432491139819366)	2359 (0.034)	#SAKNAS!
lucerne	1	5110(0.011432491139819366)	2359 (0.034)	#SAKNAS!
lucioperca	1	5110(0.011432491139819366)	2359 (0.034)	#SAKNAS!
lucioperca	1	5110(0.011432491139819366)	2359 (0.034)	#SAKNAS!
lucius	1	5110(0.011432491139819366)	#SAKNAS!	2548 (0.033)
lucy	1	5110(0.011432491139819366)	#SAKNAS!	#SAKNAS!
lugar	1	5110(0.011432491139819366)	#SAKNAS!	#SAKNAS!
lumbar	1	5110(0.011432491139819366)	#SAKNAS!	#SAKNAS!
lumen	1	5110(0.011432491139819366)	2359 (0.034)	#SAKNAS!
lumen	1	5110(0.011432491139819366)	2359 (0.034)	#SAKNAS!
lunch	1	1678(0.0685949468389162)	2359 (0.034)	1233 (0.1)
lunchboxes	1	5110(0.011432491139819366)	#SAKNAS!	#SAKNAS!
lunches	1	5110(0.011432491139819366)	#SAKNAS!	2548 (0.033)
lunch-lung	1	5110(0.011432491139819366)	#SAKNAS!	#SAKNAS!
lupin	1	5110(0.011432491139819366)	2359 (0.034)	#SAKNAS!
lupin	1	5110(0.011432491139819366)	2359 (0.034)	#SAKNAS!
luteinizing	1	5110(0.011432491139819366)	2359 (0.034)	#SAKNAS!

luteinizing	1	5110(0.011432491139819366)	2359 (0.034)	#SAKNAS!
lutris	1	5110(0.011432491139819366)	2359 (0.034)	#SAKNAS!
lutris	1	5110(0.011432491139819366)	2359 (0.034)	#SAKNAS!
lutungs	1	5110(0.011432491139819366)	#SAKNAS!	#SAKNAS!
lvbh107	1	5110(0.011432491139819366)	#SAKNAS!	#SAKNAS!
lycalopex	1	5110(0.011432491139819366)	2359 (0.034)	#SAKNAS!
lycalopex	1	5110(0.011432491139819366)	2359 (0.034)	#SAKNAS!
lygus	1	5110(0.011432491139819366)	#SAKNAS!	#SAKNAS!
lymnaea	1	5110(0.011432491139819366)	#SAKNAS!	#SAKNAS!
lymph	1	5110(0.011432491139819366)	#SAKNAS!	#SAKNAS!
lymphocyte	1	5110(0.011432491139819366)	2359 (0.034)	#SAKNAS!
lymphocyte	1	5110(0.011432491139819366)	2359 (0.034)	#SAKNAS!
lymphocytic	1	5110(0.011432491139819366)	#SAKNAS!	#SAKNAS!
lymphorrhoea	1	5110(0.011432491139819366)	2359 (0.034)	#SAKNAS!
lymphorrhoea	1	5110(0.011432491139819366)	2359 (0.034)	#SAKNAS!
lysmata	1	5110(0.011432491139819366)	2359 (0.034)	#SAKNAS!
lysmata	1	5110(0.011432491139819366)	2359 (0.034)	#SAKNAS!
lysophospholipids	1	5110(0.011432491139819366)	#SAKNAS!	#SAKNAS!
lysozyme	1	5110(0.011432491139819366)	2359 (0.034)	#SAKNAS!
lysozyme	1	5110(0.011432491139819366)	2359 (0.034)	#SAKNAS!
m2	1	5110(0.011432491139819366)	#SAKNAS!	#SAKNAS!
m3243a&gt	1	5110(0.011432491139819366)	#SAKNAS!	2548 (0.033)
macadamia	1	5110(0.011432491139819366)	#SAKNAS!	#SAKNAS!
macadamieae	1	5110(0.011432491139819366)	#SAKNAS!	#SAKNAS!
macaque	1	5110(0.011432491139819366)	#SAKNAS!	#SAKNAS!
macerated	1	5110(0.011432491139819366)	#SAKNAS!	#SAKNAS!
machaon	1	5110(0.011432491139819366)	#SAKNAS!	2548 (0.033)
machine-learning	1	5110(0.011432491139819366)	#SAKNAS!	#SAKNAS!
mackerel	1	5110(0.011432491139819366)	#SAKNAS!	2548 (0.033)
macrobrachium	1	2693(0.0342974734194581)	2359 (0.034)	1668 (0.066)
macrodon	1	5110(0.011432491139819366)	#SAKNAS!	2548 (0.033)
macroglossia	1	5110(0.011432491139819366)	#SAKNAS!	#SAKNAS!
macroinvertebrates	1	5110(0.011432491139819366)	#SAKNAS!	2548 (0.033)
macrophyte	1	5110(0.011432491139819366)	2359 (0.034)	#SAKNAS!
macrophyte	1	5110(0.011432491139819366)	2359 (0.034)	#SAKNAS!
macropus	1	5110(0.011432491139819366)	2359 (0.034)	#SAKNAS!
macropus	1	5110(0.011432491139819366)	2359 (0.034)	#SAKNAS!
macroura	1	5110(0.011432491139819366)	#SAKNAS!	#SAKNAS!
maculata	1	3437(0.02286498227963873)	2359 (0.034)	2548 (0.033)
madrid	1	2693(0.0342974734194581)	2359 (0.034)	1668 (0.066)
maea	1	5110(0.011432491139819366)	#SAKNAS!	2548 (0.033)
magazine	1	5110(0.011432491139819366)	2359 (0.034)	#SAKNAS!
magazine	1	5110(0.011432491139819366)	2359 (0.034)	#SAKNAS!

magellanicus	1	5110(0.011432491139819366)	2359 (0.034)	#SAKNAS!
magellanicus	1	5110(0.011432491139819366)	2359 (0.034)	#SAKNAS!
magnetic	1	671(0.2057848405167486)	2359 (0.034)	816 (0.16)
mahale	1	5110(0.011432491139819366)	#SAKNAS!	2548 (0.033)
mahi	1	5110(0.011432491139819366)	#SAKNAS!	2548 (0.033)
maillard	1	5110(0.011432491139819366)	#SAKNAS!	2548 (0.033)
mainly	1	5110(0.011432491139819366)	#SAKNAS!	#SAKNAS!
maintain	1	1485(0.08002743797873557)	2359 (0.034)	1233 (0.1)
maintaining	1	2214(0.04572996455927746)	2359 (0.034)	1668 (0.066)
maize-dependent	1	5110(0.011432491139819366)	2359 (0.034)	#SAKNAS!
maize-dependent	1	5110(0.011432491139819366)	2359 (0.034)	#SAKNAS!
malabsorption	1	5110(0.011432491139819366)	#SAKNAS!	#SAKNAS!
maladaptation	1	5110(0.011432491139819366)	#SAKNAS!	#SAKNAS!
malagasy	1	5110(0.011432491139819366)	#SAKNAS!	2548 (0.033)
malalignments	1	5110(0.011432491139819366)	2359 (0.034)	#SAKNAS!
malalignments	1	5110(0.011432491139819366)	2359 (0.034)	#SAKNAS!
malawian	1	5110(0.011432491139819366)	2359 (0.034)	#SAKNAS!
malawian	1	5110(0.011432491139819366)	2359 (0.034)	#SAKNAS!
malaysian	1	2214(0.04572996455927746)	2359 (0.034)	1668 (0.066)
males	1	1335(0.09145992911855493)	2359 (0.034)	816 (0.16)
malfunctioning	1	5110(0.011432491139819366)	2359 (0.034)	#SAKNAS!
malfunctioning	1	5110(0.011432491139819366)	2359 (0.034)	#SAKNAS!
malhamensis	1	5110(0.011432491139819366)	#SAKNAS!	#SAKNAS!
mali	1	5110(0.011432491139819366)	2359 (0.034)	#SAKNAS!
mali	1	5110(0.011432491139819366)	2359 (0.034)	#SAKNAS!
malignancies	1	1894(0.05716245569909683)	2359 (0.034)	1233 (0.1)
malignancy	1	5110(0.011432491139819366)	2359 (0.034)	#SAKNAS!
malignancy	1	5110(0.011432491139819366)	2359 (0.034)	#SAKNAS!
mallorca	1	5110(0.011432491139819366)	#SAKNAS!	#SAKNAS!
malnourishment	1	5110(0.011432491139819366)	#SAKNAS!	#SAKNAS!
malnutrition-defined	1	5110(0.011432491139819366)	#SAKNAS!	2548 (0.033)
malocclusions	1	3437(0.02286498227963873)	2359 (0.034)	2548 (0.033)
malonyl-coa	1	5110(0.011432491139819366)	#SAKNAS!	2548 (0.033)
mal-reduced	1	5110(0.011432491139819366)	#SAKNAS!	#SAKNAS!
maltase	1	5110(0.011432491139819366)	2359 (0.034)	#SAKNAS!
maltase	1	5110(0.011432491139819366)	2359 (0.034)	#SAKNAS!
maltodextrin	1	5110(0.011432491139819366)	#SAKNAS!	#SAKNAS!
maltreatment	1	5110(0.011432491139819366)	#SAKNAS!	#SAKNAS!
mammalia	1	5110(0.011432491139819366)	2359 (0.034)	#SAKNAS!
mammalia	1	5110(0.011432491139819366)	2359 (0.034)	#SAKNAS!
mammals	1	3437(0.02286498227963873)	2359 (0.034)	2548 (0.033)
manage	1	5110(0.011432491139819366)	#SAKNAS!	2548 (0.033)
manavgat	1	5110(0.011432491139819366)	#SAKNAS!	2548 (0.033)

mandarin	1	5110(0.011432491139819366)	#SAKNAS!	#SAKNAS!
mandible	1	1894(0.05716245569909683)	2359 (0.034)	2548 (0.033)
mandibles	1	5110(0.011432491139819366)	#SAKNAS!	2548 (0.033)
mandrills	1	5110(0.011432491139819366)	#SAKNAS!	2548 (0.033)
maneuvers	1	5110(0.011432491139819366)	2359 (0.034)	#SAKNAS!
maneuvers	1	5110(0.011432491139819366)	2359 (0.034)	#SAKNAS!
mangalarga	1	5110(0.011432491139819366)	#SAKNAS!	2548 (0.033)
manganese	1	5110(0.011432491139819366)	#SAKNAS!	2548 (0.033)
manhattan	1	5110(0.011432491139819366)	2359 (0.034)	#SAKNAS!
manhattan	1	5110(0.011432491139819366)	2359 (0.034)	#SAKNAS!
manifesto	1	5110(0.011432491139819366)	#SAKNAS!	2548 (0.033)
manipulates	1	5110(0.011432491139819366)	#SAKNAS!	2548 (0.033)
manipulations	1	5110(0.011432491139819366)	#SAKNAS!	#SAKNAS!
manitoba	1	5110(0.011432491139819366)	#SAKNAS!	2548 (0.033)
mankind	1	5110(0.011432491139819366)	#SAKNAS!	2548 (0.033)
mann	1	5110(0.011432491139819366)	#SAKNAS!	2548 (0.033)
manna-feeding	1	5110(0.011432491139819366)	#SAKNAS!	2548 (0.033)
manner	1	3437(0.02286498227963873)	2359 (0.034)	2548 (0.033)
manometric	1	2214(0.04572996455927746)	2359 (0.034)	2548 (0.033)
mantis	1	5110(0.011432491139819366)	#SAKNAS!	2548 (0.033)
manufacture	1	5110(0.011432491139819366)	#SAKNAS!	#SAKNAS!
manuka	1	5110(0.011432491139819366)	#SAKNAS!	2548 (0.033)
maori	1	5110(0.011432491139819366)	2359 (0.034)	#SAKNAS!
maori	1	5110(0.011432491139819366)	2359 (0.034)	#SAKNAS!
mapuches	1	5110(0.011432491139819366)	#SAKNAS!	2548 (0.033)
maputo	1	5110(0.011432491139819366)	2359 (0.034)	#SAKNAS!
maputo	1	5110(0.011432491139819366)	2359 (0.034)	#SAKNAS!
maraca	1	5110(0.011432491139819366)	2359 (0.034)	#SAKNAS!
maraca	1	5110(0.011432491139819366)	2359 (0.034)	#SAKNAS!
marbles	1	5110(0.011432491139819366)	2359 (0.034)	#SAKNAS!
marbles	1	5110(0.011432491139819366)	2359 (0.034)	#SAKNAS!
marburg	1	3437(0.02286498227963873)	2359 (0.034)	#SAKNAS!
marchador	1	5110(0.011432491139819366)	#SAKNAS!	2548 (0.033)
marchesianus	1	5110(0.011432491139819366)	2359 (0.034)	#SAKNAS!
marchesianus	1	5110(0.011432491139819366)	2359 (0.034)	#SAKNAS!
marchiafava-bignami	1	5110(0.011432491139819366)	2359 (0.034)	#SAKNAS!
marchiafava-bignami	1	5110(0.011432491139819366)	2359 (0.034)	#SAKNAS!
mare	1	5110(0.011432491139819366)	#SAKNAS!	2548 (0.033)
marennes-oleron	1	5110(0.011432491139819366)	2359 (0.034)	#SAKNAS!
marennes-oleron	1	5110(0.011432491139819366)	2359 (0.034)	#SAKNAS!
mares	1	3437(0.02286498227963873)	2359 (0.034)	#SAKNAS!
marfan	1	5110(0.011432491139819366)	2359 (0.034)	#SAKNAS!
marfan	1	5110(0.011432491139819366)	2359 (0.034)	#SAKNAS!

marginales	1	5110(0.011432491139819366)	#SAKNAS!	#SAKNAS!
marginalised	1	5110(0.011432491139819366)	2359 (0.034)	#SAKNAS!
marginalised	1	5110(0.011432491139819366)	2359 (0.034)	#SAKNAS!
maria	1	5110(0.011432491139819366)	#SAKNAS!	#SAKNAS!
marijuana-smoking	1	5110(0.011432491139819366)	#SAKNAS!	#SAKNAS!
marital	1	5110(0.011432491139819366)	#SAKNAS!	2548 (0.033)
marker	1	5110(0.011432491139819366)	#SAKNAS!	#SAKNAS!
market	1	3437(0.02286498227963873)	2359 (0.034)	#SAKNAS!
markets	1	5110(0.011432491139819366)	#SAKNAS!	#SAKNAS!
markhoz	1	5110(0.011432491139819366)	2359 (0.034)	#SAKNAS!
markhoz	1	5110(0.011432491139819366)	2359 (0.034)	#SAKNAS!
marmoratus	1	5110(0.011432491139819366)	#SAKNAS!	2548 (0.033)
marmoratuso	1	5110(0.011432491139819366)	#SAKNAS!	2548 (0.033)
marmoreus	1	5110(0.011432491139819366)	#SAKNAS!	#SAKNAS!
marmoset	1	5110(0.011432491139819366)	#SAKNAS!	2548 (0.033)
marmosets	1	5110(0.011432491139819366)	#SAKNAS!	#SAKNAS!
marmot	1	5110(0.011432491139819366)	#SAKNAS!	#SAKNAS!
marmota	1	3437(0.02286498227963873)	2359 (0.034)	#SAKNAS!
marmots	1	5110(0.011432491139819366)	2359 (0.034)	#SAKNAS!
marmots	1	5110(0.011432491139819366)	2359 (0.034)	#SAKNAS!
marsh	1	5110(0.011432491139819366)	#SAKNAS!	2548 (0.033)
marshall	1	5110(0.011432491139819366)	#SAKNAS!	#SAKNAS!
marsupial	1	3437(0.02286498227963873)	2359 (0.034)	#SAKNAS!
marsupials	1	5110(0.011432491139819366)	#SAKNAS!	#SAKNAS!
mart	1	5110(0.011432491139819366)	#SAKNAS!	#SAKNAS!
martens	1	5110(0.011432491139819366)	2359 (0.034)	#SAKNAS!
martens	1	5110(0.011432491139819366)	2359 (0.034)	#SAKNAS!
martes	1	5110(0.011432491139819366)	2359 (0.034)	#SAKNAS!
martes	1	5110(0.011432491139819366)	2359 (0.034)	#SAKNAS!
maryland	1	5110(0.011432491139819366)	#SAKNAS!	2548 (0.033)
masai	1	5110(0.011432491139819366)	#SAKNAS!	2548 (0.033)
mask	1	5110(0.011432491139819366)	2359 (0.034)	#SAKNAS!
mask	1	5110(0.011432491139819366)	2359 (0.034)	#SAKNAS!
masking	1	3437(0.02286498227963873)	2359 (0.034)	2548 (0.033)
masticability	1	5110(0.011432491139819366)	#SAKNAS!	#SAKNAS!
masticaticatory	1	5110(0.011432491139819366)	#SAKNAS!	#SAKNAS!
mastitis-metritis-agalactia	1	5110(0.011432491139819366)	#SAKNAS!	#SAKNAS!
matar籀	1	5110(0.011432491139819366)	#SAKNAS!	#SAKNAS!
matched-cohort	1	5110(0.011432491139819366)	#SAKNAS!	#SAKNAS!
match-mismatch	1	5110(0.011432491139819366)	#SAKNAS!	2548 (0.033)
mate	1	5110(0.011432491139819366)	#SAKNAS!	#SAKNAS!
mate-guarding	1	5110(0.011432491139819366)	#SAKNAS!	2548 (0.033)
material	1	2214(0.04572996455927746)	2359 (0.034)	1668 (0.066)

materials	1	2693(0.0342974734194581)	2359 (0.034)	#SAKNAS!
maternal-child	1	3437(0.02286498227963873)	2359 (0.034)	#SAKNAS!
maternal-neonatal	1	5110(0.011432491139819366)	#SAKNAS!	#SAKNAS!
mathematical	1	2693(0.0342974734194581)	2359 (0.034)	2548 (0.033)
matlab	1	5110(0.011432491139819366)	2359 (0.034)	#SAKNAS!
matlab	1	5110(0.011432491139819366)	2359 (0.034)	#SAKNAS!
matricaria	1	5110(0.011432491139819366)	#SAKNAS!	2548 (0.033)
matrices	1	5110(0.011432491139819366)	#SAKNAS!	2548 (0.033)
maturation	1	2693(0.0342974734194581)	2359 (0.034)	1668 (0.066)
mature	1	5110(0.011432491139819366)	2359 (0.034)	#SAKNAS!
mature	1	5110(0.011432491139819366)	2359 (0.034)	#SAKNAS!
maturity	1	5110(0.011432491139819366)	#SAKNAS!	2548 (0.033)
maura	1	5110(0.011432491139819366)	#SAKNAS!	2548 (0.033)
mausoleum	1	5110(0.011432491139819366)	#SAKNAS!	2548 (0.033)
mawsoni	1	5110(0.011432491139819366)	#SAKNAS!	#SAKNAS!
maxillary	1	5110(0.011432491139819366)	#SAKNAS!	#SAKNAS!
maxillofacial	1	3437(0.02286498227963873)	2359 (0.034)	#SAKNAS!
maxillomandibular	1	5110(0.011432491139819366)	#SAKNAS!	#SAKNAS!
maximal	1	5110(0.011432491139819366)	2359 (0.034)	#SAKNAS!
maximal	1	5110(0.011432491139819366)	2359 (0.034)	#SAKNAS!
maximization	1	5110(0.011432491139819366)	2359 (0.034)	#SAKNAS!
maximization	1	5110(0.011432491139819366)	2359 (0.034)	#SAKNAS!
maximizing	1	5110(0.011432491139819366)	2359 (0.034)	#SAKNAS!
maximizing	1	5110(0.011432491139819366)	2359 (0.034)	#SAKNAS!
maximus	1	2693(0.0342974734194581)	2359 (0.034)	#SAKNAS!
mayotte	1	5110(0.011432491139819366)	2359 (0.034)	#SAKNAS!
mayotte	1	5110(0.011432491139819366)	2359 (0.034)	#SAKNAS!
mbs	1	5110(0.011432491139819366)	2359 (0.034)	#SAKNAS!
mbs	1	5110(0.011432491139819366)	2359 (0.034)	#SAKNAS!
mc4r	1	3437(0.02286498227963873)	2359 (0.034)	#SAKNAS!
mcc-spain	1	5110(0.011432491139819366)	#SAKNAS!	#SAKNAS!
mch	1	5110(0.011432491139819366)	2359 (0.034)	#SAKNAS!
mch	1	5110(0.011432491139819366)	2359 (0.034)	#SAKNAS!
mci	1	5110(0.011432491139819366)	#SAKNAS!	2548 (0.033)
mckeown	1	5110(0.011432491139819366)	#SAKNAS!	2548 (0.033)
mcp-1	1	5110(0.011432491139819366)	#SAKNAS!	2548 (0.033)
md	1	5110(0.011432491139819366)	#SAKNAS!	#SAKNAS!
mdt	1	5110(0.011432491139819366)	#SAKNAS!	#SAKNAS!
mdt-pd	1	5110(0.011432491139819366)	#SAKNAS!	2548 (0.033)
me	1	5110(0.011432491139819366)	#SAKNAS!	#SAKNAS!
meadow	1	5110(0.011432491139819366)	2359 (0.034)	#SAKNAS!
meadow	1	5110(0.011432491139819366)	2359 (0.034)	#SAKNAS!
meagre	1	5110(0.011432491139819366)	#SAKNAS!	#SAKNAS!

meal-based	1	5110(0.011432491139819366)	#SAKNAS!	#SAKNAS!
meal-eating	1	5110(0.011432491139819366)	#SAKNAS!	#SAKNAS!
mealtime	1	1335(0.09145992911855493)	2359 (0.034)	816 (0.16)
mealworms	1	5110(0.011432491139819366)	#SAKNAS!	#SAKNAS!
mealy	1	5110(0.011432491139819366)	#SAKNAS!	2548 (0.033)
meaning	1	5110(0.011432491139819366)	#SAKNAS!	#SAKNAS!
meat	1	1678(0.0685949468389162)	2359 (0.034)	2548 (0.033)
meat-type	1	5110(0.011432491139819366)	2359 (0.034)	#SAKNAS!
meat-type	1	5110(0.011432491139819366)	2359 (0.034)	#SAKNAS!
mechanical	1	1335(0.09145992911855493)	2359 (0.034)	1233 (0.1)
mechanistic	1	3437(0.02286498227963873)	2359 (0.034)	2548 (0.033)
mechanosensitive	1	5110(0.011432491139819366)	#SAKNAS!	#SAKNAS!
mediastinitis	1	5110(0.011432491139819366)	#SAKNAS!	2548 (0.033)
mediated	1	5110(0.011432491139819366)	#SAKNAS!	#SAKNAS!
mediation	1	743(0.18291985823710985)	2359 (0.034)	1233 (0.1)
mediators	1	1894(0.05716245569909683)	2359 (0.034)	1668 (0.066)
medicaid	1	2214(0.04572996455927746)	2359 (0.034)	#SAKNAS!
medicaid-insured	1	5110(0.011432491139819366)	2359 (0.034)	#SAKNAS!
medicaid-insured	1	5110(0.011432491139819366)	2359 (0.034)	#SAKNAS!
medicare	1	5110(0.011432491139819366)	#SAKNAS!	#SAKNAS!
medication-induced	1	5110(0.011432491139819366)	#SAKNAS!	2548 (0.033)
medicines	1	5110(0.011432491139819366)	#SAKNAS!	2548 (0.033)
medico-oral	1	5110(0.011432491139819366)	#SAKNAS!	#SAKNAS!
medina	1	5110(0.011432491139819366)	#SAKNAS!	2548 (0.033)
medium-chain	1	5110(0.011432491139819366)	2359 (0.034)	#SAKNAS!
medium-chain	1	5110(0.011432491139819366)	2359 (0.034)	#SAKNAS!
medroxyprogesterone	1	5110(0.011432491139819366)	2359 (0.034)	#SAKNAS!
medroxyprogesterone	1	5110(0.011432491139819366)	2359 (0.034)	#SAKNAS!
medullary	1	5110(0.011432491139819366)	#SAKNAS!	2548 (0.033)
meetings	1	5110(0.011432491139819366)	#SAKNAS!	2548 (0.033)
mega	1	5110(0.011432491139819366)	#SAKNAS!	#SAKNAS!
mega-consumer	1	5110(0.011432491139819366)	#SAKNAS!	2548 (0.033)
megaesophagus	1	3437(0.02286498227963873)	2359 (0.034)	2548 (0.033)
megathyrsus	1	5110(0.011432491139819366)	#SAKNAS!	#SAKNAS!
mehelyi	1	5110(0.011432491139819366)	#SAKNAS!	2548 (0.033)
mekelle	1	5110(0.011432491139819366)	#SAKNAS!	#SAKNAS!
mela	1	5110(0.011432491139819366)	#SAKNAS!	2548 (0.033)
melaleuca	1	5110(0.011432491139819366)	2359 (0.034)	#SAKNAS!
melaleuca	1	5110(0.011432491139819366)	2359 (0.034)	#SAKNAS!
melaleucae	1	5110(0.011432491139819366)	2359 (0.034)	#SAKNAS!
melaleucae	1	5110(0.011432491139819366)	2359 (0.034)	#SAKNAS!
melanaphis	1	5110(0.011432491139819366)	#SAKNAS!	2548 (0.033)
melanoleuca	1	5110(0.011432491139819366)	2359 (0.034)	#SAKNAS!

melanoleuca	1	5110(0.011432491139819366)	2359 (0.034)	#SAKNAS!
melanura	1	5110(0.011432491139819366)	2359 (0.034)	#SAKNAS!
melanura	1	5110(0.011432491139819366)	2359 (0.034)	#SAKNAS!
mellitus-associated	1	5110(0.011432491139819366)	2359 (0.034)	#SAKNAS!
mellitus-associated	1	5110(0.011432491139819366)	2359 (0.034)	#SAKNAS!
melphalan	1	5110(0.011432491139819366)	2359 (0.034)	#SAKNAS!
melphalan	1	5110(0.011432491139819366)	2359 (0.034)	#SAKNAS!
member	1	5110(0.011432491139819366)	2359 (0.034)	#SAKNAS!
member	1	5110(0.011432491139819366)	2359 (0.034)	#SAKNAS!
members	1	5110(0.011432491139819366)	#SAKNAS!	2548 (0.033)
memorial	1	5110(0.011432491139819366)	2359 (0.034)	#SAKNAS!
memorial	1	5110(0.011432491139819366)	2359 (0.034)	#SAKNAS!
memories	1	5110(0.011432491139819366)	2359 (0.034)	#SAKNAS!
memories	1	5110(0.011432491139819366)	2359 (0.034)	#SAKNAS!
mendocinus	1	5110(0.011432491139819366)	2359 (0.034)	#SAKNAS!
mendocinus	1	5110(0.011432491139819366)	2359 (0.034)	#SAKNAS!
menippe	1	5110(0.011432491139819366)	#SAKNAS!	2548 (0.033)
menstrual	1	2693(0.0342974734194581)	2359 (0.034)	1668 (0.066)
mental	1	966(0.1371898936778324)	2359 (0.034)	999 (0.13)
mentha	1	5110(0.011432491139819366)	#SAKNAS!	2548 (0.033)
mentors	1	5110(0.011432491139819366)	#SAKNAS!	2548 (0.033)
menuch	1	5110(0.011432491139819366)	#SAKNAS!	2548 (0.033)
merganetta	1	5110(0.011432491139819366)	#SAKNAS!	2548 (0.033)
meridionalis	1	5110(0.011432491139819366)	2359 (0.034)	#SAKNAS!
meridionalis	1	5110(0.011432491139819366)	2359 (0.034)	#SAKNAS!
merit	1	5110(0.011432491139819366)	#SAKNAS!	#SAKNAS!
merobenthic	1	5110(0.011432491139819366)	#SAKNAS!	#SAKNAS!
mesalazine	1	5110(0.011432491139819366)	2359 (0.034)	#SAKNAS!
mesalazine	1	5110(0.011432491139819366)	2359 (0.034)	#SAKNAS!
mesencephalic	1	5110(0.011432491139819366)	2359 (0.034)	#SAKNAS!
mesencephalic	1	5110(0.011432491139819366)	2359 (0.034)	#SAKNAS!
mesenchymal	1	5110(0.011432491139819366)	#SAKNAS!	#SAKNAS!
mesenteroides	1	5110(0.011432491139819366)	#SAKNAS!	#SAKNAS!
meshes	1	5110(0.011432491139819366)	#SAKNAS!	2548 (0.033)
mesial	1	5110(0.011432491139819366)	2359 (0.034)	#SAKNAS!
mesial	1	5110(0.011432491139819366)	2359 (0.034)	#SAKNAS!
mesocarp	1	5110(0.011432491139819366)	2359 (0.034)	#SAKNAS!
mesocarp	1	5110(0.011432491139819366)	2359 (0.034)	#SAKNAS!
mesograzers	1	5110(0.011432491139819366)	#SAKNAS!	2548 (0.033)
mesowear	1	5110(0.011432491139819366)	#SAKNAS!	2548 (0.033)
mesozooplankton	1	5110(0.011432491139819366)	#SAKNAS!	2548 (0.033)
mesquite	1	5110(0.011432491139819366)	#SAKNAS!	2548 (0.033)
message	1	3437(0.02286498227963873)	2359 (0.034)	2548 (0.033)

messenian	1	5110(0.011432491139819366)	#SAKNAS!	#SAKNAS!
mesylate	1	5110(0.011432491139819366)	#SAKNAS!	#SAKNAS!
meta-analysis	1	628(0.21721733165656798)	2359 (0.034)	816 (0.16)
metabolically	1	5110(0.011432491139819366)	#SAKNAS!	#SAKNAS!
metabolite	1	3437(0.02286498227963873)	2359 (0.034)	2548 (0.033)
metabolites	1	836(0.16005487595747114)	2359 (0.034)	1668 (0.066)
metabolome	1	5110(0.011432491139819366)	#SAKNAS!	2548 (0.033)
metabolomic	1	2214(0.04572996455927746)	2359 (0.034)	#SAKNAS!
metabolomics	1	5110(0.011432491139819366)	#SAKNAS!	#SAKNAS!
metalloproteinase	1	5110(0.011432491139819366)	#SAKNAS!	2548 (0.033)
metalloproteinase-1	1	5110(0.011432491139819366)	2359 (0.034)	#SAKNAS!
metalloproteinase-1	1	5110(0.011432491139819366)	2359 (0.034)	#SAKNAS!
metalloproteinases-8	1	5110(0.011432491139819366)	2359 (0.034)	#SAKNAS!
metalloproteinases-8	1	5110(0.011432491139819366)	2359 (0.034)	#SAKNAS!
metals	1	1894(0.05716245569909683)	2359 (0.034)	1668 (0.066)
metaphor	1	5110(0.011432491139819366)	#SAKNAS!	#SAKNAS!
metaplasia	1	5110(0.011432491139819366)	2359 (0.034)	#SAKNAS!
metaplasia	1	5110(0.011432491139819366)	2359 (0.034)	#SAKNAS!
metastases	1	5110(0.011432491139819366)	#SAKNAS!	2548 (0.033)
metastasis	1	5110(0.011432491139819366)	2359 (0.034)	#SAKNAS!
metastasis	1	5110(0.011432491139819366)	2359 (0.034)	#SAKNAS!
metastasis-induced	1	5110(0.011432491139819366)	#SAKNAS!	#SAKNAS!
metchnikoff	1	5110(0.011432491139819366)	#SAKNAS!	2548 (0.033)
methacrylate	1	5110(0.011432491139819366)	#SAKNAS!	#SAKNAS!
methadone	1	3437(0.02286498227963873)	2359 (0.034)	2548 (0.033)
methamphetamine-related	1	5110(0.011432491139819366)	#SAKNAS!	2548 (0.033)
methionine	1	5110(0.011432491139819366)	#SAKNAS!	#SAKNAS!
methotrexate	1	5110(0.011432491139819366)	2359 (0.034)	#SAKNAS!
methotrexate	1	5110(0.011432491139819366)	2359 (0.034)	#SAKNAS!
methyl	1	5110(0.011432491139819366)	#SAKNAS!	#SAKNAS!
methylphenidate	1	3437(0.02286498227963873)	2359 (0.034)	#SAKNAS!
metrics	1	5110(0.011432491139819366)	#SAKNAS!	#SAKNAS!
metricus	1	5110(0.011432491139819366)	#SAKNAS!	#SAKNAS!
metroparks	1	5110(0.011432491139819366)	#SAKNAS!	2548 (0.033)
metropolis	1	5110(0.011432491139819366)	#SAKNAS!	2548 (0.033)
met-selective	1	5110(0.011432491139819366)	#SAKNAS!	#SAKNAS!
mets-ir	1	5110(0.011432491139819366)	#SAKNAS!	#SAKNAS!
mexicana	1	5110(0.011432491139819366)	#SAKNAS!	2548 (0.033)
mexicans	1	5110(0.011432491139819366)	#SAKNAS!	2548 (0.033)
mia	1	5110(0.011432491139819366)	#SAKNAS!	#SAKNAS!
miang	1	5110(0.011432491139819366)	#SAKNAS!	2548 (0.033)
micellar	1	5110(0.011432491139819366)	#SAKNAS!	#SAKNAS!
michigan	1	5110(0.011432491139819366)	#SAKNAS!	#SAKNAS!

michoacan	1	5110(0.011432491139819366)	2359 (0.034)	#SAKNAS!
michoacan	1	5110(0.011432491139819366)	2359 (0.034)	#SAKNAS!
microalgae	1	2693(0.0342974734194581)	2359 (0.034)	1668 (0.066)
microbe	1	5110(0.011432491139819366)	#SAKNAS!	2548 (0.033)
microbiologic	1	5110(0.011432491139819366)	2359 (0.034)	#SAKNAS!
microbiologic	1	5110(0.011432491139819366)	2359 (0.034)	#SAKNAS!
microbiome-by-ethanol	1	5110(0.011432491139819366)	#SAKNAS!	#SAKNAS!
microbleeds	1	5110(0.011432491139819366)	#SAKNAS!	2548 (0.033)
microbound	1	5110(0.011432491139819366)	2359 (0.034)	#SAKNAS!
microbound	1	5110(0.011432491139819366)	2359 (0.034)	#SAKNAS!
microcapsules	1	5110(0.011432491139819366)	#SAKNAS!	#SAKNAS!
microcephaly	1	5110(0.011432491139819366)	#SAKNAS!	#SAKNAS!
microcirculation	1	5110(0.011432491139819366)	#SAKNAS!	2548 (0.033)
microclimates	1	5110(0.011432491139819366)	2359 (0.034)	#SAKNAS!
microclimates	1	5110(0.011432491139819366)	2359 (0.034)	#SAKNAS!
microcosm	1	5110(0.011432491139819366)	#SAKNAS!	#SAKNAS!
microduplication	1	5110(0.011432491139819366)	2359 (0.034)	#SAKNAS!
microduplication	1	5110(0.011432491139819366)	2359 (0.034)	#SAKNAS!
microecology	1	5110(0.011432491139819366)	#SAKNAS!	#SAKNAS!
microelements	1	5110(0.011432491139819366)	#SAKNAS!	#SAKNAS!
microgel-reinforced	1	5110(0.011432491139819366)	#SAKNAS!	#SAKNAS!
microgels-based	1	5110(0.011432491139819366)	#SAKNAS!	#SAKNAS!
microgravity	1	5110(0.011432491139819366)	#SAKNAS!	#SAKNAS!
microhabitat	1	5110(0.011432491139819366)	#SAKNAS!	2548 (0.033)
microhylidae	1	5110(0.011432491139819366)	#SAKNAS!	2548 (0.033)
microinflammation	1	5110(0.011432491139819366)	#SAKNAS!	#SAKNAS!
micronesia	1	5110(0.011432491139819366)	#SAKNAS!	#SAKNAS!
micronucleus	1	5110(0.011432491139819366)	2359 (0.034)	#SAKNAS!
micronucleus	1	5110(0.011432491139819366)	2359 (0.034)	#SAKNAS!
micronutrients	1	2214(0.04572996455927746)	2359 (0.034)	2548 (0.033)
microorganisms	1	1678(0.0685949468389162)	2359 (0.034)	1233 (0.1)
microparticles	1	5110(0.011432491139819366)	#SAKNAS!	2548 (0.033)
microplastic	1	5110(0.011432491139819366)	#SAKNAS!	2548 (0.033)
microrna	1	5110(0.011432491139819366)	2359 (0.034)	#SAKNAS!
microrna	1	5110(0.011432491139819366)	2359 (0.034)	#SAKNAS!
micrornas	1	5110(0.011432491139819366)	#SAKNAS!	#SAKNAS!
microsphere-in-capsule	1	5110(0.011432491139819366)	#SAKNAS!	2548 (0.033)
microstomia	1	5110(0.011432491139819366)	#SAKNAS!	2548 (0.033)
microtus	1	5110(0.011432491139819366)	2359 (0.034)	#SAKNAS!
microtus	1	5110(0.011432491139819366)	2359 (0.034)	#SAKNAS!
microwear	1	5110(0.011432491139819366)	#SAKNAS!	2548 (0.033)
mid-afternoon	1	5110(0.011432491139819366)	#SAKNAS!	#SAKNAS!
midazolam	1	5110(0.011432491139819366)	#SAKNAS!	#SAKNAS!

midbrain	1 5110(0.011432491139819366)	#SAKNAS!	2548 (0.033)
mid-buccal	1 5110(0.011432491139819366)	#SAKNAS!	2548 (0.033)
mid-childhood	1 5110(0.011432491139819366)	#SAKNAS!	2548 (0.033)
mid-decade	1 5110(0.011432491139819366)	2359 (0.034)	#SAKNAS!
mid-decade	1 5110(0.011432491139819366)	2359 (0.034)	#SAKNAS!
middle-and	1 5110(0.011432491139819366)	#SAKNAS!	2548 (0.033)
middle-income	1 3437(0.02286498227963873)	2359 (0.034)	#SAKNAS!
mid-gestation	1 5110(0.011432491139819366)	#SAKNAS!	#SAKNAS!
mid-lactation	1 5110(0.011432491139819366)	2359 (0.034)	#SAKNAS!
mid-lactation	1 5110(0.011432491139819366)	2359 (0.034)	#SAKNAS!
midlife	1 5110(0.011432491139819366)	#SAKNAS!	#SAKNAS!
mid-long	1 5110(0.011432491139819366)	#SAKNAS!	2548 (0.033)
mid-nineteenth-century	1 5110(0.011432491139819366)	#SAKNAS!	2548 (0.033)
mid-term	1 5110(0.011432491139819366)	#SAKNAS!	2548 (0.033)
mid-to	1 5110(0.011432491139819366)	2359 (0.034)	#SAKNAS!
mid-to	1 5110(0.011432491139819366)	2359 (0.034)	#SAKNAS!
midwifery	1 5110(0.011432491139819366)	2359 (0.034)	#SAKNAS!
midwifery	1 5110(0.011432491139819366)	2359 (0.034)	#SAKNAS!
midwives	1 5110(0.011432491139819366)	#SAKNAS!	2548 (0.033)
migrating	1 5110(0.011432491139819366)	#SAKNAS!	2548 (0.033)
migratoria	1 5110(0.011432491139819366)	2359 (0.034)	#SAKNAS!
migratoria	1 5110(0.011432491139819366)	2359 (0.034)	#SAKNAS!
migratory	1 5110(0.011432491139819366)	#SAKNAS!	2548 (0.033)
mildew	1 5110(0.011432491139819366)	#SAKNAS!	2548 (0.033)
mild-to-moderate	1 5110(0.011432491139819366)	#SAKNAS!	#SAKNAS!
miles	1 5110(0.011432491139819366)	#SAKNAS!	#SAKNAS!
milestone	1 5110(0.011432491139819366)	#SAKNAS!	#SAKNAS!
milking	1 3437(0.02286498227963873)	2359 (0.034)	2548 (0.033)
milk-products	1 5110(0.011432491139819366)	2359 (0.034)	#SAKNAS!
milk-products	1 5110(0.011432491139819366)	2359 (0.034)	#SAKNAS!
milkybar	1 5110(0.011432491139819366)	#SAKNAS!	2548 (0.033)
millennium	1 5110(0.011432491139819366)	2359 (0.034)	#SAKNAS!
millennium	1 5110(0.011432491139819366)	2359 (0.034)	#SAKNAS!
millipede	1 5110(0.011432491139819366)	#SAKNAS!	#SAKNAS!
milne	1 5110(0.011432491139819366)	2359 (0.034)	#SAKNAS!
milne	1 5110(0.011432491139819366)	2359 (0.034)	#SAKNAS!
mimicking	1 5110(0.011432491139819366)	#SAKNAS!	2548 (0.033)
mimics	1 5110(0.011432491139819366)	2359 (0.034)	#SAKNAS!
mimics	1 5110(0.011432491139819366)	2359 (0.034)	#SAKNAS!
mindanao	1 5110(0.011432491139819366)	2359 (0.034)	#SAKNAS!
mindanao	1 5110(0.011432491139819366)	2359 (0.034)	#SAKNAS!
mine	1 5110(0.011432491139819366)	2359 (0.034)	#SAKNAS!
mine	1 5110(0.011432491139819366)	2359 (0.034)	#SAKNAS!

mini	1	5110(0.011432491139819366)	#SAKNAS!	#SAKNAS!
mini-gastric	1	5110(0.011432491139819366)	#SAKNAS!	2548 (0.033)
mini-laparotomy	1	5110(0.011432491139819366)	2359 (0.034)	#SAKNAS!
mini-laparotomy	1	5110(0.011432491139819366)	2359 (0.034)	#SAKNAS!
minimal	1	2693(0.0342974734194581)	2359 (0.034)	#SAKNAS!
minimally	1	1894(0.0571624556990968З)	2359 (0.034)	1668 (0.066)
minimal-massive	1	5110(0.011432491139819366)	#SAKNAS!	2548 (0.033)
minimize	1	5110(0.011432491139819366)	#SAKNAS!	#SAKNAS!
minimum	1	1894(0.0571624556990968З)	2359 (0.034)	#SAKNAS!
mining	1	2214(0.04572996455927746)	2359 (0.034)	1233 (0.1)
mini-nutritional	1	5110(0.011432491139819366)	2359 (0.034)	#SAKNAS!
mini-nutritional	1	5110(0.011432491139819366)	2359 (0.034)	#SAKNAS!
minipigs	1	5110(0.011432491139819366)	2359 (0.034)	#SAKNAS!
minipigs	1	5110(0.011432491139819366)	2359 (0.034)	#SAKNAS!
mink	1	5110(0.011432491139819366)	2359 (0.034)	#SAKNAS!
mink	1	5110(0.011432491139819366)	2359 (0.034)	#SAKNAS!
minke	1	5110(0.011432491139819366)	#SAKNAS!	#SAKNAS!
minnesota	1	5110(0.011432491139819366)	#SAKNAS!	2548 (0.033)
minnie	1	5110(0.011432491139819366)	2359 (0.034)	#SAKNAS!
minnie	1	5110(0.011432491139819366)	2359 (0.034)	#SAKNAS!
minocycline	1	5110(0.011432491139819366)	#SAKNAS!	2548 (0.033)
minor	1	3437(0.02286498227963873)	2359 (0.034)	#SAKNAS!
mir155	1	5110(0.011432491139819366)	#SAKNAS!	#SAKNAS!
mir-423-5p	1	5110(0.011432491139819366)	#SAKNAS!	#SAKNAS!
mirabilis	1	5110(0.011432491139819366)	2359 (0.034)	#SAKNAS!
mirabilis	1	5110(0.011432491139819366)	2359 (0.034)	#SAKNAS!
misadventure	1	5110(0.011432491139819366)	#SAKNAS!	2548 (0.033)
miscanthi	1	5110(0.011432491139819366)	#SAKNAS!	#SAKNAS!
misconceptions	1	5110(0.011432491139819366)	#SAKNAS!	#SAKNAS!
misreporting	1	5110(0.011432491139819366)	#SAKNAS!	#SAKNAS!
misuse	1	3437(0.02286498227963873)	2359 (0.034)	#SAKNAS!
misusing	1	5110(0.011432491139819366)	2359 (0.034)	#SAKNAS!
misusing	1	5110(0.011432491139819366)	2359 (0.034)	#SAKNAS!
mite	1	5110(0.011432491139819366)	#SAKNAS!	2548 (0.033)
mitigates	1	3437(0.02286498227963873)	2359 (0.034)	2548 (0.033)
mitochondrial-related	1	5110(0.011432491139819366)	2359 (0.034)	#SAKNAS!
mitochondrial-related	1	5110(0.011432491139819366)	2359 (0.034)	#SAKNAS!
mitotic	1	5110(0.011432491139819366)	2359 (0.034)	#SAKNAS!
mitotic	1	5110(0.011432491139819366)	2359 (0.034)	#SAKNAS!
mix	1	5110(0.011432491139819366)	2359 (0.034)	#SAKNAS!
mix	1	5110(0.011432491139819366)	2359 (0.034)	#SAKNAS!
mixed-macronutrient	1	5110(0.011432491139819366)	2359 (0.034)	#SAKNAS!
mixed-macronutrient	1	5110(0.011432491139819366)	2359 (0.034)	#SAKNAS!

mixed-method	1	5110(0.011432491139819366)	#SAKNAS!	2548 (0.033)
mixed-parity	1	5110(0.011432491139819366)	2359 (0.034)	#SAKNAS!
mixed-parity	1	5110(0.011432491139819366)	2359 (0.034)	#SAKNAS!
mixed-pollen	1	5110(0.011432491139819366)	#SAKNAS!	#SAKNAS!
mixed-sediment	1	5110(0.011432491139819366)	2359 (0.034)	#SAKNAS!
mixed-sediment	1	5110(0.011432491139819366)	2359 (0.034)	#SAKNAS!
mixture	1	5110(0.011432491139819366)	#SAKNAS!	#SAKNAS!
mixtures	1	1678(0.0685949468389162)	2359 (0.034)	2548 (0.033)
miyagi	1	5110(0.011432491139819366)	#SAKNAS!	2548 (0.033)
m-mode	1	5110(0.011432491139819366)	#SAKNAS!	2548 (0.033)
mmp-8	1	5110(0.011432491139819366)	#SAKNAS!	2548 (0.033)
mna	1	5110(0.011432491139819366)	#SAKNAS!	#SAKNAS!
mna簣	1	5110(0.011432491139819366)	2359 (0.034)	#SAKNAS!
mna簣	1	5110(0.011432491139819366)	2359 (0.034)	#SAKNAS!
mnp	1	5110(0.011432491139819366)	#SAKNAS!	2548 (0.033)
mo	1	2693(0.0342974734194581)	2359 (0.034)	2548 (0.033)
mobile	1	1335(0.09145992911855493)	2359 (0.034)	1233 (0.1)
mod	1	5110(0.011432491139819366)	2359 (0.034)	#SAKNAS!
mod	1	5110(0.011432491139819366)	2359 (0.034)	#SAKNAS!
modality	1	2693(0.0342974734194581)	2359 (0.034)	1668 (0.066)
mode	1	3437(0.02286498227963873)	2359 (0.034)	#SAKNAS!
modelling	1	1485(0.08002743797873557)	2359 (0.034)	999 (0.13)
modem	1	5110(0.011432491139819366)	2359 (0.034)	#SAKNAS!
modem	1	5110(0.011432491139819366)	2359 (0.034)	#SAKNAS!
moderate-severe	1	5110(0.011432491139819366)	#SAKNAS!	2548 (0.033)
moderate-to-severe	1	5110(0.011432491139819366)	#SAKNAS!	2548 (0.033)
moderation	1	3437(0.02286498227963873)	2359 (0.034)	#SAKNAS!
moderator	1	5110(0.011432491139819366)	#SAKNAS!	2548 (0.033)
modern	1	5110(0.011432491139819366)	2359 (0.034)	#SAKNAS!
modern	1	5110(0.011432491139819366)	2359 (0.034)	#SAKNAS!
modes	1	5110(0.011432491139819366)	#SAKNAS!	#SAKNAS!
modifiable	1	1485(0.08002743797873557)	2359 (0.034)	2548 (0.033)
modified-texture	1	5110(0.011432491139819366)	#SAKNAS!	2548 (0.033)
modifiers	1	5110(0.011432491139819366)	#SAKNAS!	2548 (0.033)
modify	1	2693(0.0342974734194581)	2359 (0.034)	2548 (0.033)
modifying	1	2693(0.0342974734194581)	2359 (0.034)	1668 (0.066)
modulators	1	5110(0.011432491139819366)	#SAKNAS!	#SAKNAS!
module	1	1894(0.05716245569909683)	2359 (0.034)	2548 (0.033)
mofid	1	5110(0.011432491139819366)	2359 (0.034)	#SAKNAS!
mofid	1	5110(0.011432491139819366)	2359 (0.034)	#SAKNAS!
mohepi	1	5110(0.011432491139819366)	#SAKNAS!	#SAKNAS!
moisture	1	5110(0.011432491139819366)	2359 (0.034)	#SAKNAS!
moisture	1	5110(0.011432491139819366)	2359 (0.034)	#SAKNAS!

mokolodi	1	5110(0.011432491139819366)	2359 (0.034)	#SAKNAS!
mokolodi	1	5110(0.011432491139819366)	2359 (0.034)	#SAKNAS!
molar-incisor-hypomineralisatio	1	5110(0.011432491139819366)	#SAKNAS!	2548 (0.033)
molecular	1	1485(0.08002743797873557)	2359 (0.034)	1233 (0.1)
molecule	1	5110(0.011432491139819366)	#SAKNAS!	2548 (0.033)
mollis	1	3437(0.02286498227963873)	2359 (0.034)	2548 (0.033)
molluscan	1	5110(0.011432491139819366)	#SAKNAS!	2548 (0.033)
molluscs	1	5110(0.011432491139819366)	2359 (0.034)	#SAKNAS!
molluscs	1	5110(0.011432491139819366)	2359 (0.034)	#SAKNAS!
molossidae	1	5110(0.011432491139819366)	#SAKNAS!	2548 (0.033)
molossus	1	5110(0.011432491139819366)	#SAKNAS!	2548 (0.033)
molting	1	5110(0.011432491139819366)	2359 (0.034)	#SAKNAS!
molting	1	5110(0.011432491139819366)	2359 (0.034)	#SAKNAS!
momba瘸a	1	5110(0.011432491139819366)	#SAKNAS!	#SAKNAS!
moments	1	5110(0.011432491139819366)	#SAKNAS!	2548 (0.033)
monarch	1	5110(0.011432491139819366)	#SAKNAS!	#SAKNAS!
monastery	1	5110(0.011432491139819366)	#SAKNAS!	2548 (0.033)
monechma	1	5110(0.011432491139819366)	#SAKNAS!	2548 (0.033)
money	1	5110(0.011432491139819366)	2359 (0.034)	#SAKNAS!
money	1	5110(0.011432491139819366)	2359 (0.034)	#SAKNAS!
mongoose	1	5110(0.011432491139819366)	2359 (0.034)	#SAKNAS!
mongoose	1	5110(0.011432491139819366)	2359 (0.034)	#SAKNAS!
mongoz	1	5110(0.011432491139819366)	2359 (0.034)	#SAKNAS!
mongoz	1	5110(0.011432491139819366)	2359 (0.034)	#SAKNAS!
monitor	1	5110(0.011432491139819366)	2359 (0.034)	#SAKNAS!
monitor	1	5110(0.011432491139819366)	2359 (0.034)	#SAKNAS!
monitoring	1	1335(0.09145992911855493)	2359 (0.034)	999 (0.13)
monoacylglycerol	1	5110(0.011432491139819366)	#SAKNAS!	2548 (0.033)
monocultures	1	5110(0.011432491139819366)	2359 (0.034)	#SAKNAS!
monocultures	1	5110(0.011432491139819366)	2359 (0.034)	#SAKNAS!
monodon	1	3437(0.02286498227963873)	2359 (0.034)	#SAKNAS!
monosaccharide-enriched	1	5110(0.011432491139819366)	2359 (0.034)	#SAKNAS!
monosaccharide-enriched	1	5110(0.011432491139819366)	2359 (0.034)	#SAKNAS!
monosiga	1	5110(0.011432491139819366)	2359 (0.034)	#SAKNAS!
monosiga	1	5110(0.011432491139819366)	2359 (0.034)	#SAKNAS!
monosodium	1	5110(0.011432491139819366)	#SAKNAS!	#SAKNAS!
monospecific	1	5110(0.011432491139819366)	#SAKNAS!	2548 (0.033)
montane	1	5110(0.011432491139819366)	2359 (0.034)	#SAKNAS!
montane	1	5110(0.011432491139819366)	2359 (0.034)	#SAKNAS!
montenegro	1	5110(0.011432491139819366)	#SAKNAS!	#SAKNAS!
month-old	1	5110(0.011432491139819366)	#SAKNAS!	2548 (0.033)
montoro	1	5110(0.011432491139819366)	#SAKNAS!	#SAKNAS!
montreal	1	5110(0.011432491139819366)	#SAKNAS!	2548 (0.033)

mood	1	3437(0.0228649822796387З)	2359 (0.034)	2548 (0.033)
moor	1	5110(0.011432491139819366)	#SAKNAS!	2548 (0.033)
moquini	1	5110(0.011432491139819366)	2359 (0.034)	#SAKNAS!
moquini	1	5110(0.011432491139819366)	2359 (0.034)	#SAKNAS!
morbidities	1	5110(0.011432491139819366)	#SAKNAS!	#SAKNAS!
morhua	1	5110(0.011432491139819366)	#SAKNAS!	2548 (0.033)
mori	1	2693(0.0342974734194581)	2359 (0.034)	2548 (0.033)
moringa	1	5110(0.011432491139819366)	#SAKNAS!	#SAKNAS!
morio	1	5110(0.011432491139819366)	#SAKNAS!	2548 (0.033)
morning	1	5110(0.011432491139819366)	#SAKNAS!	#SAKNAS!
morosus	1	5110(0.011432491139819366)	2359 (0.034)	#SAKNAS!
morosus	1	5110(0.011432491139819366)	2359 (0.034)	#SAKNAS!
morphogenetic	1	5110(0.011432491139819366)	2359 (0.034)	#SAKNAS!
morphogenetic	1	5110(0.011432491139819366)	2359 (0.034)	#SAKNAS!
morphological	1	2693(0.0342974734194581)	2359 (0.034)	1668 (0.066)
morphologically	1	5110(0.011432491139819366)	2359 (0.034)	#SAKNAS!
morphologically	1	5110(0.011432491139819366)	2359 (0.034)	#SAKNAS!
morphometrics	1	5110(0.011432491139819366)	#SAKNAS!	2548 (0.033)
morphometry	1	5110(0.011432491139819366)	2359 (0.034)	#SAKNAS!
morphometry	1	5110(0.011432491139819366)	2359 (0.034)	#SAKNAS!
morrope	1	5110(0.011432491139819366)	2359 (0.034)	#SAKNAS!
morrope	1	5110(0.011432491139819366)	2359 (0.034)	#SAKNAS!
mosaic	1	5110(0.011432491139819366)	#SAKNAS!	#SAKNAS!
moscow	1	5110(0.011432491139819366)	2359 (0.034)	#SAKNAS!
moscow	1	5110(0.011432491139819366)	2359 (0.034)	#SAKNAS!
mosquito	1	5110(0.011432491139819366)	#SAKNAS!	2548 (0.033)
mossambicus	1	5110(0.011432491139819366)	#SAKNAS!	2548 (0.033)
motherhood	1	3437(0.0228649822796387З)	2359 (0.034)	2548 (0.033)
mothers<acute	1	5110(0.011432491139819366)	#SAKNAS!	#SAKNAS!
mother-young	1	3437(0.0228649822796387З)	2359 (0.034)	2548 (0.033)
motion	1	3437(0.0228649822796387З)	2359 (0.034)	#SAKNAS!
motivation	1	1335(0.09145992911855493)	2359 (0.034)	999 (0.13)
motivational	1	3437(0.0228649822796387З)	2359 (0.034)	2548 (0.033)
motivations	1	5110(0.011432491139819366)	#SAKNAS!	2548 (0.033)
motives	1	5110(0.011432491139819366)	#SAKNAS!	2548 (0.033)
moukalabense	1	5110(0.011432491139819366)	#SAKNAS!	2548 (0.033)
moult	1	5110(0.011432491139819366)	#SAKNAS!	2548 (0.033)
moulting	1	5110(0.011432491139819366)	2359 (0.034)	#SAKNAS!
moulting	1	5110(0.011432491139819366)	2359 (0.034)	#SAKNAS!
mountain	1	1678(0.0685949468389162)	2359 (0.034)	1233 (0.1)
mountains	1	5110(0.011432491139819366)	#SAKNAS!	2548 (0.033)
mousebirds	1	5110(0.011432491139819366)	#SAKNAS!	2548 (0.033)
mouth-care	1	5110(0.011432491139819366)	2359 (0.034)	#SAKNAS!

mouth-care	1	5110(0.011432491139819366)	2359 (0.034)	#SAKNAS!
mouthfeel	1	5110(0.011432491139819366)	#SAKNAS!	2548 (0.033)
mouthful	1	5110(0.011432491139819366)	#SAKNAS!	2548 (0.033)
mouthpart	1	5110(0.011432491139819366)	#SAKNAS!	2548 (0.033)
mouthparts	1	5110(0.011432491139819366)	#SAKNAS!	2548 (0.033)
mouths	1	2693(0.0342974734194581)	2359 (0.034)	1668 (0.066)
mouth-washings	1	5110(0.011432491139819366)	2359 (0.034)	#SAKNAS!
mouth-washings	1	5110(0.011432491139819366)	2359 (0.034)	#SAKNAS!
move	1	5110(0.011432491139819366)	2359 (0.034)	#SAKNAS!
move	1	5110(0.011432491139819366)	2359 (0.034)	#SAKNAS!
movements	1	5110(0.011432491139819366)	#SAKNAS!	2548 (0.033)
mozambique	1	5110(0.011432491139819366)	2359 (0.034)	#SAKNAS!
mozambique	1	5110(0.011432491139819366)	2359 (0.034)	#SAKNAS!
mri	1	5110(0.011432491139819366)	#SAKNAS!	2548 (0.033)
mrigala	1	5110(0.011432491139819366)	2359 (0.034)	#SAKNAS!
mrigala	1	5110(0.011432491139819366)	2359 (0.034)	#SAKNAS!
mri-visible	1	5110(0.011432491139819366)	#SAKNAS!	2548 (0.033)
mrna-1273	1	5110(0.011432491139819366)	#SAKNAS!	#SAKNAS!
mros	1	5110(0.011432491139819366)	#SAKNAS!	2548 (0.033)
ms	1	5110(0.011432491139819366)	#SAKNAS!	2548 (0.033)
msbi	1	5110(0.011432491139819366)	#SAKNAS!	#SAKNAS!
msi	1	5110(0.011432491139819366)	#SAKNAS!	#SAKNAS!
mt	1	5110(0.011432491139819366)	#SAKNAS!	2548 (0.033)
mtdna	1	5110(0.011432491139819366)	#SAKNAS!	2548 (0.033)
mtrr	1	5110(0.011432491139819366)	#SAKNAS!	2548 (0.033)
muc5b	1	5110(0.011432491139819366)	#SAKNAS!	#SAKNAS!
mucosectomy	1	5110(0.011432491139819366)	#SAKNAS!	#SAKNAS!
mucss	1	5110(0.011432491139819366)	#SAKNAS!	#SAKNAS!
mucticentric	1	5110(0.011432491139819366)	#SAKNAS!	2548 (0.033)
mucus	1	5110(0.011432491139819366)	2359 (0.034)	#SAKNAS!
mucus	1	5110(0.011432491139819366)	2359 (0.034)	#SAKNAS!
mud	1	5110(0.011432491139819366)	2359 (0.034)	#SAKNAS!
mud	1	5110(0.011432491139819366)	2359 (0.034)	#SAKNAS!
mudflats	1	5110(0.011432491139819366)	2359 (0.034)	#SAKNAS!
mudflats	1	5110(0.011432491139819366)	2359 (0.034)	#SAKNAS!
muell	1	5110(0.011432491139819366)	#SAKNAS!	#SAKNAS!
muga	1	5110(0.011432491139819366)	2359 (0.034)	#SAKNAS!
muga	1	5110(0.011432491139819366)	2359 (0.034)	#SAKNAS!
mugil	1	5110(0.011432491139819366)	2359 (0.034)	#SAKNAS!
mugil	1	5110(0.011432491139819366)	2359 (0.034)	#SAKNAS!
mulatta	1	5110(0.011432491139819366)	#SAKNAS!	2548 (0.033)
mule	1	5110(0.011432491139819366)	2359 (0.034)	#SAKNAS!
mule	1	5110(0.011432491139819366)	2359 (0.034)	#SAKNAS!

mullus	1	5110(0.011432491139819366)	2359 (0.034)	#SAKNAS!
mullus	1	5110(0.011432491139819366)	2359 (0.034)	#SAKNAS!
multiagent	1	5110(0.011432491139819366)	#SAKNAS!	2548 (0.033)
multi-approach	1	5110(0.011432491139819366)	#SAKNAS!	#SAKNAS!
multi-center	1	1678(0.0685949468389162)	2359 (0.034)	1668 (0.066)
multi-centre	1	2693(0.0342974734194581)	2359 (0.034)	1668 (0.066)
multicentric	1	3437(0.02286498227963873)	2359 (0.034)	2548 (0.033)
multi-channelization	1	5110(0.011432491139819366)	#SAKNAS!	2548 (0.033)
multi-comorbidities	1	5110(0.011432491139819366)	#SAKNAS!	#SAKNAS!
multi-component	1	5110(0.011432491139819366)	2359 (0.034)	#SAKNAS!
multi-component	1	5110(0.011432491139819366)	2359 (0.034)	#SAKNAS!
multidisciplinar	1	5110(0.011432491139819366)	2359 (0.034)	#SAKNAS!
multidisciplinar	1	5110(0.011432491139819366)	2359 (0.034)	#SAKNAS!
multi-domain	1	5110(0.011432491139819366)	#SAKNAS!	#SAKNAS!
multiethnic	1	5110(0.011432491139819366)	2359 (0.034)	#SAKNAS!
multiethnic	1	5110(0.011432491139819366)	2359 (0.034)	#SAKNAS!
multi-ethnic	1	5110(0.011432491139819366)	#SAKNAS!	#SAKNAS!
multifactorial	1	5110(0.011432491139819366)	2359 (0.034)	#SAKNAS!
multifactorial	1	5110(0.011432491139819366)	2359 (0.034)	#SAKNAS!
multi-fibre	1	5110(0.011432491139819366)	2359 (0.034)	#SAKNAS!
multi-fibre	1	5110(0.011432491139819366)	2359 (0.034)	#SAKNAS!
multiflorum	1	5110(0.011432491139819366)	#SAKNAS!	2548 (0.033)
multi-fundoplication	1	5110(0.011432491139819366)	#SAKNAS!	2548 (0.033)
multigenerational	1	5110(0.011432491139819366)	#SAKNAS!	2548 (0.033)
multi-locus	1	5110(0.011432491139819366)	#SAKNAS!	2548 (0.033)
multi-modal	1	5110(0.011432491139819366)	2359 (0.034)	#SAKNAS!
multi-modal	1	5110(0.011432491139819366)	2359 (0.034)	#SAKNAS!
multimodality	1	5110(0.011432491139819366)	#SAKNAS!	2548 (0.033)
multi-morbid	1	5110(0.011432491139819366)	2359 (0.034)	#SAKNAS!
multi-morbid	1	5110(0.011432491139819366)	2359 (0.034)	#SAKNAS!
multinational	1	3437(0.02286498227963873)	2359 (0.034)	2548 (0.033)
multiorgan	1	5110(0.011432491139819366)	#SAKNAS!	#SAKNAS!
multi-parametric	1	5110(0.011432491139819366)	#SAKNAS!	#SAKNAS!
multiparous	1	5110(0.011432491139819366)	#SAKNAS!	#SAKNAS!
multi-phasic	1	5110(0.011432491139819366)	2359 (0.034)	#SAKNAS!
multi-phasic	1	5110(0.011432491139819366)	2359 (0.034)	#SAKNAS!
multiple-visit	1	5110(0.011432491139819366)	#SAKNAS!	#SAKNAS!
multi-proxy	1	5110(0.011432491139819366)	#SAKNAS!	#SAKNAS!
multi-scale	1	5110(0.011432491139819366)	#SAKNAS!	2548 (0.033)
multisector	1	5110(0.011432491139819366)	#SAKNAS!	2548 (0.033)
multi-site	1	5110(0.011432491139819366)	#SAKNAS!	2548 (0.033)
multistage	1	5110(0.011432491139819366)	2359 (0.034)	#SAKNAS!
multistage	1	5110(0.011432491139819366)	2359 (0.034)	#SAKNAS!

multistrain	1	5110(0.011432491139819366)	2359 (0.034)	#SAKNAS!
multistrain	1	5110(0.011432491139819366)	2359 (0.034)	#SAKNAS!
multisystemic	1	5110(0.011432491139819366)	#SAKNAS!	#SAKNAS!
mummy	1	5110(0.011432491139819366)	#SAKNAS!	#SAKNAS!
mundgesundheitsstudie	1	5110(0.011432491139819366)	#SAKNAS!	2548 (0.033)
municipal	1	5110(0.011432491139819366)	#SAKNAS!	#SAKNAS!
muricatum	1	5110(0.011432491139819366)	#SAKNAS!	2548 (0.033)
murrill	1	5110(0.011432491139819366)	#SAKNAS!	#SAKNAS!
muscarinic	1	5110(0.011432491139819366)	2359 (0.034)	#SAKNAS!
muscarinic	1	5110(0.011432491139819366)	2359 (0.034)	#SAKNAS!
mushroom	1	5110(0.011432491139819366)	#SAKNAS!	#SAKNAS!
mushrooms	1	5110(0.011432491139819366)	#SAKNAS!	2548 (0.033)
music	1	5110(0.011432491139819366)	#SAKNAS!	2548 (0.033)
musical	1	5110(0.011432491139819366)	#SAKNAS!	2548 (0.033)
musk	1	5110(0.011432491139819366)	#SAKNAS!	2548 (0.033)
mustelidae	1	5110(0.011432491139819366)	2359 (0.034)	#SAKNAS!
mustelidae	1	5110(0.011432491139819366)	2359 (0.034)	#SAKNAS!
mutans-streptococci	1	5110(0.011432491139819366)	2359 (0.034)	#SAKNAS!
mutans-streptococci	1	5110(0.011432491139819366)	2359 (0.034)	#SAKNAS!
mutant	1	5110(0.011432491139819366)	#SAKNAS!	#SAKNAS!
mutants	1	5110(0.011432491139819366)	2359 (0.034)	#SAKNAS!
mutants	1	5110(0.011432491139819366)	2359 (0.034)	#SAKNAS!
mutation	1	2693(0.0342974734194581)	2359 (0.034)	2548 (0.033)
mutations	1	5110(0.011432491139819366)	#SAKNAS!	2548 (0.033)
mutton	1	5110(0.011432491139819366)	#SAKNAS!	2548 (0.033)
mutualistic	1	3437(0.02286498227963873)	2359 (0.034)	2548 (0.033)
myanmar	1	5110(0.011432491139819366)	#SAKNAS!	2548 (0.033)
mycobiome	1	5110(0.011432491139819366)	#SAKNAS!	2548 (0.033)
mycostop	1	5110(0.011432491139819366)	#SAKNAS!	#SAKNAS!
myelin	1	5110(0.011432491139819366)	2359 (0.034)	#SAKNAS!
myelin	1	5110(0.011432491139819366)	2359 (0.034)	#SAKNAS!
myelinolysis	1	5110(0.011432491139819366)	2359 (0.034)	#SAKNAS!
myelinolysis	1	5110(0.011432491139819366)	2359 (0.034)	#SAKNAS!
myeloma	1	5110(0.011432491139819366)	#SAKNAS!	#SAKNAS!
myelopathy	1	3437(0.02286498227963873)	2359 (0.034)	#SAKNAS!
myeloperoxidase	1	3437(0.02286498227963873)	2359 (0.034)	#SAKNAS!
myocutaneous	1	5110(0.011432491139819366)	2359 (0.034)	#SAKNAS!
myocutaneous	1	5110(0.011432491139819366)	2359 (0.034)	#SAKNAS!
myodegeneration	1	5110(0.011432491139819366)	2359 (0.034)	#SAKNAS!
myodegeneration	1	5110(0.011432491139819366)	2359 (0.034)	#SAKNAS!
myopathies	1	5110(0.011432491139819366)	#SAKNAS!	2548 (0.033)
myopathy	1	5110(0.011432491139819366)	#SAKNAS!	#SAKNAS!
myopia	1	5110(0.011432491139819366)	#SAKNAS!	#SAKNAS!

myotonic	1	5110(0.011432491139819366)	#SAKNAS!	2548 (0.033)
myrmecophagy	1	5110(0.011432491139819366)	2359 (0.034)	#SAKNAS!
myrmecophagy	1	5110(0.011432491139819366)	2359 (0.034)	#SAKNAS!
myrsinaceae	1	5110(0.011432491139819366)	2359 (0.034)	#SAKNAS!
myrsinaceae	1	5110(0.011432491139819366)	2359 (0.034)	#SAKNAS!
myrsine	1	5110(0.011432491139819366)	2359 (0.034)	#SAKNAS!
myrsine	1	5110(0.011432491139819366)	2359 (0.034)	#SAKNAS!
myself	1	5110(0.011432491139819366)	#SAKNAS!	2548 (0.033)
mysore	1	5110(0.011432491139819366)	2359 (0.034)	#SAKNAS!
mysore	1	5110(0.011432491139819366)	2359 (0.034)	#SAKNAS!
myth	1	2693(0.0342974734194581)	2359 (0.034)	1668 (0.066)
mytilidae	1	5110(0.011432491139819366)	2359 (0.034)	#SAKNAS!
mytilidae	1	5110(0.011432491139819366)	2359 (0.034)	#SAKNAS!
n-6	1	5110(0.011432491139819366)	#SAKNAS!	2548 (0.033)
nabuccod	1	5110(0.011432491139819366)	#SAKNAS!	2548 (0.033)
n-acetylglucosamine	1	5110(0.011432491139819366)	2359 (0.034)	#SAKNAS!
n-acetylglucosamine	1	5110(0.011432491139819366)	2359 (0.034)	#SAKNAS!
nacional	1	5110(0.011432491139819366)	#SAKNAS!	2548 (0.033)
nacl	1	5110(0.011432491139819366)	#SAKNAS!	#SAKNAS!
nacl-stressed	1	5110(0.011432491139819366)	#SAKNAS!	2548 (0.033)
n-acylated	1	5110(0.011432491139819366)	#SAKNAS!	2548 (0.033)
nadph	1	5110(0.011432491139819366)	2359 (0.034)	#SAKNAS!
nadph	1	5110(0.011432491139819366)	2359 (0.034)	#SAKNAS!
nagahama	1	5110(0.011432491139819366)	#SAKNAS!	2548 (0.033)
nagasaki	1	5110(0.011432491139819366)	#SAKNAS!	#SAKNAS!
nahrungsangebotes	1	5110(0.011432491139819366)	2359 (0.034)	#SAKNAS!
nahrungsangebotes	1	5110(0.011432491139819366)	2359 (0.034)	#SAKNAS!
nairobi	1	5110(0.011432491139819366)	#SAKNAS!	2548 (0.033)
nakalanga	1	5110(0.011432491139819366)	2359 (0.034)	#SAKNAS!
nakalanga	1	5110(0.011432491139819366)	2359 (0.034)	#SAKNAS!
namibia	1	5110(0.011432491139819366)	2359 (0.034)	#SAKNAS!
namibia	1	5110(0.011432491139819366)	2359 (0.034)	#SAKNAS!
nano	1	5110(0.011432491139819366)	#SAKNAS!	#SAKNAS!
nanocomplex-based	1	5110(0.011432491139819366)	#SAKNAS!	#SAKNAS!
nanocrystals	1	5110(0.011432491139819366)	#SAKNAS!	#SAKNAS!
nanoemulgel	1	5110(0.011432491139819366)	#SAKNAS!	#SAKNAS!
nanoflagellates	1	5110(0.011432491139819366)	2359 (0.034)	#SAKNAS!
nanoflagellates	1	5110(0.011432491139819366)	2359 (0.034)	#SAKNAS!
nanoparticle	1	5110(0.011432491139819366)	#SAKNAS!	#SAKNAS!
nanophotosensitizer	1	5110(0.011432491139819366)	#SAKNAS!	#SAKNAS!
nanoplastics	1	5110(0.011432491139819366)	#SAKNAS!	#SAKNAS!
napus	1	5110(0.011432491139819366)	#SAKNAS!	2548 (0.033)
narcotic	1	5110(0.011432491139819366)	#SAKNAS!	2548 (0.033)

narrative	1	2693(0.0342974734194581)	2359 (0.034)	#SAKNAS!
nasalis	1	5110(0.011432491139819366)	#SAKNAS!	2548 (0.033)
nash	1	5110(0.011432491139819366)	#SAKNAS!	#SAKNAS!
nasiriyah	1	5110(0.011432491139819366)	#SAKNAS!	#SAKNAS!
nasuta	1	5110(0.011432491139819366)	2359 (0.034)	#SAKNAS!
nasuta	1	5110(0.011432491139819366)	2359 (0.034)	#SAKNAS!
nation	1	2693(0.0342974734194581)	2359 (0.034)	1668 (0.066)
nationality	1	5110(0.011432491139819366)	#SAKNAS!	#SAKNAS!
nation-based	1	5110(0.011432491139819366)	#SAKNAS!	#SAKNAS!
nation-wide	1	5110(0.011432491139819366)	#SAKNAS!	#SAKNAS!
native-american	1	5110(0.011432491139819366)	2359 (0.034)	#SAKNAS!
native-american	1	5110(0.011432491139819366)	2359 (0.034)	#SAKNAS!
natives	1	5110(0.011432491139819366)	#SAKNAS!	2548 (0.033)
nativity	1	5110(0.011432491139819366)	#SAKNAS!	2548 (0.033)
naturally	1	3437(0.02286498227963873)	2359 (0.034)	2548 (0.033)
naturally-occurring	1	5110(0.011432491139819366)	2359 (0.034)	#SAKNAS!
naturally-occurring	1	5110(0.011432491139819366)	2359 (0.034)	#SAKNAS!
nauplii	1	3437(0.02286498227963873)	2359 (0.034)	2548 (0.033)
nauseosus	1	5110(0.011432491139819366)	2359 (0.034)	#SAKNAS!
nauseosus	1	5110(0.011432491139819366)	2359 (0.034)	#SAKNAS!
navajo	1	5110(0.011432491139819366)	#SAKNAS!	2548 (0.033)
navigating	1	5110(0.011432491139819366)	#SAKNAS!	#SAKNAS!
nayarit	1	5110(0.011432491139819366)	#SAKNAS!	2548 (0.033)
near	1	5110(0.011432491139819366)	#SAKNAS!	2548 (0.033)
near-total	1	5110(0.011432491139819366)	2359 (0.034)	#SAKNAS!
near-total	1	5110(0.011432491139819366)	2359 (0.034)	#SAKNAS!
nebularum	1	5110(0.011432491139819366)	#SAKNAS!	2548 (0.033)
necessary	1	2693(0.0342974734194581)	2359 (0.034)	1668 (0.066)
necessity	1	5110(0.011432491139819366)	2359 (0.034)	#SAKNAS!
necessity	1	5110(0.011432491139819366)	2359 (0.034)	#SAKNAS!
necrolysis	1	5110(0.011432491139819366)	2359 (0.034)	#SAKNAS!
necrolysis	1	5110(0.011432491139819366)	2359 (0.034)	#SAKNAS!
necrotising	1	2693(0.0342974734194581)	2359 (0.034)	2548 (0.033)
nectar-feeding	1	5110(0.011432491139819366)	2359 (0.034)	#SAKNAS!
nectar-feeding	1	5110(0.011432491139819366)	2359 (0.034)	#SAKNAS!
nectar-foraging	1	5110(0.011432491139819366)	#SAKNAS!	2548 (0.033)
nectarivore	1	5110(0.011432491139819366)	#SAKNAS!	#SAKNAS!
needing	1	5110(0.011432491139819366)	#SAKNAS!	2548 (0.033)
needs-related	1	5110(0.011432491139819366)	2359 (0.034)	#SAKNAS!
needs-related	1	5110(0.011432491139819366)	2359 (0.034)	#SAKNAS!
negative	1	2214(0.04572996455927746)	2359 (0.034)	1668 (0.066)
neglect	1	5110(0.011432491139819366)	2359 (0.034)	#SAKNAS!
neglect	1	5110(0.011432491139819366)	2359 (0.034)	#SAKNAS!

negligible	1 5110(0.011432491139819366)	2359 (0.034)	#SAKNAS!
negligible	1 5110(0.011432491139819366)	2359 (0.034)	#SAKNAS!
negr1	1 5110(0.011432491139819366)	2359 (0.034)	#SAKNAS!
negr1	1 5110(0.011432491139819366)	2359 (0.034)	#SAKNAS!
neighborhood	1 5110(0.011432491139819366)	2359 (0.034)	#SAKNAS!
neighborhood	1 5110(0.011432491139819366)	2359 (0.034)	#SAKNAS!
neither	1 5110(0.011432491139819366)	2359 (0.034)	#SAKNAS!
neither	1 5110(0.011432491139819366)	2359 (0.034)	#SAKNAS!
nemaline	1 5110(0.011432491139819366)	#SAKNAS!	#SAKNAS!
nematode	1 5110(0.011432491139819366)	#SAKNAS!	2548 (0.033)
nematodes	1 3437(0.02286498227963873)	2359 (0.034)	2548 (0.033)
nemipteridae	1 5110(0.011432491139819366)	2359 (0.034)	#SAKNAS!
nemipteridae	1 5110(0.011432491139819366)	2359 (0.034)	#SAKNAS!
neo-adjuvant	1 2214(0.04572996455927746)	2359 (0.034)	1233 (0.1)
neocalanus	1 5110(0.011432491139819366)	2359 (0.034)	#SAKNAS!
neocalanus	1 5110(0.011432491139819366)	2359 (0.034)	#SAKNAS!
neocaridina	1 5110(0.011432491139819366)	#SAKNAS!	2548 (0.033)
neoclerodane	1 5110(0.011432491139819366)	2359 (0.034)	#SAKNAS!
neoclerodane	1 5110(0.011432491139819366)	2359 (0.034)	#SAKNAS!
neoeat-bottle-feeding	1 5110(0.011432491139819366)	#SAKNAS!	2548 (0.033)
neolithic	1 2214(0.04572996455927746)	2359 (0.034)	2548 (0.033)
neo-marxian	1 5110(0.011432491139819366)	#SAKNAS!	2548 (0.033)
neo-mom	1 5110(0.011432491139819366)	#SAKNAS!	2548 (0.033)
neon	1 5110(0.011432491139819366)	#SAKNAS!	#SAKNAS!
neonate	1 5110(0.011432491139819366)	2359 (0.034)	#SAKNAS!
neonate	1 5110(0.011432491139819366)	2359 (0.034)	#SAKNAS!
neoplasm	1 5110(0.011432491139819366)	#SAKNAS!	2548 (0.033)
neoplasms	1 3437(0.02286498227963873)	2359 (0.034)	#SAKNAS!
nepalese	1 5110(0.011432491139819366)	#SAKNAS!	2548 (0.033)
nepali	1 3437(0.02286498227963873)	2359 (0.034)	2548 (0.033)
nephrectomized	1 5110(0.011432491139819366)	#SAKNAS!	2548 (0.033)
nephridial	1 5110(0.011432491139819366)	#SAKNAS!	2548 (0.033)
nephritis	1 5110(0.011432491139819366)	2359 (0.034)	#SAKNAS!
nephritis	1 5110(0.011432491139819366)	2359 (0.034)	#SAKNAS!
nephropathy	1 3437(0.02286498227963873)	2359 (0.034)	#SAKNAS!
nephropathy-related	1 5110(0.011432491139819366)	#SAKNAS!	2548 (0.033)
nephrops	1 5110(0.011432491139819366)	#SAKNAS!	2548 (0.033)
nephrothrix	1 5110(0.011432491139819366)	#SAKNAS!	2548 (0.033)
nephrotic	1 5110(0.011432491139819366)	#SAKNAS!	2548 (0.033)
nerveless	1 5110(0.011432491139819366)	#SAKNAS!	#SAKNAS!
nervion	1 5110(0.011432491139819366)	2359 (0.034)	#SAKNAS!
nervion	1 5110(0.011432491139819366)	2359 (0.034)	#SAKNAS!
nervous	1 5110(0.011432491139819366)	#SAKNAS!	2548 (0.033)

nesfatin-1	1	2214(0.04572996455927746)	2359 (0.034)	1668 (0.066)
nest	1	5110(0.011432491139819366)	#SAKNAS!	2548 (0.033)
nesting	1	5110(0.011432491139819366)	2359 (0.034)	#SAKNAS!
nesting	1	5110(0.011432491139819366)	2359 (0.034)	#SAKNAS!
nestle	1	5110(0.011432491139819366)	#SAKNAS!	2548 (0.033)
nestlings	1	5110(0.011432491139819366)	2359 (0.034)	#SAKNAS!
nestlings	1	5110(0.011432491139819366)	2359 (0.034)	#SAKNAS!
nests	1	5110(0.011432491139819366)	2359 (0.034)	#SAKNAS!
nests	1	5110(0.011432491139819366)	2359 (0.034)	#SAKNAS!
netherlands	1	5110(0.011432491139819366)	2359 (0.034)	#SAKNAS!
netherlands	1	5110(0.011432491139819366)	2359 (0.034)	#SAKNAS!
net-pd	1	5110(0.011432491139819366)	#SAKNAS!	2548 (0.033)
neuogenic	1	5110(0.011432491139819366)	2359 (0.034)	#SAKNAS!
neuogenic	1	5110(0.011432491139819366)	2359 (0.034)	#SAKNAS!
neurobeachin	1	5110(0.011432491139819366)	2359 (0.034)	#SAKNAS!
neurobeachin	1	5110(0.011432491139819366)	2359 (0.034)	#SAKNAS!
neurobehavioral	1	5110(0.011432491139819366)	#SAKNAS!	2548 (0.033)
neurobiological	1	3437(0.02286498227963873)	2359 (0.034)	2548 (0.033)
neurochemistry	1	5110(0.011432491139819366)	2359 (0.034)	#SAKNAS!
neurochemistry	1	5110(0.011432491139819366)	2359 (0.034)	#SAKNAS!
neurocircuit	1	5110(0.011432491139819366)	#SAKNAS!	#SAKNAS!
neurocritically	1	5110(0.011432491139819366)	#SAKNAS!	#SAKNAS!
neurodegeneration	1	5110(0.011432491139819366)	#SAKNAS!	#SAKNAS!
neurodegenerative	1	2214(0.04572996455927746)	2359 (0.034)	2548 (0.033)
neurodevelopment	1	5110(0.011432491139819366)	#SAKNAS!	2548 (0.033)
neurodevelopmental	1	2693(0.0342974734194581)	2359 (0.034)	#SAKNAS!
neuroelectric	1	5110(0.011432491139819366)	#SAKNAS!	2548 (0.033)
neuroethology	1	5110(0.011432491139819366)	2359 (0.034)	#SAKNAS!
neuroethology	1	5110(0.011432491139819366)	2359 (0.034)	#SAKNAS!
neurofilament	1	5110(0.011432491139819366)	#SAKNAS!	#SAKNAS!
neurohormonal	1	5110(0.011432491139819366)	2359 (0.034)	#SAKNAS!
neurohormonal	1	5110(0.011432491139819366)	2359 (0.034)	#SAKNAS!
neurointensivists	1	5110(0.011432491139819366)	#SAKNAS!	#SAKNAS!
neurologists	1	5110(0.011432491139819366)	#SAKNAS!	#SAKNAS!
neuromodulation	1	5110(0.011432491139819366)	#SAKNAS!	2548 (0.033)
neuron-specific	1	5110(0.011432491139819366)	#SAKNAS!	#SAKNAS!
neuropathy	1	5110(0.011432491139819366)	2359 (0.034)	#SAKNAS!
neuropathy	1	5110(0.011432491139819366)	2359 (0.034)	#SAKNAS!
neuropeptides	1	5110(0.011432491139819366)	#SAKNAS!	2548 (0.033)
neuropsychiatric	1	5110(0.011432491139819366)	#SAKNAS!	#SAKNAS!
neurorehabilitation	1	3437(0.02286498227963873)	2359 (0.034)	#SAKNAS!
neurostimulation	1	5110(0.011432491139819366)	#SAKNAS!	2548 (0.033)
neurostimulator	1	5110(0.011432491139819366)	#SAKNAS!	2548 (0.033)

neurosurgical	1 5110(0.011432491139819366)	2359 (0.034)	#SAKNAS!
neurosurgical	1 5110(0.011432491139819366)	2359 (0.034)	#SAKNAS!
neurotized	1 5110(0.011432491139819366)	#SAKNAS!	#SAKNAS!
neurotoxicity	1 5110(0.011432491139819366)	#SAKNAS!	#SAKNAS!
neutrophil	1 2214(0.04572996455927746)	2359 (0.034)	2548 (0.033)
neutrophils	1 5110(0.011432491139819366)	#SAKNAS!	2548 (0.033)
newborn	1 5110(0.011432491139819366)	#SAKNAS!	2548 (0.033)
new-england	1 5110(0.011432491139819366)	2359 (0.034)	#SAKNAS!
new-england	1 5110(0.011432491139819366)	2359 (0.034)	#SAKNAS!
newly-hatched	1 5110(0.011432491139819366)	#SAKNAS!	#SAKNAS!
new-onset	1 5110(0.011432491139819366)	#SAKNAS!	#SAKNAS!
new-zealand	1 5110(0.011432491139819366)	2359 (0.034)	#SAKNAS!
new-zealand	1 5110(0.011432491139819366)	2359 (0.034)	#SAKNAS!
next-generation	1 5110(0.011432491139819366)	#SAKNAS!	2548 (0.033)
nhanes2009-2014	1 5110(0.011432491139819366)	#SAKNAS!	#SAKNAS!
nhanes-based	1 5110(0.011432491139819366)	#SAKNAS!	#SAKNAS!
nhanes-iii	1 5110(0.011432491139819366)	2359 (0.034)	#SAKNAS!
nhanes-iii	1 5110(0.011432491139819366)	2359 (0.034)	#SAKNAS!
nhhr	1 5110(0.011432491139819366)	#SAKNAS!	#SAKNAS!
nhs	1 5110(0.011432491139819366)	#SAKNAS!	2548 (0.033)
niacin	1 5110(0.011432491139819366)	#SAKNAS!	2548 (0.033)
nicaragua	1 5110(0.011432491139819366)	#SAKNAS!	2548 (0.033)
nicaraguan	1 5110(0.011432491139819366)	2359 (0.034)	#SAKNAS!
nicaraguan	1 5110(0.011432491139819366)	2359 (0.034)	#SAKNAS!
nice	1 5110(0.011432491139819366)	#SAKNAS!	2548 (0.033)
niche	1 5110(0.011432491139819366)	2359 (0.034)	#SAKNAS!
niche	1 5110(0.011432491139819366)	2359 (0.034)	#SAKNAS!
nicobar	1 5110(0.011432491139819366)	#SAKNAS!	#SAKNAS!
nicolet	1 5110(0.011432491139819366)	#SAKNAS!	2548 (0.033)
nicotine	1 5110(0.011432491139819366)	#SAKNAS!	#SAKNAS!
nicotinic	1 5110(0.011432491139819366)	#SAKNAS!	#SAKNAS!
nidulans	1 5110(0.011432491139819366)	2359 (0.034)	#SAKNAS!
nidulans	1 5110(0.011432491139819366)	2359 (0.034)	#SAKNAS!
niemann-pick	1 5110(0.011432491139819366)	#SAKNAS!	2548 (0.033)
nigerian	1 5110(0.011432491139819366)	#SAKNAS!	#SAKNAS!
nigerians	1 5110(0.011432491139819366)	2359 (0.034)	#SAKNAS!
nigerians	1 5110(0.011432491139819366)	2359 (0.034)	#SAKNAS!
night-shift	1 5110(0.011432491139819366)	#SAKNAS!	2548 (0.033)
nigrescens	1 5110(0.011432491139819366)	2359 (0.034)	#SAKNAS!
nigrescens	1 5110(0.011432491139819366)	2359 (0.034)	#SAKNAS!
nigriceps	1 5110(0.011432491139819366)	#SAKNAS!	2548 (0.033)
nihss	1 5110(0.011432491139819366)	#SAKNAS!	2548 (0.033)
niigata	1 5110(0.011432491139819366)	#SAKNAS!	2548 (0.033)

nil	1	5110(0.011432491139819366)	#SAKNAS!	2548 (0.033)
niloticus	1	3437(0.02286498227963873)	2359 (0.034)	2548 (0.033)
nine	1	5110(0.011432491139819366)	#SAKNAS!	#SAKNAS!
nine-year	1	3437(0.02286498227963873)	2359 (0.034)	2548 (0.033)
nino	1	5110(0.011432491139819366)	#SAKNAS!	#SAKNAS!
nipple	1	5110(0.011432491139819366)	#SAKNAS!	2548 (0.033)
nippon	1	5110(0.011432491139819366)	#SAKNAS!	2548 (0.033)
nisami	1	5110(0.011432491139819366)	#SAKNAS!	#SAKNAS!
nissen-fundoplication	1	5110(0.011432491139819366)	#SAKNAS!	#SAKNAS!
nissen-hill	1	5110(0.011432491139819366)	#SAKNAS!	2548 (0.033)
nitidulidae	1	5110(0.011432491139819366)	#SAKNAS!	#SAKNAS!
nitinol	1	5110(0.011432491139819366)	2359 (0.034)	#SAKNAS!
nitinol	1	5110(0.011432491139819366)	2359 (0.034)	#SAKNAS!
niti-s	1	5110(0.011432491139819366)	2359 (0.034)	#SAKNAS!
niti-s	1	5110(0.011432491139819366)	2359 (0.034)	#SAKNAS!
nitrate	1	5110(0.011432491139819366)	#SAKNAS!	2548 (0.033)
nitrate-rich	1	5110(0.011432491139819366)	#SAKNAS!	2548 (0.033)
nitric	1	5110(0.011432491139819366)	2359 (0.034)	#SAKNAS!
nitric	1	5110(0.011432491139819366)	2359 (0.034)	#SAKNAS!
nivalis	1	5110(0.011432491139819366)	2359 (0.034)	#SAKNAS!
nivalis	1	5110(0.011432491139819366)	2359 (0.034)	#SAKNAS!
niveatus	1	5110(0.011432491139819366)	#SAKNAS!	#SAKNAS!
njombe	1	5110(0.011432491139819366)	#SAKNAS!	#SAKNAS!
nlrp3	1	5110(0.011432491139819366)	#SAKNAS!	#SAKNAS!
nmes	1	5110(0.011432491139819366)	2359 (0.034)	#SAKNAS!
nmes	1	5110(0.011432491139819366)	2359 (0.034)	#SAKNAS!
nociceptin	1	5110(0.011432491139819366)	#SAKNAS!	#SAKNAS!
noctuid	1	5110(0.011432491139819366)	2359 (0.034)	#SAKNAS!
noctuid	1	5110(0.011432491139819366)	2359 (0.034)	#SAKNAS!
noctuidae	1	2693(0.0342974734194581)	2359 (0.034)	1668 (0.066)
node	1	5110(0.011432491139819366)	#SAKNAS!	#SAKNAS!
noises	1	5110(0.011432491139819366)	#SAKNAS!	#SAKNAS!
nomads	1	5110(0.011432491139819366)	#SAKNAS!	2548 (0.033)
nomascus	1	5110(0.011432491139819366)	#SAKNAS!	2548 (0.033)
nomenclature	1	5110(0.011432491139819366)	#SAKNAS!	2548 (0.033)
nominally	1	5110(0.011432491139819366)	2359 (0.034)	#SAKNAS!
nominally	1	5110(0.011432491139819366)	2359 (0.034)	#SAKNAS!
non-abdominal	1	5110(0.011432491139819366)	#SAKNAS!	2548 (0.033)
non-achievers	1	5110(0.011432491139819366)	2359 (0.034)	#SAKNAS!
non-achievers	1	5110(0.011432491139819366)	2359 (0.034)	#SAKNAS!
non-acute	1	5110(0.011432491139819366)	#SAKNAS!	#SAKNAS!
nonadjustable	1	5110(0.011432491139819366)	2359 (0.034)	#SAKNAS!
nonadjustable	1	5110(0.011432491139819366)	2359 (0.034)	#SAKNAS!

non-adult	1	5110(0.011432491139819366)	#SAKNAS!	2548 (0.033)
non-alcoholic	1	2214(0.04572996455927746)	2359 (0.034)	1668 (0.066)
nonbanded	1	5110(0.011432491139819366)	#SAKNAS!	2548 (0.033)
non-binary	1	5110(0.011432491139819366)	#SAKNAS!	2548 (0.033)
nonbuffered	1	5110(0.011432491139819366)	#SAKNAS!	#SAKNAS!
non-cancer	1	5110(0.011432491139819366)	2359 (0.034)	#SAKNAS!
non-cancer	1	5110(0.011432491139819366)	2359 (0.034)	#SAKNAS!
non-carious	1	5110(0.011432491139819366)	#SAKNAS!	2548 (0.033)
non-cavitated	1	5110(0.011432491139819366)	#SAKNAS!	2548 (0.033)
non-citizens	1	5110(0.011432491139819366)	#SAKNAS!	2548 (0.033)
noncommunicable	1	2214(0.04572996455927746)	2359 (0.034)	1668 (0.066)
non-competitive	1	5110(0.011432491139819366)	#SAKNAS!	2548 (0.033)
non-consumptive	1	5110(0.011432491139819366)	#SAKNAS!	2548 (0.033)
non-core	1	5110(0.011432491139819366)	#SAKNAS!	#SAKNAS!
non-deglutitive	1	5110(0.011432491139819366)	#SAKNAS!	#SAKNAS!
nondiabetic	1	5110(0.011432491139819366)	#SAKNAS!	#SAKNAS!
nondiabetics	1	5110(0.011432491139819366)	2359 (0.034)	#SAKNAS!
nondiabetics	1	5110(0.011432491139819366)	2359 (0.034)	#SAKNAS!
non-dietary	1	5110(0.011432491139819366)	#SAKNAS!	#SAKNAS!
nondirective	1	5110(0.011432491139819366)	2359 (0.034)	#SAKNAS!
nondirective	1	5110(0.011432491139819366)	2359 (0.034)	#SAKNAS!
nondisseminated	1	5110(0.011432491139819366)	2359 (0.034)	#SAKNAS!
nondisseminated	1	5110(0.011432491139819366)	2359 (0.034)	#SAKNAS!
nondistended	1	5110(0.011432491139819366)	#SAKNAS!	2548 (0.033)
nondysphagic	1	2693(0.0342974734194581)	2359 (0.034)	#SAKNAS!
non-eating	1	5110(0.011432491139819366)	#SAKNAS!	2548 (0.033)
nonelderly	1	5110(0.011432491139819366)	2359 (0.034)	#SAKNAS!
nonelderly	1	5110(0.011432491139819366)	2359 (0.034)	#SAKNAS!
non-elderly	1	5110(0.011432491139819366)	2359 (0.034)	#SAKNAS!
non-elderly	1	5110(0.011432491139819366)	2359 (0.034)	#SAKNAS!
none-mesh	1	5110(0.011432491139819366)	#SAKNAS!	2548 (0.033)
non-endoscopic	1	5110(0.011432491139819366)	2359 (0.034)	#SAKNAS!
non-endoscopic	1	5110(0.011432491139819366)	2359 (0.034)	#SAKNAS!
non-energy-restricted	1	5110(0.011432491139819366)	#SAKNAS!	#SAKNAS!
non-essential	1	5110(0.011432491139819366)	#SAKNAS!	#SAKNAS!
non-exhaustive	1	5110(0.011432491139819366)	#SAKNAS!	2548 (0.033)
non-fatal	1	5110(0.011432491139819366)	2359 (0.034)	#SAKNAS!
non-fatal	1	5110(0.011432491139819366)	2359 (0.034)	#SAKNAS!
nonforage	1	5110(0.011432491139819366)	#SAKNAS!	2548 (0.033)
non-frail	1	5110(0.011432491139819366)	2359 (0.034)	#SAKNAS!
non-frail	1	5110(0.011432491139819366)	2359 (0.034)	#SAKNAS!
non-frequent	1	5110(0.011432491139819366)	#SAKNAS!	#SAKNAS!
non-head	1	5110(0.011432491139819366)	2359 (0.034)	#SAKNAS!

non-head	1	5110(0.011432491139819366)	2359 (0.034)	#SAKNAS!
non-health	1	5110(0.011432491139819366)	#SAKNAS!	#SAKNAS!
non-high-density	1	5110(0.011432491139819366)	#SAKNAS!	#SAKNAS!
non-hispanic	1	3437(0.02286498227963873)	2359 (0.034)	2548 (0.033)
non-homeostatic	1	5110(0.011432491139819366)	#SAKNAS!	2548 (0.033)
non-honeydew	1	5110(0.011432491139819366)	2359 (0.034)	#SAKNAS!
non-honeydew	1	5110(0.011432491139819366)	2359 (0.034)	#SAKNAS!
nonhuman	1	5110(0.011432491139819366)	2359 (0.034)	#SAKNAS!
nonhuman	1	5110(0.011432491139819366)	2359 (0.034)	#SAKNAS!
non-hypertensive	1	5110(0.011432491139819366)	#SAKNAS!	2548 (0.033)
non-ignorable	1	5110(0.011432491139819366)	#SAKNAS!	#SAKNAS!
non-invasive	1	2693(0.0342974734194581)	2359 (0.034)	2548 (0.033)
non-linear	1	3437(0.02286498227963873)	2359 (0.034)	2548 (0.033)
non-malignant	1	5110(0.011432491139819366)	#SAKNAS!	#SAKNAS!
non-maori	1	5110(0.011432491139819366)	2359 (0.034)	#SAKNAS!
non-maori	1	5110(0.011432491139819366)	2359 (0.034)	#SAKNAS!
non-milk	1	5110(0.011432491139819366)	2359 (0.034)	#SAKNAS!
non-milk	1	5110(0.011432491139819366)	2359 (0.034)	#SAKNAS!
non-natural	1	5110(0.011432491139819366)	#SAKNAS!	#SAKNAS!
non-neurologically	1	5110(0.011432491139819366)	#SAKNAS!	2548 (0.033)
non-nutritive	1	5110(0.011432491139819366)	#SAKNAS!	#SAKNAS!
non-obese	1	1219(0.1028924202583743)	2359 (0.034)	694 (0.2)
non-oral	1	5110(0.011432491139819366)	#SAKNAS!	2548 (0.033)
non-orally	1	5110(0.011432491139819366)	#SAKNAS!	2548 (0.033)
non-parasitized	1	5110(0.011432491139819366)	#SAKNAS!	2548 (0.033)
nonpharmacological	1	5110(0.011432491139819366)	#SAKNAS!	#SAKNAS!
nonpulmonary	1	5110(0.011432491139819366)	2359 (0.034)	#SAKNAS!
nonpulmonary	1	5110(0.011432491139819366)	2359 (0.034)	#SAKNAS!
nonresponses	1	5110(0.011432491139819366)	#SAKNAS!	#SAKNAS!
non-robotic	1	5110(0.011432491139819366)	#SAKNAS!	#SAKNAS!
non-sarcopenic	1	5110(0.011432491139819366)	#SAKNAS!	#SAKNAS!
non-scleractinian	1	5110(0.011432491139819366)	#SAKNAS!	2548 (0.033)
nonsmokers	1	3437(0.02286498227963873)	2359 (0.034)	2548 (0.033)
non-standardized	1	5110(0.011432491139819366)	#SAKNAS!	#SAKNAS!
non-surgical	1	1111(0.11432491139819366)	2359 (0.034)	694 (0.2)
non-syndromic	1	5110(0.011432491139819366)	2359 (0.034)	#SAKNAS!
non-syndromic	1	5110(0.011432491139819366)	2359 (0.034)	#SAKNAS!
nonsystematic	1	5110(0.011432491139819366)	#SAKNAS!	2548 (0.033)
non-thrombolysed	1	5110(0.011432491139819366)	#SAKNAS!	2548 (0.033)
nontraditional	1	5110(0.011432491139819366)	2359 (0.034)	#SAKNAS!
nontraditional	1	5110(0.011432491139819366)	2359 (0.034)	#SAKNAS!
non-urate	1	5110(0.011432491139819366)	#SAKNAS!	2548 (0.033)
nonusers	1	5110(0.011432491139819366)	2359 (0.034)	#SAKNAS!

nonusers	1	5110(0.011432491139819366)	2359 (0.034)	#SAKNAS!
non-vector	1	5110(0.011432491139819366)	#SAKNAS!	2548 (0.033)
non-vegetarians	1	5110(0.011432491139819366)	#SAKNAS!	#SAKNAS!
non-working	1	5110(0.011432491139819366)	#SAKNAS!	2548 (0.033)
noorabademamasani	1	5110(0.011432491139819366)	2359 (0.034)	#SAKNAS!
noorabademamasani	1	5110(0.011432491139819366)	2359 (0.034)	#SAKNAS!
nor	1	3437(0.02286498227963873)	2359 (0.034)	2548 (0.033)
noradrenaline-induced	1	5110(0.011432491139819366)	2359 (0.034)	#SAKNAS!
noradrenaline-induced	1	5110(0.011432491139819366)	2359 (0.034)	#SAKNAS!
norethisterone	1	5110(0.011432491139819366)	#SAKNAS!	#SAKNAS!
norm	1	5110(0.011432491139819366)	#SAKNAS!	2548 (0.033)
normalcy	1	5110(0.011432491139819366)	#SAKNAS!	2548 (0.033)
normally	1	5110(0.011432491139819366)	2359 (0.034)	#SAKNAS!
normally	1	5110(0.011432491139819366)	2359 (0.034)	#SAKNAS!
normal-weight	1	1894(0.05716245569909683)	2359 (0.034)	1668 (0.066)
normal-weighted	1	5110(0.011432491139819366)	#SAKNAS!	2548 (0.033)
normoglycemic	1	5110(0.011432491139819366)	#SAKNAS!	2548 (0.033)
norsjo	1	5110(0.011432491139819366)	2359 (0.034)	#SAKNAS!
norsjo	1	5110(0.011432491139819366)	2359 (0.034)	#SAKNAS!
north-central	1	5110(0.011432491139819366)	2359 (0.034)	#SAKNAS!
north-central	1	5110(0.011432491139819366)	2359 (0.034)	#SAKNAS!
north-east	1	3437(0.02286498227963873)	2359 (0.034)	#SAKNAS!
north-eastern	1	5110(0.011432491139819366)	2359 (0.034)	#SAKNAS!
north-eastern	1	5110(0.011432491139819366)	2359 (0.034)	#SAKNAS!
north-german	1	5110(0.011432491139819366)	2359 (0.034)	#SAKNAS!
north-german	1	5110(0.011432491139819366)	2359 (0.034)	#SAKNAS!
northwest	1	2214(0.04572996455927746)	2359 (0.034)	1668 (0.066)
north-west	1	5110(0.011432491139819366)	#SAKNAS!	#SAKNAS!
northwestern	1	5110(0.011432491139819366)	2359 (0.034)	#SAKNAS!
northwestern	1	5110(0.011432491139819366)	2359 (0.034)	#SAKNAS!
norvegicus	1	3437(0.02286498227963873)	2359 (0.034)	2548 (0.033)
norway	1	1485(0.08002743797873557)	2359 (0.034)	1668 (0.066)
nosocomial	1	3437(0.02286498227963873)	2359 (0.034)	#SAKNAS!
nosological	1	5110(0.011432491139819366)	#SAKNAS!	2548 (0.033)
notes	1	5110(0.011432491139819366)	#SAKNAS!	#SAKNAS!
noteworthy	1	5110(0.011432491139819366)	#SAKNAS!	#SAKNAS!
no-tillage	1	5110(0.011432491139819366)	#SAKNAS!	#SAKNAS!
notochord	1	5110(0.011432491139819366)	2359 (0.034)	#SAKNAS!
notochord	1	5110(0.011432491139819366)	2359 (0.034)	#SAKNAS!
notoginseng	1	5110(0.011432491139819366)	2359 (0.034)	#SAKNAS!
notoginseng	1	5110(0.011432491139819366)	2359 (0.034)	#SAKNAS!
notornis-mantelli	1	5110(0.011432491139819366)	2359 (0.034)	#SAKNAS!
notornis-mantelli	1	5110(0.011432491139819366)	2359 (0.034)	#SAKNAS!

notoungulates	1	5110(0.011432491139819366)	2359 (0.034)	#SAKNAS!
notoungulates	1	5110(0.011432491139819366)	2359 (0.034)	#SAKNAS!
nouakchott	1	5110(0.011432491139819366)	#SAKNAS!	2548 (0.033)
nourish-t	1	5110(0.011432491139819366)	#SAKNAS!	2548 (0.033)
nov	1	5110(0.011432491139819366)	#SAKNAS!	2548 (0.033)
novo	1	5110(0.011432491139819366)	#SAKNAS!	#SAKNAS!
noxia	1	5110(0.011432491139819366)	#SAKNAS!	#SAKNAS!
nozoji-ato	1	5110(0.011432491139819366)	#SAKNAS!	2548 (0.033)
nozzle	1	5110(0.011432491139819366)	#SAKNAS!	#SAKNAS!
npf	1	5110(0.011432491139819366)	#SAKNAS!	#SAKNAS!
npffr2	1	3437(0.02286498227963873)	2359 (0.034)	#SAKNAS!
npy	1	2214(0.04572996455927746)	2359 (0.034)	2548 (0.033)
npy2r	1	5110(0.011432491139819366)	2359 (0.034)	#SAKNAS!
npy2r	1	5110(0.011432491139819366)	2359 (0.034)	#SAKNAS!
nq-e	1	5110(0.011432491139819366)	#SAKNAS!	#SAKNAS!
nrf2	1	5110(0.011432491139819366)	#SAKNAS!	#SAKNAS!
nrg	1	5110(0.011432491139819366)	#SAKNAS!	2548 (0.033)
nrs-2002	1	5110(0.011432491139819366)	#SAKNAS!	#SAKNAS!
nsw	1	5110(0.011432491139819366)	#SAKNAS!	#SAKNAS!
nucella-heyseana	1	5110(0.011432491139819366)	2359 (0.034)	#SAKNAS!
nucella-heyseana	1	5110(0.011432491139819366)	2359 (0.034)	#SAKNAS!
nuclear	1	2214(0.04572996455927746)	2359 (0.034)	2548 (0.033)
nuclei	1	3437(0.02286498227963873)	2359 (0.034)	2548 (0.033)
nucleobindin-2	1	5110(0.011432491139819366)	#SAKNAS!	#SAKNAS!
nude	1	5110(0.011432491139819366)	#SAKNAS!	#SAKNAS!
nunavut	1	5110(0.011432491139819366)	2359 (0.034)	#SAKNAS!
nunavut	1	5110(0.011432491139819366)	2359 (0.034)	#SAKNAS!
nunoa	1	5110(0.011432491139819366)	2359 (0.034)	#SAKNAS!
nunoa	1	5110(0.011432491139819366)	2359 (0.034)	#SAKNAS!
nurse-delivered	1	5110(0.011432491139819366)	#SAKNAS!	#SAKNAS!
nurseries	1	5110(0.011432491139819366)	#SAKNAS!	2548 (0.033)
nursing-dental	1	5110(0.011432491139819366)	2359 (0.034)	#SAKNAS!
nursing-dental	1	5110(0.011432491139819366)	2359 (0.034)	#SAKNAS!
nursing-home	1	5110(0.011432491139819366)	#SAKNAS!	2548 (0.033)
nurturance	1	5110(0.011432491139819366)	2359 (0.034)	#SAKNAS!
nurturance	1	5110(0.011432491139819366)	2359 (0.034)	#SAKNAS!
nurture	1	5110(0.011432491139819366)	#SAKNAS!	#SAKNAS!
nut	1	2693(0.0342974734194581)	2359 (0.034)	#SAKNAS!
nutilis	1	5110(0.011432491139819366)	#SAKNAS!	2548 (0.033)
nutraceutical	1	5110(0.011432491139819366)	#SAKNAS!	2548 (0.033)
nutricare	1	5110(0.011432491139819366)	#SAKNAS!	2548 (0.033)
nutricion	1	5110(0.011432491139819366)	#SAKNAS!	2548 (0.033)
nutricional	1	5110(0.011432491139819366)	#SAKNAS!	2548 (0.033)

nutrient-dense	1 5110(0.011432491139819366)	#SAKNAS!	#SAKNAS!
nutrient-dependent	1 5110(0.011432491139819366)	#SAKNAS!	2548 (0.033)
nutrient-sensing	1 5110(0.011432491139819366)	#SAKNAS!	#SAKNAS!
nutrient-specific	1 5110(0.011432491139819366)	#SAKNAS!	2548 (0.033)
nutrifrag	1 5110(0.011432491139819366)	#SAKNAS!	#SAKNAS!
nutrigenetic	1 5110(0.011432491139819366)	2359 (0.034)	#SAKNAS!
nutrigenetic	1 5110(0.011432491139819366)	2359 (0.034)	#SAKNAS!
nutrigenomics	1 5110(0.011432491139819366)	#SAKNAS!	2548 (0.033)
nutri-hab	1 5110(0.011432491139819366)	#SAKNAS!	2548 (0.033)
nutri-jelly	1 5110(0.011432491139819366)	#SAKNAS!	2548 (0.033)
nutrinet-salud	1 5110(0.011432491139819366)	#SAKNAS!	2548 (0.033)
nutrinet-sante	1 5110(0.011432491139819366)	#SAKNAS!	#SAKNAS!
nutri-score	1 5110(0.011432491139819366)	#SAKNAS!	2548 (0.033)
nutrisep	1 5110(0.011432491139819366)	#SAKNAS!	#SAKNAS!
nutritional-related	1 5110(0.011432491139819366)	#SAKNAS!	#SAKNAS!
nutritional-requirements	1 5110(0.011432491139819366)	2359 (0.034)	#SAKNAS!
nutritional-requirements	1 5110(0.011432491139819366)	2359 (0.034)	#SAKNAS!
nutrition-based	1 5110(0.011432491139819366)	#SAKNAS!	#SAKNAS!
nutritionday	1 2214(0.04572996455927746)	2359 (0.034)	1233 (0.1)
nutrition-focused	1 5110(0.011432491139819366)	2359 (0.034)	#SAKNAS!
nutrition-focused	1 5110(0.011432491139819366)	2359 (0.034)	#SAKNAS!
nutrition-intake	1 5110(0.011432491139819366)	#SAKNAS!	#SAKNAS!
nutritionists	1 5110(0.011432491139819366)	2359 (0.034)	#SAKNAS!
nutritionists	1 5110(0.011432491139819366)	2359 (0.034)	#SAKNAS!
nutrition-management	1 5110(0.011432491139819366)	2359 (0.034)	#SAKNAS!
nutrition-management	1 5110(0.011432491139819366)	2359 (0.034)	#SAKNAS!
ny	1 5110(0.011432491139819366)	#SAKNAS!	2548 (0.033)
nymph	1 5110(0.011432491139819366)	#SAKNAS!	2548 (0.033)
nymphalidae	1 5110(0.011432491139819366)	2359 (0.034)	#SAKNAS!
nymphalidae	1 5110(0.011432491139819366)	2359 (0.034)	#SAKNAS!
nymphs	1 2214(0.04572996455927746)	2359 (0.034)	1233 (0.1)
nyu	1 5110(0.011432491139819366)	#SAKNAS!	#SAKNAS!
oak	1 3437(0.02286498227963873)	2359 (0.034)	2548 (0.033)
oak-hickory	1 5110(0.011432491139819366)	#SAKNAS!	#SAKNAS!
oat-based	1 5110(0.011432491139819366)	2359 (0.034)	#SAKNAS!
oat-based	1 5110(0.011432491139819366)	2359 (0.034)	#SAKNAS!
oaten	1 5110(0.011432491139819366)	#SAKNAS!	2548 (0.033)
obesity-induced	1 5110(0.011432491139819366)	#SAKNAS!	2548 (0.033)
obestatin	1 5110(0.011432491139819366)	2359 (0.034)	#SAKNAS!
obestatin	1 5110(0.011432491139819366)	2359 (0.034)	#SAKNAS!
objectively	1 5110(0.011432491139819366)	#SAKNAS!	2548 (0.033)
objectives	1 5110(0.011432491139819366)	2359 (0.034)	#SAKNAS!
objectives	1 5110(0.011432491139819366)	2359 (0.034)	#SAKNAS!

object-oriented	1	5110(0.011432491139819366)	2359 (0.034)	#SAKNAS!
object-oriented	1	5110(0.011432491139819366)	2359 (0.034)	#SAKNAS!
oblada	1	5110(0.011432491139819366)	2359 (0.034)	#SAKNAS!
oblada	1	5110(0.011432491139819366)	2359 (0.034)	#SAKNAS!
oblique-end	1	5110(0.011432491139819366)	2359 (0.034)	#SAKNAS!
oblique-end	1	5110(0.011432491139819366)	2359 (0.034)	#SAKNAS!
observations	1	2693(0.0342974734194581)	2359 (0.034)	1668 (0.066)
observing	1	5110(0.011432491139819366)	#SAKNAS!	2548 (0.033)
obsessive	1	5110(0.011432491139819366)	2359 (0.034)	#SAKNAS!
obsessive	1	5110(0.011432491139819366)	2359 (0.034)	#SAKNAS!
obstruction-related	1	5110(0.011432491139819366)	#SAKNAS!	2548 (0.033)
obstructions	1	5110(0.011432491139819366)	#SAKNAS!	2548 (0.033)
obtech	1	5110(0.011432491139819366)	2359 (0.034)	#SAKNAS!
obtech	1	5110(0.011432491139819366)	2359 (0.034)	#SAKNAS!
obturator	1	5110(0.011432491139819366)	#SAKNAS!	#SAKNAS!
o-c2	1	3437(0.02286498227963873)	2359 (0.034)	2548 (0.033)
occidentalis	1	5110(0.011432491139819366)	2359 (0.034)	#SAKNAS!
occidentalis	1	5110(0.011432491139819366)	2359 (0.034)	#SAKNAS!
occlusions	1	5110(0.011432491139819366)	#SAKNAS!	#SAKNAS!
occult	1	5110(0.011432491139819366)	#SAKNAS!	#SAKNAS!
occupation	1	5110(0.011432491139819366)	#SAKNAS!	#SAKNAS!
occurring	1	2693(0.0342974734194581)	2359 (0.034)	1668 (0.066)
oceanica	1	5110(0.011432491139819366)	#SAKNAS!	2548 (0.033)
oceanographic	1	5110(0.011432491139819366)	#SAKNAS!	2548 (0.033)
ocellatus	1	5110(0.011432491139819366)	2359 (0.034)	#SAKNAS!
ocellatus	1	5110(0.011432491139819366)	2359 (0.034)	#SAKNAS!
octapeptide	1	5110(0.011432491139819366)	#SAKNAS!	2548 (0.033)
octn1	1	5110(0.011432491139819366)	#SAKNAS!	#SAKNAS!
octogenarians	1	3437(0.02286498227963873)	2359 (0.034)	2548 (0.033)
octopamine	1	5110(0.011432491139819366)	#SAKNAS!	2548 (0.033)
oculopharyngeal	1	2693(0.0342974734194581)	2359 (0.034)	#SAKNAS!
odds	1	5110(0.011432491139819366)	#SAKNAS!	2548 (0.033)
odocoileus	1	5110(0.011432491139819366)	2359 (0.034)	#SAKNAS!
odocoileus	1	5110(0.011432491139819366)	2359 (0.034)	#SAKNAS!
odontaster	1	5110(0.011432491139819366)	2359 (0.034)	#SAKNAS!
odontaster	1	5110(0.011432491139819366)	2359 (0.034)	#SAKNAS!
odontogenic	1	5110(0.011432491139819366)	#SAKNAS!	2548 (0.033)
odontological	1	2693(0.0342974734194581)	2359 (0.034)	#SAKNAS!
odorant	1	5110(0.011432491139819366)	#SAKNAS!	2548 (0.033)
odorant-stimulated	1	5110(0.011432491139819366)	#SAKNAS!	#SAKNAS!
odynophagia	1	5110(0.011432491139819366)	#SAKNAS!	2548 (0.033)
odynophagia-associated	1	5110(0.011432491139819366)	#SAKNAS!	2548 (0.033)
oeso-gastric	1	5110(0.011432491139819366)	#SAKNAS!	2548 (0.033)

oesophago-airway	1	5110(0.011432491139819366)	#SAKNAS!	2548 (0.033)
oesophagogastric	1	3437(0.02286498227963873)	2359 (0.034)	2548 (0.033)
oesophagostomy	1	5110(0.011432491139819366)	#SAKNAS!	2548 (0.033)
off	1	5110(0.011432491139819366)	#SAKNAS!	#SAKNAS!
offering	1	5110(0.011432491139819366)	#SAKNAS!	#SAKNAS!
offices	1	5110(0.011432491139819366)	#SAKNAS!	2548 (0.033)
officinale	1	5110(0.011432491139819366)	#SAKNAS!	#SAKNAS!
offset	1	5110(0.011432491139819366)	2359 (0.034)	#SAKNAS!
offset	1	5110(0.011432491139819366)	2359 (0.034)	#SAKNAS!
offsets	1	3437(0.02286498227963873)	2359 (0.034)	2548 (0.033)
oftrim2-associatedcharcot-marie	1	5110(0.011432491139819366)	#SAKNAS!	2548 (0.033)
ogg1	1	5110(0.011432491139819366)	2359 (0.034)	#SAKNAS!
ogg1	1	5110(0.011432491139819366)	2359 (0.034)	#SAKNAS!
oh	1	5110(0.011432491139819366)	#SAKNAS!	#SAKNAS!
ohat	1	5110(0.011432491139819366)	#SAKNAS!	2548 (0.033)
ohep	1	5110(0.011432491139819366)	#SAKNAS!	2548 (0.033)
ohrqol	1	2214(0.04572996455927746)	2359 (0.034)	#SAKNAS!
ohsaka	1	5110(0.011432491139819366)	#SAKNAS!	#SAKNAS!
okinawa	1	5110(0.011432491139819366)	#SAKNAS!	#SAKNAS!
okinawa-jima	1	5110(0.011432491139819366)	#SAKNAS!	2548 (0.033)
oklahoma	1	5110(0.011432491139819366)	#SAKNAS!	2548 (0.033)
olanzapine	1	5110(0.011432491139819366)	#SAKNAS!	#SAKNAS!
older-onset	1	5110(0.011432491139819366)	#SAKNAS!	#SAKNAS!
olds	1	5110(0.011432491139819366)	#SAKNAS!	2548 (0.033)
olduvai	1	5110(0.011432491139819366)	2359 (0.034)	#SAKNAS!
olduvai	1	5110(0.011432491139819366)	2359 (0.034)	#SAKNAS!
oleic	1	5110(0.011432491139819366)	#SAKNAS!	#SAKNAS!
oleifera	1	5110(0.011432491139819366)	#SAKNAS!	#SAKNAS!
oleoylethanolamide	1	5110(0.011432491139819366)	#SAKNAS!	#SAKNAS!
oleracea	1	3437(0.02286498227963873)	2359 (0.034)	#SAKNAS!
olfaction	1	5110(0.011432491139819366)	2359 (0.034)	#SAKNAS!
olfaction	1	5110(0.011432491139819366)	2359 (0.034)	#SAKNAS!
oligoarticular	1	5110(0.011432491139819366)	#SAKNAS!	#SAKNAS!
oligochaeta	1	5110(0.011432491139819366)	2359 (0.034)	#SAKNAS!
oligochaeta	1	5110(0.011432491139819366)	2359 (0.034)	#SAKNAS!
oligometastatic	1	5110(0.011432491139819366)	#SAKNAS!	#SAKNAS!
oligopeptides	1	5110(0.011432491139819366)	#SAKNAS!	2548 (0.033)
olive	1	5110(0.011432491139819366)	2359 (0.034)	#SAKNAS!
olive	1	5110(0.011432491139819366)	2359 (0.034)	#SAKNAS!
olympics	1	5110(0.011432491139819366)	2359 (0.034)	#SAKNAS!
olympics	1	5110(0.011432491139819366)	2359 (0.034)	#SAKNAS!
om	1	5110(0.011432491139819366)	2359 (0.034)	#SAKNAS!
om	1	5110(0.011432491139819366)	2359 (0.034)	#SAKNAS!

omaha	1	5110(0.011432491139819366)	#SAKNAS!	#SAKNAS!
ombe	1	5110(0.011432491139819366)	#SAKNAS!	#SAKNAS!
omega	1	5110(0.011432491139819366)	#SAKNAS!	#SAKNAS!
omission	1	5110(0.011432491139819366)	#SAKNAS!	2548 (0.033)
omissions	1	5110(0.011432491139819366)	#SAKNAS!	2548 (0.033)
omnivore	1	5110(0.011432491139819366)	#SAKNAS!	2548 (0.033)
omnivorous	1	5110(0.011432491139819366)	#SAKNAS!	#SAKNAS!
onabotulinum	1	5110(0.011432491139819366)	#SAKNAS!	2548 (0.033)
onasemnogene	1	5110(0.011432491139819366)	#SAKNAS!	#SAKNAS!
onbemisia	1	5110(0.011432491139819366)	#SAKNAS!	2548 (0.033)
onbtsoybean	1	5110(0.011432491139819366)	#SAKNAS!	2548 (0.033)
oncologists	1	5110(0.011432491139819366)	#SAKNAS!	#SAKNAS!
oncommunity	1	5110(0.011432491139819366)	#SAKNAS!	2548 (0.033)
one-bite-sized	1	5110(0.011432491139819366)	#SAKNAS!	#SAKNAS!
one-center	1	5110(0.011432491139819366)	#SAKNAS!	2548 (0.033)
one-humped	1	5110(0.011432491139819366)	2359 (0.034)	#SAKNAS!
one-humped	1	5110(0.011432491139819366)	2359 (0.034)	#SAKNAS!
one-level	1	5110(0.011432491139819366)	#SAKNAS!	#SAKNAS!
one-to-one	1	5110(0.011432491139819366)	#SAKNAS!	#SAKNAS!
one-to-two	1	5110(0.011432491139819366)	#SAKNAS!	#SAKNAS!
online	1	1678(0.0685949468389162)	2359 (0.034)	1233 (0.1)
onobrychis	1	5110(0.011432491139819366)	#SAKNAS!	2548 (0.033)
ontogenesis	1	5110(0.011432491139819366)	#SAKNAS!	#SAKNAS!
ontogenetic	1	2693(0.0342974734194581)	2359 (0.034)	2548 (0.033)
onychognathus	1	5110(0.011432491139819366)	#SAKNAS!	2548 (0.033)
oocyte	1	5110(0.011432491139819366)	#SAKNAS!	2548 (0.033)
oomycetes	1	5110(0.011432491139819366)	2359 (0.034)	#SAKNAS!
oomycetes	1	5110(0.011432491139819366)	2359 (0.034)	#SAKNAS!
oophagous	1	5110(0.011432491139819366)	2359 (0.034)	#SAKNAS!
oophagous	1	5110(0.011432491139819366)	2359 (0.034)	#SAKNAS!
open-airway	1	5110(0.011432491139819366)	#SAKNAS!	#SAKNAS!
operating	1	5110(0.011432491139819366)	2359 (0.034)	#SAKNAS!
operating	1	5110(0.011432491139819366)	2359 (0.034)	#SAKNAS!
operations	1	5110(0.011432491139819366)	#SAKNAS!	2548 (0.033)
operative	1	2214(0.04572996455927746)	2359 (0.034)	2548 (0.033)
operator	1	5110(0.011432491139819366)	#SAKNAS!	2548 (0.033)
ophiostomatoid	1	5110(0.011432491139819366)	#SAKNAS!	2548 (0.033)
ophiuroidea	1	5110(0.011432491139819366)	2359 (0.034)	#SAKNAS!
ophiuroidea	1	5110(0.011432491139819366)	2359 (0.034)	#SAKNAS!
opinions	1	2693(0.0342974734194581)	2359 (0.034)	2548 (0.033)
opioid-dependent	1	5110(0.011432491139819366)	2359 (0.034)	#SAKNAS!
opioid-dependent	1	5110(0.011432491139819366)	2359 (0.034)	#SAKNAS!
opiorphin	1	5110(0.011432491139819366)	#SAKNAS!	#SAKNAS!

opportunistic	1	1678(0.0685949468389162)	2359 (0.034)	1668 (0.066)
opportunity	1	3437(0.0228649822796387 3)	2359 (0.034)	#SAKNAS!
opposing	1	5110(0.011432491139819366)	#SAKNAS!	2548 (0.033)
opposite	1	5110(0.011432491139819366)	2359 (0.034)	#SAKNAS!
opposite	1	5110(0.011432491139819366)	2359 (0.034)	#SAKNAS!
optic	1	5110(0.011432491139819366)	#SAKNAS!	2548 (0.033)
optimality	1	5110(0.011432491139819366)	2359 (0.034)	#SAKNAS!
optimality	1	5110(0.011432491139819366)	2359 (0.034)	#SAKNAS!
optimize	1	5110(0.011432491139819366)	#SAKNAS!	#SAKNAS!
optimizes	1	5110(0.011432491139819366)	#SAKNAS!	2548 (0.033)
optimum	1	5110(0.011432491139819366)	2359 (0.034)	#SAKNAS!
optimum	1	5110(0.011432491139819366)	2359 (0.034)	#SAKNAS!
optinam	1	5110(0.011432491139819366)	#SAKNAS!	#SAKNAS!
option	1	5110(0.011432491139819366)	#SAKNAS!	2548 (0.033)
optogenetics	1	5110(0.011432491139819366)	#SAKNAS!	2548 (0.033)
optopad	1	5110(0.011432491139819366)	#SAKNAS!	2548 (0.033)
oral-cancer	1	5110(0.011432491139819366)	2359 (0.034)	#SAKNAS!
oral-cancer	1	5110(0.011432491139819366)	2359 (0.034)	#SAKNAS!
oral-d	1	5110(0.011432491139819366)	2359 (0.034)	#SAKNAS!
oral-d	1	5110(0.011432491139819366)	2359 (0.034)	#SAKNAS!
oral-gut	1	5110(0.011432491139819366)	#SAKNAS!	#SAKNAS!
orally	1	2693(0.0342974734194581)	2359 (0.034)	2548 (0.033)
oral-motor	1	5110(0.011432491139819366)	2359 (0.034)	#SAKNAS!
oral-motor	1	5110(0.011432491139819366)	2359 (0.034)	#SAKNAS!
oral-related	1	5110(0.011432491139819366)	#SAKNAS!	#SAKNAS!
orange	1	2693(0.0342974734194581)	2359 (0.034)	2548 (0.033)
orangutan	1	5110(0.011432491139819366)	#SAKNAS!	2548 (0.033)
oratest	1	5110(0.011432491139819366)	#SAKNAS!	2548 (0.033)
orator	1	5110(0.011432491139819366)	#SAKNAS!	#SAKNAS!
orbigny	1	5110(0.011432491139819366)	#SAKNAS!	#SAKNAS!
orbignyanus	1	5110(0.011432491139819366)	#SAKNAS!	2548 (0.033)
orchards	1	5110(0.011432491139819366)	#SAKNAS!	#SAKNAS!
orcinus	1	5110(0.011432491139819366)	#SAKNAS!	#SAKNAS!
orders	1	5110(0.011432491139819366)	#SAKNAS!	#SAKNAS!
ordinary	1	5110(0.011432491139819366)	#SAKNAS!	2548 (0.033)
oregano	1	5110(0.011432491139819366)	#SAKNAS!	2548 (0.033)
orelha	1	5110(0.011432491139819366)	#SAKNAS!	2548 (0.033)
oreochromis	1	2214(0.04572996455927746)	2359 (0.034)	1668 (0.066)
orestias	1	5110(0.011432491139819366)	#SAKNAS!	#SAKNAS!
orexin-a	1	3437(0.02286498227963873)	2359 (0.034)	2548 (0.033)
organic	1	1219(0.1028924202583743)	2359 (0.034)	2548 (0.033)
organically	1	5110(0.011432491139819366)	2359 (0.034)	#SAKNAS!
organically	1	5110(0.011432491139819366)	2359 (0.034)	#SAKNAS!

organic-chemicals	1	5110(0.011432491139819366)	2359 (0.034)	#SAKNAS!
organic-chemicals	1	5110(0.011432491139819366)	2359 (0.034)	#SAKNAS!
organisation	1	5110(0.011432491139819366)	2359 (0.034)	#SAKNAS!
organisation	1	5110(0.011432491139819366)	2359 (0.034)	#SAKNAS!
organism	1	5110(0.011432491139819366)	2359 (0.034)	#SAKNAS!
organism	1	5110(0.011432491139819366)	2359 (0.034)	#SAKNAS!
organismal	1	5110(0.011432491139819366)	#SAKNAS!	2548 (0.033)
organizations	1	5110(0.011432491139819366)	#SAKNAS!	2548 (0.033)
organochlorinated	1	5110(0.011432491139819366)	2359 (0.034)	#SAKNAS!
organochlorinated	1	5110(0.011432491139819366)	2359 (0.034)	#SAKNAS!
organoids	1	5110(0.011432491139819366)	2359 (0.034)	#SAKNAS!
organoids	1	5110(0.011432491139819366)	2359 (0.034)	#SAKNAS!
organoleptic	1	3437(0.02286498227963873)	2359 (0.034)	2548 (0.033)
organ-preservation	1	5110(0.011432491139819366)	2359 (0.034)	#SAKNAS!
organ-preservation	1	5110(0.011432491139819366)	2359 (0.034)	#SAKNAS!
organs	1	3437(0.02286498227963873)	2359 (0.034)	2548 (0.033)
oriental	1	5110(0.011432491139819366)	#SAKNAS!	#SAKNAS!
orientalis	1	5110(0.011432491139819366)	#SAKNAS!	#SAKNAS!
orientation	1	5110(0.011432491139819366)	2359 (0.034)	#SAKNAS!
orientation	1	5110(0.011432491139819366)	2359 (0.034)	#SAKNAS!
orientation-related	1	5110(0.011432491139819366)	#SAKNAS!	2548 (0.033)
originating	1	5110(0.011432491139819366)	#SAKNAS!	#SAKNAS!
oris	1	5110(0.011432491139819366)	2359 (0.034)	#SAKNAS!
oris	1	5110(0.011432491139819366)	2359 (0.034)	#SAKNAS!
ornatrix	1	5110(0.011432491139819366)	2359 (0.034)	#SAKNAS!
ornatrix	1	5110(0.011432491139819366)	2359 (0.034)	#SAKNAS!
ornithorhynchus	1	5110(0.011432491139819366)	2359 (0.034)	#SAKNAS!
ornithorhynchus	1	5110(0.011432491139819366)	2359 (0.034)	#SAKNAS!
oro-dental	1	5110(0.011432491139819366)	2359 (0.034)	#SAKNAS!
oro-dental	1	5110(0.011432491139819366)	2359 (0.034)	#SAKNAS!
orodigestive	1	5110(0.011432491139819366)	2359 (0.034)	#SAKNAS!
orodigestive	1	5110(0.011432491139819366)	2359 (0.034)	#SAKNAS!
oro-esophageal	1	5110(0.011432491139819366)	#SAKNAS!	#SAKNAS!
oromandibular	1	5110(0.011432491139819366)	#SAKNAS!	2548 (0.033)
oromandibulomaxillary	1	5110(0.011432491139819366)	2359 (0.034)	#SAKNAS!
oromandibulomaxillary	1	5110(0.011432491139819366)	2359 (0.034)	#SAKNAS!
oro-pharygo-laryngeal	1	5110(0.011432491139819366)	#SAKNAS!	2548 (0.033)
oro-pharyngeal	1	2214(0.04572996455927746)	2359 (0.034)	2548 (0.033)
oropharynx	1	2214(0.04572996455927746)	2359 (0.034)	1668 (0.066)
orosomucoid	1	5110(0.011432491139819366)	2359 (0.034)	#SAKNAS!
orosomucoid	1	5110(0.011432491139819366)	2359 (0.034)	#SAKNAS!
orphanin	1	5110(0.011432491139819366)	#SAKNAS!	#SAKNAS!
orthodontics	1	5110(0.011432491139819366)	#SAKNAS!	#SAKNAS!

orthogeriatric	1	5110(0.011432491139819366)	#SAKNAS!	2548 (0.033)
orthognathic	1	2214(0.04572996455927746)	2359 (0.034)	#SAKNAS!
orthoptera	1	3437(0.02286498227963873)	2359 (0.034)	#SAKNAS!
oryctolagus	1	5110(0.011432491139819366)	2359 (0.034)	#SAKNAS!
oryctolagus	1	5110(0.011432491139819366)	2359 (0.034)	#SAKNAS!
os	1	5110(0.011432491139819366)	#SAKNAS!	2548 (0.033)
osaka	1	5110(0.011432491139819366)	2359 (0.034)	#SAKNAS!
osaka	1	5110(0.011432491139819366)	2359 (0.034)	#SAKNAS!
osas	1	3437(0.02286498227963873)	2359 (0.034)	#SAKNAS!
oscillation	1	2693(0.0342974734194581)	2359 (0.034)	2548 (0.033)
oscillations	1	3437(0.02286498227963873)	2359 (0.034)	2548 (0.033)
oscillatory	1	3437(0.02286498227963873)	2359 (0.034)	#SAKNAS!
osimertinib	1	5110(0.011432491139819366)	#SAKNAS!	#SAKNAS!
osmolality	1	5110(0.011432491139819366)	#SAKNAS!	2548 (0.033)
osmoregulation	1	5110(0.011432491139819366)	#SAKNAS!	2548 (0.033)
osmotic	1	5110(0.011432491139819366)	#SAKNAS!	#SAKNAS!
ossification	1	5110(0.011432491139819366)	#SAKNAS!	#SAKNAS!
ossing	1	5110(0.011432491139819366)	#SAKNAS!	#SAKNAS!
osteo	1	5110(0.011432491139819366)	2359 (0.034)	#SAKNAS!
osteo	1	5110(0.011432491139819366)	2359 (0.034)	#SAKNAS!
osteoarthritis	1	1678(0.0685949468389162)	2359 (0.034)	1233 (0.1)
osteoclast	1	5110(0.011432491139819366)	#SAKNAS!	#SAKNAS!
osteoclastic	1	5110(0.011432491139819366)	2359 (0.034)	#SAKNAS!
osteoclastic	1	5110(0.011432491139819366)	2359 (0.034)	#SAKNAS!
osteogenic	1	5110(0.011432491139819366)	#SAKNAS!	#SAKNAS!
osteonecrosis	1	2693(0.0342974734194581)	2359 (0.034)	2548 (0.033)
osteopathology	1	5110(0.011432491139819366)	2359 (0.034)	#SAKNAS!
osteopathology	1	5110(0.011432491139819366)	2359 (0.034)	#SAKNAS!
osteoperio	1	2214(0.04572996455927746)	2359 (0.034)	2548 (0.033)
osteophytes	1	5110(0.011432491139819366)	2359 (0.034)	#SAKNAS!
osteophytes	1	5110(0.011432491139819366)	2359 (0.034)	#SAKNAS!
osteoprotegerin	1	5110(0.011432491139819366)	2359 (0.034)	#SAKNAS!
osteoprotegerin	1	5110(0.011432491139819366)	2359 (0.034)	#SAKNAS!
osteoradionecrosis	1	5110(0.011432491139819366)	#SAKNAS!	#SAKNAS!
osteosyntheses	1	5110(0.011432491139819366)	2359 (0.034)	#SAKNAS!
osteosyntheses	1	5110(0.011432491139819366)	2359 (0.034)	#SAKNAS!
ostomy	1	5110(0.011432491139819366)	#SAKNAS!	2548 (0.033)
ostracoda	1	5110(0.011432491139819366)	2359 (0.034)	#SAKNAS!
ostracoda	1	5110(0.011432491139819366)	2359 (0.034)	#SAKNAS!
ostrinia	1	5110(0.011432491139819366)	2359 (0.034)	#SAKNAS!
ostrinia	1	5110(0.011432491139819366)	2359 (0.034)	#SAKNAS!
otaria	1	5110(0.011432491139819366)	#SAKNAS!	2548 (0.033)
otariids	1	5110(0.011432491139819366)	2359 (0.034)	#SAKNAS!

otariids	1 5110(0.011432491139819366)	2359 (0.034)	#SAKNAS!
otiorhynchus	1 5110(0.011432491139819366)	2359 (0.034)	#SAKNAS!
otiorhynchus	1 5110(0.011432491139819366)	2359 (0.034)	#SAKNAS!
otis	1 5110(0.011432491139819366)	#SAKNAS!	2548 (0.033)
oto-rhino-laryngology	1 5110(0.011432491139819366)	#SAKNAS!	2548 (0.033)
otters	1 5110(0.011432491139819366)	2359 (0.034)	#SAKNAS!
otters	1 5110(0.011432491139819366)	2359 (0.034)	#SAKNAS!
otzi	1 5110(0.011432491139819366)	#SAKNAS!	#SAKNAS!
outbreaking	1 5110(0.011432491139819366)	2359 (0.034)	#SAKNAS!
outbreaking	1 5110(0.011432491139819366)	2359 (0.034)	#SAKNAS!
outbreaks	1 5110(0.011432491139819366)	2359 (0.034)	#SAKNAS!
outbreaks	1 5110(0.011432491139819366)	2359 (0.034)	#SAKNAS!
outcome-blinded	1 5110(0.011432491139819366)	#SAKNAS!	#SAKNAS!
outdoor	1 3437(0.02286498227963873)	2359 (0.034)	2548 (0.033)
outer	1 5110(0.011432491139819366)	2359 (0.034)	#SAKNAS!
outer	1 5110(0.011432491139819366)	2359 (0.034)	#SAKNAS!
outflow	1 5110(0.011432491139819366)	#SAKNAS!	#SAKNAS!
outliers	1 5110(0.011432491139819366)	#SAKNAS!	2548 (0.033)
out-of-home	1 5110(0.011432491139819366)	#SAKNAS!	#SAKNAS!
ovariectomy	1 5110(0.011432491139819366)	2359 (0.034)	#SAKNAS!
ovariectomy	1 5110(0.011432491139819366)	2359 (0.034)	#SAKNAS!
ovata	1 5110(0.011432491139819366)	2359 (0.034)	#SAKNAS!
ovata	1 5110(0.011432491139819366)	2359 (0.034)	#SAKNAS!
ovenbird	1 5110(0.011432491139819366)	2359 (0.034)	#SAKNAS!
ovenbird	1 5110(0.011432491139819366)	2359 (0.034)	#SAKNAS!
overactive	1 5110(0.011432491139819366)	#SAKNAS!	2548 (0.033)
overdenture	1 5110(0.011432491139819366)	2359 (0.034)	#SAKNAS!
overdenture	1 5110(0.011432491139819366)	2359 (0.034)	#SAKNAS!
overeating	1 5110(0.011432491139819366)	#SAKNAS!	#SAKNAS!
overexpression	1 3437(0.02286498227963873)	2359 (0.034)	2548 (0.033)
overfeeding	1 5110(0.011432491139819366)	2359 (0.034)	#SAKNAS!
overfeeding	1 5110(0.011432491139819366)	2359 (0.034)	#SAKNAS!
overgrowth	1 5110(0.011432491139819366)	2359 (0.034)	#SAKNAS!
overgrowth	1 5110(0.011432491139819366)	2359 (0.034)	#SAKNAS!
overhydration	1 5110(0.011432491139819366)	2359 (0.034)	#SAKNAS!
overhydration	1 5110(0.011432491139819366)	2359 (0.034)	#SAKNAS!
overlap	1 2693(0.0342974734194581)	2359 (0.034)	#SAKNAS!
overlapping	1 5110(0.011432491139819366)	2359 (0.034)	#SAKNAS!
overlapping	1 5110(0.011432491139819366)	2359 (0.034)	#SAKNAS!
overrides	1 5110(0.011432491139819366)	#SAKNAS!	#SAKNAS!
oversee	1 5110(0.011432491139819366)	#SAKNAS!	2548 (0.033)
overt	1 5110(0.011432491139819366)	2359 (0.034)	#SAKNAS!
overt	1 5110(0.011432491139819366)	2359 (0.034)	#SAKNAS!

overtubes	1	5110(0.011432491139819366)	#SAKNAS!	2548 (0.033)
ovine	1	5110(0.011432491139819366)	2359 (0.034)	#SAKNAS!
ovine	1	5110(0.011432491139819366)	2359 (0.034)	#SAKNAS!
ovipositional	1	5110(0.011432491139819366)	2359 (0.034)	#SAKNAS!
ovipositional	1	5110(0.011432491139819366)	2359 (0.034)	#SAKNAS!
ovo-lacto-vegetarian	1	5110(0.011432491139819366)	#SAKNAS!	2548 (0.033)
owing	1	5110(0.011432491139819366)	2359 (0.034)	#SAKNAS!
owing	1	5110(0.011432491139819366)	2359 (0.034)	#SAKNAS!
oxalic	1	5110(0.011432491139819366)	#SAKNAS!	2548 (0.033)
oxaliplatin	1	5110(0.011432491139819366)	2359 (0.034)	#SAKNAS!
oxaliplatin	1	5110(0.011432491139819366)	2359 (0.034)	#SAKNAS!
oxaliplatin-related	1	5110(0.011432491139819366)	#SAKNAS!	#SAKNAS!
oxen	1	5110(0.011432491139819366)	2359 (0.034)	#SAKNAS!
oxen	1	5110(0.011432491139819366)	2359 (0.034)	#SAKNAS!
oxidant	1	5110(0.011432491139819366)	#SAKNAS!	#SAKNAS!
oxidase-dependent	1	5110(0.011432491139819366)	2359 (0.034)	#SAKNAS!
oxidase-dependent	1	5110(0.011432491139819366)	2359 (0.034)	#SAKNAS!
oxidation	1	5110(0.011432491139819366)	#SAKNAS!	2548 (0.033)
oxidative	1	836(0.16005487595747114)	2359 (0.034)	461 (0.3)
oxide	1	1894(0.05716245569909683)	2359 (0.034)	#SAKNAS!
oximetry	1	5110(0.011432491139819366)	2359 (0.034)	#SAKNAS!
oximetry	1	5110(0.011432491139819366)	2359 (0.034)	#SAKNAS!
oxygenation	1	5110(0.011432491139819366)	#SAKNAS!	#SAKNAS!
oxylipins	1	5110(0.011432491139819366)	2359 (0.034)	#SAKNAS!
oxylipins	1	5110(0.011432491139819366)	2359 (0.034)	#SAKNAS!
oystercatchers	1	5110(0.011432491139819366)	2359 (0.034)	#SAKNAS!
oystercatchers	1	5110(0.011432491139819366)	2359 (0.034)	#SAKNAS!
oysters	1	5110(0.011432491139819366)	#SAKNAS!	#SAKNAS!
ozone-poplar-chrysomela	1	5110(0.011432491139819366)	#SAKNAS!	#SAKNAS!
p21	1	5110(0.011432491139819366)	2359 (0.034)	#SAKNAS!
p21	1	5110(0.011432491139819366)	2359 (0.034)	#SAKNAS!
pac1	1	5110(0.011432491139819366)	#SAKNAS!	#SAKNAS!
pace	1	5110(0.011432491139819366)	#SAKNAS!	#SAKNAS!
pacifastacus	1	5110(0.011432491139819366)	2359 (0.034)	#SAKNAS!
pacifastacus	1	5110(0.011432491139819366)	2359 (0.034)	#SAKNAS!
package	1	3437(0.02286498227963873)	2359 (0.034)	#SAKNAS!
packaging	1	5110(0.011432491139819366)	#SAKNAS!	2548 (0.033)
packet	1	5110(0.011432491139819366)	#SAKNAS!	#SAKNAS!
padu	1	5110(0.011432491139819366)	#SAKNAS!	#SAKNAS!
pagrus	1	5110(0.011432491139819366)	2359 (0.034)	#SAKNAS!
pagrus	1	5110(0.011432491139819366)	2359 (0.034)	#SAKNAS!
painful	1	5110(0.011432491139819366)	#SAKNAS!	2548 (0.033)
pair	1	3437(0.02286498227963873)	2359 (0.034)	#SAKNAS!

pairs	1	1678(0.0685949468389162)	2359 (0.034)	1668 (0.066)
pakistan	1	1219(0.1028924202583743)	2359 (0.034)	1233 (0.1)
palaemonidae	1	5110(0.011432491139819366)	#SAKNAS!	2548 (0.033)
palatable	1	2214(0.04572996455927746)	2359 (0.034)	1668 (0.066)
palatal	1	5110(0.011432491139819366)	#SAKNAS!	2548 (0.033)
palau	1	5110(0.011432491139819366)	#SAKNAS!	#SAKNAS!
pale	1	5110(0.011432491139819366)	#SAKNAS!	2548 (0.033)
paleo	1	5110(0.011432491139819366)	#SAKNAS!	#SAKNAS!
paleoecology	1	5110(0.011432491139819366)	#SAKNAS!	2548 (0.033)
paleo-odontological	1	5110(0.011432491139819366)	#SAKNAS!	#SAKNAS!
paleopathological	1	5110(0.011432491139819366)	#SAKNAS!	#SAKNAS!
paleosyndemics	1	5110(0.011432491139819366)	#SAKNAS!	#SAKNAS!
paliative	1	5110(0.011432491139819366)	#SAKNAS!	2548 (0.033)
palinurid	1	5110(0.011432491139819366)	#SAKNAS!	2548 (0.033)
pallas	1	5110(0.011432491139819366)	#SAKNAS!	2548 (0.033)
pallens	1	5110(0.011432491139819366)	#SAKNAS!	2548 (0.033)
pallidus	1	5110(0.011432491139819366)	#SAKNAS!	2548 (0.033)
palm	1	5110(0.011432491139819366)	#SAKNAS!	#SAKNAS!
palmitate	1	5110(0.011432491139819366)	#SAKNAS!	2548 (0.033)
pals	1	5110(0.011432491139819366)	#SAKNAS!	2548 (0.033)
palsies	1	5110(0.011432491139819366)	#SAKNAS!	#SAKNAS!
panauti	1	5110(0.011432491139819366)	#SAKNAS!	2548 (0.033)
panax	1	5110(0.011432491139819366)	2359 (0.034)	#SAKNAS!
panax	1	5110(0.011432491139819366)	2359 (0.034)	#SAKNAS!
pancreatitis	1	5110(0.011432491139819366)	2359 (0.034)	#SAKNAS!
pancreatitis	1	5110(0.011432491139819366)	2359 (0.034)	#SAKNAS!
pancreatoduodenectomy	1	3437(0.02286498227963873)	2359 (0.034)	2548 (0.033)
pandas	1	2693(0.0342974734194581)	2359 (0.034)	1668 (0.066)
pangolin	1	5110(0.011432491139819366)	#SAKNAS!	#SAKNAS!
pangolins	1	5110(0.011432491139819366)	#SAKNAS!	#SAKNAS!
panic-study	1	5110(0.011432491139819366)	#SAKNAS!	2548 (0.033)
panoramic	1	5110(0.011432491139819366)	#SAKNAS!	2548 (0.033)
pan-paniscus	1	5110(0.011432491139819366)	2359 (0.034)	#SAKNAS!
pan-paniscus	1	5110(0.011432491139819366)	2359 (0.034)	#SAKNAS!
pansteatitis	1	5110(0.011432491139819366)	#SAKNAS!	2548 (0.033)
pan-troglodytes	1	5110(0.011432491139819366)	2359 (0.034)	#SAKNAS!
pan-troglodytes	1	5110(0.011432491139819366)	2359 (0.034)	#SAKNAS!
pap	1	5110(0.011432491139819366)	#SAKNAS!	2548 (0.033)
paper	1	1894(0.0571624556990968З)	2359 (0.034)	1233 (0.1)
papilio	1	5110(0.011432491139819366)	#SAKNAS!	2548 (0.033)
papilionid	1	5110(0.011432491139819366)	#SAKNAS!	2548 (0.033)
papilloma	1	5110(0.011432491139819366)	2359 (0.034)	#SAKNAS!
papilloma	1	5110(0.011432491139819366)	2359 (0.034)	#SAKNAS!

papillomavirus-unrelated	1	5110(0.011432491139819366)	#SAKNAS!	2548 (0.033)
papionins	1	5110(0.011432491139819366)	#SAKNAS!	#SAKNAS!
para-adiacens	1	5110(0.011432491139819366)	#SAKNAS!	2548 (0.033)
parabens	1	5110(0.011432491139819366)	#SAKNAS!	2548 (0.033)
parabiotic	1	5110(0.011432491139819366)	2359 (0.034)	#SAKNAS!
parabiotic	1	5110(0.011432491139819366)	2359 (0.034)	#SAKNAS!
parabrachial	1	5110(0.011432491139819366)	2359 (0.034)	#SAKNAS!
parabrachial	1	5110(0.011432491139819366)	2359 (0.034)	#SAKNAS!
paracasei	1	5110(0.011432491139819366)	#SAKNAS!	#SAKNAS!
paracellular	1	5110(0.011432491139819366)	2359 (0.034)	#SAKNAS!
paracellular	1	5110(0.011432491139819366)	2359 (0.034)	#SAKNAS!
paracentrotus	1	5110(0.011432491139819366)	#SAKNAS!	#SAKNAS!
para-clinical	1	5110(0.011432491139819366)	#SAKNAS!	#SAKNAS!
paradies	1	5110(0.011432491139819366)	#SAKNAS!	#SAKNAS!
paradigm	1	3437(0.02286498227963873)	2359 (0.034)	2548 (0.033)
paradisiaca	1	5110(0.011432491139819366)	#SAKNAS!	#SAKNAS!
paradox	1	2693(0.0342974734194581)	2359 (0.034)	2548 (0.033)
paradoxical	1	5110(0.011432491139819366)	2359 (0.034)	#SAKNAS!
paradoxical	1	5110(0.011432491139819366)	2359 (0.034)	#SAKNAS!
paragymnodinium	1	5110(0.011432491139819366)	#SAKNAS!	2548 (0.033)
parallel	1	5110(0.011432491139819366)	#SAKNAS!	#SAKNAS!
parallel-group	1	5110(0.011432491139819366)	#SAKNAS!	2548 (0.033)
paralysis	1	3437(0.02286498227963873)	2359 (0.034)	#SAKNAS!
paralytic	1	5110(0.011432491139819366)	2359 (0.034)	#SAKNAS!
paralytic	1	5110(0.011432491139819366)	2359 (0.034)	#SAKNAS!
parameter	1	5110(0.011432491139819366)	#SAKNAS!	#SAKNAS!
parametric	1	5110(0.011432491139819366)	#SAKNAS!	2548 (0.033)
paraneoplastic	1	5110(0.011432491139819366)	#SAKNAS!	#SAKNAS!
para-oesophageal	1	5110(0.011432491139819366)	#SAKNAS!	2548 (0.033)
paraplegias	1	5110(0.011432491139819366)	#SAKNAS!	2548 (0.033)
paraponera	1	5110(0.011432491139819366)	#SAKNAS!	2548 (0.033)
parasite	1	3437(0.02286498227963873)	2359 (0.034)	#SAKNAS!
parasite-induced	1	5110(0.011432491139819366)	2359 (0.034)	#SAKNAS!
parasite-induced	1	5110(0.011432491139819366)	2359 (0.034)	#SAKNAS!
parasite-mediated	1	5110(0.011432491139819366)	#SAKNAS!	2548 (0.033)
parasites	1	5110(0.011432491139819366)	#SAKNAS!	#SAKNAS!
parasitic	1	2214(0.04572996455927746)	2359 (0.034)	1233 (0.1)
parasitized	1	5110(0.011432491139819366)	#SAKNAS!	2548 (0.033)
parasitoids	1	3437(0.02286498227963873)	2359 (0.034)	#SAKNAS!
parathyroid	1	3437(0.02286498227963873)	2359 (0.034)	#SAKNAS!
paratrichosis	1	5110(0.011432491139819366)	#SAKNAS!	2548 (0.033)
pardosa-hortensis	1	5110(0.011432491139819366)	2359 (0.034)	#SAKNAS!
pardosa-hortensis	1	5110(0.011432491139819366)	2359 (0.034)	#SAKNAS!

parental-oriented	1	5110(0.011432491139819366)	#SAKNAS!	2548 (0.033)
parental-reported	1	3437(0.02286498227963873)	2359 (0.034)	#SAKNAS!
parenteral-feeding	1	5110(0.011432491139819366)	2359 (0.034)	#SAKNAS!
parenteral-feeding	1	5110(0.011432491139819366)	2359 (0.034)	#SAKNAS!
parent-focused	1	5110(0.011432491139819366)	2359 (0.034)	#SAKNAS!
parent-focused	1	5110(0.011432491139819366)	2359 (0.034)	#SAKNAS!
paresis	1	5110(0.011432491139819366)	2359 (0.034)	#SAKNAS!
paresis	1	5110(0.011432491139819366)	2359 (0.034)	#SAKNAS!
parkinsons-disease	1	5110(0.011432491139819366)	2359 (0.034)	#SAKNAS!
parkinsons-disease	1	5110(0.011432491139819366)	2359 (0.034)	#SAKNAS!
parodontitis	1	5110(0.011432491139819366)	2359 (0.034)	#SAKNAS!
parodontitis	1	5110(0.011432491139819366)	2359 (0.034)	#SAKNAS!
parotidectomy	1	5110(0.011432491139819366)	#SAKNAS!	#SAKNAS!
parotid-sparing	1	5110(0.011432491139819366)	2359 (0.034)	#SAKNAS!
parotid-sparing	1	5110(0.011432491139819366)	2359 (0.034)	#SAKNAS!
parrotfish	1	5110(0.011432491139819366)	#SAKNAS!	2548 (0.033)
parrotlet	1	5110(0.011432491139819366)	2359 (0.034)	#SAKNAS!
parrotlet	1	5110(0.011432491139819366)	2359 (0.034)	#SAKNAS!
parrots	1	5110(0.011432491139819366)	#SAKNAS!	2548 (0.033)
part	1	1111(0.11432491139819366)	1121 (0.1)	816 (0.16)
part1	1	5110(0.011432491139819366)	#SAKNAS!	#SAKNAS!
parthenium	1	5110(0.011432491139819366)	2359 (0.034)	#SAKNAS!
parthenium	1	5110(0.011432491139819366)	2359 (0.034)	#SAKNAS!
partially-dentate	1	5110(0.011432491139819366)	2359 (0.034)	#SAKNAS!
partially-dentate	1	5110(0.011432491139819366)	2359 (0.034)	#SAKNAS!
participant	1	5110(0.011432491139819366)	#SAKNAS!	#SAKNAS!
participates	1	5110(0.011432491139819366)	#SAKNAS!	#SAKNAS!
participating	1	1485(0.08002743797873557)	2359 (0.034)	1233 (0.1)
particolored	1	5110(0.011432491139819366)	#SAKNAS!	#SAKNAS!
particulates	1	5110(0.011432491139819366)	2359 (0.034)	#SAKNAS!
particulates	1	5110(0.011432491139819366)	2359 (0.034)	#SAKNAS!
partner	1	5110(0.011432491139819366)	2359 (0.034)	#SAKNAS!
partner	1	5110(0.011432491139819366)	2359 (0.034)	#SAKNAS!
partnering	1	5110(0.011432491139819366)	2359 (0.034)	#SAKNAS!
partnering	1	5110(0.011432491139819366)	2359 (0.034)	#SAKNAS!
partners	1	5110(0.011432491139819366)	#SAKNAS!	#SAKNAS!
partridges	1	5110(0.011432491139819366)	#SAKNAS!	#SAKNAS!
party	1	5110(0.011432491139819366)	#SAKNAS!	2548 (0.033)
pase-brasil	1	5110(0.011432491139819366)	#SAKNAS!	2548 (0.033)
passage	1	5110(0.011432491139819366)	2359 (0.034)	#SAKNAS!
passage	1	5110(0.011432491139819366)	2359 (0.034)	#SAKNAS!
passion	1	5110(0.011432491139819366)	2359 (0.034)	#SAKNAS!
passion	1	5110(0.011432491139819366)	2359 (0.034)	#SAKNAS!

paste	1	5110(0.011432491139819366)	#SAKNAS!	#SAKNAS!
pastoral	1	5110(0.011432491139819366)	#SAKNAS!	2548 (0.033)
pasture-based	1	5110(0.011432491139819366)	#SAKNAS!	2548 (0.033)
past-year	1	5110(0.011432491139819366)	#SAKNAS!	2548 (0.033)
patagonia	1	2693(0.0342974734194581)	2359 (0.034)	2548 (0.033)
patch	1	2693(0.0342974734194581)	2359 (0.034)	1668 (0.066)
patent	1	5110(0.011432491139819366)	2359 (0.034)	#SAKNAS!
patent	1	5110(0.011432491139819366)	2359 (0.034)	#SAKNAS!
pathogen	1	1219(0.1028924202583743)	2359 (0.034)	816 (0.16)
pathogenic	1	1894(0.05716245569909683)	2359 (0.034)	1668 (0.066)
pathogenicity	1	5110(0.011432491139819366)	#SAKNAS!	#SAKNAS!
pathologists	1	1894(0.05716245569909683)	2359 (0.034)	2548 (0.033)
pathophysiologic	1	5110(0.011432491139819366)	2359 (0.034)	#SAKNAS!
pathophysiologic	1	5110(0.011432491139819366)	2359 (0.034)	#SAKNAS!
pathumthani	1	5110(0.011432491139819366)	#SAKNAS!	2548 (0.033)
patient-centric	1	5110(0.011432491139819366)	#SAKNAS!	#SAKNAS!
patient-gene	1	5110(0.011432491139819366)	2359 (0.034)	#SAKNAS!
patient-gene	1	5110(0.011432491139819366)	2359 (0.034)	#SAKNAS!
patient-generated	1	3437(0.02286498227963873)	2359 (0.034)	2548 (0.033)
patient-specific	1	5110(0.011432491139819366)	#SAKNAS!	2548 (0.033)
patients-severe	1	5110(0.011432491139819366)	#SAKNAS!	#SAKNAS!
patterning	1	5110(0.011432491139819366)	#SAKNAS!	2548 (0.033)
patulous	1	5110(0.011432491139819366)	#SAKNAS!	#SAKNAS!
pause	1	5110(0.011432491139819366)	2359 (0.034)	#SAKNAS!
pause	1	5110(0.011432491139819366)	2359 (0.034)	#SAKNAS!
pausing	1	5110(0.011432491139819366)	#SAKNAS!	2548 (0.033)
pay-per-kilo	1	5110(0.011432491139819366)	2359 (0.034)	#SAKNAS!
pay-per-kilo	1	5110(0.011432491139819366)	2359 (0.034)	#SAKNAS!
pays	1	5110(0.011432491139819366)	#SAKNAS!	2548 (0.033)
pb	1	5110(0.011432491139819366)	#SAKNAS!	2548 (0.033)
pcb	1	5110(0.011432491139819366)	#SAKNAS!	2548 (0.033)
pcos	1	5110(0.011432491139819366)	#SAKNAS!	#SAKNAS!
pd1	1	5110(0.011432491139819366)	#SAKNAS!	#SAKNAS!
pdcd5	1	5110(0.011432491139819366)	#SAKNAS!	#SAKNAS!
pdlcs	1	5110(0.011432491139819366)	#SAKNAS!	#SAKNAS!
peak-lactation	1	5110(0.011432491139819366)	#SAKNAS!	#SAKNAS!
pearl	1	5110(0.011432491139819366)	2359 (0.034)	#SAKNAS!
pearl	1	5110(0.011432491139819366)	2359 (0.034)	#SAKNAS!
pecking	1	2693(0.0342974734194581)	2359 (0.034)	1668 (0.066)
pecten	1	5110(0.011432491139819366)	2359 (0.034)	#SAKNAS!
pecten	1	5110(0.011432491139819366)	2359 (0.034)	#SAKNAS!
pectin	1	5110(0.011432491139819366)	#SAKNAS!	2548 (0.033)
peculiarities	1	5110(0.011432491139819366)	#SAKNAS!	2548 (0.033)

pediatrician	1 3437(0.0228649822796387З)	2359 (0.034)	#SAKNAS!
pediatrics	1 2214(0.0457299645592774б)	2359 (0.034)	1668 (0.066)
pedicle	1 5110(0.0114324911398193бб)	#SAKNAS!	#SAKNAS!
pedicled	1 5110(0.011432491139819366)	#SAKNAS!	2548 (0.033)
pegs	1 5110(0.011432491139819366)	2359 (0.034)	#SAKNAS!
pegs	1 5110(0.011432491139819366)	2359 (0.034)	#SAKNAS!
peg-tube	1 5110(0.011432491139819366)	#SAKNAS!	2548 (0.033)
pelage	1 5110(0.011432491139819366)	2359 (0.034)	#SAKNAS!
pelage	1 5110(0.011432491139819366)	2359 (0.034)	#SAKNAS!
pellagra	1 5110(0.011432491139819366)	2359 (0.034)	#SAKNAS!
pellagra	1 5110(0.011432491139819366)	2359 (0.034)	#SAKNAS!
pelotas	1 2693(0.0342974734194581)	2359 (0.034)	2548 (0.033)
pem	1 5110(0.011432491139819366)	2359 (0.034)	#SAKNAS!
pem	1 5110(0.011432491139819366)	2359 (0.034)	#SAKNAS!
pembrolizumab-induced	1 5110(0.011432491139819366)	#SAKNAS!	2548 (0.033)
penaeid	1 5110(0.011432491139819366)	2359 (0.034)	#SAKNAS!
penaeid	1 5110(0.011432491139819366)	2359 (0.034)	#SAKNAS!
penaeidae	1 5110(0.011432491139819366)	#SAKNAS!	2548 (0.033)
penaeus-monodon	1 5110(0.011432491139819366)	2359 (0.034)	#SAKNAS!
penaeus-monodon	1 5110(0.011432491139819366)	2359 (0.034)	#SAKNAS!
penang	1 5110(0.011432491139819366)	2359 (0.034)	#SAKNAS!
penang	1 5110(0.011432491139819366)	2359 (0.034)	#SAKNAS!
penetrating	1 5110(0.011432491139819366)	#SAKNAS!	#SAKNAS!
penetration-aspiration	1 3437(0.02286498227963873)	2359 (0.034)	2548 (0.033)
penguins	1 3437(0.02286498227963873)	2359 (0.034)	#SAKNAS!
penh	1 5110(0.011432491139819366)	#SAKNAS!	2548 (0.033)
peniight	1 5110(0.011432491139819366)	#SAKNAS!	2548 (0.033)
peninsula	1 3437(0.02286498227963873)	2359 (0.034)	#SAKNAS!
pennant	1 5110(0.011432491139819366)	2359 (0.034)	#SAKNAS!
pennant	1 5110(0.011432491139819366)	2359 (0.034)	#SAKNAS!
pennicornis	1 5110(0.011432491139819366)	2359 (0.034)	#SAKNAS!
pennicornis	1 5110(0.011432491139819366)	2359 (0.034)	#SAKNAS!
pennsylvania	1 5110(0.011432491139819366)	#SAKNAS!	#SAKNAS!
pens	1 5110(0.011432491139819366)	2359 (0.034)	#SAKNAS!
pens	1 5110(0.011432491139819366)	2359 (0.034)	#SAKNAS!
pentose-phosphate	1 5110(0.011432491139819366)	2359 (0.034)	#SAKNAS!
pentose-phosphate	1 5110(0.011432491139819366)	2359 (0.034)	#SAKNAS!
pen-urban	1 5110(0.011432491139819366)	2359 (0.034)	#SAKNAS!
pen-urban	1 5110(0.011432491139819366)	2359 (0.034)	#SAKNAS!
peoples	1 5110(0.011432491139819366)	2359 (0.034)	#SAKNAS!
peoples	1 5110(0.011432491139819366)	2359 (0.034)	#SAKNAS!
peppermint	1 5110(0.011432491139819366)	2359 (0.034)	#SAKNAS!
peppermint	1 5110(0.011432491139819366)	2359 (0.034)	#SAKNAS!

peptic	1	3437(0.02286498227963873)	2359 (0.034)	2548 (0.033)
peptidoglycan	1	5110(0.011432491139819366)	#SAKNAS!	#SAKNAS!
per	1	1335(0.09145992911855493)	2359 (0.034)	1668 (0.066)
perca	1	5110(0.011432491139819366)	2359 (0.034)	#SAKNAS!
perca	1	5110(0.011432491139819366)	2359 (0.034)	#SAKNAS!
perca-fluviatilis	1	5110(0.011432491139819366)	2359 (0.034)	#SAKNAS!
perca-fluviatilis	1	5110(0.011432491139819366)	2359 (0.034)	#SAKNAS!
percent	1	5110(0.011432491139819366)	2359 (0.034)	#SAKNAS!
percent	1	5110(0.011432491139819366)	2359 (0.034)	#SAKNAS!
percentile	1	5110(0.011432491139819366)	#SAKNAS!	#SAKNAS!
perfluorinated	1	5110(0.011432491139819366)	#SAKNAS!	2548 (0.033)
perfluoroalkyls	1	5110(0.011432491139819366)	#SAKNAS!	2548 (0.033)
perforations	1	5110(0.011432491139819366)	2359 (0.034)	#SAKNAS!
perforations	1	5110(0.011432491139819366)	2359 (0.034)	#SAKNAS!
perforatum	1	5110(0.011432491139819366)	#SAKNAS!	2548 (0.033)
performers	1	5110(0.011432491139819366)	#SAKNAS!	2548 (0.033)
peri	1	3437(0.02286498227963873)	2359 (0.034)	2548 (0.033)
peri-	1	5110(0.011432491139819366)	2359 (0.034)	#SAKNAS!
peri-	1	5110(0.011432491139819366)	2359 (0.034)	#SAKNAS!
periapical	1	5110(0.011432491139819366)	#SAKNAS!	#SAKNAS!
periaqueductal	1	5110(0.011432491139819366)	#SAKNAS!	#SAKNAS!
pericardial	1	5110(0.011432491139819366)	#SAKNAS!	2548 (0.033)
pericoronitis	1	5110(0.011432491139819366)	2359 (0.034)	#SAKNAS!
pericoronitis	1	5110(0.011432491139819366)	2359 (0.034)	#SAKNAS!
peridroma-saucia	1	5110(0.011432491139819366)	2359 (0.034)	#SAKNAS!
peridroma-saucia	1	5110(0.011432491139819366)	2359 (0.034)	#SAKNAS!
perilla	1	5110(0.011432491139819366)	#SAKNAS!	#SAKNAS!
perio	1	5110(0.011432491139819366)	2359 (0.034)	#SAKNAS!
perio	1	5110(0.011432491139819366)	2359 (0.034)	#SAKNAS!
periocardio	1	5110(0.011432491139819366)	#SAKNAS!	2548 (0.033)
periodic	1	5110(0.011432491139819366)	#SAKNAS!	#SAKNAS!
periodontal-diseases	1	5110(0.011432491139819366)	2359 (0.034)	#SAKNAS!
periodontal-diseases	1	5110(0.011432491139819366)	2359 (0.034)	#SAKNAS!
periodontia	1	5110(0.011432491139819366)	2359 (0.034)	#SAKNAS!
periodontia	1	5110(0.011432491139819366)	2359 (0.034)	#SAKNAS!
periodontitis-associated	1	2214(0.04572996455927746)	2359 (0.034)	1668 (0.066)
periodontitis-evidence	1	5110(0.011432491139819366)	#SAKNAS!	#SAKNAS!
periodontium	1	5110(0.011432491139819366)	#SAKNAS!	#SAKNAS!
periodontology	1	5110(0.011432491139819366)	#SAKNAS!	#SAKNAS!
periodontopathic	1	5110(0.011432491139819366)	2359 (0.034)	#SAKNAS!
periodontopathic	1	5110(0.011432491139819366)	2359 (0.034)	#SAKNAS!
periodontopathogen	1	5110(0.011432491139819366)	2359 (0.034)	#SAKNAS!
periodontopathogen	1	5110(0.011432491139819366)	2359 (0.034)	#SAKNAS!

period-regulated	1	5110(0.011432491139819366)	#SAKNAS!	2548 (0.033)
peri-operative	1	5110(0.011432491139819366)	#SAKNAS!	#SAKNAS!
perio-systemic	1	5110(0.011432491139819366)	#SAKNAS!	#SAKNAS!
periphery	1	5110(0.011432491139819366)	#SAKNAS!	#SAKNAS!
periphytic	1	5110(0.011432491139819366)	#SAKNAS!	#SAKNAS!
periplasmic	1	5110(0.011432491139819366)	#SAKNAS!	2548 (0.033)
periscope	1	5110(0.011432491139819366)	#SAKNAS!	#SAKNAS!
peristaltic	1	5110(0.011432491139819366)	#SAKNAS!	#SAKNAS!
peritoneal	1	1894(0.05716245569909683)	2359 (0.034)	1668 (0.066)
periwinkle	1	5110(0.011432491139819366)	2359 (0.034)	#SAKNAS!
periwinkle	1	5110(0.011432491139819366)	2359 (0.034)	#SAKNAS!
permissive	1	5110(0.011432491139819366)	2359 (0.034)	#SAKNAS!
permissive	1	5110(0.011432491139819366)	2359 (0.034)	#SAKNAS!
perna	1	5110(0.011432491139819366)	2359 (0.034)	#SAKNAS!
perna	1	5110(0.011432491139819366)	2359 (0.034)	#SAKNAS!
pernambuco	1	5110(0.011432491139819366)	2359 (0.034)	#SAKNAS!
pernambuco	1	5110(0.011432491139819366)	2359 (0.034)	#SAKNAS!
peroxinectin-like	1	5110(0.011432491139819366)	#SAKNAS!	2548 (0.033)
peroxisome	1	5110(0.011432491139819366)	#SAKNAS!	2548 (0.033)
pers	1	5110(0.011432491139819366)	#SAKNAS!	2548 (0.033)
persist	1	3437(0.02286498227963873)	2359 (0.034)	2548 (0.033)
persistently	1	5110(0.011432491139819366)	2359 (0.034)	#SAKNAS!
persistently	1	5110(0.011432491139819366)	2359 (0.034)	#SAKNAS!
persisting	1	3437(0.02286498227963873)	2359 (0.034)	2548 (0.033)
person	1	2693(0.0342974734194581)	2359 (0.034)	2548 (0.033)
personnel	1	5110(0.011432491139819366)	#SAKNAS!	#SAKNAS!
personsa	1	5110(0.011432491139819366)	#SAKNAS!	2548 (0.033)
persons-a	1	5110(0.011432491139819366)	#SAKNAS!	2548 (0.033)
pertinence	1	5110(0.011432491139819366)	#SAKNAS!	2548 (0.033)
perturbations	1	5110(0.011432491139819366)	#SAKNAS!	2548 (0.033)
peruvian	1	5110(0.011432491139819366)	#SAKNAS!	2548 (0.033)
pest	1	2214(0.04572996455927746)	2359 (0.034)	1668 (0.066)
pesticides	1	5110(0.011432491139819366)	#SAKNAS!	2548 (0.033)
pests	1	5110(0.011432491139819366)	2359 (0.034)	#SAKNAS!
pests	1	5110(0.011432491139819366)	2359 (0.034)	#SAKNAS!
pet	1	3437(0.02286498227963873)	2359 (0.034)	2548 (0.033)
petauroides	1	5110(0.011432491139819366)	#SAKNAS!	2548 (0.033)
petaurus	1	5110(0.011432491139819366)	#SAKNAS!	2548 (0.033)
peter-the-great	1	5110(0.011432491139819366)	2359 (0.034)	#SAKNAS!
peter-the-great	1	5110(0.011432491139819366)	2359 (0.034)	#SAKNAS!
petromuridae	1	5110(0.011432491139819366)	2359 (0.034)	#SAKNAS!
petromuridae	1	5110(0.011432491139819366)	2359 (0.034)	#SAKNAS!
petromus	1	5110(0.011432491139819366)	2359 (0.034)	#SAKNAS!

petromus	1	5110(0.011432491139819366)	2359 (0.034)	#SAKNAS!
pets@11	1	5110(0.011432491139819366)	#SAKNAS!	2548 (0.033)
pfas	1	5110(0.011432491139819366)	#SAKNAS!	#SAKNAS!
pfc	1	5110(0.011432491139819366)	2359 (0.034)	#SAKNAS!
pfc	1	5110(0.011432491139819366)	2359 (0.034)	#SAKNAS!
pge2-independent	1	5110(0.011432491139819366)	2359 (0.034)	#SAKNAS!
pge2-independent	1	5110(0.011432491139819366)	2359 (0.034)	#SAKNAS!
pg-sga	1	3437(0.02286498227963873)	2359 (0.034)	2548 (0.033)
ph4b-mesh	1	5110(0.011432491139819366)	#SAKNAS!	#SAKNAS!
phagotrophic	1	5110(0.011432491139819366)	2359 (0.034)	#SAKNAS!
phagotrophic	1	5110(0.011432491139819366)	2359 (0.034)	#SAKNAS!
phagotrophy	1	5110(0.011432491139819366)	2359 (0.034)	#SAKNAS!
phagotrophy	1	5110(0.011432491139819366)	2359 (0.034)	#SAKNAS!
pharmaceutical	1	5110(0.011432491139819366)	2359 (0.034)	#SAKNAS!
pharmaceutical	1	5110(0.011432491139819366)	2359 (0.034)	#SAKNAS!
pharmacists	1	5110(0.011432491139819366)	#SAKNAS!	#SAKNAS!
pharmacophagy	1	5110(0.011432491139819366)	2359 (0.034)	#SAKNAS!
pharmacophagy	1	5110(0.011432491139819366)	2359 (0.034)	#SAKNAS!
pharyngocise	1	5110(0.011432491139819366)	2359 (0.034)	#SAKNAS!
pharyngocise	1	5110(0.011432491139819366)	2359 (0.034)	#SAKNAS!
pharyngoesophageal	1	5110(0.011432491139819366)	2359 (0.034)	#SAKNAS!
pharyngoesophageal	1	5110(0.011432491139819366)	2359 (0.034)	#SAKNAS!
pharyngolaryngeal	1	5110(0.011432491139819366)	#SAKNAS!	#SAKNAS!
pharyngo-laryngeal	1	5110(0.011432491139819366)	#SAKNAS!	2548 (0.033)
pharyngo-laryngo-esophagecton	1	5110(0.011432491139819366)	#SAKNAS!	2548 (0.033)
pharyngoplasty	1	5110(0.011432491139819366)	2359 (0.034)	#SAKNAS!
pharyngoplasty	1	5110(0.011432491139819366)	2359 (0.034)	#SAKNAS!
pharynx	1	3437(0.02286498227963873)	2359 (0.034)	2548 (0.033)
phascolarctos-cinereus-adustus	1	5110(0.011432491139819366)	2359 (0.034)	#SAKNAS!
phascolarctos-cinereus-adustus	1	5110(0.011432491139819366)	2359 (0.034)	#SAKNAS!
phaseolus	1	5110(0.011432491139819366)	#SAKNAS!	2548 (0.033)
phases	1	2214(0.04572996455927746)	2359 (0.034)	#SAKNAS!
phasix?□	1	5110(0.011432491139819366)	#SAKNAS!	#SAKNAS!
phasmatidae	1	5110(0.011432491139819366)	2359 (0.034)	#SAKNAS!
phasmatidae	1	5110(0.011432491139819366)	2359 (0.034)	#SAKNAS!
phasmida	1	5110(0.011432491139819366)	2359 (0.034)	#SAKNAS!
phasmida	1	5110(0.011432491139819366)	2359 (0.034)	#SAKNAS!
phataginus	1	5110(0.011432491139819366)	#SAKNAS!	#SAKNAS!
phayre	1	5110(0.011432491139819366)	#SAKNAS!	2548 (0.033)
pheidole	1	5110(0.011432491139819366)	2359 (0.034)	#SAKNAS!
pheidole	1	5110(0.011432491139819366)	2359 (0.034)	#SAKNAS!
phenols	1	5110(0.011432491139819366)	#SAKNAS!	2548 (0.033)
phenomena	1	5110(0.011432491139819366)	2359 (0.034)	#SAKNAS!

phenomena	1	5110(0.011432491139819366)	2359 (0.034)	#SAKNAS!
phenomenalogic	1	5110(0.011432491139819366)	#SAKNAS!	2548 (0.033)
phenomenon	1	2214(0.04572996455927746)	2359 (0.034)	2548 (0.033)
phenotypic	1	2214(0.04572996455927746)	2359 (0.034)	1668 (0.066)
phenylalanine	1	5110(0.011432491139819366)	2359 (0.034)	#SAKNAS!
phenylalanine	1	5110(0.011432491139819366)	2359 (0.034)	#SAKNAS!
phenylpropanolamine	1	5110(0.011432491139819366)	2359 (0.034)	#SAKNAS!
phenylpropanolamine	1	5110(0.011432491139819366)	2359 (0.034)	#SAKNAS!
pheromone	1	3437(0.02286498227963873)	2359 (0.034)	#SAKNAS!
pheromones	1	5110(0.011432491139819366)	2359 (0.034)	#SAKNAS!
pheromones	1	5110(0.011432491139819366)	2359 (0.034)	#SAKNAS!
philadelphia	1	5110(0.011432491139819366)	#SAKNAS!	#SAKNAS!
philippine	1	5110(0.011432491139819366)	2359 (0.034)	#SAKNAS!
philippine	1	5110(0.011432491139819366)	2359 (0.034)	#SAKNAS!
philosophy	1	5110(0.011432491139819366)	#SAKNAS!	#SAKNAS!
phinest	1	5110(0.011432491139819366)	#SAKNAS!	2548 (0.033)
ph-metric	1	5110(0.011432491139819366)	2359 (0.034)	#SAKNAS!
ph-metric	1	5110(0.011432491139819366)	2359 (0.034)	#SAKNAS!
phnom	1	5110(0.011432491139819366)	#SAKNAS!	2548 (0.033)
phobia	1	5110(0.011432491139819366)	#SAKNAS!	2548 (0.033)
phones	1	5110(0.011432491139819366)	2359 (0.034)	#SAKNAS!
phones	1	5110(0.011432491139819366)	2359 (0.034)	#SAKNAS!
phoniatrics	1	5110(0.011432491139819366)	#SAKNAS!	2548 (0.033)
phosphate	1	5110(0.011432491139819366)	#SAKNAS!	#SAKNAS!
phosphoglycerate	1	5110(0.011432491139819366)	2359 (0.034)	#SAKNAS!
phosphoglycerate	1	5110(0.011432491139819366)	2359 (0.034)	#SAKNAS!
phospholipases	1	5110(0.011432491139819366)	#SAKNAS!	2548 (0.033)
phosphorus-nutrition	1	5110(0.011432491139819366)	2359 (0.034)	#SAKNAS!
phosphorus-nutrition	1	5110(0.011432491139819366)	2359 (0.034)	#SAKNAS!
photography-aided	1	5110(0.011432491139819366)	#SAKNAS!	#SAKNAS!
photosynthesis	1	5110(0.011432491139819366)	2359 (0.034)	#SAKNAS!
photosynthesis	1	5110(0.011432491139819366)	2359 (0.034)	#SAKNAS!
phototherapy	1	5110(0.011432491139819366)	#SAKNAS!	2548 (0.033)
phototroph	1	5110(0.011432491139819366)	2359 (0.034)	#SAKNAS!
phototroph	1	5110(0.011432491139819366)	2359 (0.034)	#SAKNAS!
phrenic	1	5110(0.011432491139819366)	2359 (0.034)	#SAKNAS!
phrenic	1	5110(0.011432491139819366)	2359 (0.034)	#SAKNAS!
phrenoesophagopexy	1	5110(0.011432491139819366)	#SAKNAS!	2548 (0.033)
phthalates	1	5110(0.011432491139819366)	#SAKNAS!	2548 (0.033)
phyllanthus	1	5110(0.011432491139819366)	#SAKNAS!	2548 (0.033)
phyllis	1	5110(0.011432491139819366)	2359 (0.034)	#SAKNAS!
phyllis	1	5110(0.011432491139819366)	2359 (0.034)	#SAKNAS!
phylloquinone	1	5110(0.011432491139819366)	2359 (0.034)	#SAKNAS!

phylloquinone	1 5110(0.011432491139819366)	2359 (0.034)	#SAKNAS!
phyllostomidae	1 5110(0.011432491139819366)	#SAKNAS!	2548 (0.033)
phyllotis	1 5110(0.011432491139819366)	2359 (0.034)	#SAKNAS!
phyllotis	1 5110(0.011432491139819366)	2359 (0.034)	#SAKNAS!
phylogenetically	1 5110(0.011432491139819366)	#SAKNAS!	2548 (0.033)
phylogeny	1 5110(0.011432491139819366)	2359 (0.034)	#SAKNAS!
phylogeny	1 5110(0.011432491139819366)	2359 (0.034)	#SAKNAS!
physically	1 2693(0.0342974734194581)	2359 (0.034)	#SAKNAS!
physician	1 2214(0.04572996455927746)	2359 (0.034)	1233 (0.1)
physiologic	1 2214(0.04572996455927746)	2359 (0.034)	2548 (0.033)
phytase	1 5110(0.011432491139819366)	2359 (0.034)	#SAKNAS!
phytase	1 5110(0.011432491139819366)	2359 (0.034)	#SAKNAS!
phytochemical	1 5110(0.011432491139819366)	2359 (0.034)	#SAKNAS!
phytochemical	1 5110(0.011432491139819366)	2359 (0.034)	#SAKNAS!
phytoflagellates	1 5110(0.011432491139819366)	#SAKNAS!	2548 (0.033)
phytogenic	1 5110(0.011432491139819366)	#SAKNAS!	#SAKNAS!
phytohormone-dependent	1 5110(0.011432491139819366)	#SAKNAS!	#SAKNAS!
phytomyxea	1 5110(0.011432491139819366)	#SAKNAS!	#SAKNAS!
phytophagous	1 5110(0.011432491139819366)	#SAKNAS!	2548 (0.033)
phytosterol	1 5110(0.011432491139819366)	2359 (0.034)	#SAKNAS!
phytosterol	1 5110(0.011432491139819366)	2359 (0.034)	#SAKNAS!
piau	1 5110(0.011432491139819366)	2359 (0.034)	#SAKNAS!
piau	1 5110(0.011432491139819366)	2359 (0.034)	#SAKNAS!
pied	1 5110(0.011432491139819366)	#SAKNAS!	#SAKNAS!
pieper	1 5110(0.011432491139819366)	2359 (0.034)	#SAKNAS!
pieper	1 5110(0.011432491139819366)	2359 (0.034)	#SAKNAS!
pies	1 5110(0.011432491139819366)	#SAKNAS!	2548 (0.033)
piezo1	1 5110(0.011432491139819366)	#SAKNAS!	#SAKNAS!
pig	1 5110(0.011432491139819366)	#SAKNAS!	#SAKNAS!
pigeons	1 5110(0.011432491139819366)	2359 (0.034)	#SAKNAS!
pigeons	1 5110(0.011432491139819366)	2359 (0.034)	#SAKNAS!
piglet	1 5110(0.011432491139819366)	#SAKNAS!	#SAKNAS!
pigra	1 3437(0.022864982279638730)	2359 (0.034)	2548 (0.033)
pike	1 5110(0.011432491139819366)	#SAKNAS!	2548 (0.033)
pikeperch	1 5110(0.011432491139819366)	2359 (0.034)	#SAKNAS!
pikeperch	1 5110(0.011432491139819366)	2359 (0.034)	#SAKNAS!
pilah	1 5110(0.011432491139819366)	#SAKNAS!	2548 (0.033)
pill-induced	1 5110(0.011432491139819366)	2359 (0.034)	#SAKNAS!
pill-induced	1 5110(0.011432491139819366)	2359 (0.034)	#SAKNAS!
pilocarpine	1 5110(0.011432491139819366)	2359 (0.034)	#SAKNAS!
pilocarpine	1 5110(0.011432491139819366)	2359 (0.034)	#SAKNAS!
pilosocereus-arrabidae	1 5110(0.011432491139819366)	2359 (0.034)	#SAKNAS!
pilosocereus-arrabidae	1 5110(0.011432491139819366)	2359 (0.034)	#SAKNAS!

piloting	1 5110(0.011432491139819366)	#SAKNAS!	2548 (0.033)
pilot-randomized	1 5110(0.011432491139819366)	#SAKNAS!	#SAKNAS!
pilots	1 5110(0.011432491139819366)	#SAKNAS!	2548 (0.033)
pilot-study	1 5110(0.011432491139819366)	#SAKNAS!	2548 (0.033)
pinghu	1 5110(0.011432491139819366)	#SAKNAS!	2548 (0.033)
piperine	1 5110(0.011432491139819366)	#SAKNAS!	2548 (0.033)
piperita	1 5110(0.011432491139819366)	#SAKNAS!	2548 (0.033)
pipiens	1 5110(0.011432491139819366)	#SAKNAS!	2548 (0.033)
pirarucu	1 5110(0.011432491139819366)	#SAKNAS!	2548 (0.033)
piriform	1 5110(0.011432491139819366)	2359 (0.034)	#SAKNAS!
piriform	1 5110(0.011432491139819366)	2359 (0.034)	#SAKNAS!
pisa	1 5110(0.011432491139819366)	#SAKNAS!	#SAKNAS!
pisaura	1 5110(0.011432491139819366)	2359 (0.034)	#SAKNAS!
pisaura	1 5110(0.011432491139819366)	2359 (0.034)	#SAKNAS!
pisauridae	1 5110(0.011432491139819366)	2359 (0.034)	#SAKNAS!
pisauridae	1 5110(0.011432491139819366)	2359 (0.034)	#SAKNAS!
pisces	1 5110(0.011432491139819366)	2359 (0.034)	#SAKNAS!
pisces	1 5110(0.011432491139819366)	2359 (0.034)	#SAKNAS!
piscivory	1 5110(0.011432491139819366)	#SAKNAS!	2548 (0.033)
pisonii	1 5110(0.011432491139819366)	2359 (0.034)	#SAKNAS!
pisonii	1 5110(0.011432491139819366)	2359 (0.034)	#SAKNAS!
pistillata	1 5110(0.011432491139819366)	2359 (0.034)	#SAKNAS!
pistillata	1 5110(0.011432491139819366)	2359 (0.034)	#SAKNAS!
pit	1 5110(0.011432491139819366)	#SAKNAS!	2548 (0.033)
pit-and-fissure	1 5110(0.011432491139819366)	#SAKNAS!	2548 (0.033)
pittsburgh	1 5110(0.011432491139819366)	2359 (0.034)	#SAKNAS!
pittsburgh	1 5110(0.011432491139819366)	2359 (0.034)	#SAKNAS!
pku	1 5110(0.011432491139819366)	2359 (0.034)	#SAKNAS!
pku	1 5110(0.011432491139819366)	2359 (0.034)	#SAKNAS!
place	1 5110(0.011432491139819366)	#SAKNAS!	#SAKNAS!
placebo	1 5110(0.011432491139819366)	#SAKNAS!	2548 (0.033)
placebo-control	1 5110(0.011432491139819366)	#SAKNAS!	#SAKNAS!
placental	1 2693(0.0342974734194581)	2359 (0.034)	#SAKNAS!
places	1 5110(0.011432491139819366)	2359 (0.034)	#SAKNAS!
places	1 5110(0.011432491139819366)	2359 (0.034)	#SAKNAS!
placetron	1 5110(0.011432491139819366)	2359 (0.034)	#SAKNAS!
placetron	1 5110(0.011432491139819366)	2359 (0.034)	#SAKNAS!
placopecten	1 5110(0.011432491139819366)	2359 (0.034)	#SAKNAS!
placopecten	1 5110(0.011432491139819366)	2359 (0.034)	#SAKNAS!
placozoans	1 5110(0.011432491139819366)	#SAKNAS!	#SAKNAS!
plague	1 5110(0.011432491139819366)	2359 (0.034)	#SAKNAS!
plague	1 5110(0.011432491139819366)	2359 (0.034)	#SAKNAS!
plaice	1 5110(0.011432491139819366)	2359 (0.034)	#SAKNAS!

plaice	1	5110(0.011432491139819366)	2359 (0.034)	#SAKNAS!
plain	1	2214(0.04572996455927746)	2359 (0.034)	1668 (0.066)
plakobranchus	1	5110(0.011432491139819366)	2359 (0.034)	#SAKNAS!
plakobranchus	1	5110(0.011432491139819366)	2359 (0.034)	#SAKNAS!
planing	1	5110(0.011432491139819366)	#SAKNAS!	#SAKNAS!
planktivorous	1	5110(0.011432491139819366)	#SAKNAS!	2548 (0.033)
plankton	1	5110(0.011432491139819366)	2359 (0.034)	#SAKNAS!
plankton	1	5110(0.011432491139819366)	2359 (0.034)	#SAKNAS!
planning	1	5110(0.011432491139819366)	#SAKNAS!	2548 (0.033)
plantations	1	5110(0.011432491139819366)	2359 (0.034)	#SAKNAS!
plantations	1	5110(0.011432491139819366)	2359 (0.034)	#SAKNAS!
plant-chemical	1	5110(0.011432491139819366)	2359 (0.034)	#SAKNAS!
plant-chemical	1	5110(0.011432491139819366)	2359 (0.034)	#SAKNAS!
plantcutter	1	5110(0.011432491139819366)	2359 (0.034)	#SAKNAS!
plantcutter	1	5110(0.011432491139819366)	2359 (0.034)	#SAKNAS!
plant-density	1	5110(0.011432491139819366)	2359 (0.034)	#SAKNAS!
plant-density	1	5110(0.011432491139819366)	2359 (0.034)	#SAKNAS!
plant-ectotherm	1	5110(0.011432491139819366)	#SAKNAS!	#SAKNAS!
plant-flower	1	5110(0.011432491139819366)	2359 (0.034)	#SAKNAS!
plant-flower	1	5110(0.011432491139819366)	2359 (0.034)	#SAKNAS!
plant-mediated	1	5110(0.011432491139819366)	2359 (0.034)	#SAKNAS!
plant-mediated	1	5110(0.011432491139819366)	2359 (0.034)	#SAKNAS!
planus	1	2693(0.0342974734194581)	2359 (0.034)	2548 (0.033)
plaques	1	5110(0.011432491139819366)	#SAKNAS!	#SAKNAS!
plasma-mass	1	5110(0.011432491139819366)	#SAKNAS!	2548 (0.033)
plasmatic	1	5110(0.011432491139819366)	#SAKNAS!	2548 (0.033)
plasminogen	1	3437(0.02286498227963873)	2359 (0.034)	#SAKNAS!
plasmodium	1	5110(0.011432491139819366)	2359 (0.034)	#SAKNAS!
plasmodium	1	5110(0.011432491139819366)	2359 (0.034)	#SAKNAS!
plastic-covered	1	5110(0.011432491139819366)	2359 (0.034)	#SAKNAS!
plastic-covered	1	5110(0.011432491139819366)	2359 (0.034)	#SAKNAS!
plate	1	2693(0.0342974734194581)	2359 (0.034)	2548 (0.033)
plateau	1	5110(0.011432491139819366)	2359 (0.034)	#SAKNAS!
plateau	1	5110(0.011432491139819366)	2359 (0.034)	#SAKNAS!
platelet-rich	1	5110(0.011432491139819366)	#SAKNAS!	#SAKNAS!
platypus	1	5110(0.011432491139819366)	2359 (0.034)	#SAKNAS!
platypus	1	5110(0.011432491139819366)	2359 (0.034)	#SAKNAS!
plausibility	1	5110(0.011432491139819366)	2359 (0.034)	#SAKNAS!
plausibility	1	5110(0.011432491139819366)	2359 (0.034)	#SAKNAS!
play	1	2693(0.0342974734194581)	2359 (0.034)	2548 (0.033)
players	1	5110(0.011432491139819366)	#SAKNAS!	2548 (0.033)
playing	1	5110(0.011432491139819366)	#SAKNAS!	2548 (0.033)
pledgeted	1	5110(0.011432491139819366)	#SAKNAS!	2548 (0.033)

pleiotropy	1	5110(0.011432491139819366)	#SAKNAS!	2548 (0.033)
pleistocene	1	5110(0.011432491139819366)	#SAKNAS!	2548 (0.033)
plicatilis	1	5110(0.011432491139819366)	#SAKNAS!	#SAKNAS!
plr	1	5110(0.011432491139819366)	#SAKNAS!	#SAKNAS!
plummer-vinson	1	2693(0.0342974734194581)	2359 (0.034)	2548 (0.033)
plus	1	1335(0.09145992911855493)	2359 (0.034)	816 (0.16)
plutella	1	5110(0.011432491139819366)	#SAKNAS!	2548 (0.033)
plutellidae	1	5110(0.011432491139819366)	#SAKNAS!	2548 (0.033)
plymouth	1	5110(0.011432491139819366)	#SAKNAS!	2548 (0.033)
pm	1	5110(0.011432491139819366)	2359 (0.034)	#SAKNAS!
pm	1	5110(0.011432491139819366)	2359 (0.034)	#SAKNAS!
pneumatic	1	3437(0.02286498227963873)	2359 (0.034)	#SAKNAS!
pneumoperitoneum	1	5110(0.011432491139819366)	2359 (0.034)	#SAKNAS!
pneumoperitoneum	1	5110(0.011432491139819366)	2359 (0.034)	#SAKNAS!
pocketing-results	1	5110(0.011432491139819366)	#SAKNAS!	2548 (0.033)
pockets	1	3437(0.02286498227963873)	2359 (0.034)	2548 (0.033)
poculata	1	5110(0.011432491139819366)	#SAKNAS!	2548 (0.033)
pod	1	5110(0.011432491139819366)	#SAKNAS!	2548 (0.033)
pods	1	5110(0.011432491139819366)	#SAKNAS!	2548 (0.033)
poem	1	3437(0.02286498227963873)	2359 (0.034)	#SAKNAS!
poisson	1	5110(0.011432491139819366)	#SAKNAS!	2548 (0.033)
poland-dental	1	5110(0.011432491139819366)	#SAKNAS!	#SAKNAS!
poles	1	5110(0.011432491139819366)	#SAKNAS!	2548 (0.033)
police	1	5110(0.011432491139819366)	2359 (0.034)	#SAKNAS!
police	1	5110(0.011432491139819366)	2359 (0.034)	#SAKNAS!
policies	1	5110(0.011432491139819366)	#SAKNAS!	2548 (0.033)
polish	1	1894(0.05716245569909683)	2359 (0.034)	1668 (0.066)
polistes	1	5110(0.011432491139819366)	#SAKNAS!	#SAKNAS!
political	1	5110(0.011432491139819366)	#SAKNAS!	2548 (0.033)
pollen-feeding	1	5110(0.011432491139819366)	#SAKNAS!	#SAKNAS!
pollinator	1	5110(0.011432491139819366)	#SAKNAS!	2548 (0.033)
pollinators	1	5110(0.011432491139819366)	#SAKNAS!	2548 (0.033)
pollutant	1	5110(0.011432491139819366)	#SAKNAS!	#SAKNAS!
pollutants	1	5110(0.011432491139819366)	2359 (0.034)	#SAKNAS!
pollutants	1	5110(0.011432491139819366)	2359 (0.034)	#SAKNAS!
pollution	1	5110(0.011432491139819366)	#SAKNAS!	2548 (0.033)
polsenior	1	5110(0.011432491139819366)	#SAKNAS!	2548 (0.033)
polski	1	5110(0.011432491139819366)	#SAKNAS!	#SAKNAS!
poly	1	5110(0.011432491139819366)	#SAKNAS!	#SAKNAS!
polychaete	1	5110(0.011432491139819366)	#SAKNAS!	2548 (0.033)
polychaetes	1	5110(0.011432491139819366)	2359 (0.034)	#SAKNAS!
polychaetes	1	5110(0.011432491139819366)	2359 (0.034)	#SAKNAS!
polychemotherapy	1	5110(0.011432491139819366)	2359 (0.034)	#SAKNAS!

polychemotherapy	1	5110(0.011432491139819366)	2359 (0.034)	#SAKNAS!
polyester	1	5110(0.011432491139819366)	2359 (0.034)	#SAKNAS!
polyester	1	5110(0.011432491139819366)	2359 (0.034)	#SAKNAS!
polyfluorinated	1	5110(0.011432491139819366)	#SAKNAS!	2548 (0.033)
polygonatum	1	5110(0.011432491139819366)	#SAKNAS!	#SAKNAS!
polylectic	1	5110(0.011432491139819366)	#SAKNAS!	2548 (0.033)
polymorpha	1	3437(0.02286498227963873)	2359 (0.034)	2548 (0.033)
polymorphic	1	5110(0.011432491139819366)	#SAKNAS!	2548 (0.033)
polypeptide	1	5110(0.011432491139819366)	#SAKNAS!	#SAKNAS!
polypropylene	1	5110(0.011432491139819366)	2359 (0.034)	#SAKNAS!
polypropylene	1	5110(0.011432491139819366)	2359 (0.034)	#SAKNAS!
polysaccharides	1	5110(0.011432491139819366)	#SAKNAS!	#SAKNAS!
polysomnographic	1	5110(0.011432491139819366)	2359 (0.034)	#SAKNAS!
polysomnographic	1	5110(0.011432491139819366)	2359 (0.034)	#SAKNAS!
polysubstance	1	5110(0.011432491139819366)	#SAKNAS!	2548 (0.033)
poly-traumatized	1	5110(0.011432491139819366)	2359 (0.034)	#SAKNAS!
poly-traumatized	1	5110(0.011432491139819366)	2359 (0.034)	#SAKNAS!
pomerania	1	5110(0.011432491139819366)	2359 (0.034)	#SAKNAS!
pomerania	1	5110(0.011432491139819366)	2359 (0.034)	#SAKNAS!
pompe	1	2214(0.04572996455927746)	2359 (0.034)	2548 (0.033)
pon1	1	5110(0.011432491139819366)	#SAKNAS!	2548 (0.033)
pond	1	3437(0.02286498227963873)	2359 (0.034)	#SAKNAS!
ponderal	1	5110(0.011432491139819366)	#SAKNAS!	#SAKNAS!
ponderostat	1	5110(0.011432491139819366)	2359 (0.034)	#SAKNAS!
ponderostat	1	5110(0.011432491139819366)	2359 (0.034)	#SAKNAS!
ponds	1	5110(0.011432491139819366)	2359 (0.034)	#SAKNAS!
ponds	1	5110(0.011432491139819366)	2359 (0.034)	#SAKNAS!
pongo	1	5110(0.011432491139819366)	#SAKNAS!	2548 (0.033)
ponies	1	5110(0.011432491139819366)	#SAKNAS!	#SAKNAS!
pontica	1	5110(0.011432491139819366)	#SAKNAS!	2548 (0.033)
pony	1	5110(0.011432491139819366)	2359 (0.034)	#SAKNAS!
pony	1	5110(0.011432491139819366)	2359 (0.034)	#SAKNAS!
poorer	1	3437(0.02286498227963873)	2359 (0.034)	2548 (0.033)
population-a	1	5110(0.011432491139819366)	#SAKNAS!	#SAKNAS!
population-representative	1	5110(0.011432491139819366)	#SAKNAS!	2548 (0.033)
populi	1	5110(0.011432491139819366)	#SAKNAS!	#SAKNAS!
por	1	5110(0.011432491139819366)	#SAKNAS!	#SAKNAS!
porcine	1	5110(0.011432491139819366)	#SAKNAS!	2548 (0.033)
porphyria	1	5110(0.011432491139819366)	#SAKNAS!	#SAKNAS!
portable	1	3437(0.02286498227963873)	2359 (0.034)	#SAKNAS!
portion	1	3437(0.02286498227963873)	2359 (0.034)	2548 (0.033)
portioning	1	5110(0.011432491139819366)	#SAKNAS!	2548 (0.033)
portugal	1	3437(0.02286498227963873)	2359 (0.034)	#SAKNAS!

portunidae	1	5110(0.011432491139819366)	2359 (0.034)	#SAKNAS!
portunidae	1	5110(0.011432491139819366)	2359 (0.034)	#SAKNAS!
pose	1	5110(0.011432491139819366)	#SAKNAS!	#SAKNAS!
posidonia	1	5110(0.011432491139819366)	#SAKNAS!	2548 (0.033)
positional	1	5110(0.011432491139819366)	#SAKNAS!	2548 (0.033)
positioning	1	5110(0.011432491139819366)	2359 (0.034)	#SAKNAS!
positioning	1	5110(0.011432491139819366)	2359 (0.034)	#SAKNAS!
possessing	1	5110(0.011432491139819366)	#SAKNAS!	#SAKNAS!
possibilities	1	5110(0.011432491139819366)	2359 (0.034)	#SAKNAS!
possibilities	1	5110(0.011432491139819366)	2359 (0.034)	#SAKNAS!
possibility	1	5110(0.011432491139819366)	2359 (0.034)	#SAKNAS!
possibility	1	5110(0.011432491139819366)	2359 (0.034)	#SAKNAS!
possum	1	5110(0.011432491139819366)	2359 (0.034)	#SAKNAS!
possum	1	5110(0.011432491139819366)	2359 (0.034)	#SAKNAS!
possums	1	5110(0.011432491139819366)	2359 (0.034)	#SAKNAS!
possums	1	5110(0.011432491139819366)	2359 (0.034)	#SAKNAS!
postbariatric	1	5110(0.011432491139819366)	#SAKNAS!	2548 (0.033)
post-bariatric	1	5110(0.011432491139819366)	#SAKNAS!	#SAKNAS!
post-carotid	1	5110(0.011432491139819366)	2359 (0.034)	#SAKNAS!
post-carotid	1	5110(0.011432491139819366)	2359 (0.034)	#SAKNAS!
post-conflict	1	5110(0.011432491139819366)	#SAKNAS!	2548 (0.033)
postcontact	1	5110(0.011432491139819366)	2359 (0.034)	#SAKNAS!
postcontact	1	5110(0.011432491139819366)	2359 (0.034)	#SAKNAS!
post-discharge	1	5110(0.011432491139819366)	#SAKNAS!	#SAKNAS!
post-earthquake	1	5110(0.011432491139819366)	2359 (0.034)	#SAKNAS!
post-earthquake	1	5110(0.011432491139819366)	2359 (0.034)	#SAKNAS!
postegression	1	5110(0.011432491139819366)	#SAKNAS!	2548 (0.033)
posterior	1	710(0.19435234937692925)	2359 (0.034)	584 (0.23)
post-extubated	1	5110(0.011432491139819366)	#SAKNAS!	#SAKNAS!
post-feeding	1	5110(0.011432491139819366)	2359 (0.034)	#SAKNAS!
post-feeding	1	5110(0.011432491139819366)	2359 (0.034)	#SAKNAS!
postfundoplication	1	5110(0.011432491139819366)	2359 (0.034)	#SAKNAS!
postfundoplication	1	5110(0.011432491139819366)	2359 (0.034)	#SAKNAS!
post-hospitalisation	1	5110(0.011432491139819366)	2359 (0.034)	#SAKNAS!
post-hospitalisation	1	5110(0.011432491139819366)	2359 (0.034)	#SAKNAS!
posthospitalization	1	5110(0.011432491139819366)	2359 (0.034)	#SAKNAS!
posthospitalization	1	5110(0.011432491139819366)	2359 (0.034)	#SAKNAS!
postingestion	1	5110(0.011432491139819366)	#SAKNAS!	2548 (0.033)
postingestive-driven	1	5110(0.011432491139819366)	#SAKNAS!	#SAKNAS!
post-intervention	1	5110(0.011432491139819366)	#SAKNAS!	2548 (0.033)
postintubation	1	5110(0.011432491139819366)	#SAKNAS!	#SAKNAS!
post-irradiated	1	5110(0.011432491139819366)	#SAKNAS!	2548 (0.033)
post-irradiation	1	5110(0.011432491139819366)	#SAKNAS!	#SAKNAS!

postischemic	1	5110(0.011432491139819366)	#SAKNAS!	#SAKNAS!
post-ischemic	1	5110(0.011432491139819366)	#SAKNAS!	#SAKNAS!
post-laparoscopic	1	5110(0.011432491139819366)	#SAKNAS!	2548 (0.033)
post-larvae	1	5110(0.011432491139819366)	#SAKNAS!	2548 (0.033)
post-larval	1	5110(0.011432491139819366)	2359 (0.034)	#SAKNAS!
post-larval	1	5110(0.011432491139819366)	2359 (0.034)	#SAKNAS!
postoesophageal	1	5110(0.011432491139819366)	#SAKNAS!	#SAKNAS!
postoesophagectomy	1	5110(0.011432491139819366)	#SAKNAS!	2548 (0.033)
postprandial	1	2693(0.0342974734194581)	2359 (0.034)	#SAKNAS!
post-prandial	1	5110(0.011432491139819366)	#SAKNAS!	2548 (0.033)
post-procedural	1	5110(0.011432491139819366)	#SAKNAS!	2548 (0.033)
postprocessing	1	5110(0.011432491139819366)	#SAKNAS!	#SAKNAS!
post-pyloric	1	5110(0.011432491139819366)	#SAKNAS!	#SAKNAS!
postradiation	1	5110(0.011432491139819366)	#SAKNAS!	2548 (0.033)
post-soviet	1	5110(0.011432491139819366)	#SAKNAS!	#SAKNAS!
poststroke	1	787(0.1714873670972905)	2359 (0.034)	999 (0.13)
post-surgery	1	5110(0.011432491139819366)	#SAKNAS!	#SAKNAS!
postsurgical	1	5110(0.011432491139819366)	2359 (0.034)	#SAKNAS!
postsurgical	1	5110(0.011432491139819366)	2359 (0.034)	#SAKNAS!
post-surgical	1	5110(0.011432491139819366)	#SAKNAS!	2548 (0.033)
postsynaptic	1	5110(0.011432491139819366)	2359 (0.034)	#SAKNAS!
postsynaptic	1	5110(0.011432491139819366)	2359 (0.034)	#SAKNAS!
post-tracheostomy	1	5110(0.011432491139819366)	#SAKNAS!	2548 (0.033)
posttraumatic	1	5110(0.011432491139819366)	#SAKNAS!	2548 (0.033)
postulate	1	5110(0.011432491139819366)	#SAKNAS!	2548 (0.033)
postures	1	5110(0.011432491139819366)	#SAKNAS!	2548 (0.033)
postweaned	1	5110(0.011432491139819366)	#SAKNAS!	#SAKNAS!
postweaning	1	5110(0.011432491139819366)	#SAKNAS!	#SAKNAS!
potentialities	1	5110(0.011432491139819366)	2359 (0.034)	#SAKNAS!
potentialities	1	5110(0.011432491139819366)	2359 (0.034)	#SAKNAS!
potentially	1	1111(0.11432491139819366)	2359 (0.034)	1233 (0.1)
potentials	1	5110(0.011432491139819366)	2359 (0.034)	#SAKNAS!
potentials	1	5110(0.011432491139819366)	2359 (0.034)	#SAKNAS!
poterioochromonas	1	5110(0.011432491139819366)	#SAKNAS!	#SAKNAS!
poterioochromonas-malhamens	1	5110(0.011432491139819366)	2359 (0.034)	#SAKNAS!
poterioochromonas-malhamens	1	5110(0.011432491139819366)	2359 (0.034)	#SAKNAS!
potocki-lupski	1	5110(0.011432491139819366)	2359 (0.034)	#SAKNAS!
potocki-lupski	1	5110(0.011432491139819366)	2359 (0.034)	#SAKNAS!
pouch	1	3437(0.02286498227963873)	2359 (0.034)	2548 (0.033)
poultry	1	2693(0.0342974734194581)	2359 (0.034)	#SAKNAS!
poults	1	5110(0.011432491139819366)	2359 (0.034)	#SAKNAS!
poults	1	5110(0.011432491139819366)	2359 (0.034)	#SAKNAS!
povidone-iodine	1	5110(0.011432491139819366)	2359 (0.034)	#SAKNAS!

povidone-iodine	1	5110(0.011432491139819366)	2359 (0.034)	#SAKNAS!
powder-based	1	5110(0.011432491139819366)	#SAKNAS!	#SAKNAS!
powdery	1	5110(0.011432491139819366)	#SAKNAS!	2548 (0.033)
power	1	2693(0.0342974734194581)	2359 (0.034)	#SAKNAS!
ppar	1	5110(0.011432491139819366)	#SAKNAS!	2548 (0.033)
ppk	1	5110(0.011432491139819366)	2359 (0.034)	#SAKNAS!
ppk	1	5110(0.011432491139819366)	2359 (0.034)	#SAKNAS!
pr	1	5110(0.011432491139819366)	#SAKNAS!	2548 (0.033)
practice-based	1	3437(0.02286498227963873)	2359 (0.034)	#SAKNAS!
practise	1	5110(0.011432491139819366)	#SAKNAS!	2548 (0.033)
practitioner	1	5110(0.011432491139819366)	2359 (0.034)	#SAKNAS!
practitioner	1	5110(0.011432491139819366)	2359 (0.034)	#SAKNAS!
practitioner-dentist	1	5110(0.011432491139819366)	#SAKNAS!	2548 (0.033)
practitioners	1	1485(0.08002743797873557)	2359 (0.034)	1233 (0.1)
prandial	1	5110(0.011432491139819366)	#SAKNAS!	#SAKNAS!
pravastatin	1	5110(0.011432491139819366)	#SAKNAS!	2548 (0.033)
prawn	1	5110(0.011432491139819366)	2359 (0.034)	#SAKNAS!
prawn	1	5110(0.011432491139819366)	2359 (0.034)	#SAKNAS!
praying	1	5110(0.011432491139819366)	#SAKNAS!	2548 (0.033)
pre-adolescents	1	5110(0.011432491139819366)	2359 (0.034)	#SAKNAS!
pre-adolescents	1	5110(0.011432491139819366)	2359 (0.034)	#SAKNAS!
prealbumin	1	5110(0.011432491139819366)	#SAKNAS!	#SAKNAS!
pre-and	1	5110(0.011432491139819366)	#SAKNAS!	2548 (0.033)
precancerous	1	5110(0.011432491139819366)	2359 (0.034)	#SAKNAS!
precancerous	1	5110(0.011432491139819366)	2359 (0.034)	#SAKNAS!
preceramic	1	5110(0.011432491139819366)	#SAKNAS!	#SAKNAS!
precious	1	5110(0.011432491139819366)	#SAKNAS!	#SAKNAS!
precision	1	2214(0.04572996455927746)	2359 (0.034)	2548 (0.033)
preclinical	1	5110(0.011432491139819366)	#SAKNAS!	2548 (0.033)
pre-columbian	1	5110(0.011432491139819366)	#SAKNAS!	2548 (0.033)
precursors	1	5110(0.011432491139819366)	2359 (0.034)	#SAKNAS!
precursors	1	5110(0.011432491139819366)	2359 (0.034)	#SAKNAS!
predaceous	1	5110(0.011432491139819366)	#SAKNAS!	2548 (0.033)
predator-prey	1	5110(0.011432491139819366)	2359 (0.034)	#SAKNAS!
predator-prey	1	5110(0.011432491139819366)	2359 (0.034)	#SAKNAS!
predators	1	2214(0.04572996455927746)	2359 (0.034)	1668 (0.066)
prediagnostic	1	5110(0.011432491139819366)	2359 (0.034)	#SAKNAS!
prediagnostic	1	5110(0.011432491139819366)	2359 (0.034)	#SAKNAS!
predict-cp	1	5110(0.011432491139819366)	#SAKNAS!	2548 (0.033)
predictions	1	5110(0.011432491139819366)	#SAKNAS!	2548 (0.033)
predisposition	1	5110(0.011432491139819366)	#SAKNAS!	#SAKNAS!
predoctoral	1	5110(0.011432491139819366)	2359 (0.034)	#SAKNAS!
predoctoral	1	5110(0.011432491139819366)	2359 (0.034)	#SAKNAS!

pre-doctoral	1	5110(0.011432491139819366)	#SAKNAS!	#SAKNAS!
predominantly	1	5110(0.011432491139819366)	#SAKNAS!	2548 (0.033)
predyces	1	5110(0.011432491139819366)	#SAKNAS!	#SAKNAS!
predyces簣	1	3437(0.02286498227963873)	2359 (0.034)	2548 (0.033)
predyscore	1	5110(0.011432491139819366)	#SAKNAS!	2548 (0.033)
preeclampsia	1	3437(0.02286498227963873)	2359 (0.034)	#SAKNAS!
preeclampsia-a	1	5110(0.011432491139819366)	#SAKNAS!	#SAKNAS!
pre-existing	1	3437(0.02286498227963873)	2359 (0.034)	#SAKNAS!
prefecture	1	5110(0.011432491139819366)	#SAKNAS!	2548 (0.033)
pre-feeding	1	5110(0.011432491139819366)	#SAKNAS!	2548 (0.033)
prefer	1	5110(0.011432491139819366)	#SAKNAS!	#SAKNAS!
preferable	1	5110(0.011432491139819366)	2359 (0.034)	#SAKNAS!
preferable	1	5110(0.011432491139819366)	2359 (0.034)	#SAKNAS!
preferentially	1	5110(0.011432491139819366)	2359 (0.034)	#SAKNAS!
preferentially	1	5110(0.011432491139819366)	2359 (0.034)	#SAKNAS!
pre-frail	1	5110(0.011432491139819366)	#SAKNAS!	#SAKNAS!
prefrailty	1	5110(0.011432491139819366)	#SAKNAS!	#SAKNAS!
prehabilitation	1	5110(0.011432491139819366)	#SAKNAS!	#SAKNAS!
prehaps	1	5110(0.011432491139819366)	#SAKNAS!	#SAKNAS!
prehispanic	1	5110(0.011432491139819366)	2359 (0.034)	#SAKNAS!
prehispanic	1	5110(0.011432491139819366)	2359 (0.034)	#SAKNAS!
pre-hospitalization	1	5110(0.011432491139819366)	#SAKNAS!	#SAKNAS!
preimaginal	1	5110(0.011432491139819366)	#SAKNAS!	2548 (0.033)
pre-infestation	1	5110(0.011432491139819366)	#SAKNAS!	2548 (0.033)
pre-kindergarten	1	5110(0.011432491139819366)	#SAKNAS!	#SAKNAS!
pre-lgm	1	5110(0.011432491139819366)	#SAKNAS!	2548 (0.033)
preload	1	5110(0.011432491139819366)	2359 (0.034)	#SAKNAS!
preload	1	5110(0.011432491139819366)	2359 (0.034)	#SAKNAS!
preloads	1	5110(0.011432491139819366)	2359 (0.034)	#SAKNAS!
preloads	1	5110(0.011432491139819366)	2359 (0.034)	#SAKNAS!
premastication	1	5110(0.011432491139819366)	#SAKNAS!	2548 (0.033)
prematurity	1	3437(0.02286498227963873)	2359 (0.034)	#SAKNAS!
premiefeed	1	5110(0.011432491139819366)	#SAKNAS!	#SAKNAS!
premorbid	1	5110(0.011432491139819366)	#SAKNAS!	2548 (0.033)
preparations	1	5110(0.011432491139819366)	#SAKNAS!	#SAKNAS!
preparatory	1	5110(0.011432491139819366)	#SAKNAS!	2548 (0.033)
prepartum	1	5110(0.011432491139819366)	#SAKNAS!	#SAKNAS!
pre-post	1	5110(0.011432491139819366)	#SAKNAS!	#SAKNAS!
preprosthetic	1	5110(0.011432491139819366)	#SAKNAS!	2548 (0.033)
prepubertal	1	5110(0.011432491139819366)	2359 (0.034)	#SAKNAS!
prepubertal	1	5110(0.011432491139819366)	2359 (0.034)	#SAKNAS!
pre-pubertal	1	5110(0.011432491139819366)	#SAKNAS!	2548 (0.033)
preschool-adapted	1	5110(0.011432491139819366)	#SAKNAS!	2548 (0.033)

preschool-age	1	5110(0.011432491139819366)	2359 (0.034)	#SAKNAS!
preschool-age	1	5110(0.011432491139819366)	2359 (0.034)	#SAKNAS!
preschool-children	1	3437(0.02286498227963873)	2359 (0.034)	#SAKNAS!
pre-schoolers	1	5110(0.011432491139819366)	#SAKNAS!	2548 (0.033)
prescribing	1	5110(0.011432491139819366)	#SAKNAS!	2548 (0.033)
preservation	1	1678(0.0685949468389162)	2359 (0.034)	1233 (0.1)
preserving	1	5110(0.011432491139819366)	2359 (0.034)	#SAKNAS!
preserving	1	5110(0.011432491139819366)	2359 (0.034)	#SAKNAS!
pressure-generation	1	5110(0.011432491139819366)	2359 (0.034)	#SAKNAS!
pressure-generation	1	5110(0.011432491139819366)	2359 (0.034)	#SAKNAS!
prestige-lp	1	5110(0.011432491139819366)	#SAKNAS!	#SAKNAS!
presyncope	1	5110(0.011432491139819366)	#SAKNAS!	2548 (0.033)
pre-term	1	5110(0.011432491139819366)	2359 (0.034)	#SAKNAS!
pre-term	1	5110(0.011432491139819366)	2359 (0.034)	#SAKNAS!
prethickened	1	5110(0.011432491139819366)	#SAKNAS!	2548 (0.033)
pre-thickened	1	5110(0.011432491139819366)	#SAKNAS!	2548 (0.033)
pretreating	1	5110(0.011432491139819366)	2359 (0.034)	#SAKNAS!
pretreating	1	5110(0.011432491139819366)	2359 (0.034)	#SAKNAS!
pretreatment	1	1894(0.05716245569909683)	2359 (0.034)	999 (0.13)
pre-treatment	1	5110(0.011432491139819366)	#SAKNAS!	#SAKNAS!
preventable	1	5110(0.011432491139819366)	2359 (0.034)	#SAKNAS!
preventable	1	5110(0.011432491139819366)	2359 (0.034)	#SAKNAS!
preventative	1	3437(0.02286498227963873)	2359 (0.034)	#SAKNAS!
preventing	1	1335(0.09145992911855493)	2359 (0.034)	816 (0.16)
prevertebral	1	2214(0.04572996455927746)	2359 (0.034)	2548 (0.033)
previous	1	2214(0.04572996455927746)	2359 (0.034)	2548 (0.033)
prevotella	1	3437(0.02286498227963873)	2359 (0.034)	2548 (0.033)
pre-weaned	1	5110(0.011432491139819366)	#SAKNAS!	2548 (0.033)
prey-choice	1	5110(0.011432491139819366)	#SAKNAS!	#SAKNAS!
primarily	1	5110(0.011432491139819366)	2359 (0.034)	#SAKNAS!
primarily	1	5110(0.011432491139819366)	2359 (0.034)	#SAKNAS!
primates	1	5110(0.011432491139819366)	2359 (0.034)	#SAKNAS!
primates	1	5110(0.011432491139819366)	2359 (0.034)	#SAKNAS!
prime	1	5110(0.011432491139819366)	#SAKNAS!	2548 (0.033)
primitive	1	5110(0.011432491139819366)	#SAKNAS!	2548 (0.033)
principal	1	5110(0.011432491139819366)	2359 (0.034)	#SAKNAS!
principal	1	5110(0.011432491139819366)	2359 (0.034)	#SAKNAS!
printability	1	5110(0.011432491139819366)	#SAKNAS!	#SAKNAS!
printable	1	5110(0.011432491139819366)	#SAKNAS!	#SAKNAS!
priorities	1	5110(0.011432491139819366)	#SAKNAS!	#SAKNAS!
prioritization	1	5110(0.011432491139819366)	#SAKNAS!	2548 (0.033)
priority	1	5110(0.011432491139819366)	#SAKNAS!	2548 (0.033)
pristionchus	1	5110(0.011432491139819366)	#SAKNAS!	2548 (0.033)

private	1	1678(0.0685949468389162)	2359 (0.034)	1668 (0.066)
pro	1	5110(0.011432491139819366)	#SAKNAS!	2548 (0.033)
proactive	1	2214(0.04572996455927746)	2359 (0.034)	2548 (0.033)
proanthocyanidin	1	5110(0.011432491139819366)	2359 (0.034)	#SAKNAS!
proanthocyanidin	1	5110(0.011432491139819366)	2359 (0.034)	#SAKNAS!
probability	1	5110(0.011432491139819366)	#SAKNAS!	2548 (0.033)
probes	1	5110(0.011432491139819366)	2359 (0.034)	#SAKNAS!
probes	1	5110(0.011432491139819366)	2359 (0.034)	#SAKNAS!
probiotic-kefir	1	5110(0.011432491139819366)	2359 (0.034)	#SAKNAS!
probiotic-kefir	1	5110(0.011432491139819366)	2359 (0.034)	#SAKNAS!
problem	1	2214(0.04572996455927746)	2359 (0.034)	2548 (0.033)
problematic	1	5110(0.011432491139819366)	#SAKNAS!	2548 (0.033)
proboscidea	1	5110(0.011432491139819366)	#SAKNAS!	2548 (0.033)
proboscis	1	5110(0.011432491139819366)	#SAKNAS!	2548 (0.033)
procalcitonin	1	5110(0.011432491139819366)	#SAKNAS!	2548 (0.033)
procedures-the	1	5110(0.011432491139819366)	#SAKNAS!	#SAKNAS!
proceedings	1	2693(0.0342974734194581)	2359 (0.034)	1668 (0.066)
procera	1	3437(0.02286498227963873)	2359 (0.034)	2548 (0.033)
prochilodus	1	5110(0.011432491139819366)	#SAKNAS!	#SAKNAS!
proctolin	1	5110(0.011432491139819366)	2359 (0.034)	#SAKNAS!
proctolin	1	5110(0.011432491139819366)	2359 (0.034)	#SAKNAS!
produce	1	3437(0.02286498227963873)	2359 (0.034)	#SAKNAS!
producer-grazer	1	5110(0.011432491139819366)	#SAKNAS!	2548 (0.033)
produces	1	3437(0.02286498227963873)	2359 (0.034)	2548 (0.033)
productive	1	2693(0.0342974734194581)	2359 (0.034)	1668 (0.066)
profession	1	5110(0.011432491139819366)	#SAKNAS!	#SAKNAS!
proficiency	1	5110(0.011432491139819366)	#SAKNAS!	#SAKNAS!
profiling	1	5110(0.011432491139819366)	#SAKNAS!	2548 (0.033)
profitability	1	3437(0.02286498227963873)	2359 (0.034)	#SAKNAS!
profound	1	5110(0.011432491139819366)	#SAKNAS!	2548 (0.033)
profoundly	1	5110(0.011432491139819366)	2359 (0.034)	#SAKNAS!
profoundly	1	5110(0.011432491139819366)	2359 (0.034)	#SAKNAS!
progas	1	5110(0.011432491139819366)	#SAKNAS!	2548 (0.033)
progenitor	1	2693(0.0342974734194581)	2359 (0.034)	2548 (0.033)
progeny	1	5110(0.011432491139819366)	2359 (0.034)	#SAKNAS!
progeny	1	5110(0.011432491139819366)	2359 (0.034)	#SAKNAS!
proglumide	1	5110(0.011432491139819366)	2359 (0.034)	#SAKNAS!
proglumide	1	5110(0.011432491139819366)	2359 (0.034)	#SAKNAS!
prognosis-related	1	5110(0.011432491139819366)	#SAKNAS!	2548 (0.033)
prognostication	1	5110(0.011432491139819366)	#SAKNAS!	2548 (0.033)
programs	1	896(0.14862238481765175)	2359 (0.034)	694 (0.2)
progress	1	1485(0.08002743797873557)	2359 (0.034)	2548 (0.033)
progressive	1	1894(0.05716245569909683)	2359 (0.034)	2548 (0.033)

prohibiting	1	5110(0.011432491139819366)	#SAKNAS!	#SAKNAS!
proinflammatory	1	2693(0.0342974734194581)	2359 (0.034)	2548 (0.033)
pro-inflammatory	1	3437(0.02286498227963873)	2359 (0.034)	2548 (0.033)
prokineticin	1	5110(0.011432491139819366)	#SAKNAS!	2548 (0.033)
prolactinoma	1	5110(0.011432491139819366)	#SAKNAS!	2548 (0.033)
proliferating	1	5110(0.011432491139819366)	2359 (0.034)	#SAKNAS!
proliferating	1	5110(0.011432491139819366)	2359 (0.034)	#SAKNAS!
prolongation	1	5110(0.011432491139819366)	#SAKNAS!	#SAKNAS!
prolongs	1	5110(0.011432491139819366)	2359 (0.034)	#SAKNAS!
prolongs	1	5110(0.011432491139819366)	2359 (0.034)	#SAKNAS!
prom	1	5110(0.011432491139819366)	#SAKNAS!	#SAKNAS!
prominent	1	5110(0.011432491139819366)	2359 (0.034)	#SAKNAS!
prominent	1	5110(0.011432491139819366)	2359 (0.034)	#SAKNAS!
promise	1	5110(0.011432491139819366)	#SAKNAS!	#SAKNAS!
proof	1	5110(0.011432491139819366)	#SAKNAS!	2548 (0.033)
prop	1	5110(0.011432491139819366)	2359 (0.034)	#SAKNAS!
prop	1	5110(0.011432491139819366)	2359 (0.034)	#SAKNAS!
propensity	1	787(0.1714873670972905)	2359 (0.034)	584 (0.23)
propensity-matched	1	5110(0.011432491139819366)	#SAKNAS!	2548 (0.033)
propensity-score	1	5110(0.011432491139819366)	#SAKNAS!	#SAKNAS!
proper	1	2693(0.0342974734194581)	2359 (0.034)	2548 (0.033)
prophylactic	1	743(0.18291985823710985)	2359 (0.034)	422 (0.33)
propionate	1	3437(0.02286498227963873)	2359 (0.034)	2548 (0.033)
propithecus	1	2693(0.0342974734194581)	2359 (0.034)	1668 (0.066)
propolis	1	3437(0.02286498227963873)	2359 (0.034)	2548 (0.033)
proportion	1	3437(0.02286498227963873)	2359 (0.034)	#SAKNAS!
proposal	1	2214(0.04572996455927746)	2359 (0.034)	2548 (0.033)
proposals	1	5110(0.011432491139819366)	2359 (0.034)	#SAKNAS!
proposals	1	5110(0.011432491139819366)	2359 (0.034)	#SAKNAS!
proposition	1	5110(0.011432491139819366)	2359 (0.034)	#SAKNAS!
proposition	1	5110(0.011432491139819366)	2359 (0.034)	#SAKNAS!
pro-resolving	1	5110(0.011432491139819366)	#SAKNAS!	#SAKNAS!
pro-saude	1	5110(0.011432491139819366)	#SAKNAS!	2548 (0.033)
proseal	1	5110(0.011432491139819366)	2359 (0.034)	#SAKNAS!
proseal	1	5110(0.011432491139819366)	2359 (0.034)	#SAKNAS!
prosobranch	1	5110(0.011432491139819366)	2359 (0.034)	#SAKNAS!
prosobranch	1	5110(0.011432491139819366)	2359 (0.034)	#SAKNAS!
prospective-study	1	5110(0.011432491139819366)	2359 (0.034)	#SAKNAS!
prospective-study	1	5110(0.011432491139819366)	2359 (0.034)	#SAKNAS!
prostaglandin	1	3437(0.02286498227963873)	2359 (0.034)	#SAKNAS!
prostaglandin-e2	1	5110(0.011432491139819366)	2359 (0.034)	#SAKNAS!
prostaglandin-e2	1	5110(0.011432491139819366)	2359 (0.034)	#SAKNAS!
prostate	1	5110(0.011432491139819366)	#SAKNAS!	2548 (0.033)

prostate-specific	1	5110(0.011432491139819366)	#SAKNAS!	2548 (0.033)
prosthodontically	1	5110(0.011432491139819366)	2359 (0.034)	#SAKNAS!
prosthodontically	1	5110(0.011432491139819366)	2359 (0.034)	#SAKNAS!
protease	1	3437(0.02286498227963873)	2359 (0.034)	2548 (0.033)
protectants	1	5110(0.011432491139819366)	2359 (0.034)	#SAKNAS!
protectants	1	5110(0.011432491139819366)	2359 (0.034)	#SAKNAS!
protection	1	2693(0.0342974734194581)	2359 (0.034)	1668 (0.066)
protective	1	966(0.1371898936778324)	2359 (0.034)	816 (0.16)
protects	1	5110(0.011432491139819366)	#SAKNAS!	2548 (0.033)
protein-1	1	5110(0.011432491139819366)	2359 (0.034)	#SAKNAS!
protein-1	1	5110(0.011432491139819366)	2359 (0.034)	#SAKNAS!
protein-2	1	5110(0.011432491139819366)	2359 (0.034)	#SAKNAS!
protein-2	1	5110(0.011432491139819366)	2359 (0.034)	#SAKNAS!
protein-alginate	1	5110(0.011432491139819366)	#SAKNAS!	#SAKNAS!
proteinase	1	5110(0.011432491139819366)	#SAKNAS!	2548 (0.033)
protein-associated	1	5110(0.011432491139819366)	2359 (0.034)	#SAKNAS!
protein-associated	1	5110(0.011432491139819366)	2359 (0.034)	#SAKNAS!
protein-calorie	1	5110(0.011432491139819366)	2359 (0.034)	#SAKNAS!
protein-calorie	1	5110(0.011432491139819366)	2359 (0.034)	#SAKNAS!
protein-carbohydrate	1	5110(0.011432491139819366)	#SAKNAS!	2548 (0.033)
protein-polysaccharide	1	5110(0.011432491139819366)	#SAKNAS!	#SAKNAS!
protein-positive	1	5110(0.011432491139819366)	#SAKNAS!	#SAKNAS!
protein-reduced	1	5110(0.011432491139819366)	#SAKNAS!	2548 (0.033)
protein-rich	1	5110(0.011432491139819366)	#SAKNAS!	2548 (0.033)
protein-unbalanced	1	5110(0.011432491139819366)	2359 (0.034)	#SAKNAS!
protein-unbalanced	1	5110(0.011432491139819366)	2359 (0.034)	#SAKNAS!
proteinuria	1	5110(0.011432491139819366)	#SAKNAS!	#SAKNAS!
proteome	1	3437(0.02286498227963873)	2359 (0.034)	#SAKNAS!
protists	1	5110(0.011432491139819366)	2359 (0.034)	#SAKNAS!
protists	1	5110(0.011432491139819366)	2359 (0.034)	#SAKNAS!
protocol-from	1	5110(0.011432491139819366)	#SAKNAS!	#SAKNAS!
proton	1	5110(0.011432491139819366)	#SAKNAS!	#SAKNAS!
protoporphyrin	1	5110(0.011432491139819366)	2359 (0.034)	#SAKNAS!
protoporphyrin	1	5110(0.011432491139819366)	2359 (0.034)	#SAKNAS!
prototypes	1	5110(0.011432491139819366)	#SAKNAS!	#SAKNAS!
protozoa	1	5110(0.011432491139819366)	2359 (0.034)	#SAKNAS!
protozoa	1	5110(0.011432491139819366)	2359 (0.034)	#SAKNAS!
protrusion	1	5110(0.011432491139819366)	#SAKNAS!	2548 (0.033)
provide	1	5110(0.011432491139819366)	#SAKNAS!	2548 (0.033)
provider	1	5110(0.011432491139819366)	2359 (0.034)	#SAKNAS!
provider	1	5110(0.011432491139819366)	2359 (0.034)	#SAKNAS!
providers	1	1485(0.08002743797873557)	2359 (0.034)	1233 (0.1)
providing	1	1485(0.08002743797873557)	2359 (0.034)	694 (0.2)

provinces	1	2693(0.0342974734194581)	2359 (0.034)	#SAKNAS!
provocation	1	5110(0.011432491139819366)	#SAKNAS!	2548 (0.033)
proximal	1	1111(0.11432491139819366)	2359 (0.034)	999 (0.13)
proximity	1	3437(0.02286498227963873)	2359 (0.034)	#SAKNAS!
prudent	1	5110(0.011432491139819366)	#SAKNAS!	#SAKNAS!
prunorum	1	5110(0.011432491139819366)	#SAKNAS!	2548 (0.033)
prunus	1	5110(0.011432491139819366)	#SAKNAS!	2548 (0.033)
psammodromus-algirus	1	5110(0.011432491139819366)	2359 (0.034)	#SAKNAS!
psammodromus-algirus	1	5110(0.011432491139819366)	2359 (0.034)	#SAKNAS!
pseudarthrosis	1	5110(0.011432491139819366)	2359 (0.034)	#SAKNAS!
pseudarthrosis	1	5110(0.011432491139819366)	2359 (0.034)	#SAKNAS!
pseudoaneurysm	1	5110(0.011432491139819366)	#SAKNAS!	2548 (0.033)
pseudodiverticulosis	1	5110(0.011432491139819366)	2359 (0.034)	#SAKNAS!
pseudodiverticulosis	1	5110(0.011432491139819366)	2359 (0.034)	#SAKNAS!
pseudomonas	1	3437(0.02286498227963873)	2359 (0.034)	#SAKNAS!
pseudo-nitzschia	1	5110(0.011432491139819366)	2359 (0.034)	#SAKNAS!
pseudo-nitzschia	1	5110(0.011432491139819366)	2359 (0.034)	#SAKNAS!
pseudo-obstruction	1	5110(0.011432491139819366)	2359 (0.034)	#SAKNAS!
pseudo-obstruction	1	5110(0.011432491139819366)	2359 (0.034)	#SAKNAS!
pseudophyllidea	1	5110(0.011432491139819366)	2359 (0.034)	#SAKNAS!
pseudophyllidea	1	5110(0.011432491139819366)	2359 (0.034)	#SAKNAS!
pseudoplatystoma	1	5110(0.011432491139819366)	#SAKNAS!	2548 (0.033)
pseudostem	1	5110(0.011432491139819366)	#SAKNAS!	2548 (0.033)
pseudotropheus	1	5110(0.011432491139819366)	2359 (0.034)	#SAKNAS!
pseudotropheus	1	5110(0.011432491139819366)	2359 (0.034)	#SAKNAS!
psittacidae	1	5110(0.011432491139819366)	2359 (0.034)	#SAKNAS!
psittacidae	1	5110(0.011432491139819366)	2359 (0.034)	#SAKNAS!
psoriasis	1	2214(0.04572996455927746)	2359 (0.034)	1668 (0.066)
psychiatric	1	1678(0.0685949468389162)	2359 (0.034)	1233 (0.1)
psychoemotional	1	5110(0.011432491139819366)	#SAKNAS!	#SAKNAS!
psychologist	1	5110(0.011432491139819366)	2359 (0.034)	#SAKNAS!
psychologist	1	5110(0.011432491139819366)	2359 (0.034)	#SAKNAS!
psychology	1	5110(0.011432491139819366)	#SAKNAS!	#SAKNAS!
psychometrics	1	5110(0.011432491139819366)	#SAKNAS!	2548 (0.033)
psychopathology	1	3437(0.02286498227963873)	2359 (0.034)	#SAKNAS!
psychophysical	1	5110(0.011432491139819366)	#SAKNAS!	2548 (0.033)
psychosis	1	3437(0.02286498227963873)	2359 (0.034)	#SAKNAS!
psylloidea	1	5110(0.011432491139819366)	#SAKNAS!	2548 (0.033)
pta-acka	1	5110(0.011432491139819366)	#SAKNAS!	2548 (0.033)
pterodactyloids	1	5110(0.011432491139819366)	#SAKNAS!	#SAKNAS!
pterois	1	5110(0.011432491139819366)	#SAKNAS!	#SAKNAS!
pteropus	1	5110(0.011432491139819366)	2359 (0.034)	#SAKNAS!
pteropus	1	5110(0.011432491139819366)	2359 (0.034)	#SAKNAS!

pterosauria	1	5110(0.011432491139819366)	#SAKNAS!	#SAKNAS!
puas	1	5110(0.011432491139819366)	2359 (0.034)	#SAKNAS!
puas	1	5110(0.011432491139819366)	2359 (0.034)	#SAKNAS!
puberty	1	3437(0.02286498227963873)	2359 (0.034)	2548 (0.033)
publica	1	5110(0.011432491139819366)	#SAKNAS!	2548 (0.033)
publication	1	5110(0.011432491139819366)	#SAKNAS!	2548 (0.033)
public-health	1	5110(0.011432491139819366)	2359 (0.034)	#SAKNAS!
public-health	1	5110(0.011432491139819366)	2359 (0.034)	#SAKNAS!
publicly	1	5110(0.011432491139819366)	2359 (0.034)	#SAKNAS!
publicly	1	5110(0.011432491139819366)	2359 (0.034)	#SAKNAS!
puddings	1	5110(0.011432491139819366)	2359 (0.034)	#SAKNAS!
puddings	1	5110(0.011432491139819366)	2359 (0.034)	#SAKNAS!
pufa	1	5110(0.011432491139819366)	#SAKNAS!	2548 (0.033)
puffins	1	5110(0.011432491139819366)	2359 (0.034)	#SAKNAS!
puffins	1	5110(0.011432491139819366)	2359 (0.034)	#SAKNAS!
puffs	1	5110(0.011432491139819366)	#SAKNAS!	#SAKNAS!
pulchripecta	1	5110(0.011432491139819366)	#SAKNAS!	#SAKNAS!
pullastra	1	5110(0.011432491139819366)	2359 (0.034)	#SAKNAS!
pullastra	1	5110(0.011432491139819366)	2359 (0.034)	#SAKNAS!
pullulan-based	1	5110(0.011432491139819366)	#SAKNAS!	#SAKNAS!
pull-up	1	5110(0.011432491139819366)	#SAKNAS!	2548 (0.033)
pulmonary-function	1	5110(0.011432491139819366)	2359 (0.034)	#SAKNAS!
pulmonary-function	1	5110(0.011432491139819366)	2359 (0.034)	#SAKNAS!
pulpal	1	5110(0.011432491139819366)	#SAKNAS!	#SAKNAS!
pulpally	1	5110(0.011432491139819366)	2359 (0.034)	#SAKNAS!
pulpally	1	5110(0.011432491139819366)	2359 (0.034)	#SAKNAS!
pulpitis	1	5110(0.011432491139819366)	#SAKNAS!	#SAKNAS!
pulsatile	1	5110(0.011432491139819366)	2359 (0.034)	#SAKNAS!
pulsatile	1	5110(0.011432491139819366)	2359 (0.034)	#SAKNAS!
pulse	1	2214(0.04572996455927746)	2359 (0.034)	2548 (0.033)
pulses	1	5110(0.011432491139819366)	2359 (0.034)	#SAKNAS!
pulses	1	5110(0.011432491139819366)	2359 (0.034)	#SAKNAS!
pump	1	5110(0.011432491139819366)	#SAKNAS!	#SAKNAS!
pumps	1	5110(0.011432491139819366)	#SAKNAS!	#SAKNAS!
pup	1	5110(0.011432491139819366)	2359 (0.034)	#SAKNAS!
pup	1	5110(0.011432491139819366)	2359 (0.034)	#SAKNAS!
purchase	1	5110(0.011432491139819366)	#SAKNAS!	2548 (0.033)
purging	1	5110(0.011432491139819366)	#SAKNAS!	2548 (0.033)
purinergic	1	5110(0.011432491139819366)	2359 (0.034)	#SAKNAS!
purinergic	1	5110(0.011432491139819366)	2359 (0.034)	#SAKNAS!
purple	1	5110(0.011432491139819366)	#SAKNAS!	#SAKNAS!
purpose	1	5110(0.011432491139819366)	2359 (0.034)	#SAKNAS!
purpose	1	5110(0.011432491139819366)	2359 (0.034)	#SAKNAS!

purposes	1	3437(0.0228649822796387З)	2359 (0.034)	#SAKNAS!
put	1	5110(0.011432491139819366)	2359 (0.034)	#SAKNAS!
put	1	5110(0.011432491139819366)	2359 (0.034)	#SAKNAS!
putrescine	1	5110(0.011432491139819366)	2359 (0.034)	#SAKNAS!
putrescine	1	5110(0.011432491139819366)	2359 (0.034)	#SAKNAS!
pvy-infected	1	5110(0.011432491139819366)	#SAKNAS!	#SAKNAS!
pycnopsyche	1	5110(0.011432491139819366)	2359 (0.034)	#SAKNAS!
pycnopsyche	1	5110(0.011432491139819366)	2359 (0.034)	#SAKNAS!
pygmaeus	1	5110(0.011432491139819366)	#SAKNAS!	2548 (0.033)
pyloric	1	5110(0.011432491139819366)	2359 (0.034)	#SAKNAS!
pyloric	1	5110(0.011432491139819366)	2359 (0.034)	#SAKNAS!
pyogenic	1	5110(0.011432491139819366)	#SAKNAS!	2548 (0.033)
pyongyang	1	5110(0.011432491139819366)	#SAKNAS!	2548 (0.033)
pyramid	1	3437(0.02286498227963873)	2359 (0.034)	2548 (0.033)
pyroptotic	1	5110(0.011432491139819366)	#SAKNAS!	#SAKNAS!
pyrrolizidine	1	5110(0.011432491139819366)	2359 (0.034)	#SAKNAS!
pyrrolizidine	1	5110(0.011432491139819366)	2359 (0.034)	#SAKNAS!
pyrroloquinoline	1	5110(0.011432491139819366)	#SAKNAS!	#SAKNAS!
pyruvate	1	5110(0.011432491139819366)	#SAKNAS!	2548 (0.033)
pythium	1	5110(0.011432491139819366)	2359 (0.034)	#SAKNAS!
pythium	1	5110(0.011432491139819366)	2359 (0.034)	#SAKNAS!
p矇cs	1	5110(0.011432491139819366)	#SAKNAS!	#SAKNAS!
qashqai	1	5110(0.011432491139819366)	#SAKNAS!	2548 (0.033)
qinling	1	5110(0.011432491139819366)	#SAKNAS!	2548 (0.033)
qinshihuang	1	5110(0.011432491139819366)	#SAKNAS!	2548 (0.033)
qlq-c30	1	5110(0.011432491139819366)	2359 (0.034)	#SAKNAS!
qlq-c30	1	5110(0.011432491139819366)	2359 (0.034)	#SAKNAS!
qlq-h&n35	1	5110(0.011432491139819366)	#SAKNAS!	2548 (0.033)
qlq-oh17	1	5110(0.011432491139819366)	2359 (0.034)	#SAKNAS!
qlq-oh17	1	5110(0.011432491139819366)	2359 (0.034)	#SAKNAS!
qol	1	5110(0.011432491139819366)	#SAKNAS!	2548 (0.033)
qpsg	1	5110(0.011432491139819366)	2359 (0.034)	#SAKNAS!
qpsg	1	5110(0.011432491139819366)	2359 (0.034)	#SAKNAS!
qrfp	1	5110(0.011432491139819366)	2359 (0.034)	#SAKNAS!
qrfp	1	5110(0.011432491139819366)	2359 (0.034)	#SAKNAS!
quadrimaculatus	1	5110(0.011432491139819366)	#SAKNAS!	2548 (0.033)
quadriparetic	1	5110(0.011432491139819366)	#SAKNAS!	2548 (0.033)
quaking	1	5110(0.011432491139819366)	2359 (0.034)	#SAKNAS!
quaking	1	5110(0.011432491139819366)	2359 (0.034)	#SAKNAS!
quality-a	1	5110(0.011432491139819366)	#SAKNAS!	#SAKNAS!
quantification	1	1678(0.0685949468389162)	2359 (0.034)	816 (0.16)
quantify	1	5110(0.011432491139819366)	#SAKNAS!	2548 (0.033)
quantile	1	5110(0.011432491139819366)	#SAKNAS!	#SAKNAS!

quarter	1	5110(0.011432491139819366)	#SAKNAS!	2548 (0.033)
quasi-clinical	1	5110(0.011432491139819366)	#SAKNAS!	#SAKNAS!
quebec	1	3437(0.022864982279638733)	2359 (0.034)	2548 (0.033)
queen	1	5110(0.011432491139819366)	2359 (0.034)	#SAKNAS!
queen	1	5110(0.011432491139819366)	2359 (0.034)	#SAKNAS!
quercus	1	5110(0.011432491139819366)	2359 (0.034)	#SAKNAS!
quercus	1	5110(0.011432491139819366)	2359 (0.034)	#SAKNAS!
questionnaire-r21	1	5110(0.011432491139819366)	2359 (0.034)	#SAKNAS!
questionnaire-r21	1	5110(0.011432491139819366)	2359 (0.034)	#SAKNAS!
questions	1	5110(0.011432491139819366)	2359 (0.034)	#SAKNAS!
questions	1	5110(0.011432491139819366)	2359 (0.034)	#SAKNAS!
quick	1	3437(0.022864982279638733)	2359 (0.034)	2548 (0.033)
quid	1	5110(0.011432491139819366)	2359 (0.034)	#SAKNAS!
quid	1	5110(0.011432491139819366)	2359 (0.034)	#SAKNAS!
quindio	1	5110(0.011432491139819366)	#SAKNAS!	2548 (0.033)
quinone	1	5110(0.011432491139819366)	#SAKNAS!	#SAKNAS!
quinquenervia	1	5110(0.011432491139819366)	2359 (0.034)	#SAKNAS!
quinquenervia	1	5110(0.011432491139819366)	2359 (0.034)	#SAKNAS!
quintile	1	5110(0.011432491139819366)	#SAKNAS!	2548 (0.033)
quitting	1	5110(0.011432491139819366)	#SAKNAS!	2548 (0.033)
quotient	1	5110(0.011432491139819366)	#SAKNAS!	#SAKNAS!
r	1	2693(0.0342974734194581)	2359 (0.034)	2548 (0.033)
rabbit	1	3437(0.022864982279638733)	2359 (0.034)	2548 (0.033)
rabbitbrush	1	5110(0.011432491139819366)	2359 (0.034)	#SAKNAS!
rabbitbrush	1	5110(0.011432491139819366)	2359 (0.034)	#SAKNAS!
race	1	2214(0.04572996455927746)	2359 (0.034)	1668 (0.066)
races	1	3437(0.022864982279638733)	2359 (0.034)	#SAKNAS!
racially	1	5110(0.011432491139819366)	#SAKNAS!	#SAKNAS!
raclopride	1	5110(0.011432491139819366)	2359 (0.034)	#SAKNAS!
raclopride	1	5110(0.011432491139819366)	2359 (0.034)	#SAKNAS!
radia-tion	1	5110(0.011432491139819366)	#SAKNAS!	#SAKNAS!
radiation-therapy	1	5110(0.011432491139819366)	2359 (0.034)	#SAKNAS!
radiation-therapy	1	5110(0.011432491139819366)	2359 (0.034)	#SAKNAS!
radical	1	3437(0.022864982279638733)	2359 (0.034)	#SAKNAS!
radio	1	5110(0.011432491139819366)	#SAKNAS!	2548 (0.033)
radioactive	1	5110(0.011432491139819366)	#SAKNAS!	2548 (0.033)
radiofrequency	1	5110(0.011432491139819366)	2359 (0.034)	#SAKNAS!
radiofrequency	1	5110(0.011432491139819366)	2359 (0.034)	#SAKNAS!
radiograph	1	5110(0.011432491139819366)	#SAKNAS!	2548 (0.033)
radiologically	1	5110(0.011432491139819366)	#SAKNAS!	#SAKNAS!
radiology	1	5110(0.011432491139819366)	2359 (0.034)	#SAKNAS!
radiology	1	5110(0.011432491139819366)	2359 (0.034)	#SAKNAS!
radiomics	1	5110(0.011432491139819366)	#SAKNAS!	#SAKNAS!

radiotherapy-induced	1	5110(0.011432491139819366)	#SAKNAS!	#SAKNAS!
radioxerostomia	1	5110(0.011432491139819366)	2359 (0.034)	#SAKNAS!
radioxerostomia	1	5110(0.011432491139819366)	2359 (0.034)	#SAKNAS!
radius	1	5110(0.011432491139819366)	#SAKNAS!	#SAKNAS!
rage	1	5110(0.011432491139819366)	#SAKNAS!	2548 (0.033)
ragwort	1	5110(0.011432491139819366)	#SAKNAS!	#SAKNAS!
raider	1	5110(0.011432491139819366)	#SAKNAS!	#SAKNAS!
rainy	1	5110(0.011432491139819366)	#SAKNAS!	2548 (0.033)
raja	1	5110(0.011432491139819366)	#SAKNAS!	#SAKNAS!
raman	1	5110(0.011432491139819366)	#SAKNAS!	2548 (0.033)
rampant	1	5110(0.011432491139819366)	2359 (0.034)	#SAKNAS!
rampant	1	5110(0.011432491139819366)	2359 (0.034)	#SAKNAS!
rams	1	5110(0.011432491139819366)	#SAKNAS!	#SAKNAS!
ramus	1	5110(0.011432491139819366)	#SAKNAS!	2548 (0.033)
rancd	1	5110(0.011432491139819366)	#SAKNAS!	#SAKNAS!
rancho	1	5110(0.011432491139819366)	#SAKNAS!	2548 (0.033)
random	1	3437(0.02286498227963873)	2359 (0.034)	2548 (0.033)
randomisation	1	5110(0.011432491139819366)	#SAKNAS!	#SAKNAS!
rangeland	1	5110(0.011432491139819366)	#SAKNAS!	2548 (0.033)
rank	1	3437(0.02286498227963873)	2359 (0.034)	2548 (0.033)
rantes	1	5110(0.011432491139819366)	#SAKNAS!	#SAKNAS!
rapa	1	5110(0.011432491139819366)	#SAKNAS!	#SAKNAS!
rapalogue	1	5110(0.011432491139819366)	#SAKNAS!	2548 (0.033)
rapamycin	1	5110(0.011432491139819366)	#SAKNAS!	2548 (0.033)
rapana	1	5110(0.011432491139819366)	#SAKNAS!	#SAKNAS!
rapeseed	1	5110(0.011432491139819366)	#SAKNAS!	#SAKNAS!
rapidly	1	5110(0.011432491139819366)	#SAKNAS!	#SAKNAS!
raptorial-feeding	1	5110(0.011432491139819366)	2359 (0.034)	#SAKNAS!
raptorial-feeding	1	5110(0.011432491139819366)	2359 (0.034)	#SAKNAS!
ras	1	5110(0.011432491139819366)	#SAKNAS!	#SAKNAS!
raspberry	1	5110(0.011432491139819366)	2359 (0.034)	#SAKNAS!
raspberry	1	5110(0.011432491139819366)	2359 (0.034)	#SAKNAS!
raster-scanned	1	5110(0.011432491139819366)	#SAKNAS!	2548 (0.033)
rat-feeding	1	5110(0.011432491139819366)	2359 (0.034)	#SAKNAS!
rat-feeding	1	5110(0.011432491139819366)	2359 (0.034)	#SAKNAS!
ratio-based	1	5110(0.011432491139819366)	#SAKNAS!	#SAKNAS!
rationale	1	1894(0.05716245569909683)	2359 (0.034)	999 (0.13)
rations	1	3437(0.02286498227963873)	2359 (0.034)	#SAKNAS!
ratios	1	2214(0.04572996455927746)	2359 (0.034)	2548 (0.033)
rattus	1	5110(0.011432491139819366)	2359 (0.034)	#SAKNAS!
rattus	1	5110(0.011432491139819366)	2359 (0.034)	#SAKNAS!
ratufa	1	5110(0.011432491139819366)	#SAKNAS!	#SAKNAS!
rau	1	5110(0.011432491139819366)	#SAKNAS!	2548 (0.033)

raw	1	1678(0.0685949468389162)	2359 (0.034)	#SAKNAS!
ray	1	5110(0.011432491139819366)	#SAKNAS!	#SAKNAS!
raynaud	1	5110(0.011432491139819366)	2359 (0.034)	#SAKNAS!
raynaud	1	5110(0.011432491139819366)	2359 (0.034)	#SAKNAS!
reaches	1	5110(0.011432491139819366)	#SAKNAS!	#SAKNAS!
reaction	1	5110(0.011432491139819366)	#SAKNAS!	2548 (0.033)
reactive	1	1678(0.0685949468389162)	2359 (0.034)	1668 (0.066)
reactivity	1	5110(0.011432491139819366)	#SAKNAS!	2548 (0.033)
readily	1	5110(0.011432491139819366)	#SAKNAS!	#SAKNAS!
readiness	1	2693(0.0342974734194581)	2359 (0.034)	#SAKNAS!
reading	1	5110(0.011432491139819366)	#SAKNAS!	2548 (0.033)
readmissions	1	5110(0.011432491139819366)	#SAKNAS!	#SAKNAS!
ready	1	5110(0.011432491139819366)	#SAKNAS!	#SAKNAS!
ready-to-drink	1	5110(0.011432491139819366)	#SAKNAS!	2548 (0.033)
ready-to-eat	1	5110(0.011432491139819366)	#SAKNAS!	#SAKNAS!
ready-to-feed	1	5110(0.011432491139819366)	#SAKNAS!	2548 (0.033)
re-aim	1	5110(0.011432491139819366)	#SAKNAS!	#SAKNAS!
reality	1	2693(0.0342974734194581)	2359 (0.034)	1668 (0.066)
realize	1	5110(0.011432491139819366)	2359 (0.034)	#SAKNAS!
realize	1	5110(0.011432491139819366)	2359 (0.034)	#SAKNAS!
reappearance	1	5110(0.011432491139819366)	#SAKNAS!	2548 (0.033)
reappraisal	1	5110(0.011432491139819366)	#SAKNAS!	2548 (0.033)
rearing	1	2214(0.04572996455927746)	2359 (0.034)	1668 (0.066)
reason	1	5110(0.011432491139819366)	2359 (0.034)	#SAKNAS!
reason	1	5110(0.011432491139819366)	2359 (0.034)	#SAKNAS!
reasonable	1	5110(0.011432491139819366)	#SAKNAS!	#SAKNAS!
reasons	1	2693(0.0342974734194581)	2359 (0.034)	2548 (0.033)
recalcitrant	1	5110(0.011432491139819366)	#SAKNAS!	2548 (0.033)
recall	1	5110(0.011432491139819366)	#SAKNAS!	2548 (0.033)
receiver	1	5110(0.011432491139819366)	#SAKNAS!	2548 (0.033)
recently	1	5110(0.011432491139819366)	#SAKNAS!	#SAKNAS!
recent-onset	1	5110(0.011432491139819366)	2359 (0.034)	#SAKNAS!
recent-onset	1	5110(0.011432491139819366)	2359 (0.034)	#SAKNAS!
reception	1	5110(0.011432491139819366)	#SAKNAS!	2548 (0.033)
receptor-deficient	1	5110(0.011432491139819366)	#SAKNAS!	2548 (0.033)
receptor-expressing	1	5110(0.011432491139819366)	2359 (0.034)	#SAKNAS!
receptor-expressing	1	5110(0.011432491139819366)	2359 (0.034)	#SAKNAS!
receptor-mutated	1	5110(0.011432491139819366)	#SAKNAS!	#SAKNAS!
recessions	1	5110(0.011432491139819366)	#SAKNAS!	2548 (0.033)
recipes	1	2693(0.0342974734194581)	2359 (0.034)	2548 (0.033)
recipient	1	5110(0.011432491139819366)	#SAKNAS!	#SAKNAS!
recline	1	5110(0.011432491139819366)	#SAKNAS!	2548 (0.033)
recognizable	1	5110(0.011432491139819366)	#SAKNAS!	#SAKNAS!

recollection	1	5110(0.011432491139819366)	#SAKNAS!	#SAKNAS!
recombination	1	5110(0.011432491139819366)	2359 (0.034)	#SAKNAS!
recombination	1	5110(0.011432491139819366)	2359 (0.034)	#SAKNAS!
recommend	1	5110(0.011432491139819366)	2359 (0.034)	#SAKNAS!
recommend	1	5110(0.011432491139819366)	2359 (0.034)	#SAKNAS!
reconciles	1	5110(0.011432491139819366)	#SAKNAS!	#SAKNAS!
reconciling	1	5110(0.011432491139819366)	#SAKNAS!	#SAKNAS!
reconstructive	1	5110(0.011432491139819366)	#SAKNAS!	2548 (0.033)
recover	1	5110(0.011432491139819366)	2359 (0.034)	#SAKNAS!
recover	1	5110(0.011432491139819366)	2359 (0.034)	#SAKNAS!
recovering	1	5110(0.011432491139819366)	#SAKNAS!	#SAKNAS!
rectus	1	5110(0.011432491139819366)	2359 (0.034)	#SAKNAS!
rectus	1	5110(0.011432491139819366)	2359 (0.034)	#SAKNAS!
recurrences	1	5110(0.011432491139819366)	#SAKNAS!	2548 (0.033)
recutita	1	5110(0.011432491139819366)	#SAKNAS!	2548 (0.033)
red-complex	1	5110(0.011432491139819366)	#SAKNAS!	#SAKNAS!
redefining	1	5110(0.011432491139819366)	#SAKNAS!	2548 (0.033)
redic	1	5110(0.011432491139819366)	#SAKNAS!	#SAKNAS!
redo-laparoscopic	1	5110(0.011432491139819366)	#SAKNAS!	2548 (0.033)
redox	1	3437(0.02286498227963873)	2359 (0.034)	#SAKNAS!
redundancy	1	5110(0.011432491139819366)	2359 (0.034)	#SAKNAS!
redundancy	1	5110(0.011432491139819366)	2359 (0.034)	#SAKNAS!
red-winged	1	5110(0.011432491139819366)	#SAKNAS!	2548 (0.033)
reefs	1	5110(0.011432491139819366)	2359 (0.034)	#SAKNAS!
reefs	1	5110(0.011432491139819366)	2359 (0.034)	#SAKNAS!
reevaluating	1	5110(0.011432491139819366)	2359 (0.034)	#SAKNAS!
reevaluating	1	5110(0.011432491139819366)	2359 (0.034)	#SAKNAS!
re-examining	1	5110(0.011432491139819366)	#SAKNAS!	#SAKNAS!
reference	1	3437(0.02286498227963873)	2359 (0.034)	#SAKNAS!
reference-invariant	1	5110(0.011432491139819366)	2359 (0.034)	#SAKNAS!
reference-invariant	1	5110(0.011432491139819366)	2359 (0.034)	#SAKNAS!
referral	1	2693(0.0342974734194581)	2359 (0.034)	2548 (0.033)
referrals	1	5110(0.011432491139819366)	2359 (0.034)	#SAKNAS!
referrals	1	5110(0.011432491139819366)	2359 (0.034)	#SAKNAS!
refinement	1	5110(0.011432491139819366)	#SAKNAS!	#SAKNAS!
reflection	1	3437(0.02286498227963873)	2359 (0.034)	2548 (0.033)
reflections	1	2693(0.0342974734194581)	2359 (0.034)	2548 (0.033)
reflexes	1	5110(0.011432491139819366)	#SAKNAS!	2548 (0.033)
reflexive	1	5110(0.011432491139819366)	#SAKNAS!	2548 (0.033)
refractory	1	1894(0.05716245569909683)	2359 (0.034)	1668 (0.066)
refrigeration	1	5110(0.011432491139819366)	#SAKNAS!	#SAKNAS!
refuge	1	5110(0.011432491139819366)	2359 (0.034)	#SAKNAS!
refuge	1	5110(0.011432491139819366)	2359 (0.034)	#SAKNAS!

refusal	1	5110(0.011432491139819366)	2359 (0.034)	#SAKNAS!
refusal	1	5110(0.011432491139819366)	2359 (0.034)	#SAKNAS!
regain	1	1894(0.05716245569909683)	2359 (0.034)	1233 (0.1)
regarding	1	1219(0.1028924202583743)	2359 (0.034)	999 (0.13)
regardless	1	5110(0.011432491139819366)	#SAKNAS!	2548 (0.033)
regeneration	1	5110(0.011432491139819366)	#SAKNAS!	#SAKNAS!
regime	1	5110(0.011432491139819366)	2359 (0.034)	#SAKNAS!
regime	1	5110(0.011432491139819366)	2359 (0.034)	#SAKNAS!
regimens	1	5110(0.011432491139819366)	#SAKNAS!	#SAKNAS!
regrowth	1	5110(0.011432491139819366)	2359 (0.034)	#SAKNAS!
regrowth	1	5110(0.011432491139819366)	2359 (0.034)	#SAKNAS!
regular	1	2214(0.04572996455927746)	2359 (0.034)	1668 (0.066)
regulate	1	1485(0.08002743797873557)	2359 (0.034)	1668 (0.066)
regulations	1	3437(0.02286498227963873)	2359 (0.034)	#SAKNAS!
regulator	1	3437(0.02286498227963873)	2359 (0.034)	#SAKNAS!
regulators	1	5110(0.011432491139819366)	#SAKNAS!	2548 (0.033)
regulatory	1	5110(0.011432491139819366)	#SAKNAS!	#SAKNAS!
regurgitation	1	3437(0.02286498227963873)	2359 (0.034)	#SAKNAS!
rehydrated	1	5110(0.011432491139819366)	#SAKNAS!	2548 (0.033)
rehydration	1	5110(0.011432491139819366)	2359 (0.034)	#SAKNAS!
rehydration	1	5110(0.011432491139819366)	2359 (0.034)	#SAKNAS!
reinforcing	1	5110(0.011432491139819366)	2359 (0.034)	#SAKNAS!
reinforcing	1	5110(0.011432491139819366)	2359 (0.034)	#SAKNAS!
reingestion	1	5110(0.011432491139819366)	2359 (0.034)	#SAKNAS!
reingestion	1	5110(0.011432491139819366)	2359 (0.034)	#SAKNAS!
re-initiation	1	5110(0.011432491139819366)	#SAKNAS!	2548 (0.033)
reintervention	1	5110(0.011432491139819366)	#SAKNAS!	2548 (0.033)
rejects	1	5110(0.011432491139819366)	2359 (0.034)	#SAKNAS!
rejects	1	5110(0.011432491139819366)	2359 (0.034)	#SAKNAS!
relapse	1	5110(0.011432491139819366)	2359 (0.034)	#SAKNAS!
relapse	1	5110(0.011432491139819366)	2359 (0.034)	#SAKNAS!
relapsing-remitting	1	5110(0.011432491139819366)	#SAKNAS!	#SAKNAS!
related-protein	1	5110(0.011432491139819366)	2359 (0.034)	#SAKNAS!
related-protein	1	5110(0.011432491139819366)	2359 (0.034)	#SAKNAS!
relational	1	5110(0.011432491139819366)	#SAKNAS!	#SAKNAS!
relative	1	1111(0.11432491139819366)	2359 (0.034)	999 (0.13)
relatively	1	5110(0.011432491139819366)	#SAKNAS!	2548 (0.033)
releases	1	5110(0.011432491139819366)	#SAKNAS!	2548 (0.033)
relevant	1	1894(0.05716245569909683)	2359 (0.034)	2548 (0.033)
reliable	1	5110(0.011432491139819366)	#SAKNAS!	2548 (0.033)
relictual	1	5110(0.011432491139819366)	#SAKNAS!	#SAKNAS!
relieves	1	5110(0.011432491139819366)	#SAKNAS!	2548 (0.033)
relieving	1	5110(0.011432491139819366)	#SAKNAS!	#SAKNAS!

religious	1	5110(0.011432491139819366)	#SAKNAS!	2548 (0.033)
rely	1	5110(0.011432491139819366)	2359 (0.034)	#SAKNAS!
rely	1	5110(0.011432491139819366)	2359 (0.034)	#SAKNAS!
remains	1	3437(0.02286498227963873)	2359 (0.034)	#SAKNAS!
remarks	1	5110(0.011432491139819366)	#SAKNAS!	2548 (0.033)
remediation	1	5110(0.011432491139819366)	#SAKNAS!	2548 (0.033)
remember	1	5110(0.011432491139819366)	#SAKNAS!	2548 (0.033)
remise	1	5110(0.011432491139819366)	#SAKNAS!	#SAKNAS!
remnant	1	5110(0.011432491139819366)	#SAKNAS!	#SAKNAS!
remoteness	1	5110(0.011432491139819366)	#SAKNAS!	#SAKNAS!
removable-partial-denture	1	5110(0.011432491139819366)	#SAKNAS!	#SAKNAS!
removals	1	5110(0.011432491139819366)	#SAKNAS!	2548 (0.033)
removing	1	5110(0.011432491139819366)	2359 (0.034)	#SAKNAS!
removing	1	5110(0.011432491139819366)	2359 (0.034)	#SAKNAS!
renin-angiotensin	1	5110(0.011432491139819366)	#SAKNAS!	2548 (0.033)
renyi	1	5110(0.011432491139819366)	2359 (0.034)	#SAKNAS!
renyi	1	5110(0.011432491139819366)	2359 (0.034)	#SAKNAS!
reoperation	1	5110(0.011432491139819366)	2359 (0.034)	#SAKNAS!
reoperation	1	5110(0.011432491139819366)	2359 (0.034)	#SAKNAS!
re-operative	1	5110(0.011432491139819366)	#SAKNAS!	#SAKNAS!
reorganization	1	5110(0.011432491139819366)	2359 (0.034)	#SAKNAS!
reorganization	1	5110(0.011432491139819366)	2359 (0.034)	#SAKNAS!
repair-a	1	5110(0.011432491139819366)	#SAKNAS!	2548 (0.033)
repairs	1	5110(0.011432491139819366)	#SAKNAS!	2548 (0.033)
repellents	1	5110(0.011432491139819366)	#SAKNAS!	2548 (0.033)
repercussions	1	5110(0.011432491139819366)	2359 (0.034)	#SAKNAS!
repercussions	1	5110(0.011432491139819366)	2359 (0.034)	#SAKNAS!
reperfusion	1	5110(0.011432491139819366)	2359 (0.034)	#SAKNAS!
reperfusion	1	5110(0.011432491139819366)	2359 (0.034)	#SAKNAS!
replacing	1	2214(0.04572996455927746)	2359 (0.034)	2548 (0.033)
representation	1	5110(0.011432491139819366)	2359 (0.034)	#SAKNAS!
representation	1	5110(0.011432491139819366)	2359 (0.034)	#SAKNAS!
represses	1	5110(0.011432491139819366)	#SAKNAS!	#SAKNAS!
reproducibility	1	5110(0.011432491139819366)	2359 (0.034)	#SAKNAS!
reproducibility	1	5110(0.011432491139819366)	2359 (0.034)	#SAKNAS!
repurposing	1	5110(0.011432491139819366)	#SAKNAS!	#SAKNAS!
require	1	3437(0.02286498227963873)	2359 (0.034)	2548 (0.033)
requirement	1	2693(0.0342974734194581)	2359 (0.034)	2548 (0.033)
requirements	1	3437(0.02286498227963873)	2359 (0.034)	2548 (0.033)
requiring	1	671(0.2057848405167486)	2359 (0.034)	584 (0.23)
reranking	1	5110(0.011432491139819366)	#SAKNAS!	#SAKNAS!
rerio	1	5110(0.011432491139819366)	2359 (0.034)	#SAKNAS!
rerio	1	5110(0.011432491139819366)	2359 (0.034)	#SAKNAS!

resectable	1	3437(0.0228649822796387 3)	2359 (0.034)	2548 (0.033)
resectional	1	5110(0.011432491139819366)	2359 (0.034)	#SAKNAS!
resectional	1	5110(0.011432491139819366)	2359 (0.034)	#SAKNAS!
reserve	1	3437(0.02286498227963873)	2359 (0.034)	#SAKNAS!
reserves	1	5110(0.011432491139819366)	2359 (0.034)	#SAKNAS!
reserves	1	5110(0.011432491139819366)	2359 (0.034)	#SAKNAS!
reservoir	1	5110(0.011432491139819366)	#SAKNAS!	2548 (0.033)
resettled	1	5110(0.011432491139819366)	#SAKNAS!	#SAKNAS!
reshapes	1	5110(0.011432491139819366)	#SAKNAS!	#SAKNAS!
residence	1	3437(0.02286498227963873)	2359 (0.034)	#SAKNAS!
residences	1	5110(0.011432491139819366)	2359 (0.034)	#SAKNAS!
residences	1	5110(0.011432491139819366)	2359 (0.034)	#SAKNAS!
resident	1	2214(0.04572996455927746)	2359 (0.034)	1668 (0.066)
residential	1	1111(0.11432491139819366)	2359 (0.034)	694 (0.2)
residents-results	1	5110(0.011432491139819366)	#SAKNAS!	2548 (0.033)
residues	1	3437(0.02286498227963873)	2359 (0.034)	#SAKNAS!
resin	1	5110(0.011432491139819366)	#SAKNAS!	#SAKNAS!
resistant	1	3437(0.02286498227963873)	2359 (0.034)	2548 (0.033)
resonance	1	2214(0.04572996455927746)	2359 (0.034)	2548 (0.033)
resorbable	1	5110(0.011432491139819366)	2359 (0.034)	#SAKNAS!
resorbable	1	5110(0.011432491139819366)	2359 (0.034)	#SAKNAS!
resorption	1	3437(0.02286498227963873)	2359 (0.034)	#SAKNAS!
resorptive	1	5110(0.011432491139819366)	2359 (0.034)	#SAKNAS!
resorptive	1	5110(0.011432491139819366)	2359 (0.034)	#SAKNAS!
respectful	1	5110(0.011432491139819366)	#SAKNAS!	#SAKNAS!
respective	1	5110(0.011432491139819366)	#SAKNAS!	#SAKNAS!
respiration	1	3437(0.02286498227963873)	2359 (0.034)	2548 (0.033)
respond	1	5110(0.011432491139819366)	2359 (0.034)	#SAKNAS!
respond	1	5110(0.011432491139819366)	2359 (0.034)	#SAKNAS!
respondents	1	5110(0.011432491139819366)	#SAKNAS!	#SAKNAS!
responders	1	3437(0.02286498227963873)	2359 (0.034)	#SAKNAS!
responds	1	5110(0.011432491139819366)	#SAKNAS!	2548 (0.033)
responsive	1	1485(0.08002743797873557)	2359 (0.034)	#SAKNAS!
responsiveness	1	1894(0.05716245569909683)	2359 (0.034)	1668 (0.066)
rest	1	3437(0.02286498227963873)	2359 (0.034)	2548 (0.033)
restaurant	1	5110(0.011432491139819366)	#SAKNAS!	#SAKNAS!
restaurants	1	5110(0.011432491139819366)	2359 (0.034)	#SAKNAS!
restaurants	1	5110(0.011432491139819366)	2359 (0.034)	#SAKNAS!
resting-state	1	5110(0.011432491139819366)	#SAKNAS!	2548 (0.033)
restraint	1	5110(0.011432491139819366)	#SAKNAS!	2548 (0.033)
restricted-fed	1	5110(0.011432491139819366)	2359 (0.034)	#SAKNAS!
restricted-fed	1	5110(0.011432491139819366)	2359 (0.034)	#SAKNAS!
restrictedly	1	5110(0.011432491139819366)	2359 (0.034)	#SAKNAS!

restrictedly	1	5110(0.011432491139819366)	2359 (0.034)	#SAKNAS!
restrictions	1	5110(0.011432491139819366)	#SAKNAS!	#SAKNAS!
restricts	1	5110(0.011432491139819366)	#SAKNAS!	2548 (0.033)
resumption	1	3437(0.02286498227963873)	2359 (0.034)	2548 (0.033)
resurgence	1	5110(0.011432491139819366)	2359 (0.034)	#SAKNAS!
resurgence	1	5110(0.011432491139819366)	2359 (0.034)	#SAKNAS!
resuspension	1	5110(0.011432491139819366)	2359 (0.034)	#SAKNAS!
resuspension	1	5110(0.011432491139819366)	2359 (0.034)	#SAKNAS!
retainers	1	5110(0.011432491139819366)	#SAKNAS!	2548 (0.033)
retching	1	5110(0.011432491139819366)	#SAKNAS!	2548 (0.033)
reticulatum	1	5110(0.011432491139819366)	2359 (0.034)	#SAKNAS!
reticulatum	1	5110(0.011432491139819366)	2359 (0.034)	#SAKNAS!
reticulatumfxleiarius	1	5110(0.011432491139819366)	#SAKNAS!	2548 (0.033)
reticulum	1	5110(0.011432491139819366)	#SAKNAS!	2548 (0.033)
retinal	1	5110(0.011432491139819366)	#SAKNAS!	2548 (0.033)
retinol-binding	1	5110(0.011432491139819366)	#SAKNAS!	2548 (0.033)
retornus-2	1	5110(0.011432491139819366)	#SAKNAS!	#SAKNAS!
retracted	1	5110(0.011432491139819366)	#SAKNAS!	2548 (0.033)
retrocolic-supracolic	1	5110(0.011432491139819366)	#SAKNAS!	2548 (0.033)
retrodiction	1	5110(0.011432491139819366)	#SAKNAS!	2548 (0.033)
retrognathic	1	5110(0.011432491139819366)	2359 (0.034)	#SAKNAS!
retrognathic	1	5110(0.011432491139819366)	2359 (0.034)	#SAKNAS!
retro-nasal	1	5110(0.011432491139819366)	#SAKNAS!	2548 (0.033)
retropharyngeal	1	5110(0.011432491139819366)	#SAKNAS!	#SAKNAS!
retro-pro	1	5110(0.011432491139819366)	2359 (0.034)	#SAKNAS!
retro-pro	1	5110(0.011432491139819366)	2359 (0.034)	#SAKNAS!
rett	1	5110(0.011432491139819366)	2359 (0.034)	#SAKNAS!
rett	1	5110(0.011432491139819366)	2359 (0.034)	#SAKNAS!
return	1	3437(0.02286498227963873)	2359 (0.034)	2548 (0.033)
returning	1	5110(0.011432491139819366)	#SAKNAS!	2548 (0.033)
returns	1	5110(0.011432491139819366)	#SAKNAS!	2548 (0.033)
reuptake	1	5110(0.011432491139819366)	2359 (0.034)	#SAKNAS!
reuptake	1	5110(0.011432491139819366)	2359 (0.034)	#SAKNAS!
rev-erb	1	5110(0.011432491139819366)	#SAKNAS!	#SAKNAS!
reversal	1	5110(0.011432491139819366)	#SAKNAS!	#SAKNAS!
reverse	1	5110(0.011432491139819366)	#SAKNAS!	2548 (0.033)
reverses	1	5110(0.011432491139819366)	2359 (0.034)	#SAKNAS!
reverses	1	5110(0.011432491139819366)	2359 (0.034)	#SAKNAS!
reversible	1	5110(0.011432491139819366)	2359 (0.034)	#SAKNAS!
reversible	1	5110(0.011432491139819366)	2359 (0.034)	#SAKNAS!
reversibly	1	5110(0.011432491139819366)	2359 (0.034)	#SAKNAS!
reversibly	1	5110(0.011432491139819366)	2359 (0.034)	#SAKNAS!
reviews	1	5110(0.011432491139819366)	#SAKNAS!	#SAKNAS!

revision	1	1485(0.08002743797873557)	2359 (0.034)	1233 (0.1)
revisional	1	3437(0.02286498227963873)	2359 (0.034)	#SAKNAS!
revista	1	5110(0.011432491139819366)	#SAKNAS!	2548 (0.033)
reward-based	1	5110(0.011432491139819366)	#SAKNAS!	2548 (0.033)
rewards	1	3437(0.02286498227963873)	2359 (0.034)	2548 (0.033)
reynosa	1	5110(0.011432491139819366)	#SAKNAS!	#SAKNAS!
rfi	1	5110(0.011432491139819366)	#SAKNAS!	#SAKNAS!
rhabdomyolysis	1	5110(0.011432491139819366)	2359 (0.034)	#SAKNAS!
rhabdomyolysis	1	5110(0.011432491139819366)	2359 (0.034)	#SAKNAS!
rhacophoridae	1	5110(0.011432491139819366)	2359 (0.034)	#SAKNAS!
rhacophoridae	1	5110(0.011432491139819366)	2359 (0.034)	#SAKNAS!
rhamnoides	1	5110(0.011432491139819366)	#SAKNAS!	#SAKNAS!
rhamnosus	1	5110(0.011432491139819366)	#SAKNAS!	2548 (0.033)
rhesus	1	3437(0.02286498227963873)	2359 (0.034)	2548 (0.033)
rheumatic	1	3437(0.02286498227963873)	2359 (0.034)	2548 (0.033)
rhgm-csf	1	5110(0.011432491139819366)	2359 (0.034)	#SAKNAS!
rhgm-csf	1	5110(0.011432491139819366)	2359 (0.034)	#SAKNAS!
rhineland-palatinate	1	5110(0.011432491139819366)	#SAKNAS!	2548 (0.033)
rhinoceroses	1	3437(0.02286498227963873)	2359 (0.034)	2548 (0.033)
rhinosinusitis	1	5110(0.011432491139819366)	#SAKNAS!	#SAKNAS!
rhinotermitidae	1	5110(0.011432491139819366)	2359 (0.034)	#SAKNAS!
rhinotermitidae	1	5110(0.011432491139819366)	2359 (0.034)	#SAKNAS!
rhizaria	1	5110(0.011432491139819366)	#SAKNAS!	#SAKNAS!
rhizoprionodon	1	5110(0.011432491139819366)	#SAKNAS!	2548 (0.033)
rhodnius	1	5110(0.011432491139819366)	2359 (0.034)	#SAKNAS!
rhodnius	1	5110(0.011432491139819366)	2359 (0.034)	#SAKNAS!
rhynchomonas	1	5110(0.011432491139819366)	2359 (0.034)	#SAKNAS!
rhynchomonas	1	5110(0.011432491139819366)	2359 (0.034)	#SAKNAS!
rhythmic	1	5110(0.011432491139819366)	#SAKNAS!	2548 (0.033)
rhythms	1	2214(0.04572996455927746)	2359 (0.034)	1668 (0.066)
riboflavin	1	5110(0.011432491139819366)	#SAKNAS!	2548 (0.033)
rica	1	5110(0.011432491139819366)	#SAKNAS!	2548 (0.033)
riceberry	1	5110(0.011432491139819366)	#SAKNAS!	#SAKNAS!
rice-specific	1	5110(0.011432491139819366)	#SAKNAS!	2548 (0.033)
rich-diets	1	5110(0.011432491139819366)	#SAKNAS!	2548 (0.033)
rickets	1	2693(0.0342974734194581)	2359 (0.034)	1668 (0.066)
ridge	1	5110(0.011432491139819366)	2359 (0.034)	#SAKNAS!
ridge	1	5110(0.011432491139819366)	2359 (0.034)	#SAKNAS!
rifampin	1	5110(0.011432491139819366)	2359 (0.034)	#SAKNAS!
rifampin	1	5110(0.011432491139819366)	2359 (0.034)	#SAKNAS!
rig	1	5110(0.011432491139819366)	#SAKNAS!	#SAKNAS!
righ	1	5110(0.011432491139819366)	2359 (0.034)	#SAKNAS!
righ	1	5110(0.011432491139819366)	2359 (0.034)	#SAKNAS!

rigid	1	5110(0.011432491139819366)	2359 (0.034)	#SAKNAS!
rigid	1	5110(0.011432491139819366)	2359 (0.034)	#SAKNAS!
rinse	1	5110(0.011432491139819366)	#SAKNAS!	2548 (0.033)
rio	1	5110(0.011432491139819366)	2359 (0.034)	#SAKNAS!
rio	1	5110(0.011432491139819366)	2359 (0.034)	#SAKNAS!
ripe	1	5110(0.011432491139819366)	2359 (0.034)	#SAKNAS!
ripe	1	5110(0.011432491139819366)	2359 (0.034)	#SAKNAS!
risk-results	1	5110(0.011432491139819366)	#SAKNAS!	#SAKNAS!
risk-sensitive	1	5110(0.011432491139819366)	2359 (0.034)	#SAKNAS!
risk-sensitive	1	5110(0.011432491139819366)	2359 (0.034)	#SAKNAS!
risky	1	3437(0.02286498227963873)	2359 (0.034)	#SAKNAS!
riyadh	1	2214(0.04572996455927746)	2359 (0.034)	2548 (0.033)
rnai	1	5110(0.011432491139819366)	#SAKNAS!	#SAKNAS!
roasted	1	5110(0.011432491139819366)	#SAKNAS!	#SAKNAS!
robust	1	5110(0.011432491139819366)	#SAKNAS!	#SAKNAS!
robustness	1	5110(0.011432491139819366)	#SAKNAS!	2548 (0.033)
rocaglamide	1	5110(0.011432491139819366)	2359 (0.034)	#SAKNAS!
rocaglamide	1	5110(0.011432491139819366)	2359 (0.034)	#SAKNAS!
rock	1	2693(0.0342974734194581)	2359 (0.034)	2548 (0.033)
rocky	1	5110(0.011432491139819366)	#SAKNAS!	#SAKNAS!
rocs	1	5110(0.011432491139819366)	#SAKNAS!	#SAKNAS!
rodent	1	2214(0.04572996455927746)	2359 (0.034)	1233 (0.1)
rodentia	1	5110(0.011432491139819366)	2359 (0.034)	#SAKNAS!
rodentia	1	5110(0.011432491139819366)	2359 (0.034)	#SAKNAS!
roe	1	3437(0.02286498227963873)	2359 (0.034)	#SAKNAS!
roeselii	1	5110(0.011432491139819366)	2359 (0.034)	#SAKNAS!
roeselii	1	5110(0.011432491139819366)	2359 (0.034)	#SAKNAS!
rogercresseyi	1	5110(0.011432491139819366)	#SAKNAS!	2548 (0.033)
rohita	1	5110(0.011432491139819366)	2359 (0.034)	#SAKNAS!
rohita	1	5110(0.011432491139819366)	2359 (0.034)	#SAKNAS!
roma	1	2693(0.0342974734194581)	2359 (0.034)	2548 (0.033)
roman	1	3437(0.02286498227963873)	2359 (0.034)	2548 (0.033)
romania-a	1	5110(0.011432491139819366)	#SAKNAS!	#SAKNAS!
romanian	1	5110(0.011432491139819366)	#SAKNAS!	2548 (0.033)
romanization	1	5110(0.011432491139819366)	2359 (0.034)	#SAKNAS!
romanization	1	5110(0.011432491139819366)	2359 (0.034)	#SAKNAS!
roraima	1	5110(0.011432491139819366)	2359 (0.034)	#SAKNAS!
roraima	1	5110(0.011432491139819366)	2359 (0.034)	#SAKNAS!
rosario	1	5110(0.011432491139819366)	2359 (0.034)	#SAKNAS!
rosario	1	5110(0.011432491139819366)	2359 (0.034)	#SAKNAS!
rosenbergii	1	5110(0.011432491139819366)	2359 (0.034)	#SAKNAS!
rosenbergii	1	5110(0.011432491139819366)	2359 (0.034)	#SAKNAS!
rosf	1	5110(0.011432491139819366)	#SAKNAS!	#SAKNAS!

rosmarinic	1	5110(0.011432491139819366)	#SAKNAS!	2548 (0.033)
rotaria	1	5110(0.011432491139819366)	#SAKNAS!	#SAKNAS!
rotation	1	5110(0.011432491139819366)	#SAKNAS!	2548 (0.033)
rotatoria	1	5110(0.011432491139819366)	#SAKNAS!	#SAKNAS!
rotifer	1	5110(0.011432491139819366)	#SAKNAS!	#SAKNAS!
rotifera	1	5110(0.011432491139819366)	#SAKNAS!	#SAKNAS!
rotifers	1	5110(0.011432491139819366)	#SAKNAS!	#SAKNAS!
rotundiformis	1	5110(0.011432491139819366)	#SAKNAS!	#SAKNAS!
rough	1	5110(0.011432491139819366)	#SAKNAS!	#SAKNAS!
roughage	1	2693(0.0342974734194581)	2359 (0.034)	1668 (0.066)
roughage-free	1	5110(0.011432491139819366)	#SAKNAS!	2548 (0.033)
routinely	1	5110(0.011432491139819366)	2359 (0.034)	#SAKNAS!
routinely	1	5110(0.011432491139819366)	2359 (0.034)	#SAKNAS!
roxellana	1	5110(0.011432491139819366)	#SAKNAS!	2548 (0.033)
roy	1	5110(0.011432491139819366)	2359 (0.034)	#SAKNAS!
roy	1	5110(0.011432491139819366)	2359 (0.034)	#SAKNAS!
royal	1	5110(0.011432491139819366)	#SAKNAS!	2548 (0.033)
rs1333049	1	5110(0.011432491139819366)	#SAKNAS!	2548 (0.033)
rs3217992	1	5110(0.011432491139819366)	#SAKNAS!	2548 (0.033)
rs6815464	1	5110(0.011432491139819366)	#SAKNAS!	2548 (0.033)
rs925946	1	5110(0.011432491139819366)	#SAKNAS!	#SAKNAS!
r-sleeve	1	5110(0.011432491139819366)	#SAKNAS!	2548 (0.033)
rsp	1	5110(0.011432491139819366)	#SAKNAS!	2548 (0.033)
rst	1	5110(0.011432491139819366)	#SAKNAS!	#SAKNAS!
rtog	1	3437(0.02286498227963873)	2359 (0.034)	2548 (0.033)
rubicunda	1	5110(0.011432491139819366)	#SAKNAS!	2548 (0.033)
rubus	1	5110(0.011432491139819366)	#SAKNAS!	#SAKNAS!
rubusoside	1	5110(0.011432491139819366)	#SAKNAS!	#SAKNAS!
ruditapes	1	5110(0.011432491139819366)	2359 (0.034)	#SAKNAS!
ruditapes	1	5110(0.011432491139819366)	2359 (0.034)	#SAKNAS!
ruffed	1	5110(0.011432491139819366)	2359 (0.034)	#SAKNAS!
ruffed	1	5110(0.011432491139819366)	2359 (0.034)	#SAKNAS!
rufous-tailed	1	5110(0.011432491139819366)	2359 (0.034)	#SAKNAS!
rufous-tailed	1	5110(0.011432491139819366)	2359 (0.034)	#SAKNAS!
rufus	1	5110(0.011432491139819366)	2359 (0.034)	#SAKNAS!
rufus	1	5110(0.011432491139819366)	2359 (0.034)	#SAKNAS!
rumen-protected	1	5110(0.011432491139819366)	#SAKNAS!	#SAKNAS!
ruminal	1	1678(0.0685949468389162)	2359 (0.034)	1233 (0.1)
ruminant	1	3437(0.02286498227963873)	2359 (0.034)	2548 (0.033)
runners	1	5110(0.011432491139819366)	#SAKNAS!	2548 (0.033)
rural-urban	1	5110(0.011432491139819366)	#SAKNAS!	2548 (0.033)
russet	1	5110(0.011432491139819366)	#SAKNAS!	2548 (0.033)
rvd1	1	5110(0.011432491139819366)	#SAKNAS!	#SAKNAS!

rve1	1	5110(0.011432491139819366)	#SAKNAS!	#SAKNAS!
ryegrass	1	5110(0.011432491139819366)	#SAKNAS!	2548 (0.033)
s148	1	5110(0.011432491139819366)	#SAKNAS!	#SAKNAS!
saanen	1	5110(0.011432491139819366)	#SAKNAS!	2548 (0.033)
sacchari	1	5110(0.011432491139819366)	#SAKNAS!	2548 (0.033)
saccharin	1	5110(0.011432491139819366)	2359 (0.034)	#SAKNAS!
saccharin	1	5110(0.011432491139819366)	2359 (0.034)	#SAKNAS!
saddled	1	5110(0.011432491139819366)	2359 (0.034)	#SAKNAS!
saddled	1	5110(0.011432491139819366)	2359 (0.034)	#SAKNAS!
safely	1	5110(0.011432491139819366)	#SAKNAS!	2548 (0.033)
safeness	1	5110(0.011432491139819366)	#SAKNAS!	#SAKNAS!
safe-swallowing	1	5110(0.011432491139819366)	#SAKNAS!	#SAKNAS!
sagb	1	5110(0.011432491139819366)	2359 (0.034)	#SAKNAS!
sagb	1	5110(0.011432491139819366)	2359 (0.034)	#SAKNAS!
sage	1	5110(0.011432491139819366)	#SAKNAS!	2548 (0.033)
sages	1	5110(0.011432491139819366)	#SAKNAS!	#SAKNAS!
sahel	1	5110(0.011432491139819366)	2359 (0.034)	#SAKNAS!
sahel	1	5110(0.011432491139819366)	2359 (0.034)	#SAKNAS!
saidi	1	5110(0.011432491139819366)	#SAKNAS!	2548 (0.033)
saimiri	1	5110(0.011432491139819366)	#SAKNAS!	2548 (0.033)
sainfoin	1	5110(0.011432491139819366)	#SAKNAS!	2548 (0.033)
sakaka	1	5110(0.011432491139819366)	#SAKNAS!	#SAKNAS!
salaam	1	5110(0.011432491139819366)	2359 (0.034)	#SAKNAS!
salaam	1	5110(0.011432491139819366)	2359 (0.034)	#SAKNAS!
salamanders	1	5110(0.011432491139819366)	#SAKNAS!	#SAKNAS!
salar	1	5110(0.011432491139819366)	2359 (0.034)	#SAKNAS!
salar	1	5110(0.011432491139819366)	2359 (0.034)	#SAKNAS!
saline	1	5110(0.011432491139819366)	2359 (0.034)	#SAKNAS!
saline	1	5110(0.011432491139819366)	2359 (0.034)	#SAKNAS!
salinities	1	5110(0.011432491139819366)	#SAKNAS!	#SAKNAS!
saliva-an	1	5110(0.011432491139819366)	#SAKNAS!	2548 (0.033)
salivary-glands	1	5110(0.011432491139819366)	2359 (0.034)	#SAKNAS!
salivary-glands	1	5110(0.011432491139819366)	2359 (0.034)	#SAKNAS!
salivation	1	5110(0.011432491139819366)	#SAKNAS!	#SAKNAS!
salmo	1	3437(0.02286498227963873)	2359 (0.034)	#SAKNAS!
salmonella	1	2693(0.0342974734194581)	2359 (0.034)	#SAKNAS!
salmonids	1	5110(0.011432491139819366)	#SAKNAS!	2548 (0.033)
salmonis	1	5110(0.011432491139819366)	#SAKNAS!	2548 (0.033)
salpa	1	5110(0.011432491139819366)	#SAKNAS!	2548 (0.033)
salts	1	3437(0.02286498227963873)	2359 (0.034)	#SAKNAS!
salud	1	5110(0.011432491139819366)	2359 (0.034)	#SAKNAS!
salud	1	5110(0.011432491139819366)	2359 (0.034)	#SAKNAS!
salus	1	5110(0.011432491139819366)	#SAKNAS!	#SAKNAS!

salvelinus	1	5110(0.011432491139819366)	2359 (0.034)	#SAKNAS!
salvelinus	1	5110(0.011432491139819366)	2359 (0.034)	#SAKNAS!
samital簀	1	5110(0.011432491139819366)	2359 (0.034)	#SAKNAS!
samital簀	1	5110(0.011432491139819366)	2359 (0.034)	#SAKNAS!
sampling	1	2693(0.0342974734194581)	2359 (0.034)	2548 (0.033)
samurai	1	5110(0.011432491139819366)	2359 (0.034)	#SAKNAS!
samurai	1	5110(0.011432491139819366)	2359 (0.034)	#SAKNAS!
sanative	1	5110(0.011432491139819366)	#SAKNAS!	#SAKNAS!
sander	1	5110(0.011432491139819366)	2359 (0.034)	#SAKNAS!
sander	1	5110(0.011432491139819366)	2359 (0.034)	#SAKNAS!
sandhopper	1	5110(0.011432491139819366)	2359 (0.034)	#SAKNAS!
sandhopper	1	5110(0.011432491139819366)	2359 (0.034)	#SAKNAS!
san-francisco-zoo	1	5110(0.011432491139819366)	2359 (0.034)	#SAKNAS!
san-francisco-zoo	1	5110(0.011432491139819366)	2359 (0.034)	#SAKNAS!
sanitary	1	5110(0.011432491139819366)	2359 (0.034)	#SAKNAS!
sanitary	1	5110(0.011432491139819366)	2359 (0.034)	#SAKNAS!
sanitation	1	5110(0.011432491139819366)	#SAKNAS!	2548 (0.033)
santa	1	5110(0.011432491139819366)	2359 (0.034)	#SAKNAS!
santa	1	5110(0.011432491139819366)	2359 (0.034)	#SAKNAS!
santana	1	5110(0.011432491139819366)	#SAKNAS!	2548 (0.033)
santander	1	5110(0.011432491139819366)	#SAKNAS!	2548 (0.033)
santiago	1	5110(0.011432491139819366)	#SAKNAS!	2548 (0.033)
santos	1	5110(0.011432491139819366)	#SAKNAS!	#SAKNAS!
sap	1	5110(0.011432491139819366)	#SAKNAS!	2548 (0.033)
sapidissima	1	5110(0.011432491139819366)	#SAKNAS!	2548 (0.033)
saplings	1	5110(0.011432491139819366)	2359 (0.034)	#SAKNAS!
saplings	1	5110(0.011432491139819366)	2359 (0.034)	#SAKNAS!
saps	1	5110(0.011432491139819366)	2359 (0.034)	#SAKNAS!
saps	1	5110(0.011432491139819366)	2359 (0.034)	#SAKNAS!
sara	1	3437(0.02286498227963873)	2359 (0.034)	2548 (0.033)
sarasinula-plebeia	1	5110(0.011432491139819366)	2359 (0.034)	#SAKNAS!
sarasinula-plebeia	1	5110(0.011432491139819366)	2359 (0.034)	#SAKNAS!
sardinia	1	5110(0.011432491139819366)	#SAKNAS!	2548 (0.033)
sarpa	1	5110(0.011432491139819366)	#SAKNAS!	2548 (0.033)
sasi	1	5110(0.011432491139819366)	#SAKNAS!	#SAKNAS!
saturation	1	5110(0.011432491139819366)	#SAKNAS!	#SAKNAS!
saude	1	5110(0.011432491139819366)	#SAKNAS!	2548 (0.033)
savannah	1	5110(0.011432491139819366)	#SAKNAS!	#SAKNAS!
savary	1	5110(0.011432491139819366)	#SAKNAS!	2548 (0.033)
savings	1	5110(0.011432491139819366)	#SAKNAS!	2548 (0.033)
saxagliptin	1	5110(0.011432491139819366)	#SAKNAS!	2548 (0.033)
sbma	1	5110(0.011432491139819366)	#SAKNAS!	2548 (0.033)
scalable	1	5110(0.011432491139819366)	#SAKNAS!	2548 (0.033)

scales	1	2214(0.04572996455927746)	2359 (0.034)	1233 (0.1)
scallop	1	3437(0.02286498227963873)	2359 (0.034)	2548 (0.033)
scallops	1	5110(0.011432491139819366)	2359 (0.034)	#SAKNAS!
scallops	1	5110(0.011432491139819366)	2359 (0.034)	#SAKNAS!
scapula	1	5110(0.011432491139819366)	#SAKNAS!	2548 (0.033)
scapular	1	5110(0.011432491139819366)	#SAKNAS!	2548 (0.033)
scarcity	1	5110(0.011432491139819366)	#SAKNAS!	#SAKNAS!
sceloporus	1	5110(0.011432491139819366)	2359 (0.034)	#SAKNAS!
sceloporus	1	5110(0.011432491139819366)	2359 (0.034)	#SAKNAS!
scenario	1	5110(0.011432491139819366)	2359 (0.034)	#SAKNAS!
scenario	1	5110(0.011432491139819366)	2359 (0.034)	#SAKNAS!
scenarios	1	5110(0.011432491139819366)	#SAKNAS!	#SAKNAS!
scent	1	5110(0.011432491139819366)	#SAKNAS!	#SAKNAS!
scfa	1	5110(0.011432491139819366)	#SAKNAS!	2548 (0.033)
schalfeew	1	5110(0.011432491139819366)	2359 (0.034)	#SAKNAS!
schalfeew	1	5110(0.011432491139819366)	2359 (0.034)	#SAKNAS!
schinus	1	5110(0.011432491139819366)	2359 (0.034)	#SAKNAS!
schinus	1	5110(0.011432491139819366)	2359 (0.034)	#SAKNAS!
schistocephalus	1	5110(0.011432491139819366)	2359 (0.034)	#SAKNAS!
schistocephalus	1	5110(0.011432491139819366)	2359 (0.034)	#SAKNAS!
schistocerca-americana	1	5110(0.011432491139819366)	2359 (0.034)	#SAKNAS!
schistocerca-americana	1	5110(0.011432491139819366)	2359 (0.034)	#SAKNAS!
schistosoma-mansoni	1	5110(0.011432491139819366)	2359 (0.034)	#SAKNAS!
schistosoma-mansoni	1	5110(0.011432491139819366)	2359 (0.034)	#SAKNAS!
schizophrenia	1	2214(0.04572996455927746)	2359 (0.034)	2548 (0.033)
school-aged	1	1111(0.11432491139819366)	2359 (0.034)	584 (0.23)
school-based	1	1219(0.1028924202583743)	2359 (0.034)	1233 (0.1)
schoolbooks	1	5110(0.011432491139819366)	2359 (0.034)	#SAKNAS!
schoolbooks	1	5110(0.011432491139819366)	2359 (0.034)	#SAKNAS!
schoolboys	1	5110(0.011432491139819366)	2359 (0.034)	#SAKNAS!
schoolboys	1	5110(0.011432491139819366)	2359 (0.034)	#SAKNAS!
schooled	1	5110(0.011432491139819366)	#SAKNAS!	#SAKNAS!
schooling	1	5110(0.011432491139819366)	#SAKNAS!	2548 (0.033)
schoolteachers	1	5110(0.011432491139819366)	#SAKNAS!	#SAKNAS!
science	1	1894(0.05716245569909683)	2359 (0.034)	1668 (0.066)
scientific	1	1678(0.0685949468389162)	2359 (0.034)	1233 (0.1)
scientists	1	5110(0.011432491139819366)	#SAKNAS!	2548 (0.033)
scientometric	1	5110(0.011432491139819366)	#SAKNAS!	#SAKNAS!
scintigraphic	1	5110(0.011432491139819366)	2359 (0.034)	#SAKNAS!
scintigraphic	1	5110(0.011432491139819366)	2359 (0.034)	#SAKNAS!
scintigraphy	1	5110(0.011432491139819366)	2359 (0.034)	#SAKNAS!
scintigraphy	1	5110(0.011432491139819366)	2359 (0.034)	#SAKNAS!
sciomyzidae	1	5110(0.011432491139819366)	2359 (0.034)	#SAKNAS!

sciomyzidae	1	5110(0.011432491139819366)	2359 (0.034)	#SAKNAS!
scleractinian	1	5110(0.011432491139819366)	#SAKNAS!	2548 (0.033)
scleroderma	1	2693(0.0342974734194581)	2359 (0.034)	2548 (0.033)
sclerosis-validity	1	5110(0.011432491139819366)	#SAKNAS!	#SAKNAS!
scolopendra	1	5110(0.011432491139819366)	#SAKNAS!	2548 (0.033)
scolopendridae	1	5110(0.011432491139819366)	#SAKNAS!	2548 (0.033)
scolopsis	1	5110(0.011432491139819366)	2359 (0.034)	#SAKNAS!
scolopsis	1	5110(0.011432491139819366)	2359 (0.034)	#SAKNAS!
scolytidae	1	5110(0.011432491139819366)	2359 (0.034)	#SAKNAS!
scolytidae	1	5110(0.011432491139819366)	2359 (0.034)	#SAKNAS!
scope	1	5110(0.011432491139819366)	2359 (0.034)	#SAKNAS!
scope	1	5110(0.011432491139819366)	2359 (0.034)	#SAKNAS!
scopolamine	1	5110(0.011432491139819366)	#SAKNAS!	2548 (0.033)
score	1	244(0.5258945924316909)	2359 (0.034)	228 (0.59)
scores	1	1894(0.05716245569909683)	2359 (0.034)	1668 (0.066)
score-weighted	1	5110(0.011432491139819366)	#SAKNAS!	#SAKNAS!
scoring	1	3437(0.02286498227963873)	2359 (0.034)	2548 (0.033)
scrapes	1	5110(0.011432491139819366)	2359 (0.034)	#SAKNAS!
scrapes	1	5110(0.011432491139819366)	2359 (0.034)	#SAKNAS!
screen	1	1335(0.09145992911855493)	2359 (0.034)	2548 (0.033)
screw	1	5110(0.011432491139819366)	#SAKNAS!	#SAKNAS!
screw-rod	1	5110(0.011432491139819366)	#SAKNAS!	#SAKNAS!
scrofa	1	5110(0.011432491139819366)	#SAKNAS!	#SAKNAS!
scrubland	1	5110(0.011432491139819366)	2359 (0.034)	#SAKNAS!
scrubland	1	5110(0.011432491139819366)	2359 (0.034)	#SAKNAS!
sct	1	5110(0.011432491139819366)	2359 (0.034)	#SAKNAS!
sct	1	5110(0.011432491139819366)	2359 (0.034)	#SAKNAS!
scurvy	1	5110(0.011432491139819366)	2359 (0.034)	#SAKNAS!
scurvy	1	5110(0.011432491139819366)	2359 (0.034)	#SAKNAS!
scylla	1	5110(0.011432491139819366)	2359 (0.034)	#SAKNAS!
scylla	1	5110(0.011432491139819366)	2359 (0.034)	#SAKNAS!
scyphomedusan	1	5110(0.011432491139819366)	2359 (0.034)	#SAKNAS!
scyphomedusan	1	5110(0.011432491139819366)	2359 (0.034)	#SAKNAS!
sdor.2-6y?□	1	5110(0.011432491139819366)	#SAKNAS!	#SAKNAS!
sdq-dp	1	5110(0.011432491139819366)	#SAKNAS!	#SAKNAS!
seahorse	1	5110(0.011432491139819366)	#SAKNAS!	2548 (0.033)
seal	1	5110(0.011432491139819366)	2359 (0.034)	#SAKNAS!
seal	1	5110(0.011432491139819366)	2359 (0.034)	#SAKNAS!
sealant	1	2693(0.0342974734194581)	2359 (0.034)	2548 (0.033)
searching	1	5110(0.011432491139819366)	2359 (0.034)	#SAKNAS!
searching	1	5110(0.011432491139819366)	2359 (0.034)	#SAKNAS!
seasonally-breeding	1	5110(0.011432491139819366)	2359 (0.034)	#SAKNAS!
seasonally-breeding	1	5110(0.011432491139819366)	2359 (0.034)	#SAKNAS!

seasonal-variation	1	5110(0.011432491139819366)	2359 (0.034)	#SAKNAS!
seasonal-variation	1	5110(0.011432491139819366)	2359 (0.034)	#SAKNAS!
sea-urchin	1	5110(0.011432491139819366)	2359 (0.034)	#SAKNAS!
sea-urchin	1	5110(0.011432491139819366)	2359 (0.034)	#SAKNAS!
seaweed	1	3437(0.02286498227963873)	2359 (0.034)	#SAKNAS!
seaweeds	1	5110(0.011432491139819366)	2359 (0.034)	#SAKNAS!
seaweeds	1	5110(0.011432491139819366)	2359 (0.034)	#SAKNAS!
secondary-human	1	5110(0.011432491139819366)	#SAKNAS!	#SAKNAS!
second-grade	1	5110(0.011432491139819366)	#SAKNAS!	#SAKNAS!
secondhand	1	2214(0.04572996455927746)	2359 (0.034)	2548 (0.033)
second-hand	1	5110(0.011432491139819366)	#SAKNAS!	2548 (0.033)
second-year	1	5110(0.011432491139819366)	#SAKNAS!	#SAKNAS!
secretory	1	5110(0.011432491139819366)	#SAKNAS!	2548 (0.033)
section	1	5110(0.011432491139819366)	2359 (0.034)	#SAKNAS!
section	1	5110(0.011432491139819366)	2359 (0.034)	#SAKNAS!
secular	1	5110(0.011432491139819366)	#SAKNAS!	#SAKNAS!
sedentary	1	1485(0.08002743797873557)	2359 (0.034)	2548 (0.033)
sedreno	1	5110(0.011432491139819366)	#SAKNAS!	#SAKNAS!
seed-caching	1	5110(0.011432491139819366)	#SAKNAS!	2548 (0.033)
seeding	1	5110(0.011432491139819366)	#SAKNAS!	#SAKNAS!
seek	1	5110(0.011432491139819366)	#SAKNAS!	#SAKNAS!
seeking	1	3437(0.02286498227963873)	2359 (0.034)	2548 (0.033)
seesaw	1	5110(0.011432491139819366)	#SAKNAS!	#SAKNAS!
seferihisar	1	5110(0.011432491139819366)	#SAKNAS!	#SAKNAS!
segment	1	2693(0.0342974734194581)	2359 (0.034)	2548 (0.033)
segmental	1	5110(0.011432491139819366)	#SAKNAS!	#SAKNAS!
segmentation	1	3437(0.02286498227963873)	2359 (0.034)	2548 (0.033)
selecting	1	5110(0.011432491139819366)	#SAKNAS!	2548 (0.033)
selenium	1	5110(0.011432491139819366)	#SAKNAS!	2548 (0.033)
selenomonas	1	5110(0.011432491139819366)	#SAKNAS!	#SAKNAS!
self	1	5110(0.011432491139819366)	2359 (0.034)	#SAKNAS!
self	1	5110(0.011432491139819366)	2359 (0.034)	#SAKNAS!
self-cognitive	1	5110(0.011432491139819366)	#SAKNAS!	#SAKNAS!
self-controlled	1	5110(0.011432491139819366)	#SAKNAS!	#SAKNAS!
self-design	1	5110(0.011432491139819366)	2359 (0.034)	#SAKNAS!
self-design	1	5110(0.011432491139819366)	2359 (0.034)	#SAKNAS!
self-dilation	1	5110(0.011432491139819366)	#SAKNAS!	2548 (0.033)
self-discrepancy	1	5110(0.011432491139819366)	2359 (0.034)	#SAKNAS!
self-discrepancy	1	5110(0.011432491139819366)	2359 (0.034)	#SAKNAS!
self-disintegrating	1	5110(0.011432491139819366)	#SAKNAS!	#SAKNAS!
self-educational	1	5110(0.011432491139819366)	#SAKNAS!	2548 (0.033)
self-esophageal	1	5110(0.011432491139819366)	#SAKNAS!	2548 (0.033)
self-fed	1	5110(0.011432491139819366)	#SAKNAS!	2548 (0.033)

selffeeders	1	5110(0.011432491139819366)	2359 (0.034)	#SAKNAS!
selffeeders	1	5110(0.011432491139819366)	2359 (0.034)	#SAKNAS!
self-feeding	1	2693(0.0342974734194581)	2359 (0.034)	1668 (0.066)
self-guided	1	5110(0.011432491139819366)	#SAKNAS!	#SAKNAS!
self-inflatable	1	5110(0.011432491139819366)	#SAKNAS!	#SAKNAS!
self-inflicted	1	5110(0.011432491139819366)	#SAKNAS!	#SAKNAS!
self-regulation	1	3437(0.02286498227963873)	2359 (0.034)	2548 (0.033)
self-schema	1	5110(0.011432491139819366)	#SAKNAS!	2548 (0.033)
self-selecting	1	5110(0.011432491139819366)	2359 (0.034)	#SAKNAS!
self-selecting	1	5110(0.011432491139819366)	2359 (0.034)	#SAKNAS!
self-sufficient	1	5110(0.011432491139819366)	#SAKNAS!	#SAKNAS!
selseyense	1	5110(0.011432491139819366)	#SAKNAS!	#SAKNAS!
semaglutide-associated	1	5110(0.011432491139819366)	#SAKNAS!	#SAKNAS!
semi	1	5110(0.011432491139819366)	2359 (0.034)	#SAKNAS!
semi	1	5110(0.011432491139819366)	2359 (0.034)	#SAKNAS!
semiarid	1	3437(0.02286498227963873)	2359 (0.034)	#SAKNAS!
semi-arid	1	3437(0.02286498227963873)	2359 (0.034)	#SAKNAS!
semiconductor	1	5110(0.011432491139819366)	#SAKNAS!	2548 (0.033)
semi-dynamic	1	5110(0.011432491139819366)	#SAKNAS!	#SAKNAS!
semiology	1	5110(0.011432491139819366)	#SAKNAS!	#SAKNAS!
semi-quantitative	1	5110(0.011432491139819366)	#SAKNAS!	#SAKNAS!
semisynthetic	1	3437(0.02286498227963873)	2359 (0.034)	#SAKNAS!
senc	1	5110(0.011432491139819366)	#SAKNAS!	2548 (0.033)
senecio	1	5110(0.011432491139819366)	#SAKNAS!	#SAKNAS!
senegal	1	5110(0.011432491139819366)	2359 (0.034)	#SAKNAS!
senegal	1	5110(0.011432491139819366)	2359 (0.034)	#SAKNAS!
senegalese	1	3437(0.02286498227963873)	2359 (0.034)	2548 (0.033)
senex	1	5110(0.011432491139819366)	#SAKNAS!	2548 (0.033)
senorita	1	5110(0.011432491139819366)	#SAKNAS!	#SAKNAS!
sensation	1	5110(0.011432491139819366)	#SAKNAS!	2548 (0.033)
sense	1	5110(0.011432491139819366)	#SAKNAS!	2548 (0.033)
sensilla	1	5110(0.011432491139819366)	#SAKNAS!	2548 (0.033)
sensitive	1	5110(0.011432491139819366)	#SAKNAS!	2548 (0.033)
sensitive-feeding	1	5110(0.011432491139819366)	#SAKNAS!	#SAKNAS!
sensor	1	3437(0.02286498227963873)	2359 (0.034)	2548 (0.033)
separating	1	5110(0.011432491139819366)	2359 (0.034)	#SAKNAS!
separating	1	5110(0.011432491139819366)	2359 (0.034)	#SAKNAS!
sepia	1	5110(0.011432491139819366)	2359 (0.034)	#SAKNAS!
sepia	1	5110(0.011432491139819366)	2359 (0.034)	#SAKNAS!
sepsis	1	5110(0.011432491139819366)	#SAKNAS!	2548 (0.033)
septic	1	5110(0.011432491139819366)	#SAKNAS!	#SAKNAS!
sequelae	1	2693(0.0342974734194581)	2359 (0.034)	1668 (0.066)
sequence	1	5110(0.011432491139819366)	2359 (0.034)	#SAKNAS!

sequence	1	5110(0.011432491139819366)	2359 (0.034)	#SAKNAS!
ser326cys	1	5110(0.011432491139819366)	2359 (0.034)	#SAKNAS!
ser326cys	1	5110(0.011432491139819366)	2359 (0.034)	#SAKNAS!
serbia	1	5110(0.011432491139819366)	#SAKNAS!	#SAKNAS!
serial	1	5110(0.011432491139819366)	#SAKNAS!	#SAKNAS!
sericin	1	5110(0.011432491139819366)	#SAKNAS!	#SAKNAS!
serologic	1	5110(0.011432491139819366)	#SAKNAS!	2548 (0.033)
serotonergic	1	3437(0.02286498227963873)	2359 (0.034)	#SAKNAS!
serotonin2c	1	5110(0.011432491139819366)	2359 (0.034)	#SAKNAS!
serotonin2c	1	5110(0.011432491139819366)	2359 (0.034)	#SAKNAS!
serotype	1	5110(0.011432491139819366)	#SAKNAS!	2548 (0.033)
serovar	1	5110(0.011432491139819366)	#SAKNAS!	#SAKNAS!
serovars	1	5110(0.011432491139819366)	2359 (0.034)	#SAKNAS!
serovars	1	5110(0.011432491139819366)	2359 (0.034)	#SAKNAS!
serrata	1	5110(0.011432491139819366)	2359 (0.034)	#SAKNAS!
serrata	1	5110(0.011432491139819366)	2359 (0.034)	#SAKNAS!
sertoli	1	5110(0.011432491139819366)	#SAKNAS!	2548 (0.033)
serum-saliva	1	5110(0.011432491139819366)	#SAKNAS!	2548 (0.033)
service-health	1	5110(0.011432491139819366)	#SAKNAS!	2548 (0.033)
service-learning	1	5110(0.011432491139819366)	#SAKNAS!	#SAKNAS!
ses	1	5110(0.011432491139819366)	2359 (0.034)	#SAKNAS!
ses	1	5110(0.011432491139819366)	2359 (0.034)	#SAKNAS!
sesarma	1	5110(0.011432491139819366)	#SAKNAS!	2548 (0.033)
sesarma-messa	1	5110(0.011432491139819366)	2359 (0.034)	#SAKNAS!
sesarma-messa	1	5110(0.011432491139819366)	2359 (0.034)	#SAKNAS!
sesarma-smithii	1	5110(0.011432491139819366)	2359 (0.034)	#SAKNAS!
sesarma-smithii	1	5110(0.011432491139819366)	2359 (0.034)	#SAKNAS!
sesarmidae	1	3437(0.02286498227963873)	2359 (0.034)	2548 (0.033)
set	1	2693(0.0342974734194581)	2359 (0.034)	#SAKNAS!
settings-a	1	5110(0.011432491139819366)	#SAKNAS!	#SAKNAS!
settlement	1	5110(0.011432491139819366)	#SAKNAS!	#SAKNAS!
settlements	1	5110(0.011432491139819366)	#SAKNAS!	#SAKNAS!
seven	1	5110(0.011432491139819366)	#SAKNAS!	#SAKNAS!
seven-repeat	1	5110(0.011432491139819366)	#SAKNAS!	2548 (0.033)
seven-year	1	2693(0.0342974734194581)	2359 (0.034)	2548 (0.033)
seville	1	5110(0.011432491139819366)	2359 (0.034)	#SAKNAS!
seville	1	5110(0.011432491139819366)	2359 (0.034)	#SAKNAS!
sex-dependent	1	5110(0.011432491139819366)	#SAKNAS!	#SAKNAS!
sexually	1	1894(0.05716245569909683)	2359 (0.034)	1233 (0.1)
shad	1	5110(0.011432491139819366)	#SAKNAS!	2548 (0.033)
shapes	1	5110(0.011432491139819366)	#SAKNAS!	#SAKNAS!
shaping	1	3437(0.02286498227963873)	2359 (0.034)	#SAKNAS!
share	1	5110(0.011432491139819366)	#SAKNAS!	2548 (0.033)

sharpi	1	3437(0.0228649822796387З)	2359 (0.034)	2548 (0.033)
sharpnose	1	5110(0.011432491139819366)	#SAKNAS!	2548 (0.033)
she	1	5110(0.011432491139819366)	#SAKNAS!	2548 (0.033)
shearwater	1	3437(0.02286498227963873)	2359 (0.034)	2548 (0.033)
shear-wave	1	5110(0.011432491139819366)	#SAKNAS!	#SAKNAS!
shellfish	1	3437(0.02286498227963873)	2359 (0.034)	2548 (0.033)
sheltering	1	5110(0.011432491139819366)	#SAKNAS!	#SAKNAS!
shelters	1	5110(0.011432491139819366)	#SAKNAS!	#SAKNAS!
shemya	1	5110(0.011432491139819366)	2359 (0.034)	#SAKNAS!
shemya	1	5110(0.011432491139819366)	2359 (0.034)	#SAKNAS!
shenzhen	1	5110(0.011432491139819366)	#SAKNAS!	2548 (0.033)
shift	1	1894(0.05716245569909683)	2359 (0.034)	1668 (0.066)
shifts	1	2214(0.04572996455927746)	2359 (0.034)	2548 (0.033)
shinkaia	1	5110(0.011432491139819366)	#SAKNAS!	2548 (0.033)
ship-0	1	5110(0.011432491139819366)	2359 (0.034)	#SAKNAS!
ship-0	1	5110(0.011432491139819366)	2359 (0.034)	#SAKNAS!
shoaling	1	5110(0.011432491139819366)	2359 (0.034)	#SAKNAS!
shoaling	1	5110(0.011432491139819366)	2359 (0.034)	#SAKNAS!
shock	1	5110(0.011432491139819366)	#SAKNAS!	#SAKNAS!
short-acting	1	5110(0.011432491139819366)	2359 (0.034)	#SAKNAS!
short-acting	1	5110(0.011432491139819366)	2359 (0.034)	#SAKNAS!
shortage	1	2693(0.0342974734194581)	2359 (0.034)	1668 (0.066)
shortages	1	5110(0.011432491139819366)	#SAKNAS!	#SAKNAS!
short-bowel	1	5110(0.011432491139819366)	2359 (0.034)	#SAKNAS!
short-bowel	1	5110(0.011432491139819366)	2359 (0.034)	#SAKNAS!
short-chain	1	3437(0.02286498227963873)	2359 (0.034)	#SAKNAS!
shorter	1	3437(0.02286498227963873)	2359 (0.034)	#SAKNAS!
short-limb	1	5110(0.011432491139819366)	2359 (0.034)	#SAKNAS!
short-limb	1	5110(0.011432491139819366)	2359 (0.034)	#SAKNAS!
shredder	1	5110(0.011432491139819366)	2359 (0.034)	#SAKNAS!
shredder	1	5110(0.011432491139819366)	2359 (0.034)	#SAKNAS!
shredders	1	5110(0.011432491139819366)	2359 (0.034)	#SAKNAS!
shredders	1	5110(0.011432491139819366)	2359 (0.034)	#SAKNAS!
shrikhand	1	5110(0.011432491139819366)	#SAKNAS!	#SAKNAS!
shrinkage	1	5110(0.011432491139819366)	#SAKNAS!	2548 (0.033)
sialorrhea	1	2693(0.0342974734194581)	2359 (0.034)	2548 (0.033)
sibship	1	5110(0.011432491139819366)	#SAKNAS!	#SAKNAS!
sibutramine	1	5110(0.011432491139819366)	2359 (0.034)	#SAKNAS!
sibutramine	1	5110(0.011432491139819366)	2359 (0.034)	#SAKNAS!
sichuan	1	5110(0.011432491139819366)	#SAKNAS!	2548 (0.033)
sicilian	1	5110(0.011432491139819366)	2359 (0.034)	#SAKNAS!
sicilian	1	5110(0.011432491139819366)	2359 (0.034)	#SAKNAS!
sickness-like	1	5110(0.011432491139819366)	2359 (0.034)	#SAKNAS!

sickness-like	1	5110(0.011432491139819366)	2359 (0.034)	#SAKNAS!
side	1	2693(0.0342974734194581)	2359 (0.034)	1668 (0.066)
side-effects	1	5110(0.011432491139819366)	#SAKNAS!	#SAKNAS!
sid-sidp-amd	1	5110(0.011432491139819366)	#SAKNAS!	#SAKNAS!
sierra	1	5110(0.011432491139819366)	2359 (0.034)	#SAKNAS!
sierra	1	5110(0.011432491139819366)	2359 (0.034)	#SAKNAS!
sieving	1	5110(0.011432491139819366)	#SAKNAS!	2548 (0.033)
significantly	1	1894(0.0571624556990968З)	2359 (0.034)	2548 (0.033)
signs	1	1485(0.08002743797873557)	2359 (0.034)	999 (0.13)
sii	1	5110(0.011432491139819366)	#SAKNAS!	#SAKNAS!
sika	1	5110(0.011432491139819366)	#SAKNAS!	2548 (0.033)
silage-based	1	5110(0.011432491139819366)	#SAKNAS!	2548 (0.033)
silastic	1	5110(0.011432491139819366)	2359 (0.034)	#SAKNAS!
silastic	1	5110(0.011432491139819366)	2359 (0.034)	#SAKNAS!
silenus	1	5110(0.011432491139819366)	2359 (0.034)	#SAKNAS!
silenus	1	5110(0.011432491139819366)	2359 (0.034)	#SAKNAS!
siler	1	5110(0.011432491139819366)	2359 (0.034)	#SAKNAS!
siler	1	5110(0.011432491139819366)	2359 (0.034)	#SAKNAS!
silhouette	1	5110(0.011432491139819366)	#SAKNAS!	#SAKNAS!
silicon	1	5110(0.011432491139819366)	2359 (0.034)	#SAKNAS!
silicon	1	5110(0.011432491139819366)	2359 (0.034)	#SAKNAS!
silicon-based	1	5110(0.011432491139819366)	#SAKNAS!	2548 (0.033)
silicon-covered	1	5110(0.011432491139819366)	2359 (0.034)	#SAKNAS!
silicon-covered	1	5110(0.011432491139819366)	2359 (0.034)	#SAKNAS!
silkworms	1	5110(0.011432491139819366)	#SAKNAS!	2548 (0.033)
silver	1	5110(0.011432491139819366)	#SAKNAS!	#SAKNAS!
silvery	1	5110(0.011432491139819366)	#SAKNAS!	#SAKNAS!
similarities	1	5110(0.011432491139819366)	#SAKNAS!	#SAKNAS!
similarity	1	5110(0.011432491139819366)	2359 (0.034)	#SAKNAS!
similarity	1	5110(0.011432491139819366)	2359 (0.034)	#SAKNAS!
similarly	1	5110(0.011432491139819366)	#SAKNAS!	#SAKNAS!
simmental	1	5110(0.011432491139819366)	#SAKNAS!	2548 (0.033)
simple-to-use	1	5110(0.011432491139819366)	#SAKNAS!	#SAKNAS!
simulant	1	5110(0.011432491139819366)	#SAKNAS!	#SAKNAS!
simulations	1	5110(0.011432491139819366)	#SAKNAS!	2548 (0.033)
simultaneously	1	5110(0.011432491139819366)	#SAKNAS!	#SAKNAS!
sin	1	5110(0.011432491139819366)	2359 (0.034)	#SAKNAS!
sin	1	5110(0.011432491139819366)	2359 (0.034)	#SAKNAS!
since	1	2214(0.04572996455927746)	2359 (0.034)	1233 (0.1)
singaporean	1	5110(0.011432491139819366)	#SAKNAS!	#SAKNAS!
single-bearing	1	5110(0.011432491139819366)	#SAKNAS!	2548 (0.033)
single-dose	1	5110(0.011432491139819366)	#SAKNAS!	2548 (0.033)
single-flap	1	5110(0.011432491139819366)	#SAKNAS!	#SAKNAS!

single-food	1	5110(0.011432491139819366)	#SAKNAS!	#SAKNAS!
single-item	1	5110(0.011432491139819366)	2359 (0.034)	#SAKNAS!
single-item	1	5110(0.011432491139819366)	2359 (0.034)	#SAKNAS!
single-nucleus	1	5110(0.011432491139819366)	#SAKNAS!	#SAKNAS!
single-pan	1	5110(0.011432491139819366)	#SAKNAS!	2548 (0.033)
single-site	1	5110(0.011432491139819366)	2359 (0.034)	#SAKNAS!
single-site	1	5110(0.011432491139819366)	2359 (0.034)	#SAKNAS!
single-space	1	5110(0.011432491139819366)	2359 (0.034)	#SAKNAS!
single-space	1	5110(0.011432491139819366)	2359 (0.034)	#SAKNAS!
single-stage	1	5110(0.011432491139819366)	2359 (0.034)	#SAKNAS!
single-stage	1	5110(0.011432491139819366)	2359 (0.034)	#SAKNAS!
siniperca	1	5110(0.011432491139819366)	#SAKNAS!	#SAKNAS!
sinpe	1	5110(0.011432491139819366)	#SAKNAS!	#SAKNAS!
sinus	1	5110(0.011432491139819366)	2359 (0.034)	#SAKNAS!
sinus	1	5110(0.011432491139819366)	2359 (0.034)	#SAKNAS!
siri	1	5110(0.011432491139819366)	#SAKNAS!	#SAKNAS!
sirt1	1	5110(0.011432491139819366)	#SAKNAS!	#SAKNAS!
sit-and-wait	1	5110(0.011432491139819366)	2359 (0.034)	#SAKNAS!
sit-and-wait	1	5110(0.011432491139819366)	2359 (0.034)	#SAKNAS!
sitchensis	1	5110(0.011432491139819366)	2359 (0.034)	#SAKNAS!
sitchensis	1	5110(0.011432491139819366)	2359 (0.034)	#SAKNAS!
site-specific	1	5110(0.011432491139819366)	#SAKNAS!	#SAKNAS!
sitka	1	5110(0.011432491139819366)	2359 (0.034)	#SAKNAS!
sitka	1	5110(0.011432491139819366)	2359 (0.034)	#SAKNAS!
sitophilus	1	5110(0.011432491139819366)	#SAKNAS!	#SAKNAS!
situ	1	5110(0.011432491139819366)	2359 (0.034)	#SAKNAS!
situ	1	5110(0.011432491139819366)	2359 (0.034)	#SAKNAS!
situacion	1	5110(0.011432491139819366)	#SAKNAS!	2548 (0.033)
situation	1	2214(0.04572996455927746)	2359 (0.034)	1668 (0.066)
sixmonth	1	5110(0.011432491139819366)	#SAKNAS!	2548 (0.033)
six-month	1	5110(0.011432491139819366)	2359 (0.034)	#SAKNAS!
six-month	1	5110(0.011432491139819366)	2359 (0.034)	#SAKNAS!
sjogren	1	2214(0.04572996455927746)	2359 (0.034)	1668 (0.066)
sjs	1	5110(0.011432491139819366)	2359 (0.034)	#SAKNAS!
sjs	1	5110(0.011432491139819366)	2359 (0.034)	#SAKNAS!
skeletal-dental	1	5110(0.011432491139819366)	2359 (0.034)	#SAKNAS!
skeletal-dental	1	5110(0.011432491139819366)	2359 (0.034)	#SAKNAS!
skills	1	2214(0.04572996455927746)	2359 (0.034)	1233 (0.1)
skin	1	1894(0.05716245569909683)	2359 (0.034)	1668 (0.066)
skinfold	1	5110(0.011432491139819366)	2359 (0.034)	#SAKNAS!
skinfold	1	5110(0.011432491139819366)	2359 (0.034)	#SAKNAS!
sleeping	1	5110(0.011432491139819366)	#SAKNAS!	2548 (0.033)
slight	1	5110(0.011432491139819366)	#SAKNAS!	2548 (0.033)

slings	1	5110(0.011432491139819366)	#SAKNAS!	#SAKNAS!
slippage	1	5110(0.011432491139819366)	2359 (0.034)	#SAKNAS!
slippage	1	5110(0.011432491139819366)	2359 (0.034)	#SAKNAS!
slipper	1	5110(0.011432491139819366)	#SAKNAS!	2548 (0.033)
sloan-kettering	1	5110(0.011432491139819366)	2359 (0.034)	#SAKNAS!
sloan-kettering	1	5110(0.011432491139819366)	2359 (0.034)	#SAKNAS!
slovakia	1	5110(0.011432491139819366)	#SAKNAS!	2548 (0.033)
slovenia	1	5110(0.011432491139819366)	#SAKNAS!	2548 (0.033)
slower	1	5110(0.011432491139819366)	2359 (0.034)	#SAKNAS!
slower	1	5110(0.011432491139819366)	2359 (0.034)	#SAKNAS!
slpi	1	5110(0.011432491139819366)	#SAKNAS!	2548 (0.033)
slums	1	5110(0.011432491139819366)	#SAKNAS!	2548 (0.033)
small-area	1	5110(0.011432491139819366)	#SAKNAS!	#SAKNAS!
small-scale	1	5110(0.011432491139819366)	#SAKNAS!	2548 (0.033)
smart	1	5110(0.011432491139819366)	#SAKNAS!	#SAKNAS!
smart-card-based	1	5110(0.011432491139819366)	2359 (0.034)	#SAKNAS!
smart-card-based	1	5110(0.011432491139819366)	2359 (0.034)	#SAKNAS!
smartfeeding4kids	1	5110(0.011432491139819366)	#SAKNAS!	#SAKNAS!
smartphone	1	5110(0.011432491139819366)	#SAKNAS!	2548 (0.033)
smiles	1	5110(0.011432491139819366)	#SAKNAS!	2548 (0.033)
smokeless	1	5110(0.011432491139819366)	2359 (0.034)	#SAKNAS!
smokeless	1	5110(0.011432491139819366)	2359 (0.034)	#SAKNAS!
smoking-attributable	1	5110(0.011432491139819366)	2359 (0.034)	#SAKNAS!
smoking-attributable	1	5110(0.011432491139819366)	2359 (0.034)	#SAKNAS!
smooth	1	5110(0.011432491139819366)	#SAKNAS!	2548 (0.033)
smooth-surface	1	5110(0.011432491139819366)	2359 (0.034)	#SAKNAS!
smooth-surface	1	5110(0.011432491139819366)	2359 (0.034)	#SAKNAS!
snack	1	1678(0.0685949468389162)	2359 (0.034)	1233 (0.1)
snail-killing	1	5110(0.011432491139819366)	2359 (0.034)	#SAKNAS!
snail-killing	1	5110(0.011432491139819366)	2359 (0.034)	#SAKNAS!
snakes	1	5110(0.011432491139819366)	#SAKNAS!	#SAKNAS!
snaq	1	5110(0.011432491139819366)	#SAKNAS!	#SAKNAS!
snare	1	5110(0.011432491139819366)	#SAKNAS!	2548 (0.033)
snoring	1	5110(0.011432491139819366)	2359 (0.034)	#SAKNAS!
snoring	1	5110(0.011432491139819366)	2359 (0.034)	#SAKNAS!
snow-covered	1	5110(0.011432491139819366)	#SAKNAS!	2548 (0.033)
snpf	1	5110(0.011432491139819366)	2359 (0.034)	#SAKNAS!
snpf	1	5110(0.011432491139819366)	2359 (0.034)	#SAKNAS!
snuffers	1	5110(0.011432491139819366)	#SAKNAS!	2548 (0.033)
snuffing	1	5110(0.011432491139819366)	#SAKNAS!	2548 (0.033)
sobral	1	5110(0.011432491139819366)	2359 (0.034)	#SAKNAS!
sobral	1	5110(0.011432491139819366)	2359 (0.034)	#SAKNAS!
sociality	1	5110(0.011432491139819366)	2359 (0.034)	#SAKNAS!

sociality	1	5110(0.011432491139819366)	2359 (0.034)	#SAKNAS!
socialization-based	1	5110(0.011432491139819366)	#SAKNAS!	#SAKNAS!
sociedad	1	5110(0.011432491139819366)	#SAKNAS!	2548 (0.033)
societal	1	5110(0.011432491139819366)	#SAKNAS!	#SAKNAS!
societies	1	3437(0.02286498227963873)	2359 (0.034)	#SAKNAS!
socio-behavioural	1	5110(0.011432491139819366)	2359 (0.034)	#SAKNAS!
socio-behavioural	1	5110(0.011432491139819366)	2359 (0.034)	#SAKNAS!
socio-ecological	1	5110(0.011432491139819366)	#SAKNAS!	#SAKNAS!
socioeconomic-oral	1	5110(0.011432491139819366)	2359 (0.034)	#SAKNAS!
socioeconomic-oral	1	5110(0.011432491139819366)	2359 (0.034)	#SAKNAS!
socioeconomics	1	5110(0.011432491139819366)	#SAKNAS!	#SAKNAS!
sociofamilial	1	5110(0.011432491139819366)	2359 (0.034)	#SAKNAS!
sociofamilial	1	5110(0.011432491139819366)	2359 (0.034)	#SAKNAS!
socolofi	1	5110(0.011432491139819366)	2359 (0.034)	#SAKNAS!
socolofi	1	5110(0.011432491139819366)	2359 (0.034)	#SAKNAS!
sod1	1	5110(0.011432491139819366)	#SAKNAS!	#SAKNAS!
sod1g93a	1	5110(0.011432491139819366)	#SAKNAS!	#SAKNAS!
sodium	1	1219(0.1028924202583743)	2359 (0.034)	816 (0.16)
sofia	1	5110(0.011432491139819366)	#SAKNAS!	2548 (0.033)
soft-bottom	1	5110(0.011432491139819366)	2359 (0.034)	#SAKNAS!
soft-bottom	1	5110(0.011432491139819366)	2359 (0.034)	#SAKNAS!
softening	1	5110(0.011432491139819366)	2359 (0.034)	#SAKNAS!
softening	1	5110(0.011432491139819366)	2359 (0.034)	#SAKNAS!
soft-pellet	1	5110(0.011432491139819366)	#SAKNAS!	#SAKNAS!
soft-textured	1	5110(0.011432491139819366)	#SAKNAS!	#SAKNAS!
soft-tissue	1	5110(0.011432491139819366)	2359 (0.034)	#SAKNAS!
soft-tissue	1	5110(0.011432491139819366)	2359 (0.034)	#SAKNAS!
soil	1	2693(0.0342974734194581)	2359 (0.034)	1668 (0.066)
sol	1	5110(0.011432491139819366)	#SAKNAS!	2548 (0.033)
sold	1	5110(0.011432491139819366)	#SAKNAS!	#SAKNAS!
soldiers	1	5110(0.011432491139819366)	#SAKNAS!	2548 (0.033)
sole	1	5110(0.011432491139819366)	2359 (0.034)	#SAKNAS!
sole	1	5110(0.011432491139819366)	2359 (0.034)	#SAKNAS!
solid	1	1485(0.08002743797873557)	2359 (0.034)	694 (0.2)
solidus	1	5110(0.011432491139819366)	2359 (0.034)	#SAKNAS!
solidus	1	5110(0.011432491139819366)	2359 (0.034)	#SAKNAS!
solitarii	1	5110(0.011432491139819366)	2359 (0.034)	#SAKNAS!
solitarii	1	5110(0.011432491139819366)	2359 (0.034)	#SAKNAS!
solomon	1	5110(0.011432491139819366)	#SAKNAS!	#SAKNAS!
solubility	1	5110(0.011432491139819366)	#SAKNAS!	2548 (0.033)
solution	1	2693(0.0342974734194581)	2359 (0.034)	1668 (0.066)
somali	1	5110(0.011432491139819366)	#SAKNAS!	2548 (0.033)
sonnic	1	5110(0.011432491139819366)	#SAKNAS!	#SAKNAS!

sonrisa	1	5110(0.011432491139819366)	#SAKNAS!	#SAKNAS!
soothe	1	5110(0.011432491139819366)	#SAKNAS!	#SAKNAS!
sophias	1	5110(0.011432491139819366)	#SAKNAS!	2548 (0.033)
sorbet	1	5110(0.011432491139819366)	#SAKNAS!	#SAKNAS!
sorbitol	1	5110(0.011432491139819366)	#SAKNAS!	2548 (0.033)
sore	1	5110(0.011432491139819366)	2359 (0.034)	#SAKNAS!
sore	1	5110(0.011432491139819366)	2359 (0.034)	#SAKNAS!
sorghum-based	1	5110(0.011432491139819366)	#SAKNAS!	#SAKNAS!
sort	1	5110(0.011432491139819366)	#SAKNAS!	#SAKNAS!
sothern	1	5110(0.011432491139819366)	#SAKNAS!	#SAKNAS!
sound	1	5110(0.011432491139819366)	2359 (0.034)	#SAKNAS!
sound	1	5110(0.011432491139819366)	2359 (0.034)	#SAKNAS!
sounds	1	5110(0.011432491139819366)	#SAKNAS!	2548 (0.033)
sous-vide	1	5110(0.011432491139819366)	#SAKNAS!	#SAKNAS!
south-bahia	1	5110(0.011432491139819366)	#SAKNAS!	2548 (0.033)
southeast	1	2214(0.04572996455927746)	2359 (0.034)	#SAKNAS!
south-east	1	5110(0.011432491139819366)	2359 (0.034)	#SAKNAS!
south-east	1	5110(0.011432491139819366)	2359 (0.034)	#SAKNAS!
southeastern	1	2693(0.0342974734194581)	2359 (0.034)	1668 (0.066)
south-eastern	1	5110(0.011432491139819366)	#SAKNAS!	2548 (0.033)
south-east-european	1	5110(0.011432491139819366)	2359 (0.034)	#SAKNAS!
south-east-european	1	5110(0.011432491139819366)	2359 (0.034)	#SAKNAS!
southwest	1	1678(0.0685949468389162)	2359 (0.034)	999 (0.13)
south-west	1	5110(0.011432491139819366)	2359 (0.034)	#SAKNAS!
south-west	1	5110(0.011432491139819366)	2359 (0.034)	#SAKNAS!
southwestern	1	5110(0.011432491139819366)	#SAKNAS!	2548 (0.033)
sow	1	3437(0.02286498227963873)	2359 (0.034)	#SAKNAS!
soy	1	2693(0.0342974734194581)	2359 (0.034)	2548 (0.033)
soybean	1	1894(0.05716245569909683)	2359 (0.034)	#SAKNAS!
spacers	1	5110(0.011432491139819366)	#SAKNAS!	#SAKNAS!
spadonia	1	5110(0.011432491139819366)	2359 (0.034)	#SAKNAS!
spadonia	1	5110(0.011432491139819366)	2359 (0.034)	#SAKNAS!
span	1	5110(0.011432491139819366)	2359 (0.034)	#SAKNAS!
span	1	5110(0.011432491139819366)	2359 (0.034)	#SAKNAS!
spanish-speaking	1	5110(0.011432491139819366)	#SAKNAS!	2548 (0.033)
sparid	1	5110(0.011432491139819366)	2359 (0.034)	#SAKNAS!
sparid	1	5110(0.011432491139819366)	2359 (0.034)	#SAKNAS!
sparidae	1	5110(0.011432491139819366)	2359 (0.034)	#SAKNAS!
sparidae	1	5110(0.011432491139819366)	2359 (0.034)	#SAKNAS!
spasmodic	1	5110(0.011432491139819366)	#SAKNAS!	2548 (0.033)
spatangoids	1	5110(0.011432491139819366)	#SAKNAS!	#SAKNAS!
specialists	1	5110(0.011432491139819366)	2359 (0.034)	#SAKNAS!
specialists	1	5110(0.011432491139819366)	2359 (0.034)	#SAKNAS!

specialization	1	3437(0.02286498227963873)	2359 (0.034)	2548 (0.033)
species-specific	1	5110(0.011432491139819366)	2359 (0.034)	#SAKNAS!
species-specific	1	5110(0.011432491139819366)	2359 (0.034)	#SAKNAS!
specification	1	5110(0.011432491139819366)	#SAKNAS!	2548 (0.033)
specificity	1	2214(0.04572996455927746)	2359 (0.034)	2548 (0.033)
speckled	1	5110(0.011432491139819366)	#SAKNAS!	2548 (0.033)
spectral	1	5110(0.011432491139819366)	#SAKNAS!	#SAKNAS!
spectroscopy	1	5110(0.011432491139819366)	#SAKNAS!	2548 (0.033)
speech-language	1	1335(0.09145992911855493)	2359 (0.034)	1668 (0.066)
spent	1	5110(0.011432491139819366)	#SAKNAS!	#SAKNAS!
spermatophylax	1	5110(0.011432491139819366)	#SAKNAS!	2548 (0.033)
sphincter	1	1027(0.12575740253801304)	2359 (0.034)	1233 (0.1)
spiders	1	5110(0.011432491139819366)	2359 (0.034)	#SAKNAS!
spiders	1	5110(0.011432491139819366)	2359 (0.034)	#SAKNAS!
spillover	1	5110(0.011432491139819366)	#SAKNAS!	2548 (0.033)
spines	1	5110(0.011432491139819366)	#SAKNAS!	2548 (0.033)
spinocerebellar	1	3437(0.02286498227963873)	2359 (0.034)	#SAKNAS!
spionid	1	5110(0.011432491139819366)	2359 (0.034)	#SAKNAS!
spionid	1	5110(0.011432491139819366)	2359 (0.034)	#SAKNAS!
spiraecolaandtoxoptera	1	5110(0.011432491139819366)	#SAKNAS!	2548 (0.033)
spiritual	1	5110(0.011432491139819366)	#SAKNAS!	#SAKNAS!
spondylitis	1	3437(0.02286498227963873)	2359 (0.034)	2548 (0.033)
spondylotic	1	3437(0.02286498227963873)	2359 (0.034)	#SAKNAS!
sponge	1	5110(0.011432491139819366)	#SAKNAS!	2548 (0.033)
spongiform	1	5110(0.011432491139819366)	2359 (0.034)	#SAKNAS!
spongiform	1	5110(0.011432491139819366)	2359 (0.034)	#SAKNAS!
spoon	1	5110(0.011432491139819366)	#SAKNAS!	#SAKNAS!
spoon-thick	1	5110(0.011432491139819366)	2359 (0.034)	#SAKNAS!
spoon-thick	1	5110(0.011432491139819366)	2359 (0.034)	#SAKNAS!
sport	1	2214(0.04572996455927746)	2359 (0.034)	1233 (0.1)
spot	1	5110(0.011432491139819366)	#SAKNAS!	2548 (0.033)
spots	1	5110(0.011432491139819366)	#SAKNAS!	#SAKNAS!
sprague	1	5110(0.011432491139819366)	#SAKNAS!	#SAKNAS!
spray-drying	1	5110(0.011432491139819366)	#SAKNAS!	#SAKNAS!
spread	1	3437(0.02286498227963873)	2359 (0.034)	2548 (0.033)
sprlm	1	5110(0.011432491139819366)	#SAKNAS!	#SAKNAS!
sprouting	1	5110(0.011432491139819366)	#SAKNAS!	2548 (0.033)
sprue	1	5110(0.011432491139819366)	2359 (0.034)	#SAKNAS!
sprue	1	5110(0.011432491139819366)	2359 (0.034)	#SAKNAS!
spurge	1	5110(0.011432491139819366)	2359 (0.034)	#SAKNAS!
spurge	1	5110(0.011432491139819366)	2359 (0.034)	#SAKNAS!
spurious	1	5110(0.011432491139819366)	2359 (0.034)	#SAKNAS!
spurious	1	5110(0.011432491139819366)	2359 (0.034)	#SAKNAS!

squalius	1	5110(0.011432491139819366)	2359 (0.034)	#SAKNAS!
squalius	1	5110(0.011432491139819366)	2359 (0.034)	#SAKNAS!
squid	1	5110(0.011432491139819366)	#SAKNAS!	#SAKNAS!
squids	1	5110(0.011432491139819366)	2359 (0.034)	#SAKNAS!
squids	1	5110(0.011432491139819366)	2359 (0.034)	#SAKNAS!
squirrel	1	2693(0.0342974734194581)	2359 (0.034)	2548 (0.033)
squirrels	1	3437(0.02286498227963873)	2359 (0.034)	#SAKNAS!
sr	1	5110(0.011432491139819366)	2359 (0.034)	#SAKNAS!
sr	1	5110(0.011432491139819366)	2359 (0.034)	#SAKNAS!
srebp-1	1	5110(0.011432491139819366)	2359 (0.034)	#SAKNAS!
srebp-1	1	5110(0.011432491139819366)	2359 (0.034)	#SAKNAS!
st	1	3437(0.02286498227963873)	2359 (0.034)	2548 (0.033)
st2	1	5110(0.011432491139819366)	#SAKNAS!	2548 (0.033)
stabheuschrecke	1	5110(0.011432491139819366)	2359 (0.034)	#SAKNAS!
stabheuschrecke	1	5110(0.011432491139819366)	2359 (0.034)	#SAKNAS!
stable-isotope	1	5110(0.011432491139819366)	2359 (0.034)	#SAKNAS!
stable-isotope	1	5110(0.011432491139819366)	2359 (0.034)	#SAKNAS!
staffing	1	5110(0.011432491139819366)	#SAKNAS!	2548 (0.033)
stag	1	5110(0.011432491139819366)	#SAKNAS!	2548 (0.033)
stage-iii	1	5110(0.011432491139819366)	2359 (0.034)	#SAKNAS!
stage-iii	1	5110(0.011432491139819366)	2359 (0.034)	#SAKNAS!
staging	1	5110(0.011432491139819366)	2359 (0.034)	#SAKNAS!
staging	1	5110(0.011432491139819366)	2359 (0.034)	#SAKNAS!
stagnalis	1	5110(0.011432491139819366)	#SAKNAS!	#SAKNAS!
stair	1	5110(0.011432491139819366)	#SAKNAS!	#SAKNAS!
stakeholders	1	5110(0.011432491139819366)	#SAKNAS!	#SAKNAS!
stalk	1	5110(0.011432491139819366)	#SAKNAS!	#SAKNAS!
stalls	1	5110(0.011432491139819366)	#SAKNAS!	2548 (0.033)
standalone	1	5110(0.011432491139819366)	#SAKNAS!	#SAKNAS!
stand-alone	1	5110(0.011432491139819366)	#SAKNAS!	#SAKNAS!
standard	1	1335(0.09145992911855493)	2359 (0.034)	999 (0.13)
standing	1	3437(0.02286498227963873)	2359 (0.034)	#SAKNAS!
staple	1	5110(0.011432491139819366)	#SAKNAS!	#SAKNAS!
stapled	1	5110(0.011432491139819366)	2359 (0.034)	#SAKNAS!
stapled	1	5110(0.011432491139819366)	2359 (0.034)	#SAKNAS!
stapler	1	5110(0.011432491139819366)	2359 (0.034)	#SAKNAS!
stapler	1	5110(0.011432491139819366)	2359 (0.034)	#SAKNAS!
starch-based	1	5110(0.011432491139819366)	#SAKNAS!	2548 (0.033)
starch-protein	1	5110(0.011432491139819366)	#SAKNAS!	#SAKNAS!
starch-soybean	1	5110(0.011432491139819366)	#SAKNAS!	#SAKNAS!
starlings	1	5110(0.011432491139819366)	#SAKNAS!	2548 (0.033)
stars	1	5110(0.011432491139819366)	2359 (0.034)	#SAKNAS!
stars	1	5110(0.011432491139819366)	2359 (0.034)	#SAKNAS!

starter	1	5110(0.011432491139819366)	#SAKNAS!	2548 (0.033)
starting	1	5110(0.011432491139819366)	#SAKNAS!	#SAKNAS!
starvation-induced	1	5110(0.011432491139819366)	#SAKNAS!	2548 (0.033)
state-dependent	1	5110(0.011432491139819366)	#SAKNAS!	#SAKNAS!
stated-preference	1	5110(0.011432491139819366)	#SAKNAS!	2548 (0.033)
statement	1	2214(0.04572996455927746)	2359 (0.034)	1233 (0.1)
state-ofthe-art	1	5110(0.011432491139819366)	#SAKNAS!	#SAKNAS!
statewide	1	5110(0.011432491139819366)	#SAKNAS!	2548 (0.033)
state-wide	1	5110(0.011432491139819366)	#SAKNAS!	#SAKNAS!
statin	1	5110(0.011432491139819366)	#SAKNAS!	#SAKNAS!
station	1	5110(0.011432491139819366)	2359 (0.034)	#SAKNAS!
station	1	5110(0.011432491139819366)	2359 (0.034)	#SAKNAS!
station-tested	1	5110(0.011432491139819366)	2359 (0.034)	#SAKNAS!
station-tested	1	5110(0.011432491139819366)	2359 (0.034)	#SAKNAS!
status-by-environment	1	5110(0.011432491139819366)	2359 (0.034)	#SAKNAS!
status-by-environment	1	5110(0.011432491139819366)	2359 (0.034)	#SAKNAS!
stays	1	5110(0.011432491139819366)	#SAKNAS!	#SAKNAS!
steady-state	1	5110(0.011432491139819366)	#SAKNAS!	#SAKNAS!
steatohepatitis-related	1	5110(0.011432491139819366)	#SAKNAS!	2548 (0.033)
steep	1	5110(0.011432491139819366)	#SAKNAS!	2548 (0.033)
steer	1	5110(0.011432491139819366)	#SAKNAS!	#SAKNAS!
stemlage	1	5110(0.011432491139819366)	#SAKNAS!	2548 (0.033)
stenotic	1	5110(0.011432491139819366)	2359 (0.034)	#SAKNAS!
stenotic	1	5110(0.011432491139819366)	2359 (0.034)	#SAKNAS!
step	1	5110(0.011432491139819366)	2359 (0.034)	#SAKNAS!
step	1	5110(0.011432491139819366)	2359 (0.034)	#SAKNAS!
stephanolepis	1	5110(0.011432491139819366)	#SAKNAS!	#SAKNAS!
stepheni	1	5110(0.011432491139819366)	2359 (0.034)	#SAKNAS!
stepheni	1	5110(0.011432491139819366)	2359 (0.034)	#SAKNAS!
stephensi	1	5110(0.011432491139819366)	2359 (0.034)	#SAKNAS!
stephensi	1	5110(0.011432491139819366)	2359 (0.034)	#SAKNAS!
stephensimidgut	1	5110(0.011432491139819366)	#SAKNAS!	2548 (0.033)
steppe	1	5110(0.011432491139819366)	#SAKNAS!	2548 (0.033)
steppe-vegetation	1	5110(0.011432491139819366)	#SAKNAS!	#SAKNAS!
steps	1	5110(0.011432491139819366)	#SAKNAS!	2548 (0.033)
stepwise	1	2693(0.0342974734194581)	2359 (0.034)	#SAKNAS!
stereognostic	1	5110(0.011432491139819366)	#SAKNAS!	2548 (0.033)
sterilisation	1	5110(0.011432491139819366)	#SAKNAS!	#SAKNAS!
steroid	1	5110(0.011432491139819366)	#SAKNAS!	2548 (0.033)
sterol	1	5110(0.011432491139819366)	2359 (0.034)	#SAKNAS!
sterol	1	5110(0.011432491139819366)	2359 (0.034)	#SAKNAS!
stevens-johnson	1	5110(0.011432491139819366)	2359 (0.034)	#SAKNAS!
stevens-johnson	1	5110(0.011432491139819366)	2359 (0.034)	#SAKNAS!

stevia	1	5110(0.011432491139819366)	#SAKNAS!	2548 (0.033)
stickleback	1	5110(0.011432491139819366)	2359 (0.034)	#SAKNAS!
stickleback	1	5110(0.011432491139819366)	2359 (0.034)	#SAKNAS!
sticklebacks	1	5110(0.011432491139819366)	2359 (0.034)	#SAKNAS!
sticklebacks	1	5110(0.011432491139819366)	2359 (0.034)	#SAKNAS!
still	1	2693(0.0342974734194581)	2359 (0.034)	#SAKNAS!
stim	1	5110(0.011432491139819366)	#SAKNAS!	#SAKNAS!
stimulants	1	5110(0.011432491139819366)	2359 (0.034)	#SAKNAS!
stimulants	1	5110(0.011432491139819366)	2359 (0.034)	#SAKNAS!
stimulate	1	5110(0.011432491139819366)	2359 (0.034)	#SAKNAS!
stimulate	1	5110(0.011432491139819366)	2359 (0.034)	#SAKNAS!
stimulates	1	3437(0.02286498227963873)	2359 (0.034)	2548 (0.033)
stimulating	1	2693(0.0342974734194581)	2359 (0.034)	#SAKNAS!
stimuli	1	2693(0.0342974734194581)	2359 (0.034)	1668 (0.066)
stimulus	1	5110(0.011432491139819366)	#SAKNAS!	2548 (0.033)
stochastic	1	5110(0.011432491139819366)	#SAKNAS!	2548 (0.033)
stoichiometric	1	5110(0.011432491139819366)	#SAKNAS!	2548 (0.033)
stomach-preserving	1	5110(0.011432491139819366)	#SAKNAS!	#SAKNAS!
stomachs	1	5110(0.011432491139819366)	2359 (0.034)	#SAKNAS!
stomachs	1	5110(0.011432491139819366)	2359 (0.034)	#SAKNAS!
stop	1	5110(0.011432491139819366)	#SAKNAS!	2548 (0.033)
stop-bang	1	5110(0.011432491139819366)	#SAKNAS!	#SAKNAS!
stopping	1	5110(0.011432491139819366)	#SAKNAS!	2548 (0.033)
stops	1	5110(0.011432491139819366)	2359 (0.034)	#SAKNAS!
stops	1	5110(0.011432491139819366)	2359 (0.034)	#SAKNAS!
storage	1	2214(0.04572996455927746)	2359 (0.034)	2548 (0.033)
stores	1	5110(0.011432491139819366)	2359 (0.034)	#SAKNAS!
stores	1	5110(0.011432491139819366)	2359 (0.034)	#SAKNAS!
storm-induced	1	5110(0.011432491139819366)	2359 (0.034)	#SAKNAS!
storm-induced	1	5110(0.011432491139819366)	2359 (0.034)	#SAKNAS!
storm-petrel	1	5110(0.011432491139819366)	2359 (0.034)	#SAKNAS!
storm-petrel	1	5110(0.011432491139819366)	2359 (0.034)	#SAKNAS!
story	1	5110(0.011432491139819366)	2359 (0.034)	#SAKNAS!
story	1	5110(0.011432491139819366)	2359 (0.034)	#SAKNAS!
strain	1	2693(0.0342974734194581)	2359 (0.034)	#SAKNAS!
strait	1	5110(0.011432491139819366)	#SAKNAS!	#SAKNAS!
strategic	1	5110(0.011432491139819366)	#SAKNAS!	2548 (0.033)
streaked	1	5110(0.011432491139819366)	#SAKNAS!	2548 (0.033)
stream	1	2693(0.0342974734194581)	2359 (0.034)	2548 (0.033)
street-recruited	1	5110(0.011432491139819366)	2359 (0.034)	#SAKNAS!
street-recruited	1	5110(0.011432491139819366)	2359 (0.034)	#SAKNAS!
strength	1	427(0.3201097519149423)	2359 (0.034)	305 (0.46)
stress-eating	1	5110(0.011432491139819366)	#SAKNAS!	2548 (0.033)

stress-sensitive	1	5110(0.011432491139819366)	#SAKNAS!	#SAKNAS!
striatal	1	5110(0.011432491139819366)	#SAKNAS!	#SAKNAS!
stricturotomy	1	5110(0.011432491139819366)	#SAKNAS!	2548 (0.033)
stridor	1	5110(0.011432491139819366)	#SAKNAS!	#SAKNAS!
stripping	1	5110(0.011432491139819366)	2359 (0.034)	#SAKNAS!
stripping	1	5110(0.011432491139819366)	2359 (0.034)	#SAKNAS!
stroke-associated	1	1894(0.05716245569909683)	2359 (0.034)	#SAKNAS!
stronger	1	5110(0.011432491139819366)	#SAKNAS!	2548 (0.033)
strongylocentrotus	1	5110(0.011432491139819366)	2359 (0.034)	#SAKNAS!
strongylocentrotus	1	5110(0.011432491139819366)	2359 (0.034)	#SAKNAS!
structure-related	1	5110(0.011432491139819366)	#SAKNAS!	2548 (0.033)
structures	1	1894(0.05716245569909683)	2359 (0.034)	1233 (0.1)
struggling	1	5110(0.011432491139819366)	#SAKNAS!	2548 (0.033)
strykeri	1	5110(0.011432491139819366)	#SAKNAS!	2548 (0.033)
stubble	1	5110(0.011432491139819366)	#SAKNAS!	#SAKNAS!
sturgeon	1	5110(0.011432491139819366)	2359 (0.034)	#SAKNAS!
sturgeon	1	5110(0.011432491139819366)	2359 (0.034)	#SAKNAS!
stylohyoid	1	5110(0.011432491139819366)	#SAKNAS!	2548 (0.033)
stylophora	1	5110(0.011432491139819366)	2359 (0.034)	#SAKNAS!
stylophora	1	5110(0.011432491139819366)	2359 (0.034)	#SAKNAS!
suavissimus	1	5110(0.011432491139819366)	#SAKNAS!	#SAKNAS!
subalpine	1	5110(0.011432491139819366)	#SAKNAS!	2548 (0.033)
subanalysis	1	5110(0.011432491139819366)	#SAKNAS!	2548 (0.033)
subarachnoid	1	3437(0.022864982279638733)	2359 (0.034)	#SAKNAS!
subcastes	1	5110(0.011432491139819366)	2359 (0.034)	#SAKNAS!
subcastes	1	5110(0.011432491139819366)	2359 (0.034)	#SAKNAS!
subchronic	1	5110(0.011432491139819366)	2359 (0.034)	#SAKNAS!
subchronic	1	5110(0.011432491139819366)	2359 (0.034)	#SAKNAS!
subclavian	1	5110(0.011432491139819366)	#SAKNAS!	#SAKNAS!
subclinical	1	2214(0.04572996455927746)	2359 (0.034)	1668 (0.066)
subcortical	1	5110(0.011432491139819366)	#SAKNAS!	2548 (0.033)
sub-county	1	5110(0.011432491139819366)	#SAKNAS!	#SAKNAS!
subcutaneous	1	5110(0.011432491139819366)	2359 (0.034)	#SAKNAS!
subcutaneous	1	5110(0.011432491139819366)	2359 (0.034)	#SAKNAS!
subdiaphragmatic	1	5110(0.011432491139819366)	2359 (0.034)	#SAKNAS!
subdiaphragmatic	1	5110(0.011432491139819366)	2359 (0.034)	#SAKNAS!
subdistricts	1	5110(0.011432491139819366)	2359 (0.034)	#SAKNAS!
subdistricts	1	5110(0.011432491139819366)	2359 (0.034)	#SAKNAS!
subepithelial	1	5110(0.011432491139819366)	2359 (0.034)	#SAKNAS!
subepithelial	1	5110(0.011432491139819366)	2359 (0.034)	#SAKNAS!
subject	1	5110(0.011432491139819366)	#SAKNAS!	2548 (0.033)
subjectively	1	5110(0.011432491139819366)	#SAKNAS!	2548 (0.033)
sub-lethal	1	5110(0.011432491139819366)	#SAKNAS!	2548 (0.033)

sublingual	1	5110(0.011432491139819366)	#SAKNAS!	#SAKNAS!
submucosa	1	5110(0.011432491139819366)	2359 (0.034)	#SAKNAS!
submucosa	1	5110(0.011432491139819366)	2359 (0.034)	#SAKNAS!
submucosal	1	3437(0.02286498227963873)	2359 (0.034)	#SAKNAS!
submucous	1	5110(0.011432491139819366)	2359 (0.034)	#SAKNAS!
submucous	1	5110(0.011432491139819366)	2359 (0.034)	#SAKNAS!
sub-national	1	5110(0.011432491139819366)	#SAKNAS!	#SAKNAS!
subpolar	1	5110(0.011432491139819366)	2359 (0.034)	#SAKNAS!
subpolar	1	5110(0.011432491139819366)	2359 (0.034)	#SAKNAS!
subpopulations	1	5110(0.011432491139819366)	2359 (0.034)	#SAKNAS!
subpopulations	1	5110(0.011432491139819366)	2359 (0.034)	#SAKNAS!
sub-region	1	5110(0.011432491139819366)	#SAKNAS!	2548 (0.033)
sub-saharan	1	5110(0.011432491139819366)	2359 (0.034)	#SAKNAS!
sub-saharan	1	5110(0.011432491139819366)	2359 (0.034)	#SAKNAS!
subset	1	5110(0.011432491139819366)	2359 (0.034)	#SAKNAS!
subset	1	5110(0.011432491139819366)	2359 (0.034)	#SAKNAS!
subsidy	1	5110(0.011432491139819366)	#SAKNAS!	2548 (0.033)
subsistence	1	5110(0.011432491139819366)	#SAKNAS!	2548 (0.033)
subsocial	1	5110(0.011432491139819366)	#SAKNAS!	2548 (0.033)
substance	1	3437(0.02286498227963873)	2359 (0.034)	2548 (0.033)
substances	1	2214(0.04572996455927746)	2359 (0.034)	2548 (0.033)
substituting	1	2693(0.0342974734194581)	2359 (0.034)	2548 (0.033)
subterranean	1	5110(0.011432491139819366)	2359 (0.034)	#SAKNAS!
subterranean	1	5110(0.011432491139819366)	2359 (0.034)	#SAKNAS!
subthalamic	1	5110(0.011432491139819366)	#SAKNAS!	#SAKNAS!
subtypes	1	2214(0.04572996455927746)	2359 (0.034)	#SAKNAS!
subtyping	1	5110(0.011432491139819366)	#SAKNAS!	2548 (0.033)
suburban	1	3437(0.02286498227963873)	2359 (0.034)	2548 (0.033)
successive	1	5110(0.011432491139819366)	#SAKNAS!	#SAKNAS!
succinylated	1	5110(0.011432491139819366)	#SAKNAS!	#SAKNAS!
suckling	1	5110(0.011432491139819366)	#SAKNAS!	2548 (0.033)
sucralfate	1	5110(0.011432491139819366)	2359 (0.034)	#SAKNAS!
sucralfate	1	5110(0.011432491139819366)	2359 (0.034)	#SAKNAS!
suction	1	5110(0.011432491139819366)	#SAKNAS!	#SAKNAS!
suction-swallowing	1	5110(0.011432491139819366)	#SAKNAS!	2548 (0.033)
sudden	1	5110(0.011432491139819366)	2359 (0.034)	#SAKNAS!
sudden	1	5110(0.011432491139819366)	2359 (0.034)	#SAKNAS!
sufeetube	1	5110(0.011432491139819366)	#SAKNAS!	#SAKNAS!
sufficient	1	5110(0.011432491139819366)	#SAKNAS!	#SAKNAS!
sugarcane	1	5110(0.011432491139819366)	#SAKNAS!	2548 (0.033)
sugarcane-based	1	5110(0.011432491139819366)	#SAKNAS!	2548 (0.033)
sugar-containing	1	5110(0.011432491139819366)	2359 (0.034)	#SAKNAS!
sugar-containing	1	5110(0.011432491139819366)	2359 (0.034)	#SAKNAS!

sugared	1	5110(0.011432491139819366)	2359 (0.034)	#SAKNAS!
sugared	1	5110(0.011432491139819366)	2359 (0.034)	#SAKNAS!
sugar-related	1	5110(0.011432491139819366)	#SAKNAS!	2548 (0.033)
sugar-rich	1	5110(0.011432491139819366)	#SAKNAS!	2548 (0.033)
sugar-sweetened	1	743(0.18291985823710985)	2359 (0.034)	526 (0.26)
suicidal	1	5110(0.011432491139819366)	#SAKNAS!	2548 (0.033)
suita	1	5110(0.011432491139819366)	#SAKNAS!	#SAKNAS!
sul	1	5110(0.011432491139819366)	2359 (0.034)	#SAKNAS!
sul	1	5110(0.011432491139819366)	2359 (0.034)	#SAKNAS!
sulawesi	1	5110(0.011432491139819366)	#SAKNAS!	2548 (0.033)
sulcatus	1	5110(0.011432491139819366)	2359 (0.034)	#SAKNAS!
sulcatus	1	5110(0.011432491139819366)	2359 (0.034)	#SAKNAS!
sulfate	1	5110(0.011432491139819366)	2359 (0.034)	#SAKNAS!
sulfate	1	5110(0.011432491139819366)	2359 (0.034)	#SAKNAS!
sulforaphene	1	5110(0.011432491139819366)	#SAKNAS!	#SAKNAS!
sulphur	1	5110(0.011432491139819366)	2359 (0.034)	#SAKNAS!
sulphur	1	5110(0.011432491139819366)	2359 (0.034)	#SAKNAS!
sumatra	1	5110(0.011432491139819366)	#SAKNAS!	#SAKNAS!
summer	1	2214(0.04572996455927746)	2359 (0.034)	#SAKNAS!
summit	1	5110(0.011432491139819366)	#SAKNAS!	2548 (0.033)
sunflower	1	5110(0.011432491139819366)	2359 (0.034)	#SAKNAS!
sunflower	1	5110(0.011432491139819366)	2359 (0.034)	#SAKNAS!
super-conditioning	1	5110(0.011432491139819366)	#SAKNAS!	#SAKNAS!
superficial	1	5110(0.011432491139819366)	#SAKNAS!	2548 (0.033)
super-intensive	1	5110(0.011432491139819366)	2359 (0.034)	#SAKNAS!
super-intensive	1	5110(0.011432491139819366)	2359 (0.034)	#SAKNAS!
superior	1	5110(0.011432491139819366)	#SAKNAS!	2548 (0.033)
superiority	1	5110(0.011432491139819366)	#SAKNAS!	#SAKNAS!
superoxide	1	5110(0.011432491139819366)	#SAKNAS!	#SAKNAS!
superselective	1	3437(0.02286498227963873)	2359 (0.034)	2548 (0.033)
supervise	1	5110(0.011432491139819366)	2359 (0.034)	#SAKNAS!
supervise	1	5110(0.011432491139819366)	2359 (0.034)	#SAKNAS!
supervision	1	5110(0.011432491139819366)	#SAKNAS!	2548 (0.033)
supplementary	1	1485(0.08002743797873557)	2359 (0.034)	999 (0.13)
suppresses	1	1335(0.09145992911855493)	2359 (0.034)	816 (0.16)
suppurativa	1	5110(0.011432491139819366)	#SAKNAS!	#SAKNAS!
suprachiasmatic	1	5110(0.011432491139819366)	#SAKNAS!	#SAKNAS!
supraglottic	1	3437(0.02286498227963873)	2359 (0.034)	#SAKNAS!
supraglottoplasty	1	5110(0.011432491139819366)	2359 (0.034)	#SAKNAS!
supraglottoplasty	1	5110(0.011432491139819366)	2359 (0.034)	#SAKNAS!
supranormal	1	5110(0.011432491139819366)	#SAKNAS!	#SAKNAS!
supratentorial	1	5110(0.011432491139819366)	#SAKNAS!	2548 (0.033)
supreme	1	5110(0.011432491139819366)	2359 (0.034)	#SAKNAS!

supreme	1 5110(0.011432491139819366)	2359 (0.034)	#SAKNAS!
surfaces	1 2693(0.0342974734194581)	2359 (0.034)	2548 (0.033)
surgeon	1 5110(0.011432491139819366)	#SAKNAS!	#SAKNAS!
surgery-does	1 5110(0.011432491139819366)	#SAKNAS!	#SAKNAS!
surgery-related	1 5110(0.011432491139819366)	#SAKNAS!	2548 (0.033)
surgically	1 3437(0.02286498227963873)	2359 (0.034)	2548 (0.033)
surgical-management	1 5110(0.011432491139819366)	2359 (0.034)	#SAKNAS!
surgical-management	1 5110(0.011432491139819366)	2359 (0.034)	#SAKNAS!
surimi	1 5110(0.011432491139819366)	#SAKNAS!	#SAKNAS!
surrogate	1 5110(0.011432491139819366)	#SAKNAS!	#SAKNAS!
surrounding	1 5110(0.011432491139819366)	2359 (0.034)	#SAKNAS!
surrounding	1 5110(0.011432491139819366)	2359 (0.034)	#SAKNAS!
surveying	1 5110(0.011432491139819366)	#SAKNAS!	#SAKNAS!
survey-wide	1 5110(0.011432491139819366)	#SAKNAS!	#SAKNAS!
survival-determining	1 5110(0.011432491139819366)	2359 (0.034)	#SAKNAS!
survival-determining	1 5110(0.011432491139819366)	2359 (0.034)	#SAKNAS!
survivals	1 5110(0.011432491139819366)	#SAKNAS!	#SAKNAS!
surviving	1 3437(0.02286498227963873)	2359 (0.034)	2548 (0.033)
survivors	1 439(0.30867726077512286)	2359 (0.034)	251 (0.56)
sus	1 5110(0.011432491139819366)	#SAKNAS!	#SAKNAS!
suspension-feeding	1 3437(0.02286498227963873)	2359 (0.034)	2548 (0.033)
sustain	1 5110(0.011432491139819366)	#SAKNAS!	#SAKNAS!
sw	1 5110(0.011432491139819366)	#SAKNAS!	#SAKNAS!
swall	1 5110(0.011432491139819366)	#SAKNAS!	#SAKNAS!
swallowing-breathing	1 5110(0.011432491139819366)	#SAKNAS!	2548 (0.033)
swallowing-feeding	1 5110(0.011432491139819366)	#SAKNAS!	#SAKNAS!
swallowing-quality	1 5110(0.011432491139819366)	2359 (0.034)	#SAKNAS!
swallowing-quality	1 5110(0.011432491139819366)	2359 (0.034)	#SAKNAS!
swallowing-the	1 5110(0.011432491139819366)	#SAKNAS!	2548 (0.033)
swallow-related	1 5110(0.011432491139819366)	#SAKNAS!	2548 (0.033)
swallows	1 5110(0.011432491139819366)	#SAKNAS!	#SAKNAS!
sweating	1 5110(0.011432491139819366)	#SAKNAS!	#SAKNAS!
swedes	1 5110(0.011432491139819366)	2359 (0.034)	#SAKNAS!
swedes	1 5110(0.011432491139819366)	2359 (0.034)	#SAKNAS!
sweetener	1 5110(0.011432491139819366)	#SAKNAS!	2548 (0.033)
sweetness	1 5110(0.011432491139819366)	2359 (0.034)	#SAKNAS!
sweetness	1 5110(0.011432491139819366)	2359 (0.034)	#SAKNAS!
sweet-potato	1 5110(0.011432491139819366)	2359 (0.034)	#SAKNAS!
sweet-potato	1 5110(0.011432491139819366)	2359 (0.034)	#SAKNAS!
sweets	1 3437(0.02286498227963873)	2359 (0.034)	2548 (0.033)
swelling	1 2693(0.0342974734194581)	2359 (0.034)	2548 (0.033)
swimming	1 5110(0.011432491139819366)	#SAKNAS!	2548 (0.033)
swine	1 3437(0.02286498227963873)	2359 (0.034)	#SAKNAS!

swines	1	5110(0.011432491139819366)	2359 (0.034)	#SAKNAS!
swines	1	5110(0.011432491139819366)	2359 (0.034)	#SAKNAS!
swiss	1	2214(0.04572996455927746)	2359 (0.034)	2548 (0.033)
switching	1	2693(0.0342974734194581)	2359 (0.034)	#SAKNAS!
switzerland	1	5110(0.011432491139819366)	#SAKNAS!	2548 (0.033)
swordfish	1	5110(0.011432491139819366)	#SAKNAS!	#SAKNAS!
syagrus	1	5110(0.011432491139819366)	#SAKNAS!	#SAKNAS!
sydney	1	2693(0.0342974734194581)	2359 (0.034)	#SAKNAS!
sylvia	1	5110(0.011432491139819366)	2359 (0.034)	#SAKNAS!
sylvia	1	5110(0.011432491139819366)	2359 (0.034)	#SAKNAS!
symbionts	1	3437(0.02286498227963873)	2359 (0.034)	2548 (0.033)
symbiosis-independent	1	5110(0.011432491139819366)	#SAKNAS!	#SAKNAS!
symbiotic	1	5110(0.011432491139819366)	#SAKNAS!	2548 (0.033)
symmetrized	1	5110(0.011432491139819366)	2359 (0.034)	#SAKNAS!
symmetrized	1	5110(0.011432491139819366)	2359 (0.034)	#SAKNAS!
symposium	1	5110(0.011432491139819366)	#SAKNAS!	#SAKNAS!
symptomatic	1	2214(0.04572996455927746)	2359 (0.034)	1668 (0.066)
symptomatology	1	5110(0.011432491139819366)	#SAKNAS!	#SAKNAS!
synapses	1	5110(0.011432491139819366)	#SAKNAS!	#SAKNAS!
synaptic	1	3437(0.02286498227963873)	2359 (0.034)	2548 (0.033)
synbiotic	1	5110(0.011432491139819366)	2359 (0.034)	#SAKNAS!
synbiotic	1	5110(0.011432491139819366)	2359 (0.034)	#SAKNAS!
synchronizes	1	5110(0.011432491139819366)	#SAKNAS!	2548 (0.033)
synchronous	1	3437(0.02286498227963873)	2359 (0.034)	2548 (0.033)
synchrony	1	5110(0.011432491139819366)	2359 (0.034)	#SAKNAS!
synchrony	1	5110(0.011432491139819366)	2359 (0.034)	#SAKNAS!
syndecan-3	1	5110(0.011432491139819366)	2359 (0.034)	#SAKNAS!
syndecan-3	1	5110(0.011432491139819366)	2359 (0.034)	#SAKNAS!
syndromes	1	1485(0.08002743797873557)	2359 (0.034)	1668 (0.066)
synergism	1	5110(0.011432491139819366)	2359 (0.034)	#SAKNAS!
synergism	1	5110(0.011432491139819366)	2359 (0.034)	#SAKNAS!
synopsis	1	5110(0.011432491139819366)	2359 (0.034)	#SAKNAS!
synopsis	1	5110(0.011432491139819366)	2359 (0.034)	#SAKNAS!
synthesis	1	5110(0.011432491139819366)	#SAKNAS!	2548 (0.033)
synthetic	1	3437(0.02286498227963873)	2359 (0.034)	2548 (0.033)
syntrophic	1	5110(0.011432491139819366)	#SAKNAS!	#SAKNAS!
systematics	1	5110(0.011432491139819366)	#SAKNAS!	2548 (0.033)
systemically	1	5110(0.011432491139819366)	#SAKNAS!	#SAKNAS!
systolic	1	5110(0.011432491139819366)	#SAKNAS!	2548 (0.033)
t1n0m0	1	5110(0.011432491139819366)	#SAKNAS!	2548 (0.033)
t-2	1	5110(0.011432491139819366)	#SAKNAS!	#SAKNAS!
t2*-weighted	1	5110(0.011432491139819366)	#SAKNAS!	2548 (0.033)
t2dm	1	5110(0.011432491139819366)	#SAKNAS!	#SAKNAS!

tabaci	1	2693(0.0342974734194581)	2359 (0.034)	1668 (0.066)
tabacimeam1	1	5110(0.011432491139819366)	#SAKNAS!	2548 (0.033)
tablet	1	5110(0.011432491139819366)	#SAKNAS!	#SAKNAS!
tachypleus-gigas	1	5110(0.011432491139819366)	2359 (0.034)	#SAKNAS!
tachypleus-gigas	1	5110(0.011432491139819366)	2359 (0.034)	#SAKNAS!
tackle	1	5110(0.011432491139819366)	#SAKNAS!	2548 (0.033)
tacr1	1	5110(0.011432491139819366)	#SAKNAS!	#SAKNAS!
tactics	1	5110(0.011432491139819366)	#SAKNAS!	2548 (0.033)
tadpole	1	5110(0.011432491139819366)	#SAKNAS!	2548 (0.033)
tadpoles	1	5110(0.011432491139819366)	2359 (0.034)	#SAKNAS!
tadpoles	1	5110(0.011432491139819366)	2359 (0.034)	#SAKNAS!
taichung	1	5110(0.011432491139819366)	#SAKNAS!	#SAKNAS!
tail	1	5110(0.011432491139819366)	2359 (0.034)	#SAKNAS!
tail	1	5110(0.011432491139819366)	2359 (0.034)	#SAKNAS!
taipei	1	5110(0.011432491139819366)	#SAKNAS!	#SAKNAS!
taiwanese	1	1678(0.0685949468389162)	2359 (0.034)	1233 (0.1)
takahashi	1	5110(0.011432491139819366)	#SAKNAS!	#SAKNAS!
takahata	1	5110(0.011432491139819366)	#SAKNAS!	2548 (0.033)
takahe	1	5110(0.011432491139819366)	2359 (0.034)	#SAKNAS!
takahe	1	5110(0.011432491139819366)	2359 (0.034)	#SAKNAS!
takeout	1	5110(0.011432491139819366)	2359 (0.034)	#SAKNAS!
takeout	1	5110(0.011432491139819366)	2359 (0.034)	#SAKNAS!
talarum	1	5110(0.011432491139819366)	#SAKNAS!	2548 (0.033)
tall	1	5110(0.011432491139819366)	2359 (0.034)	#SAKNAS!
tall	1	5110(0.011432491139819366)	2359 (0.034)	#SAKNAS!
tallgrass	1	5110(0.011432491139819366)	#SAKNAS!	2548 (0.033)
tamarins	1	2693(0.0342974734194581)	2359 (0.034)	#SAKNAS!
tamaulipas	1	5110(0.011432491139819366)	#SAKNAS!	#SAKNAS!
tammar	1	5110(0.011432491139819366)	2359 (0.034)	#SAKNAS!
tammar	1	5110(0.011432491139819366)	2359 (0.034)	#SAKNAS!
tanacetum	1	5110(0.011432491139819366)	2359 (0.034)	#SAKNAS!
tanacetum	1	5110(0.011432491139819366)	2359 (0.034)	#SAKNAS!
tannic	1	5110(0.011432491139819366)	#SAKNAS!	#SAKNAS!
tannin	1	3437(0.02286498227963873)	2359 (0.034)	2548 (0.033)
tannin-rich	1	3437(0.02286498227963873)	2359 (0.034)	2548 (0.033)
tanycytes	1	5110(0.011432491139819366)	#SAKNAS!	#SAKNAS!
tanzanians	1	5110(0.011432491139819366)	2359 (0.034)	#SAKNAS!
tanzanians	1	5110(0.011432491139819366)	2359 (0.034)	#SAKNAS!
taotie	1	5110(0.011432491139819366)	#SAKNAS!	2548 (0.033)
tapeworm	1	5110(0.011432491139819366)	2359 (0.034)	#SAKNAS!
tapeworm	1	5110(0.011432491139819366)	2359 (0.034)	#SAKNAS!
taping	1	5110(0.011432491139819366)	#SAKNAS!	#SAKNAS!
tardigrade	1	5110(0.011432491139819366)	#SAKNAS!	#SAKNAS!

tardigrades	1	5110(0.011432491139819366)	#SAKNAS!	#SAKNAS!
tardive	1	5110(0.011432491139819366)	#SAKNAS!	2548 (0.033)
tarf	1	5110(0.011432491139819366)	#SAKNAS!	#SAKNAS!
target-controlled	1	5110(0.011432491139819366)	#SAKNAS!	2548 (0.033)
targeting	1	1894(0.05716245569909683)	2359 (0.034)	1668 (0.066)
targets	1	5110(0.011432491139819366)	#SAKNAS!	2548 (0.033)
tas2r1	1	5110(0.011432491139819366)	#SAKNAS!	2548 (0.033)
tas2r4	1	5110(0.011432491139819366)	#SAKNAS!	2548 (0.033)
tasar	1	5110(0.011432491139819366)	2359 (0.034)	#SAKNAS!
tasar	1	5110(0.011432491139819366)	2359 (0.034)	#SAKNAS!
tasks	1	5110(0.011432491139819366)	#SAKNAS!	#SAKNAS!
tastants	1	5110(0.011432491139819366)	#SAKNAS!	2548 (0.033)
taste-independent	1	5110(0.011432491139819366)	2359 (0.034)	#SAKNAS!
taste-independent	1	5110(0.011432491139819366)	2359 (0.034)	#SAKNAS!
tastes	1	2214(0.04572996455927746)	2359 (0.034)	1668 (0.066)
tasting	1	5110(0.011432491139819366)	2359 (0.034)	#SAKNAS!
tasting	1	5110(0.011432491139819366)	2359 (0.034)	#SAKNAS!
taumako	1	5110(0.011432491139819366)	#SAKNAS!	#SAKNAS!
taurine	1	3437(0.02286498227963873)	2359 (0.034)	2548 (0.033)
tax	1	5110(0.011432491139819366)	2359 (0.034)	#SAKNAS!
tax	1	5110(0.011432491139819366)	2359 (0.034)	#SAKNAS!
taxation	1	5110(0.011432491139819366)	#SAKNAS!	2548 (0.033)
tbc1d1	1	5110(0.011432491139819366)	#SAKNAS!	2548 (0.033)
tbi	1	5110(0.011432491139819366)	2359 (0.034)	#SAKNAS!
tbi	1	5110(0.011432491139819366)	2359 (0.034)	#SAKNAS!
tcbi	1	5110(0.011432491139819366)	#SAKNAS!	#SAKNAS!
tcdd	1	5110(0.011432491139819366)	2359 (0.034)	#SAKNAS!
tcdd	1	5110(0.011432491139819366)	2359 (0.034)	#SAKNAS!
t-cell	1	3437(0.02286498227963873)	2359 (0.034)	#SAKNAS!
tdp-43	1	5110(0.011432491139819366)	#SAKNAS!	2548 (0.033)
teachers	1	5110(0.011432491139819366)	#SAKNAS!	2548 (0.033)
teams	1	3437(0.02286498227963873)	2359 (0.034)	#SAKNAS!
technology-enabled	1	5110(0.011432491139819366)	#SAKNAS!	2548 (0.033)
tegula	1	5110(0.011432491139819366)	#SAKNAS!	#SAKNAS!
tegumentary	1	5110(0.011432491139819366)	2359 (0.034)	#SAKNAS!
tegumentary	1	5110(0.011432491139819366)	2359 (0.034)	#SAKNAS!
tehran	1	5110(0.011432491139819366)	2359 (0.034)	#SAKNAS!
tehran	1	5110(0.011432491139819366)	2359 (0.034)	#SAKNAS!
telangiectasia	1	3437(0.02286498227963873)	2359 (0.034)	2548 (0.033)
telecommuting	1	5110(0.011432491139819366)	#SAKNAS!	#SAKNAS!
telemetry	1	5110(0.011432491139819366)	#SAKNAS!	#SAKNAS!
teleoste	1	5110(0.011432491139819366)	2359 (0.034)	#SAKNAS!
teleoste	1	5110(0.011432491139819366)	2359 (0.034)	#SAKNAS!

telerehabilitation	1	5110(0.011432491139819366)	#SAKNAS!	#SAKNAS!
tell	1	5110(0.011432491139819366)	#SAKNAS!	2548 (0.033)
telomere	1	5110(0.011432491139819366)	#SAKNAS!	#SAKNAS!
temminck	1	5110(0.011432491139819366)	#SAKNAS!	#SAKNAS!
temperature-dependent	1	5110(0.011432491139819366)	#SAKNAS!	#SAKNAS!
temperature-regulated	1	5110(0.011432491139819366)	#SAKNAS!	#SAKNAS!
template	1	5110(0.011432491139819366)	#SAKNAS!	#SAKNAS!
tempo	1	5110(0.011432491139819366)	2359 (0.034)	#SAKNAS!
tempo	1	5110(0.011432491139819366)	2359 (0.034)	#SAKNAS!
temporalis	1	5110(0.011432491139819366)	#SAKNAS!	#SAKNAS!
temporally	1	5110(0.011432491139819366)	#SAKNAS!	#SAKNAS!
temporary	1	2214(0.04572996455927746)	2359 (0.034)	1668 (0.066)
tems	1	5110(0.011432491139819366)	#SAKNAS!	2548 (0.033)
ten	1	2693(0.0342974734194581)	2359 (0.034)	#SAKNAS!
tenebrio-molitor	1	5110(0.011432491139819366)	2359 (0.034)	#SAKNAS!
tenebrio-molitor	1	5110(0.011432491139819366)	2359 (0.034)	#SAKNAS!
tenebrionidae	1	5110(0.011432491139819366)	#SAKNAS!	2548 (0.033)
tenerife	1	5110(0.011432491139819366)	2359 (0.034)	#SAKNAS!
tenerife	1	5110(0.011432491139819366)	2359 (0.034)	#SAKNAS!
tennessee	1	5110(0.011432491139819366)	2359 (0.034)	#SAKNAS!
tennessee	1	5110(0.011432491139819366)	2359 (0.034)	#SAKNAS!
tensor	1	5110(0.011432491139819366)	#SAKNAS!	#SAKNAS!
tentative	1	5110(0.011432491139819366)	#SAKNAS!	2548 (0.033)
tenth	1	5110(0.011432491139819366)	#SAKNAS!	2548 (0.033)
ten-year	1	5110(0.011432491139819366)	#SAKNAS!	#SAKNAS!
tephritid	1	5110(0.011432491139819366)	#SAKNAS!	#SAKNAS!
teratoma	1	5110(0.011432491139819366)	#SAKNAS!	2548 (0.033)
terebinthifolius	1	5110(0.011432491139819366)	2359 (0.034)	#SAKNAS!
terebinthifolius	1	5110(0.011432491139819366)	2359 (0.034)	#SAKNAS!
term-equivalent	1	5110(0.011432491139819366)	#SAKNAS!	#SAKNAS!
terminal	1	2693(0.0342974734194581)	2359 (0.034)	2548 (0.033)
terminalia	1	5110(0.011432491139819366)	2359 (0.034)	#SAKNAS!
terminalia	1	5110(0.011432491139819366)	2359 (0.034)	#SAKNAS!
terminally	1	5110(0.011432491139819366)	2359 (0.034)	#SAKNAS!
terminally	1	5110(0.011432491139819366)	2359 (0.034)	#SAKNAS!
termite	1	5110(0.011432491139819366)	2359 (0.034)	#SAKNAS!
termite	1	5110(0.011432491139819366)	2359 (0.034)	#SAKNAS!
termites	1	5110(0.011432491139819366)	#SAKNAS!	#SAKNAS!
termitoidae	1	5110(0.011432491139819366)	#SAKNAS!	#SAKNAS!
terms	1	2214(0.04572996455927746)	2359 (0.034)	2548 (0.033)
terrestrial	1	2693(0.0342974734194581)	2359 (0.034)	1668 (0.066)
terrestris	1	2214(0.04572996455927746)	2359 (0.034)	#SAKNAS!
territory	1	5110(0.011432491139819366)	2359 (0.034)	#SAKNAS!

territory	1	5110(0.011432491139819366)	2359 (0.034)	#SAKNAS!
tert	1	5110(0.011432491139819366)	2359 (0.034)	#SAKNAS!
tert	1	5110(0.011432491139819366)	2359 (0.034)	#SAKNAS!
teso	1	5110(0.011432491139819366)	#SAKNAS!	#SAKNAS!
test-day	1	5110(0.011432491139819366)	2359 (0.034)	#SAKNAS!
test-day	1	5110(0.011432491139819366)	2359 (0.034)	#SAKNAS!
test-meal	1	5110(0.011432491139819366)	2359 (0.034)	#SAKNAS!
test-meal	1	5110(0.011432491139819366)	2359 (0.034)	#SAKNAS!
testosterone	1	5110(0.011432491139819366)	2359 (0.034)	#SAKNAS!
testosterone	1	5110(0.011432491139819366)	2359 (0.034)	#SAKNAS!
test-parkinson	1	5110(0.011432491139819366)	#SAKNAS!	2548 (0.033)
test-retest	1	3437(0.02286498227963873)	2359 (0.034)	2548 (0.033)
testudineus	1	5110(0.011432491139819366)	#SAKNAS!	#SAKNAS!
tetracyclines	1	5110(0.011432491139819366)	2359 (0.034)	#SAKNAS!
tetracyclines	1	5110(0.011432491139819366)	2359 (0.034)	#SAKNAS!
tetrahydrocannabinol	1	5110(0.011432491139819366)	2359 (0.034)	#SAKNAS!
tetrahydrocannabinol	1	5110(0.011432491139819366)	2359 (0.034)	#SAKNAS!
tetranychus	1	5110(0.011432491139819366)	#SAKNAS!	2548 (0.033)
tetrao	1	5110(0.011432491139819366)	2359 (0.034)	#SAKNAS!
tetrao	1	5110(0.011432491139819366)	2359 (0.034)	#SAKNAS!
teucrium	1	5110(0.011432491139819366)	2359 (0.034)	#SAKNAS!
teucrium	1	5110(0.011432491139819366)	2359 (0.034)	#SAKNAS!
texas	1	5110(0.011432491139819366)	2359 (0.034)	#SAKNAS!
texas	1	5110(0.011432491139819366)	2359 (0.034)	#SAKNAS!
texel	1	5110(0.011432491139819366)	#SAKNAS!	2548 (0.033)
text	1	5110(0.011432491139819366)	#SAKNAS!	2548 (0.033)
texture-modified	1	743(0.18291985823710985)	2359 (0.034)	1233 (0.1)
textures	1	2693(0.0342974734194581)	2359 (0.034)	1668 (0.066)
tfap2b	1	5110(0.011432491139819366)	#SAKNAS!	2548 (0.033)
tff2	1	5110(0.011432491139819366)	2359 (0.034)	#SAKNAS!
tff2	1	5110(0.011432491139819366)	2359 (0.034)	#SAKNAS!
tg	1	5110(0.011432491139819366)	#SAKNAS!	#SAKNAS!
tgf-劵	1	5110(0.011432491139819366)	#SAKNAS!	#SAKNAS!
thailand-myanmar	1	5110(0.011432491139819366)	#SAKNAS!	2548 (0.033)
thal	1	5110(0.011432491139819366)	#SAKNAS!	2548 (0.033)
thalamita	1	5110(0.011432491139819366)	2359 (0.034)	#SAKNAS!
thalamita	1	5110(0.011432491139819366)	2359 (0.034)	#SAKNAS!
thalassemia	1	5110(0.011432491139819366)	#SAKNAS!	2548 (0.033)
thalassiosira	1	5110(0.011432491139819366)	2359 (0.034)	#SAKNAS!
thalassiosira	1	5110(0.011432491139819366)	2359 (0.034)	#SAKNAS!
thaliana	1	5110(0.011432491139819366)	2359 (0.034)	#SAKNAS!
thaliana	1	5110(0.011432491139819366)	2359 (0.034)	#SAKNAS!
theil	1	5110(0.011432491139819366)	2359 (0.034)	#SAKNAS!

theil	1 5110(0.011432491139819366)	2359 (0.034)	#SAKNAS!
them	1 2693(0.0342974734194581)	2359 (0.034)	#SAKNAS!
theory-based	1 5110(0.011432491139819366)	2359 (0.034)	#SAKNAS!
theory-based	1 5110(0.011432491139819366)	2359 (0.034)	#SAKNAS!
therapeutical	1 5110(0.011432491139819366)	2359 (0.034)	#SAKNAS!
therapeutical	1 5110(0.011432491139819366)	2359 (0.034)	#SAKNAS!
therapist	1 5110(0.011432491139819366)	#SAKNAS!	#SAKNAS!
thereby	1 5110(0.011432491139819366)	#SAKNAS!	#SAKNAS!
theridiidae	1 5110(0.011432491139819366)	2359 (0.034)	#SAKNAS!
theridiidae	1 5110(0.011432491139819366)	2359 (0.034)	#SAKNAS!
therisk	1 5110(0.011432491139819366)	#SAKNAS!	2548 (0.033)
thermally	1 5110(0.011432491139819366)	#SAKNAS!	#SAKNAS!
thermodynamic	1 5110(0.011432491139819366)	2359 (0.034)	#SAKNAS!
thermodynamic	1 5110(0.011432491139819366)	2359 (0.034)	#SAKNAS!
thermogenetic	1 5110(0.011432491139819366)	#SAKNAS!	2548 (0.033)
thermogenic	1 5110(0.011432491139819366)	#SAKNAS!	#SAKNAS!
thermoregulation	1 5110(0.011432491139819366)	2359 (0.034)	#SAKNAS!
thermoregulation	1 5110(0.011432491139819366)	2359 (0.034)	#SAKNAS!
thiamin	1 5110(0.011432491139819366)	#SAKNAS!	#SAKNAS!
thiamine	1 5110(0.011432491139819366)	#SAKNAS!	2548 (0.033)
thibetanus	1 5110(0.011432491139819366)	2359 (0.034)	#SAKNAS!
thibetanus	1 5110(0.011432491139819366)	2359 (0.034)	#SAKNAS!
thick	1 3437(0.02286498227963873)	2359 (0.034)	#SAKNAS!
thief	1 5110(0.011432491139819366)	#SAKNAS!	2548 (0.033)
thigh	1 5110(0.011432491139819366)	#SAKNAS!	#SAKNAS!
thin	1 3437(0.02286498227963873)	2359 (0.034)	#SAKNAS!
third-grade	1 5110(0.011432491139819366)	2359 (0.034)	#SAKNAS!
third-grade	1 5110(0.011432491139819366)	2359 (0.034)	#SAKNAS!
third-level	1 5110(0.011432491139819366)	#SAKNAS!	2548 (0.033)
thirst	1 5110(0.011432491139819366)	#SAKNAS!	2548 (0.033)
thirsty	1 5110(0.011432491139819366)	#SAKNAS!	#SAKNAS!
thirty-six	1 5110(0.011432491139819366)	#SAKNAS!	#SAKNAS!
thixotropy	1 3437(0.02286498227963873)	2359 (0.034)	2548 (0.033)
thoracotomy	1 5110(0.011432491139819366)	#SAKNAS!	2548 (0.033)
thornback	1 5110(0.011432491139819366)	#SAKNAS!	#SAKNAS!
thoughts	1 5110(0.011432491139819366)	#SAKNAS!	#SAKNAS!
threadsail	1 5110(0.011432491139819366)	#SAKNAS!	#SAKNAS!
threats	1 5110(0.011432491139819366)	#SAKNAS!	2548 (0.033)
three-arm	1 5110(0.011432491139819366)	#SAKNAS!	2548 (0.033)
three-spined	1 5110(0.011432491139819366)	2359 (0.034)	#SAKNAS!
three-spined	1 5110(0.011432491139819366)	2359 (0.034)	#SAKNAS!
threshold	1 3437(0.02286498227963873)	2359 (0.034)	2548 (0.033)
thrips	1 5110(0.011432491139819366)	#SAKNAS!	2548 (0.033)

thrombolysed	1 5110(0.011432491139819366)	#SAKNAS!	2548 (0.033)
thrombolysis	1 5110(0.011432491139819366)	#SAKNAS!	#SAKNAS!
thrush	1 5110(0.011432491139819366)	#SAKNAS!	2548 (0.033)
thymallus	1 5110(0.011432491139819366)	#SAKNAS!	2548 (0.033)
thyme	1 5110(0.011432491139819366)	#SAKNAS!	#SAKNAS!
thymus	1 3437(0.02286498227963873)	2359 (0.034)	#SAKNAS!
thyroidectomy	1 2693(0.0342974734194581)	2359 (0.034)	#SAKNAS!
thyroid-function	1 5110(0.011432491139819366)	2359 (0.034)	#SAKNAS!
thyroid-function	1 5110(0.011432491139819366)	2359 (0.034)	#SAKNAS!
thyroiditis	1 5110(0.011432491139819366)	#SAKNAS!	2548 (0.033)
thyropharyngeal	1 5110(0.011432491139819366)	#SAKNAS!	#SAKNAS!
tibet	1 5110(0.011432491139819366)	#SAKNAS!	2548 (0.033)
tidal	1 5110(0.011432491139819366)	2359 (0.034)	#SAKNAS!
tidal	1 5110(0.011432491139819366)	2359 (0.034)	#SAKNAS!
tide	1 5110(0.011432491139819366)	2359 (0.034)	#SAKNAS!
tide	1 5110(0.011432491139819366)	2359 (0.034)	#SAKNAS!
tiger	1 5110(0.011432491139819366)	2359 (0.034)	#SAKNAS!
tiger	1 5110(0.011432491139819366)	2359 (0.034)	#SAKNAS!
tillage	1 5110(0.011432491139819366)	#SAKNAS!	#SAKNAS!
time-based	1 5110(0.011432491139819366)	#SAKNAS!	2548 (0.033)
time-dependent	1 5110(0.011432491139819366)	#SAKNAS!	2548 (0.033)
time-economic-social	1 5110(0.011432491139819366)	#SAKNAS!	#SAKNAS!
time-place	1 5110(0.011432491139819366)	2359 (0.034)	#SAKNAS!
time-place	1 5110(0.011432491139819366)	2359 (0.034)	#SAKNAS!
time-restricted	1 5110(0.011432491139819366)	#SAKNAS!	2548 (0.033)
timor-leste	1 5110(0.011432491139819366)	#SAKNAS!	2548 (0.033)
tinigua	1 5110(0.011432491139819366)	2359 (0.034)	#SAKNAS!
tinigua	1 5110(0.011432491139819366)	2359 (0.034)	#SAKNAS!
tip	1 5110(0.011432491139819366)	2359 (0.034)	#SAKNAS!
tip	1 5110(0.011432491139819366)	2359 (0.034)	#SAKNAS!
tipo	1 5110(0.011432491139819366)	#SAKNAS!	#SAKNAS!
tippelskirchi	1 5110(0.011432491139819366)	#SAKNAS!	2548 (0.033)
tips	1 5110(0.011432491139819366)	#SAKNAS!	2548 (0.033)
tissue-specific	1 5110(0.011432491139819366)	#SAKNAS!	2548 (0.033)
titanium	1 5110(0.011432491139819366)	2359 (0.034)	#SAKNAS!
titanium	1 5110(0.011432491139819366)	2359 (0.034)	#SAKNAS!
titanized	1 5110(0.011432491139819366)	2359 (0.034)	#SAKNAS!
titanized	1 5110(0.011432491139819366)	2359 (0.034)	#SAKNAS!
titers	1 5110(0.011432491139819366)	2359 (0.034)	#SAKNAS!
titers	1 5110(0.011432491139819366)	2359 (0.034)	#SAKNAS!
tlr	1 5110(0.011432491139819366)	#SAKNAS!	#SAKNAS!
tmem18	1 5110(0.011432491139819366)	2359 (0.034)	#SAKNAS!
tmem18	1 5110(0.011432491139819366)	2359 (0.034)	#SAKNAS!

tnf-related	1	5110(0.011432491139819366)	#SAKNAS!	#SAKNAS!
tnf帢	1	5110(0.011432491139819366)	2359 (0.034)	#SAKNAS!
tnf帢	1	5110(0.011432491139819366)	2359 (0.034)	#SAKNAS!
tnik	1	5110(0.011432491139819366)	#SAKNAS!	#SAKNAS!
toads	1	5110(0.011432491139819366)	#SAKNAS!	2548 (0.033)
together	1	5110(0.011432491139819366)	2359 (0.034)	#SAKNAS!
together	1	5110(0.011432491139819366)	2359 (0.034)	#SAKNAS!
toilet	1	5110(0.011432491139819366)	#SAKNAS!	2548 (0.033)
tokyo	1	3437(0.02286498227963873)	2359 (0.034)	2548 (0.033)
toll-like	1	5110(0.011432491139819366)	#SAKNAS!	#SAKNAS!
tomato-based	1	5110(0.011432491139819366)	#SAKNAS!	#SAKNAS!
tomorrow	1	3437(0.02286498227963873)	2359 (0.034)	2548 (0.033)
tone	1	5110(0.011432491139819366)	#SAKNAS!	#SAKNAS!
tongue-lip	1	5110(0.011432491139819366)	#SAKNAS!	#SAKNAS!
tongue-pressure	1	5110(0.011432491139819366)	2359 (0.034)	#SAKNAS!
tongue-pressure	1	5110(0.011432491139819366)	2359 (0.034)	#SAKNAS!
tool-10	1	1678(0.0685949468389162)	2359 (0.034)	1233 (0.1)
tool-bottle-feeding	1	5110(0.011432491139819366)	#SAKNAS!	2548 (0.033)
toolkit	1	5110(0.011432491139819366)	#SAKNAS!	#SAKNAS!
tools	1	1335(0.09145992911855493)	2359 (0.034)	694 (0.2)
toothfish	1	5110(0.011432491139819366)	#SAKNAS!	#SAKNAS!
topic	1	5110(0.011432491139819366)	#SAKNAS!	2548 (0.033)
topics	1	5110(0.011432491139819366)	#SAKNAS!	#SAKNAS!
topography	1	5110(0.011432491139819366)	#SAKNAS!	2548 (0.033)
tor	1	5110(0.011432491139819366)	#SAKNAS!	2548 (0.033)
tor-dependent	1	5110(0.011432491139819366)	#SAKNAS!	2548 (0.033)
tormes	1	5110(0.011432491139819366)	2359 (0.034)	#SAKNAS!
tormes	1	5110(0.011432491139819366)	2359 (0.034)	#SAKNAS!
torrent	1	5110(0.011432491139819366)	#SAKNAS!	2548 (0.033)
torres	1	5110(0.011432491139819366)	#SAKNAS!	#SAKNAS!
tors	1	5110(0.011432491139819366)	#SAKNAS!	#SAKNAS!
tortoises	1	5110(0.011432491139819366)	#SAKNAS!	2548 (0.033)
tortricidae	1	5110(0.011432491139819366)	2359 (0.034)	#SAKNAS!
tortricidae	1	5110(0.011432491139819366)	2359 (0.034)	#SAKNAS!
totally	1	5110(0.011432491139819366)	2359 (0.034)	#SAKNAS!
totally	1	5110(0.011432491139819366)	2359 (0.034)	#SAKNAS!
tottori	1	5110(0.011432491139819366)	#SAKNAS!	#SAKNAS!
touch	1	5110(0.011432491139819366)	#SAKNAS!	2548 (0.033)
tough	1	5110(0.011432491139819366)	#SAKNAS!	#SAKNAS!
toughness	1	5110(0.011432491139819366)	2359 (0.034)	#SAKNAS!
toughness	1	5110(0.011432491139819366)	2359 (0.034)	#SAKNAS!
toupet	1	5110(0.011432491139819366)	#SAKNAS!	2548 (0.033)
toupet-sleeve	1	5110(0.011432491139819366)	#SAKNAS!	#SAKNAS!

toward	1	1678(0.0685949468389162)	2359 (0.034)	1233 (0.1)
towards	1	1335(0.0914599291185549З)	2359 (0.034)	#SAKNAS!
township	1	5110(0.0114324911398193б6)	#SAKNAS!	2548 (0.033)
toxicological	1	5110(0.011432491139819366)	2359 (0.034)	#SAKNAS!
toxicological	1	5110(0.011432491139819366)	2359 (0.034)	#SAKNAS!
toxin-producing	1	5110(0.011432491139819366)	2359 (0.034)	#SAKNAS!
toxin-producing	1	5110(0.011432491139819366)	2359 (0.034)	#SAKNAS!
toxoneuron	1	5110(0.011432491139819366)	#SAKNAS!	2548 (0.033)
toys	1	5110(0.011432491139819366)	#SAKNAS!	#SAKNAS!
tpf	1	5110(0.011432491139819366)	#SAKNAS!	#SAKNAS!
tpn	1	5110(0.011432491139819366)	2359 (0.034)	#SAKNAS!
tpn	1	5110(0.011432491139819366)	2359 (0.034)	#SAKNAS!
tppt	1	5110(0.011432491139819366)	2359 (0.034)	#SAKNAS!
tppt	1	5110(0.011432491139819366)	2359 (0.034)	#SAKNAS!
trabecular	1	5110(0.011432491139819366)	#SAKNAS!	#SAKNAS!
tracers	1	5110(0.011432491139819366)	2359 (0.034)	#SAKNAS!
tracers	1	5110(0.011432491139819366)	2359 (0.034)	#SAKNAS!
tracheal	1	2693(0.0342974734194581)	2359 (0.034)	2548 (0.033)
tracheoesophageal	1	2214(0.04572996455927746)	2359 (0.034)	1668 (0.066)
tracheo-neo-esophageal	1	5110(0.011432491139819366)	2359 (0.034)	#SAKNAS!
tracheo-neo-esophageal	1	5110(0.011432491139819366)	2359 (0.034)	#SAKNAS!
tracheostomy-related	1	5110(0.011432491139819366)	#SAKNAS!	#SAKNAS!
tracheotomized	1	5110(0.011432491139819366)	#SAKNAS!	2548 (0.033)
tracheotomy	1	3437(0.02286498227963873)	2359 (0.034)	#SAKNAS!
trachomatis	1	5110(0.011432491139819366)	#SAKNAS!	#SAKNAS!
trachurus	1	5110(0.011432491139819366)	#SAKNAS!	2548 (0.033)
trachypithecus	1	5110(0.011432491139819366)	#SAKNAS!	#SAKNAS!
tracing	1	5110(0.011432491139819366)	#SAKNAS!	2548 (0.033)
traction	1	3437(0.02286498227963873)	2359 (0.034)	#SAKNAS!
tractus	1	5110(0.011432491139819366)	2359 (0.034)	#SAKNAS!
tractus	1	5110(0.011432491139819366)	2359 (0.034)	#SAKNAS!
trade	1	5110(0.011432491139819366)	#SAKNAS!	2548 (0.033)
tradeoff	1	5110(0.011432491139819366)	2359 (0.034)	#SAKNAS!
tradeoff	1	5110(0.011432491139819366)	2359 (0.034)	#SAKNAS!
trade-off	1	5110(0.011432491139819366)	2359 (0.034)	#SAKNAS!
trade-off	1	5110(0.011432491139819366)	2359 (0.034)	#SAKNAS!
tradeoffs	1	5110(0.011432491139819366)	2359 (0.034)	#SAKNAS!
tradeoffs	1	5110(0.011432491139819366)	2359 (0.034)	#SAKNAS!
trade-offs	1	5110(0.011432491139819366)	#SAKNAS!	2548 (0.033)
trail-following	1	5110(0.011432491139819366)	2359 (0.034)	#SAKNAS!
trail-following	1	5110(0.011432491139819366)	2359 (0.034)	#SAKNAS!
trails	1	5110(0.011432491139819366)	2359 (0.034)	#SAKNAS!
trails	1	5110(0.011432491139819366)	2359 (0.034)	#SAKNAS!

trainers	1	5110(0.011432491139819366)	#SAKNAS!	2548 (0.033)
trajectories	1	1485(0.08002743797873557)	2359 (0.034)	2548 (0.033)
trans	1	5110(0.011432491139819366)	#SAKNAS!	#SAKNAS!
transcatheter	1	5110(0.011432491139819366)	#SAKNAS!	#SAKNAS!
transcription	1	3437(0.02286498227963873)	2359 (0.034)	#SAKNAS!
transcriptional	1	3437(0.02286498227963873)	2359 (0.034)	#SAKNAS!
transcriptomics	1	5110(0.011432491139819366)	#SAKNAS!	#SAKNAS!
transcultural	1	5110(0.011432491139819366)	#SAKNAS!	#SAKNAS!
transcutaneous	1	2214(0.04572996455927746)	2359 (0.034)	1668 (0.066)
transdisciplinary	1	5110(0.011432491139819366)	#SAKNAS!	#SAKNAS!
transesophageal	1	5110(0.011432491139819366)	#SAKNAS!	2548 (0.033)
transformations	1	5110(0.011432491139819366)	#SAKNAS!	2548 (0.033)
transglutaminase	1	5110(0.011432491139819366)	#SAKNAS!	#SAKNAS!
transient	1	5110(0.011432491139819366)	#SAKNAS!	#SAKNAS!
transitional	1	5110(0.011432491139819366)	#SAKNAS!	2548 (0.033)
transitional-state	1	5110(0.011432491139819366)	#SAKNAS!	#SAKNAS!
transitivity	1	5110(0.011432491139819366)	#SAKNAS!	2548 (0.033)
translating	1	3437(0.02286498227963873)	2359 (0.034)	2548 (0.033)
trans-mesocolic	1	5110(0.011432491139819366)	#SAKNAS!	2548 (0.033)
transnasal	1	2214(0.04572996455927746)	2359 (0.034)	1233 (0.1)
trans-nasal	1	5110(0.011432491139819366)	#SAKNAS!	2548 (0.033)
trans-oral	1	5110(0.011432491139819366)	#SAKNAS!	#SAKNAS!
transport	1	2693(0.0342974734194581)	2359 (0.034)	#SAKNAS!
trans-sheath	1	5110(0.011432491139819366)	#SAKNAS!	#SAKNAS!
transtheoretical	1	2693(0.0342974734194581)	2359 (0.034)	2548 (0.033)
transthyretin	1	5110(0.011432491139819366)	#SAKNAS!	#SAKNAS!
transylvania	1	5110(0.011432491139819366)	#SAKNAS!	#SAKNAS!
trapability	1	5110(0.011432491139819366)	2359 (0.034)	#SAKNAS!
trapability	1	5110(0.011432491139819366)	2359 (0.034)	#SAKNAS!
traplining	1	5110(0.011432491139819366)	2359 (0.034)	#SAKNAS!
traplining	1	5110(0.011432491139819366)	2359 (0.034)	#SAKNAS!
traps	1	5110(0.011432491139819366)	#SAKNAS!	2548 (0.033)
treacher	1	5110(0.011432491139819366)	#SAKNAS!	#SAKNAS!
treatment-a	1	5110(0.011432491139819366)	#SAKNAS!	#SAKNAS!
treatment-refractory	1	5110(0.011432491139819366)	#SAKNAS!	2548 (0.033)
treatment-seeking	1	5110(0.011432491139819366)	#SAKNAS!	2548 (0.033)
tree	1	3437(0.02286498227963873)	2359 (0.034)	2548 (0.033)
tree-feeding	1	5110(0.011432491139819366)	2359 (0.034)	#SAKNAS!
tree-feeding	1	5110(0.011432491139819366)	2359 (0.034)	#SAKNAS!
trees	1	3437(0.02286498227963873)	2359 (0.034)	#SAKNAS!
trefoil	1	3437(0.02286498227963873)	2359 (0.034)	#SAKNAS!
trehalose	1	5110(0.011432491139819366)	2359 (0.034)	#SAKNAS!
trehalose	1	5110(0.011432491139819366)	2359 (0.034)	#SAKNAS!

treponema	1	3437(0.02286498227963873)	2359 (0.034)	2548 (0.033)
triacylglycerols	1	5110(0.011432491139819366)	2359 (0.034)	#SAKNAS!
triacylglycerols	1	5110(0.011432491139819366)	2359 (0.034)	#SAKNAS!
triad	1	5110(0.011432491139819366)	#SAKNAS!	#SAKNAS!
trials-a	1	5110(0.011432491139819366)	#SAKNAS!	2548 (0.033)
triangle	1	5110(0.011432491139819366)	#SAKNAS!	#SAKNAS!
triangular	1	5110(0.011432491139819366)	2359 (0.034)	#SAKNAS!
triangular	1	5110(0.011432491139819366)	2359 (0.034)	#SAKNAS!
tribes	1	5110(0.011432491139819366)	2359 (0.034)	#SAKNAS!
tribes	1	5110(0.011432491139819366)	2359 (0.034)	#SAKNAS!
tribolium	1	2693(0.0342974734194581)	2359 (0.034)	1668 (0.066)
tribute	1	5110(0.011432491139819366)	2359 (0.034)	#SAKNAS!
tribute	1	5110(0.011432491139819366)	2359 (0.034)	#SAKNAS!
tricarboxylic	1	5110(0.011432491139819366)	#SAKNAS!	2548 (0.033)
trichogramma	1	2693(0.0342974734194581)	2359 (0.034)	1668 (0.066)
trichogrammatidae	1	5110(0.011432491139819366)	2359 (0.034)	#SAKNAS!
trichogrammatidae	1	5110(0.011432491139819366)	2359 (0.034)	#SAKNAS!
trichoptera	1	3437(0.02286498227963873)	2359 (0.034)	#SAKNAS!
trichosurus	1	5110(0.011432491139819366)	2359 (0.034)	#SAKNAS!
trichosurus	1	5110(0.011432491139819366)	2359 (0.034)	#SAKNAS!
trichosurus-vulpecula	1	5110(0.011432491139819366)	2359 (0.034)	#SAKNAS!
trichosurus-vulpecula	1	5110(0.011432491139819366)	2359 (0.034)	#SAKNAS!
trichromacy	1	5110(0.011432491139819366)	#SAKNAS!	2548 (0.033)
triclosan	1	5110(0.011432491139819366)	#SAKNAS!	2548 (0.033)
tri-comparison	1	5110(0.011432491139819366)	#SAKNAS!	2548 (0.033)
tricuspis	1	5110(0.011432491139819366)	#SAKNAS!	#SAKNAS!
trigger-based	1	5110(0.011432491139819366)	#SAKNAS!	2548 (0.033)
triggers	1	5110(0.011432491139819366)	2359 (0.034)	#SAKNAS!
triggers	1	5110(0.011432491139819366)	2359 (0.034)	#SAKNAS!
triglyceride-glucose	1	5110(0.011432491139819366)	#SAKNAS!	#SAKNAS!
triglycerides	1	2693(0.0342974734194581)	2359 (0.034)	#SAKNAS!
trinidad	1	3437(0.02286498227963873)	2359 (0.034)	2548 (0.033)
trios	1	5110(0.011432491139819366)	2359 (0.034)	#SAKNAS!
trios	1	5110(0.011432491139819366)	2359 (0.034)	#SAKNAS!
tripartite	1	5110(0.011432491139819366)	#SAKNAS!	#SAKNAS!
tripeptides	1	5110(0.011432491139819366)	#SAKNAS!	2548 (0.033)
triple	1	3437(0.02286498227963873)	2359 (0.034)	2548 (0.033)
trirhabda	1	5110(0.011432491139819366)	2359 (0.034)	#SAKNAS!
trirhabda	1	5110(0.011432491139819366)	2359 (0.034)	#SAKNAS!
trismus	1	2214(0.04572996455927746)	2359 (0.034)	1668 (0.066)
trisomy	1	5110(0.011432491139819366)	2359 (0.034)	#SAKNAS!
trisomy	1	5110(0.011432491139819366)	2359 (0.034)	#SAKNAS!
trochoidea	1	5110(0.011432491139819366)	#SAKNAS!	#SAKNAS!

troms	1	5110(0.011432491139819366)	#SAKNAS!	2548 (0.033)
trophodynamics	1	5110(0.011432491139819366)	#SAKNAS!	#SAKNAS!
tropical	1	710(0.19435234937692925)	2359 (0.034)	422 (0.33)
tropicalization	1	5110(0.011432491139819366)	#SAKNAS!	#SAKNAS!
tropics	1	5110(0.011432491139819366)	2359 (0.034)	#SAKNAS!
tropics	1	5110(0.011432491139819366)	2359 (0.034)	#SAKNAS!
tropisetron	1	5110(0.011432491139819366)	2359 (0.034)	#SAKNAS!
tropisetron	1	5110(0.011432491139819366)	2359 (0.034)	#SAKNAS!
trossulus	1	5110(0.011432491139819366)	2359 (0.034)	#SAKNAS!
trossulus	1	5110(0.011432491139819366)	2359 (0.034)	#SAKNAS!
troutsalmo	1	5110(0.011432491139819366)	#SAKNAS!	2548 (0.033)
trujillo	1	5110(0.011432491139819366)	2359 (0.034)	#SAKNAS!
trujillo	1	5110(0.011432491139819366)	2359 (0.034)	#SAKNAS!
trunk	1	5110(0.011432491139819366)	#SAKNAS!	#SAKNAS!
trutta	1	5110(0.011432491139819366)	#SAKNAS!	#SAKNAS!
truttal	1	5110(0.011432491139819366)	#SAKNAS!	2548 (0.033)
tryptophan	1	5110(0.011432491139819366)	2359 (0.034)	#SAKNAS!
tryptophan	1	5110(0.011432491139819366)	2359 (0.034)	#SAKNAS!
tsetse-flies	1	5110(0.011432491139819366)	2359 (0.034)	#SAKNAS!
tsetse-flies	1	5110(0.011432491139819366)	2359 (0.034)	#SAKNAS!
tubal	1	5110(0.011432491139819366)	#SAKNAS!	2548 (0.033)
tube-dependent	1	5110(0.011432491139819366)	2359 (0.034)	#SAKNAS!
tube-dependent	1	5110(0.011432491139819366)	2359 (0.034)	#SAKNAS!
tube-feeding	1	5110(0.011432491139819366)	2359 (0.034)	#SAKNAS!
tube-feeding	1	5110(0.011432491139819366)	2359 (0.034)	#SAKNAS!
tubercle	1	5110(0.011432491139819366)	#SAKNAS!	#SAKNAS!
tube-the	1	5110(0.011432491139819366)	#SAKNAS!	#SAKNAS!
tubifex	1	5110(0.011432491139819366)	2359 (0.034)	#SAKNAS!
tubifex	1	5110(0.011432491139819366)	2359 (0.034)	#SAKNAS!
tubularized	1	5110(0.011432491139819366)	#SAKNAS!	2548 (0.033)
tuco-tuco	1	5110(0.011432491139819366)	2359 (0.034)	#SAKNAS!
tuco-tuco	1	5110(0.011432491139819366)	2359 (0.034)	#SAKNAS!
tufted	1	5110(0.011432491139819366)	2359 (0.034)	#SAKNAS!
tufted	1	5110(0.011432491139819366)	2359 (0.034)	#SAKNAS!
tulear	1	5110(0.011432491139819366)	#SAKNAS!	#SAKNAS!
tumours	1	3437(0.02286498227963873)	2359 (0.034)	2548 (0.033)
tuned	1	5110(0.011432491139819366)	#SAKNAS!	2548 (0.033)
tunisia	1	3437(0.02286498227963873)	2359 (0.034)	2548 (0.033)
tunneled	1	5110(0.011432491139819366)	#SAKNAS!	2548 (0.033)
turbid	1	5110(0.011432491139819366)	2359 (0.034)	#SAKNAS!
turbid	1	5110(0.011432491139819366)	2359 (0.034)	#SAKNAS!
turdus	1	5110(0.011432491139819366)	#SAKNAS!	2548 (0.033)
turin	1	5110(0.011432491139819366)	#SAKNAS!	2548 (0.033)

turmeric	1	5110(0.011432491139819366)	#SAKNAS!	#SAKNAS!
turtle	1	5110(0.011432491139819366)	2359 (0.034)	#SAKNAS!
turtle	1	5110(0.011432491139819366)	2359 (0.034)	#SAKNAS!
tuscan	1	5110(0.011432491139819366)	#SAKNAS!	2548 (0.033)
tuzla	1	5110(0.011432491139819366)	2359 (0.034)	#SAKNAS!
tuzla	1	5110(0.011432491139819366)	2359 (0.034)	#SAKNAS!
tv	1	3437(0.02286498227963873)	2359 (0.034)	2548 (0.033)
tvss	1	5110(0.011432491139819366)	2359 (0.034)	#SAKNAS!
tvss	1	5110(0.011432491139819366)	2359 (0.034)	#SAKNAS!
twelve-year-old	1	5110(0.011432491139819366)	#SAKNAS!	#SAKNAS!
twice	1	3437(0.02286498227963873)	2359 (0.034)	2548 (0.033)
twin	1	2693(0.0342974734194581)	2359 (0.034)	2548 (0.033)
twins	1	3437(0.02286498227963873)	2359 (0.034)	2548 (0.033)
twist	1	5110(0.011432491139819366)	#SAKNAS!	2548 (0.033)
two-coloured	1	5110(0.011432491139819366)	#SAKNAS!	2548 (0.033)
two-domain	1	5110(0.011432491139819366)	#SAKNAS!	2548 (0.033)
two-implant	1	5110(0.011432491139819366)	2359 (0.034)	#SAKNAS!
two-implant	1	5110(0.011432491139819366)	2359 (0.034)	#SAKNAS!
two-item	1	5110(0.011432491139819366)	#SAKNAS!	#SAKNAS!
two-level	1	5110(0.011432491139819366)	#SAKNAS!	#SAKNAS!
two-month	1	5110(0.011432491139819366)	#SAKNAS!	#SAKNAS!
two-stage	1	5110(0.011432491139819366)	2359 (0.034)	#SAKNAS!
two-stage	1	5110(0.011432491139819366)	2359 (0.034)	#SAKNAS!
two-step	1	5110(0.011432491139819366)	#SAKNAS!	2548 (0.033)
two-way	1	5110(0.011432491139819366)	#SAKNAS!	2548 (0.033)
tyg	1	5110(0.011432491139819366)	#SAKNAS!	#SAKNAS!
tygon	1	5110(0.011432491139819366)	2359 (0.034)	#SAKNAS!
tygon	1	5110(0.011432491139819366)	2359 (0.034)	#SAKNAS!
tympanic	1	5110(0.011432491139819366)	#SAKNAS!	2548 (0.033)
type-1	1	5110(0.011432491139819366)	#SAKNAS!	#SAKNAS!
type-i	1	5110(0.011432491139819366)	2359 (0.034)	#SAKNAS!
type-i	1	5110(0.011432491139819366)	2359 (0.034)	#SAKNAS!
typhimurium	1	3437(0.02286498227963873)	2359 (0.034)	#SAKNAS!
typhimurium-induced	1	5110(0.011432491139819366)	#SAKNAS!	#SAKNAS!
typical	1	3437(0.02286498227963873)	2359 (0.034)	2548 (0.033)
typicus	1	5110(0.011432491139819366)	2359 (0.034)	#SAKNAS!
typicus	1	5110(0.011432491139819366)	2359 (0.034)	#SAKNAS!
tyramine-mediated	1	5110(0.011432491139819366)	#SAKNAS!	#SAKNAS!
t羹rkiye	1	5110(0.011432491139819366)	#SAKNAS!	#SAKNAS!
u.s	1	3437(0.02286498227963873)	2359 (0.034)	2548 (0.033)
u.s.a	1	5110(0.011432491139819366)	2359 (0.034)	#SAKNAS!
u.s.a	1	5110(0.011432491139819366)	2359 (0.034)	#SAKNAS!
ucides	1	5110(0.011432491139819366)	#SAKNAS!	2548 (0.033)

ucididae	1	5110(0.011432491139819366)	#SAKNAS!	2548 (0.033)
ufm1	1	5110(0.011432491139819366)	#SAKNAS!	#SAKNAS!
ugandan	1	5110(0.011432491139819366)	#SAKNAS!	#SAKNAS!
ugt46a1	1	5110(0.011432491139819366)	#SAKNAS!	#SAKNAS!
ukrainian	1	5110(0.011432491139819366)	2359 (0.034)	#SAKNAS!
ukrainian	1	5110(0.011432491139819366)	2359 (0.034)	#SAKNAS!
ulaanbaatar	1	5110(0.011432491139819366)	2359 (0.034)	#SAKNAS!
ulaanbaatar	1	5110(0.011432491139819366)	2359 (0.034)	#SAKNAS!
ulceration	1	3437(0.02286498227963873)	2359 (0.034)	2548 (0.033)
ulcerations	1	5110(0.011432491139819366)	2359 (0.034)	#SAKNAS!
ulcerations	1	5110(0.011432491139819366)	2359 (0.034)	#SAKNAS!
ultimum	1	5110(0.011432491139819366)	2359 (0.034)	#SAKNAS!
ultimum	1	5110(0.011432491139819366)	2359 (0.034)	#SAKNAS!
ultradian	1	3437(0.02286498227963873)	2359 (0.034)	#SAKNAS!
ultra-high	1	5110(0.011432491139819366)	#SAKNAS!	#SAKNAS!
ultrasonic	1	2214(0.04572996455927746)	2359 (0.034)	2548 (0.033)
ultrasound-based	1	5110(0.011432491139819366)	#SAKNAS!	#SAKNAS!
ultrastructural	1	5110(0.011432491139819366)	#SAKNAS!	#SAKNAS!
ultrastructure	1	5110(0.011432491139819366)	#SAKNAS!	2548 (0.033)
ultrathin	1	5110(0.011432491139819366)	2359 (0.034)	#SAKNAS!
ultrathin	1	5110(0.011432491139819366)	2359 (0.034)	#SAKNAS!
ultraviolet	1	5110(0.011432491139819366)	2359 (0.034)	#SAKNAS!
ultraviolet	1	5110(0.011432491139819366)	2359 (0.034)	#SAKNAS!
ulvae	1	5110(0.011432491139819366)	2359 (0.034)	#SAKNAS!
ulvae	1	5110(0.011432491139819366)	2359 (0.034)	#SAKNAS!
umbrosus	1	5110(0.011432491139819366)	#SAKNAS!	#SAKNAS!
umsars	1	5110(0.011432491139819366)	#SAKNAS!	#SAKNAS!
una	1	5110(0.011432491139819366)	#SAKNAS!	#SAKNAS!
uncommon	1	5110(0.011432491139819366)	#SAKNAS!	#SAKNAS!
uncooperative	1	5110(0.011432491139819366)	#SAKNAS!	2548 (0.033)
unculturables	1	5110(0.011432491139819366)	#SAKNAS!	2548 (0.033)
undergraduate	1	2214(0.04572996455927746)	2359 (0.034)	2548 (0.033)
undergraduates	1	5110(0.011432491139819366)	#SAKNAS!	#SAKNAS!
underlie	1	5110(0.011432491139819366)	2359 (0.034)	#SAKNAS!
underlie	1	5110(0.011432491139819366)	2359 (0.034)	#SAKNAS!
undernourishment	1	5110(0.011432491139819366)	2359 (0.034)	#SAKNAS!
undernourishment	1	5110(0.011432491139819366)	2359 (0.034)	#SAKNAS!
undernutrition-induced	1	5110(0.011432491139819366)	2359 (0.034)	#SAKNAS!
undernutrition-induced	1	5110(0.011432491139819366)	2359 (0.034)	#SAKNAS!
underpinning	1	3437(0.02286498227963873)	2359 (0.034)	2548 (0.033)
underpins	1	5110(0.011432491139819366)	#SAKNAS!	#SAKNAS!
under-recognised	1	5110(0.011432491139819366)	#SAKNAS!	#SAKNAS!
undervalued	1	5110(0.011432491139819366)	#SAKNAS!	2548 (0.033)

undesirable	1 5110(0.011432491139819366)	#SAKNAS!	2548 (0.033)
undone	1 5110(0.011432491139819366)	2359 (0.034)	#SAKNAS!
undone	1 5110(0.011432491139819366)	2359 (0.034)	#SAKNAS!
unequal	1 5110(0.011432491139819366)	#SAKNAS!	#SAKNAS!
ungulate	1 5110(0.011432491139819366)	#SAKNAS!	2548 (0.033)
ungulates	1 5110(0.011432491139819366)	#SAKNAS!	2548 (0.033)
unicellular	1 5110(0.011432491139819366)	2359 (0.034)	#SAKNAS!
unicellular	1 5110(0.011432491139819366)	2359 (0.034)	#SAKNAS!
unicentric	1 5110(0.011432491139819366)	#SAKNAS!	#SAKNAS!
unifying	1 5110(0.011432491139819366)	2359 (0.034)	#SAKNAS!
unifying	1 5110(0.011432491139819366)	2359 (0.034)	#SAKNAS!
unilateral	1 5110(0.011432491139819366)	#SAKNAS!	#SAKNAS!
unintentional	1 3437(0.02286498227963873)	2359 (0.034)	2548 (0.033)
union	1 5110(0.011432491139819366)	#SAKNAS!	2548 (0.033)
unionid	1 5110(0.011432491139819366)	2359 (0.034)	#SAKNAS!
unionid	1 5110(0.011432491139819366)	2359 (0.034)	#SAKNAS!
unique	1 5110(0.011432491139819366)	#SAKNAS!	#SAKNAS!
united-states	1 5110(0.011432491139819366)	2359 (0.034)	#SAKNAS!
united-states	1 5110(0.011432491139819366)	2359 (0.034)	#SAKNAS!
univentricle	1 5110(0.011432491139819366)	2359 (0.034)	#SAKNAS!
univentricle	1 5110(0.011432491139819366)	2359 (0.034)	#SAKNAS!
universal	1 5110(0.011432491139819366)	#SAKNAS!	2548 (0.033)
unpredictable	1 3437(0.02286498227963873)	2359 (0.034)	2548 (0.033)
unraveling	1 5110(0.011432491139819366)	#SAKNAS!	2548 (0.033)
unravelling	1 5110(0.011432491139819366)	#SAKNAS!	#SAKNAS!
unreleasable	1 5110(0.011432491139819366)	2359 (0.034)	#SAKNAS!
unreleasable	1 5110(0.011432491139819366)	2359 (0.034)	#SAKNAS!
unreplaced	1 5110(0.011432491139819366)	2359 (0.034)	#SAKNAS!
unreplaced	1 5110(0.011432491139819366)	2359 (0.034)	#SAKNAS!
unripe	1 5110(0.011432491139819366)	2359 (0.034)	#SAKNAS!
unripe	1 5110(0.011432491139819366)	2359 (0.034)	#SAKNAS!
unrwa	1 5110(0.011432491139819366)	#SAKNAS!	2548 (0.033)
unsafe	1 5110(0.011432491139819366)	#SAKNAS!	2548 (0.033)
unsatisfactory	1 5110(0.011432491139819366)	#SAKNAS!	#SAKNAS!
unsedated	1 5110(0.011432491139819366)	#SAKNAS!	2548 (0.033)
unstable	1 5110(0.011432491139819366)	#SAKNAS!	#SAKNAS!
until	1 5110(0.011432491139819366)	#SAKNAS!	2548 (0.033)
unveil	1 5110(0.011432491139819366)	#SAKNAS!	#SAKNAS!
unveils	1 5110(0.011432491139819366)	#SAKNAS!	#SAKNAS!
unvesity	1 5110(0.011432491139819366)	2359 (0.034)	#SAKNAS!
unvesity	1 5110(0.011432491139819366)	2359 (0.034)	#SAKNAS!
uoridated	1 5110(0.011432491139819366)	#SAKNAS!	#SAKNAS!
upbeat	1 5110(0.011432491139819366)	#SAKNAS!	#SAKNAS!

updating	1	5110(0.011432491139819366)	2359 (0.034)	#SAKNAS!
updating	1	5110(0.011432491139819366)	2359 (0.034)	#SAKNAS!
uppp	1	5110(0.011432491139819366)	2359 (0.034)	#SAKNAS!
uppp	1	5110(0.011432491139819366)	2359 (0.034)	#SAKNAS!
upregulates	1	5110(0.011432491139819366)	#SAKNAS!	2548 (0.033)
up-regulation	1	5110(0.011432491139819366)	#SAKNAS!	2548 (0.033)
uprer-immunity	1	5110(0.011432491139819366)	#SAKNAS!	#SAKNAS!
up-to-the-minute	1	5110(0.011432491139819366)	2359 (0.034)	#SAKNAS!
up-to-the-minute	1	5110(0.011432491139819366)	2359 (0.034)	#SAKNAS!
urban-dwelling	1	5110(0.011432491139819366)	#SAKNAS!	#SAKNAS!
urbanisation	1	5110(0.011432491139819366)	2359 (0.034)	#SAKNAS!
urbanisation	1	5110(0.011432491139819366)	2359 (0.034)	#SAKNAS!
urbanization	1	5110(0.011432491139819366)	#SAKNAS!	#SAKNAS!
urchin	1	5110(0.011432491139819366)	2359 (0.034)	#SAKNAS!
urchin	1	5110(0.011432491139819366)	2359 (0.034)	#SAKNAS!
urchins	1	5110(0.011432491139819366)	#SAKNAS!	2548 (0.033)
urea	1	5110(0.011432491139819366)	2359 (0.034)	#SAKNAS!
urea	1	5110(0.011432491139819366)	2359 (0.034)	#SAKNAS!
uria	1	5110(0.011432491139819366)	2359 (0.034)	#SAKNAS!
uria	1	5110(0.011432491139819366)	2359 (0.034)	#SAKNAS!
uridine	1	5110(0.011432491139819366)	#SAKNAS!	#SAKNAS!
urinary	1	1111(0.11432491139819366)	2359 (0.034)	816 (0.16)
urine	1	5110(0.011432491139819366)	#SAKNAS!	#SAKNAS!
urogallus	1	5110(0.011432491139819366)	2359 (0.034)	#SAKNAS!
urogallus	1	5110(0.011432491139819366)	2359 (0.034)	#SAKNAS!
urokinase	1	5110(0.011432491139819366)	#SAKNAS!	#SAKNAS!
urolithiasis	1	5110(0.011432491139819366)	#SAKNAS!	2548 (0.033)
ursus	1	5110(0.011432491139819366)	2359 (0.034)	#SAKNAS!
ursus	1	5110(0.011432491139819366)	2359 (0.034)	#SAKNAS!
urticae	1	5110(0.011432491139819366)	#SAKNAS!	2548 (0.033)
uruguayan	1	5110(0.011432491139819366)	2359 (0.034)	#SAKNAS!
uruguayan	1	5110(0.011432491139819366)	2359 (0.034)	#SAKNAS!
usability	1	5110(0.011432491139819366)	#SAKNAS!	#SAKNAS!
user-centered	1	5110(0.011432491139819366)	#SAKNAS!	#SAKNAS!
usual	1	3437(0.02286498227963873)	2359 (0.034)	#SAKNAS!
utero	1	5110(0.011432491139819366)	#SAKNAS!	2548 (0.033)
utetheisa	1	5110(0.011432491139819366)	2359 (0.034)	#SAKNAS!
utetheisa	1	5110(0.011432491139819366)	2359 (0.034)	#SAKNAS!
utilisation	1	3437(0.02286498227963873)	2359 (0.034)	#SAKNAS!
utility	1	1111(0.11432491139819366)	2359 (0.034)	816 (0.16)
utilizes	1	5110(0.011432491139819366)	#SAKNAS!	#SAKNAS!
uvulopalatal	1	5110(0.011432491139819366)	2359 (0.034)	#SAKNAS!
uvulopalatal	1	5110(0.011432491139819366)	2359 (0.034)	#SAKNAS!

uvulopalatopharyngoplasty	1	5110(0.011432491139819366)	#SAKNAS!	2548 (0.033)
uwsfr	1	5110(0.011432491139819366)	#SAKNAS!	2548 (0.033)
va	1	5110(0.011432491139819366)	#SAKNAS!	2548 (0.033)
vaccine	1	5110(0.011432491139819366)	#SAKNAS!	#SAKNAS!
vaccinium	1	5110(0.011432491139819366)	#SAKNAS!	#SAKNAS!
vaginal	1	3437(0.02286498227963873)	2359 (0.034)	2548 (0.033)
vaginosis	1	5110(0.011432491139819366)	#SAKNAS!	#SAKNAS!
val66met	1	5110(0.011432491139819366)	2359 (0.034)	#SAKNAS!
val66met	1	5110(0.011432491139819366)	2359 (0.034)	#SAKNAS!
validate	1	2693(0.0342974734194581)	2359 (0.034)	#SAKNAS!
validus	1	5110(0.011432491139819366)	2359 (0.034)	#SAKNAS!
validus	1	5110(0.011432491139819366)	2359 (0.034)	#SAKNAS!
valine-deficient	1	5110(0.011432491139819366)	2359 (0.034)	#SAKNAS!
valine-deficient	1	5110(0.011432491139819366)	2359 (0.034)	#SAKNAS!
valuable	1	5110(0.011432491139819366)	#SAKNAS!	#SAKNAS!
valuation	1	5110(0.011432491139819366)	2359 (0.034)	#SAKNAS!
valuation	1	5110(0.011432491139819366)	2359 (0.034)	#SAKNAS!
value-based	1	5110(0.011432491139819366)	#SAKNAS!	2548 (0.033)
valvarum	1	5110(0.011432491139819366)	2359 (0.034)	#SAKNAS!
valvarum	1	5110(0.011432491139819366)	2359 (0.034)	#SAKNAS!
valve	1	3437(0.02286498227963873)	2359 (0.034)	#SAKNAS!
valvuloplasty	1	5110(0.011432491139819366)	#SAKNAS!	#SAKNAS!
vanessa	1	5110(0.011432491139819366)	#SAKNAS!	2548 (0.033)
vannamei	1	2693(0.0342974734194581)	2359 (0.034)	1668 (0.066)
vanzolinii	1	5110(0.011432491139819366)	#SAKNAS!	2548 (0.033)
vaping	1	5110(0.011432491139819366)	#SAKNAS!	#SAKNAS!
variant	1	2693(0.0342974734194581)	2359 (0.034)	2548 (0.033)
variants	1	2214(0.04572996455927746)	2359 (0.034)	1668 (0.066)
variegata	1	5110(0.011432491139819366)	#SAKNAS!	2548 (0.033)
variegated	1	5110(0.011432491139819366)	2359 (0.034)	#SAKNAS!
variegated	1	5110(0.011432491139819366)	2359 (0.034)	#SAKNAS!
varies	1	2214(0.04572996455927746)	2359 (0.034)	#SAKNAS!
varieties	1	5110(0.011432491139819366)	#SAKNAS!	#SAKNAS!
variety-seeking	1	5110(0.011432491139819366)	2359 (0.034)	#SAKNAS!
variety-seeking	1	5110(0.011432491139819366)	2359 (0.034)	#SAKNAS!
varmland	1	5110(0.011432491139819366)	2359 (0.034)	#SAKNAS!
varmland	1	5110(0.011432491139819366)	2359 (0.034)	#SAKNAS!
varnish	1	5110(0.011432491139819366)	#SAKNAS!	#SAKNAS!
vary	1	5110(0.011432491139819366)	#SAKNAS!	2548 (0.033)
varying	1	2693(0.0342974734194581)	2359 (0.034)	2548 (0.033)
vasopressin	1	5110(0.011432491139819366)	2359 (0.034)	#SAKNAS!
vasopressin	1	5110(0.011432491139819366)	2359 (0.034)	#SAKNAS!
vaspin	1	5110(0.011432491139819366)	#SAKNAS!	2548 (0.033)

veal	1	5110(0.011432491139819366)	2359 (0.034)	#SAKNAS!
veal	1	5110(0.011432491139819366)	2359 (0.034)	#SAKNAS!
vector	1	5110(0.011432491139819366)	#SAKNAS!	#SAKNAS!
vegetarians	1	3437(0.02286498227963873)	2359 (0.034)	#SAKNAS!
vehicle	1	5110(0.011432491139819366)	2359 (0.034)	#SAKNAS!
vehicle	1	5110(0.011432491139819366)	2359 (0.034)	#SAKNAS!
veillonella	1	5110(0.011432491139819366)	2359 (0.034)	#SAKNAS!
veillonella	1	5110(0.011432491139819366)	2359 (0.034)	#SAKNAS!
velocity	1	5110(0.011432491139819366)	2359 (0.034)	#SAKNAS!
velocity	1	5110(0.011432491139819366)	2359 (0.034)	#SAKNAS!
velvetbean	1	5110(0.011432491139819366)	2359 (0.034)	#SAKNAS!
velvetbean	1	5110(0.011432491139819366)	2359 (0.034)	#SAKNAS!
vemurafenib	1	5110(0.011432491139819366)	#SAKNAS!	2548 (0.033)
venerupis	1	5110(0.011432491139819366)	2359 (0.034)	#SAKNAS!
venerupis	1	5110(0.011432491139819366)	2359 (0.034)	#SAKNAS!
venezuela	1	5110(0.011432491139819366)	2359 (0.034)	#SAKNAS!
venezuela	1	5110(0.011432491139819366)	2359 (0.034)	#SAKNAS!
venosa	1	5110(0.011432491139819366)	#SAKNAS!	#SAKNAS!
ventilator	1	5110(0.011432491139819366)	#SAKNAS!	#SAKNAS!
ventilators	1	5110(0.011432491139819366)	#SAKNAS!	#SAKNAS!
ventilatory	1	3437(0.02286498227963873)	2359 (0.034)	2548 (0.033)
ventricle	1	5110(0.011432491139819366)	#SAKNAS!	2548 (0.033)
ventrolateral	1	5110(0.011432491139819366)	#SAKNAS!	#SAKNAS!
venturia	1	5110(0.011432491139819366)	2359 (0.034)	#SAKNAS!
venturia	1	5110(0.011432491139819366)	2359 (0.034)	#SAKNAS!
verminephrobacter	1	5110(0.011432491139819366)	#SAKNAS!	2548 (0.033)
vernonanthura	1	5110(0.011432491139819366)	#SAKNAS!	2548 (0.033)
vers瓊o	1	5110(0.011432491139819366)	#SAKNAS!	#SAKNAS!
vertebral	1	5110(0.011432491139819366)	2359 (0.034)	#SAKNAS!
vertebral	1	5110(0.011432491139819366)	2359 (0.034)	#SAKNAS!
vertebrates	1	5110(0.011432491139819366)	#SAKNAS!	2548 (0.033)
vervet	1	5110(0.011432491139819366)	#SAKNAS!	2548 (0.033)
vessel	1	5110(0.011432491139819366)	2359 (0.034)	#SAKNAS!
vessel	1	5110(0.011432491139819366)	2359 (0.034)	#SAKNAS!
veterinary	1	5110(0.011432491139819366)	2359 (0.034)	#SAKNAS!
veterinary	1	5110(0.011432491139819366)	2359 (0.034)	#SAKNAS!
viable	1	3437(0.02286498227963873)	2359 (0.034)	#SAKNAS!
vibrations	1	5110(0.011432491139819366)	#SAKNAS!	#SAKNAS!
viciifolia	1	5110(0.011432491139819366)	#SAKNAS!	2548 (0.033)
vicious	1	5110(0.011432491139819366)	#SAKNAS!	2548 (0.033)
victim	1	5110(0.011432491139819366)	2359 (0.034)	#SAKNAS!
victim	1	5110(0.011432491139819366)	2359 (0.034)	#SAKNAS!
victims	1	3437(0.02286498227963873)	2359 (0.034)	2548 (0.033)

victoria	1	5110(0.011432491139819366)	#SAKNAS!	2548 (0.033)
videoendoscopy	1	5110(0.011432491139819366)	#SAKNAS!	2548 (0.033)
videofluorography	1	3437(0.02286498227963873)	2359 (0.034)	2548 (0.033)
videofluoromanometric	1	5110(0.011432491139819366)	#SAKNAS!	2548 (0.033)
videofluoroscopy	1	1335(0.09145992911855493)	2359 (0.034)	816 (0.16)
video-laparoscopic	1	5110(0.011432491139819366)	#SAKNAS!	2548 (0.033)
videoradiographic	1	5110(0.011432491139819366)	2359 (0.034)	#SAKNAS!
videoradiographic	1	5110(0.011432491139819366)	2359 (0.034)	#SAKNAS!
view	1	1894(0.05716245569909683)	2359 (0.034)	999 (0.13)
viewing	1	2693(0.0342974734194581)	2359 (0.034)	1668 (0.066)
views	1	1485(0.08002743797873557)	2359 (0.034)	999 (0.13)
vigilance	1	5110(0.011432491139819366)	#SAKNAS!	#SAKNAS!
villages	1	5110(0.011432491139819366)	#SAKNAS!	2548 (0.033)
villosum	1	5110(0.011432491139819366)	#SAKNAS!	2548 (0.033)
vine	1	5110(0.011432491139819366)	2359 (0.034)	#SAKNAS!
vine	1	5110(0.011432491139819366)	2359 (0.034)	#SAKNAS!
vines	1	5110(0.011432491139819366)	2359 (0.034)	#SAKNAS!
vines	1	5110(0.011432491139819366)	2359 (0.034)	#SAKNAS!
vinifera	1	5110(0.011432491139819366)	2359 (0.034)	#SAKNAS!
vinifera	1	5110(0.011432491139819366)	2359 (0.034)	#SAKNAS!
viral	1	5110(0.011432491139819366)	#SAKNAS!	2548 (0.033)
virens	1	5110(0.011432491139819366)	#SAKNAS!	2548 (0.033)
virgatus	1	5110(0.011432491139819366)	2359 (0.034)	#SAKNAS!
virgatus	1	5110(0.011432491139819366)	2359 (0.034)	#SAKNAS!
virgifera	1	5110(0.011432491139819366)	2359 (0.034)	#SAKNAS!
virgifera	1	5110(0.011432491139819366)	2359 (0.034)	#SAKNAS!
virgin	1	5110(0.011432491139819366)	2359 (0.034)	#SAKNAS!
virgin	1	5110(0.011432491139819366)	2359 (0.034)	#SAKNAS!
virginiana	1	5110(0.011432491139819366)	#SAKNAS!	#SAKNAS!
viridicornis	1	5110(0.011432491139819366)	#SAKNAS!	2548 (0.033)
viridis	1	5110(0.011432491139819366)	2359 (0.034)	#SAKNAS!
viridis	1	5110(0.011432491139819366)	2359 (0.034)	#SAKNAS!
virtual	1	5110(0.011432491139819366)	#SAKNAS!	2548 (0.033)
virus	1	966(0.1371898936778324)	2359 (0.034)	999 (0.13)
virus-enriched	1	5110(0.011432491139819366)	#SAKNAS!	2548 (0.033)
viruses	1	5110(0.011432491139819366)	#SAKNAS!	#SAKNAS!
virus-mediated	1	5110(0.011432491139819366)	2359 (0.034)	#SAKNAS!
virus-mediated	1	5110(0.011432491139819366)	2359 (0.034)	#SAKNAS!
virus-negative	1	5110(0.011432491139819366)	#SAKNAS!	#SAKNAS!
visceral	1	1894(0.05716245569909683)	2359 (0.034)	2548 (0.033)
viscoelasticity	1	5110(0.011432491139819366)	2359 (0.034)	#SAKNAS!
viscoelasticity	1	5110(0.011432491139819366)	2359 (0.034)	#SAKNAS!
viscous	1	5110(0.011432491139819366)	#SAKNAS!	2548 (0.033)

visfatin	1	5110(0.011432491139819366)	#SAKNAS!	2548 (0.033)
visitor	1	5110(0.011432491139819366)	2359 (0.034)	#SAKNAS!
visitor	1	5110(0.011432491139819366)	2359 (0.034)	#SAKNAS!
visitors	1	3437(0.02286498227963873)	2359 (0.034)	#SAKNAS!
vison	1	5110(0.011432491139819366)	2359 (0.034)	#SAKNAS!
vison	1	5110(0.011432491139819366)	2359 (0.034)	#SAKNAS!
visual	1	1335(0.09145992911855493)	2359 (0.034)	1668 (0.066)
visualization	1	5110(0.011432491139819366)	#SAKNAS!	#SAKNAS!
visually	1	5110(0.011432491139819366)	#SAKNAS!	#SAKNAS!
vitalstim	1	5110(0.011432491139819366)	2359 (0.034)	#SAKNAS!
vitalstim	1	5110(0.011432491139819366)	2359 (0.034)	#SAKNAS!
vitamin-b12	1	5110(0.011432491139819366)	2359 (0.034)	#SAKNAS!
vitamin-b12	1	5110(0.011432491139819366)	2359 (0.034)	#SAKNAS!
vitis	1	5110(0.011432491139819366)	2359 (0.034)	#SAKNAS!
vitis	1	5110(0.011432491139819366)	2359 (0.034)	#SAKNAS!
vitis-idaea	1	5110(0.011432491139819366)	#SAKNAS!	#SAKNAS!
vivomicrofluidic	1	5110(0.011432491139819366)	#SAKNAS!	2548 (0.033)
vocalisations	1	5110(0.011432491139819366)	#SAKNAS!	#SAKNAS!
volans	1	5110(0.011432491139819366)	#SAKNAS!	2548 (0.033)
volatile	1	1894(0.05716245569909683)	2359 (0.034)	2548 (0.033)
volcanic	1	5110(0.011432491139819366)	#SAKNAS!	#SAKNAS!
vole	1	5110(0.011432491139819366)	2359 (0.034)	#SAKNAS!
vole	1	5110(0.011432491139819366)	2359 (0.034)	#SAKNAS!
voles	1	5110(0.011432491139819366)	#SAKNAS!	2548 (0.033)
volitional	1	5110(0.011432491139819366)	#SAKNAS!	#SAKNAS!
volleyball	1	5110(0.011432491139819366)	#SAKNAS!	2548 (0.033)
volumes	1	5110(0.011432491139819366)	2359 (0.034)	#SAKNAS!
volumes	1	5110(0.011432491139819366)	2359 (0.034)	#SAKNAS!
volvulus	1	3437(0.02286498227963873)	2359 (0.034)	2548 (0.033)
vomiting	1	5110(0.011432491139819366)	2359 (0.034)	#SAKNAS!
vomiting	1	5110(0.011432491139819366)	2359 (0.034)	#SAKNAS!
vorticosus	1	5110(0.011432491139819366)	#SAKNAS!	#SAKNAS!
vulgare	1	5110(0.011432491139819366)	2359 (0.034)	#SAKNAS!
vulgare	1	5110(0.011432491139819366)	2359 (0.034)	#SAKNAS!
vulnerable	1	3437(0.02286498227963873)	2359 (0.034)	2548 (0.033)
vulpecula	1	5110(0.011432491139819366)	2359 (0.034)	#SAKNAS!
vulpecula	1	5110(0.011432491139819366)	2359 (0.034)	#SAKNAS!
wage	1	5110(0.011432491139819366)	#SAKNAS!	#SAKNAS!
waist-to-height	1	5110(0.011432491139819366)	#SAKNAS!	2548 (0.033)
wait	1	5110(0.011432491139819366)	#SAKNAS!	2548 (0.033)
walbaum	1	5110(0.011432491139819366)	2359 (0.034)	#SAKNAS!
walbaum	1	5110(0.011432491139819366)	2359 (0.034)	#SAKNAS!
wall	1	2214(0.04572996455927746)	2359 (0.034)	2548 (0.033)

wallabies	1	5110(0.011432491139819366)	2359 (0.034)	#SAKNAS!
wallabies	1	5110(0.011432491139819366)	2359 (0.034)	#SAKNAS!
wallflex簀	1	5110(0.011432491139819366)	#SAKNAS!	2548 (0.033)
wanging	1	5110(0.011432491139819366)	#SAKNAS!	#SAKNAS!
want	1	5110(0.011432491139819366)	#SAKNAS!	2548 (0.033)
wanting	1	5110(0.011432491139819366)	2359 (0.034)	#SAKNAS!
wanting	1	5110(0.011432491139819366)	2359 (0.034)	#SAKNAS!
wapiti	1	5110(0.011432491139819366)	2359 (0.034)	#SAKNAS!
wapiti	1	5110(0.011432491139819366)	2359 (0.034)	#SAKNAS!
ward	1	1335(0.09145992911855493)	2359 (0.034)	1233 (0.1)
warm-temperate	1	5110(0.011432491139819366)	#SAKNAS!	2548 (0.033)
wash	1	5110(0.011432491139819366)	#SAKNAS!	2548 (0.033)
washington	1	5110(0.011432491139819366)	2359 (0.034)	#SAKNAS!
washington	1	5110(0.011432491139819366)	2359 (0.034)	#SAKNAS!
washington-state	1	5110(0.011432491139819366)	2359 (0.034)	#SAKNAS!
washington-state	1	5110(0.011432491139819366)	2359 (0.034)	#SAKNAS!
wasp	1	2693(0.0342974734194581)	2359 (0.034)	2548 (0.033)
wasps	1	5110(0.011432491139819366)	2359 (0.034)	#SAKNAS!
wasps	1	5110(0.011432491139819366)	2359 (0.034)	#SAKNAS!
waste	1	2693(0.0342974734194581)	2359 (0.034)	#SAKNAS!
wasting	1	2214(0.04572996455927746)	2359 (0.034)	2548 (0.033)
waterborne	1	5110(0.011432491139819366)	2359 (0.034)	#SAKNAS!
waterborne	1	5110(0.011432491139819366)	2359 (0.034)	#SAKNAS!
water-quality	1	5110(0.011432491139819366)	2359 (0.034)	#SAKNAS!
water-quality	1	5110(0.011432491139819366)	2359 (0.034)	#SAKNAS!
wave	1	2214(0.04572996455927746)	2359 (0.034)	1668 (0.066)
waveform	1	5110(0.011432491139819366)	#SAKNAS!	#SAKNAS!
wavelet	1	5110(0.011432491139819366)	2359 (0.034)	#SAKNAS!
wavelet	1	5110(0.011432491139819366)	2359 (0.034)	#SAKNAS!
wax	1	5110(0.011432491139819366)	#SAKNAS!	#SAKNAS!
way	1	5110(0.011432491139819366)	#SAKNAS!	#SAKNAS!
way-100635	1	5110(0.011432491139819366)	2359 (0.034)	#SAKNAS!
way-100635	1	5110(0.011432491139819366)	2359 (0.034)	#SAKNAS!
ways	1	3437(0.02286498227963873)	2359 (0.034)	2548 (0.033)
weakly	1	5110(0.011432491139819366)	2359 (0.034)	#SAKNAS!
weakly	1	5110(0.011432491139819366)	2359 (0.034)	#SAKNAS!
weakness	1	3437(0.02286498227963873)	2359 (0.034)	2548 (0.033)
wealth	1	5110(0.011432491139819366)	#SAKNAS!	2548 (0.033)
wean	1	5110(0.011432491139819366)	#SAKNAS!	2548 (0.033)
weancare	1	5110(0.011432491139819366)	#SAKNAS!	#SAKNAS!
weaner	1	5110(0.011432491139819366)	#SAKNAS!	#SAKNAS!
weanling	1	5110(0.011432491139819366)	2359 (0.034)	#SAKNAS!
weanling	1	5110(0.011432491139819366)	2359 (0.034)	#SAKNAS!

weanlings	1	5110(0.011432491139819366)	#SAKNAS!	2548 (0.033)
wearer	1	5110(0.011432491139819366)	#SAKNAS!	#SAKNAS!
weasel	1	5110(0.011432491139819366)	2359 (0.034)	#SAKNAS!
weasel	1	5110(0.011432491139819366)	2359 (0.034)	#SAKNAS!
webs	1	5110(0.011432491139819366)	#SAKNAS!	2548 (0.033)
websites	1	5110(0.011432491139819366)	#SAKNAS!	#SAKNAS!
weeds	1	5110(0.011432491139819366)	#SAKNAS!	2548 (0.033)
week	1	2693(0.0342974734194581)	2359 (0.034)	1668 (0.066)
weekdays	1	5110(0.011432491139819366)	#SAKNAS!	2548 (0.033)
weekend-weekday	1	5110(0.011432491139819366)	#SAKNAS!	#SAKNAS!
weekly	1	3437(0.02286498227963873)	2359 (0.034)	2548 (0.033)
wehncke	1	3437(0.02286498227963873)	2359 (0.034)	2548 (0.033)
weighing	1	3437(0.02286498227963873)	2359 (0.034)	2548 (0.033)
weight-a	1	5110(0.011432491139819366)	#SAKNAS!	2548 (0.033)
welfare	1	1335(0.09145992911855493)	2359 (0.034)	2548 (0.033)
well-being	1	896(0.14862238481765175)	2359 (0.034)	694 (0.2)
well-child	1	5110(0.011432491139819366)	#SAKNAS!	#SAKNAS!
well-functioning	1	5110(0.011432491139819366)	2359 (0.034)	#SAKNAS!
well-functioning	1	5110(0.011432491139819366)	2359 (0.034)	#SAKNAS!
wellingtonensis	1	5110(0.011432491139819366)	#SAKNAS!	2548 (0.033)
well-planned	1	5110(0.011432491139819366)	#SAKNAS!	2548 (0.033)
wenchuan	1	5110(0.011432491139819366)	2359 (0.034)	#SAKNAS!
wenchuan	1	5110(0.011432491139819366)	2359 (0.034)	#SAKNAS!
wernicke	1	5110(0.011432491139819366)	2359 (0.034)	#SAKNAS!
wernicke	1	5110(0.011432491139819366)	2359 (0.034)	#SAKNAS!
wernicke-korsakoff	1	5110(0.011432491139819366)	2359 (0.034)	#SAKNAS!
wernicke-korsakoff	1	5110(0.011432491139819366)	2359 (0.034)	#SAKNAS!
wetland	1	5110(0.011432491139819366)	#SAKNAS!	#SAKNAS!
wetting	1	5110(0.011432491139819366)	#SAKNAS!	#SAKNAS!
whale	1	5110(0.011432491139819366)	#SAKNAS!	#SAKNAS!
whales	1	2693(0.0342974734194581)	2359 (0.034)	#SAKNAS!
wheel	1	5110(0.011432491139819366)	#SAKNAS!	2548 (0.033)
whether	1	5110(0.011432491139819366)	#SAKNAS!	#SAKNAS!
whiplash	1	5110(0.011432491139819366)	2359 (0.034)	#SAKNAS!
whiplash	1	5110(0.011432491139819366)	2359 (0.034)	#SAKNAS!
white-bellied	1	5110(0.011432491139819366)	#SAKNAS!	#SAKNAS!
white-eyes	1	5110(0.011432491139819366)	#SAKNAS!	2548 (0.033)
whitefish	1	5110(0.011432491139819366)	2359 (0.034)	#SAKNAS!
whitefish	1	5110(0.011432491139819366)	2359 (0.034)	#SAKNAS!
whitefly	1	5110(0.011432491139819366)	2359 (0.034)	#SAKNAS!
whitefly	1	5110(0.011432491139819366)	2359 (0.034)	#SAKNAS!
white-tailed	1	5110(0.011432491139819366)	2359 (0.034)	#SAKNAS!
white-tailed	1	5110(0.011432491139819366)	2359 (0.034)	#SAKNAS!

whole-body	1	5110(0.011432491139819366)	#SAKNAS!	#SAKNAS!
whole-grain	1	5110(0.011432491139819366)	2359 (0.034)	#SAKNAS!
whole-grain	1	5110(0.011432491139819366)	2359 (0.034)	#SAKNAS!
wichealth.org	1	5110(0.011432491139819366)	2359 (0.034)	#SAKNAS!
wichealth.org	1	5110(0.011432491139819366)	2359 (0.034)	#SAKNAS!
widespread	1	5110(0.011432491139819366)	#SAKNAS!	#SAKNAS!
wiedemann	1	5110(0.011432491139819366)	2359 (0.034)	#SAKNAS!
wiedemann	1	5110(0.011432491139819366)	2359 (0.034)	#SAKNAS!
wild-type	1	5110(0.011432491139819366)	#SAKNAS!	2548 (0.033)
willow	1	5110(0.011432491139819366)	2359 (0.034)	#SAKNAS!
willow	1	5110(0.011432491139819366)	2359 (0.034)	#SAKNAS!
willow-feeding	1	5110(0.011432491139819366)	2359 (0.034)	#SAKNAS!
willow-feeding	1	5110(0.011432491139819366)	2359 (0.034)	#SAKNAS!
wilsons	1	5110(0.011432491139819366)	2359 (0.034)	#SAKNAS!
wilsons	1	5110(0.011432491139819366)	2359 (0.034)	#SAKNAS!
window	1	5110(0.011432491139819366)	2359 (0.034)	#SAKNAS!
window	1	5110(0.011432491139819366)	2359 (0.034)	#SAKNAS!
windows	1	5110(0.011432491139819366)	2359 (0.034)	#SAKNAS!
windows	1	5110(0.011432491139819366)	2359 (0.034)	#SAKNAS!
wing-polymorphic	1	5110(0.011432491139819366)	#SAKNAS!	2548 (0.033)
winter	1	2693(0.0342974734194581)	2359 (0.034)	#SAKNAS!
wireless	1	5110(0.011432491139819366)	#SAKNAS!	#SAKNAS!
wisdom	1	3437(0.02286498227963873)	2359 (0.034)	2548 (0.033)
within-group	1	5110(0.011432491139819366)	2359 (0.034)	#SAKNAS!
within-group	1	5110(0.011432491139819366)	2359 (0.034)	#SAKNAS!
within-subject	1	5110(0.011432491139819366)	2359 (0.034)	#SAKNAS!
within-subject	1	5110(0.011432491139819366)	2359 (0.034)	#SAKNAS!
withother	1	5110(0.011432491139819366)	#SAKNAS!	2548 (0.033)
wolf	1	5110(0.011432491139819366)	2359 (0.034)	#SAKNAS!
wolf	1	5110(0.011432491139819366)	2359 (0.034)	#SAKNAS!
wolves	1	3437(0.02286498227963873)	2359 (0.034)	#SAKNAS!
women-specific	1	5110(0.011432491139819366)	#SAKNAS!	#SAKNAS!
woodlice	1	5110(0.011432491139819366)	2359 (0.034)	#SAKNAS!
woodlice	1	5110(0.011432491139819366)	2359 (0.034)	#SAKNAS!
woody	1	3437(0.02286498227963873)	2359 (0.034)	#SAKNAS!
woralth	1	5110(0.011432491139819366)	#SAKNAS!	#SAKNAS!
workforce	1	5110(0.011432491139819366)	#SAKNAS!	#SAKNAS!
workload	1	5110(0.011432491139819366)	#SAKNAS!	#SAKNAS!
workplace-based	1	5110(0.011432491139819366)	#SAKNAS!	2548 (0.033)
works	1	5110(0.011432491139819366)	#SAKNAS!	2548 (0.033)
worksite	1	5110(0.011432491139819366)	2359 (0.034)	#SAKNAS!
worksite	1	5110(0.011432491139819366)	2359 (0.034)	#SAKNAS!
workup	1	5110(0.011432491139819366)	#SAKNAS!	2548 (0.033)

worldwide	1 5110(0.011432491139819366)	2359 (0.034)	#SAKNAS!
worldwide	1 5110(0.011432491139819366)	2359 (0.034)	#SAKNAS!
worm	1 5110(0.011432491139819366)	#SAKNAS!	2548 (0.033)
worms	1 5110(0.011432491139819366)	#SAKNAS!	#SAKNAS!
worn	1 5110(0.011432491139819366)	2359 (0.034)	#SAKNAS!
worn	1 5110(0.011432491139819366)	2359 (0.034)	#SAKNAS!
worse	1 3437(0.02286498227963873)	2359 (0.034)	2548 (0.033)
worsen	1 2693(0.0342974734194581)	2359 (0.034)	2548 (0.033)
worsening	1 5110(0.011432491139819366)	#SAKNAS!	#SAKNAS!
wort	1 5110(0.011432491139819366)	#SAKNAS!	2548 (0.033)
wosnessenskii	1 5110(0.011432491139819366)	2359 (0.034)	#SAKNAS!
wosnessenskii	1 5110(0.011432491139819366)	2359 (0.034)	#SAKNAS!
wound	1 5110(0.011432491139819366)	#SAKNAS!	2548 (0.033)
wrap-ipf	1 5110(0.011432491139819366)	#SAKNAS!	2548 (0.033)
wrinkle-faced	1 5110(0.011432491139819366)	#SAKNAS!	2548 (0.033)
wrist-worn	1 5110(0.011432491139819366)	#SAKNAS!	#SAKNAS!
wroclaw	1 5110(0.011432491139819366)	#SAKNAS!	#SAKNAS!
wrong	1 5110(0.011432491139819366)	#SAKNAS!	#SAKNAS!
wurdemanni	1 5110(0.011432491139819366)	2359 (0.034)	#SAKNAS!
wurdemanni	1 5110(0.011432491139819366)	2359 (0.034)	#SAKNAS!
xanthopterygius	1 5110(0.011432491139819366)	2359 (0.034)	#SAKNAS!
xanthopterygius	1 5110(0.011432491139819366)	2359 (0.034)	#SAKNAS!
xenin	1 5110(0.011432491139819366)	#SAKNAS!	2548 (0.033)
xenopus	1 3437(0.02286498227963873)	2359 (0.034)	#SAKNAS!
xerogenic	1 5110(0.011432491139819366)	#SAKNAS!	#SAKNAS!
xerus	1 5110(0.011432491139819366)	2359 (0.034)	#SAKNAS!
xerus	1 5110(0.011432491139819366)	2359 (0.034)	#SAKNAS!
xiamen	1 5110(0.011432491139819366)	#SAKNAS!	#SAKNAS!
xiangyun	1 5110(0.011432491139819366)	#SAKNAS!	#SAKNAS!
xinjiang	1 5110(0.011432491139819366)	#SAKNAS!	2548 (0.033)
xiphias	1 5110(0.011432491139819366)	#SAKNAS!	#SAKNAS!
xq25.1-27.2	1 5110(0.011432491139819366)	#SAKNAS!	2548 (0.033)
xq26.1-27.3	1 5110(0.011432491139819366)	#SAKNAS!	#SAKNAS!
x-ray	1 2693(0.0342974734194581)	2359 (0.034)	#SAKNAS!
xylem-feeding	1 5110(0.011432491139819366)	2359 (0.034)	#SAKNAS!
xylem-feeding	1 5110(0.011432491139819366)	2359 (0.034)	#SAKNAS!
xylostella	1 5110(0.011432491139819366)	#SAKNAS!	2548 (0.033)
xysticus	1 5110(0.011432491139819366)	#SAKNAS!	2548 (0.033)
y1	1 5110(0.011432491139819366)	2359 (0.034)	#SAKNAS!
y1	1 5110(0.011432491139819366)	2359 (0.034)	#SAKNAS!
y2	1 5110(0.011432491139819366)	2359 (0.034)	#SAKNAS!
y2	1 5110(0.011432491139819366)	2359 (0.034)	#SAKNAS!
y5	1 5110(0.011432491139819366)	2359 (0.034)	#SAKNAS!

y5	1	5110(0.011432491139819366)	2359 (0.034)	#SAKNAS!
yakushima	1	5110(0.011432491139819366)	#SAKNAS!	2548 (0.033)
yakutia	1	5110(0.011432491139819366)	2359 (0.034)	#SAKNAS!
yakutia	1	5110(0.011432491139819366)	2359 (0.034)	#SAKNAS!
yangpyeong	1	5110(0.011432491139819366)	#SAKNAS!	2548 (0.033)
yangtze	1	5110(0.011432491139819366)	#SAKNAS!	#SAKNAS!
yap	1	5110(0.011432491139819366)	#SAKNAS!	2548 (0.033)
yards	1	5110(0.011432491139819366)	2359 (0.034)	#SAKNAS!
yards	1	5110(0.011432491139819366)	2359 (0.034)	#SAKNAS!
year-olds	1	5110(0.011432491139819366)	2359 (0.034)	#SAKNAS!
year-olds	1	5110(0.011432491139819366)	2359 (0.034)	#SAKNAS!
years-a	1	5110(0.011432491139819366)	#SAKNAS!	#SAKNAS!
yeasts	1	3437(0.02286498227963873)	2359 (0.034)	2548 (0.033)
yellow	1	5110(0.011432491139819366)	#SAKNAS!	2548 (0.033)
yellow-cheeked	1	5110(0.011432491139819366)	#SAKNAS!	2548 (0.033)
yellow-shouldered	1	5110(0.011432491139819366)	2359 (0.034)	#SAKNAS!
yellow-shouldered	1	5110(0.011432491139819366)	2359 (0.034)	#SAKNAS!
yemen	1	5110(0.011432491139819366)	#SAKNAS!	2548 (0.033)
yesterday	1	3437(0.02286498227963873)	2359 (0.034)	2548 (0.033)
yielding	1	5110(0.011432491139819366)	2359 (0.034)	#SAKNAS!
yielding	1	5110(0.011432491139819366)	2359 (0.034)	#SAKNAS!
ykl-40	1	5110(0.011432491139819366)	#SAKNAS!	#SAKNAS!
y-like	1	5110(0.011432491139819366)	2359 (0.034)	#SAKNAS!
y-like	1	5110(0.011432491139819366)	2359 (0.034)	#SAKNAS!
yoghurt	1	5110(0.011432491139819366)	#SAKNAS!	#SAKNAS!
yogurt	1	2693(0.0342974734194581)	2359 (0.034)	1668 (0.066)
yoking	1	5110(0.011432491139819366)	2359 (0.034)	#SAKNAS!
yoking	1	5110(0.011432491139819366)	2359 (0.034)	#SAKNAS!
yolk	1	5110(0.011432491139819366)	#SAKNAS!	2548 (0.033)
york	1	5110(0.011432491139819366)	#SAKNAS!	2548 (0.033)
your	1	3437(0.02286498227963873)	2359 (0.034)	2548 (0.033)
yukmijihwang-tang	1	5110(0.011432491139819366)	#SAKNAS!	2548 (0.033)
yy	1	3437(0.02286498227963873)	2359 (0.034)	2548 (0.033)
zagreb	1	5110(0.011432491139819366)	#SAKNAS!	2548 (0.033)
zaire	1	5110(0.011432491139819366)	2359 (0.034)	#SAKNAS!
zaire	1	5110(0.011432491139819366)	2359 (0.034)	#SAKNAS!
zealanders	1	5110(0.011432491139819366)	#SAKNAS!	#SAKNAS!
zebra	1	3437(0.02286498227963873)	2359 (0.034)	2548 (0.033)
zebras	1	5110(0.011432491139819366)	#SAKNAS!	2548 (0.033)
zenkers	1	5110(0.011432491139819366)	2359 (0.034)	#SAKNAS!
zenkers	1	5110(0.011432491139819366)	2359 (0.034)	#SAKNAS!
zero	1	5110(0.011432491139819366)	2359 (0.034)	#SAKNAS!
zero	1	5110(0.011432491139819366)	2359 (0.034)	#SAKNAS!

zero-in-flated	1	5110(0.011432491139819366)	#SAKNAS!	2548 (0.033)
zhejiang	1	5110(0.011432491139819366)	#SAKNAS!	2548 (0.033)
zimbabwe	1	2693(0.0342974734194581)	2359 (0.034)	#SAKNAS!
zingiber	1	5110(0.011432491139819366)	#SAKNAS!	#SAKNAS!
zirconia	1	5110(0.011432491139819366)	#SAKNAS!	#SAKNAS!
zoeae	1	5110(0.011432491139819366)	2359 (0.034)	#SAKNAS!
zoeae	1	5110(0.011432491139819366)	2359 (0.034)	#SAKNAS!
zona	1	5110(0.011432491139819366)	#SAKNAS!	2548 (0.033)
zone	1	5110(0.011432491139819366)	#SAKNAS!	2548 (0.033)
zoophytophagy	1	5110(0.011432491139819366)	2359 (0.034)	#SAKNAS!
zoophytophagy	1	5110(0.011432491139819366)	2359 (0.034)	#SAKNAS!
zosterops	1	5110(0.011432491139819366)	#SAKNAS!	2548 (0.033)
z-score	1	5110(0.011432491139819366)	#SAKNAS!	#SAKNAS!
z-scores	1	5110(0.011432491139819366)	#SAKNAS!	2548 (0.033)
zucker	1	5110(0.011432491139819366)	2359 (0.034)	#SAKNAS!
zucker	1	5110(0.011432491139819366)	2359 (0.034)	#SAKNAS!
zurich	1	5110(0.011432491139819366)	2359 (0.034)	#SAKNAS!
zurich	1	5110(0.011432491139819366)	2359 (0.034)	#SAKNAS!

aning and
processing
anking,

21-24 R (%)
1 (17)
2 (16)
3 (14)
12 (7.8)
4 (13)
11 (8)
5 (11)
10 (8.2)
13 (7.6)
7 (10)
17 (6.5)
6 (10)
15 (6.8)
14 (7.4)
20 (5.3)
21 (5.1)
9 (8.3)
18 (6.4)
22 (4.9)
16 (6.5)
23 (4.7)
8 (10)
19 (5.3)
23 (4.7)
12 (7.8)
40 (2.9)
25 (4.2)
2 (16)
26 (4.1)
31 (3.5)
30 (3.6)
28 (3.9)
1 (17)
3 (14)
37 (3.2)
35 (3.3)
26 (4.1)

40 (2.9)
11 (8)
51 (2.4)
31 (3.5)
34 (3.4)
43 (2.7)
33 (3.4)
29 (3.9)
39 (2.9)
20 (5.3)
69 (1.8)
10 (8.2)
46 (2.6)
13 (7.6)
21 (5.1)
38 (3.1)
46 (2.6)
54 (2.2)
51 (2.4)
101 (1.3)
61 (2.1)
17 (6.5)
67 (1.9)
53 (2.3)
49 (2.5)
56 (2.2)
48 (2.5)
42 (2.7)
64 (2)
98 (1.4)
57 (2.1)
64 (2)
5 (11)
54 (2.2)
43 (2.7)
69 (1.8)
57 (2.1)
15 (6.8)
108 (1.2)
45 (2.6)
57 (2.1)
57 (2.1)
105 (1.3)

23 (4.7)
14 (7.4)
36 (3.2)
22 (4.9)
73 (1.8)
103 (1.3)
89 (1.5)
92 (1.5)
50 (2.5)
75 (1.8)
69 (1.8)
62 (2)
73 (1.8)
148 (0.92)
69 (1.8)
79 (1.6)
114 (1.2)
96 (1.4)
4 (13)
78 (1.7)
173 (0.78)
84 (1.6)
92 (1.5)
84 (1.6)
114 (1.2)
105 (1.3)
6 (10)
80 (1.6)
66 (2)
23 (4.7)
80 (1.6)
75 (1.8)
19 (5.3)
137 (1)
114 (1.2)
18 (6.4)
108 (1.2)
89 (1.5)
128 (1.1)
84 (1.6)
92 (1.5)
40 (2.9)
128 (1.1)

108 (1.2)
101 (1.3)
7 (10)
209 (0.67)
80 (1.6)
31 (3.5)
141 (1)
101 (1.3)
92 (1.5)
137 (1)
98 (1.4)
16 (6.5)
137 (1)
84 (1.6)
62 (2)
108 (1.2)
114 (1.2)
89 (1.5)
159 (0.85)
121 (1.1)
80 (1.6)
37 (3.2)
121 (1.1)
173 (0.78)
98 (1.4)
96 (1.4)
84 (1.6)
243 (0.6)
121 (1.1)
30 (3.6)
165 (0.82)
173 (0.78)
108 (1.2)
132 (1.1)
114 (1.2)
209 (0.67)
198 (0.71)
132 (1.1)
282 (0.53)
69 (1.8)
198 (0.71)
187 (0.75)
40 (2.9)

51 (2.4)
103 (1.3)
128 (1.1)
142 (1)
98 (1.4)
148 (0.92)
114 (1.2)
9 (8.3)
28 (3.9)
75 (1.8)
148 (0.92)
773 (0.18)
108 (1.2)
25 (4.2)
121 (1.1)
108 (1.2)
198 (0.71)
152 (0.89)
121 (1.1)
173 (0.78)
148 (0.92)
165 (0.82)
121 (1.1)
64 (2)
152 (0.89)
165 (0.82)
282 (0.53)
506 (0.28)
152 (0.89)
61 (2.1)
132 (1.1)
198 (0.71)
152 (0.89)
282 (0.53)
187 (0.75)
159 (0.85)
121 (1.1)
225 (0.64)
225 (0.64)
67 (1.9)
225 (0.64)
173 (0.78)
51 (2.4)

142 (1)
209 (0.67)
173 (0.78)
137 (1)
173 (0.78)
198 (0.71)
152 (0.89)
225 (0.64)
173 (0.78)
411 (0.36)
159 (0.85)
187 (0.75)
225 (0.64)
43 (2.7)
26 (4.1)
148 (0.92)
54 (2.2)
173 (0.78)
105 (1.3)
341 (0.43)
105 (1.3)
165 (0.82)
282 (0.53)
34 (3.4)
165 (0.82)
173 (0.78)
53 (2.3)
114 (1.2)
187 (0.75)
165 (0.82)
319 (0.46)
69 (1.8)
319 (0.46)
49 (2.5)
243 (0.6)
370 (0.39)
209 (0.67)
64 (2)
159 (0.85)
56 (2.2)
152 (0.89)
198 (0.71)
209 (0.67)

54 (2.2)
225 (0.64)
46 (2.6)
173 (0.78)
341 (0.43)
319 (0.46)
209 (0.67)
67 (1.9)
198 (0.71)
165 (0.82)
261 (0.57)
142 (1)
159 (0.85)
187 (0.75)
261 (0.57)
57 (2.1)
187 (0.75)
26 (4.1)
282 (0.53)
39 (2.9)
114 (1.2)
446 (0.32)
73 (1.8)
173 (0.78)
261 (0.57)
33 (3.4)
142 (1)
773 (0.18)
187 (0.75)
301 (0.5)
57 (2.1)
209 (0.67)
35 (3.3)
282 (0.53)
114 (1.2)
142 (1)
446 (0.32)
92 (1.5)
187 (0.75)
225 (0.64)
282 (0.53)
43 (2.7)
301 (0.5)

31 (3.5)
57 (2.1)
261 (0.57)
159 (0.85)
319 (0.46)
261 (0.57)
243 (0.6)
243 (0.6)
73 (1.8)
301 (0.5)
261 (0.57)
198 (0.71)
137 (1)
128 (1.1)
48 (2.5)
209 (0.67)
225 (0.64)
341 (0.43)
261 (0.57)
225 (0.64)
225 (0.64)
225 (0.64)
142 (1)
187 (0.75)
225 (0.64)
282 (0.53)
773 (0.18)
301 (0.5)
198 (0.71)
243 (0.6)
561 (0.25)
243 (0.6)
152 (0.89)
198 (0.71)
506 (0.28)
561 (0.25)
243 (0.6)
105 (1.3)
561 (0.25)
370 (0.39)
446 (0.32)
446 (0.32)
243 (0.6)

225 (0.64)
261 (0.57)
446 (0.32)
243 (0.6)
341 (0.43)
411 (0.36)
341 (0.43)
261 (0.57)
370 (0.39)
936 (0.14)
225 (0.64)
506 (0.28)
132 (1.1)
446 (0.32)
370 (0.39)
341 (0.43)
370 (0.39)
225 (0.64)
301 (0.5)
79 (1.6)
411 (0.36)
173 (0.78)
165 (0.82)
319 (0.46)
225 (0.64)
648 (0.21)
370 (0.39)
773 (0.18)
319 (0.46)
319 (0.46)
341 (0.43)
243 (0.6)
209 (0.67)
506 (0.28)
261 (0.57)
506 (0.28)
198 (0.71)
84 (1.6)
38 (3.1)
187 (0.75)
411 (0.36)
243 (0.6)
209 (0.67)

648 (0.21)
57 (2.1)
243 (0.6)
282 (0.53)
173 (0.78)
128 (1.1)
114 (1.2)
243 (0.6)
75 (1.8)
341 (0.43)
370 (0.39)
45 (2.6)
341 (0.43)
411 (0.36)
506 (0.28)
209 (0.67)
506 (0.28)
132 (1.1)
282 (0.53)
198 (0.71)
1164 (0.11)
92 (1.5)
773 (0.18)
506 (0.28)
446 (0.32)
282 (0.53)
1164 (0.11)
411 (0.36)
319 (0.46)
282 (0.53)
411 (0.36)
173 (0.78)
341 (0.43)
370 (0.39)
225 (0.64)
103 (1.3)
243 (0.6)
282 (0.53)
370 (0.39)
561 (0.25)
69 (1.8)
370 (0.39)
370 (0.39)

648 (0.21)
446 (0.32)
411 (0.36)
936 (0.14)
446 (0.32)
370 (0.39)
78 (1.7)
319 (0.46)
446 (0.32)
187 (0.75)
282 (0.53)
648 (0.21)
261 (0.57)
209 (0.67)
159 (0.85)
50 (2.5)
936 (0.14)
370 (0.39)
209 (0.67)
261 (0.57)
301 (0.5)
282 (0.53)
1164 (0.11)
282 (0.53)
301 (0.5)
282 (0.53)
936 (0.14)
648 (0.21)
84 (1.6)
561 (0.25)
108 (1.2)
648 (0.21)
301 (0.5)
46 (2.6)
209 (0.67)
319 (0.46)
561 (0.25)
936 (0.14)
301 (0.5)
96 (1.4)
2395 (0.036)
301 (0.5)
446 (0.32)

80 (1.6)
370 (0.39)
261 (0.57)
341 (0.43)
301 (0.5)
446 (0.32)
128 (1.1)
261 (0.57)
89 (1.5)
341 (0.43)
506 (0.28)
446 (0.32)
173 (0.78)
446 (0.32)
411 (0.36)
446 (0.32)
243 (0.6)
446 (0.32)
209 (0.67)
411 (0.36)
198 (0.71)
370 (0.39)
773 (0.18)
446 (0.32)
282 (0.53)
561 (0.25)
561 (0.25)
42 (2.7)
243 (0.6)
243 (0.6)
341 (0.43)
648 (0.21)
370 (0.39)
648 (0.21)
282 (0.53)
319 (0.46)
411 (0.36)
561 (0.25)
561 (0.25)
319 (0.46)
261 (0.57)
446 (0.32)
506 (0.28)

137 (1)
301 (0.5)
341 (0.43)
370 (0.39)
370 (0.39)
301 (0.5)
506 (0.28)
648 (0.21)
411 (0.36)
137 (1)
261 (0.57)
261 (0.57)
648 (0.21)
261 (0.57)
936 (0.14)
261 (0.57)
319 (0.46)
446 (0.32)
319 (0.46)
341 (0.43)
446 (0.32)
341 (0.43)
648 (0.21)
66 (2)
446 (0.32)
261 (0.57)
648 (0.21)
411 (0.36)
319 (0.46)
80 (1.6)
1573 (0.071)
561 (0.25)
411 (0.36)
319 (0.46)
561 (0.25)
561 (0.25)
370 (0.39)
98 (1.4)
341 (0.43)
411 (0.36)
446 (0.32)
446 (0.32)
773 (0.18)

1573 (0.071)
141 (1)
446 (0.32)
370 (0.39)
319 (0.46)
936 (0.14)
370 (0.39)
936 (0.14)
165 (0.82)
121 (1.1)
341 (0.43)
446 (0.32)
370 (0.39)
773 (0.18)
773 (0.18)
1164 (0.11)
152 (0.89)
446 (0.32)
341 (0.43)
411 (0.36)
319 (0.46)
446 (0.32)
198 (0.71)
370 (0.39)
561 (0.25)
#SAKNAS!
370 (0.39)
108 (1.2)
648 (0.21)
648 (0.21)
411 (0.36)
243 (0.6)
187 (0.75)
648 (0.21)
648 (0.21)
648 (0.21)
319 (0.46)
301 (0.5)
225 (0.64)
1164 (0.11)
506 (0.28)
561 (0.25)
411 (0.36)

341 (0.43)
108 (1.2)
142 (1)
370 (0.39)
69 (1.8)
506 (0.28)
648 (0.21)
101 (1.3)
773 (0.18)
411 (0.36)
1164 (0.11)
648 (0.21)
121 (1.1)
561 (0.25)
506 (0.28)
370 (0.39)
114 (1.2)
92 (1.5)
561 (0.25)
446 (0.32)
411 (0.36)
209 (0.67)
446 (0.32)
773 (0.18)
411 (0.36)
2395 (0.036)
121 (1.1)
411 (0.36)
282 (0.53)
301 (0.5)
341 (0.43)
1164 (0.11)
936 (0.14)
446 (0.32)
114 (1.2)
29 (3.9)
165 (0.82)
506 (0.28)
1573 (0.071)
#SAKNAS!
936 (0.14)
773 (0.18)
341 (0.43)

936 (0.14)
225 (0.64)
319 (0.46)
506 (0.28)
198 (0.71)
261 (0.57)
936 (0.14)
648 (0.21)
370 (0.39)
209 (0.67)
341 (0.43)
446 (0.32)
446 (0.32)
561 (0.25)
128 (1.1)
561 (0.25)
209 (0.67)
319 (0.46)
62 (2)
773 (0.18)
446 (0.32)
108 (1.2)
187 (0.75)
92 (1.5)
341 (0.43)
1164 (0.11)
506 (0.28)
#SAKNAS!
648 (0.21)
370 (0.39)
1164 (0.11)
773 (0.18)
341 (0.43)
648 (0.21)
96 (1.4)
936 (0.14)
561 (0.25)
80 (1.6)
648 (0.21)
561 (0.25)
173 (0.78)
561 (0.25)
411 (0.36)

411 (0.36)
148 (0.92)
648 (0.21)
411 (0.36)
411 (0.36)
75 (1.8)
#SAKNAS!
506 (0.28)
561 (0.25)
648 (0.21)
506 (0.28)
370 (0.39)
936 (0.14)
173 (0.78)
936 (0.14)
506 (0.28)
936 (0.14)
152 (0.89)
301 (0.5)
121 (1.1)
773 (0.18)
506 (0.28)
773 (0.18)
648 (0.21)
209 (0.67)
506 (0.28)
319 (0.46)
198 (0.71)
561 (0.25)
370 (0.39)
648 (0.21)
301 (0.5)
773 (0.18)
506 (0.28)
936 (0.14)
648 (0.21)
648 (0.21)
446 (0.32)
506 (0.28)
319 (0.46)
370 (0.39)
411 (0.36)
648 (0.21)

225 (0.64)
561 (0.25)
773 (0.18)
773 (0.18)
1573 (0.071)
319 (0.46)
936 (0.14)
341 (0.43)
173 (0.78)
773 (0.18)
1164 (0.11)
1164 (0.11)
1164 (0.11)
261 (0.57)
84 (1.6)
773 (0.18)
773 (0.18)
446 (0.32)
1164 (0.11)
773 (0.18)
561 (0.25)
132 (1.1)
506 (0.28)
936 (0.14)
561 (0.25)
446 (0.32)
648 (0.21)
561 (0.25)
1164 (0.11)
411 (0.36)
506 (0.28)
936 (0.14)
370 (0.39)
506 (0.28)
648 (0.21)
773 (0.18)
370 (0.39)
936 (0.14)
936 (0.14)
561 (0.25)
773 (0.18)
1164 (0.11)
152 (0.89)

446 (0.32)
773 (0.18)
773 (0.18)
301 (0.5)
411 (0.36)
648 (0.21)
114 (1.2)
370 (0.39)
561 (0.25)
561 (0.25)
173 (0.78)
561 (0.25)
1573 (0.071)
506 (0.28)
561 (0.25)
1573 (0.071)
411 (0.36)
506 (0.28)
936 (0.14)
225 (0.64)
773 (0.18)
561 (0.25)
446 (0.32)
506 (0.28)
648 (0.21)
506 (0.28)
#SAKNAS!
142 (1)
411 (0.36)
411 (0.36)
648 (0.21)
89 (1.5)
370 (0.39)
561 (0.25)
1164 (0.11)
1164 (0.11)
209 (0.67)
132 (1.1)
1164 (0.11)
648 (0.21)
1164 (0.11)
370 (0.39)
209 (0.67)

1573 (0.071)
446 (0.32)
411 (0.36)
936 (0.14)
561 (0.25)
301 (0.5)
370 (0.39)
446 (0.32)
506 (0.28)
1164 (0.11)
773 (0.18)
773 (0.18)
648 (0.21)
1164 (0.11)
341 (0.43)
506 (0.28)
648 (0.21)
137 (1)
936 (0.14)
561 (0.25)
936 (0.14)
561 (0.25)
648 (0.21)
225 (0.64)
1164 (0.11)
773 (0.18)
648 (0.21)
506 (0.28)
225 (0.64)
319 (0.46)
165 (0.82)
936 (0.14)
773 (0.18)
36 (3.2)
561 (0.25)
936 (0.14)
84 (1.6)
1164 (0.11)
936 (0.14)
1164 (0.11)
173 (0.78)
936 (0.14)
370 (0.39)

773 (0.18)
370 (0.39)
648 (0.21)
648 (0.21)
446 (0.32)
561 (0.25)
446 (0.32)
108 (1.2)
561 (0.25)
936 (0.14)
936 (0.14)
648 (0.21)
243 (0.6)
648 (0.21)
446 (0.32)
506 (0.28)
1573 (0.071)
648 (0.21)
561 (0.25)
446 (0.32)
561 (0.25)
648 (0.21)
370 (0.39)
1164 (0.11)
198 (0.71)
209 (0.67)
446 (0.32)
561 (0.25)
648 (0.21)
773 (0.18)
773 (0.18)
446 (0.32)
936 (0.14)
148 (0.92)
936 (0.14)
341 (0.43)
773 (0.18)
561 (0.25)
561 (0.25)
773 (0.18)
446 (0.32)
773 (0.18)
561 (0.25)

773 (0.18)
243 (0.6)
561 (0.25)
411 (0.36)
561 (0.25)
370 (0.39)
173 (0.78)
341 (0.43)
446 (0.32)
773 (0.18)
411 (0.36)
446 (0.32)
1573 (0.071)
282 (0.53)
936 (0.14)
648 (0.21)
561 (0.25)
936 (0.14)
648 (0.21)
561 (0.25)
187 (0.75)
936 (0.14)
506 (0.28)
648 (0.21)
80 (1.6)
936 (0.14)
773 (0.18)
209 (0.67)
648 (0.21)
506 (0.28)
301 (0.5)
243 (0.6)
2395 (0.036)
370 (0.39)
446 (0.32)
282 (0.53)
773 (0.18)
773 (0.18)
173 (0.78)
773 (0.18)
446 (0.32)
446 (0.32)
936 (0.14)

773 (0.18)
187 (0.75)
1164 (0.11)
261 (0.57)
773 (0.18)
648 (0.21)
446 (0.32)
773 (0.18)
773 (0.18)
648 (0.21)
561 (0.25)
773 (0.18)
370 (0.39)
648 (0.21)
1573 (0.071)
446 (0.32)
648 (0.21)
506 (0.28)
936 (0.14)
773 (0.18)
773 (0.18)
#SAKNAS!
1164 (0.11)
446 (0.32)
936 (0.14)
936 (0.14)
561 (0.25)
411 (0.36)
1164 (0.11)
261 (0.57)
152 (0.89)
370 (0.39)
165 (0.82)
936 (0.14)
#SAKNAS!
#SAKNAS!
506 (0.28)
1164 (0.11)
1573 (0.071)
506 (0.28)
446 (0.32)
936 (0.14)
561 (0.25)

561 (0.25)
1164 (0.11)
446 (0.32)
773 (0.18)
341 (0.43)
561 (0.25)
648 (0.21)
1573 (0.071)
648 (0.21)
301 (0.5)
341 (0.43)
648 (0.21)
648 (0.21)
773 (0.18)
1164 (0.11)
506 (0.28)
561 (0.25)
936 (0.14)
152 (0.89)
561 (0.25)
370 (0.39)
773 (0.18)
341 (0.43)
561 (0.25)
506 (0.28)
187 (0.75)
773 (0.18)
561 (0.25)
936 (0.14)
936 (0.14)
446 (0.32)
648 (0.21)
1164 (0.11)
282 (0.53)
561 (0.25)
341 (0.43)
1164 (0.11)
936 (0.14)
1164 (0.11)
1573 (0.071)
648 (0.21)
225 (0.64)
506 (0.28)

773 (0.18)
561 (0.25)
159 (0.85)
936 (0.14)
261 (0.57)
773 (0.18)
261 (0.57)
187 (0.75)
506 (0.28)
319 (0.46)
561 (0.25)
936 (0.14)
561 (0.25)
225 (0.64)
936 (0.14)
648 (0.21)
1573 (0.071)
936 (0.14)
282 (0.53)
506 (0.28)
506 (0.28)
198 (0.71)
446 (0.32)
648 (0.21)
506 (0.28)
648 (0.21)
773 (0.18)
506 (0.28)
370 (0.39)
370 (0.39)
1164 (0.11)
446 (0.32)
411 (0.36)
506 (0.28)
936 (0.14)
446 (0.32)
1573 (0.071)
1573 (0.071)
561 (0.25)
1164 (0.11)
1573 (0.071)
506 (0.28)
1164 (0.11)

561 (0.25)
773 (0.18)
561 (0.25)
319 (0.46)
411 (0.36)
936 (0.14)
648 (0.21)
282 (0.53)
1164 (0.11)
561 (0.25)
1164 (0.11)
936 (0.14)
370 (0.39)
2395 (0.036)
773 (0.18)
2395 (0.036)
1164 (0.11)
446 (0.32)
243 (0.6)
1164 (0.11)
773 (0.18)
648 (0.21)
225 (0.64)
561 (0.25)
773 (0.18)
773 (0.18)
648 (0.21)
648 (0.21)
173 (0.78)
773 (0.18)
243 (0.6)
773 (0.18)
936 (0.14)
773 (0.18)
936 (0.14)
1164 (0.11)
1573 (0.071)
506 (0.28)
282 (0.53)
936 (0.14)
1573 (0.071)
446 (0.32)
936 (0.14)

301 (0.5)
936 (0.14)
1164 (0.11)
446 (0.32)
773 (0.18)
936 (0.14)
936 (0.14)
648 (0.21)
506 (0.28)
936 (0.14)
648 (0.21)
446 (0.32)
341 (0.43)
936 (0.14)
121 (1.1)
1164 (0.11)
648 (0.21)
1573 (0.071)
165 (0.82)
1164 (0.11)
936 (0.14)
1164 (0.11)
773 (0.18)
506 (0.28)
209 (0.67)
370 (0.39)
936 (0.14)
1573 (0.071)
2395 (0.036)
773 (0.18)
936 (0.14)
936 (0.14)
936 (0.14)
773 (0.18)
446 (0.32)
1164 (0.11)
648 (0.21)
648 (0.21)
506 (0.28)
936 (0.14)
648 (0.21)
936 (0.14)
773 (0.18)

2395 (0.036)
936 (0.14)
1164 (0.11)
121 (1.1)
648 (0.21)
2395 (0.036)
648 (0.21)
936 (0.14)
2395 (0.036)
648 (0.21)
648 (0.21)
936 (0.14)
1573 (0.071)
1164 (0.11)
561 (0.25)
446 (0.32)
2395 (0.036)
506 (0.28)
648 (0.21)
2395 (0.036)
561 (0.25)
411 (0.36)
1164 (0.11)
648 (0.21)
773 (0.18)
773 (0.18)
648 (0.21)
1164 (0.11)
159 (0.85)
648 (0.21)
648 (0.21)
561 (0.25)
773 (0.18)
370 (0.39)
773 (0.18)
1164 (0.11)
1164 (0.11)
8 (10)
1164 (0.11)
1164 (0.11)
561 (0.25)
1164 (0.11)
506 (0.28)

648 (0.21)
1164 (0.11)
1573 (0.071)
2395 (0.036)
773 (0.18)
282 (0.53)
319 (0.46)
648 (0.21)
1164 (0.11)
1573 (0.071)
773 (0.18)
936 (0.14)
446 (0.32)
2395 (0.036)
936 (0.14)
773 (0.18)
243 (0.6)
773 (0.18)
1573 (0.071)
1164 (0.11)
936 (0.14)
936 (0.14)
2395 (0.036)
648 (0.21)
936 (0.14)
1164 (0.11)
773 (0.18)
773 (0.18)
1573 (0.071)
506 (0.28)
773 (0.18)
648 (0.21)
446 (0.32)
1573 (0.071)
1573 (0.071)
1573 (0.071)
282 (0.53)
1164 (0.11)
#SAKNAS!
648 (0.21)
648 (0.21)
773 (0.18)
936 (0.14)

1573 (0.071)
1164 (0.11)
1164 (0.11)
2395 (0.036)
2395 (0.036)
1164 (0.11)
198 (0.71)
1573 (0.071)
936 (0.14)
561 (0.25)
1573 (0.071)
1164 (0.11)
142 (1)
2395 (0.036)
936 (0.14)
1164 (0.11)
648 (0.21)
1164 (0.11)
2395 (0.036)
187 (0.75)
936 (0.14)
936 (0.14)
648 (0.21)
1164 (0.11)
561 (0.25)
773 (0.18)
#SAKNAS!
773 (0.18)
1573 (0.071)
1164 (0.11)
773 (0.18)
936 (0.14)
936 (0.14)
341 (0.43)
370 (0.39)
1573 (0.071)
936 (0.14)
936 (0.14)
936 (0.14)
341 (0.43)
648 (0.21)
561 (0.25)
282 (0.53)

936 (0.14)
773 (0.18)
#SAKNAS!
773 (0.18)
773 (0.18)
506 (0.28)
648 (0.21)
1164 (0.11)
936 (0.14)
773 (0.18)
1164 (0.11)
1164 (0.11)
225 (0.64)
648 (0.21)
936 (0.14)
648 (0.21)
773 (0.18)
648 (0.21)
773 (0.18)
411 (0.36)
561 (0.25)
261 (0.57)
648 (0.21)
1164 (0.11)
1164 (0.11)
1164 (0.11)
1573 (0.071)
506 (0.28)
209 (0.67)
103 (1.3)
773 (0.18)
1573 (0.071)
936 (0.14)
1573 (0.071)
2395 (0.036)
243 (0.6)
648 (0.21)
1573 (0.071)
936 (0.14)
446 (0.32)
1573 (0.071)
648 (0.21)
1164 (0.11)

936 (0.14)
936 (0.14)
648 (0.21)
561 (0.25)
773 (0.18)
243 (0.6)
282 (0.53)
2395 (0.036)
561 (0.25)
173 (0.78)
62 (2)
1573 (0.071)
#SAKNAS!
2395 (0.036)
1573 (0.071)
2395 (0.036)
2395 (0.036)
773 (0.18)
446 (0.32)
936 (0.14)
648 (0.21)
773 (0.18)
411 (0.36)
561 (0.25)
773 (0.18)
561 (0.25)
341 (0.43)
370 (0.39)
648 (0.21)
773 (0.18)
773 (0.18)
225 (0.64)
1164 (0.11)
936 (0.14)
1164 (0.11)
2395 (0.036)
561 (0.25)
561 (0.25)
648 (0.21)
773 (0.18)
1573 (0.071)
561 (0.25)
2395 (0.036)

446 (0.32)
936 (0.14)
#SAKNAS!
#SAKNAS!
1164 (0.11)
2395 (0.036)
411 (0.36)
1164 (0.11)
648 (0.21)
187 (0.75)
936 (0.14)
936 (0.14)
225 (0.64)
936 (0.14)
936 (0.14)
773 (0.18)
506 (0.28)
773 (0.18)
561 (0.25)
648 (0.21)
936 (0.14)
773 (0.18)
2395 (0.036)
1164 (0.11)
1164 (0.11)
773 (0.18)
1573 (0.071)
1164 (0.11)
648 (0.21)
1573 (0.071)
773 (0.18)
936 (0.14)
561 (0.25)
1164 (0.11)
936 (0.14)
1573 (0.071)
561 (0.25)
1164 (0.11)
1573 (0.071)
#SAKNAS!
1164 (0.11)
165 (0.82)
1573 (0.071)

936 (0.14)
1164 (0.11)
561 (0.25)
1164 (0.11)
1573 (0.071)
936 (0.14)
773 (0.18)
1164 (0.11)
1573 (0.071)
648 (0.21)
1573 (0.071)
936 (0.14)
561 (0.25)
936 (0.14)
411 (0.36)
225 (0.64)
1164 (0.11)
648 (0.21)
648 (0.21)
1164 (0.11)
1573 (0.071)
648 (0.21)
#SAKNAS!
1164 (0.11)
2395 (0.036)
936 (0.14)
2395 (0.036)
198 (0.71)
#SAKNAS!
1164 (0.11)
446 (0.32)
1164 (0.11)
152 (0.89)
648 (0.21)
561 (0.25)
936 (0.14)
648 (0.21)
936 (0.14)
1164 (0.11)
936 (0.14)
1164 (0.11)
1573 (0.071)
1164 (0.11)

936 (0.14)
936 (0.14)
561 (0.25)
1573 (0.071)
1164 (0.11)
1164 (0.11)
773 (0.18)
936 (0.14)
936 (0.14)
1164 (0.11)
#SAKNAS!
936 (0.14)
506 (0.28)
2395 (0.036)
#SAKNAS!
1164 (0.11)
2395 (0.036)
648 (0.21)
1573 (0.071)
936 (0.14)
1164 (0.11)
#SAKNAS!
1573 (0.071)
561 (0.25)
2395 (0.036)
936 (0.14)
1164 (0.11)
648 (0.21)
773 (0.18)
1164 (0.11)
1573 (0.071)
243 (0.6)
648 (0.21)
1573 (0.071)
648 (0.21)
1164 (0.11)
1573 (0.071)
2395 (0.036)
2395 (0.036)
936 (0.14)
1573 (0.071)
1164 (0.11)
1164 (0.11)

2395 (0.036)
301 (0.5)
2395 (0.036)
1573 (0.071)
1573 (0.071)
411 (0.36)
936 (0.14)
1164 (0.11)
1164 (0.11)
2395 (0.036)
773 (0.18)
561 (0.25)
1573 (0.071)
506 (0.28)
561 (0.25)
936 (0.14)
1164 (0.11)
936 (0.14)
1164 (0.11)
936 (0.14)
773 (0.18)
1164 (0.11)
1164 (0.11)
1164 (0.11)
2395 (0.036)
648 (0.21)
1573 (0.071)
1164 (0.11)
648 (0.21)
648 (0.21)
2395 (0.036)
98 (1.4)
936 (0.14)
1164 (0.11)
648 (0.21)
1573 (0.071)
1164 (0.11)
936 (0.14)
1164 (0.11)
1573 (0.071)
1164 (0.11)
142 (1)
1164 (0.11)

1573 (0.071)
1164 (0.11)
1573 (0.071)
1573 (0.071)
773 (0.18)
159 (0.85)
370 (0.39)
2395 (0.036)
446 (0.32)
1164 (0.11)
648 (0.21)
1573 (0.071)
1164 (0.11)
1164 (0.11)
282 (0.53)
198 (0.71)
648 (0.21)
1164 (0.11)
1164 (0.11)
1164 (0.11)
1164 (0.11)
773 (0.18)
1573 (0.071)
936 (0.14)
319 (0.46)
648 (0.21)
165 (0.82)
648 (0.21)
773 (0.18)
1573 (0.071)
159 (0.85)
936 (0.14)
936 (0.14)
648 (0.21)
1164 (0.11)
773 (0.18)
561 (0.25)
1164 (0.11)
1573 (0.071)
1573 (0.071)
209 (0.67)
773 (0.18)
561 (0.25)

1164 (0.11)
370 (0.39)
1573 (0.071)
1573 (0.071)
773 (0.18)
1164 (0.11)
1164 (0.11)
446 (0.32)
2395 (0.036)
1573 (0.071)
936 (0.14)
773 (0.18)
1164 (0.11)
1573 (0.071)
1164 (0.11)
1164 (0.11)
1573 (0.071)
370 (0.39)
1164 (0.11)
773 (0.18)
1573 (0.071)
1573 (0.071)
148 (0.92)
936 (0.14)
936 (0.14)
187 (0.75)
446 (0.32)
1164 (0.11)
773 (0.18)
936 (0.14)
1573 (0.071)
506 (0.28)
#SAKNAS!
#SAKNAS!
#SAKNAS!
#SAKNAS!
#SAKNAS!
1573 (0.071)
773 (0.18)
446 (0.32)
1573 (0.071)
341 (0.43)
1573 (0.071)

#SAKNAS!
341 (0.43)
561 (0.25)
1573 (0.071)
#SAKNAS!
#SAKNAS!
1164 (0.11)
1573 (0.071)
936 (0.14)
#SAKNAS!
1573 (0.071)
773 (0.18)
370 (0.39)
1573 (0.071)
225 (0.64)
1573 (0.071)
936 (0.14)
121 (1.1)
2395 (0.036)
936 (0.14)
773 (0.18)
209 (0.67)
#SAKNAS!
370 (0.39)
648 (0.21)
936 (0.14)
1573 (0.071)
1573 (0.071)
#SAKNAS!
#SAKNAS!
#SAKNAS!
936 (0.14)
2395 (0.036)
1573 (0.071)
2395 (0.036)
1573 (0.071)
561 (0.25)
1164 (0.11)
1164 (0.11)
#SAKNAS!
1164 (0.11)
773 (0.18)
936 (0.14)

2395 (0.036)
1164 (0.11)
1164 (0.11)
648 (0.21)
1573 (0.071)
773 (0.18)
#SAKNAS!
1164 (0.11)
773 (0.18)
936 (0.14)
506 (0.28)
936 (0.14)
1164 (0.11)
936 (0.14)
319 (0.46)
773 (0.18)
#SAKNAS!
648 (0.21)
1164 (0.11)
2395 (0.036)
936 (0.14)
1164 (0.11)
2395 (0.036)
1164 (0.11)
1164 (0.11)
1164 (0.11)
648 (0.21)
1164 (0.11)
936 (0.14)
446 (0.32)
2395 (0.036)
89 (1.5)
2395 (0.036)
936 (0.14)
1573 (0.071)
1573 (0.071)
2395 (0.036)
773 (0.18)
936 (0.14)
1164 (0.11)
936 (0.14)
773 (0.18)
561 (0.25)

1573 (0.071)
648 (0.21)
2395 (0.036)
411 (0.36)
936 (0.14)
936 (0.14)
1573 (0.071)
2395 (0.036)
648 (0.21)
936 (0.14)
936 (0.14)
#SAKNAS!
1573 (0.071)
1573 (0.071)
1164 (0.11)
225 (0.64)
2395 (0.036)
2395 (0.036)
561 (0.25)
773 (0.18)
261 (0.57)
1164 (0.11)
1164 (0.11)
1573 (0.071)
561 (0.25)
561 (0.25)
1164 (0.11)
506 (0.28)
1164 (0.11)
446 (0.32)
225 (0.64)
1164 (0.11)
1164 (0.11)
411 (0.36)
198 (0.71)
1164 (0.11)
282 (0.53)
2395 (0.036)
936 (0.14)
341 (0.43)
1164 (0.11)
#SAKNAS!
243 (0.6)

1164 (0.11)
1164 (0.11)
1164 (0.11)
261 (0.57)
773 (0.18)
561 (0.25)
2395 (0.036)
2395 (0.036)
1164 (0.11)
1164 (0.11)
773 (0.18)
1573 (0.071)
446 (0.32)
1164 (0.11)
773 (0.18)
1164 (0.11)
2395 (0.036)
936 (0.14)
1573 (0.071)
506 (0.28)
159 (0.85)
#SAKNAS!
1573 (0.071)
936 (0.14)
773 (0.18)
773 (0.18)
1164 (0.11)
2395 (0.036)
319 (0.46)
2395 (0.036)
936 (0.14)
773 (0.18)
648 (0.21)
1164 (0.11)
#SAKNAS!
#SAKNAS!
561 (0.25)
1164 (0.11)
2395 (0.036)
#SAKNAS!
370 (0.39)
1164 (0.11)
261 (0.57)

341 (0.43)
773 (0.18)
#SAKNAS!
#SAKNAS!
936 (0.14)
2395 (0.036)
2395 (0.036)
1164 (0.11)
648 (0.21)
1573 (0.071)
1164 (0.11)
648 (0.21)
936 (0.14)
2395 (0.036)
1573 (0.071)
261 (0.57)
370 (0.39)
1573 (0.071)
2395 (0.036)
#SAKNAS!
2395 (0.036)
1164 (0.11)
648 (0.21)
773 (0.18)
#SAKNAS!
936 (0.14)
1573 (0.071)
2395 (0.036)
#SAKNAS!
2395 (0.036)
#SAKNAS!
261 (0.57)
1164 (0.11)
936 (0.14)
341 (0.43)
2395 (0.036)
1164 (0.11)
1164 (0.11)
#SAKNAS!
370 (0.39)
1573 (0.071)
561 (0.25)
1164 (0.11)

773 (0.18)
1164 (0.11)
446 (0.32)
1573 (0.071)
936 (0.14)
1164 (0.11)
411 (0.36)
648 (0.21)
#SAKNAS!
936 (0.14)
1573 (0.071)
1573 (0.071)
773 (0.18)
2395 (0.036)
1164 (0.11)
1164 (0.11)
#SAKNAS!
2395 (0.036)
773 (0.18)
936 (0.14)
2395 (0.036)
#SAKNAS!
411 (0.36)
561 (0.25)
1573 (0.071)
1164 (0.11)
1164 (0.11)
1573 (0.071)
1573 (0.071)
561 (0.25)
561 (0.25)
1573 (0.071)
2395 (0.036)
1164 (0.11)
2395 (0.036)
506 (0.28)
2395 (0.036)
936 (0.14)
#SAKNAS!
2395 (0.036)
2395 (0.036)
#SAKNAS!
773 (0.18)

2395 (0.036)
648 (0.21)
1573 (0.071)
936 (0.14)
1164 (0.11)
301 (0.5)
#SAKNAS!
1164 (0.11)
773 (0.18)
#SAKNAS!
773 (0.18)
773 (0.18)
1573 (0.071)
1573 (0.071)
1164 (0.11)
1573 (0.071)
936 (0.14)
773 (0.18)
936 (0.14)
1164 (0.11)
1164 (0.11)
936 (0.14)
2395 (0.036)
1164 (0.11)
301 (0.5)
1573 (0.071)
1164 (0.11)
1164 (0.11)
1573 (0.071)
261 (0.57)
773 (0.18)
936 (0.14)
773 (0.18)
1573 (0.071)
773 (0.18)
506 (0.28)
648 (0.21)
773 (0.18)
2395 (0.036)
1573 (0.071)
1164 (0.11)
773 (0.18)
773 (0.18)

648 (0.21)
2395 (0.036)
773 (0.18)
1573 (0.071)
2395 (0.036)
773 (0.18)
773 (0.18)
1164 (0.11)
1164 (0.11)
#SAKNAS!
319 (0.46)
282 (0.53)
1573 (0.071)
1573 (0.071)
506 (0.28)
1164 (0.11)
2395 (0.036)
2395 (0.036)
561 (0.25)
561 (0.25)
1164 (0.11)
561 (0.25)
648 (0.21)
1164 (0.11)
2395 (0.036)
#SAKNAS!
773 (0.18)
2395 (0.036)
2395 (0.036)
773 (0.18)
936 (0.14)
1164 (0.11)
1573 (0.071)
1164 (0.11)
773 (0.18)
341 (0.43)
1164 (0.11)
773 (0.18)
#SAKNAS!
936 (0.14)
301 (0.5)
#SAKNAS!
773 (0.18)

1573 (0.071)
1164 (0.11)
1573 (0.071)
1164 (0.11)
1573 (0.071)
648 (0.21)
#SAKNAS!
1573 (0.071)
446 (0.32)
1573 (0.071)
2395 (0.036)
561 (0.25)
1164 (0.11)
2395 (0.036)
1164 (0.11)
1573 (0.071)
1164 (0.11)
648 (0.21)
936 (0.14)
1164 (0.11)
1573 (0.071)
1164 (0.11)
648 (0.21)
2395 (0.036)
1573 (0.071)
1573 (0.071)
773 (0.18)
936 (0.14)
648 (0.21)
648 (0.21)
773 (0.18)
1573 (0.071)
1573 (0.071)
2395 (0.036)
1573 (0.071)
225 (0.64)
2395 (0.036)
773 (0.18)
1164 (0.11)
370 (0.39)
1164 (0.11)
2395 (0.036)
1573 (0.071)

1164 (0.11)
1164 (0.11)
243 (0.6)
1573 (0.071)
#SAKNAS!
936 (0.14)
1164 (0.11)
1164 (0.11)
1573 (0.071)
936 (0.14)
243 (0.6)
411 (0.36)
446 (0.32)
561 (0.25)
370 (0.39)
1573 (0.071)
1573 (0.071)
2395 (0.036)
1573 (0.071)
1573 (0.071)
1164 (0.11)
#SAKNAS!
936 (0.14)
301 (0.5)
2395 (0.036)
2395 (0.036)
1573 (0.071)
936 (0.14)
2395 (0.036)
1573 (0.071)
936 (0.14)
446 (0.32)
1164 (0.11)
#SAKNAS!
561 (0.25)
#SAKNAS!
1164 (0.11)
1164 (0.11)
2395 (0.036)
2395 (0.036)
341 (0.43)
773 (0.18)
1573 (0.071)

936 (0.14)
#SAKNAS!
1573 (0.071)
261 (0.57)
#SAKNAS!
2395 (0.036)
1573 (0.071)
1573 (0.071)
936 (0.14)
648 (0.21)
2395 (0.036)
936 (0.14)
1164 (0.11)
936 (0.14)
1164 (0.11)
936 (0.14)
2395 (0.036)
#SAKNAS!
1164 (0.11)
#SAKNAS!
1164 (0.11)
1573 (0.071)
1164 (0.11)
1164 (0.11)
370 (0.39)
1164 (0.11)
1164 (0.11)
506 (0.28)
1164 (0.11)
2395 (0.036)
1573 (0.071)
2395 (0.036)
1164 (0.11)
648 (0.21)
2395 (0.036)
2395 (0.036)
209 (0.67)
282 (0.53)
1573 (0.071)
2395 (0.036)
1573 (0.071)
1164 (0.11)
#SAKNAS!

1164 (0.11)
773 (0.18)
1573 (0.071)
1573 (0.071)
319 (0.46)
936 (0.14)
506 (0.28)
#SAKNAS!
561 (0.25)
1164 (0.11)
936 (0.14)
446 (0.32)
#SAKNAS!
1164 (0.11)
261 (0.57)
2395 (0.036)
936 (0.14)
1164 (0.11)
1573 (0.071)
773 (0.18)
936 (0.14)
648 (0.21)
#SAKNAS!
411 (0.36)
2395 (0.036)
936 (0.14)
#SAKNAS!
#SAKNAS!
1164 (0.11)
1164 (0.11)
936 (0.14)
1573 (0.071)
#SAKNAS!
2395 (0.036)
1164 (0.11)
261 (0.57)
1573 (0.071)
1573 (0.071)
936 (0.14)
#SAKNAS!
1164 (0.11)
#SAKNAS!
1573 (0.071)

2395 (0.036)
773 (0.18)
936 (0.14)
2395 (0.036)
936 (0.14)
282 (0.53)
1573 (0.071)
648 (0.21)
561 (0.25)
1573 (0.071)
773 (0.18)
1164 (0.11)
1164 (0.11)
648 (0.21)
648 (0.21)
2395 (0.036)
936 (0.14)
2395 (0.036)
#SAKNAS!
936 (0.14)
132 (1.1)
2395 (0.036)
2395 (0.036)
1164 (0.11)
2395 (0.036)
1164 (0.11)
#SAKNAS!
1573 (0.071)
1164 (0.11)
1164 (0.11)
1573 (0.071)
773 (0.18)
1164 (0.11)
#SAKNAS!
1573 (0.071)
1164 (0.11)
773 (0.18)
506 (0.28)
1573 (0.071)
#SAKNAS!
1164 (0.11)
936 (0.14)
1164 (0.11)

2395 (0.036)
1573 (0.071)
319 (0.46)
936 (0.14)
1573 (0.071)
773 (0.18)
506 (0.28)
1573 (0.071)
773 (0.18)
1573 (0.071)
1164 (0.11)
1164 (0.11)
#SAKNAS!
1164 (0.11)
1164 (0.11)
1164 (0.11)
446 (0.32)
2395 (0.036)
2395 (0.036)
2395 (0.036)
1573 (0.071)
936 (0.14)
648 (0.21)
2395 (0.036)
1164 (0.11)
773 (0.18)
1573 (0.071)
773 (0.18)
411 (0.36)
1573 (0.071)
936 (0.14)
773 (0.18)
2395 (0.036)
773 (0.18)
2395 (0.036)
1573 (0.071)
1573 (0.071)
2395 (0.036)
936 (0.14)
2395 (0.036)
2395 (0.036)
#SAKNAS!
773 (0.18)

2395 (0.036)
2395 (0.036)
2395 (0.036)
1164 (0.11)
773 (0.18)
936 (0.14)
1573 (0.071)
506 (0.28)
2395 (0.036)
936 (0.14)
648 (0.21)
2395 (0.036)
1573 (0.071)
648 (0.21)
#SAKNAS!
#SAKNAS!
1573 (0.071)
561 (0.25)
#SAKNAS!
773 (0.18)
648 (0.21)
773 (0.18)
1573 (0.071)
2395 (0.036)
561 (0.25)
1573 (0.071)
648 (0.21)
936 (0.14)
1573 (0.071)
1573 (0.071)
2395 (0.036)
773 (0.18)
1573 (0.071)
1573 (0.071)
1164 (0.11)
2395 (0.036)
936 (0.14)
773 (0.18)
936 (0.14)
1164 (0.11)
1573 (0.071)
1164 (0.11)
1573 (0.071)

2395 (0.036)
1573 (0.071)
936 (0.14)
1164 (0.11)
2395 (0.036)
936 (0.14)
1573 (0.071)
1164 (0.11)
341 (0.43)
506 (0.28)
2395 (0.036)
#SAKNAS!
411 (0.36)
2395 (0.036)
773 (0.18)
561 (0.25)
243 (0.6)
936 (0.14)
#SAKNAS!
1164 (0.11)
1573 (0.071)
1573 (0.071)
#SAKNAS!
648 (0.21)
2395 (0.036)
446 (0.32)
936 (0.14)
648 (0.21)
446 (0.32)
2395 (0.036)
2395 (0.036)
936 (0.14)
#SAKNAS!
773 (0.18)
#SAKNAS!
370 (0.39)
2395 (0.036)
2395 (0.036)
1164 (0.11)
1573 (0.071)
936 (0.14)
#SAKNAS!
2395 (0.036)

411 (0.36)
1573 (0.071)
1573 (0.071)
2395 (0.036)
2395 (0.036)
936 (0.14)
1573 (0.071)
2395 (0.036)
648 (0.21)
301 (0.5)
1573 (0.071)
2395 (0.036)
#SAKNAS!
561 (0.25)
2395 (0.036)
936 (0.14)
2395 (0.036)
1164 (0.11)
2395 (0.036)
1573 (0.071)
1164 (0.11)
1573 (0.071)
1164 (0.11)
#SAKNAS!
2395 (0.036)
561 (0.25)
936 (0.14)
1573 (0.071)
1573 (0.071)
648 (0.21)
1164 (0.11)
2395 (0.036)
1164 (0.11)
#SAKNAS!
1164 (0.11)
2395 (0.036)
1164 (0.11)
2395 (0.036)
1164 (0.11)
#SAKNAS!
1164 (0.11)
2395 (0.036)
936 (0.14)

1573 (0.071)
1164 (0.11)
1164 (0.11)
506 (0.28)
1573 (0.071)
2395 (0.036)
#SAKNAS!
#SAKNAS!
1573 (0.071)
1164 (0.11)
2395 (0.036)
773 (0.18)
1573 (0.071)
1573 (0.071)
773 (0.18)
1164 (0.11)
2395 (0.036)
243 (0.6)
1164 (0.11)
1164 (0.11)
1573 (0.071)
773 (0.18)
1164 (0.11)
936 (0.14)
506 (0.28)
2395 (0.036)
1573 (0.071)
1164 (0.11)
648 (0.21)
#SAKNAS!
#SAKNAS!
936 (0.14)
#SAKNAS!
#SAKNAS!
936 (0.14)
1573 (0.071)
773 (0.18)
506 (0.28)
1164 (0.11)
1573 (0.071)
1164 (0.11)
#SAKNAS!
648 (0.21)

1573 (0.071)
1573 (0.071)
1573 (0.071)
773 (0.18)
936 (0.14)
561 (0.25)
#SAKNAS!
2395 (0.036)
1164 (0.11)
1573 (0.071)
1573 (0.071)
#SAKNAS!
1573 (0.071)
#SAKNAS!
936 (0.14)
#SAKNAS!
2395 (0.036)
2395 (0.036)
1573 (0.071)
#SAKNAS!
2395 (0.036)
1573 (0.071)
2395 (0.036)
1164 (0.11)
1573 (0.071)
2395 (0.036)
648 (0.21)
2395 (0.036)
773 (0.18)
301 (0.5)
648 (0.21)
341 (0.43)
1164 (0.11)
648 (0.21)
1573 (0.071)
2395 (0.036)
#SAKNAS!
2395 (0.036)
1573 (0.071)
936 (0.14)
#SAKNAS!
446 (0.32)
773 (0.18)

1573 (0.071)
1573 (0.071)
1573 (0.071)
1164 (0.11)
1573 (0.071)
773 (0.18)
936 (0.14)
411 (0.36)
#SAKNAS!
936 (0.14)
#SAKNAS!
#SAKNAS!
936 (0.14)
1573 (0.071)
1573 (0.071)
#SAKNAS!
225 (0.64)
1164 (0.11)
1164 (0.11)
1573 (0.071)
411 (0.36)
1573 (0.071)
1573 (0.071)
1573 (0.071)
#SAKNAS!
#SAKNAS!
1573 (0.071)
936 (0.14)
370 (0.39)
1573 (0.071)
142 (1)
1573 (0.071)
936 (0.14)
1164 (0.11)
2395 (0.036)
2395 (0.036)
1573 (0.071)
#SAKNAS!
773 (0.18)
1164 (0.11)
2395 (0.036)
1573 (0.071)
1573 (0.071)

1573 (0.071)
2395 (0.036)
411 (0.36)
2395 (0.036)
1573 (0.071)
1164 (0.11)
1573 (0.071)
2395 (0.036)
1164 (0.11)
1573 (0.071)
2395 (0.036)
#SAKNAS!
2395 (0.036)
1164 (0.11)
1164 (0.11)
773 (0.18)
#SAKNAS!
#SAKNAS!
341 (0.43)
2395 (0.036)
301 (0.5)
1164 (0.11)
370 (0.39)
187 (0.75)
1573 (0.071)
773 (0.18)
#SAKNAS!
1573 (0.071)
1164 (0.11)
1164 (0.11)
411 (0.36)
446 (0.32)
1164 (0.11)
114 (1.2)
1573 (0.071)
#SAKNAS!
#SAKNAS!
2395 (0.036)
1164 (0.11)
1573 (0.071)
1573 (0.071)
1573 (0.071)
2395 (0.036)

773 (0.18)
1164 (0.11)
2395 (0.036)
2395 (0.036)
#SAKNAS!
2395 (0.036)
1164 (0.11)
341 (0.43)
2395 (0.036)
2395 (0.036)
773 (0.18)
648 (0.21)
1573 (0.071)
1573 (0.071)
#SAKNAS!
#SAKNAS!
1573 (0.071)
#SAKNAS!
1573 (0.071)
648 (0.21)
#SAKNAS!
2395 (0.036)
936 (0.14)
936 (0.14)
1573 (0.071)
936 (0.14)
2395 (0.036)
936 (0.14)
2395 (0.036)
936 (0.14)
2395 (0.036)
1573 (0.071)
2395 (0.036)
2395 (0.036)
411 (0.36)
1164 (0.11)
1573 (0.071)
648 (0.21)
1164 (0.11)
773 (0.18)
1573 (0.071)
648 (0.21)
209 (0.67)

1573 (0.071)
936 (0.14)
1573 (0.071)
936 (0.14)
773 (0.18)
1573 (0.071)
506 (0.28)
936 (0.14)
1573 (0.071)
1573 (0.071)
2395 (0.036)
#SAKNAS!
243 (0.6)
1164 (0.11)
1573 (0.071)
2395 (0.036)
936 (0.14)
1573 (0.071)
1573 (0.071)
1573 (0.071)
648 (0.21)
1164 (0.11)
936 (0.14)
1573 (0.071)
1573 (0.071)
1573 (0.071)
1164 (0.11)
1164 (0.11)
#SAKNAS!
561 (0.25)
1573 (0.071)
1573 (0.071)
446 (0.32)
1573 (0.071)
2395 (0.036)
1164 (0.11)
936 (0.14)
1164 (0.11)
1573 (0.071)
1164 (0.11)
2395 (0.036)
1573 (0.071)
#SAKNAS!

773 (0.18)
1573 (0.071)
1164 (0.11)
648 (0.21)
2395 (0.036)
#SAKNAS!
#SAKNAS!
773 (0.18)
2395 (0.036)
2395 (0.036)
#SAKNAS!
1573 (0.071)
1573 (0.071)
1573 (0.071)
2395 (0.036)
1573 (0.071)
936 (0.14)
1164 (0.11)
#SAKNAS!
#SAKNAS!
2395 (0.036)
2395 (0.036)
1573 (0.071)
2395 (0.036)
2395 (0.036)
1164 (0.11)
#SAKNAS!
1164 (0.11)
2395 (0.036)
2395 (0.036)
1164 (0.11)
773 (0.18)
2395 (0.036)
2395 (0.036)
#SAKNAS!
#SAKNAS!
446 (0.32)
2395 (0.036)
2395 (0.036)
#SAKNAS!
1164 (0.11)
1164 (0.11)
2395 (0.036)

773 (0.18)
1573 (0.071)
1573 (0.071)
1573 (0.071)
936 (0.14)
#SAKNAS!
2395 (0.036)
2395 (0.036)
506 (0.28)
1573 (0.071)
2395 (0.036)
#SAKNAS!
#SAKNAS!
1573 (0.071)
2395 (0.036)
1164 (0.11)
2395 (0.036)
#SAKNAS!
#SAKNAS!
1164 (0.11)
2395 (0.036)
1573 (0.071)
2395 (0.036)
1573 (0.071)
1164 (0.11)
2395 (0.036)
2395 (0.036)
2395 (0.036)
1164 (0.11)
1573 (0.071)
648 (0.21)
561 (0.25)
1573 (0.071)
1573 (0.071)
446 (0.32)
561 (0.25)
773 (0.18)
936 (0.14)
936 (0.14)
2395 (0.036)
1573 (0.071)
1573 (0.071)
773 (0.18)

2395 (0.036)
2395 (0.036)
773 (0.18)
#SAKNAS!
2395 (0.036)
#SAKNAS!
#SAKNAS!
1164 (0.11)
1573 (0.071)
1573 (0.071)
936 (0.14)
936 (0.14)
2395 (0.036)
1573 (0.071)
1573 (0.071)
936 (0.14)
#SAKNAS!
1573 (0.071)
936 (0.14)
1164 (0.11)
#SAKNAS!
1164 (0.11)
2395 (0.036)
#SAKNAS!
936 (0.14)
2395 (0.036)
2395 (0.036)
936 (0.14)
2395 (0.036)
2395 (0.036)
#SAKNAS!
1164 (0.11)
2395 (0.036)
#SAKNAS!
2395 (0.036)
936 (0.14)
936 (0.14)
773 (0.18)
2395 (0.036)
#SAKNAS!
#SAKNAS!
2395 (0.036)
1573 (0.071)

341 (0.43)
773 (0.18)
#SAKNAS!
#SAKNAS!
1573 (0.071)
1164 (0.11)
2395 (0.036)
319 (0.46)
1573 (0.071)
936 (0.14)
#SAKNAS!
1573 (0.071)
1164 (0.11)
1164 (0.11)
936 (0.14)
2395 (0.036)
2395 (0.036)
1573 (0.071)
648 (0.21)
1573 (0.071)
2395 (0.036)
1164 (0.11)
#SAKNAS!
#SAKNAS!
1164 (0.11)
1573 (0.071)
1164 (0.11)
#SAKNAS!
1164 (0.11)
773 (0.18)
1573 (0.071)
773 (0.18)
#SAKNAS!
#SAKNAS!
1573 (0.071)
#SAKNAS!
#SAKNAS!
1573 (0.071)
1164 (0.11)
#SAKNAS!
#SAKNAS!
936 (0.14)
1164 (0.11)

1164 (0.11)
1573 (0.071)
1573 (0.071)
#SAKNAS!
2395 (0.036)
#SAKNAS!
1164 (0.11)
1573 (0.071)
#SAKNAS!
2395 (0.036)
1573 (0.071)
1164 (0.11)
#SAKNAS!
#SAKNAS!
936 (0.14)
1164 (0.11)
1573 (0.071)
#SAKNAS!
1164 (0.11)
2395 (0.036)
#SAKNAS!
1573 (0.071)
936 (0.14)
2395 (0.036)
1164 (0.11)
1573 (0.071)
1164 (0.11)
2395 (0.036)
1573 (0.071)
1573 (0.071)
1573 (0.071)
446 (0.32)
2395 (0.036)
1164 (0.11)
#SAKNAS!
2395 (0.036)
1573 (0.071)
1164 (0.11)
1164 (0.11)
1164 (0.11)
#SAKNAS!
1164 (0.11)
1573 (0.071)

2395 (0.036)
1164 (0.11)
561 (0.25)
2395 (0.036)
1164 (0.11)
2395 (0.036)
1164 (0.11)
936 (0.14)
773 (0.18)
1573 (0.071)
#SAKNAS!
261 (0.57)
1573 (0.071)
#SAKNAS!
2395 (0.036)
2395 (0.036)
1573 (0.071)
1573 (0.071)
2395 (0.036)
1164 (0.11)
936 (0.14)
319 (0.46)
1573 (0.071)
#SAKNAS!
#SAKNAS!
648 (0.21)
2395 (0.036)
261 (0.57)
#SAKNAS!
1164 (0.11)
#SAKNAS!
1573 (0.071)
1573 (0.071)
1573 (0.071)
#SAKNAS!
1573 (0.071)
936 (0.14)
#SAKNAS!
#SAKNAS!
#SAKNAS!
#SAKNAS!
1164 (0.11)
648 (0.21)

1164 (0.11)
936 (0.14)
2395 (0.036)
1573 (0.071)
773 (0.18)
2395 (0.036)
#SAKNAS!
1573 (0.071)
2395 (0.036)
1164 (0.11)
#SAKNAS!
1573 (0.071)
2395 (0.036)
2395 (0.036)
561 (0.25)
2395 (0.036)
1573 (0.071)
1573 (0.071)
1573 (0.071)
1164 (0.11)
2395 (0.036)
1573 (0.071)
773 (0.18)
773 (0.18)
2395 (0.036)
1164 (0.11)
1164 (0.11)
1573 (0.071)
936 (0.14)
1164 (0.11)
1573 (0.071)
2395 (0.036)
1164 (0.11)
2395 (0.036)
#SAKNAS!
1573 (0.071)
1573 (0.071)
2395 (0.036)
1164 (0.11)
#SAKNAS!
1573 (0.071)
1164 (0.11)
2395 (0.036)

1164 (0.11)
2395 (0.036)
446 (0.32)
#SAKNAS!
2395 (0.036)
2395 (0.036)
2395 (0.036)
#SAKNAS!
1573 (0.071)
2395 (0.036)
936 (0.14)
936 (0.14)
2395 (0.036)
#SAKNAS!
#SAKNAS!
773 (0.18)
2395 (0.036)
1164 (0.11)
2395 (0.036)
561 (0.25)
#SAKNAS!
#SAKNAS!
1573 (0.071)
#SAKNAS!
1573 (0.071)
936 (0.14)
936 (0.14)
2395 (0.036)
2395 (0.036)
#SAKNAS!
2395 (0.036)
#SAKNAS!
1573 (0.071)
1164 (0.11)
#SAKNAS!
1164 (0.11)
#SAKNAS!
1573 (0.071)
2395 (0.036)
1573 (0.071)
2395 (0.036)
1164 (0.11)
1573 (0.071)

#SAKNAS!
2395 (0.036)
1573 (0.071)
936 (0.14)
#SAKNAS!
#SAKNAS!
1164 (0.11)
#SAKNAS!
1573 (0.071)
301 (0.5)
301 (0.5)
1164 (0.11)
1573 (0.071)
1164 (0.11)
#SAKNAS!
773 (0.18)
1573 (0.071)
936 (0.14)
773 (0.18)
506 (0.28)
936 (0.14)
1164 (0.11)
561 (0.25)
#SAKNAS!
2395 (0.036)
1573 (0.071)
446 (0.32)
2395 (0.036)
446 (0.32)
1164 (0.11)
261 (0.57)
648 (0.21)
2395 (0.036)
2395 (0.036)
#SAKNAS!
936 (0.14)
1164 (0.11)
2395 (0.036)
1573 (0.071)
1573 (0.071)
2395 (0.036)
1573 (0.071)
#SAKNAS!

1164 (0.11)
1573 (0.071)
1164 (0.11)
773 (0.18)
1573 (0.071)
936 (0.14)
1573 (0.071)
411 (0.36)
1573 (0.071)
773 (0.18)
1573 (0.071)
1573 (0.071)
2395 (0.036)
#SAKNAS!
#SAKNAS!
#SAKNAS!
1164 (0.11)
#SAKNAS!
2395 (0.036)
#SAKNAS!
#SAKNAS!
936 (0.14)
1573 (0.071)
1573 (0.071)
370 (0.39)
1164 (0.11)
2395 (0.036)
1573 (0.071)
411 (0.36)
#SAKNAS!
2395 (0.036)
1573 (0.071)
2395 (0.036)
#SAKNAS!
2395 (0.036)
1164 (0.11)
1164 (0.11)
2395 (0.036)
1573 (0.071)
1573 (0.071)
#SAKNAS!
506 (0.28)
936 (0.14)

936 (0.14)
319 (0.46)
936 (0.14)
446 (0.32)
411 (0.36)
2395 (0.036)
1573 (0.071)
2395 (0.036)
2395 (0.036)
936 (0.14)
773 (0.18)
2395 (0.036)
1573 (0.071)
319 (0.46)
936 (0.14)
2395 (0.036)
1573 (0.071)
411 (0.36)
1573 (0.071)
1573 (0.071)
#SAKNAS!
936 (0.14)
2395 (0.036)
1573 (0.071)
#SAKNAS!
1164 (0.11)
2395 (0.036)
1164 (0.11)
1573 (0.071)
1573 (0.071)
561 (0.25)
936 (0.14)
1573 (0.071)
936 (0.14)
1573 (0.071)
936 (0.14)
936 (0.14)
209 (0.67)
1164 (0.11)
1573 (0.071)
1164 (0.11)
1573 (0.071)
1573 (0.071)

1164 (0.11)
2395 (0.036)
243 (0.6)
#SAKNAS!
1573 (0.071)
2395 (0.036)
1164 (0.11)
1164 (0.11)
773 (0.18)
#SAKNAS!
1164 (0.11)
2395 (0.036)
1164 (0.11)
1573 (0.071)
2395 (0.036)
561 (0.25)
2395 (0.036)
773 (0.18)
1573 (0.071)
2395 (0.036)
2395 (0.036)
936 (0.14)
2395 (0.036)
936 (0.14)
2395 (0.036)
1573 (0.071)
2395 (0.036)
#SAKNAS!
1573 (0.071)
#SAKNAS!
1573 (0.071)
2395 (0.036)
#SAKNAS!
#SAKNAS!
#SAKNAS!
1573 (0.071)
1573 (0.071)
#SAKNAS!
1573 (0.071)
773 (0.18)
936 (0.14)
#SAKNAS!
2395 (0.036)

#SAKNAS!
561 (0.25)
2395 (0.036)
#SAKNAS!
2395 (0.036)
2395 (0.036)
1164 (0.11)
#SAKNAS!
1164 (0.11)
#SAKNAS!
2395 (0.036)
936 (0.14)
1573 (0.071)
2395 (0.036)
#SAKNAS!
1164 (0.11)
1573 (0.071)
#SAKNAS!
2395 (0.036)
#SAKNAS!
936 (0.14)
1573 (0.071)
#SAKNAS!
2395 (0.036)
2395 (0.036)
936 (0.14)
1164 (0.11)
2395 (0.036)
#SAKNAS!
#SAKNAS!
1573 (0.071)
#SAKNAS!
1573 (0.071)
1573 (0.071)
1164 (0.11)
2395 (0.036)
#SAKNAS!
1164 (0.11)
1164 (0.11)
2395 (0.036)
#SAKNAS!
1164 (0.11)
2395 (0.036)

#SAKNAS!
2395 (0.036)
1573 (0.071)
1164 (0.11)
936 (0.14)
1164 (0.11)
446 (0.32)
2395 (0.036)
1164 (0.11)
#SAKNAS!
2395 (0.036)
1164 (0.11)
2395 (0.036)
1164 (0.11)
773 (0.18)
1573 (0.071)
#SAKNAS!
2395 (0.036)
1164 (0.11)
1164 (0.11)
#SAKNAS!
1164 (0.11)
506 (0.28)
936 (0.14)
#SAKNAS!
#SAKNAS!
#SAKNAS!
#SAKNAS!
#SAKNAS!
#SAKNAS!
1573 (0.071)
2395 (0.036)
#SAKNAS!
1573 (0.071)
1573 (0.071)
936 (0.14)
#SAKNAS!
648 (0.21)
2395 (0.036)
1164 (0.11)
2395 (0.036)
773 (0.18)
#SAKNAS!

2395 (0.036)
2395 (0.036)
2395 (0.036)
2395 (0.036)
2395 (0.036)
#SAKNAS!
2395 (0.036)
2395 (0.036)
1573 (0.071)
1573 (0.071)
1573 (0.071)
2395 (0.036)
936 (0.14)
1164 (0.11)
2395 (0.036)
2395 (0.036)
#SAKNAS!
#SAKNAS!
2395 (0.036)
2395 (0.036)
506 (0.28)
1164 (0.11)
#SAKNAS!
#SAKNAS!
2395 (0.036)
2395 (0.036)
#SAKNAS!
#SAKNAS!
1573 (0.071)
2395 (0.036)
#SAKNAS!
#SAKNAS!
#SAKNAS!
936 (0.14)
1573 (0.071)
#SAKNAS!
2395 (0.036)
#SAKNAS!
2395 (0.036)
2395 (0.036)
#SAKNAS!
2395 (0.036)
2395 (0.036)

561 (0.25)
1573 (0.071)
1573 (0.071)
2395 (0.036)
2395 (0.036)
1573 (0.071)
2395 (0.036)
648 (0.21)
2395 (0.036)
2395 (0.036)
1164 (0.11)
1164 (0.11)
2395 (0.036)
#SAKNAS!
2395 (0.036)
1164 (0.11)
#SAKNAS!
1573 (0.071)
#SAKNAS!
773 (0.18)
1164 (0.11)
773 (0.18)
2395 (0.036)
#SAKNAS!
1573 (0.071)
2395 (0.036)
1164 (0.11)
#SAKNAS!
1573 (0.071)
#SAKNAS!
#SAKNAS!
2395 (0.036)
1573 (0.071)
1164 (0.11)
#SAKNAS!
506 (0.28)
773 (0.18)
936 (0.14)
1573 (0.071)
1573 (0.071)
#SAKNAS!
#SAKNAS!
1164 (0.11)

1164 (0.11)
2395 (0.036)
2395 (0.036)
#SAKNAS!
#SAKNAS!
1573 (0.071)
1164 (0.11)
2395 (0.036)
2395 (0.036)
#SAKNAS!
2395 (0.036)
561 (0.25)
#SAKNAS!
2395 (0.036)
#SAKNAS!
2395 (0.036)
1573 (0.071)
#SAKNAS!
2395 (0.036)
2395 (0.036)
1573 (0.071)
#SAKNAS!
2395 (0.036)
#SAKNAS!
1573 (0.071)
936 (0.14)
#SAKNAS!
1164 (0.11)
1164 (0.11)
#SAKNAS!
1573 (0.071)
2395 (0.036)
561 (0.25)
2395 (0.036)
773 (0.18)
2395 (0.036)
#SAKNAS!
#SAKNAS!
1164 (0.11)
2395 (0.036)
1573 (0.071)
648 (0.21)
936 (0.14)

#SAKNAS!
1573 (0.071)
#SAKNAS!
936 (0.14)
2395 (0.036)
#SAKNAS!
#SAKNAS!
2395 (0.036)
2395 (0.036)
648 (0.21)
1573 (0.071)
#SAKNAS!
282 (0.53)
1573 (0.071)
2395 (0.036)
936 (0.14)
#SAKNAS!
2395 (0.036)
2395 (0.036)
1164 (0.11)
506 (0.28)
773 (0.18)
2395 (0.036)
2395 (0.036)
#SAKNAS!
561 (0.25)
2395 (0.036)
1573 (0.071)
2395 (0.036)
2395 (0.036)
#SAKNAS!
#SAKNAS!
#SAKNAS!
1573 (0.071)
1573 (0.071)
#SAKNAS!
2395 (0.036)
773 (0.18)
2395 (0.036)
#SAKNAS!
1164 (0.11)
1164 (0.11)
2395 (0.036)

1164 (0.11)
1573 (0.071)
1573 (0.071)
1573 (0.071)
1573 (0.071)
#SAKNAS!
#SAKNAS!
1573 (0.071)
#SAKNAS!
2395 (0.036)
1573 (0.071)
1573 (0.071)
#SAKNAS!
#SAKNAS!
1164 (0.11)
1573 (0.071)
#SAKNAS!
1573 (0.071)
2395 (0.036)
1573 (0.071)
2395 (0.036)
1573 (0.071)
370 (0.39)
2395 (0.036)
506 (0.28)
#SAKNAS!
#SAKNAS!
648 (0.21)
1573 (0.071)
1164 (0.11)
2395 (0.036)
1164 (0.11)
1573 (0.071)
#SAKNAS!
#SAKNAS!
446 (0.32)
2395 (0.036)
1164 (0.11)
1573 (0.071)
2395 (0.036)
2395 (0.036)
1573 (0.071)
243 (0.6)

#SAKNAS!
2395 (0.036)
2395 (0.036)
#SAKNAS!
2395 (0.036)
1164 (0.11)
2395 (0.036)
1573 (0.071)
2395 (0.036)
2395 (0.036)
#SAKNAS!
1164 (0.11)
2395 (0.036)
1164 (0.11)
1164 (0.11)
#SAKNAS!
1573 (0.071)
2395 (0.036)
#SAKNAS!
#SAKNAS!
1573 (0.071)
#SAKNAS!
2395 (0.036)
#SAKNAS!
2395 (0.036)
2395 (0.036)
1573 (0.071)
1573 (0.071)
506 (0.28)
1573 (0.071)
506 (0.28)
#SAKNAS!
936 (0.14)
773 (0.18)
1164 (0.11)
2395 (0.036)
#SAKNAS!
#SAKNAS!
2395 (0.036)
2395 (0.036)
1573 (0.071)
2395 (0.036)
1573 (0.071)

2395 (0.036)
#SAKNAS!
648 (0.21)
#SAKNAS!
1573 (0.071)
1164 (0.11)
2395 (0.036)
2395 (0.036)
#SAKNAS!
1164 (0.11)
561 (0.25)
1164 (0.11)
1573 (0.071)
2395 (0.036)
2395 (0.036)
#SAKNAS!
2395 (0.036)
2395 (0.036)
2395 (0.036)
1164 (0.11)
936 (0.14)
1164 (0.11)
936 (0.14)
1573 (0.071)
#SAKNAS!
2395 (0.036)
1164 (0.11)
1164 (0.11)
#SAKNAS!
#SAKNAS!
173 (0.78)
2395 (0.036)
2395 (0.036)
1573 (0.071)
#SAKNAS!
#SAKNAS!
446 (0.32)
648 (0.21)
2395 (0.036)
2395 (0.036)
2395 (0.036)
#SAKNAS!
1573 (0.071)

2395 (0.036)
1573 (0.071)
1573 (0.071)
2395 (0.036)
936 (0.14)
1573 (0.071)
2395 (0.036)
2395 (0.036)
#SAKNAS!
2395 (0.036)
1573 (0.071)
648 (0.21)
1573 (0.071)
506 (0.28)
2395 (0.036)
2395 (0.036)
2395 (0.036)
#SAKNAS!
2395 (0.036)
2395 (0.036)
1573 (0.071)
1573 (0.071)
2395 (0.036)
#SAKNAS!
2395 (0.036)
#SAKNAS!
105 (1.3)
1573 (0.071)
#SAKNAS!
936 (0.14)
1164 (0.11)
1573 (0.071)
1573 (0.071)
2395 (0.036)
2395 (0.036)
1573 (0.071)
2395 (0.036)
2395 (0.036)
#SAKNAS!
1164 (0.11)
#SAKNAS!
#SAKNAS!
1573 (0.071)

1573 (0.071)
2395 (0.036)
1164 (0.11)
1573 (0.071)
1164 (0.11)
#SAKNAS!
1164 (0.11)
#SAKNAS!
2395 (0.036)
1573 (0.071)
2395 (0.036)
1573 (0.071)
#SAKNAS!
1573 (0.071)
#SAKNAS!
#SAKNAS!
2395 (0.036)
561 (0.25)
2395 (0.036)
2395 (0.036)
2395 (0.036)
1164 (0.11)
1573 (0.071)
2395 (0.036)
1573 (0.071)
#SAKNAS!
1573 (0.071)
1573 (0.071)
2395 (0.036)
2395 (0.036)
#SAKNAS!
2395 (0.036)
2395 (0.036)
#SAKNAS!
261 (0.57)
1164 (0.11)
2395 (0.036)
1573 (0.071)
#SAKNAS!
1573 (0.071)
2395 (0.036)
1164 (0.11)
1573 (0.071)

2395 (0.036)
#SAKNAS!
#SAKNAS!
#SAKNAS!
1573 (0.071)
1164 (0.11)
936 (0.14)
2395 (0.036)
#SAKNAS!
1573 (0.071)
936 (0.14)
1573 (0.071)
1573 (0.071)
2395 (0.036)
#SAKNAS!
#SAKNAS!
132 (1.1)
#SAKNAS!
1573 (0.071)
2395 (0.036)
2395 (0.036)
#SAKNAS!
1573 (0.071)
936 (0.14)
1573 (0.071)
648 (0.21)
1573 (0.071)
1573 (0.071)
2395 (0.036)
#SAKNAS!
1573 (0.071)
1573 (0.071)
#SAKNAS!
561 (0.25)
411 (0.36)
#SAKNAS!
#SAKNAS!
2395 (0.036)
1573 (0.071)
1164 (0.11)
2395 (0.036)
936 (0.14)
2395 (0.036)

1573 (0.071)
2395 (0.036)
2395 (0.036)
2395 (0.036)
1573 (0.071)
1573 (0.071)
1573 (0.071)
2395 (0.036)
#SAKNAS!
2395 (0.036)
2395 (0.036)
561 (0.25)
2395 (0.036)
1573 (0.071)
506 (0.28)
1573 (0.071)
2395 (0.036)
#SAKNAS!
2395 (0.036)
1573 (0.071)
2395 (0.036)
2395 (0.036)
936 (0.14)
773 (0.18)
1573 (0.071)
936 (0.14)
2395 (0.036)
1573 (0.071)
#SAKNAS!
#SAKNAS!
1573 (0.071)
#SAKNAS!
2395 (0.036)
2395 (0.036)
2395 (0.036)
2395 (0.036)
2395 (0.036)
370 (0.39)
1573 (0.071)
2395 (0.036)
2395 (0.036)
#SAKNAS!
1573 (0.071)

#SAKNAS!
1164 (0.11)
#SAKNAS!
2395 (0.036)
1573 (0.071)
#SAKNAS!
#SAKNAS!
1573 (0.071)
1164 (0.11)
1573 (0.071)
1573 (0.071)
1164 (0.11)
1573 (0.071)
1573 (0.071)
1164 (0.11)
341 (0.43)
#SAKNAS!
#SAKNAS!
1573 (0.071)
411 (0.36)
1573 (0.071)
2395 (0.036)
1573 (0.071)
#SAKNAS!
370 (0.39)
1573 (0.071)
2395 (0.036)
2395 (0.036)
936 (0.14)
#SAKNAS!
1164 (0.11)
1573 (0.071)
#SAKNAS!
1573 (0.071)
#SAKNAS!
2395 (0.036)
1164 (0.11)
1164 (0.11)
1573 (0.071)
#SAKNAS!
#SAKNAS!
2395 (0.036)
#SAKNAS!

#SAKNAS!
#SAKNAS!
1573 (0.071)
1164 (0.11)
#SAKNAS!
1573 (0.071)
2395 (0.036)
2395 (0.036)
2395 (0.036)
2395 (0.036)
1573 (0.071)
1573 (0.071)
1164 (0.11)
2395 (0.036)
#SAKNAS!
1164 (0.11)
1164 (0.11)
#SAKNAS!
#SAKNAS!
648 (0.21)
#SAKNAS!
1573 (0.071)
2395 (0.036)
2395 (0.036)
446 (0.32)
1164 (0.11)
1573 (0.071)
2395 (0.036)
#SAKNAS!
1573 (0.071)
#SAKNAS!
2395 (0.036)
1164 (0.11)
1573 (0.071)
1164 (0.11)
2395 (0.036)
2395 (0.036)
1164 (0.11)
2395 (0.036)
2395 (0.036)
2395 (0.036)
2395 (0.036)
1164 (0.11)

#SAKNAS!
1573 (0.071)
1573 (0.071)
341 (0.43)
2395 (0.036)
1573 (0.071)
2395 (0.036)
#SAKNAS!
#SAKNAS!
648 (0.21)
1573 (0.071)
1573 (0.071)
#SAKNAS!
#SAKNAS!
#SAKNAS!
1164 (0.11)
936 (0.14)
#SAKNAS!
648 (0.21)
#SAKNAS!
2395 (0.036)
1164 (0.11)
446 (0.32)
2395 (0.036)
1164 (0.11)
1573 (0.071)
1573 (0.071)
1573 (0.071)
2395 (0.036)
2395 (0.036)
#SAKNAS!
936 (0.14)
936 (0.14)
1164 (0.11)
1164 (0.11)
1573 (0.071)
1164 (0.11)
1164 (0.11)
2395 (0.036)
1573 (0.071)
2395 (0.036)
2395 (0.036)
#SAKNAS!

#SAKNAS!
#SAKNAS!
2395 (0.036)
1164 (0.11)
#SAKNAS!
#SAKNAS!
1573 (0.071)
1164 (0.11)
#SAKNAS!
2395 (0.036)
1573 (0.071)
1164 (0.11)
1573 (0.071)
2395 (0.036)
2395 (0.036)
#SAKNAS!
1573 (0.071)
#SAKNAS!
#SAKNAS!
2395 (0.036)
1573 (0.071)
#SAKNAS!
#SAKNAS!
1573 (0.071)
2395 (0.036)
#SAKNAS!
2395 (0.036)
2395 (0.036)
2395 (0.036)
2395 (0.036)
506 (0.28)
2395 (0.036)
1573 (0.071)
648 (0.21)
2395 (0.036)
2395 (0.036)
#SAKNAS!
2395 (0.036)
1573 (0.071)
1573 (0.071)
1573 (0.071)
2395 (0.036)
#SAKNAS!

1164 (0.11)
2395 (0.036)
1573 (0.071)
1573 (0.071)
#SAKNAS!
#SAKNAS!
#SAKNAS!
1573 (0.071)
#SAKNAS!
#SAKNAS!
319 (0.46)
#SAKNAS!
2395 (0.036)
#SAKNAS!
2395 (0.036)
1573 (0.071)
1573 (0.071)
936 (0.14)
2395 (0.036)
1573 (0.071)
2395 (0.036)
2395 (0.036)
773 (0.18)
1573 (0.071)
2395 (0.036)
2395 (0.036)
1573 (0.071)
411 (0.36)
1573 (0.071)
2395 (0.036)
561 (0.25)
1573 (0.071)
2395 (0.036)
2395 (0.036)
2395 (0.036)
1573 (0.071)
2395 (0.036)
#SAKNAS!
1573 (0.071)
#SAKNAS!
936 (0.14)
2395 (0.036)
2395 (0.036)

#SAKNAS!
#SAKNAS!
2395 (0.036)
1573 (0.071)
#SAKNAS!
773 (0.18)
#SAKNAS!
2395 (0.036)
1573 (0.071)
1164 (0.11)
1573 (0.071)
#SAKNAS!
#SAKNAS!
1164 (0.11)
#SAKNAS!
1164 (0.11)
#SAKNAS!
#SAKNAS!
#SAKNAS!
#SAKNAS!
#SAKNAS!
#SAKNAS!
1573 (0.071)
#SAKNAS!
773 (0.18)
#SAKNAS!
2395 (0.036)
#SAKNAS!
#SAKNAS!
773 (0.18)
2395 (0.036)
2395 (0.036)
2395 (0.036)
#SAKNAS!
2395 (0.036)
1573 (0.071)
#SAKNAS!
1573 (0.071)
648 (0.21)
936 (0.14)
1573 (0.071)
1573 (0.071)
2395 (0.036)

2395 (0.036)
506 (0.28)
1573 (0.071)
#SAKNAS!
2395 (0.036)
2395 (0.036)
#SAKNAS!
#SAKNAS!
#SAKNAS!
1573 (0.071)
2395 (0.036)
2395 (0.036)
#SAKNAS!
1164 (0.11)
2395 (0.036)
#SAKNAS!
2395 (0.036)
2395 (0.036)
2395 (0.036)
648 (0.21)
773 (0.18)
2395 (0.036)
#SAKNAS!
2395 (0.036)
2395 (0.036)
2395 (0.036)
2395 (0.036)
#SAKNAS!
2395 (0.036)
#SAKNAS!
#SAKNAS!
1573 (0.071)
1573 (0.071)
2395 (0.036)
#SAKNAS!
#SAKNAS!
2395 (0.036)
1164 (0.11)
2395 (0.036)
#SAKNAS!
1164 (0.11)
2395 (0.036)
2395 (0.036)

1164 (0.11)
648 (0.21)
#SAKNAS!
2395 (0.036)
2395 (0.036)
#SAKNAS!
2395 (0.036)
2395 (0.036)
2395 (0.036)
2395 (0.036)
1573 (0.071)
1573 (0.071)
#SAKNAS!
#SAKNAS!
#SAKNAS!
#SAKNAS!
#SAKNAS!
2395 (0.036)
1164 (0.11)
773 (0.18)
2395 (0.036)
1164 (0.11)
1573 (0.071)
1573 (0.071)
#SAKNAS!
#SAKNAS!
#SAKNAS!
#SAKNAS!
2395 (0.036)
#SAKNAS!
1573 (0.071)
936 (0.14)
1573 (0.071)
2395 (0.036)
2395 (0.036)
506 (0.28)
1164 (0.11)
2395 (0.036)
2395 (0.036)
1164 (0.11)
#SAKNAS!
#SAKNAS!
1164 (0.11)

2395 (0.036)
#SAKNAS!
1573 (0.071)
1573 (0.071)
1573 (0.071)
2395 (0.036)
2395 (0.036)
1573 (0.071)
773 (0.18)
2395 (0.036)
2395 (0.036)
#SAKNAS!
2395 (0.036)
2395 (0.036)
1573 (0.071)
#SAKNAS!
#SAKNAS!
2395 (0.036)
#SAKNAS!
2395 (0.036)
#SAKNAS!
#SAKNAS!
1573 (0.071)
2395 (0.036)
#SAKNAS!
2395 (0.036)
773 (0.18)
#SAKNAS!
2395 (0.036)
2395 (0.036)
#SAKNAS!
1573 (0.071)
#SAKNAS!
2395 (0.036)
2395 (0.036)
2395 (0.036)
2395 (0.036)
#SAKNAS!
2395 (0.036)
1164 (0.11)
#SAKNAS!
2395 (0.036)
#SAKNAS!

2395 (0.036)
2395 (0.036)
2395 (0.036)
1573 (0.071)
1573 (0.071)
1164 (0.11)
1164 (0.11)
1573 (0.071)
2395 (0.036)
2395 (0.036)
2395 (0.036)
1164 (0.11)
2395 (0.036)
936 (0.14)
#SAKNAS!
1164 (0.11)
2395 (0.036)
1573 (0.071)
2395 (0.036)
#SAKNAS!
2395 (0.036)
2395 (0.036)
#SAKNAS!
2395 (0.036)
#SAKNAS!
#SAKNAS!
1573 (0.071)
2395 (0.036)
2395 (0.036)
#SAKNAS!
2395 (0.036)
648 (0.21)
1573 (0.071)
#SAKNAS!
1573 (0.071)
#SAKNAS!
773 (0.18)
1573 (0.071)
#SAKNAS!
2395 (0.036)
1573 (0.071)
#SAKNAS!
1164 (0.11)

1573 (0.071)
1164 (0.11)
#SAKNAS!
370 (0.39)
#SAKNAS!
#SAKNAS!
2395 (0.036)
#SAKNAS!
#SAKNAS!
#SAKNAS!
#SAKNAS!
2395 (0.036)
1573 (0.071)
1164 (0.11)
2395 (0.036)
773 (0.18)
2395 (0.036)
1164 (0.11)
1573 (0.071)
1573 (0.071)
2395 (0.036)
#SAKNAS!
1573 (0.071)
#SAKNAS!
1164 (0.11)
#SAKNAS!
#SAKNAS!
936 (0.14)
2395 (0.036)
2395 (0.036)
1164 (0.11)
#SAKNAS!
2395 (0.036)
#SAKNAS!
2395 (0.036)
#SAKNAS!
1573 (0.071)
2395 (0.036)
1573 (0.071)
1573 (0.071)
1573 (0.071)
#SAKNAS!
#SAKNAS!

1164 (0.11)
2395 (0.036)
2395 (0.036)
2395 (0.036)
2395 (0.036)
2395 (0.036)
1164 (0.11)
446 (0.32)
#SAKNAS!
2395 (0.036)
1164 (0.11)
1573 (0.071)
1164 (0.11)
#SAKNAS!
#SAKNAS!
936 (0.14)
648 (0.21)
#SAKNAS!
#SAKNAS!
773 (0.18)
2395 (0.036)
2395 (0.036)
648 (0.21)
773 (0.18)
#SAKNAS!
#SAKNAS!
#SAKNAS!
2395 (0.036)
2395 (0.036)
1573 (0.071)
#SAKNAS!
#SAKNAS!
446 (0.32)
#SAKNAS!
1573 (0.071)
2395 (0.036)
#SAKNAS!
#SAKNAS!
1573 (0.071)
#SAKNAS!
#SAKNAS!
#SAKNAS!
#SAKNAS!

#SAKNAS!
1573 (0.071)
#SAKNAS!
1573 (0.071)
2395 (0.036)
#SAKNAS!
1164 (0.11)
2395 (0.036)
2395 (0.036)
1573 (0.071)
2395 (0.036)
1164 (0.11)
#SAKNAS!
648 (0.21)
1573 (0.071)
2395 (0.036)
2395 (0.036)
936 (0.14)
2395 (0.036)
1164 (0.11)
1573 (0.071)
2395 (0.036)
#SAKNAS!
2395 (0.036)
1573 (0.071)
2395 (0.036)
2395 (0.036)
2395 (0.036)
2395 (0.036)
#SAKNAS!
#SAKNAS!
#SAKNAS!
2395 (0.036)
#SAKNAS!
2395 (0.036)
1573 (0.071)
1164 (0.11)
1573 (0.071)
1573 (0.071)
2395 (0.036)
#SAKNAS!
#SAKNAS!
1164 (0.11)

2395 (0.036)
1164 (0.11)
#SAKNAS!
#SAKNAS!
#SAKNAS!
1573 (0.071)
#SAKNAS!
2395 (0.036)
#SAKNAS!
#SAKNAS!
2395 (0.036)
2395 (0.036)
2395 (0.036)
1573 (0.071)
#SAKNAS!
2395 (0.036)
#SAKNAS!
#SAKNAS!
2395 (0.036)
2395 (0.036)
2395 (0.036)
370 (0.39)
1573 (0.071)
1573 (0.071)
936 (0.14)
#SAKNAS!
#SAKNAS!
#SAKNAS!
2395 (0.036)
1164 (0.11)
2395 (0.036)
2395 (0.036)
1573 (0.071)
1164 (0.11)
2395 (0.036)
1164 (0.11)
1573 (0.071)
2395 (0.036)
#SAKNAS!
1573 (0.071)
#SAKNAS!
#SAKNAS!
#SAKNAS!

#SAKNAS!
1164 (0.11)
#SAKNAS!
#SAKNAS!
#SAKNAS!
2395 (0.036)
2395 (0.036)
2395 (0.036)
2395 (0.036)
2395 (0.036)
#SAKNAS!
#SAKNAS!
1573 (0.071)
2395 (0.036)
2395 (0.036)
1164 (0.11)
1573 (0.071)
2395 (0.036)
2395 (0.036)
#SAKNAS!
#SAKNAS!
648 (0.21)
2395 (0.036)
2395 (0.036)
2395 (0.036)
2395 (0.036)
1573 (0.071)
1164 (0.11)
1164 (0.11)
1164 (0.11)
2395 (0.036)
2395 (0.036)
1573 (0.071)
1164 (0.11)
773 (0.18)
2395 (0.036)
2395 (0.036)
#SAKNAS!
2395 (0.036)
2395 (0.036)
1573 (0.071)
2395 (0.036)
936 (0.14)

1573 (0.071)
#SAKNAS!
1573 (0.071)
1164 (0.11)
2395 (0.036)
2395 (0.036)
2395 (0.036)
#SAKNAS!
#SAKNAS!
1573 (0.071)
2395 (0.036)
2395 (0.036)
2395 (0.036)
2395 (0.036)
936 (0.14)
#SAKNAS!
#SAKNAS!
936 (0.14)
1573 (0.071)
#SAKNAS!
#SAKNAS!
2395 (0.036)
1573 (0.071)
#SAKNAS!
#SAKNAS!
2395 (0.036)
1573 (0.071)
2395 (0.036)
#SAKNAS!
1164 (0.11)
#SAKNAS!
#SAKNAS!
2395 (0.036)
936 (0.14)
1573 (0.071)
#SAKNAS!
2395 (0.036)
2395 (0.036)
1573 (0.071)
#SAKNAS!
#SAKNAS!
#SAKNAS!
1573 (0.071)

2395 (0.036)
2395 (0.036)
1164 (0.11)
#SAKNAS!
2395 (0.036)
2395 (0.036)
1573 (0.071)
2395 (0.036)
#SAKNAS!
#SAKNAS!
2395 (0.036)
1164 (0.11)
2395 (0.036)
#SAKNAS!
#SAKNAS!
2395 (0.036)
1573 (0.071)
2395 (0.036)
936 (0.14)
2395 (0.036)
2395 (0.036)
1573 (0.071)
#SAKNAS!
#SAKNAS!
#SAKNAS!
#SAKNAS!
2395 (0.036)
#SAKNAS!
2395 (0.036)
648 (0.21)
#SAKNAS!
2395 (0.036)
2395 (0.036)
2395 (0.036)
#SAKNAS!
773 (0.18)
#SAKNAS!
1573 (0.071)
#SAKNAS!
#SAKNAS!
1573 (0.071)
#SAKNAS!
#SAKNAS!

#SAKNAS!
#SAKNAS!
936 (0.14)
1573 (0.071)
2395 (0.036)
#SAKNAS!
1573 (0.071)
1573 (0.071)
1164 (0.11)
2395 (0.036)
1573 (0.071)
#SAKNAS!
#SAKNAS!
#SAKNAS!
2395 (0.036)
936 (0.14)
1573 (0.071)
2395 (0.036)
#SAKNAS!
#SAKNAS!
#SAKNAS!
#SAKNAS!
2395 (0.036)
#SAKNAS!
#SAKNAS!
1164 (0.11)
2395 (0.036)
#SAKNAS!
#SAKNAS!
1573 (0.071)
#SAKNAS!
2395 (0.036)
1573 (0.071)
2395 (0.036)
1573 (0.071)
#SAKNAS!
773 (0.18)
2395 (0.036)
#SAKNAS!
1164 (0.11)
2395 (0.036)
#SAKNAS!
1573 (0.071)

1573 (0.071)
#SAKNAS!
#SAKNAS!
#SAKNAS!
#SAKNAS!
2395 (0.036)
#SAKNAS!
1164 (0.11)
2395 (0.036)
1573 (0.071)
#SAKNAS!
#SAKNAS!
1573 (0.071)
2395 (0.036)
2395 (0.036)
2395 (0.036)
152 (0.89)
2395 (0.036)
2395 (0.036)
#SAKNAS!
#SAKNAS!
1573 (0.071)
#SAKNAS!
2395 (0.036)
2395 (0.036)
1573 (0.071)
#SAKNAS!
165 (0.82)
2395 (0.036)
1573 (0.071)
#SAKNAS!
2395 (0.036)
2395 (0.036)
1573 (0.071)
2395 (0.036)
1573 (0.071)
1573 (0.071)
#SAKNAS!
2395 (0.036)
1573 (0.071)
1573 (0.071)
#SAKNAS!
1573 (0.071)

1573 (0.071)
2395 (0.036)
1573 (0.071)
2395 (0.036)
2395 (0.036)
1573 (0.071)
2395 (0.036)
2395 (0.036)
2395 (0.036)
2395 (0.036)
#SAKNAS!
2395 (0.036)
2395 (0.036)
1573 (0.071)
2395 (0.036)
#SAKNAS!
370 (0.39)
#SAKNAS!
1164 (0.11)
1573 (0.071)
1573 (0.071)
2395 (0.036)
#SAKNAS!
2395 (0.036)
1573 (0.071)
#SAKNAS!
#SAKNAS!
2395 (0.036)
1573 (0.071)
2395 (0.036)
2395 (0.036)
1573 (0.071)
2395 (0.036)
1573 (0.071)
1164 (0.11)
2395 (0.036)
2395 (0.036)
2395 (0.036)
#SAKNAS!
1164 (0.11)
#SAKNAS!
936 (0.14)
2395 (0.036)

2395 (0.036)
2395 (0.036)
1164 (0.11)
2395 (0.036)
773 (0.18)
1573 (0.071)
#SAKNAS!
561 (0.25)
#SAKNAS!
#SAKNAS!
#SAKNAS!
1164 (0.11)
2395 (0.036)
2395 (0.036)
#SAKNAS!
#SAKNAS!
#SAKNAS!
1573 (0.071)
1164 (0.11)
1164 (0.11)
#SAKNAS!
1573 (0.071)
2395 (0.036)
#SAKNAS!
#SAKNAS!
#SAKNAS!
#SAKNAS!
2395 (0.036)
#SAKNAS!
1573 (0.071)
2395 (0.036)
1573 (0.071)
936 (0.14)
#SAKNAS!
2395 (0.036)
#SAKNAS!
#SAKNAS!
2395 (0.036)
2395 (0.036)
2395 (0.036)
#SAKNAS!
2395 (0.036)
1573 (0.071)

#SAKNAS!
#SAKNAS!
#SAKNAS!
1164 (0.11)
446 (0.32)
#SAKNAS!
2395 (0.036)
2395 (0.036)
1573 (0.071)
936 (0.14)
1164 (0.11)
2395 (0.036)
2395 (0.036)
2395 (0.036)
1573 (0.071)
1164 (0.11)
1164 (0.11)
2395 (0.036)
#SAKNAS!
#SAKNAS!
2395 (0.036)
1573 (0.071)
1573 (0.071)
2395 (0.036)
2395 (0.036)
2395 (0.036)
#SAKNAS!
2395 (0.036)
2395 (0.036)
2395 (0.036)
1573 (0.071)
773 (0.18)
#SAKNAS!
561 (0.25)
1573 (0.071)
2395 (0.036)
1573 (0.071)
1573 (0.071)
1573 (0.071)
2395 (0.036)
#SAKNAS!
1573 (0.071)
#SAKNAS!

1164 (0.11)
1573 (0.071)
2395 (0.036)
1164 (0.11)
648 (0.21)
#SAKNAS!
#SAKNAS!
1573 (0.071)
#SAKNAS!
2395 (0.036)
2395 (0.036)
936 (0.14)
2395 (0.036)
2395 (0.036)
261 (0.57)
1164 (0.11)
#SAKNAS!
648 (0.21)
#SAKNAS!
#SAKNAS!
#SAKNAS!
1573 (0.071)
#SAKNAS!
1573 (0.071)
2395 (0.036)
1573 (0.071)
1164 (0.11)
1164 (0.11)
1573 (0.071)
2395 (0.036)
2395 (0.036)
1164 (0.11)
2395 (0.036)
1573 (0.071)
#SAKNAS!
#SAKNAS!
773 (0.18)
1573 (0.071)
1164 (0.11)
#SAKNAS!
#SAKNAS!
2395 (0.036)
#SAKNAS!

#SAKNAS!
2395 (0.036)
1573 (0.071)
#SAKNAS!
#SAKNAS!
#SAKNAS!
#SAKNAS!
1573 (0.071)
2395 (0.036)
936 (0.14)
2395 (0.036)
2395 (0.036)
2395 (0.036)
2395 (0.036)
2395 (0.036)
1573 (0.071)
1573 (0.071)
#SAKNAS!
2395 (0.036)
#SAKNAS!
2395 (0.036)
1573 (0.071)
2395 (0.036)
#SAKNAS!
2395 (0.036)
#SAKNAS!
1573 (0.071)
#SAKNAS!
#SAKNAS!
1164 (0.11)
2395 (0.036)
2395 (0.036)
936 (0.14)
#SAKNAS!
#SAKNAS!
1164 (0.11)
#SAKNAS!
1573 (0.071)
1573 (0.071)
2395 (0.036)
370 (0.39)
#SAKNAS!
1573 (0.071)

2395 (0.036)
2395 (0.036)
2395 (0.036)
2395 (0.036)
1573 (0.071)
1573 (0.071)
773 (0.18)
2395 (0.036)
2395 (0.036)
2395 (0.036)
2395 (0.036)
#SAKNAS!
#SAKNAS!
1164 (0.11)
#SAKNAS!
#SAKNAS!
1573 (0.071)
2395 (0.036)
1573 (0.071)
#SAKNAS!
#SAKNAS!
2395 (0.036)
#SAKNAS!
1573 (0.071)
1164 (0.11)
2395 (0.036)
2395 (0.036)
2395 (0.036)
446 (0.32)
#SAKNAS!
2395 (0.036)
#SAKNAS!
#SAKNAS!
#SAKNAS!
1573 (0.071)
2395 (0.036)
370 (0.39)
2395 (0.036)
936 (0.14)
341 (0.43)
#SAKNAS!
#SAKNAS!
1164 (0.11)

#SAKNAS!
#SAKNAS!
2395 (0.036)
#SAKNAS!
1573 (0.071)
#SAKNAS!
#SAKNAS!
2395 (0.036)
#SAKNAS!
2395 (0.036)
#SAKNAS!
1164 (0.11)
#SAKNAS!
#SAKNAS!
#SAKNAS!
#SAKNAS!
#SAKNAS!
561 (0.25)
2395 (0.036)
#SAKNAS!
2395 (0.036)
370 (0.39)
2395 (0.036)
648 (0.21)
#SAKNAS!
1573 (0.071)
2395 (0.036)
1164 (0.11)
1573 (0.071)
1573 (0.071)
1573 (0.071)
#SAKNAS!
#SAKNAS!
1573 (0.071)
2395 (0.036)
2395 (0.036)
2395 (0.036)
773 (0.18)
561 (0.25)
#SAKNAS!
1573 (0.071)
1573 (0.071)
301 (0.5)

2395 (0.036)
2395 (0.036)
1573 (0.071)
2395 (0.036)
#SAKNAS!
#SAKNAS!
#SAKNAS!
1573 (0.071)
2395 (0.036)
#SAKNAS!
1573 (0.071)
1573 (0.071)
1164 (0.11)
2395 (0.036)
1573 (0.071)
1164 (0.11)
1164 (0.11)
#SAKNAS!
1164 (0.11)
2395 (0.036)
2395 (0.036)
2395 (0.036)
2395 (0.036)
1573 (0.071)
1573 (0.071)
1573 (0.071)
1573 (0.071)
#SAKNAS!
2395 (0.036)
1573 (0.071)
2395 (0.036)
#SAKNAS!
#SAKNAS!
#SAKNAS!
#SAKNAS!
#SAKNAS!
#SAKNAS!
2395 (0.036)
2395 (0.036)
#SAKNAS!
936 (0.14)
#SAKNAS!
1573 (0.071)

2395 (0.036)
1573 (0.071)
#SAKNAS!
#SAKNAS!
1164 (0.11)
370 (0.39)
#SAKNAS!
1573 (0.071)
1573 (0.071)
2395 (0.036)
2395 (0.036)
1573 (0.071)
#SAKNAS!
#SAKNAS!
1164 (0.11)
#SAKNAS!
1573 (0.071)
1573 (0.071)
1573 (0.071)
936 (0.14)
2395 (0.036)
648 (0.21)
2395 (0.036)
1573 (0.071)
1573 (0.071)
1573 (0.071)
2395 (0.036)
#SAKNAS!
#SAKNAS!
1573 (0.071)
1573 (0.071)
#SAKNAS!
#SAKNAS!
#SAKNAS!
1573 (0.071)
#SAKNAS!
#SAKNAS!
2395 (0.036)
1164 (0.11)
#SAKNAS!
1573 (0.071)
648 (0.21)
2395 (0.036)

2395 (0.036)
1164 (0.11)
936 (0.14)
#SAKNAS!
#SAKNAS!
#SAKNAS!
#SAKNAS!
1573 (0.071)
#SAKNAS!
2395 (0.036)
2395 (0.036)
1573 (0.071)
#SAKNAS!
#SAKNAS!
2395 (0.036)
446 (0.32)
1573 (0.071)
#SAKNAS!
1573 (0.071)
936 (0.14)
#SAKNAS!
1164 (0.11)
1573 (0.071)
#SAKNAS!
936 (0.14)
#SAKNAS!
1573 (0.071)
#SAKNAS!
2395 (0.036)
1573 (0.071)
1164 (0.11)
#SAKNAS!
#SAKNAS!
#SAKNAS!
#SAKNAS!
1573 (0.071)
2395 (0.036)
#SAKNAS!
2395 (0.036)
1573 (0.071)
#SAKNAS!
#SAKNAS!
1164 (0.11)

2395 (0.036)
1573 (0.071)
2395 (0.036)
#SAKNAS!
1164 (0.11)
2395 (0.036)
1573 (0.071)
446 (0.32)
2395 (0.036)
#SAKNAS!
#SAKNAS!
#SAKNAS!
#SAKNAS!
1573 (0.071)
#SAKNAS!
#SAKNAS!
#SAKNAS!
#SAKNAS!
#SAKNAS!
1573 (0.071)
1573 (0.071)
2395 (0.036)
2395 (0.036)
2395 (0.036)
2395 (0.036)
2395 (0.036)
2395 (0.036)
#SAKNAS!
2395 (0.036)
#SAKNAS!
#SAKNAS!
#SAKNAS!
#SAKNAS!
#SAKNAS!
2395 (0.036)
#SAKNAS!
#SAKNAS!
#SAKNAS!
#SAKNAS!
#SAKNAS!
#SAKNAS!
#SAKNAS!
#SAKNAS!

1573 (0.071)
#SAKNAS!
2395 (0.036)
1573 (0.071)
#SAKNAS!
1573 (0.071)
1573 (0.071)
1573 (0.071)
2395 (0.036)
#SAKNAS!
2395 (0.036)
2395 (0.036)
1164 (0.11)
#SAKNAS!
#SAKNAS!
#SAKNAS!
2395 (0.036)
2395 (0.036)
2395 (0.036)
1573 (0.071)
#SAKNAS!
2395 (0.036)
#SAKNAS!
#SAKNAS!
#SAKNAS!
#SAKNAS!
#SAKNAS!
#SAKNAS!
#SAKNAS!
#SAKNAS!
#SAKNAS!
2395 (0.036)
#SAKNAS!
#SAKNAS!
2395 (0.036)
1573 (0.071)
#SAKNAS!
1573 (0.071)
#SAKNAS!
1573 (0.071)
1573 (0.071)
1573 (0.071)
#SAKNAS!

1573 (0.071)
2395 (0.036)
#SAKNAS!
#SAKNAS!
#SAKNAS!
1573 (0.071)
773 (0.18)
2395 (0.036)
2395 (0.036)
#SAKNAS!
#SAKNAS!
2395 (0.036)
#SAKNAS!
#SAKNAS!
1573 (0.071)
2395 (0.036)
#SAKNAS!
2395 (0.036)
2395 (0.036)
#SAKNAS!
#SAKNAS!
#SAKNAS!
2395 (0.036)
2395 (0.036)
#SAKNAS!
#SAKNAS!
2395 (0.036)
#SAKNAS!
2395 (0.036)
2395 (0.036)
1164 (0.11)
2395 (0.036)
2395 (0.036)
2395 (0.036)
#SAKNAS!
773 (0.18)
1573 (0.071)
#SAKNAS!
2395 (0.036)
2395 (0.036)
#SAKNAS!
1573 (0.071)
2395 (0.036)

2395 (0.036)
#SAKNAS!
#SAKNAS!
#SAKNAS!
936 (0.14)
#SAKNAS!
1164 (0.11)
#SAKNAS!
2395 (0.036)
#SAKNAS!
#SAKNAS!
2395 (0.036)
#SAKNAS!
1573 (0.071)
#SAKNAS!
#SAKNAS!
#SAKNAS!
#SAKNAS!
#SAKNAS!
#SAKNAS!
2395 (0.036)
#SAKNAS!
#SAKNAS!
2395 (0.036)
2395 (0.036)
2395 (0.036)
1573 (0.071)
2395 (0.036)
#SAKNAS!
#SAKNAS!
#SAKNAS!
1573 (0.071)
2395 (0.036)
#SAKNAS!
2395 (0.036)
#SAKNAS!
2395 (0.036)
#SAKNAS!
2395 (0.036)
#SAKNAS!
2395 (0.036)
#SAKNAS!
2395 (0.036)

#SAKNAS!
#SAKNAS!
1573 (0.071)
#SAKNAS!
#SAKNAS!
2395 (0.036)
1573 (0.071)
1573 (0.071)
2395 (0.036)
#SAKNAS!
2395 (0.036)
#SAKNAS!
#SAKNAS!
2395 (0.036)
2395 (0.036)
#SAKNAS!
#SAKNAS!
#SAKNAS!
#SAKNAS!
1573 (0.071)
2395 (0.036)
#SAKNAS!
#SAKNAS!
#SAKNAS!
#SAKNAS!
#SAKNAS!
#SAKNAS!
#SAKNAS!
1573 (0.071)
#SAKNAS!
2395 (0.036)
#SAKNAS!
#SAKNAS!
2395 (0.036)
1573 (0.071)
2395 (0.036)
2395 (0.036)
2395 (0.036)
#SAKNAS!
#SAKNAS!
#SAKNAS!
2395 (0.036)
2395 (0.036)

2395 (0.036)
2395 (0.036)
2395 (0.036)
#SAKNAS!
#SAKNAS!
#SAKNAS!
#SAKNAS!
#SAKNAS!
2395 (0.036)
2395 (0.036)
#SAKNAS!
#SAKNAS!
2395 (0.036)
2395 (0.036)
2395 (0.036)
446 (0.32)
#SAKNAS!
#SAKNAS!
#SAKNAS!
2395 (0.036)
1573 (0.071)
#SAKNAS!
#SAKNAS!
2395 (0.036)
1573 (0.071)
2395 (0.036)
2395 (0.036)
#SAKNAS!
#SAKNAS!
#SAKNAS!
#SAKNAS!
#SAKNAS!
2395 (0.036)
#SAKNAS!
#SAKNAS!
#SAKNAS!
1573 (0.071)
2395 (0.036)
2395 (0.036)
#SAKNAS!
#SAKNAS!
2395 (0.036)
#SAKNAS!

2395 (0.036)
#SAKNAS!
#SAKNAS!
#SAKNAS!
#SAKNAS!
#SAKNAS!
2395 (0.036)
#SAKNAS!
#SAKNAS!
#SAKNAS!
#SAKNAS!
2395 (0.036)
2395 (0.036)
#SAKNAS!
#SAKNAS!
#SAKNAS!
#SAKNAS!
#SAKNAS!
1573 (0.071)
1573 (0.071)
2395 (0.036)
#SAKNAS!
#SAKNAS!
#SAKNAS!
#SAKNAS!
1573 (0.071)
#SAKNAS!
648 (0.21)
2395 (0.036)
1573 (0.071)
#SAKNAS!
2395 (0.036)
#SAKNAS!
#SAKNAS!
1573 (0.071)
#SAKNAS!
#SAKNAS!
#SAKNAS!
2395 (0.036)
#SAKNAS!
#SAKNAS!
2395 (0.036)
2395 (0.036)

773 (0.18)
#SAKNAS!
#SAKNAS!
2395 (0.036)
1573 (0.071)
#SAKNAS!
#SAKNAS!
#SAKNAS!
936 (0.14)
561 (0.25)
#SAKNAS!
#SAKNAS!
#SAKNAS!
#SAKNAS!
#SAKNAS!
936 (0.14)
#SAKNAS!
#SAKNAS!
#SAKNAS!
1573 (0.071)
1573 (0.071)
2395 (0.036)
#SAKNAS!
#SAKNAS!
#SAKNAS!
#SAKNAS!
2395 (0.036)
#SAKNAS!
2395 (0.036)
#SAKNAS!
2395 (0.036)
1573 (0.071)
2395 (0.036)
411 (0.36)
#SAKNAS!
2395 (0.036)
1573 (0.071)
2395 (0.036)
1573 (0.071)
2395 (0.036)
2395 (0.036)
1573 (0.071)
#SAKNAS!

#SAKNAS!
#SAKNAS!
#SAKNAS!
1573 (0.071)
#SAKNAS!
#SAKNAS!
#SAKNAS!
1573 (0.071)
#SAKNAS!
2395 (0.036)
1573 (0.071)
#SAKNAS!
#SAKNAS!
2395 (0.036)
1573 (0.071)
#SAKNAS!
1573 (0.071)
2395 (0.036)
#SAKNAS!
#SAKNAS!
2395 (0.036)
2395 (0.036)
2395 (0.036)
#SAKNAS!
1573 (0.071)
#SAKNAS!
#SAKNAS!
1573 (0.071)
#SAKNAS!
2395 (0.036)
2395 (0.036)
#SAKNAS!
#SAKNAS!
#SAKNAS!
#SAKNAS!
1164 (0.11)
#SAKNAS!
2395 (0.036)
#SAKNAS!
#SAKNAS!
#SAKNAS!
1573 (0.071)
2395 (0.036)

648 (0.21)
#SAKNAS!
#SAKNAS!
1573 (0.071)
2395 (0.036)
1164 (0.11)
2395 (0.036)
#SAKNAS!
#SAKNAS!
2395 (0.036)
#SAKNAS!
#SAKNAS!
#SAKNAS!
#SAKNAS!
#SAKNAS!
#SAKNAS!
#SAKNAS!
#SAKNAS!
2395 (0.036)
#SAKNAS!
2395 (0.036)
#SAKNAS!
#SAKNAS!
2395 (0.036)
1573 (0.071)
2395 (0.036)
1573 (0.071)
#SAKNAS!
#SAKNAS!
#SAKNAS!
2395 (0.036)
2395 (0.036)
2395 (0.036)
#SAKNAS!
#SAKNAS!
1573 (0.071)
#SAKNAS!
#SAKNAS!
#SAKNAS!
#SAKNAS!
#SAKNAS!
#SAKNAS!
#SAKNAS!

#SAKNAS!
#SAKNAS!
1164 (0.11)
#SAKNAS!
#SAKNAS!
#SAKNAS!
2395 (0.036)
#SAKNAS!
#SAKNAS!
2395 (0.036)
#SAKNAS!
#SAKNAS!
#SAKNAS!
#SAKNAS!
#SAKNAS!
#SAKNAS!
#SAKNAS!
2395 (0.036)
#SAKNAS!
#SAKNAS!
2395 (0.036)
2395 (0.036)
#SAKNAS!
#SAKNAS!
#SAKNAS!
2395 (0.036)
1573 (0.071)
2395 (0.036)
#SAKNAS!
#SAKNAS!
#SAKNAS!
#SAKNAS!
2395 (0.036)
2395 (0.036)
#SAKNAS!
#SAKNAS!
#SAKNAS!
#SAKNAS!
#SAKNAS!
2395 (0.036)
#SAKNAS!
#SAKNAS!
2395 (0.036)

#SAKNAS!
936 (0.14)
#SAKNAS!
#SAKNAS!
1573 (0.071)
2395 (0.036)
#SAKNAS!
#SAKNAS!
2395 (0.036)
1573 (0.071)
2395 (0.036)
2395 (0.036)
#SAKNAS!
648 (0.21)
2395 (0.036)
#SAKNAS!
2395 (0.036)
#SAKNAS!
2395 (0.036)
1164 (0.11)
2395 (0.036)
2395 (0.036)
#SAKNAS!
#SAKNAS!
2395 (0.036)
1573 (0.071)
1573 (0.071)
1573 (0.071)
2395 (0.036)
2395 (0.036)
#SAKNAS!
2395 (0.036)
2395 (0.036)
#SAKNAS!
#SAKNAS!
#SAKNAS!
2395 (0.036)
#SAKNAS!
2395 (0.036)
1573 (0.071)
#SAKNAS!
2395 (0.036)
#SAKNAS!

1573 (0.071)
2395 (0.036)
1573 (0.071)
#SAKNAS!
936 (0.14)
2395 (0.036)
2395 (0.036)
1164 (0.11)
1573 (0.071)
#SAKNAS!
2395 (0.036)
2395 (0.036)
#SAKNAS!
#SAKNAS!
2395 (0.036)
1573 (0.071)
773 (0.18)
#SAKNAS!
561 (0.25)
2395 (0.036)
2395 (0.036)
2395 (0.036)
#SAKNAS!
#SAKNAS!
2395 (0.036)
1573 (0.071)
2395 (0.036)
#SAKNAS!
#SAKNAS!
#SAKNAS!
2395 (0.036)
#SAKNAS!
2395 (0.036)
2395 (0.036)
2395 (0.036)
#SAKNAS!
#SAKNAS!
#SAKNAS!
1573 (0.071)
#SAKNAS!
#SAKNAS!
#SAKNAS!
1573 (0.071)

#SAKNAS!
#SAKNAS!
2395 (0.036)
#SAKNAS!
1573 (0.071)
2395 (0.036)
1573 (0.071)
2395 (0.036)
#SAKNAS!
#SAKNAS!
#SAKNAS!
#SAKNAS!
#SAKNAS!
2395 (0.036)
2395 (0.036)
#SAKNAS!
1573 (0.071)
#SAKNAS!
1573 (0.071)
1573 (0.071)
2395 (0.036)
#SAKNAS!
#SAKNAS!
1573 (0.071)
#SAKNAS!
648 (0.21)
#SAKNAS!
#SAKNAS!
#SAKNAS!
1164 (0.11)
#SAKNAS!
#SAKNAS!
#SAKNAS!
2395 (0.036)
1573 (0.071)
2395 (0.036)
936 (0.14)
#SAKNAS!
1573 (0.071)
2395 (0.036)
#SAKNAS!
#SAKNAS!
#SAKNAS!

#SAKNAS!
2395 (0.036)
#SAKNAS!
#SAKNAS!
#SAKNAS!
#SAKNAS!
#SAKNAS!
2395 (0.036)
2395 (0.036)
#SAKNAS!
2395 (0.036)
2395 (0.036)
2395 (0.036)
2395 (0.036)
1164 (0.11)
773 (0.18)
#SAKNAS!
2395 (0.036)
2395 (0.036)
1573 (0.071)
#SAKNAS!
2395 (0.036)
2395 (0.036)
#SAKNAS!
#SAKNAS!
1573 (0.071)
2395 (0.036)
#SAKNAS!
#SAKNAS!
2395 (0.036)
#SAKNAS!
#SAKNAS!
#SAKNAS!
2395 (0.036)
1164 (0.11)
2395 (0.036)
1573 (0.071)
1573 (0.071)
936 (0.14)
#SAKNAS!
#SAKNAS!
1573 (0.071)
1573 (0.071)

2395 (0.036)
#SAKNAS!
#SAKNAS!
2395 (0.036)
2395 (0.036)
#SAKNAS!
2395 (0.036)
2395 (0.036)
#SAKNAS!
#SAKNAS!
2395 (0.036)
2395 (0.036)
1164 (0.11)
#SAKNAS!
#SAKNAS!
2395 (0.036)
#SAKNAS!
2395 (0.036)
#SAKNAS!
#SAKNAS!
2395 (0.036)
#SAKNAS!
936 (0.14)
#SAKNAS!
2395 (0.036)
#SAKNAS!
#SAKNAS!
#SAKNAS!
#SAKNAS!
2395 (0.036)
2395 (0.036)
2395 (0.036)
2395 (0.036)
#SAKNAS!
#SAKNAS!
#SAKNAS!
#SAKNAS!
2395 (0.036)
2395 (0.036)
2395 (0.036)
#SAKNAS!
#SAKNAS!
2395 (0.036)

#SAKNAS!
#SAKNAS!
2395 (0.036)
561 (0.25)
2395 (0.036)
#SAKNAS!
#SAKNAS!
#SAKNAS!
#SAKNAS!
#SAKNAS!
#SAKNAS!
#SAKNAS!
#SAKNAS!
#SAKNAS!
#SAKNAS!
773 (0.18)
#SAKNAS!
#SAKNAS!
#SAKNAS!
341 (0.43)
#SAKNAS!
#SAKNAS!
#SAKNAS!
#SAKNAS!
#SAKNAS!
#SAKNAS!
#SAKNAS!
#SAKNAS!
#SAKNAS!
#SAKNAS!
2395 (0.036)
506 (0.28)
#SAKNAS!
#SAKNAS!
#SAKNAS!
2395 (0.036)
#SAKNAS!
#SAKNAS!
1573 (0.071)
#SAKNAS!
#SAKNAS!
#SAKNAS!
#SAKNAS!

1573 (0.071)
#SAKNAS!
2395 (0.036)
2395 (0.036)
#SAKNAS!
#SAKNAS!
#SAKNAS!
#SAKNAS!
#SAKNAS!
#SAKNAS!
1573 (0.071)
#SAKNAS!
#SAKNAS!
1573 (0.071)
1164 (0.11)
1573 (0.071)
1573 (0.071)
#SAKNAS!
#SAKNAS!
2395 (0.036)
#SAKNAS!
#SAKNAS!
2395 (0.036)
1573 (0.071)
#SAKNAS!
1573 (0.071)
#SAKNAS!
1573 (0.071)
#SAKNAS!
#SAKNAS!
#SAKNAS!
#SAKNAS!
2395 (0.036)
#SAKNAS!
#SAKNAS!
2395 (0.036)
#SAKNAS!
#SAKNAS!
2395 (0.036)
1573 (0.071)
1164 (0.11)
506 (0.28)
#SAKNAS!

#SAKNAS!
#SAKNAS!
#SAKNAS!
2395 (0.036)
#SAKNAS!
#SAKNAS!
2395 (0.036)
2395 (0.036)
1573 (0.071)
2395 (0.036)
#SAKNAS!
2395 (0.036)
#SAKNAS!
#SAKNAS!
#SAKNAS!
#SAKNAS!
1573 (0.071)
2395 (0.036)
2395 (0.036)
1573 (0.071)
1164 (0.11)
1573 (0.071)
2395 (0.036)
#SAKNAS!
1573 (0.071)
#SAKNAS!
#SAKNAS!
2395 (0.036)
#SAKNAS!
#SAKNAS!
1573 (0.071)
1573 (0.071)
#SAKNAS!
#SAKNAS!
1573 (0.071)
2395 (0.036)
1573 (0.071)
#SAKNAS!
#SAKNAS!
#SAKNAS!
#SAKNAS!
#SAKNAS!
#SAKNAS!

#SAKNAS!
#SAKNAS!
#SAKNAS!
1573 (0.071)
#SAKNAS!
2395 (0.036)
#SAKNAS!
#SAKNAS!
#SAKNAS!
#SAKNAS!
2395 (0.036)
1573 (0.071)
1164 (0.11)
#SAKNAS!
#SAKNAS!
1573 (0.071)
#SAKNAS!
1573 (0.071)
#SAKNAS!
506 (0.28)
2395 (0.036)
#SAKNAS!
#SAKNAS!
1573 (0.071)
#SAKNAS!
#SAKNAS!
#SAKNAS!
1573 (0.071)
2395 (0.036)
#SAKNAS!
1164 (0.11)
#SAKNAS!
#SAKNAS!
#SAKNAS!
#SAKNAS!
2395 (0.036)
2395 (0.036)
2395 (0.036)
#SAKNAS!
#SAKNAS!
#SAKNAS!
#SAKNAS!
2395 (0.036)

2395 (0.036)
2395 (0.036)
2395 (0.036)
2395 (0.036)
#SAKNAS!
#SAKNAS!
#SAKNAS!
#SAKNAS!
#SAKNAS!
506 (0.28)
#SAKNAS!
2395 (0.036)
2395 (0.036)
2395 (0.036)
2395 (0.036)
#SAKNAS!
#SAKNAS!
#SAKNAS!
#SAKNAS!
#SAKNAS!
1573 (0.071)
#SAKNAS!
#SAKNAS!
#SAKNAS!
1573 (0.071)
#SAKNAS!
#SAKNAS!
#SAKNAS!
319 (0.46)
1164 (0.11)
#SAKNAS!
2395 (0.036)
2395 (0.036)
#SAKNAS!
#SAKNAS!
2395 (0.036)
2395 (0.036)
#SAKNAS!
#SAKNAS!
2395 (0.036)
2395 (0.036)
#SAKNAS!
1573 (0.071)

#SAKNAS!
#SAKNAS!
#SAKNAS!
2395 (0.036)
2395 (0.036)
2395 (0.036)
506 (0.28)
#SAKNAS!
#SAKNAS!
#SAKNAS!
#SAKNAS!
#SAKNAS!
1573 (0.071)
1573 (0.071)
1573 (0.071)
#SAKNAS!
#SAKNAS!
#SAKNAS!
#SAKNAS!
#SAKNAS!
#SAKNAS!
2395 (0.036)
#SAKNAS!
1573 (0.071)
2395 (0.036)
#SAKNAS!
1573 (0.071)
561 (0.25)
#SAKNAS!
#SAKNAS!
506 (0.28)
#SAKNAS!
#SAKNAS!
#SAKNAS!
#SAKNAS!
#SAKNAS!
#SAKNAS!
#SAKNAS!
#SAKNAS!
2395 (0.036)
1573 (0.071)
2395 (0.036)
#SAKNAS!

1573 (0.071)
1573 (0.071)
#SAKNAS!
#SAKNAS!
2395 (0.036)
#SAKNAS!
#SAKNAS!
2395 (0.036)
#SAKNAS!
#SAKNAS!
#SAKNAS!
1573 (0.071)
#SAKNAS!
2395 (0.036)
#SAKNAS!
#SAKNAS!
2395 (0.036)
1573 (0.071)
#SAKNAS!
#SAKNAS!
1573 (0.071)
2395 (0.036)
2395 (0.036)
1573 (0.071)
#SAKNAS!
#SAKNAS!
2395 (0.036)
#SAKNAS!
#SAKNAS!
#SAKNAS!
#SAKNAS!
#SAKNAS!
#SAKNAS!
#SAKNAS!
1573 (0.071)
#SAKNAS!
#SAKNAS!
#SAKNAS!
#SAKNAS!
2395 (0.036)
#SAKNAS!
1573 (0.071)
2395 (0.036)

#SAKNAS!
#SAKNAS!
#SAKNAS!
75 (1.8)
#SAKNAS!
#SAKNAS!
1573 (0.071)
2395 (0.036)
1573 (0.071)
#SAKNAS!
#SAKNAS!
#SAKNAS!
1164 (0.11)
#SAKNAS!
#SAKNAS!
#SAKNAS!
341 (0.43)
#SAKNAS!
2395 (0.036)
2395 (0.036)
#SAKNAS!
#SAKNAS!
2395 (0.036)
#SAKNAS!
1573 (0.071)
2395 (0.036)
2395 (0.036)
2395 (0.036)
2395 (0.036)
#SAKNAS!
#SAKNAS!
#SAKNAS!
2395 (0.036)
2395 (0.036)
#SAKNAS!
2395 (0.036)
#SAKNAS!
#SAKNAS!
1573 (0.071)
1573 (0.071)
#SAKNAS!
#SAKNAS!
2395 (0.036)

#SAKNAS!
#SAKNAS!
#SAKNAS!
#SAKNAS!
2395 (0.036)
2395 (0.036)
#SAKNAS!
#SAKNAS!
2395 (0.036)
1573 (0.071)
#SAKNAS!
#SAKNAS!
#SAKNAS!
446 (0.32)
#SAKNAS!
#SAKNAS!
2395 (0.036)
#SAKNAS!
#SAKNAS!
2395 (0.036)
#SAKNAS!
#SAKNAS!
2395 (0.036)
#SAKNAS!
2395 (0.036)
936 (0.14)
#SAKNAS!
#SAKNAS!
#SAKNAS!
2395 (0.036)
#SAKNAS!
#SAKNAS!
#SAKNAS!
1573 (0.071)
1164 (0.11)
#SAKNAS!
#SAKNAS!
#SAKNAS!
#SAKNAS!
#SAKNAS!
#SAKNAS!
2395 (0.036)
2395 (0.036)

2395 (0.036)
#SAKNAS!
2395 (0.036)
2395 (0.036)
#SAKNAS!
#SAKNAS!
1573 (0.071)
#SAKNAS!
773 (0.18)
2395 (0.036)
#SAKNAS!
1573 (0.071)
#SAKNAS!
#SAKNAS!
#SAKNAS!
#SAKNAS!
#SAKNAS!
#SAKNAS!
#SAKNAS!
#SAKNAS!
#SAKNAS!
#SAKNAS!
2395 (0.036)
#SAKNAS!
#SAKNAS!
2395 (0.036)
1573 (0.071)
2395 (0.036)
#SAKNAS!
2395 (0.036)
1164 (0.11)
#SAKNAS!
1573 (0.071)
#SAKNAS!
2395 (0.036)
1573 (0.071)
2395 (0.036)
#SAKNAS!
#SAKNAS!
411 (0.36)
#SAKNAS!
#SAKNAS!
#SAKNAS!

#SAKNAS!
2395 (0.036)
1573 (0.071)
#SAKNAS!
#SAKNAS!
#SAKNAS!
#SAKNAS!
#SAKNAS!
#SAKNAS!
#SAKNAS!
#SAKNAS!
2395 (0.036)
#SAKNAS!
#SAKNAS!
2395 (0.036)
2395 (0.036)
#SAKNAS!
#SAKNAS!
#SAKNAS!
#SAKNAS!
#SAKNAS!
2395 (0.036)
1164 (0.11)
#SAKNAS!
#SAKNAS!
561 (0.25)
#SAKNAS!
#SAKNAS!
1573 (0.071)
648 (0.21)
#SAKNAS!
#SAKNAS!
#SAKNAS!
2395 (0.036)
2395 (0.036)
#SAKNAS!
#SAKNAS!
#SAKNAS!
#SAKNAS!
2395 (0.036)
#SAKNAS!
#SAKNAS!
2395 (0.036)

2395 (0.036)
#SAKNAS!
#SAKNAS!
2395 (0.036)
2395 (0.036)
2395 (0.036)
#SAKNAS!
#SAKNAS!
2395 (0.036)
936 (0.14)
#SAKNAS!
1164 (0.11)
#SAKNAS!
1573 (0.071)
1573 (0.071)
1573 (0.071)
1573 (0.071)
2395 (0.036)
2395 (0.036)
648 (0.21)
2395 (0.036)
2395 (0.036)
1164 (0.11)
#SAKNAS!
2395 (0.036)
1164 (0.11)
648 (0.21)
2395 (0.036)
1573 (0.071)
#SAKNAS!
2395 (0.036)
936 (0.14)
2395 (0.036)
1573 (0.071)
506 (0.28)
#SAKNAS!
#SAKNAS!
2395 (0.036)
936 (0.14)
#SAKNAS!
1573 (0.071)
2395 (0.036)
261 (0.57)

2395 (0.036)
1573 (0.071)
#SAKNAS!
2395 (0.036)
2395 (0.036)
1573 (0.071)
#SAKNAS!
#SAKNAS!
#SAKNAS!
1573 (0.071)
1573 (0.071)
2395 (0.036)
648 (0.21)
#SAKNAS!
2395 (0.036)
#SAKNAS!
#SAKNAS!
#SAKNAS!
2395 (0.036)
#SAKNAS!
2395 (0.036)
1573 (0.071)
#SAKNAS!
#SAKNAS!
2395 (0.036)
2395 (0.036)
#SAKNAS!
#SAKNAS!
1164 (0.11)
1164 (0.11)
#SAKNAS!
2395 (0.036)
#SAKNAS!
2395 (0.036)
2395 (0.036)
2395 (0.036)
2395 (0.036)
2395 (0.036)
1573 (0.071)
#SAKNAS!
#SAKNAS!
2395 (0.036)
#SAKNAS!

2395 (0.036)
#SAKNAS!
#SAKNAS!
#SAKNAS!
1573 (0.071)
1573 (0.071)
1573 (0.071)
#SAKNAS!
#SAKNAS!
#SAKNAS!
1573 (0.071)
#SAKNAS!
#SAKNAS!
#SAKNAS!
2395 (0.036)
1164 (0.11)
#SAKNAS!
2395 (0.036)
#SAKNAS!
2395 (0.036)
2395 (0.036)
2395 (0.036)
2395 (0.036)
2395 (0.036)
1164 (0.11)
#SAKNAS!
#SAKNAS!
#SAKNAS!
1573 (0.071)
#SAKNAS!
2395 (0.036)
1573 (0.071)
#SAKNAS!
2395 (0.036)
2395 (0.036)
2395 (0.036)
#SAKNAS!
2395 (0.036)
2395 (0.036)
1573 (0.071)
2395 (0.036)
2395 (0.036)
#SAKNAS!

84 (1.6)
773 (0.18)
#SAKNAS!
#SAKNAS!
#SAKNAS!
1573 (0.071)
#SAKNAS!
#SAKNAS!
2395 (0.036)
#SAKNAS!
#SAKNAS!
#SAKNAS!
#SAKNAS!
#SAKNAS!
2395 (0.036)
2395 (0.036)
#SAKNAS!
#SAKNAS!
#SAKNAS!
#SAKNAS!
1573 (0.071)
2395 (0.036)
2395 (0.036)
1573 (0.071)
#SAKNAS!
2395 (0.036)
1573 (0.071)
#SAKNAS!
2395 (0.036)
2395 (0.036)
1573 (0.071)
2395 (0.036)
#SAKNAS!
1573 (0.071)
2395 (0.036)
1573 (0.071)
#SAKNAS!
#SAKNAS!
#SAKNAS!
#SAKNAS!
#SAKNAS!
1573 (0.071)
#SAKNAS!

#SAKNAS!
#SAKNAS!
#SAKNAS!
#SAKNAS!
1573 (0.071)
#SAKNAS!
#SAKNAS!
2395 (0.036)
#SAKNAS!
#SAKNAS!
2395 (0.036)
#SAKNAS!
#SAKNAS!
2395 (0.036)
1164 (0.11)
1573 (0.071)
2395 (0.036)
936 (0.14)
#SAKNAS!
#SAKNAS!
1573 (0.071)
1164 (0.11)
#SAKNAS!
2395 (0.036)
#SAKNAS!
#SAKNAS!
2395 (0.036)
#SAKNAS!
#SAKNAS!
2395 (0.036)
#SAKNAS!
#SAKNAS!
#SAKNAS!
#SAKNAS!
1573 (0.071)
#SAKNAS!
2395 (0.036)
#SAKNAS!
#SAKNAS!
#SAKNAS!
2395 (0.036)
2395 (0.036)
#SAKNAS!

#SAKNAS!
#SAKNAS!
#SAKNAS!
#SAKNAS!
1573 (0.071)
2395 (0.036)
#SAKNAS!
#SAKNAS!
#SAKNAS!
2395 (0.036)
#SAKNAS!
2395 (0.036)
1573 (0.071)
1573 (0.071)
1573 (0.071)
2395 (0.036)
2395 (0.036)
#SAKNAS!
2395 (0.036)
#SAKNAS!
#SAKNAS!
1164 (0.11)
#SAKNAS!
#SAKNAS!
1573 (0.071)
#SAKNAS!
#SAKNAS!
#SAKNAS!
#SAKNAS!
#SAKNAS!
#SAKNAS!
2395 (0.036)
2395 (0.036)
2395 (0.036)
#SAKNAS!
#SAKNAS!
2395 (0.036)
2395 (0.036)
2395 (0.036)
#SAKNAS!
#SAKNAS!
2395 (0.036)
#SAKNAS!

#SAKNAS!
#SAKNAS!
2395 (0.036)
1573 (0.071)
936 (0.14)
2395 (0.036)
2395 (0.036)
2395 (0.036)
2395 (0.036)
936 (0.14)
2395 (0.036)
2395 (0.036)
#SAKNAS!
#SAKNAS!
2395 (0.036)
1573 (0.071)
#SAKNAS!
#SAKNAS!
#SAKNAS!
#SAKNAS!
561 (0.25)
#SAKNAS!
#SAKNAS!
1573 (0.071)
#SAKNAS!
#SAKNAS!
2395 (0.036)
#SAKNAS!
2395 (0.036)
2395 (0.036)
#SAKNAS!
1573 (0.071)
#SAKNAS!
#SAKNAS!
#SAKNAS!
#SAKNAS!
#SAKNAS!
2395 (0.036)
#SAKNAS!
2395 (0.036)
#SAKNAS!
1573 (0.071)
2395 (0.036)

#SAKNAS!
#SAKNAS!
1573 (0.071)
187 (0.75)
1573 (0.071)
2395 (0.036)
#SAKNAS!
2395 (0.036)
#SAKNAS!
#SAKNAS!
#SAKNAS!
#SAKNAS!
2395 (0.036)
2395 (0.036)
1164 (0.11)
1164 (0.11)
#SAKNAS!
#SAKNAS!
2395 (0.036)
2395 (0.036)
#SAKNAS!
2395 (0.036)
648 (0.21)
2395 (0.036)
#SAKNAS!
1573 (0.071)
2395 (0.036)
2395 (0.036)
2395 (0.036)
648 (0.21)
2395 (0.036)
2395 (0.036)
2395 (0.036)
936 (0.14)
2395 (0.036)
648 (0.21)
2395 (0.036)
#SAKNAS!
1573 (0.071)
2395 (0.036)
2395 (0.036)
#SAKNAS!
#SAKNAS!

#SAKNAS!
#SAKNAS!
#SAKNAS!
1573 (0.071)
2395 (0.036)
#SAKNAS!
#SAKNAS!
2395 (0.036)
#SAKNAS!
648 (0.21)
1573 (0.071)
2395 (0.036)
2395 (0.036)
2395 (0.036)
1573 (0.071)
2395 (0.036)
#SAKNAS!
#SAKNAS!
1164 (0.11)
#SAKNAS!
446 (0.32)
1573 (0.071)
2395 (0.036)
1573 (0.071)
1573 (0.071)
#SAKNAS!
1573 (0.071)
1573 (0.071)
2395 (0.036)
2395 (0.036)
2395 (0.036)
2395 (0.036)
2395 (0.036)
#SAKNAS!
#SAKNAS!
#SAKNAS!
#SAKNAS!
1164 (0.11)
2395 (0.036)
2395 (0.036)
2395 (0.036)
2395 (0.036)
2395 (0.036)

#SAKNAS!
#SAKNAS!
#SAKNAS!
936 (0.14)
#SAKNAS!
#SAKNAS!
#SAKNAS!
#SAKNAS!
#SAKNAS!
2395 (0.036)
2395 (0.036)
#SAKNAS!
#SAKNAS!
2395 (0.036)
#SAKNAS!
#SAKNAS!
1573 (0.071)
#SAKNAS!
2395 (0.036)
#SAKNAS!
773 (0.18)
#SAKNAS!
#SAKNAS!
2395 (0.036)
2395 (0.036)
#SAKNAS!
#SAKNAS!
#SAKNAS!
#SAKNAS!
2395 (0.036)
#SAKNAS!
#SAKNAS!
1573 (0.071)
1573 (0.071)
#SAKNAS!
#SAKNAS!
2395 (0.036)
#SAKNAS!
#SAKNAS!
#SAKNAS!
#SAKNAS!
#SAKNAS!
2395 (0.036)

2395 (0.036)
#SAKNAS!
#SAKNAS!
#SAKNAS!
#SAKNAS!
#SAKNAS!
#SAKNAS!
2395 (0.036)
#SAKNAS!
2395 (0.036)
2395 (0.036)
2395 (0.036)
#SAKNAS!
2395 (0.036)
#SAKNAS!
#SAKNAS!
2395 (0.036)
#SAKNAS!
#SAKNAS!
#SAKNAS!
#SAKNAS!
2395 (0.036)
2395 (0.036)
#SAKNAS!
#SAKNAS!
#SAKNAS!
#SAKNAS!
#SAKNAS!
2395 (0.036)
1573 (0.071)
#SAKNAS!
#SAKNAS!
#SAKNAS!
2395 (0.036)
773 (0.18)
2395 (0.036)
#SAKNAS!
2395 (0.036)
773 (0.18)
#SAKNAS!
#SAKNAS!
2395 (0.036)
#SAKNAS!

#SAKNAS!
2395 (0.036)
#SAKNAS!
#SAKNAS!
2395 (0.036)
2395 (0.036)
1573 (0.071)
1164 (0.11)
1573 (0.071)
2395 (0.036)
2395 (0.036)
2395 (0.036)
#SAKNAS!
1164 (0.11)
2395 (0.036)
1573 (0.071)
2395 (0.036)
#SAKNAS!
2395 (0.036)
#SAKNAS!
#SAKNAS!
#SAKNAS!
1573 (0.071)
#SAKNAS!
#SAKNAS!
#SAKNAS!
2395 (0.036)
#SAKNAS!
#SAKNAS!
#SAKNAS!
2395 (0.036)
2395 (0.036)
2395 (0.036)
#SAKNAS!
2395 (0.036)
#SAKNAS!
2395 (0.036)
1573 (0.071)
1573 (0.071)
1573 (0.071)
#SAKNAS!
#SAKNAS!
561 (0.25)

2395 (0.036)
2395 (0.036)
#SAKNAS!
#SAKNAS!
#SAKNAS!
#SAKNAS!
1164 (0.11)
#SAKNAS!
#SAKNAS!
2395 (0.036)
2395 (0.036)
#SAKNAS!
#SAKNAS!
2395 (0.036)
#SAKNAS!
2395 (0.036)
1573 (0.071)
#SAKNAS!
2395 (0.036)
#SAKNAS!
#SAKNAS!
1573 (0.071)
2395 (0.036)
#SAKNAS!
#SAKNAS!
2395 (0.036)
2395 (0.036)
2395 (0.036)
561 (0.25)
2395 (0.036)
#SAKNAS!
#SAKNAS!
#SAKNAS!
1573 (0.071)
2395 (0.036)
#SAKNAS!
#SAKNAS!
2395 (0.036)
#SAKNAS!
#SAKNAS!
#SAKNAS!
#SAKNAS!
2395 (0.036)

#SAKNAS!
#SAKNAS!
#SAKNAS!
#SAKNAS!
#SAKNAS!
2395 (0.036)
936 (0.14)
#SAKNAS!
#SAKNAS!
446 (0.32)
1573 (0.071)
#SAKNAS!
#SAKNAS!
#SAKNAS!
2395 (0.036)
#SAKNAS!
2395 (0.036)
#SAKNAS!
2395 (0.036)
2395 (0.036)
#SAKNAS!
#SAKNAS!
2395 (0.036)
2395 (0.036)
2395 (0.036)
1573 (0.071)
2395 (0.036)
1573 (0.071)
#SAKNAS!
1573 (0.071)
#SAKNAS!
1573 (0.071)
2395 (0.036)
1573 (0.071)
#SAKNAS!
#SAKNAS!
2395 (0.036)
#SAKNAS!
#SAKNAS!
2395 (0.036)
#SAKNAS!
#SAKNAS!
1573 (0.071)

#SAKNAS!
#SAKNAS!
#SAKNAS!
#SAKNAS!
#SAKNAS!
2395 (0.036)
2395 (0.036)
2395 (0.036)
2395 (0.036)
2395 (0.036)
#SAKNAS!
1573 (0.071)
1164 (0.11)
#SAKNAS!
#SAKNAS!
2395 (0.036)
#SAKNAS!
2395 (0.036)
#SAKNAS!
#SAKNAS!
#SAKNAS!
2395 (0.036)
#SAKNAS!
#SAKNAS!
#SAKNAS!
1573 (0.071)
2395 (0.036)
1164 (0.11)
2395 (0.036)
2395 (0.036)
#SAKNAS!
#SAKNAS!
2395 (0.036)
#SAKNAS!
2395 (0.036)
2395 (0.036)
2395 (0.036)
#SAKNAS!
2395 (0.036)
2395 (0.036)
2395 (0.036)
1573 (0.071)
#SAKNAS!

#SAKNAS!
2395 (0.036)
#SAKNAS!
#SAKNAS!
2395 (0.036)
#SAKNAS!
2395 (0.036)
#SAKNAS!
#SAKNAS!
#SAKNAS!
2395 (0.036)
2395 (0.036)
1573 (0.071)
2395 (0.036)
#SAKNAS!
#SAKNAS!
#SAKNAS!
2395 (0.036)
#SAKNAS!
#SAKNAS!
#SAKNAS!
1164 (0.11)
1573 (0.071)
2395 (0.036)
2395 (0.036)
2395 (0.036)
1573 (0.071)
#SAKNAS!
#SAKNAS!
2395 (0.036)
2395 (0.036)
2395 (0.036)
2395 (0.036)
2395 (0.036)
2395 (0.036)
1573 (0.071)
#SAKNAS!
2395 (0.036)
#SAKNAS!
1164 (0.11)
2395 (0.036)
#SAKNAS!
#SAKNAS!

#SAKNAS!
#SAKNAS!
2395 (0.036)
1573 (0.071)
936 (0.14)
#SAKNAS!
2395 (0.036)
2395 (0.036)
2395 (0.036)
#SAKNAS!
#SAKNAS!
2395 (0.036)
1573 (0.071)
648 (0.21)
1573 (0.071)
2395 (0.036)
1573 (0.071)
2395 (0.036)
#SAKNAS!
#SAKNAS!
936 (0.14)
2395 (0.036)
2395 (0.036)
#SAKNAS!
2395 (0.036)
#SAKNAS!
2395 (0.036)
1573 (0.071)
#SAKNAS!
648 (0.21)
2395 (0.036)
#SAKNAS!
#SAKNAS!
#SAKNAS!
773 (0.18)
#SAKNAS!
2395 (0.036)
1573 (0.071)
319 (0.46)
#SAKNAS!
#SAKNAS!
#SAKNAS!
#SAKNAS!

1573 (0.071)
648 (0.21)
2395 (0.036)
#SAKNAS!
#SAKNAS!
2395 (0.036)
#SAKNAS!
2395 (0.036)
2395 (0.036)
#SAKNAS!
#SAKNAS!
#SAKNAS!
2395 (0.036)
2395 (0.036)
1573 (0.071)
#SAKNAS!
2395 (0.036)
2395 (0.036)
1573 (0.071)
#SAKNAS!
#SAKNAS!
#SAKNAS!
2395 (0.036)
2395 (0.036)
2395 (0.036)
2395 (0.036)
2395 (0.036)
#SAKNAS!
#SAKNAS!
#SAKNAS!
#SAKNAS!
2395 (0.036)
#SAKNAS!
#SAKNAS!
2395 (0.036)
#SAKNAS!
#SAKNAS!
#SAKNAS!
#SAKNAS!
1573 (0.071)
#SAKNAS!
2395 (0.036)
2395 (0.036)

#SAKNAS!
#SAKNAS!
2395 (0.036)
2395 (0.036)
#SAKNAS!
2395 (0.036)
#SAKNAS!
#SAKNAS!
#SAKNAS!
#SAKNAS!
2395 (0.036)
1164 (0.11)
#SAKNAS!
#SAKNAS!
2395 (0.036)
2395 (0.036)
2395 (0.036)
1573 (0.071)
#SAKNAS!
#SAKNAS!
#SAKNAS!
2395 (0.036)
2395 (0.036)
#SAKNAS!
#SAKNAS!
#SAKNAS!
2395 (0.036)
2395 (0.036)
#SAKNAS!
2395 (0.036)
#SAKNAS!
773 (0.18)
2395 (0.036)
1573 (0.071)
2395 (0.036)
2395 (0.036)
2395 (0.036)
2395 (0.036)
2395 (0.036)
2395 (0.036)
2395 (0.036)
2395 (0.036)
2395 (0.036)

2395 (0.036)
1573 (0.071)
#SAKNAS!
2395 (0.036)
1573 (0.071)
#SAKNAS!
#SAKNAS!
2395 (0.036)
1573 (0.071)
1573 (0.071)
2395 (0.036)
2395 (0.036)
2395 (0.036)
#SAKNAS!
#SAKNAS!
2395 (0.036)
2395 (0.036)
936 (0.14)
2395 (0.036)
#SAKNAS!
1573 (0.071)
2395 (0.036)
1573 (0.071)
#SAKNAS!
2395 (0.036)
#SAKNAS!
#SAKNAS!
#SAKNAS!
2395 (0.036)
2395 (0.036)
1573 (0.071)
1573 (0.071)
2395 (0.036)
#SAKNAS!
2395 (0.036)
2395 (0.036)
#SAKNAS!
2395 (0.036)
#SAKNAS!
#SAKNAS!
#SAKNAS!
#SAKNAS!
2395 (0.036)

1164 (0.11)
#SAKNAS!
1573 (0.071)
1573 (0.071)
#SAKNAS!
#SAKNAS!
1573 (0.071)
#SAKNAS!
#SAKNAS!
#SAKNAS!
2395 (0.036)
#SAKNAS!
#SAKNAS!
1573 (0.071)
#SAKNAS!
#SAKNAS!
#SAKNAS!
#SAKNAS!
2395 (0.036)
#SAKNAS!
#SAKNAS!
2395 (0.036)
#SAKNAS!
773 (0.18)
#SAKNAS!
#SAKNAS!
2395 (0.036)
#SAKNAS!
#SAKNAS!
2395 (0.036)
2395 (0.036)
2395 (0.036)
#SAKNAS!
2395 (0.036)
#SAKNAS!
#SAKNAS!
#SAKNAS!
1164 (0.11)
#SAKNAS!
#SAKNAS!
2395 (0.036)
#SAKNAS!
1573 (0.071)

#SAKNAS!
2395 (0.036)
2395 (0.036)
2395 (0.036)
2395 (0.036)
#SAKNAS!
#SAKNAS!
1573 (0.071)
#SAKNAS!
1573 (0.071)
301 (0.5)
#SAKNAS!
#SAKNAS!
#SAKNAS!
#SAKNAS!
#SAKNAS!
#SAKNAS!
2395 (0.036)
#SAKNAS!
#SAKNAS!
#SAKNAS!
#SAKNAS!
#SAKNAS!
1573 (0.071)
2395 (0.036)
1573 (0.071)
2395 (0.036)
#SAKNAS!
#SAKNAS!
1573 (0.071)
2395 (0.036)
#SAKNAS!
#SAKNAS!
2395 (0.036)
#SAKNAS!
2395 (0.036)
2395 (0.036)
1573 (0.071)
2395 (0.036)
#SAKNAS!
#SAKNAS!
#SAKNAS!
#SAKNAS!

2395 (0.036)
2395 (0.036)
2395 (0.036)
2395 (0.036)
2395 (0.036)
#SAKNAS!
#SAKNAS!
#SAKNAS!
2395 (0.036)
2395 (0.036)
648 (0.21)
#SAKNAS!
1573 (0.071)
#SAKNAS!
#SAKNAS!
#SAKNAS!
2395 (0.036)
1573 (0.071)
#SAKNAS!
1573 (0.071)
773 (0.18)
936 (0.14)
2395 (0.036)
2395 (0.036)
#SAKNAS!
#SAKNAS!
#SAKNAS!
2395 (0.036)
#SAKNAS!
#SAKNAS!
2395 (0.036)
1573 (0.071)
2395 (0.036)
2395 (0.036)
#SAKNAS!
2395 (0.036)
#SAKNAS!
#SAKNAS!
#SAKNAS!
2395 (0.036)
#SAKNAS!
773 (0.18)
1573 (0.071)

1573 (0.071)
#SAKNAS!
#SAKNAS!
2395 (0.036)
2395 (0.036)
1573 (0.071)
2395 (0.036)
1573 (0.071)
2395 (0.036)
1573 (0.071)
#SAKNAS!
#SAKNAS!
1573 (0.071)
2395 (0.036)
2395 (0.036)
#SAKNAS!
#SAKNAS!
2395 (0.036)
#SAKNAS!
#SAKNAS!
2395 (0.036)
1573 (0.071)
2395 (0.036)
#SAKNAS!
#SAKNAS!
#SAKNAS!
2395 (0.036)
2395 (0.036)
936 (0.14)
#SAKNAS!
#SAKNAS!
2395 (0.036)
#SAKNAS!
#SAKNAS!
1573 (0.071)
#SAKNAS!
#SAKNAS!
#SAKNAS!
#SAKNAS!
#SAKNAS!
#SAKNAS!
2395 (0.036)
#SAKNAS!

2395 (0.036)
1573 (0.071)
#SAKNAS!
2395 (0.036)
#SAKNAS!
2395 (0.036)
773 (0.18)
#SAKNAS!
#SAKNAS!
2395 (0.036)
2395 (0.036)
#SAKNAS!
#SAKNAS!
2395 (0.036)
2395 (0.036)
2395 (0.036)
#SAKNAS!
2395 (0.036)
#SAKNAS!
#SAKNAS!
#SAKNAS!
2395 (0.036)
#SAKNAS!
#SAKNAS!
2395 (0.036)
2395 (0.036)
1573 (0.071)
2395 (0.036)
1573 (0.071)
#SAKNAS!
2395 (0.036)
2395 (0.036)
#SAKNAS!
2395 (0.036)
1573 (0.071)
2395 (0.036)
2395 (0.036)
2395 (0.036)
#SAKNAS!
#SAKNAS!
#SAKNAS!
#SAKNAS!
#SAKNAS!

#SAKNAS!
1164 (0.11)
#SAKNAS!
773 (0.18)
2395 (0.036)
2395 (0.036)
#SAKNAS!
#SAKNAS!
2395 (0.036)
936 (0.14)
#SAKNAS!
2395 (0.036)
#SAKNAS!
#SAKNAS!
#SAKNAS!
1573 (0.071)
#SAKNAS!
#SAKNAS!
#SAKNAS!
#SAKNAS!
2395 (0.036)
#SAKNAS!
#SAKNAS!
1573 (0.071)
#SAKNAS!
2395 (0.036)
#SAKNAS!
936 (0.14)
1573 (0.071)
2395 (0.036)
#SAKNAS!
#SAKNAS!
#SAKNAS!
2395 (0.036)
#SAKNAS!
2395 (0.036)
#SAKNAS!
#SAKNAS!
#SAKNAS!
#SAKNAS!
2395 (0.036)
2395 (0.036)
#SAKNAS!

2395 (0.036)
2395 (0.036)
#SAKNAS!
#SAKNAS!
#SAKNAS!
#SAKNAS!
#SAKNAS!
#SAKNAS!
#SAKNAS!
2395 (0.036)
2395 (0.036)
2395 (0.036)
#SAKNAS!
#SAKNAS!
2395 (0.036)
#SAKNAS!
#SAKNAS!
#SAKNAS!
#SAKNAS!
2395 (0.036)
#SAKNAS!
2395 (0.036)
2395 (0.036)
2395 (0.036)
1573 (0.071)
2395 (0.036)
1164 (0.11)
2395 (0.036)
#SAKNAS!
#SAKNAS!
1573 (0.071)
2395 (0.036)
#SAKNAS!
#SAKNAS!
#SAKNAS!
#SAKNAS!
1573 (0.071)
936 (0.14)
2395 (0.036)
2395 (0.036)
#SAKNAS!
2395 (0.036)
#SAKNAS!

1573 (0.071)
#SAKNAS!
1573 (0.071)
#SAKNAS!
#SAKNAS!
2395 (0.036)
1573 (0.071)
#SAKNAS!
#SAKNAS!
2395 (0.036)
2395 (0.036)
#SAKNAS!
2395 (0.036)
1573 (0.071)
#SAKNAS!
#SAKNAS!
#SAKNAS!
#SAKNAS!
#SAKNAS!
#SAKNAS!
#SAKNAS!
1573 (0.071)
2395 (0.036)
2395 (0.036)
#SAKNAS!
#SAKNAS!
2395 (0.036)
#SAKNAS!
2395 (0.036)
1164 (0.11)
2395 (0.036)
2395 (0.036)
2395 (0.036)
1573 (0.071)
2395 (0.036)
1573 (0.071)
#SAKNAS!
#SAKNAS!
#SAKNAS!
1573 (0.071)
2395 (0.036)
#SAKNAS!
#SAKNAS!

1164 (0.11)
1573 (0.071)
648 (0.21)
773 (0.18)
936 (0.14)
#SAKNAS!
2395 (0.036)
2395 (0.036)
#SAKNAS!
2395 (0.036)
2395 (0.036)
1164 (0.11)
#SAKNAS!
#SAKNAS!
1573 (0.071)
2395 (0.036)
#SAKNAS!
#SAKNAS!
2395 (0.036)
#SAKNAS!
#SAKNAS!
#SAKNAS!
#SAKNAS!
#SAKNAS!
#SAKNAS!
2395 (0.036)
#SAKNAS!
1573 (0.071)
#SAKNAS!
#SAKNAS!
#SAKNAS!
1573 (0.071)
2395 (0.036)
1573 (0.071)
2395 (0.036)
1164 (0.11)
1573 (0.071)
1573 (0.071)
#SAKNAS!
#SAKNAS!
1573 (0.071)
#SAKNAS!
#SAKNAS!

1573 (0.071)
1573 (0.071)
2395 (0.036)
#SAKNAS!
#SAKNAS!
1164 (0.11)
#SAKNAS!
2395 (0.036)
2395 (0.036)
1573 (0.071)
2395 (0.036)
#SAKNAS!
#SAKNAS!
2395 (0.036)
2395 (0.036)
#SAKNAS!
#SAKNAS!
2395 (0.036)
936 (0.14)
2395 (0.036)
#SAKNAS!
2395 (0.036)
2395 (0.036)
#SAKNAS!
#SAKNAS!
2395 (0.036)
#SAKNAS!
#SAKNAS!
2395 (0.036)
2395 (0.036)
1573 (0.071)
1573 (0.071)
1573 (0.071)
2395 (0.036)
506 (0.28)
773 (0.18)
#SAKNAS!
506 (0.28)
#SAKNAS!
#SAKNAS!
#SAKNAS!
#SAKNAS!
2395 (0.036)

#SAKNAS!
#SAKNAS!
#SAKNAS!
2395 (0.036)
1164 (0.11)
1573 (0.071)
2395 (0.036)
2395 (0.036)
1573 (0.071)
#SAKNAS!
#SAKNAS!
773 (0.18)
1573 (0.071)
648 (0.21)
#SAKNAS!
#SAKNAS!
#SAKNAS!
#SAKNAS!
#SAKNAS!
1573 (0.071)
#SAKNAS!
#SAKNAS!
#SAKNAS!
#SAKNAS!
#SAKNAS!
2395 (0.036)
2395 (0.036)
2395 (0.036)
#SAKNAS!
2395 (0.036)
2395 (0.036)
#SAKNAS!
#SAKNAS!
2395 (0.036)
2395 (0.036)
2395 (0.036)
#SAKNAS!
#SAKNAS!
2395 (0.036)
#SAKNAS!
1573 (0.071)
1573 (0.071)
#SAKNAS!

2395 (0.036)
2395 (0.036)
#SAKNAS!
#SAKNAS!
2395 (0.036)
2395 (0.036)
1164 (0.11)
#SAKNAS!
#SAKNAS!
#SAKNAS!
#SAKNAS!
1573 (0.071)
#SAKNAS!
1573 (0.071)
#SAKNAS!
#SAKNAS!
2395 (0.036)
#SAKNAS!
#SAKNAS!
#SAKNAS!
2395 (0.036)
1573 (0.071)
2395 (0.036)
2395 (0.036)
#SAKNAS!
#SAKNAS!
#SAKNAS!
#SAKNAS!
#SAKNAS!
1573 (0.071)
1573 (0.071)
#SAKNAS!
2395 (0.036)
2395 (0.036)
2395 (0.036)
2395 (0.036)
#SAKNAS!
#SAKNAS!
2395 (0.036)
2395 (0.036)
#SAKNAS!
#SAKNAS!
2395 (0.036)

#SAKNAS!
#SAKNAS!
#SAKNAS!
#SAKNAS!
#SAKNAS!
#SAKNAS!
#SAKNAS!
#SAKNAS!
#SAKNAS!
#SAKNAS!
#SAKNAS!
2395 (0.036)
#SAKNAS!
#SAKNAS!
#SAKNAS!
#SAKNAS!
#SAKNAS!
#SAKNAS!
773 (0.18)
2395 (0.036)
1164 (0.11)
2395 (0.036)
2395 (0.036)
#SAKNAS!
#SAKNAS!
#SAKNAS!
#SAKNAS!
#SAKNAS!
#SAKNAS!
#SAKNAS!
#SAKNAS!
#SAKNAS!
2395 (0.036)
#SAKNAS!
#SAKNAS!
#SAKNAS!
#SAKNAS!
#SAKNAS!
#SAKNAS!
#SAKNAS!
2395 (0.036)
#SAKNAS!
#SAKNAS!

#SAKNAS!
#SAKNAS!
#SAKNAS!
2395 (0.036)
#SAKNAS!
#SAKNAS!
2395 (0.036)
#SAKNAS!
#SAKNAS!
#SAKNAS!
#SAKNAS!
#SAKNAS!
#SAKNAS!
#SAKNAS!
#SAKNAS!
#SAKNAS!
#SAKNAS!
#SAKNAS!
#SAKNAS!
2395 (0.036)
#SAKNAS!
#SAKNAS!
#SAKNAS!
2395 (0.036)
#SAKNAS!
#SAKNAS!
#SAKNAS!
#SAKNAS!
#SAKNAS!
#SAKNAS!
#SAKNAS!
#SAKNAS!
#SAKNAS!
#SAKNAS!
#SAKNAS!
#SAKNAS!
#SAKNAS!
#SAKNAS!
#SAKNAS!
#SAKNAS!
#SAKNAS!
#SAKNAS!
#SAKNAS!

#SAKNAS!
#SAKNAS!
#SAKNAS!
#SAKNAS!
2395 (0.036)
2395 (0.036)
2395 (0.036)
#SAKNAS!
#SAKNAS!
#SAKNAS!
#SAKNAS!
2395 (0.036)
#SAKNAS!
#SAKNAS!
#SAKNAS!
#SAKNAS!
#SAKNAS!
#SAKNAS!
#SAKNAS!
#SAKNAS!
#SAKNAS!
2395 (0.036)
2395 (0.036)
#SAKNAS!
2395 (0.036)
#SAKNAS!
#SAKNAS!
#SAKNAS!
2395 (0.036)
#SAKNAS!
#SAKNAS!
#SAKNAS!
#SAKNAS!
#SAKNAS!
#SAKNAS!
2395 (0.036)
#SAKNAS!
2395 (0.036)
#SAKNAS!
#SAKNAS!
#SAKNAS!
#SAKNAS!
#SAKNAS!

#SAKNAS!
#SAKNAS!
#SAKNAS!
#SAKNAS!
#SAKNAS!
#SAKNAS!
#SAKNAS!
#SAKNAS!
2395 (0.036)
#SAKNAS!
#SAKNAS!
#SAKNAS!
#SAKNAS!
#SAKNAS!
#SAKNAS!
2395 (0.036)
2395 (0.036)
#SAKNAS!
#SAKNAS!
#SAKNAS!
#SAKNAS!
#SAKNAS!
2395 (0.036)
#SAKNAS!
#SAKNAS!
#SAKNAS!
#SAKNAS!
#SAKNAS!
#SAKNAS!
2395 (0.036)
#SAKNAS!
#SAKNAS!
#SAKNAS!
#SAKNAS!
2395 (0.036)
2395 (0.036)
#SAKNAS!
#SAKNAS!
2395 (0.036)
2395 (0.036)
#SAKNAS!
#SAKNAS!
2395 (0.036)

2395 (0.036)
2395 (0.036)
2395 (0.036)
2395 (0.036)
2395 (0.036)
2395 (0.036)
2395 (0.036)
2395 (0.036)
2395 (0.036)
2395 (0.036)
#SAKNAS!
2395 (0.036)
2395 (0.036)
#SAKNAS!
#SAKNAS!
#SAKNAS!
#SAKNAS!
#SAKNAS!
#SAKNAS!
#SAKNAS!
#SAKNAS!
#SAKNAS!
#SAKNAS!
#SAKNAS!
2395 (0.036)
#SAKNAS!
#SAKNAS!
#SAKNAS!
2395 (0.036)
#SAKNAS!
#SAKNAS!
#SAKNAS!
#SAKNAS!
#SAKNAS!
2395 (0.036)
#SAKNAS!
#SAKNAS!
2395 (0.036)
#SAKNAS!
#SAKNAS!
#SAKNAS!
#SAKNAS!
#SAKNAS!

#SAKNAS!
#SAKNAS!
#SAKNAS!
#SAKNAS!
2395 (0.036)
#SAKNAS!
2395 (0.036)
2395 (0.036)
#SAKNAS!
#SAKNAS!
2395 (0.036)
#SAKNAS!
2395 (0.036)
#SAKNAS!
#SAKNAS!
#SAKNAS!
#SAKNAS!
#SAKNAS!
2395 (0.036)
#SAKNAS!
#SAKNAS!
#SAKNAS!
#SAKNAS!
#SAKNAS!
#SAKNAS!
#SAKNAS!
#SAKNAS!
#SAKNAS!
#SAKNAS!
2395 (0.036)
#SAKNAS!
#SAKNAS!
#SAKNAS!
#SAKNAS!
#SAKNAS!
2395 (0.036)
#SAKNAS!
#SAKNAS!
#SAKNAS!
#SAKNAS!
#SAKNAS!
#SAKNAS!
#SAKNAS!

#SAKNAS!
#SAKNAS!
#SAKNAS!
#SAKNAS!
#SAKNAS!
#SAKNAS!
#SAKNAS!
2395 (0.036)
2395 (0.036)
#SAKNAS!
#SAKNAS!
648 (0.21)
#SAKNAS!
#SAKNAS!
#SAKNAS!
#SAKNAS!
2395 (0.036)
#SAKNAS!
#SAKNAS!
#SAKNAS!
#SAKNAS!
#SAKNAS!
#SAKNAS!
2395 (0.036)
#SAKNAS!
#SAKNAS!
#SAKNAS!
#SAKNAS!
#SAKNAS!
#SAKNAS!
#SAKNAS!
#SAKNAS!
#SAKNAS!
#SAKNAS!
#SAKNAS!
#SAKNAS!
2395 (0.036)
2395 (0.036)
#SAKNAS!
#SAKNAS!
#SAKNAS!
#SAKNAS!
#SAKNAS!

#SAKNAS!
#SAKNAS!
2395 (0.036)
936 (0.14)
1573 (0.071)
2395 (0.036)
#SAKNAS!
#SAKNAS!
#SAKNAS!
#SAKNAS!
#SAKNAS!
2395 (0.036)
2395 (0.036)
#SAKNAS!
#SAKNAS!
#SAKNAS!
#SAKNAS!
#SAKNAS!
#SAKNAS!
2395 (0.036)
#SAKNAS!
#SAKNAS!
2395 (0.036)
#SAKNAS!
#SAKNAS!
#SAKNAS!
2395 (0.036)
#SAKNAS!
#SAKNAS!
#SAKNAS!
2395 (0.036)
2395 (0.036)
#SAKNAS!
#SAKNAS!
2395 (0.036)
2395 (0.036)
#SAKNAS!
#SAKNAS!
#SAKNAS!
2395 (0.036)
#SAKNAS!
2395 (0.036)
#SAKNAS!

#SAKNAS!
#SAKNAS!
2395 (0.036)
2395 (0.036)
#SAKNAS!
#SAKNAS!
#SAKNAS!
#SAKNAS!
#SAKNAS!
#SAKNAS!
#SAKNAS!
2395 (0.036)
#SAKNAS!
#SAKNAS!
2395 (0.036)
#SAKNAS!
#SAKNAS!
#SAKNAS!
2395 (0.036)
#SAKNAS!
#SAKNAS!
2395 (0.036)
2395 (0.036)
#SAKNAS!
#SAKNAS!
#SAKNAS!
#SAKNAS!
#SAKNAS!
2395 (0.036)
#SAKNAS!
#SAKNAS!
#SAKNAS!
2395 (0.036)
2395 (0.036)
#SAKNAS!
2395 (0.036)
2395 (0.036)
#SAKNAS!
#SAKNAS!
#SAKNAS!
#SAKNAS!
#SAKNAS!
2395 (0.036)

2395 (0.036)
#SAKNAS!
#SAKNAS!
#SAKNAS!
2395 (0.036)
2395 (0.036)
2395 (0.036)
#SAKNAS!
#SAKNAS!
#SAKNAS!
#SAKNAS!
#SAKNAS!
#SAKNAS!
2395 (0.036)
#SAKNAS!
#SAKNAS!
#SAKNAS!
2395 (0.036)
#SAKNAS!
#SAKNAS!
#SAKNAS!
#SAKNAS!
#SAKNAS!
#SAKNAS!
2395 (0.036)
#SAKNAS!
#SAKNAS!
#SAKNAS!
#SAKNAS!
#SAKNAS!
2395 (0.036)
#SAKNAS!
#SAKNAS!
#SAKNAS!
#SAKNAS!
#SAKNAS!
2395 (0.036)
#SAKNAS!
#SAKNAS!
1164 (0.11)
2395 (0.036)
#SAKNAS!
2395 (0.036)

#SAKNAS!
#SAKNAS!
#SAKNAS!
#SAKNAS!
#SAKNAS!
#SAKNAS!
#SAKNAS!
#SAKNAS!
1573 (0.071)
#SAKNAS!
#SAKNAS!
#SAKNAS!
#SAKNAS!
#SAKNAS!
2395 (0.036)
#SAKNAS!
2395 (0.036)
2395 (0.036)
2395 (0.036)
773 (0.18)
#SAKNAS!
2395 (0.036)
#SAKNAS!
#SAKNAS!
#SAKNAS!
#SAKNAS!
2395 (0.036)
#SAKNAS!
#SAKNAS!
#SAKNAS!
2395 (0.036)
2395 (0.036)
#SAKNAS!
2395 (0.036)
2395 (0.036)
#SAKNAS!
#SAKNAS!
#SAKNAS!
#SAKNAS!
#SAKNAS!
#SAKNAS!
#SAKNAS!
#SAKNAS!

2395 (0.036)
2395 (0.036)
#SAKNAS!
2395 (0.036)
#SAKNAS!
#SAKNAS!
#SAKNAS!
2395 (0.036)
2395 (0.036)
#SAKNAS!
2395 (0.036)
2395 (0.036)
#SAKNAS!
2395 (0.036)
2395 (0.036)
2395 (0.036)
#SAKNAS!
2395 (0.036)
#SAKNAS!
#SAKNAS!
#SAKNAS!
#SAKNAS!
#SAKNAS!
#SAKNAS!
#SAKNAS!
#SAKNAS!
2395 (0.036)
#SAKNAS!
#SAKNAS!
#SAKNAS!
2395 (0.036)
2395 (0.036)
2395 (0.036)
2395 (0.036)
2395 (0.036)
#SAKNAS!
#SAKNAS!
#SAKNAS!
#SAKNAS!
#SAKNAS!
#SAKNAS!
2395 (0.036)
#SAKNAS!

2395 (0.036)
2395 (0.036)
#SAKNAS!
#SAKNAS!
#SAKNAS!
#SAKNAS!
2395 (0.036)
#SAKNAS!
#SAKNAS!
2395 (0.036)
2395 (0.036)
#SAKNAS!
#SAKNAS!
2395 (0.036)
#SAKNAS!
#SAKNAS!
#SAKNAS!
#SAKNAS!
#SAKNAS!
2395 (0.036)
#SAKNAS!
#SAKNAS!
#SAKNAS!
#SAKNAS!
#SAKNAS!
#SAKNAS!
#SAKNAS!
2395 (0.036)
#SAKNAS!
#SAKNAS!
#SAKNAS!
#SAKNAS!
#SAKNAS!
#SAKNAS!
#SAKNAS!
2395 (0.036)
#SAKNAS!
#SAKNAS!
2395 (0.036)
#SAKNAS!
#SAKNAS!
#SAKNAS!
#SAKNAS!

1573 (0.071)
#SAKNAS!
#SAKNAS!
#SAKNAS!
#SAKNAS!
2395 (0.036)
#SAKNAS!
#SAKNAS!
2395 (0.036)
#SAKNAS!
#SAKNAS!
2395 (0.036)
#SAKNAS!
#SAKNAS!
2395 (0.036)
#SAKNAS!
#SAKNAS!
2395 (0.036)
1573 (0.071)
#SAKNAS!
#SAKNAS!
#SAKNAS!
#SAKNAS!
#SAKNAS!
2395 (0.036)
2395 (0.036)
#SAKNAS!
#SAKNAS!
2395 (0.036)
#SAKNAS!
#SAKNAS!
#SAKNAS!
#SAKNAS!
#SAKNAS!
#SAKNAS!
#SAKNAS!
2395 (0.036)
#SAKNAS!
#SAKNAS!
#SAKNAS!
2395 (0.036)
1573 (0.071)
2395 (0.036)

2395 (0.036)
#SAKNAS!
#SAKNAS!
#SAKNAS!
#SAKNAS!
#SAKNAS!
2395 (0.036)
#SAKNAS!
#SAKNAS!
#SAKNAS!
#SAKNAS!
#SAKNAS!
#SAKNAS!
#SAKNAS!
#SAKNAS!
#SAKNAS!
#SAKNAS!
#SAKNAS!
#SAKNAS!
#SAKNAS!
#SAKNAS!
#SAKNAS!
2395 (0.036)
#SAKNAS!
#SAKNAS!
2395 (0.036)
2395 (0.036)
#SAKNAS!
2395 (0.036)
2395 (0.036)
#SAKNAS!
#SAKNAS!
#SAKNAS!
#SAKNAS!
#SAKNAS!
#SAKNAS!
#SAKNAS!
#SAKNAS!
#SAKNAS!
#SAKNAS!
#SAKNAS!
1573 (0.071)
#SAKNAS!

#SAKNAS!
2395 (0.036)
#SAKNAS!
#SAKNAS!
#SAKNAS!
#SAKNAS!
#SAKNAS!
2395 (0.036)
2395 (0.036)
#SAKNAS!
#SAKNAS!
#SAKNAS!
#SAKNAS!
1573 (0.071)
#SAKNAS!
2395 (0.036)
2395 (0.036)
#SAKNAS!
#SAKNAS!
#SAKNAS!
2395 (0.036)
#SAKNAS!
#SAKNAS!
2395 (0.036)
2395 (0.036)
2395 (0.036)
2395 (0.036)
#SAKNAS!
2395 (0.036)
#SAKNAS!
#SAKNAS!
#SAKNAS!
2395 (0.036)
#SAKNAS!
#SAKNAS!
#SAKNAS!
#SAKNAS!
#SAKNAS!
#SAKNAS!
#SAKNAS!
#SAKNAS!
#SAKNAS!
2395 (0.036)

2395 (0.036)
#SAKNAS!
#SAKNAS!
#SAKNAS!
2395 (0.036)
2395 (0.036)
2395 (0.036)
2395 (0.036)
#SAKNAS!
#SAKNAS!
2395 (0.036)
#SAKNAS!
#SAKNAS!
2395 (0.036)
#SAKNAS!
#SAKNAS!
#SAKNAS!
2395 (0.036)
#SAKNAS!
#SAKNAS!
#SAKNAS!
2395 (0.036)
2395 (0.036)
1573 (0.071)
2395 (0.036)
2395 (0.036)
#SAKNAS!
#SAKNAS!
#SAKNAS!
1164 (0.11)
#SAKNAS!
#SAKNAS!
2395 (0.036)
1573 (0.071)
2395 (0.036)
#SAKNAS!
#SAKNAS!
#SAKNAS!
#SAKNAS!
2395 (0.036)
#SAKNAS!
#SAKNAS!
#SAKNAS!

#SAKNAS!
#SAKNAS!
#SAKNAS!
#SAKNAS!
#SAKNAS!
#SAKNAS!
#SAKNAS!
#SAKNAS!
#SAKNAS!
#SAKNAS!
#SAKNAS!
#SAKNAS!
1164 (0.11)
#SAKNAS!
#SAKNAS!
#SAKNAS!
#SAKNAS!
#SAKNAS!
#SAKNAS!
2395 (0.036)
2395 (0.036)
#SAKNAS!
#SAKNAS!
#SAKNAS!
2395 (0.036)
#SAKNAS!
2395 (0.036)
2395 (0.036)
#SAKNAS!
#SAKNAS!
#SAKNAS!
#SAKNAS!
#SAKNAS!
2395 (0.036)
#SAKNAS!
#SAKNAS!
#SAKNAS!
#SAKNAS!
#SAKNAS!
#SAKNAS!
#SAKNAS!
#SAKNAS!
#SAKNAS!

#SAKNAS!
#SAKNAS!
2395 (0.036)
#SAKNAS!
#SAKNAS!
2395 (0.036)
1573 (0.071)
#SAKNAS!
#SAKNAS!
#SAKNAS!
#SAKNAS!
2395 (0.036)
2395 (0.036)
#SAKNAS!
2395 (0.036)
#SAKNAS!
#SAKNAS!
2395 (0.036)
2395 (0.036)
2395 (0.036)
#SAKNAS!
#SAKNAS!
2395 (0.036)
2395 (0.036)
2395 (0.036)
2395 (0.036)
#SAKNAS!
#SAKNAS!
#SAKNAS!
#SAKNAS!
#SAKNAS!
1573 (0.071)
2395 (0.036)
#SAKNAS!
#SAKNAS!
#SAKNAS!
2395 (0.036)
#SAKNAS!
#SAKNAS!
#SAKNAS!
#SAKNAS!
1164 (0.11)
2395 (0.036)

#SAKNAS!
#SAKNAS!
2395 (0.036)
#SAKNAS!
#SAKNAS!
#SAKNAS!
#SAKNAS!
#SAKNAS!
2395 (0.036)
#SAKNAS!
#SAKNAS!
#SAKNAS!
#SAKNAS!
#SAKNAS!
#SAKNAS!
2395 (0.036)
#SAKNAS!
#SAKNAS!
1573 (0.071)
2395 (0.036)
1164 (0.11)
#SAKNAS!
1164 (0.11)
#SAKNAS!
#SAKNAS!
#SAKNAS!
#SAKNAS!
2395 (0.036)
#SAKNAS!
#SAKNAS!
#SAKNAS!
#SAKNAS!
2395 (0.036)
#SAKNAS!
#SAKNAS!
#SAKNAS!
#SAKNAS!
#SAKNAS!
2395 (0.036)
2395 (0.036)
#SAKNAS!
#SAKNAS!
#SAKNAS!

1164 (0.11)
#SAKNAS!
#SAKNAS!
#SAKNAS!
#SAKNAS!
#SAKNAS!
#SAKNAS!
#SAKNAS!
#SAKNAS!
#SAKNAS!
#SAKNAS!
2395 (0.036)
#SAKNAS!
#SAKNAS!
1164 (0.11)
2395 (0.036)
#SAKNAS!
#SAKNAS!
#SAKNAS!
2395 (0.036)
#SAKNAS!
2395 (0.036)
2395 (0.036)
#SAKNAS!
2395 (0.036)
2395 (0.036)
2395 (0.036)
2395 (0.036)
#SAKNAS!
#SAKNAS!
#SAKNAS!
#SAKNAS!
#SAKNAS!
#SAKNAS!
#SAKNAS!
#SAKNAS!
2395 (0.036)
#SAKNAS!
#SAKNAS!
#SAKNAS!
#SAKNAS!
1573 (0.071)
#SAKNAS!

#SAKNAS!
#SAKNAS!
2395 (0.036)
#SAKNAS!
#SAKNAS!
#SAKNAS!
#SAKNAS!
2395 (0.036)
#SAKNAS!
#SAKNAS!
#SAKNAS!
2395 (0.036)
#SAKNAS!
#SAKNAS!
#SAKNAS!
#SAKNAS!
#SAKNAS!
#SAKNAS!
#SAKNAS!
#SAKNAS!
2395 (0.036)
#SAKNAS!
#SAKNAS!
2395 (0.036)
#SAKNAS!
#SAKNAS!
#SAKNAS!
#SAKNAS!
#SAKNAS!
#SAKNAS!
#SAKNAS!
#SAKNAS!
#SAKNAS!
#SAKNAS!
2395 (0.036)
#SAKNAS!
#SAKNAS!
#SAKNAS!
#SAKNAS!
#SAKNAS!
#SAKNAS!
#SAKNAS!
#SAKNAS!

#SAKNAS!
561 (0.25)
#SAKNAS!
#SAKNAS!
#SAKNAS!
#SAKNAS!
#SAKNAS!
2395 (0.036)
#SAKNAS!
#SAKNAS!
#SAKNAS!
#SAKNAS!
#SAKNAS!
2395 (0.036)
2395 (0.036)
2395 (0.036)
2395 (0.036)
#SAKNAS!
#SAKNAS!
2395 (0.036)
#SAKNAS!
#SAKNAS!
#SAKNAS!
#SAKNAS!
#SAKNAS!
#SAKNAS!
#SAKNAS!
1573 (0.071)
#SAKNAS!
#SAKNAS!
#SAKNAS!
#SAKNAS!
#SAKNAS!
#SAKNAS!
#SAKNAS!
#SAKNAS!
#SAKNAS!
#SAKNAS!
#SAKNAS!
2395 (0.036)
#SAKNAS!
#SAKNAS!
#SAKNAS!

#SAKNAS!
#SAKNAS!
#SAKNAS!
#SAKNAS!
1573 (0.071)
#SAKNAS!
2395 (0.036)
#SAKNAS!
#SAKNAS!
#SAKNAS!
#SAKNAS!
#SAKNAS!
#SAKNAS!
#SAKNAS!
#SAKNAS!
#SAKNAS!
#SAKNAS!
1573 (0.071)
2395 (0.036)
2395 (0.036)
#SAKNAS!
#SAKNAS!
#SAKNAS!
#SAKNAS!
#SAKNAS!
2395 (0.036)
#SAKNAS!
#SAKNAS!
#SAKNAS!
#SAKNAS!
2395 (0.036)
#SAKNAS!
#SAKNAS!
#SAKNAS!
2395 (0.036)
#SAKNAS!
#SAKNAS!
#SAKNAS!
#SAKNAS!
2395 (0.036)
2395 (0.036)
#SAKNAS!
#SAKNAS!

2395 (0.036)
#SAKNAS!
#SAKNAS!
#SAKNAS!
#SAKNAS!
#SAKNAS!
#SAKNAS!
#SAKNAS!
#SAKNAS!
#SAKNAS!
648 (0.21)
#SAKNAS!
#SAKNAS!
#SAKNAS!
#SAKNAS!
#SAKNAS!
#SAKNAS!
#SAKNAS!
#SAKNAS!
#SAKNAS!
#SAKNAS!
#SAKNAS!
936 (0.14)
1573 (0.071)
#SAKNAS!
#SAKNAS!
#SAKNAS!
#SAKNAS!
2395 (0.036)
#SAKNAS!
#SAKNAS!
#SAKNAS!
#SAKNAS!
2395 (0.036)
2395 (0.036)
#SAKNAS!
#SAKNAS!
#SAKNAS!
#SAKNAS!
2395 (0.036)
#SAKNAS!
#SAKNAS!
#SAKNAS!

2395 (0.036)
#SAKNAS!
#SAKNAS!
#SAKNAS!
#SAKNAS!
#SAKNAS!
#SAKNAS!
2395 (0.036)
#SAKNAS!
#SAKNAS!
#SAKNAS!
2395 (0.036)
2395 (0.036)
#SAKNAS!
#SAKNAS!
2395 (0.036)
2395 (0.036)
2395 (0.036)
#SAKNAS!
#SAKNAS!
#SAKNAS!
#SAKNAS!
2395 (0.036)
#SAKNAS!
2395 (0.036)
#SAKNAS!
2395 (0.036)
2395 (0.036)
#SAKNAS!
#SAKNAS!
#SAKNAS!
#SAKNAS!
#SAKNAS!
#SAKNAS!
2395 (0.036)
#SAKNAS!
#SAKNAS!
#SAKNAS!
2395 (0.036)
#SAKNAS!
2395 (0.036)
#SAKNAS!
#SAKNAS!

#SAKNAS!
#SAKNAS!
#SAKNAS!
#SAKNAS!
#SAKNAS!
#SAKNAS!
#SAKNAS!
#SAKNAS!
#SAKNAS!
#SAKNAS!
#SAKNAS!
#SAKNAS!
#SAKNAS!
#SAKNAS!
#SAKNAS!
#SAKNAS!
#SAKNAS!
#SAKNAS!
#SAKNAS!
2395 (0.036)
#SAKNAS!
#SAKNAS!
2395 (0.036)
#SAKNAS!
#SAKNAS!
2395 (0.036)
#SAKNAS!
2395 (0.036)
#SAKNAS!
#SAKNAS!
2395 (0.036)
2395 (0.036)
2395 (0.036)
2395 (0.036)
#SAKNAS!
#SAKNAS!
#SAKNAS!
#SAKNAS!
2395 (0.036)
2395 (0.036)
#SAKNAS!
#SAKNAS!
#SAKNAS!

#SAKNAS!
#SAKNAS!
2395 (0.036)
2395 (0.036)
#SAKNAS!
#SAKNAS!
#SAKNAS!
#SAKNAS!
#SAKNAS!
#SAKNAS!
2395 (0.036)
#SAKNAS!
#SAKNAS!
2395 (0.036)
#SAKNAS!
#SAKNAS!
#SAKNAS!
#SAKNAS!
1573 (0.071)
#SAKNAS!
#SAKNAS!
#SAKNAS!
#SAKNAS!
#SAKNAS!
#SAKNAS!
#SAKNAS!
#SAKNAS!
#SAKNAS!
#SAKNAS!
2395 (0.036)
2395 (0.036)
#SAKNAS!
#SAKNAS!
2395 (0.036)
#SAKNAS!
2395 (0.036)
#SAKNAS!
#SAKNAS!
#SAKNAS!
#SAKNAS!
#SAKNAS!
2395 (0.036)
2395 (0.036)

#SAKNAS!
#SAKNAS!
2395 (0.036)
#SAKNAS!
#SAKNAS!
2395 (0.036)
#SAKNAS!
2395 (0.036)
#SAKNAS!
#SAKNAS!
#SAKNAS!
#SAKNAS!
#SAKNAS!
#SAKNAS!
#SAKNAS!
#SAKNAS!
#SAKNAS!
#SAKNAS!
#SAKNAS!
#SAKNAS!
#SAKNAS!
2395 (0.036)
#SAKNAS!
#SAKNAS!
#SAKNAS!
#SAKNAS!
#SAKNAS!
561 (0.25)
#SAKNAS!
#SAKNAS!
2395 (0.036)
2395 (0.036)
#SAKNAS!
2395 (0.036)
2395 (0.036)
#SAKNAS!
#SAKNAS!
#SAKNAS!
#SAKNAS!
1164 (0.11)
#SAKNAS!
#SAKNAS!
#SAKNAS!

#SAKNAS!
#SAKNAS!
#SAKNAS!
#SAKNAS!
#SAKNAS!
1573 (0.071)
#SAKNAS!
#SAKNAS!
#SAKNAS!
#SAKNAS!
#SAKNAS!
2395 (0.036)
#SAKNAS!
2395 (0.036)
2395 (0.036)
#SAKNAS!
#SAKNAS!
#SAKNAS!
#SAKNAS!
#SAKNAS!
#SAKNAS!
#SAKNAS!
#SAKNAS!
2395 (0.036)
2395 (0.036)
#SAKNAS!
#SAKNAS!
#SAKNAS!
#SAKNAS!
#SAKNAS!
2395 (0.036)
#SAKNAS!
#SAKNAS!
#SAKNAS!
2395 (0.036)
#SAKNAS!
#SAKNAS!
#SAKNAS!
#SAKNAS!
#SAKNAS!
#SAKNAS!
#SAKNAS!
#SAKNAS!

#SAKNAS!
#SAKNAS!
#SAKNAS!
#SAKNAS!
#SAKNAS!
#SAKNAS!
2395 (0.036)
2395 (0.036)
2395 (0.036)
2395 (0.036)
2395 (0.036)
#SAKNAS!
2395 (0.036)
#SAKNAS!
#SAKNAS!
#SAKNAS!
#SAKNAS!
2395 (0.036)
#SAKNAS!
#SAKNAS!
#SAKNAS!
#SAKNAS!
2395 (0.036)
#SAKNAS!
#SAKNAS!
#SAKNAS!
#SAKNAS!
#SAKNAS!
#SAKNAS!
936 (0.14)
#SAKNAS!
2395 (0.036)
#SAKNAS!
#SAKNAS!
2395 (0.036)
2395 (0.036)
2395 (0.036)
#SAKNAS!
#SAKNAS!
#SAKNAS!
#SAKNAS!
#SAKNAS!
#SAKNAS!

#SAKNAS!
#SAKNAS!
#SAKNAS!
2395 (0.036)
#SAKNAS!
#SAKNAS!
2395 (0.036)
2395 (0.036)
2395 (0.036)
2395 (0.036)
#SAKNAS!
2395 (0.036)
#SAKNAS!
#SAKNAS!
#SAKNAS!
#SAKNAS!
#SAKNAS!
#SAKNAS!
#SAKNAS!
#SAKNAS!
2395 (0.036)
#SAKNAS!
#SAKNAS!
#SAKNAS!
#SAKNAS!
2395 (0.036)
2395 (0.036)
2395 (0.036)
#SAKNAS!
2395 (0.036)
#SAKNAS!
#SAKNAS!
#SAKNAS!
#SAKNAS!
#SAKNAS!
#SAKNAS!
#SAKNAS!
#SAKNAS!
2395 (0.036)
2395 (0.036)
2395 (0.036)
#SAKNAS!
#SAKNAS!

#SAKNAS!
1164 (0.11)
2395 (0.036)
#SAKNAS!
#SAKNAS!
#SAKNAS!
#SAKNAS!
2395 (0.036)
#SAKNAS!
2395 (0.036)
#SAKNAS!
#SAKNAS!
#SAKNAS!
#SAKNAS!
#SAKNAS!
#SAKNAS!
#SAKNAS!
#SAKNAS!
#SAKNAS!
#SAKNAS!
#SAKNAS!
#SAKNAS!
#SAKNAS!
#SAKNAS!
#SAKNAS!
#SAKNAS!
#SAKNAS!
#SAKNAS!
#SAKNAS!
#SAKNAS!
#SAKNAS!
#SAKNAS!
#SAKNAS!
#SAKNAS!
2395 (0.036)
2395 (0.036)
2395 (0.036)
#SAKNAS!
#SAKNAS!
2395 (0.036)
#SAKNAS!
936 (0.14)
2395 (0.036)

#SAKNAS!
#SAKNAS!
#SAKNAS!
#SAKNAS!
#SAKNAS!
#SAKNAS!
#SAKNAS!
#SAKNAS!
#SAKNAS!
2395 (0.036)
2395 (0.036)
#SAKNAS!
#SAKNAS!
#SAKNAS!
#SAKNAS!
2395 (0.036)
#SAKNAS!
2395 (0.036)
#SAKNAS!
#SAKNAS!
#SAKNAS!
#SAKNAS!
#SAKNAS!
2395 (0.036)
#SAKNAS!
#SAKNAS!
#SAKNAS!
#SAKNAS!
#SAKNAS!
#SAKNAS!
#SAKNAS!
#SAKNAS!
#SAKNAS!
#SAKNAS!
#SAKNAS!
#SAKNAS!
#SAKNAS!
#SAKNAS!
#SAKNAS!
#SAKNAS!
#SAKNAS!
#SAKNAS!
2395 (0.036)

2395 (0.036)
1164 (0.11)
2395 (0.036)
2395 (0.036)
#SAKNAS!
#SAKNAS!
#SAKNAS!
#SAKNAS!
#SAKNAS!
#SAKNAS!
#SAKNAS!
#SAKNAS!
#SAKNAS!
#SAKNAS!
#SAKNAS!
#SAKNAS!
#SAKNAS!
#SAKNAS!
2395 (0.036)
2395 (0.036)
#SAKNAS!
#SAKNAS!
#SAKNAS!
#SAKNAS!
#SAKNAS!
#SAKNAS!
#SAKNAS!
#SAKNAS!
#SAKNAS!
2395 (0.036)
2395 (0.036)
#SAKNAS!
#SAKNAS!
#SAKNAS!
#SAKNAS!
#SAKNAS!
2395 (0.036)
#SAKNAS!
#SAKNAS!
#SAKNAS!
#SAKNAS!
#SAKNAS!
#SAKNAS!

#SAKNAS!
2395 (0.036)
2395 (0.036)
#SAKNAS!
#SAKNAS!
#SAKNAS!
#SAKNAS!
#SAKNAS!
#SAKNAS!
#SAKNAS!
#SAKNAS!
#SAKNAS!
#SAKNAS!
#SAKNAS!
#SAKNAS!
#SAKNAS!
#SAKNAS!
#SAKNAS!
#SAKNAS!
#SAKNAS!
#SAKNAS!
#SAKNAS!
#SAKNAS!
2395 (0.036)
#SAKNAS!
#SAKNAS!
#SAKNAS!
2395 (0.036)
#SAKNAS!
1164 (0.11)
2395 (0.036)
#SAKNAS!
#SAKNAS!
#SAKNAS!
#SAKNAS!
#SAKNAS!
#SAKNAS!
#SAKNAS!
2395 (0.036)
2395 (0.036)
#SAKNAS!
#SAKNAS!
2395 (0.036)

2395 (0.036)
2395 (0.036)
#SAKNAS!
#SAKNAS!
#SAKNAS!
#SAKNAS!
2395 (0.036)
2395 (0.036)
#SAKNAS!
2395 (0.036)
#SAKNAS!
936 (0.14)
1573 (0.071)
#SAKNAS!
2395 (0.036)
#SAKNAS!
2395 (0.036)
#SAKNAS!
2395 (0.036)
#SAKNAS!
2395 (0.036)
#SAKNAS!
2395 (0.036)
#SAKNAS!
#SAKNAS!
#SAKNAS!
#SAKNAS!
#SAKNAS!
2395 (0.036)
#SAKNAS!
#SAKNAS!
#SAKNAS!
#SAKNAS!
#SAKNAS!
#SAKNAS!
2395 (0.036)
2395 (0.036)
#SAKNAS!
2395 (0.036)
#SAKNAS!
#SAKNAS!
#SAKNAS!
#SAKNAS!

#SAKNAS!
#SAKNAS!
#SAKNAS!
2395 (0.036)
#SAKNAS!
#SAKNAS!
2395 (0.036)
#SAKNAS!
2395 (0.036)
#SAKNAS!
2395 (0.036)
#SAKNAS!
#SAKNAS!
#SAKNAS!
#SAKNAS!
#SAKNAS!
#SAKNAS!
#SAKNAS!
#SAKNAS!
#SAKNAS!
#SAKNAS!
#SAKNAS!
#SAKNAS!
2395 (0.036)
#SAKNAS!
#SAKNAS!
#SAKNAS!
#SAKNAS!
#SAKNAS!
#SAKNAS!
#SAKNAS!
#SAKNAS!
#SAKNAS!
#SAKNAS!
#SAKNAS!
#SAKNAS!
#SAKNAS!
2395 (0.036)
#SAKNAS!
#SAKNAS!
#SAKNAS!
#SAKNAS!
#SAKNAS!

#SAKNAS!
#SAKNAS!
#SAKNAS!
#SAKNAS!
#SAKNAS!
2395 (0.036)
#SAKNAS!
#SAKNAS!
2395 (0.036)
2395 (0.036)
#SAKNAS!
2395 (0.036)
#SAKNAS!
#SAKNAS!
2395 (0.036)
#SAKNAS!
#SAKNAS!
2395 (0.036)
#SAKNAS!
2395 (0.036)
#SAKNAS!
#SAKNAS!
2395 (0.036)
#SAKNAS!
#SAKNAS!
#SAKNAS!
2395 (0.036)
1573 (0.071)
#SAKNAS!
#SAKNAS!
#SAKNAS!
#SAKNAS!
2395 (0.036)
#SAKNAS!
#SAKNAS!
936 (0.14)
#SAKNAS!
#SAKNAS!
2395 (0.036)
#SAKNAS!
#SAKNAS!
#SAKNAS!
#SAKNAS!

#SAKNAS!
506 (0.28)
#SAKNAS!
#SAKNAS!
2395 (0.036)
#SAKNAS!
2395 (0.036)
#SAKNAS!
#SAKNAS!
#SAKNAS!
2395 (0.036)
#SAKNAS!
2395 (0.036)
#SAKNAS!
#SAKNAS!
#SAKNAS!
2395 (0.036)
#SAKNAS!
2395 (0.036)
2395 (0.036)
#SAKNAS!
#SAKNAS!
2395 (0.036)
#SAKNAS!
2395 (0.036)
1573 (0.071)
#SAKNAS!
#SAKNAS!
2395 (0.036)
#SAKNAS!
#SAKNAS!
2395 (0.036)
#SAKNAS!
#SAKNAS!
#SAKNAS!
#SAKNAS!
#SAKNAS!
#SAKNAS!
#SAKNAS!
#SAKNAS!
#SAKNAS!
#SAKNAS!
2395 (0.036)

#SAKNAS!
#SAKNAS!
#SAKNAS!
#SAKNAS!
#SAKNAS!
#SAKNAS!
#SAKNAS!
#SAKNAS!
#SAKNAS!
2395 (0.036)
#SAKNAS!
#SAKNAS!
#SAKNAS!
#SAKNAS!
#SAKNAS!
#SAKNAS!
#SAKNAS!
2395 (0.036)
2395 (0.036)
#SAKNAS!
#SAKNAS!
#SAKNAS!
#SAKNAS!
#SAKNAS!
2395 (0.036)
#SAKNAS!
2395 (0.036)
#SAKNAS!
#SAKNAS!
#SAKNAS!
2395 (0.036)
#SAKNAS!
2395 (0.036)
#SAKNAS!
#SAKNAS!
#SAKNAS!
#SAKNAS!
#SAKNAS!
#SAKNAS!
2395 (0.036)
#SAKNAS!
#SAKNAS!
#SAKNAS!

2395 (0.036)
319 (0.46)
#SAKNAS!
#SAKNAS!
#SAKNAS!
#SAKNAS!
2395 (0.036)
2395 (0.036)
2395 (0.036)
2395 (0.036)
#SAKNAS!
#SAKNAS!
#SAKNAS!
#SAKNAS!
#SAKNAS!
#SAKNAS!
#SAKNAS!
2395 (0.036)
2395 (0.036)
#SAKNAS!
#SAKNAS!
#SAKNAS!
#SAKNAS!
#SAKNAS!
#SAKNAS!
#SAKNAS!
#SAKNAS!
#SAKNAS!
1164 (0.11)
#SAKNAS!
#SAKNAS!
#SAKNAS!
2395 (0.036)
#SAKNAS!
#SAKNAS!
#SAKNAS!
#SAKNAS!
#SAKNAS!
#SAKNAS!
#SAKNAS!
#SAKNAS!
#SAKNAS!
1573 (0.071)

#SAKNAS!
#SAKNAS!
#SAKNAS!
#SAKNAS!
#SAKNAS!
#SAKNAS!
#SAKNAS!
#SAKNAS!
2395 (0.036)
2395 (0.036)
#SAKNAS!
#SAKNAS!
2395 (0.036)
#SAKNAS!
#SAKNAS!
#SAKNAS!
#SAKNAS!
#SAKNAS!
#SAKNAS!
2395 (0.036)
#SAKNAS!
#SAKNAS!
#SAKNAS!
#SAKNAS!
#SAKNAS!
#SAKNAS!
2395 (0.036)
#SAKNAS!
#SAKNAS!
#SAKNAS!
#SAKNAS!
#SAKNAS!
#SAKNAS!
#SAKNAS!
#SAKNAS!
#SAKNAS!
#SAKNAS!
2395 (0.036)
2395 (0.036)
2395 (0.036)
#SAKNAS!
#SAKNAS!
2395 (0.036)

#SAKNAS!
#SAKNAS!
#SAKNAS!
2395 (0.036)
#SAKNAS!
#SAKNAS!
2395 (0.036)
#SAKNAS!
#SAKNAS!
#SAKNAS!
#SAKNAS!
#SAKNAS!
2395 (0.036)
#SAKNAS!
#SAKNAS!
#SAKNAS!
2395 (0.036)
2395 (0.036)
#SAKNAS!
#SAKNAS!
#SAKNAS!
2395 (0.036)
#SAKNAS!
#SAKNAS!
#SAKNAS!
#SAKNAS!
#SAKNAS!
2395 (0.036)
#SAKNAS!
#SAKNAS!
#SAKNAS!
#SAKNAS!
2395 (0.036)
#SAKNAS!
#SAKNAS!
#SAKNAS!
#SAKNAS!
2395 (0.036)
#SAKNAS!
#SAKNAS!
2395 (0.036)
#SAKNAS!
#SAKNAS!

#SAKNAS!
#SAKNAS!
#SAKNAS!
2395 (0.036)
#SAKNAS!
#SAKNAS!
#SAKNAS!
#SAKNAS!
1573 (0.071)
#SAKNAS!
#SAKNAS!
#SAKNAS!
#SAKNAS!
#SAKNAS!
1573 (0.071)
2395 (0.036)
#SAKNAS!
2395 (0.036)
2395 (0.036)
2395 (0.036)
#SAKNAS!
#SAKNAS!
2395 (0.036)
#SAKNAS!
#SAKNAS!
2395 (0.036)
#SAKNAS!
#SAKNAS!
#SAKNAS!
#SAKNAS!
2395 (0.036)
#SAKNAS!
2395 (0.036)
#SAKNAS!
#SAKNAS!
2395 (0.036)
#SAKNAS!
#SAKNAS!
#SAKNAS!
#SAKNAS!
#SAKNAS!
2395 (0.036)
2395 (0.036)

#SAKNAS!
#SAKNAS!
2395 (0.036)
2395 (0.036)
#SAKNAS!
#SAKNAS!
#SAKNAS!
#SAKNAS!
2395 (0.036)
#SAKNAS!
#SAKNAS!
#SAKNAS!
#SAKNAS!
#SAKNAS!
#SAKNAS!
2395 (0.036)
#SAKNAS!
2395 (0.036)
#SAKNAS!
#SAKNAS!
#SAKNAS!
#SAKNAS!
#SAKNAS!
2395 (0.036)
2395 (0.036)
#SAKNAS!
#SAKNAS!
#SAKNAS!
2395 (0.036)
#SAKNAS!
#SAKNAS!
2395 (0.036)
#SAKNAS!
#SAKNAS!
#SAKNAS!
#SAKNAS!
#SAKNAS!
#SAKNAS!
#SAKNAS!
#SAKNAS!
#SAKNAS!
#SAKNAS!
#SAKNAS!

#SAKNAS!
#SAKNAS!
#SAKNAS!
#SAKNAS!
#SAKNAS!
#SAKNAS!
#SAKNAS!
#SAKNAS!
#SAKNAS!
2395 (0.036)
#SAKNAS!
#SAKNAS!
#SAKNAS!
#SAKNAS!
#SAKNAS!
#SAKNAS!
#SAKNAS!
#SAKNAS!
#SAKNAS!
2395 (0.036)
#SAKNAS!
#SAKNAS!
#SAKNAS!
#SAKNAS!
#SAKNAS!
#SAKNAS!
#SAKNAS!
#SAKNAS!
2395 (0.036)
2395 (0.036)
#SAKNAS!
#SAKNAS!
#SAKNAS!
#SAKNAS!
#SAKNAS!
#SAKNAS!
#SAKNAS!
#SAKNAS!
2395 (0.036)
2395 (0.036)
#SAKNAS!
#SAKNAS!
#SAKNAS!

#SAKNAS!
#SAKNAS!
#SAKNAS!
#SAKNAS!
2395 (0.036)
#SAKNAS!
#SAKNAS!
#SAKNAS!
#SAKNAS!
#SAKNAS!
2395 (0.036)
#SAKNAS!
#SAKNAS!
#SAKNAS!
2395 (0.036)
2395 (0.036)
#SAKNAS!
#SAKNAS!
#SAKNAS!
#SAKNAS!
#SAKNAS!
#SAKNAS!
2395 (0.036)
2395 (0.036)
#SAKNAS!
#SAKNAS!
#SAKNAS!
#SAKNAS!
2395 (0.036)
#SAKNAS!
#SAKNAS!
2395 (0.036)
2395 (0.036)
2395 (0.036)
#SAKNAS!
#SAKNAS!
#SAKNAS!
#SAKNAS!
2395 (0.036)
#SAKNAS!
#SAKNAS!
2395 (0.036)
#SAKNAS!

#SAKNAS!
#SAKNAS!
#SAKNAS!
#SAKNAS!
#SAKNAS!
#SAKNAS!
#SAKNAS!
#SAKNAS!
#SAKNAS!
#SAKNAS!
#SAKNAS!
#SAKNAS!
#SAKNAS!
#SAKNAS!
#SAKNAS!
#SAKNAS!
#SAKNAS!
#SAKNAS!
2395 (0.036)
#SAKNAS!
2395 (0.036)
#SAKNAS!
#SAKNAS!
2395 (0.036)
2395 (0.036)
2395 (0.036)
#SAKNAS!
2395 (0.036)
#SAKNAS!
#SAKNAS!
#SAKNAS!
#SAKNAS!
2395 (0.036)
2395 (0.036)
2395 (0.036)
2395 (0.036)
#SAKNAS!
2395 (0.036)
#SAKNAS!
#SAKNAS!
#SAKNAS!
#SAKNAS!
#SAKNAS!

#SAKNAS!
#SAKNAS!
#SAKNAS!
#SAKNAS!
#SAKNAS!
#SAKNAS!
#SAKNAS!
#SAKNAS!
2395 (0.036)
2395 (0.036)
#SAKNAS!
#SAKNAS!
#SAKNAS!
#SAKNAS!
#SAKNAS!
#SAKNAS!
2395 (0.036)
2395 (0.036)
#SAKNAS!
#SAKNAS!
#SAKNAS!
#SAKNAS!
#SAKNAS!
#SAKNAS!
#SAKNAS!
#SAKNAS!
2395 (0.036)
#SAKNAS!
#SAKNAS!
#SAKNAS!
2395 (0.036)
#SAKNAS!
2395 (0.036)
2395 (0.036)
1164 (0.11)
#SAKNAS!
#SAKNAS!
#SAKNAS!
2395 (0.036)
2395 (0.036)
#SAKNAS!
#SAKNAS!
#SAKNAS!

#SAKNAS!
#SAKNAS!
#SAKNAS!
2395 (0.036)
#SAKNAS!
#SAKNAS!
2395 (0.036)
2395 (0.036)
#SAKNAS!
#SAKNAS!
#SAKNAS!
#SAKNAS!
#SAKNAS!
#SAKNAS!
#SAKNAS!
#SAKNAS!
#SAKNAS!
#SAKNAS!
#SAKNAS!
#SAKNAS!
#SAKNAS!
2395 (0.036)
1573 (0.071)
#SAKNAS!
#SAKNAS!
#SAKNAS!
2395 (0.036)
2395 (0.036)
#SAKNAS!
#SAKNAS!
#SAKNAS!
#SAKNAS!
#SAKNAS!
#SAKNAS!
#SAKNAS!
1573 (0.071)
#SAKNAS!
#SAKNAS!
2395 (0.036)
#SAKNAS!
1573 (0.071)
2395 (0.036)
#SAKNAS!

#SAKNAS!
#SAKNAS!
#SAKNAS!
#SAKNAS!
1573 (0.071)
2395 (0.036)
#SAKNAS!
#SAKNAS!
936 (0.14)
2395 (0.036)
2395 (0.036)
2395 (0.036)
648 (0.21)
2395 (0.036)
#SAKNAS!
2395 (0.036)
#SAKNAS!
936 (0.14)
#SAKNAS!
#SAKNAS!
#SAKNAS!
#SAKNAS!
2395 (0.036)
#SAKNAS!
#SAKNAS!
#SAKNAS!
1573 (0.071)
#SAKNAS!
#SAKNAS!
2395 (0.036)
#SAKNAS!
2395 (0.036)
#SAKNAS!
#SAKNAS!
#SAKNAS!
2395 (0.036)
2395 (0.036)
2395 (0.036)
#SAKNAS!
2395 (0.036)
#SAKNAS!
2395 (0.036)
#SAKNAS!

2395 (0.036)
2395 (0.036)
#SAKNAS!
#SAKNAS!
#SAKNAS!
#SAKNAS!
#SAKNAS!
2395 (0.036)
2395 (0.036)
#SAKNAS!
2395 (0.036)
#SAKNAS!
2395 (0.036)
#SAKNAS!
#SAKNAS!
#SAKNAS!
#SAKNAS!
#SAKNAS!
2395 (0.036)
#SAKNAS!
2395 (0.036)
#SAKNAS!
#SAKNAS!
2395 (0.036)
#SAKNAS!
#SAKNAS!
#SAKNAS!
#SAKNAS!
#SAKNAS!
#SAKNAS!
2395 (0.036)
#SAKNAS!
#SAKNAS!
#SAKNAS!
2395 (0.036)
#SAKNAS!
#SAKNAS!
#SAKNAS!
2395 (0.036)
#SAKNAS!
#SAKNAS!
#SAKNAS!
#SAKNAS!

#SAKNAS!
2395 (0.036)
1573 (0.071)
2395 (0.036)
#SAKNAS!
#SAKNAS!
#SAKNAS!
#SAKNAS!
#SAKNAS!
#SAKNAS!
#SAKNAS!
#SAKNAS!
#SAKNAS!
#SAKNAS!
#SAKNAS!
#SAKNAS!
#SAKNAS!
#SAKNAS!
2395 (0.036)
#SAKNAS!
#SAKNAS!
#SAKNAS!
2395 (0.036)
2395 (0.036)
#SAKNAS!
#SAKNAS!
#SAKNAS!
#SAKNAS!
#SAKNAS!
#SAKNAS!
2395 (0.036)
#SAKNAS!
#SAKNAS!
2395 (0.036)
2395 (0.036)
2395 (0.036)
#SAKNAS!
2395 (0.036)
#SAKNAS!
#SAKNAS!
936 (0.14)
2395 (0.036)
2395 (0.036)

2395 (0.036)
1164 (0.11)
2395 (0.036)
2395 (0.036)
2395 (0.036)
#SAKNAS!
#SAKNAS!
#SAKNAS!
1164 (0.11)
2395 (0.036)
#SAKNAS!
#SAKNAS!
2395 (0.036)
#SAKNAS!
#SAKNAS!
#SAKNAS!
2395 (0.036)
#SAKNAS!
#SAKNAS!
#SAKNAS!
1164 (0.11)
#SAKNAS!
#SAKNAS!
#SAKNAS!
773 (0.18)
2395 (0.036)
#SAKNAS!
#SAKNAS!
#SAKNAS!
#SAKNAS!
#SAKNAS!
#SAKNAS!
2395 (0.036)
#SAKNAS!
#SAKNAS!
2395 (0.036)
2395 (0.036)
#SAKNAS!
#SAKNAS!
2395 (0.036)
#SAKNAS!
#SAKNAS!
#SAKNAS!

#SAKNAS!
2395 (0.036)
#SAKNAS!
#SAKNAS!
#SAKNAS!
#SAKNAS!
#SAKNAS!
#SAKNAS!
#SAKNAS!
2395 (0.036)
2395 (0.036)
2395 (0.036)
#SAKNAS!
2395 (0.036)
#SAKNAS!
#SAKNAS!
#SAKNAS!
#SAKNAS!
#SAKNAS!
#SAKNAS!
#SAKNAS!
#SAKNAS!
#SAKNAS!
#SAKNAS!
936 (0.14)
2395 (0.036)
2395 (0.036)
2395 (0.036)
#SAKNAS!
2395 (0.036)
2395 (0.036)
2395 (0.036)
#SAKNAS!
2395 (0.036)
#SAKNAS!
#SAKNAS!
2395 (0.036)
2395 (0.036)
#SAKNAS!
#SAKNAS!
#SAKNAS!
2395 (0.036)
#SAKNAS!

#SAKNAS!
#SAKNAS!
#SAKNAS!
2395 (0.036)
#SAKNAS!
2395 (0.036)
#SAKNAS!
#SAKNAS!
#SAKNAS!
#SAKNAS!
#SAKNAS!
#SAKNAS!
2395 (0.036)
#SAKNAS!
#SAKNAS!
2395 (0.036)
2395 (0.036)
#SAKNAS!
#SAKNAS!
2395 (0.036)
370 (0.39)
#SAKNAS!
#SAKNAS!
#SAKNAS!
#SAKNAS!
#SAKNAS!
#SAKNAS!
#SAKNAS!
#SAKNAS!
1164 (0.11)
#SAKNAS!
2395 (0.036)
#SAKNAS!
#SAKNAS!
1573 (0.071)
2395 (0.036)
#SAKNAS!
#SAKNAS!
2395 (0.036)
2395 (0.036)
#SAKNAS!
#SAKNAS!
2395 (0.036)

#SAKNAS!
#SAKNAS!
#SAKNAS!
#SAKNAS!
#SAKNAS!
#SAKNAS!
#SAKNAS!
2395 (0.036)
#SAKNAS!
#SAKNAS!
#SAKNAS!
#SAKNAS!
#SAKNAS!
2395 (0.036)
#SAKNAS!
#SAKNAS!
2395 (0.036)
#SAKNAS!
#SAKNAS!
#SAKNAS!
2395 (0.036)
2395 (0.036)
#SAKNAS!
#SAKNAS!
2395 (0.036)
#SAKNAS!
#SAKNAS!
#SAKNAS!
#SAKNAS!
#SAKNAS!
#SAKNAS!
2395 (0.036)
2395 (0.036)
#SAKNAS!
2395 (0.036)
#SAKNAS!
#SAKNAS!
2395 (0.036)
#SAKNAS!
1573 (0.071)
2395 (0.036)
#SAKNAS!
#SAKNAS!

#SAKNAS!
#SAKNAS!
2395 (0.036)
#SAKNAS!
2395 (0.036)
#SAKNAS!
#SAKNAS!
#SAKNAS!
#SAKNAS!
#SAKNAS!
#SAKNAS!
#SAKNAS!
#SAKNAS!
#SAKNAS!
#SAKNAS!
#SAKNAS!
2395 (0.036)
#SAKNAS!
#SAKNAS!
#SAKNAS!
2395 (0.036)
2395 (0.036)
2395 (0.036)
#SAKNAS!
#SAKNAS!
#SAKNAS!
#SAKNAS!
#SAKNAS!
#SAKNAS!
#SAKNAS!
#SAKNAS!
2395 (0.036)
2395 (0.036)
648 (0.21)
#SAKNAS!
#SAKNAS!
#SAKNAS!
#SAKNAS!
#SAKNAS!
#SAKNAS!
2395 (0.036)
#SAKNAS!
2395 (0.036)

2395 (0.036)
#SAKNAS!
#SAKNAS!
2395 (0.036)
#SAKNAS!
#SAKNAS!
#SAKNAS!
#SAKNAS!
#SAKNAS!
#SAKNAS!
#SAKNAS!
#SAKNAS!
#SAKNAS!
#SAKNAS!
#SAKNAS!
#SAKNAS!
#SAKNAS!
#SAKNAS!
2395 (0.036)
#SAKNAS!
#SAKNAS!
#SAKNAS!
#SAKNAS!
#SAKNAS!
#SAKNAS!
#SAKNAS!
#SAKNAS!
301 (0.5)
#SAKNAS!
#SAKNAS!
2395 (0.036)
2395 (0.036)
#SAKNAS!
#SAKNAS!
#SAKNAS!
#SAKNAS!
#SAKNAS!
2395 (0.036)
2395 (0.036)
#SAKNAS!
#SAKNAS!
2395 (0.036)
936 (0.14)

#SAKNAS!
#SAKNAS!
2395 (0.036)
#SAKNAS!
#SAKNAS!
#SAKNAS!
#SAKNAS!
#SAKNAS!
2395 (0.036)
#SAKNAS!
#SAKNAS!
#SAKNAS!
#SAKNAS!
#SAKNAS!
#SAKNAS!
#SAKNAS!
2395 (0.036)
#SAKNAS!
#SAKNAS!
#SAKNAS!
#SAKNAS!
#SAKNAS!
2395 (0.036)
2395 (0.036)
#SAKNAS!
#SAKNAS!
#SAKNAS!
1164 (0.11)
#SAKNAS!
#SAKNAS!
2395 (0.036)
2395 (0.036)
#SAKNAS!
#SAKNAS!
2395 (0.036)
1573 (0.071)
1573 (0.071)
2395 (0.036)
2395 (0.036)
#SAKNAS!
#SAKNAS!
#SAKNAS!
#SAKNAS!

#SAKNAS!
#SAKNAS!
2395 (0.036)
2395 (0.036)
#SAKNAS!
#SAKNAS!
#SAKNAS!
#SAKNAS!
2395 (0.036)
2395 (0.036)
2395 (0.036)
2395 (0.036)
#SAKNAS!
#SAKNAS!
#SAKNAS!
#SAKNAS!
#SAKNAS!
#SAKNAS!
#SAKNAS!
#SAKNAS!
2395 (0.036)
#SAKNAS!
#SAKNAS!
#SAKNAS!
#SAKNAS!
2395 (0.036)
#SAKNAS!
#SAKNAS!
#SAKNAS!
#SAKNAS!
#SAKNAS!
1573 (0.071)
#SAKNAS!
2395 (0.036)
#SAKNAS!
#SAKNAS!
#SAKNAS!
#SAKNAS!
#SAKNAS!
#SAKNAS!
#SAKNAS!
#SAKNAS!
#SAKNAS!

411 (0.36)
#SAKNAS!
#SAKNAS!
#SAKNAS!
#SAKNAS!
#SAKNAS!
#SAKNAS!
#SAKNAS!
#SAKNAS!
#SAKNAS!
#SAKNAS!
#SAKNAS!
#SAKNAS!
#SAKNAS!
#SAKNAS!
#SAKNAS!
#SAKNAS!
#SAKNAS!
#SAKNAS!
#SAKNAS!
#SAKNAS!
#SAKNAS!
2395 (0.036)
#SAKNAS!
#SAKNAS!
#SAKNAS!
2395 (0.036)
2395 (0.036)
2395 (0.036)
2395 (0.036)
#SAKNAS!
2395 (0.036)
#SAKNAS!
#SAKNAS!
#SAKNAS!
2395 (0.036)
2395 (0.036)
#SAKNAS!
#SAKNAS!
#SAKNAS!
#SAKNAS!
#SAKNAS!
#SAKNAS!

#SAKNAS!
#SAKNAS!
2395 (0.036)
#SAKNAS!
#SAKNAS!
#SAKNAS!
#SAKNAS!
1164 (0.11)
2395 (0.036)
#SAKNAS!
2395 (0.036)
#SAKNAS!
2395 (0.036)
#SAKNAS!
#SAKNAS!
#SAKNAS!
#SAKNAS!
#SAKNAS!
2395 (0.036)
2395 (0.036)
2395 (0.036)
2395 (0.036)
2395 (0.036)
2395 (0.036)
2395 (0.036)
#SAKNAS!
#SAKNAS!
#SAKNAS!
#SAKNAS!
2395 (0.036)
#SAKNAS!
2395 (0.036)
#SAKNAS!
#SAKNAS!
2395 (0.036)
#SAKNAS!
2395 (0.036)
2395 (0.036)
#SAKNAS!
2395 (0.036)
#SAKNAS!
#SAKNAS!
#SAKNAS!

#SAKNAS!
#SAKNAS!
2395 (0.036)
#SAKNAS!
#SAKNAS!
#SAKNAS!
#SAKNAS!
#SAKNAS!
#SAKNAS!
#SAKNAS!
#SAKNAS!
#SAKNAS!
#SAKNAS!
2395 (0.036)
#SAKNAS!
#SAKNAS!
#SAKNAS!
#SAKNAS!
#SAKNAS!
2395 (0.036)
446 (0.32)
#SAKNAS!
#SAKNAS!
2395 (0.036)
2395 (0.036)
2395 (0.036)
#SAKNAS!
#SAKNAS!
2395 (0.036)
2395 (0.036)
2395 (0.036)
#SAKNAS!
#SAKNAS!
2395 (0.036)
#SAKNAS!
#SAKNAS!
2395 (0.036)
#SAKNAS!
#SAKNAS!
2395 (0.036)
#SAKNAS!
#SAKNAS!
#SAKNAS!

#SAKNAS!
#SAKNAS!
2395 (0.036)
#SAKNAS!
#SAKNAS!
2395 (0.036)
#SAKNAS!
#SAKNAS!
2395 (0.036)
2395 (0.036)
#SAKNAS!
#SAKNAS!
#SAKNAS!
#SAKNAS!
#SAKNAS!
2395 (0.036)
#SAKNAS!
#SAKNAS!
2395 (0.036)
#SAKNAS!
#SAKNAS!
#SAKNAS!
#SAKNAS!
773 (0.18)
#SAKNAS!
2395 (0.036)
#SAKNAS!
#SAKNAS!
#SAKNAS!
#SAKNAS!
#SAKNAS!
#SAKNAS!
2395 (0.036)
2395 (0.036)
#SAKNAS!
#SAKNAS!
#SAKNAS!
#SAKNAS!
#SAKNAS!
2395 (0.036)
2395 (0.036)
2395 (0.036)
#SAKNAS!

2395 (0.036)
2395 (0.036)
2395 (0.036)
2395 (0.036)
#SAKNAS!
#SAKNAS!
#SAKNAS!
2395 (0.036)
1573 (0.071)
2395 (0.036)
#SAKNAS!
#SAKNAS!
#SAKNAS!
#SAKNAS!
#SAKNAS!
2395 (0.036)
#SAKNAS!
2395 (0.036)
1164 (0.11)
2395 (0.036)
#SAKNAS!
#SAKNAS!
2395 (0.036)
2395 (0.036)
2395 (0.036)
2395 (0.036)
2395 (0.036)
2395 (0.036)
#SAKNAS!
#SAKNAS!
#SAKNAS!
#SAKNAS!
1164 (0.11)
#SAKNAS!
#SAKNAS!
#SAKNAS!
#SAKNAS!
#SAKNAS!
#SAKNAS!
2395 (0.036)
773 (0.18)
#SAKNAS!
#SAKNAS!

#SAKNAS!
#SAKNAS!
#SAKNAS!
2395 (0.036)
2395 (0.036)
#SAKNAS!
#SAKNAS!
#SAKNAS!
2395 (0.036)
2395 (0.036)
2395 (0.036)
#SAKNAS!
#SAKNAS!
2395 (0.036)
#SAKNAS!
#SAKNAS!
2395 (0.036)
#SAKNAS!
2395 (0.036)
2395 (0.036)
#SAKNAS!
#SAKNAS!
#SAKNAS!
#SAKNAS!
#SAKNAS!
#SAKNAS!
#SAKNAS!
#SAKNAS!
2395 (0.036)
#SAKNAS!
#SAKNAS!
2395 (0.036)
2395 (0.036)
2395 (0.036)
#SAKNAS!
1164 (0.11)
2395 (0.036)
#SAKNAS!
#SAKNAS!
#SAKNAS!
#SAKNAS!
#SAKNAS!
#SAKNAS!

#SAKNAS!
#SAKNAS!
#SAKNAS!
#SAKNAS!
#SAKNAS!
#SAKNAS!
#SAKNAS!
2395 (0.036)
1573 (0.071)
2395 (0.036)
2395 (0.036)
2395 (0.036)
#SAKNAS!
#SAKNAS!
#SAKNAS!
#SAKNAS!
#SAKNAS!
#SAKNAS!
#SAKNAS!
#SAKNAS!
#SAKNAS!
1164 (0.11)
#SAKNAS!
2395 (0.036)
2395 (0.036)
#SAKNAS!
#SAKNAS!
#SAKNAS!
#SAKNAS!
2395 (0.036)
#SAKNAS!
#SAKNAS!
#SAKNAS!
#SAKNAS!
2395 (0.036)
#SAKNAS!
#SAKNAS!
#SAKNAS!
#SAKNAS!
#SAKNAS!
2395 (0.036)
2395 (0.036)
1164 (0.11)

#SAKNAS!
#SAKNAS!
2395 (0.036)
2395 (0.036)
2395 (0.036)
2395 (0.036)
2395 (0.036)
#SAKNAS!
#SAKNAS!
#SAKNAS!
#SAKNAS!
#SAKNAS!
#SAKNAS!
#SAKNAS!
2395 (0.036)
2395 (0.036)
#SAKNAS!
936 (0.14)
2395 (0.036)
2395 (0.036)
#SAKNAS!
#SAKNAS!
#SAKNAS!
2395 (0.036)
2395 (0.036)
#SAKNAS!
#SAKNAS!
#SAKNAS!
#SAKNAS!
2395 (0.036)
#SAKNAS!
#SAKNAS!
2395 (0.036)
#SAKNAS!
#SAKNAS!
#SAKNAS!
#SAKNAS!
2395 (0.036)
2395 (0.036)
#SAKNAS!
#SAKNAS!
1573 (0.071)
#SAKNAS!

#SAKNAS!
2395 (0.036)
2395 (0.036)
2395 (0.036)
#SAKNAS!
#SAKNAS!
#SAKNAS!
2395 (0.036)
2395 (0.036)
#SAKNAS!
#SAKNAS!
#SAKNAS!
2395 (0.036)
#SAKNAS!
#SAKNAS!
2395 (0.036)
#SAKNAS!
#SAKNAS!
#SAKNAS!
2395 (0.036)
#SAKNAS!
#SAKNAS!
2395 (0.036)
#SAKNAS!
#SAKNAS!
2395 (0.036)
2395 (0.036)
2395 (0.036)
#SAKNAS!
#SAKNAS!
#SAKNAS!
#SAKNAS!
2395 (0.036)
#SAKNAS!
#SAKNAS!
#SAKNAS!
#SAKNAS!
#SAKNAS!
#SAKNAS!
#SAKNAS!
2395 (0.036)
#SAKNAS!
#SAKNAS!

#SAKNAS!
#SAKNAS!
#SAKNAS!
#SAKNAS!
#SAKNAS!
#SAKNAS!
#SAKNAS!
#SAKNAS!
#SAKNAS!
#SAKNAS!
#SAKNAS!
#SAKNAS!
2395 (0.036)
#SAKNAS!
#SAKNAS!
936 (0.14)
#SAKNAS!
#SAKNAS!
#SAKNAS!
#SAKNAS!
2395 (0.036)
#SAKNAS!
#SAKNAS!
#SAKNAS!
#SAKNAS!
#SAKNAS!
#SAKNAS!
#SAKNAS!
#SAKNAS!
#SAKNAS!
2395 (0.036)
2395 (0.036)
#SAKNAS!
2395 (0.036)
#SAKNAS!
2395 (0.036)
#SAKNAS!
2395 (0.036)
#SAKNAS!
#SAKNAS!
#SAKNAS!
#SAKNAS!
#SAKNAS!

2395 (0.036)
2395 (0.036)
#SAKNAS!
#SAKNAS!
2395 (0.036)
2395 (0.036)
2395 (0.036)
#SAKNAS!
#SAKNAS!
#SAKNAS!
#SAKNAS!
#SAKNAS!
#SAKNAS!
#SAKNAS!
#SAKNAS!
2395 (0.036)
#SAKNAS!
#SAKNAS!
#SAKNAS!
#SAKNAS!
2395 (0.036)
2395 (0.036)
2395 (0.036)
2395 (0.036)
2395 (0.036)
#SAKNAS!
2395 (0.036)
2395 (0.036)
2395 (0.036)
#SAKNAS!
#SAKNAS!
2395 (0.036)
#SAKNAS!
#SAKNAS!
#SAKNAS!
#SAKNAS!
#SAKNAS!
1164 (0.11)
2395 (0.036)
2395 (0.036)
2395 (0.036)
#SAKNAS!
#SAKNAS!

#SAKNAS!
#SAKNAS!
2395 (0.036)
2395 (0.036)
2395 (0.036)
#SAKNAS!
2395 (0.036)
2395 (0.036)
2395 (0.036)
#SAKNAS!
#SAKNAS!
#SAKNAS!
#SAKNAS!
#SAKNAS!
#SAKNAS!
#SAKNAS!
#SAKNAS!
#SAKNAS!
#SAKNAS!
#SAKNAS!
#SAKNAS!
2395 (0.036)
2395 (0.036)
#SAKNAS!
2395 (0.036)
#SAKNAS!
2395 (0.036)
#SAKNAS!
#SAKNAS!
#SAKNAS!
#SAKNAS!
#SAKNAS!
2395 (0.036)
#SAKNAS!
#SAKNAS!
2395 (0.036)
#SAKNAS!
#SAKNAS!
#SAKNAS!
#SAKNAS!
1573 (0.071)
#SAKNAS!
#SAKNAS!

#SAKNAS!
#SAKNAS!
#SAKNAS!
#SAKNAS!
#SAKNAS!
#SAKNAS!
#SAKNAS!
#SAKNAS!
#SAKNAS!
#SAKNAS!
#SAKNAS!
#SAKNAS!
#SAKNAS!
#SAKNAS!
2395 (0.036)
#SAKNAS!
#SAKNAS!
#SAKNAS!
2395 (0.036)
#SAKNAS!
#SAKNAS!
#SAKNAS!
#SAKNAS!
#SAKNAS!
#SAKNAS!
2395 (0.036)
2395 (0.036)
#SAKNAS!
#SAKNAS!
2395 (0.036)
2395 (0.036)
2395 (0.036)
2395 (0.036)
2395 (0.036)
#SAKNAS!
2395 (0.036)
2395 (0.036)
2395 (0.036)
#SAKNAS!
#SAKNAS!
#SAKNAS!
#SAKNAS!
#SAKNAS!

2395 (0.036)
#SAKNAS!
2395 (0.036)
2395 (0.036)
#SAKNAS!
#SAKNAS!
#SAKNAS!
#SAKNAS!
#SAKNAS!
#SAKNAS!
2395 (0.036)
2395 (0.036)
2395 (0.036)
#SAKNAS!
2395 (0.036)
#SAKNAS!
#SAKNAS!
#SAKNAS!
#SAKNAS!
#SAKNAS!
#SAKNAS!
#SAKNAS!
#SAKNAS!
2395 (0.036)
2395 (0.036)
#SAKNAS!
2395 (0.036)
773 (0.18)
#SAKNAS!
#SAKNAS!
#SAKNAS!
#SAKNAS!
#SAKNAS!
2395 (0.036)
#SAKNAS!
#SAKNAS!
2395 (0.036)
#SAKNAS!
#SAKNAS!
#SAKNAS!
#SAKNAS!
#SAKNAS!
#SAKNAS!

#SAKNAS!
#SAKNAS!
#SAKNAS!
#SAKNAS!
#SAKNAS!
2395 (0.036)
#SAKNAS!
#SAKNAS!
#SAKNAS!
2395 (0.036)
#SAKNAS!
#SAKNAS!
#SAKNAS!
#SAKNAS!
#SAKNAS!
#SAKNAS!
#SAKNAS!
#SAKNAS!
#SAKNAS!
#SAKNAS!
#SAKNAS!
#SAKNAS!
#SAKNAS!
#SAKNAS!
#SAKNAS!
#SAKNAS!
#SAKNAS!
#SAKNAS!
#SAKNAS!
#SAKNAS!
#SAKNAS!
2395 (0.036)
#SAKNAS!
#SAKNAS!
#SAKNAS!
#SAKNAS!
1573 (0.071)
#SAKNAS!
#SAKNAS!
2395 (0.036)
#SAKNAS!
#SAKNAS!
#SAKNAS!

1164 (0.11)
2395 (0.036)
#SAKNAS!
#SAKNAS!
2395 (0.036)
#SAKNAS!
#SAKNAS!
#SAKNAS!
2395 (0.036)
#SAKNAS!
2395 (0.036)
2395 (0.036)
2395 (0.036)
648 (0.21)
#SAKNAS!
#SAKNAS!
2395 (0.036)
1573 (0.071)
2395 (0.036)
2395 (0.036)
2395 (0.036)
#SAKNAS!
#SAKNAS!
#SAKNAS!
#SAKNAS!
#SAKNAS!
#SAKNAS!
#SAKNAS!
#SAKNAS!
#SAKNAS!
#SAKNAS!
#SAKNAS!
#SAKNAS!
#SAKNAS!
#SAKNAS!
#SAKNAS!
#SAKNAS!
#SAKNAS!
2395 (0.036)
#SAKNAS!
#SAKNAS!
2395 (0.036)
2395 (0.036)

#SAKNAS!
#SAKNAS!
2395 (0.036)
2395 (0.036)
2395 (0.036)
#SAKNAS!
2395 (0.036)
#SAKNAS!
2395 (0.036)
1573 (0.071)
#SAKNAS!
2395 (0.036)
2395 (0.036)
#SAKNAS!
#SAKNAS!
#SAKNAS!
#SAKNAS!
#SAKNAS!
2395 (0.036)
#SAKNAS!
#SAKNAS!
2395 (0.036)
#SAKNAS!
#SAKNAS!
#SAKNAS!
#SAKNAS!
#SAKNAS!
#SAKNAS!
#SAKNAS!
#SAKNAS!
1573 (0.071)
#SAKNAS!
#SAKNAS!
#SAKNAS!
2395 (0.036)
2395 (0.036)
#SAKNAS!
#SAKNAS!
#SAKNAS!
#SAKNAS!
#SAKNAS!
#SAKNAS!
#SAKNAS!

#SAKNAS!
#SAKNAS!
#SAKNAS!
#SAKNAS!
#SAKNAS!
#SAKNAS!
#SAKNAS!
2395 (0.036)
2395 (0.036)
#SAKNAS!
#SAKNAS!
#SAKNAS!
#SAKNAS!
1573 (0.071)
2395 (0.036)
2395 (0.036)
#SAKNAS!
#SAKNAS!
#SAKNAS!
#SAKNAS!
#SAKNAS!
#SAKNAS!
1573 (0.071)
#SAKNAS!
#SAKNAS!
#SAKNAS!
#SAKNAS!
#SAKNAS!
#SAKNAS!
#SAKNAS!
#SAKNAS!
#SAKNAS!
#SAKNAS!
936 (0.14)
2395 (0.036)
2395 (0.036)
#SAKNAS!
#SAKNAS!
#SAKNAS!
#SAKNAS!
2395 (0.036)
#SAKNAS!
#SAKNAS!

#SAKNAS!
#SAKNAS!
2395 (0.036)
#SAKNAS!
#SAKNAS!
#SAKNAS!
#SAKNAS!
2395 (0.036)
2395 (0.036)
1164 (0.11)
2395 (0.036)
#SAKNAS!
2395 (0.036)
2395 (0.036)
#SAKNAS!
#SAKNAS!
2395 (0.036)
#SAKNAS!
#SAKNAS!
#SAKNAS!
#SAKNAS!
2395 (0.036)
#SAKNAS!
#SAKNAS!
#SAKNAS!
2395 (0.036)
#SAKNAS!
#SAKNAS!
#SAKNAS!
#SAKNAS!
#SAKNAS!
#SAKNAS!
#SAKNAS!
2395 (0.036)
2395 (0.036)
#SAKNAS!
#SAKNAS!
#SAKNAS!
#SAKNAS!
#SAKNAS!
#SAKNAS!
#SAKNAS!
2395 (0.036)

#SAKNAS!
2395 (0.036)
2395 (0.036)
2395 (0.036)
#SAKNAS!
2395 (0.036)
2395 (0.036)
#SAKNAS!
2395 (0.036)
2395 (0.036)
#SAKNAS!
#SAKNAS!
#SAKNAS!
#SAKNAS!
#SAKNAS!
#SAKNAS!
#SAKNAS!
#SAKNAS!
#SAKNAS!
#SAKNAS!
2395 (0.036)
#SAKNAS!
#SAKNAS!
2395 (0.036)
2395 (0.036)
#SAKNAS!
#SAKNAS!
#SAKNAS!
#SAKNAS!
#SAKNAS!
#SAKNAS!
#SAKNAS!
#SAKNAS!
#SAKNAS!
#SAKNAS!
2395 (0.036)
2395 (0.036)
#SAKNAS!
#SAKNAS!
2395 (0.036)
#SAKNAS!
#SAKNAS!
773 (0.18)

#SAKNAS!
#SAKNAS!
#SAKNAS!
#SAKNAS!
1573 (0.071)
#SAKNAS!
#SAKNAS!
#SAKNAS!
2395 (0.036)
#SAKNAS!
#SAKNAS!
#SAKNAS!
#SAKNAS!
#SAKNAS!
#SAKNAS!
#SAKNAS!
2395 (0.036)
#SAKNAS!
#SAKNAS!
2395 (0.036)
#SAKNAS!
#SAKNAS!
#SAKNAS!
2395 (0.036)
1164 (0.11)
2395 (0.036)
#SAKNAS!
2395 (0.036)
2395 (0.036)
2395 (0.036)
#SAKNAS!
2395 (0.036)
2395 (0.036)
2395 (0.036)
#SAKNAS!
#SAKNAS!
#SAKNAS!
#SAKNAS!
#SAKNAS!
#SAKNAS!
#SAKNAS!
#SAKNAS!
#SAKNAS!

2395 (0.036)
2395 (0.036)
#SAKNAS!
#SAKNAS!
#SAKNAS!
#SAKNAS!
2395 (0.036)
2395 (0.036)
2395 (0.036)
#SAKNAS!
2395 (0.036)
#SAKNAS!
936 (0.14)
1573 (0.071)
2395 (0.036)
#SAKNAS!
#SAKNAS!
#SAKNAS!
2395 (0.036)
#SAKNAS!
#SAKNAS!
#SAKNAS!
#SAKNAS!
2395 (0.036)
#SAKNAS!
#SAKNAS!
#SAKNAS!
2395 (0.036)
2395 (0.036)
2395 (0.036)
#SAKNAS!
2395 (0.036)
2395 (0.036)
2395 (0.036)
#SAKNAS!
#SAKNAS!
#SAKNAS!
2395 (0.036)
1573 (0.071)
#SAKNAS!
#SAKNAS!
#SAKNAS!
2395 (0.036)

#SAKNAS!
#SAKNAS!
#SAKNAS!
#SAKNAS!
#SAKNAS!
2395 (0.036)
2395 (0.036)
#SAKNAS!
#SAKNAS!
#SAKNAS!
#SAKNAS!
1573 (0.071)
2395 (0.036)
#SAKNAS!
#SAKNAS!
#SAKNAS!
#SAKNAS!
#SAKNAS!
2395 (0.036)
#SAKNAS!
#SAKNAS!
2395 (0.036)
#SAKNAS!
#SAKNAS!
2395 (0.036)
2395 (0.036)
2395 (0.036)
2395 (0.036)
#SAKNAS!
#SAKNAS!
#SAKNAS!
#SAKNAS!
#SAKNAS!
#SAKNAS!
#SAKNAS!
#SAKNAS!
#SAKNAS!
#SAKNAS!
2395 (0.036)
#SAKNAS!
#SAKNAS!
2395 (0.036)
#SAKNAS!

2395 (0.036)
#SAKNAS!
2395 (0.036)
2395 (0.036)
#SAKNAS!
2395 (0.036)
2395 (0.036)
1164 (0.11)
#SAKNAS!
#SAKNAS!
#SAKNAS!
2395 (0.036)
2395 (0.036)
1573 (0.071)
#SAKNAS!
#SAKNAS!
#SAKNAS!
#SAKNAS!
#SAKNAS!
#SAKNAS!
#SAKNAS!
#SAKNAS!
#SAKNAS!
#SAKNAS!
#SAKNAS!
#SAKNAS!
#SAKNAS!
#SAKNAS!
#SAKNAS!
#SAKNAS!
#SAKNAS!
#SAKNAS!
2395 (0.036)
2395 (0.036)
#SAKNAS!
#SAKNAS!
#SAKNAS!
#SAKNAS!
#SAKNAS!
#SAKNAS!
2395 (0.036)
#SAKNAS!
#SAKNAS!

2395 (0.036)
2395 (0.036)
1573 (0.071)
#SAKNAS!
#SAKNAS!
#SAKNAS!
#SAKNAS!
#SAKNAS!
2395 (0.036)
2395 (0.036)
#SAKNAS!
#SAKNAS!
2395 (0.036)
#SAKNAS!
#SAKNAS!
2395 (0.036)
#SAKNAS!
#SAKNAS!
2395 (0.036)
#SAKNAS!
#SAKNAS!
#SAKNAS!
#SAKNAS!
1573 (0.071)
2395 (0.036)
#SAKNAS!
#SAKNAS!
2395 (0.036)
#SAKNAS!
#SAKNAS!
#SAKNAS!
2395 (0.036)
#SAKNAS!
2395 (0.036)
#SAKNAS!
#SAKNAS!
#SAKNAS!
#SAKNAS!
#SAKNAS!
#SAKNAS!
#SAKNAS!
2395 (0.036)
2395 (0.036)

#SAKNAS!
#SAKNAS!
#SAKNAS!
#SAKNAS!
#SAKNAS!
1164 (0.11)
2395 (0.036)
#SAKNAS!
#SAKNAS!
#SAKNAS!
#SAKNAS!
1573 (0.071)
#SAKNAS!
#SAKNAS!
#SAKNAS!
#SAKNAS!
#SAKNAS!
#SAKNAS!
#SAKNAS!
#SAKNAS!
2395 (0.036)
#SAKNAS!
2395 (0.036)
#SAKNAS!
2395 (0.036)
1573 (0.071)
#SAKNAS!
#SAKNAS!
#SAKNAS!
#SAKNAS!
2395 (0.036)
#SAKNAS!
#SAKNAS!
2395 (0.036)
#SAKNAS!
#SAKNAS!
2395 (0.036)
#SAKNAS!
#SAKNAS!
#SAKNAS!
#SAKNAS!
#SAKNAS!
2395 (0.036)

2395 (0.036)
2395 (0.036)
#SAKNAS!
#SAKNAS!
#SAKNAS!
#SAKNAS!
#SAKNAS!
#SAKNAS!
2395 (0.036)
#SAKNAS!
#SAKNAS!
#SAKNAS!
#SAKNAS!
1164 (0.11)
2395 (0.036)
#SAKNAS!
#SAKNAS!
2395 (0.036)
#SAKNAS!
#SAKNAS!
#SAKNAS!
2395 (0.036)
#SAKNAS!
2395 (0.036)
2395 (0.036)
#SAKNAS!
2395 (0.036)
#SAKNAS!
#SAKNAS!
#SAKNAS!
#SAKNAS!
#SAKNAS!
2395 (0.036)
#SAKNAS!
#SAKNAS!
#SAKNAS!
2395 (0.036)
#SAKNAS!
#SAKNAS!
#SAKNAS!
#SAKNAS!
2395 (0.036)
#SAKNAS!

#SAKNAS!
2395 (0.036)
#SAKNAS!
#SAKNAS!
#SAKNAS!
#SAKNAS!
#SAKNAS!
#SAKNAS!
#SAKNAS!
2395 (0.036)
#SAKNAS!
2395 (0.036)
2395 (0.036)
#SAKNAS!
#SAKNAS!
#SAKNAS!
#SAKNAS!
#SAKNAS!
#SAKNAS!
#SAKNAS!
#SAKNAS!
2395 (0.036)
#SAKNAS!
#SAKNAS!
#SAKNAS!
#SAKNAS!
2395 (0.036)
#SAKNAS!
#SAKNAS!
#SAKNAS!
2395 (0.036)
2395 (0.036)
#SAKNAS!
#SAKNAS!
#SAKNAS!
#SAKNAS!
#SAKNAS!
2395 (0.036)
#SAKNAS!
#SAKNAS!
#SAKNAS!
#SAKNAS!
#SAKNAS!

#SAKNAS!
#SAKNAS!
#SAKNAS!
#SAKNAS!
#SAKNAS!
#SAKNAS!
#SAKNAS!
#SAKNAS!
#SAKNAS!
#SAKNAS!
1573 (0.071)
2395 (0.036)
1573 (0.071)
2395 (0.036)
2395 (0.036)
2395 (0.036)
2395 (0.036)
#SAKNAS!
#SAKNAS!
#SAKNAS!
#SAKNAS!
#SAKNAS!
2395 (0.036)
2395 (0.036)
#SAKNAS!
2395 (0.036)
#SAKNAS!
#SAKNAS!
#SAKNAS!
#SAKNAS!
2395 (0.036)
1573 (0.071)
#SAKNAS!
#SAKNAS!
#SAKNAS!
2395 (0.036)
2395 (0.036)
#SAKNAS!
#SAKNAS!
#SAKNAS!
#SAKNAS!
#SAKNAS!
#SAKNAS!

#SAKNAS!
1573 (0.071)
#SAKNAS!
#SAKNAS!
#SAKNAS!
#SAKNAS!
#SAKNAS!
#SAKNAS!
#SAKNAS!
#SAKNAS!
#SAKNAS!
2395 (0.036)
#SAKNAS!
#SAKNAS!
#SAKNAS!
#SAKNAS!
#SAKNAS!
#SAKNAS!
#SAKNAS!
2395 (0.036)
#SAKNAS!
#SAKNAS!
#SAKNAS!
#SAKNAS!
#SAKNAS!
#SAKNAS!
#SAKNAS!
#SAKNAS!
#SAKNAS!
#SAKNAS!
#SAKNAS!
#SAKNAS!
#SAKNAS!
#SAKNAS!
#SAKNAS!
#SAKNAS!
#SAKNAS!
#SAKNAS!
#SAKNAS!
2395 (0.036)
#SAKNAS!
#SAKNAS!
#SAKNAS!

#SAKNAS!
#SAKNAS!
#SAKNAS!
#SAKNAS!
2395 (0.036)
2395 (0.036)
2395 (0.036)
2395 (0.036)
#SAKNAS!
#SAKNAS!
#SAKNAS!
#SAKNAS!
#SAKNAS!
#SAKNAS!
#SAKNAS!
#SAKNAS!
#SAKNAS!
#SAKNAS!
#SAKNAS!
2395 (0.036)
#SAKNAS!
#SAKNAS!
2395 (0.036)
#SAKNAS!
#SAKNAS!
#SAKNAS!
#SAKNAS!
#SAKNAS!
2395 (0.036)
#SAKNAS!
#SAKNAS!
#SAKNAS!
#SAKNAS!
#SAKNAS!
#SAKNAS!
2395 (0.036)
2395 (0.036)
2395 (0.036)
1164 (0.11)
2395 (0.036)
#SAKNAS!
#SAKNAS!
#SAKNAS!

#SAKNAS!
#SAKNAS!
2395 (0.036)
2395 (0.036)
2395 (0.036)
#SAKNAS!
#SAKNAS!
2395 (0.036)
2395 (0.036)
#SAKNAS!
2395 (0.036)
2395 (0.036)
#SAKNAS!
#SAKNAS!
#SAKNAS!
#SAKNAS!
#SAKNAS!
#SAKNAS!
#SAKNAS!
2395 (0.036)
1573 (0.071)
#SAKNAS!
#SAKNAS!
#SAKNAS!
#SAKNAS!
#SAKNAS!
2395 (0.036)
1573 (0.071)
2395 (0.036)
2395 (0.036)
#SAKNAS!
#SAKNAS!
#SAKNAS!
#SAKNAS!
#SAKNAS!
#SAKNAS!
#SAKNAS!
#SAKNAS!
2395 (0.036)
2395 (0.036)
2395 (0.036)
#SAKNAS!
773 (0.18)

2395 (0.036)
2395 (0.036)
2395 (0.036)
#SAKNAS!
#SAKNAS!
#SAKNAS!
2395 (0.036)
#SAKNAS!
2395 (0.036)
#SAKNAS!
#SAKNAS!
#SAKNAS!
#SAKNAS!
#SAKNAS!
#SAKNAS!
#SAKNAS!
2395 (0.036)
#SAKNAS!
2395 (0.036)
#SAKNAS!
#SAKNAS!
#SAKNAS!
#SAKNAS!
#SAKNAS!
#SAKNAS!
1573 (0.071)
#SAKNAS!
#SAKNAS!
#SAKNAS!
#SAKNAS!
#SAKNAS!
#SAKNAS!
#SAKNAS!
#SAKNAS!
2395 (0.036)
#SAKNAS!
#SAKNAS!
#SAKNAS!
#SAKNAS!
#SAKNAS!
#SAKNAS!
#SAKNAS!
#SAKNAS!

#SAKNAS!
#SAKNAS!
#SAKNAS!
#SAKNAS!
#SAKNAS!
2395 (0.036)
#SAKNAS!
#SAKNAS!
2395 (0.036)
#SAKNAS!
#SAKNAS!
#SAKNAS!
#SAKNAS!
#SAKNAS!
#SAKNAS!
2395 (0.036)
2395 (0.036)
#SAKNAS!
#SAKNAS!
#SAKNAS!
#SAKNAS!
#SAKNAS!
2395 (0.036)
#SAKNAS!
#SAKNAS!
#SAKNAS!
#SAKNAS!
2395 (0.036)
#SAKNAS!
#SAKNAS!
#SAKNAS!
#SAKNAS!
#SAKNAS!
2395 (0.036)
#SAKNAS!
#SAKNAS!
#SAKNAS!
#SAKNAS!
#SAKNAS!
#SAKNAS!
2395 (0.036)
#SAKNAS!
2395 (0.036)

#SAKNAS!
#SAKNAS!
#SAKNAS!
2395 (0.036)
2395 (0.036)
#SAKNAS!
2395 (0.036)
#SAKNAS!
#SAKNAS!
#SAKNAS!
#SAKNAS!
#SAKNAS!
2395 (0.036)
#SAKNAS!
#SAKNAS!
2395 (0.036)
2395 (0.036)
2395 (0.036)
2395 (0.036)
2395 (0.036)
#SAKNAS!
2395 (0.036)
#SAKNAS!
#SAKNAS!
2395 (0.036)
#SAKNAS!
#SAKNAS!
2395 (0.036)
936 (0.14)
2395 (0.036)
2395 (0.036)
2395 (0.036)
#SAKNAS!
#SAKNAS!
2395 (0.036)
#SAKNAS!
#SAKNAS!
#SAKNAS!
2395 (0.036)
#SAKNAS!
#SAKNAS!
#SAKNAS!
#SAKNAS!

2395 (0.036)
#SAKNAS!
#SAKNAS!
#SAKNAS!
#SAKNAS!
2395 (0.036)
1573 (0.071)
2395 (0.036)
#SAKNAS!
#SAKNAS!
#SAKNAS!
#SAKNAS!
#SAKNAS!
#SAKNAS!
#SAKNAS!
#SAKNAS!
#SAKNAS!
#SAKNAS!
#SAKNAS!
2395 (0.036)
#SAKNAS!
#SAKNAS!
#SAKNAS!
#SAKNAS!
#SAKNAS!
2395 (0.036)
#SAKNAS!
#SAKNAS!
#SAKNAS!
#SAKNAS!
#SAKNAS!
#SAKNAS!
#SAKNAS!
2395 (0.036)
2395 (0.036)
#SAKNAS!
#SAKNAS!
#SAKNAS!
#SAKNAS!
#SAKNAS!
#SAKNAS!
2395 (0.036)
#SAKNAS!

#SAKNAS!
#SAKNAS!
#SAKNAS!
#SAKNAS!
2395 (0.036)
#SAKNAS!
#SAKNAS!
#SAKNAS!
2395 (0.036)
#SAKNAS!
#SAKNAS!
#SAKNAS!
#SAKNAS!
2395 (0.036)
2395 (0.036)
#SAKNAS!
#SAKNAS!
2395 (0.036)
1164 (0.11)
#SAKNAS!
#SAKNAS!
#SAKNAS!
#SAKNAS!
#SAKNAS!
#SAKNAS!
#SAKNAS!
#SAKNAS!
2395 (0.036)
2395 (0.036)
#SAKNAS!
2395 (0.036)
#SAKNAS!
2395 (0.036)
341 (0.43)
#SAKNAS!
#SAKNAS!
#SAKNAS!
1164 (0.11)
#SAKNAS!
#SAKNAS!
#SAKNAS!
2395 (0.036)
#SAKNAS!

#SAKNAS!
1164 (0.11)
#SAKNAS!
2395 (0.036)
2395 (0.036)
1573 (0.071)
#SAKNAS!
#SAKNAS!
2395 (0.036)
#SAKNAS!
#SAKNAS!
#SAKNAS!
#SAKNAS!
#SAKNAS!
#SAKNAS!
#SAKNAS!
#SAKNAS!
#SAKNAS!
#SAKNAS!
#SAKNAS!
2395 (0.036)
#SAKNAS!
#SAKNAS!
#SAKNAS!
2395 (0.036)
#SAKNAS!
#SAKNAS!
#SAKNAS!
#SAKNAS!
#SAKNAS!
2395 (0.036)
#SAKNAS!
2395 (0.036)
#SAKNAS!
#SAKNAS!
#SAKNAS!
#SAKNAS!
1573 (0.071)
#SAKNAS!
#SAKNAS!
#SAKNAS!
#SAKNAS!
#SAKNAS!

#SAKNAS!
1573 (0.071)
#SAKNAS!
#SAKNAS!
#SAKNAS!
#SAKNAS!
#SAKNAS!
#SAKNAS!
#SAKNAS!
#SAKNAS!
1573 (0.071)
#SAKNAS!
#SAKNAS!
2395 (0.036)
#SAKNAS!
#SAKNAS!
#SAKNAS!
2395 (0.036)
#SAKNAS!
#SAKNAS!
2395 (0.036)
#SAKNAS!
#SAKNAS!
2395 (0.036)
2395 (0.036)
2395 (0.036)
#SAKNAS!
#SAKNAS!
#SAKNAS!
#SAKNAS!
#SAKNAS!
#SAKNAS!
2395 (0.036)
2395 (0.036)
#SAKNAS!
#SAKNAS!
#SAKNAS!
#SAKNAS!
2395 (0.036)
2395 (0.036)
561 (0.25)
#SAKNAS!
#SAKNAS!

2395 (0.036)
#SAKNAS!
2395 (0.036)
#SAKNAS!
2395 (0.036)
2395 (0.036)
#SAKNAS!
#SAKNAS!
#SAKNAS!
2395 (0.036)
2395 (0.036)
2395 (0.036)
#SAKNAS!
#SAKNAS!
1573 (0.071)
2395 (0.036)
#SAKNAS!
#SAKNAS!
2395 (0.036)
936 (0.14)
#SAKNAS!
#SAKNAS!
1573 (0.071)
#SAKNAS!
#SAKNAS!
#SAKNAS!
2395 (0.036)
#SAKNAS!
#SAKNAS!
#SAKNAS!
#SAKNAS!
#SAKNAS!
2395 (0.036)
#SAKNAS!
#SAKNAS!
#SAKNAS!
2395 (0.036)
#SAKNAS!
2395 (0.036)
2395 (0.036)
#SAKNAS!
#SAKNAS!
2395 (0.036)

2395 (0.036)
#SAKNAS!
#SAKNAS!
#SAKNAS!
#SAKNAS!
2395 (0.036)
#SAKNAS!
2395 (0.036)
2395 (0.036)
2395 (0.036)
#SAKNAS!
2395 (0.036)
#SAKNAS!
#SAKNAS!
2395 (0.036)
#SAKNAS!
#SAKNAS!
#SAKNAS!
#SAKNAS!
#SAKNAS!
#SAKNAS!
#SAKNAS!
#SAKNAS!
2395 (0.036)
#SAKNAS!
#SAKNAS!
#SAKNAS!
#SAKNAS!
2395 (0.036)
#SAKNAS!
#SAKNAS!
#SAKNAS!
2395 (0.036)
#SAKNAS!
2395 (0.036)
#SAKNAS!
#SAKNAS!
#SAKNAS!
#SAKNAS!
#SAKNAS!
#SAKNAS!
2395 (0.036)
#SAKNAS!

2395 (0.036)
2395 (0.036)
#SAKNAS!
#SAKNAS!
2395 (0.036)
#SAKNAS!
#SAKNAS!
2395 (0.036)
#SAKNAS!
#SAKNAS!
#SAKNAS!
#SAKNAS!
#SAKNAS!
1164 (0.11)
#SAKNAS!
#SAKNAS!
#SAKNAS!
2395 (0.036)
2395 (0.036)
#SAKNAS!
#SAKNAS!
2395 (0.036)
#SAKNAS!
#SAKNAS!
#SAKNAS!
#SAKNAS!
2395 (0.036)
#SAKNAS!
#SAKNAS!
#SAKNAS!
#SAKNAS!
2395 (0.036)
#SAKNAS!
#SAKNAS!
#SAKNAS!
2395 (0.036)
#SAKNAS!
#SAKNAS!
2395 (0.036)
#SAKNAS!
#SAKNAS!
1573 (0.071)
#SAKNAS!

#SAKNAS!
2395 (0.036)
2395 (0.036)
#SAKNAS!
#SAKNAS!
#SAKNAS!
#SAKNAS!
2395 (0.036)
2395 (0.036)
#SAKNAS!
#SAKNAS!
#SAKNAS!
#SAKNAS!
#SAKNAS!
#SAKNAS!
2395 (0.036)
2395 (0.036)
2395 (0.036)
#SAKNAS!
#SAKNAS!
#SAKNAS!
#SAKNAS!
#SAKNAS!
2395 (0.036)
2395 (0.036)
1164 (0.11)
#SAKNAS!
#SAKNAS!
#SAKNAS!
#SAKNAS!
#SAKNAS!
#SAKNAS!
#SAKNAS!
#SAKNAS!
#SAKNAS!
#SAKNAS!
#SAKNAS!
2395 (0.036)
#SAKNAS!
#SAKNAS!
#SAKNAS!
2395 (0.036)
2395 (0.036)

#SAKNAS!
2395 (0.036)
#SAKNAS!
#SAKNAS!
#SAKNAS!
#SAKNAS!
#SAKNAS!
#SAKNAS!
#SAKNAS!
#SAKNAS!
#SAKNAS!
#SAKNAS!
#SAKNAS!
2395 (0.036)
#SAKNAS!
#SAKNAS!
2395 (0.036)
#SAKNAS!
#SAKNAS!
#SAKNAS!
2395 (0.036)
2395 (0.036)
2395 (0.036)
2395 (0.036)
#SAKNAS!
2395 (0.036)
#SAKNAS!
#SAKNAS!
2395 (0.036)
2395 (0.036)
2395 (0.036)
2395 (0.036)
2395 (0.036)
2395 (0.036)
#SAKNAS!
#SAKNAS!
2395 (0.036)
#SAKNAS!
#SAKNAS!
2395 (0.036)
#SAKNAS!
2395 (0.036)
#SAKNAS!

#SAKNAS!
#SAKNAS!
#SAKNAS!
2395 (0.036)
#SAKNAS!
#SAKNAS!
#SAKNAS!
#SAKNAS!
#SAKNAS!
#SAKNAS!
#SAKNAS!
#SAKNAS!
#SAKNAS!
#SAKNAS!
#SAKNAS!
#SAKNAS!
#SAKNAS!
2395 (0.036)
#SAKNAS!
2395 (0.036)
#SAKNAS!
#SAKNAS!
#SAKNAS!
#SAKNAS!
#SAKNAS!
#SAKNAS!
#SAKNAS!
#SAKNAS!
#SAKNAS!
#SAKNAS!
#SAKNAS!
#SAKNAS!
#SAKNAS!
#SAKNAS!
#SAKNAS!
#SAKNAS!
#SAKNAS!
#SAKNAS!
#SAKNAS!
#SAKNAS!
#SAKNAS!
#SAKNAS!
#SAKNAS!

#SAKNAS!
#SAKNAS!
#SAKNAS!
#SAKNAS!
#SAKNAS!
#SAKNAS!
#SAKNAS!
#SAKNAS!
#SAKNAS!
#SAKNAS!
#SAKNAS!
#SAKNAS!
2395 (0.036)
2395 (0.036)
#SAKNAS!
#SAKNAS!
2395 (0.036)
#SAKNAS!
#SAKNAS!
#SAKNAS!
#SAKNAS!
1573 (0.071)
#SAKNAS!
#SAKNAS!
#SAKNAS!
#SAKNAS!
#SAKNAS!
1164 (0.11)
#SAKNAS!
#SAKNAS!
2395 (0.036)
#SAKNAS!
#SAKNAS!
2395 (0.036)
#SAKNAS!
2395 (0.036)
#SAKNAS!
#SAKNAS!
#SAKNAS!
2395 (0.036)
2395 (0.036)
2395 (0.036)
#SAKNAS!

#SAKNAS!
#SAKNAS!
#SAKNAS!
#SAKNAS!
#SAKNAS!
#SAKNAS!
2395 (0.036)
#SAKNAS!
#SAKNAS!
2395 (0.036)
2395 (0.036)
2395 (0.036)
1573 (0.071)
#SAKNAS!
#SAKNAS!
2395 (0.036)
2395 (0.036)
#SAKNAS!
#SAKNAS!
#SAKNAS!
#SAKNAS!
#SAKNAS!
2395 (0.036)
#SAKNAS!
#SAKNAS!
#SAKNAS!
#SAKNAS!
#SAKNAS!
#SAKNAS!
#SAKNAS!
#SAKNAS!
#SAKNAS!
2395 (0.036)
2395 (0.036)
2395 (0.036)
#SAKNAS!
2395 (0.036)
#SAKNAS!
#SAKNAS!
#SAKNAS!
#SAKNAS!
#SAKNAS!
2395 (0.036)

#SAKNAS!
#SAKNAS!
#SAKNAS!
2395 (0.036)
#SAKNAS!
#SAKNAS!
#SAKNAS!
#SAKNAS!
#SAKNAS!
#SAKNAS!
2395 (0.036)
#SAKNAS!
#SAKNAS!
2395 (0.036)
#SAKNAS!
#SAKNAS!
#SAKNAS!
#SAKNAS!
2395 (0.036)
#SAKNAS!
648 (0.21)
#SAKNAS!
#SAKNAS!
#SAKNAS!
#SAKNAS!
2395 (0.036)
936 (0.14)
#SAKNAS!
#SAKNAS!
2395 (0.036)
2395 (0.036)
2395 (0.036)
#SAKNAS!
#SAKNAS!
2395 (0.036)
#SAKNAS!
#SAKNAS!
#SAKNAS!
#SAKNAS!
#SAKNAS!
2395 (0.036)
#SAKNAS!
2395 (0.036)

2395 (0.036)
#SAKNAS!
2395 (0.036)
#SAKNAS!
#SAKNAS!
#SAKNAS!
#SAKNAS!
#SAKNAS!
#SAKNAS!
#SAKNAS!
#SAKNAS!
2395 (0.036)
#SAKNAS!
#SAKNAS!
#SAKNAS!
2395 (0.036)
#SAKNAS!
#SAKNAS!
#SAKNAS!
#SAKNAS!
2395 (0.036)
#SAKNAS!
#SAKNAS!
#SAKNAS!
2395 (0.036)
2395 (0.036)
#SAKNAS!
#SAKNAS!
2395 (0.036)
#SAKNAS!
#SAKNAS!
#SAKNAS!
#SAKNAS!
#SAKNAS!
#SAKNAS!
#SAKNAS!
#SAKNAS!
#SAKNAS!
#SAKNAS!
#SAKNAS!
1573 (0.071)
2395 (0.036)
#SAKNAS!

#SAKNAS!
2395 (0.036)
#SAKNAS!
#SAKNAS!
2395 (0.036)
2395 (0.036)
1164 (0.11)
#SAKNAS!
#SAKNAS!
1164 (0.11)
648 (0.21)
#SAKNAS!
#SAKNAS!
#SAKNAS!
2395 (0.036)
#SAKNAS!
#SAKNAS!
#SAKNAS!
#SAKNAS!
#SAKNAS!
2395 (0.036)
#SAKNAS!
1573 (0.071)
#SAKNAS!
2395 (0.036)
#SAKNAS!
#SAKNAS!
2395 (0.036)
370 (0.39)
2395 (0.036)
2395 (0.036)
2395 (0.036)
209 (0.67)
#SAKNAS!
2395 (0.036)
#SAKNAS!
2395 (0.036)
#SAKNAS!
#SAKNAS!
#SAKNAS!
#SAKNAS!
#SAKNAS!
#SAKNAS!

2395 (0.036)
#SAKNAS!
#SAKNAS!
2395 (0.036)
#SAKNAS!
2395 (0.036)
2395 (0.036)
#SAKNAS!
#SAKNAS!
2395 (0.036)
2395 (0.036)
#SAKNAS!
2395 (0.036)
2395 (0.036)
1573 (0.071)
#SAKNAS!
#SAKNAS!
2395 (0.036)
#SAKNAS!
2395 (0.036)
936 (0.14)
1573 (0.071)
2395 (0.036)
2395 (0.036)
#SAKNAS!
2395 (0.036)
#SAKNAS!
2395 (0.036)
#SAKNAS!
#SAKNAS!
#SAKNAS!
#SAKNAS!
#SAKNAS!
#SAKNAS!
#SAKNAS!
#SAKNAS!
#SAKNAS!
#SAKNAS!
#SAKNAS!
#SAKNAS!
#SAKNAS!
#SAKNAS!
#SAKNAS!

#SAKNAS!
#SAKNAS!
#SAKNAS!
#SAKNAS!
#SAKNAS!
#SAKNAS!
#SAKNAS!
#SAKNAS!
#SAKNAS!
#SAKNAS!
#SAKNAS!
#SAKNAS!
#SAKNAS!
#SAKNAS!
2395 (0.036)
2395 (0.036)
#SAKNAS!
1573 (0.071)
2395 (0.036)
#SAKNAS!
2395 (0.036)
#SAKNAS!
#SAKNAS!
#SAKNAS!
2395 (0.036)
2395 (0.036)
#SAKNAS!
#SAKNAS!
#SAKNAS!
#SAKNAS!
2395 (0.036)
#SAKNAS!
936 (0.14)
1164 (0.11)
2395 (0.036)
#SAKNAS!
#SAKNAS!
#SAKNAS!
2395 (0.036)
#SAKNAS!
#SAKNAS!
2395 (0.036)
#SAKNAS!

2395 (0.036)
1573 (0.071)
#SAKNAS!
2395 (0.036)
#SAKNAS!
#SAKNAS!
#SAKNAS!
#SAKNAS!
2395 (0.036)
#SAKNAS!
#SAKNAS!
#SAKNAS!
#SAKNAS!
#SAKNAS!
#SAKNAS!
#SAKNAS!
#SAKNAS!
#SAKNAS!
#SAKNAS!
#SAKNAS!
#SAKNAS!
#SAKNAS!
#SAKNAS!
936 (0.14)
#SAKNAS!
#SAKNAS!
2395 (0.036)
#SAKNAS!
#SAKNAS!
2395 (0.036)
#SAKNAS!
2395 (0.036)
2395 (0.036)
1164 (0.11)
2395 (0.036)
#SAKNAS!
2395 (0.036)
#SAKNAS!
#SAKNAS!
#SAKNAS!
2395 (0.036)
2395 (0.036)
#SAKNAS!

#SAKNAS!
#SAKNAS!
2395 (0.036)
#SAKNAS!
#SAKNAS!
#SAKNAS!
2395 (0.036)
1164 (0.11)
#SAKNAS!
2395 (0.036)
#SAKNAS!
#SAKNAS!
#SAKNAS!
#SAKNAS!
#SAKNAS!
#SAKNAS!
#SAKNAS!
#SAKNAS!
#SAKNAS!
#SAKNAS!
#SAKNAS!
#SAKNAS!
#SAKNAS!
2395 (0.036)
2395 (0.036)
#SAKNAS!
#SAKNAS!
#SAKNAS!
#SAKNAS!
#SAKNAS!
#SAKNAS!
#SAKNAS!
#SAKNAS!
2395 (0.036)
#SAKNAS!
#SAKNAS!
#SAKNAS!
#SAKNAS!
#SAKNAS!
2395 (0.036)
2395 (0.036)
2395 (0.036)
#SAKNAS!

#SAKNAS!
446 (0.32)
#SAKNAS!
#SAKNAS!
#SAKNAS!
2395 (0.036)
#SAKNAS!
#SAKNAS!
#SAKNAS!
#SAKNAS!
#SAKNAS!
#SAKNAS!
#SAKNAS!
#SAKNAS!
#SAKNAS!
2395 (0.036)
#SAKNAS!
#SAKNAS!
2395 (0.036)
#SAKNAS!
#SAKNAS!
#SAKNAS!
#SAKNAS!
#SAKNAS!
#SAKNAS!
#SAKNAS!
2395 (0.036)
1573 (0.071)
#SAKNAS!
#SAKNAS!
2395 (0.036)
#SAKNAS!
2395 (0.036)
#SAKNAS!
#SAKNAS!
#SAKNAS!
#SAKNAS!
#SAKNAS!
2395 (0.036)
2395 (0.036)
#SAKNAS!
2395 (0.036)
#SAKNAS!

#SAKNAS!
2395 (0.036)
#SAKNAS!
2395 (0.036)
#SAKNAS!
#SAKNAS!
#SAKNAS!
#SAKNAS!
#SAKNAS!
2395 (0.036)
2395 (0.036)
#SAKNAS!
#SAKNAS!
#SAKNAS!
#SAKNAS!
2395 (0.036)
2395 (0.036)
#SAKNAS!
#SAKNAS!
#SAKNAS!
#SAKNAS!
2395 (0.036)
#SAKNAS!
#SAKNAS!
#SAKNAS!
#SAKNAS!
#SAKNAS!
#SAKNAS!
#SAKNAS!
2395 (0.036)
2395 (0.036)
#SAKNAS!
#SAKNAS!
2395 (0.036)
2395 (0.036)
#SAKNAS!
2395 (0.036)
#SAKNAS!
#SAKNAS!
#SAKNAS!
#SAKNAS!
#SAKNAS!
2395 (0.036)

#SAKNAS!
#SAKNAS!
2395 (0.036)
#SAKNAS!
#SAKNAS!
2395 (0.036)
#SAKNAS!
#SAKNAS!
2395 (0.036)
2395 (0.036)
#SAKNAS!
#SAKNAS!
#SAKNAS!
#SAKNAS!
#SAKNAS!
#SAKNAS!
#SAKNAS!
#SAKNAS!
#SAKNAS!
#SAKNAS!
#SAKNAS!
#SAKNAS!
#SAKNAS!
1573 (0.071)
#SAKNAS!
2395 (0.036)
#SAKNAS!
#SAKNAS!
#SAKNAS!
2395 (0.036)
#SAKNAS!
#SAKNAS!
#SAKNAS!
#SAKNAS!
2395 (0.036)
1164 (0.11)
#SAKNAS!
#SAKNAS!
2395 (0.036)
#SAKNAS!
#SAKNAS!
#SAKNAS!
#SAKNAS!

#SAKNAS!
#SAKNAS!
2395 (0.036)
2395 (0.036)
#SAKNAS!
#SAKNAS!
2395 (0.036)
2395 (0.036)
#SAKNAS!
#SAKNAS!
#SAKNAS!
#SAKNAS!
#SAKNAS!
#SAKNAS!
#SAKNAS!
2395 (0.036)
#SAKNAS!
2395 (0.036)
#SAKNAS!
#SAKNAS!
#SAKNAS!
#SAKNAS!
#SAKNAS!
#SAKNAS!
#SAKNAS!
#SAKNAS!
#SAKNAS!
#SAKNAS!
#SAKNAS!
#SAKNAS!
2395 (0.036)
#SAKNAS!
#SAKNAS!
#SAKNAS!
#SAKNAS!
2395 (0.036)
#SAKNAS!
#SAKNAS!
#SAKNAS!
#SAKNAS!
#SAKNAS!
#SAKNAS!
#SAKNAS!

#SAKNAS!
#SAKNAS!
#SAKNAS!
2395 (0.036)
#SAKNAS!
#SAKNAS!
#SAKNAS!
#SAKNAS!
936 (0.14)
2395 (0.036)
2395 (0.036)
#SAKNAS!
2395 (0.036)
#SAKNAS!
#SAKNAS!
#SAKNAS!
#SAKNAS!
#SAKNAS!
#SAKNAS!
#SAKNAS!
2395 (0.036)
1573 (0.071)
2395 (0.036)
1573 (0.071)
#SAKNAS!
#SAKNAS!
#SAKNAS!
2395 (0.036)
2395 (0.036)
#SAKNAS!
#SAKNAS!
#SAKNAS!
#SAKNAS!
2395 (0.036)
2395 (0.036)
#SAKNAS!
#SAKNAS!
#SAKNAS!
2395 (0.036)
2395 (0.036)
#SAKNAS!
#SAKNAS!
2395 (0.036)

#SAKNAS!
#SAKNAS!
2395 (0.036)
1573 (0.071)
#SAKNAS!
#SAKNAS!
#SAKNAS!
2395 (0.036)
#SAKNAS!
#SAKNAS!
#SAKNAS!
#SAKNAS!
#SAKNAS!
2395 (0.036)
2395 (0.036)
#SAKNAS!
#SAKNAS!
2395 (0.036)
#SAKNAS!
#SAKNAS!
#SAKNAS!
#SAKNAS!
#SAKNAS!
#SAKNAS!
#SAKNAS!
1164 (0.11)
2395 (0.036)
#SAKNAS!
#SAKNAS!
2395 (0.036)
2395 (0.036)
1573 (0.071)
#SAKNAS!
#SAKNAS!
#SAKNAS!
#SAKNAS!
#SAKNAS!
#SAKNAS!
#SAKNAS!
#SAKNAS!
#SAKNAS!
#SAKNAS!
#SAKNAS!

#SAKNAS!
#SAKNAS!
#SAKNAS!
#SAKNAS!
2395 (0.036)
2395 (0.036)
#SAKNAS!
#SAKNAS!
#SAKNAS!
2395 (0.036)
#SAKNAS!
#SAKNAS!
#SAKNAS!
#SAKNAS!
#SAKNAS!
#SAKNAS!
#SAKNAS!
#SAKNAS!
#SAKNAS!
2395 (0.036)
#SAKNAS!
#SAKNAS!
#SAKNAS!
2395 (0.036)
1573 (0.071)
#SAKNAS!
#SAKNAS!
#SAKNAS!
#SAKNAS!
#SAKNAS!
#SAKNAS!
#SAKNAS!
#SAKNAS!
#SAKNAS!
#SAKNAS!
#SAKNAS!
#SAKNAS!
#SAKNAS!
1573 (0.071)
2395 (0.036)
#SAKNAS!
2395 (0.036)
2395 (0.036)

2395 (0.036)
2395 (0.036)
#SAKNAS!
#SAKNAS!
#SAKNAS!
2395 (0.036)
2395 (0.036)
#SAKNAS!
#SAKNAS!
#SAKNAS!
#SAKNAS!
#SAKNAS!
#SAKNAS!
#SAKNAS!
#SAKNAS!
#SAKNAS!
#SAKNAS!
#SAKNAS!
#SAKNAS!
2395 (0.036)
#SAKNAS!
#SAKNAS!
#SAKNAS!
2395 (0.036)
2395 (0.036)
2395 (0.036)
#SAKNAS!
2395 (0.036)
2395 (0.036)
#SAKNAS!
#SAKNAS!
2395 (0.036)
2395 (0.036)
#SAKNAS!
#SAKNAS!
#SAKNAS!
#SAKNAS!
#SAKNAS!
1573 (0.071)
#SAKNAS!
#SAKNAS!
#SAKNAS!
2395 (0.036)

1164 (0.11)
#SAKNAS!
#SAKNAS!
#SAKNAS!
#SAKNAS!
2395 (0.036)
1573 (0.071)
#SAKNAS!
#SAKNAS!
2395 (0.036)
#SAKNAS!
#SAKNAS!
#SAKNAS!
2395 (0.036)
#SAKNAS!
#SAKNAS!
#SAKNAS!
2395 (0.036)
2395 (0.036)
2395 (0.036)
#SAKNAS!
#SAKNAS!
#SAKNAS!
2395 (0.036)
1573 (0.071)
2395 (0.036)
2395 (0.036)
2395 (0.036)
#SAKNAS!
#SAKNAS!
#SAKNAS!
#SAKNAS!
2395 (0.036)
#SAKNAS!
#SAKNAS!
2395 (0.036)
#SAKNAS!
#SAKNAS!
2395 (0.036)
#SAKNAS!
#SAKNAS!
#SAKNAS!
#SAKNAS!

#SAKNAS!
#SAKNAS!
#SAKNAS!
2395 (0.036)
#SAKNAS!
#SAKNAS!
#SAKNAS!
#SAKNAS!
773 (0.18)
2395 (0.036)
#SAKNAS!
#SAKNAS!
2395 (0.036)
2395 (0.036)
2395 (0.036)
#SAKNAS!
#SAKNAS!
#SAKNAS!
#SAKNAS!
#SAKNAS!
#SAKNAS!
#SAKNAS!
2395 (0.036)
2395 (0.036)
#SAKNAS!
#SAKNAS!
1573 (0.071)
2395 (0.036)
#SAKNAS!
#SAKNAS!
2395 (0.036)
#SAKNAS!
#SAKNAS!
2395 (0.036)
#SAKNAS!
#SAKNAS!
2395 (0.036)
#SAKNAS!
#SAKNAS!
#SAKNAS!
#SAKNAS!
#SAKNAS!
#SAKNAS!

#SAKNAS!
#SAKNAS!
2395 (0.036)
#SAKNAS!
#SAKNAS!
#SAKNAS!
#SAKNAS!
#SAKNAS!
#SAKNAS!
#SAKNAS!
2395 (0.036)
#SAKNAS!
#SAKNAS!
#SAKNAS!
#SAKNAS!
2395 (0.036)
#SAKNAS!
#SAKNAS!
#SAKNAS!
#SAKNAS!
#SAKNAS!
2395 (0.036)
#SAKNAS!
#SAKNAS!
#SAKNAS!
#SAKNAS!
#SAKNAS!
#SAKNAS!
#SAKNAS!
2395 (0.036)
#SAKNAS!
2395 (0.036)
#SAKNAS!
#SAKNAS!
#SAKNAS!
#SAKNAS!
#SAKNAS!
2395 (0.036)
#SAKNAS!
2395 (0.036)
#SAKNAS!
#SAKNAS!
#SAKNAS!

#SAKNAS!
#SAKNAS!
#SAKNAS!
#SAKNAS!
#SAKNAS!
#SAKNAS!
#SAKNAS!
#SAKNAS!
#SAKNAS!
#SAKNAS!
2395 (0.036)
#SAKNAS!
2395 (0.036)
2395 (0.036)
2395 (0.036)
#SAKNAS!
#SAKNAS!
#SAKNAS!
2395 (0.036)
#SAKNAS!
#SAKNAS!
#SAKNAS!
#SAKNAS!
2395 (0.036)
2395 (0.036)
2395 (0.036)
#SAKNAS!
#SAKNAS!
#SAKNAS!
#SAKNAS!
#SAKNAS!
2395 (0.036)
2395 (0.036)
2395 (0.036)
#SAKNAS!
#SAKNAS!
1573 (0.071)
2395 (0.036)
#SAKNAS!
2395 (0.036)
#SAKNAS!
#SAKNAS!
#SAKNAS!

#SAKNAS!
#SAKNAS!
#SAKNAS!
2395 (0.036)
2395 (0.036)
#SAKNAS!
#SAKNAS!
2395 (0.036)
2395 (0.036)
2395 (0.036)
#SAKNAS!
#SAKNAS!
2395 (0.036)
#SAKNAS!
#SAKNAS!
#SAKNAS!
#SAKNAS!
2395 (0.036)
#SAKNAS!
#SAKNAS!
2395 (0.036)
#SAKNAS!
2395 (0.036)
2395 (0.036)
2395 (0.036)
2395 (0.036)
#SAKNAS!
2395 (0.036)
#SAKNAS!
#SAKNAS!
#SAKNAS!
2395 (0.036)
#SAKNAS!
#SAKNAS!
#SAKNAS!
#SAKNAS!
#SAKNAS!
2395 (0.036)
#SAKNAS!
#SAKNAS!
#SAKNAS!
#SAKNAS!
#SAKNAS!

#SAKNAS!
#SAKNAS!
341 (0.43)
#SAKNAS!
#SAKNAS!
#SAKNAS!
2395 (0.036)
1164 (0.11)
2395 (0.036)
#SAKNAS!
#SAKNAS!
2395 (0.036)
2395 (0.036)
#SAKNAS!
#SAKNAS!
#SAKNAS!
#SAKNAS!
#SAKNAS!
2395 (0.036)
1573 (0.071)
#SAKNAS!
#SAKNAS!
2395 (0.036)
#SAKNAS!
#SAKNAS!
2395 (0.036)
#SAKNAS!
#SAKNAS!
2395 (0.036)
2395 (0.036)
#SAKNAS!
#SAKNAS!
#SAKNAS!
2395 (0.036)
#SAKNAS!
#SAKNAS!
2395 (0.036)
2395 (0.036)
#SAKNAS!
#SAKNAS!
#SAKNAS!
#SAKNAS!
#SAKNAS!

2395 (0.036)
1164 (0.11)
#SAKNAS!
#SAKNAS!
#SAKNAS!
#SAKNAS!
#SAKNAS!
#SAKNAS!
#SAKNAS!
#SAKNAS!
#SAKNAS!
#SAKNAS!
2395 (0.036)
#SAKNAS!
#SAKNAS!
#SAKNAS!
#SAKNAS!
#SAKNAS!
1573 (0.071)
#SAKNAS!
2395 (0.036)
#SAKNAS!
#SAKNAS!
#SAKNAS!
#SAKNAS!
#SAKNAS!
#SAKNAS!
#SAKNAS!
#SAKNAS!
#SAKNAS!
#SAKNAS!
2395 (0.036)
#SAKNAS!
#SAKNAS!
#SAKNAS!
#SAKNAS!
#SAKNAS!
#SAKNAS!
#SAKNAS!
#SAKNAS!
2395 (0.036)
#SAKNAS!
#SAKNAS!

2395 (0.036)
#SAKNAS!
#SAKNAS!
2395 (0.036)
2395 (0.036)
#SAKNAS!
2395 (0.036)
2395 (0.036)
2395 (0.036)
#SAKNAS!
#SAKNAS!
#SAKNAS!
#SAKNAS!
2395 (0.036)
#SAKNAS!
2395 (0.036)
2395 (0.036)
2395 (0.036)
#SAKNAS!
#SAKNAS!
#SAKNAS!
2395 (0.036)
2395 (0.036)
2395 (0.036)
2395 (0.036)
#SAKNAS!
#SAKNAS!
#SAKNAS!
#SAKNAS!
#SAKNAS!
#SAKNAS!
#SAKNAS!
#SAKNAS!
#SAKNAS!
2395 (0.036)
2395 (0.036)
2395 (0.036)
2395 (0.036)
2395 (0.036)
#SAKNAS!
2395 (0.036)
#SAKNAS!
2395 (0.036)

1573 (0.071)
2395 (0.036)
2395 (0.036)
2395 (0.036)
#SAKNAS!
#SAKNAS!
#SAKNAS!
#SAKNAS!
#SAKNAS!
#SAKNAS!
#SAKNAS!
#SAKNAS!
#SAKNAS!
#SAKNAS!
2395 (0.036)
2395 (0.036)
2395 (0.036)
2395 (0.036)
#SAKNAS!
#SAKNAS!
#SAKNAS!
#SAKNAS!
#SAKNAS!
#SAKNAS!
1573 (0.071)
#SAKNAS!
#SAKNAS!
#SAKNAS!
#SAKNAS!
2395 (0.036)
2395 (0.036)
#SAKNAS!
#SAKNAS!
#SAKNAS!
#SAKNAS!
#SAKNAS!
2395 (0.036)
2395 (0.036)
#SAKNAS!
2395 (0.036)
#SAKNAS!
#SAKNAS!
2395 (0.036)

2395 (0.036)
2395 (0.036)
1573 (0.071)
2395 (0.036)
#SAKNAS!
2395 (0.036)
936 (0.14)
#SAKNAS!
#SAKNAS!
936 (0.14)
#SAKNAS!
2395 (0.036)
#SAKNAS!
2395 (0.036)
341 (0.43)
1573 (0.071)
1164 (0.11)
#SAKNAS!
#SAKNAS!
2395 (0.036)
#SAKNAS!
#SAKNAS!
2395 (0.036)
#SAKNAS!
#SAKNAS!
#SAKNAS!
#SAKNAS!
#SAKNAS!
#SAKNAS!
#SAKNAS!
2395 (0.036)
#SAKNAS!
#SAKNAS!
2395 (0.036)
#SAKNAS!
2395 (0.036)
#SAKNAS!
#SAKNAS!
#SAKNAS!
#SAKNAS!
#SAKNAS!
#SAKNAS!
#SAKNAS!

#SAKNAS!
#SAKNAS!
#SAKNAS!
#SAKNAS!
#SAKNAS!
#SAKNAS!
#SAKNAS!
#SAKNAS!
#SAKNAS!
#SAKNAS!
#SAKNAS!
#SAKNAS!
#SAKNAS!
#SAKNAS!
#SAKNAS!
#SAKNAS!
#SAKNAS!
#SAKNAS!
561 (0.25)
#SAKNAS!
#SAKNAS!
#SAKNAS!
#SAKNAS!
#SAKNAS!
#SAKNAS!
2395 (0.036)
2395 (0.036)
#SAKNAS!
#SAKNAS!
#SAKNAS!
#SAKNAS!
2395 (0.036)
2395 (0.036)
#SAKNAS!
#SAKNAS!
#SAKNAS!
#SAKNAS!
#SAKNAS!
#SAKNAS!
#SAKNAS!
#SAKNAS!
#SAKNAS!
#SAKNAS!

2395 (0.036)
2395 (0.036)
319 (0.46)
2395 (0.036)
#SAKNAS!
370 (0.39)
#SAKNAS!
1164 (0.11)
2395 (0.036)
#SAKNAS!
#SAKNAS!
#SAKNAS!
#SAKNAS!
#SAKNAS!
1573 (0.071)
2395 (0.036)
#SAKNAS!
#SAKNAS!
#SAKNAS!
#SAKNAS!
#SAKNAS!
2395 (0.036)
#SAKNAS!
2395 (0.036)
#SAKNAS!
#SAKNAS!
2395 (0.036)
#SAKNAS!
#SAKNAS!
2395 (0.036)
2395 (0.036)
2395 (0.036)
2395 (0.036)
#SAKNAS!
#SAKNAS!
2395 (0.036)
2395 (0.036)
#SAKNAS!
#SAKNAS!
2395 (0.036)
#SAKNAS!
2395 (0.036)
2395 (0.036)

#SAKNAS!
#SAKNAS!
#SAKNAS!
#SAKNAS!
#SAKNAS!
#SAKNAS!
2395 (0.036)
#SAKNAS!
#SAKNAS!
#SAKNAS!
2395 (0.036)
2395 (0.036)
#SAKNAS!
#SAKNAS!
#SAKNAS!
2395 (0.036)
#SAKNAS!
#SAKNAS!
2395 (0.036)
2395 (0.036)
2395 (0.036)
2395 (0.036)
2395 (0.036)
#SAKNAS!
#SAKNAS!
2395 (0.036)
2395 (0.036)
#SAKNAS!
#SAKNAS!
1573 (0.071)
1573 (0.071)
#SAKNAS!
#SAKNAS!
#SAKNAS!
#SAKNAS!
2395 (0.036)
#SAKNAS!
#SAKNAS!
#SAKNAS!
#SAKNAS!
#SAKNAS!
2395 (0.036)
2395 (0.036)

#SAKNAS!
#SAKNAS!
#SAKNAS!
#SAKNAS!
#SAKNAS!
#SAKNAS!
2395 (0.036)
2395 (0.036)
#SAKNAS!
#SAKNAS!
2395 (0.036)
#SAKNAS!
#SAKNAS!
#SAKNAS!
#SAKNAS!
#SAKNAS!
#SAKNAS!
#SAKNAS!
#SAKNAS!
#SAKNAS!
#SAKNAS!
#SAKNAS!
#SAKNAS!
#SAKNAS!
2395 (0.036)
2395 (0.036)
2395 (0.036)
#SAKNAS!
#SAKNAS!
#SAKNAS!
#SAKNAS!
#SAKNAS!
#SAKNAS!
2395 (0.036)
#SAKNAS!
#SAKNAS!
#SAKNAS!
#SAKNAS!
#SAKNAS!
#SAKNAS!
#SAKNAS!
#SAKNAS!
#SAKNAS!

2395 (0.036)
#SAKNAS!
#SAKNAS!
#SAKNAS!
1573 (0.071)
1573 (0.071)
#SAKNAS!
2395 (0.036)
936 (0.14)
#SAKNAS!
#SAKNAS!
#SAKNAS!
#SAKNAS!
#SAKNAS!
#SAKNAS!
#SAKNAS!
2395 (0.036)
#SAKNAS!
#SAKNAS!
#SAKNAS!
#SAKNAS!
2395 (0.036)
2395 (0.036)
2395 (0.036)
#SAKNAS!
#SAKNAS!
#SAKNAS!
2395 (0.036)
2395 (0.036)
2395 (0.036)
2395 (0.036)
#SAKNAS!
#SAKNAS!
#SAKNAS!
#SAKNAS!
#SAKNAS!
#SAKNAS!
#SAKNAS!
#SAKNAS!
#SAKNAS!
#SAKNAS!
#SAKNAS!
#SAKNAS!

#SAKNAS!
#SAKNAS!
#SAKNAS!
2395 (0.036)
#SAKNAS!
#SAKNAS!
2395 (0.036)
936 (0.14)
#SAKNAS!
#SAKNAS!
#SAKNAS!
2395 (0.036)
#SAKNAS!
#SAKNAS!
#SAKNAS!
2395 (0.036)
936 (0.14)
#SAKNAS!
#SAKNAS!
#SAKNAS!
2395 (0.036)
1573 (0.071)
#SAKNAS!
#SAKNAS!
#SAKNAS!
#SAKNAS!
2395 (0.036)
#SAKNAS!
#SAKNAS!
#SAKNAS!
2395 (0.036)
773 (0.18)
#SAKNAS!
#SAKNAS!
2395 (0.036)
#SAKNAS!
2395 (0.036)
1164 (0.11)
#SAKNAS!
#SAKNAS!
2395 (0.036)
#SAKNAS!
#SAKNAS!

#SAKNAS!
#SAKNAS!
#SAKNAS!
1164 (0.11)
#SAKNAS!
#SAKNAS!
#SAKNAS!
#SAKNAS!
#SAKNAS!
#SAKNAS!
#SAKNAS!
#SAKNAS!
#SAKNAS!
2395 (0.036)
#SAKNAS!
2395 (0.036)
#SAKNAS!
#SAKNAS!
#SAKNAS!
#SAKNAS!
#SAKNAS!
#SAKNAS!
#SAKNAS!
#SAKNAS!
#SAKNAS!
#SAKNAS!
1164 (0.11)
#SAKNAS!
#SAKNAS!
#SAKNAS!
2395 (0.036)
#SAKNAS!
#SAKNAS!
#SAKNAS!
#SAKNAS!
2395 (0.036)
#SAKNAS!
#SAKNAS!
#SAKNAS!
2395 (0.036)
#SAKNAS!
2395 (0.036)
#SAKNAS!

#SAKNAS!
#SAKNAS!
#SAKNAS!
#SAKNAS!
2395 (0.036)
#SAKNAS!
2395 (0.036)
2395 (0.036)
#SAKNAS!
2395 (0.036)
#SAKNAS!
#SAKNAS!
#SAKNAS!
#SAKNAS!
#SAKNAS!
#SAKNAS!
#SAKNAS!
#SAKNAS!
#SAKNAS!
#SAKNAS!
#SAKNAS!
#SAKNAS!
2395 (0.036)
#SAKNAS!
#SAKNAS!
#SAKNAS!
#SAKNAS!
#SAKNAS!
2395 (0.036)
#SAKNAS!
2395 (0.036)
1164 (0.11)
#SAKNAS!
#SAKNAS!
#SAKNAS!
#SAKNAS!
#SAKNAS!
#SAKNAS!
#SAKNAS!
1573 (0.071)
#SAKNAS!
#SAKNAS!
#SAKNAS!

#SAKNAS!
#SAKNAS!
#SAKNAS!
#SAKNAS!
#SAKNAS!
#SAKNAS!
#SAKNAS!
#SAKNAS!
#SAKNAS!
#SAKNAS!
#SAKNAS!
#SAKNAS!
#SAKNAS!
#SAKNAS!
#SAKNAS!
#SAKNAS!
#SAKNAS!
2395 (0.036)
#SAKNAS!
#SAKNAS!
2395 (0.036)
2395 (0.036)
#SAKNAS!
#SAKNAS!
#SAKNAS!
2395 (0.036)
2395 (0.036)
2395 (0.036)
#SAKNAS!
#SAKNAS!
#SAKNAS!
#SAKNAS!
#SAKNAS!
#SAKNAS!
#SAKNAS!
2395 (0.036)
#SAKNAS!
#SAKNAS!
#SAKNAS!
#SAKNAS!
#SAKNAS!
#SAKNAS!
#SAKNAS!

#SAKNAS!
#SAKNAS!
#SAKNAS!
2395 (0.036)
1164 (0.11)
#SAKNAS!
#SAKNAS!
#SAKNAS!
2395 (0.036)
#SAKNAS!
#SAKNAS!
#SAKNAS!
#SAKNAS!
2395 (0.036)
#SAKNAS!
#SAKNAS!
2395 (0.036)
#SAKNAS!
#SAKNAS!
#SAKNAS!
#SAKNAS!
#SAKNAS!
#SAKNAS!
#SAKNAS!
#SAKNAS!
#SAKNAS!
#SAKNAS!
#SAKNAS!
#SAKNAS!
#SAKNAS!
#SAKNAS!
2395 (0.036)
2395 (0.036)
2395 (0.036)
#SAKNAS!
#SAKNAS!
2395 (0.036)
2395 (0.036)
#SAKNAS!
#SAKNAS!
#SAKNAS!
#SAKNAS!
#SAKNAS!

#SAKNAS!
#SAKNAS!
2395 (0.036)
2395 (0.036)
#SAKNAS!
2395 (0.036)
#SAKNAS!
2395 (0.036)
#SAKNAS!
#SAKNAS!
2395 (0.036)
#SAKNAS!
#SAKNAS!
#SAKNAS!
#SAKNAS!
#SAKNAS!
#SAKNAS!
#SAKNAS!
#SAKNAS!
2395 (0.036)
#SAKNAS!
#SAKNAS!
2395 (0.036)
#SAKNAS!
#SAKNAS!
#SAKNAS!
#SAKNAS!
#SAKNAS!
2395 (0.036)
#SAKNAS!
#SAKNAS!
#SAKNAS!
#SAKNAS!
2395 (0.036)
2395 (0.036)
2395 (0.036)
#SAKNAS!
#SAKNAS!
#SAKNAS!
#SAKNAS!
#SAKNAS!
2395 (0.036)
2395 (0.036)

#SAKNAS!
#SAKNAS!
#SAKNAS!
#SAKNAS!
#SAKNAS!
#SAKNAS!
#SAKNAS!
#SAKNAS!
#SAKNAS!
#SAKNAS!
#SAKNAS!
#SAKNAS!
#SAKNAS!
#SAKNAS!
#SAKNAS!
#SAKNAS!
#SAKNAS!
#SAKNAS!
2395 (0.036)
#SAKNAS!
#SAKNAS!
#SAKNAS!
#SAKNAS!
#SAKNAS!
2395 (0.036)
#SAKNAS!
#SAKNAS!
#SAKNAS!
#SAKNAS!
#SAKNAS!
#SAKNAS!
#SAKNAS!
2395 (0.036)
2395 (0.036)
2395 (0.036)
2395 (0.036)
#SAKNAS!
#SAKNAS!
2395 (0.036)
2395 (0.036)
2395 (0.036)
#SAKNAS!
#SAKNAS!

1573 (0.071)
#SAKNAS!
2395 (0.036)
2395 (0.036)
#SAKNAS!
#SAKNAS!
#SAKNAS!
2395 (0.036)
2395 (0.036)
2395 (0.036)
#SAKNAS!
#SAKNAS!
#SAKNAS!
#SAKNAS!
#SAKNAS!
#SAKNAS!
#SAKNAS!
#SAKNAS!
#SAKNAS!
#SAKNAS!
#SAKNAS!
2395 (0.036)
#SAKNAS!
#SAKNAS!
#SAKNAS!
#SAKNAS!
#SAKNAS!
#SAKNAS!
#SAKNAS!
#SAKNAS!
#SAKNAS!
#SAKNAS!
2395 (0.036)
#SAKNAS!
#SAKNAS!
#SAKNAS!
2395 (0.036)
#SAKNAS!
#SAKNAS!
#SAKNAS!
2395 (0.036)
#SAKNAS!
#SAKNAS!

#SAKNAS!
#SAKNAS!
#SAKNAS!
#SAKNAS!
#SAKNAS!
#SAKNAS!
#SAKNAS!
#SAKNAS!
2395 (0.036)
#SAKNAS!
#SAKNAS!
#SAKNAS!
#SAKNAS!
#SAKNAS!
#SAKNAS!
#SAKNAS!
#SAKNAS!
#SAKNAS!
#SAKNAS!
#SAKNAS!
1573 (0.071)
#SAKNAS!
#SAKNAS!
2395 (0.036)
#SAKNAS!
#SAKNAS!
#SAKNAS!
2395 (0.036)
#SAKNAS!
#SAKNAS!
#SAKNAS!
#SAKNAS!
#SAKNAS!
#SAKNAS!
2395 (0.036)
#SAKNAS!
#SAKNAS!
#SAKNAS!
#SAKNAS!
2395 (0.036)
#SAKNAS!
#SAKNAS!
#SAKNAS!

2395 (0.036)
#SAKNAS!
#SAKNAS!
#SAKNAS!
#SAKNAS!
#SAKNAS!
#SAKNAS!
#SAKNAS!
#SAKNAS!
#SAKNAS!
#SAKNAS!
#SAKNAS!
#SAKNAS!
#SAKNAS!
#SAKNAS!
#SAKNAS!
#SAKNAS!
#SAKNAS!
#SAKNAS!
#SAKNAS!
2395 (0.036)
2395 (0.036)
2395 (0.036)
1573 (0.071)
#SAKNAS!
1573 (0.071)
#SAKNAS!
#SAKNAS!
#SAKNAS!
2395 (0.036)
#SAKNAS!
#SAKNAS!
2395 (0.036)
2395 (0.036)
#SAKNAS!
2395 (0.036)
#SAKNAS!
#SAKNAS!
#SAKNAS!
2395 (0.036)
2395 (0.036)
#SAKNAS!
#SAKNAS!

#SAKNAS!
#SAKNAS!
2395 (0.036)
2395 (0.036)
1573 (0.071)
#SAKNAS!
#SAKNAS!
#SAKNAS!
#SAKNAS!
2395 (0.036)
2395 (0.036)
#SAKNAS!
#SAKNAS!
#SAKNAS!
2395 (0.036)
2395 (0.036)
#SAKNAS!
#SAKNAS!
2395 (0.036)
#SAKNAS!
#SAKNAS!
#SAKNAS!
#SAKNAS!
#SAKNAS!
#SAKNAS!
#SAKNAS!
#SAKNAS!
2395 (0.036)
#SAKNAS!
2395 (0.036)
2395 (0.036)
#SAKNAS!
#SAKNAS!
#SAKNAS!
2395 (0.036)
#SAKNAS!
#SAKNAS!
#SAKNAS!
#SAKNAS!
#SAKNAS!
#SAKNAS!
#SAKNAS!
#SAKNAS!

#SAKNAS!
#SAKNAS!
2395 (0.036)
#SAKNAS!
2395 (0.036)
#SAKNAS!
#SAKNAS!
2395 (0.036)
2395 (0.036)
#SAKNAS!
2395 (0.036)
#SAKNAS!
#SAKNAS!
#SAKNAS!
#SAKNAS!
#SAKNAS!
#SAKNAS!
#SAKNAS!
#SAKNAS!
#SAKNAS!
#SAKNAS!
2395 (0.036)
2395 (0.036)
2395 (0.036)
#SAKNAS!
#SAKNAS!
2395 (0.036)
#SAKNAS!
#SAKNAS!
#SAKNAS!
2395 (0.036)
2395 (0.036)
#SAKNAS!
#SAKNAS!
#SAKNAS!
#SAKNAS!
#SAKNAS!
#SAKNAS!
#SAKNAS!
#SAKNAS!
2395 (0.036)
#SAKNAS!
#SAKNAS!

#SAKNAS!
2395 (0.036)
#SAKNAS!
#SAKNAS!
2395 (0.036)
#SAKNAS!
#SAKNAS!
#SAKNAS!
#SAKNAS!
#SAKNAS!
2395 (0.036)
#SAKNAS!
#SAKNAS!
2395 (0.036)
2395 (0.036)
2395 (0.036)
#SAKNAS!
#SAKNAS!
2395 (0.036)
#SAKNAS!
#SAKNAS!
#SAKNAS!
#SAKNAS!
#SAKNAS!
1573 (0.071)
#SAKNAS!
#SAKNAS!
#SAKNAS!
#SAKNAS!
#SAKNAS!
#SAKNAS!
#SAKNAS!
#SAKNAS!
2395 (0.036)
2395 (0.036)
#SAKNAS!
#SAKNAS!
#SAKNAS!
#SAKNAS!
#SAKNAS!
#SAKNAS!
2395 (0.036)
#SAKNAS!

#SAKNAS!
2395 (0.036)
2395 (0.036)
#SAKNAS!
#SAKNAS!
#SAKNAS!
#SAKNAS!
#SAKNAS!
#SAKNAS!
#SAKNAS!
2395 (0.036)
2395 (0.036)
#SAKNAS!
2395 (0.036)
#SAKNAS!
#SAKNAS!
#SAKNAS!
#SAKNAS!
2395 (0.036)
#SAKNAS!
2395 (0.036)
1573 (0.071)
#SAKNAS!
#SAKNAS!
#SAKNAS!
2395 (0.036)
#SAKNAS!
#SAKNAS!
2395 (0.036)
2395 (0.036)
2395 (0.036)
#SAKNAS!
#SAKNAS!
2395 (0.036)
1164 (0.11)
#SAKNAS!
#SAKNAS!
#SAKNAS!
#SAKNAS!
#SAKNAS!
#SAKNAS!
#SAKNAS!
#SAKNAS!

#SAKNAS!
#SAKNAS!
2395 (0.036)
#SAKNAS!
#SAKNAS!
#SAKNAS!
#SAKNAS!
#SAKNAS!
#SAKNAS!
2395 (0.036)
#SAKNAS!
#SAKNAS!
#SAKNAS!
#SAKNAS!
1573 (0.071)
#SAKNAS!
#SAKNAS!
#SAKNAS!
#SAKNAS!
#SAKNAS!
#SAKNAS!
2395 (0.036)
#SAKNAS!
#SAKNAS!
#SAKNAS!
#SAKNAS!
2395 (0.036)
2395 (0.036)
#SAKNAS!
#SAKNAS!
#SAKNAS!
936 (0.14)
2395 (0.036)
#SAKNAS!
2395 (0.036)
2395 (0.036)
2395 (0.036)
#SAKNAS!
#SAKNAS!
#SAKNAS!
#SAKNAS!
#SAKNAS!
#SAKNAS!

#SAKNAS!
#SAKNAS!
#SAKNAS!
#SAKNAS!
#SAKNAS!
2395 (0.036)
2395 (0.036)
#SAKNAS!
2395 (0.036)
2395 (0.036)
2395 (0.036)
1573 (0.071)
#SAKNAS!
#SAKNAS!
2395 (0.036)
2395 (0.036)
#SAKNAS!
2395 (0.036)
2395 (0.036)
#SAKNAS!
#SAKNAS!
1573 (0.071)
#SAKNAS!
2395 (0.036)
2395 (0.036)
#SAKNAS!
#SAKNAS!
#SAKNAS!
#SAKNAS!
2395 (0.036)
#SAKNAS!
#SAKNAS!
#SAKNAS!
#SAKNAS!
#SAKNAS!
#SAKNAS!
2395 (0.036)
1573 (0.071)
#SAKNAS!
#SAKNAS!
#SAKNAS!
#SAKNAS!
#SAKNAS!

2395 (0.036)
#SAKNAS!
2395 (0.036)
#SAKNAS!
2395 (0.036)
#SAKNAS!
#SAKNAS!
#SAKNAS!
#SAKNAS!
#SAKNAS!
#SAKNAS!
2395 (0.036)
#SAKNAS!
2395 (0.036)
2395 (0.036)
#SAKNAS!
#SAKNAS!
2395 (0.036)
#SAKNAS!
#SAKNAS!
#SAKNAS!
2395 (0.036)
#SAKNAS!
#SAKNAS!
#SAKNAS!
#SAKNAS!
#SAKNAS!
#SAKNAS!
#SAKNAS!
#SAKNAS!
#SAKNAS!
2395 (0.036)
#SAKNAS!
2395 (0.036)
#SAKNAS!
#SAKNAS!
#SAKNAS!
#SAKNAS!
#SAKNAS!
#SAKNAS!
#SAKNAS!
#SAKNAS!
#SAKNAS!

#SAKNAS!
#SAKNAS!
#SAKNAS!
#SAKNAS!
#SAKNAS!
#SAKNAS!
#SAKNAS!
#SAKNAS!
#SAKNAS!
#SAKNAS!
#SAKNAS!
#SAKNAS!
#SAKNAS!
#SAKNAS!
2395 (0.036)
#SAKNAS!
#SAKNAS!
#SAKNAS!
2395 (0.036)
2395 (0.036)
2395 (0.036)
#SAKNAS!
#SAKNAS!
#SAKNAS!
#SAKNAS!
#SAKNAS!
#SAKNAS!
2395 (0.036)
#SAKNAS!
#SAKNAS!
1573 (0.071)
#SAKNAS!
#SAKNAS!
#SAKNAS!
#SAKNAS!
#SAKNAS!
#SAKNAS!
1573 (0.071)
#SAKNAS!
2395 (0.036)
#SAKNAS!
#SAKNAS!
#SAKNAS!

#SAKNAS!
#SAKNAS!
#SAKNAS!
2395 (0.036)
2395 (0.036)
#SAKNAS!
2395 (0.036)
#SAKNAS!
#SAKNAS!
#SAKNAS!
#SAKNAS!
#SAKNAS!
#SAKNAS!
2395 (0.036)
#SAKNAS!
#SAKNAS!
1164 (0.11)
2395 (0.036)
2395 (0.036)
#SAKNAS!
#SAKNAS!
2395 (0.036)
2395 (0.036)
#SAKNAS!
#SAKNAS!
#SAKNAS!
2395 (0.036)
2395 (0.036)
2395 (0.036)
2395 (0.036)
#SAKNAS!
#SAKNAS!
2395 (0.036)
#SAKNAS!
#SAKNAS!
2395 (0.036)
#SAKNAS!
#SAKNAS!
#SAKNAS!
#SAKNAS!
#SAKNAS!
#SAKNAS!
#SAKNAS!

2395 (0.036)
2395 (0.036)
2395 (0.036)
#SAKNAS!
#SAKNAS!
#SAKNAS!
2395 (0.036)
#SAKNAS!
2395 (0.036)
#SAKNAS!
#SAKNAS!
2395 (0.036)
#SAKNAS!
2395 (0.036)
#SAKNAS!
#SAKNAS!
#SAKNAS!
2395 (0.036)
2395 (0.036)
2395 (0.036)
1573 (0.071)
#SAKNAS!
2395 (0.036)
2395 (0.036)
#SAKNAS!
#SAKNAS!
#SAKNAS!
#SAKNAS!
#SAKNAS!
#SAKNAS!
2395 (0.036)
#SAKNAS!
#SAKNAS!
#SAKNAS!
1573 (0.071)
#SAKNAS!
#SAKNAS!
#SAKNAS!
#SAKNAS!
2395 (0.036)
#SAKNAS!
#SAKNAS!
2395 (0.036)

1164 (0.11)
2395 (0.036)
#SAKNAS!
#SAKNAS!
#SAKNAS!
#SAKNAS!
#SAKNAS!
#SAKNAS!
2395 (0.036)
#SAKNAS!
#SAKNAS!
#SAKNAS!
2395 (0.036)
#SAKNAS!
#SAKNAS!
#SAKNAS!
#SAKNAS!
#SAKNAS!
#SAKNAS!
#SAKNAS!
2395 (0.036)
2395 (0.036)
#SAKNAS!
#SAKNAS!
2395 (0.036)
2395 (0.036)
#SAKNAS!
#SAKNAS!
2395 (0.036)
2395 (0.036)
#SAKNAS!
2395 (0.036)
2395 (0.036)
2395 (0.036)
#SAKNAS!
#SAKNAS!
#SAKNAS!
2395 (0.036)
2395 (0.036)
#SAKNAS!
561 (0.25)
#SAKNAS!
#SAKNAS!

#SAKNAS!
#SAKNAS!
#SAKNAS!
#SAKNAS!
#SAKNAS!
#SAKNAS!
#SAKNAS!
#SAKNAS!
#SAKNAS!
#SAKNAS!
#SAKNAS!
#SAKNAS!
#SAKNAS!
#SAKNAS!
#SAKNAS!
#SAKNAS!
2395 (0.036)
2395 (0.036)
#SAKNAS!
#SAKNAS!
#SAKNAS!
2395 (0.036)
#SAKNAS!
#SAKNAS!
#SAKNAS!
#SAKNAS!
#SAKNAS!
#SAKNAS!
#SAKNAS!
#SAKNAS!
#SAKNAS!
#SAKNAS!
2395 (0.036)
#SAKNAS!
#SAKNAS!
#SAKNAS!
#SAKNAS!
1573 (0.071)
2395 (0.036)
#SAKNAS!
#SAKNAS!
2395 (0.036)
2395 (0.036)

#SAKNAS!
1164 (0.11)
2395 (0.036)
#SAKNAS!
#SAKNAS!
#SAKNAS!
#SAKNAS!
#SAKNAS!
2395 (0.036)
2395 (0.036)
#SAKNAS!
2395 (0.036)
2395 (0.036)
#SAKNAS!
#SAKNAS!
2395 (0.036)
2395 (0.036)
2395 (0.036)
#SAKNAS!
#SAKNAS!
1573 (0.071)
2395 (0.036)
#SAKNAS!
#SAKNAS!
2395 (0.036)
2395 (0.036)
#SAKNAS!
#SAKNAS!
1573 (0.071)
#SAKNAS!
#SAKNAS!
#SAKNAS!
#SAKNAS!
2395 (0.036)
#SAKNAS!
#SAKNAS!
#SAKNAS!
#SAKNAS!
#SAKNAS!
#SAKNAS!
#SAKNAS!
#SAKNAS!
#SAKNAS!

#SAKNAS!
#SAKNAS!
#SAKNAS!
#SAKNAS!
#SAKNAS!
#SAKNAS!
#SAKNAS!
2395 (0.036)
#SAKNAS!
#SAKNAS!
#SAKNAS!
#SAKNAS!
2395 (0.036)
#SAKNAS!
#SAKNAS!
#SAKNAS!
2395 (0.036)
#SAKNAS!
2395 (0.036)
#SAKNAS!
#SAKNAS!
#SAKNAS!
#SAKNAS!
#SAKNAS!
#SAKNAS!
#SAKNAS!
#SAKNAS!
#SAKNAS!
2395 (0.036)
#SAKNAS!
#SAKNAS!
#SAKNAS!
#SAKNAS!
#SAKNAS!
#SAKNAS!
#SAKNAS!
1573 (0.071)
#SAKNAS!
#SAKNAS!
2395 (0.036)
#SAKNAS!
#SAKNAS!
#SAKNAS!

#SAKNAS!
#SAKNAS!
#SAKNAS!
#SAKNAS!
#SAKNAS!
#SAKNAS!
#SAKNAS!
#SAKNAS!
#SAKNAS!
#SAKNAS!
#SAKNAS!
2395 (0.036)
#SAKNAS!
#SAKNAS!
2395 (0.036)
#SAKNAS!
#SAKNAS!
#SAKNAS!
936 (0.14)
936 (0.14)
#SAKNAS!
#SAKNAS!
2395 (0.036)
#SAKNAS!
#SAKNAS!
#SAKNAS!
#SAKNAS!
2395 (0.036)
2395 (0.036)
#SAKNAS!
#SAKNAS!
2395 (0.036)
2395 (0.036)
#SAKNAS!
#SAKNAS!
2395 (0.036)
#SAKNAS!
2395 (0.036)
2395 (0.036)
#SAKNAS!
#SAKNAS!
#SAKNAS!
2395 (0.036)

1164 (0.11)
773 (0.18)
#SAKNAS!
2395 (0.036)
#SAKNAS!
2395 (0.036)
#SAKNAS!
2395 (0.036)
#SAKNAS!
2395 (0.036)
2395 (0.036)
2395 (0.036)
#SAKNAS!
#SAKNAS!
#SAKNAS!
#SAKNAS!
#SAKNAS!
2395 (0.036)
#SAKNAS!
#SAKNAS!
2395 (0.036)
#SAKNAS!
#SAKNAS!
#SAKNAS!
#SAKNAS!
#SAKNAS!
#SAKNAS!
#SAKNAS!
2395 (0.036)
2395 (0.036)
#SAKNAS!
#SAKNAS!
#SAKNAS!
#SAKNAS!
#SAKNAS!
#SAKNAS!
#SAKNAS!
#SAKNAS!
2395 (0.036)
#SAKNAS!
#SAKNAS!
#SAKNAS!
#SAKNAS!

#SAKNAS!
2395 (0.036)
#SAKNAS!
#SAKNAS!
#SAKNAS!
#SAKNAS!
#SAKNAS!
#SAKNAS!
2395 (0.036)
#SAKNAS!
#SAKNAS!
2395 (0.036)
2395 (0.036)
2395 (0.036)
#SAKNAS!
2395 (0.036)
2395 (0.036)
#SAKNAS!
#SAKNAS!
#SAKNAS!
2395 (0.036)
#SAKNAS!
2395 (0.036)
#SAKNAS!
#SAKNAS!
2395 (0.036)
#SAKNAS!
2395 (0.036)
#SAKNAS!
#SAKNAS!
#SAKNAS!
2395 (0.036)
#SAKNAS!
#SAKNAS!
#SAKNAS!
2395 (0.036)
#SAKNAS!
#SAKNAS!
2395 (0.036)
2395 (0.036)
#SAKNAS!
#SAKNAS!
#SAKNAS!

#SAKNAS!
2395 (0.036)
#SAKNAS!
#SAKNAS!
#SAKNAS!
#SAKNAS!
#SAKNAS!
#SAKNAS!
#SAKNAS!
#SAKNAS!
#SAKNAS!
#SAKNAS!
2395 (0.036)
#SAKNAS!
#SAKNAS!
#SAKNAS!
#SAKNAS!
#SAKNAS!
#SAKNAS!
1573 (0.071)
2395 (0.036)
#SAKNAS!
#SAKNAS!
#SAKNAS!
#SAKNAS!
2395 (0.036)
2395 (0.036)
1164 (0.11)
2395 (0.036)
#SAKNAS!
#SAKNAS!
#SAKNAS!
#SAKNAS!
#SAKNAS!
#SAKNAS!
2395 (0.036)
2395 (0.036)
#SAKNAS!
#SAKNAS!
#SAKNAS!
#SAKNAS!
#SAKNAS!
#SAKNAS!

2395 (0.036)
#SAKNAS!
#SAKNAS!
#SAKNAS!
2395 (0.036)
#SAKNAS!
#SAKNAS!
#SAKNAS!
1164 (0.11)
1573 (0.071)
2395 (0.036)
1164 (0.11)
#SAKNAS!
#SAKNAS!
#SAKNAS!
2395 (0.036)
#SAKNAS!
#SAKNAS!
#SAKNAS!
#SAKNAS!
2395 (0.036)
#SAKNAS!
2395 (0.036)
#SAKNAS!
#SAKNAS!
#SAKNAS!
#SAKNAS!
#SAKNAS!
#SAKNAS!
#SAKNAS!
#SAKNAS!
2395 (0.036)
2395 (0.036)
2395 (0.036)
2395 (0.036)
2395 (0.036)
#SAKNAS!
#SAKNAS!
#SAKNAS!
#SAKNAS!
#SAKNAS!
#SAKNAS!
#SAKNAS!

2395 (0.036)
2395 (0.036)
2395 (0.036)
#SAKNAS!
#SAKNAS!
#SAKNAS!
#SAKNAS!
#SAKNAS!
#SAKNAS!
#SAKNAS!
#SAKNAS!
2395 (0.036)
#SAKNAS!
#SAKNAS!
#SAKNAS!
#SAKNAS!
#SAKNAS!
#SAKNAS!
#SAKNAS!
#SAKNAS!
#SAKNAS!
#SAKNAS!
2395 (0.036)
#SAKNAS!
2395 (0.036)
#SAKNAS!
#SAKNAS!
2395 (0.036)
#SAKNAS!
#SAKNAS!
#SAKNAS!
#SAKNAS!
2395 (0.036)
#SAKNAS!
#SAKNAS!
#SAKNAS!
#SAKNAS!
#SAKNAS!
#SAKNAS!
#SAKNAS!
#SAKNAS!
#SAKNAS!
#SAKNAS!

#SAKNAS!
2395 (0.036)
773 (0.18)
#SAKNAS!
#SAKNAS!
#SAKNAS!
#SAKNAS!
#SAKNAS!
#SAKNAS!
2395 (0.036)
#SAKNAS!
#SAKNAS!
#SAKNAS!
#SAKNAS!
#SAKNAS!
#SAKNAS!
#SAKNAS!
#SAKNAS!
#SAKNAS!
2395 (0.036)
2395 (0.036)
#SAKNAS!
#SAKNAS!
#SAKNAS!
#SAKNAS!
#SAKNAS!
2395 (0.036)
#SAKNAS!
#SAKNAS!
#SAKNAS!
2395 (0.036)
#SAKNAS!
#SAKNAS!
#SAKNAS!
#SAKNAS!
2395 (0.036)
2395 (0.036)
2395 (0.036)
2395 (0.036)
#SAKNAS!
#SAKNAS!
#SAKNAS!
#SAKNAS!

#SAKNAS!
2395 (0.036)
2395 (0.036)
2395 (0.036)
2395 (0.036)
#SAKNAS!
2395 (0.036)
2395 (0.036)
1573 (0.071)
#SAKNAS!
#SAKNAS!
#SAKNAS!
#SAKNAS!
#SAKNAS!
#SAKNAS!
#SAKNAS!
#SAKNAS!
#SAKNAS!
#SAKNAS!
#SAKNAS!
#SAKNAS!
#SAKNAS!
#SAKNAS!
#SAKNAS!
2395 (0.036)
2395 (0.036)
#SAKNAS!
#SAKNAS!
#SAKNAS!
#SAKNAS!
#SAKNAS!
2395 (0.036)
#SAKNAS!
#SAKNAS!
#SAKNAS!
#SAKNAS!
#SAKNAS!
#SAKNAS!
#SAKNAS!
#SAKNAS!
#SAKNAS!
#SAKNAS!
#SAKNAS!

#SAKNAS!
#SAKNAS!
2395 (0.036)
#SAKNAS!
#SAKNAS!
#SAKNAS!
#SAKNAS!
#SAKNAS!
2395 (0.036)
#SAKNAS!
#SAKNAS!
#SAKNAS!
#SAKNAS!
#SAKNAS!
#SAKNAS!
2395 (0.036)
#SAKNAS!
#SAKNAS!
#SAKNAS!
#SAKNAS!
#SAKNAS!
#SAKNAS!
2395 (0.036)
#SAKNAS!
#SAKNAS!
#SAKNAS!
#SAKNAS!
#SAKNAS!
#SAKNAS!
#SAKNAS!
#SAKNAS!
1164 (0.11)
2395 (0.036)
#SAKNAS!
#SAKNAS!
#SAKNAS!
#SAKNAS!
2395 (0.036)
#SAKNAS!
#SAKNAS!
#SAKNAS!
#SAKNAS!
#SAKNAS!

#SAKNAS!
#SAKNAS!
1573 (0.071)
2395 (0.036)
#SAKNAS!
#SAKNAS!
#SAKNAS!
#SAKNAS!
2395 (0.036)
#SAKNAS!
#SAKNAS!
2395 (0.036)
#SAKNAS!
#SAKNAS!
2395 (0.036)
#SAKNAS!
#SAKNAS!
#SAKNAS!
#SAKNAS!
#SAKNAS!
#SAKNAS!
#SAKNAS!
#SAKNAS!
2395 (0.036)
#SAKNAS!
#SAKNAS!
#SAKNAS!
#SAKNAS!
#SAKNAS!
2395 (0.036)
#SAKNAS!
#SAKNAS!
#SAKNAS!
#SAKNAS!
#SAKNAS!
#SAKNAS!
#SAKNAS!
#SAKNAS!
#SAKNAS!
#SAKNAS!
#SAKNAS!
#SAKNAS!
#SAKNAS!

#SAKNAS!
#SAKNAS!
#SAKNAS!
#SAKNAS!
#SAKNAS!
#SAKNAS!
#SAKNAS!
1573 (0.071)
#SAKNAS!
1573 (0.071)
#SAKNAS!
#SAKNAS!
#SAKNAS!
#SAKNAS!
#SAKNAS!
2395 (0.036)
2395 (0.036)
2395 (0.036)
#SAKNAS!
#SAKNAS!
#SAKNAS!
#SAKNAS!
#SAKNAS!
2395 (0.036)
#SAKNAS!
#SAKNAS!
#SAKNAS!
2395 (0.036)
2395 (0.036)
#SAKNAS!
#SAKNAS!
2395 (0.036)
#SAKNAS!
#SAKNAS!
#SAKNAS!
#SAKNAS!
#SAKNAS!
#SAKNAS!
#SAKNAS!
#SAKNAS!
#SAKNAS!
#SAKNAS!
#SAKNAS!

#SAKNAS!
2395 (0.036)
#SAKNAS!
#SAKNAS!
#SAKNAS!
#SAKNAS!
#SAKNAS!
#SAKNAS!
#SAKNAS!
#SAKNAS!
#SAKNAS!
2395 (0.036)
#SAKNAS!
#SAKNAS!
#SAKNAS!
#SAKNAS!
#SAKNAS!
#SAKNAS!
#SAKNAS!
#SAKNAS!
#SAKNAS!
#SAKNAS!
#SAKNAS!
#SAKNAS!
#SAKNAS!
#SAKNAS!
#SAKNAS!
#SAKNAS!
#SAKNAS!
2395 (0.036)
#SAKNAS!
2395 (0.036)
1573 (0.071)
#SAKNAS!
#SAKNAS!
#SAKNAS!
#SAKNAS!
#SAKNAS!
#SAKNAS!
2395 (0.036)
#SAKNAS!
#SAKNAS!
#SAKNAS!

#SAKNAS!
2395 (0.036)
#SAKNAS!
#SAKNAS!
2395 (0.036)
#SAKNAS!
#SAKNAS!
#SAKNAS!
#SAKNAS!
#SAKNAS!
#SAKNAS!
#SAKNAS!
#SAKNAS!
#SAKNAS!
#SAKNAS!
#SAKNAS!
#SAKNAS!
2395 (0.036)
#SAKNAS!
#SAKNAS!
#SAKNAS!
#SAKNAS!
2395 (0.036)
2395 (0.036)
#SAKNAS!
#SAKNAS!
2395 (0.036)
#SAKNAS!
#SAKNAS!
#SAKNAS!
#SAKNAS!
2395 (0.036)
#SAKNAS!
#SAKNAS!
2395 (0.036)
#SAKNAS!
#SAKNAS!
#SAKNAS!
#SAKNAS!
2395 (0.036)
#SAKNAS!
#SAKNAS!
#SAKNAS!

#SAKNAS!
#SAKNAS!
2395 (0.036)
2395 (0.036)
2395 (0.036)
1573 (0.071)
#SAKNAS!
#SAKNAS!
#SAKNAS!
#SAKNAS!
#SAKNAS!
2395 (0.036)
#SAKNAS!
#SAKNAS!
#SAKNAS!
#SAKNAS!
#SAKNAS!
#SAKNAS!
#SAKNAS!
2395 (0.036)
#SAKNAS!
2395 (0.036)
#SAKNAS!
#SAKNAS!
#SAKNAS!
#SAKNAS!
1573 (0.071)
2395 (0.036)
#SAKNAS!
2395 (0.036)
#SAKNAS!
#SAKNAS!
2395 (0.036)
#SAKNAS!
#SAKNAS!
#SAKNAS!
#SAKNAS!
2395 (0.036)
2395 (0.036)
#SAKNAS!
#SAKNAS!
#SAKNAS!
#SAKNAS!

#SAKNAS!
#SAKNAS!
#SAKNAS!
#SAKNAS!
2395 (0.036)
#SAKNAS!
#SAKNAS!
#SAKNAS!
2395 (0.036)
#SAKNAS!
#SAKNAS!
2395 (0.036)
#SAKNAS!
#SAKNAS!
#SAKNAS!
#SAKNAS!
#SAKNAS!
#SAKNAS!
#SAKNAS!
1573 (0.071)
#SAKNAS!
2395 (0.036)
2395 (0.036)
#SAKNAS!
#SAKNAS!
#SAKNAS!
#SAKNAS!
#SAKNAS!
2395 (0.036)
#SAKNAS!
#SAKNAS!
#SAKNAS!
#SAKNAS!
2395 (0.036)
#SAKNAS!
2395 (0.036)
2395 (0.036)
#SAKNAS!
2395 (0.036)
2395 (0.036)
#SAKNAS!
#SAKNAS!
2395 (0.036)

#SAKNAS!
#SAKNAS!
2395 (0.036)
#SAKNAS!
#SAKNAS!
#SAKNAS!
#SAKNAS!
2395 (0.036)
#SAKNAS!
#SAKNAS!
#SAKNAS!
#SAKNAS!
#SAKNAS!
#SAKNAS!
#SAKNAS!
#SAKNAS!
#SAKNAS!
2395 (0.036)
#SAKNAS!
#SAKNAS!
#SAKNAS!
#SAKNAS!
#SAKNAS!
2395 (0.036)
#SAKNAS!
#SAKNAS!
#SAKNAS!
446 (0.32)
2395 (0.036)
#SAKNAS!
#SAKNAS!
#SAKNAS!
#SAKNAS!
#SAKNAS!
#SAKNAS!
#SAKNAS!
#SAKNAS!
#SAKNAS!
2395 (0.036)
#SAKNAS!
2395 (0.036)
#SAKNAS!
2395 (0.036)

2395 (0.036)
2395 (0.036)
#SAKNAS!
#SAKNAS!
#SAKNAS!
#SAKNAS!
2395 (0.036)
#SAKNAS!
1573 (0.071)
#SAKNAS!
#SAKNAS!
2395 (0.036)
2395 (0.036)
#SAKNAS!
2395 (0.036)
411 (0.36)
2395 (0.036)
#SAKNAS!
#SAKNAS!
#SAKNAS!
#SAKNAS!
#SAKNAS!
#SAKNAS!
#SAKNAS!
#SAKNAS!
#SAKNAS!
2395 (0.036)
2395 (0.036)
#SAKNAS!
#SAKNAS!
648 (0.21)
#SAKNAS!
#SAKNAS!
2395 (0.036)
#SAKNAS!
#SAKNAS!
#SAKNAS!
#SAKNAS!
#SAKNAS!
1573 (0.071)
#SAKNAS!
#SAKNAS!
#SAKNAS!

#SAKNAS!
2395 (0.036)
#SAKNAS!
1573 (0.071)
#SAKNAS!
#SAKNAS!
#SAKNAS!
#SAKNAS!
2395 (0.036)
#SAKNAS!
#SAKNAS!
#SAKNAS!
#SAKNAS!
1164 (0.11)
2395 (0.036)
#SAKNAS!
#SAKNAS!
#SAKNAS!
#SAKNAS!
#SAKNAS!
#SAKNAS!
2395 (0.036)
#SAKNAS!
#SAKNAS!
#SAKNAS!
2395 (0.036)
2395 (0.036)
1573 (0.071)
#SAKNAS!
#SAKNAS!
#SAKNAS!
#SAKNAS!
#SAKNAS!
#SAKNAS!
#SAKNAS!
2395 (0.036)
#SAKNAS!
#SAKNAS!
#SAKNAS!
#SAKNAS!
2395 (0.036)
#SAKNAS!
#SAKNAS!

2395 (0.036)
#SAKNAS!
2395 (0.036)
#SAKNAS!
#SAKNAS!
2395 (0.036)
2395 (0.036)
2395 (0.036)
#SAKNAS!
#SAKNAS!
2395 (0.036)
#SAKNAS!
#SAKNAS!
#SAKNAS!
#SAKNAS!
2395 (0.036)
2395 (0.036)
2395 (0.036)
2395 (0.036)
#SAKNAS!
#SAKNAS!
2395 (0.036)
#SAKNAS!
#SAKNAS!
2395 (0.036)
#SAKNAS!
#SAKNAS!
#SAKNAS!
#SAKNAS!
#SAKNAS!
#SAKNAS!
2395 (0.036)
2395 (0.036)
#SAKNAS!
2395 (0.036)
#SAKNAS!
2395 (0.036)
2395 (0.036)
#SAKNAS!
#SAKNAS!
#SAKNAS!
#SAKNAS!
#SAKNAS!

#SAKNAS!
#SAKNAS!
2395 (0.036)
#SAKNAS!
#SAKNAS!
1573 (0.071)
#SAKNAS!
#SAKNAS!
#SAKNAS!
#SAKNAS!
2395 (0.036)
#SAKNAS!
#SAKNAS!
#SAKNAS!
#SAKNAS!
#SAKNAS!
#SAKNAS!
#SAKNAS!
#SAKNAS!
2395 (0.036)
#SAKNAS!
#SAKNAS!
2395 (0.036)
1573 (0.071)
1573 (0.071)
1573 (0.071)
#SAKNAS!
#SAKNAS!
2395 (0.036)
#SAKNAS!
#SAKNAS!
#SAKNAS!
#SAKNAS!
#SAKNAS!
#SAKNAS!
#SAKNAS!
#SAKNAS!
2395 (0.036)
2395 (0.036)
2395 (0.036)
#SAKNAS!
#SAKNAS!
#SAKNAS!

1164 (0.11)
#SAKNAS!
1573 (0.071)
#SAKNAS!
#SAKNAS!
#SAKNAS!
#SAKNAS!
#SAKNAS!
#SAKNAS!
#SAKNAS!
1573 (0.071)
#SAKNAS!
#SAKNAS!
#SAKNAS!
#SAKNAS!
2395 (0.036)
#SAKNAS!
#SAKNAS!
2395 (0.036)
#SAKNAS!
#SAKNAS!
2395 (0.036)
#SAKNAS!
#SAKNAS!
#SAKNAS!
2395 (0.036)
2395 (0.036)
#SAKNAS!
2395 (0.036)
#SAKNAS!
#SAKNAS!
#SAKNAS!
#SAKNAS!
2395 (0.036)
#SAKNAS!
#SAKNAS!
#SAKNAS!
#SAKNAS!
#SAKNAS!
#SAKNAS!
648 (0.21)
773 (0.18)
1164 (0.11)

2395 (0.036)
2395 (0.036)
#SAKNAS!
#SAKNAS!
#SAKNAS!
#SAKNAS!
#SAKNAS!
2395 (0.036)
#SAKNAS!
#SAKNAS!
2395 (0.036)
#SAKNAS!
#SAKNAS!
2395 (0.036)
#SAKNAS!
#SAKNAS!
#SAKNAS!
561 (0.25)
#SAKNAS!
2395 (0.036)
2395 (0.036)
773 (0.18)
#SAKNAS!
#SAKNAS!
#SAKNAS!
2395 (0.036)
1573 (0.071)
#SAKNAS!
#SAKNAS!
#SAKNAS!
#SAKNAS!
2395 (0.036)
#SAKNAS!
#SAKNAS!
#SAKNAS!
#SAKNAS!
#SAKNAS!
#SAKNAS!
#SAKNAS!
2395 (0.036)
#SAKNAS!
#SAKNAS!
#SAKNAS!

#SAKNAS!
#SAKNAS!
#SAKNAS!
#SAKNAS!
#SAKNAS!
#SAKNAS!
#SAKNAS!
648 (0.21)
#SAKNAS!
#SAKNAS!
#SAKNAS!
#SAKNAS!
#SAKNAS!
2395 (0.036)
#SAKNAS!
#SAKNAS!
#SAKNAS!
#SAKNAS!
#SAKNAS!
#SAKNAS!
2395 (0.036)
2395 (0.036)
#SAKNAS!
#SAKNAS!
#SAKNAS!
#SAKNAS!
2395 (0.036)
2395 (0.036)
#SAKNAS!
#SAKNAS!
2395 (0.036)
2395 (0.036)
#SAKNAS!
#SAKNAS!
2395 (0.036)
#SAKNAS!
#SAKNAS!
#SAKNAS!
#SAKNAS!
#SAKNAS!
#SAKNAS!
1164 (0.11)
#SAKNAS!

1573 (0.071)
#SAKNAS!
773 (0.18)
2395 (0.036)
2395 (0.036)
#SAKNAS!
#SAKNAS!
#SAKNAS!
#SAKNAS!
#SAKNAS!
#SAKNAS!
#SAKNAS!
#SAKNAS!
#SAKNAS!
2395 (0.036)
#SAKNAS!
#SAKNAS!
#SAKNAS!
#SAKNAS!
#SAKNAS!
#SAKNAS!
#SAKNAS!
#SAKNAS!
#SAKNAS!
#SAKNAS!
#SAKNAS!
#SAKNAS!
2395 (0.036)
1573 (0.071)
2395 (0.036)
#SAKNAS!
#SAKNAS!
2395 (0.036)
#SAKNAS!
2395 (0.036)
#SAKNAS!
2395 (0.036)
#SAKNAS!
#SAKNAS!
2395 (0.036)
2395 (0.036)
#SAKNAS!
#SAKNAS!

2395 (0.036)
#SAKNAS!
#SAKNAS!
#SAKNAS!
#SAKNAS!
#SAKNAS!
#SAKNAS!
#SAKNAS!
#SAKNAS!
#SAKNAS!
#SAKNAS!
#SAKNAS!
#SAKNAS!
#SAKNAS!
#SAKNAS!
2395 (0.036)
2395 (0.036)
#SAKNAS!
#SAKNAS!
2395 (0.036)
#SAKNAS!
#SAKNAS!
#SAKNAS!
2395 (0.036)
#SAKNAS!
#SAKNAS!
2395 (0.036)
#SAKNAS!
#SAKNAS!
1573 (0.071)
#SAKNAS!
#SAKNAS!
2395 (0.036)
2395 (0.036)
#SAKNAS!
#SAKNAS!
#SAKNAS!
#SAKNAS!
#SAKNAS!
#SAKNAS!
2395 (0.036)
#SAKNAS!
#SAKNAS!

2395 (0.036)
#SAKNAS!
#SAKNAS!
#SAKNAS!
#SAKNAS!
2395 (0.036)
#SAKNAS!
#SAKNAS!
#SAKNAS!
#SAKNAS!
#SAKNAS!
#SAKNAS!
#SAKNAS!
#SAKNAS!
2395 (0.036)
#SAKNAS!
#SAKNAS!
2395 (0.036)
#SAKNAS!
#SAKNAS!
#SAKNAS!
2395 (0.036)
#SAKNAS!
#SAKNAS!
#SAKNAS!
#SAKNAS!
#SAKNAS!
#SAKNAS!
#SAKNAS!
#SAKNAS!
#SAKNAS!
#SAKNAS!
#SAKNAS!
#SAKNAS!
#SAKNAS!
#SAKNAS!
#SAKNAS!
#SAKNAS!
#SAKNAS!
2395 (0.036)
#SAKNAS!
#SAKNAS!
2395 (0.036)

#SAKNAS!
2395 (0.036)
#SAKNAS!
#SAKNAS!
#SAKNAS!
#SAKNAS!
#SAKNAS!
#SAKNAS!
#SAKNAS!
#SAKNAS!
#SAKNAS!
#SAKNAS!
#SAKNAS!
#SAKNAS!
#SAKNAS!
2395 (0.036)
#SAKNAS!
#SAKNAS!
#SAKNAS!
#SAKNAS!
2395 (0.036)
2395 (0.036)
#SAKNAS!
#SAKNAS!
#SAKNAS!
2395 (0.036)
2395 (0.036)
2395 (0.036)
#SAKNAS!
#SAKNAS!
2395 (0.036)
#SAKNAS!
#SAKNAS!
2395 (0.036)
#SAKNAS!
#SAKNAS!
#SAKNAS!
#SAKNAS!
#SAKNAS!
2395 (0.036)
#SAKNAS!
#SAKNAS!
2395 (0.036)

2395 (0.036)
#SAKNAS!
#SAKNAS!
2395 (0.036)
#SAKNAS!
2395 (0.036)
2395 (0.036)
#SAKNAS!
2395 (0.036)
#SAKNAS!
#SAKNAS!
#SAKNAS!
2395 (0.036)
#SAKNAS!
2395 (0.036)
#SAKNAS!
#SAKNAS!
2395 (0.036)
#SAKNAS!
#SAKNAS!
2395 (0.036)
2395 (0.036)
#SAKNAS!
#SAKNAS!
2395 (0.036)
2395 (0.036)
2395 (0.036)
#SAKNAS!
#SAKNAS!
2395 (0.036)
#SAKNAS!
#SAKNAS!
#SAKNAS!
#SAKNAS!
#SAKNAS!
2395 (0.036)
#SAKNAS!
2395 (0.036)
1573 (0.071)
#SAKNAS!
#SAKNAS!
2395 (0.036)
#SAKNAS!

773 (0.18)
2395 (0.036)
#SAKNAS!
#SAKNAS!
2395 (0.036)
#SAKNAS!
1164 (0.11)
#SAKNAS!
2395 (0.036)
1573 (0.071)
#SAKNAS!
2395 (0.036)
2395 (0.036)
#SAKNAS!
2395 (0.036)
#SAKNAS!
2395 (0.036)
#SAKNAS!
#SAKNAS!
#SAKNAS!
#SAKNAS!
#SAKNAS!
2395 (0.036)
#SAKNAS!
#SAKNAS!
2395 (0.036)
2395 (0.036)
#SAKNAS!
#SAKNAS!
#SAKNAS!
2395 (0.036)
#SAKNAS!
#SAKNAS!
#SAKNAS!
#SAKNAS!
#SAKNAS!
#SAKNAS!
2395 (0.036)
#SAKNAS!
2395 (0.036)
2395 (0.036)
#SAKNAS!
2395 (0.036)

2395 (0.036)
#SAKNAS!
#SAKNAS!
#SAKNAS!
#SAKNAS!
2395 (0.036)
2395 (0.036)
#SAKNAS!
#SAKNAS!
#SAKNAS!
2395 (0.036)
#SAKNAS!
#SAKNAS!
#SAKNAS!
#SAKNAS!
2395 (0.036)
#SAKNAS!
2395 (0.036)
#SAKNAS!
2395 (0.036)
#SAKNAS!
#SAKNAS!
#SAKNAS!
#SAKNAS!
#SAKNAS!
#SAKNAS!
#SAKNAS!
2395 (0.036)
2395 (0.036)
#SAKNAS!
#SAKNAS!
2395 (0.036)
#SAKNAS!
#SAKNAS!
2395 (0.036)
#SAKNAS!
2395 (0.036)
#SAKNAS!
#SAKNAS!
1573 (0.071)
2395 (0.036)
#SAKNAS!
#SAKNAS!

#SAKNAS!
#SAKNAS!
2395 (0.036)
936 (0.14)
#SAKNAS!
2395 (0.036)
#SAKNAS!
#SAKNAS!
2395 (0.036)
#SAKNAS!
#SAKNAS!
2395 (0.036)
936 (0.14)
2395 (0.036)
2395 (0.036)
#SAKNAS!
2395 (0.036)
2395 (0.036)
#SAKNAS!
#SAKNAS!
#SAKNAS!
#SAKNAS!
#SAKNAS!
#SAKNAS!
#SAKNAS!
#SAKNAS!
#SAKNAS!
#SAKNAS!
#SAKNAS!
#SAKNAS!
#SAKNAS!
2395 (0.036)
#SAKNAS!
#SAKNAS!
2395 (0.036)
773 (0.18)
#SAKNAS!
#SAKNAS!
1164 (0.11)
#SAKNAS!
2395 (0.036)
#SAKNAS!
2395 (0.036)

#SAKNAS!
#SAKNAS!
#SAKNAS!
2395 (0.036)
#SAKNAS!
#SAKNAS!
#SAKNAS!
2395 (0.036)
2395 (0.036)
2395 (0.036)
2395 (0.036)
#SAKNAS!
#SAKNAS!
#SAKNAS!
#SAKNAS!
#SAKNAS!
#SAKNAS!
#SAKNAS!
#SAKNAS!
2395 (0.036)
#SAKNAS!
#SAKNAS!
#SAKNAS!
#SAKNAS!
#SAKNAS!
#SAKNAS!
#SAKNAS!
#SAKNAS!
#SAKNAS!
1573 (0.071)
#SAKNAS!
#SAKNAS!
2395 (0.036)
#SAKNAS!
#SAKNAS!
2395 (0.036)
#SAKNAS!
2395 (0.036)
#SAKNAS!
411 (0.36)
2395 (0.036)
#SAKNAS!
#SAKNAS!

#SAKNAS!
#SAKNAS!
#SAKNAS!
2395 (0.036)
#SAKNAS!
#SAKNAS!
#SAKNAS!
2395 (0.036)
2395 (0.036)
2395 (0.036)
#SAKNAS!
#SAKNAS!
2395 (0.036)
1164 (0.11)
#SAKNAS!
2395 (0.036)
2395 (0.036)
#SAKNAS!
1573 (0.071)
#SAKNAS!
#SAKNAS!
2395 (0.036)
#SAKNAS!
#SAKNAS!
2395 (0.036)
2395 (0.036)
#SAKNAS!
#SAKNAS!
#SAKNAS!
2395 (0.036)
2395 (0.036)
#SAKNAS!
648 (0.21)
1573 (0.071)
#SAKNAS!
2395 (0.036)
#SAKNAS!
#SAKNAS!
#SAKNAS!
#SAKNAS!
#SAKNAS!
#SAKNAS!
#SAKNAS!

#SAKNAS!
2395 (0.036)
#SAKNAS!
#SAKNAS!
#SAKNAS!
#SAKNAS!
#SAKNAS!
#SAKNAS!
#SAKNAS!
#SAKNAS!
#SAKNAS!
#SAKNAS!
#SAKNAS!
#SAKNAS!
#SAKNAS!
2395 (0.036)
#SAKNAS!
#SAKNAS!
#SAKNAS!
#SAKNAS!
#SAKNAS!
#SAKNAS!
2395 (0.036)
#SAKNAS!
#SAKNAS!
#SAKNAS!
#SAKNAS!
#SAKNAS!
#SAKNAS!
#SAKNAS!
#SAKNAS!
#SAKNAS!
2395 (0.036)
2395 (0.036)
#SAKNAS!
#SAKNAS!
#SAKNAS!
#SAKNAS!
#SAKNAS!
#SAKNAS!
#SAKNAS!
2395 (0.036)

1164 (0.11)
2395 (0.036)
#SAKNAS!
#SAKNAS!
#SAKNAS!
2395 (0.036)
2395 (0.036)
#SAKNAS!
#SAKNAS!
#SAKNAS!
#SAKNAS!
2395 (0.036)
#SAKNAS!
#SAKNAS!
#SAKNAS!
#SAKNAS!
#SAKNAS!
#SAKNAS!
#SAKNAS!
2395 (0.036)
#SAKNAS!
#SAKNAS!
2395 (0.036)
#SAKNAS!
#SAKNAS!
#SAKNAS!
#SAKNAS!
#SAKNAS!
#SAKNAS!
2395 (0.036)
#SAKNAS!
#SAKNAS!
2395 (0.036)
#SAKNAS!
#SAKNAS!
#SAKNAS!
#SAKNAS!
#SAKNAS!
#SAKNAS!
#SAKNAS!
2395 (0.036)
#SAKNAS!
#SAKNAS!

#SAKNAS!
#SAKNAS!
#SAKNAS!
#SAKNAS!
#SAKNAS!
#SAKNAS!
#SAKNAS!
2395 (0.036)
#SAKNAS!
#SAKNAS!
2395 (0.036)
1573 (0.071)
2395 (0.036)
2395 (0.036)
2395 (0.036)
#SAKNAS!
#SAKNAS!
#SAKNAS!
2395 (0.036)
2395 (0.036)
2395 (0.036)
#SAKNAS!
#SAKNAS!
#SAKNAS!
2395 (0.036)
#SAKNAS!
#SAKNAS!
#SAKNAS!
#SAKNAS!
#SAKNAS!
2395 (0.036)
#SAKNAS!
2395 (0.036)
#SAKNAS!
#SAKNAS!
#SAKNAS!
#SAKNAS!
#SAKNAS!
#SAKNAS!
#SAKNAS!
#SAKNAS!
#SAKNAS!
2395 (0.036)

#SAKNAS!
2395 (0.036)
#SAKNAS!
2395 (0.036)
2395 (0.036)
2395 (0.036)
2395 (0.036)
2395 (0.036)
2395 (0.036)
#SAKNAS!
#SAKNAS!
#SAKNAS!
#SAKNAS!
#SAKNAS!
#SAKNAS!
#SAKNAS!
#SAKNAS!
#SAKNAS!
#SAKNAS!
#SAKNAS!
2395 (0.036)
#SAKNAS!
#SAKNAS!
2395 (0.036)
#SAKNAS!
#SAKNAS!
2395 (0.036)
2395 (0.036)
#SAKNAS!
#SAKNAS!
#SAKNAS!
#SAKNAS!
#SAKNAS!
#SAKNAS!
#SAKNAS!
#SAKNAS!
2395 (0.036)
1573 (0.071)
#SAKNAS!
#SAKNAS!
#SAKNAS!
#SAKNAS!
2395 (0.036)

2395 (0.036)
#SAKNAS!
2395 (0.036)
#SAKNAS!
#SAKNAS!
#SAKNAS!
#SAKNAS!
#SAKNAS!
#SAKNAS!
#SAKNAS!
2395 (0.036)
2395 (0.036)
#SAKNAS!
#SAKNAS!
#SAKNAS!
2395 (0.036)
#SAKNAS!
#SAKNAS!
#SAKNAS!
#SAKNAS!
#SAKNAS!
2395 (0.036)
#SAKNAS!
#SAKNAS!
2395 (0.036)
#SAKNAS!
#SAKNAS!
#SAKNAS!
#SAKNAS!
2395 (0.036)
#SAKNAS!
#SAKNAS!
#SAKNAS!
2395 (0.036)
2395 (0.036)
1573 (0.071)
#SAKNAS!
#SAKNAS!
#SAKNAS!
2395 (0.036)
#SAKNAS!
#SAKNAS!
2395 (0.036)

#SAKNAS!
#SAKNAS!
#SAKNAS!
#SAKNAS!
2395 (0.036)
#SAKNAS!
#SAKNAS!
2395 (0.036)
#SAKNAS!
#SAKNAS!
#SAKNAS!
#SAKNAS!
#SAKNAS!
#SAKNAS!
#SAKNAS!
#SAKNAS!
#SAKNAS!
#SAKNAS!
#SAKNAS!
#SAKNAS!
#SAKNAS!
#SAKNAS!
2395 (0.036)
#SAKNAS!
#SAKNAS!
#SAKNAS!
#SAKNAS!
#SAKNAS!
#SAKNAS!
#SAKNAS!
#SAKNAS!
#SAKNAS!
#SAKNAS!
#SAKNAS!
2395 (0.036)
2395 (0.036)
#SAKNAS!
2395 (0.036)
#SAKNAS!
#SAKNAS!
#SAKNAS!
#SAKNAS!
#SAKNAS!

#SAKNAS!
#SAKNAS!
#SAKNAS!
#SAKNAS!
#SAKNAS!
#SAKNAS!
2395 (0.036)
#SAKNAS!
#SAKNAS!
#SAKNAS!
#SAKNAS!
2395 (0.036)
2395 (0.036)
#SAKNAS!
#SAKNAS!
#SAKNAS!
#SAKNAS!
#SAKNAS!
#SAKNAS!
#SAKNAS!
#SAKNAS!
#SAKNAS!
#SAKNAS!
#SAKNAS!
1573 (0.071)
1573 (0.071)
773 (0.18)
#SAKNAS!
#SAKNAS!
#SAKNAS!
#SAKNAS!
2395 (0.036)
#SAKNAS!
2395 (0.036)
1573 (0.071)
1573 (0.071)
#SAKNAS!
2395 (0.036)
#SAKNAS!
#SAKNAS!
#SAKNAS!
#SAKNAS!
#SAKNAS!

#SAKNAS!
#SAKNAS!
2395 (0.036)
2395 (0.036)
#SAKNAS!
#SAKNAS!
#SAKNAS!
#SAKNAS!
#SAKNAS!
#SAKNAS!
#SAKNAS!
#SAKNAS!
#SAKNAS!
142 (1)
1573 (0.071)
2395 (0.036)
#SAKNAS!
#SAKNAS!
#SAKNAS!
648 (0.21)
2395 (0.036)
2395 (0.036)
2395 (0.036)
#SAKNAS!
#SAKNAS!
#SAKNAS!
#SAKNAS!
#SAKNAS!
#SAKNAS!
#SAKNAS!
#SAKNAS!
#SAKNAS!
#SAKNAS!
2395 (0.036)
2395 (0.036)
#SAKNAS!
#SAKNAS!
#SAKNAS!
2395 (0.036)
#SAKNAS!
#SAKNAS!
#SAKNAS!
#SAKNAS!

#SAKNAS!
#SAKNAS!
#SAKNAS!
#SAKNAS!
2395 (0.036)
#SAKNAS!
#SAKNAS!
2395 (0.036)
2395 (0.036)
1573 (0.071)
#SAKNAS!
2395 (0.036)
#SAKNAS!
#SAKNAS!
#SAKNAS!
2395 (0.036)
773 (0.18)
2395 (0.036)
#SAKNAS!
2395 (0.036)
2395 (0.036)
#SAKNAS!
2395 (0.036)
2395 (0.036)
2395 (0.036)
2395 (0.036)
#SAKNAS!
#SAKNAS!
#SAKNAS!
2395 (0.036)
#SAKNAS!
#SAKNAS!
2395 (0.036)
2395 (0.036)
#SAKNAS!
#SAKNAS!
#SAKNAS!
#SAKNAS!
#SAKNAS!
2395 (0.036)
#SAKNAS!
#SAKNAS!
#SAKNAS!

#SAKNAS!
#SAKNAS!
#SAKNAS!
2395 (0.036)
2395 (0.036)
2395 (0.036)
#SAKNAS!
#SAKNAS!
#SAKNAS!
#SAKNAS!
2395 (0.036)
2395 (0.036)
2395 (0.036)
#SAKNAS!
#SAKNAS!
2395 (0.036)
2395 (0.036)
#SAKNAS!
2395 (0.036)
2395 (0.036)
2395 (0.036)
2395 (0.036)
#SAKNAS!
2395 (0.036)
#SAKNAS!
#SAKNAS!
#SAKNAS!
#SAKNAS!
2395 (0.036)
#SAKNAS!
#SAKNAS!
#SAKNAS!
#SAKNAS!
2395 (0.036)
#SAKNAS!
#SAKNAS!
#SAKNAS!
#SAKNAS!
#SAKNAS!
#SAKNAS!
2395 (0.036)
#SAKNAS!
#SAKNAS!

#SAKNAS!
#SAKNAS!
#SAKNAS!
2395 (0.036)
2395 (0.036)
2395 (0.036)
#SAKNAS!
2395 (0.036)
#SAKNAS!
#SAKNAS!
#SAKNAS!
2395 (0.036)
#SAKNAS!
#SAKNAS!
#SAKNAS!
#SAKNAS!
#SAKNAS!
#SAKNAS!
#SAKNAS!
2395 (0.036)
#SAKNAS!
#SAKNAS!
#SAKNAS!
#SAKNAS!
#SAKNAS!
#SAKNAS!
#SAKNAS!
#SAKNAS!
1573 (0.071)
2395 (0.036)
2395 (0.036)
2395 (0.036)
2395 (0.036)
#SAKNAS!
2395 (0.036)
#SAKNAS!
#SAKNAS!
2395 (0.036)
2395 (0.036)
#SAKNAS!
2395 (0.036)
2395 (0.036)
#SAKNAS!

#SAKNAS!
#SAKNAS!
#SAKNAS!
#SAKNAS!
2395 (0.036)
#SAKNAS!
2395 (0.036)
2395 (0.036)
#SAKNAS!
#SAKNAS!
#SAKNAS!
1573 (0.071)
1573 (0.071)
#SAKNAS!
#SAKNAS!
#SAKNAS!
#SAKNAS!
#SAKNAS!
2395 (0.036)
#SAKNAS!
#SAKNAS!
#SAKNAS!
2395 (0.036)
#SAKNAS!
#SAKNAS!
2395 (0.036)
2395 (0.036)
#SAKNAS!
#SAKNAS!
#SAKNAS!
#SAKNAS!
#SAKNAS!
#SAKNAS!
2395 (0.036)
#SAKNAS!
2395 (0.036)
2395 (0.036)
#SAKNAS!
#SAKNAS!
#SAKNAS!
#SAKNAS!
#SAKNAS!
#SAKNAS!

#SAKNAS!
#SAKNAS!
2395 (0.036)
2395 (0.036)
#SAKNAS!
#SAKNAS!
#SAKNAS!
1164 (0.11)
1573 (0.071)
2395 (0.036)
#SAKNAS!
#SAKNAS!
#SAKNAS!
#SAKNAS!
#SAKNAS!
#SAKNAS!
#SAKNAS!
#SAKNAS!
2395 (0.036)
#SAKNAS!
#SAKNAS!
#SAKNAS!
#SAKNAS!
#SAKNAS!
#SAKNAS!
2395 (0.036)
2395 (0.036)
2395 (0.036)
#SAKNAS!
#SAKNAS!
2395 (0.036)
#SAKNAS!
2395 (0.036)
2395 (0.036)
#SAKNAS!
2395 (0.036)
#SAKNAS!
#SAKNAS!
#SAKNAS!
2395 (0.036)
#SAKNAS!
#SAKNAS!
2395 (0.036)

2395 (0.036)
#SAKNAS!
#SAKNAS!
2395 (0.036)
#SAKNAS!
#SAKNAS!
#SAKNAS!
#SAKNAS!
#SAKNAS!
#SAKNAS!
#SAKNAS!
2395 (0.036)
2395 (0.036)
#SAKNAS!
#SAKNAS!
2395 (0.036)
2395 (0.036)
#SAKNAS!
#SAKNAS!
#SAKNAS!
#SAKNAS!
2395 (0.036)
#SAKNAS!
#SAKNAS!
2395 (0.036)
#SAKNAS!
#SAKNAS!
#SAKNAS!
2395 (0.036)
#SAKNAS!
#SAKNAS!
#SAKNAS!
2395 (0.036)
#SAKNAS!
#SAKNAS!
#SAKNAS!
#SAKNAS!
#SAKNAS!
1573 (0.071)
#SAKNAS!
#SAKNAS!
#SAKNAS!
#SAKNAS!

2395 (0.036)
#SAKNAS!
#SAKNAS!
#SAKNAS!
#SAKNAS!
#SAKNAS!
#SAKNAS!
#SAKNAS!
#SAKNAS!
#SAKNAS!
#SAKNAS!
#SAKNAS!
2395 (0.036)
#SAKNAS!
2395 (0.036)
#SAKNAS!
#SAKNAS!
2395 (0.036)
#SAKNAS!
#SAKNAS!
#SAKNAS!
#SAKNAS!
#SAKNAS!
#SAKNAS!
#SAKNAS!
#SAKNAS!
#SAKNAS!
1573 (0.071)
#SAKNAS!
#SAKNAS!
2395 (0.036)
2395 (0.036)
#SAKNAS!
#SAKNAS!
#SAKNAS!
#SAKNAS!
#SAKNAS!
#SAKNAS!
#SAKNAS!
#SAKNAS!
#SAKNAS!
#SAKNAS!
#SAKNAS!

#SAKNAS!
2395 (0.036)
#SAKNAS!
2395 (0.036)
2395 (0.036)
#SAKNAS!
#SAKNAS!
2395 (0.036)
#SAKNAS!
#SAKNAS!
2395 (0.036)
#SAKNAS!
#SAKNAS!
#SAKNAS!
#SAKNAS!
2395 (0.036)
2395 (0.036)
1164 (0.11)
#SAKNAS!
#SAKNAS!
#SAKNAS!
#SAKNAS!
#SAKNAS!
2395 (0.036)
2395 (0.036)
#SAKNAS!
#SAKNAS!
#SAKNAS!
#SAKNAS!
2395 (0.036)
#SAKNAS!
#SAKNAS!
#SAKNAS!
#SAKNAS!
#SAKNAS!
#SAKNAS!
#SAKNAS!
#SAKNAS!
2395 (0.036)
#SAKNAS!
#SAKNAS!
#SAKNAS!
2395 (0.036)

2395 (0.036)
2395 (0.036)
#SAKNAS!
2395 (0.036)
#SAKNAS!
#SAKNAS!
#SAKNAS!
2395 (0.036)
2395 (0.036)
2395 (0.036)
#SAKNAS!
#SAKNAS!
#SAKNAS!
2395 (0.036)
#SAKNAS!
1164 (0.11)
#SAKNAS!
#SAKNAS!
#SAKNAS!
#SAKNAS!
#SAKNAS!
#SAKNAS!
2395 (0.036)
#SAKNAS!
#SAKNAS!
#SAKNAS!
2395 (0.036)
2395 (0.036)
936 (0.14)
2395 (0.036)
#SAKNAS!
#SAKNAS!
#SAKNAS!
#SAKNAS!
#SAKNAS!
#SAKNAS!
#SAKNAS!
#SAKNAS!
#SAKNAS!
#SAKNAS!
2395 (0.036)
#SAKNAS!
#SAKNAS!

#SAKNAS!
#SAKNAS!
#SAKNAS!
#SAKNAS!
1573 (0.071)
#SAKNAS!
2395 (0.036)
#SAKNAS!
773 (0.18)
2395 (0.036)
#SAKNAS!
561 (0.25)
#SAKNAS!
#SAKNAS!
#SAKNAS!
#SAKNAS!
2395 (0.036)
#SAKNAS!
#SAKNAS!
#SAKNAS!
2395 (0.036)
#SAKNAS!
2395 (0.036)
#SAKNAS!
#SAKNAS!
#SAKNAS!
2395 (0.036)
#SAKNAS!
#SAKNAS!
#SAKNAS!
#SAKNAS!
2395 (0.036)
2395 (0.036)
2395 (0.036)
#SAKNAS!
2395 (0.036)
#SAKNAS!
#SAKNAS!
#SAKNAS!
#SAKNAS!
#SAKNAS!
#SAKNAS!
#SAKNAS!

#SAKNAS!
#SAKNAS!
2395 (0.036)
#SAKNAS!
#SAKNAS!
2395 (0.036)
2395 (0.036)
#SAKNAS!
#SAKNAS!
#SAKNAS!
#SAKNAS!
#SAKNAS!
#SAKNAS!
#SAKNAS!
#SAKNAS!
#SAKNAS!
#SAKNAS!
#SAKNAS!
#SAKNAS!
#SAKNAS!
#SAKNAS!
#SAKNAS!
#SAKNAS!
2395 (0.036)
2395 (0.036)
2395 (0.036)
2395 (0.036)
#SAKNAS!
2395 (0.036)
2395 (0.036)
1164 (0.11)
2395 (0.036)
2395 (0.036)
#SAKNAS!
#SAKNAS!
#SAKNAS!
#SAKNAS!
#SAKNAS!
2395 (0.036)
2395 (0.036)
#SAKNAS!
#SAKNAS!
#SAKNAS!

#SAKNAS!
2395 (0.036)
#SAKNAS!
2395 (0.036)
#SAKNAS!
#SAKNAS!
2395 (0.036)
#SAKNAS!
2395 (0.036)
2395 (0.036)
#SAKNAS!
#SAKNAS!
#SAKNAS!
#SAKNAS!
#SAKNAS!
#SAKNAS!
2395 (0.036)
2395 (0.036)
#SAKNAS!
#SAKNAS!
2395 (0.036)
#SAKNAS!
#SAKNAS!
#SAKNAS!
#SAKNAS!
#SAKNAS!
2395 (0.036)
#SAKNAS!
#SAKNAS!
#SAKNAS!
#SAKNAS!
#SAKNAS!
#SAKNAS!
2395 (0.036)
#SAKNAS!
1573 (0.071)
#SAKNAS!
2395 (0.036)
#SAKNAS!
#SAKNAS!
#SAKNAS!
#SAKNAS!
#SAKNAS!

#SAKNAS!
#SAKNAS!
#SAKNAS!
#SAKNAS!
#SAKNAS!
1573 (0.071)
2395 (0.036)
#SAKNAS!
#SAKNAS!
#SAKNAS!
#SAKNAS!
#SAKNAS!
1573 (0.071)
#SAKNAS!
#SAKNAS!
#SAKNAS!
#SAKNAS!
2395 (0.036)
#SAKNAS!
#SAKNAS!
#SAKNAS!
2395 (0.036)
#SAKNAS!
#SAKNAS!
#SAKNAS!
1573 (0.071)
#SAKNAS!
#SAKNAS!
#SAKNAS!
#SAKNAS!
#SAKNAS!
#SAKNAS!
#SAKNAS!
#SAKNAS!
1573 (0.071)
2395 (0.036)
#SAKNAS!
#SAKNAS!
2395 (0.036)
#SAKNAS!
#SAKNAS!
319 (0.46)
#SAKNAS!

2395 (0.036)
2395 (0.036)
#SAKNAS!
2395 (0.036)
#SAKNAS!
#SAKNAS!
936 (0.14)
#SAKNAS!
#SAKNAS!
#SAKNAS!
#SAKNAS!
2395 (0.036)
#SAKNAS!
#SAKNAS!
2395 (0.036)
#SAKNAS!
#SAKNAS!
#SAKNAS!
#SAKNAS!
#SAKNAS!
2395 (0.036)
#SAKNAS!
#SAKNAS!
2395 (0.036)
#SAKNAS!
#SAKNAS!
#SAKNAS!
#SAKNAS!
2395 (0.036)
2395 (0.036)
#SAKNAS!
2395 (0.036)
#SAKNAS!
#SAKNAS!
#SAKNAS!
#SAKNAS!
#SAKNAS!
#SAKNAS!
#SAKNAS!
#SAKNAS!
#SAKNAS!
#SAKNAS!
#SAKNAS!

2395 (0.036)
#SAKNAS!
#SAKNAS!
2395 (0.036)
#SAKNAS!
#SAKNAS!
2395 (0.036)
#SAKNAS!
#SAKNAS!
#SAKNAS!
#SAKNAS!
#SAKNAS!
#SAKNAS!
#SAKNAS!
#SAKNAS!
#SAKNAS!
#SAKNAS!
#SAKNAS!
#SAKNAS!
#SAKNAS!
1573 (0.071)
2395 (0.036)
#SAKNAS!
#SAKNAS!
2395 (0.036)
1164 (0.11)
#SAKNAS!
#SAKNAS!
2395 (0.036)
2395 (0.036)
#SAKNAS!
#SAKNAS!
#SAKNAS!
2395 (0.036)
#SAKNAS!
#SAKNAS!
#SAKNAS!
2395 (0.036)
2395 (0.036)
#SAKNAS!
#SAKNAS!
#SAKNAS!
#SAKNAS!

#SAKNAS!
#SAKNAS!
#SAKNAS!
#SAKNAS!
561 (0.25)
#SAKNAS!
2395 (0.036)
#SAKNAS!
#SAKNAS!
#SAKNAS!
#SAKNAS!
#SAKNAS!
#SAKNAS!
#SAKNAS!
2395 (0.036)
#SAKNAS!
#SAKNAS!
2395 (0.036)
1164 (0.11)
#SAKNAS!
#SAKNAS!
#SAKNAS!
2395 (0.036)
#SAKNAS!
#SAKNAS!
#SAKNAS!
#SAKNAS!
2395 (0.036)
2395 (0.036)
#SAKNAS!
#SAKNAS!
#SAKNAS!
#SAKNAS!
1573 (0.071)
1573 (0.071)
2395 (0.036)
2395 (0.036)
2395 (0.036)
#SAKNAS!
#SAKNAS!
2395 (0.036)
#SAKNAS!
#SAKNAS!

#SAKNAS!
2395 (0.036)
2395 (0.036)
2395 (0.036)
#SAKNAS!
#SAKNAS!
#SAKNAS!
#SAKNAS!
2395 (0.036)
2395 (0.036)
#SAKNAS!
#SAKNAS!
2395 (0.036)
2395 (0.036)
#SAKNAS!
#SAKNAS!
2395 (0.036)
#SAKNAS!
446 (0.32)
2395 (0.036)
#SAKNAS!
2395 (0.036)
2395 (0.036)
2395 (0.036)
#SAKNAS!
2395 (0.036)
#SAKNAS!
#SAKNAS!
#SAKNAS!
#SAKNAS!
2395 (0.036)
2395 (0.036)
#SAKNAS!
#SAKNAS!
#SAKNAS!
#SAKNAS!
#SAKNAS!
#SAKNAS!
#SAKNAS!
#SAKNAS!
2395 (0.036)
#SAKNAS!
2395 (0.036)

#SAKNAS!
#SAKNAS!
1573 (0.071)
1573 (0.071)
#SAKNAS!
2395 (0.036)
2395 (0.036)
1573 (0.071)
#SAKNAS!
#SAKNAS!
#SAKNAS!
2395 (0.036)
#SAKNAS!
#SAKNAS!
#SAKNAS!
2395 (0.036)
2395 (0.036)
2395 (0.036)
2395 (0.036)
#SAKNAS!
#SAKNAS!
#SAKNAS!
#SAKNAS!
#SAKNAS!
#SAKNAS!
#SAKNAS!
#SAKNAS!
#SAKNAS!
936 (0.14)
#SAKNAS!
#SAKNAS!
#SAKNAS!
#SAKNAS!
#SAKNAS!
#SAKNAS!
2395 (0.036)
#SAKNAS!
2395 (0.036)
#SAKNAS!
#SAKNAS!
2395 (0.036)
#SAKNAS!
2395 (0.036)

#SAKNAS!
#SAKNAS!
2395 (0.036)
#SAKNAS!
#SAKNAS!
#SAKNAS!
2395 (0.036)
#SAKNAS!
#SAKNAS!
#SAKNAS!
#SAKNAS!
2395 (0.036)
#SAKNAS!
#SAKNAS!
2395 (0.036)
1573 (0.071)
2395 (0.036)
#SAKNAS!
#SAKNAS!
#SAKNAS!
#SAKNAS!
#SAKNAS!
#SAKNAS!
#SAKNAS!
#SAKNAS!
#SAKNAS!
1573 (0.071)
2395 (0.036)
#SAKNAS!
#SAKNAS!
#SAKNAS!
#SAKNAS!
2395 (0.036)
#SAKNAS!
#SAKNAS!
2395 (0.036)
#SAKNAS!
#SAKNAS!
#SAKNAS!
#SAKNAS!
#SAKNAS!
2395 (0.036)
2395 (0.036)

2395 (0.036)
#SAKNAS!
2395 (0.036)
#SAKNAS!
1573 (0.071)
#SAKNAS!
#SAKNAS!
#SAKNAS!
#SAKNAS!
#SAKNAS!
2395 (0.036)
#SAKNAS!
#SAKNAS!
#SAKNAS!
2395 (0.036)
#SAKNAS!
#SAKNAS!
2395 (0.036)
#SAKNAS!
#SAKNAS!
#SAKNAS!
#SAKNAS!
#SAKNAS!
#SAKNAS!
#SAKNAS!
2395 (0.036)
#SAKNAS!
#SAKNAS!
2395 (0.036)
#SAKNAS!
#SAKNAS!
2395 (0.036)
#SAKNAS!
2395 (0.036)
#SAKNAS!
#SAKNAS!
#SAKNAS!
#SAKNAS!
#SAKNAS!
2395 (0.036)
2395 (0.036)
#SAKNAS!
#SAKNAS!

2395 (0.036)
#SAKNAS!
2395 (0.036)
2395 (0.036)
2395 (0.036)
2395 (0.036)
2395 (0.036)
#SAKNAS!
#SAKNAS!
2395 (0.036)
2395 (0.036)
2395 (0.036)
#SAKNAS!
1573 (0.071)
#SAKNAS!
#SAKNAS!
#SAKNAS!
#SAKNAS!
#SAKNAS!
#SAKNAS!
#SAKNAS!
2395 (0.036)
#SAKNAS!
#SAKNAS!
2395 (0.036)
2395 (0.036)
#SAKNAS!
#SAKNAS!
#SAKNAS!
2395 (0.036)
2395 (0.036)
#SAKNAS!
#SAKNAS!
#SAKNAS!
#SAKNAS!
#SAKNAS!
#SAKNAS!
2395 (0.036)
2395 (0.036)
1573 (0.071)
#SAKNAS!
1164 (0.11)
#SAKNAS!

#SAKNAS!
#SAKNAS!
#SAKNAS!
2395 (0.036)
#SAKNAS!
#SAKNAS!
#SAKNAS!
#SAKNAS!
#SAKNAS!
#SAKNAS!
#SAKNAS!
#SAKNAS!
2395 (0.036)
#SAKNAS!
#SAKNAS!
#SAKNAS!
#SAKNAS!
#SAKNAS!
#SAKNAS!
#SAKNAS!
#SAKNAS!
#SAKNAS!
#SAKNAS!
#SAKNAS!
#SAKNAS!
#SAKNAS!
341 (0.43)
#SAKNAS!
#SAKNAS!
#SAKNAS!
#SAKNAS!
2395 (0.036)
2395 (0.036)
#SAKNAS!
#SAKNAS!
#SAKNAS!
#SAKNAS!
#SAKNAS!
#SAKNAS!
#SAKNAS!
#SAKNAS!
#SAKNAS!
#SAKNAS!

#SAKNAS!
1573 (0.071)
#SAKNAS!
#SAKNAS!
#SAKNAS!
#SAKNAS!
2395 (0.036)
2395 (0.036)
#SAKNAS!
#SAKNAS!
#SAKNAS!
2395 (0.036)
#SAKNAS!
#SAKNAS!
#SAKNAS!
2395 (0.036)
#SAKNAS!
#SAKNAS!
2395 (0.036)
#SAKNAS!
#SAKNAS!
#SAKNAS!
2395 (0.036)
#SAKNAS!
2395 (0.036)
2395 (0.036)
#SAKNAS!
#SAKNAS!
#SAKNAS!
#SAKNAS!
2395 (0.036)
2395 (0.036)
#SAKNAS!
#SAKNAS!
2395 (0.036)
2395 (0.036)
2395 (0.036)
#SAKNAS!
#SAKNAS!
#SAKNAS!
#SAKNAS!
#SAKNAS!
#SAKNAS!

#SAKNAS!
2395 (0.036)
#SAKNAS!
#SAKNAS!
2395 (0.036)
2395 (0.036)
1573 (0.071)
#SAKNAS!
#SAKNAS!
#SAKNAS!
2395 (0.036)
#SAKNAS!
#SAKNAS!
#SAKNAS!
#SAKNAS!
#SAKNAS!
#SAKNAS!
#SAKNAS!
2395 (0.036)
#SAKNAS!
#SAKNAS!
2395 (0.036)
#SAKNAS!
#SAKNAS!
#SAKNAS!
#SAKNAS!
#SAKNAS!
#SAKNAS!
#SAKNAS!
#SAKNAS!
2395 (0.036)
#SAKNAS!
#SAKNAS!
#SAKNAS!
#SAKNAS!
#SAKNAS!
#SAKNAS!
#SAKNAS!
#SAKNAS!
#SAKNAS!
2395 (0.036)
#SAKNAS!
#SAKNAS!

2395 (0.036)
#SAKNAS!
#SAKNAS!
2395 (0.036)
#SAKNAS!
#SAKNAS!
#SAKNAS!
#SAKNAS!
#SAKNAS!
2395 (0.036)
2395 (0.036)
#SAKNAS!
2395 (0.036)
2395 (0.036)
#SAKNAS!
#SAKNAS!
1573 (0.071)
#SAKNAS!
2395 (0.036)
2395 (0.036)
2395 (0.036)
#SAKNAS!
2395 (0.036)
#SAKNAS!
#SAKNAS!
#SAKNAS!
#SAKNAS!
#SAKNAS!
#SAKNAS!
2395 (0.036)
2395 (0.036)
#SAKNAS!
#SAKNAS!
#SAKNAS!
#SAKNAS!
#SAKNAS!
2395 (0.036)
#SAKNAS!
2395 (0.036)
#SAKNAS!
#SAKNAS!
#SAKNAS!
2395 (0.036)

1573 (0.071)
561 (0.25)
#SAKNAS!
#SAKNAS!
#SAKNAS!
#SAKNAS!
#SAKNAS!
#SAKNAS!
2395 (0.036)
2395 (0.036)
#SAKNAS!
#SAKNAS!
#SAKNAS!
#SAKNAS!
2395 (0.036)
#SAKNAS!
#SAKNAS!
2395 (0.036)
2395 (0.036)
#SAKNAS!
#SAKNAS!
2395 (0.036)
#SAKNAS!
2395 (0.036)
2395 (0.036)
#SAKNAS!
2395 (0.036)
#SAKNAS!
2395 (0.036)
#SAKNAS!
#SAKNAS!
#SAKNAS!
#SAKNAS!
#SAKNAS!
#SAKNAS!
#SAKNAS!
#SAKNAS!
#SAKNAS!
#SAKNAS!
#SAKNAS!
#SAKNAS!
#SAKNAS!
#SAKNAS!

#SAKNAS!
773 (0.18)
2395 (0.036)
2395 (0.036)
2395 (0.036)
2395 (0.036)
2395 (0.036)
2395 (0.036)
2395 (0.036)
2395 (0.036)
#SAKNAS!
#SAKNAS!
2395 (0.036)
2395 (0.036)
#SAKNAS!
2395 (0.036)
#SAKNAS!
#SAKNAS!
#SAKNAS!
#SAKNAS!
#SAKNAS!
2395 (0.036)
1573 (0.071)
2395 (0.036)
2395 (0.036)
2395 (0.036)
2395 (0.036)
#SAKNAS!
#SAKNAS!
#SAKNAS!
#SAKNAS!
#SAKNAS!
2395 (0.036)
2395 (0.036)
#SAKNAS!
#SAKNAS!
#SAKNAS!
#SAKNAS!
#SAKNAS!
2395 (0.036)
2395 (0.036)
#SAKNAS!
#SAKNAS!

#SAKNAS!
#SAKNAS!
#SAKNAS!
2395 (0.036)
#SAKNAS!
2395 (0.036)
#SAKNAS!
#SAKNAS!
#SAKNAS!
#SAKNAS!
#SAKNAS!
#SAKNAS!
#SAKNAS!
#SAKNAS!
#SAKNAS!
#SAKNAS!
#SAKNAS!
2395 (0.036)
#SAKNAS!
#SAKNAS!
#SAKNAS!
#SAKNAS!
#SAKNAS!
#SAKNAS!
#SAKNAS!
2395 (0.036)
#SAKNAS!
#SAKNAS!
#SAKNAS!
2395 (0.036)
1573 (0.071)
#SAKNAS!
#SAKNAS!
#SAKNAS!
2395 (0.036)
#SAKNAS!
#SAKNAS!
#SAKNAS!
#SAKNAS!
2395 (0.036)
#SAKNAS!
#SAKNAS!
2395 (0.036)

#SAKNAS!
2395 (0.036)
648 (0.21)
2395 (0.036)
#SAKNAS!
#SAKNAS!
#SAKNAS!
#SAKNAS!
#SAKNAS!
#SAKNAS!
#SAKNAS!
#SAKNAS!
#SAKNAS!
2395 (0.036)
2395 (0.036)
#SAKNAS!
#SAKNAS!
#SAKNAS!
#SAKNAS!
#SAKNAS!
#SAKNAS!
#SAKNAS!
#SAKNAS!
#SAKNAS!
#SAKNAS!
2395 (0.036)
2395 (0.036)
#SAKNAS!
#SAKNAS!
#SAKNAS!
#SAKNAS!
#SAKNAS!
#SAKNAS!
#SAKNAS!
2395 (0.036)
#SAKNAS!
#SAKNAS!
#SAKNAS!
#SAKNAS!
#SAKNAS!
#SAKNAS!
#SAKNAS!
#SAKNAS!

2395 (0.036)
#SAKNAS!
#SAKNAS!
#SAKNAS!
#SAKNAS!
#SAKNAS!
#SAKNAS!
#SAKNAS!
#SAKNAS!
2395 (0.036)
#SAKNAS!
2395 (0.036)
#SAKNAS!
#SAKNAS!
#SAKNAS!
#SAKNAS!
#SAKNAS!
#SAKNAS!
2395 (0.036)
2395 (0.036)
2395 (0.036)
#SAKNAS!
#SAKNAS!
#SAKNAS!
#SAKNAS!
2395 (0.036)
#SAKNAS!
#SAKNAS!
#SAKNAS!
2395 (0.036)
#SAKNAS!
#SAKNAS!
2395 (0.036)
2395 (0.036)
#SAKNAS!
#SAKNAS!
#SAKNAS!
2395 (0.036)
2395 (0.036)
#SAKNAS!
#SAKNAS!
#SAKNAS!
#SAKNAS!

#SAKNAS!
2395 (0.036)
2395 (0.036)
2395 (0.036)
#SAKNAS!
#SAKNAS!
#SAKNAS!
#SAKNAS!
#SAKNAS!
#SAKNAS!
#SAKNAS!
#SAKNAS!
#SAKNAS!
2395 (0.036)
2395 (0.036)
1573 (0.071)
2395 (0.036)
2395 (0.036)
#SAKNAS!
#SAKNAS!
#SAKNAS!
#SAKNAS!
#SAKNAS!
#SAKNAS!
#SAKNAS!
2395 (0.036)
2395 (0.036)
2395 (0.036)
2395 (0.036)
#SAKNAS!
#SAKNAS!
1573 (0.071)
2395 (0.036)
#SAKNAS!
#SAKNAS!
#SAKNAS!
#SAKNAS!
#SAKNAS!
#SAKNAS!
#SAKNAS!
2395 (0.036)
2395 (0.036)
#SAKNAS!

#SAKNAS!
#SAKNAS!
#SAKNAS!
2395 (0.036)
#SAKNAS!
#SAKNAS!
#SAKNAS!
#SAKNAS!
2395 (0.036)
#SAKNAS!
#SAKNAS!
2395 (0.036)
#SAKNAS!
#SAKNAS!
#SAKNAS!
#SAKNAS!
2395 (0.036)
#SAKNAS!
#SAKNAS!
#SAKNAS!
#SAKNAS!
#SAKNAS!
#SAKNAS!
#SAKNAS!
2395 (0.036)
#SAKNAS!
#SAKNAS!
#SAKNAS!
#SAKNAS!
#SAKNAS!
#SAKNAS!
#SAKNAS!
#SAKNAS!
2395 (0.036)
#SAKNAS!
2395 (0.036)
#SAKNAS!
2395 (0.036)
2395 (0.036)
#SAKNAS!
#SAKNAS!
2395 (0.036)
2395 (0.036)

#SAKNAS!
#SAKNAS!
#SAKNAS!
#SAKNAS!
#SAKNAS!
#SAKNAS!
2395 (0.036)
#SAKNAS!
#SAKNAS!
2395 (0.036)
#SAKNAS!
#SAKNAS!
2395 (0.036)
#SAKNAS!
#SAKNAS!
#SAKNAS!
#SAKNAS!
#SAKNAS!
#SAKNAS!
#SAKNAS!
2395 (0.036)
936 (0.14)
2395 (0.036)
#SAKNAS!
#SAKNAS!
2395 (0.036)
#SAKNAS!
#SAKNAS!
#SAKNAS!
#SAKNAS!
#SAKNAS!
#SAKNAS!
2395 (0.036)
2395 (0.036)
2395 (0.036)
#SAKNAS!
#SAKNAS!
#SAKNAS!
2395 (0.036)
936 (0.14)
2395 (0.036)
#SAKNAS!
#SAKNAS!

#SAKNAS!
#SAKNAS!
#SAKNAS!
2395 (0.036)
2395 (0.036)
#SAKNAS!
2395 (0.036)
#SAKNAS!
#SAKNAS!
1573 (0.071)
#SAKNAS!
#SAKNAS!
#SAKNAS!
#SAKNAS!
2395 (0.036)
#SAKNAS!
#SAKNAS!
#SAKNAS!
#SAKNAS!
#SAKNAS!
2395 (0.036)
2395 (0.036)
#SAKNAS!
#SAKNAS!
#SAKNAS!
2395 (0.036)
2395 (0.036)
2395 (0.036)
#SAKNAS!
#SAKNAS!
#SAKNAS!
1164 (0.11)
2395 (0.036)
#SAKNAS!
#SAKNAS!
#SAKNAS!
#SAKNAS!
2395 (0.036)
#SAKNAS!
2395 (0.036)
#SAKNAS!
#SAKNAS!
#SAKNAS!

#SAKNAS!
#SAKNAS!
2395 (0.036)
2395 (0.036)
#SAKNAS!
#SAKNAS!
#SAKNAS!
#SAKNAS!
#SAKNAS!
#SAKNAS!
#SAKNAS!
#SAKNAS!
#SAKNAS!
#SAKNAS!
#SAKNAS!
#SAKNAS!
#SAKNAS!
2395 (0.036)
2395 (0.036)
2395 (0.036)
#SAKNAS!
#SAKNAS!
2395 (0.036)
#SAKNAS!
#SAKNAS!
#SAKNAS!
#SAKNAS!
2395 (0.036)
#SAKNAS!
#SAKNAS!
#SAKNAS!
#SAKNAS!
#SAKNAS!
#SAKNAS!
#SAKNAS!
#SAKNAS!
2395 (0.036)
2395 (0.036)
#SAKNAS!
#SAKNAS!
#SAKNAS!
#SAKNAS!
#SAKNAS!

#SAKNAS!
#SAKNAS!
#SAKNAS!
#SAKNAS!
1573 (0.071)
#SAKNAS!
#SAKNAS!
#SAKNAS!
#SAKNAS!
#SAKNAS!
1573 (0.071)
2395 (0.036)
#SAKNAS!
#SAKNAS!
#SAKNAS!
#SAKNAS!
#SAKNAS!
#SAKNAS!
#SAKNAS!
#SAKNAS!
#SAKNAS!
#SAKNAS!
#SAKNAS!
#SAKNAS!
#SAKNAS!
#SAKNAS!
#SAKNAS!
#SAKNAS!
2395 (0.036)
#SAKNAS!
#SAKNAS!
#SAKNAS!
#SAKNAS!
561 (0.25)
#SAKNAS!
2395 (0.036)
#SAKNAS!
#SAKNAS!
2395 (0.036)
1164 (0.11)
#SAKNAS!
#SAKNAS!
#SAKNAS!

#SAKNAS!
#SAKNAS!
#SAKNAS!
2395 (0.036)
#SAKNAS!
#SAKNAS!
773 (0.18)
2395 (0.036)
2395 (0.036)
#SAKNAS!
#SAKNAS!
#SAKNAS!
#SAKNAS!
#SAKNAS!
#SAKNAS!
2395 (0.036)
#SAKNAS!
2395 (0.036)
#SAKNAS!
1164 (0.11)
2395 (0.036)
#SAKNAS!
#SAKNAS!
#SAKNAS!
2395 (0.036)
#SAKNAS!
#SAKNAS!
#SAKNAS!
#SAKNAS!
#SAKNAS!
#SAKNAS!
2395 (0.036)
#SAKNAS!
#SAKNAS!
#SAKNAS!
#SAKNAS!
#SAKNAS!
2395 (0.036)
#SAKNAS!
#SAKNAS!
#SAKNAS!
#SAKNAS!
1573 (0.071)

#SAKNAS!
#SAKNAS!
#SAKNAS!
2395 (0.036)
#SAKNAS!
#SAKNAS!
#SAKNAS!
#SAKNAS!
#SAKNAS!
936 (0.14)
#SAKNAS!
#SAKNAS!
#SAKNAS!
#SAKNAS!
#SAKNAS!
#SAKNAS!
2395 (0.036)
#SAKNAS!
#SAKNAS!
1573 (0.071)
1573 (0.071)
#SAKNAS!
#SAKNAS!
#SAKNAS!
#SAKNAS!
2395 (0.036)
2395 (0.036)
#SAKNAS!
#SAKNAS!
2395 (0.036)
2395 (0.036)
#SAKNAS!
#SAKNAS!
#SAKNAS!
#SAKNAS!
#SAKNAS!
#SAKNAS!
#SAKNAS!
#SAKNAS!
2395 (0.036)
2395 (0.036)
#SAKNAS!
#SAKNAS!

#SAKNAS!
2395 (0.036)
#SAKNAS!
#SAKNAS!
#SAKNAS!
2395 (0.036)
#SAKNAS!
#SAKNAS!
#SAKNAS!
2395 (0.036)
#SAKNAS!
#SAKNAS!
#SAKNAS!
#SAKNAS!
648 (0.21)
648 (0.21)
2395 (0.036)
#SAKNAS!
#SAKNAS!
#SAKNAS!
#SAKNAS!
#SAKNAS!
#SAKNAS!
#SAKNAS!
#SAKNAS!
#SAKNAS!
#SAKNAS!
2395 (0.036)
2395 (0.036)
2395 (0.036)
1573 (0.071)
#SAKNAS!
2395 (0.036)
#SAKNAS!
#SAKNAS!
2395 (0.036)
#SAKNAS!
#SAKNAS!
#SAKNAS!
#SAKNAS!
#SAKNAS!
#SAKNAS!
#SAKNAS!

2395 (0.036)
#SAKNAS!
#SAKNAS!
#SAKNAS!
#SAKNAS!
2395 (0.036)
#SAKNAS!
#SAKNAS!
#SAKNAS!
#SAKNAS!
#SAKNAS!
#SAKNAS!
#SAKNAS!
#SAKNAS!
#SAKNAS!
#SAKNAS!
#SAKNAS!
#SAKNAS!
#SAKNAS!
#SAKNAS!
1573 (0.071)
2395 (0.036)
#SAKNAS!
#SAKNAS!
#SAKNAS!
#SAKNAS!
#SAKNAS!
#SAKNAS!
#SAKNAS!
#SAKNAS!
2395 (0.036)
2395 (0.036)
#SAKNAS!
#SAKNAS!
2395 (0.036)
2395 (0.036)
2395 (0.036)
2395 (0.036)
#SAKNAS!
#SAKNAS!
#SAKNAS!
#SAKNAS!
#SAKNAS!

#SAKNAS!
#SAKNAS!
#SAKNAS!
2395 (0.036)
#SAKNAS!
#SAKNAS!
#SAKNAS!
2395 (0.036)
2395 (0.036)
#SAKNAS!
#SAKNAS!
#SAKNAS!
#SAKNAS!
#SAKNAS!
#SAKNAS!
2395 (0.036)
2395 (0.036)
2395 (0.036)
#SAKNAS!
#SAKNAS!
#SAKNAS!
#SAKNAS!
#SAKNAS!
2395 (0.036)
2395 (0.036)
#SAKNAS!
#SAKNAS!
2395 (0.036)
2395 (0.036)
#SAKNAS!
2395 (0.036)
#SAKNAS!
2395 (0.036)
1573 (0.071)
#SAKNAS!
#SAKNAS!
#SAKNAS!
#SAKNAS!
#SAKNAS!
#SAKNAS!
#SAKNAS!
#SAKNAS!
#SAKNAS!

#SAKNAS!
#SAKNAS!
#SAKNAS!
#SAKNAS!
#SAKNAS!
2395 (0.036)
#SAKNAS!
#SAKNAS!
#SAKNAS!
#SAKNAS!
#SAKNAS!
2395 (0.036)
#SAKNAS!
#SAKNAS!
#SAKNAS!
#SAKNAS!
#SAKNAS!
#SAKNAS!
#SAKNAS!
#SAKNAS!
#SAKNAS!
2395 (0.036)
#SAKNAS!
#SAKNAS!
2395 (0.036)
#SAKNAS!
#SAKNAS!
#SAKNAS!
#SAKNAS!
#SAKNAS!
#SAKNAS!
#SAKNAS!
#SAKNAS!
#SAKNAS!
#SAKNAS!
#SAKNAS!
2395 (0.036)
#SAKNAS!
#SAKNAS!
#SAKNAS!
#SAKNAS!
#SAKNAS!
#SAKNAS!

#SAKNAS!
#SAKNAS!
1573 (0.071)
2395 (0.036)
2395 (0.036)
#SAKNAS!
#SAKNAS!
#SAKNAS!
#SAKNAS!
#SAKNAS!
#SAKNAS!
#SAKNAS!
2395 (0.036)
#SAKNAS!
#SAKNAS!
#SAKNAS!
#SAKNAS!
#SAKNAS!

Supplementary material 3 - *Part 2 - Words in title without search keywords* . Full cleaned datase
analysis. Data cleaning and standardization were performed in Microsoft Excel 365 using commo
bibliometric data-processing procedures, including text standardization, duplicate handling, sorti
functions, citation ranking, lookup utilities, and pivot table analyses

Words in title (without search keywords)	TP	91-24 R (%)	91-13 R (%)	14-20 R (%)
patients	1188	1 (14)	1 (11)	1 (14)
association	775	2 (8.9)	3 (4.5)	3 (9.1)
children	769	3 (8.8)	2 (8.8)	2 (10)
adults	613	4 (7)	6 (3.6)	4 (7.5)
older	476	5 (5.4)	12 (2.7)	5 (5.5)
factors	442	6 (5.1)	7 (3.4)	6 (5.2)
cross-sectional	415	7 (4.7)	237 (0.45)	10 (4.1)
cancer	394	8 (4.5)	4 (4.1)	7 (4.7)
obesity	321	9 (3.7)	5 (3.7)	9 (4.4)
care	313	10 (3.6)	14 (2.5)	11 (4)
survey	306	11 (3.5)	31 (1.9)	8 (4.5)
quality	296	12 (3.4)	12 (2.7)	13 (3.6)
relationship	279	13 (3.2)	8 (3.3)	17 (3.1)
national	264	14 (3)	36 (1.8)	12 (4)
elderly	248	15 (2.8)	10 (2.8)	14 (3.3)
life	240	16 (2.7)	40 (1.7)	18 (3.1)
clinical	239	17 (2.7)	23 (2.1)	22 (2.8)
prevalence	238	18 (2.7)	20 (2.1)	14 (3.3)
cohort	225	19 (2.6)	102 (0.83)	19 (3)
impact	224	20 (2.6)	33 (1.9)	21 (2.9)
treatment	216	21 (2.5)	9 (2.9)	23 (2.7)
eating	210	22 (2.4)	29 (2)	23 (2.7)
outcomes	202	23 (2.3)	48 (1.4)	27 (2.4)
examination	200	24 (2.3)	66 (1.1)	16 (3.1)
weight	200	24 (2.3)	20 (2.1)	25 (2.5)
population	198	26 (2.3)	17 (2.2)	33 (2.2)
esophageal	190	27 (2.2)	17 (2.2)	28 (2.4)
levels	187	28 (2.1)	23 (2.1)	39 (2)
stroke	186	29 (2.1)	25 (2)	44 (1.9)
neck	184	30 (2.1)	27 (2)	35 (2.1)
trial	183	31 (2.1)	72 (1.1)	26 (2.5)
women	183	31 (2.1)	16 (2.4)	44 (1.9)
gastrostomy	182	33 (2.1)	11 (2.7)	35 (2.1)
head	181	34 (2.1)	27 (2)	31 (2.2)
assessment	177	35 (2)	29 (2)	42 (1.9)
management	177	35 (2)	25 (2)	33 (2.2)
diabetes	175	37 (2)	36 (1.8)	35 (2.1)

endoscopic	174	38 (2)	15 (2.5)	30 (2.2)
evaluation	174	38 (2)	55 (1.3)	38 (2.1)
childhood	173	40 (2)	31 (1.9)	40 (1.9)
adolescents	165	41 (1.9)	50 (1.3)	31 (2.2)
chronic	155	42 (1.8)	22 (2.1)	42 (1.9)
nhanes	152	43 (1.7)	149 (0.66)	71 (1.4)
protein	145	44 (1.7)	41 (1.7)	59 (1.5)
consumption	143	45 (1.6)	58 (1.2)	29 (2.3)
korean	143	45 (1.6)	352 (0.31)	19 (3)
therapy	142	47 (1.6)	38 (1.7)	48 (1.7)
us	142	47 (1.6)	60 (1.2)	79 (1.3)
type	140	49 (1.6)	50 (1.3)	48 (1.7)
prospective	138	50 (1.6)	58 (1.2)	51 (1.6)
associations	136	51 (1.6)	113 (0.79)	47 (1.8)
role	136	51 (1.6)	33 (1.9)	99 (1)
surgery	135	53 (1.5)	88 (0.9)	44 (1.9)
long-term	134	54 (1.5)	47 (1.4)	56 (1.6)
influence	132	55 (1.5)	33 (1.9)	61 (1.5)
japanese	132	55 (1.5)	66 (1.1)	40 (1.9)
development	129	57 (1.5)	60 (1.2)	56 (1.6)
percutaneous	129	57 (1.5)	17 (2.2)	68 (1.4)
syndrome	126	59 (1.4)	64 (1.2)	53 (1.6)
acute	125	60 (1.4)	57 (1.3)	56 (1.6)
oropharyngeal	125	60 (1.4)	48 (1.4)	73 (1.4)
gastric	124	62 (1.4)	38 (1.7)	73 (1.4)
young	124	62 (1.4)	44 (1.5)	64 (1.5)
practices	122	64 (1.4)	78 (1)	52 (1.6)
people	119	65 (1.4)	66 (1.1)	73 (1.4)
experience	117	66 (1.3)	42 (1.6)	73 (1.4)
severe	117	66 (1.3)	50 (1.3)	61 (1.5)
age	112	68 (1.3)	66 (1.1)	61 (1.5)
case	112	68 (1.3)	70 (1.1)	82 (1.2)
growth	112	68 (1.3)	42 (1.6)	89 (1.1)
mortality	109	71 (1.2)	130 (0.72)	64 (1.5)
characteristics	108	72 (1.2)	113 (0.79)	64 (1.5)
serum	106	73 (1.2)	50 (1.3)	84 (1.2)
activity	104	74 (1.2)	95 (0.86)	73 (1.4)
child	104	74 (1.2)	64 (1.2)	48 (1.7)
community-dwelling	104	74 (1.2)	170 (0.59)	71 (1.4)
healthy	103	77 (1.2)	80 (0.93)	53 (1.6)
functional	102	78 (1.2)	95 (0.86)	84 (1.2)
home	102	78 (1.2)	77 (1)	64 (1.5)
adult	101	80 (1.2)	80 (0.93)	87 (1.2)

obese	100	81 (1.1)	74 (1)	73 (1.4)
physical	99	82 (1.1)	149 (0.66)	84 (1.2)
retrospective	99	82 (1.1)	260 (0.41)	103 (1)
salivary	98	84 (1.1)	88 (0.9)	79 (1.3)
rehabilitation	96	85 (1.1)	135 (0.69)	95 (1.1)
tube	96	85 (1.1)	60 (1.2)	82 (1.2)
longitudinal	95	87 (1.1)	95 (0.86)	79 (1.3)
model	95	87 (1.1)	113 (0.79)	114 (0.89)
patterns	94	89 (1.1)	80 (0.93)	87 (1.2)
relation	93	90 (1.1)	50 (1.3)	97 (1.1)
individuals	92	91 (1.1)	320 (0.34)	68 (1.4)
intervention	92	91 (1.1)	113 (0.79)	108 (1)
korea	92	91 (1.1)	1457 (0.069)	59 (1.5)
laparoscopic	92	91 (1.1)	46 (1.5)	97 (1.1)
changes	91	95 (1)	95 (0.86)	89 (1.1)
metabolic	90	96 (1)	149 (0.66)	53 (1.6)
enteral	88	97 (1)	70 (1.1)	89 (1.1)
diets	85	98 (1)	88 (0.9)	126 (0.82)
pilot	85	98 (1)	130 (0.72)	89 (1.1)
disorders	84	100 (1)	95 (0.86)	117 (0.86)
overweight	84	100 (1)	123 (0.76)	68 (1.4)
states	84	100 (1)	55 (1.3)	117 (0.86)
systemic	84	100 (1)	135 (0.69)	89 (1.1)
nutrient	83	104 (0.95)	60 (1.2)	95 (1.1)
infants	81	105 (0.93)	72 (1.1)	103 (1)
maternal	81	105 (0.93)	88 (0.9)	89 (1.1)
nursing	80	107 (0.91)	260 (0.41)	99 (1)
report	80	107 (0.91)	102 (0.83)	126 (0.82)
screening	79	109 (0.9)	88 (0.9)	117 (0.86)
sclerosis	78	110 (0.89)	113 (0.79)	108 (1)
blood	77	111 (0.88)	102 (0.83)	170 (0.66)
living	75	112 (0.86)	159 (0.62)	99 (1)
vitamin	75	112 (0.86)	149 (0.66)	151 (0.73)
symptoms	72	114 (0.82)	80 (0.93)	126 (0.82)
cardiovascular	71	115 (0.81)	135 (0.69)	117 (0.86)
hospital	71	115 (0.81)	217 (0.48)	126 (0.82)
conditions	70	117 (0.8)	123 (0.76)	117 (0.86)
patient	70	117 (0.8)	80 (0.93)	170 (0.66)
support	70	117 (0.8)	217 (0.48)	134 (0.79)
composition	69	120 (0.79)	182 (0.55)	110 (0.92)
control	69	120 (0.79)	102 (0.83)	151 (0.73)
does	69	120 (0.79)	74 (1)	134 (0.79)
rats	69	120 (0.79)	44 (1.5)	210 (0.56)

review	69	120 (0.79)	80 (0.93)	210 (0.56)
number	68	125 (0.78)	471 (0.24)	99 (1)
program	68	125 (0.78)	123 (0.76)	117 (0.86)
evidence	67	127 (0.77)	123 (0.76)	170 (0.66)
rural	66	128 (0.75)	95 (0.86)	117 (0.86)
approach	65	129 (0.74)	170 (0.59)	114 (0.89)
cervical	65	129 (0.74)	237 (0.45)	110 (0.92)
china	65	129 (0.74)	352 (0.31)	134 (0.79)
human	65	129 (0.74)	102 (0.83)	141 (0.76)
lateral	65	129 (0.74)	135 (0.69)	114 (0.89)
predictors	64	134 (0.73)	135 (0.69)	210 (0.56)
preschool	64	134 (0.73)	102 (0.83)	160 (0.69)
residents	64	134 (0.73)	260 (0.41)	103 (1)
foods	63	137 (0.72)	149 (0.66)	238 (0.49)
general	63	137 (0.72)	130 (0.72)	170 (0.66)
infant	63	137 (0.72)	102 (0.83)	181 (0.63)
pediatric	63	137 (0.72)	182 (0.55)	151 (0.73)
primary	63	137 (0.72)	88 (0.9)	181 (0.63)
school	63	137 (0.72)	159 (0.62)	141 (0.76)
south	63	137 (0.72)	113 (0.79)	103 (1)
aspiration	62	144 (0.71)	197 (0.52)	141 (0.76)
bone	62	144 (0.71)	113 (0.79)	191 (0.59)
complications	62	144 (0.71)	130 (0.72)	141 (0.76)
undergoing	62	144 (0.71)	320 (0.34)	117 (0.86)
case-control	61	148 (0.7)	217 (0.48)	126 (0.82)
gastrointestinal	61	148 (0.7)	102 (0.83)	126 (0.82)
observational	61	148 (0.7)	844 (0.14)	160 (0.69)
poor	61	148 (0.7)	320 (0.34)	110 (0.92)
sugar	61	148 (0.7)	182 (0.55)	103 (1)
dairy	60	153 (0.69)	182 (0.55)	160 (0.69)
follow-up	60	153 (0.69)	135 (0.69)	191 (0.59)
protocol	60	153 (0.69)	546 (0.21)	160 (0.69)
radiotherapy	60	153 (0.69)	170 (0.59)	151 (0.73)
hygiene	59	157 (0.67)	287 (0.38)	126 (0.82)
implications	59	157 (0.67)	102 (0.83)	151 (0.73)
outcome	59	157 (0.67)	78 (1)	191 (0.59)
amyotrophic	58	160 (0.66)	159 (0.62)	141 (0.76)
brazilian	58	160 (0.66)	149 (0.66)	110 (0.92)
factor	58	160 (0.66)	159 (0.62)	160 (0.69)
pneumonia	58	160 (0.66)	287 (0.38)	151 (0.73)
sleep	58	160 (0.66)	237 (0.45)	126 (0.82)
social	58	160 (0.66)	102 (0.83)	238 (0.49)
community	57	166 (0.65)	197 (0.52)	134 (0.79)

parameters	57	166 (0.65)	170 (0.59)	191 (0.59)
problems	57	166 (0.65)	102 (0.83)	160 (0.69)
subjects	57	166 (0.65)	80 (0.93)	210 (0.56)
system	57	166 (0.65)	135 (0.69)	191 (0.59)
covid-19	56	171 (0.64)	#SAKNAS!	1612 (0.066)
inflammatory	56	171 (0.64)	217 (0.48)	181 (0.63)
mellitus	56	171 (0.64)	130 (0.72)	181 (0.63)
students	56	171 (0.64)	237 (0.45)	222 (0.53)
tongue	56	171 (0.64)	352 (0.31)	141 (0.76)
versus	56	171 (0.64)	237 (0.45)	151 (0.73)
bypass	55	177 (0.63)	197 (0.52)	134 (0.79)
cognitive	55	177 (0.63)	287 (0.38)	141 (0.76)
survival	55	177 (0.63)	159 (0.62)	151 (0.73)
men	54	180 (0.62)	74 (1)	238 (0.49)
prevention	53	181 (0.61)	149 (0.66)	317 (0.4)
knowledge	52	182 (0.59)	320 (0.34)	238 (0.49)
carcinoma	51	183 (0.58)	197 (0.52)	238 (0.49)
determinants	51	183 (0.58)	217 (0.48)	141 (0.76)
exposure	51	183 (0.58)	170 (0.59)	238 (0.49)
cows	50	186 (0.57)	217 (0.48)	160 (0.69)
microbiota	50	186 (0.57)	546 (0.21)	210 (0.56)
placement	50	186 (0.57)	170 (0.59)	134 (0.79)
validation	50	186 (0.57)	320 (0.34)	181 (0.63)
acid	49	190 (0.56)	197 (0.52)	222 (0.53)
dementia	49	190 (0.56)	260 (0.41)	151 (0.73)
group	49	190 (0.56)	123 (0.76)	290 (0.43)
parental	49	190 (0.56)	352 (0.31)	134 (0.79)
pressure	49	190 (0.56)	320 (0.34)	238 (0.49)
appetite	48	195 (0.55)	113 (0.79)	317 (0.4)
birth	48	195 (0.55)	197 (0.52)	210 (0.56)
cerebral	48	195 (0.55)	217 (0.48)	210 (0.56)
heart	48	195 (0.55)	170 (0.59)	263 (0.46)
brazil	47	199 (0.54)	149 (0.66)	263 (0.46)
chinese	47	199 (0.54)	402 (0.28)	141 (0.76)
education	47	199 (0.54)	197 (0.52)	317 (0.4)
medical	47	199 (0.54)	217 (0.48)	263 (0.46)
novel	47	199 (0.54)	352 (0.31)	170 (0.66)
receptor	47	199 (0.54)	182 (0.55)	181 (0.63)
reflux	47	199 (0.54)	170 (0.59)	263 (0.46)
american	46	206 (0.53)	217 (0.48)	263 (0.46)
condition	46	206 (0.53)	287 (0.38)	210 (0.56)
indicators	46	206 (0.53)	260 (0.41)	222 (0.53)
score	46	206 (0.53)	2293 (0.034)	191 (0.59)

severity	46	206 (0.53)	182 (0.55)	416 (0.3)
according	45	211 (0.51)	471 (0.24)	170 (0.66)
dentition	45	211 (0.51)	197 (0.52)	238 (0.49)
foraging	45	211 (0.51)	80 (0.93)	290 (0.43)
geriatric	45	211 (0.51)	170 (0.59)	263 (0.46)
palsy	45	211 (0.51)	287 (0.38)	263 (0.46)
parkinson	45	211 (0.51)	260 (0.41)	222 (0.53)
regulation	45	211 (0.51)	88 (0.9)	317 (0.4)
muscle	44	218 (0.5)	1067 (0.1)	222 (0.53)
profile	44	218 (0.5)	352 (0.31)	238 (0.49)
smoking	44	218 (0.5)	135 (0.69)	210 (0.56)
time	44	218 (0.5)	197 (0.52)	317 (0.4)
central	43	222 (0.49)	123 (0.76)	263 (0.46)
c-reactive	43	222 (0.49)	182 (0.55)	222 (0.53)
fat	43	222 (0.49)	182 (0.55)	191 (0.59)
milk	43	222 (0.49)	287 (0.38)	263 (0.46)
saliva	43	222 (0.49)	352 (0.31)	191 (0.59)
state	43	222 (0.49)	159 (0.62)	222 (0.53)
anterior	42	228 (0.48)	471 (0.24)	191 (0.59)
change	42	228 (0.48)	287 (0.38)	160 (0.69)
correlation	42	228 (0.48)	260 (0.41)	191 (0.59)
daily	42	228 (0.48)	402 (0.28)	191 (0.59)
frequency	42	228 (0.48)	197 (0.52)	222 (0.53)
leptin	42	228 (0.48)	113 (0.79)	238 (0.49)
lifestyle	42	228 (0.48)	546 (0.21)	191 (0.59)
plasma	42	228 (0.48)	135 (0.69)	263 (0.46)
sarcopenia	42	228 (0.48)	#SAKNAS!	317 (0.4)
species	42	228 (0.48)	159 (0.62)	238 (0.49)
surgical	42	228 (0.48)	170 (0.59)	263 (0.46)
water	42	228 (0.48)	260 (0.41)	191 (0.59)
brain	41	240 (0.47)	260 (0.41)	191 (0.59)
cell	41	240 (0.47)	352 (0.31)	263 (0.46)
japan	41	240 (0.47)	402 (0.28)	181 (0.63)
mice	41	240 (0.47)	287 (0.38)	170 (0.66)
multicenter	41	240 (0.47)	844 (0.14)	238 (0.49)
nationwide	41	240 (0.47)	1067 (0.1)	238 (0.49)
parents	41	240 (0.47)	197 (0.52)	290 (0.43)
predictive	41	240 (0.47)	352 (0.31)	263 (0.46)
rate	41	240 (0.47)	135 (0.69)	238 (0.49)
responses	41	240 (0.47)	159 (0.62)	317 (0.4)
socioeconomic	41	240 (0.47)	320 (0.34)	117 (0.86)
stress	41	240 (0.47)	666 (0.17)	141 (0.76)
ability	40	252 (0.46)	402 (0.28)	181 (0.63)

affect	40	252 (0.46)	352 (0.31)	181 (0.63)
assessing	40	252 (0.46)	471 (0.24)	222 (0.53)
comparative	40	252 (0.46)	352 (0.31)	317 (0.4)
male	40	252 (0.46)	217 (0.48)	191 (0.59)
metabolism	40	252 (0.46)	260 (0.41)	317 (0.4)
mothers	40	252 (0.46)	237 (0.45)	181 (0.63)
multiple	40	252 (0.46)	320 (0.34)	377 (0.33)
postoperative	40	252 (0.46)	546 (0.21)	290 (0.43)
preference	40	252 (0.46)	135 (0.69)	377 (0.33)
receiving	40	252 (0.46)	260 (0.41)	344 (0.36)
recovery	40	252 (0.46)	320 (0.34)	344 (0.36)
achalasia	39	264 (0.45)	237 (0.45)	170 (0.66)
incidence	39	264 (0.45)	237 (0.45)	416 (0.3)
meal	39	264 (0.45)	471 (0.24)	210 (0.56)
training	39	264 (0.45)	546 (0.21)	222 (0.53)
disorder	38	268 (0.43)	666 (0.17)	160 (0.69)
drosophila	38	268 (0.43)	471 (0.24)	170 (0.66)
fundoplication	38	268 (0.43)	217 (0.48)	317 (0.4)
markers	38	268 (0.43)	260 (0.41)	222 (0.53)
natural	38	268 (0.43)	260 (0.41)	191 (0.59)
needs	38	268 (0.43)	260 (0.41)	537 (0.23)
old	38	268 (0.43)	182 (0.55)	263 (0.46)
post-stroke	38	268 (0.43)	844 (0.14)	947 (0.13)
reconstruction	38	268 (0.43)	260 (0.41)	344 (0.36)
variation	38	268 (0.43)	135 (0.69)	238 (0.49)
concentrations	37	278 (0.42)	217 (0.48)	191 (0.59)
expression	37	278 (0.42)	182 (0.55)	290 (0.43)
female	37	278 (0.42)	159 (0.62)	377 (0.33)
gastrectomy	37	278 (0.42)	844 (0.14)	191 (0.59)
gene	37	278 (0.42)	237 (0.45)	160 (0.69)
improve	37	278 (0.42)	197 (0.52)	317 (0.4)
pattern	37	278 (0.42)	320 (0.34)	263 (0.46)
preliminary	37	278 (0.42)	237 (0.45)	416 (0.3)
safety	37	278 (0.42)	471 (0.24)	170 (0.66)
sarcopenic	37	278 (0.42)	#SAKNAS!	238 (0.49)
southern	37	278 (0.42)	159 (0.62)	537 (0.23)
taste	37	278 (0.42)	320 (0.34)	222 (0.53)
aging	36	290 (0.41)	287 (0.38)	537 (0.23)
efficacy	36	290 (0.41)	546 (0.21)	290 (0.43)
family	36	290 (0.41)	237 (0.45)	480 (0.26)
gastroesophageal	36	290 (0.41)	197 (0.52)	377 (0.33)
obstructive	36	290 (0.41)	197 (0.52)	263 (0.46)
roux-en-y	36	290 (0.41)	320 (0.34)	238 (0.49)

schoolchildren	36	290 (0.41)	197 (0.52)	377 (0.33)
sea	36	290 (0.41)	113 (0.79)	537 (0.23)
stimulation	36	290 (0.41)	352 (0.31)	191 (0.59)
upper	36	290 (0.41)	260 (0.41)	263 (0.46)
urban	36	290 (0.41)	182 (0.55)	222 (0.53)
behavioral	35	301 (0.4)	287 (0.38)	238 (0.49)
content	35	301 (0.4)	237 (0.45)	344 (0.36)
fatty	35	301 (0.4)	237 (0.45)	416 (0.3)
guidelines	35	301 (0.4)	237 (0.45)	290 (0.43)
microbiome	35	301 (0.4)	1457 (0.069)	317 (0.4)
postmenopausal	35	301 (0.4)	217 (0.48)	238 (0.49)
properties	35	301 (0.4)	1067 (0.1)	222 (0.53)
systematic	35	301 (0.4)	546 (0.21)	377 (0.33)
affects	34	309 (0.39)	135 (0.69)	377 (0.33)
bacterial	34	309 (0.39)	471 (0.24)	222 (0.53)
comprehensive	34	309 (0.39)	471 (0.24)	344 (0.36)
diagnosis	34	309 (0.39)	320 (0.34)	377 (0.33)
failure	34	309 (0.39)	471 (0.24)	317 (0.4)
mouth	34	309 (0.39)	320 (0.34)	416 (0.3)
predicting	34	309 (0.39)	666 (0.17)	238 (0.49)
pregnant	34	309 (0.39)	402 (0.28)	344 (0.36)
reduces	34	309 (0.39)	197 (0.52)	238 (0.49)
strategies	34	309 (0.39)	170 (0.59)	344 (0.36)
tool	34	309 (0.39)	471 (0.24)	170 (0.66)
chemoradiotherapy	33	320 (0.38)	237 (0.45)	263 (0.46)
chemotherapy	33	320 (0.38)	546 (0.21)	290 (0.43)
exploring	33	320 (0.38)	471 (0.24)	537 (0.23)
gain	33	320 (0.38)	471 (0.24)	290 (0.43)
glucose	33	320 (0.38)	287 (0.38)	238 (0.49)
hypothalamic	33	320 (0.38)	149 (0.66)	377 (0.33)
impairment	33	320 (0.38)	546 (0.21)	290 (0.43)
larvae	33	320 (0.38)	95 (0.86)	537 (0.23)
months	33	320 (0.38)	402 (0.28)	344 (0.36)
palliative	33	320 (0.38)	237 (0.45)	344 (0.36)
pregnancy	33	320 (0.38)	182 (0.55)	647 (0.2)
qualitative	33	320 (0.38)	666 (0.17)	317 (0.4)
relationships	33	320 (0.38)	352 (0.31)	317 (0.4)
supplementation	33	320 (0.38)	402 (0.28)	344 (0.36)
value	33	320 (0.38)	320 (0.34)	263 (0.46)
2009-2014	32	335 (0.37)	#SAKNAS!	2492 (0.033)
area	32	335 (0.37)	402 (0.28)	480 (0.26)
common	32	335 (0.37)	471 (0.24)	290 (0.43)
current	32	335 (0.37)	287 (0.38)	290 (0.43)

density	32 335 (0.37)	217 (0.48)	416 (0.3)
fruit	32 335 (0.37)	217 (0.48)	416 (0.3)
long	32 335 (0.37)	546 (0.21)	222 (0.53)
methods	32 335 (0.37)	320 (0.34)	290 (0.43)
research	32 335 (0.37)	352 (0.31)	647 (0.2)
swallow	32 335 (0.37)	260 (0.41)	344 (0.36)
traits	32 335 (0.37)	666 (0.17)	480 (0.26)
anorexia	31 346 (0.35)	237 (0.45)	480 (0.26)
measures	31 346 (0.35)	320 (0.34)	377 (0.33)
oesophageal	31 346 (0.35)	197 (0.52)	344 (0.36)
practice	31 346 (0.35)	352 (0.31)	290 (0.43)
questionnaire	31 346 (0.35)	352 (0.31)	537 (0.23)
repair	31 346 (0.35)	320 (0.34)	263 (0.46)
size	31 346 (0.35)	182 (0.55)	480 (0.26)
university	31 346 (0.35)	666 (0.17)	416 (0.3)
affecting	30 354 (0.34)	546 (0.21)	537 (0.23)
alzheimer	30 354 (0.34)	352 (0.31)	416 (0.3)
balance	30 354 (0.34)	287 (0.38)	290 (0.43)
choice	30 354 (0.34)	402 (0.28)	344 (0.36)
coronary	30 354 (0.34)	197 (0.52)	416 (0.3)
duration	30 354 (0.34)	666 (0.17)	317 (0.4)
dysphagic	30 354 (0.34)	320 (0.34)	377 (0.33)
fluid	30 354 (0.34)	320 (0.34)	290 (0.43)
fluoride	30 354 (0.34)	182 (0.55)	537 (0.23)
genetic	30 354 (0.34)	352 (0.31)	480 (0.26)
inpatients	30 354 (0.34)	666 (0.17)	416 (0.3)
nasogastric	30 354 (0.34)	197 (0.52)	537 (0.23)
phase	30 354 (0.34)	546 (0.21)	377 (0.33)
sleeve	30 354 (0.34)	1067 (0.1)	238 (0.49)
against	29 368 (0.33)	471 (0.24)	344 (0.36)
bariatric	29 368 (0.33)	546 (0.21)	317 (0.4)
breastfeeding	29 368 (0.33)	471 (0.24)	480 (0.26)
fish	29 368 (0.33)	197 (0.52)	480 (0.26)
hernia	29 368 (0.33)	402 (0.28)	344 (0.36)
hypertension	29 368 (0.33)	471 (0.24)	647 (0.2)
ischemic	29 368 (0.33)	666 (0.17)	480 (0.26)
low-income	29 368 (0.33)	287 (0.38)	317 (0.4)
muscular	29 368 (0.33)	402 (0.28)	768 (0.16)
peg	29 368 (0.33)	149 (0.66)	647 (0.2)
perception	29 368 (0.33)	844 (0.14)	416 (0.3)
preoperative	29 368 (0.33)	471 (0.24)	416 (0.3)
project	29 368 (0.33)	546 (0.21)	263 (0.46)
spanish	29 368 (0.33)	666 (0.17)	344 (0.36)

trends	29	368 (0.33)	844 (0.14)	290 (0.43)
activities	28	383 (0.32)	546 (0.21)	377 (0.33)
environmental	28	383 (0.32)	287 (0.38)	344 (0.36)
interaction	28	383 (0.32)	546 (0.21)	290 (0.43)
learning	28	383 (0.32)	471 (0.24)	768 (0.16)
myotomy	28	383 (0.32)	352 (0.31)	290 (0.43)
northern	28	383 (0.32)	546 (0.21)	317 (0.4)
preferences	28	383 (0.32)	260 (0.41)	263 (0.46)
radiation	28	383 (0.32)	260 (0.41)	416 (0.3)
sensory	28	383 (0.32)	666 (0.17)	290 (0.43)
strength	28	383 (0.32)	2293 (0.034)	263 (0.46)
utilization	28	383 (0.32)	260 (0.41)	416 (0.3)
wild	28	383 (0.32)	217 (0.48)	537 (0.23)
acids	27	395 (0.31)	287 (0.38)	768 (0.16)
attitudes	27	395 (0.31)	402 (0.28)	537 (0.23)
burden	27	395 (0.31)	546 (0.21)	344 (0.36)
environment	27	395 (0.31)	402 (0.28)	377 (0.33)
feasibility	27	395 (0.31)	1067 (0.1)	238 (0.49)
ghrelin	27	395 (0.31)	135 (0.69)	768 (0.16)
history	27	395 (0.31)	471 (0.24)	344 (0.36)
improves	27	395 (0.31)	844 (0.14)	317 (0.4)
interventions	27	395 (0.31)	666 (0.17)	416 (0.3)
macronutrient	27	395 (0.31)	159 (0.62)	768 (0.16)
mediterranean	27	395 (0.31)	546 (0.21)	377 (0.33)
persons	27	395 (0.31)	546 (0.21)	416 (0.3)
pigs	27	395 (0.31)	260 (0.41)	647 (0.2)
possible	27	395 (0.31)	546 (0.21)	377 (0.33)
predict	27	395 (0.31)	320 (0.34)	317 (0.4)
resistance	27	395 (0.31)	666 (0.17)	210 (0.56)
restriction	27	395 (0.31)	237 (0.45)	344 (0.36)
services	27	395 (0.31)	320 (0.34)	480 (0.26)
stage	27	395 (0.31)	402 (0.28)	537 (0.23)
strategy	27	395 (0.31)	546 (0.21)	344 (0.36)
survivors	27	395 (0.31)	2293 (0.034)	210 (0.56)
aspects	26	416 (0.3)	217 (0.48)	768 (0.16)
banding	26	416 (0.3)	123 (0.76)	947 (0.13)
caregivers	26	416 (0.3)	352 (0.31)	647 (0.2)
esophagectomy	26	416 (0.3)	471 (0.24)	290 (0.43)
exercise	26	416 (0.3)	402 (0.28)	480 (0.26)
investigation	26	416 (0.3)	844 (0.14)	768 (0.16)
permanent	26	416 (0.3)	260 (0.41)	480 (0.26)
plant	26	416 (0.3)	320 (0.34)	377 (0.33)
populations	26	416 (0.3)	287 (0.38)	416 (0.3)

prediction	26	416 (0.3)	402 (0.28)	768 (0.16)
predicts	26	416 (0.3)	666 (0.17)	537 (0.23)
rat	26	416 (0.3)	170 (0.59)	647 (0.2)
recommendations	26	416 (0.3)	402 (0.28)	377 (0.33)
representative	26	416 (0.3)	666 (0.17)	344 (0.36)
respiratory	26	416 (0.3)	546 (0.21)	480 (0.26)
single	26	416 (0.3)	844 (0.14)	344 (0.36)
tissue	26	416 (0.3)	352 (0.31)	416 (0.3)
undernutrition	26	416 (0.3)	546 (0.21)	263 (0.46)
adiposity	25	434 (0.29)	287 (0.38)	416 (0.3)
adolescent	25	434 (0.29)	546 (0.21)	416 (0.3)
bacteria	25	434 (0.29)	352 (0.31)	290 (0.43)
black	25	434 (0.29)	237 (0.45)	647 (0.2)
correlates	25	434 (0.29)	546 (0.21)	317 (0.4)
diabetic	25	434 (0.29)	546 (0.21)	344 (0.36)
difficulties	25	434 (0.29)	402 (0.28)	647 (0.2)
hemodialysis	25	434 (0.29)	287 (0.38)	537 (0.23)
homes	25	434 (0.29)	666 (0.17)	344 (0.36)
injury	25	434 (0.29)	471 (0.24)	480 (0.26)
insights	25	434 (0.29)	2293 (0.034)	768 (0.16)
intensive	25	434 (0.29)	352 (0.31)	537 (0.23)
interactions	25	434 (0.29)	402 (0.28)	317 (0.4)
kidney	25	434 (0.29)	546 (0.21)	416 (0.3)
method	25	434 (0.29)	352 (0.31)	647 (0.2)
saudi	25	434 (0.29)	1457 (0.069)	416 (0.3)
series	25	434 (0.29)	844 (0.14)	416 (0.3)
sheep	25	434 (0.29)	402 (0.28)	263 (0.46)
toddlers	25	434 (0.29)	546 (0.21)	238 (0.49)
arthritis	24	453 (0.27)	546 (0.21)	416 (0.3)
effectiveness	24	453 (0.27)	471 (0.24)	416 (0.3)
european	24	453 (0.27)	182 (0.55)	647 (0.2)
flow	24	453 (0.27)	287 (0.38)	416 (0.3)
free	24	453 (0.27)	402 (0.28)	344 (0.36)
host	24	453 (0.27)	237 (0.45)	537 (0.23)
india	24	453 (0.27)	402 (0.28)	480 (0.26)
influencing	24	453 (0.27)	1457 (0.069)	647 (0.2)
insulin	24	453 (0.27)	352 (0.31)	344 (0.36)
nervosa	24	453 (0.27)	287 (0.38)	537 (0.23)
occlusal	24	453 (0.27)	1067 (0.1)	377 (0.33)
psychological	24	453 (0.27)	666 (0.17)	377 (0.33)
public	24	453 (0.27)	666 (0.17)	377 (0.33)
pulmonary	24	453 (0.27)	320 (0.34)	537 (0.23)
short-term	24	453 (0.27)	352 (0.31)	480 (0.26)

atherosclerosis	23 468 (0.26)	217 (0.48)	947 (0.13)
attending	23 468 (0.26)	471 (0.24)	537 (0.23)
denture	23 468 (0.26)	402 (0.28)	1180 (0.1)
design	23 468 (0.26)	546 (0.21)	263 (0.46)
esophagitis	23 468 (0.26)	546 (0.21)	416 (0.3)
forest	23 468 (0.26)	844 (0.14)	290 (0.43)
gut	23 468 (0.26)	1067 (0.1)	416 (0.3)
hormone	23 468 (0.26)	320 (0.34)	377 (0.33)
humans	23 468 (0.26)	237 (0.45)	768 (0.16)
improvement	23 468 (0.26)	546 (0.21)	768 (0.16)
increases	23 468 (0.26)	546 (0.21)	344 (0.36)
malignant	23 468 (0.26)	352 (0.31)	537 (0.23)
normal	23 468 (0.26)	402 (0.28)	416 (0.3)
pain	23 468 (0.26)	1067 (0.1)	290 (0.43)
pandemic	23 468 (0.26)	#SAKNAS!	2492 (0.033)
physiological	23 468 (0.26)	352 (0.31)	537 (0.23)
preterm	23 468 (0.26)	352 (0.31)	947 (0.13)
products	23 468 (0.26)	546 (0.21)	537 (0.23)
subjective	23 468 (0.26)	666 (0.17)	480 (0.26)
behavioural	22 487 (0.25)	666 (0.17)	416 (0.3)
characterization	22 487 (0.25)	546 (0.21)	377 (0.33)
cluster	22 487 (0.25)	1067 (0.1)	344 (0.36)
complete	22 487 (0.25)	352 (0.31)	1612 (0.066)
concentration	22 487 (0.25)	287 (0.38)	537 (0.23)
disparities	22 487 (0.25)	352 (0.31)	416 (0.3)
ecology	22 487 (0.25)	260 (0.41)	1612 (0.066)
esophagus	22 487 (0.25)	217 (0.48)	947 (0.13)
late	22 487 (0.25)	352 (0.31)	480 (0.26)
lipid	22 487 (0.25)	844 (0.14)	377 (0.33)
locally	22 487 (0.25)	402 (0.28)	416 (0.3)
mechanisms	22 487 (0.25)	352 (0.31)	537 (0.23)
production	22 487 (0.25)	471 (0.24)	537 (0.23)
prognosis	22 487 (0.25)	1457 (0.069)	537 (0.23)
promotion	22 487 (0.25)	666 (0.17)	377 (0.33)
secondary	22 487 (0.25)	666 (0.17)	647 (0.2)
setting	22 487 (0.25)	546 (0.21)	377 (0.33)
subgingival	22 487 (0.25)	1067 (0.1)	768 (0.16)
sugars	22 487 (0.25)	471 (0.24)	377 (0.33)
supplements	22 487 (0.25)	471 (0.24)	537 (0.23)
third	22 487 (0.25)	287 (0.38)	537 (0.23)
tubes	22 487 (0.25)	237 (0.45)	1180 (0.1)
variables	22 487 (0.25)	471 (0.24)	416 (0.3)
vegetable	22 487 (0.25)	844 (0.14)	416 (0.3)

adaptation	21 511 (0.24)	546 (0.21)	537 (0.23)
all-cause	21 511 (0.24)	1067 (0.1)	768 (0.16)
anthropometric	21 511 (0.24)	402 (0.28)	1612 (0.066)
atrophy	21 511 (0.24)	471 (0.24)	947 (0.13)
beverages	21 511 (0.24)	1067 (0.1)	317 (0.4)
dry	21 511 (0.24)	546 (0.21)	377 (0.33)
edentulous	21 511 (0.24)	402 (0.28)	480 (0.26)
elders	21 511 (0.24)	287 (0.38)	480 (0.26)
facilities	21 511 (0.24)	1457 (0.069)	647 (0.2)
fasting	21 511 (0.24)	402 (0.28)	416 (0.3)
fusion	21 511 (0.24)	1457 (0.069)	537 (0.23)
gingivitis	21 511 (0.24)	352 (0.31)	537 (0.23)
ingestion	21 511 (0.24)	287 (0.38)	537 (0.23)
issues	21 511 (0.24)	217 (0.48)	947 (0.13)
larval	21 511 (0.24)	287 (0.38)	537 (0.23)
mucositis	21 511 (0.24)	546 (0.21)	377 (0.33)
mutans	21 511 (0.24)	402 (0.28)	480 (0.26)
neurological	21 511 (0.24)	666 (0.17)	537 (0.23)
nutrients	21 511 (0.24)	287 (0.38)	537 (0.23)
paediatric	21 511 (0.24)	320 (0.34)	647 (0.2)
pharyngeal	21 511 (0.24)	352 (0.31)	768 (0.16)
plaque	21 511 (0.24)	471 (0.24)	647 (0.2)
prey	21 511 (0.24)	287 (0.38)	647 (0.2)
profiles	21 511 (0.24)	844 (0.14)	377 (0.33)
reduce	21 511 (0.24)	471 (0.24)	768 (0.16)
rheumatoid	21 511 (0.24)	546 (0.21)	416 (0.3)
schools	21 511 (0.24)	402 (0.28)	647 (0.2)
seasonal	21 511 (0.24)	402 (0.28)	377 (0.33)
sex	21 511 (0.24)	844 (0.14)	537 (0.23)
sociodemographic	21 511 (0.24)	666 (0.17)	480 (0.26)
sources	21 511 (0.24)	546 (0.21)	344 (0.36)
squamous	21 511 (0.24)	1067 (0.1)	537 (0.23)
tobacco	21 511 (0.24)	546 (0.21)	537 (0.23)
uk	21 511 (0.24)	1067 (0.1)	317 (0.4)
unit	21 511 (0.24)	260 (0.41)	947 (0.13)
adherence	20 546 (0.23)	#SAKNAS!	416 (0.3)
alcohol	20 546 (0.23)	546 (0.21)	377 (0.33)
als	20 546 (0.23)	402 (0.28)	768 (0.16)
atresia	20 546 (0.23)	546 (0.21)	416 (0.3)
australian	20 546 (0.23)	1067 (0.1)	263 (0.46)
cattle	20 546 (0.23)	666 (0.17)	647 (0.2)
complementary	20 546 (0.23)	1457 (0.069)	290 (0.43)
consequences	20 546 (0.23)	402 (0.28)	537 (0.23)

database	20 546 (0.23)	2293 (0.034)	768 (0.16)
depression	20 546 (0.23)	2293 (0.034)	416 (0.3)
digestive	20 546 (0.23)	1067 (0.1)	344 (0.36)
diversity	20 546 (0.23)	1067 (0.1)	647 (0.2)
drinking	20 546 (0.23)	402 (0.28)	768 (0.16)
eosinophilic	20 546 (0.23)	844 (0.14)	416 (0.3)
gestational	20 546 (0.23)	546 (0.21)	537 (0.23)
grazing	20 546 (0.23)	471 (0.24)	768 (0.16)
groups	20 546 (0.23)	471 (0.24)	647 (0.2)
herbivore	20 546 (0.23)	287 (0.38)	537 (0.23)
hiatal	20 546 (0.23)	1457 (0.069)	480 (0.26)
independent	20 546 (0.23)	844 (0.14)	480 (0.26)
influences	20 546 (0.23)	402 (0.28)	480 (0.26)
local	20 546 (0.23)	402 (0.28)	537 (0.23)
mineral	20 546 (0.23)	471 (0.24)	647 (0.2)
multidisciplinary	20 546 (0.23)	1457 (0.069)	416 (0.3)
nitrogen	20 546 (0.23)	844 (0.14)	480 (0.26)
perceptions	20 546 (0.23)	402 (0.28)	647 (0.2)
polymorphism	20 546 (0.23)	471 (0.24)	290 (0.43)
prognostic	20 546 (0.23)	844 (0.14)	647 (0.2)
scale	20 546 (0.23)	1067 (0.1)	647 (0.2)
stents	20 546 (0.23)	287 (0.38)	647 (0.2)
strictures	20 546 (0.23)	320 (0.34)	537 (0.23)
term	20 546 (0.23)	844 (0.14)	290 (0.43)
test	20 546 (0.23)	402 (0.28)	647 (0.2)
2011-2014	19 579 (0.22)	#SAKNAS!	480 (0.26)
3d	19 579 (0.22)	#SAKNAS!	#SAKNAS!
african	19 579 (0.22)	260 (0.41)	768 (0.16)
amino	19 579 (0.22)	546 (0.21)	947 (0.13)
antioxidant	19 579 (0.22)	1067 (0.1)	647 (0.2)
apnea	19 579 (0.22)	546 (0.21)	416 (0.3)
availability	19 579 (0.22)	471 (0.24)	768 (0.16)
calves	19 579 (0.22)	844 (0.14)	647 (0.2)
center	19 579 (0.22)	844 (0.14)	537 (0.23)
chemoradiation	19 579 (0.22)	287 (0.38)	768 (0.16)
city	19 579 (0.22)	666 (0.17)	416 (0.3)
communities	19 579 (0.22)	471 (0.24)	537 (0.23)
concurrent	19 579 (0.22)	402 (0.28)	768 (0.16)
context	19 579 (0.22)	546 (0.21)	377 (0.33)
digestibility	19 579 (0.22)	1457 (0.069)	768 (0.16)
epidemiological	19 579 (0.22)	1457 (0.069)	416 (0.3)
evaluating	19 579 (0.22)	1067 (0.1)	377 (0.33)
gingivalis	19 579 (0.22)	471 (0.24)	480 (0.26)

global	19 579 (0.22)	844 (0.14)	480 (0.26)
identification	19 579 (0.22)	402 (0.28)	947 (0.13)
implant	19 579 (0.22)	546 (0.21)	537 (0.23)
lactating	19 579 (0.22)	402 (0.28)	647 (0.2)
lesions	19 579 (0.22)	666 (0.17)	480 (0.26)
link	19 579 (0.22)	844 (0.14)	537 (0.23)
mendelian	19 579 (0.22)	#SAKNAS!	2492 (0.033)
meta-analysis	19 579 (0.22)	2293 (0.034)	768 (0.16)
mexican	19 579 (0.22)	402 (0.28)	537 (0.23)
neuropeptide	19 579 (0.22)	237 (0.45)	2492 (0.033)
parenting	19 579 (0.22)	844 (0.14)	480 (0.26)
physiology	19 579 (0.22)	352 (0.31)	537 (0.23)
porphyromonas	19 579 (0.22)	471 (0.24)	480 (0.26)
position	19 579 (0.22)	352 (0.31)	537 (0.23)
progression	19 579 (0.22)	320 (0.34)	768 (0.16)
rheological	19 579 (0.22)	844 (0.14)	947 (0.13)
service	19 579 (0.22)	402 (0.28)	647 (0.2)
single-center	19 579 (0.22)	1067 (0.1)	768 (0.16)
taiwan	19 579 (0.22)	471 (0.24)	647 (0.2)
temporal	19 579 (0.22)	471 (0.24)	647 (0.2)
therapeutic	19 579 (0.22)	402 (0.28)	1612 (0.066)
thickness	19 579 (0.22)	666 (0.17)	537 (0.23)
version	19 579 (0.22)	1067 (0.1)	537 (0.23)
western	19 579 (0.22)	666 (0.17)	537 (0.23)
access	18 621 (0.21)	546 (0.21)	480 (0.26)
airway	18 621 (0.21)	666 (0.17)	416 (0.3)
alterations	18 621 (0.21)	546 (0.21)	647 (0.2)
artery	18 621 (0.21)	546 (0.21)	647 (0.2)
artificial	18 621 (0.21)	666 (0.17)	947 (0.13)
biomarkers	18 621 (0.21)	#SAKNAS!	377 (0.33)
calcium	18 621 (0.21)	402 (0.28)	480 (0.26)
carbohydrate	18 621 (0.21)	666 (0.17)	537 (0.23)
cariogenic	18 621 (0.21)	666 (0.17)	647 (0.2)
cases	18 621 (0.21)	471 (0.24)	1612 (0.066)
cholesterol	18 621 (0.21)	471 (0.24)	947 (0.13)
costs	18 621 (0.21)	546 (0.21)	647 (0.2)
criteria	18 621 (0.21)	2293 (0.034)	647 (0.2)
distribution	18 621 (0.21)	287 (0.38)	947 (0.13)
improving	18 621 (0.21)	666 (0.17)	647 (0.2)
individual	18 621 (0.21)	471 (0.24)	768 (0.16)
infarction	18 621 (0.21)	666 (0.17)	480 (0.26)
juvenile	18 621 (0.21)	546 (0.21)	537 (0.23)
knhanes	18 621 (0.21)	#SAKNAS!	416 (0.3)

leaf	18 621 (0.21)	666 (0.17)	416 (0.3)
magnetic	18 621 (0.21)	2293 (0.034)	768 (0.16)
marine	18 621 (0.21)	471 (0.24)	647 (0.2)
meals	18 621 (0.21)	666 (0.17)	647 (0.2)
measurement	18 621 (0.21)	844 (0.14)	537 (0.23)
mexico	18 621 (0.21)	666 (0.17)	480 (0.26)
nasopharyngeal	18 621 (0.21)	844 (0.14)	768 (0.16)
nissen	18 621 (0.21)	402 (0.28)	768 (0.16)
onset	18 621 (0.21)	666 (0.17)	647 (0.2)
pathogens	18 621 (0.21)	546 (0.21)	537 (0.23)
randomization	18 621 (0.21)	#SAKNAS!	2492 (0.033)
reproductive	18 621 (0.21)	402 (0.28)	1180 (0.1)
requiring	18 621 (0.21)	2293 (0.034)	537 (0.23)
resection	18 621 (0.21)	402 (0.28)	1180 (0.1)
spectrum	18 621 (0.21)	1457 (0.069)	344 (0.36)
spinal	18 621 (0.21)	666 (0.17)	1180 (0.1)
stent	18 621 (0.21)	352 (0.31)	537 (0.23)
tract	18 621 (0.21)	666 (0.17)	416 (0.3)
validity	18 621 (0.21)	844 (0.14)	480 (0.26)
workers	18 621 (0.21)	546 (0.21)	768 (0.16)
action	17 660 (0.19)	402 (0.28)	768 (0.16)
administration	17 660 (0.19)	402 (0.28)	1180 (0.1)
advice	17 660 (0.19)	546 (0.21)	947 (0.13)
autism	17 660 (0.19)	2293 (0.034)	317 (0.4)
biofilm	17 660 (0.19)	1067 (0.1)	647 (0.2)
cells	17 660 (0.19)	402 (0.28)	1180 (0.1)
developmental	17 660 (0.19)	666 (0.17)	480 (0.26)
evolution	17 660 (0.19)	666 (0.17)	537 (0.23)
formula	17 660 (0.19)	546 (0.21)	768 (0.16)
gender	17 660 (0.19)	844 (0.14)	768 (0.16)
high-fat	17 660 (0.19)	546 (0.21)	377 (0.33)
insecurity	17 660 (0.19)	2293 (0.034)	768 (0.16)
italian	17 660 (0.19)	2293 (0.034)	537 (0.23)
mediating	17 660 (0.19)	#SAKNAS!	1612 (0.066)
metal	17 660 (0.19)	402 (0.28)	947 (0.13)
oil	17 660 (0.19)	666 (0.17)	537 (0.23)
perspective	17 660 (0.19)	402 (0.28)	947 (0.13)
posterior	17 660 (0.19)	2293 (0.034)	537 (0.23)
predictor	17 660 (0.19)	1457 (0.069)	344 (0.36)
preventive	17 660 (0.19)	666 (0.17)	537 (0.23)
safe	17 660 (0.19)	471 (0.24)	768 (0.16)
sensitivity	17 660 (0.19)	1457 (0.069)	416 (0.3)
short	17 660 (0.19)	1067 (0.1)	768 (0.16)

spine		17 660 (0.19)	471 (0.24)	768 (0.16)
stenosis		17 660 (0.19)	666 (0.17)	537 (0.23)
structural		17 660 (0.19)	1067 (0.1)	537 (0.23)
structure		17 660 (0.19)	402 (0.28)	947 (0.13)
surveys		17 660 (0.19)	546 (0.21)	768 (0.16)
swedish		17 660 (0.19)	320 (0.34)	768 (0.16)
tertiary		17 660 (0.19)	#SAKNAS!	480 (0.26)
tropical		17 660 (0.19)	2293 (0.034)	377 (0.33)
volume		17 660 (0.19)	1457 (0.069)	537 (0.23)
white		17 660 (0.19)	471 (0.24)	647 (0.2)
	2009	16 693 (0.18)	2293 (0.034)	377 (0.33)
active		16 693 (0.18)	471 (0.24)	947 (0.13)
adjustable		16 693 (0.18)	260 (0.41)	947 (0.13)
alters		16 693 (0.18)	471 (0.24)	647 (0.2)
arabia		16 693 (0.18)	1457 (0.069)	768 (0.16)
areas		16 693 (0.18)	1067 (0.1)	416 (0.3)
benefits		16 693 (0.18)	844 (0.14)	480 (0.26)
beverage		16 693 (0.18)	2293 (0.034)	480 (0.26)
biological		16 693 (0.18)	1457 (0.069)	947 (0.13)
cancers		16 693 (0.18)	844 (0.14)	416 (0.3)
deer		16 693 (0.18)	402 (0.28)	947 (0.13)
dystrophy		16 693 (0.18)	844 (0.14)	947 (0.13)
electrical		16 693 (0.18)	844 (0.14)	416 (0.3)
females		16 693 (0.18)	666 (0.17)	537 (0.23)
fiber		16 693 (0.18)	844 (0.14)	768 (0.16)
german		16 693 (0.18)	666 (0.17)	480 (0.26)
goats		16 693 (0.18)	402 (0.28)	1180 (0.1)
healthcare		16 693 (0.18)	2293 (0.034)	1180 (0.1)
hospitalization		16 693 (0.18)	1457 (0.069)	537 (0.23)
iron		16 693 (0.18)	666 (0.17)	768 (0.16)
lactation		16 693 (0.18)	666 (0.17)	416 (0.3)
liver		16 693 (0.18)	1067 (0.1)	537 (0.23)
machine		16 693 (0.18)	#SAKNAS!	1612 (0.066)
mediation		16 693 (0.18)	2293 (0.034)	1180 (0.1)
motility		16 693 (0.18)	471 (0.24)	1180 (0.1)
nationally		16 693 (0.18)	844 (0.14)	647 (0.2)
neurogenic		16 693 (0.18)	402 (0.28)	1180 (0.1)
nucleus		16 693 (0.18)	320 (0.34)	1180 (0.1)
parent		16 693 (0.18)	1457 (0.069)	537 (0.23)
pathway		16 693 (0.18)	1067 (0.1)	416 (0.3)
positive		16 693 (0.18)	1067 (0.1)	647 (0.2)
prophylactic		16 693 (0.18)	2293 (0.034)	377 (0.33)
recurrent		16 693 (0.18)	844 (0.14)	768 (0.16)

region		16 693 (0.18)	844 (0.14)	947 (0.13)
signaling		16 693 (0.18)	1457 (0.069)	416 (0.3)
skeletal		16 693 (0.18)	844 (0.14)	480 (0.26)
soft		16 693 (0.18)	546 (0.21)	647 (0.2)
special		16 693 (0.18)	666 (0.17)	1612 (0.066)
stages		16 693 (0.18)	1067 (0.1)	537 (0.23)
successful		16 693 (0.18)	352 (0.31)	947 (0.13)
sugar-sweetened		16 693 (0.18)	2293 (0.034)	480 (0.26)
texture-modified		16 693 (0.18)	2293 (0.034)	1180 (0.1)
thai		16 693 (0.18)	666 (0.17)	480 (0.26)
	2010	15 736 (0.17)	1067 (0.1)	416 (0.3)
12-year-old		15 736 (0.17)	471 (0.24)	647 (0.2)
5-year		15 736 (0.17)	2293 (0.034)	480 (0.26)
across		15 736 (0.17)	1067 (0.1)	947 (0.13)
antibodies		15 736 (0.17)	1067 (0.1)	537 (0.23)
aphid		15 736 (0.17)	1067 (0.1)	768 (0.16)
assess		15 736 (0.17)	844 (0.14)	537 (0.23)
breast		15 736 (0.17)	1067 (0.1)	537 (0.23)
cardiometabolic		15 736 (0.17)	2293 (0.034)	647 (0.2)
causal		15 736 (0.17)	2293 (0.034)	2492 (0.033)
cause		15 736 (0.17)	666 (0.17)	1180 (0.1)
causes		15 736 (0.17)	546 (0.21)	768 (0.16)
classification		15 736 (0.17)	546 (0.21)	1612 (0.066)
comparing		15 736 (0.17)	471 (0.24)	947 (0.13)
crevicular		15 736 (0.17)	471 (0.24)	647 (0.2)
dentists		15 736 (0.17)	546 (0.21)	768 (0.16)
discectomy		15 736 (0.17)	1457 (0.069)	768 (0.16)
eat		15 736 (0.17)	1457 (0.069)	768 (0.16)
essential		15 736 (0.17)	1067 (0.1)	1180 (0.1)
expenditure		15 736 (0.17)	546 (0.21)	1180 (0.1)
features		15 736 (0.17)	1457 (0.069)	768 (0.16)
fitness		15 736 (0.17)	844 (0.14)	647 (0.2)
flap		15 736 (0.17)	546 (0.21)	2492 (0.033)
giant		15 736 (0.17)	1067 (0.1)	416 (0.3)
glycemic		15 736 (0.17)	1457 (0.069)	947 (0.13)
infancy		15 736 (0.17)	666 (0.17)	647 (0.2)
international		15 736 (0.17)	844 (0.14)	1612 (0.066)
lead		15 736 (0.17)	546 (0.21)	537 (0.23)
maintenance		15 736 (0.17)	287 (0.38)	1612 (0.066)
medicine		15 736 (0.17)	1457 (0.069)	647 (0.2)
models		15 736 (0.17)	546 (0.21)	647 (0.2)
morbid		15 736 (0.17)	402 (0.28)	768 (0.16)
north		15 736 (0.17)	352 (0.31)	1180 (0.1)

outpatient	15 736 (0.17)	1457 (0.069)	647 (0.2)
poststroke	15 736 (0.17)	2293 (0.034)	947 (0.13)
presence	15 736 (0.17)	844 (0.14)	537 (0.23)
prevent	15 736 (0.17)	844 (0.14)	768 (0.16)
printing	15 736 (0.17)	#SAKNAS!	#SAKNAS!
procedure	15 736 (0.17)	546 (0.21)	647 (0.2)
propensity	15 736 (0.17)	2293 (0.034)	537 (0.23)
rates	15 736 (0.17)	471 (0.24)	768 (0.16)
serotonin	15 736 (0.17)	260 (0.41)	1612 (0.066)
specific	15 736 (0.17)	352 (0.31)	2492 (0.033)
stricture	15 736 (0.17)	471 (0.24)	537 (0.23)
sweden	15 736 (0.17)	320 (0.34)	1612 (0.066)
systems	15 736 (0.17)	844 (0.14)	1612 (0.066)
toxicity	15 736 (0.17)	402 (0.28)	947 (0.13)
wic	15 736 (0.17)	471 (0.24)	1180 (0.1)
abdominal	14 784 (0.16)	666 (0.17)	1180 (0.1)
asthma	14 784 (0.16)	1067 (0.1)	647 (0.2)
breakfast	14 784 (0.16)	1067 (0.1)	768 (0.16)
capacity	14 784 (0.16)	1067 (0.1)	768 (0.16)
cavity	14 784 (0.16)	666 (0.17)	947 (0.13)
challenge	14 784 (0.16)	1067 (0.1)	647 (0.2)
challenges	14 784 (0.16)	666 (0.17)	768 (0.16)
considerations	14 784 (0.16)	666 (0.17)	768 (0.16)
continuous	14 784 (0.16)	666 (0.17)	647 (0.2)
contribution	14 784 (0.16)	352 (0.31)	1180 (0.1)
conventional	14 784 (0.16)	844 (0.14)	1612 (0.066)
cricopharyngeal	14 784 (0.16)	666 (0.17)	768 (0.16)
dentistry	14 784 (0.16)	471 (0.24)	1180 (0.1)
developing	14 784 (0.16)	666 (0.17)	768 (0.16)
differential	14 784 (0.16)	471 (0.24)	947 (0.13)
difficulty	14 784 (0.16)	2293 (0.034)	947 (0.13)
disabilities	14 784 (0.16)	844 (0.14)	768 (0.16)
effective	14 784 (0.16)	844 (0.14)	647 (0.2)
ethiopia	14 784 (0.16)	2293 (0.034)	537 (0.23)
experiences	14 784 (0.16)	1457 (0.069)	947 (0.13)
families	14 784 (0.16)	546 (0.21)	947 (0.13)
force	14 784 (0.16)	1067 (0.1)	768 (0.16)
gland	14 784 (0.16)	666 (0.17)	768 (0.16)
impacts	14 784 (0.16)	1457 (0.069)	416 (0.3)
implementation	14 784 (0.16)	1457 (0.069)	647 (0.2)
independently	14 784 (0.16)	546 (0.21)	647 (0.2)
insect	14 784 (0.16)	352 (0.31)	1612 (0.066)
insurance	14 784 (0.16)	1067 (0.1)	537 (0.23)

lung		14 784 (0.16)	1067 (0.1)	647 (0.2)
metabolites		14 784 (0.16)	2293 (0.034)	1612 (0.066)
neoadjuvant		14 784 (0.16)	546 (0.21)	647 (0.2)
neuromuscular		14 784 (0.16)	844 (0.14)	416 (0.3)
neurons		14 784 (0.16)	1457 (0.069)	537 (0.23)
obstruction		14 784 (0.16)	666 (0.17)	768 (0.16)
offspring		14 784 (0.16)	844 (0.14)	647 (0.2)
oxidative		14 784 (0.16)	2293 (0.034)	416 (0.3)
partial		14 784 (0.16)	471 (0.24)	1612 (0.066)
perioperative		14 784 (0.16)	1067 (0.1)	1612 (0.066)
polymorphisms		14 784 (0.16)	471 (0.24)	947 (0.13)
prior		14 784 (0.16)	1067 (0.1)	768 (0.16)
professionals		14 784 (0.16)	666 (0.17)	2492 (0.033)
programme		14 784 (0.16)	666 (0.17)	768 (0.16)
quantitative		14 784 (0.16)	666 (0.17)	1180 (0.1)
reliability		14 784 (0.16)	844 (0.14)	647 (0.2)
renal		14 784 (0.16)	844 (0.14)	768 (0.16)
reward		14 784 (0.16)	844 (0.14)	416 (0.3)
root		14 784 (0.16)	1067 (0.1)	537 (0.23)
secretion		14 784 (0.16)	844 (0.14)	537 (0.23)
surface		14 784 (0.16)	844 (0.14)	947 (0.13)
surveillance		14 784 (0.16)	471 (0.24)	647 (0.2)
symptom		14 784 (0.16)	546 (0.21)	1612 (0.066)
toothbrushing		14 784 (0.16)	1067 (0.1)	537 (0.23)
toxin		14 784 (0.16)	402 (0.28)	768 (0.16)
traditional		14 784 (0.16)	1067 (0.1)	768 (0.16)
transplantation		14 784 (0.16)	1457 (0.069)	647 (0.2)
trophic		14 784 (0.16)	546 (0.21)	947 (0.13)
users		14 784 (0.16)	666 (0.17)	537 (0.23)
variability		14 784 (0.16)	666 (0.17)	647 (0.2)
zinc		14 784 (0.16)	1067 (0.1)	1612 (0.066)
	2012	13 843 (0.15)	#SAKNAS!	317 (0.4)
	2018	13 843 (0.15)	#SAKNAS!	768 (0.16)
africa		13 843 (0.15)	320 (0.34)	1612 (0.066)
animal		13 843 (0.15)	844 (0.14)	1612 (0.066)
attachment		13 843 (0.15)	666 (0.17)	768 (0.16)
augmentation		13 843 (0.15)	2293 (0.034)	647 (0.2)
australia		13 843 (0.15)	666 (0.17)	1180 (0.1)
bangladesh		13 843 (0.15)	1067 (0.1)	768 (0.16)
cardiac		13 843 (0.15)	844 (0.14)	647 (0.2)
clinic		13 843 (0.15)	844 (0.14)	1180 (0.1)
complex		13 843 (0.15)	1457 (0.069)	947 (0.13)
congenital		13 843 (0.15)	1067 (0.1)	768 (0.16)

corn	13 843 (0.15)	2293 (0.034)	537 (0.23)
countries	13 843 (0.15)	546 (0.21)	947 (0.13)
crab	13 843 (0.15)	1457 (0.069)	480 (0.26)
death	13 843 (0.15)	471 (0.24)	1180 (0.1)
dentures	13 843 (0.15)	666 (0.17)	768 (0.16)
diagnostic	13 843 (0.15)	1457 (0.069)	947 (0.13)
drinks	13 843 (0.15)	666 (0.17)	768 (0.16)
enamel	13 843 (0.15)	666 (0.17)	1612 (0.066)
epidemiology	13 843 (0.15)	546 (0.21)	947 (0.13)
epidermolysis	13 843 (0.15)	402 (0.28)	1180 (0.1)
events	13 843 (0.15)	546 (0.21)	647 (0.2)
examining	13 843 (0.15)	666 (0.17)	1180 (0.1)
generalist	13 843 (0.15)	546 (0.21)	768 (0.16)
genes	13 843 (0.15)	844 (0.14)	947 (0.13)
germany	13 843 (0.15)	546 (0.21)	1180 (0.1)
honey	13 843 (0.15)	666 (0.17)	537 (0.23)
incident	13 843 (0.15)	1067 (0.1)	768 (0.16)
invasive	13 843 (0.15)	844 (0.14)	537 (0.23)
italy	13 843 (0.15)	844 (0.14)	647 (0.2)
laboratory	13 843 (0.15)	546 (0.21)	1180 (0.1)
laryngeal	13 843 (0.15)	546 (0.21)	1180 (0.1)
macaques	13 843 (0.15)	844 (0.14)	480 (0.26)
mandibular	13 843 (0.15)	844 (0.14)	1180 (0.1)
mastication	13 843 (0.15)	1067 (0.1)	647 (0.2)
measure	13 843 (0.15)	844 (0.14)	647 (0.2)
microbial	13 843 (0.15)	666 (0.17)	1180 (0.1)
monkeys	13 843 (0.15)	1067 (0.1)	480 (0.26)
newly	13 843 (0.15)	844 (0.14)	1180 (0.1)
paraesophageal	13 843 (0.15)	471 (0.24)	947 (0.13)
peripheral	13 843 (0.15)	844 (0.14)	537 (0.23)
plants	13 843 (0.15)	1067 (0.1)	480 (0.26)
preschoolers	13 843 (0.15)	666 (0.17)	768 (0.16)
process	13 843 (0.15)	844 (0.14)	416 (0.3)
programs	13 843 (0.15)	2293 (0.034)	647 (0.2)
receptors	13 843 (0.15)	1457 (0.069)	647 (0.2)
reduction	13 843 (0.15)	1067 (0.1)	647 (0.2)
replacement	13 843 (0.15)	666 (0.17)	647 (0.2)
selective	13 843 (0.15)	471 (0.24)	768 (0.16)
self-expanding	13 843 (0.15)	402 (0.28)	947 (0.13)
simple	13 843 (0.15)	666 (0.17)	768 (0.16)
socio-economic	13 843 (0.15)	402 (0.28)	1180 (0.1)
spain	13 843 (0.15)	546 (0.21)	1180 (0.1)
speech	13 843 (0.15)	1067 (0.1)	1180 (0.1)

staff	13	843 (0.15)	844 (0.14)	537 (0.23)
streptococci	13	843 (0.15)	546 (0.21)	947 (0.13)
sucrose	13	843 (0.15)	844 (0.14)	480 (0.26)
temperature	13	843 (0.15)	1067 (0.1)	647 (0.2)
transition	13	843 (0.15)	666 (0.17)	768 (0.16)
update	13	843 (0.15)	844 (0.14)	647 (0.2)
vascular	13	843 (0.15)	402 (0.28)	947 (0.13)
vertical	13	843 (0.15)	546 (0.21)	1180 (0.1)
videofluoroscopic	13	843 (0.15)	1067 (0.1)	647 (0.2)
well-being	13	843 (0.15)	2293 (0.034)	647 (0.2)
whole	13	843 (0.15)	546 (0.21)	947 (0.13)
2013-2014	12	909 (0.14)	#SAKNAS!	768 (0.16)
acceptability	12	909 (0.14)	1457 (0.069)	768 (0.16)
adverse	12	909 (0.14)	546 (0.21)	2492 (0.033)
albumin	12	909 (0.14)	666 (0.17)	947 (0.13)
alternative	12	909 (0.14)	844 (0.14)	1180 (0.1)
alveolar	12	909 (0.14)	844 (0.14)	947 (0.13)
banded	12	909 (0.14)	844 (0.14)	947 (0.13)
bullosa	12	909 (0.14)	471 (0.24)	1180 (0.1)
circumference	12	909 (0.14)	844 (0.14)	947 (0.13)
commercial	12	909 (0.14)	1457 (0.069)	647 (0.2)
components	12	909 (0.14)	844 (0.14)	947 (0.13)
course	12	909 (0.14)	1457 (0.069)	947 (0.13)
decay	12	909 (0.14)	844 (0.14)	2492 (0.033)
descriptive	12	909 (0.14)	1067 (0.1)	768 (0.16)
dilation	12	909 (0.14)	844 (0.14)	1180 (0.1)
diptera	12	909 (0.14)	352 (0.31)	1180 (0.1)
disability	12	909 (0.14)	1067 (0.1)	947 (0.13)
ecological	12	909 (0.14)	844 (0.14)	1180 (0.1)
exploration	12	909 (0.14)	1067 (0.1)	1180 (0.1)
goat	12	909 (0.14)	546 (0.21)	1180 (0.1)
growing	12	909 (0.14)	546 (0.21)	1612 (0.066)
guideline	12	909 (0.14)	844 (0.14)	1180 (0.1)
hemiptera	12	909 (0.14)	1457 (0.069)	537 (0.23)
income	12	909 (0.14)	666 (0.17)	947 (0.13)
indigenous	12	909 (0.14)	2293 (0.034)	768 (0.16)
inequalities	12	909 (0.14)	1457 (0.069)	647 (0.2)
ingestive	12	909 (0.14)	844 (0.14)	1612 (0.066)
intestinal	12	909 (0.14)	546 (0.21)	2492 (0.033)
investigating	12	909 (0.14)	#SAKNAS!	647 (0.2)
liquid	12	909 (0.14)	844 (0.14)	1180 (0.1)
mental	12	909 (0.14)	2293 (0.034)	947 (0.13)
modeling	12	909 (0.14)	1457 (0.069)	947 (0.13)

modulation	12 909 (0.14)	844 (0.14)	768 (0.16)
morbidity	12 909 (0.14)	402 (0.28)	2492 (0.033)
morbidly	12 909 (0.14)	471 (0.24)	1612 (0.066)
myocardial	12 909 (0.14)	1067 (0.1)	537 (0.23)
occurrence	12 909 (0.14)	1457 (0.069)	768 (0.16)
olfactory	12 909 (0.14)	1067 (0.1)	537 (0.23)
overview	12 909 (0.14)	666 (0.17)	1180 (0.1)
palatability	12 909 (0.14)	844 (0.14)	647 (0.2)
palliation	12 909 (0.14)	546 (0.21)	768 (0.16)
perspectives	12 909 (0.14)	844 (0.14)	647 (0.2)
ph	12 909 (0.14)	1067 (0.1)	768 (0.16)
preparation	12 909 (0.14)	1457 (0.069)	947 (0.13)
pre-school	12 909 (0.14)	666 (0.17)	947 (0.13)
prevalent	12 909 (0.14)	1457 (0.069)	647 (0.2)
promote	12 909 (0.14)	1067 (0.1)	768 (0.16)
protective	12 909 (0.14)	2293 (0.034)	768 (0.16)
ratio	12 909 (0.14)	1067 (0.1)	947 (0.13)
reducing	12 909 (0.14)	471 (0.24)	947 (0.13)
regulates	12 909 (0.14)	#SAKNAS!	768 (0.16)
selectivity	12 909 (0.14)	844 (0.14)	647 (0.2)
self-selection	12 909 (0.14)	402 (0.28)	947 (0.13)
significance	12 909 (0.14)	471 (0.24)	1180 (0.1)
spontaneous	12 909 (0.14)	471 (0.24)	947 (0.13)
stable	12 909 (0.14)	666 (0.17)	647 (0.2)
subacute	12 909 (0.14)	1457 (0.069)	768 (0.16)
tilapia	12 909 (0.14)	666 (0.17)	1180 (0.1)
turkey	12 909 (0.14)	844 (0.14)	1612 (0.066)
various	12 909 (0.14)	666 (0.17)	1612 (0.066)
virus	12 909 (0.14)	2293 (0.034)	947 (0.13)
ad	11 970 (0.13)	1067 (0.1)	1612 (0.066)
admission	11 970 (0.13)	1457 (0.069)	947 (0.13)
algorithm	11 970 (0.13)	546 (0.21)	1612 (0.066)
anesthesia	11 970 (0.13)	1067 (0.1)	947 (0.13)
ant	11 970 (0.13)	666 (0.17)	1180 (0.1)
antireflux	11 970 (0.13)	666 (0.17)	768 (0.16)
arterial	11 970 (0.13)	#SAKNAS!	947 (0.13)
asian	11 970 (0.13)	2293 (0.034)	1180 (0.1)
barriers	11 970 (0.13)	1067 (0.1)	1180 (0.1)
baseline	11 970 (0.13)	844 (0.14)	768 (0.16)
beef	11 970 (0.13)	1457 (0.069)	1612 (0.066)
beetle	11 970 (0.13)	546 (0.21)	947 (0.13)
beyond	11 970 (0.13)	1067 (0.1)	947 (0.13)
birds	11 970 (0.13)	352 (0.31)	1612 (0.066)

botulinum	11 970 (0.13)	471 (0.24)	947 (0.13)
brown	11 970 (0.13)	844 (0.14)	947 (0.13)
captive	11 970 (0.13)	546 (0.21)	947 (0.13)
cleaning	11 970 (0.13)	#SAKNAS!	768 (0.16)
complication	11 970 (0.13)	666 (0.17)	1612 (0.066)
containing	11 970 (0.13)	666 (0.17)	1612 (0.066)
coverage	11 970 (0.13)	2293 (0.034)	647 (0.2)
cross	11 970 (0.13)	1067 (0.1)	1180 (0.1)
cytokines	11 970 (0.13)	1457 (0.069)	947 (0.13)
decline	11 970 (0.13)	#SAKNAS!	947 (0.13)
delivery	11 970 (0.13)	844 (0.14)	2492 (0.033)
depressive	11 970 (0.13)	1457 (0.069)	768 (0.16)
dietitians	11 970 (0.13)	1457 (0.069)	947 (0.13)
digestion	11 970 (0.13)	666 (0.17)	768 (0.16)
district	11 970 (0.13)	1067 (0.1)	768 (0.16)
duchenne	11 970 (0.13)	1067 (0.1)	1180 (0.1)
elementary	11 970 (0.13)	1067 (0.1)	768 (0.16)
excessive	11 970 (0.13)	#SAKNAS!	416 (0.3)
exploratory	11 970 (0.13)	1067 (0.1)	1180 (0.1)
hay	11 970 (0.13)	844 (0.14)	947 (0.13)
hba1c	11 970 (0.13)	1067 (0.1)	947 (0.13)
height	11 970 (0.13)	844 (0.14)	768 (0.16)
hemoglobin	11 970 (0.13)	666 (0.17)	1180 (0.1)
hispanic	11 970 (0.13)	1067 (0.1)	480 (0.26)
holstein	11 970 (0.13)	#SAKNAS!	480 (0.26)
indicator	11 970 (0.13)	1457 (0.069)	537 (0.23)
indonesia	11 970 (0.13)	2293 (0.034)	647 (0.2)
interdental	11 970 (0.13)	#SAKNAS!	947 (0.13)
involvement	11 970 (0.13)	1067 (0.1)	768 (0.16)
joint	11 970 (0.13)	1067 (0.1)	647 (0.2)
key	11 970 (0.13)	546 (0.21)	2492 (0.033)
koreans	11 970 (0.13)	1457 (0.069)	947 (0.13)
load	11 970 (0.13)	666 (0.17)	947 (0.13)
matter	11 970 (0.13)	1457 (0.069)	768 (0.16)
mediates	11 970 (0.13)	#SAKNAS!	947 (0.13)
melanogaster	11 970 (0.13)	1067 (0.1)	947 (0.13)
modulates	11 970 (0.13)	844 (0.14)	947 (0.13)
molar	11 970 (0.13)	1457 (0.069)	1180 (0.1)
mouse	11 970 (0.13)	1457 (0.069)	1180 (0.1)
multicentre	11 970 (0.13)	1457 (0.069)	647 (0.2)
neonatal	11 970 (0.13)	546 (0.21)	1180 (0.1)
neuronal	11 970 (0.13)	352 (0.31)	1612 (0.066)
optimal	11 970 (0.13)	352 (0.31)	2492 (0.033)

orofacial	11	970 (0.13)	1067 (0.1)	947 (0.13)
orthodontic	11	970 (0.13)	1457 (0.069)	480 (0.26)
parenteral	11	970 (0.13)	546 (0.21)	768 (0.16)
participants	11	970 (0.13)	844 (0.14)	947 (0.13)
pea	11	970 (0.13)	1457 (0.069)	#SAKNAS!
peptide	11	970 (0.13)	666 (0.17)	1180 (0.1)
postpartum	11	970 (0.13)	#SAKNAS!	947 (0.13)
processing	11	970 (0.13)	1457 (0.069)	1180 (0.1)
provision	11	970 (0.13)	844 (0.14)	1180 (0.1)
recent	11	970 (0.13)	666 (0.17)	1180 (0.1)
remaining	11	970 (0.13)	1457 (0.069)	768 (0.16)
resource	11	970 (0.13)	546 (0.21)	1612 (0.066)
resources	11	970 (0.13)	666 (0.17)	947 (0.13)
roles	11	970 (0.13)	844 (0.14)	947 (0.13)
site	11	970 (0.13)	1067 (0.1)	947 (0.13)
spatial	11	970 (0.13)	666 (0.17)	1612 (0.066)
sphincter	11	970 (0.13)	2293 (0.034)	1180 (0.1)
starch	11	970 (0.13)	1067 (0.1)	647 (0.2)
stem	11	970 (0.13)	1067 (0.1)	768 (0.16)
streptococcus	11	970 (0.13)	1067 (0.1)	1180 (0.1)
traumatic	11	970 (0.13)	1457 (0.069)	768 (0.16)
treating	11	970 (0.13)	666 (0.17)	1180 (0.1)
underweight	11	970 (0.13)	844 (0.14)	537 (0.23)
usa	11	970 (0.13)	1067 (0.1)	947 (0.13)
vitro	11	970 (0.13)	1457 (0.069)	2492 (0.033)
weaning	11	970 (0.13)	471 (0.24)	1180 (0.1)
why	11	970 (0.13)	546 (0.21)	768 (0.16)
2014	10	1054 (0.11)	#SAKNAS!	537 (0.23)
1999-2004	10	1054 (0.11)	844 (0.14)	768 (0.16)
1-year	10	1054 (0.11)	#SAKNAS!	947 (0.13)
25-hydroxyvitamin	10	1054 (0.11)	666 (0.17)	1180 (0.1)
abnormal	10	1054 (0.11)	844 (0.14)	1612 (0.066)
abnormalities	10	1054 (0.11)	546 (0.21)	2492 (0.033)
absorption	10	1054 (0.11)	666 (0.17)	768 (0.16)
acceptance	10	1054 (0.11)	1067 (0.1)	768 (0.16)
adolescence	10	1054 (0.11)	#SAKNAS!	1180 (0.1)
ageing	10	1054 (0.11)	1457 (0.069)	947 (0.13)
athletes	10	1054 (0.11)	1067 (0.1)	947 (0.13)
awareness	10	1054 (0.11)	1457 (0.069)	1612 (0.066)
baby	10	1054 (0.11)	#SAKNAS!	768 (0.16)
benign	10	1054 (0.11)	844 (0.14)	1180 (0.1)
biochemical	10	1054 (0.11)	666 (0.17)	1180 (0.1)
canadian	10	1054 (0.11)	1457 (0.069)	1612 (0.066)

chemical	10	1054 (0.11)	1067 (0.1)	768 (0.16)
circulating	10	1054 (0.11)	1457 (0.069)	1612 (0.066)
class	10	1054 (0.11)	1457 (0.069)	768 (0.16)
coffee	10	1054 (0.11)	#SAKNAS!	480 (0.26)
colonization	10	1054 (0.11)	844 (0.14)	768 (0.16)
component	10	1054 (0.11)	844 (0.14)	768 (0.16)
concentrate	10	1054 (0.11)	2293 (0.034)	768 (0.16)
count	10	1054 (0.11)	1457 (0.069)	647 (0.2)
de	10	1054 (0.11)	#SAKNAS!	537 (0.23)
defects	10	1054 (0.11)	546 (0.21)	2492 (0.033)
detection	10	1054 (0.11)	1457 (0.069)	537 (0.23)
diagnostics	10	1054 (0.11)	666 (0.17)	947 (0.13)
drink	10	1054 (0.11)	2293 (0.034)	537 (0.23)
dynamics	10	1054 (0.11)	1067 (0.1)	768 (0.16)
educational	10	1054 (0.11)	1067 (0.1)	1180 (0.1)
emptying	10	1054 (0.11)	844 (0.14)	1180 (0.1)
erosion	10	1054 (0.11)	1067 (0.1)	947 (0.13)
estimating	10	1054 (0.11)	1457 (0.069)	537 (0.23)
feedlot	10	1054 (0.11)	2293 (0.034)	947 (0.13)
fibrosis	10	1054 (0.11)	1457 (0.069)	2492 (0.033)
field	10	1054 (0.11)	666 (0.17)	1180 (0.1)
finishing	10	1054 (0.11)	1457 (0.069)	#SAKNAS!
form	10	1054 (0.11)	1067 (0.1)	1180 (0.1)
formation	10	1054 (0.11)	1067 (0.1)	947 (0.13)
gastroplasty	10	1054 (0.11)	844 (0.14)	1612 (0.066)
gerd	10	1054 (0.11)	1067 (0.1)	947 (0.13)
gum	10	1054 (0.11)	2293 (0.034)	768 (0.16)
helicobacter	10	1054 (0.11)	1067 (0.1)	947 (0.13)
hemorrhage	10	1054 (0.11)	1067 (0.1)	947 (0.13)
horses	10	1054 (0.11)	1067 (0.1)	647 (0.2)
household	10	1054 (0.11)	1457 (0.069)	1612 (0.066)
hypopharyngeal	10	1054 (0.11)	844 (0.14)	947 (0.13)
hypothalamus	10	1054 (0.11)	666 (0.17)	1612 (0.066)
ill	10	1054 (0.11)	1067 (0.1)	2492 (0.033)
immune	10	1054 (0.11)	1457 (0.069)	1612 (0.066)
indian	10	1054 (0.11)	546 (0.21)	1180 (0.1)
induction	10	1054 (0.11)	1067 (0.1)	1180 (0.1)
inpatient	10	1054 (0.11)	1457 (0.069)	1612 (0.066)
insertion	10	1054 (0.11)	666 (0.17)	1180 (0.1)
intensity-modulated	10	1054 (0.11)	1067 (0.1)	1180 (0.1)
internal	10	1054 (0.11)	1457 (0.069)	768 (0.16)
iran	10	1054 (0.11)	546 (0.21)	1612 (0.066)
iv	10	1054 (0.11)	1457 (0.069)	768 (0.16)

length	10	1054 (0.11)	666 (0.17)	1612 (0.066)
liquids	10	1054 (0.11)	1457 (0.069)	647 (0.2)
literature	10	1054 (0.11)	546 (0.21)	2492 (0.033)
macaca	10	1054 (0.11)	1067 (0.1)	768 (0.16)
manifestations	10	1054 (0.11)	666 (0.17)	1180 (0.1)
manometry	10	1054 (0.11)	#SAKNAS!	768 (0.16)
measuring	10	1054 (0.11)	666 (0.17)	947 (0.13)
microbiological	10	1054 (0.11)	844 (0.14)	1180 (0.1)
missing	10	1054 (0.11)	1457 (0.069)	1612 (0.066)
mixed-methods	10	1054 (0.11)	#SAKNAS!	480 (0.26)
modulate	10	1054 (0.11)	1067 (0.1)	947 (0.13)
morphology	10	1054 (0.11)	1067 (0.1)	947 (0.13)
muscles	10	1054 (0.11)	#SAKNAS!	947 (0.13)
necrosis	10	1054 (0.11)	666 (0.17)	1612 (0.066)
non-surgical	10	1054 (0.11)	2293 (0.034)	647 (0.2)
outpatients	10	1054 (0.11)	844 (0.14)	1180 (0.1)
part	10	1054 (0.11)	1067 (0.1)	768 (0.16)
pathways	10	1054 (0.11)	1457 (0.069)	947 (0.13)
potentially	10	1054 (0.11)	2293 (0.034)	1180 (0.1)
presentation	10	1054 (0.11)	844 (0.14)	947 (0.13)
procedures	10	1054 (0.11)	666 (0.17)	2492 (0.033)
promoting	10	1054 (0.11)	1457 (0.069)	1612 (0.066)
proximal	10	1054 (0.11)	2293 (0.034)	947 (0.13)
pylori	10	1054 (0.11)	1067 (0.1)	947 (0.13)
rare	10	1054 (0.11)	546 (0.21)	1612 (0.066)
recipients	10	1054 (0.11)	1067 (0.1)	768 (0.16)
relative	10	1054 (0.11)	2293 (0.034)	947 (0.13)
residential	10	1054 (0.11)	2293 (0.034)	647 (0.2)
satisfaction	10	1054 (0.11)	844 (0.14)	947 (0.13)
school-aged	10	1054 (0.11)	2293 (0.034)	537 (0.23)
sectional	10	1054 (0.11)	1067 (0.1)	1612 (0.066)
self-efficacy	10	1054 (0.11)	1067 (0.1)	947 (0.13)
self-report	10	1054 (0.11)	844 (0.14)	1180 (0.1)
significant	10	1054 (0.11)	666 (0.17)	947 (0.13)
silage	10	1054 (0.11)	1457 (0.069)	647 (0.2)
society	10	1054 (0.11)	1457 (0.069)	947 (0.13)
success	10	1054 (0.11)	666 (0.17)	1180 (0.1)
supplement	10	1054 (0.11)	844 (0.14)	1180 (0.1)
transfer	10	1054 (0.11)	844 (0.14)	1180 (0.1)
treatments	10	1054 (0.11)	#SAKNAS!	768 (0.16)
tumor	10	1054 (0.11)	546 (0.21)	1612 (0.066)
turkish	10	1054 (0.11)	666 (0.17)	1180 (0.1)
urinary	10	1054 (0.11)	2293 (0.034)	768 (0.16)

utility		10	1054 (0.11)	2293 (0.034)	768 (0.16)
variations		10	1054 (0.11)	666 (0.17)	1612 (0.066)
variety		10	1054 (0.11)	#SAKNAS!	768 (0.16)
vegetables		10	1054 (0.11)	1457 (0.069)	947 (0.13)
	2015	9	1160 (0.1)	#SAKNAS!	537 (0.23)
	2019	9	1160 (0.1)	#SAKNAS!	2492 (0.033)
1988-1991		9	1160 (0.1)	352 (0.31)	#SAKNAS!
1988-1994		9	1160 (0.1)	352 (0.31)	#SAKNAS!
2008-2010		9	1160 (0.1)	2293 (0.034)	480 (0.26)
2011-2012		9	1160 (0.1)	#SAKNAS!	537 (0.23)
2013-2015		9	1160 (0.1)	#SAKNAS!	947 (0.13)
2016-2018		9	1160 (0.1)	#SAKNAS!	#SAKNAS!
adequacy		9	1160 (0.1)	844 (0.14)	1180 (0.1)
agents		9	1160 (0.1)	666 (0.17)	1612 (0.066)
aggressive		9	1160 (0.1)	844 (0.14)	768 (0.16)
appetitive		9	1160 (0.1)	#SAKNAS!	1612 (0.066)
approaches		9	1160 (0.1)	1067 (0.1)	947 (0.13)
atlantic		9	1160 (0.1)	666 (0.17)	947 (0.13)
audit		9	1160 (0.1)	546 (0.21)	1180 (0.1)
beliefs		9	1160 (0.1)	666 (0.17)	1612 (0.066)
benefit		9	1160 (0.1)	1457 (0.069)	1180 (0.1)
breathing		9	1160 (0.1)	1457 (0.069)	768 (0.16)
caregiver		9	1160 (0.1)	844 (0.14)	#SAKNAS!
carotid		9	1160 (0.1)	666 (0.17)	1612 (0.066)
chickens		9	1160 (0.1)	666 (0.17)	1612 (0.066)
chilean		9	1160 (0.1)	1457 (0.069)	1180 (0.1)
cigarette		9	1160 (0.1)	546 (0.21)	1612 (0.066)
cleft		9	1160 (0.1)	1067 (0.1)	1612 (0.066)
clustering		9	1160 (0.1)	844 (0.14)	#SAKNAS!
compensatory		9	1160 (0.1)	471 (0.24)	1612 (0.066)
compounds		9	1160 (0.1)	2293 (0.034)	1612 (0.066)
consensus		9	1160 (0.1)	#SAKNAS!	768 (0.16)
consuming		9	1160 (0.1)	666 (0.17)	1612 (0.066)
copepod		9	1160 (0.1)	352 (0.31)	#SAKNAS!
counseling		9	1160 (0.1)	844 (0.14)	768 (0.16)
critical		9	1160 (0.1)	1457 (0.069)	1180 (0.1)
culture		9	1160 (0.1)	402 (0.28)	#SAKNAS!
deciduous		9	1160 (0.1)	844 (0.14)	1180 (0.1)
decisions		9	1160 (0.1)	1457 (0.069)	647 (0.2)
dentate		9	1160 (0.1)	1457 (0.069)	947 (0.13)
dialysis		9	1160 (0.1)	844 (0.14)	1612 (0.066)
direct		9	1160 (0.1)	844 (0.14)	1612 (0.066)
discharge		9	1160 (0.1)	2293 (0.034)	1180 (0.1)

drug	9	1160 (0.1)	666 (0.17)	1180 (0.1)
dyslipidemia	9	1160 (0.1)	844 (0.14)	1612 (0.066)
eastern	9	1160 (0.1)	1067 (0.1)	1180 (0.1)
egg	9	1160 (0.1)	844 (0.14)	1180 (0.1)
equation	9	1160 (0.1)	2293 (0.034)	1180 (0.1)
estimates	9	1160 (0.1)	666 (0.17)	947 (0.13)
ethnic	9	1160 (0.1)	1067 (0.1)	1180 (0.1)
exclusive	9	1160 (0.1)	1067 (0.1)	2492 (0.033)
fecal	9	1160 (0.1)	#SAKNAS!	768 (0.16)
fermentation	9	1160 (0.1)	#SAKNAS!	2492 (0.033)
finland	9	1160 (0.1)	2293 (0.034)	947 (0.13)
forage	9	1160 (0.1)	1067 (0.1)	1180 (0.1)
framework	9	1160 (0.1)	844 (0.14)	2492 (0.033)
gustatory	9	1160 (0.1)	1457 (0.069)	537 (0.23)
handgrip	9	1160 (0.1)	#SAKNAS!	1180 (0.1)
head-and-neck	9	1160 (0.1)	844 (0.14)	1612 (0.066)
heller	9	1160 (0.1)	1457 (0.069)	647 (0.2)
high-resolution	9	1160 (0.1)	#SAKNAS!	768 (0.16)
high-risk	9	1160 (0.1)	1067 (0.1)	1180 (0.1)
homeostasis	9	1160 (0.1)	546 (0.21)	1612 (0.066)
impairments	9	1160 (0.1)	1067 (0.1)	1180 (0.1)
inappropriate	9	1160 (0.1)	1067 (0.1)	1180 (0.1)
inequality	9	1160 (0.1)	#SAKNAS!	647 (0.2)
initiative	9	1160 (0.1)	2293 (0.034)	1612 (0.066)
island	9	1160 (0.1)	844 (0.14)	947 (0.13)
lack	9	1160 (0.1)	1067 (0.1)	768 (0.16)
lactobacillus	9	1160 (0.1)	2293 (0.034)	947 (0.13)
lambs	9	1160 (0.1)	2293 (0.034)	647 (0.2)
language	9	1160 (0.1)	666 (0.17)	1612 (0.066)
laryngectomy	9	1160 (0.1)	844 (0.14)	947 (0.13)
libitum	9	1160 (0.1)	1067 (0.1)	1612 (0.066)
links	9	1160 (0.1)	1457 (0.069)	947 (0.13)
lipoprotein	9	1160 (0.1)	844 (0.14)	2492 (0.033)
live	9	1160 (0.1)	2293 (0.034)	1180 (0.1)
mechanism	9	1160 (0.1)	666 (0.17)	1612 (0.066)
methamphetamine	9	1160 (0.1)	1457 (0.069)	647 (0.2)
micronutrient	9	1160 (0.1)	844 (0.14)	2492 (0.033)
modifies	9	1160 (0.1)	1457 (0.069)	1180 (0.1)
mucosal	9	1160 (0.1)	666 (0.17)	1612 (0.066)
multivariate	9	1160 (0.1)	1067 (0.1)	768 (0.16)
negatively	9	1160 (0.1)	#SAKNAS!	768 (0.16)
network	9	1160 (0.1)	844 (0.14)	2492 (0.033)
nile	9	1160 (0.1)	844 (0.14)	1180 (0.1)

non-obese		9	1160 (0.1)	2293 (0.034)	647 (0.2)
objective		9	1160 (0.1)	1067 (0.1)	768 (0.16)
organic		9	1160 (0.1)	2293 (0.034)	2492 (0.033)
pakistan		9	1160 (0.1)	2293 (0.034)	1180 (0.1)
particle		9	1160 (0.1)	1067 (0.1)	1180 (0.1)
pathogen		9	1160 (0.1)	2293 (0.034)	768 (0.16)
pathology		9	1160 (0.1)	844 (0.14)	1180 (0.1)
portuguese		9	1160 (0.1)	#SAKNAS!	2492 (0.033)
prenatal		9	1160 (0.1)	1067 (0.1)	1180 (0.1)
presenting		9	1160 (0.1)	1067 (0.1)	1180 (0.1)
prosthetic		9	1160 (0.1)	844 (0.14)	1612 (0.066)
recurrence		9	1160 (0.1)	1457 (0.069)	647 (0.2)
regarding		9	1160 (0.1)	2293 (0.034)	947 (0.13)
removal		9	1160 (0.1)	666 (0.17)	2492 (0.033)
reproduction		9	1160 (0.1)	546 (0.21)	1180 (0.1)
river		9	1160 (0.1)	1067 (0.1)	1180 (0.1)
routine		9	1160 (0.1)	1457 (0.069)	947 (0.13)
rumen		9	1160 (0.1)	1067 (0.1)	1180 (0.1)
school-based		9	1160 (0.1)	2293 (0.034)	1180 (0.1)
shrimp		9	1160 (0.1)	666 (0.17)	1180 (0.1)
signals		9	1160 (0.1)	546 (0.21)	1612 (0.066)
smoke		9	1160 (0.1)	1067 (0.1)	1180 (0.1)
sodium		9	1160 (0.1)	2293 (0.034)	768 (0.16)
sweet		9	1160 (0.1)	#SAKNAS!	947 (0.13)
technique		9	1160 (0.1)	1457 (0.069)	947 (0.13)
tests		9	1160 (0.1)	844 (0.14)	947 (0.13)
thickeners		9	1160 (0.1)	#SAKNAS!	768 (0.16)
thoracic		9	1160 (0.1)	1457 (0.069)	768 (0.16)
unresectable		9	1160 (0.1)	844 (0.14)	768 (0.16)
visits		9	1160 (0.1)	1067 (0.1)	947 (0.13)
voice		9	1160 (0.1)	1067 (0.1)	1612 (0.066)
wear		9	1160 (0.1)	1457 (0.069)	1612 (0.066)
wheat		9	1160 (0.1)	#SAKNAS!	947 (0.13)
zealand		9	1160 (0.1)	844 (0.14)	1612 (0.066)
	2011	8	1276 (0.091)	2293 (0.034)	647 (0.2)
actinomycetemcomitans		8	1276 (0.091)	844 (0.14)	1180 (0.1)
additives		8	1276 (0.091)	2293 (0.034)	1612 (0.066)
agent		8	1276 (0.091)	1457 (0.069)	1180 (0.1)
aids		8	1276 (0.091)	402 (0.28)	#SAKNAS!
anastomosis		8	1276 (0.091)	2293 (0.034)	947 (0.13)
anatomy		8	1276 (0.091)	844 (0.14)	1180 (0.1)
anemia		8	1276 (0.091)	844 (0.14)	947 (0.13)
aphididae		8	1276 (0.091)	2293 (0.034)	947 (0.13)

basic	8	1276 (0.091)	1457 (0.069)	2492 (0.033)
bass	8	1276 (0.091)	402 (0.28)	#SAKNAS!
bedside	8	1276 (0.091)	1457 (0.069)	1612 (0.066)
bee	8	1276 (0.091)	844 (0.14)	1180 (0.1)
biology	8	1276 (0.091)	666 (0.17)	1612 (0.066)
bottle	8	1276 (0.091)	844 (0.14)	2492 (0.033)
bulbar	8	1276 (0.091)	#SAKNAS!	1612 (0.066)
carbohydrates	8	1276 (0.091)	844 (0.14)	2492 (0.033)
centers	8	1276 (0.091)	844 (0.14)	#SAKNAS!
child-feeding	8	1276 (0.091)	666 (0.17)	1612 (0.066)
chimpanzees	8	1276 (0.091)	1457 (0.069)	947 (0.13)
choices	8	1276 (0.091)	1457 (0.069)	947 (0.13)
coleoptera	8	1276 (0.091)	844 (0.14)	1180 (0.1)
college	8	1276 (0.091)	666 (0.17)	1180 (0.1)
communication	8	1276 (0.091)	844 (0.14)	1180 (0.1)
comorbidities	8	1276 (0.091)	1457 (0.069)	1180 (0.1)
controls	8	1276 (0.091)	1067 (0.1)	768 (0.16)
convalescent	8	1276 (0.091)	2293 (0.034)	1180 (0.1)
counselling	8	1276 (0.091)	1067 (0.1)	1180 (0.1)
cues	8	1276 (0.091)	666 (0.17)	1612 (0.066)
cultural	8	1276 (0.091)	1457 (0.069)	647 (0.2)
cycle	8	1276 (0.091)	844 (0.14)	947 (0.13)
decrease	8	1276 (0.091)	1067 (0.1)	1612 (0.066)
dependence	8	1276 (0.091)	844 (0.14)	1180 (0.1)
dependent	8	1276 (0.091)	#SAKNAS!	768 (0.16)
deprivation	8	1276 (0.091)	1067 (0.1)	1612 (0.066)
dietetic	8	1276 (0.091)	471 (0.24)	2492 (0.033)
double	8	1276 (0.091)	1457 (0.069)	947 (0.13)
dynamic	8	1276 (0.091)	1067 (0.1)	1612 (0.066)
emotional	8	1276 (0.091)	#SAKNAS!	768 (0.16)
evaluate	8	1276 (0.091)	1067 (0.1)	2492 (0.033)
excess	8	1276 (0.091)	#SAKNAS!	768 (0.16)
expansion	8	1276 (0.091)	#SAKNAS!	1612 (0.066)
extract	8	1276 (0.091)	666 (0.17)	1180 (0.1)
fistula	8	1276 (0.091)	1457 (0.069)	947 (0.13)
frail	8	1276 (0.091)	2293 (0.034)	768 (0.16)
free-ranging	8	1276 (0.091)	844 (0.14)	1180 (0.1)
fruits	8	1276 (0.091)	2293 (0.034)	947 (0.13)
full	8	1276 (0.091)	#SAKNAS!	768 (0.16)
gastro-oesophageal	8	1276 (0.091)	666 (0.17)	1180 (0.1)
gestation	8	1276 (0.091)	844 (0.14)	1180 (0.1)
grade	8	1276 (0.091)	1457 (0.069)	1612 (0.066)
great	8	1276 (0.091)	666 (0.17)	1612 (0.066)

habitat	8	1276 (0.091)	844 (0.14)	947 (0.13)
high-density	8	1276 (0.091)	2293 (0.034)	#SAKNAS!
hiv	8	1276 (0.091)	666 (0.17)	2492 (0.033)
hormones	8	1276 (0.091)	1457 (0.069)	1180 (0.1)
hydration	8	1276 (0.091)	1067 (0.1)	1612 (0.066)
hymenoptera	8	1276 (0.091)	844 (0.14)	1612 (0.066)
hyperactivity	8	1276 (0.091)	1067 (0.1)	1180 (0.1)
hypofunction	8	1276 (0.091)	#SAKNAS!	2492 (0.033)
identifying	8	1276 (0.091)	844 (0.14)	1612 (0.066)
image	8	1276 (0.091)	1067 (0.1)	947 (0.13)
immunity	8	1276 (0.091)	2293 (0.034)	947 (0.13)
immunoglobulin	8	1276 (0.091)	#SAKNAS!	537 (0.23)
indications	8	1276 (0.091)	1067 (0.1)	947 (0.13)
information	8	1276 (0.091)	844 (0.14)	947 (0.13)
infusion	8	1276 (0.091)	1457 (0.069)	768 (0.16)
insects	8	1276 (0.091)	1457 (0.069)	768 (0.16)
integration	8	1276 (0.091)	546 (0.21)	1612 (0.066)
interleukin-6	8	1276 (0.091)	666 (0.17)	1612 (0.066)
interplay	8	1276 (0.091)	#SAKNAS!	1612 (0.066)
interprofessional	8	1276 (0.091)	2293 (0.034)	768 (0.16)
introduction	8	1276 (0.091)	2293 (0.034)	1180 (0.1)
items	8	1276 (0.091)	666 (0.17)	#SAKNAS!
jejunostomy	8	1276 (0.091)	666 (0.17)	1180 (0.1)
kingdom	8	1276 (0.091)	2293 (0.034)	947 (0.13)
lepidoptera	8	1276 (0.091)	546 (0.21)	1612 (0.066)
longevity	8	1276 (0.091)	546 (0.21)	#SAKNAS!
males	8	1276 (0.091)	2293 (0.034)	768 (0.16)
malocclusion	8	1276 (0.091)	1457 (0.069)	1180 (0.1)
mealtime	8	1276 (0.091)	2293 (0.034)	768 (0.16)
mechanical	8	1276 (0.091)	2293 (0.034)	1180 (0.1)
middle	8	1276 (0.091)	1067 (0.1)	1612 (0.066)
mild	8	1276 (0.091)	1457 (0.069)	1180 (0.1)
mobile	8	1276 (0.091)	2293 (0.034)	1180 (0.1)
moderate	8	1276 (0.091)	#SAKNAS!	537 (0.23)
monitoring	8	1276 (0.091)	2293 (0.034)	947 (0.13)
motivation	8	1276 (0.091)	2293 (0.034)	947 (0.13)
multilevel	8	1276 (0.091)	1457 (0.069)	947 (0.13)
neural	8	1276 (0.091)	#SAKNAS!	947 (0.13)
neurologically	8	1276 (0.091)	1067 (0.1)	1180 (0.1)
nomogram	8	1276 (0.091)	#SAKNAS!	#SAKNAS!
observation	8	1276 (0.091)	1457 (0.069)	2492 (0.033)
oncology	8	1276 (0.091)	666 (0.17)	1612 (0.066)
one-year	8	1276 (0.091)	1067 (0.1)	1180 (0.1)

osteoporosis	8	1276 (0.091)	844 (0.14)	2492 (0.033)
ovary	8	1276 (0.091)	1067 (0.1)	#SAKNAS!
per	8	1276 (0.091)	2293 (0.034)	1612 (0.066)
persistent	8	1276 (0.091)	666 (0.17)	1612 (0.066)
plus	8	1276 (0.091)	2293 (0.034)	768 (0.16)
point	8	1276 (0.091)	1457 (0.069)	768 (0.16)
poland	8	1276 (0.091)	#SAKNAS!	1180 (0.1)
polycystic	8	1276 (0.091)	1067 (0.1)	#SAKNAS!
post	8	1276 (0.091)	1457 (0.069)	1180 (0.1)
practical	8	1276 (0.091)	1457 (0.069)	1180 (0.1)
predation	8	1276 (0.091)	666 (0.17)	1180 (0.1)
preventing	8	1276 (0.091)	2293 (0.034)	768 (0.16)
radiological	8	1276 (0.091)	1067 (0.1)	1180 (0.1)
registry	8	1276 (0.091)	#SAKNAS!	768 (0.16)
regression	8	1276 (0.091)	1067 (0.1)	947 (0.13)
relevance	8	1276 (0.091)	844 (0.14)	1180 (0.1)
restrictive	8	1276 (0.091)	1457 (0.069)	1180 (0.1)
screen	8	1276 (0.091)	2293 (0.034)	2492 (0.033)
settings	8	1276 (0.091)	#SAKNAS!	537 (0.23)
sexual	8	1276 (0.091)	1067 (0.1)	947 (0.13)
shanghai	8	1276 (0.091)	1457 (0.069)	2492 (0.033)
shape	8	1276 (0.091)	1457 (0.069)	1612 (0.066)
sows	8	1276 (0.091)	844 (0.14)	2492 (0.033)
speech-language	8	1276 (0.091)	2293 (0.034)	1612 (0.066)
standard	8	1276 (0.091)	2293 (0.034)	947 (0.13)
style	8	1276 (0.091)	1457 (0.069)	647 (0.2)
subsequent	8	1276 (0.091)	1457 (0.069)	1612 (0.066)
supply	8	1276 (0.091)	666 (0.17)	1180 (0.1)
supportive	8	1276 (0.091)	#SAKNAS!	1180 (0.1)
suppresses	8	1276 (0.091)	2293 (0.034)	768 (0.16)
susceptibility	8	1276 (0.091)	1067 (0.1)	1612 (0.066)
sympatric	8	1276 (0.091)	546 (0.21)	#SAKNAS!
tanzania	8	1276 (0.091)	844 (0.14)	2492 (0.033)
tea	8	1276 (0.091)	#SAKNAS!	647 (0.2)
testing	8	1276 (0.091)	1067 (0.1)	768 (0.16)
thailand	8	1276 (0.091)	1067 (0.1)	1180 (0.1)
thermal	8	1276 (0.091)	666 (0.17)	2492 (0.033)
tools	8	1276 (0.091)	2293 (0.034)	647 (0.2)
towards	8	1276 (0.091)	2293 (0.034)	#SAKNAS!
transoral	8	1276 (0.091)	1457 (0.069)	1180 (0.1)
treat	8	1276 (0.091)	#SAKNAS!	647 (0.2)
triglyceride	8	1276 (0.091)	1457 (0.069)	1180 (0.1)
ultra-processed	8	1276 (0.091)	#SAKNAS!	1612 (0.066)

ultrasonography	8	1276 (0.091)	#SAKNAS!	947 (0.13)
ultrasound	8	1276 (0.091)	1457 (0.069)	1180 (0.1)
underwent	8	1276 (0.091)	1457 (0.069)	947 (0.13)
units	8	1276 (0.091)	1067 (0.1)	1612 (0.066)
unmet	8	1276 (0.091)	#SAKNAS!	1612 (0.066)
ventilation	8	1276 (0.091)	1067 (0.1)	1180 (0.1)
videofluoroscopy	8	1276 (0.091)	2293 (0.034)	768 (0.16)
visual	8	1276 (0.091)	2293 (0.034)	1612 (0.066)
ward	8	1276 (0.091)	2293 (0.034)	1180 (0.1)
wearing	8	1276 (0.091)	1457 (0.069)	#SAKNAS!
welfare	8	1276 (0.091)	2293 (0.034)	2492 (0.033)
working	8	1276 (0.091)	#SAKNAS!	1180 (0.1)
3-year-old	7	1426 (0.08)	1067 (0.1)	1612 (0.066)
5-year-old	7	1426 (0.08)	1067 (0.1)	1180 (0.1)
abundance	7	1426 (0.08)	1067 (0.1)	1612 (0.066)
accuracy	7	1426 (0.08)	1067 (0.1)	1180 (0.1)
adipose	7	1426 (0.08)	1457 (0.069)	1612 (0.066)
americans	7	1426 (0.08)	666 (0.17)	2492 (0.033)
amongst	7	1426 (0.08)	1457 (0.069)	1180 (0.1)
amount	7	1426 (0.08)	#SAKNAS!	947 (0.13)
angle	7	1426 (0.08)	2293 (0.034)	1180 (0.1)
anthropometry	7	1426 (0.08)	1457 (0.069)	1180 (0.1)
antibody	7	1426 (0.08)	666 (0.17)	1612 (0.066)
anti-reflux	7	1426 (0.08)	#SAKNAS!	1180 (0.1)
anxiety	7	1426 (0.08)	2293 (0.034)	1180 (0.1)
aortic	7	1426 (0.08)	2293 (0.034)	1180 (0.1)
aphids	7	1426 (0.08)	1067 (0.1)	1612 (0.066)
apical	7	1426 (0.08)	#SAKNAS!	1180 (0.1)
balloon	7	1426 (0.08)	1067 (0.1)	1612 (0.066)
band	7	1426 (0.08)	1457 (0.069)	1180 (0.1)
bees	7	1426 (0.08)	#SAKNAS!	647 (0.2)
benthic	7	1426 (0.08)	1067 (0.1)	1612 (0.066)
bleeding	7	1426 (0.08)	1457 (0.069)	1180 (0.1)
bolus	7	1426 (0.08)	1067 (0.1)	2492 (0.033)
bowel	7	1426 (0.08)	2293 (0.034)	1612 (0.066)
breast-feeding	7	1426 (0.08)	1067 (0.1)	1180 (0.1)
bulimia	7	1426 (0.08)	844 (0.14)	1612 (0.066)
cachexia	7	1426 (0.08)	2293 (0.034)	1612 (0.066)
caloric	7	1426 (0.08)	1067 (0.1)	1180 (0.1)
carbon	7	1426 (0.08)	2293 (0.034)	947 (0.13)
carcass	7	1426 (0.08)	2293 (0.034)	1612 (0.066)
cats	7	1426 (0.08)	1457 (0.069)	1612 (0.066)
chemistry	7	1426 (0.08)	1457 (0.069)	768 (0.16)

circadian	7	1426 (0.08)	546 (0.21)	2492 (0.033)
coastal	7	1426 (0.08)	1067 (0.1)	2492 (0.033)
colony	7	1426 (0.08)	1067 (0.1)	2492 (0.033)
combination	7	1426 (0.08)	1457 (0.069)	947 (0.13)
comfort	7	1426 (0.08)	2293 (0.034)	768 (0.16)
comorbidity	7	1426 (0.08)	1457 (0.069)	1180 (0.1)
compliance	7	1426 (0.08)	1067 (0.1)	1612 (0.066)
concept	7	1426 (0.08)	1457 (0.069)	768 (0.16)
contrasting	7	1426 (0.08)	1067 (0.1)	1180 (0.1)
cortex	7	1426 (0.08)	1457 (0.069)	1180 (0.1)
crossbred	7	1426 (0.08)	#SAKNAS!	1180 (0.1)
cystic	7	1426 (0.08)	2293 (0.034)	#SAKNAS!
day	7	1426 (0.08)	844 (0.14)	1612 (0.066)
days	7	1426 (0.08)	2293 (0.034)	1612 (0.066)
decreases	7	1426 (0.08)	1457 (0.069)	2492 (0.033)
definitive	7	1426 (0.08)	2293 (0.034)	1180 (0.1)
description	7	1426 (0.08)	1457 (0.069)	947 (0.13)
dilution	7	1426 (0.08)	546 (0.21)	2492 (0.033)
dissection	7	1426 (0.08)	1067 (0.1)	2492 (0.033)
distinct	7	1426 (0.08)	1457 (0.069)	1180 (0.1)
dose	7	1426 (0.08)	1457 (0.069)	1612 (0.066)
double-blind	7	1426 (0.08)	2293 (0.034)	1180 (0.1)
dutch	7	1426 (0.08)	2293 (0.034)	1180 (0.1)
east	7	1426 (0.08)	1457 (0.069)	1612 (0.066)
elective	7	1426 (0.08)	1457 (0.069)	1612 (0.066)
elevation	7	1426 (0.08)	1067 (0.1)	1180 (0.1)
end-stage	7	1426 (0.08)	1457 (0.069)	1180 (0.1)
energetic	7	1426 (0.08)	1457 (0.069)	947 (0.13)
environments	7	1426 (0.08)	1457 (0.069)	1612 (0.066)
europe	7	1426 (0.08)	#SAKNAS!	647 (0.2)
evolutionary	7	1426 (0.08)	844 (0.14)	1612 (0.066)
fecundity	7	1426 (0.08)	1067 (0.1)	1612 (0.066)
fetal	7	1426 (0.08)	1457 (0.069)	947 (0.13)
fibre	7	1426 (0.08)	1067 (0.1)	1612 (0.066)
flexible	7	1426 (0.08)	844 (0.14)	#SAKNAS!
fourth	7	1426 (0.08)	844 (0.14)	1180 (0.1)
fracture	7	1426 (0.08)	2293 (0.034)	1180 (0.1)
fractures	7	1426 (0.08)	#SAKNAS!	1612 (0.066)
free-living	7	1426 (0.08)	1067 (0.1)	1612 (0.066)
french	7	1426 (0.08)	2293 (0.034)	947 (0.13)
green	7	1426 (0.08)	1067 (0.1)	947 (0.13)
having	7	1426 (0.08)	2293 (0.034)	1180 (0.1)
hip	7	1426 (0.08)	2293 (0.034)	1612 (0.066)

hong	7	1426 (0.08)	1457 (0.069)	768 (0.16)
hygienists	7	1426 (0.08)	2293 (0.034)	1180 (0.1)
hyposalivation	7	1426 (0.08)	1457 (0.069)	947 (0.13)
identify	7	1426 (0.08)	1457 (0.069)	2492 (0.033)
idiopathic	7	1426 (0.08)	1457 (0.069)	1612 (0.066)
imaging	7	1426 (0.08)	1457 (0.069)	1180 (0.1)
independence	7	1426 (0.08)	#SAKNAS!	1612 (0.066)
injection	7	1426 (0.08)	1067 (0.1)	947 (0.13)
intellectual	7	1426 (0.08)	2293 (0.034)	768 (0.16)
intensity	7	1426 (0.08)	2293 (0.034)	1612 (0.066)
interposition	7	1426 (0.08)	1067 (0.1)	947 (0.13)
inversely	7	1426 (0.08)	1457 (0.069)	768 (0.16)
iranian	7	1426 (0.08)	1457 (0.069)	1612 (0.066)
ireland	7	1426 (0.08)	2293 (0.034)	1180 (0.1)
islands	7	1426 (0.08)	1067 (0.1)	#SAKNAS!
junction	7	1426 (0.08)	1457 (0.069)	1180 (0.1)
kong	7	1426 (0.08)	1457 (0.069)	768 (0.16)
laser	7	1426 (0.08)	1457 (0.069)	947 (0.13)
laying	7	1426 (0.08)	2293 (0.034)	1180 (0.1)
lesion	7	1426 (0.08)	2293 (0.034)	947 (0.13)
limitation	7	1426 (0.08)	2293 (0.034)	947 (0.13)
lip	7	1426 (0.08)	1457 (0.069)	2492 (0.033)
locust	7	1426 (0.08)	666 (0.17)	1612 (0.066)
maintain	7	1426 (0.08)	2293 (0.034)	1180 (0.1)
making	7	1426 (0.08)	#SAKNAS!	537 (0.23)
mammalian	7	1426 (0.08)	1457 (0.069)	1180 (0.1)
marginal	7	1426 (0.08)	1457 (0.069)	947 (0.13)
matching	7	1426 (0.08)	#SAKNAS!	1180 (0.1)
matrix	7	1426 (0.08)	844 (0.14)	1612 (0.066)
maximum	7	1426 (0.08)	1457 (0.069)	2492 (0.033)
mediate	7	1426 (0.08)	1457 (0.069)	1180 (0.1)
medication	7	1426 (0.08)	#SAKNAS!	2492 (0.033)
modelling	7	1426 (0.08)	2293 (0.034)	947 (0.13)
modifiable	7	1426 (0.08)	2293 (0.034)	2492 (0.033)
molecular	7	1426 (0.08)	2293 (0.034)	1180 (0.1)
multimorbidity	7	1426 (0.08)	#SAKNAS!	2492 (0.033)
mussel	7	1426 (0.08)	1067 (0.1)	1180 (0.1)
nasal	7	1426 (0.08)	1457 (0.069)	2492 (0.033)
nigeria	7	1426 (0.08)	666 (0.17)	2492 (0.033)
noninvasive	7	1426 (0.08)	666 (0.17)	1612 (0.066)
norway	7	1426 (0.08)	2293 (0.034)	1612 (0.066)
nutritionally	7	1426 (0.08)	844 (0.14)	1612 (0.066)
nutritive	7	1426 (0.08)	844 (0.14)	1612 (0.066)

occupational	7	1426 (0.08)	#SAKNAS!	947 (0.13)
operation	7	1426 (0.08)	844 (0.14)	1612 (0.066)
opportunities	7	1426 (0.08)	1457 (0.069)	1612 (0.066)
orexin	7	1426 (0.08)	844 (0.14)	1180 (0.1)
overdentures	7	1426 (0.08)	666 (0.17)	#SAKNAS!
oxygen	7	1426 (0.08)	1457 (0.069)	1612 (0.066)
panel	7	1426 (0.08)	1067 (0.1)	2492 (0.033)
participating	7	1426 (0.08)	2293 (0.034)	1180 (0.1)
perinatal	7	1426 (0.08)	1067 (0.1)	947 (0.13)
peroral	7	1426 (0.08)	#SAKNAS!	768 (0.16)
phenotype	7	1426 (0.08)	1067 (0.1)	2492 (0.033)
phenotypes	7	1426 (0.08)	#SAKNAS!	1612 (0.066)
pollen	7	1426 (0.08)	1067 (0.1)	1612 (0.066)
practitioners	7	1426 (0.08)	2293 (0.034)	1180 (0.1)
predator	7	1426 (0.08)	844 (0.14)	1612 (0.066)
predatory	7	1426 (0.08)	1457 (0.069)	1180 (0.1)
prevents	7	1426 (0.08)	#SAKNAS!	1180 (0.1)
processes	7	1426 (0.08)	1067 (0.1)	1612 (0.066)
product	7	1426 (0.08)	1457 (0.069)	#SAKNAS!
professional	7	1426 (0.08)	#SAKNAS!	768 (0.16)
progress	7	1426 (0.08)	2293 (0.034)	2492 (0.033)
promotes	7	1426 (0.08)	1457 (0.069)	1180 (0.1)
prophylaxis	7	1426 (0.08)	1067 (0.1)	2492 (0.033)
providers	7	1426 (0.08)	2293 (0.034)	1180 (0.1)
providing	7	1426 (0.08)	2293 (0.034)	647 (0.2)
racial	7	1426 (0.08)	1457 (0.069)	1612 (0.066)
rct	7	1426 (0.08)	#SAKNAS!	947 (0.13)
refeeding	7	1426 (0.08)	666 (0.17)	2492 (0.033)
regional	7	1426 (0.08)	1457 (0.069)	947 (0.13)
regions	7	1426 (0.08)	844 (0.14)	2492 (0.033)
regulate	7	1426 (0.08)	2293 (0.034)	1612 (0.066)
responsive	7	1426 (0.08)	2293 (0.034)	#SAKNAS!
retention	7	1426 (0.08)	844 (0.14)	1612 (0.066)
revision	7	1426 (0.08)	2293 (0.034)	1180 (0.1)
rice	7	1426 (0.08)	1457 (0.069)	#SAKNAS!
right	7	1426 (0.08)	1067 (0.1)	1612 (0.066)
satiety	7	1426 (0.08)	1457 (0.069)	1180 (0.1)
scaling	7	1426 (0.08)	#SAKNAS!	1180 (0.1)
school-age	7	1426 (0.08)	#SAKNAS!	1612 (0.066)
season	7	1426 (0.08)	1067 (0.1)	1180 (0.1)
sedentary	7	1426 (0.08)	2293 (0.034)	2492 (0.033)
signs	7	1426 (0.08)	2293 (0.034)	947 (0.13)
simultaneous	7	1426 (0.08)	666 (0.17)	2492 (0.033)

six		7	1426 (0.08)	#SAKNAS!	1180 (0.1)
smokers		7	1426 (0.08)	#SAKNAS!	647 (0.2)
socio-demographic		7	1426 (0.08)	1457 (0.069)	947 (0.13)
solid		7	1426 (0.08)	2293 (0.034)	647 (0.2)
strains		7	1426 (0.08)	546 (0.21)	#SAKNAS!
stunting		7	1426 (0.08)	1457 (0.069)	1612 (0.066)
supplemental		7	1426 (0.08)	1457 (0.069)	2492 (0.033)
supplementary		7	1426 (0.08)	2293 (0.034)	947 (0.13)
syndromes		7	1426 (0.08)	2293 (0.034)	1612 (0.066)
techniques		7	1426 (0.08)	1067 (0.1)	1180 (0.1)
teenagers		7	1426 (0.08)	844 (0.14)	2492 (0.033)
tephritidae		7	1426 (0.08)	471 (0.24)	#SAKNAS!
tolerance		7	1426 (0.08)	1457 (0.069)	1180 (0.1)
toxins		7	1426 (0.08)	546 (0.21)	2492 (0.033)
trajectories		7	1426 (0.08)	2293 (0.034)	2492 (0.033)
transcranial		7	1426 (0.08)	#SAKNAS!	2492 (0.033)
trauma		7	1426 (0.08)	1457 (0.069)	#SAKNAS!
uganda		7	1426 (0.08)	1067 (0.1)	1612 (0.066)
underlying		7	1426 (0.08)	1067 (0.1)	1180 (0.1)
unhealthy		7	1426 (0.08)	#SAKNAS!	#SAKNAS!
unusual		7	1426 (0.08)	1457 (0.069)	1180 (0.1)
vegetation		7	1426 (0.08)	#SAKNAS!	1180 (0.1)
veterans		7	1426 (0.08)	1067 (0.1)	1612 (0.066)
views		7	1426 (0.08)	2293 (0.034)	947 (0.13)
virulence		7	1426 (0.08)	844 (0.14)	1612 (0.066)
vivo		7	1426 (0.08)	1457 (0.069)	#SAKNAS!
voluntary		7	1426 (0.08)	1067 (0.1)	1612 (0.066)
waist		7	1426 (0.08)	844 (0.14)	2492 (0.033)
wards		7	1426 (0.08)	1457 (0.069)	1612 (0.066)
xanthan		7	1426 (0.08)	#SAKNAS!	1180 (0.1)
yeast		7	1426 (0.08)	1457 (0.069)	1612 (0.066)
younger		7	1426 (0.08)	1457 (0.069)	1180 (0.1)
	2013	6	1618 (0.069)	2293 (0.034)	1612 (0.066)
	2016	6	1618 (0.069)	#SAKNAS!	947 (0.13)
	45721	6	1618 (0.069)	1457 (0.069)	1180 (0.1)
3-year		6	1618 (0.069)	1457 (0.069)	1612 (0.066)
6-year		6	1618 (0.069)	2293 (0.034)	2492 (0.033)
academy		6	1618 (0.069)	844 (0.14)	2492 (0.033)
accumulation		6	1618 (0.069)	1457 (0.069)	1612 (0.066)
activation		6	1618 (0.069)	2293 (0.034)	1612 (0.066)
adaptive		6	1618 (0.069)	666 (0.17)	#SAKNAS!
adenocarcinoma		6	1618 (0.069)	1067 (0.1)	1180 (0.1)
adulthood		6	1618 (0.069)	1457 (0.069)	1180 (0.1)

algal	6	1618 (0.069)	666 (0.17)	2492 (0.033)
allergic	6	1618 (0.069)	1457 (0.069)	1612 (0.066)
alone	6	1618 (0.069)	2293 (0.034)	1180 (0.1)
alter	6	1618 (0.069)	1457 (0.069)	947 (0.13)
ambulatory	6	1618 (0.069)	666 (0.17)	#SAKNAS!
assessments	6	1618 (0.069)	1067 (0.1)	1612 (0.066)
assimilation	6	1618 (0.069)	666 (0.17)	#SAKNAS!
associates	6	1618 (0.069)	#SAKNAS!	1612 (0.066)
assurance	6	1618 (0.069)	1067 (0.1)	1180 (0.1)
barium	6	1618 (0.069)	666 (0.17)	#SAKNAS!
bidirectional	6	1618 (0.069)	2293 (0.034)	1612 (0.066)
biomarker	6	1618 (0.069)	1067 (0.1)	2492 (0.033)
bite	6	1618 (0.069)	2293 (0.034)	2492 (0.033)
born	6	1618 (0.069)	1067 (0.1)	1180 (0.1)
budget	6	1618 (0.069)	1067 (0.1)	1612 (0.066)
burn	6	1618 (0.069)	1067 (0.1)	2492 (0.033)
cactus	6	1618 (0.069)	2293 (0.034)	1180 (0.1)
caterpillars	6	1618 (0.069)	844 (0.14)	2492 (0.033)
centre	6	1618 (0.069)	2293 (0.034)	1180 (0.1)
cessation	6	1618 (0.069)	2293 (0.034)	947 (0.13)
chile	6	1618 (0.069)	844 (0.14)	#SAKNAS!
clinically	6	1618 (0.069)	2293 (0.034)	947 (0.13)
cognition	6	1618 (0.069)	#SAKNAS!	1612 (0.066)
colombia	6	1618 (0.069)	1457 (0.069)	1180 (0.1)
comparisons	6	1618 (0.069)	#SAKNAS!	1180 (0.1)
consecutive	6	1618 (0.069)	1457 (0.069)	1180 (0.1)
construct	6	1618 (0.069)	2293 (0.034)	1180 (0.1)
contribute	6	1618 (0.069)	1457 (0.069)	1612 (0.066)
conversion	6	1618 (0.069)	2293 (0.034)	1612 (0.066)
coronal	6	1618 (0.069)	1457 (0.069)	947 (0.13)
correlate	6	1618 (0.069)	1067 (0.1)	1612 (0.066)
correlations	6	1618 (0.069)	#SAKNAS!	1612 (0.066)
corrosive	6	1618 (0.069)	#SAKNAS!	947 (0.13)
cost	6	1618 (0.069)	1457 (0.069)	1612 (0.066)
country	6	1618 (0.069)	1067 (0.1)	1612 (0.066)
cross-cultural	6	1618 (0.069)	2293 (0.034)	1612 (0.066)
curative	6	1618 (0.069)	844 (0.14)	1612 (0.066)
cytokine	6	1618 (0.069)	1457 (0.069)	2492 (0.033)
damage	6	1618 (0.069)	844 (0.14)	2492 (0.033)
decision	6	1618 (0.069)	1457 (0.069)	2492 (0.033)
decision-making	6	1618 (0.069)	1067 (0.1)	1612 (0.066)
deficit	6	1618 (0.069)	1067 (0.1)	1180 (0.1)
demographic	6	1618 (0.069)	1067 (0.1)	#SAKNAS!

dentist	6	1618 (0.069)	1067 (0.1)	1612 (0.066)
department	6	1618 (0.069)	2293 (0.034)	1180 (0.1)
device	6	1618 (0.069)	2293 (0.034)	1612 (0.066)
difference	6	1618 (0.069)	#SAKNAS!	947 (0.13)
differentiation	6	1618 (0.069)	1067 (0.1)	1612 (0.066)
dilatation	6	1618 (0.069)	1067 (0.1)	1180 (0.1)
diurnal	6	1618 (0.069)	844 (0.14)	#SAKNAS!
diverse	6	1618 (0.069)	2293 (0.034)	1612 (0.066)
diverticulum	6	1618 (0.069)	1457 (0.069)	1180 (0.1)
dmft	6	1618 (0.069)	2293 (0.034)	#SAKNAS!
domestic	6	1618 (0.069)	844 (0.14)	#SAKNAS!
dopamine	6	1618 (0.069)	1457 (0.069)	1612 (0.066)
dryness	6	1618 (0.069)	2293 (0.034)	1612 (0.066)
ecosystem	6	1618 (0.069)	1457 (0.069)	1180 (0.1)
el	6	1618 (0.069)	#SAKNAS!	1180 (0.1)
emergency	6	1618 (0.069)	2293 (0.034)	1612 (0.066)
endoscopy	6	1618 (0.069)	1067 (0.1)	2492 (0.033)
enhancing	6	1618 (0.069)	#SAKNAS!	2492 (0.033)
erosive	6	1618 (0.069)	#SAKNAS!	1612 (0.066)
exercises	6	1618 (0.069)	2293 (0.034)	1180 (0.1)
expanding	6	1618 (0.069)	#SAKNAS!	1180 (0.1)
expert	6	1618 (0.069)	1457 (0.069)	947 (0.13)
extent	6	1618 (0.069)	844 (0.14)	1612 (0.066)
external	6	1618 (0.069)	2293 (0.034)	2492 (0.033)
extracts	6	1618 (0.069)	666 (0.17)	2492 (0.033)
familial	6	1618 (0.069)	1067 (0.1)	1180 (0.1)
finnish	6	1618 (0.069)	2293 (0.034)	1180 (0.1)
fixation	6	1618 (0.069)	#SAKNAS!	2492 (0.033)
flies	6	1618 (0.069)	1067 (0.1)	#SAKNAS!
fluorosis	6	1618 (0.069)	1457 (0.069)	1612 (0.066)
focus	6	1618 (0.069)	2293 (0.034)	1612 (0.066)
follow	6	1618 (0.069)	2293 (0.034)	768 (0.16)
fresh	6	1618 (0.069)	1457 (0.069)	1612 (0.066)
gastrojejunostomy	6	1618 (0.069)	2293 (0.034)	1180 (0.1)
generation	6	1618 (0.069)	#SAKNAS!	2492 (0.033)
ghana	6	1618 (0.069)	666 (0.17)	2492 (0.033)
girls	6	1618 (0.069)	1457 (0.069)	1180 (0.1)
grain	6	1618 (0.069)	2293 (0.034)	1180 (0.1)
grass	6	1618 (0.069)	#SAKNAS!	947 (0.13)
greater	6	1618 (0.069)	1457 (0.069)	947 (0.13)
greek	6	1618 (0.069)	1067 (0.1)	2492 (0.033)
gross	6	1618 (0.069)	666 (0.17)	2492 (0.033)
guide	6	1618 (0.069)	1457 (0.069)	947 (0.13)

heat	6	1618 (0.069)	1067 (0.1)	1612 (0.066)
hematopoietic	6	1618 (0.069)	#SAKNAS!	768 (0.16)
hens	6	1618 (0.069)	2293 (0.034)	1180 (0.1)
herbivorous	6	1618 (0.069)	1067 (0.1)	1180 (0.1)
hippocampal	6	1618 (0.069)	2293 (0.034)	1180 (0.1)
hospitals	6	1618 (0.069)	#SAKNAS!	1180 (0.1)
hosts	6	1618 (0.069)	844 (0.14)	#SAKNAS!
hunger	6	1618 (0.069)	1067 (0.1)	1612 (0.066)
hydrocolloids	6	1618 (0.069)	#SAKNAS!	#SAKNAS!
hyperglycemia	6	1618 (0.069)	2293 (0.034)	947 (0.13)
iddsi	6	1618 (0.069)	#SAKNAS!	2492 (0.033)
inclusion	6	1618 (0.069)	#SAKNAS!	1180 (0.1)
induces	6	1618 (0.069)	1457 (0.069)	947 (0.13)
inhibitor	6	1618 (0.069)	1067 (0.1)	1612 (0.066)
injuries	6	1618 (0.069)	1457 (0.069)	1612 (0.066)
innovative	6	1618 (0.069)	#SAKNAS!	1612 (0.066)
intima-media	6	1618 (0.069)	1457 (0.069)	1612 (0.066)
intracerebral	6	1618 (0.069)	2293 (0.034)	1180 (0.1)
intrinsic	6	1618 (0.069)	1457 (0.069)	1612 (0.066)
inverse	6	1618 (0.069)	2293 (0.034)	1612 (0.066)
la	6	1618 (0.069)	1457 (0.069)	947 (0.13)
labrax	6	1618 (0.069)	546 (0.21)	#SAKNAS!
larynx	6	1618 (0.069)	2293 (0.034)	1180 (0.1)
literacy	6	1618 (0.069)	1457 (0.069)	2492 (0.033)
lobster	6	1618 (0.069)	2293 (0.034)	1180 (0.1)
lunch	6	1618 (0.069)	2293 (0.034)	1180 (0.1)
macronutrients	6	1618 (0.069)	1457 (0.069)	1612 (0.066)
manduca	6	1618 (0.069)	666 (0.17)	2492 (0.033)
mangrove	6	1618 (0.069)	1067 (0.1)	1180 (0.1)
mating	6	1618 (0.069)	1457 (0.069)	1612 (0.066)
meat	6	1618 (0.069)	2293 (0.034)	2492 (0.033)
methodology	6	1618 (0.069)	1457 (0.069)	1612 (0.066)
microorganisms	6	1618 (0.069)	2293 (0.034)	1180 (0.1)
mixtures	6	1618 (0.069)	2293 (0.034)	2492 (0.033)
molars	6	1618 (0.069)	1067 (0.1)	1612 (0.066)
mountain	6	1618 (0.069)	2293 (0.034)	1180 (0.1)
mrna	6	1618 (0.069)	666 (0.17)	2492 (0.033)
multi-center	6	1618 (0.069)	2293 (0.034)	1612 (0.066)
mussels	6	1618 (0.069)	666 (0.17)	#SAKNAS!
night	6	1618 (0.069)	1457 (0.069)	1612 (0.066)
nonsurgical	6	1618 (0.069)	1457 (0.069)	1180 (0.1)
nurses	6	1618 (0.069)	1067 (0.1)	1612 (0.066)
online	6	1618 (0.069)	2293 (0.034)	1180 (0.1)

opportunistic	6	1618 (0.069)	2293 (0.034)	1612 (0.066)
osteoarthritis	6	1618 (0.069)	2293 (0.034)	1180 (0.1)
outlet	6	1618 (0.069)	1067 (0.1)	1612 (0.066)
pairs	6	1618 (0.069)	2293 (0.034)	1612 (0.066)
palate	6	1618 (0.069)	844 (0.14)	#SAKNAS!
parotid	6	1618 (0.069)	1457 (0.069)	1180 (0.1)
participation	6	1618 (0.069)	1067 (0.1)	#SAKNAS!
pasture	6	1618 (0.069)	#SAKNAS!	1180 (0.1)
paulo	6	1618 (0.069)	666 (0.17)	2492 (0.033)
perforation	6	1618 (0.069)	666 (0.17)	2492 (0.033)
peru	6	1618 (0.069)	666 (0.17)	#SAKNAS!
plasticity	6	1618 (0.069)	1457 (0.069)	2492 (0.033)
potato	6	1618 (0.069)	1457 (0.069)	1612 (0.066)
powder	6	1618 (0.069)	#SAKNAS!	2492 (0.033)
prader-willi	6	1618 (0.069)	844 (0.14)	1612 (0.066)
preservation	6	1618 (0.069)	2293 (0.034)	1180 (0.1)
private	6	1618 (0.069)	2293 (0.034)	1612 (0.066)
psychiatric	6	1618 (0.069)	2293 (0.034)	1180 (0.1)
puerto	6	1618 (0.069)	1457 (0.069)	947 (0.13)
quantification	6	1618 (0.069)	2293 (0.034)	768 (0.16)
quantity	6	1618 (0.069)	844 (0.14)	2492 (0.033)
quasi-experimental	6	1618 (0.069)	#SAKNAS!	1180 (0.1)
radiologic	6	1618 (0.069)	1457 (0.069)	1612 (0.066)
rapid	6	1618 (0.069)	844 (0.14)	#SAKNAS!
ration	6	1618 (0.069)	1457 (0.069)	1180 (0.1)
raw	6	1618 (0.069)	2293 (0.034)	#SAKNAS!
reactive	6	1618 (0.069)	2293 (0.034)	1612 (0.066)
refugee	6	1618 (0.069)	1457 (0.069)	1180 (0.1)
regulating	6	1618 (0.069)	1067 (0.1)	1612 (0.066)
republic	6	1618 (0.069)	1457 (0.069)	1180 (0.1)
restoration	6	1618 (0.069)	666 (0.17)	#SAKNAS!
reveal	6	1618 (0.069)	#SAKNAS!	768 (0.16)
reveals	6	1618 (0.069)	#SAKNAS!	2492 (0.033)
robotic	6	1618 (0.069)	#SAKNAS!	947 (0.13)
ruminal	6	1618 (0.069)	2293 (0.034)	1180 (0.1)
ruminants	6	1618 (0.069)	666 (0.17)	2492 (0.033)
salmon	6	1618 (0.069)	1067 (0.1)	1180 (0.1)
salvage	6	1618 (0.069)	#SAKNAS!	947 (0.13)
sao	6	1618 (0.069)	666 (0.17)	2492 (0.033)
scientific	6	1618 (0.069)	2293 (0.034)	1180 (0.1)
sexta	6	1618 (0.069)	666 (0.17)	2492 (0.033)
smell	6	1618 (0.069)	#SAKNAS!	1612 (0.066)
smile	6	1618 (0.069)	#SAKNAS!	1612 (0.066)

snack	6	1618 (0.069)	2293 (0.034)	1180 (0.1)
source	6	1618 (0.069)	1067 (0.1)	1612 (0.066)
southwest	6	1618 (0.069)	2293 (0.034)	947 (0.13)
starvation	6	1618 (0.069)	1067 (0.1)	1612 (0.066)
stay	6	1618 (0.069)	1457 (0.069)	1612 (0.066)
stomach	6	1618 (0.069)	1457 (0.069)	1180 (0.1)
styles	6	1618 (0.069)	1067 (0.1)	2492 (0.033)
suspension	6	1618 (0.069)	1067 (0.1)	#SAKNAS!
swallowing-related	6	1618 (0.069)	#SAKNAS!	2492 (0.033)
taiwanese	6	1618 (0.069)	2293 (0.034)	1180 (0.1)
team	6	1618 (0.069)	#SAKNAS!	1612 (0.066)
temporomandibular	6	1618 (0.069)	1457 (0.069)	1612 (0.066)
therapies	6	1618 (0.069)	1457 (0.069)	#SAKNAS!
thickening	6	1618 (0.069)	#SAKNAS!	2492 (0.033)
thyroid	6	1618 (0.069)	1457 (0.069)	1612 (0.066)
tool-10	6	1618 (0.069)	2293 (0.034)	1180 (0.1)
toward	6	1618 (0.069)	2293 (0.034)	1180 (0.1)
toxic	6	1618 (0.069)	844 (0.14)	2492 (0.033)
trajectory	6	1618 (0.069)	1457 (0.069)	2492 (0.033)
transporter	6	1618 (0.069)	1067 (0.1)	2492 (0.033)
trials	6	1618 (0.069)	1457 (0.069)	1180 (0.1)
trout	6	1618 (0.069)	844 (0.14)	#SAKNAS!
ulcers	6	1618 (0.069)	1457 (0.069)	1612 (0.066)
usefulness	6	1618 (0.069)	1457 (0.069)	2492 (0.033)
video	6	1618 (0.069)	1067 (0.1)	2492 (0.033)
vietnam	6	1618 (0.069)	#SAKNAS!	947 (0.13)
visit	6	1618 (0.069)	1457 (0.069)	1612 (0.066)
wearers	6	1618 (0.069)	1067 (0.1)	#SAKNAS!
workshop	6	1618 (0.069)	#SAKNAS!	947 (0.13)
world	6	1618 (0.069)	1457 (0.069)	1612 (0.066)
youth	6	1618 (0.069)	1457 (0.069)	1612 (0.066)
zenker	6	1618 (0.069)	1067 (0.1)	1612 (0.066)
2017	5	1833 (0.057)	#SAKNAS!	1612 (0.066)
2020	5	1833 (0.057)	#SAKNAS!	2492 (0.033)
45820	5	1833 (0.057)	#SAKNAS!	1612 (0.066)
&lt	5	1833 (0.057)	#SAKNAS!	1180 (0.1)
10-year	5	1833 (0.057)	#SAKNAS!	1612 (0.066)
2009-2010	5	1833 (0.057)	#SAKNAS!	947 (0.13)
2009-2012	5	1833 (0.057)	#SAKNAS!	1180 (0.1)
2010-2012	5	1833 (0.057)	#SAKNAS!	768 (0.16)
2015-2018	5	1833 (0.057)	#SAKNAS!	#SAKNAS!
2-year	5	1833 (0.057)	1457 (0.069)	#SAKNAS!
aboriginal	5	1833 (0.057)	2293 (0.034)	1180 (0.1)

abuse	5	1833 (0.057)	1067 (0.1)	1612 (0.066)
acyrthosiphon	5	1833 (0.057)	#SAKNAS!	#SAKNAS!
adhd	5	1833 (0.057)	2293 (0.034)	1180 (0.1)
adiponectin	5	1833 (0.057)	2293 (0.034)	947 (0.13)
advances	5	1833 (0.057)	1457 (0.069)	2492 (0.033)
ages	5	1833 (0.057)	#SAKNAS!	1180 (0.1)
aggregatibacter	5	1833 (0.057)	1457 (0.069)	1612 (0.066)
agrp	5	1833 (0.057)	1457 (0.069)	2492 (0.033)
alimentary	5	1833 (0.057)	1067 (0.1)	1612 (0.066)
allostatic	5	1833 (0.057)	1457 (0.069)	1612 (0.066)
america	5	1833 (0.057)	1457 (0.069)	2492 (0.033)
animals	5	1833 (0.057)	1067 (0.1)	2492 (0.033)
antimicrobial	5	1833 (0.057)	2293 (0.034)	2492 (0.033)
aphthous	5	1833 (0.057)	1457 (0.069)	1612 (0.066)
apparent	5	1833 (0.057)	2293 (0.034)	2492 (0.033)
applications	5	1833 (0.057)	#SAKNAS!	#SAKNAS!
appropriate	5	1833 (0.057)	1457 (0.069)	1612 (0.066)
aquaculture	5	1833 (0.057)	1457 (0.069)	1612 (0.066)
arctic	5	1833 (0.057)	1067 (0.1)	1612 (0.066)
article	5	1833 (0.057)	1457 (0.069)	1180 (0.1)
atherogenic	5	1833 (0.057)	1067 (0.1)	2492 (0.033)
atrial	5	1833 (0.057)	#SAKNAS!	1612 (0.066)
attributes	5	1833 (0.057)	1457 (0.069)	1612 (0.066)
autologous	5	1833 (0.057)	1457 (0.069)	2492 (0.033)
automatic	5	1833 (0.057)	1457 (0.069)	2492 (0.033)
aversion	5	1833 (0.057)	1457 (0.069)	2492 (0.033)
axis	5	1833 (0.057)	#SAKNAS!	2492 (0.033)
balancing	5	1833 (0.057)	1067 (0.1)	1612 (0.066)
base	5	1833 (0.057)	1067 (0.1)	2492 (0.033)
beneficial	5	1833 (0.057)	844 (0.14)	#SAKNAS!
binding	5	1833 (0.057)	666 (0.17)	#SAKNAS!
boys	5	1833 (0.057)	#SAKNAS!	1612 (0.066)
brainstem	5	1833 (0.057)	1067 (0.1)	2492 (0.033)
bream	5	1833 (0.057)	666 (0.17)	#SAKNAS!
brief	5	1833 (0.057)	2293 (0.034)	2492 (0.033)
britain	5	1833 (0.057)	1067 (0.1)	2492 (0.033)
broiler	5	1833 (0.057)	1457 (0.069)	2492 (0.033)
browsing	5	1833 (0.057)	1067 (0.1)	2492 (0.033)
butterfly	5	1833 (0.057)	1457 (0.069)	2492 (0.033)
calculus	5	1833 (0.057)	1457 (0.069)	#SAKNAS!
california	5	1833 (0.057)	#SAKNAS!	947 (0.13)
call	5	1833 (0.057)	2293 (0.034)	1180 (0.1)
cambodia	5	1833 (0.057)	#SAKNAS!	947 (0.13)

candida	5	1833 (0.057)	1457 (0.069)	#SAKNAS!
cannabinoid	5	1833 (0.057)	#SAKNAS!	1180 (0.1)
carious	5	1833 (0.057)	1457 (0.069)	2492 (0.033)
checklist	5	1833 (0.057)	#SAKNAS!	2492 (0.033)
chemosensory	5	1833 (0.057)	2293 (0.034)	1180 (0.1)
chicken	5	1833 (0.057)	2293 (0.034)	1612 (0.066)
chief	5	1833 (0.057)	#SAKNAS!	#SAKNAS!
childbearing	5	1833 (0.057)	1457 (0.069)	1612 (0.066)
clients	5	1833 (0.057)	1457 (0.069)	1180 (0.1)
cluster-randomized	5	1833 (0.057)	2293 (0.034)	1180 (0.1)
colon	5	1833 (0.057)	1067 (0.1)	1612 (0.066)
colonies	5	1833 (0.057)	2293 (0.034)	2492 (0.033)
colorectal	5	1833 (0.057)	#SAKNAS!	1180 (0.1)
competition	5	1833 (0.057)	1457 (0.069)	2492 (0.033)
composite	5	1833 (0.057)	2293 (0.034)	#SAKNAS!
concerns	5	1833 (0.057)	#SAKNAS!	947 (0.13)
concomitant	5	1833 (0.057)	2293 (0.034)	1612 (0.066)
connectivity	5	1833 (0.057)	#SAKNAS!	1180 (0.1)
consistency	5	1833 (0.057)	1457 (0.069)	1180 (0.1)
coral	5	1833 (0.057)	2293 (0.034)	947 (0.13)
cord	5	1833 (0.057)	2293 (0.034)	2492 (0.033)
cost-effectiveness	5	1833 (0.057)	#SAKNAS!	947 (0.13)
counts	5	1833 (0.057)	1067 (0.1)	1612 (0.066)
crp	5	1833 (0.057)	1457 (0.069)	1612 (0.066)
decade	5	1833 (0.057)	#SAKNAS!	2492 (0.033)
deep	5	1833 (0.057)	#SAKNAS!	947 (0.13)
defense	5	1833 (0.057)	2293 (0.034)	1180 (0.1)
deletion	5	1833 (0.057)	#SAKNAS!	768 (0.16)
derivation	5	1833 (0.057)	844 (0.14)	#SAKNAS!
desert	5	1833 (0.057)	1067 (0.1)	1612 (0.066)
determining	5	1833 (0.057)	2293 (0.034)	1180 (0.1)
devices	5	1833 (0.057)	#SAKNAS!	#SAKNAS!
dicentrarchus	5	1833 (0.057)	666 (0.17)	#SAKNAS!
discomfort	5	1833 (0.057)	#SAKNAS!	1180 (0.1)
disturbance	5	1833 (0.057)	#SAKNAS!	#SAKNAS!
dogs	5	1833 (0.057)	1457 (0.069)	1180 (0.1)
dominance	5	1833 (0.057)	1067 (0.1)	1612 (0.066)
down	5	1833 (0.057)	#SAKNAS!	1180 (0.1)
dwelling	5	1833 (0.057)	#SAKNAS!	1180 (0.1)
dysphagia-oriented	5	1833 (0.057)	2293 (0.034)	#SAKNAS!
dystrophic	5	1833 (0.057)	844 (0.14)	2492 (0.033)
eat-10	5	1833 (0.057)	2293 (0.034)	1612 (0.066)
economic	5	1833 (0.057)	#SAKNAS!	1612 (0.066)

edible	5	1833 (0.057)	#SAKNAS!	1612 (0.066)
egyptian	5	1833 (0.057)	2293 (0.034)	#SAKNAS!
electronic	5	1833 (0.057)	2293 (0.034)	1180 (0.1)
elite	5	1833 (0.057)	2293 (0.034)	1180 (0.1)
emerging	5	1833 (0.057)	1457 (0.069)	2492 (0.033)
endovascular	5	1833 (0.057)	#SAKNAS!	1180 (0.1)
england	5	1833 (0.057)	1067 (0.1)	2492 (0.033)
enhances	5	1833 (0.057)	#SAKNAS!	1612 (0.066)
enzyme	5	1833 (0.057)	1457 (0.069)	1180 (0.1)
eruption	5	1833 (0.057)	844 (0.14)	2492 (0.033)
esophagogastric	5	1833 (0.057)	1457 (0.069)	#SAKNAS!
establishing	5	1833 (0.057)	2293 (0.034)	1612 (0.066)
estimate	5	1833 (0.057)	2293 (0.034)	2492 (0.033)
estimation	5	1833 (0.057)	#SAKNAS!	1612 (0.066)
ewes	5	1833 (0.057)	2293 (0.034)	1612 (0.066)
excretion	5	1833 (0.057)	1457 (0.069)	1180 (0.1)
experiments	5	1833 (0.057)	844 (0.14)	2492 (0.033)
explorative	5	1833 (0.057)	2293 (0.034)	1612 (0.066)
facial	5	1833 (0.057)	1457 (0.069)	2492 (0.033)
facility	5	1833 (0.057)	1457 (0.069)	#SAKNAS!
factor-帢	5	1833 (0.057)	666 (0.17)	#SAKNAS!
fiberoptic	5	1833 (0.057)	2293 (0.034)	2492 (0.033)
fibrillation	5	1833 (0.057)	#SAKNAS!	1612 (0.066)
fluids	5	1833 (0.057)	1457 (0.069)	2492 (0.033)
fluoridation	5	1833 (0.057)	1457 (0.069)	1180 (0.1)
food-based	5	1833 (0.057)	1067 (0.1)	2492 (0.033)
fto	5	1833 (0.057)	1457 (0.069)	1180 (0.1)
fungal	5	1833 (0.057)	1457 (0.069)	#SAKNAS!
gastro-esophageal	5	1833 (0.057)	1457 (0.069)	2492 (0.033)
genetics	5	1833 (0.057)	1457 (0.069)	1612 (0.066)
genotype	5	1833 (0.057)	1067 (0.1)	#SAKNAS!
given	5	1833 (0.057)	844 (0.14)	2492 (0.033)
glands	5	1833 (0.057)	2293 (0.034)	2492 (0.033)
glycaemic	5	1833 (0.057)	2293 (0.034)	1612 (0.066)
grasshopper	5	1833 (0.057)	844 (0.14)	2492 (0.033)
gravis	5	1833 (0.057)	1457 (0.069)	#SAKNAS!
guangzhou	5	1833 (0.057)	#SAKNAS!	1180 (0.1)
hamburg	5	1833 (0.057)	#SAKNAS!	2492 (0.033)
health-promoting	5	1833 (0.057)	1067 (0.1)	2492 (0.033)
heavy	5	1833 (0.057)	2293 (0.034)	2492 (0.033)
heifers	5	1833 (0.057)	2293 (0.034)	1612 (0.066)
herbivory	5	1833 (0.057)	#SAKNAS!	947 (0.13)
hernias	5	1833 (0.057)	1067 (0.1)	#SAKNAS!

high-sensitivity	5	1833 (0.057)	2293 (0.034)	1612 (0.066)
hours	5	1833 (0.057)	2293 (0.034)	2492 (0.033)
housing	5	1833 (0.057)	844 (0.14)	2492 (0.033)
huntington	5	1833 (0.057)	1067 (0.1)	2492 (0.033)
hybrid	5	1833 (0.057)	#SAKNAS!	1180 (0.1)
hypertensive	5	1833 (0.057)	#SAKNAS!	1612 (0.066)
hyperuricemia	5	1833 (0.057)	#SAKNAS!	1612 (0.066)
hypothesis	5	1833 (0.057)	1067 (0.1)	1612 (0.066)
icu	5	1833 (0.057)	#SAKNAS!	1612 (0.066)
illness	5	1833 (0.057)	1457 (0.069)	2492 (0.033)
immigrant	5	1833 (0.057)	1457 (0.069)	1612 (0.066)
implant-supported	5	1833 (0.057)	1457 (0.069)	#SAKNAS!
imrt	5	1833 (0.057)	#SAKNAS!	947 (0.13)
individually	5	1833 (0.057)	1067 (0.1)	2492 (0.033)
industry	5	1833 (0.057)	#SAKNAS!	1612 (0.066)
inhibition	5	1833 (0.057)	1457 (0.069)	2492 (0.033)
inoperable	5	1833 (0.057)	1067 (0.1)	1612 (0.066)
institution	5	1833 (0.057)	#SAKNAS!	768 (0.16)
instrument	5	1833 (0.057)	1067 (0.1)	2492 (0.033)
integrating	5	1833 (0.057)	2293 (0.034)	1180 (0.1)
integrative	5	1833 (0.057)	2293 (0.034)	1612 (0.066)
interactive	5	1833 (0.057)	2293 (0.034)	1612 (0.066)
intermittent	5	1833 (0.057)	1457 (0.069)	#SAKNAS!
interventional	5	1833 (0.057)	2293 (0.034)	#SAKNAS!
intolerance	5	1833 (0.057)	#SAKNAS!	1180 (0.1)
intrauterine	5	1833 (0.057)	666 (0.17)	#SAKNAS!
ischaemic	5	1833 (0.057)	1457 (0.069)	1612 (0.066)
isotopes	5	1833 (0.057)	844 (0.14)	2492 (0.033)
kenya	5	1833 (0.057)	#SAKNAS!	1180 (0.1)
latinos	5	1833 (0.057)	#SAKNAS!	768 (0.16)
leads	5	1833 (0.057)	2293 (0.034)	1612 (0.066)
leaves	5	1833 (0.057)	1067 (0.1)	1612 (0.066)
lemur	5	1833 (0.057)	844 (0.14)	2492 (0.033)
lemurs	5	1833 (0.057)	666 (0.17)	#SAKNAS!
light	5	1833 (0.057)	2293 (0.034)	2492 (0.033)
limb	5	1833 (0.057)	#SAKNAS!	2492 (0.033)
litter	5	1833 (0.057)	1067 (0.1)	2492 (0.033)
lockdown	5	1833 (0.057)	#SAKNAS!	#SAKNAS!
locomotor	5	1833 (0.057)	844 (0.14)	#SAKNAS!
main	5	1833 (0.057)	#SAKNAS!	1180 (0.1)
malignancies	5	1833 (0.057)	2293 (0.034)	1180 (0.1)
mandible	5	1833 (0.057)	2293 (0.034)	2492 (0.033)
mapping	5	1833 (0.057)	#SAKNAS!	947 (0.13)

matters	5	1833 (0.057)	1457 (0.069)	1612 (0.066)
mediators	5	1833 (0.057)	2293 (0.034)	1612 (0.066)
melatonin	5	1833 (0.057)	1457 (0.069)	2492 (0.033)
menopausal	5	1833 (0.057)	#SAKNAS!	1612 (0.066)
messages	5	1833 (0.057)	1457 (0.069)	1612 (0.066)
metals	5	1833 (0.057)	2293 (0.034)	1612 (0.066)
migration	5	1833 (0.057)	1457 (0.069)	1612 (0.066)
military	5	1833 (0.057)	1457 (0.069)	#SAKNAS!
minerals	5	1833 (0.057)	#SAKNAS!	1612 (0.066)
minimally	5	1833 (0.057)	2293 (0.034)	1612 (0.066)
minimum	5	1833 (0.057)	2293 (0.034)	#SAKNAS!
mitochondrial	5	1833 (0.057)	#SAKNAS!	1612 (0.066)
mixing	5	1833 (0.057)	1067 (0.1)	#SAKNAS!
module	5	1833 (0.057)	2293 (0.034)	2492 (0.033)
movement	5	1833 (0.057)	1457 (0.069)	1612 (0.066)
municipality	5	1833 (0.057)	#SAKNAS!	1180 (0.1)
myasthenia	5	1833 (0.057)	1457 (0.069)	#SAKNAS!
mytilus	5	1833 (0.057)	844 (0.14)	2492 (0.033)
nature	5	1833 (0.057)	1067 (0.1)	#SAKNAS!
necrotizing	5	1833 (0.057)	844 (0.14)	#SAKNAS!
nellore	5	1833 (0.057)	#SAKNAS!	1612 (0.066)
neurologic	5	1833 (0.057)	844 (0.14)	#SAKNAS!
neurone	5	1833 (0.057)	1067 (0.1)	1612 (0.066)
newborns	5	1833 (0.057)	1457 (0.069)	1612 (0.066)
nocturnal	5	1833 (0.057)	844 (0.14)	#SAKNAS!
normal-weight	5	1833 (0.057)	2293 (0.034)	1612 (0.066)
normative	5	1833 (0.057)	1067 (0.1)	2492 (0.033)
ocean	5	1833 (0.057)	#SAKNAS!	2492 (0.033)
oesophagectomy	5	1833 (0.057)	1457 (0.069)	1612 (0.066)
oesophagus	5	1833 (0.057)	844 (0.14)	2492 (0.033)
office	5	1833 (0.057)	1067 (0.1)	1612 (0.066)
oldest	5	1833 (0.057)	1067 (0.1)	2492 (0.033)
oncological	5	1833 (0.057)	1457 (0.069)	#SAKNAS!
options	5	1833 (0.057)	666 (0.17)	#SAKNAS!
organization	5	1833 (0.057)	1457 (0.069)	2492 (0.033)
origin	5	1833 (0.057)	844 (0.14)	2492 (0.033)
oxide	5	1833 (0.057)	2293 (0.034)	#SAKNAS!
pacific	5	1833 (0.057)	#SAKNAS!	947 (0.13)
paper	5	1833 (0.057)	2293 (0.034)	1180 (0.1)
parasitoid	5	1833 (0.057)	1067 (0.1)	1612 (0.066)
parts	5	1833 (0.057)	1067 (0.1)	2492 (0.033)
pathogenesis	5	1833 (0.057)	844 (0.14)	2492 (0.033)
pathogenic	5	1833 (0.057)	2293 (0.034)	1612 (0.066)

pathologists	5	1833 (0.057)	2293 (0.034)	2492 (0.033)
peritoneal	5	1833 (0.057)	2293 (0.034)	1612 (0.066)
personal	5	1833 (0.057)	1067 (0.1)	#SAKNAS!
photodynamic	5	1833 (0.057)	1457 (0.069)	#SAKNAS!
plan	5	1833 (0.057)	#SAKNAS!	1180 (0.1)
plastic	5	1833 (0.057)	844 (0.14)	2492 (0.033)
policy	5	1833 (0.057)	1067 (0.1)	2492 (0.033)
polish	5	1833 (0.057)	2293 (0.034)	1612 (0.066)
polypharmacy	5	1833 (0.057)	#SAKNAS!	2492 (0.033)
postnatal	5	1833 (0.057)	844 (0.14)	2492 (0.033)
poverty	5	1833 (0.057)	1457 (0.069)	1180 (0.1)
pre	5	1833 (0.057)	#SAKNAS!	1612 (0.066)
prediabetes	5	1833 (0.057)	#SAKNAS!	1180 (0.1)
preschool-aged	5	1833 (0.057)	1457 (0.069)	2492 (0.033)
pretreatment	5	1833 (0.057)	2293 (0.034)	947 (0.13)
probiotics	5	1833 (0.057)	1457 (0.069)	2492 (0.033)
productivity	5	1833 (0.057)	#SAKNAS!	#SAKNAS!
progressive	5	1833 (0.057)	2293 (0.034)	2492 (0.033)
protein-energy	5	1833 (0.057)	1067 (0.1)	1612 (0.066)
proteins	5	1833 (0.057)	1457 (0.069)	1612 (0.066)
protocols	5	1833 (0.057)	1457 (0.069)	2492 (0.033)
psychosocial	5	1833 (0.057)	1457 (0.069)	1612 (0.066)
pureed	5	1833 (0.057)	#SAKNAS!	1612 (0.066)
radiographic	5	1833 (0.057)	1457 (0.069)	1612 (0.066)
range	5	1833 (0.057)	1457 (0.069)	1612 (0.066)
rationale	5	1833 (0.057)	2293 (0.034)	947 (0.13)
real-world	5	1833 (0.057)	#SAKNAS!	2492 (0.033)
reflect	5	1833 (0.057)	1457 (0.069)	2492 (0.033)
refractory	5	1833 (0.057)	2293 (0.034)	1612 (0.066)
regain	5	1833 (0.057)	2293 (0.034)	1180 (0.1)
relates	5	1833 (0.057)	1067 (0.1)	#SAKNAS!
relations	5	1833 (0.057)	1457 (0.069)	1180 (0.1)
relevant	5	1833 (0.057)	2293 (0.034)	2492 (0.033)
removable	5	1833 (0.057)	1457 (0.069)	2492 (0.033)
repetitive	5	1833 (0.057)	#SAKNAS!	#SAKNAS!
replacer	5	1833 (0.057)	#SAKNAS!	2492 (0.033)
reports	5	1833 (0.057)	1457 (0.069)	2492 (0.033)
residual	5	1833 (0.057)	1457 (0.069)	2492 (0.033)
responsiveness	5	1833 (0.057)	2293 (0.034)	1612 (0.066)
revisiting	5	1833 (0.057)	1067 (0.1)	1612 (0.066)
risk-factors	5	1833 (0.057)	666 (0.17)	#SAKNAS!
salt	5	1833 (0.057)	1457 (0.069)	1612 (0.066)
san	5	1833 (0.057)	1457 (0.069)	1180 (0.1)

sars-cov-2	5	1833 (0.057)	#SAKNAS!	2492 (0.033)
science	5	1833 (0.057)	2293 (0.034)	1612 (0.066)
scores	5	1833 (0.057)	2293 (0.034)	1612 (0.066)
security	5	1833 (0.057)	#SAKNAS!	1612 (0.066)
self-expandable	5	1833 (0.057)	1457 (0.069)	1612 (0.066)
sensing	5	1833 (0.057)	#SAKNAS!	1180 (0.1)
sexually	5	1833 (0.057)	2293 (0.034)	1180 (0.1)
shift	5	1833 (0.057)	2293 (0.034)	1612 (0.066)
signal	5	1833 (0.057)	1457 (0.069)	1180 (0.1)
significantly	5	1833 (0.057)	2293 (0.034)	2492 (0.033)
sites	5	1833 (0.057)	1457 (0.069)	2492 (0.033)
skin	5	1833 (0.057)	2293 (0.034)	1612 (0.066)
snacks	5	1833 (0.057)	844 (0.14)	2492 (0.033)
solutions	5	1833 (0.057)	#SAKNAS!	2492 (0.033)
soybean	5	1833 (0.057)	2293 (0.034)	#SAKNAS!
spiny	5	1833 (0.057)	1457 (0.069)	1612 (0.066)
spray	5	1833 (0.057)	#SAKNAS!	1180 (0.1)
stability	5	1833 (0.057)	#SAKNAS!	947 (0.13)
steers	5	1833 (0.057)	#SAKNAS!	2492 (0.033)
stenting	5	1833 (0.057)	1457 (0.069)	1612 (0.066)
stomatitis	5	1833 (0.057)	1457 (0.069)	2492 (0.033)
stroke-associated	5	1833 (0.057)	2293 (0.034)	#SAKNAS!
structures	5	1833 (0.057)	2293 (0.034)	1180 (0.1)
studying	5	1833 (0.057)	1067 (0.1)	#SAKNAS!
substitute	5	1833 (0.057)	1067 (0.1)	#SAKNAS!
sustainable	5	1833 (0.057)	#SAKNAS!	2492 (0.033)
sweeteners	5	1833 (0.057)	1067 (0.1)	2492 (0.033)
targeting	5	1833 (0.057)	2293 (0.034)	1612 (0.066)
television	5	1833 (0.057)	1457 (0.069)	1180 (0.1)
theory	5	1833 (0.057)	1067 (0.1)	2492 (0.033)
therapists	5	1833 (0.057)	#SAKNAS!	1612 (0.066)
thickener	5	1833 (0.057)	#SAKNAS!	1180 (0.1)
toddler	5	1833 (0.057)	1067 (0.1)	#SAKNAS!
toxicities	5	1833 (0.057)	#SAKNAS!	2492 (0.033)
trace	5	1833 (0.057)	1457 (0.069)	2492 (0.033)
tracheostomy	5	1833 (0.057)	#SAKNAS!	2492 (0.033)
translation	5	1833 (0.057)	1457 (0.069)	2492 (0.033)
tumors	5	1833 (0.057)	1067 (0.1)	#SAKNAS!
two-year	5	1833 (0.057)	#SAKNAS!	1612 (0.066)
ulcerative	5	1833 (0.057)	1067 (0.1)	2492 (0.033)
useful	5	1833 (0.057)	1067 (0.1)	1612 (0.066)
valid	5	1833 (0.057)	1457 (0.069)	1612 (0.066)
view	5	1833 (0.057)	2293 (0.034)	947 (0.13)

visceral		5	1833 (0.057)	2293 (0.034)	2492 (0.033)
viscosity		5	1833 (0.057)	1457 (0.069)	1612 (0.066)
volatile		5	1833 (0.057)	2293 (0.034)	2492 (0.033)
weeks		5	1833 (0.057)	1457 (0.069)	2492 (0.033)
west		5	1833 (0.057)	1457 (0.069)	1612 (0.066)
whey		5	1833 (0.057)	#SAKNAS!	947 (0.13)
xxi		5	1833 (0.057)	#SAKNAS!	#SAKNAS!
you		5	1833 (0.057)	#SAKNAS!	1180 (0.1)
	1999	4	2153 (0.046)	#SAKNAS!	1180 (0.1)
2017-2020		4	2153 (0.046)	#SAKNAS!	#SAKNAS!
4-year		4	2153 (0.046)	#SAKNAS!	1180 (0.1)
act		4	2153 (0.046)	#SAKNAS!	1612 (0.066)
actions		4	2153 (0.046)	2293 (0.034)	1180 (0.1)
acutely		4	2153 (0.046)	2293 (0.034)	2492 (0.033)
adaptations		4	2153 (0.046)	1457 (0.069)	1612 (0.066)
adipokines		4	2153 (0.046)	1457 (0.069)	2492 (0.033)
advertisements		4	2153 (0.046)	2293 (0.034)	1180 (0.1)
agenda		4	2153 (0.046)	1457 (0.069)	1612 (0.066)
aid		4	2153 (0.046)	2293 (0.034)	2492 (0.033)
algae		4	2153 (0.046)	2293 (0.034)	2492 (0.033)
alteration		4	2153 (0.046)	1067 (0.1)	2492 (0.033)
altering		4	2153 (0.046)	2293 (0.034)	2492 (0.033)
anastomotic		4	2153 (0.046)	1457 (0.069)	1612 (0.066)
antarctic		4	2153 (0.046)	1457 (0.069)	2492 (0.033)
antioxidants		4	2153 (0.046)	1457 (0.069)	1612 (0.066)
apidae		4	2153 (0.046)	1457 (0.069)	2492 (0.033)
apis		4	2153 (0.046)	#SAKNAS!	1180 (0.1)
applicability		4	2153 (0.046)	#SAKNAS!	2492 (0.033)
arab		4	2153 (0.046)	1067 (0.1)	2492 (0.033)
arabian		4	2153 (0.046)	#SAKNAS!	2492 (0.033)
argentina		4	2153 (0.046)	2293 (0.034)	1180 (0.1)
assamese		4	2153 (0.046)	2293 (0.034)	1612 (0.066)
asymptomatic		4	2153 (0.046)	2293 (0.034)	2492 (0.033)
ataxia		4	2153 (0.046)	1457 (0.069)	2492 (0.033)
attention		4	2153 (0.046)	1457 (0.069)	1612 (0.066)
attitude		4	2153 (0.046)	#SAKNAS!	2492 (0.033)
autistic		4	2153 (0.046)	2293 (0.034)	2492 (0.033)
avian		4	2153 (0.046)	1067 (0.1)	2492 (0.033)
avoidance		4	2153 (0.046)	#SAKNAS!	1180 (0.1)
back		4	2153 (0.046)	1067 (0.1)	2492 (0.033)
bat		4	2153 (0.046)	2293 (0.034)	1180 (0.1)
bias		4	2153 (0.046)	#SAKNAS!	1180 (0.1)
bimaculatus		4	2153 (0.046)	#SAKNAS!	947 (0.13)

binge	4	2153 (0.046)	1457 (0.069)	1612 (0.066)
biobank	4	2153 (0.046)	#SAKNAS!	1612 (0.066)
biofilms	4	2153 (0.046)	2293 (0.034)	1180 (0.1)
bitter	4	2153 (0.046)	#SAKNAS!	1612 (0.066)
blenderized	4	2153 (0.046)	#SAKNAS!	1612 (0.066)
bombus	4	2153 (0.046)	2293 (0.034)	#SAKNAS!
border	4	2153 (0.046)	#SAKNAS!	1180 (0.1)
borer	4	2153 (0.046)	1457 (0.069)	1612 (0.066)
branched-chain	4	2153 (0.046)	#SAKNAS!	2492 (0.033)
british	4	2153 (0.046)	1067 (0.1)	#SAKNAS!
bronze	4	2153 (0.046)	#SAKNAS!	947 (0.13)
buffalo	4	2153 (0.046)	1457 (0.069)	2492 (0.033)
building	4	2153 (0.046)	2293 (0.034)	1180 (0.1)
bulls	4	2153 (0.046)	1457 (0.069)	#SAKNAS!
burning	4	2153 (0.046)	2293 (0.034)	#SAKNAS!
cake	4	2153 (0.046)	#SAKNAS!	#SAKNAS!
calf	4	2153 (0.046)	#SAKNAS!	2492 (0.033)
canada	4	2153 (0.046)	1457 (0.069)	2492 (0.033)
candidiasis	4	2153 (0.046)	1457 (0.069)	1612 (0.066)
canine	4	2153 (0.046)	2293 (0.034)	1612 (0.066)
carcinomas	4	2153 (0.046)	1067 (0.1)	#SAKNAS!
cariogenicity	4	2153 (0.046)	2293 (0.034)	2492 (0.033)
caterpillar	4	2153 (0.046)	1067 (0.1)	2492 (0.033)
cause-specific	4	2153 (0.046)	#SAKNAS!	2492 (0.033)
causing	4	2153 (0.046)	#SAKNAS!	2492 (0.033)
caustic	4	2153 (0.046)	1457 (0.069)	1612 (0.066)
celiac	4	2153 (0.046)	2293 (0.034)	#SAKNAS!
century	4	2153 (0.046)	1067 (0.1)	2492 (0.033)
cereal	4	2153 (0.046)	1457 (0.069)	1612 (0.066)
cerebrovascular	4	2153 (0.046)	844 (0.14)	#SAKNAS!
cervus	4	2153 (0.046)	844 (0.14)	#SAKNAS!
changing	4	2153 (0.046)	1457 (0.069)	#SAKNAS!
characterizing	4	2153 (0.046)	2293 (0.034)	1612 (0.066)
chemo	4	2153 (0.046)	2293 (0.034)	1180 (0.1)
circuit	4	2153 (0.046)	#SAKNAS!	1612 (0.066)
cirrhosis	4	2153 (0.046)	#SAKNAS!	947 (0.13)
clinics	4	2153 (0.046)	1067 (0.1)	#SAKNAS!
closely	4	2153 (0.046)	#SAKNAS!	2492 (0.033)
closure	4	2153 (0.046)	1067 (0.1)	#SAKNAS!
co2	4	2153 (0.046)	2293 (0.034)	1180 (0.1)
cocaine	4	2153 (0.046)	1457 (0.069)	2492 (0.033)
cohorts	4	2153 (0.046)	#SAKNAS!	1180 (0.1)
collective	4	2153 (0.046)	1457 (0.069)	2492 (0.033)

committee	4	2153 (0.046)	1067 (0.1)	2492 (0.033)
compensate	4	2153 (0.046)	#SAKNAS!	1180 (0.1)
competitive	4	2153 (0.046)	2293 (0.034)	2492 (0.033)
complaints	4	2153 (0.046)	2293 (0.034)	2492 (0.033)
complexity	4	2153 (0.046)	2293 (0.034)	2492 (0.033)
concern	4	2153 (0.046)	#SAKNAS!	2492 (0.033)
conduit	4	2153 (0.046)	2293 (0.034)	1612 (0.066)
conservation	4	2153 (0.046)	2293 (0.034)	1612 (0.066)
constraints	4	2153 (0.046)	844 (0.14)	#SAKNAS!
consumer	4	2153 (0.046)	#SAKNAS!	1180 (0.1)
contents	4	2153 (0.046)	#SAKNAS!	1180 (0.1)
copd	4	2153 (0.046)	2293 (0.034)	#SAKNAS!
copepods	4	2153 (0.046)	844 (0.14)	#SAKNAS!
coping	4	2153 (0.046)	2293 (0.034)	1612 (0.066)
cortical	4	2153 (0.046)	2293 (0.034)	1612 (0.066)
cortisol	4	2153 (0.046)	844 (0.14)	#SAKNAS!
cotton	4	2153 (0.046)	#SAKNAS!	1612 (0.066)
cough	4	2153 (0.046)	#SAKNAS!	1180 (0.1)
county	4	2153 (0.046)	2293 (0.034)	1180 (0.1)
cricket	4	2153 (0.046)	#SAKNAS!	947 (0.13)
critically	4	2153 (0.046)	#SAKNAS!	#SAKNAS!
crossover	4	2153 (0.046)	2293 (0.034)	2492 (0.033)
curve	4	2153 (0.046)	1457 (0.069)	2492 (0.033)
cyclic	4	2153 (0.046)	1457 (0.069)	2492 (0.033)
danish	4	2153 (0.046)	2293 (0.034)	1180 (0.1)
daphnia	4	2153 (0.046)	1457 (0.069)	2492 (0.033)
decompression	4	2153 (0.046)	#SAKNAS!	1612 (0.066)
defining	4	2153 (0.046)	1457 (0.069)	2492 (0.033)
degeneration	4	2153 (0.046)	2293 (0.034)	1612 (0.066)
degree	4	2153 (0.046)	2293 (0.034)	2492 (0.033)
delay	4	2153 (0.046)	1457 (0.069)	2492 (0.033)
dessert	4	2153 (0.046)	#SAKNAS!	2492 (0.033)
detect	4	2153 (0.046)	#SAKNAS!	2492 (0.033)
detecting	4	2153 (0.046)	#SAKNAS!	1180 (0.1)
determination	4	2153 (0.046)	1067 (0.1)	2492 (0.033)
diagnosing	4	2153 (0.046)	1457 (0.069)	2492 (0.033)
dietetics	4	2153 (0.046)	1067 (0.1)	2492 (0.033)
diet-related	4	2153 (0.046)	#SAKNAS!	1612 (0.066)
differing	4	2153 (0.046)	1067 (0.1)	2492 (0.033)
disaster	4	2153 (0.046)	#SAKNAS!	1180 (0.1)
discrimination	4	2153 (0.046)	1067 (0.1)	2492 (0.033)
disease-specific	4	2153 (0.046)	1457 (0.069)	#SAKNAS!
disparity	4	2153 (0.046)	2293 (0.034)	1612 (0.066)

distal	4	2153 (0.046)	2293 (0.034)	#SAKNAS!
disturbances	4	2153 (0.046)	1457 (0.069)	1612 (0.066)
diverticulectomy	4	2153 (0.046)	1067 (0.1)	2492 (0.033)
diving	4	2153 (0.046)	1067 (0.1)	2492 (0.033)
doing	4	2153 (0.046)	2293 (0.034)	1612 (0.066)
efp	4	2153 (0.046)	#SAKNAS!	1612 (0.066)
elaphus	4	2153 (0.046)	844 (0.14)	#SAKNAS!
electromyographic	4	2153 (0.046)	#SAKNAS!	2492 (0.033)
electromyography	4	2153 (0.046)	1457 (0.069)	2492 (0.033)
element	4	2153 (0.046)	1067 (0.1)	#SAKNAS!
elephant	4	2153 (0.046)	1457 (0.069)	1612 (0.066)
emirates	4	2153 (0.046)	1067 (0.1)	2492 (0.033)
emission	4	2153 (0.046)	1457 (0.069)	#SAKNAS!
endocrine	4	2153 (0.046)	2293 (0.034)	1612 (0.066)
endodontic	4	2153 (0.046)	2293 (0.034)	1612 (0.066)
english	4	2153 (0.046)	1457 (0.069)	1612 (0.066)
ent	4	2153 (0.046)	1067 (0.1)	#SAKNAS!
epidemiologic	4	2153 (0.046)	1457 (0.069)	1612 (0.066)
epigenetic	4	2153 (0.046)	#SAKNAS!	1180 (0.1)
esnupi	4	2153 (0.046)	#SAKNAS!	1180 (0.1)
espen	4	2153 (0.046)	#SAKNAS!	1612 (0.066)
etiology	4	2153 (0.046)	2293 (0.034)	1180 (0.1)
exacerbation	4	2153 (0.046)	2293 (0.034)	1612 (0.066)
existing	4	2153 (0.046)	#SAKNAS!	1612 (0.066)
expectations	4	2153 (0.046)	1457 (0.069)	1612 (0.066)
experiencing	4	2153 (0.046)	1067 (0.1)	#SAKNAS!
explain	4	2153 (0.046)	2293 (0.034)	2492 (0.033)
exposures	4	2153 (0.046)	#SAKNAS!	#SAKNAS!
facilitators	4	2153 (0.046)	2293 (0.034)	#SAKNAS!
falls	4	2153 (0.046)	#SAKNAS!	2492 (0.033)
feeds	4	2153 (0.046)	1067 (0.1)	2492 (0.033)
fees	4	2153 (0.046)	2293 (0.034)	2492 (0.033)
fertilization	4	2153 (0.046)	2293 (0.034)	1612 (0.066)
fifth	4	2153 (0.046)	#SAKNAS!	1180 (0.1)
flavor	4	2153 (0.046)	1067 (0.1)	#SAKNAS!
flemish	4	2153 (0.046)	844 (0.14)	#SAKNAS!
flossing	4	2153 (0.046)	2293 (0.034)	1612 (0.066)
folate	4	2153 (0.046)	1457 (0.069)	#SAKNAS!
formative	4	2153 (0.046)	#SAKNAS!	#SAKNAS!
forms	4	2153 (0.046)	1457 (0.069)	1612 (0.066)
formulas	4	2153 (0.046)	2293 (0.034)	2492 (0.033)
freshwater	4	2153 (0.046)	2293 (0.034)	1180 (0.1)
fructose	4	2153 (0.046)	2293 (0.034)	1180 (0.1)

fully	4	2153 (0.046)	2293 (0.034)	1612 (0.066)
functioning	4	2153 (0.046)	#SAKNAS!	1180 (0.1)
gait	4	2153 (0.046)	#SAKNAS!	1612 (0.066)
galanin	4	2153 (0.046)	1067 (0.1)	2492 (0.033)
gastroenterology	4	2153 (0.046)	2293 (0.034)	2492 (0.033)
gastro-intestinal	4	2153 (0.046)	1457 (0.069)	2492 (0.033)
gastrostomies	4	2153 (0.046)	1067 (0.1)	2492 (0.033)
geese	4	2153 (0.046)	1067 (0.1)	#SAKNAS!
gel	4	2153 (0.046)	#SAKNAS!	1180 (0.1)
gels	4	2153 (0.046)	#SAKNAS!	2492 (0.033)
gerontological	4	2153 (0.046)	1067 (0.1)	#SAKNAS!
glossectomy	4	2153 (0.046)	#SAKNAS!	1180 (0.1)
glutamate	4	2153 (0.046)	2293 (0.034)	2492 (0.033)
glycation	4	2153 (0.046)	2293 (0.034)	1612 (0.066)
gorillas	4	2153 (0.046)	1457 (0.069)	2492 (0.033)
gradient	4	2153 (0.046)	844 (0.14)	#SAKNAS!
grasshoppers	4	2153 (0.046)	1457 (0.069)	#SAKNAS!
grip	4	2153 (0.046)	#SAKNAS!	1612 (0.066)
gryllus	4	2153 (0.046)	#SAKNAS!	947 (0.13)
guangxi	4	2153 (0.046)	#SAKNAS!	1180 (0.1)
gum-based	4	2153 (0.046)	#SAKNAS!	1180 (0.1)
healing	4	2153 (0.046)	2293 (0.034)	1612 (0.066)
healthier	4	2153 (0.046)	2293 (0.034)	#SAKNAS!
hepatic	4	2153 (0.046)	1457 (0.069)	#SAKNAS!
hepatology	4	2153 (0.046)	2293 (0.034)	2492 (0.033)
hereditary	4	2153 (0.046)	2293 (0.034)	2492 (0.033)
heterogeneity	4	2153 (0.046)	844 (0.14)	#SAKNAS!
histological	4	2153 (0.046)	#SAKNAS!	1612 (0.066)
honeybee	4	2153 (0.046)	2293 (0.034)	1180 (0.1)
hormonal	4	2153 (0.046)	1067 (0.1)	#SAKNAS!
households	4	2153 (0.046)	#SAKNAS!	1612 (0.066)
hpv	4	2153 (0.046)	2293 (0.034)	1180 (0.1)
hydrocarbons	4	2153 (0.046)	#SAKNAS!	2492 (0.033)
hyoid	4	2153 (0.046)	1457 (0.069)	1612 (0.066)
hypoplasia	4	2153 (0.046)	1457 (0.069)	2492 (0.033)
immunological	4	2153 (0.046)	1457 (0.069)	2492 (0.033)
impedance	4	2153 (0.046)	#SAKNAS!	1612 (0.066)
incremental	4	2153 (0.046)	1457 (0.069)	2492 (0.033)
infant-feeding	4	2153 (0.046)	1457 (0.069)	1612 (0.066)
ingredients	4	2153 (0.046)	2293 (0.034)	2492 (0.033)
in-hospital	4	2153 (0.046)	2293 (0.034)	#SAKNAS!
initial	4	2153 (0.046)	1067 (0.1)	#SAKNAS!
initiation	4	2153 (0.046)	2293 (0.034)	#SAKNAS!

institute	4	2153 (0.046)	#SAKNAS!	1612 (0.066)
insufficient	4	2153 (0.046)	1457 (0.069)	1612 (0.066)
interdisciplinary	4	2153 (0.046)	2293 (0.034)	1180 (0.1)
interrelationship	4	2153 (0.046)	1457 (0.069)	2492 (0.033)
interview	4	2153 (0.046)	2293 (0.034)	#SAKNAS!
intraoral	4	2153 (0.046)	#SAKNAS!	1612 (0.066)
intubation	4	2153 (0.046)	2293 (0.034)	1612 (0.066)
invasion	4	2153 (0.046)	2293 (0.034)	1612 (0.066)
involving	4	2153 (0.046)	1457 (0.069)	1612 (0.066)
isotope	4	2153 (0.046)	1457 (0.069)	2492 (0.033)
israel	4	2153 (0.046)	2293 (0.034)	1612 (0.066)
japonica	4	2153 (0.046)	1067 (0.1)	#SAKNAS!
jaw	4	2153 (0.046)	2293 (0.034)	2492 (0.033)
jejunal	4	2153 (0.046)	2293 (0.034)	1612 (0.066)
juice	4	2153 (0.046)	2293 (0.034)	1612 (0.066)
juveniles	4	2153 (0.046)	2293 (0.034)	1180 (0.1)
karachi	4	2153 (0.046)	#SAKNAS!	2492 (0.033)
kinase	4	2153 (0.046)	1067 (0.1)	#SAKNAS!
kindergarten	4	2153 (0.046)	#SAKNAS!	2492 (0.033)
knee	4	2153 (0.046)	#SAKNAS!	2492 (0.033)
labor	4	2153 (0.046)	1457 (0.069)	1612 (0.066)
landscape	4	2153 (0.046)	2293 (0.034)	2492 (0.033)
later	4	2153 (0.046)	2293 (0.034)	1180 (0.1)
latino	4	2153 (0.046)	2293 (0.034)	1180 (0.1)
leadership	4	2153 (0.046)	#SAKNAS!	1612 (0.066)
lean	4	2153 (0.046)	1067 (0.1)	2492 (0.033)
left	4	2153 (0.046)	1067 (0.1)	#SAKNAS!
lichen	4	2153 (0.046)	2293 (0.034)	1612 (0.066)
life-style	4	2153 (0.046)	1067 (0.1)	#SAKNAS!
lifestyles	4	2153 (0.046)	#SAKNAS!	1612 (0.066)
lifetime	4	2153 (0.046)	1457 (0.069)	#SAKNAS!
linking	4	2153 (0.046)	2293 (0.034)	2492 (0.033)
lipids	4	2153 (0.046)	2293 (0.034)	1180 (0.1)
location	4	2153 (0.046)	#SAKNAS!	2492 (0.033)
long-evans	4	2153 (0.046)	844 (0.14)	#SAKNAS!
madagascar	4	2153 (0.046)	1457 (0.069)	2492 (0.033)
magnesium	4	2153 (0.046)	1457 (0.069)	#SAKNAS!
maintaining	4	2153 (0.046)	2293 (0.034)	1612 (0.066)
maize	4	2153 (0.046)	1457 (0.069)	#SAKNAS!
malaysian	4	2153 (0.046)	2293 (0.034)	1612 (0.066)
managing	4	2153 (0.046)	1457 (0.069)	1612 (0.066)
manipulation	4	2153 (0.046)	1067 (0.1)	2492 (0.033)
manometric	4	2153 (0.046)	2293 (0.034)	2492 (0.033)

masseter	4	2153 (0.046)	#SAKNAS!	2492 (0.033)
material	4	2153 (0.046)	2293 (0.034)	1612 (0.066)
mealtimes	4	2153 (0.046)	#SAKNAS!	947 (0.13)
medicaid	4	2153 (0.046)	2293 (0.034)	#SAKNAS!
medications	4	2153 (0.046)	#SAKNAS!	1612 (0.066)
medieval	4	2153 (0.046)	#SAKNAS!	1180 (0.1)
medium	4	2153 (0.046)	1067 (0.1)	#SAKNAS!
melanin-concentrating	4	2153 (0.046)	1067 (0.1)	2492 (0.033)
melanocortin	4	2153 (0.046)	1067 (0.1)	#SAKNAS!
mellifera	4	2153 (0.046)	#SAKNAS!	1180 (0.1)
memory	4	2153 (0.046)	#SAKNAS!	1180 (0.1)
metabolomic	4	2153 (0.046)	2293 (0.034)	#SAKNAS!
methane	4	2153 (0.046)	#SAKNAS!	#SAKNAS!
micronutrients	4	2153 (0.046)	2293 (0.034)	2492 (0.033)
mining	4	2153 (0.046)	2293 (0.034)	1180 (0.1)
mixotrophic	4	2153 (0.046)	1457 (0.069)	2492 (0.033)
moth	4	2153 (0.046)	844 (0.14)	#SAKNAS!
multidimensional	4	2153 (0.046)	1067 (0.1)	#SAKNAS!
murine	4	2153 (0.046)	1457 (0.069)	2492 (0.033)
myositis	4	2153 (0.046)	#SAKNAS!	1612 (0.066)
naive	4	2153 (0.046)	#SAKNAS!	947 (0.13)
nectar	4	2153 (0.046)	#SAKNAS!	1180 (0.1)
negative	4	2153 (0.046)	2293 (0.034)	1612 (0.066)
neo-adjuvant	4	2153 (0.046)	2293 (0.034)	1180 (0.1)
neolithic	4	2153 (0.046)	2293 (0.034)	2492 (0.033)
neonates	4	2153 (0.046)	1457 (0.069)	2492 (0.033)
neophobia	4	2153 (0.046)	#SAKNAS!	1612 (0.066)
nerve	4	2153 (0.046)	#SAKNAS!	1612 (0.066)
nesfatin-1	4	2153 (0.046)	2293 (0.034)	1612 (0.066)
neurodegenerative	4	2153 (0.046)	2293 (0.034)	2492 (0.033)
neuron	4	2153 (0.046)	1457 (0.069)	1612 (0.066)
neurotrophic	4	2153 (0.046)	1067 (0.1)	#SAKNAS!
neutrophil	4	2153 (0.046)	2293 (0.034)	2492 (0.033)
non-alcoholic	4	2153 (0.046)	2293 (0.034)	1612 (0.066)
noncommunicable	4	2153 (0.046)	2293 (0.034)	1612 (0.066)
non-smoking	4	2153 (0.046)	1457 (0.069)	1612 (0.066)
northeast	4	2153 (0.046)	1457 (0.069)	2492 (0.033)
northwest	4	2153 (0.046)	2293 (0.034)	1612 (0.066)
npy	4	2153 (0.046)	2293 (0.034)	2492 (0.033)
nuclear	4	2153 (0.046)	2293 (0.034)	2492 (0.033)
nutritionday	4	2153 (0.046)	2293 (0.034)	1180 (0.1)
nymphs	4	2153 (0.046)	2293 (0.034)	1180 (0.1)
occlusion	4	2153 (0.046)	#SAKNAS!	1612 (0.066)

ohrqol	4	2153 (0.046)	2293 (0.034)	#SAKNAS!
omega-3	4	2153 (0.046)	1457 (0.069)	#SAKNAS!
oncologic	4	2153 (0.046)	#SAKNAS!	1612 (0.066)
opening	4	2153 (0.046)	1457 (0.069)	2492 (0.033)
operative	4	2153 (0.046)	2293 (0.034)	2492 (0.033)
optimizing	4	2153 (0.046)	1457 (0.069)	2492 (0.033)
oreochromis	4	2153 (0.046)	2293 (0.034)	1612 (0.066)
organ	4	2153 (0.046)	#SAKNAS!	2492 (0.033)
organisms	4	2153 (0.046)	1457 (0.069)	1612 (0.066)
ornatus	4	2153 (0.046)	#SAKNAS!	1612 (0.066)
oro-pharyngeal	4	2153 (0.046)	2293 (0.034)	2492 (0.033)
oropharynx	4	2153 (0.046)	2293 (0.034)	1612 (0.066)
orthognathic	4	2153 (0.046)	2293 (0.034)	#SAKNAS!
osteoperio	4	2153 (0.046)	2293 (0.034)	2492 (0.033)
osteoporotic	4	2153 (0.046)	1457 (0.069)	1612 (0.066)
oviposition	4	2153 (0.046)	1067 (0.1)	2492 (0.033)
oxytocin	4	2153 (0.046)	1457 (0.069)	2492 (0.033)
palatable	4	2153 (0.046)	2293 (0.034)	1612 (0.066)
pancreatic	4	2153 (0.046)	1067 (0.1)	2492 (0.033)
panulirus	4	2153 (0.046)	#SAKNAS!	1612 (0.066)
papillomavirus	4	2153 (0.046)	#SAKNAS!	947 (0.13)
parasitic	4	2153 (0.046)	2293 (0.034)	1180 (0.1)
parasitism	4	2153 (0.046)	1067 (0.1)	#SAKNAS!
parity	4	2153 (0.046)	1457 (0.069)	2492 (0.033)
partially	4	2153 (0.046)	#SAKNAS!	1180 (0.1)
partial-mouth	4	2153 (0.046)	#SAKNAS!	1180 (0.1)
participatory	4	2153 (0.046)	#SAKNAS!	1612 (0.066)
particular	4	2153 (0.046)	1457 (0.069)	2492 (0.033)
pastures	4	2153 (0.046)	#SAKNAS!	1612 (0.066)
pathologies	4	2153 (0.046)	#SAKNAS!	1612 (0.066)
pathophysiology	4	2153 (0.046)	#SAKNAS!	1180 (0.1)
pediatrics	4	2153 (0.046)	2293 (0.034)	1612 (0.066)
pelleted	4	2153 (0.046)	1067 (0.1)	2492 (0.033)
penaeus	4	2153 (0.046)	1067 (0.1)	#SAKNAS!
penetration	4	2153 (0.046)	#SAKNAS!	1180 (0.1)
pepper	4	2153 (0.046)	1457 (0.069)	1612 (0.066)
periodontitis-a	4	2153 (0.046)	#SAKNAS!	2492 (0.033)
periodontitis-associated	4	2153 (0.046)	2293 (0.034)	1612 (0.066)
per-oral	4	2153 (0.046)	#SAKNAS!	947 (0.13)
pest	4	2153 (0.046)	2293 (0.034)	1612 (0.066)
phases	4	2153 (0.046)	2293 (0.034)	#SAKNAS!
phenomenon	4	2153 (0.046)	2293 (0.034)	2492 (0.033)
phenotypic	4	2153 (0.046)	2293 (0.034)	1612 (0.066)

phloem	4	2153 (0.046)	1457 (0.069)	1612 (0.066)
physician	4	2153 (0.046)	2293 (0.034)	1180 (0.1)
physicians	4	2153 (0.046)	1457 (0.069)	1612 (0.066)
physiologic	4	2153 (0.046)	2293 (0.034)	2492 (0.033)
piglets	4	2153 (0.046)	#SAKNAS!	2492 (0.033)
pisum	4	2153 (0.046)	#SAKNAS!	#SAKNAS!
plain	4	2153 (0.046)	2293 (0.034)	1612 (0.066)
poisoning	4	2153 (0.046)	#SAKNAS!	1612 (0.066)
polysaccharide	4	2153 (0.046)	#SAKNAS!	#SAKNAS!
pompe	4	2153 (0.046)	2293 (0.034)	2492 (0.033)
positively	4	2153 (0.046)	#SAKNAS!	1612 (0.066)
post-acute	4	2153 (0.046)	#SAKNAS!	1180 (0.1)
post-operative	4	2153 (0.046)	#SAKNAS!	947 (0.13)
precision	4	2153 (0.046)	2293 (0.034)	2492 (0.033)
predators	4	2153 (0.046)	2293 (0.034)	1612 (0.066)
pre-eclampsia	4	2153 (0.046)	1067 (0.1)	2492 (0.033)
presbyphagia	4	2153 (0.046)	#SAKNAS!	1612 (0.066)
prevertebral	4	2153 (0.046)	2293 (0.034)	2492 (0.033)
previous	4	2153 (0.046)	2293 (0.034)	2492 (0.033)
previously	4	2153 (0.046)	1457 (0.069)	2492 (0.033)
primate	4	2153 (0.046)	#SAKNAS!	947 (0.13)
proactive	4	2153 (0.046)	2293 (0.034)	2492 (0.033)
problem	4	2153 (0.046)	2293 (0.034)	2492 (0.033)
programmes	4	2153 (0.046)	844 (0.14)	#SAKNAS!
promoter	4	2153 (0.046)	1067 (0.1)	#SAKNAS!
proposal	4	2153 (0.046)	2293 (0.034)	2492 (0.033)
province	4	2153 (0.046)	#SAKNAS!	1180 (0.1)
psoriasis	4	2153 (0.046)	2293 (0.034)	1612 (0.066)
pulse	4	2153 (0.046)	2293 (0.034)	2492 (0.033)
pupils	4	2153 (0.046)	1457 (0.069)	2492 (0.033)
race	4	2153 (0.046)	2293 (0.034)	1612 (0.066)
ratios	4	2153 (0.046)	2293 (0.034)	2492 (0.033)
real-time	4	2153 (0.046)	#SAKNAS!	1612 (0.066)
rearing	4	2153 (0.046)	2293 (0.034)	1612 (0.066)
receipt	4	2153 (0.046)	1457 (0.069)	1612 (0.066)
recession	4	2153 (0.046)	1457 (0.069)	2492 (0.033)
recording	4	2153 (0.046)	1457 (0.069)	2492 (0.033)
reflex	4	2153 (0.046)	#SAKNAS!	1180 (0.1)
refugees	4	2153 (0.046)	#SAKNAS!	1612 (0.066)
regular	4	2153 (0.046)	2293 (0.034)	1612 (0.066)
reinforcement	4	2153 (0.046)	1457 (0.069)	1612 (0.066)
relate	4	2153 (0.046)	1067 (0.1)	#SAKNAS!
relatives	4	2153 (0.046)	#SAKNAS!	1612 (0.066)

release	4	2153 (0.046)	1457 (0.069)	2492 (0.033)
replacing	4	2153 (0.046)	2293 (0.034)	2492 (0.033)
reporting	4	2153 (0.046)	#SAKNAS!	#SAKNAS!
resident	4	2153 (0.046)	2293 (0.034)	1612 (0.066)
residing	4	2153 (0.046)	1457 (0.069)	#SAKNAS!
residue	4	2153 (0.046)	#SAKNAS!	947 (0.13)
resistin	4	2153 (0.046)	#SAKNAS!	947 (0.13)
resonance	4	2153 (0.046)	2293 (0.034)	2492 (0.033)
restorations	4	2153 (0.046)	1457 (0.069)	1612 (0.066)
rethinking	4	2153 (0.046)	1067 (0.1)	#SAKNAS!
retinopathy	4	2153 (0.046)	#SAKNAS!	1180 (0.1)
reuteri	4	2153 (0.046)	#SAKNAS!	1180 (0.1)
rheology	4	2153 (0.046)	1457 (0.069)	#SAKNAS!
rhythm	4	2153 (0.046)	1457 (0.069)	2492 (0.033)
rhythms	4	2153 (0.046)	2293 (0.034)	1612 (0.066)
rico	4	2153 (0.046)	1457 (0.069)	1612 (0.066)
riyadh	4	2153 (0.046)	2293 (0.034)	2492 (0.033)
rodent	4	2153 (0.046)	2293 (0.034)	1180 (0.1)
route	4	2153 (0.046)	1067 (0.1)	2492 (0.033)
salvador	4	2153 (0.046)	#SAKNAS!	1180 (0.1)
scales	4	2153 (0.046)	2293 (0.034)	1180 (0.1)
schistocerca	4	2153 (0.046)	1067 (0.1)	2492 (0.033)
schizophrenia	4	2153 (0.046)	2293 (0.034)	2492 (0.033)
scoping	4	2153 (0.046)	#SAKNAS!	2492 (0.033)
sealants	4	2153 (0.046)	#SAKNAS!	1612 (0.066)
secondhand	4	2153 (0.046)	2293 (0.034)	2492 (0.033)
self-care	4	2153 (0.046)	#SAKNAS!	1612 (0.066)
seston	4	2153 (0.046)	844 (0.14)	#SAKNAS!
severely	4	2153 (0.046)	1457 (0.069)	#SAKNAS!
shifts	4	2153 (0.046)	2293 (0.034)	2492 (0.033)
simplex	4	2153 (0.046)	1457 (0.069)	2492 (0.033)
simulation	4	2153 (0.046)	#SAKNAS!	1612 (0.066)
since	4	2153 (0.046)	2293 (0.034)	1180 (0.1)
single-level	4	2153 (0.046)	#SAKNAS!	1612 (0.066)
situation	4	2153 (0.046)	2293 (0.034)	1612 (0.066)
sjogren	4	2153 (0.046)	2293 (0.034)	1612 (0.066)
skills	4	2153 (0.046)	2293 (0.034)	1180 (0.1)
skipping	4	2153 (0.046)	#SAKNAS!	2492 (0.033)
skull	4	2153 (0.046)	1457 (0.069)	#SAKNAS!
snail	4	2153 (0.046)	#SAKNAS!	1612 (0.066)
solitary	4	2153 (0.046)	1457 (0.069)	1612 (0.066)
sonic	4	2153 (0.046)	#SAKNAS!	1180 (0.1)
southeast	4	2153 (0.046)	2293 (0.034)	#SAKNAS!

specificity	4	2153 (0.046)	2293 (0.034)	2492 (0.033)
spider	4	2153 (0.046)	1457 (0.069)	1612 (0.066)
sport	4	2153 (0.046)	2293 (0.034)	1180 (0.1)
spp	4	2153 (0.046)	1457 (0.069)	2492 (0.033)
spruce	4	2153 (0.046)	844 (0.14)	#SAKNAS!
sri	4	2153 (0.046)	1457 (0.069)	1612 (0.066)
standardization	4	2153 (0.046)	#SAKNAS!	2492 (0.033)
statement	4	2153 (0.046)	2293 (0.034)	1180 (0.1)
storage	4	2153 (0.046)	2293 (0.034)	2492 (0.033)
straw	4	2153 (0.046)	1457 (0.069)	2492 (0.033)
strongly	4	2153 (0.046)	#SAKNAS!	1180 (0.1)
subclinical	4	2153 (0.046)	2293 (0.034)	1612 (0.066)
submandibular	4	2153 (0.046)	1067 (0.1)	#SAKNAS!
suboptimal	4	2153 (0.046)	#SAKNAS!	1612 (0.066)
substances	4	2153 (0.046)	2293 (0.034)	2492 (0.033)
subtypes	4	2153 (0.046)	2293 (0.034)	#SAKNAS!
suitability	4	2153 (0.046)	1457 (0.069)	1612 (0.066)
summer	4	2153 (0.046)	2293 (0.034)	#SAKNAS!
suppression	4	2153 (0.046)	844 (0.14)	#SAKNAS!
sweetened	4	2153 (0.046)	#SAKNAS!	947 (0.13)
swiss	4	2153 (0.046)	2293 (0.034)	2492 (0.033)
switch	4	2153 (0.046)	1457 (0.069)	2492 (0.033)
symptomatic	4	2153 (0.046)	2293 (0.034)	1612 (0.066)
target	4	2153 (0.046)	#SAKNAS!	1612 (0.066)
tastes	4	2153 (0.046)	2293 (0.034)	1612 (0.066)
technical	4	2153 (0.046)	1067 (0.1)	2492 (0.033)
temperate	4	2153 (0.046)	1067 (0.1)	2492 (0.033)
temporary	4	2153 (0.046)	2293 (0.034)	1612 (0.066)
terms	4	2153 (0.046)	2293 (0.034)	2492 (0.033)
terrestris	4	2153 (0.046)	2293 (0.034)	#SAKNAS!
theoretical	4	2153 (0.046)	1067 (0.1)	#SAKNAS!
three-dimensional	4	2153 (0.046)	#SAKNAS!	2492 (0.033)
tracheoesophageal	4	2153 (0.046)	2293 (0.034)	1612 (0.066)
transcript	4	2153 (0.046)	1457 (0.069)	2492 (0.033)
transcutaneous	4	2153 (0.046)	2293 (0.034)	1612 (0.066)
transnasal	4	2153 (0.046)	2293 (0.034)	1180 (0.1)
transplant	4	2153 (0.046)	1457 (0.069)	#SAKNAS!
trend	4	2153 (0.046)	#SAKNAS!	1612 (0.066)
trismus	4	2153 (0.046)	2293 (0.034)	1612 (0.066)
tube-fed	4	2153 (0.046)	1457 (0.069)	2492 (0.033)
tumour	4	2153 (0.046)	844 (0.14)	#SAKNAS!
ultrasonic	4	2153 (0.046)	2293 (0.034)	2492 (0.033)
undergraduate	4	2153 (0.046)	2293 (0.034)	2492 (0.033)

understand		4	2153 (0.046)	1457 (0.069)	1612 (0.066)
uric		4	2153 (0.046)	#SAKNAS!	#SAKNAS!
v		4	2153 (0.046)	#SAKNAS!	947 (0.13)
variable		4	2153 (0.046)	1457 (0.069)	1612 (0.066)
variants		4	2153 (0.046)	2293 (0.034)	1612 (0.066)
varies		4	2153 (0.046)	2293 (0.034)	#SAKNAS!
vitamins		4	2153 (0.046)	#SAKNAS!	2492 (0.033)
wall		4	2153 (0.046)	2293 (0.034)	2492 (0.033)
wasting		4	2153 (0.046)	2293 (0.034)	2492 (0.033)
waters		4	2153 (0.046)	1457 (0.069)	2492 (0.033)
wave		4	2153 (0.046)	2293 (0.034)	1612 (0.066)
wintering		4	2153 (0.046)	1457 (0.069)	1612 (0.066)
woman		4	2153 (0.046)	#SAKNAS!	#SAKNAS!
yield		4	2153 (0.046)	#SAKNAS!	#SAKNAS!
	1758	3	2632 (0.034)	#SAKNAS!	#SAKNAS!
	1966	3	2632 (0.034)	#SAKNAS!	2492 (0.033)
	2021	3	2632 (0.034)	#SAKNAS!	#SAKNAS!
10-year-old		3	2632 (0.034)	2293 (0.034)	2492 (0.033)
12-month		3	2632 (0.034)	#SAKNAS!	2492 (0.033)
12-to		3	2632 (0.034)	#SAKNAS!	1612 (0.066)
12-year-olds		3	2632 (0.034)	#SAKNAS!	1612 (0.066)
18-year-old		3	2632 (0.034)	2293 (0.034)	1612 (0.066)
1999-2002		3	2632 (0.034)	1067 (0.1)	#SAKNAS!
2008-2009		3	2632 (0.034)	2293 (0.034)	2492 (0.033)
2011-2018		3	2632 (0.034)	#SAKNAS!	#SAKNAS!
2012-2013		3	2632 (0.034)	#SAKNAS!	1180 (0.1)
2012-2014		3	2632 (0.034)	#SAKNAS!	1180 (0.1)
2015-2016		3	2632 (0.034)	#SAKNAS!	2492 (0.033)
2017-2018		3	2632 (0.034)	#SAKNAS!	#SAKNAS!
21st		3	2632 (0.034)	1457 (0.069)	2492 (0.033)
2-to		3	2632 (0.034)	2293 (0.034)	2492 (0.033)
30-day		3	2632 (0.034)	#SAKNAS!	2492 (0.033)
3d-printed		3	2632 (0.034)	#SAKNAS!	#SAKNAS!
5-to		3	2632 (0.034)	2293 (0.034)	2492 (0.033)
6-to		3	2632 (0.034)	1457 (0.069)	2492 (0.033)
6-year-old		3	2632 (0.034)	2293 (0.034)	#SAKNAS!
7-year-old		3	2632 (0.034)	1457 (0.069)	2492 (0.033)
80-year-old		3	2632 (0.034)	1457 (0.069)	2492 (0.033)
absence		3	2632 (0.034)	2293 (0.034)	2492 (0.033)
abundant		3	2632 (0.034)	1457 (0.069)	2492 (0.033)
acellular		3	2632 (0.034)	1457 (0.069)	2492 (0.033)
acetate		3	2632 (0.034)	2293 (0.034)	2492 (0.033)
achieving		3	2632 (0.034)	#SAKNAS!	1612 (0.066)

acidic	3	2632 (0.034)	#SAKNAS!	1612 (0.066)
acquisition	3	2632 (0.034)	#SAKNAS!	1612 (0.066)
activates	3	2632 (0.034)	2293 (0.034)	2492 (0.033)
acupuncture	3	2632 (0.034)	#SAKNAS!	2492 (0.033)
additive	3	2632 (0.034)	2293 (0.034)	2492 (0.033)
addressing	3	2632 (0.034)	2293 (0.034)	2492 (0.033)
adipokine	3	2632 (0.034)	#SAKNAS!	1612 (0.066)
adjunct	3	2632 (0.034)	#SAKNAS!	2492 (0.033)
adjunctive	3	2632 (0.034)	#SAKNAS!	2492 (0.033)
adjuvant	3	2632 (0.034)	#SAKNAS!	2492 (0.033)
advanced-stage	3	2632 (0.034)	1457 (0.069)	#SAKNAS!
advancement	3	2632 (0.034)	1457 (0.069)	2492 (0.033)
advancing	3	2632 (0.034)	#SAKNAS!	#SAKNAS!
aedes	3	2632 (0.034)	2293 (0.034)	2492 (0.033)
aerodigestive	3	2632 (0.034)	#SAKNAS!	2492 (0.033)
agriculture	3	2632 (0.034)	#SAKNAS!	2492 (0.033)
alaska	3	2632 (0.034)	1067 (0.1)	#SAKNAS!
alaskan	3	2632 (0.034)	#SAKNAS!	1180 (0.1)
albicans	3	2632 (0.034)	2293 (0.034)	#SAKNAS!
aleyrodidae	3	2632 (0.034)	1457 (0.069)	2492 (0.033)
alternate	3	2632 (0.034)	1457 (0.069)	#SAKNAS!
amazon	3	2632 (0.034)	2293 (0.034)	1612 (0.066)
amphetamine-regulated	3	2632 (0.034)	1457 (0.069)	#SAKNAS!
analytical	3	2632 (0.034)	2293 (0.034)	#SAKNAS!
anatomical	3	2632 (0.034)	2293 (0.034)	#SAKNAS!
antagonistic	3	2632 (0.034)	2293 (0.034)	#SAKNAS!
anthropogenic	3	2632 (0.034)	#SAKNAS!	1612 (0.066)
antibiotics	3	2632 (0.034)	1457 (0.069)	#SAKNAS!
anticholinergic	3	2632 (0.034)	2293 (0.034)	#SAKNAS!
anticipatory	3	2632 (0.034)	2293 (0.034)	2492 (0.033)
antigen	3	2632 (0.034)	2293 (0.034)	2492 (0.033)
anti-inflammatory	3	2632 (0.034)	#SAKNAS!	2492 (0.033)
ants	3	2632 (0.034)	2293 (0.034)	#SAKNAS!
anura	3	2632 (0.034)	2293 (0.034)	2492 (0.033)
aphis	3	2632 (0.034)	#SAKNAS!	1612 (0.066)
apnea-hypopnea	3	2632 (0.034)	#SAKNAS!	1612 (0.066)
apple	3	2632 (0.034)	#SAKNAS!	1612 (0.066)
aquatic	3	2632 (0.034)	#SAKNAS!	1612 (0.066)
architecture	3	2632 (0.034)	2293 (0.034)	#SAKNAS!
aric	3	2632 (0.034)	2293 (0.034)	2492 (0.033)
armigera	3	2632 (0.034)	#SAKNAS!	1612 (0.066)
ascia	3	2632 (0.034)	1067 (0.1)	#SAKNAS!
assistance	3	2632 (0.034)	2293 (0.034)	#SAKNAS!

asymmetric	3	2632 (0.034)	2293 (0.034)	1612 (0.066)
atherosclerotic	3	2632 (0.034)	2293 (0.034)	#SAKNAS!
attenuates	3	2632 (0.034)	#SAKNAS!	2492 (0.033)
aurata	3	2632 (0.034)	1067 (0.1)	#SAKNAS!
avoidant	3	2632 (0.034)	#SAKNAS!	#SAKNAS!
away-from-home	3	2632 (0.034)	2293 (0.034)	1612 (0.066)
bacillus	3	2632 (0.034)	1457 (0.069)	2492 (0.033)
bad	3	2632 (0.034)	1067 (0.1)	#SAKNAS!
banana	3	2632 (0.034)	#SAKNAS!	1180 (0.1)
bangladeshi	3	2632 (0.034)	1457 (0.069)	2492 (0.033)
barcelona	3	2632 (0.034)	2293 (0.034)	2492 (0.033)
bark	3	2632 (0.034)	1067 (0.1)	#SAKNAS!
basal	3	2632 (0.034)	#SAKNAS!	1612 (0.066)
bases	3	2632 (0.034)	2293 (0.034)	2492 (0.033)
bay	3	2632 (0.034)	1457 (0.069)	2492 (0.033)
bdnf	3	2632 (0.034)	1457 (0.069)	#SAKNAS!
beetles	3	2632 (0.034)	2293 (0.034)	#SAKNAS!
bemisia	3	2632 (0.034)	2293 (0.034)	1612 (0.066)
big	3	2632 (0.034)	#SAKNAS!	2492 (0.033)
biliopancreatic	3	2632 (0.034)	2293 (0.034)	#SAKNAS!
bioavailability	3	2632 (0.034)	#SAKNAS!	1612 (0.066)
biofeedback	3	2632 (0.034)	#SAKNAS!	1612 (0.066)
biomechanics	3	2632 (0.034)	2293 (0.034)	1612 (0.066)
bites	3	2632 (0.034)	#SAKNAS!	1612 (0.066)
bivalve	3	2632 (0.034)	2293 (0.034)	2492 (0.033)
bivalvia	3	2632 (0.034)	1067 (0.1)	#SAKNAS!
bladder	3	2632 (0.034)	#SAKNAS!	1180 (0.1)
blends	3	2632 (0.034)	#SAKNAS!	2492 (0.033)
blind	3	2632 (0.034)	2293 (0.034)	2492 (0.033)
blue	3	2632 (0.034)	1457 (0.069)	#SAKNAS!
bombyx	3	2632 (0.034)	2293 (0.034)	2492 (0.033)
box	3	2632 (0.034)	2293 (0.034)	2492 (0.033)
brazilians	3	2632 (0.034)	1457 (0.069)	#SAKNAS!
breeding	3	2632 (0.034)	1457 (0.069)	2492 (0.033)
bridge	3	2632 (0.034)	#SAKNAS!	1612 (0.066)
buccal	3	2632 (0.034)	2293 (0.034)	1612 (0.066)
budworm	3	2632 (0.034)	1067 (0.1)	#SAKNAS!
c1-2	3	2632 (0.034)	#SAKNAS!	2492 (0.033)
c57bl	3	2632 (0.034)	2293 (0.034)	2492 (0.033)
cadmium	3	2632 (0.034)	1067 (0.1)	#SAKNAS!
cafeteria	3	2632 (0.034)	2293 (0.034)	2492 (0.033)
calcification	3	2632 (0.034)	#SAKNAS!	#SAKNAS!
calorie	3	2632 (0.034)	2293 (0.034)	1612 (0.066)

cambodian	3	2632 (0.034)	#SAKNAS!	#SAKNAS!
candy	3	2632 (0.034)	#SAKNAS!	1612 (0.066)
cane	3	2632 (0.034)	#SAKNAS!	1612 (0.066)
capability	3	2632 (0.034)	#SAKNAS!	#SAKNAS!
cape	3	2632 (0.034)	2293 (0.034)	1612 (0.066)
cardiorespiratory	3	2632 (0.034)	#SAKNAS!	1612 (0.066)
carers	3	2632 (0.034)	#SAKNAS!	1180 (0.1)
caries-related	3	2632 (0.034)	2293 (0.034)	2492 (0.033)
carolina	3	2632 (0.034)	1457 (0.069)	#SAKNAS!
carp	3	2632 (0.034)	#SAKNAS!	1612 (0.066)
cart	3	2632 (0.034)	1457 (0.069)	#SAKNAS!
casein	3	2632 (0.034)	#SAKNAS!	2492 (0.033)
category	3	2632 (0.034)	#SAKNAS!	2492 (0.033)
cd	3	2632 (0.034)	1457 (0.069)	2492 (0.033)
ceratitis-capitata	3	2632 (0.034)	1067 (0.1)	#SAKNAS!
cerebrospinal	3	2632 (0.034)	2293 (0.034)	2492 (0.033)
chain	3	2632 (0.034)	#SAKNAS!	#SAKNAS!
channel	3	2632 (0.034)	2293 (0.034)	2492 (0.033)
checkups	3	2632 (0.034)	#SAKNAS!	#SAKNAS!
chew	3	2632 (0.034)	2293 (0.034)	#SAKNAS!
cholecystokinin	3	2632 (0.034)	1067 (0.1)	#SAKNAS!
choose	3	2632 (0.034)	1067 (0.1)	#SAKNAS!
chronotype	3	2632 (0.034)	#SAKNAS!	1612 (0.066)
circuits	3	2632 (0.034)	1457 (0.069)	2492 (0.033)
citrus	3	2632 (0.034)	#SAKNAS!	2492 (0.033)
clinicopathological	3	2632 (0.034)	#SAKNAS!	2492 (0.033)
cod	3	2632 (0.034)	2293 (0.034)	2492 (0.033)
collaboration	3	2632 (0.034)	2293 (0.034)	2492 (0.033)
collaborative	3	2632 (0.034)	#SAKNAS!	#SAKNAS!
colonic	3	2632 (0.034)	#SAKNAS!	1180 (0.1)
commensal	3	2632 (0.034)	#SAKNAS!	1612 (0.066)
community-living	3	2632 (0.034)	#SAKNAS!	2492 (0.033)
comorbid	3	2632 (0.034)	2293 (0.034)	#SAKNAS!
complicating	3	2632 (0.034)	1457 (0.069)	#SAKNAS!
compression	3	2632 (0.034)	#SAKNAS!	1612 (0.066)
concerning	3	2632 (0.034)	#SAKNAS!	2492 (0.033)
congo	3	2632 (0.034)	1457 (0.069)	#SAKNAS!
considering	3	2632 (0.034)	2293 (0.034)	2492 (0.033)
contemporary	3	2632 (0.034)	2293 (0.034)	1612 (0.066)
contributing	3	2632 (0.034)	2293 (0.034)	2492 (0.033)
contributions	3	2632 (0.034)	#SAKNAS!	2492 (0.033)
controlling	3	2632 (0.034)	2293 (0.034)	1612 (0.066)
cope	3	2632 (0.034)	2293 (0.034)	2492 (0.033)

corals	3	2632 (0.034)	1457 (0.069)	2492 (0.033)
corticosterone	3	2632 (0.034)	1067 (0.1)	#SAKNAS!
croatian	3	2632 (0.034)	#SAKNAS!	1612 (0.066)
crude	3	2632 (0.034)	1457 (0.069)	2492 (0.033)
cruzi	3	2632 (0.034)	#SAKNAS!	1612 (0.066)
cu	3	2632 (0.034)	2293 (0.034)	2492 (0.033)
cucumber	3	2632 (0.034)	2293 (0.034)	1612 (0.066)
curriculum	3	2632 (0.034)	2293 (0.034)	#SAKNAS!
cycles	3	2632 (0.034)	2293 (0.034)	1612 (0.066)
d2	3	2632 (0.034)	2293 (0.034)	2492 (0.033)
d3	3	2632 (0.034)	1457 (0.069)	#SAKNAS!
decades	3	2632 (0.034)	#SAKNAS!	1612 (0.066)
decannulation	3	2632 (0.034)	#SAKNAS!	1612 (0.066)
decapoda	3	2632 (0.034)	1457 (0.069)	2492 (0.033)
defect	3	2632 (0.034)	#SAKNAS!	1180 (0.1)
defenses	3	2632 (0.034)	2293 (0.034)	2492 (0.033)
definition	3	2632 (0.034)	#SAKNAS!	1612 (0.066)
definitions	3	2632 (0.034)	1457 (0.069)	#SAKNAS!
deformity	3	2632 (0.034)	#SAKNAS!	1612 (0.066)
dehydration	3	2632 (0.034)	2293 (0.034)	#SAKNAS!
delirium	3	2632 (0.034)	#SAKNAS!	2492 (0.033)
delivering	3	2632 (0.034)	#SAKNAS!	#SAKNAS!
dentin	3	2632 (0.034)	#SAKNAS!	2492 (0.033)
dependency	3	2632 (0.034)	2293 (0.034)	2492 (0.033)
depletion	3	2632 (0.034)	1457 (0.069)	#SAKNAS!
depth	3	2632 (0.034)	#SAKNAS!	1612 (0.066)
designing	3	2632 (0.034)	#SAKNAS!	#SAKNAS!
destructive	3	2632 (0.034)	1457 (0.069)	#SAKNAS!
detergent	3	2632 (0.034)	#SAKNAS!	2492 (0.033)
deterioration	3	2632 (0.034)	2293 (0.034)	2492 (0.033)
develop	3	2632 (0.034)	1457 (0.069)	#SAKNAS!
diabetics	3	2632 (0.034)	2293 (0.034)	2492 (0.033)
diagnoses	3	2632 (0.034)	1457 (0.069)	2492 (0.033)
diarrhea	3	2632 (0.034)	2293 (0.034)	2492 (0.033)
diastolic	3	2632 (0.034)	2293 (0.034)	1612 (0.066)
dietitian	3	2632 (0.034)	#SAKNAS!	2492 (0.033)
differ	3	2632 (0.034)	2293 (0.034)	2492 (0.033)
difficult	3	2632 (0.034)	2293 (0.034)	2492 (0.033)
digital	3	2632 (0.034)	#SAKNAS!	2492 (0.033)
dimensions	3	2632 (0.034)	#SAKNAS!	2492 (0.033)
dimorphic	3	2632 (0.034)	2293 (0.034)	1612 (0.066)
disc	3	2632 (0.034)	#SAKNAS!	2492 (0.033)
display	3	2632 (0.034)	2293 (0.034)	2492 (0.033)

disruption	3	2632 (0.034)	2293 (0.034)	#SAKNAS!
dissatisfaction	3	2632 (0.034)	2293 (0.034)	1612 (0.066)
distress	3	2632 (0.034)	#SAKNAS!	2492 (0.033)
diversion	3	2632 (0.034)	2293 (0.034)	1612 (0.066)
dog	3	2632 (0.034)	#SAKNAS!	1612 (0.066)
dopaminergic	3	2632 (0.034)	1067 (0.1)	#SAKNAS!
dosimetric	3	2632 (0.034)	2293 (0.034)	1612 (0.066)
drive	3	2632 (0.034)	2293 (0.034)	2492 (0.033)
drives	3	2632 (0.034)	#SAKNAS!	2492 (0.033)
drugs	3	2632 (0.034)	#SAKNAS!	#SAKNAS!
dsm	3	2632 (0.034)	#SAKNAS!	1612 (0.066)
dying	3	2632 (0.034)	2293 (0.034)	2492 (0.033)
dysmotility	3	2632 (0.034)	2293 (0.034)	2492 (0.033)
dyspepsia	3	2632 (0.034)	#SAKNAS!	1180 (0.1)
early-stage	3	2632 (0.034)	#SAKNAS!	2492 (0.033)
earthworm	3	2632 (0.034)	#SAKNAS!	1180 (0.1)
eaten	3	2632 (0.034)	1457 (0.069)	2492 (0.033)
edentulousness	3	2632 (0.034)	1457 (0.069)	2492 (0.033)
ehlers-danlos	3	2632 (0.034)	#SAKNAS!	1612 (0.066)
elements	3	2632 (0.034)	2293 (0.034)	1612 (0.066)
employees	3	2632 (0.034)	#SAKNAS!	1612 (0.066)
employment	3	2632 (0.034)	#SAKNAS!	1180 (0.1)
emulsion	3	2632 (0.034)	#SAKNAS!	2492 (0.033)
encephalopathy	3	2632 (0.034)	1457 (0.069)	#SAKNAS!
endothelial	3	2632 (0.034)	#SAKNAS!	2492 (0.033)
endotracheal	3	2632 (0.034)	2293 (0.034)	#SAKNAS!
engagement	3	2632 (0.034)	#SAKNAS!	#SAKNAS!
enjoyment	3	2632 (0.034)	#SAKNAS!	1612 (0.066)
entry	3	2632 (0.034)	#SAKNAS!	1612 (0.066)
erica	3	2632 (0.034)	#SAKNAS!	1612 (0.066)
ethical	3	2632 (0.034)	1457 (0.069)	2492 (0.033)
ethics	3	2632 (0.034)	2293 (0.034)	2492 (0.033)
ethnicity	3	2632 (0.034)	2293 (0.034)	#SAKNAS!
ethylene	3	2632 (0.034)	#SAKNAS!	#SAKNAS!
eulemur	3	2632 (0.034)	1067 (0.1)	#SAKNAS!
example	3	2632 (0.034)	1067 (0.1)	#SAKNAS!
experiment	3	2632 (0.034)	2293 (0.034)	2492 (0.033)
facilitates	3	2632 (0.034)	1457 (0.069)	2492 (0.033)
faecal	3	2632 (0.034)	1457 (0.069)	2492 (0.033)
farm	3	2632 (0.034)	1457 (0.069)	#SAKNAS!
farming	3	2632 (0.034)	2293 (0.034)	2492 (0.033)
fascicularis	3	2632 (0.034)	#SAKNAS!	2492 (0.033)
fatal	3	2632 (0.034)	1067 (0.1)	#SAKNAS!

feasible	3	2632 (0.034)	2293 (0.034)	1612 (0.066)
feeder	3	2632 (0.034)	2293 (0.034)	2492 (0.033)
feeders	3	2632 (0.034)	1457 (0.069)	2492 (0.033)
fibrinogen	3	2632 (0.034)	1457 (0.069)	2492 (0.033)
filipino	3	2632 (0.034)	1067 (0.1)	#SAKNAS!
fire	3	2632 (0.034)	1067 (0.1)	#SAKNAS!
five-year	3	2632 (0.034)	2293 (0.034)	#SAKNAS!
flaps	3	2632 (0.034)	1457 (0.069)	2492 (0.033)
flavonoids	3	2632 (0.034)	2293 (0.034)	#SAKNAS!
flexibility	3	2632 (0.034)	1457 (0.069)	2492 (0.033)
flower	3	2632 (0.034)	2293 (0.034)	2492 (0.033)
foliage	3	2632 (0.034)	1457 (0.069)	#SAKNAS!
folic	3	2632 (0.034)	2293 (0.034)	#SAKNAS!
foragers	3	2632 (0.034)	2293 (0.034)	1612 (0.066)
forces	3	2632 (0.034)	2293 (0.034)	#SAKNAS!
forebrain	3	2632 (0.034)	#SAKNAS!	1612 (0.066)
forests	3	2632 (0.034)	2293 (0.034)	2492 (0.033)
format	3	2632 (0.034)	#SAKNAS!	1612 (0.066)
formulations	3	2632 (0.034)	#SAKNAS!	2492 (0.033)
forsythia	3	2632 (0.034)	1457 (0.069)	#SAKNAS!
fragments	3	2632 (0.034)	#SAKNAS!	1180 (0.1)
free-choice	3	2632 (0.034)	2293 (0.034)	2492 (0.033)
frequent	3	2632 (0.034)	2293 (0.034)	2492 (0.033)
frugivorous	3	2632 (0.034)	2293 (0.034)	2492 (0.033)
fungi	3	2632 (0.034)	2293 (0.034)	2492 (0.033)
further	3	2632 (0.034)	2293 (0.034)	2492 (0.033)
fuscata	3	2632 (0.034)	1457 (0.069)	2492 (0.033)
fusobacterium	3	2632 (0.034)	1457 (0.069)	2492 (0.033)
gastrojejunal	3	2632 (0.034)	2293 (0.034)	2492 (0.033)
gelatin	3	2632 (0.034)	2293 (0.034)	#SAKNAS!
gelling	3	2632 (0.034)	#SAKNAS!	#SAKNAS!
gender-specific	3	2632 (0.034)	2293 (0.034)	#SAKNAS!
genotypes	3	2632 (0.034)	#SAKNAS!	1612 (0.066)
genus	3	2632 (0.034)	1457 (0.069)	2492 (0.033)
geographic	3	2632 (0.034)	2293 (0.034)	1612 (0.066)
gigas	3	2632 (0.034)	1457 (0.069)	2492 (0.033)
gilthead	3	2632 (0.034)	1067 (0.1)	#SAKNAS!
glim	3	2632 (0.034)	#SAKNAS!	2492 (0.033)
glucocorticoid	3	2632 (0.034)	2293 (0.034)	1612 (0.066)
goals	3	2632 (0.034)	#SAKNAS!	1612 (0.066)
goldfish	3	2632 (0.034)	1457 (0.069)	2492 (0.033)
gorilla	3	2632 (0.034)	1457 (0.069)	2492 (0.033)
gossypii	3	2632 (0.034)	#SAKNAS!	1612 (0.066)

grains	3	2632 (0.034)	1457 (0.069)	#SAKNAS!
gray	3	2632 (0.034)	2293 (0.034)	2492 (0.033)
ground	3	2632 (0.034)	1457 (0.069)	#SAKNAS!
group-housed	3	2632 (0.034)	1067 (0.1)	#SAKNAS!
guidance	3	2632 (0.034)	2293 (0.034)	2492 (0.033)
guinea	3	2632 (0.034)	2293 (0.034)	2492 (0.033)
gulf	3	2632 (0.034)	2293 (0.034)	2492 (0.033)
habitats	3	2632 (0.034)	2293 (0.034)	#SAKNAS!
hand	3	2632 (0.034)	#SAKNAS!	2492 (0.033)
health-care	3	2632 (0.034)	1067 (0.1)	#SAKNAS!
healthful	3	2632 (0.034)	2293 (0.034)	1612 (0.066)
hearing	3	2632 (0.034)	#SAKNAS!	#SAKNAS!
hedonic	3	2632 (0.034)	2293 (0.034)	2492 (0.033)
helical	3	2632 (0.034)	#SAKNAS!	2492 (0.033)
helicoverpa	3	2632 (0.034)	#SAKNAS!	1612 (0.066)
hepatitis	3	2632 (0.034)	#SAKNAS!	2492 (0.033)
herbivores	3	2632 (0.034)	1457 (0.069)	#SAKNAS!
heterotrophic	3	2632 (0.034)	1457 (0.069)	2492 (0.033)
high-calorie	3	2632 (0.034)	1067 (0.1)	#SAKNAS!
high-fiber	3	2632 (0.034)	1457 (0.069)	2492 (0.033)
hill	3	2632 (0.034)	#SAKNAS!	1180 (0.1)
homeostatic	3	2632 (0.034)	2293 (0.034)	1612 (0.066)
homocysteine	3	2632 (0.034)	1457 (0.069)	#SAKNAS!
horse	3	2632 (0.034)	2293 (0.034)	2492 (0.033)
hospitalisation	3	2632 (0.034)	#SAKNAS!	1612 (0.066)
hummingbirds	3	2632 (0.034)	1067 (0.1)	#SAKNAS!
hungarian	3	2632 (0.034)	2293 (0.034)	#SAKNAS!
hyperphagia	3	2632 (0.034)	2293 (0.034)	2492 (0.033)
hypopharynx	3	2632 (0.034)	2293 (0.034)	2492 (0.033)
il-6	3	2632 (0.034)	2293 (0.034)	1612 (0.066)
images	3	2632 (0.034)	#SAKNAS!	1612 (0.066)
imbalance	3	2632 (0.034)	2293 (0.034)	2492 (0.033)
immature	3	2632 (0.034)	#SAKNAS!	1612 (0.066)
immediate	3	2632 (0.034)	2293 (0.034)	#SAKNAS!
immune-inflammation	3	2632 (0.034)	#SAKNAS!	#SAKNAS!
immunodeficiency	3	2632 (0.034)	#SAKNAS!	#SAKNAS!
implants	3	2632 (0.034)	#SAKNAS!	1612 (0.066)
implication	3	2632 (0.034)	2293 (0.034)	2492 (0.033)
improvements	3	2632 (0.034)	2293 (0.034)	#SAKNAS!
incisor	3	2632 (0.034)	2293 (0.034)	2492 (0.033)
incorporating	3	2632 (0.034)	#SAKNAS!	1612 (0.066)
increment	3	2632 (0.034)	2293 (0.034)	1612 (0.066)
indicate	3	2632 (0.034)	1457 (0.069)	#SAKNAS!

indirect	3	2632 (0.034)	#SAKNAS!	2492 (0.033)
infection-related	3	2632 (0.034)	#SAKNAS!	2492 (0.033)
inhabiting	3	2632 (0.034)	2293 (0.034)	1612 (0.066)
inhalation	3	2632 (0.034)	#SAKNAS!	2492 (0.033)
inhibiting	3	2632 (0.034)	#SAKNAS!	#SAKNAS!
inhibits	3	2632 (0.034)	2293 (0.034)	2492 (0.033)
inorganic	3	2632 (0.034)	2293 (0.034)	#SAKNAS!
instrumental	3	2632 (0.034)	#SAKNAS!	1612 (0.066)
instrumentation	3	2632 (0.034)	2293 (0.034)	2492 (0.033)
insufficiency	3	2632 (0.034)	2293 (0.034)	2492 (0.033)
intense	3	2632 (0.034)	1067 (0.1)	#SAKNAS!
interdialytic	3	2632 (0.034)	#SAKNAS!	2492 (0.033)
interleukin	3	2632 (0.034)	2293 (0.034)	1612 (0.066)
intertidal	3	2632 (0.034)	1457 (0.069)	#SAKNAS!
intervertebral	3	2632 (0.034)	#SAKNAS!	2492 (0.033)
intestine	3	2632 (0.034)	1067 (0.1)	#SAKNAS!
intramural	3	2632 (0.034)	1457 (0.069)	#SAKNAS!
intramuscular	3	2632 (0.034)	#SAKNAS!	2492 (0.033)
intuitive	3	2632 (0.034)	#SAKNAS!	1612 (0.066)
invest	3	2632 (0.034)	1457 (0.069)	2492 (0.033)
irradiation	3	2632 (0.034)	1457 (0.069)	2492 (0.033)
irregularity	3	2632 (0.034)	#SAKNAS!	1180 (0.1)
irritable	3	2632 (0.034)	#SAKNAS!	1612 (0.066)
isolate	3	2632 (0.034)	#SAKNAS!	2492 (0.033)
isotopic	3	2632 (0.034)	#SAKNAS!	1180 (0.1)
java	3	2632 (0.034)	2293 (0.034)	2492 (0.033)
jordan	3	2632 (0.034)	1067 (0.1)	#SAKNAS!
juan	3	2632 (0.034)	1457 (0.069)	2492 (0.033)
kids	3	2632 (0.034)	2293 (0.034)	#SAKNAS!
koges	3	2632 (0.034)	#SAKNAS!	1612 (0.066)
konjac	3	2632 (0.034)	#SAKNAS!	#SAKNAS!
krill	3	2632 (0.034)	2293 (0.034)	1612 (0.066)
kyoto-kameoka	3	2632 (0.034)	#SAKNAS!	1612 (0.066)
lactose	3	2632 (0.034)	#SAKNAS!	2492 (0.033)
lake	3	2632 (0.034)	1457 (0.069)	#SAKNAS!
lanka	3	2632 (0.034)	2293 (0.034)	1612 (0.066)
large-scale	3	2632 (0.034)	#SAKNAS!	1180 (0.1)
laryngotracheal	3	2632 (0.034)	1457 (0.069)	#SAKNAS!
latent	3	2632 (0.034)	#SAKNAS!	#SAKNAS!
late-onset	3	2632 (0.034)	#SAKNAS!	1612 (0.066)
lens	3	2632 (0.034)	2293 (0.034)	#SAKNAS!
leukocyte	3	2632 (0.034)	#SAKNAS!	1612 (0.066)
lifespan	3	2632 (0.034)	#SAKNAS!	1612 (0.066)

ligament	3	2632 (0.034)	2293 (0.034)	#SAKNAS!
like	3	2632 (0.034)	2293 (0.034)	1612 (0.066)
linear	3	2632 (0.034)	2293 (0.034)	1612 (0.066)
lingual	3	2632 (0.034)	1067 (0.1)	#SAKNAS!
linxian	3	2632 (0.034)	#SAKNAS!	1612 (0.066)
litopenaeus	3	2632 (0.034)	2293 (0.034)	1612 (0.066)
lizard	3	2632 (0.034)	1457 (0.069)	2492 (0.033)
locations	3	2632 (0.034)	2293 (0.034)	2492 (0.033)
low-level	3	2632 (0.034)	#SAKNAS!	1612 (0.066)
low-resource	3	2632 (0.034)	#SAKNAS!	1612 (0.066)
low-risk	3	2632 (0.034)	2293 (0.034)	1612 (0.066)
m3	3	2632 (0.034)	#SAKNAS!	1180 (0.1)
macrobrachium	3	2632 (0.034)	2293 (0.034)	1612 (0.066)
madrid	3	2632 (0.034)	2293 (0.034)	1612 (0.066)
malaysia	3	2632 (0.034)	#SAKNAS!	2492 (0.033)
mammal	3	2632 (0.034)	1457 (0.069)	2492 (0.033)
marina	3	2632 (0.034)	1457 (0.069)	2492 (0.033)
materials	3	2632 (0.034)	2293 (0.034)	#SAKNAS!
mathematical	3	2632 (0.034)	2293 (0.034)	2492 (0.033)
maturation	3	2632 (0.034)	2293 (0.034)	1612 (0.066)
maximus	3	2632 (0.034)	2293 (0.034)	#SAKNAS!
media	3	2632 (0.034)	#SAKNAS!	2492 (0.033)
mediator	3	2632 (0.034)	#SAKNAS!	#SAKNAS!
medium-term	3	2632 (0.034)	#SAKNAS!	2492 (0.033)
meeting	3	2632 (0.034)	1067 (0.1)	#SAKNAS!
membrane	3	2632 (0.034)	#SAKNAS!	2492 (0.033)
menopause	3	2632 (0.034)	#SAKNAS!	1612 (0.066)
menstrual	3	2632 (0.034)	2293 (0.034)	1612 (0.066)
mentally	3	2632 (0.034)	1067 (0.1)	#SAKNAS!
menu	3	2632 (0.034)	#SAKNAS!	1180 (0.1)
mesh	3	2632 (0.034)	1457 (0.069)	#SAKNAS!
microalgae	3	2632 (0.034)	2293 (0.034)	1612 (0.066)
microvascular	3	2632 (0.034)	1457 (0.069)	2492 (0.033)
migrant	3	2632 (0.034)	#SAKNAS!	1612 (0.066)
miniature	3	2632 (0.034)	1067 (0.1)	#SAKNAS!
minimal	3	2632 (0.034)	2293 (0.034)	#SAKNAS!
miocene	3	2632 (0.034)	1457 (0.069)	2492 (0.033)
misclassification	3	2632 (0.034)	#SAKNAS!	1612 (0.066)
mo	3	2632 (0.034)	2293 (0.034)	2492 (0.033)
mobility	3	2632 (0.034)	#SAKNAS!	2492 (0.033)
modality	3	2632 (0.034)	2293 (0.034)	1612 (0.066)
modify	3	2632 (0.034)	2293 (0.034)	2492 (0.033)
modifying	3	2632 (0.034)	2293 (0.034)	1612 (0.066)

monkey	3	2632 (0.034)	#SAKNAS!	1180 (0.1)
month	3	2632 (0.034)	#SAKNAS!	2492 (0.033)
monuste	3	2632 (0.034)	1067 (0.1)	#SAKNAS!
moose	3	2632 (0.034)	1067 (0.1)	#SAKNAS!
mori	3	2632 (0.034)	2293 (0.034)	2492 (0.033)
morphological	3	2632 (0.034)	2293 (0.034)	1612 (0.066)
mosquitoes	3	2632 (0.034)	#SAKNAS!	1612 (0.066)
mother	3	2632 (0.034)	#SAKNAS!	2492 (0.033)
mouths	3	2632 (0.034)	2293 (0.034)	1612 (0.066)
multi-centre	3	2632 (0.034)	2293 (0.034)	1612 (0.066)
multicomponent	3	2632 (0.034)	#SAKNAS!	2492 (0.033)
multi-professional	3	2632 (0.034)	#SAKNAS!	1612 (0.066)
mutation	3	2632 (0.034)	2293 (0.034)	2492 (0.033)
mykiss	3	2632 (0.034)	1067 (0.1)	#SAKNAS!
myth	3	2632 (0.034)	2293 (0.034)	1612 (0.066)
myzus	3	2632 (0.034)	#SAKNAS!	1612 (0.066)
nadya-senpe	3	2632 (0.034)	#SAKNAS!	1612 (0.066)
narrative	3	2632 (0.034)	2293 (0.034)	#SAKNAS!
nation	3	2632 (0.034)	2293 (0.034)	1612 (0.066)
native	3	2632 (0.034)	#SAKNAS!	1612 (0.066)
necessary	3	2632 (0.034)	2293 (0.034)	1612 (0.066)
necrotising	3	2632 (0.034)	2293 (0.034)	2492 (0.033)
neotropical	3	2632 (0.034)	#SAKNAS!	1612 (0.066)
neurodevelopmental	3	2632 (0.034)	2293 (0.034)	#SAKNAS!
neuroendocrine	3	2632 (0.034)	1457 (0.069)	#SAKNAS!
neurology	3	2632 (0.034)	#SAKNAS!	2492 (0.033)
neutral	3	2632 (0.034)	#SAKNAS!	2492 (0.033)
nicu	3	2632 (0.034)	#SAKNAS!	2492 (0.033)
noctuidae	3	2632 (0.034)	2293 (0.034)	1612 (0.066)
noma	3	2632 (0.034)	#SAKNAS!	2492 (0.033)
nonalcoholic	3	2632 (0.034)	#SAKNAS!	2492 (0.033)
non-diabetic	3	2632 (0.034)	#SAKNAS!	1612 (0.066)
nondysphagic	3	2632 (0.034)	2293 (0.034)	#SAKNAS!
non-invasive	3	2632 (0.034)	2293 (0.034)	2492 (0.033)
nonnutritive	3	2632 (0.034)	1457 (0.069)	#SAKNAS!
non-smokers	3	2632 (0.034)	#SAKNAS!	2492 (0.033)
nordic	3	2632 (0.034)	#SAKNAS!	1612 (0.066)
northeastern	3	2632 (0.034)	#SAKNAS!	1612 (0.066)
norwegian	3	2632 (0.034)	#SAKNAS!	1612 (0.066)
nucleatum	3	2632 (0.034)	1457 (0.069)	2492 (0.033)
nurse	3	2632 (0.034)	#SAKNAS!	1612 (0.066)
nut	3	2632 (0.034)	2293 (0.034)	#SAKNAS!
observations	3	2632 (0.034)	2293 (0.034)	1612 (0.066)

occurring	3	2632 (0.034)	2293 (0.034)	1612 (0.066)
octopus	3	2632 (0.034)	#SAKNAS!	#SAKNAS!
oculopharyngeal	3	2632 (0.034)	2293 (0.034)	#SAKNAS!
odontological	3	2632 (0.034)	2293 (0.034)	#SAKNAS!
oesophagitis	3	2632 (0.034)	#SAKNAS!	1612 (0.066)
oncorhynchus	3	2632 (0.034)	1067 (0.1)	#SAKNAS!
one-stage	3	2632 (0.034)	1457 (0.069)	#SAKNAS!
ontogenetic	3	2632 (0.034)	2293 (0.034)	2492 (0.033)
ontogeny	3	2632 (0.034)	1457 (0.069)	#SAKNAS!
opinion	3	2632 (0.034)	#SAKNAS!	2492 (0.033)
opinions	3	2632 (0.034)	2293 (0.034)	2492 (0.033)
opioid	3	2632 (0.034)	#SAKNAS!	2492 (0.033)
orally	3	2632 (0.034)	2293 (0.034)	2492 (0.033)
orange	3	2632 (0.034)	2293 (0.034)	2492 (0.033)
orca	3	2632 (0.034)	#SAKNAS!	1612 (0.066)
orchestoidea	3	2632 (0.034)	1457 (0.069)	2492 (0.033)
original	3	2632 (0.034)	#SAKNAS!	#SAKNAS!
origins	3	2632 (0.034)	1457 (0.069)	2492 (0.033)
oscillation	3	2632 (0.034)	2293 (0.034)	2492 (0.033)
osteonecrosis	3	2632 (0.034)	2293 (0.034)	2492 (0.033)
osteophyte	3	2632 (0.034)	#SAKNAS!	1612 (0.066)
outbreak	3	2632 (0.034)	#SAKNAS!	#SAKNAS!
overlap	3	2632 (0.034)	2293 (0.034)	#SAKNAS!
pan	3	2632 (0.034)	#SAKNAS!	1180 (0.1)
pandas	3	2632 (0.034)	2293 (0.034)	1612 (0.066)
paradox	3	2632 (0.034)	2293 (0.034)	2492 (0.033)
paraventricular	3	2632 (0.034)	1067 (0.1)	#SAKNAS!
partitioning	3	2632 (0.034)	1457 (0.069)	2492 (0.033)
partnership	3	2632 (0.034)	#SAKNAS!	2492 (0.033)
passerine	3	2632 (0.034)	1067 (0.1)	#SAKNAS!
passive	3	2632 (0.034)	1067 (0.1)	#SAKNAS!
patagonia	3	2632 (0.034)	2293 (0.034)	2492 (0.033)
patch	3	2632 (0.034)	2293 (0.034)	1612 (0.066)
paternal	3	2632 (0.034)	#SAKNAS!	2492 (0.033)
path	3	2632 (0.034)	#SAKNAS!	1612 (0.066)
pathological	3	2632 (0.034)	#SAKNAS!	2492 (0.033)
pcr	3	2632 (0.034)	#SAKNAS!	1180 (0.1)
peak	3	2632 (0.034)	#SAKNAS!	1612 (0.066)
pecking	3	2632 (0.034)	2293 (0.034)	1612 (0.066)
pelotas	3	2632 (0.034)	2293 (0.034)	2492 (0.033)
peptides	3	2632 (0.034)	1457 (0.069)	#SAKNAS!
percentage	3	2632 (0.034)	#SAKNAS!	1180 (0.1)
perch	3	2632 (0.034)	1457 (0.069)	#SAKNAS!

peri-implant	3	2632 (0.034)	#SAKNAS!	2492 (0.033)
periodontally	3	2632 (0.034)	#SAKNAS!	#SAKNAS!
peristalsis	3	2632 (0.034)	#SAKNAS!	1180 (0.1)
persicae	3	2632 (0.034)	#SAKNAS!	1612 (0.066)
person	3	2632 (0.034)	2293 (0.034)	2492 (0.033)
phenylketonuria	3	2632 (0.034)	1457 (0.069)	#SAKNAS!
philippines	3	2632 (0.034)	1457 (0.069)	#SAKNAS!
phosphorus	3	2632 (0.034)	#SAKNAS!	1612 (0.066)
physically	3	2632 (0.034)	2293 (0.034)	#SAKNAS!
physicochemical	3	2632 (0.034)	#SAKNAS!	#SAKNAS!
physico-chemical	3	2632 (0.034)	#SAKNAS!	#SAKNAS!
phytoplankton	3	2632 (0.034)	1457 (0.069)	#SAKNAS!
pieridae	3	2632 (0.034)	1067 (0.1)	#SAKNAS!
pituitary	3	2632 (0.034)	#SAKNAS!	1612 (0.066)
placental	3	2632 (0.034)	2293 (0.034)	#SAKNAS!
planus	3	2632 (0.034)	2293 (0.034)	2492 (0.033)
plate	3	2632 (0.034)	2293 (0.034)	2492 (0.033)
play	3	2632 (0.034)	2293 (0.034)	2492 (0.033)
plication	3	2632 (0.034)	1457 (0.069)	2492 (0.033)
plummer-vinson	3	2632 (0.034)	2293 (0.034)	2492 (0.033)
point-of-care	3	2632 (0.034)	#SAKNAS!	1612 (0.066)
polyphagous	3	2632 (0.034)	1457 (0.069)	2492 (0.033)
post-extubation	3	2632 (0.034)	#SAKNAS!	2492 (0.033)
post-ingestive	3	2632 (0.034)	1457 (0.069)	#SAKNAS!
postprandial	3	2632 (0.034)	2293 (0.034)	#SAKNAS!
poultry	3	2632 (0.034)	2293 (0.034)	#SAKNAS!
power	3	2632 (0.034)	2293 (0.034)	#SAKNAS!
pre-diabetes	3	2632 (0.034)	#SAKNAS!	1612 (0.066)
prefrontal	3	2632 (0.034)	#SAKNAS!	2492 (0.033)
prehistoric	3	2632 (0.034)	1457 (0.069)	2492 (0.033)
prescription	3	2632 (0.034)	#SAKNAS!	2492 (0.033)
presentations	3	2632 (0.034)	#SAKNAS!	1612 (0.066)
probing	3	2632 (0.034)	#SAKNAS!	1180 (0.1)
proceedings	3	2632 (0.034)	2293 (0.034)	1612 (0.066)
productive	3	2632 (0.034)	2293 (0.034)	1612 (0.066)
progenitor	3	2632 (0.034)	2293 (0.034)	2492 (0.033)
proinflammatory	3	2632 (0.034)	2293 (0.034)	2492 (0.033)
proper	3	2632 (0.034)	2293 (0.034)	2492 (0.033)
propithecus	3	2632 (0.034)	2293 (0.034)	1612 (0.066)
prosthodontic	3	2632 (0.034)	#SAKNAS!	1180 (0.1)
protection	3	2632 (0.034)	2293 (0.034)	1612 (0.066)
proteomic	3	2632 (0.034)	#SAKNAS!	2492 (0.033)
provides	3	2632 (0.034)	1457 (0.069)	2492 (0.033)

provinces	3	2632 (0.034)	2293 (0.034)	#SAKNAS!
psychometric	3	2632 (0.034)	#SAKNAS!	1612 (0.066)
psyllid	3	2632 (0.034)	#SAKNAS!	2492 (0.033)
puree	3	2632 (0.034)	#SAKNAS!	#SAKNAS!
qatar	3	2632 (0.034)	#SAKNAS!	2492 (0.033)
quality-of-life	3	2632 (0.034)	1457 (0.069)	#SAKNAS!
quantifying	3	2632 (0.034)	1457 (0.069)	#SAKNAS!
quarantine	3	2632 (0.034)	#SAKNAS!	#SAKNAS!
r	3	2632 (0.034)	2293 (0.034)	2492 (0.033)
rainbow	3	2632 (0.034)	1067 (0.1)	#SAKNAS!
readiness	3	2632 (0.034)	2293 (0.034)	#SAKNAS!
real	3	2632 (0.034)	#SAKNAS!	#SAKNAS!
reality	3	2632 (0.034)	2293 (0.034)	1612 (0.066)
really	3	2632 (0.034)	1457 (0.069)	2492 (0.033)
reasons	3	2632 (0.034)	2293 (0.034)	2492 (0.033)
recessive	3	2632 (0.034)	1067 (0.1)	#SAKNAS!
recipes	3	2632 (0.034)	2293 (0.034)	2492 (0.033)
recombinant	3	2632 (0.034)	1067 (0.1)	#SAKNAS!
record	3	2632 (0.034)	1067 (0.1)	#SAKNAS!
recordings	3	2632 (0.034)	#SAKNAS!	2492 (0.033)
redo	3	2632 (0.034)	#SAKNAS!	1612 (0.066)
referral	3	2632 (0.034)	2293 (0.034)	2492 (0.033)
reflections	3	2632 (0.034)	2293 (0.034)	2492 (0.033)
replace	3	2632 (0.034)	#SAKNAS!	2492 (0.033)
representations	3	2632 (0.034)	1457 (0.069)	2492 (0.033)
requirement	3	2632 (0.034)	2293 (0.034)	2492 (0.033)
restorative	3	2632 (0.034)	1457 (0.069)	2492 (0.033)
restricting	3	2632 (0.034)	#SAKNAS!	1612 (0.066)
result	3	2632 (0.034)	#SAKNAS!	2492 (0.033)
rhinitis	3	2632 (0.034)	#SAKNAS!	1612 (0.066)
rickets	3	2632 (0.034)	2293 (0.034)	1612 (0.066)
ring-tailed	3	2632 (0.034)	1067 (0.1)	#SAKNAS!
rock	3	2632 (0.034)	2293 (0.034)	2492 (0.033)
roma	3	2632 (0.034)	2293 (0.034)	2492 (0.033)
roughage	3	2632 (0.034)	2293 (0.034)	1612 (0.066)
salmonella	3	2632 (0.034)	2293 (0.034)	#SAKNAS!
samples	3	2632 (0.034)	#SAKNAS!	2492 (0.033)
sampling	3	2632 (0.034)	2293 (0.034)	2492 (0.033)
schizophrenic	3	2632 (0.034)	#SAKNAS!	2492 (0.033)
scleroderma	3	2632 (0.034)	2293 (0.034)	2492 (0.033)
screenings	3	2632 (0.034)	#SAKNAS!	1612 (0.066)
sealant	3	2632 (0.034)	2293 (0.034)	2492 (0.033)
seasons	3	2632 (0.034)	#SAKNAS!	2492 (0.033)

second	3	2632 (0.034)	#SAKNAS!	2492 (0.033)
sediment	3	2632 (0.034)	1457 (0.069)	#SAKNAS!
seen	3	2632 (0.034)	1457 (0.069)	#SAKNAS!
segment	3	2632 (0.034)	2293 (0.034)	2492 (0.033)
segregation	3	2632 (0.034)	1457 (0.069)	#SAKNAS!
selectively	3	2632 (0.034)	1067 (0.1)	#SAKNAS!
self-feeding	3	2632 (0.034)	2293 (0.034)	1612 (0.066)
self-medication	3	2632 (0.034)	1457 (0.069)	2492 (0.033)
self-perception	3	2632 (0.034)	#SAKNAS!	2492 (0.033)
semi-intensive	3	2632 (0.034)	1457 (0.069)	#SAKNAS!
seniors	3	2632 (0.034)	#SAKNAS!	2492 (0.033)
separation	3	2632 (0.034)	1457 (0.069)	2492 (0.033)
sequelae	3	2632 (0.034)	2293 (0.034)	1612 (0.066)
sequencing	3	2632 (0.034)	#SAKNAS!	1612 (0.066)
sequential	3	2632 (0.034)	1457 (0.069)	2492 (0.033)
set	3	2632 (0.034)	2293 (0.034)	#SAKNAS!
seventh	3	2632 (0.034)	#SAKNAS!	#SAKNAS!
seven-year	3	2632 (0.034)	2293 (0.034)	2492 (0.033)
shelter	3	2632 (0.034)	1457 (0.069)	#SAKNAS!
shortage	3	2632 (0.034)	2293 (0.034)	1612 (0.066)
sialorrhea	3	2632 (0.034)	2293 (0.034)	2492 (0.033)
side	3	2632 (0.034)	2293 (0.034)	1612 (0.066)
signalling	3	2632 (0.034)	1067 (0.1)	#SAKNAS!
silent	3	2632 (0.034)	#SAKNAS!	2492 (0.033)
silkworm	3	2632 (0.034)	1457 (0.069)	#SAKNAS!
single-centre	3	2632 (0.034)	#SAKNAS!	1612 (0.066)
slug	3	2632 (0.034)	1067 (0.1)	#SAKNAS!
snacking	3	2632 (0.034)	1067 (0.1)	#SAKNAS!
socially	3	2632 (0.034)	1457 (0.069)	#SAKNAS!
soil	3	2632 (0.034)	2293 (0.034)	1612 (0.066)
solids	3	2632 (0.034)	#SAKNAS!	2492 (0.033)
solution	3	2632 (0.034)	2293 (0.034)	1612 (0.066)
sorghum	3	2632 (0.034)	#SAKNAS!	1180 (0.1)
southeastern	3	2632 (0.034)	2293 (0.034)	1612 (0.066)
soy	3	2632 (0.034)	2293 (0.034)	2492 (0.033)
sp	3	2632 (0.034)	#SAKNAS!	1180 (0.1)
space	3	2632 (0.034)	#SAKNAS!	#SAKNAS!
sparus	3	2632 (0.034)	1067 (0.1)	#SAKNAS!
specialist	3	2632 (0.034)	1067 (0.1)	#SAKNAS!
specimens	3	2632 (0.034)	#SAKNAS!	1180 (0.1)
spodoptera	3	2632 (0.034)	#SAKNAS!	1612 (0.066)
sports	3	2632 (0.034)	#SAKNAS!	1180 (0.1)
spring	3	2632 (0.034)	#SAKNAS!	1612 (0.066)

squirrel	3	2632 (0.034)	2293 (0.034)	2492 (0.033)
standards	3	2632 (0.034)	1457 (0.069)	2492 (0.033)
stepwise	3	2632 (0.034)	2293 (0.034)	#SAKNAS!
still	3	2632 (0.034)	2293 (0.034)	#SAKNAS!
stimulating	3	2632 (0.034)	2293 (0.034)	#SAKNAS!
stimuli	3	2632 (0.034)	2293 (0.034)	1612 (0.066)
stone	3	2632 (0.034)	#SAKNAS!	1180 (0.1)
strain	3	2632 (0.034)	2293 (0.034)	#SAKNAS!
stratification	3	2632 (0.034)	#SAKNAS!	1612 (0.066)
stream	3	2632 (0.034)	2293 (0.034)	2492 (0.033)
streams	3	2632 (0.034)	1457 (0.069)	#SAKNAS!
student	3	2632 (0.034)	1457 (0.069)	2492 (0.033)
subpopulation	3	2632 (0.034)	#SAKNAS!	1612 (0.066)
substituting	3	2632 (0.034)	2293 (0.034)	2492 (0.033)
substrate	3	2632 (0.034)	1457 (0.069)	2492 (0.033)
subtype	3	2632 (0.034)	1457 (0.069)	2492 (0.033)
suffering	3	2632 (0.034)	1457 (0.069)	2492 (0.033)
suitable	3	2632 (0.034)	#SAKNAS!	2492 (0.033)
summary	3	2632 (0.034)	1457 (0.069)	#SAKNAS!
supracricoid	3	2632 (0.034)	1457 (0.069)	2492 (0.033)
supragingival	3	2632 (0.034)	#SAKNAS!	2492 (0.033)
suprahyoid	3	2632 (0.034)	#SAKNAS!	2492 (0.033)
supranuclear	3	2632 (0.034)	#SAKNAS!	#SAKNAS!
surfaces	3	2632 (0.034)	2293 (0.034)	2492 (0.033)
survive	3	2632 (0.034)	#SAKNAS!	2492 (0.033)
sustainability	3	2632 (0.034)	#SAKNAS!	1180 (0.1)
swallowability	3	2632 (0.034)	#SAKNAS!	#SAKNAS!
swal-qol	3	2632 (0.034)	1457 (0.069)	#SAKNAS!
sward	3	2632 (0.034)	1457 (0.069)	#SAKNAS!
swelling	3	2632 (0.034)	2293 (0.034)	2492 (0.033)
switching	3	2632 (0.034)	2293 (0.034)	#SAKNAS!
sydney	3	2632 (0.034)	2293 (0.034)	#SAKNAS!
synergistic	3	2632 (0.034)	#SAKNAS!	2492 (0.033)
syrian	3	2632 (0.034)	#SAKNAS!	2492 (0.033)
tabaci	3	2632 (0.034)	2293 (0.034)	1612 (0.066)
takashimadaira	3	2632 (0.034)	#SAKNAS!	1612 (0.066)
tamarins	3	2632 (0.034)	2293 (0.034)	#SAKNAS!
tannerella	3	2632 (0.034)	1457 (0.069)	#SAKNAS!
tannins	3	2632 (0.034)	1067 (0.1)	#SAKNAS!
tap	3	2632 (0.034)	#SAKNAS!	1180 (0.1)
task	3	2632 (0.034)	1067 (0.1)	#SAKNAS!
temperament	3	2632 (0.034)	#SAKNAS!	1612 (0.066)
temperatures	3	2632 (0.034)	#SAKNAS!	1180 (0.1)

ten	3	2632 (0.034)	2293 (0.034)	#SAKNAS!
terminal	3	2632 (0.034)	2293 (0.034)	2492 (0.033)
terminology	3	2632 (0.034)	#SAKNAS!	2492 (0.033)
terrestrial	3	2632 (0.034)	2293 (0.034)	1612 (0.066)
textural	3	2632 (0.034)	#SAKNAS!	#SAKNAS!
textures	3	2632 (0.034)	2293 (0.034)	1612 (0.066)
them	3	2632 (0.034)	2293 (0.034)	#SAKNAS!
think	3	2632 (0.034)	#SAKNAS!	2492 (0.033)
thrive	3	2632 (0.034)	1067 (0.1)	#SAKNAS!
thrombectomy	3	2632 (0.034)	#SAKNAS!	#SAKNAS!
thuringiensis	3	2632 (0.034)	1457 (0.069)	2492 (0.033)
thyroidectomy	3	2632 (0.034)	2293 (0.034)	#SAKNAS!
tnf-帢	3	2632 (0.034)	#SAKNAS!	2492 (0.033)
tracheal	3	2632 (0.034)	2293 (0.034)	2492 (0.033)
transcriptome	3	2632 (0.034)	#SAKNAS!	1612 (0.066)
transit	3	2632 (0.034)	#SAKNAS!	2492 (0.033)
transport	3	2632 (0.034)	2293 (0.034)	#SAKNAS!
transtheoretical	3	2632 (0.034)	2293 (0.034)	2492 (0.033)
tribolium	3	2632 (0.034)	2293 (0.034)	1612 (0.066)
trichogramma	3	2632 (0.034)	2293 (0.034)	1612 (0.066)
triglycerides	3	2632 (0.034)	2293 (0.034)	#SAKNAS!
trimester	3	2632 (0.034)	#SAKNAS!	2492 (0.033)
troglodytes	3	2632 (0.034)	#SAKNAS!	1180 (0.1)
tsurugaya	3	2632 (0.034)	#SAKNAS!	1180 (0.1)
tuberculata	3	2632 (0.034)	1457 (0.069)	2492 (0.033)
turnover	3	2632 (0.034)	1067 (0.1)	#SAKNAS!
twin	3	2632 (0.034)	2293 (0.034)	2492 (0.033)
two-sample	3	2632 (0.034)	#SAKNAS!	#SAKNAS!
two-spotted	3	2632 (0.034)	#SAKNAS!	1180 (0.1)
vagal	3	2632 (0.034)	1457 (0.069)	2492 (0.033)
vagotomy	3	2632 (0.034)	1067 (0.1)	#SAKNAS!
validate	3	2632 (0.034)	2293 (0.034)	#SAKNAS!
vannamei	3	2632 (0.034)	2293 (0.034)	1612 (0.066)
variant	3	2632 (0.034)	2293 (0.034)	2492 (0.033)
varying	3	2632 (0.034)	2293 (0.034)	2492 (0.033)
ventricular	3	2632 (0.034)	1067 (0.1)	#SAKNAS!
ventromedial	3	2632 (0.034)	1457 (0.069)	#SAKNAS!
vi	3	2632 (0.034)	#SAKNAS!	1180 (0.1)
viewing	3	2632 (0.034)	2293 (0.034)	1612 (0.066)
vii	3	2632 (0.034)	#SAKNAS!	#SAKNAS!
vocal	3	2632 (0.034)	1457 (0.069)	2492 (0.033)
volume-viscosity	3	2632 (0.034)	1457 (0.069)	2492 (0.033)
volunteers	3	2632 (0.034)	1457 (0.069)	2492 (0.033)

wasp		3	2632 (0.034)	2293 (0.034)	2492 (0.033)
waste		3	2632 (0.034)	2293 (0.034)	#SAKNAS!
week		3	2632 (0.034)	2293 (0.034)	1612 (0.066)
weevil		3	2632 (0.034)	1067 (0.1)	#SAKNAS!
wet		3	2632 (0.034)	#SAKNAS!	1180 (0.1)
whales		3	2632 (0.034)	2293 (0.034)	#SAKNAS!
whelk		3	2632 (0.034)	1457 (0.069)	#SAKNAS!
whose		3	2632 (0.034)	1457 (0.069)	2492 (0.033)
will		3	2632 (0.034)	#SAKNAS!	1612 (0.066)
winter		3	2632 (0.034)	2293 (0.034)	#SAKNAS!
woolly		3	2632 (0.034)	1457 (0.069)	2492 (0.033)
worker		3	2632 (0.034)	1457 (0.069)	2492 (0.033)
working-age		3	2632 (0.034)	#SAKNAS!	#SAKNAS!
worsen		3	2632 (0.034)	2293 (0.034)	2492 (0.033)
x-ray		3	2632 (0.034)	2293 (0.034)	#SAKNAS!
years-results		3	2632 (0.034)	#SAKNAS!	1612 (0.066)
yogurt		3	2632 (0.034)	2293 (0.034)	1612 (0.066)
zebrafish		3	2632 (0.034)	#SAKNAS!	#SAKNAS!
zimbabwe		3	2632 (0.034)	2293 (0.034)	#SAKNAS!
zn		3	2632 (0.034)	#SAKNAS!	1612 (0.066)
zooplankton		3	2632 (0.034)	1067 (0.1)	#SAKNAS!
	0,2	2	3370 (0.023)	1457 (0.069)	#SAKNAS!
	2	2	3370 (0.023)	2293 (0.034)	#SAKNAS!
	1924	2	3370 (0.023)	#SAKNAS!	1612 (0.066)
	1995	2	3370 (0.023)	1457 (0.069)	#SAKNAS!
	2001	2	3370 (0.023)	1457 (0.069)	#SAKNAS!
	2002	2	3370 (0.023)	#SAKNAS!	#SAKNAS!
	2030	2	3370 (0.023)	#SAKNAS!	#SAKNAS!
	5289	2	3370 (0.023)	#SAKNAS!	2492 (0.033)
	17938	2	3370 (0.023)	#SAKNAS!	2492 (0.033)
	45693	2	3370 (0.023)	2293 (0.034)	#SAKNAS!
	45722	2	3370 (0.023)	#SAKNAS!	2492 (0.033)
	45942	2	3370 (0.023)	#SAKNAS!	2492 (0.033)
	46005	2	3370 (0.023)	2293 (0.034)	2492 (0.033)
&gt		2	3370 (0.023)	#SAKNAS!	2492 (0.033)
10-item		2	3370 (0.023)	#SAKNAS!	1612 (0.066)
11-year		2	3370 (0.023)	2293 (0.034)	2492 (0.033)
13-to		2	3370 (0.023)	#SAKNAS!	1612 (0.066)
13-year-old		2	3370 (0.023)	1457 (0.069)	#SAKNAS!
15-year		2	3370 (0.023)	#SAKNAS!	2492 (0.033)
1999-2012		2	3370 (0.023)	#SAKNAS!	1612 (0.066)
1-year-old		2	3370 (0.023)	1457 (0.069)	#SAKNAS!
2.5-year-old		2	3370 (0.023)	1457 (0.069)	#SAKNAS!

2003-2004	2	3370 (0.023)	1457 (0.069)	#SAKNAS!
2005-2006	2	3370 (0.023)	#SAKNAS!	1612 (0.066)
2005-2016	2	3370 (0.023)	#SAKNAS!	#SAKNAS!
2007-2008	2	3370 (0.023)	2293 (0.034)	2492 (0.033)
2007-2015	2	3370 (0.023)	#SAKNAS!	2492 (0.033)
2007-2018	2	3370 (0.023)	#SAKNAS!	#SAKNAS!
2008-2014	2	3370 (0.023)	#SAKNAS!	1612 (0.066)
2011-2016	2	3370 (0.023)	#SAKNAS!	#SAKNAS!
2013-2018	2	3370 (0.023)	#SAKNAS!	#SAKNAS!
2020-2021	2	3370 (0.023)	#SAKNAS!	#SAKNAS!
20-year	2	3370 (0.023)	#SAKNAS!	2492 (0.033)
24-month	2	3370 (0.023)	#SAKNAS!	2492 (0.033)
2-ethylhexyl	2	3370 (0.023)	#SAKNAS!	2492 (0.033)
30-year	2	3370 (0.023)	2293 (0.034)	2492 (0.033)
35-	2	3370 (0.023)	1457 (0.069)	#SAKNAS!
3-5-year-old	2	3370 (0.023)	#SAKNAS!	2492 (0.033)
3-month	2	3370 (0.023)	#SAKNAS!	2492 (0.033)
3-to	2	3370 (0.023)	#SAKNAS!	2492 (0.033)
4-and	2	3370 (0.023)	2293 (0.034)	2492 (0.033)
4th	2	3370 (0.023)	#SAKNAS!	2492 (0.033)
4-year-old	2	3370 (0.023)	2293 (0.034)	2492 (0.033)
50-	2	3370 (0.023)	1457 (0.069)	#SAKNAS!
55-84	2	3370 (0.023)	2293 (0.034)	2492 (0.033)
5-fluorouracil	2	3370 (0.023)	1457 (0.069)	#SAKNAS!
60-70-year-old	2	3370 (0.023)	1457 (0.069)	#SAKNAS!
6-and	2	3370 (0.023)	2293 (0.034)	2492 (0.033)
6j	2	3370 (0.023)	2293 (0.034)	#SAKNAS!
6-month	2	3370 (0.023)	#SAKNAS!	1612 (0.066)
70-year-old	2	3370 (0.023)	#SAKNAS!	2492 (0.033)
7th	2	3370 (0.023)	#SAKNAS!	#SAKNAS!
7-year-olds	2	3370 (0.023)	#SAKNAS!	#SAKNAS!
8th	2	3370 (0.023)	#SAKNAS!	#SAKNAS!
90-year-old	2	3370 (0.023)	#SAKNAS!	#SAKNAS!
aap	2	3370 (0.023)	#SAKNAS!	#SAKNAS!
abalone	2	3370 (0.023)	2293 (0.034)	2492 (0.033)
ablation	2	3370 (0.023)	#SAKNAS!	#SAKNAS!
able	2	3370 (0.023)	#SAKNAS!	2492 (0.033)
absolute	2	3370 (0.023)	1457 (0.069)	#SAKNAS!
acceleration	2	3370 (0.023)	#SAKNAS!	#SAKNAS!
accessible	2	3370 (0.023)	2293 (0.034)	#SAKNAS!
accommodation	2	3370 (0.023)	2293 (0.034)	#SAKNAS!
accra	2	3370 (0.023)	1457 (0.069)	#SAKNAS!
acculturation	2	3370 (0.023)	#SAKNAS!	2492 (0.033)

accumbens	2	3370 (0.023)	1457 (0.069)	#SAKNAS!
acdf	2	3370 (0.023)	#SAKNAS!	2492 (0.033)
achieve	2	3370 (0.023)	2293 (0.034)	#SAKNAS!
acidification	2	3370 (0.023)	#SAKNAS!	2492 (0.033)
acidosis	2	3370 (0.023)	2293 (0.034)	2492 (0.033)
actinobacillus	2	3370 (0.023)	1457 (0.069)	#SAKNAS!
activator	2	3370 (0.023)	2293 (0.034)	#SAKNAS!
acylpeptidyl	2	3370 (0.023)	#SAKNAS!	2492 (0.033)
addiction	2	3370 (0.023)	#SAKNAS!	1612 (0.066)
adductor	2	3370 (0.023)	#SAKNAS!	2492 (0.033)
adequate	2	3370 (0.023)	2293 (0.034)	2492 (0.033)
adequately	2	3370 (0.023)	2293 (0.034)	#SAKNAS!
adjacent	2	3370 (0.023)	2293 (0.034)	#SAKNAS!
adjusting	2	3370 (0.023)	#SAKNAS!	2492 (0.033)
adjustment	2	3370 (0.023)	#SAKNAS!	2492 (0.033)
ad-libitum	2	3370 (0.023)	2293 (0.034)	2492 (0.033)
admissions	2	3370 (0.023)	#SAKNAS!	#SAKNAS!
adrenergic	2	3370 (0.023)	#SAKNAS!	1612 (0.066)
adversely	2	3370 (0.023)	#SAKNAS!	2492 (0.033)
aegypti	2	3370 (0.023)	2293 (0.034)	#SAKNAS!
affairs	2	3370 (0.023)	1457 (0.069)	#SAKNAS!
african-american	2	3370 (0.023)	2293 (0.034)	2492 (0.033)
age-specific	2	3370 (0.023)	#SAKNAS!	1612 (0.066)
aggregation	2	3370 (0.023)	2293 (0.034)	2492 (0.033)
agonist	2	3370 (0.023)	2293 (0.034)	2492 (0.033)
agonistic	2	3370 (0.023)	2293 (0.034)	#SAKNAS!
agreement	2	3370 (0.023)	2293 (0.034)	2492 (0.033)
airflow	2	3370 (0.023)	#SAKNAS!	1612 (0.066)
ajman	2	3370 (0.023)	1457 (0.069)	#SAKNAS!
albopictus	2	3370 (0.023)	2293 (0.034)	2492 (0.033)
alces	2	3370 (0.023)	1457 (0.069)	#SAKNAS!
alert	2	3370 (0.023)	#SAKNAS!	2492 (0.033)
alfalfa	2	3370 (0.023)	#SAKNAS!	1612 (0.066)
algeria	2	3370 (0.023)	2293 (0.034)	#SAKNAS!
algerian	2	3370 (0.023)	#SAKNAS!	2492 (0.033)
alginate	2	3370 (0.023)	#SAKNAS!	2492 (0.033)
algorithms	2	3370 (0.023)	#SAKNAS!	#SAKNAS!
alignment	2	3370 (0.023)	#SAKNAS!	2492 (0.033)
alkaloid	2	3370 (0.023)	1457 (0.069)	#SAKNAS!
allele	2	3370 (0.023)	#SAKNAS!	1612 (0.066)
alleviates	2	3370 (0.023)	2293 (0.034)	#SAKNAS!
alleviation	2	3370 (0.023)	#SAKNAS!	1612 (0.066)
allobates	2	3370 (0.023)	2293 (0.034)	#SAKNAS!

allocation	2	3370 (0.023)	2293 (0.034)	2492 (0.033)
alouatta	2	3370 (0.023)	2293 (0.034)	2492 (0.033)
alpine	2	3370 (0.023)	2293 (0.034)	#SAKNAS!
altitude	2	3370 (0.023)	#SAKNAS!	1612 (0.066)
amazonia	2	3370 (0.023)	#SAKNAS!	2492 (0.033)
ambient	2	3370 (0.023)	1457 (0.069)	#SAKNAS!
americana	2	3370 (0.023)	1457 (0.069)	#SAKNAS!
ammp-8	2	3370 (0.023)	#SAKNAS!	2492 (0.033)
amounts	2	3370 (0.023)	#SAKNAS!	#SAKNAS!
amphipod	2	3370 (0.023)	2293 (0.034)	2492 (0.033)
amyloidosis	2	3370 (0.023)	#SAKNAS!	2492 (0.033)
amyotrophic-lateral-sclerosis	2	3370 (0.023)	1457 (0.069)	#SAKNAS!
anaerobic	2	3370 (0.023)	#SAKNAS!	2492 (0.033)
analgesic	2	3370 (0.023)	#SAKNAS!	2492 (0.033)
analyzing	2	3370 (0.023)	2293 (0.034)	2492 (0.033)
anastrepha	2	3370 (0.023)	1457 (0.069)	#SAKNAS!
ancient	2	3370 (0.023)	#SAKNAS!	#SAKNAS!
andean	2	3370 (0.023)	#SAKNAS!	2492 (0.033)
angina	2	3370 (0.023)	#SAKNAS!	1612 (0.066)
ankylosing	2	3370 (0.023)	2293 (0.034)	2492 (0.033)
anodal	2	3370 (0.023)	#SAKNAS!	1612 (0.066)
anopheles	2	3370 (0.023)	2293 (0.034)	2492 (0.033)
anorectic	2	3370 (0.023)	1457 (0.069)	#SAKNAS!
anorexic	2	3370 (0.023)	2293 (0.034)	2492 (0.033)
anorexigenic	2	3370 (0.023)	#SAKNAS!	#SAKNAS!
anril	2	3370 (0.023)	#SAKNAS!	1612 (0.066)
anser	2	3370 (0.023)	2293 (0.034)	#SAKNAS!
antagonist	2	3370 (0.023)	1457 (0.069)	#SAKNAS!
antibacterial	2	3370 (0.023)	#SAKNAS!	2492 (0.033)
antibiotic	2	3370 (0.023)	#SAKNAS!	2492 (0.033)
anticholinergics	2	3370 (0.023)	#SAKNAS!	1612 (0.066)
antidiabetic	2	3370 (0.023)	#SAKNAS!	1612 (0.066)
antipsychotic	2	3370 (0.023)	2293 (0.034)	#SAKNAS!
apes	2	3370 (0.023)	2293 (0.034)	#SAKNAS!
app	2	3370 (0.023)	#SAKNAS!	2492 (0.033)
appearance	2	3370 (0.023)	1457 (0.069)	#SAKNAS!
appetite-regulating	2	3370 (0.023)	2293 (0.034)	2492 (0.033)
appetites	2	3370 (0.023)	1457 (0.069)	#SAKNAS!
applying	2	3370 (0.023)	#SAKNAS!	2492 (0.033)
approximal	2	3370 (0.023)	1457 (0.069)	#SAKNAS!
approximation	2	3370 (0.023)	#SAKNAS!	2492 (0.033)
aqueous	2	3370 (0.023)	#SAKNAS!	2492 (0.033)
araneae	2	3370 (0.023)	1457 (0.069)	#SAKNAS!

arch	2	3370 (0.023)	#SAKNAS!	1612 (0.066)
arcuate	2	3370 (0.023)	#SAKNAS!	2492 (0.033)
argyrosomus	2	3370 (0.023)	#SAKNAS!	#SAKNAS!
arizona	2	3370 (0.023)	1457 (0.069)	#SAKNAS!
arm	2	3370 (0.023)	2293 (0.034)	#SAKNAS!
aromatic	2	3370 (0.023)	#SAKNAS!	#SAKNAS!
aromobatidae	2	3370 (0.023)	2293 (0.034)	#SAKNAS!
arsenic	2	3370 (0.023)	#SAKNAS!	2492 (0.033)
artemia	2	3370 (0.023)	2293 (0.034)	2492 (0.033)
ash	2	3370 (0.023)	1457 (0.069)	#SAKNAS!
asians	2	3370 (0.023)	2293 (0.034)	2492 (0.033)
aspect	2	3370 (0.023)	2293 (0.034)	#SAKNAS!
aspirin	2	3370 (0.023)	#SAKNAS!	1612 (0.066)
assay	2	3370 (0.023)	#SAKNAS!	1612 (0.066)
assignment	2	3370 (0.023)	2293 (0.034)	2492 (0.033)
asthmatics	2	3370 (0.023)	#SAKNAS!	#SAKNAS!
atcc	2	3370 (0.023)	#SAKNAS!	2492 (0.033)
atkinson	2	3370 (0.023)	1457 (0.069)	#SAKNAS!
atlas	2	3370 (0.023)	#SAKNAS!	2492 (0.033)
atmospheric	2	3370 (0.023)	2293 (0.034)	2492 (0.033)
atopic	2	3370 (0.023)	#SAKNAS!	2492 (0.033)
atp	2	3370 (0.023)	2293 (0.034)	2492 (0.033)
attainment	2	3370 (0.023)	2293 (0.034)	2492 (0.033)
attendance	2	3370 (0.023)	#SAKNAS!	1612 (0.066)
attenuation	2	3370 (0.023)	2293 (0.034)	2492 (0.033)
atypical	2	3370 (0.023)	1457 (0.069)	#SAKNAS!
auckland	2	3370 (0.023)	#SAKNAS!	1612 (0.066)
auratus	2	3370 (0.023)	#SAKNAS!	1612 (0.066)
auscultation	2	3370 (0.023)	#SAKNAS!	2492 (0.033)
australasian	2	3370 (0.023)	#SAKNAS!	2492 (0.033)
australostichopus	2	3370 (0.023)	2293 (0.034)	2492 (0.033)
austria	2	3370 (0.023)	1457 (0.069)	#SAKNAS!
authoritative	2	3370 (0.023)	1457 (0.069)	#SAKNAS!
auxiliary	2	3370 (0.023)	#SAKNAS!	2492 (0.033)
avahi	2	3370 (0.023)	1457 (0.069)	#SAKNAS!
avenues	2	3370 (0.023)	1457 (0.069)	#SAKNAS!
awaiting	2	3370 (0.023)	#SAKNAS!	1612 (0.066)
away	2	3370 (0.023)	2293 (0.034)	#SAKNAS!
azar	2	3370 (0.023)	#SAKNAS!	#SAKNAS!
background	2	3370 (0.023)	2293 (0.034)	#SAKNAS!
balsam	2	3370 (0.023)	1457 (0.069)	#SAKNAS!
bands	2	3370 (0.023)	2293 (0.034)	2492 (0.033)
bar	2	3370 (0.023)	#SAKNAS!	1612 (0.066)

barley	2	3370 (0.023)	#SAKNAS!	2492 (0.033)
barrett	2	3370 (0.023)	1457 (0.069)	#SAKNAS!
barrier	2	3370 (0.023)	2293 (0.034)	#SAKNAS!
based	2	3370 (0.023)	#SAKNAS!	2492 (0.033)
basin	2	3370 (0.023)	#SAKNAS!	1612 (0.066)
beads	2	3370 (0.023)	#SAKNAS!	#SAKNAS!
bear	2	3370 (0.023)	1457 (0.069)	#SAKNAS!
bears	2	3370 (0.023)	2293 (0.034)	2492 (0.033)
beginning	2	3370 (0.023)	#SAKNAS!	1612 (0.066)
behavioral-assessment	2	3370 (0.023)	1457 (0.069)	#SAKNAS!
belgium	2	3370 (0.023)	1457 (0.069)	#SAKNAS!
benthos	2	3370 (0.023)	1457 (0.069)	#SAKNAS!
biases	2	3370 (0.023)	2293 (0.034)	2492 (0.033)
bibliometric	2	3370 (0.023)	#SAKNAS!	#SAKNAS!
bigel	2	3370 (0.023)	#SAKNAS!	#SAKNAS!
bilateral	2	3370 (0.023)	#SAKNAS!	2492 (0.033)
bioaccumulation	2	3370 (0.023)	1457 (0.069)	#SAKNAS!
bioactive	2	3370 (0.023)	2293 (0.034)	2492 (0.033)
bioactivity	2	3370 (0.023)	2293 (0.034)	#SAKNAS!
biochemistry	2	3370 (0.023)	2293 (0.034)	2492 (0.033)
biocultural	2	3370 (0.023)	#SAKNAS!	2492 (0.033)
biodegradable	2	3370 (0.023)	2293 (0.034)	2492 (0.033)
biodiesel	2	3370 (0.023)	#SAKNAS!	2492 (0.033)
bioelectrical	2	3370 (0.023)	#SAKNAS!	#SAKNAS!
biologic	2	3370 (0.023)	2293 (0.034)	#SAKNAS!
biomass	2	3370 (0.023)	2293 (0.034)	2492 (0.033)
biopsies	2	3370 (0.023)	#SAKNAS!	2492 (0.033)
biopsy	2	3370 (0.023)	#SAKNAS!	#SAKNAS!
biting	2	3370 (0.023)	2293 (0.034)	#SAKNAS!
bmi-1	2	3370 (0.023)	2293 (0.034)	#SAKNAS!
bmi-for-age	2	3370 (0.023)	#SAKNAS!	#SAKNAS!
body-weight	2	3370 (0.023)	1457 (0.069)	#SAKNAS!
boron	2	3370 (0.023)	#SAKNAS!	#SAKNAS!
boundaries	2	3370 (0.023)	#SAKNAS!	1612 (0.066)
bovine	2	3370 (0.023)	2293 (0.034)	2492 (0.033)
bp	2	3370 (0.023)	2293 (0.034)	2492 (0.033)
brachionus	2	3370 (0.023)	#SAKNAS!	#SAKNAS!
braconidae	2	3370 (0.023)	2293 (0.034)	2492 (0.033)
breeds	2	3370 (0.023)	2293 (0.034)	2492 (0.033)
bridging	2	3370 (0.023)	2293 (0.034)	#SAKNAS!
broken	2	3370 (0.023)	2293 (0.034)	2492 (0.033)
brominated	2	3370 (0.023)	#SAKNAS!	#SAKNAS!
bronchial	2	3370 (0.023)	#SAKNAS!	2492 (0.033)

browser	2	3370 (0.023)	#SAKNAS!	1612 (0.066)
brush	2	3370 (0.023)	2293 (0.034)	2492 (0.033)
brushing	2	3370 (0.023)	#SAKNAS!	2492 (0.033)
buckthorn	2	3370 (0.023)	2293 (0.034)	#SAKNAS!
buffers	2	3370 (0.023)	#SAKNAS!	1612 (0.066)
bumblebees	2	3370 (0.023)	2293 (0.034)	2492 (0.033)
burns	2	3370 (0.023)	#SAKNAS!	1612 (0.066)
butterflyfish	2	3370 (0.023)	2293 (0.034)	2492 (0.033)
byproducts	2	3370 (0.023)	#SAKNAS!	2492 (0.033)
by-products	2	3370 (0.023)	#SAKNAS!	2492 (0.033)
c2-c7	2	3370 (0.023)	2293 (0.034)	2492 (0.033)
cacopsylla	2	3370 (0.023)	#SAKNAS!	1612 (0.066)
calanoid	2	3370 (0.023)	1457 (0.069)	#SAKNAS!
calonectris	2	3370 (0.023)	2293 (0.034)	2492 (0.033)
calories	2	3370 (0.023)	#SAKNAS!	2492 (0.033)
calotropis	2	3370 (0.023)	2293 (0.034)	2492 (0.033)
camel	2	3370 (0.023)	1457 (0.069)	#SAKNAS!
cameroon	2	3370 (0.023)	#SAKNAS!	#SAKNAS!
canadians	2	3370 (0.023)	1457 (0.069)	#SAKNAS!
canal	2	3370 (0.023)	#SAKNAS!	#SAKNAS!
cancer-a	2	3370 (0.023)	2293 (0.034)	#SAKNAS!
candidates	2	3370 (0.023)	#SAKNAS!	2492 (0.033)
candidatus	2	3370 (0.023)	#SAKNAS!	1612 (0.066)
cannabis	2	3370 (0.023)	#SAKNAS!	2492 (0.033)
cannibalistic	2	3370 (0.023)	2293 (0.034)	2492 (0.033)
capitata	2	3370 (0.023)	1457 (0.069)	#SAKNAS!
capra	2	3370 (0.023)	1457 (0.069)	#SAKNAS!
capsules	2	3370 (0.023)	1457 (0.069)	#SAKNAS!
captivity	2	3370 (0.023)	2293 (0.034)	2492 (0.033)
capture	2	3370 (0.023)	2293 (0.034)	#SAKNAS!
carassius	2	3370 (0.023)	#SAKNAS!	1612 (0.066)
carbohydrate-rich	2	3370 (0.023)	2293 (0.034)	#SAKNAS!
cardia	2	3370 (0.023)	1457 (0.069)	#SAKNAS!
caregiver-infant	2	3370 (0.023)	#SAKNAS!	2492 (0.033)
caries-a	2	3370 (0.023)	#SAKNAS!	#SAKNAS!
caring	2	3370 (0.023)	#SAKNAS!	2492 (0.033)
carnivora	2	3370 (0.023)	1457 (0.069)	#SAKNAS!
carnivore	2	3370 (0.023)	2293 (0.034)	2492 (0.033)
carnivory	2	3370 (0.023)	#SAKNAS!	2492 (0.033)
cartilage	2	3370 (0.023)	#SAKNAS!	1612 (0.066)
case-matched	2	3370 (0.023)	2293 (0.034)	2492 (0.033)
case-series	2	3370 (0.023)	#SAKNAS!	#SAKNAS!
castaneum	2	3370 (0.023)	#SAKNAS!	1612 (0.066)

cat	2	3370 (0.023)	1457 (0.069)	#SAKNAS!
cataract	2	3370 (0.023)	1457 (0.069)	#SAKNAS!
catfish	2	3370 (0.023)	2293 (0.034)	2492 (0.033)
catheter	2	3370 (0.023)	1457 (0.069)	#SAKNAS!
catla	2	3370 (0.023)	2293 (0.034)	2492 (0.033)
catta	2	3370 (0.023)	1457 (0.069)	#SAKNAS!
cavia	2	3370 (0.023)	#SAKNAS!	2492 (0.033)
cellular	2	3370 (0.023)	2293 (0.034)	2492 (0.033)
cellulose	2	3370 (0.023)	2293 (0.034)	#SAKNAS!
centres	2	3370 (0.023)	1457 (0.069)	#SAKNAS!
cerastoderma	2	3370 (0.023)	1457 (0.069)	#SAKNAS!
ceratitis	2	3370 (0.023)	1457 (0.069)	#SAKNAS!
cerebellum	2	3370 (0.023)	2293 (0.034)	#SAKNAS!
cerebral-palsy	2	3370 (0.023)	1457 (0.069)	#SAKNAS!
cerevisiae	2	3370 (0.023)	#SAKNAS!	2492 (0.033)
certain	2	3370 (0.023)	#SAKNAS!	2492 (0.033)
chaetodon	2	3370 (0.023)	2293 (0.034)	2492 (0.033)
chagasic	2	3370 (0.023)	2293 (0.034)	2492 (0.033)
chairside	2	3370 (0.023)	2293 (0.034)	2492 (0.033)
chance	2	3370 (0.023)	2293 (0.034)	#SAKNAS!
characterisation	2	3370 (0.023)	#SAKNAS!	2492 (0.033)
characteristic	2	3370 (0.023)	2293 (0.034)	2492 (0.033)
charolais	2	3370 (0.023)	1457 (0.069)	#SAKNAS!
chd	2	3370 (0.023)	2293 (0.034)	2492 (0.033)
cheap	2	3370 (0.023)	2293 (0.034)	#SAKNAS!
checkpoint	2	3370 (0.023)	#SAKNAS!	2492 (0.033)
chemerin	2	3370 (0.023)	#SAKNAS!	1612 (0.066)
chemical-composition	2	3370 (0.023)	1457 (0.069)	#SAKNAS!
chemically	2	3370 (0.023)	2293 (0.034)	2492 (0.033)
chemo-radiotherapy	2	3370 (0.023)	#SAKNAS!	2492 (0.033)
chicks	2	3370 (0.023)	1457 (0.069)	#SAKNAS!
childbirth	2	3370 (0.023)	#SAKNAS!	1612 (0.066)
chilonis	2	3370 (0.023)	#SAKNAS!	1612 (0.066)
chimpanzee	2	3370 (0.023)	2293 (0.034)	2492 (0.033)
chinese-american	2	3370 (0.023)	2293 (0.034)	#SAKNAS!
chlorhexidine	2	3370 (0.023)	1457 (0.069)	#SAKNAS!
chloride	2	3370 (0.023)	2293 (0.034)	#SAKNAS!
chrysomelas	2	3370 (0.023)	#SAKNAS!	2492 (0.033)
chrysophyte	2	3370 (0.023)	2293 (0.034)	#SAKNAS!
circulation	2	3370 (0.023)	#SAKNAS!	2492 (0.033)
cisplatin	2	3370 (0.023)	2293 (0.034)	2492 (0.033)
citri	2	3370 (0.023)	#SAKNAS!	2492 (0.033)
claim	2	3370 (0.023)	#SAKNAS!	1612 (0.066)

clams	2	3370 (0.023)	2293 (0.034)	#SAKNAS!
clavata	2	3370 (0.023)	#SAKNAS!	2492 (0.033)
clefts	2	3370 (0.023)	2293 (0.034)	#SAKNAS!
cleveland	2	3370 (0.023)	2293 (0.034)	2492 (0.033)
climate	2	3370 (0.023)	#SAKNAS!	2492 (0.033)
clinician	2	3370 (0.023)	#SAKNAS!	#SAKNAS!
clusters	2	3370 (0.023)	#SAKNAS!	#SAKNAS!
cmi	2	3370 (0.023)	#SAKNAS!	#SAKNAS!
cncm	2	3370 (0.023)	#SAKNAS!	2492 (0.033)
coccinellidae	2	3370 (0.023)	2293 (0.034)	2492 (0.033)
cockroach	2	3370 (0.023)	2293 (0.034)	#SAKNAS!
coconut	2	3370 (0.023)	#SAKNAS!	2492 (0.033)
coincidence	2	3370 (0.023)	1457 (0.069)	#SAKNAS!
coleomegilla	2	3370 (0.023)	2293 (0.034)	2492 (0.033)
collis	2	3370 (0.023)	#SAKNAS!	1612 (0.066)
colombian	2	3370 (0.023)	#SAKNAS!	#SAKNAS!
colorado	2	3370 (0.023)	2293 (0.034)	2492 (0.033)
color-changeable	2	3370 (0.023)	#SAKNAS!	2492 (0.033)
colossoma	2	3370 (0.023)	#SAKNAS!	1612 (0.066)
com-b	2	3370 (0.023)	#SAKNAS!	#SAKNAS!
combinations	2	3370 (0.023)	#SAKNAS!	#SAKNAS!
come	2	3370 (0.023)	2293 (0.034)	2492 (0.033)
communicating	2	3370 (0.023)	#SAKNAS!	2492 (0.033)
community-level	2	3370 (0.023)	#SAKNAS!	2492 (0.033)
companion	2	3370 (0.023)	2293 (0.034)	2492 (0.033)
comparable	2	3370 (0.023)	#SAKNAS!	2492 (0.033)
compare	2	3370 (0.023)	#SAKNAS!	2492 (0.033)
compensation	2	3370 (0.023)	1457 (0.069)	#SAKNAS!
competing	2	3370 (0.023)	#SAKNAS!	2492 (0.033)
completion	2	3370 (0.023)	2293 (0.034)	2492 (0.033)
compositions	2	3370 (0.023)	2293 (0.034)	#SAKNAS!
computational	2	3370 (0.023)	2293 (0.034)	#SAKNAS!
computer	2	3370 (0.023)	#SAKNAS!	1612 (0.066)
concepts	2	3370 (0.023)	2293 (0.034)	#SAKNAS!
concord	2	3370 (0.023)	#SAKNAS!	#SAKNAS!
conditional	2	3370 (0.023)	#SAKNAS!	2492 (0.033)
conditioning	2	3370 (0.023)	2293 (0.034)	2492 (0.033)
confectionery	2	3370 (0.023)	2293 (0.034)	2492 (0.033)
conference	2	3370 (0.023)	#SAKNAS!	1612 (0.066)
confirmatory	2	3370 (0.023)	2293 (0.034)	#SAKNAS!
congestive	2	3370 (0.023)	2293 (0.034)	2492 (0.033)
conservative	2	3370 (0.023)	#SAKNAS!	2492 (0.033)
conspecific	2	3370 (0.023)	2293 (0.034)	2492 (0.033)

constituents	2	3370 (0.023)	2293 (0.034)	2492 (0.033)
constrictor	2	3370 (0.023)	#SAKNAS!	#SAKNAS!
consumers	2	3370 (0.023)	2293 (0.034)	2492 (0.033)
contaminant	2	3370 (0.023)	2293 (0.034)	2492 (0.033)
contaminants	2	3370 (0.023)	2293 (0.034)	2492 (0.033)
context-dependent	2	3370 (0.023)	#SAKNAS!	#SAKNAS!
continuing	2	3370 (0.023)	1457 (0.069)	#SAKNAS!
continuously	2	3370 (0.023)	2293 (0.034)	#SAKNAS!
contraindication	2	3370 (0.023)	2293 (0.034)	2492 (0.033)
contributes	2	3370 (0.023)	2293 (0.034)	#SAKNAS!
co-occurrence	2	3370 (0.023)	#SAKNAS!	#SAKNAS!
cooking	2	3370 (0.023)	#SAKNAS!	2492 (0.033)
cooperation	2	3370 (0.023)	2293 (0.034)	2492 (0.033)
coordination	2	3370 (0.023)	#SAKNAS!	1612 (0.066)
copper	2	3370 (0.023)	#SAKNAS!	1612 (0.066)
coprophagy	2	3370 (0.023)	2293 (0.034)	2492 (0.033)
cordatus	2	3370 (0.023)	#SAKNAS!	2492 (0.033)
core	2	3370 (0.023)	#SAKNAS!	2492 (0.033)
coronavirus	2	3370 (0.023)	#SAKNAS!	#SAKNAS!
correction	2	3370 (0.023)	2293 (0.034)	2492 (0.033)
cotinine	2	3370 (0.023)	#SAKNAS!	2492 (0.033)
coturnix	2	3370 (0.023)	1457 (0.069)	#SAKNAS!
counterparts	2	3370 (0.023)	2293 (0.034)	2492 (0.033)
cow	2	3370 (0.023)	1457 (0.069)	#SAKNAS!
cpd	2	3370 (0.023)	#SAKNAS!	1612 (0.066)
crabs	2	3370 (0.023)	1457 (0.069)	#SAKNAS!
cranial	2	3370 (0.023)	2293 (0.034)	2492 (0.033)
crickets	2	3370 (0.023)	#SAKNAS!	2492 (0.033)
cricopharyngeus	2	3370 (0.023)	#SAKNAS!	#SAKNAS!
cristatus	2	3370 (0.023)	2293 (0.034)	#SAKNAS!
crop	2	3370 (0.023)	2293 (0.034)	2492 (0.033)
crowns	2	3370 (0.023)	#SAKNAS!	#SAKNAS!
crucial	2	3370 (0.023)	#SAKNAS!	1612 (0.066)
crural	2	3370 (0.023)	1457 (0.069)	#SAKNAS!
cruroplasty	2	3370 (0.023)	2293 (0.034)	#SAKNAS!
crustacea	2	3370 (0.023)	1457 (0.069)	#SAKNAS!
ctenomys	2	3370 (0.023)	2293 (0.034)	2492 (0.033)
cue	2	3370 (0.023)	2293 (0.034)	2492 (0.033)
culicidae	2	3370 (0.023)	2293 (0.034)	2492 (0.033)
culinary	2	3370 (0.023)	#SAKNAS!	2492 (0.033)
cultivars	2	3370 (0.023)	2293 (0.034)	#SAKNAS!
curricula	2	3370 (0.023)	2293 (0.034)	#SAKNAS!
cynodon	2	3370 (0.023)	#SAKNAS!	2492 (0.033)

czech	2	3370 (0.023)	2293 (0.034)	2492 (0.033)
dactylon	2	3370 (0.023)	#SAKNAS!	2492 (0.033)
dalmatian	2	3370 (0.023)	#SAKNAS!	#SAKNAS!
danes	2	3370 (0.023)	2293 (0.034)	2492 (0.033)
danger	2	3370 (0.023)	#SAKNAS!	2492 (0.033)
databases	2	3370 (0.023)	1457 (0.069)	#SAKNAS!
dataset	2	3370 (0.023)	#SAKNAS!	#SAKNAS!
daycare	2	3370 (0.023)	2293 (0.034)	#SAKNAS!
day-case	2	3370 (0.023)	1457 (0.069)	#SAKNAS!
daytime	2	3370 (0.023)	#SAKNAS!	2492 (0.033)
decide	2	3370 (0.023)	1457 (0.069)	#SAKNAS!
deciphering	2	3370 (0.023)	2293 (0.034)	#SAKNAS!
decomposition	2	3370 (0.023)	#SAKNAS!	#SAKNAS!
decorated	2	3370 (0.023)	#SAKNAS!	#SAKNAS!
deep-sea	2	3370 (0.023)	2293 (0.034)	2492 (0.033)
defences	2	3370 (0.023)	2293 (0.034)	2492 (0.033)
deficient	2	3370 (0.023)	#SAKNAS!	#SAKNAS!
define	2	3370 (0.023)	#SAKNAS!	#SAKNAS!
definite	2	3370 (0.023)	1457 (0.069)	#SAKNAS!
deglutition	2	3370 (0.023)	#SAKNAS!	2492 (0.033)
dehydrogenase	2	3370 (0.023)	#SAKNAS!	1612 (0.066)
delphi	2	3370 (0.023)	#SAKNAS!	2492 (0.033)
demand	2	3370 (0.023)	#SAKNAS!	1612 (0.066)
demand-feeding	2	3370 (0.023)	2293 (0.034)	2492 (0.033)
democratic	2	3370 (0.023)	#SAKNAS!	2492 (0.033)
demonstrate	2	3370 (0.023)	#SAKNAS!	2492 (0.033)
denticola	2	3370 (0.023)	2293 (0.034)	2492 (0.033)
deoxynivalenol	2	3370 (0.023)	2293 (0.034)	2492 (0.033)
departments	2	3370 (0.023)	#SAKNAS!	2492 (0.033)
depend	2	3370 (0.023)	2293 (0.034)	2492 (0.033)
depending	2	3370 (0.023)	#SAKNAS!	2492 (0.033)
depends	2	3370 (0.023)	1457 (0.069)	#SAKNAS!
deposit	2	3370 (0.023)	2293 (0.034)	2492 (0.033)
dermal	2	3370 (0.023)	2293 (0.034)	2492 (0.033)
dermatitis	2	3370 (0.023)	#SAKNAS!	2492 (0.033)
dermatomyositis	2	3370 (0.023)	1457 (0.069)	#SAKNAS!
desalivated	2	3370 (0.023)	1457 (0.069)	#SAKNAS!
desire	2	3370 (0.023)	#SAKNAS!	2492 (0.033)
determinant	2	3370 (0.023)	2293 (0.034)	2492 (0.033)
determines	2	3370 (0.023)	1457 (0.069)	#SAKNAS!
deterrent	2	3370 (0.023)	#SAKNAS!	1612 (0.066)
developments	2	3370 (0.023)	2293 (0.034)	#SAKNAS!
deviance	2	3370 (0.023)	#SAKNAS!	2492 (0.033)

d-fructose	2	3370 (0.023)	#SAKNAS!	1612 (0.066)
dha	2	3370 (0.023)	#SAKNAS!	1612 (0.066)
dhaka	2	3370 (0.023)	#SAKNAS!	2492 (0.033)
di	2	3370 (0.023)	#SAKNAS!	1612 (0.066)
diaphragmatic	2	3370 (0.023)	2293 (0.034)	#SAKNAS!
diaries	2	3370 (0.023)	#SAKNAS!	1612 (0.066)
diatom	2	3370 (0.023)	1457 (0.069)	#SAKNAS!
diatoms	2	3370 (0.023)	1457 (0.069)	#SAKNAS!
diego	2	3370 (0.023)	#SAKNAS!	1612 (0.066)
diel	2	3370 (0.023)	1457 (0.069)	#SAKNAS!
differentially	2	3370 (0.023)	2293 (0.034)	2492 (0.033)
differently	2	3370 (0.023)	2293 (0.034)	2492 (0.033)
digeorge	2	3370 (0.023)	#SAKNAS!	1612 (0.066)
dimorphism	2	3370 (0.023)	2293 (0.034)	2492 (0.033)
dinner	2	3370 (0.023)	2293 (0.034)	2492 (0.033)
dinoflagellate	2	3370 (0.023)	2293 (0.034)	2492 (0.033)
diomedea	2	3370 (0.023)	1457 (0.069)	#SAKNAS!
dioxide	2	3370 (0.023)	#SAKNAS!	1612 (0.066)
diplodus	2	3370 (0.023)	1457 (0.069)	#SAKNAS!
direction	2	3370 (0.023)	2293 (0.034)	2492 (0.033)
directly	2	3370 (0.023)	#SAKNAS!	1612 (0.066)
disadvantage	2	3370 (0.023)	2293 (0.034)	#SAKNAS!
discordant	2	3370 (0.023)	1457 (0.069)	#SAKNAS!
discriminant	2	3370 (0.023)	#SAKNAS!	1612 (0.066)
discriminate	2	3370 (0.023)	#SAKNAS!	2492 (0.033)
discussion	2	3370 (0.023)	1457 (0.069)	#SAKNAS!
displacement	2	3370 (0.023)	#SAKNAS!	1612 (0.066)
displacements	2	3370 (0.023)	2293 (0.034)	2492 (0.033)
distance	2	3370 (0.023)	1457 (0.069)	#SAKNAS!
divergent	2	3370 (0.023)	#SAKNAS!	2492 (0.033)
division	2	3370 (0.023)	1457 (0.069)	#SAKNAS!
dna	2	3370 (0.023)	2293 (0.034)	2492 (0.033)
docosahexaenoic	2	3370 (0.023)	1457 (0.069)	#SAKNAS!
dome	2	3370 (0.023)	#SAKNAS!	#SAKNAS!
dominant	2	3370 (0.023)	1457 (0.069)	#SAKNAS!
dose-response	2	3370 (0.023)	#SAKNAS!	#SAKNAS!
dreissena	2	3370 (0.023)	2293 (0.034)	2492 (0.033)
drift-feeding	2	3370 (0.023)	#SAKNAS!	1612 (0.066)
driven	2	3370 (0.023)	#SAKNAS!	2492 (0.033)
drooling	2	3370 (0.023)	2293 (0.034)	2492 (0.033)
drosophilidae	2	3370 (0.023)	#SAKNAS!	1612 (0.066)
dual-energy	2	3370 (0.023)	2293 (0.034)	#SAKNAS!
dung	2	3370 (0.023)	#SAKNAS!	#SAKNAS!

duodenal	2	3370 (0.023)	2293 (0.034)	2492 (0.033)
duty	2	3370 (0.023)	#SAKNAS!	#SAKNAS!
dysarthria	2	3370 (0.023)	#SAKNAS!	1612 (0.066)
dysbiosis	2	3370 (0.023)	#SAKNAS!	#SAKNAS!
dyskinesia	2	3370 (0.023)	2293 (0.034)	2492 (0.033)
dysphagia-related	2	3370 (0.023)	#SAKNAS!	1612 (0.066)
dysphonia	2	3370 (0.023)	#SAKNAS!	1612 (0.066)
dysplasia	2	3370 (0.023)	2293 (0.034)	2492 (0.033)
dystonia	2	3370 (0.023)	2293 (0.034)	#SAKNAS!
dytiscidae	2	3370 (0.023)	2293 (0.034)	2492 (0.033)
dytiscus	2	3370 (0.023)	2293 (0.034)	2492 (0.033)
e2	2	3370 (0.023)	2293 (0.034)	#SAKNAS!
earlier	2	3370 (0.023)	2293 (0.034)	2492 (0.033)
earliest	2	3370 (0.023)	#SAKNAS!	1612 (0.066)
early-life	2	3370 (0.023)	2293 (0.034)	#SAKNAS!
early-phase	2	3370 (0.023)	#SAKNAS!	#SAKNAS!
ec	2	3370 (0.023)	#SAKNAS!	1612 (0.066)
echinodermata	2	3370 (0.023)	1457 (0.069)	#SAKNAS!
ecosystems	2	3370 (0.023)	1457 (0.069)	#SAKNAS!
ecuador	2	3370 (0.023)	#SAKNAS!	2492 (0.033)
ecuadorian	2	3370 (0.023)	#SAKNAS!	2492 (0.033)
e-delphi	2	3370 (0.023)	#SAKNAS!	#SAKNAS!
edema	2	3370 (0.023)	2293 (0.034)	#SAKNAS!
edinburgh	2	3370 (0.023)	2293 (0.034)	#SAKNAS!
edule	2	3370 (0.023)	1457 (0.069)	#SAKNAS!
edulis	2	3370 (0.023)	1457 (0.069)	#SAKNAS!
egypt	2	3370 (0.023)	#SAKNAS!	#SAKNAS!
elastase	2	3370 (0.023)	2293 (0.034)	#SAKNAS!
elder	2	3370 (0.023)	2293 (0.034)	#SAKNAS!
elder-friendly	2	3370 (0.023)	#SAKNAS!	#SAKNAS!
electroencephalography	2	3370 (0.023)	#SAKNAS!	#SAKNAS!
eligible	2	3370 (0.023)	#SAKNAS!	#SAKNAS!
eliminating	2	3370 (0.023)	1457 (0.069)	#SAKNAS!
elimination	2	3370 (0.023)	2293 (0.034)	#SAKNAS!
emerald	2	3370 (0.023)	1457 (0.069)	#SAKNAS!
emphasis	2	3370 (0.023)	1457 (0.069)	#SAKNAS!
empirical	2	3370 (0.023)	#SAKNAS!	#SAKNAS!
endocrinological	2	3370 (0.023)	#SAKNAS!	#SAKNAS!
end-of-life	2	3370 (0.023)	2293 (0.034)	2492 (0.033)
endogeic	2	3370 (0.023)	#SAKNAS!	1612 (0.066)
endogenous	2	3370 (0.023)	1457 (0.069)	#SAKNAS!
endoluminal	2	3370 (0.023)	1457 (0.069)	#SAKNAS!
endoparasitoid	2	3370 (0.023)	2293 (0.034)	2492 (0.033)

endoscopically	2	3370 (0.023)	1457 (0.069)	#SAKNAS!
endurance	2	3370 (0.023)	#SAKNAS!	1612 (0.066)
engaging	2	3370 (0.023)	2293 (0.034)	2492 (0.033)
enrichment	2	3370 (0.023)	#SAKNAS!	1612 (0.066)
ensure	2	3370 (0.023)	#SAKNAS!	1612 (0.066)
enterica	2	3370 (0.023)	2293 (0.034)	#SAKNAS!
environmentally	2	3370 (0.023)	#SAKNAS!	#SAKNAS!
enzymes	2	3370 (0.023)	#SAKNAS!	1612 (0.066)
eoe	2	3370 (0.023)	#SAKNAS!	#SAKNAS!
eortc	2	3370 (0.023)	2293 (0.034)	2492 (0.033)
epidemic	2	3370 (0.023)	#SAKNAS!	#SAKNAS!
epidermal	2	3370 (0.023)	2293 (0.034)	#SAKNAS!
epidermolysis-bullosa	2	3370 (0.023)	1457 (0.069)	#SAKNAS!
epigenetics	2	3370 (0.023)	#SAKNAS!	#SAKNAS!
epiphrenic	2	3370 (0.023)	2293 (0.034)	2492 (0.033)
era	2	3370 (0.023)	2293 (0.034)	2492 (0.033)
erectus	2	3370 (0.023)	2293 (0.034)	2492 (0.033)
eriocheir	2	3370 (0.023)	#SAKNAS!	#SAKNAS!
erythrocyte	2	3370 (0.023)	2293 (0.034)	#SAKNAS!
esophagocoloplasty	2	3370 (0.023)	2293 (0.034)	2492 (0.033)
esophago-gastric	2	3370 (0.023)	1457 (0.069)	#SAKNAS!
esrd	2	3370 (0.023)	1457 (0.069)	#SAKNAS!
establishment	2	3370 (0.023)	#SAKNAS!	2492 (0.033)
estradiol	2	3370 (0.023)	2293 (0.034)	#SAKNAS!
estrogen	2	3370 (0.023)	1457 (0.069)	#SAKNAS!
etv5	2	3370 (0.023)	2293 (0.034)	2492 (0.033)
eutrophic	2	3370 (0.023)	2293 (0.034)	#SAKNAS!
event	2	3370 (0.023)	#SAKNAS!	2492 (0.033)
every	2	3370 (0.023)	2293 (0.034)	#SAKNAS!
exacerbations	2	3370 (0.023)	2293 (0.034)	#SAKNAS!
exchange	2	3370 (0.023)	2293 (0.034)	#SAKNAS!
exclusion	2	3370 (0.023)	#SAKNAS!	1612 (0.066)
excreta	2	3370 (0.023)	#SAKNAS!	1612 (0.066)
expenditures	2	3370 (0.023)	2293 (0.034)	2492 (0.033)
expiratory	2	3370 (0.023)	#SAKNAS!	2492 (0.033)
explaining	2	3370 (0.023)	2293 (0.034)	#SAKNAS!
exploitation	2	3370 (0.023)	#SAKNAS!	2492 (0.033)
extension	2	3370 (0.023)	#SAKNAS!	2492 (0.033)
extracellular	2	3370 (0.023)	#SAKNAS!	1612 (0.066)
extraction	2	3370 (0.023)	1457 (0.069)	#SAKNAS!
extreme	2	3370 (0.023)	1457 (0.069)	#SAKNAS!
extubation	2	3370 (0.023)	#SAKNAS!	#SAKNAS!
f1	2	3370 (0.023)	#SAKNAS!	1612 (0.066)

fabricius	2	3370 (0.023)	1457 (0.069)	#SAKNAS!
facilitating	2	3370 (0.023)	1457 (0.069)	#SAKNAS!
faisal	2	3370 (0.023)	#SAKNAS!	2492 (0.033)
fall	2	3370 (0.023)	#SAKNAS!	2492 (0.033)
fallow	2	3370 (0.023)	1457 (0.069)	#SAKNAS!
far	2	3370 (0.023)	#SAKNAS!	2492 (0.033)
farmed	2	3370 (0.023)	1457 (0.069)	#SAKNAS!
farmers	2	3370 (0.023)	#SAKNAS!	2492 (0.033)
faster	2	3370 (0.023)	#SAKNAS!	2492 (0.033)
fathers	2	3370 (0.023)	#SAKNAS!	1612 (0.066)
fatigue	2	3370 (0.023)	#SAKNAS!	#SAKNAS!
fattening	2	3370 (0.023)	1457 (0.069)	#SAKNAS!
favor	2	3370 (0.023)	2293 (0.034)	2492 (0.033)
favorable	2	3370 (0.023)	#SAKNAS!	2492 (0.033)
fear	2	3370 (0.023)	#SAKNAS!	2492 (0.033)
feature	2	3370 (0.023)	#SAKNAS!	2492 (0.033)
feces	2	3370 (0.023)	1457 (0.069)	#SAKNAS!
feedback	2	3370 (0.023)	2293 (0.034)	2492 (0.033)
feedings	2	3370 (0.023)	2293 (0.034)	2492 (0.033)
feeding-tube	2	3370 (0.023)	#SAKNAS!	#SAKNAS!
feline	2	3370 (0.023)	#SAKNAS!	#SAKNAS!
femoralis	2	3370 (0.023)	#SAKNAS!	#SAKNAS!
fenugreek	2	3370 (0.023)	1457 (0.069)	#SAKNAS!
feral	2	3370 (0.023)	1457 (0.069)	#SAKNAS!
fertile	2	3370 (0.023)	#SAKNAS!	1612 (0.066)
fertility	2	3370 (0.023)	#SAKNAS!	1612 (0.066)
fewer	2	3370 (0.023)	#SAKNAS!	1612 (0.066)
fgf21	2	3370 (0.023)	#SAKNAS!	#SAKNAS!
fibrin	2	3370 (0.023)	2293 (0.034)	#SAKNAS!
fibromyalgia	2	3370 (0.023)	#SAKNAS!	#SAKNAS!
filter-feeding	2	3370 (0.023)	1457 (0.069)	#SAKNAS!
fir	2	3370 (0.023)	1457 (0.069)	#SAKNAS!
first-degree	2	3370 (0.023)	#SAKNAS!	1612 (0.066)
first-grade	2	3370 (0.023)	1457 (0.069)	#SAKNAS!
first-time	2	3370 (0.023)	2293 (0.034)	2492 (0.033)
fishes	2	3370 (0.023)	1457 (0.069)	#SAKNAS!
fishmeal	2	3370 (0.023)	2293 (0.034)	#SAKNAS!
fit	2	3370 (0.023)	2293 (0.034)	#SAKNAS!
fits	2	3370 (0.023)	2293 (0.034)	2492 (0.033)
fl	2	3370 (0.023)	#SAKNAS!	#SAKNAS!
flame	2	3370 (0.023)	#SAKNAS!	#SAKNAS!
flora	2	3370 (0.023)	2293 (0.034)	#SAKNAS!
floral	2	3370 (0.023)	1457 (0.069)	#SAKNAS!

flour	2	3370 (0.023)	2293 (0.034)	#SAKNAS!
flowering	2	3370 (0.023)	#SAKNAS!	1612 (0.066)
fluctuations	2	3370 (0.023)	1457 (0.069)	#SAKNAS!
fluoroscopic	2	3370 (0.023)	2293 (0.034)	#SAKNAS!
fluoroscopically	2	3370 (0.023)	1457 (0.069)	#SAKNAS!
fluoroscopy	2	3370 (0.023)	2293 (0.034)	#SAKNAS!
fluviatilis	2	3370 (0.023)	1457 (0.069)	#SAKNAS!
fly	2	3370 (0.023)	#SAKNAS!	#SAKNAS!
focal	2	3370 (0.023)	#SAKNAS!	2492 (0.033)
fodder	2	3370 (0.023)	#SAKNAS!	2492 (0.033)
fold	2	3370 (0.023)	1457 (0.069)	#SAKNAS!
folivore	2	3370 (0.023)	2293 (0.034)	2492 (0.033)
folivorous	2	3370 (0.023)	2293 (0.034)	2492 (0.033)
food-consumption	2	3370 (0.023)	1457 (0.069)	#SAKNAS!
food-deprivation	2	3370 (0.023)	1457 (0.069)	#SAKNAS!
forager	2	3370 (0.023)	2293 (0.034)	2492 (0.033)
forceps	2	3370 (0.023)	#SAKNAS!	#SAKNAS!
forearm	2	3370 (0.023)	1457 (0.069)	#SAKNAS!
foregut	2	3370 (0.023)	#SAKNAS!	1612 (0.066)
foreign	2	3370 (0.023)	2293 (0.034)	2492 (0.033)
forgotten	2	3370 (0.023)	#SAKNAS!	2492 (0.033)
formulation	2	3370 (0.023)	#SAKNAS!	#SAKNAS!
fossil	2	3370 (0.023)	2293 (0.034)	2492 (0.033)
four-year-old	2	3370 (0.023)	1457 (0.069)	#SAKNAS!
france	2	3370 (0.023)	1457 (0.069)	#SAKNAS!
freshmen	2	3370 (0.023)	2293 (0.034)	#SAKNAS!
frontiers	2	3370 (0.023)	#SAKNAS!	#SAKNAS!
fulvus	2	3370 (0.023)	1457 (0.069)	#SAKNAS!
fur	2	3370 (0.023)	1457 (0.069)	#SAKNAS!
futures	2	3370 (0.023)	#SAKNAS!	2492 (0.033)
gains	2	3370 (0.023)	2293 (0.034)	#SAKNAS!
galapagos	2	3370 (0.023)	2293 (0.034)	2492 (0.033)
gall-inducing	2	3370 (0.023)	2293 (0.034)	2492 (0.033)
gaps	2	3370 (0.023)	2293 (0.034)	#SAKNAS!
gastroduodenal	2	3370 (0.023)	2293 (0.034)	2492 (0.033)
gastrooesophageal	2	3370 (0.023)	2293 (0.034)	#SAKNAS!
gastroparesis	2	3370 (0.023)	#SAKNAS!	2492 (0.033)
gastropexy	2	3370 (0.023)	2293 (0.034)	#SAKNAS!
gelatinous	2	3370 (0.023)	2293 (0.034)	2492 (0.033)
gene-environment	2	3370 (0.023)	#SAKNAS!	1612 (0.066)
generates	2	3370 (0.023)	2293 (0.034)	2492 (0.033)
genome-wide	2	3370 (0.023)	#SAKNAS!	2492 (0.033)
geometric	2	3370 (0.023)	#SAKNAS!	2492 (0.033)

geometry	2	3370 (0.023)	1457 (0.069)	#SAKNAS!
gestating	2	3370 (0.023)	#SAKNAS!	#SAKNAS!
ghats	2	3370 (0.023)	#SAKNAS!	#SAKNAS!
gianturco	2	3370 (0.023)	1457 (0.069)	#SAKNAS!
gift	2	3370 (0.023)	#SAKNAS!	2492 (0.033)
gingiva	2	3370 (0.023)	1457 (0.069)	#SAKNAS!
giraffe	2	3370 (0.023)	2293 (0.034)	2492 (0.033)
gl	2	3370 (0.023)	#SAKNAS!	2492 (0.033)
glossina-pallidipes	2	3370 (0.023)	1457 (0.069)	#SAKNAS!
glucagon-like	2	3370 (0.023)	#SAKNAS!	2492 (0.033)
glucomannan	2	3370 (0.023)	#SAKNAS!	#SAKNAS!
glutamatergic	2	3370 (0.023)	2293 (0.034)	2492 (0.033)
glutathione	2	3370 (0.023)	1457 (0.069)	#SAKNAS!
glycol	2	3370 (0.023)	1457 (0.069)	#SAKNAS!
go	2	3370 (0.023)	#SAKNAS!	2492 (0.033)
godart	2	3370 (0.023)	1457 (0.069)	#SAKNAS!
going	2	3370 (0.023)	#SAKNAS!	2492 (0.033)
gothenburg	2	3370 (0.023)	2293 (0.034)	2492 (0.033)
government	2	3370 (0.023)	2293 (0.034)	#SAKNAS!
graft	2	3370 (0.023)	#SAKNAS!	1612 (0.066)
graft-versus-host	2	3370 (0.023)	1457 (0.069)	#SAKNAS!
granulocyte-macrophage	2	3370 (0.023)	1457 (0.069)	#SAKNAS!
graph	2	3370 (0.023)	#SAKNAS!	1612 (0.066)
gregaria	2	3370 (0.023)	2293 (0.034)	2492 (0.033)
greylag	2	3370 (0.023)	1457 (0.069)	#SAKNAS!
grocery	2	3370 (0.023)	1457 (0.069)	#SAKNAS!
grown	2	3370 (0.023)	2293 (0.034)	#SAKNAS!
gugging	2	3370 (0.023)	2293 (0.034)	#SAKNAS!
guillain-barre	2	3370 (0.023)	#SAKNAS!	2492 (0.033)
guilt	2	3370 (0.023)	#SAKNAS!	2492 (0.033)
gullah	2	3370 (0.023)	1457 (0.069)	#SAKNAS!
gum-chewing	2	3370 (0.023)	#SAKNAS!	#SAKNAS!
habitual	2	3370 (0.023)	2293 (0.034)	#SAKNAS!
haematological	2	3370 (0.023)	2293 (0.034)	2492 (0.033)
haemodialysis	2	3370 (0.023)	#SAKNAS!	#SAKNAS!
haitian	2	3370 (0.023)	1457 (0.069)	#SAKNAS!
half	2	3370 (0.023)	1457 (0.069)	#SAKNAS!
haliotis	2	3370 (0.023)	2293 (0.034)	2492 (0.033)
halitosis	2	3370 (0.023)	#SAKNAS!	1612 (0.066)
han	2	3370 (0.023)	#SAKNAS!	1612 (0.066)
hand-oral	2	3370 (0.023)	1457 (0.069)	#SAKNAS!
hard	2	3370 (0.023)	2293 (0.034)	2492 (0.033)
hardness	2	3370 (0.023)	#SAKNAS!	2492 (0.033)

harmful	2	3370 (0.023)	#SAKNAS!	1612 (0.066)
haw	2	3370 (0.023)	#SAKNAS!	1612 (0.066)
heartburn	2	3370 (0.023)	2293 (0.034)	#SAKNAS!
heating	2	3370 (0.023)	#SAKNAS!	#SAKNAS!
helsinki	2	3370 (0.023)	#SAKNAS!	1612 (0.066)
hematoma	2	3370 (0.023)	2293 (0.034)	#SAKNAS!
hemolymph	2	3370 (0.023)	2293 (0.034)	2492 (0.033)
herbal	2	3370 (0.023)	#SAKNAS!	1612 (0.066)
hermetia	2	3370 (0.023)	#SAKNAS!	#SAKNAS!
herpes	2	3370 (0.023)	2293 (0.034)	#SAKNAS!
hexa	2	3370 (0.023)	#SAKNAS!	1612 (0.066)
high-energy	2	3370 (0.023)	#SAKNAS!	1612 (0.066)
high-fibre	2	3370 (0.023)	1457 (0.069)	#SAKNAS!
highlights	2	3370 (0.023)	2293 (0.034)	#SAKNAS!
high-pressure	2	3370 (0.023)	#SAKNAS!	#SAKNAS!
hinds	2	3370 (0.023)	1457 (0.069)	#SAKNAS!
hircus	2	3370 (0.023)	1457 (0.069)	#SAKNAS!
hisayama	2	3370 (0.023)	2293 (0.034)	2492 (0.033)
hispanics	2	3370 (0.023)	2293 (0.034)	2492 (0.033)
histology	2	3370 (0.023)	2293 (0.034)	2492 (0.033)
historical	2	3370 (0.023)	2293 (0.034)	2492 (0.033)
holstein-friesian	2	3370 (0.023)	#SAKNAS!	2492 (0.033)
homebound	2	3370 (0.023)	2293 (0.034)	2492 (0.033)
home-care	2	3370 (0.023)	#SAKNAS!	1612 (0.066)
hominin	2	3370 (0.023)	#SAKNAS!	2492 (0.033)
homoptera	2	3370 (0.023)	1457 (0.069)	#SAKNAS!
honeydew	2	3370 (0.023)	1457 (0.069)	#SAKNAS!
horizontal	2	3370 (0.023)	#SAKNAS!	2492 (0.033)
hornworm	2	3370 (0.023)	2293 (0.034)	2492 (0.033)
hospital-associated	2	3370 (0.023)	#SAKNAS!	1612 (0.066)
host-feeding	2	3370 (0.023)	1457 (0.069)	#SAKNAS!
hotspots	2	3370 (0.023)	#SAKNAS!	#SAKNAS!
howler	2	3370 (0.023)	#SAKNAS!	1612 (0.066)
huemul	2	3370 (0.023)	1457 (0.069)	#SAKNAS!
humidity	2	3370 (0.023)	2293 (0.034)	#SAKNAS!
hunting	2	3370 (0.023)	2293 (0.034)	#SAKNAS!
hydrolysates	2	3370 (0.023)	#SAKNAS!	1612 (0.066)
hyperborea	2	3370 (0.023)	1457 (0.069)	#SAKNAS!
hypercaloric	2	3370 (0.023)	2293 (0.034)	#SAKNAS!
hyperparathyroidism	2	3370 (0.023)	1457 (0.069)	#SAKNAS!
hypertriglyceridemia	2	3370 (0.023)	2293 (0.034)	#SAKNAS!
hypoglossal	2	3370 (0.023)	#SAKNAS!	2492 (0.033)
hypophosphatemia	2	3370 (0.023)	1457 (0.069)	#SAKNAS!

hypothetical	2	3370 (0.023)	#SAKNAS!	2492 (0.033)
iatrogenic	2	3370 (0.023)	2293 (0.034)	2492 (0.033)
icdas	2	3370 (0.023)	#SAKNAS!	1612 (0.066)
identifies	2	3370 (0.023)	#SAKNAS!	2492 (0.033)
identities	2	3370 (0.023)	#SAKNAS!	1612 (0.066)
igg	2	3370 (0.023)	2293 (0.034)	2492 (0.033)
ilembula	2	3370 (0.023)	#SAKNAS!	#SAKNAS!
illucens	2	3370 (0.023)	#SAKNAS!	#SAKNAS!
immigration	2	3370 (0.023)	#SAKNAS!	2492 (0.033)
impacting	2	3370 (0.023)	#SAKNAS!	2492 (0.033)
impairs	2	3370 (0.023)	#SAKNAS!	1612 (0.066)
implant-based	2	3370 (0.023)	#SAKNAS!	2492 (0.033)
imprinting	2	3370 (0.023)	2293 (0.034)	#SAKNAS!
impulsivity	2	3370 (0.023)	#SAKNAS!	#SAKNAS!
inadequate	2	3370 (0.023)	2293 (0.034)	2492 (0.033)
indirectly	2	3370 (0.023)	#SAKNAS!	1612 (0.066)
ineffective	2	3370 (0.023)	#SAKNAS!	2492 (0.033)
infectious	2	3370 (0.023)	#SAKNAS!	#SAKNAS!
infestation	2	3370 (0.023)	#SAKNAS!	2492 (0.033)
influential	2	3370 (0.023)	#SAKNAS!	2492 (0.033)
informal	2	3370 (0.023)	#SAKNAS!	#SAKNAS!
informing	2	3370 (0.023)	#SAKNAS!	1612 (0.066)
inhibit	2	3370 (0.023)	2293 (0.034)	#SAKNAS!
inhibitory	2	3370 (0.023)	1457 (0.069)	#SAKNAS!
injections	2	3370 (0.023)	2293 (0.034)	2492 (0.033)
inks	2	3370 (0.023)	#SAKNAS!	#SAKNAS!
innovation	2	3370 (0.023)	#SAKNAS!	2492 (0.033)
insecticidal	2	3370 (0.023)	2293 (0.034)	2492 (0.033)
insensitivity	2	3370 (0.023)	1457 (0.069)	#SAKNAS!
institutional	2	3370 (0.023)	2293 (0.034)	2492 (0.033)
instructions	2	3370 (0.023)	#SAKNAS!	2492 (0.033)
intact	2	3370 (0.023)	2293 (0.034)	#SAKNAS!
intelligence-based	2	3370 (0.023)	#SAKNAS!	#SAKNAS!
intensification	2	3370 (0.023)	1457 (0.069)	#SAKNAS!
interact	2	3370 (0.023)	#SAKNAS!	2492 (0.033)
interannual	2	3370 (0.023)	2293 (0.034)	#SAKNAS!
interest	2	3370 (0.023)	2293 (0.034)	2492 (0.033)
interferential	2	3370 (0.023)	#SAKNAS!	1612 (0.066)
internet	2	3370 (0.023)	2293 (0.034)	2492 (0.033)
interpretation	2	3370 (0.023)	2293 (0.034)	#SAKNAS!
interrelation	2	3370 (0.023)	2293 (0.034)	2492 (0.033)
interval	2	3370 (0.023)	2293 (0.034)	#SAKNAS!
interviewing	2	3370 (0.023)	2293 (0.034)	#SAKNAS!

interviews	2	3370 (0.023)	#SAKNAS!	2492 (0.033)
intra-arterial	2	3370 (0.023)	2293 (0.034)	2492 (0.033)
intracerebroventricular	2	3370 (0.023)	2293 (0.034)	2492 (0.033)
intracranial	2	3370 (0.023)	2293 (0.034)	#SAKNAS!
intractable	2	3370 (0.023)	2293 (0.034)	2492 (0.033)
intrasphincteric	2	3370 (0.023)	2293 (0.034)	2492 (0.033)
intrathoracic	2	3370 (0.023)	#SAKNAS!	1612 (0.066)
intra-thoracic	2	3370 (0.023)	2293 (0.034)	2492 (0.033)
intravenous	2	3370 (0.023)	#SAKNAS!	#SAKNAS!
introduce	2	3370 (0.023)	#SAKNAS!	#SAKNAS!
invertebrate	2	3370 (0.023)	2293 (0.034)	2492 (0.033)
investigations	2	3370 (0.023)	#SAKNAS!	#SAKNAS!
invicta	2	3370 (0.023)	1457 (0.069)	#SAKNAS!
ionic	2	3370 (0.023)	#SAKNAS!	2492 (0.033)
irish	2	3370 (0.023)	2293 (0.034)	2492 (0.033)
irisin	2	3370 (0.023)	#SAKNAS!	2492 (0.033)
isolates	2	3370 (0.023)	#SAKNAS!	2492 (0.033)
isolation	2	3370 (0.023)	#SAKNAS!	2492 (0.033)
israeli	2	3370 (0.023)	2293 (0.034)	#SAKNAS!
ivb	2	3370 (0.023)	2293 (0.034)	2492 (0.033)
jages	2	3370 (0.023)	#SAKNAS!	1612 (0.066)
jejunum	2	3370 (0.023)	2293 (0.034)	2492 (0.033)
jelly	2	3370 (0.023)	#SAKNAS!	#SAKNAS!
juices	2	3370 (0.023)	2293 (0.034)	#SAKNAS!
junk	2	3370 (0.023)	2293 (0.034)	2492 (0.033)
just	2	3370 (0.023)	#SAKNAS!	2492 (0.033)
karnataka	2	3370 (0.023)	2293 (0.034)	#SAKNAS!
kazakhstan	2	3370 (0.023)	#SAKNAS!	2492 (0.033)
kid	2	3370 (0.023)	2293 (0.034)	2492 (0.033)
killifish	2	3370 (0.023)	#SAKNAS!	2492 (0.033)
kinematic	2	3370 (0.023)	#SAKNAS!	2492 (0.033)
kinematics	2	3370 (0.023)	#SAKNAS!	#SAKNAS!
king	2	3370 (0.023)	2293 (0.034)	#SAKNAS!
know	2	3370 (0.023)	#SAKNAS!	2492 (0.033)
kuchi-kara	2	3370 (0.023)	#SAKNAS!	1612 (0.066)
kwazulu-natal	2	3370 (0.023)	2293 (0.034)	2492 (0.033)
labeling	2	3370 (0.023)	#SAKNAS!	#SAKNAS!
labelling	2	3370 (0.023)	#SAKNAS!	1612 (0.066)
labels	2	3370 (0.023)	#SAKNAS!	1612 (0.066)
labial	2	3370 (0.023)	#SAKNAS!	2492 (0.033)
lacertid	2	3370 (0.023)	1457 (0.069)	#SAKNAS!
lactobacilli	2	3370 (0.023)	1457 (0.069)	#SAKNAS!
laevis	2	3370 (0.023)	2293 (0.034)	#SAKNAS!

lakes	2	3370 (0.023)	2293 (0.034)	2492 (0.033)
lam	2	3370 (0.023)	2293 (0.034)	2492 (0.033)
lao	2	3370 (0.023)	#SAKNAS!	1612 (0.066)
l-arginine	2	3370 (0.023)	2293 (0.034)	2492 (0.033)
laryngopharyngeal	2	3370 (0.023)	#SAKNAS!	#SAKNAS!
latinx	2	3370 (0.023)	#SAKNAS!	#SAKNAS!
leaflets	2	3370 (0.023)	2293 (0.034)	2492 (0.033)
leaks	2	3370 (0.023)	2293 (0.034)	#SAKNAS!
learning-based	2	3370 (0.023)	#SAKNAS!	#SAKNAS!
least	2	3370 (0.023)	2293 (0.034)	2492 (0.033)
lebanon	2	3370 (0.023)	#SAKNAS!	2492 (0.033)
legal	2	3370 (0.023)	#SAKNAS!	2492 (0.033)
leisure-time	2	3370 (0.023)	#SAKNAS!	#SAKNAS!
leontopithecus	2	3370 (0.023)	#SAKNAS!	2492 (0.033)
lessons	2	3370 (0.023)	#SAKNAS!	1612 (0.066)
libitum-fed	2	3370 (0.023)	#SAKNAS!	#SAKNAS!
libyan	2	3370 (0.023)	2293 (0.034)	#SAKNAS!
licury	2	3370 (0.023)	#SAKNAS!	#SAKNAS!
life-course	2	3370 (0.023)	1457 (0.069)	#SAKNAS!
life-history	2	3370 (0.023)	2293 (0.034)	2492 (0.033)
life-threatening	2	3370 (0.023)	1457 (0.069)	#SAKNAS!
lifting	2	3370 (0.023)	2293 (0.034)	2492 (0.033)
lightweight	2	3370 (0.023)	1457 (0.069)	#SAKNAS!
liking	2	3370 (0.023)	#SAKNAS!	1612 (0.066)
limestone	2	3370 (0.023)	#SAKNAS!	1612 (0.066)
limitations	2	3370 (0.023)	#SAKNAS!	#SAKNAS!
limiting	2	3370 (0.023)	2293 (0.034)	2492 (0.033)
limits	2	3370 (0.023)	2293 (0.034)	2492 (0.033)
limnephilidae	2	3370 (0.023)	2293 (0.034)	#SAKNAS!
limousin	2	3370 (0.023)	1457 (0.069)	#SAKNAS!
line	2	3370 (0.023)	2293 (0.034)	#SAKNAS!
lines	2	3370 (0.023)	1457 (0.069)	#SAKNAS!
linkage	2	3370 (0.023)	2293 (0.034)	#SAKNAS!
linnaeus	2	3370 (0.023)	#SAKNAS!	#SAKNAS!
lion	2	3370 (0.023)	#SAKNAS!	2492 (0.033)
lipolysis	2	3370 (0.023)	1457 (0.069)	#SAKNAS!
locus	2	3370 (0.023)	2293 (0.034)	#SAKNAS!
logistic	2	3370 (0.023)	2293 (0.034)	2492 (0.033)
longer	2	3370 (0.023)	#SAKNAS!	1612 (0.066)
long-gap	2	3370 (0.023)	#SAKNAS!	2492 (0.033)
longitudinal-study	2	3370 (0.023)	1457 (0.069)	#SAKNAS!
long-tailed	2	3370 (0.023)	#SAKNAS!	2492 (0.033)
looped	2	3370 (0.023)	1457 (0.069)	#SAKNAS!

loose-housed	2	3370 (0.023)	1457 (0.069)	#SAKNAS!
lost	2	3370 (0.023)	#SAKNAS!	#SAKNAS!
low-cost	2	3370 (0.023)	2293 (0.034)	2492 (0.033)
low-density	2	3370 (0.023)	2293 (0.034)	#SAKNAS!
lowland	2	3370 (0.023)	2293 (0.034)	2492 (0.033)
low-levels	2	3370 (0.023)	1457 (0.069)	#SAKNAS!
lusoria	2	3370 (0.023)	#SAKNAS!	2492 (0.033)
lymphedema	2	3370 (0.023)	#SAKNAS!	2492 (0.033)
lymphoma	2	3370 (0.023)	1457 (0.069)	#SAKNAS!
macquart	2	3370 (0.023)	1457 (0.069)	#SAKNAS!
macroalgal	2	3370 (0.023)	#SAKNAS!	1612 (0.066)
macrophages	2	3370 (0.023)	#SAKNAS!	2492 (0.033)
macropomum	2	3370 (0.023)	#SAKNAS!	1612 (0.066)
macular	2	3370 (0.023)	#SAKNAS!	1612 (0.066)
maculata	2	3370 (0.023)	2293 (0.034)	2492 (0.033)
madinah	2	3370 (0.023)	#SAKNAS!	1612 (0.066)
magna	2	3370 (0.023)	#SAKNAS!	2492 (0.033)
maintains	2	3370 (0.023)	#SAKNAS!	#SAKNAS!
makes	2	3370 (0.023)	#SAKNAS!	2492 (0.033)
makkah	2	3370 (0.023)	#SAKNAS!	2492 (0.033)
malaria	2	3370 (0.023)	#SAKNAS!	1612 (0.066)
malawi	2	3370 (0.023)	#SAKNAS!	2492 (0.033)
malformations	2	3370 (0.023)	1457 (0.069)	#SAKNAS!
malocclusions	2	3370 (0.023)	2293 (0.034)	2492 (0.033)
mammals	2	3370 (0.023)	2293 (0.034)	2492 (0.033)
man	2	3370 (0.023)	1457 (0.069)	#SAKNAS!
manner	2	3370 (0.023)	2293 (0.034)	2492 (0.033)
mannitol	2	3370 (0.023)	#SAKNAS!	1612 (0.066)
marburg	2	3370 (0.023)	2293 (0.034)	#SAKNAS!
mares	2	3370 (0.023)	2293 (0.034)	#SAKNAS!
marijuana	2	3370 (0.023)	#SAKNAS!	1612 (0.066)
market	2	3370 (0.023)	2293 (0.034)	#SAKNAS!
marketing	2	3370 (0.023)	#SAKNAS!	2492 (0.033)
marmota	2	3370 (0.023)	2293 (0.034)	#SAKNAS!
marrow	2	3370 (0.023)	1457 (0.069)	#SAKNAS!
marsupial	2	3370 (0.023)	2293 (0.034)	#SAKNAS!
masking	2	3370 (0.023)	2293 (0.034)	2492 (0.033)
massive	2	3370 (0.023)	1457 (0.069)	#SAKNAS!
maternal-child	2	3370 (0.023)	2293 (0.034)	#SAKNAS!
maxillectomy	2	3370 (0.023)	#SAKNAS!	2492 (0.033)
maxillofacial	2	3370 (0.023)	2293 (0.034)	#SAKNAS!
maximize	2	3370 (0.023)	#SAKNAS!	2492 (0.033)
mc4r	2	3370 (0.023)	2293 (0.034)	#SAKNAS!

mealworm	2	3370 (0.023)	#SAKNAS!	2492 (0.033)
mechanics	2	3370 (0.023)	#SAKNAS!	1612 (0.066)
mechanistic	2	3370 (0.023)	2293 (0.034)	2492 (0.033)
medial	2	3370 (0.023)	#SAKNAS!	2492 (0.033)
medicaid-eligible	2	3370 (0.023)	1457 (0.069)	#SAKNAS!
medically	2	3370 (0.023)	1457 (0.069)	#SAKNAS!
megaesophagus	2	3370 (0.023)	2293 (0.034)	2492 (0.033)
megalopa	2	3370 (0.023)	#SAKNAS!	1612 (0.066)
mercury	2	3370 (0.023)	1457 (0.069)	#SAKNAS!
message	2	3370 (0.023)	2293 (0.034)	2492 (0.033)
metabarcoding	2	3370 (0.023)	#SAKNAS!	2492 (0.033)
metabolite	2	3370 (0.023)	2293 (0.034)	2492 (0.033)
metabolizable	2	3370 (0.023)	#SAKNAS!	#SAKNAS!
metallic	2	3370 (0.023)	#SAKNAS!	1612 (0.066)
metalloproteinase-9	2	3370 (0.023)	1457 (0.069)	#SAKNAS!
metamorphosis	2	3370 (0.023)	#SAKNAS!	1612 (0.066)
metastatic	2	3370 (0.023)	#SAKNAS!	1612 (0.066)
metformin	2	3370 (0.023)	#SAKNAS!	2492 (0.033)
methadone	2	3370 (0.023)	2293 (0.034)	2492 (0.033)
methodological	2	3370 (0.023)	#SAKNAS!	1612 (0.066)
methylphenidate	2	3370 (0.023)	2293 (0.034)	#SAKNAS!
mexican-american	2	3370 (0.023)	1457 (0.069)	#SAKNAS!
microbes	2	3370 (0.023)	#SAKNAS!	#SAKNAS!
microplastics	2	3370 (0.023)	#SAKNAS!	#SAKNAS!
microstructure	2	3370 (0.023)	#SAKNAS!	2492 (0.033)
middle-income	2	3370 (0.023)	2293 (0.034)	#SAKNAS!
midgut	2	3370 (0.023)	1457 (0.069)	#SAKNAS!
might	2	3370 (0.023)	1457 (0.069)	#SAKNAS!
migrants	2	3370 (0.023)	#SAKNAS!	2492 (0.033)
milking	2	3370 (0.023)	2293 (0.034)	2492 (0.033)
mimicry	2	3370 (0.023)	#SAKNAS!	1612 (0.066)
mineralization	2	3370 (0.023)	#SAKNAS!	2492 (0.033)
minor	2	3370 (0.023)	2293 (0.034)	#SAKNAS!
misperception	2	3370 (0.023)	#SAKNAS!	2492 (0.033)
misuse	2	3370 (0.023)	2293 (0.034)	#SAKNAS!
mitigates	2	3370 (0.023)	2293 (0.034)	2492 (0.033)
mitten	2	3370 (0.023)	#SAKNAS!	#SAKNAS!
modalities	2	3370 (0.023)	#SAKNAS!	2492 (0.033)
mode	2	3370 (0.023)	2293 (0.034)	#SAKNAS!
moderating	2	3370 (0.023)	#SAKNAS!	2492 (0.033)
moderation	2	3370 (0.023)	2293 (0.034)	#SAKNAS!
modifier	2	3370 (0.023)	#SAKNAS!	2492 (0.033)
modulating	2	3370 (0.023)	#SAKNAS!	2492 (0.033)

molitor	2	3370 (0.023)	#SAKNAS!	2492 (0.033)
mollis	2	3370 (0.023)	2293 (0.034)	2492 (0.033)
monensin	2	3370 (0.023)	#SAKNAS!	#SAKNAS!
monocentric	2	3370 (0.023)	#SAKNAS!	2492 (0.033)
monodon	2	3370 (0.023)	2293 (0.034)	#SAKNAS!
monoglyceride	2	3370 (0.023)	1457 (0.069)	#SAKNAS!
monozygotic	2	3370 (0.023)	1457 (0.069)	#SAKNAS!
mood	2	3370 (0.023)	2293 (0.034)	2492 (0.033)
morphofunctional	2	3370 (0.023)	#SAKNAS!	#SAKNAS!
mother-child	2	3370 (0.023)	#SAKNAS!	2492 (0.033)
motherhood	2	3370 (0.023)	2293 (0.034)	2492 (0.033)
mother-young	2	3370 (0.023)	2293 (0.034)	2492 (0.033)
motion	2	3370 (0.023)	2293 (0.034)	#SAKNAS!
motivational	2	3370 (0.023)	2293 (0.034)	2492 (0.033)
mousse	2	3370 (0.023)	#SAKNAS!	#SAKNAS!
mouthwash	2	3370 (0.023)	#SAKNAS!	2492 (0.033)
mouthwashes	2	3370 (0.023)	#SAKNAS!	2492 (0.033)
moving	2	3370 (0.023)	#SAKNAS!	#SAKNAS!
mucosa	2	3370 (0.023)	#SAKNAS!	2492 (0.033)
muller	2	3370 (0.023)	1457 (0.069)	#SAKNAS!
multicentric	2	3370 (0.023)	2293 (0.034)	2492 (0.033)
multigrain	2	3370 (0.023)	#SAKNAS!	#SAKNAS!
multi-institutional	2	3370 (0.023)	#SAKNAS!	2492 (0.033)
multimodal	2	3370 (0.023)	#SAKNAS!	#SAKNAS!
multinational	2	3370 (0.023)	2293 (0.034)	2492 (0.033)
multi-omics	2	3370 (0.023)	#SAKNAS!	#SAKNAS!
multivariable	2	3370 (0.023)	#SAKNAS!	#SAKNAS!
mumbai	2	3370 (0.023)	#SAKNAS!	2492 (0.033)
munich	2	3370 (0.023)	#SAKNAS!	2492 (0.033)
musa	2	3370 (0.023)	#SAKNAS!	2492 (0.033)
musculoskeletal	2	3370 (0.023)	#SAKNAS!	2492 (0.033)
mustela	2	3370 (0.023)	1457 (0.069)	#SAKNAS!
mutualistic	2	3370 (0.023)	2293 (0.034)	2492 (0.033)
my	2	3370 (0.023)	#SAKNAS!	1612 (0.066)
mycotoxin	2	3370 (0.023)	#SAKNAS!	2492 (0.033)
myectomy	2	3370 (0.023)	#SAKNAS!	2492 (0.033)
myelopathy	2	3370 (0.023)	2293 (0.034)	#SAKNAS!
myeloperoxidase	2	3370 (0.023)	2293 (0.034)	#SAKNAS!
myths	2	3370 (0.023)	#SAKNAS!	2492 (0.033)
n-3	2	3370 (0.023)	1457 (0.069)	#SAKNAS!
nap	2	3370 (0.023)	#SAKNAS!	1612 (0.066)
naturally	2	3370 (0.023)	2293 (0.034)	2492 (0.033)
nauplii	2	3370 (0.023)	2293 (0.034)	2492 (0.033)

ne	2	3370 (0.023)	1457 (0.069)	#SAKNAS!
nematodes	2	3370 (0.023)	2293 (0.034)	2492 (0.033)
neoplasms	2	3370 (0.023)	2293 (0.034)	#SAKNAS!
nepal	2	3370 (0.023)	#SAKNAS!	1612 (0.066)
nepali	2	3370 (0.023)	2293 (0.034)	2492 (0.033)
nephropathy	2	3370 (0.023)	2293 (0.034)	#SAKNAS!
net	2	3370 (0.023)	1457 (0.069)	#SAKNAS!
networks	2	3370 (0.023)	#SAKNAS!	#SAKNAS!
neurobiological	2	3370 (0.023)	2293 (0.034)	2492 (0.033)
neuropeptide-y	2	3370 (0.023)	1457 (0.069)	#SAKNAS!
neurorehabilitation	2	3370 (0.023)	2293 (0.034)	#SAKNAS!
neurosecretory	2	3370 (0.023)	#SAKNAS!	2492 (0.033)
neurotensin	2	3370 (0.023)	1457 (0.069)	#SAKNAS!
nevada	2	3370 (0.023)	1457 (0.069)	#SAKNAS!
next	2	3370 (0.023)	#SAKNAS!	1612 (0.066)
niger	2	3370 (0.023)	#SAKNAS!	1612 (0.066)
nighttime	2	3370 (0.023)	#SAKNAS!	#SAKNAS!
niloticus	2	3370 (0.023)	2293 (0.034)	2492 (0.033)
nine-year	2	3370 (0.023)	2293 (0.034)	2492 (0.033)
noise	2	3370 (0.023)	#SAKNAS!	2492 (0.033)
non	2	3370 (0.023)	1457 (0.069)	#SAKNAS!
non-hispanic	2	3370 (0.023)	2293 (0.034)	2492 (0.033)
non-indigenous	2	3370 (0.023)	#SAKNAS!	1612 (0.066)
non-linear	2	3370 (0.023)	2293 (0.034)	2492 (0.033)
nonobese	2	3370 (0.023)	#SAKNAS!	2492 (0.033)
nonoral	2	3370 (0.023)	1457 (0.069)	#SAKNAS!
non-plaque-induced	2	3370 (0.023)	#SAKNAS!	1612 (0.066)
nonpregnant	2	3370 (0.023)	#SAKNAS!	1612 (0.066)
nonsmokers	2	3370 (0.023)	2293 (0.034)	2492 (0.033)
nor	2	3370 (0.023)	2293 (0.034)	2492 (0.033)
north-east	2	3370 (0.023)	2293 (0.034)	#SAKNAS!
north-western	2	3370 (0.023)	#SAKNAS!	2492 (0.033)
norvegicus	2	3370 (0.023)	2293 (0.034)	2492 (0.033)
nosocomial	2	3370 (0.023)	2293 (0.034)	#SAKNAS!
note	2	3370 (0.023)	1457 (0.069)	#SAKNAS!
npffr2	2	3370 (0.023)	2293 (0.034)	#SAKNAS!
nuclei	2	3370 (0.023)	2293 (0.034)	2492 (0.033)
nuculanidae	2	3370 (0.023)	1457 (0.069)	#SAKNAS!
nuptial	2	3370 (0.023)	#SAKNAS!	2492 (0.033)
nurse-led	2	3370 (0.023)	#SAKNAS!	2492 (0.033)
nursery	2	3370 (0.023)	#SAKNAS!	1612 (0.066)
nutritional-value	2	3370 (0.023)	1457 (0.069)	#SAKNAS!
nutrition-related	2	3370 (0.023)	#SAKNAS!	2492 (0.033)

nutritious	2	3370 (0.023)	#SAKNAS!	#SAKNAS!
oak	2	3370 (0.023)	2293 (0.034)	2492 (0.033)
obliqua	2	3370 (0.023)	1457 (0.069)	#SAKNAS!
observable	2	3370 (0.023)	#SAKNAS!	2492 (0.033)
obtain	2	3370 (0.023)	#SAKNAS!	2492 (0.033)
o-c2	2	3370 (0.023)	2293 (0.034)	2492 (0.033)
occluding	2	3370 (0.023)	#SAKNAS!	2492 (0.033)
octogenarians	2	3370 (0.023)	2293 (0.034)	2492 (0.033)
odontoid	2	3370 (0.023)	#SAKNAS!	1612 (0.066)
oesophagogastric	2	3370 (0.023)	2293 (0.034)	2492 (0.033)
offsets	2	3370 (0.023)	2293 (0.034)	2492 (0.033)
ohasama	2	3370 (0.023)	#SAKNAS!	2492 (0.033)
ohio	2	3370 (0.023)	1457 (0.069)	#SAKNAS!
oleracea	2	3370 (0.023)	2293 (0.034)	#SAKNAS!
oligopeptidase	2	3370 (0.023)	#SAKNAS!	2492 (0.033)
one-anastomosis	2	3370 (0.023)	#SAKNAS!	#SAKNAS!
open-label	2	3370 (0.023)	#SAKNAS!	1612 (0.066)
opportunity	2	3370 (0.023)	2293 (0.034)	#SAKNAS!
optimization	2	3370 (0.023)	#SAKNAS!	#SAKNAS!
opuntia	2	3370 (0.023)	#SAKNAS!	1612 (0.066)
oral-health-related	2	3370 (0.023)	#SAKNAS!	2492 (0.033)
orexin-a	2	3370 (0.023)	2293 (0.034)	2492 (0.033)
organoleptic	2	3370 (0.023)	2293 (0.034)	2492 (0.033)
organs	2	3370 (0.023)	2293 (0.034)	2492 (0.033)
orodispersible	2	3370 (0.023)	#SAKNAS!	2492 (0.033)
orosensory	2	3370 (0.023)	#SAKNAS!	1612 (0.066)
orseis	2	3370 (0.023)	1457 (0.069)	#SAKNAS!
orthoptera	2	3370 (0.023)	2293 (0.034)	#SAKNAS!
osas	2	3370 (0.023)	2293 (0.034)	#SAKNAS!
oscillations	2	3370 (0.023)	2293 (0.034)	2492 (0.033)
oscillatory	2	3370 (0.023)	2293 (0.034)	#SAKNAS!
osteotomy	2	3370 (0.023)	#SAKNAS!	2492 (0.033)
otassha	2	3370 (0.023)	#SAKNAS!	#SAKNAS!
otolaryngologist	2	3370 (0.023)	#SAKNAS!	#SAKNAS!
otolaryngology	2	3370 (0.023)	#SAKNAS!	#SAKNAS!
otorhinolaryngological	2	3370 (0.023)	#SAKNAS!	1612 (0.066)
outdoor	2	3370 (0.023)	2293 (0.034)	2492 (0.033)
outside	2	3370 (0.023)	#SAKNAS!	2492 (0.033)
overcoming	2	3370 (0.023)	#SAKNAS!	2492 (0.033)
overexpression	2	3370 (0.023)	2293 (0.034)	2492 (0.033)
overload	2	3370 (0.023)	#SAKNAS!	1612 (0.066)
overnutrition	2	3370 (0.023)	#SAKNAS!	1612 (0.066)
owner	2	3370 (0.023)	#SAKNAS!	1612 (0.066)

oxyrrhis	2	3370 (0.023)	1457 (0.069)	#SAKNAS!
package	2	3370 (0.023)	2293 (0.034)	#SAKNAS!
paclitaxel	2	3370 (0.023)	1457 (0.069)	#SAKNAS!
pair	2	3370 (0.023)	2293 (0.034)	#SAKNAS!
paleopathology	2	3370 (0.023)	#SAKNAS!	1612 (0.066)
palestine	2	3370 (0.023)	#SAKNAS!	2492 (0.033)
palestinian	2	3370 (0.023)	#SAKNAS!	#SAKNAS!
pancreatoduodenectomy	2	3370 (0.023)	2293 (0.034)	2492 (0.033)
papillae	2	3370 (0.023)	#SAKNAS!	2492 (0.033)
papillomavirus-positive	2	3370 (0.023)	#SAKNAS!	1612 (0.066)
paradigm	2	3370 (0.023)	2293 (0.034)	2492 (0.033)
paralarvae	2	3370 (0.023)	#SAKNAS!	#SAKNAS!
paralysis	2	3370 (0.023)	2293 (0.034)	#SAKNAS!
parana	2	3370 (0.023)	1457 (0.069)	#SAKNAS!
parasite	2	3370 (0.023)	2293 (0.034)	#SAKNAS!
parasitoids	2	3370 (0.023)	2293 (0.034)	#SAKNAS!
parathyroid	2	3370 (0.023)	2293 (0.034)	#SAKNAS!
parental-reported	2	3370 (0.023)	2293 (0.034)	#SAKNAS!
parent-child	2	3370 (0.023)	#SAKNAS!	1612 (0.066)
parenteral-nutrition	2	3370 (0.023)	1457 (0.069)	#SAKNAS!
paris	2	3370 (0.023)	#SAKNAS!	#SAKNAS!
park	2	3370 (0.023)	1457 (0.069)	#SAKNAS!
participate	2	3370 (0.023)	1457 (0.069)	#SAKNAS!
particles	2	3370 (0.023)	1457 (0.069)	#SAKNAS!
patagonian	2	3370 (0.023)	1457 (0.069)	#SAKNAS!
patelliferum	2	3370 (0.023)	1457 (0.069)	#SAKNAS!
pathologist	2	3370 (0.023)	#SAKNAS!	2492 (0.033)
patient-generated	2	3370 (0.023)	2293 (0.034)	2492 (0.033)
pdr	2	3370 (0.023)	#SAKNAS!	1612 (0.066)
pear	2	3370 (0.023)	#SAKNAS!	#SAKNAS!
pectoralis	2	3370 (0.023)	1457 (0.069)	#SAKNAS!
pectoris	2	3370 (0.023)	#SAKNAS!	1612 (0.066)
pediatrician	2	3370 (0.023)	2293 (0.034)	#SAKNAS!
peer	2	3370 (0.023)	#SAKNAS!	1612 (0.066)
pellets	2	3370 (0.023)	#SAKNAS!	2492 (0.033)
penetration-aspiration	2	3370 (0.023)	2293 (0.034)	2492 (0.033)
penguins	2	3370 (0.023)	2293 (0.034)	#SAKNAS!
peninsula	2	3370 (0.023)	2293 (0.034)	#SAKNAS!
peptic	2	3370 (0.023)	2293 (0.034)	2492 (0.033)
peptide-1	2	3370 (0.023)	#SAKNAS!	2492 (0.033)
perforator	2	3370 (0.023)	#SAKNAS!	#SAKNAS!
performing	2	3370 (0.023)	#SAKNAS!	#SAKNAS!
peri	2	3370 (0.023)	2293 (0.034)	2492 (0.033)

peri-implantitis	2	3370 (0.023)	#SAKNAS!	#SAKNAS!
periodontopathogenic	2	3370 (0.023)	#SAKNAS!	1612 (0.066)
peripherally	2	3370 (0.023)	#SAKNAS!	#SAKNAS!
periprosthetic	2	3370 (0.023)	#SAKNAS!	2492 (0.033)
persian	2	3370 (0.023)	#SAKNAS!	#SAKNAS!
persist	2	3370 (0.023)	2293 (0.034)	2492 (0.033)
persisting	2	3370 (0.023)	2293 (0.034)	2492 (0.033)
personality	2	3370 (0.023)	#SAKNAS!	2492 (0.033)
pet	2	3370 (0.023)	2293 (0.034)	2492 (0.033)
pg-sga	2	3370 (0.023)	2293 (0.034)	2492 (0.033)
phagocytosis	2	3370 (0.023)	#SAKNAS!	2492 (0.033)
phagostimulants	2	3370 (0.023)	1457 (0.069)	#SAKNAS!
pharmacovigilance	2	3370 (0.023)	#SAKNAS!	#SAKNAS!
pharynx	2	3370 (0.023)	2293 (0.034)	2492 (0.033)
phenolic	2	3370 (0.023)	1457 (0.069)	#SAKNAS!
phenology	2	3370 (0.023)	1457 (0.069)	#SAKNAS!
phenome-wide	2	3370 (0.023)	#SAKNAS!	2492 (0.033)
phenotyping	2	3370 (0.023)	#SAKNAS!	2492 (0.033)
pheromone	2	3370 (0.023)	2293 (0.034)	#SAKNAS!
phoniatric	2	3370 (0.023)	#SAKNAS!	#SAKNAS!
photoperiod	2	3370 (0.023)	1457 (0.069)	#SAKNAS!
phthalate	2	3370 (0.023)	#SAKNAS!	2492 (0.033)
phytoplasma	2	3370 (0.023)	#SAKNAS!	1612 (0.066)
picea	2	3370 (0.023)	1457 (0.069)	#SAKNAS!
picky	2	3370 (0.023)	#SAKNAS!	2492 (0.033)
pigra	2	3370 (0.023)	2293 (0.034)	2492 (0.033)
pine	2	3370 (0.023)	1457 (0.069)	#SAKNAS!
plant-insect	2	3370 (0.023)	#SAKNAS!	#SAKNAS!
plasminogen	2	3370 (0.023)	2293 (0.034)	#SAKNAS!
platelet	2	3370 (0.023)	#SAKNAS!	2492 (0.033)
plating	2	3370 (0.023)	#SAKNAS!	2492 (0.033)
platinum	2	3370 (0.023)	1457 (0.069)	#SAKNAS!
plays	2	3370 (0.023)	#SAKNAS!	2492 (0.033)
pneumatic	2	3370 (0.023)	2293 (0.034)	#SAKNAS!
pocket	2	3370 (0.023)	#SAKNAS!	2492 (0.033)
pocketing	2	3370 (0.023)	#SAKNAS!	2492 (0.033)
pockets	2	3370 (0.023)	2293 (0.034)	2492 (0.033)
poem	2	3370 (0.023)	2293 (0.034)	#SAKNAS!
polyaromatic	2	3370 (0.023)	#SAKNAS!	2492 (0.033)
polycyclic	2	3370 (0.023)	#SAKNAS!	#SAKNAS!
polyethylene	2	3370 (0.023)	1457 (0.069)	#SAKNAS!
polyfluoroalkyl	2	3370 (0.023)	#SAKNAS!	2492 (0.033)
polymorpha	2	3370 (0.023)	2293 (0.034)	2492 (0.033)

polyurethane	2	3370 (0.023)	#SAKNAS!	2492 (0.033)
pomc	2	3370 (0.023)	#SAKNAS!	1612 (0.066)
pond	2	3370 (0.023)	2293 (0.034)	#SAKNAS!
pontine	2	3370 (0.023)	1457 (0.069)	#SAKNAS!
poorer	2	3370 (0.023)	2293 (0.034)	2492 (0.033)
popular	2	3370 (0.023)	#SAKNAS!	2492 (0.033)
porcellus	2	3370 (0.023)	#SAKNAS!	2492 (0.033)
porridge	2	3370 (0.023)	#SAKNAS!	#SAKNAS!
portable	2	3370 (0.023)	2293 (0.034)	#SAKNAS!
portion	2	3370 (0.023)	2293 (0.034)	2492 (0.033)
porto	2	3370 (0.023)	1457 (0.069)	#SAKNAS!
portugal	2	3370 (0.023)	2293 (0.034)	#SAKNAS!
positions	2	3370 (0.023)	#SAKNAS!	1612 (0.066)
positron	2	3370 (0.023)	1457 (0.069)	#SAKNAS!
post-covid-19	2	3370 (0.023)	#SAKNAS!	#SAKNAS!
poster	2	3370 (0.023)	#SAKNAS!	2492 (0.033)
post-esophagectomy	2	3370 (0.023)	#SAKNAS!	2492 (0.033)
post-intensive	2	3370 (0.023)	#SAKNAS!	#SAKNAS!
postlaryngectomy	2	3370 (0.023)	1457 (0.069)	#SAKNAS!
post-menopausal	2	3370 (0.023)	#SAKNAS!	2492 (0.033)
post-radiotherapy	2	3370 (0.023)	#SAKNAS!	2492 (0.033)
posttreatment	2	3370 (0.023)	#SAKNAS!	2492 (0.033)
postural	2	3370 (0.023)	#SAKNAS!	2492 (0.033)
post-weaning	2	3370 (0.023)	#SAKNAS!	2492 (0.033)
potassium	2	3370 (0.023)	#SAKNAS!	2492 (0.033)
pouch	2	3370 (0.023)	2293 (0.034)	2492 (0.033)
pouches	2	3370 (0.023)	#SAKNAS!	2492 (0.033)
practice-based	2	3370 (0.023)	2293 (0.034)	#SAKNAS!
pragmatic	2	3370 (0.023)	#SAKNAS!	1612 (0.066)
prairie	2	3370 (0.023)	#SAKNAS!	1612 (0.066)
pre-	2	3370 (0.023)	1457 (0.069)	#SAKNAS!
prebiotic	2	3370 (0.023)	#SAKNAS!	2492 (0.033)
predyces簣	2	3370 (0.023)	2293 (0.034)	2492 (0.033)
preeclampsia	2	3370 (0.023)	2293 (0.034)	#SAKNAS!
pre-existing	2	3370 (0.023)	2293 (0.034)	#SAKNAS!
pregnancies	2	3370 (0.023)	#SAKNAS!	2492 (0.033)
premature	2	3370 (0.023)	#SAKNAS!	1612 (0.066)
prematurity	2	3370 (0.023)	2293 (0.034)	#SAKNAS!
pre-operative	2	3370 (0.023)	#SAKNAS!	#SAKNAS!
pre-pregnancy	2	3370 (0.023)	#SAKNAS!	#SAKNAS!
presbytis	2	3370 (0.023)	#SAKNAS!	2492 (0.033)
preschool-children	2	3370 (0.023)	2293 (0.034)	#SAKNAS!
preserves	2	3370 (0.023)	#SAKNAS!	1612 (0.066)

pressures	2	3370 (0.023)	#SAKNAS!	1612 (0.066)
preventative	2	3370 (0.023)	2293 (0.034)	#SAKNAS!
prevotella	2	3370 (0.023)	2293 (0.034)	2492 (0.033)
probabilistic	2	3370 (0.023)	#SAKNAS!	2492 (0.033)
probable	2	3370 (0.023)	#SAKNAS!	1612 (0.066)
probiotic	2	3370 (0.023)	#SAKNAS!	2492 (0.033)
procera	2	3370 (0.023)	2293 (0.034)	2492 (0.033)
produce	2	3370 (0.023)	2293 (0.034)	#SAKNAS!
producer	2	3370 (0.023)	1457 (0.069)	#SAKNAS!
produces	2	3370 (0.023)	2293 (0.034)	2492 (0.033)
professors	2	3370 (0.023)	#SAKNAS!	1612 (0.066)
profitability	2	3370 (0.023)	2293 (0.034)	#SAKNAS!
profunda	2	3370 (0.023)	#SAKNAS!	#SAKNAS!
programming	2	3370 (0.023)	1457 (0.069)	#SAKNAS!
pro-inflammatory	2	3370 (0.023)	2293 (0.034)	2492 (0.033)
projections	2	3370 (0.023)	#SAKNAS!	1612 (0.066)
proopiomelanocortin	2	3370 (0.023)	#SAKNAS!	2492 (0.033)
propionate	2	3370 (0.023)	2293 (0.034)	2492 (0.033)
propionic	2	3370 (0.023)	#SAKNAS!	1612 (0.066)
propofol	2	3370 (0.023)	#SAKNAS!	2492 (0.033)
propolis	2	3370 (0.023)	2293 (0.034)	2492 (0.033)
proportion	2	3370 (0.023)	2293 (0.034)	#SAKNAS!
prostaglandin	2	3370 (0.023)	2293 (0.034)	#SAKNAS!
protease	2	3370 (0.023)	2293 (0.034)	2492 (0.033)
protect	2	3370 (0.023)	#SAKNAS!	1612 (0.066)
protecting	2	3370 (0.023)	#SAKNAS!	1612 (0.066)
proteome	2	3370 (0.023)	2293 (0.034)	#SAKNAS!
proximate	2	3370 (0.023)	#SAKNAS!	2492 (0.033)
proximity	2	3370 (0.023)	2293 (0.034)	#SAKNAS!
proxy	2	3370 (0.023)	#SAKNAS!	#SAKNAS!
pruni	2	3370 (0.023)	#SAKNAS!	1612 (0.066)
prymnesium	2	3370 (0.023)	1457 (0.069)	#SAKNAS!
pseudomonas	2	3370 (0.023)	2293 (0.034)	#SAKNAS!
psychopathology	2	3370 (0.023)	2293 (0.034)	#SAKNAS!
psychosis	2	3370 (0.023)	2293 (0.034)	#SAKNAS!
pta	2	3370 (0.023)	#SAKNAS!	2492 (0.033)
puberty	2	3370 (0.023)	2293 (0.034)	2492 (0.033)
puntazzo	2	3370 (0.023)	1457 (0.069)	#SAKNAS!
purchasing	2	3370 (0.023)	1457 (0.069)	#SAKNAS!
purposes	2	3370 (0.023)	2293 (0.034)	#SAKNAS!
pursuant	2	3370 (0.023)	#SAKNAS!	1612 (0.066)
putative	2	3370 (0.023)	#SAKNAS!	1612 (0.066)
putting	2	3370 (0.023)	1457 (0.069)	#SAKNAS!

pycnogenol箦	2	3370 (0.023)	#SAKNAS!	#SAKNAS!
pyramid	2	3370 (0.023)	2293 (0.034)	2492 (0.033)
pyy	2	3370 (0.023)	#SAKNAS!	2492 (0.033)
quadriceps	2	3370 (0.023)	#SAKNAS!	2492 (0.033)
quail	2	3370 (0.023)	1457 (0.069)	#SAKNAS!
quebec	2	3370 (0.023)	2293 (0.034)	2492 (0.033)
questionnaires	2	3370 (0.023)	#SAKNAS!	2492 (0.033)
quick	2	3370 (0.023)	2293 (0.034)	2492 (0.033)
rabbit	2	3370 (0.023)	2293 (0.034)	2492 (0.033)
rabbits	2	3370 (0.023)	1457 (0.069)	#SAKNAS!
races	2	3370 (0.023)	2293 (0.034)	#SAKNAS!
radial	2	3370 (0.023)	1457 (0.069)	#SAKNAS!
radical	2	3370 (0.023)	2293 (0.034)	#SAKNAS!
radiographically	2	3370 (0.023)	#SAKNAS!	2492 (0.033)
ramadan	2	3370 (0.023)	1457 (0.069)	#SAKNAS!
random	2	3370 (0.023)	2293 (0.034)	2492 (0.033)
rangelands	2	3370 (0.023)	#SAKNAS!	2492 (0.033)
ranges	2	3370 (0.023)	1457 (0.069)	#SAKNAS!
rank	2	3370 (0.023)	2293 (0.034)	2492 (0.033)
rations	2	3370 (0.023)	2293 (0.034)	#SAKNAS!
reassessment	2	3370 (0.023)	#SAKNAS!	2492 (0.033)
recognition	2	3370 (0.023)	#SAKNAS!	#SAKNAS!
recommendation	2	3370 (0.023)	#SAKNAS!	2492 (0.033)
recommending	2	3370 (0.023)	#SAKNAS!	2492 (0.033)
records	2	3370 (0.023)	#SAKNAS!	2492 (0.033)
records-based	2	3370 (0.023)	#SAKNAS!	2492 (0.033)
recreational	2	3370 (0.023)	#SAKNAS!	2492 (0.033)
redox	2	3370 (0.023)	2293 (0.034)	#SAKNAS!
reef	2	3370 (0.023)	#SAKNAS!	2492 (0.033)
reference	2	3370 (0.023)	2293 (0.034)	#SAKNAS!
reflection	2	3370 (0.023)	2293 (0.034)	2492 (0.033)
regimen	2	3370 (0.023)	#SAKNAS!	#SAKNAS!
regimes	2	3370 (0.023)	1457 (0.069)	#SAKNAS!
register	2	3370 (0.023)	#SAKNAS!	#SAKNAS!
regius	2	3370 (0.023)	#SAKNAS!	#SAKNAS!
regulations	2	3370 (0.023)	2293 (0.034)	#SAKNAS!
regulator	2	3370 (0.023)	2293 (0.034)	#SAKNAS!
regurgitation	2	3370 (0.023)	2293 (0.034)	#SAKNAS!
relief	2	3370 (0.023)	#SAKNAS!	2492 (0.033)
remains	2	3370 (0.023)	2293 (0.034)	#SAKNAS!
remote	2	3370 (0.023)	#SAKNAS!	1612 (0.066)
repeat	2	3370 (0.023)	#SAKNAS!	2492 (0.033)
require	2	3370 (0.023)	2293 (0.034)	2492 (0.033)

requirements	2	3370 (0.023)	2293 (0.034)	2492 (0.033)
resectable	2	3370 (0.023)	2293 (0.034)	2492 (0.033)
reserve	2	3370 (0.023)	2293 (0.034)	#SAKNAS!
residence	2	3370 (0.023)	2293 (0.034)	#SAKNAS!
residues	2	3370 (0.023)	2293 (0.034)	#SAKNAS!
resistant	2	3370 (0.023)	2293 (0.034)	2492 (0.033)
resolution	2	3370 (0.023)	#SAKNAS!	2492 (0.033)
resorption	2	3370 (0.023)	2293 (0.034)	#SAKNAS!
respiration	2	3370 (0.023)	2293 (0.034)	2492 (0.033)
responders	2	3370 (0.023)	2293 (0.034)	#SAKNAS!
rest	2	3370 (0.023)	2293 (0.034)	2492 (0.033)
resting	2	3370 (0.023)	#SAKNAS!	1612 (0.066)
restores	2	3370 (0.023)	#SAKNAS!	2492 (0.033)
resumption	2	3370 (0.023)	2293 (0.034)	2492 (0.033)
retardants	2	3370 (0.023)	#SAKNAS!	#SAKNAS!
retinol	2	3370 (0.023)	#SAKNAS!	#SAKNAS!
retirement	2	3370 (0.023)	1457 (0.069)	#SAKNAS!
retort	2	3370 (0.023)	#SAKNAS!	#SAKNAS!
retrosternal	2	3370 (0.023)	1457 (0.069)	#SAKNAS!
return	2	3370 (0.023)	2293 (0.034)	2492 (0.033)
revisional	2	3370 (0.023)	2293 (0.034)	#SAKNAS!
rewarding	2	3370 (0.023)	#SAKNAS!	1612 (0.066)
rewards	2	3370 (0.023)	2293 (0.034)	2492 (0.033)
rhesus	2	3370 (0.023)	2293 (0.034)	2492 (0.033)
rhesus-monkeys	2	3370 (0.023)	1457 (0.069)	#SAKNAS!
rheumatic	2	3370 (0.023)	2293 (0.034)	2492 (0.033)
rhinella	2	3370 (0.023)	#SAKNAS!	2492 (0.033)
rhinoceroses	2	3370 (0.023)	2293 (0.034)	2492 (0.033)
rhinopithecus	2	3370 (0.023)	#SAKNAS!	1612 (0.066)
rican	2	3370 (0.023)	#SAKNAS!	1612 (0.066)
rich	2	3370 (0.023)	#SAKNAS!	1612 (0.066)
ring	2	3370 (0.023)	#SAKNAS!	2492 (0.033)
rings	2	3370 (0.023)	1457 (0.069)	#SAKNAS!
risky	2	3370 (0.023)	2293 (0.034)	#SAKNAS!
rna	2	3370 (0.023)	#SAKNAS!	2492 (0.033)
robot	2	3370 (0.023)	#SAKNAS!	2492 (0.033)
rodents	2	3370 (0.023)	#SAKNAS!	1612 (0.066)
roe	2	3370 (0.023)	2293 (0.034)	#SAKNAS!
roman	2	3370 (0.023)	2293 (0.034)	2492 (0.033)
rossetti	2	3370 (0.023)	#SAKNAS!	1612 (0.066)
rotationally	2	3370 (0.023)	#SAKNAS!	1612 (0.066)
roux	2	3370 (0.023)	#SAKNAS!	1612 (0.066)
rtog	2	3370 (0.023)	2293 (0.034)	2492 (0.033)

rules	2	3370 (0.023)	#SAKNAS!	2492 (0.033)
ruminant	2	3370 (0.023)	2293 (0.034)	2492 (0.033)
rumination	2	3370 (0.023)	#SAKNAS!	#SAKNAS!
sacc	2	3370 (0.023)	#SAKNAS!	1612 (0.066)
saccharomyces	2	3370 (0.023)	#SAKNAS!	2492 (0.033)
salmo	2	3370 (0.023)	2293 (0.034)	#SAKNAS!
salts	2	3370 (0.023)	2293 (0.034)	#SAKNAS!
sara	2	3370 (0.023)	2293 (0.034)	2492 (0.033)
sardinian	2	3370 (0.023)	#SAKNAS!	#SAKNAS!
satiation	2	3370 (0.023)	#SAKNAS!	1612 (0.066)
scallop	2	3370 (0.023)	2293 (0.034)	2492 (0.033)
scavenger	2	3370 (0.023)	1457 (0.069)	#SAKNAS!
schatzki	2	3370 (0.023)	1457 (0.069)	#SAKNAS!
schweinfurthii	2	3370 (0.023)	#SAKNAS!	1612 (0.066)
sciences	2	3370 (0.023)	#SAKNAS!	2492 (0.033)
scoring	2	3370 (0.023)	2293 (0.034)	2492 (0.033)
scottish	2	3370 (0.023)	1457 (0.069)	#SAKNAS!
seabream	2	3370 (0.023)	1457 (0.069)	#SAKNAS!
seagrass	2	3370 (0.023)	#SAKNAS!	#SAKNAS!
sealing	2	3370 (0.023)	#SAKNAS!	2492 (0.033)
search	2	3370 (0.023)	#SAKNAS!	#SAKNAS!
seaweed	2	3370 (0.023)	2293 (0.034)	#SAKNAS!
second-generation	2	3370 (0.023)	1457 (0.069)	#SAKNAS!
sedation	2	3370 (0.023)	#SAKNAS!	#SAKNAS!
seeds	2	3370 (0.023)	#SAKNAS!	1612 (0.066)
seeking	2	3370 (0.023)	2293 (0.034)	2492 (0.033)
segmentation	2	3370 (0.023)	2293 (0.034)	2492 (0.033)
select	2	3370 (0.023)	#SAKNAS!	2492 (0.033)
self-assessment	2	3370 (0.023)	#SAKNAS!	#SAKNAS!
self-feeders	2	3370 (0.023)	1457 (0.069)	#SAKNAS!
self-regulation	2	3370 (0.023)	2293 (0.034)	2492 (0.033)
semiarid	2	3370 (0.023)	2293 (0.034)	#SAKNAS!
semi-arid	2	3370 (0.023)	2293 (0.034)	#SAKNAS!
semi-solid	2	3370 (0.023)	#SAKNAS!	2492 (0.033)
semisynthetic	2	3370 (0.023)	2293 (0.034)	#SAKNAS!
senegalensis	2	3370 (0.023)	1457 (0.069)	#SAKNAS!
senegalese	2	3370 (0.023)	2293 (0.034)	2492 (0.033)
senescence	2	3370 (0.023)	#SAKNAS!	2492 (0.033)
senior	2	3370 (0.023)	#SAKNAS!	2492 (0.033)
sensor	2	3370 (0.023)	2293 (0.034)	2492 (0.033)
sensors	2	3370 (0.023)	#SAKNAS!	#SAKNAS!
serbian	2	3370 (0.023)	#SAKNAS!	1612 (0.066)
serotonergic	2	3370 (0.023)	2293 (0.034)	#SAKNAS!

serving	2	3370 (0.023)	#SAKNAS!	#SAKNAS!
sesarmidae	2	3370 (0.023)	2293 (0.034)	2492 (0.033)
shaping	2	3370 (0.023)	2293 (0.034)	#SAKNAS!
shark	2	3370 (0.023)	#SAKNAS!	2492 (0.033)
sharpi	2	3370 (0.023)	2293 (0.034)	2492 (0.033)
sharpsnout	2	3370 (0.023)	1457 (0.069)	#SAKNAS!
shear	2	3370 (0.023)	#SAKNAS!	2492 (0.033)
shearwater	2	3370 (0.023)	2293 (0.034)	2492 (0.033)
shellfish	2	3370 (0.023)	2293 (0.034)	2492 (0.033)
shika	2	3370 (0.023)	#SAKNAS!	#SAKNAS!
short-chain	2	3370 (0.023)	2293 (0.034)	#SAKNAS!
shorter	2	3370 (0.023)	2293 (0.034)	#SAKNAS!
shrimps	2	3370 (0.023)	#SAKNAS!	1612 (0.066)
siberian	2	3370 (0.023)	#SAKNAS!	2492 (0.033)
sibling	2	3370 (0.023)	#SAKNAS!	1612 (0.066)
siblings	2	3370 (0.023)	1457 (0.069)	#SAKNAS!
sicca	2	3370 (0.023)	#SAKNAS!	2492 (0.033)
signature	2	3370 (0.023)	#SAKNAS!	#SAKNAS!
silicone	2	3370 (0.023)	1457 (0.069)	#SAKNAS!
sinensis	2	3370 (0.023)	#SAKNAS!	#SAKNAS!
singapore	2	3370 (0.023)	#SAKNAS!	2492 (0.033)
single-arm	2	3370 (0.023)	#SAKNAS!	#SAKNAS!
single-blind	2	3370 (0.023)	#SAKNAS!	2492 (0.033)
single-institution	2	3370 (0.023)	#SAKNAS!	#SAKNAS!
single-session	2	3370 (0.023)	1457 (0.069)	#SAKNAS!
sitobion	2	3370 (0.023)	#SAKNAS!	2492 (0.033)
sixth	2	3370 (0.023)	#SAKNAS!	2492 (0.033)
sixth-instar	2	3370 (0.023)	1457 (0.069)	#SAKNAS!
sizes	2	3370 (0.023)	#SAKNAS!	2492 (0.033)
skeletons	2	3370 (0.023)	1457 (0.069)	#SAKNAS!
skill	2	3370 (0.023)	#SAKNAS!	2492 (0.033)
sleepiness	2	3370 (0.023)	#SAKNAS!	2492 (0.033)
slow-growing	2	3370 (0.023)	1457 (0.069)	#SAKNAS!
snub-nosed	2	3370 (0.023)	#SAKNAS!	1612 (0.066)
societies	2	3370 (0.023)	2293 (0.034)	#SAKNAS!
socio-behavioral	2	3370 (0.023)	1457 (0.069)	#SAKNAS!
soda	2	3370 (0.023)	1457 (0.069)	#SAKNAS!
soldier	2	3370 (0.023)	#SAKNAS!	#SAKNAS!
solea	2	3370 (0.023)	1457 (0.069)	#SAKNAS!
solenopsis	2	3370 (0.023)	1457 (0.069)	#SAKNAS!
soluble	2	3370 (0.023)	#SAKNAS!	1612 (0.066)
somatic	2	3370 (0.023)	#SAKNAS!	2492 (0.033)
sorting	2	3370 (0.023)	#SAKNAS!	1612 (0.066)

sow	2	3370 (0.023)	2293 (0.034)	#SAKNAS!
spasm	2	3370 (0.023)	#SAKNAS!	#SAKNAS!
spastic	2	3370 (0.023)	#SAKNAS!	1612 (0.066)
spatiotemporal	2	3370 (0.023)	#SAKNAS!	2492 (0.033)
specialization	2	3370 (0.023)	2293 (0.034)	2492 (0.033)
spectrometry	2	3370 (0.023)	#SAKNAS!	2492 (0.033)
spineless	2	3370 (0.023)	#SAKNAS!	1612 (0.066)
spinocerebellar	2	3370 (0.023)	2293 (0.034)	#SAKNAS!
spondylitis	2	3370 (0.023)	2293 (0.034)	2492 (0.033)
spondylosis	2	3370 (0.023)	#SAKNAS!	1612 (0.066)
spondylotic	2	3370 (0.023)	2293 (0.034)	#SAKNAS!
sporadic	2	3370 (0.023)	#SAKNAS!	2492 (0.033)
spread	2	3370 (0.023)	2293 (0.034)	2492 (0.033)
squirrels	2	3370 (0.023)	2293 (0.034)	#SAKNAS!
st	2	3370 (0.023)	2293 (0.034)	2492 (0.033)
standing	2	3370 (0.023)	2293 (0.034)	#SAKNAS!
star	2	3370 (0.023)	1457 (0.069)	#SAKNAS!
starchy	2	3370 (0.023)	#SAKNAS!	2492 (0.033)
start	2	3370 (0.023)	#SAKNAS!	2492 (0.033)
statistical	2	3370 (0.023)	#SAKNAS!	#SAKNAS!
statuses	2	3370 (0.023)	#SAKNAS!	2492 (0.033)
steady	2	3370 (0.023)	#SAKNAS!	1612 (0.066)
stiffness	2	3370 (0.023)	#SAKNAS!	2492 (0.033)
stimulates	2	3370 (0.023)	2293 (0.034)	2492 (0.033)
strata	2	3370 (0.023)	#SAKNAS!	2492 (0.033)
striatum	2	3370 (0.023)	#SAKNAS!	1612 (0.066)
striatus	2	3370 (0.023)	#SAKNAS!	1612 (0.066)
stricta	2	3370 (0.023)	#SAKNAS!	1612 (0.066)
strombus	2	3370 (0.023)	1457 (0.069)	#SAKNAS!
sub-acute	2	3370 (0.023)	#SAKNAS!	2492 (0.033)
subarachnoid	2	3370 (0.023)	2293 (0.034)	#SAKNAS!
sub-clinical	2	3370 (0.023)	1457 (0.069)	#SAKNAS!
submucosal	2	3370 (0.023)	2293 (0.034)	#SAKNAS!
substance	2	3370 (0.023)	2293 (0.034)	2492 (0.033)
substitutes	2	3370 (0.023)	#SAKNAS!	2492 (0.033)
subtilis	2	3370 (0.023)	1457 (0.069)	#SAKNAS!
subtotal	2	3370 (0.023)	#SAKNAS!	#SAKNAS!
subtropical	2	3370 (0.023)	#SAKNAS!	1612 (0.066)
suburban	2	3370 (0.023)	2293 (0.034)	2492 (0.033)
sucking	2	3370 (0.023)	#SAKNAS!	1612 (0.066)
sudan	2	3370 (0.023)	#SAKNAS!	2492 (0.033)
sugar-free	2	3370 (0.023)	#SAKNAS!	1612 (0.066)
sugary	2	3370 (0.023)	#SAKNAS!	#SAKNAS!

superselective	2	3370 (0.023)	2293 (0.034)	2492 (0.033)
supporting	2	3370 (0.023)	#SAKNAS!	#SAKNAS!
supports	2	3370 (0.023)	#SAKNAS!	2492 (0.033)
suppressive	2	3370 (0.023)	1457 (0.069)	#SAKNAS!
supraglottic	2	3370 (0.023)	2293 (0.034)	#SAKNAS!
surgically	2	3370 (0.023)	2293 (0.034)	2492 (0.033)
survey-based	2	3370 (0.023)	#SAKNAS!	2492 (0.033)
surviving	2	3370 (0.023)	2293 (0.034)	2492 (0.033)
survivorship	2	3370 (0.023)	#SAKNAS!	1612 (0.066)
suspension-feeding	2	3370 (0.023)	2293 (0.034)	2492 (0.033)
suzukii	2	3370 (0.023)	#SAKNAS!	1612 (0.066)
sweets	2	3370 (0.023)	2293 (0.034)	2492 (0.033)
swine	2	3370 (0.023)	2293 (0.034)	#SAKNAS!
symbionts	2	3370 (0.023)	2293 (0.034)	2492 (0.033)
synaptic	2	3370 (0.023)	2293 (0.034)	2492 (0.033)
synchronous	2	3370 (0.023)	2293 (0.034)	2492 (0.033)
synthetic	2	3370 (0.023)	2293 (0.034)	2492 (0.033)
taberu	2	3370 (0.023)	#SAKNAS!	1612 (0.066)
tactile	2	3370 (0.023)	1457 (0.069)	#SAKNAS!
take	2	3370 (0.023)	#SAKNAS!	2492 (0.033)
taking	2	3370 (0.023)	#SAKNAS!	2492 (0.033)
talk	2	3370 (0.023)	#SAKNAS!	2492 (0.033)
talking	2	3370 (0.023)	#SAKNAS!	1612 (0.066)
tambaqui	2	3370 (0.023)	#SAKNAS!	1612 (0.066)
tannin	2	3370 (0.023)	2293 (0.034)	2492 (0.033)
tannin-rich	2	3370 (0.023)	2293 (0.034)	2492 (0.033)
tanzanian	2	3370 (0.023)	1457 (0.069)	#SAKNAS!
tasteless	2	3370 (0.023)	#SAKNAS!	2492 (0.033)
taurine	2	3370 (0.023)	2293 (0.034)	2492 (0.033)
taxes	2	3370 (0.023)	#SAKNAS!	2492 (0.033)
t-cell	2	3370 (0.023)	2293 (0.034)	#SAKNAS!
tdcs	2	3370 (0.023)	#SAKNAS!	2492 (0.033)
teaching	2	3370 (0.023)	#SAKNAS!	#SAKNAS!
teams	2	3370 (0.023)	2293 (0.034)	#SAKNAS!
tegmental	2	3370 (0.023)	#SAKNAS!	2492 (0.033)
telangiectasia	2	3370 (0.023)	2293 (0.034)	2492 (0.033)
tenebrio	2	3370 (0.023)	#SAKNAS!	2492 (0.033)
tension	2	3370 (0.023)	1457 (0.069)	#SAKNAS!
test-retest	2	3370 (0.023)	2293 (0.034)	2492 (0.033)
tetanus	2	3370 (0.023)	#SAKNAS!	1612 (0.066)
tetricus	2	3370 (0.023)	#SAKNAS!	#SAKNAS!
thick	2	3370 (0.023)	2293 (0.034)	#SAKNAS!
thin	2	3370 (0.023)	2293 (0.034)	#SAKNAS!

thinness	2	3370 (0.023)	#SAKNAS!	1612 (0.066)
thiocyanate	2	3370 (0.023)	#SAKNAS!	2492 (0.033)
thixotropy	2	3370 (0.023)	2293 (0.034)	2492 (0.033)
thoracoscopic	2	3370 (0.023)	#SAKNAS!	1612 (0.066)
threat	2	3370 (0.023)	#SAKNAS!	#SAKNAS!
threshold	2	3370 (0.023)	2293 (0.034)	2492 (0.033)
thymus	2	3370 (0.023)	2293 (0.034)	#SAKNAS!
tibetan	2	3370 (0.023)	#SAKNAS!	1612 (0.066)
tissues	2	3370 (0.023)	#SAKNAS!	1612 (0.066)
titicaca	2	3370 (0.023)	#SAKNAS!	2492 (0.033)
toddlerhood	2	3370 (0.023)	#SAKNAS!	2492 (0.033)
tokyo	2	3370 (0.023)	2293 (0.034)	2492 (0.033)
tolerable	2	3370 (0.023)	#SAKNAS!	2492 (0.033)
tomato	2	3370 (0.023)	1457 (0.069)	#SAKNAS!
tomography	2	3370 (0.023)	1457 (0.069)	#SAKNAS!
tomorrow	2	3370 (0.023)	2293 (0.034)	2492 (0.033)
topical	2	3370 (0.023)	1457 (0.069)	#SAKNAS!
tracheo-oesophageal	2	3370 (0.023)	#SAKNAS!	2492 (0.033)
tracheotomy	2	3370 (0.023)	2293 (0.034)	#SAKNAS!
tracking	2	3370 (0.023)	1457 (0.069)	#SAKNAS!
traction	2	3370 (0.023)	2293 (0.034)	#SAKNAS!
traditions	2	3370 (0.023)	#SAKNAS!	1612 (0.066)
train	2	3370 (0.023)	#SAKNAS!	2492 (0.033)
transcription	2	3370 (0.023)	2293 (0.034)	#SAKNAS!
transcriptional	2	3370 (0.023)	2293 (0.034)	#SAKNAS!
transcriptomic	2	3370 (0.023)	#SAKNAS!	#SAKNAS!
transgastric	2	3370 (0.023)	#SAKNAS!	2492 (0.033)
transgenerational	2	3370 (0.023)	#SAKNAS!	#SAKNAS!
transhiatal	2	3370 (0.023)	1457 (0.069)	#SAKNAS!
translating	2	3370 (0.023)	2293 (0.034)	2492 (0.033)
translational	2	3370 (0.023)	#SAKNAS!	2492 (0.033)
translocation	2	3370 (0.023)	#SAKNAS!	1612 (0.066)
transmission	2	3370 (0.023)	#SAKNAS!	1612 (0.066)
tree	2	3370 (0.023)	2293 (0.034)	2492 (0.033)
trees	2	3370 (0.023)	2293 (0.034)	#SAKNAS!
trefoil	2	3370 (0.023)	2293 (0.034)	#SAKNAS!
treponema	2	3370 (0.023)	2293 (0.034)	2492 (0.033)
trichoptera	2	3370 (0.023)	2293 (0.034)	#SAKNAS!
trinidad	2	3370 (0.023)	2293 (0.034)	2492 (0.033)
triple	2	3370 (0.023)	2293 (0.034)	2492 (0.033)
tromso	2	3370 (0.023)	#SAKNAS!	#SAKNAS!
trypanosoma	2	3370 (0.023)	#SAKNAS!	2492 (0.033)
tube-enteral-fed	2	3370 (0.023)	1457 (0.069)	#SAKNAS!

tumours	2	3370 (0.023)	2293 (0.034)	2492 (0.033)
tunisia	2	3370 (0.023)	2293 (0.034)	2492 (0.033)
tv	2	3370 (0.023)	2293 (0.034)	2492 (0.033)
twice	2	3370 (0.023)	2293 (0.034)	2492 (0.033)
twins	2	3370 (0.023)	2293 (0.034)	2492 (0.033)
typhimurium	2	3370 (0.023)	2293 (0.034)	#SAKNAS!
typical	2	3370 (0.023)	2293 (0.034)	2492 (0.033)
tyrosine	2	3370 (0.023)	#SAKNAS!	#SAKNAS!
u.s	2	3370 (0.023)	2293 (0.034)	2492 (0.033)
ulcer	2	3370 (0.023)	#SAKNAS!	1612 (0.066)
ulceration	2	3370 (0.023)	2293 (0.034)	2492 (0.033)
ultradian	2	3370 (0.023)	2293 (0.034)	#SAKNAS!
ultra-endurance	2	3370 (0.023)	#SAKNAS!	#SAKNAS!
ultrasonographic	2	3370 (0.023)	#SAKNAS!	2492 (0.033)
under-five	2	3370 (0.023)	#SAKNAS!	#SAKNAS!
undergone	2	3370 (0.023)	#SAKNAS!	#SAKNAS!
underpinning	2	3370 (0.023)	2293 (0.034)	2492 (0.033)
unintentional	2	3370 (0.023)	2293 (0.034)	2492 (0.033)
unpredictable	2	3370 (0.023)	2293 (0.034)	2492 (0.033)
unsuccessful	2	3370 (0.023)	#SAKNAS!	#SAKNAS!
upwelling	2	3370 (0.023)	#SAKNAS!	2492 (0.033)
usage	2	3370 (0.023)	1457 (0.069)	#SAKNAS!
us-mexican	2	3370 (0.023)	#SAKNAS!	1612 (0.066)
usual	2	3370 (0.023)	2293 (0.034)	#SAKNAS!
utilisation	2	3370 (0.023)	2293 (0.034)	#SAKNAS!
utilizing	2	3370 (0.023)	#SAKNAS!	#SAKNAS!
vaccination	2	3370 (0.023)	#SAKNAS!	2492 (0.033)
vaginal	2	3370 (0.023)	2293 (0.034)	2492 (0.033)
valve	2	3370 (0.023)	2293 (0.034)	#SAKNAS!
vegan	2	3370 (0.023)	#SAKNAS!	1612 (0.066)
vegetable-exposure	2	3370 (0.023)	#SAKNAS!	2492 (0.033)
vegetarians	2	3370 (0.023)	2293 (0.034)	#SAKNAS!
ventilatory	2	3370 (0.023)	2293 (0.034)	2492 (0.033)
ventral	2	3370 (0.023)	#SAKNAS!	2492 (0.033)
verreauxi	2	3370 (0.023)	#SAKNAS!	1612 (0.066)
vertebrate	2	3370 (0.023)	#SAKNAS!	1612 (0.066)
viable	2	3370 (0.023)	2293 (0.034)	#SAKNAS!
victims	2	3370 (0.023)	2293 (0.034)	2492 (0.033)
videofluorography	2	3370 (0.023)	2293 (0.034)	2492 (0.033)
vietnamese	2	3370 (0.023)	#SAKNAS!	2492 (0.033)
vision	2	3370 (0.023)	#SAKNAS!	#SAKNAS!
visiting	2	3370 (0.023)	#SAKNAS!	#SAKNAS!
visitors	2	3370 (0.023)	2293 (0.034)	#SAKNAS!

volumetric	2	3370 (0.023)	#SAKNAS!	1612 (0.066)
volvulus	2	3370 (0.023)	2293 (0.034)	2492 (0.033)
vulgaris	2	3370 (0.023)	#SAKNAS!	2492 (0.033)
vulnerable	2	3370 (0.023)	2293 (0.034)	2492 (0.033)
wales	2	3370 (0.023)	1457 (0.069)	#SAKNAS!
walking	2	3370 (0.023)	#SAKNAS!	2492 (0.033)
warming	2	3370 (0.023)	#SAKNAS!	#SAKNAS!
warning	2	3370 (0.023)	#SAKNAS!	2492 (0.033)
waves	2	3370 (0.023)	#SAKNAS!	#SAKNAS!
ways	2	3370 (0.023)	2293 (0.034)	2492 (0.033)
weakness	2	3370 (0.023)	2293 (0.034)	2492 (0.033)
wearable	2	3370 (0.023)	#SAKNAS!	#SAKNAS!
weather	2	3370 (0.023)	1457 (0.069)	#SAKNAS!
web	2	3370 (0.023)	1457 (0.069)	#SAKNAS!
weekly	2	3370 (0.023)	2293 (0.034)	2492 (0.033)
wehncke	2	3370 (0.023)	2293 (0.034)	2492 (0.033)
weighing	2	3370 (0.023)	2293 (0.034)	2492 (0.033)
weight-loss	2	3370 (0.023)	1457 (0.069)	#SAKNAS!
wellbeing	2	3370 (0.023)	#SAKNAS!	1612 (0.066)
wellness	2	3370 (0.023)	#SAKNAS!	2492 (0.033)
willingness	2	3370 (0.023)	#SAKNAS!	2492 (0.033)
wilson	2	3370 (0.023)	#SAKNAS!	2492 (0.033)
wisdom	2	3370 (0.023)	2293 (0.034)	2492 (0.033)
wistar	2	3370 (0.023)	#SAKNAS!	2492 (0.033)
wolves	2	3370 (0.023)	2293 (0.034)	#SAKNAS!
woody	2	3370 (0.023)	2293 (0.034)	#SAKNAS!
workplace	2	3370 (0.023)	#SAKNAS!	2492 (0.033)
workshops	2	3370 (0.023)	#SAKNAS!	1612 (0.066)
worries	2	3370 (0.023)	#SAKNAS!	#SAKNAS!
worse	2	3370 (0.023)	2293 (0.034)	2492 (0.033)
worsens	2	3370 (0.023)	#SAKNAS!	2492 (0.033)
xenopus	2	3370 (0.023)	2293 (0.034)	#SAKNAS!
xylem	2	3370 (0.023)	#SAKNAS!	1612 (0.066)
yakui	2	3370 (0.023)	#SAKNAS!	1612 (0.066)
yamagata	2	3370 (0.023)	#SAKNAS!	2492 (0.033)
year-old	2	3370 (0.023)	#SAKNAS!	2492 (0.033)
yeasts	2	3370 (0.023)	2293 (0.034)	2492 (0.033)
yesterday	2	3370 (0.023)	2293 (0.034)	2492 (0.033)
yoldia	2	3370 (0.023)	1457 (0.069)	#SAKNAS!
your	2	3370 (0.023)	2293 (0.034)	2492 (0.033)
youths	2	3370 (0.023)	1457 (0.069)	#SAKNAS!
yunnan	2	3370 (0.023)	#SAKNAS!	2492 (0.033)
yy	2	3370 (0.023)	2293 (0.034)	2492 (0.033)

zebra		2	3370 (0.023)	2293 (0.034)	2492 (0.033)
zebu		2	3370 (0.023)	#SAKNAS!	1612 (0.066)
zero-p		2	3370 (0.023)	#SAKNAS!	2492 (0.033)
zika		2	3370 (0.023)	#SAKNAS!	#SAKNAS!
zones		2	3370 (0.023)	#SAKNAS!	2492 (0.033)
zoo		2	3370 (0.023)	#SAKNAS!	2492 (0.033)
z-stents		2	3370 (0.023)	1457 (0.069)	#SAKNAS!
	0,9	1	5033 (0.011)	#SAKNAS!	2492 (0.033)
	1	1	5033 (0.011)	2293 (0.034)	#SAKNAS!
	2	1	3370 (0.023)	2293 (0.034)	#SAKNAS!
	3	1	5033 (0.011)	#SAKNAS!	2492 (0.033)
	264,7	1	5033 (0.011)	#SAKNAS!	#SAKNAS!
	1505	1	5033 (0.011)	#SAKNAS!	#SAKNAS!
	1512	1	5033 (0.011)	#SAKNAS!	#SAKNAS!
	1790	1	5033 (0.011)	#SAKNAS!	#SAKNAS!
	1794	1	5033 (0.011)	2293 (0.034)	#SAKNAS!
	1828	1	5033 (0.011)	#SAKNAS!	#SAKNAS!
	1833	1	5033 (0.011)	#SAKNAS!	#SAKNAS!
	1837	1	5033 (0.011)	2293 (0.034)	#SAKNAS!
	1841	1	5033 (0.011)	#SAKNAS!	#SAKNAS!
	1849	1	5033 (0.011)	#SAKNAS!	2492 (0.033)
	1931	1	5033 (0.011)	#SAKNAS!	2492 (0.033)
	1945	1	5033 (0.011)	2293 (0.034)	#SAKNAS!
	1956	1	5033 (0.011)	2293 (0.034)	#SAKNAS!
	1969	1	5033 (0.011)	2293 (0.034)	#SAKNAS!
	1971	1	5033 (0.011)	#SAKNAS!	2492 (0.033)
	1982	1	5033 (0.011)	#SAKNAS!	2492 (0.033)
	1988	1	5033 (0.011)	#SAKNAS!	2492 (0.033)
	1990	1	5033 (0.011)	#SAKNAS!	#SAKNAS!
	1991	1	5033 (0.011)	2293 (0.034)	#SAKNAS!
	1994	1	5033 (0.011)	2293 (0.034)	#SAKNAS!
	1998	1	5033 (0.011)	2293 (0.034)	#SAKNAS!
	2023	1	5033 (0.011)	#SAKNAS!	#SAKNAS!
	2024	1	5033 (0.011)	#SAKNAS!	#SAKNAS!
	2071	1	5033 (0.011)	2293 (0.034)	#SAKNAS!
	2131	1	5033 (0.011)	2293 (0.034)	#SAKNAS!
	2200	1	5033 (0.011)	#SAKNAS!	2492 (0.033)
	3063	1	5033 (0.011)	#SAKNAS!	2492 (0.033)
	3111	1	5033 (0.011)	#SAKNAS!	2492 (0.033)
	4061	1	5033 (0.011)	#SAKNAS!	2492 (0.033)
	4716	1	5033 (0.011)	#SAKNAS!	2492 (0.033)
	5943	1	5033 (0.011)	#SAKNAS!	#SAKNAS!
	7589	1	5033 (0.011)	#SAKNAS!	2492 (0.033)

	8020	1	5033 (0.011)	2293 (0.034)	#SAKNAS!
	12174	1	5033 (0.011)	#SAKNAS!	2492 (0.033)
	13998	1	5033 (0.011)	#SAKNAS!	2492 (0.033)
	18476	1	5033 (0.011)	2293 (0.034)	#SAKNAS!
	45660	1	5033 (0.011)	#SAKNAS!	#SAKNAS!
	45667	1	5033 (0.011)	#SAKNAS!	2492 (0.033)
	45674	1	5033 (0.011)	2293 (0.034)	#SAKNAS!
	45676	1	5033 (0.011)	#SAKNAS!	2492 (0.033)
	45692	1	5033 (0.011)	#SAKNAS!	2492 (0.033)
	45694	1	5033 (0.011)	2293 (0.034)	#SAKNAS!
	45717	1	5033 (0.011)	#SAKNAS!	#SAKNAS!
	45727	1	5033 (0.011)	#SAKNAS!	2492 (0.033)
	45752	1	5033 (0.011)	#SAKNAS!	2492 (0.033)
	45753	1	5033 (0.011)	2293 (0.034)	#SAKNAS!
	45783	1	5033 (0.011)	#SAKNAS!	2492 (0.033)
	45791	1	5033 (0.011)	#SAKNAS!	2492 (0.033)
	45796	1	5033 (0.011)	#SAKNAS!	2492 (0.033)
	45817	1	5033 (0.011)	#SAKNAS!	2492 (0.033)
	45847	1	5033 (0.011)	#SAKNAS!	2492 (0.033)
	45848	1	5033 (0.011)	#SAKNAS!	2492 (0.033)
	45852	1	5033 (0.011)	2293 (0.034)	#SAKNAS!
	45883	1	5033 (0.011)	#SAKNAS!	#SAKNAS!
	45910	1	5033 (0.011)	2293 (0.034)	#SAKNAS!
	45911	1	5033 (0.011)	#SAKNAS!	2492 (0.033)
	45923	1	5033 (0.011)	#SAKNAS!	#SAKNAS!
	45974	1	5033 (0.011)	2293 (0.034)	#SAKNAS!
	46010	1	5033 (0.011)	2293 (0.034)	#SAKNAS!
!goggin		1	5033 (0.011)	2293 (0.034)	#SAKNAS!
!kimble		1	5033 (0.011)	2293 (0.034)	#SAKNAS!
!patel		1	5033 (0.011)	2293 (0.034)	#SAKNAS!
!roseveare		1	5033 (0.011)	2293 (0.034)	#SAKNAS!
!shepherd		1	5033 (0.011)	2293 (0.034)	#SAKNAS!
!simmonds		1	5033 (0.011)	2293 (0.034)	#SAKNAS!
?□p63		1	5033 (0.011)	2293 (0.034)	#SAKNAS!
?⍰80		1	5033 (0.011)	2293 (0.034)	#SAKNAS!
0.5-year-old		1	5033 (0.011)	2293 (0.034)	#SAKNAS!
0-2		1	5033 (0.011)	#SAKNAS!	#SAKNAS!
0-5		1	5033 (0.011)	#SAKNAS!	#SAKNAS!
0-5-month-old		1	5033 (0.011)	#SAKNAS!	#SAKNAS!
1.5-4.5		1	5033 (0.011)	2293 (0.034)	#SAKNAS!
10-species		1	5033 (0.011)	2293 (0.034)	#SAKNAS!
10-y-old		1	5033 (0.011)	2293 (0.034)	#SAKNAS!
11c		1	5033 (0.011)	2293 (0.034)	#SAKNAS!

11-year-old	1	5033 (0.011)	2293 (0.034)	#SAKNAS!
11-years	1	5033 (0.011)	#SAKNAS!	2492 (0.033)
11斧-hydroxysteroid	1	5033 (0.011)	#SAKNAS!	2492 (0.033)
12th	1	5033 (0.011)	#SAKNAS!	#SAKNAS!
12-week	1	5033 (0.011)	#SAKNAS!	#SAKNAS!
12-year	1	5033 (0.011)	#SAKNAS!	2492 (0.033)
13-year	1	5033 (0.011)	#SAKNAS!	#SAKNAS!
1438g	1	5033 (0.011)	2293 (0.034)	#SAKNAS!
14-d	1	5033 (0.011)	#SAKNAS!	2492 (0.033)
14-year-old	1	5033 (0.011)	#SAKNAS!	#SAKNAS!
15-cm	1	5033 (0.011)	#SAKNAS!	2492 (0.033)
15-year-old	1	5033 (0.011)	#SAKNAS!	2492 (0.033)
15-year-olds	1	5033 (0.011)	#SAKNAS!	2492 (0.033)
16-year-olds	1	5033 (0.011)	#SAKNAS!	2492 (0.033)
17-47	1	5033 (0.011)	2293 (0.034)	#SAKNAS!
17-year	1	5033 (0.011)	#SAKNAS!	#SAKNAS!
17-year-old	1	5033 (0.011)	#SAKNAS!	2492 (0.033)
18-39	1	5033 (0.011)	#SAKNAS!	#SAKNAS!
18-74	1	5033 (0.011)	2293 (0.034)	#SAKNAS!
18-month	1	5033 (0.011)	#SAKNAS!	2492 (0.033)
18-year-olds	1	5033 (0.011)	#SAKNAS!	2492 (0.033)
1958-1970	1	5033 (0.011)	2293 (0.034)	#SAKNAS!
1989-1995	1	5033 (0.011)	2293 (0.034)	#SAKNAS!
1989-2001	1	5033 (0.011)	2293 (0.034)	#SAKNAS!
1999-2010	1	5033 (0.011)	#SAKNAS!	2492 (0.033)
1999-2014	1	5033 (0.011)	#SAKNAS!	2492 (0.033)
1999-2020	1	5033 (0.011)	#SAKNAS!	#SAKNAS!
19-year-old	1	5033 (0.011)	#SAKNAS!	2492 (0.033)
19-years-olds	1	5033 (0.011)	2293 (0.034)	#SAKNAS!
1-day-old	1	5033 (0.011)	2293 (0.034)	#SAKNAS!
1-deoxynojirimycin	1	5033 (0.011)	#SAKNAS!	2492 (0.033)
1h-nmr	1	5033 (0.011)	#SAKNAS!	#SAKNAS!
1-y	1	5033 (0.011)	#SAKNAS!	#SAKNAS!
1-year-olds	1	5033 (0.011)	#SAKNAS!	2492 (0.033)
2.5-year	1	5033 (0.011)	#SAKNAS!	2492 (0.033)
2000-2014	1	5033 (0.011)	#SAKNAS!	2492 (0.033)
2001-2002	1	5033 (0.011)	2293 (0.034)	#SAKNAS!
2001-2004	1	5033 (0.011)	#SAKNAS!	#SAKNAS!
2002-2015	1	5033 (0.011)	#SAKNAS!	2492 (0.033)
2003-04	1	5033 (0.011)	2293 (0.034)	#SAKNAS!
2003-2012	1	5033 (0.011)	#SAKNAS!	2492 (0.033)
2003-2013	1	5033 (0.011)	#SAKNAS!	2492 (0.033)
2003-2016	1	5033 (0.011)	#SAKNAS!	2492 (0.033)

2003-2018	1	5033 (0.011)	#SAKNAS!	#SAKNAS!
2005-08	1	5033 (0.011)	2293 (0.034)	#SAKNAS!
2005-2008	1	5033 (0.011)	#SAKNAS!	2492 (0.033)
2005-2010	1	5033 (0.011)	#SAKNAS!	2492 (0.033)
2005-2018	1	5033 (0.011)	#SAKNAS!	#SAKNAS!
2005-2020	1	5033 (0.011)	#SAKNAS!	#SAKNAS!
2007-2014	1	5033 (0.011)	#SAKNAS!	2492 (0.033)
2007-2021	1	5033 (0.011)	#SAKNAS!	#SAKNAS!
2008-2015	1	5033 (0.011)	#SAKNAS!	2492 (0.033)
2009-2016	1	5033 (0.011)	#SAKNAS!	#SAKNAS!
2009-2018	1	5033 (0.011)	#SAKNAS!	#SAKNAS!
2011-16	1	5033 (0.011)	#SAKNAS!	#SAKNAS!
2011-2020	1	5033 (0.011)	#SAKNAS!	#SAKNAS!
2012-2015	1	5033 (0.011)	#SAKNAS!	#SAKNAS!
2012-2018	1	5033 (0.011)	#SAKNAS!	#SAKNAS!
2012-2020	1	5033 (0.011)	#SAKNAS!	#SAKNAS!
2013-2016	1	5033 (0.011)	#SAKNAS!	#SAKNAS!
2014-2015	1	5033 (0.011)	#SAKNAS!	#SAKNAS!
2015-2020	1	5033 (0.011)	#SAKNAS!	#SAKNAS!
2016-2019	1	5033 (0.011)	#SAKNAS!	#SAKNAS!
2018-2028	1	5033 (0.011)	#SAKNAS!	2492 (0.033)
2019-2021	1	5033 (0.011)	#SAKNAS!	#SAKNAS!
2021-2022	1	5033 (0.011)	#SAKNAS!	#SAKNAS!
20-79	1	5033 (0.011)	2293 (0.034)	#SAKNAS!
21-64	1	5033 (0.011)	#SAKNAS!	2492 (0.033)
21-mm	1	5033 (0.011)	2293 (0.034)	#SAKNAS!
22q11	1	5033 (0.011)	#SAKNAS!	2492 (0.033)
22q11.2	1	5033 (0.011)	#SAKNAS!	2492 (0.033)
23-hour	1	5033 (0.011)	#SAKNAS!	2492 (0.033)
24-h	1	5033 (0.011)	#SAKNAS!	2492 (0.033)
24-month-old	1	5033 (0.011)	2293 (0.034)	#SAKNAS!
25-44	1	5033 (0.011)	#SAKNAS!	#SAKNAS!
25-79	1	5033 (0.011)	#SAKNAS!	2492 (0.033)
25-year	1	5033 (0.011)	2293 (0.034)	#SAKNAS!
27-year	1	5033 (0.011)	#SAKNAS!	#SAKNAS!
2a	1	5033 (0.011)	2293 (0.034)	#SAKNAS!
2-arachidonoylglycerol	1	5033 (0.011)	#SAKNAS!	2492 (0.033)
2h	1	5033 (0.011)	#SAKNAS!	2492 (0.033)
2nd	1	5033 (0.011)	#SAKNAS!	2492 (0.033)
30-42	1	5033 (0.011)	2293 (0.034)	#SAKNAS!
30-and	1	5033 (0.011)	#SAKNAS!	#SAKNAS!
3-4-year-old	1	5033 (0.011)	2293 (0.034)	#SAKNAS!
35-5	1	5033 (0.011)	#SAKNAS!	2492 (0.033)

35-54	1	5033 (0.011)	#SAKNAS!	2492 (0.033)
35-79	1	5033 (0.011)	#SAKNAS!	2492 (0.033)
36-month-old	1	5033 (0.011)	#SAKNAS!	2492 (0.033)
37-year	1	5033 (0.011)	#SAKNAS!	2492 (0.033)
3-and	1	5033 (0.011)	#SAKNAS!	2492 (0.033)
3dct	1	5033 (0.011)	#SAKNAS!	#SAKNAS!
3-dimensional	1	5033 (0.011)	#SAKNAS!	2492 (0.033)
3d-printable	1	5033 (0.011)	#SAKNAS!	#SAKNAS!
3-level	1	5033 (0.011)	#SAKNAS!	#SAKNAS!
3-week	1	5033 (0.011)	2293 (0.034)	#SAKNAS!
3-weekly	1	5033 (0.011)	#SAKNAS!	#SAKNAS!
3xtg-ad	1	5033 (0.011)	#SAKNAS!	2492 (0.033)
4??-tetrabromodiphenyl	1	5033 (0.011)	#SAKNAS!	#SAKNAS!
40years	1	5033 (0.011)	#SAKNAS!	2492 (0.033)
4-24-month-old	1	5033 (0.011)	2293 (0.034)	#SAKNAS!
44-year-old	1	5033 (0.011)	2293 (0.034)	#SAKNAS!
45-80	1	5033 (0.011)	#SAKNAS!	#SAKNAS!
472-patient	1	5033 (0.011)	#SAKNAS!	2492 (0.033)
4-diaminobutane	1	5033 (0.011)	2293 (0.034)	#SAKNAS!
4-month-old	1	5033 (0.011)	2293 (0.034)	#SAKNAS!
4th-instar	1	5033 (0.011)	2293 (0.034)	#SAKNAS!
4-week	1	5033 (0.011)	#SAKNAS!	2492 (0.033)
50-to	1	5033 (0.011)	#SAKNAS!	#SAKNAS!
5-11years	1	5033 (0.011)	#SAKNAS!	2492 (0.033)
55-79	1	5033 (0.011)	#SAKNAS!	2492 (0.033)
5-7-year-old	1	5033 (0.011)	2293 (0.034)	#SAKNAS!
5-ag	1	5033 (0.011)	#SAKNAS!	#SAKNAS!
5-dimethyl-4-hydroxy-3	1	5033 (0.011)	#SAKNAS!	2492 (0.033)
5-fluorouracil-based	1	5033 (0.011)	2293 (0.034)	#SAKNAS!
5-fu	1	5033 (0.011)	2293 (0.034)	#SAKNAS!
5-ht1a	1	5033 (0.011)	2293 (0.034)	#SAKNAS!
5-ht1b	1	5033 (0.011)	2293 (0.034)	#SAKNAS!
5-ht6	1	5033 (0.011)	#SAKNAS!	2492 (0.033)
5httlpr	1	5033 (0.011)	2293 (0.034)	#SAKNAS!
5-hydroxyindoleacetic	1	5033 (0.011)	2293 (0.034)	#SAKNAS!
5-item	1	5033 (0.011)	#SAKNAS!	#SAKNAS!
5-y	1	5033 (0.011)	2293 (0.034)	#SAKNAS!
6.5-y-old	1	5033 (0.011)	2293 (0.034)	#SAKNAS!
60years	1	5033 (0.011)	2293 (0.034)	#SAKNAS!
6-12-year-old	1	5033 (0.011)	#SAKNAS!	#SAKNAS!
6-24-month-old	1	5033 (0.011)	2293 (0.034)	#SAKNAS!
6-36-month-old	1	5033 (0.011)	#SAKNAS!	2492 (0.033)
6-36months	1	5033 (0.011)	#SAKNAS!	2492 (0.033)

65-	1	5033 (0.011)	2293 (0.034)	#SAKNAS!
65+age	1	5033 (0.011)	#SAKNAS!	#SAKNAS!
653-7	1	5033 (0.011)	2293 (0.034)	#SAKNAS!
65-74-year-old	1	5033 (0.011)	2293 (0.034)	#SAKNAS!
6700-5600	1	5033 (0.011)	2293 (0.034)	#SAKNAS!
6-7-year-old	1	5033 (0.011)	2293 (0.034)	#SAKNAS!
6-8months	1	5033 (0.011)	#SAKNAS!	2492 (0.033)
68-year-old	1	5033 (0.011)	2293 (0.034)	#SAKNAS!
6-month-old	1	5033 (0.011)	#SAKNAS!	#SAKNAS!
6-months	1	5033 (0.011)	#SAKNAS!	2492 (0.033)
6-n-propylthiouracil	1	5033 (0.011)	2293 (0.034)	#SAKNAS!
6th-3rd	1	5033 (0.011)	2293 (0.034)	#SAKNAS!
6th-instar	1	5033 (0.011)	2293 (0.034)	#SAKNAS!
6-week	1	5033 (0.011)	#SAKNAS!	#SAKNAS!
6-year-olds	1	5033 (0.011)	#SAKNAS!	#SAKNAS!
75-year-old	1	5033 (0.011)	2293 (0.034)	#SAKNAS!
75-year-olds	1	5033 (0.011)	2293 (0.034)	#SAKNAS!
79-year-old	1	5033 (0.011)	#SAKNAS!	2492 (0.033)
7-9-year-old	1	5033 (0.011)	#SAKNAS!	#SAKNAS!
80-year-olds	1	5033 (0.011)	#SAKNAS!	#SAKNAS!
82-to	1	5033 (0.011)	#SAKNAS!	2492 (0.033)
84-year-old	1	5033 (0.011)	#SAKNAS!	2492 (0.033)
8-tetrachlorodibenzo-p-dioxin	1	5033 (0.011)	2293 (0.034)	#SAKNAS!
8-week	1	5033 (0.011)	2293 (0.034)	#SAKNAS!
90-day	1	5033 (0.011)	#SAKNAS!	#SAKNAS!
97-09	1	5033 (0.011)	2293 (0.034)	#SAKNAS!
9-and	1	5033 (0.011)	#SAKNAS!	#SAKNAS!
9-month	1	5033 (0.011)	#SAKNAS!	2492 (0.033)
9p	1	5033 (0.011)	2293 (0.034)	#SAKNAS!
9-year	1	5033 (0.011)	#SAKNAS!	#SAKNAS!
9-year-old	1	5033 (0.011)	#SAKNAS!	2492 (0.033)
a-1	1	5033 (0.011)	2293 (0.034)	#SAKNAS!
a10	1	5033 (0.011)	#SAKNAS!	#SAKNAS!
a1c	1	5033 (0.011)	#SAKNAS!	#SAKNAS!
a3669g	1	5033 (0.011)	#SAKNAS!	2492 (0.033)
a4	1	5033 (0.011)	#SAKNAS!	2492 (0.033)
aafp	1	5033 (0.011)	#SAKNAS!	#SAKNAS!
aaha	1	5033 (0.011)	#SAKNAS!	#SAKNAS!
aalge	1	5033 (0.011)	2293 (0.034)	#SAKNAS!
aav-mediated	1	5033 (0.011)	#SAKNAS!	2492 (0.033)
abadia	1	5033 (0.011)	#SAKNAS!	#SAKNAS!
abatus	1	5033 (0.011)	#SAKNAS!	#SAKNAS!
abdominis	1	5033 (0.011)	2293 (0.034)	#SAKNAS!

abdulrahman	1	5033 (0.011)	#SAKNAS!	2492 (0.033)
abeparvovec	1	5033 (0.011)	#SAKNAS!	#SAKNAS!
aberrant	1	5033 (0.011)	#SAKNAS!	#SAKNAS!
abha	1	5033 (0.011)	2293 (0.034)	#SAKNAS!
abies	1	5033 (0.011)	2293 (0.034)	#SAKNAS!
abilities	1	5033 (0.011)	2293 (0.034)	#SAKNAS!
abiotic	1	5033 (0.011)	#SAKNAS!	2492 (0.033)
aborigine	1	5033 (0.011)	2293 (0.034)	#SAKNAS!
about-child	1	5033 (0.011)	#SAKNAS!	#SAKNAS!
abrupt	1	5033 (0.011)	#SAKNAS!	2492 (0.033)
abscesses	1	5033 (0.011)	#SAKNAS!	2492 (0.033)
abscisic	1	5033 (0.011)	#SAKNAS!	2492 (0.033)
absent	1	5033 (0.011)	2293 (0.034)	#SAKNAS!
absi	1	5033 (0.011)	#SAKNAS!	#SAKNAS!
absorbable	1	5033 (0.011)	#SAKNAS!	#SAKNAS!
absorptiometry	1	5033 (0.011)	2293 (0.034)	#SAKNAS!
absorptive	1	5033 (0.011)	2293 (0.034)	#SAKNAS!
abuse-deterrent	1	5033 (0.011)	#SAKNAS!	2492 (0.033)
academic	1	5033 (0.011)	#SAKNAS!	#SAKNAS!
accelerates	1	5033 (0.011)	#SAKNAS!	2492 (0.033)
accelerometry	1	5033 (0.011)	2293 (0.034)	#SAKNAS!
accent>	1	5033 (0.011)	#SAKNAS!	#SAKNAS!
accessing	1	5033 (0.011)	#SAKNAS!	2492 (0.033)
accessions	1	5033 (0.011)	#SAKNAS!	#SAKNAS!
access-time	1	5033 (0.011)	#SAKNAS!	#SAKNAS!
accident	1	5033 (0.011)	2293 (0.034)	#SAKNAS!
accidental	1	5033 (0.011)	#SAKNAS!	2492 (0.033)
accidents	1	5033 (0.011)	2293 (0.034)	#SAKNAS!
account	1	5033 (0.011)	#SAKNAS!	#SAKNAS!
accountable	1	5033 (0.011)	#SAKNAS!	2492 (0.033)
accumulate	1	5033 (0.011)	2293 (0.034)	#SAKNAS!
acetylcholine	1	5033 (0.011)	#SAKNAS!	#SAKNAS!
achievers	1	5033 (0.011)	2293 (0.034)	#SAKNAS!
acholi	1	5033 (0.011)	#SAKNAS!	2492 (0.033)
acid-based	1	5033 (0.011)	2293 (0.034)	#SAKNAS!
acid-deprived	1	5033 (0.011)	#SAKNAS!	2492 (0.033)
acid-fortified	1	5033 (0.011)	#SAKNAS!	#SAKNAS!
acid-imbalanced	1	5033 (0.011)	2293 (0.034)	#SAKNAS!
acidity	1	5033 (0.011)	#SAKNAS!	2492 (0.033)
acidophilus	1	5033 (0.011)	#SAKNAS!	#SAKNAS!
aciduria	1	5033 (0.011)	2293 (0.034)	#SAKNAS!
acinar	1	5033 (0.011)	2293 (0.034)	#SAKNAS!
acorn	1	5033 (0.011)	#SAKNAS!	2492 (0.033)

acoustic	1	5033 (0.011)	#SAKNAS!	#SAKNAS!
acpas	1	5033 (0.011)	#SAKNAS!	2492 (0.033)
acquires	1	5033 (0.011)	2293 (0.034)	#SAKNAS!
acrididae	1	5033 (0.011)	2293 (0.034)	#SAKNAS!
acromegaly	1	5033 (0.011)	#SAKNAS!	#SAKNAS!
actinobacillus-actinomycetemcomitans	1	5033 (0.011)	2293 (0.034)	#SAKNAS!
actinopterygii	1	5033 (0.011)	2293 (0.034)	#SAKNAS!
activating	1	5033 (0.011)	2293 (0.034)	#SAKNAS!
actors	1	5033 (0.011)	#SAKNAS!	2492 (0.033)
acts	1	5033 (0.011)	#SAKNAS!	#SAKNAS!
actual	1	5033 (0.011)	#SAKNAS!	2492 (0.033)
actualisation	1	5033 (0.011)	#SAKNAS!	2492 (0.033)
actuator	1	5033 (0.011)	#SAKNAS!	2492 (0.033)
aculeatus	1	5033 (0.011)	2293 (0.034)	#SAKNAS!
acute-care	1	5033 (0.011)	#SAKNAS!	2492 (0.033)
acute-leukemia	1	5033 (0.011)	2293 (0.034)	#SAKNAS!
acute-phase	1	5033 (0.011)	2293 (0.034)	#SAKNAS!
acute-stroke	1	5033 (0.011)	2293 (0.034)	#SAKNAS!
adapt	1	5033 (0.011)	#SAKNAS!	2492 (0.033)
adapta癟瓊o	1	5033 (0.011)	#SAKNAS!	#SAKNAS!
adapting	1	5033 (0.011)	#SAKNAS!	2492 (0.033)
adaptively	1	5033 (0.011)	#SAKNAS!	#SAKNAS!
adcabc	1	5033 (0.011)	#SAKNAS!	#SAKNAS!
address	1	5033 (0.011)	#SAKNAS!	2492 (0.033)
adduction	1	5033 (0.011)	2293 (0.034)	#SAKNAS!
a-deficient	1	5033 (0.011)	2293 (0.034)	#SAKNAS!
adelie	1	5033 (0.011)	#SAKNAS!	#SAKNAS!
adeno-associated	1	5033 (0.011)	2293 (0.034)	#SAKNAS!
adenoma	1	5033 (0.011)	#SAKNAS!	2492 (0.033)
adenotonsillar	1	5033 (0.011)	#SAKNAS!	#SAKNAS!
adenylate	1	5033 (0.011)	#SAKNAS!	#SAKNAS!
adhesive	1	5033 (0.011)	#SAKNAS!	2492 (0.033)
adipocyte	1	5033 (0.011)	2293 (0.034)	#SAKNAS!
adipocytes	1	5033 (0.011)	#SAKNAS!	2492 (0.033)
adipoq-rs1501299	1	5033 (0.011)	#SAKNAS!	2492 (0.033)
adipostat	1	5033 (0.011)	2293 (0.034)	#SAKNAS!
adipsin	1	5033 (0.011)	#SAKNAS!	#SAKNAS!
adjustments	1	5033 (0.011)	#SAKNAS!	2492 (0.033)
adjusts	1	5033 (0.011)	#SAKNAS!	#SAKNAS!
adl	1	5033 (0.011)	#SAKNAS!	2492 (0.033)
adls	1	5033 (0.011)	#SAKNAS!	2492 (0.033)
administer	1	5033 (0.011)	#SAKNAS!	#SAKNAS!
adolescents-a	1	5033 (0.011)	#SAKNAS!	#SAKNAS!

adolescentsresults	1 5033 (0.011)	#SAKNAS!	2492 (0.033)
adrenal	1 5033 (0.011)	#SAKNAS!	#SAKNAS!
adrenomedullin	1 5033 (0.011)	#SAKNAS!	#SAKNAS!
adropin	1 5033 (0.011)	#SAKNAS!	2492 (0.033)
adsorbents	1 5033 (0.011)	#SAKNAS!	#SAKNAS!
adult-onset	1 5033 (0.011)	#SAKNAS!	#SAKNAS!
adults-a	1 5033 (0.011)	#SAKNAS!	#SAKNAS!
adults-evaluation	1 5033 (0.011)	#SAKNAS!	2492 (0.033)
advance	1 5033 (0.011)	#SAKNAS!	#SAKNAS!
advantages	1 5033 (0.011)	2293 (0.034)	#SAKNAS!
advent	1 5033 (0.011)	2293 (0.034)	#SAKNAS!
adverse-effects	1 5033 (0.011)	2293 (0.034)	#SAKNAS!
advertising	1 5033 (0.011)	#SAKNAS!	2492 (0.033)
advisability	1 5033 (0.011)	#SAKNAS!	#SAKNAS!
advise	1 5033 (0.011)	2293 (0.034)	#SAKNAS!
advocating	1 5033 (0.011)	#SAKNAS!	2492 (0.033)
aegean	1 5033 (0.011)	#SAKNAS!	#SAKNAS!
aerobic	1 5033 (0.011)	#SAKNAS!	#SAKNAS!
aeruginosa	1 5033 (0.011)	#SAKNAS!	#SAKNAS!
aetiology	1 5033 (0.011)	2293 (0.034)	#SAKNAS!
afferents	1 5033 (0.011)	#SAKNAS!	2492 (0.033)
affinis	1 5033 (0.011)	2293 (0.034)	#SAKNAS!
affluent	1 5033 (0.011)	#SAKNAS!	2492 (0.033)
afford	1 5033 (0.011)	#SAKNAS!	#SAKNAS!
affordability	1 5033 (0.011)	#SAKNAS!	2492 (0.033)
affordable	1 5033 (0.011)	#SAKNAS!	#SAKNAS!
afghan	1 5033 (0.011)	#SAKNAS!	#SAKNAS!
afghanistan	1 5033 (0.011)	#SAKNAS!	2492 (0.033)
african-americans	1 5033 (0.011)	2293 (0.034)	#SAKNAS!
agaricus	1 5033 (0.011)	#SAKNAS!	#SAKNAS!
age-at-death	1 5033 (0.011)	2293 (0.034)	#SAKNAS!
aged-care	1 5033 (0.011)	#SAKNAS!	#SAKNAS!
aged-children	1 5033 (0.011)	#SAKNAS!	#SAKNAS!
age-dependent	1 5033 (0.011)	2293 (0.034)	#SAKNAS!
age-friendly	1 5033 (0.011)	#SAKNAS!	#SAKNAS!
age-groups	1 5033 (0.011)	2293 (0.034)	#SAKNAS!
age-independent	1 5033 (0.011)	2293 (0.034)	#SAKNAS!
age-period-cohort	1 5033 (0.011)	#SAKNAS!	2492 (0.033)
age-when	1 5033 (0.011)	#SAKNAS!	#SAKNAS!
aggravate	1 5033 (0.011)	#SAKNAS!	2492 (0.033)
aggravates	1 5033 (0.011)	#SAKNAS!	2492 (0.033)
aggravation	1 5033 (0.011)	2293 (0.034)	#SAKNAS!
aging-a	1 5033 (0.011)	#SAKNAS!	2492 (0.033)

agonists	1	5033 (0.011)	#SAKNAS!	2492 (0.033)
agouti	1	5033 (0.011)	2293 (0.034)	#SAKNAS!
agouti-related	1	5033 (0.011)	#SAKNAS!	2492 (0.033)
agrestis	1	5033 (0.011)	2293 (0.034)	#SAKNAS!
agribusiness	1	5033 (0.011)	#SAKNAS!	#SAKNAS!
agro-ecological	1	5033 (0.011)	2293 (0.034)	#SAKNAS!
a-hei	1	5033 (0.011)	#SAKNAS!	#SAKNAS!
ahli	1	5033 (0.011)	2293 (0.034)	#SAKNAS!
ai	1	5033 (0.011)	2293 (0.034)	#SAKNAS!
aileu	1	5033 (0.011)	#SAKNAS!	2492 (0.033)
ailuropoda	1	5033 (0.011)	2293 (0.034)	#SAKNAS!
aiming	1	5033 (0.011)	#SAKNAS!	2492 (0.033)
aiptasia	1	5033 (0.011)	#SAKNAS!	#SAKNAS!
ait	1	5033 (0.011)	2293 (0.034)	#SAKNAS!
al	1	5033 (0.011)	#SAKNAS!	#SAKNAS!
alagille	1	5033 (0.011)	2293 (0.034)	#SAKNAS!
al-ahsa	1	5033 (0.011)	#SAKNAS!	#SAKNAS!
alarm	1	5033 (0.011)	2293 (0.034)	#SAKNAS!
albanian	1	5033 (0.011)	#SAKNAS!	#SAKNAS!
albatrosses	1	5033 (0.011)	2293 (0.034)	#SAKNAS!
alberta	1	5033 (0.011)	#SAKNAS!	#SAKNAS!
albocinereus	1	5033 (0.011)	2293 (0.034)	#SAKNAS!
albuminuria	1	5033 (0.011)	#SAKNAS!	2492 (0.033)
alcohol-dependent	1	5033 (0.011)	#SAKNAS!	2492 (0.033)
alcoholic	1	5033 (0.011)	#SAKNAS!	#SAKNAS!
aldehyde	1	5033 (0.011)	#SAKNAS!	#SAKNAS!
aldolase	1	5033 (0.011)	2293 (0.034)	#SAKNAS!
alectoris	1	5033 (0.011)	#SAKNAS!	#SAKNAS!
alegranza	1	5033 (0.011)	2293 (0.034)	#SAKNAS!
alegre	1	5033 (0.011)	2293 (0.034)	#SAKNAS!
alendronate	1	5033 (0.011)	2293 (0.034)	#SAKNAS!
alerts	1	5033 (0.011)	#SAKNAS!	2492 (0.033)
alessandro	1	5033 (0.011)	#SAKNAS!	#SAKNAS!
alexandri	1	5033 (0.011)	#SAKNAS!	2492 (0.033)
alfalfa-pellet	1	5033 (0.011)	2293 (0.034)	#SAKNAS!
alfalfa-ryegrass	1	5033 (0.011)	#SAKNAS!	#SAKNAS!
algae-herbivore	1	5033 (0.011)	#SAKNAS!	#SAKNAS!
alginates	1	5033 (0.011)	2293 (0.034)	#SAKNAS!
algirus	1	5033 (0.011)	#SAKNAS!	2492 (0.033)
algivore	1	5033 (0.011)	2293 (0.034)	#SAKNAS!
aliado	1	5033 (0.011)	#SAKNAS!	#SAKNAS!
alien	1	5033 (0.011)	2293 (0.034)	#SAKNAS!
alike	1	5033 (0.011)	2293 (0.034)	#SAKNAS!

alimentation	1	5033 (0.011)	#SAKNAS!	2492 (0.033)
alkaline-phosphatases	1	5033 (0.011)	2293 (0.034)	#SAKNAS!
alkaloids	1	5033 (0.011)	2293 (0.034)	#SAKNAS!
alkyl	1	5033 (0.011)	#SAKNAS!	2492 (0.033)
allelic	1	5033 (0.011)	#SAKNAS!	2492 (0.033)
allelochemical	1	5033 (0.011)	2293 (0.034)	#SAKNAS!
allelochemicals	1	5033 (0.011)	2293 (0.034)	#SAKNAS!
allergies	1	5033 (0.011)	#SAKNAS!	#SAKNAS!
allergy	1	5033 (0.011)	2293 (0.034)	#SAKNAS!
alleviate	1	5033 (0.011)	#SAKNAS!	#SAKNAS!
alleviating	1	5033 (0.011)	#SAKNAS!	2492 (0.033)
alliance	1	5033 (0.011)	#SAKNAS!	#SAKNAS!
allogeneic	1	5033 (0.011)	2293 (0.034)	#SAKNAS!
allosucking	1	5033 (0.011)	2293 (0.034)	#SAKNAS!
allowance	1	5033 (0.011)	#SAKNAS!	2492 (0.033)
alluvial	1	5033 (0.011)	2293 (0.034)	#SAKNAS!
almada-seixal	1	5033 (0.011)	#SAKNAS!	2492 (0.033)
almonds	1	5033 (0.011)	2293 (0.034)	#SAKNAS!
alosa	1	5033 (0.011)	#SAKNAS!	2492 (0.033)
alpelisib-related	1	5033 (0.011)	#SAKNAS!	#SAKNAS!
alpha-amylase	1	5033 (0.011)	2293 (0.034)	#SAKNAS!
alpha-ketoglutarate	1	5033 (0.011)	2293 (0.034)	#SAKNAS!
alpha-melanocyte-stimulating	1	5033 (0.011)	#SAKNAS!	2492 (0.033)
alpha-tocopherol	1	5033 (0.011)	#SAKNAS!	#SAKNAS!
alpinus	1	5033 (0.011)	2293 (0.034)	#SAKNAS!
alspac	1	5033 (0.011)	2293 (0.034)	#SAKNAS!
alternation	1	5033 (0.011)	#SAKNAS!	2492 (0.033)
alumni	1	5033 (0.011)	#SAKNAS!	#SAKNAS!
amarali	1	5033 (0.011)	#SAKNAS!	#SAKNAS!
amazona	1	5033 (0.011)	2293 (0.034)	#SAKNAS!
amazonas	1	5033 (0.011)	#SAKNAS!	#SAKNAS!
amazons	1	5033 (0.011)	2293 (0.034)	#SAKNAS!
ambidirectional	1	5033 (0.011)	#SAKNAS!	#SAKNAS!
ambulation	1	5033 (0.011)	#SAKNAS!	#SAKNAS!
ambystomatid	1	5033 (0.011)	#SAKNAS!	#SAKNAS!
ameerega	1	5033 (0.011)	#SAKNAS!	#SAKNAS!
amel	1	5033 (0.011)	#SAKNAS!	2492 (0.033)
ameliorate	1	5033 (0.011)	#SAKNAS!	#SAKNAS!
ameliorates	1	5033 (0.011)	#SAKNAS!	2492 (0.033)
amenorrhea	1	5033 (0.011)	2293 (0.034)	#SAKNAS!
amid	1	5033 (0.011)	#SAKNAS!	#SAKNAS!
amifostine	1	5033 (0.011)	2293 (0.034)	#SAKNAS!
amino-acid	1	5033 (0.011)	2293 (0.034)	#SAKNAS!

aminopeptidase	1	5033 (0.011)	2293 (0.034)	#SAKNAS!
ammonia	1	5033 (0.011)	2293 (0.034)	#SAKNAS!
amp	1	5033 (0.011)	#SAKNAS!	2492 (0.033)
amparafaravola	1	5033 (0.011)	#SAKNAS!	#SAKNAS!
amphioctopus	1	5033 (0.011)	#SAKNAS!	#SAKNAS!
amphiura	1	5033 (0.011)	2293 (0.034)	#SAKNAS!
ampk	1	5033 (0.011)	#SAKNAS!	#SAKNAS!
ampk-mtor	1	5033 (0.011)	#SAKNAS!	#SAKNAS!
amsan	1	5033 (0.011)	#SAKNAS!	#SAKNAS!
amur	1	5033 (0.011)	#SAKNAS!	#SAKNAS!
amygdalar	1	5033 (0.011)	2293 (0.034)	#SAKNAS!
anabas	1	5033 (0.011)	#SAKNAS!	#SAKNAS!
anacardiaceae	1	5033 (0.011)	2293 (0.034)	#SAKNAS!
anaemia	1	5033 (0.011)	#SAKNAS!	#SAKNAS!
anaesthesia	1	5033 (0.011)	#SAKNAS!	2492 (0.033)
anahita	1	5033 (0.011)	2293 (0.034)	#SAKNAS!
anal	1	5033 (0.011)	#SAKNAS!	#SAKNAS!
anality	1	5033 (0.011)	2293 (0.034)	#SAKNAS!
analysing	1	5033 (0.011)	#SAKNAS!	2492 (0.033)
analysis???	1	5033 (0.011)	#SAKNAS!	#SAKNAS!
analytic	1	5033 (0.011)	#SAKNAS!	#SAKNAS!
analytics	1	5033 (0.011)	#SAKNAS!	#SAKNAS!
analyze	1	5033 (0.011)	#SAKNAS!	#SAKNAS!
analyzes	1	5033 (0.011)	#SAKNAS!	2492 (0.033)
anaplerosis	1	5033 (0.011)	#SAKNAS!	2492 (0.033)
anatinus	1	5033 (0.011)	2293 (0.034)	#SAKNAS!
anatolia	1	5033 (0.011)	#SAKNAS!	#SAKNAS!
anchor-based	1	5033 (0.011)	#SAKNAS!	#SAKNAS!
ancillary	1	5033 (0.011)	2293 (0.034)	#SAKNAS!
and-9	1	5033 (0.011)	2293 (0.034)	#SAKNAS!
anderson	1	5033 (0.011)	#SAKNAS!	#SAKNAS!
andes	1	5033 (0.011)	2293 (0.034)	#SAKNAS!
andrei	1	5033 (0.011)	#SAKNAS!	2492 (0.033)
anelosimus	1	5033 (0.011)	2293 (0.034)	#SAKNAS!
anesthetic	1	5033 (0.011)	2293 (0.034)	#SAKNAS!
aneurogeriatric	1	5033 (0.011)	#SAKNAS!	2492 (0.033)
aneurysm	1	5033 (0.011)	#SAKNAS!	2492 (0.033)
anganwadi	1	5033 (0.011)	2293 (0.034)	#SAKNAS!
angeles	1	5033 (0.011)	#SAKNAS!	2492 (0.033)
angiography	1	5033 (0.011)	#SAKNAS!	2492 (0.033)
angioplasty	1	5033 (0.011)	2293 (0.034)	#SAKNAS!
anguilla	1	5033 (0.011)	2293 (0.034)	#SAKNAS!
angular	1	5033 (0.011)	2293 (0.034)	#SAKNAS!

anhidrosis	1	5033 (0.011)	2293 (0.034)	#SAKNAS!
anhydrobiosis	1	5033 (0.011)	#SAKNAS!	#SAKNAS!
anhydrobiotic	1	5033 (0.011)	#SAKNAS!	#SAKNAS!
anin	1	5033 (0.011)	#SAKNAS!	2492 (0.033)
anisotropic	1	5033 (0.011)	2293 (0.034)	#SAKNAS!
anka	1	5033 (0.011)	#SAKNAS!	#SAKNAS!
ann	1	5033 (0.011)	#SAKNAS!	#SAKNAS!
anna	1	5033 (0.011)	#SAKNAS!	#SAKNAS!
annamensis	1	5033 (0.011)	#SAKNAS!	2492 (0.033)
annelida	1	5033 (0.011)	2293 (0.034)	#SAKNAS!
annual	1	5033 (0.011)	2293 (0.034)	#SAKNAS!
anomalies	1	5033 (0.011)	#SAKNAS!	#SAKNAS!
anomaloglossus	1	5033 (0.011)	2293 (0.034)	#SAKNAS!
anomura	1	5033 (0.011)	2293 (0.034)	#SAKNAS!
anorexia-cachexia	1	5033 (0.011)	#SAKNAS!	2492 (0.033)
anorexia-nervosa	1	5033 (0.011)	2293 (0.034)	#SAKNAS!
another	1	5033 (0.011)	#SAKNAS!	2492 (0.033)
anrs	1	5033 (0.011)	#SAKNAS!	2492 (0.033)
anser-anser	1	5033 (0.011)	2293 (0.034)	#SAKNAS!
antacid	1	5033 (0.011)	#SAKNAS!	#SAKNAS!
antagonists	1	5033 (0.011)	2293 (0.034)	#SAKNAS!
ant-eating	1	5033 (0.011)	2293 (0.034)	#SAKNAS!
antegrade-retrograde	1	5033 (0.011)	#SAKNAS!	#SAKNAS!
antenatal	1	5033 (0.011)	#SAKNAS!	#SAKNAS!
antennal	1	5033 (0.011)	#SAKNAS!	2492 (0.033)
anterolateral	1	5033 (0.011)	#SAKNAS!	#SAKNAS!
antheraea	1	5033 (0.011)	2293 (0.034)	#SAKNAS!
anthranilic	1	5033 (0.011)	#SAKNAS!	#SAKNAS!
anthropological	1	5033 (0.011)	#SAKNAS!	#SAKNAS!
anthropometrics	1	5033 (0.011)	#SAKNAS!	#SAKNAS!
anti-apolipoprotein	1	5033 (0.011)	2293 (0.034)	#SAKNAS!
anti-aspiration	1	5033 (0.011)	#SAKNAS!	#SAKNAS!
anti-asthmatic	1	5033 (0.011)	#SAKNAS!	#SAKNAS!
antibody-associated	1	5033 (0.011)	#SAKNAS!	#SAKNAS!
anticancer	1	5033 (0.011)	#SAKNAS!	#SAKNAS!
anticaries	1	5033 (0.011)	#SAKNAS!	2492 (0.033)
anticariogenesis	1	5033 (0.011)	2293 (0.034)	#SAKNAS!
anticarsia-gemmatalis	1	5033 (0.011)	2293 (0.034)	#SAKNAS!
anti-defoliate	1	5033 (0.011)	2293 (0.034)	#SAKNAS!
antifeedant	1	5033 (0.011)	#SAKNAS!	2492 (0.033)
antigens	1	5033 (0.011)	2293 (0.034)	#SAKNAS!
anti-infectious	1	5033 (0.011)	2293 (0.034)	#SAKNAS!
antimuscarinic	1	5033 (0.011)	#SAKNAS!	#SAKNAS!

anti-nutritional	1	5033 (0.011)	2293 (0.034)	#SAKNAS!
anti-nxp2	1	5033 (0.011)	#SAKNAS!	#SAKNAS!
antioxidant-rich	1	5033 (0.011)	#SAKNAS!	2492 (0.033)
antiplaque	1	5033 (0.011)	2293 (0.034)	#SAKNAS!
anti-predator	1	5033 (0.011)	2293 (0.034)	#SAKNAS!
antipsychotics	1	5033 (0.011)	2293 (0.034)	#SAKNAS!
antiretroviral-experienced	1	5033 (0.011)	#SAKNAS!	2492 (0.033)
antisecretory	1	5033 (0.011)	2293 (0.034)	#SAKNAS!
antiseptic	1	5033 (0.011)	#SAKNAS!	2492 (0.033)
antixenosis	1	5033 (0.011)	#SAKNAS!	#SAKNAS!
antral	1	5033 (0.011)	2293 (0.034)	#SAKNAS!
aorta	1	5033 (0.011)	#SAKNAS!	2492 (0.033)
aortobronchial	1	5033 (0.011)	#SAKNAS!	2492 (0.033)
aortoesophageal	1	5033 (0.011)	#SAKNAS!	2492 (0.033)
aospine	1	5033 (0.011)	2293 (0.034)	#SAKNAS!
aotearoa	1	5033 (0.011)	#SAKNAS!	#SAKNAS!
apama	1	5033 (0.011)	2293 (0.034)	#SAKNAS!
apart	1	5033 (0.011)	2293 (0.034)	#SAKNAS!
apatinib	1	5033 (0.011)	#SAKNAS!	#SAKNAS!
ape	1	5033 (0.011)	#SAKNAS!	2492 (0.033)
apella	1	5033 (0.011)	2293 (0.034)	#SAKNAS!
apert	1	5033 (0.011)	2293 (0.034)	#SAKNAS!
apex	1	5033 (0.011)	#SAKNAS!	#SAKNAS!
aphagia	1	5033 (0.011)	2293 (0.034)	#SAKNAS!
aphalaridae	1	5033 (0.011)	#SAKNAS!	2492 (0.033)
aphidius	1	5033 (0.011)	2293 (0.034)	#SAKNAS!
aphytis-lingnanensis	1	5033 (0.011)	2293 (0.034)	#SAKNAS!
apiaries	1	5033 (0.011)	#SAKNAS!	2492 (0.033)
aplodactylus-punctatus	1	5033 (0.011)	2293 (0.034)	#SAKNAS!
apnoea	1	5033 (0.011)	#SAKNAS!	2492 (0.033)
apo	1	5033 (0.011)	2293 (0.034)	#SAKNAS!
apoa	1	5033 (0.011)	#SAKNAS!	#SAKNAS!
apollonia	1	5033 (0.011)	#SAKNAS!	2492 (0.033)
appalachian	1	5033 (0.011)	#SAKNAS!	2492 (0.033)
appendages	1	5033 (0.011)	#SAKNAS!	#SAKNAS!
apples	1	5033 (0.011)	#SAKNAS!	2492 (0.033)
appliances	1	5033 (0.011)	#SAKNAS!	2492 (0.033)
application-based	1	5033 (0.011)	#SAKNAS!	#SAKNAS!
appraisal	1	5033 (0.011)	#SAKNAS!	2492 (0.033)
appreciation	1	5033 (0.011)	#SAKNAS!	#SAKNAS!
apps	1	5033 (0.011)	#SAKNAS!	#SAKNAS!
aproaches	1	5033 (0.011)	#SAKNAS!	2492 (0.033)
apron	1	5033 (0.011)	#SAKNAS!	#SAKNAS!

apulia	1	5033 (0.011)	#SAKNAS!	#SAKNAS!
aquafeed	1	5033 (0.011)	#SAKNAS!	#SAKNAS!
aquaporin-3	1	5033 (0.011)	#SAKNAS!	#SAKNAS!
arabia-a	1	5033 (0.011)	#SAKNAS!	2492 (0.033)
arabidopsis	1	5033 (0.011)	2293 (0.034)	#SAKNAS!
arachidonic-acid	1	5033 (0.011)	2293 (0.034)	#SAKNAS!
arapaima	1	5033 (0.011)	#SAKNAS!	2492 (0.033)
aratus	1	5033 (0.011)	2293 (0.034)	#SAKNAS!
arboreal	1	5033 (0.011)	#SAKNAS!	#SAKNAS!
arcage	1	5033 (0.011)	#SAKNAS!	2492 (0.033)
archaeological	1	5033 (0.011)	#SAKNAS!	2492 (0.033)
archipelago	1	5033 (0.011)	2293 (0.034)	#SAKNAS!
arctica	1	5033 (0.011)	2293 (0.034)	#SAKNAS!
arcticus	1	5033 (0.011)	#SAKNAS!	2492 (0.033)
arduino-based	1	5033 (0.011)	#SAKNAS!	#SAKNAS!
area-defined	1	5033 (0.011)	2293 (0.034)	#SAKNAS!
arena	1	5033 (0.011)	2293 (0.034)	#SAKNAS!
arfid	1	5033 (0.011)	#SAKNAS!	#SAKNAS!
arginine	1	5033 (0.011)	#SAKNAS!	2492 (0.033)
argopecten	1	5033 (0.011)	#SAKNAS!	2492 (0.033)
arid	1	5033 (0.011)	2293 (0.034)	#SAKNAS!
arising	1	5033 (0.011)	2293 (0.034)	#SAKNAS!
arius	1	5033 (0.011)	2293 (0.034)	#SAKNAS!
arjuna	1	5033 (0.011)	2293 (0.034)	#SAKNAS!
armata	1	5033 (0.011)	#SAKNAS!	2492 (0.033)
aroma	1	5033 (0.011)	#SAKNAS!	2492 (0.033)
arrangement	1	5033 (0.011)	2293 (0.034)	#SAKNAS!
arrest	1	5033 (0.011)	2293 (0.034)	#SAKNAS!
arresting	1	5033 (0.011)	#SAKNAS!	2492 (0.033)
arsanjan	1	5033 (0.011)	2293 (0.034)	#SAKNAS!
arteries	1	5033 (0.011)	2293 (0.034)	#SAKNAS!
arthroplasty	1	5033 (0.011)	#SAKNAS!	#SAKNAS!
arytenoid	1	5033 (0.011)	2293 (0.034)	#SAKNAS!
ascorbic	1	5033 (0.011)	2293 (0.034)	#SAKNAS!
ascorbic-acid	1	5033 (0.011)	2293 (0.034)	#SAKNAS!
asd	1	5033 (0.011)	#SAKNAS!	2492 (0.033)
asgb	1	5033 (0.011)	2293 (0.034)	#SAKNAS!
asiatic	1	5033 (0.011)	2293 (0.034)	#SAKNAS!
asiatica	1	5033 (0.011)	2293 (0.034)	#SAKNAS!
aspen	1	5033 (0.011)	2293 (0.034)	#SAKNAS!
aspergillus	1	5033 (0.011)	2293 (0.034)	#SAKNAS!
asphaltic	1	5033 (0.011)	#SAKNAS!	2492 (0.033)
aspirational	1	5033 (0.011)	#SAKNAS!	#SAKNAS!

aspiration-related	1	5033 (0.011)	#SAKNAS!	#SAKNAS!
aspirations	1	5033 (0.011)	#SAKNAS!	#SAKNAS!
asprosin	1	5033 (0.011)	#SAKNAS!	#SAKNAS!
assamensis	1	5033 (0.011)	2293 (0.034)	#SAKNAS!
assaying	1	5033 (0.011)	#SAKNAS!	2492 (0.033)
assemblage	1	5033 (0.011)	2293 (0.034)	#SAKNAS!
assertive	1	5033 (0.011)	2293 (0.034)	#SAKNAS!
assessor-blinded	1	5033 (0.011)	#SAKNAS!	2492 (0.033)
assistant	1	5033 (0.011)	#SAKNAS!	2492 (0.033)
associate	1	5033 (0.011)	#SAKNAS!	2492 (0.033)
associative	1	5033 (0.011)	#SAKNAS!	2492 (0.033)
assortative	1	5033 (0.011)	2293 (0.034)	#SAKNAS!
assuaging	1	5033 (0.011)	2293 (0.034)	#SAKNAS!
asteraceae	1	5033 (0.011)	2293 (0.034)	#SAKNAS!
asteroidea	1	5033 (0.011)	2293 (0.034)	#SAKNAS!
asthmatic	1	5033 (0.011)	#SAKNAS!	#SAKNAS!
astrangia	1	5033 (0.011)	#SAKNAS!	2492 (0.033)
astropecten-articulatus	1	5033 (0.011)	2293 (0.034)	#SAKNAS!
asymmetricum	1	5033 (0.011)	#SAKNAS!	2492 (0.033)
asymmetry	1	5033 (0.011)	2293 (0.034)	#SAKNAS!
ataxia-a	1	5033 (0.011)	#SAKNAS!	2492 (0.033)
ataxias	1	5033 (0.011)	#SAKNAS!	#SAKNAS!
ataxia-telangiectasia	1	5033 (0.011)	2293 (0.034)	#SAKNAS!
atelerix	1	5033 (0.011)	#SAKNAS!	2492 (0.033)
ateles	1	5033 (0.011)	2293 (0.034)	#SAKNAS!
ater	1	5033 (0.011)	2293 (0.034)	#SAKNAS!
atherogenesis	1	5033 (0.011)	2293 (0.034)	#SAKNAS!
atheroprotective	1	5033 (0.011)	#SAKNAS!	2492 (0.033)
athlete	1	5033 (0.011)	#SAKNAS!	#SAKNAS!
athletes-an	1	5033 (0.011)	#SAKNAS!	#SAKNAS!
athletic	1	5033 (0.011)	#SAKNAS!	2492 (0.033)
at-home	1	5033 (0.011)	#SAKNAS!	2492 (0.033)
athospital	1	5033 (0.011)	#SAKNAS!	2492 (0.033)
atra	1	5033 (0.011)	#SAKNAS!	#SAKNAS!
atresia-tracheoesophageal	1	5033 (0.011)	#SAKNAS!	2492 (0.033)
atricapilla	1	5033 (0.011)	2293 (0.034)	#SAKNAS!
atricauda	1	5033 (0.011)	#SAKNAS!	2492 (0.033)
at-risk	1	5033 (0.011)	#SAKNAS!	#SAKNAS!
atrophic-erosive	1	5033 (0.011)	#SAKNAS!	2492 (0.033)
attack	1	5033 (0.011)	2293 (0.034)	#SAKNAS!
attainable	1	5033 (0.011)	#SAKNAS!	#SAKNAS!
attempts	1	5033 (0.011)	#SAKNAS!	2492 (0.033)
attenders	1	5033 (0.011)	2293 (0.034)	#SAKNAS!

attention-deficit	1	5033 (0.011)	#SAKNAS!	#SAKNAS!
attenuate	1	5033 (0.011)	#SAKNAS!	#SAKNAS!
attract	1	5033 (0.011)	#SAKNAS!	2492 (0.033)
attraction	1	5033 (0.011)	#SAKNAS!	#SAKNAS!
attractiveness	1	5033 (0.011)	#SAKNAS!	2492 (0.033)
attributable	1	5033 (0.011)	2293 (0.034)	#SAKNAS!
atyaephyra	1	5033 (0.011)	#SAKNAS!	2492 (0.033)
audiology	1	5033 (0.011)	#SAKNAS!	2492 (0.033)
audiovestibular	1	5033 (0.011)	#SAKNAS!	2492 (0.033)
auf	1	5033 (0.011)	2293 (0.034)	#SAKNAS!
augment	1	5033 (0.011)	#SAKNAS!	2492 (0.033)
august	1	5033 (0.011)	2293 (0.034)	#SAKNAS!
aurelia-aurita	1	5033 (0.011)	2293 (0.034)	#SAKNAS!
australian-born	1	5033 (0.011)	#SAKNAS!	2492 (0.033)
australians	1	5033 (0.011)	#SAKNAS!	2492 (0.033)
austriacus	1	5033 (0.011)	2293 (0.034)	#SAKNAS!
austrian	1	5033 (0.011)	2293 (0.034)	#SAKNAS!
autoantibodies	1	5033 (0.011)	2293 (0.034)	#SAKNAS!
autoantigens	1	5033 (0.011)	#SAKNAS!	2492 (0.033)
autograft	1	5033 (0.011)	#SAKNAS!	2492 (0.033)
autoimmune	1	5033 (0.011)	#SAKNAS!	#SAKNAS!
autonomic	1	5033 (0.011)	2293 (0.034)	#SAKNAS!
autonomous	1	5033 (0.011)	#SAKNAS!	2492 (0.033)
autonomy	1	5033 (0.011)	#SAKNAS!	2492 (0.033)
autophagy	1	5033 (0.011)	#SAKNAS!	#SAKNAS!
autopsy	1	5033 (0.011)	#SAKNAS!	#SAKNAS!
autotomization	1	5033 (0.011)	#SAKNAS!	2492 (0.033)
autumn	1	5033 (0.011)	2293 (0.034)	#SAKNAS!
avenae	1	5033 (0.011)	#SAKNAS!	2492 (0.033)
average	1	5033 (0.011)	#SAKNAS!	#SAKNAS!
aversive	1	5033 (0.011)	#SAKNAS!	2492 (0.033)
avidity	1	5033 (0.011)	2293 (0.034)	#SAKNAS!
aviz	1	5033 (0.011)	#SAKNAS!	#SAKNAS!
avoiding	1	5033 (0.011)	2293 (0.034)	#SAKNAS!
avon	1	5033 (0.011)	2293 (0.034)	#SAKNAS!
awakening	1	5033 (0.011)	2293 (0.034)	#SAKNAS!
axenic	1	5033 (0.011)	2293 (0.034)	#SAKNAS!
axis-boosted	1	5033 (0.011)	#SAKNAS!	#SAKNAS!
ayder	1	5033 (0.011)	#SAKNAS!	#SAKNAS!
azadirachtin	1	5033 (0.011)	#SAKNAS!	2492 (0.033)
azithromycin	1	5033 (0.011)	#SAKNAS!	2492 (0.033)
a瘺a穩	1	5033 (0.011)	#SAKNAS!	#SAKNAS!
b1	1	5033 (0.011)	#SAKNAS!	#SAKNAS!

b12	1	5033 (0.011)	#SAKNAS!	#SAKNAS!
ba	1	5033 (0.011)	2293 (0.034)	#SAKNAS!
babassu	1	5033 (0.011)	2293 (0.034)	#SAKNAS!
babies	1	5033 (0.011)	#SAKNAS!	#SAKNAS!
baboon	1	5033 (0.011)	#SAKNAS!	2492 (0.033)
baby-friendly	1	5033 (0.011)	#SAKNAS!	#SAKNAS!
baby-led	1	5033 (0.011)	#SAKNAS!	2492 (0.033)
backgrounds	1	5033 (0.011)	#SAKNAS!	#SAKNAS!
baclofen	1	5033 (0.011)	2293 (0.034)	#SAKNAS!
bacterial-feeding	1	5033 (0.011)	2293 (0.034)	#SAKNAS!
bacteriological	1	5033 (0.011)	2293 (0.034)	#SAKNAS!
bacterium	1	5033 (0.011)	#SAKNAS!	#SAKNAS!
bacterivory	1	5033 (0.011)	2293 (0.034)	#SAKNAS!
baculovirus-challenged	1	5033 (0.011)	#SAKNAS!	2492 (0.033)
bagasse	1	5033 (0.011)	#SAKNAS!	#SAKNAS!
bahia	1	5033 (0.011)	#SAKNAS!	2492 (0.033)
bahrain	1	5033 (0.011)	#SAKNAS!	#SAKNAS!
baladi	1	5033 (0.011)	#SAKNAS!	2492 (0.033)
balancan	1	5033 (0.011)	2293 (0.034)	#SAKNAS!
balb	1	5033 (0.011)	#SAKNAS!	2492 (0.033)
ballabio	1	5033 (0.011)	#SAKNAS!	2492 (0.033)
ballet	1	5033 (0.011)	2293 (0.034)	#SAKNAS!
balochistan	1	5033 (0.011)	#SAKNAS!	2492 (0.033)
baltimore	1	5033 (0.011)	2293 (0.034)	#SAKNAS!
bamboo	1	5033 (0.011)	2293 (0.034)	#SAKNAS!
bamboo-leaf-based	1	5033 (0.011)	#SAKNAS!	2492 (0.033)
band-c	1	5033 (0.011)	2293 (0.034)	#SAKNAS!
bandings	1	5033 (0.011)	#SAKNAS!	2492 (0.033)
banks	1	5033 (0.011)	2293 (0.034)	#SAKNAS!
barbadensis	1	5033 (0.011)	2293 (0.034)	#SAKNAS!
barbara	1	5033 (0.011)	#SAKNAS!	#SAKNAS!
barbatus	1	5033 (0.011)	2293 (0.034)	#SAKNAS!
barcoding	1	5033 (0.011)	#SAKNAS!	2492 (0.033)
bariloche	1	5033 (0.011)	#SAKNAS!	2492 (0.033)
barn	1	5033 (0.011)	#SAKNAS!	#SAKNAS!
barrel	1	5033 (0.011)	#SAKNAS!	2492 (0.033)
bar-retained	1	5033 (0.011)	#SAKNAS!	#SAKNAS!
barretts-esophagus	1	5033 (0.011)	2293 (0.034)	#SAKNAS!
bars	1	5033 (0.011)	#SAKNAS!	#SAKNAS!
based-sample	1	5033 (0.011)	#SAKNAS!	#SAKNAS!
basilar	1	5033 (0.011)	#SAKNAS!	2492 (0.033)
batatas	1	5033 (0.011)	2293 (0.034)	#SAKNAS!
bats	1	5033 (0.011)	#SAKNAS!	#SAKNAS!

bayesian	1	5033 (0.011)	#SAKNAS!	#SAKNAS!
bayley	1	5033 (0.011)	2293 (0.034)	#SAKNAS!
bc	1	5033 (0.011)	2293 (0.034)	#SAKNAS!
bce-200	1	5033 (0.011)	#SAKNAS!	2492 (0.033)
bde-47	1	5033 (0.011)	#SAKNAS!	#SAKNAS!
bdelloid	1	5033 (0.011)	#SAKNAS!	#SAKNAS!
beak	1	5033 (0.011)	#SAKNAS!	2492 (0.033)
beam	1	5033 (0.011)	2293 (0.034)	#SAKNAS!
beam-conditioning	1	5033 (0.011)	2293 (0.034)	#SAKNAS!
bean	1	5033 (0.011)	#SAKNAS!	2492 (0.033)
bean-based	1	5033 (0.011)	#SAKNAS!	#SAKNAS!
beaver	1	5033 (0.011)	#SAKNAS!	2492 (0.033)
became	1	5033 (0.011)	#SAKNAS!	#SAKNAS!
bedding	1	5033 (0.011)	2293 (0.034)	#SAKNAS!
beet	1	5033 (0.011)	2293 (0.034)	#SAKNAS!
beetroot	1	5033 (0.011)	#SAKNAS!	2492 (0.033)
beginnings	1	5033 (0.011)	2293 (0.034)	#SAKNAS!
begins	1	5033 (0.011)	#SAKNAS!	2492 (0.033)
behaviar	1	5033 (0.011)	2293 (0.034)	#SAKNAS!
behavior-change	1	5033 (0.011)	2293 (0.034)	#SAKNAS!
behcet	1	5033 (0.011)	2293 (0.034)	#SAKNAS!
beijing	1	5033 (0.011)	#SAKNAS!	#SAKNAS!
belarusian	1	5033 (0.011)	#SAKNAS!	#SAKNAS!
belgrade	1	5033 (0.011)	2293 (0.034)	#SAKNAS!
belief	1	5033 (0.011)	#SAKNAS!	#SAKNAS!
bell	1	5033 (0.011)	#SAKNAS!	2492 (0.033)
bellydent	1	5033 (0.011)	#SAKNAS!	#SAKNAS!
belowground	1	5033 (0.011)	#SAKNAS!	2492 (0.033)
belzebuth	1	5033 (0.011)	2293 (0.034)	#SAKNAS!
bench	1	5033 (0.011)	#SAKNAS!	2492 (0.033)
benchmarking	1	5033 (0.011)	#SAKNAS!	#SAKNAS!
benchmarks	1	5033 (0.011)	#SAKNAS!	#SAKNAS!
bend	1	5033 (0.011)	2293 (0.034)	#SAKNAS!
beneficiaries	1	5033 (0.011)	#SAKNAS!	#SAKNAS!
benefit-risk	1	5033 (0.011)	2293 (0.034)	#SAKNAS!
benghazi	1	5033 (0.011)	#SAKNAS!	#SAKNAS!
benguela	1	5033 (0.011)	#SAKNAS!	2492 (0.033)
bennett	1	5033 (0.011)	#SAKNAS!	#SAKNAS!
benzoate	1	5033 (0.011)	#SAKNAS!	2492 (0.033)
bergamo	1	5033 (0.011)	2293 (0.034)	#SAKNAS!
bergenia	1	5033 (0.011)	2293 (0.034)	#SAKNAS!
beringei	1	5033 (0.011)	#SAKNAS!	2492 (0.033)
bermuda	1	5033 (0.011)	#SAKNAS!	2492 (0.033)

bernicla	1 5033 (0.011)	2293 (0.034)	#SAKNAS!
berries	1 5033 (0.011)	2293 (0.034)	#SAKNAS!
bet	1 5033 (0.011)	#SAKNAS!	2492 (0.033)
beta-3	1 5033 (0.011)	#SAKNAS!	2492 (0.033)
beta-band	1 5033 (0.011)	#SAKNAS!	#SAKNAS!
beta-blockers	1 5033 (0.011)	#SAKNAS!	2492 (0.033)
betel	1 5033 (0.011)	2293 (0.034)	#SAKNAS!
better-quality	1 5033 (0.011)	#SAKNAS!	2492 (0.033)
betweenaphis	1 5033 (0.011)	#SAKNAS!	2492 (0.033)
beyond-brand	1 5033 (0.011)	2293 (0.034)	#SAKNAS!
bezoar	1 5033 (0.011)	#SAKNAS!	#SAKNAS!
bfrs	1 5033 (0.011)	#SAKNAS!	#SAKNAS!
bhopal	1 5033 (0.011)	#SAKNAS!	2492 (0.033)
bia-derived	1 5033 (0.011)	#SAKNAS!	#SAKNAS!
bialystok	1 5033 (0.011)	#SAKNAS!	#SAKNAS!
bicornis	1 5033 (0.011)	2293 (0.034)	#SAKNAS!
bictegravir	1 5033 (0.011)	#SAKNAS!	2492 (0.033)
bifidobacteriaceae	1 5033 (0.011)	2293 (0.034)	#SAKNAS!
bifidobacterium	1 5033 (0.011)	#SAKNAS!	2492 (0.033)
bighorn	1 5033 (0.011)	2293 (0.034)	#SAKNAS!
bile	1 5033 (0.011)	#SAKNAS!	2492 (0.033)
bilineatus	1 5033 (0.011)	2293 (0.034)	#SAKNAS!
bin	1 5033 (0.011)	#SAKNAS!	2492 (0.033)
binds	1 5033 (0.011)	2293 (0.034)	#SAKNAS!
binge-like	1 5033 (0.011)	2293 (0.034)	#SAKNAS!
bioaccessibility	1 5033 (0.011)	#SAKNAS!	#SAKNAS!
bioarchaeological	1 5033 (0.011)	#SAKNAS!	#SAKNAS!
bioarchaeology	1 5033 (0.011)	#SAKNAS!	2492 (0.033)
bioassay	1 5033 (0.011)	2293 (0.034)	#SAKNAS!
bioassays	1 5033 (0.011)	2293 (0.034)	#SAKNAS!
biodeposits	1 5033 (0.011)	#SAKNAS!	2492 (0.033)
biodiversity	1 5033 (0.011)	#SAKNAS!	#SAKNAS!
bioenergetic	1 5033 (0.011)	#SAKNAS!	#SAKNAS!
bioenterics	1 5033 (0.011)	2293 (0.034)	#SAKNAS!
biofilm-demineralization	1 5033 (0.011)	#SAKNAS!	2492 (0.033)
biofilm-insights	1 5033 (0.011)	#SAKNAS!	2492 (0.033)
bio-functional	1 5033 (0.011)	#SAKNAS!	#SAKNAS!
biogeochemical	1 5033 (0.011)	#SAKNAS!	2492 (0.033)
biogeographical	1 5033 (0.011)	2293 (0.034)	#SAKNAS!
biologically	1 5033 (0.011)	2293 (0.034)	#SAKNAS!
bio-markers	1 5033 (0.011)	2293 (0.034)	#SAKNAS!
biomarkers-soluble	1 5033 (0.011)	#SAKNAS!	2492 (0.033)
biomechanical	1 5033 (0.011)	#SAKNAS!	2492 (0.033)

biometrics	1 5033 (0.011)	2293 (0.034)	#SAKNAS!
biomphalaria-glabrata	1 5033 (0.011)	2293 (0.034)	#SAKNAS!
bionic	1 5033 (0.011)	2293 (0.034)	#SAKNAS!
bioqualim	1 5033 (0.011)	#SAKNAS!	#SAKNAS!
biosignals	1 5033 (0.011)	#SAKNAS!	#SAKNAS!
biosocial	1 5033 (0.011)	#SAKNAS!	#SAKNAS!
biosynthesis	1 5033 (0.011)	#SAKNAS!	#SAKNAS!
biosynthetic	1 5033 (0.011)	#SAKNAS!	#SAKNAS!
biotransformation	1 5033 (0.011)	#SAKNAS!	2492 (0.033)
biotrophic	1 5033 (0.011)	#SAKNAS!	#SAKNAS!
biracial	1 5033 (0.011)	2293 (0.034)	#SAKNAS!
bird	1 5033 (0.011)	2293 (0.034)	#SAKNAS!
birth-related	1 5033 (0.011)	#SAKNAS!	#SAKNAS!
birth-to-ten	1 5033 (0.011)	2293 (0.034)	#SAKNAS!
birthweight	1 5033 (0.011)	2293 (0.034)	#SAKNAS!
biscuit	1 5033 (0.011)	#SAKNAS!	#SAKNAS!
bisphosphate	1 5033 (0.011)	2293 (0.034)	#SAKNAS!
bisphosphonate-related	1 5033 (0.011)	2293 (0.034)	#SAKNAS!
bisulcus	1 5033 (0.011)	2293 (0.034)	#SAKNAS!
bite-force	1 5033 (0.011)	#SAKNAS!	2492 (0.033)
bits	1 5033 (0.011)	#SAKNAS!	2492 (0.033)
bitter-sensitive	1 5033 (0.011)	#SAKNAS!	2492 (0.033)
bitwin	1 5033 (0.011)	#SAKNAS!	#SAKNAS!
bivalves	1 5033 (0.011)	#SAKNAS!	#SAKNAS!
biweekly	1 5033 (0.011)	#SAKNAS!	2492 (0.033)
black-backed	1 5033 (0.011)	2293 (0.034)	#SAKNAS!
blackbird	1 5033 (0.011)	2293 (0.034)	#SAKNAS!
blackcaps	1 5033 (0.011)	2293 (0.034)	#SAKNAS!
black-children	1 5033 (0.011)	2293 (0.034)	#SAKNAS!
blatta	1 5033 (0.011)	#SAKNAS!	#SAKNAS!
blattella	1 5033 (0.011)	2293 (0.034)	#SAKNAS!
blattodea	1 5033 (0.011)	#SAKNAS!	#SAKNAS!
blazei	1 5033 (0.011)	#SAKNAS!	#SAKNAS!
blend	1 5033 (0.011)	#SAKNAS!	#SAKNAS!
blindness	1 5033 (0.011)	#SAKNAS!	2492 (0.033)
bloating	1 5033 (0.011)	#SAKNAS!	#SAKNAS!
bloch	1 5033 (0.011)	2293 (0.034)	#SAKNAS!
block	1 5033 (0.011)	2293 (0.034)	#SAKNAS!
bloodborne	1 5033 (0.011)	2293 (0.034)	#SAKNAS!
bloodstream	1 5033 (0.011)	2293 (0.034)	#SAKNAS!
blooming	1 5033 (0.011)	#SAKNAS!	#SAKNAS!
blowfly	1 5033 (0.011)	2293 (0.034)	#SAKNAS!
bluegill	1 5033 (0.011)	2293 (0.034)	#SAKNAS!

bmal1	1	5033 (0.011)	#SAKNAS!	#SAKNAS!
bmi-modified	1	5033 (0.011)	#SAKNAS!	#SAKNAS!
bmorco	1	5033 (0.011)	#SAKNAS!	2492 (0.033)
boa	1	5033 (0.011)	#SAKNAS!	#SAKNAS!
board	1	5033 (0.011)	#SAKNAS!	2492 (0.033)
boarding	1	5033 (0.011)	2293 (0.034)	#SAKNAS!
boars	1	5033 (0.011)	2293 (0.034)	#SAKNAS!
boccardia	1	5033 (0.011)	#SAKNAS!	2492 (0.033)
body-temperature	1	5033 (0.011)	2293 (0.034)	#SAKNAS!
boer	1	5033 (0.011)	#SAKNAS!	#SAKNAS!
bog	1	5033 (0.011)	#SAKNAS!	#SAKNAS!
boisd	1	5033 (0.011)	#SAKNAS!	#SAKNAS!
bolbometopon	1	5033 (0.011)	#SAKNAS!	2492 (0.033)
bolster	1	5033 (0.011)	2293 (0.034)	#SAKNAS!
bone-density	1	5033 (0.011)	2293 (0.034)	#SAKNAS!
bone-mineral	1	5033 (0.011)	2293 (0.034)	#SAKNAS!
bonnaterre	1	5033 (0.011)	#SAKNAS!	#SAKNAS!
bonobo	1	5033 (0.011)	#SAKNAS!	2492 (0.033)
book	1	5033 (0.011)	#SAKNAS!	#SAKNAS!
booklet	1	5033 (0.011)	2293 (0.034)	#SAKNAS!
boone	1	5033 (0.011)	#SAKNAS!	2492 (0.033)
boosting	1	5033 (0.011)	2293 (0.034)	#SAKNAS!
borderline	1	5033 (0.011)	#SAKNAS!	2492 (0.033)
boreioglycaspis	1	5033 (0.011)	2293 (0.034)	#SAKNAS!
bornean	1	5033 (0.011)	#SAKNAS!	2492 (0.033)
boscii	1	5033 (0.011)	2293 (0.034)	#SAKNAS!
bosnia	1	5033 (0.011)	2293 (0.034)	#SAKNAS!
bossou	1	5033 (0.011)	2293 (0.034)	#SAKNAS!
boston	1	5033 (0.011)	2293 (0.034)	#SAKNAS!
bostwick	1	5033 (0.011)	#SAKNAS!	2492 (0.033)
botanical	1	5033 (0.011)	#SAKNAS!	2492 (0.033)
botswana	1	5033 (0.011)	2293 (0.034)	#SAKNAS!
bottled	1	5033 (0.011)	#SAKNAS!	2492 (0.033)
bottle-feeding	1	5033 (0.011)	2293 (0.034)	#SAKNAS!
bottom-up	1	5033 (0.011)	#SAKNAS!	#SAKNAS!
bougie	1	5033 (0.011)	#SAKNAS!	#SAKNAS!
boulardii	1	5033 (0.011)	#SAKNAS!	#SAKNAS!
boundary	1	5033 (0.011)	2293 (0.034)	#SAKNAS!
bouquet	1	5033 (0.011)	#SAKNAS!	#SAKNAS!
bpifa1	1	5033 (0.011)	#SAKNAS!	2492 (0.033)
brains	1	5033 (0.011)	2293 (0.034)	#SAKNAS!
brain-stem	1	5033 (0.011)	2293 (0.034)	#SAKNAS!
brain-tumor	1	5033 (0.011)	2293 (0.034)	#SAKNAS!

bran	1	5033 (0.011)	#SAKNAS!	#SAKNAS!
brandt	1	5033 (0.011)	#SAKNAS!	#SAKNAS!
branta	1	5033 (0.011)	2293 (0.034)	#SAKNAS!
brasila	1	5033 (0.011)	2293 (0.034)	#SAKNAS!
brasileira	1	5033 (0.011)	#SAKNAS!	#SAKNAS!
brassica	1	5033 (0.011)	#SAKNAS!	2492 (0.033)
brazilian-portuguese	1	5033 (0.011)	#SAKNAS!	#SAKNAS!
braziliansa	1	5033 (0.011)	#SAKNAS!	2492 (0.033)
brazzaville	1	5033 (0.011)	2293 (0.034)	#SAKNAS!
brea	1	5033 (0.011)	#SAKNAS!	2492 (0.033)
breadth	1	5033 (0.011)	#SAKNAS!	2492 (0.033)
break	1	5033 (0.011)	#SAKNAS!	#SAKNAS!
breakfasts	1	5033 (0.011)	2293 (0.034)	#SAKNAS!
breakfast-three	1	5033 (0.011)	#SAKNAS!	2492 (0.033)
breaks	1	5033 (0.011)	2293 (0.034)	#SAKNAS!
breastfeeding-friendly	1	5033 (0.011)	2293 (0.034)	#SAKNAS!
breastmilk	1	5033 (0.011)	#SAKNAS!	2492 (0.033)
breath	1	5033 (0.011)	2293 (0.034)	#SAKNAS!
breathing-related	1	5033 (0.011)	#SAKNAS!	2492 (0.033)
brent	1	5033 (0.011)	2293 (0.034)	#SAKNAS!
breviceps	1	5033 (0.011)	#SAKNAS!	2492 (0.033)
brevis	1	5033 (0.011)	2293 (0.034)	#SAKNAS!
brick	1	5033 (0.011)	#SAKNAS!	2492 (0.033)
bridle	1	5033 (0.011)	#SAKNAS!	#SAKNAS!
brief-motivational	1	5033 (0.011)	#SAKNAS!	#SAKNAS!
bright	1	5033 (0.011)	#SAKNAS!	#SAKNAS!
bring	1	5033 (0.011)	2293 (0.034)	#SAKNAS!
broad	1	5033 (0.011)	2293 (0.034)	#SAKNAS!
broadbent	1	5033 (0.011)	2293 (0.034)	#SAKNAS!
broadly	1	5033 (0.011)	#SAKNAS!	2492 (0.033)
broilers	1	5033 (0.011)	2293 (0.034)	#SAKNAS!
brood	1	5033 (0.011)	2293 (0.034)	#SAKNAS!
broodnest	1	5033 (0.011)	2293 (0.034)	#SAKNAS!
brothers	1	5033 (0.011)	#SAKNAS!	#SAKNAS!
browse	1	5033 (0.011)	#SAKNAS!	#SAKNAS!
bruchid	1	5033 (0.011)	2293 (0.034)	#SAKNAS!
bruchidius	1	5033 (0.011)	2293 (0.034)	#SAKNAS!
brushes	1	5033 (0.011)	#SAKNAS!	2492 (0.033)
brushtail	1	5033 (0.011)	2293 (0.034)	#SAKNAS!
brycon	1	5033 (0.011)	#SAKNAS!	2492 (0.033)
bsg	1	5033 (0.011)	#SAKNAS!	#SAKNAS!
bspghan	1	5033 (0.011)	#SAKNAS!	#SAKNAS!
bt	1	5033 (0.011)	#SAKNAS!	2492 (0.033)

btxa	1	5033 (0.011)	#SAKNAS!	2492 (0.033)
buccinum-undatum	1	5033 (0.011)	2293 (0.034)	#SAKNAS!
bud	1	5033 (0.011)	2293 (0.034)	#SAKNAS!
budesonide	1	5033 (0.011)	#SAKNAS!	2492 (0.033)
buds	1	5033 (0.011)	2293 (0.034)	#SAKNAS!
buffer	1	5033 (0.011)	#SAKNAS!	2492 (0.033)
buffet	1	5033 (0.011)	#SAKNAS!	2492 (0.033)
bufonids	1	5033 (0.011)	#SAKNAS!	#SAKNAS!
bug	1	5033 (0.011)	#SAKNAS!	#SAKNAS!
builds	1	5033 (0.011)	2293 (0.034)	#SAKNAS!
bujumbura	1	5033 (0.011)	2293 (0.034)	#SAKNAS!
bulb	1	5033 (0.011)	#SAKNAS!	2492 (0.033)
bulgaria	1	5033 (0.011)	2293 (0.034)	#SAKNAS!
bulhosa	1	5033 (0.011)	2293 (0.034)	#SAKNAS!
bulimia-nervosa	1	5033 (0.011)	2293 (0.034)	#SAKNAS!
bulk	1	5033 (0.011)	2293 (0.034)	#SAKNAS!
bull	1	5033 (0.011)	#SAKNAS!	2492 (0.033)
bullet	1	5033 (0.011)	#SAKNAS!	2492 (0.033)
bumble	1	5033 (0.011)	2293 (0.034)	#SAKNAS!
bumphead	1	5033 (0.011)	#SAKNAS!	2492 (0.033)
bundled	1	5033 (0.011)	#SAKNAS!	#SAKNAS!
burbank	1	5033 (0.011)	#SAKNAS!	2492 (0.033)
buren	1	5033 (0.011)	2293 (0.034)	#SAKNAS!
burial	1	5033 (0.011)	#SAKNAS!	#SAKNAS!
burkina	1	5033 (0.011)	#SAKNAS!	2492 (0.033)
burnout	1	5033 (0.011)	#SAKNAS!	#SAKNAS!
burnout-related	1	5033 (0.011)	#SAKNAS!	2492 (0.033)
burst	1	5033 (0.011)	#SAKNAS!	2492 (0.033)
burundi	1	5033 (0.011)	2293 (0.034)	#SAKNAS!
bushcricket	1	5033 (0.011)	#SAKNAS!	2492 (0.033)
busting	1	5033 (0.011)	#SAKNAS!	2492 (0.033)
button	1	5033 (0.011)	#SAKNAS!	#SAKNAS!
butyrate	1	5033 (0.011)	#SAKNAS!	#SAKNAS!
bwindi	1	5033 (0.011)	2293 (0.034)	#SAKNAS!
bypasses	1	5033 (0.011)	#SAKNAS!	#SAKNAS!
by-product	1	5033 (0.011)	#SAKNAS!	#SAKNAS!
c1q	1	5033 (0.011)	#SAKNAS!	#SAKNAS!
c2-7	1	5033 (0.011)	#SAKNAS!	2492 (0.033)
c-4	1	5033 (0.011)	2293 (0.034)	#SAKNAS!
c57bl6	1	5033 (0.011)	#SAKNAS!	#SAKNAS!
c-634g	1	5033 (0.011)	#SAKNAS!	2492 (0.033)
ca	1	5033 (0.011)	#SAKNAS!	#SAKNAS!
cabbage	1	5033 (0.011)	#SAKNAS!	2492 (0.033)

cadavers	1	5033 (0.011)	#SAKNAS!	2492 (0.033)
caddisflies	1	5033 (0.011)	#SAKNAS!	#SAKNAS!
cafe	1	5033 (0.011)	#SAKNAS!	2492 (0.033)
cafeteria-style	1	5033 (0.011)	#SAKNAS!	2492 (0.033)
caffeine	1	5033 (0.011)	#SAKNAS!	#SAKNAS!
caffeine-mediated	1	5033 (0.011)	#SAKNAS!	2492 (0.033)
cage	1	5033 (0.011)	#SAKNAS!	#SAKNAS!
caged	1	5033 (0.011)	#SAKNAS!	2492 (0.033)
cage-plate	1	5033 (0.011)	#SAKNAS!	#SAKNAS!
calan	1	5033 (0.011)	2293 (0.034)	#SAKNAS!
calanus	1	5033 (0.011)	2293 (0.034)	#SAKNAS!
calcium-phosphate	1	5033 (0.011)	#SAKNAS!	2492 (0.033)
calculating	1	5033 (0.011)	#SAKNAS!	#SAKNAS!
calculi	1	5033 (0.011)	#SAKNAS!	2492 (0.033)
cali	1	5033 (0.011)	2293 (0.034)	#SAKNAS!
calibration	1	5033 (0.011)	2293 (0.034)	#SAKNAS!
caligus	1	5033 (0.011)	#SAKNAS!	2492 (0.033)
callithrix	1	5033 (0.011)	#SAKNAS!	#SAKNAS!
callitrichidae	1	5033 (0.011)	#SAKNAS!	2492 (0.033)
calls	1	5033 (0.011)	#SAKNAS!	#SAKNAS!
caloric-intake	1	5033 (0.011)	2293 (0.034)	#SAKNAS!
calorie-dense	1	5033 (0.011)	#SAKNAS!	2492 (0.033)
calyptraeotheres	1	5033 (0.011)	#SAKNAS!	2492 (0.033)
camara	1	5033 (0.011)	#SAKNAS!	#SAKNAS!
cambodians	1	5033 (0.011)	#SAKNAS!	2492 (0.033)
cambridgeshire	1	5033 (0.011)	#SAKNAS!	2492 (0.033)
camcam	1	5033 (0.011)	#SAKNAS!	#SAKNAS!
camelopardalis	1	5033 (0.011)	#SAKNAS!	2492 (0.033)
camels	1	5033 (0.011)	#SAKNAS!	#SAKNAS!
campaign	1	5033 (0.011)	#SAKNAS!	2492 (0.033)
campaigns	1	5033 (0.011)	#SAKNAS!	#SAKNAS!
campinas	1	5033 (0.011)	2293 (0.034)	#SAKNAS!
camrelizumab-containing	1	5033 (0.011)	#SAKNAS!	2492 (0.033)
canadensis	1	5033 (0.011)	2293 (0.034)	#SAKNAS!
canalized	1	5033 (0.011)	#SAKNAS!	2492 (0.033)
canaria	1	5033 (0.011)	2293 (0.034)	#SAKNAS!
canarian	1	5033 (0.011)	2293 (0.034)	#SAKNAS!
canary	1	5033 (0.011)	2293 (0.034)	#SAKNAS!
cancer-free	1	5033 (0.011)	2293 (0.034)	#SAKNAS!
cancerologia	1	5033 (0.011)	#SAKNAS!	2492 (0.033)
cancer-patients	1	5033 (0.011)	2293 (0.034)	#SAKNAS!
cancrum	1	5033 (0.011)	2293 (0.034)	#SAKNAS!
candidate!	1	5033 (0.011)	2293 (0.034)	#SAKNAS!

canescens	1	5033 (0.011)	2293 (0.034)	#SAKNAS!
canidae	1	5033 (0.011)	2293 (0.034)	#SAKNAS!
canis	1	5033 (0.011)	2293 (0.034)	#SAKNAS!
canna	1	5033 (0.011)	#SAKNAS!	2492 (0.033)
cannabinoid1	1	5033 (0.011)	2293 (0.034)	#SAKNAS!
canned	1	5033 (0.011)	2293 (0.034)	#SAKNAS!
cannibalism	1	5033 (0.011)	2293 (0.034)	#SAKNAS!
cannula	1	5033 (0.011)	#SAKNAS!	#SAKNAS!
cantabricus	1	5033 (0.011)	2293 (0.034)	#SAKNAS!
capabilities	1	5033 (0.011)	#SAKNAS!	#SAKNAS!
capacities	1	5033 (0.011)	#SAKNAS!	#SAKNAS!
capella	1	5033 (0.011)	#SAKNAS!	#SAKNAS!
capensis	1	5033 (0.011)	#SAKNAS!	2492 (0.033)
capillary	1	5033 (0.011)	#SAKNAS!	2492 (0.033)
capnography	1	5033 (0.011)	#SAKNAS!	#SAKNAS!
capsaicin	1	5033 (0.011)	2293 (0.034)	#SAKNAS!
captive-born	1	5033 (0.011)	#SAKNAS!	#SAKNAS!
capuchin	1	5033 (0.011)	2293 (0.034)	#SAKNAS!
carabid	1	5033 (0.011)	#SAKNAS!	#SAKNAS!
carausius	1	5033 (0.011)	2293 (0.034)	#SAKNAS!
carbidopa	1	5033 (0.011)	#SAKNAS!	2492 (0.033)
carbonyl11c	1	5033 (0.011)	2293 (0.034)	#SAKNAS!
carboplatin	1	5033 (0.011)	2293 (0.034)	#SAKNAS!
carboxymethylated	1	5033 (0.011)	#SAKNAS!	#SAKNAS!
carboxymethylcellulose	1	5033 (0.011)	2293 (0.034)	#SAKNAS!
carcharhinidae	1	5033 (0.011)	#SAKNAS!	2492 (0.033)
carcharhiniformes	1	5033 (0.011)	#SAKNAS!	2492 (0.033)
carcinoma-a	1	5033 (0.011)	#SAKNAS!	#SAKNAS!
carcinosarcoma	1	5033 (0.011)	2293 (0.034)	#SAKNAS!
carcinus	1	5033 (0.011)	#SAKNAS!	2492 (0.033)
cardiobacterium	1	5033 (0.011)	2293 (0.034)	#SAKNAS!
cardiomyotomy	1	5033 (0.011)	2293 (0.034)	#SAKNAS!
cardiovascular-high-risk	1	5033 (0.011)	#SAKNAS!	#SAKNAS!
cardui	1	5033 (0.011)	#SAKNAS!	2492 (0.033)
care-a	1	5033 (0.011)	#SAKNAS!	#SAKNAS!
care-dependent	1	5033 (0.011)	#SAKNAS!	2492 (0.033)
carefully	1	5033 (0.011)	#SAKNAS!	2492 (0.033)
caregiver-reported	1	5033 (0.011)	#SAKNAS!	2492 (0.033)
caregivers-intervention	1	5033 (0.011)	#SAKNAS!	#SAKNAS!
caregiving	1	5033 (0.011)	#SAKNAS!	2492 (0.033)
cares-hapin	1	5033 (0.011)	#SAKNAS!	#SAKNAS!
care-specific	1	5033 (0.011)	2293 (0.034)	#SAKNAS!
caretaking	1	5033 (0.011)	#SAKNAS!	2492 (0.033)

caretrack	1	5033 (0.011)	#SAKNAS!	#SAKNAS!
cargos	1	5033 (0.011)	#SAKNAS!	2492 (0.033)
caribbean	1	5033 (0.011)	#SAKNAS!	#SAKNAS!
caries-free	1	5033 (0.011)	#SAKNAS!	#SAKNAS!
caries-reducing	1	5033 (0.011)	2293 (0.034)	#SAKNAS!
caries-risk	1	5033 (0.011)	#SAKNAS!	2492 (0.033)
caries-suspected	1	5033 (0.011)	#SAKNAS!	#SAKNAS!
cariogram	1	5033 (0.011)	2293 (0.034)	#SAKNAS!
carnauba	1	5033 (0.011)	#SAKNAS!	#SAKNAS!
carnea	1	5033 (0.011)	2293 (0.034)	#SAKNAS!
carnitine	1	5033 (0.011)	#SAKNAS!	#SAKNAS!
carnival	1	5033 (0.011)	2293 (0.034)	#SAKNAS!
carnivores	1	5033 (0.011)	2293 (0.034)	#SAKNAS!
carnivorous	1	5033 (0.011)	#SAKNAS!	2492 (0.033)
carolitertii	1	5033 (0.011)	2293 (0.034)	#SAKNAS!
carpio	1	5033 (0.011)	2293 (0.034)	#SAKNAS!
carrier	1	5033 (0.011)	#SAKNAS!	#SAKNAS!
carrier-mediated	1	5033 (0.011)	2293 (0.034)	#SAKNAS!
carriers	1	5033 (0.011)	#SAKNAS!	2492 (0.033)
carrying	1	5033 (0.011)	#SAKNAS!	2492 (0.033)
cascading	1	5033 (0.011)	#SAKNAS!	2492 (0.033)
caseinate	1	5033 (0.011)	#SAKNAS!	2492 (0.033)
case-match	1	5033 (0.011)	2293 (0.034)	#SAKNAS!
caseous	1	5033 (0.011)	#SAKNAS!	#SAKNAS!
case-parent	1	5033 (0.011)	2293 (0.034)	#SAKNAS!
case-report	1	5033 (0.011)	2293 (0.034)	#SAKNAS!
case-reports	1	5033 (0.011)	2293 (0.034)	#SAKNAS!
case-study	1	5033 (0.011)	#SAKNAS!	2492 (0.033)
cash	1	5033 (0.011)	#SAKNAS!	#SAKNAS!
cashew	1	5033 (0.011)	#SAKNAS!	#SAKNAS!
cassava	1	5033 (0.011)	#SAKNAS!	2492 (0.033)
caste	1	5033 (0.011)	2293 (0.034)	#SAKNAS!
castration	1	5033 (0.011)	#SAKNAS!	2492 (0.033)
casts	1	5033 (0.011)	#SAKNAS!	2492 (0.033)
catalonia	1	5033 (0.011)	#SAKNAS!	#SAKNAS!
catarrhine	1	5033 (0.011)	#SAKNAS!	2492 (0.033)
catastrophic	1	5033 (0.011)	#SAKNAS!	#SAKNAS!
catch-up	1	5033 (0.011)	2293 (0.034)	#SAKNAS!
catecholamines	1	5033 (0.011)	#SAKNAS!	2492 (0.033)
categorization	1	5033 (0.011)	#SAKNAS!	#SAKNAS!
categorize	1	5033 (0.011)	#SAKNAS!	2492 (0.033)
catering	1	5033 (0.011)	#SAKNAS!	#SAKNAS!
catherines	1	5033 (0.011)	2293 (0.034)	#SAKNAS!

catus	1	5033 (0.011)	2293 (0.034)	#SAKNAS!
cauca	1	5033 (0.011)	#SAKNAS!	#SAKNAS!
caucasian	1	5033 (0.011)	2293 (0.034)	#SAKNAS!
caudomedial	1	5033 (0.011)	#SAKNAS!	2492 (0.033)
caulophyllum	1	5033 (0.011)	#SAKNAS!	#SAKNAS!
cavities	1	5033 (0.011)	#SAKNAS!	2492 (0.033)
cayo	1	5033 (0.011)	#SAKNAS!	2492 (0.033)
cb1	1	5033 (0.011)	#SAKNAS!	2492 (0.033)
cb1r-	1	5033 (0.011)	#SAKNAS!	2492 (0.033)
cbp-positive	1	5033 (0.011)	#SAKNAS!	#SAKNAS!
cchamide-1	1	5033 (0.011)	#SAKNAS!	#SAKNAS!
cchamide2-receptor	1	5033 (0.011)	#SAKNAS!	#SAKNAS!
cchs	1	5033 (0.011)	#SAKNAS!	#SAKNAS!
cci-779	1	5033 (0.011)	#SAKNAS!	2492 (0.033)
ccks	1	5033 (0.011)	#SAKNAS!	2492 (0.033)
cd4	1	5033 (0.011)	2293 (0.034)	#SAKNAS!
cd4+cell	1	5033 (0.011)	#SAKNAS!	2492 (0.033)
cd8	1	5033 (0.011)	2293 (0.034)	#SAKNAS!
cddp	1	5033 (0.011)	#SAKNAS!	#SAKNAS!
cdh	1	5033 (0.011)	#SAKNAS!	#SAKNAS!
ce	1	5033 (0.011)	#SAKNAS!	2492 (0.033)
ceara	1	5033 (0.011)	2293 (0.034)	#SAKNAS!
cebus	1	5033 (0.011)	2293 (0.034)	#SAKNAS!
cecum	1	5033 (0.011)	2293 (0.034)	#SAKNAS!
cedrelone	1	5033 (0.011)	2293 (0.034)	#SAKNAS!
cell-mediated-immunity	1	5033 (0.011)	2293 (0.034)	#SAKNAS!
cementoblast	1	5033 (0.011)	2293 (0.034)	#SAKNAS!
cementum	1	5033 (0.011)	2293 (0.034)	#SAKNAS!
center-delivered	1	5033 (0.011)	2293 (0.034)	#SAKNAS!
center-reported	1	5033 (0.011)	#SAKNAS!	2492 (0.033)
centipede	1	5033 (0.011)	#SAKNAS!	2492 (0.033)
central-acting	1	5033 (0.011)	#SAKNAS!	2492 (0.033)
centurio	1	5033 (0.011)	#SAKNAS!	2492 (0.033)
cephalopods	1	5033 (0.011)	#SAKNAS!	2492 (0.033)
cephalus	1	5033 (0.011)	2293 (0.034)	#SAKNAS!
cerastoderma-edule	1	5033 (0.011)	2293 (0.034)	#SAKNAS!
cereals	1	5033 (0.011)	#SAKNAS!	2492 (0.033)
cerebellar	1	5033 (0.011)	#SAKNAS!	2492 (0.033)
cerebrospinal-fluid	1	5033 (0.011)	2293 (0.034)	#SAKNAS!
cergas	1	5033 (0.011)	#SAKNAS!	#SAKNAS!
cerrado	1	5033 (0.011)	#SAKNAS!	2492 (0.033)
cervid	1	5033 (0.011)	2293 (0.034)	#SAKNAS!
cervidae	1	5033 (0.011)	2293 (0.034)	#SAKNAS!

cervids	1 5033 (0.011)	#SAKNAS!	2492 (0.033)
cestoda	1 5033 (0.011)	2293 (0.034)	#SAKNAS!
cfa	1 5033 (0.011)	2293 (0.034)	#SAKNAS!
cfq	1 5033 (0.011)	2293 (0.034)	#SAKNAS!
cga	1 5033 (0.011)	#SAKNAS!	#SAKNAS!
chabaudi	1 5033 (0.011)	2293 (0.034)	#SAKNAS!
chaetica	1 5033 (0.011)	#SAKNAS!	2492 (0.033)
chaff-based	1 5033 (0.011)	2293 (0.034)	#SAKNAS!
chair	1 5033 (0.011)	#SAKNAS!	2492 (0.033)
chair-side	1 5033 (0.011)	#SAKNAS!	2492 (0.033)
challenging	1 5033 (0.011)	#SAKNAS!	#SAKNAS!
chamber	1 5033 (0.011)	#SAKNAS!	#SAKNAS!
champ	1 5033 (0.011)	#SAKNAS!	#SAKNAS!
champlain	1 5033 (0.011)	2293 (0.034)	#SAKNAS!
chances	1 5033 (0.011)	#SAKNAS!	2492 (0.033)
change-based	1 5033 (0.011)	2293 (0.034)	#SAKNAS!
chaperone	1 5033 (0.011)	2293 (0.034)	#SAKNAS!
chapter	1 5033 (0.011)	#SAKNAS!	#SAKNAS!
characiforms	1 5033 (0.011)	2293 (0.034)	#SAKNAS!
character	1 5033 (0.011)	2293 (0.034)	#SAKNAS!
chara-fragilis	1 5033 (0.011)	2293 (0.034)	#SAKNAS!
charge	1 5033 (0.011)	#SAKNAS!	2492 (0.033)
charls	1 5033 (0.011)	#SAKNAS!	#SAKNAS!
charm	1 5033 (0.011)	#SAKNAS!	#SAKNAS!
charr	1 5033 (0.011)	2293 (0.034)	#SAKNAS!
cheating	1 5033 (0.011)	#SAKNAS!	2492 (0.033)
check-all-that-apply	1 5033 (0.011)	#SAKNAS!	2492 (0.033)
checklista穢	1 5033 (0.011)	2293 (0.034)	#SAKNAS!
checks	1 5033 (0.011)	#SAKNAS!	2492 (0.033)
check-up	1 5033 (0.011)	#SAKNAS!	#SAKNAS!
cheek	1 5033 (0.011)	2293 (0.034)	#SAKNAS!
cheese	1 5033 (0.011)	2293 (0.034)	#SAKNAS!
cheffia	1 5033 (0.011)	#SAKNAS!	#SAKNAS!
cheilitis	1 5033 (0.011)	2293 (0.034)	#SAKNAS!
chemo-attract	1 5033 (0.011)	#SAKNAS!	#SAKNAS!
chemoboost	1 5033 (0.011)	2293 (0.034)	#SAKNAS!
chemoembolization	1 5033 (0.011)	#SAKNAS!	#SAKNAS!
chemoirradiation	1 5033 (0.011)	2293 (0.034)	#SAKNAS!
chemo-radiation	1 5033 (0.011)	2293 (0.034)	#SAKNAS!
chemoradiation-induced	1 5033 (0.011)	#SAKNAS!	2492 (0.033)
chemotherapeutic	1 5033 (0.011)	#SAKNAS!	2492 (0.033)
chemotherapy-based	1 5033 (0.011)	#SAKNAS!	2492 (0.033)
chest	1 5033 (0.011)	2293 (0.034)	#SAKNAS!

chews	1	5033 (0.011)	#SAKNAS!	2492 (0.033)
chiari	1	5033 (0.011)	2293 (0.034)	#SAKNAS!
chiasmocleis	1	5033 (0.011)	#SAKNAS!	2492 (0.033)
chick	1	5033 (0.011)	#SAKNAS!	2492 (0.033)
chickpea-mealworm	1	5033 (0.011)	#SAKNAS!	#SAKNAS!
chicory	1	5033 (0.011)	#SAKNAS!	2492 (0.033)
child-	1	5033 (0.011)	#SAKNAS!	#SAKNAS!
childcare	1	5033 (0.011)	#SAKNAS!	#SAKNAS!
child-oidp	1	5033 (0.011)	#SAKNAS!	#SAKNAS!
child-oral	1	5033 (0.011)	#SAKNAS!	2492 (0.033)
childrearing	1	5033 (0.011)	#SAKNAS!	2492 (0.033)
child-related	1	5033 (0.011)	#SAKNAS!	#SAKNAS!
children-extent	1	5033 (0.011)	2293 (0.034)	#SAKNAS!
children-sediento	1	5033 (0.011)	#SAKNAS!	#SAKNAS!
children-the	1	5033 (0.011)	#SAKNAS!	2492 (0.033)
child-youth	1	5033 (0.011)	#SAKNAS!	2492 (0.033)
chilensis	1	5033 (0.011)	2293 (0.034)	#SAKNAS!
chimeric	1	5033 (0.011)	#SAKNAS!	#SAKNAS!
chinese-born	1	5033 (0.011)	#SAKNAS!	2492 (0.033)
chinook	1	5033 (0.011)	#SAKNAS!	2492 (0.033)
chipping	1	5033 (0.011)	#SAKNAS!	#SAKNAS!
chirixalus	1	5033 (0.011)	2293 (0.034)	#SAKNAS!
chiroderma	1	5033 (0.011)	#SAKNAS!	2492 (0.033)
chironomids	1	5033 (0.011)	#SAKNAS!	2492 (0.033)
chiroptera	1	5033 (0.011)	#SAKNAS!	2492 (0.033)
chirp	1	5033 (0.011)	#SAKNAS!	#SAKNAS!
chlamydia	1	5033 (0.011)	#SAKNAS!	#SAKNAS!
chlamydomonas	1	5033 (0.011)	2293 (0.034)	#SAKNAS!
chlorella	1	5033 (0.011)	#SAKNAS!	#SAKNAS!
choanoflagellate	1	5033 (0.011)	2293 (0.034)	#SAKNAS!
cholecystectomy	1	5033 (0.011)	2293 (0.034)	#SAKNAS!
cholecystokinin-like	1	5033 (0.011)	#SAKNAS!	#SAKNAS!
cholesterol-rich	1	5033 (0.011)	#SAKNAS!	2492 (0.033)
chongqing	1	5033 (0.011)	#SAKNAS!	2492 (0.033)
choosy	1	5033 (0.011)	#SAKNAS!	2492 (0.033)
choristoneura	1	5033 (0.011)	2293 (0.034)	#SAKNAS!
chorthippus	1	5033 (0.011)	#SAKNAS!	2492 (0.033)
chow	1	5033 (0.011)	#SAKNAS!	#SAKNAS!
chrna5	1	5033 (0.011)	#SAKNAS!	2492 (0.033)
chromatography	1	5033 (0.011)	#SAKNAS!	#SAKNAS!
chronic-alcoholism	1	5033 (0.011)	2293 (0.034)	#SAKNAS!
chronically	1	5033 (0.011)	2293 (0.034)	#SAKNAS!
chronological	1	5033 (0.011)	#SAKNAS!	2492 (0.033)

chronologies	1	5033 (0.011)	#SAKNAS!	#SAKNAS!
chrononutrition	1	5033 (0.011)	#SAKNAS!	#SAKNAS!
chrono-nutrition	1	5033 (0.011)	#SAKNAS!	2492 (0.033)
chrysoperla	1	5033 (0.011)	2293 (0.034)	#SAKNAS!
chrysothamnus	1	5033 (0.011)	2293 (0.034)	#SAKNAS!
chuatsi	1	5033 (0.011)	#SAKNAS!	#SAKNAS!
church	1	5033 (0.011)	2293 (0.034)	#SAKNAS!
chylothorax	1	5033 (0.011)	#SAKNAS!	2492 (0.033)
chymotrypsin-like	1	5033 (0.011)	#SAKNAS!	2492 (0.033)
cichlidae	1	5033 (0.011)	#SAKNAS!	2492 (0.033)
cichlids	1	5033 (0.011)	2293 (0.034)	#SAKNAS!
ciliate	1	5033 (0.011)	2293 (0.034)	#SAKNAS!
ciliatum	1	5033 (0.011)	#SAKNAS!	2492 (0.033)
circuitry	1	5033 (0.011)	#SAKNAS!	2492 (0.033)
circular	1	5033 (0.011)	2293 (0.034)	#SAKNAS!
circumferential	1	5033 (0.011)	2293 (0.034)	#SAKNAS!
circumvent	1	5033 (0.011)	2293 (0.034)	#SAKNAS!
circumventricular	1	5033 (0.011)	2293 (0.034)	#SAKNAS!
cirrhatus	1	5033 (0.011)	#SAKNAS!	2492 (0.033)
cirrhifer	1	5033 (0.011)	#SAKNAS!	#SAKNAS!
cirrhinus	1	5033 (0.011)	2293 (0.034)	#SAKNAS!
cirt	1	5033 (0.011)	#SAKNAS!	2492 (0.033)
cities	1	5033 (0.011)	#SAKNAS!	2492 (0.033)
citizen	1	5033 (0.011)	#SAKNAS!	2492 (0.033)
citizens	1	5033 (0.011)	#SAKNAS!	2492 (0.033)
citric	1	5033 (0.011)	#SAKNAS!	2492 (0.033)
citricidaon	1	5033 (0.011)	#SAKNAS!	2492 (0.033)
city-iraq	1	5033 (0.011)	#SAKNAS!	#SAKNAS!
civilian	1	5033 (0.011)	#SAKNAS!	2492 (0.033)
civilization	1	5033 (0.011)	2293 (0.034)	#SAKNAS!
ckd	1	5033 (0.011)	2293 (0.034)	#SAKNAS!
cladoceran	1	5033 (0.011)	2293 (0.034)	#SAKNAS!
cladodes	1	5033 (0.011)	#SAKNAS!	2492 (0.033)
claims	1	5033 (0.011)	#SAKNAS!	#SAKNAS!
clarity	1	5033 (0.011)	2293 (0.034)	#SAKNAS!
clarki	1	5033 (0.011)	2293 (0.034)	#SAKNAS!
class-based	1	5033 (0.011)	2293 (0.034)	#SAKNAS!
classic	1	5033 (0.011)	2293 (0.034)	#SAKNAS!
classical	1	5033 (0.011)	#SAKNAS!	#SAKNAS!
classifier	1	5033 (0.011)	#SAKNAS!	#SAKNAS!
clay	1	5033 (0.011)	2293 (0.034)	#SAKNAS!
clear	1	5033 (0.011)	#SAKNAS!	2492 (0.033)
clearance	1	5033 (0.011)	2293 (0.034)	#SAKNAS!

clear-water	1	5033 (0.011)	#SAKNAS!	2492 (0.033)
clear簣	1	5033 (0.011)	#SAKNAS!	2492 (0.033)
clem	1	5033 (0.011)	2293 (0.034)	#SAKNAS!
climatic	1	5033 (0.011)	2293 (0.034)	#SAKNAS!
climbing	1	5033 (0.011)	#SAKNAS!	#SAKNAS!
clinical-administrative	1	5033 (0.011)	#SAKNAS!	2492 (0.033)
clinical-geriatric	1	5033 (0.011)	2293 (0.034)	#SAKNAS!
clinico-biochemical	1	5033 (0.011)	#SAKNAS!	#SAKNAS!
clipping	1	5033 (0.011)	2293 (0.034)	#SAKNAS!
clm	1	5033 (0.011)	#SAKNAS!	2492 (0.033)
clock	1	5033 (0.011)	#SAKNAS!	2492 (0.033)
cloi-d-00160r2	1	5033 (0.011)	2293 (0.034)	#SAKNAS!
clonal	1	5033 (0.011)	#SAKNAS!	#SAKNAS!
clonazepam	1	5033 (0.011)	#SAKNAS!	#SAKNAS!
cloning	1	5033 (0.011)	2293 (0.034)	#SAKNAS!
closed-loop	1	5033 (0.011)	#SAKNAS!	2492 (0.033)
closing	1	5033 (0.011)	#SAKNAS!	2492 (0.033)
clotrimazole	1	5033 (0.011)	#SAKNAS!	#SAKNAS!
clover	1	5033 (0.011)	2293 (0.034)	#SAKNAS!
clozapine	1	5033 (0.011)	#SAKNAS!	#SAKNAS!
clozapine-treated	1	5033 (0.011)	#SAKNAS!	#SAKNAS!
clsa	1	5033 (0.011)	#SAKNAS!	#SAKNAS!
cluj-napoca	1	5033 (0.011)	#SAKNAS!	2492 (0.033)
clupeiform	1	5033 (0.011)	2293 (0.034)	#SAKNAS!
cluster-randomised	1	5033 (0.011)	#SAKNAS!	2492 (0.033)
cnn	1	5033 (0.011)	#SAKNAS!	2492 (0.033)
cnr1	1	5033 (0.011)	#SAKNAS!	2492 (0.033)
cns	1	5033 (0.011)	#SAKNAS!	#SAKNAS!
cntf	1	5033 (0.011)	2293 (0.034)	#SAKNAS!
co2concentration	1	5033 (0.011)	#SAKNAS!	2492 (0.033)
coadministration	1	5033 (0.011)	2293 (0.034)	#SAKNAS!
coarse	1	5033 (0.011)	2293 (0.034)	#SAKNAS!
coccidial	1	5033 (0.011)	2293 (0.034)	#SAKNAS!
coccidiosis	1	5033 (0.011)	2293 (0.034)	#SAKNAS!
coccoides	1	5033 (0.011)	2293 (0.034)	#SAKNAS!
cockle	1	5033 (0.011)	2293 (0.034)	#SAKNAS!
cockles	1	5033 (0.011)	2293 (0.034)	#SAKNAS!
cockroach-specific	1	5033 (0.011)	#SAKNAS!	2492 (0.033)
cocoa	1	5033 (0.011)	#SAKNAS!	#SAKNAS!
co-crystallized	1	5033 (0.011)	2293 (0.034)	#SAKNAS!
co-design	1	5033 (0.011)	#SAKNAS!	#SAKNAS!
codesigned	1	5033 (0.011)	#SAKNAS!	#SAKNAS!
coding	1	5033 (0.011)	#SAKNAS!	2492 (0.033)

coefficient	1	5033 (0.011)	#SAKNAS!	#SAKNAS!
coefficients	1	5033 (0.011)	2293 (0.034)	#SAKNAS!
coeliac	1	5033 (0.011)	#SAKNAS!	2492 (0.033)
coenzyme	1	5033 (0.011)	#SAKNAS!	2492 (0.033)
coevolution	1	5033 (0.011)	#SAKNAS!	#SAKNAS!
coexistence	1	5033 (0.011)	#SAKNAS!	#SAKNAS!
co-existence	1	5033 (0.011)	#SAKNAS!	#SAKNAS!
coexisting	1	5033 (0.011)	#SAKNAS!	#SAKNAS!
cofactors	1	5033 (0.011)	#SAKNAS!	2492 (0.033)
cognate	1	5033 (0.011)	#SAKNAS!	#SAKNAS!
cognitively	1	5033 (0.011)	2293 (0.034)	#SAKNAS!
coherence	1	5033 (0.011)	#SAKNAS!	2492 (0.033)
cohesion	1	5033 (0.011)	2293 (0.034)	#SAKNAS!
coho	1	5033 (0.011)	#SAKNAS!	2492 (0.033)
c-oidp	1	5033 (0.011)	#SAKNAS!	2492 (0.033)
coincident	1	5033 (0.011)	2293 (0.034)	#SAKNAS!
coix	1	5033 (0.011)	#SAKNAS!	2492 (0.033)
cola	1	5033 (0.011)	2293 (0.034)	#SAKNAS!
cold	1	5033 (0.011)	#SAKNAS!	2492 (0.033)
coldent	1	5033 (0.011)	#SAKNAS!	#SAKNAS!
cold-stressed	1	5033 (0.011)	#SAKNAS!	#SAKNAS!
cold-water	1	5033 (0.011)	#SAKNAS!	2492 (0.033)
coleopteran	1	5033 (0.011)	2293 (0.034)	#SAKNAS!
colitis	1	5033 (0.011)	#SAKNAS!	2492 (0.033)
colius	1	5033 (0.011)	#SAKNAS!	2492 (0.033)
collagen	1	5033 (0.011)	#SAKNAS!	#SAKNAS!
collapsibility	1	5033 (0.011)	2293 (0.034)	#SAKNAS!
collar	1	5033 (0.011)	2293 (0.034)	#SAKNAS!
collection	1	5033 (0.011)	#SAKNAS!	2492 (0.033)
collector-inductively	1	5033 (0.011)	#SAKNAS!	2492 (0.033)
collembola	1	5033 (0.011)	2293 (0.034)	#SAKNAS!
collembolan	1	5033 (0.011)	#SAKNAS!	#SAKNAS!
collider	1	5033 (0.011)	#SAKNAS!	2492 (0.033)
collier	1	5033 (0.011)	2293 (0.034)	#SAKNAS!
collins	1	5033 (0.011)	#SAKNAS!	#SAKNAS!
collis-nissen	1	5033 (0.011)	#SAKNAS!	2492 (0.033)
co-localized	1	5033 (0.011)	#SAKNAS!	#SAKNAS!
colocalizes	1	5033 (0.011)	#SAKNAS!	#SAKNAS!
colony-stimulating	1	5033 (0.011)	2293 (0.034)	#SAKNAS!
colorful	1	5033 (0.011)	#SAKNAS!	#SAKNAS!
colour-contrast	1	5033 (0.011)	#SAKNAS!	#SAKNAS!
colourimetric	1	5033 (0.011)	#SAKNAS!	2492 (0.033)
columbia	1	5033 (0.011)	2293 (0.034)	#SAKNAS!

combined-modality	1	5033 (0.011)	2293 (0.034)	#SAKNAS!
comfortability	1	5033 (0.011)	#SAKNAS!	2492 (0.033)
comfortable	1	5033 (0.011)	#SAKNAS!	2492 (0.033)
comfort-focused	1	5033 (0.011)	#SAKNAS!	#SAKNAS!
co-mimics	1	5033 (0.011)	#SAKNAS!	#SAKNAS!
commercially	1	5033 (0.011)	#SAKNAS!	2492 (0.033)
commercially-prepared	1	5033 (0.011)	#SAKNAS!	2492 (0.033)
commercials	1	5033 (0.011)	2293 (0.034)	#SAKNAS!
commission	1	5033 (0.011)	#SAKNAS!	2492 (0.033)
commoner	1	5033 (0.011)	2293 (0.034)	#SAKNAS!
commonly	1	5033 (0.011)	#SAKNAS!	2492 (0.033)
communal	1	5033 (0.011)	2293 (0.034)	#SAKNAS!
communicable	1	5033 (0.011)	#SAKNAS!	2492 (0.033)
community-hospital	1	5033 (0.011)	2293 (0.034)	#SAKNAS!
community-supported	1	5033 (0.011)	#SAKNAS!	#SAKNAS!
co-morbidities	1	5033 (0.011)	2293 (0.034)	#SAKNAS!
companions	1	5033 (0.011)	#SAKNAS!	#SAKNAS!
company	1	5033 (0.011)	#SAKNAS!	#SAKNAS!
compelling	1	5033 (0.011)	#SAKNAS!	2492 (0.033)
compensates	1	5033 (0.011)	2293 (0.034)	#SAKNAS!
compensating	1	5033 (0.011)	2293 (0.034)	#SAKNAS!
competency	1	5033 (0.011)	#SAKNAS!	2492 (0.033)
competent	1	5033 (0.011)	#SAKNAS!	2492 (0.033)
competitively	1	5033 (0.011)	2293 (0.034)	#SAKNAS!
competitors	1	5033 (0.011)	#SAKNAS!	#SAKNAS!
complaining	1	5033 (0.011)	#SAKNAS!	2492 (0.033)
completely	1	5033 (0.011)	#SAKNAS!	#SAKNAS!
completing	1	5033 (0.011)	2293 (0.034)	#SAKNAS!
complexing	1	5033 (0.011)	2293 (0.034)	#SAKNAS!
compliant	1	5033 (0.011)	#SAKNAS!	#SAKNAS!
complimentary	1	5033 (0.011)	#SAKNAS!	2492 (0.033)
component-specific	1	5033 (0.011)	#SAKNAS!	2492 (0.033)
composites	1	5033 (0.011)	#SAKNAS!	#SAKNAS!
composites-based	1	5033 (0.011)	#SAKNAS!	#SAKNAS!
comprising	1	5033 (0.011)	#SAKNAS!	#SAKNAS!
compromise	1	5033 (0.011)	#SAKNAS!	2492 (0.033)
comunitaria	1	5033 (0.011)	#SAKNAS!	2492 (0.033)
concepcion	1	5033 (0.011)	2293 (0.034)	#SAKNAS!
conception	1	5033 (0.011)	2293 (0.034)	#SAKNAS!
conceptual	1	5033 (0.011)	#SAKNAS!	2492 (0.033)
conch	1	5033 (0.011)	2293 (0.034)	#SAKNAS!
concomitants	1	5033 (0.011)	2293 (0.034)	#SAKNAS!
concordance	1	5033 (0.011)	#SAKNAS!	#SAKNAS!

concordant	1	5033 (0.011)	#SAKNAS!	2492 (0.033)
concurrently	1	5033 (0.011)	#SAKNAS!	#SAKNAS!
conductance	1	5033 (0.011)	2293 (0.034)	#SAKNAS!
conductive	1	5033 (0.011)	#SAKNAS!	#SAKNAS!
confectioneries	1	5033 (0.011)	#SAKNAS!	#SAKNAS!
configurations	1	5033 (0.011)	#SAKNAS!	#SAKNAS!
confirm	1	5033 (0.011)	#SAKNAS!	2492 (0.033)
confirming	1	5033 (0.011)	#SAKNAS!	2492 (0.033)
confirms	1	5033 (0.011)	#SAKNAS!	#SAKNAS!
conflict	1	5033 (0.011)	#SAKNAS!	#SAKNAS!
conform	1	5033 (0.011)	#SAKNAS!	2492 (0.033)
confounders	1	5033 (0.011)	#SAKNAS!	2492 (0.033)
confusum	1	5033 (0.011)	2293 (0.034)	#SAKNAS!
congregate	1	5033 (0.011)	#SAKNAS!	#SAKNAS!
congress	1	5033 (0.011)	#SAKNAS!	#SAKNAS!
conifers	1	5033 (0.011)	#SAKNAS!	2492 (0.033)
conjunction	1	5033 (0.011)	#SAKNAS!	#SAKNAS!
connection	1	5033 (0.011)	#SAKNAS!	2492 (0.033)
connections	1	5033 (0.011)	#SAKNAS!	#SAKNAS!
connective	1	5033 (0.011)	2293 (0.034)	#SAKNAS!
connexin	1	5033 (0.011)	#SAKNAS!	2492 (0.033)
conotrachelus	1	5033 (0.011)	2293 (0.034)	#SAKNAS!
consciousness	1	5033 (0.011)	#SAKNAS!	#SAKNAS!
conscripts	1	5033 (0.011)	#SAKNAS!	2492 (0.033)
consideration	1	5033 (0.011)	2293 (0.034)	#SAKNAS!
consistencies	1	5033 (0.011)	#SAKNAS!	2492 (0.033)
consistent	1	5033 (0.011)	2293 (0.034)	#SAKNAS!
consistometer	1	5033 (0.011)	#SAKNAS!	2492 (0.033)
consortium	1	5033 (0.011)	#SAKNAS!	2492 (0.033)
constellatus	1	5033 (0.011)	#SAKNAS!	#SAKNAS!
constipation	1	5033 (0.011)	#SAKNAS!	2492 (0.033)
constituent	1	5033 (0.011)	#SAKNAS!	2492 (0.033)
constitution	1	5033 (0.011)	#SAKNAS!	#SAKNAS!
constitutional	1	5033 (0.011)	#SAKNAS!	2492 (0.033)
constitutionally	1	5033 (0.011)	2293 (0.034)	#SAKNAS!
constraining	1	5033 (0.011)	#SAKNAS!	#SAKNAS!
constrains	1	5033 (0.011)	#SAKNAS!	2492 (0.033)
constraint	1	5033 (0.011)	2293 (0.034)	#SAKNAS!
constriction	1	5033 (0.011)	2293 (0.034)	#SAKNAS!
constrictotermes	1	5033 (0.011)	#SAKNAS!	#SAKNAS!
construction	1	5033 (0.011)	#SAKNAS!	#SAKNAS!
consult	1	5033 (0.011)	#SAKNAS!	2492 (0.033)
consultants	1	5033 (0.011)	2293 (0.034)	#SAKNAS!

consultation	1	5033 (0.011)	2293 (0.034)	#SAKNAS!
consumer-demand	1	5033 (0.011)	#SAKNAS!	2492 (0.033)
contact	1	5033 (0.011)	#SAKNAS!	2492 (0.033)
contacts	1	5033 (0.011)	#SAKNAS!	2492 (0.033)
contains	1	5033 (0.011)	2293 (0.034)	#SAKNAS!
contest	1	5033 (0.011)	#SAKNAS!	#SAKNAS!
contextual	1	5033 (0.011)	2293 (0.034)	#SAKNAS!
continent	1	5033 (0.011)	#SAKNAS!	2492 (0.033)
contingent	1	5033 (0.011)	#SAKNAS!	#SAKNAS!
contortus	1	5033 (0.011)	#SAKNAS!	2492 (0.033)
contra	1	5033 (0.011)	#SAKNAS!	2492 (0.033)
contraceptives	1	5033 (0.011)	2293 (0.034)	#SAKNAS!
contract	1	5033 (0.011)	#SAKNAS!	2492 (0.033)
contralateral	1	5033 (0.011)	#SAKNAS!	#SAKNAS!
contrast	1	5033 (0.011)	2293 (0.034)	#SAKNAS!
contributor	1	5033 (0.011)	#SAKNAS!	#SAKNAS!
conut	1	5033 (0.011)	#SAKNAS!	#SAKNAS!
convalescence	1	5033 (0.011)	2293 (0.034)	#SAKNAS!
convergent	1	5033 (0.011)	#SAKNAS!	#SAKNAS!
conversation	1	5033 (0.011)	#SAKNAS!	#SAKNAS!
conversations	1	5033 (0.011)	#SAKNAS!	#SAKNAS!
conversional	1	5033 (0.011)	#SAKNAS!	#SAKNAS!
conversions	1	5033 (0.011)	#SAKNAS!	#SAKNAS!
co-occurring	1	5033 (0.011)	#SAKNAS!	#SAKNAS!
coordinate	1	5033 (0.011)	#SAKNAS!	2492 (0.033)
coots	1	5033 (0.011)	#SAKNAS!	2492 (0.033)
copenhagen	1	5033 (0.011)	2293 (0.034)	#SAKNAS!
coprophagic	1	5033 (0.011)	2293 (0.034)	#SAKNAS!
copula	1	5033 (0.011)	#SAKNAS!	#SAKNAS!
corallivorous	1	5033 (0.011)	2293 (0.034)	#SAKNAS!
co-receptor	1	5033 (0.011)	#SAKNAS!	2492 (0.033)
coriacea	1	5033 (0.011)	2293 (0.034)	#SAKNAS!
cornelia	1	5033 (0.011)	#SAKNAS!	#SAKNAS!
cornerstone	1	5033 (0.011)	#SAKNAS!	2492 (0.033)
corn-silage	1	5033 (0.011)	2293 (0.034)	#SAKNAS!
corn-soy	1	5033 (0.011)	#SAKNAS!	2492 (0.033)
coronally	1	5033 (0.011)	2293 (0.034)	#SAKNAS!
coronata	1	5033 (0.011)	#SAKNAS!	#SAKNAS!
coronatus	1	5033 (0.011)	2293 (0.034)	#SAKNAS!
corps	1	5033 (0.011)	#SAKNAS!	#SAKNAS!
correcting	1	5033 (0.011)	#SAKNAS!	#SAKNAS!
correlating	1	5033 (0.011)	#SAKNAS!	2492 (0.033)
correlational	1	5033 (0.011)	#SAKNAS!	#SAKNAS!

corresponding	1	5033 (0.011)	#SAKNAS!	2492 (0.033)
corriedale	1	5033 (0.011)	#SAKNAS!	2492 (0.033)
corrosive-induced	1	5033 (0.011)	2293 (0.034)	#SAKNAS!
corticotropin-releasing	1	5033 (0.011)	2293 (0.034)	#SAKNAS!
cory	1	5033 (0.011)	2293 (0.034)	#SAKNAS!
coryphaena	1	5033 (0.011)	#SAKNAS!	2492 (0.033)
cosmioides	1	5033 (0.011)	#SAKNAS!	2492 (0.033)
costa	1	5033 (0.011)	#SAKNAS!	2492 (0.033)
cost-offset	1	5033 (0.011)	#SAKNAS!	#SAKNAS!
cost-of-living	1	5033 (0.011)	#SAKNAS!	#SAKNAS!
coton	1	5033 (0.011)	#SAKNAS!	#SAKNAS!
counsellors	1	5033 (0.011)	#SAKNAS!	2492 (0.033)
counselors	1	5033 (0.011)	#SAKNAS!	#SAKNAS!
counties	1	5033 (0.011)	2293 (0.034)	#SAKNAS!
counting	1	5033 (0.011)	#SAKNAS!	2492 (0.033)
county-level	1	5033 (0.011)	#SAKNAS!	2492 (0.033)
courses	1	5033 (0.011)	2293 (0.034)	#SAKNAS!
courtship	1	5033 (0.011)	2293 (0.034)	#SAKNAS!
covid-19-associated	1	5033 (0.011)	#SAKNAS!	#SAKNAS!
covid-19-induced	1	5033 (0.011)	#SAKNAS!	#SAKNAS!
coyoacan	1	5033 (0.011)	#SAKNAS!	#SAKNAS!
cpeo	1	5033 (0.011)	#SAKNAS!	2492 (0.033)
c-peptide	1	5033 (0.011)	#SAKNAS!	#SAKNAS!
cpi	1	5033 (0.011)	#SAKNAS!	#SAKNAS!
cpne7	1	5033 (0.011)	#SAKNAS!	#SAKNAS!
cpoe	1	5033 (0.011)	#SAKNAS!	2492 (0.033)
crack	1	5033 (0.011)	#SAKNAS!	#SAKNAS!
cranberries	1	5033 (0.011)	#SAKNAS!	2492 (0.033)
craniocerebral	1	5033 (0.011)	#SAKNAS!	#SAKNAS!
craniofacial	1	5033 (0.011)	#SAKNAS!	#SAKNAS!
crassifolia	1	5033 (0.011)	2293 (0.034)	#SAKNAS!
craving	1	5033 (0.011)	#SAKNAS!	2492 (0.033)
cravings	1	5033 (0.011)	#SAKNAS!	#SAKNAS!
crayfish	1	5033 (0.011)	2293 (0.034)	#SAKNAS!
creating	1	5033 (0.011)	2293 (0.034)	#SAKNAS!
creatinine	1	5033 (0.011)	#SAKNAS!	#SAKNAS!
creation	1	5033 (0.011)	#SAKNAS!	2492 (0.033)
creativity	1	5033 (0.011)	#SAKNAS!	#SAKNAS!
cree	1	5033 (0.011)	#SAKNAS!	2492 (0.033)
crenata	1	5033 (0.011)	2293 (0.034)	#SAKNAS!
crested	1	5033 (0.011)	#SAKNAS!	2492 (0.033)
crf	1	5033 (0.011)	2293 (0.034)	#SAKNAS!
cribroelphidium	1	5033 (0.011)	#SAKNAS!	#SAKNAS!

cricohyoidoepiglottopexy	1	5033 (0.011)	2293 (0.034)	#SAKNAS!
crisis	1	5033 (0.011)	#SAKNAS!	#SAKNAS!
crisponi	1	5033 (0.011)	#SAKNAS!	#SAKNAS!
crispula	1	5033 (0.011)	2293 (0.034)	#SAKNAS!
croatia	1	5033 (0.011)	2293 (0.034)	#SAKNAS!
crohn	1	5033 (0.011)	#SAKNAS!	2492 (0.033)
crops	1	5033 (0.011)	#SAKNAS!	2492 (0.033)
crosnieri	1	5033 (0.011)	#SAKNAS!	2492 (0.033)
crossbreeding	1	5033 (0.011)	#SAKNAS!	2492 (0.033)
cross-country	1	5033 (0.011)	#SAKNAS!	2492 (0.033)
crosse	1	5033 (0.011)	#SAKNAS!	2492 (0.033)
cross-examining	1	5033 (0.011)	#SAKNAS!	#SAKNAS!
cross-intestinal	1	5033 (0.011)	2293 (0.034)	#SAKNAS!
crosssectional	1	5033 (0.011)	#SAKNAS!	#SAKNAS!
cross-sucking	1	5033 (0.011)	2293 (0.034)	#SAKNAS!
cross-trait	1	5033 (0.011)	#SAKNAS!	#SAKNAS!
crushing	1	5033 (0.011)	#SAKNAS!	2492 (0.033)
cryotherapy	1	5033 (0.011)	#SAKNAS!	2492 (0.033)
crystalline	1	5033 (0.011)	#SAKNAS!	#SAKNAS!
csn1s1	1	5033 (0.011)	2293 (0.034)	#SAKNAS!
ctenopharyngodon	1	5033 (0.011)	#SAKNAS!	2492 (0.033)
c-terminal	1	5033 (0.011)	#SAKNAS!	2492 (0.033)
ct-guided	1	5033 (0.011)	2293 (0.034)	#SAKNAS!
cuba	1	5033 (0.011)	2293 (0.034)	#SAKNAS!
cucumaria	1	5033 (0.011)	#SAKNAS!	2492 (0.033)
culebras	1	5033 (0.011)	#SAKNAS!	#SAKNAS!
culex	1	5033 (0.011)	#SAKNAS!	2492 (0.033)
cull	1	5033 (0.011)	#SAKNAS!	#SAKNAS!
culpaeus	1	5033 (0.011)	2293 (0.034)	#SAKNAS!
culprits	1	5033 (0.011)	#SAKNAS!	2492 (0.033)
culturally	1	5033 (0.011)	#SAKNAS!	#SAKNAS!
culture-independent	1	5033 (0.011)	2293 (0.034)	#SAKNAS!
cuminum	1	5033 (0.011)	#SAKNAS!	#SAKNAS!
cumulative	1	5033 (0.011)	2293 (0.034)	#SAKNAS!
cuniculus	1	5033 (0.011)	2293 (0.034)	#SAKNAS!
curculionidae	1	5033 (0.011)	2293 (0.034)	#SAKNAS!
curcumin	1	5033 (0.011)	#SAKNAS!	#SAKNAS!
curdlan	1	5033 (0.011)	#SAKNAS!	#SAKNAS!
cure	1	5033 (0.011)	#SAKNAS!	2492 (0.033)
currently	1	5033 (0.011)	#SAKNAS!	#SAKNAS!
currents	1	5033 (0.011)	2293 (0.034)	#SAKNAS!
curricular	1	5033 (0.011)	#SAKNAS!	2492 (0.033)
curry	1	5033 (0.011)	#SAKNAS!	#SAKNAS!

curtipennis	1	5033 (0.011)	#SAKNAS!	2492 (0.033)
curves	1	5033 (0.011)	2293 (0.034)	#SAKNAS!
customised	1	5033 (0.011)	#SAKNAS!	2492 (0.033)
customization	1	5033 (0.011)	#SAKNAS!	#SAKNAS!
cutoff	1	5033 (0.011)	#SAKNAS!	2492 (0.033)
cut-off	1	5033 (0.011)	#SAKNAS!	#SAKNAS!
cuts	1	5033 (0.011)	2293 (0.034)	#SAKNAS!
cuttlefish	1	5033 (0.011)	2293 (0.034)	#SAKNAS!
cutworm	1	5033 (0.011)	2293 (0.034)	#SAKNAS!
cyanobacteria	1	5033 (0.011)	2293 (0.034)	#SAKNAS!
cyanobacterial	1	5033 (0.011)	#SAKNAS!	2492 (0.033)
cyanogen	1	5033 (0.011)	2293 (0.034)	#SAKNAS!
cyclase-activating	1	5033 (0.011)	#SAKNAS!	#SAKNAS!
cyclosporine	1	5033 (0.011)	2293 (0.034)	#SAKNAS!
cyminum	1	5033 (0.011)	#SAKNAS!	#SAKNAS!
cynomolgus	1	5033 (0.011)	#SAKNAS!	#SAKNAS!
cyphergaster	1	5033 (0.011)	#SAKNAS!	#SAKNAS!
cypridopsis-vidua	1	5033 (0.011)	2293 (0.034)	#SAKNAS!
cyprinidae	1	5033 (0.011)	2293 (0.034)	#SAKNAS!
cypriniformes	1	5033 (0.011)	2293 (0.034)	#SAKNAS!
cyprinus	1	5033 (0.011)	2293 (0.034)	#SAKNAS!
cyrtonema	1	5033 (0.011)	#SAKNAS!	#SAKNAS!
cystatin	1	5033 (0.011)	#SAKNAS!	#SAKNAS!
cystic-fibrosis	1	5033 (0.011)	2293 (0.034)	#SAKNAS!
cystinosis	1	5033 (0.011)	2293 (0.034)	#SAKNAS!
cytokinin-mediated	1	5033 (0.011)	2293 (0.034)	#SAKNAS!
cytomegalovirus	1	5033 (0.011)	#SAKNAS!	#SAKNAS!
da	1	5033 (0.011)	#SAKNAS!	#SAKNAS!
dairy-products	1	5033 (0.011)	2293 (0.034)	#SAKNAS!
damages	1	5033 (0.011)	#SAKNAS!	#SAKNAS!
dams	1	5033 (0.011)	#SAKNAS!	2492 (0.033)
damselfishes	1	5033 (0.011)	#SAKNAS!	2492 (0.033)
danazol	1	5033 (0.011)	2293 (0.034)	#SAKNAS!
dancers	1	5033 (0.011)	2293 (0.034)	#SAKNAS!
danio	1	5033 (0.011)	2293 (0.034)	#SAKNAS!
daphnia-ambigua	1	5033 (0.011)	2293 (0.034)	#SAKNAS!
dar	1	5033 (0.011)	2293 (0.034)	#SAKNAS!
darkness	1	5033 (0.011)	#SAKNAS!	#SAKNAS!
darwini	1	5033 (0.011)	2293 (0.034)	#SAKNAS!
dash	1	5033 (0.011)	#SAKNAS!	2492 (0.033)
dashtestan	1	5033 (0.011)	2293 (0.034)	#SAKNAS!
dassie-rat	1	5033 (0.011)	2293 (0.034)	#SAKNAS!
data2010	1	5033 (0.011)	#SAKNAS!	2492 (0.033)

data-base	1	5033 (0.011)	2293 (0.034)	#SAKNAS!
data-driven	1	5033 (0.011)	#SAKNAS!	#SAKNAS!
date	1	5033 (0.011)	#SAKNAS!	#SAKNAS!
daubentonia-madagascariensis	1	5033 (0.011)	2293 (0.034)	#SAKNAS!
daughters	1	5033 (0.011)	2293 (0.034)	#SAKNAS!
davidi	1	5033 (0.011)	#SAKNAS!	2492 (0.033)
dawley-national	1	5033 (0.011)	#SAKNAS!	#SAKNAS!
day-care	1	5033 (0.011)	2293 (0.034)	#SAKNAS!
daylong	1	5033 (0.011)	#SAKNAS!	#SAKNAS!
daytime-only	1	5033 (0.011)	#SAKNAS!	2492 (0.033)
d-binding	1	5033 (0.011)	#SAKNAS!	2492 (0.033)
dds	1	5033 (0.011)	#SAKNAS!	#SAKNAS!
debris	1	5033 (0.011)	#SAKNAS!	2492 (0.033)
decarboxylase	1	5033 (0.011)	2293 (0.034)	#SAKNAS!
deceleration	1	5033 (0.011)	2293 (0.034)	#SAKNAS!
decision-makers	1	5033 (0.011)	2293 (0.034)	#SAKNAS!
decussatus	1	5033 (0.011)	2293 (0.034)	#SAKNAS!
de-escalated	1	5033 (0.011)	#SAKNAS!	2492 (0.033)
de-escalation	1	5033 (0.011)	#SAKNAS!	2492 (0.033)
defb1	1	5033 (0.011)	#SAKNAS!	#SAKNAS!
defecation	1	5033 (0.011)	2293 (0.034)	#SAKNAS!
defence	1	5033 (0.011)	2293 (0.034)	#SAKNAS!
defensive	1	5033 (0.011)	#SAKNAS!	2492 (0.033)
definable	1	5033 (0.011)	2293 (0.034)	#SAKNAS!
deformities	1	5033 (0.011)	2293 (0.034)	#SAKNAS!
defrosted	1	5033 (0.011)	#SAKNAS!	#SAKNAS!
degenerative	1	5033 (0.011)	2293 (0.034)	#SAKNAS!
deglutitive	1	5033 (0.011)	#SAKNAS!	#SAKNAS!
delavayi	1	5033 (0.011)	#SAKNAS!	#SAKNAS!
delays	1	5033 (0.011)	#SAKNAS!	#SAKNAS!
deletions	1	5033 (0.011)	#SAKNAS!	2492 (0.033)
delhi	1	5033 (0.011)	#SAKNAS!	2492 (0.033)
deliberations	1	5033 (0.011)	#SAKNAS!	2492 (0.033)
delta	1	5033 (0.011)	2293 (0.034)	#SAKNAS!
demeester	1	5033 (0.011)	#SAKNAS!	#SAKNAS!
dementia-an	1	5033 (0.011)	#SAKNAS!	#SAKNAS!
demographics	1	5033 (0.011)	2293 (0.034)	#SAKNAS!
demonstrates	1	5033 (0.011)	#SAKNAS!	2492 (0.033)
demonstrating	1	5033 (0.011)	#SAKNAS!	2492 (0.033)
demyelination	1	5033 (0.011)	#SAKNAS!	#SAKNAS!
dendrobatidae	1	5033 (0.011)	#SAKNAS!	#SAKNAS!
denervation	1	5033 (0.011)	#SAKNAS!	#SAKNAS!
dengue	1	5033 (0.011)	#SAKNAS!	#SAKNAS!

denies	1	5033 (0.011)	2293 (0.034)	#SAKNAS!
denosumab-related	1	5033 (0.011)	#SAKNAS!	2492 (0.033)
dense	1	5033 (0.011)	2293 (0.034)	#SAKNAS!
densities	1	5033 (0.011)	2293 (0.034)	#SAKNAS!
density-dependent	1	5033 (0.011)	#SAKNAS!	2492 (0.033)
dentaisez	1	5033 (0.011)	#SAKNAS!	#SAKNAS!
dental-health	1	5033 (0.011)	2293 (0.034)	#SAKNAS!
dentally	1	5033 (0.011)	#SAKNAS!	2492 (0.033)
dentisits	1	5033 (0.011)	#SAKNAS!	#SAKNAS!
dento-alveolar	1	5033 (0.011)	2293 (0.034)	#SAKNAS!
dentognathic	1	5033 (0.011)	#SAKNAS!	2492 (0.033)
denture-wearing	1	5033 (0.011)	#SAKNAS!	2492 (0.033)
deployment	1	5033 (0.011)	#SAKNAS!	2492 (0.033)
deposit-feeder	1	5033 (0.011)	2293 (0.034)	#SAKNAS!
deposition	1	5033 (0.011)	#SAKNAS!	2492 (0.033)
depot	1	5033 (0.011)	2293 (0.034)	#SAKNAS!
depress	1	5033 (0.011)	2293 (0.034)	#SAKNAS!
der	1	5033 (0.011)	2293 (0.034)	#SAKNAS!
derivatives	1	5033 (0.011)	2293 (0.034)	#SAKNAS!
dermis	1	5033 (0.011)	2293 (0.034)	#SAKNAS!
deroceras	1	5033 (0.011)	2293 (0.034)	#SAKNAS!
des	1	5033 (0.011)	2293 (0.034)	#SAKNAS!
descending	1	5033 (0.011)	#SAKNAS!	2492 (0.033)
describing	1	5033 (0.011)	#SAKNAS!	2492 (0.033)
desipramine	1	5033 (0.011)	2293 (0.034)	#SAKNAS!
desmarest	1	5033 (0.011)	2293 (0.034)	#SAKNAS!
desmarestii	1	5033 (0.011)	#SAKNAS!	2492 (0.033)
despite	1	5033 (0.011)	#SAKNAS!	2492 (0.033)
desserts	1	5033 (0.011)	2293 (0.034)	#SAKNAS!
destination	1	5033 (0.011)	#SAKNAS!	#SAKNAS!
destiny	1	5033 (0.011)	#SAKNAS!	2492 (0.033)
destruction	1	5033 (0.011)	2293 (0.034)	#SAKNAS!
desynchronization	1	5033 (0.011)	#SAKNAS!	#SAKNAS!
detention	1	5033 (0.011)	#SAKNAS!	#SAKNAS!
deteriorating	1	5033 (0.011)	#SAKNAS!	#SAKNAS!
deters	1	5033 (0.011)	#SAKNAS!	2492 (0.033)
detoxification	1	5033 (0.011)	2293 (0.034)	#SAKNAS!
detritivores	1	5033 (0.011)	#SAKNAS!	#SAKNAS!
detritivorous	1	5033 (0.011)	#SAKNAS!	#SAKNAS!
deuterium	1	5033 (0.011)	2293 (0.034)	#SAKNAS!
deutetrabenazine	1	5033 (0.011)	#SAKNAS!	2492 (0.033)
deutsche	1	5033 (0.011)	#SAKNAS!	2492 (0.033)
devaluation	1	5033 (0.011)	2293 (0.034)	#SAKNAS!

developmentally	1	5033 (0.011)	2293 (0.034)	#SAKNAS!
development-third	1	5033 (0.011)	2293 (0.034)	#SAKNAS!
deviate	1	5033 (0.011)	2293 (0.034)	#SAKNAS!
device-assisted	1	5033 (0.011)	#SAKNAS!	#SAKNAS!
device-facilitated	1	5033 (0.011)	#SAKNAS!	2492 (0.033)
dexamethasone	1	5033 (0.011)	#SAKNAS!	#SAKNAS!
dexamethasone-treatment	1	5033 (0.011)	#SAKNAS!	2492 (0.033)
d-galactose	1	5033 (0.011)	#SAKNAS!	#SAKNAS!
dgem	1	5033 (0.011)	2293 (0.034)	#SAKNAS!
dgg	1	5033 (0.011)	2293 (0.034)	#SAKNAS!
dgkj	1	5033 (0.011)	#SAKNAS!	2492 (0.033)
diabetes-oral	1	5033 (0.011)	#SAKNAS!	#SAKNAS!
diabetes-periodontitis	1	5033 (0.011)	#SAKNAS!	#SAKNAS!
diabetes-specific	1	5033 (0.011)	#SAKNAS!	#SAKNAS!
diabetic-friendly	1	5033 (0.011)	#SAKNAS!	#SAKNAS!
diabetologists	1	5033 (0.011)	#SAKNAS!	#SAKNAS!
diabetology	1	5033 (0.011)	#SAKNAS!	#SAKNAS!
diabrotica	1	5033 (0.011)	2293 (0.034)	#SAKNAS!
diadema	1	5033 (0.011)	2293 (0.034)	#SAKNAS!
diagnose	1	5033 (0.011)	#SAKNAS!	#SAKNAS!
dialium	1	5033 (0.011)	#SAKNAS!	2492 (0.033)
diamides	1	5033 (0.011)	#SAKNAS!	#SAKNAS!
diamondback	1	5033 (0.011)	2293 (0.034)	#SAKNAS!
diaphorima	1	5033 (0.011)	#SAKNAS!	#SAKNAS!
diaphorina	1	5033 (0.011)	#SAKNAS!	2492 (0.033)
diazepam-binding	1	5033 (0.011)	#SAKNAS!	#SAKNAS!
dicentrarcus	1	5033 (0.011)	2293 (0.034)	#SAKNAS!
diceros	1	5033 (0.011)	2293 (0.034)	#SAKNAS!
dictate	1	5033 (0.011)	#SAKNAS!	2492 (0.033)
didelphis	1	5033 (0.011)	#SAKNAS!	#SAKNAS!
didn	1	5033 (0.011)	#SAKNAS!	2492 (0.033)
die	1	5033 (0.011)	2293 (0.034)	#SAKNAS!
diencephalon	1	5033 (0.011)	2293 (0.034)	#SAKNAS!
dietary-induced	1	5033 (0.011)	2293 (0.034)	#SAKNAS!
diet-borne	1	5033 (0.011)	#SAKNAS!	2492 (0.033)
diet-diaries	1	5033 (0.011)	#SAKNAS!	2492 (0.033)
diet-microbiota-host	1	5033 (0.011)	#SAKNAS!	2492 (0.033)
differentiates	1	5033 (0.011)	#SAKNAS!	2492 (0.033)
differentiating	1	5033 (0.011)	#SAKNAS!	2492 (0.033)
different-sized	1	5033 (0.011)	#SAKNAS!	2492 (0.033)
diffuse	1	5033 (0.011)	2293 (0.034)	#SAKNAS!
digastric	1	5033 (0.011)	#SAKNAS!	#SAKNAS!
digest	1	5033 (0.011)	#SAKNAS!	#SAKNAS!

digest-fees	1	5033 (0.011)	#SAKNAS!	#SAKNAS!
digluconate	1	5033 (0.011)	2293 (0.034)	#SAKNAS!
dihydroxyflavone	1	5033 (0.011)	#SAKNAS!	#SAKNAS!
dilations	1	5033 (0.011)	#SAKNAS!	#SAKNAS!
dilemma	1	5033 (0.011)	#SAKNAS!	#SAKNAS!
dilutions	1	5033 (0.011)	2293 (0.034)	#SAKNAS!
dimension	1	5033 (0.011)	#SAKNAS!	#SAKNAS!
dimethylarginine	1	5033 (0.011)	2293 (0.034)	#SAKNAS!
diminishes	1	5033 (0.011)	#SAKNAS!	#SAKNAS!
dining	1	5033 (0.011)	#SAKNAS!	#SAKNAS!
dinoflagellates	1	5033 (0.011)	#SAKNAS!	2492 (0.033)
dinophyceae	1	5033 (0.011)	#SAKNAS!	2492 (0.033)
diode	1	5033 (0.011)	#SAKNAS!	2492 (0.033)
dioscorea	1	5033 (0.011)	#SAKNAS!	#SAKNAS!
dioxin	1	5033 (0.011)	#SAKNAS!	2492 (0.033)
dip	1	5033 (0.011)	#SAKNAS!	2492 (0.033)
diphenyl	1	5033 (0.011)	#SAKNAS!	#SAKNAS!
diploid	1	5033 (0.011)	2293 (0.034)	#SAKNAS!
diplopoda	1	5033 (0.011)	#SAKNAS!	#SAKNAS!
dipping	1	5033 (0.011)	#SAKNAS!	#SAKNAS!
directions	1	5033 (0.011)	2293 (0.034)	#SAKNAS!
directive	1	5033 (0.011)	2293 (0.034)	#SAKNAS!
directives	1	5033 (0.011)	#SAKNAS!	#SAKNAS!
disabled-child	1	5033 (0.011)	2293 (0.034)	#SAKNAS!
disappearance	1	5033 (0.011)	2293 (0.034)	#SAKNAS!
disastrous	1	5033 (0.011)	#SAKNAS!	2492 (0.033)
discards	1	5033 (0.011)	#SAKNAS!	2492 (0.033)
disclosing	1	5033 (0.011)	#SAKNAS!	2492 (0.033)
disconnection-based	1	5033 (0.011)	#SAKNAS!	#SAKNAS!
discontinuity	1	5033 (0.011)	#SAKNAS!	2492 (0.033)
discrete	1	5033 (0.011)	2293 (0.034)	#SAKNAS!
discriminates	1	5033 (0.011)	#SAKNAS!	2492 (0.033)
discus	1	5033 (0.011)	2293 (0.034)	#SAKNAS!
discussing	1	5033 (0.011)	#SAKNAS!	#SAKNAS!
disease-modifying	1	5033 (0.011)	#SAKNAS!	#SAKNAS!
disease-resistant	1	5033 (0.011)	2293 (0.034)	#SAKNAS!
disease-united	1	5033 (0.011)	#SAKNAS!	2492 (0.033)
disentangling	1	5033 (0.011)	2293 (0.034)	#SAKNAS!
disinhibition	1	5033 (0.011)	#SAKNAS!	#SAKNAS!
disjunct	1	5033 (0.011)	#SAKNAS!	#SAKNAS!
diskectomy	1	5033 (0.011)	#SAKNAS!	#SAKNAS!
disks	1	5033 (0.011)	#SAKNAS!	2492 (0.033)
dislodgement	1	5033 (0.011)	#SAKNAS!	#SAKNAS!

dismutase	1 5033 (0.011)	#SAKNAS!	#SAKNAS!
disorder-a	1 5033 (0.011)	#SAKNAS!	#SAKNAS!
dispersal	1 5033 (0.011)	#SAKNAS!	2492 (0.033)
dispersions	1 5033 (0.011)	#SAKNAS!	2492 (0.033)
disruptions	1 5033 (0.011)	#SAKNAS!	#SAKNAS!
disruptive	1 5033 (0.011)	#SAKNAS!	2492 (0.033)
disrupts	1 5033 (0.011)	2293 (0.034)	#SAKNAS!
disseminating	1 5033 (0.011)	#SAKNAS!	2492 (0.033)
dissociable	1 5033 (0.011)	#SAKNAS!	#SAKNAS!
dissociation	1 5033 (0.011)	#SAKNAS!	2492 (0.033)
dissostichus	1 5033 (0.011)	#SAKNAS!	#SAKNAS!
distance-based	1 5033 (0.011)	#SAKNAS!	#SAKNAS!
distinctive	1 5033 (0.011)	#SAKNAS!	2492 (0.033)
distinguishing	1 5033 (0.011)	#SAKNAS!	2492 (0.033)
distraction	1 5033 (0.011)	#SAKNAS!	#SAKNAS!
distributional	1 5033 (0.011)	#SAKNAS!	#SAKNAS!
districts	1 5033 (0.011)	#SAKNAS!	#SAKNAS!
disturbing	1 5033 (0.011)	#SAKNAS!	#SAKNAS!
disturbs	1 5033 (0.011)	#SAKNAS!	#SAKNAS!
dit-coombe	1 5033 (0.011)	#SAKNAS!	#SAKNAS!
diterpenes	1 5033 (0.011)	2293 (0.034)	#SAKNAS!
divergence	1 5033 (0.011)	2293 (0.034)	#SAKNAS!
diverticula	1 5033 (0.011)	2293 (0.034)	#SAKNAS!
diverticulitis	1 5033 (0.011)	2293 (0.034)	#SAKNAS!
dmanisi	1 5033 (0.011)	#SAKNAS!	2492 (0.033)
dmf	1 5033 (0.011)	#SAKNAS!	#SAKNAS!
dmhf	1 5033 (0.011)	#SAKNAS!	2492 (0.033)
dms	1 5033 (0.011)	#SAKNAS!	2492 (0.033)
dnqp	1 5033 (0.011)	#SAKNAS!	2492 (0.033)
docetaxel	1 5033 (0.011)	#SAKNAS!	2492 (0.033)
docetaxel-cisplatin-fluorouracil	1 5033 (0.011)	#SAKNAS!	#SAKNAS!
doctors	1 5033 (0.011)	#SAKNAS!	#SAKNAS!
document	1 5033 (0.011)	#SAKNAS!	2492 (0.033)
documents	1 5033 (0.011)	#SAKNAS!	2492 (0.033)
doesn	1 5033 (0.011)	#SAKNAS!	2492 (0.033)
dog-human	1 5033 (0.011)	#SAKNAS!	#SAKNAS!
domesticus	1 5033 (0.011)	#SAKNAS!	#SAKNAS!
dominance-related	1 5033 (0.011)	2293 (0.034)	#SAKNAS!
dominican	1 5033 (0.011)	#SAKNAS!	#SAKNAS!
don	1 5033 (0.011)	2293 (0.034)	#SAKNAS!
door-to-oral	1 5033 (0.011)	#SAKNAS!	#SAKNAS!
dopamine-4	1 5033 (0.011)	#SAKNAS!	2492 (0.033)
dopamine-neuropeptide	1 5033 (0.011)	#SAKNAS!	#SAKNAS!

doped	1	5033 (0.011)	#SAKNAS!	#SAKNAS!
dor	1	5033 (0.011)	2293 (0.034)	#SAKNAS!
dorsal	1	5033 (0.011)	2293 (0.034)	#SAKNAS!
dorsalis	1	5033 (0.011)	2293 (0.034)	#SAKNAS!
dorset	1	5033 (0.011)	2293 (0.034)	#SAKNAS!
doryteuthis	1	5033 (0.011)	#SAKNAS!	#SAKNAS!
dose-addition	1	5033 (0.011)	2293 (0.034)	#SAKNAS!
dose-dependent	1	5033 (0.011)	#SAKNAS!	#SAKNAS!
doses	1	5033 (0.011)	#SAKNAS!	#SAKNAS!
dose-volume	1	5033 (0.011)	#SAKNAS!	#SAKNAS!
doubly	1	5033 (0.011)	#SAKNAS!	#SAKNAS!
doves	1	5033 (0.011)	2293 (0.034)	#SAKNAS!
dowling	1	5033 (0.011)	2293 (0.034)	#SAKNAS!
dr	1	5033 (0.011)	2293 (0.034)	#SAKNAS!
dramatic	1	5033 (0.011)	#SAKNAS!	2492 (0.033)
dramatically	1	5033 (0.011)	2293 (0.034)	#SAKNAS!
draught	1	5033 (0.011)	2293 (0.034)	#SAKNAS!
drd4	1	5033 (0.011)	#SAKNAS!	2492 (0.033)
drift	1	5033 (0.011)	#SAKNAS!	#SAKNAS!
drivers	1	5033 (0.011)	#SAKNAS!	#SAKNAS!
driving	1	5033 (0.011)	#SAKNAS!	#SAKNAS!
droebachiensis	1	5033 (0.011)	2293 (0.034)	#SAKNAS!
dropout	1	5033 (0.011)	#SAKNAS!	2492 (0.033)
drosophila-melanogaster	1	5033 (0.011)	2293 (0.034)	#SAKNAS!
drosophila-serido	1	5033 (0.011)	2293 (0.034)	#SAKNAS!
drought	1	5033 (0.011)	#SAKNAS!	2492 (0.033)
dryopithecine	1	5033 (0.011)	#SAKNAS!	2492 (0.033)
ds	1	5033 (0.011)	#SAKNAS!	#SAKNAS!
dsa簣	1	5033 (0.011)	#SAKNAS!	#SAKNAS!
dswal-qol	1	5033 (0.011)	#SAKNAS!	2492 (0.033)
dual	1	5033 (0.011)	#SAKNAS!	2492 (0.033)
dual-axis	1	5033 (0.011)	2293 (0.034)	#SAKNAS!
dual-tasks	1	5033 (0.011)	#SAKNAS!	2492 (0.033)
duck	1	5033 (0.011)	#SAKNAS!	2492 (0.033)
ducks	1	5033 (0.011)	2293 (0.034)	#SAKNAS!
duke	1	5033 (0.011)	#SAKNAS!	#SAKNAS!
duodena	1	5033 (0.011)	2293 (0.034)	#SAKNAS!
duodenoileal	1	5033 (0.011)	#SAKNAS!	2492 (0.033)
durability	1	5033 (0.011)	#SAKNAS!	2492 (0.033)
durum	1	5033 (0.011)	#SAKNAS!	#SAKNAS!
durvalumab	1	5033 (0.011)	#SAKNAS!	#SAKNAS!
dwindles	1	5033 (0.011)	2293 (0.034)	#SAKNAS!
dymus	1	5033 (0.011)	#SAKNAS!	#SAKNAS!

dymus-hr	1	5033 (0.011)	#SAKNAS!	#SAKNAS!
dynamically	1	5033 (0.011)	#SAKNAS!	#SAKNAS!
dynamicity	1	5033 (0.011)	#SAKNAS!	#SAKNAS!
dynamo	1	5033 (0.011)	#SAKNAS!	2492 (0.033)
dynorphin	1	5033 (0.011)	#SAKNAS!	2492 (0.033)
dysautonomia	1	5033 (0.011)	2293 (0.034)	#SAKNAS!
dysgnathic	1	5033 (0.011)	2293 (0.034)	#SAKNAS!
dysphagia-a	1	5033 (0.011)	#SAKNAS!	#SAKNAS!
dysphagia-friendly	1	5033 (0.011)	#SAKNAS!	2492 (0.033)
dysphagia-specific	1	5033 (0.011)	#SAKNAS!	#SAKNAS!
dysphaging	1	5033 (0.011)	#SAKNAS!	#SAKNAS!
dysphagy	1	5033 (0.011)	#SAKNAS!	2492 (0.033)
dyspnea	1	5033 (0.011)	#SAKNAS!	2492 (0.033)
dysregulation	1	5033 (0.011)	#SAKNAS!	#SAKNAS!
dystrophica	1	5033 (0.011)	#SAKNAS!	2492 (0.033)
e.v	1	5033 (0.011)	#SAKNAS!	2492 (0.033)
eagle	1	5033 (0.011)	#SAKNAS!	2492 (0.033)
early-onset	1	5033 (0.011)	2293 (0.034)	#SAKNAS!
early-to-advanced	1	5033 (0.011)	#SAKNAS!	2492 (0.033)
earthquake	1	5033 (0.011)	#SAKNAS!	2492 (0.033)
ease	1	5033 (0.011)	#SAKNAS!	#SAKNAS!
easo-defined	1	5033 (0.011)	#SAKNAS!	#SAKNAS!
easy	1	5033 (0.011)	2293 (0.034)	#SAKNAS!
eat-10spa	1	5033 (0.011)	#SAKNAS!	2492 (0.033)
eat-ability	1	5033 (0.011)	#SAKNAS!	2492 (0.033)
eating-related	1	5033 (0.011)	#SAKNAS!	#SAKNAS!
eat-lancet	1	5033 (0.011)	#SAKNAS!	#SAKNAS!
ecc	1	5033 (0.011)	#SAKNAS!	#SAKNAS!
echium	1	5033 (0.011)	#SAKNAS!	2492 (0.033)
echocardiographic	1	5033 (0.011)	#SAKNAS!	2492 (0.033)
echocardiography	1	5033 (0.011)	#SAKNAS!	2492 (0.033)
echogenicity	1	5033 (0.011)	#SAKNAS!	#SAKNAS!
e-cigarettes	1	5033 (0.011)	#SAKNAS!	#SAKNAS!
eckardt	1	5033 (0.011)	#SAKNAS!	2492 (0.033)
ecohistological	1	5033 (0.011)	2293 (0.034)	#SAKNAS!
ecomorphology	1	5033 (0.011)	#SAKNAS!	#SAKNAS!
economically	1	5033 (0.011)	#SAKNAS!	2492 (0.033)
economy	1	5033 (0.011)	#SAKNAS!	2492 (0.033)
ecopal	1	5033 (0.011)	#SAKNAS!	#SAKNAS!
ecophysiological	1	5033 (0.011)	#SAKNAS!	2492 (0.033)
ectoparasitoid	1	5033 (0.011)	2293 (0.034)	#SAKNAS!
edelphi	1	5033 (0.011)	#SAKNAS!	#SAKNAS!
edelstein	1	5033 (0.011)	2293 (0.034)	#SAKNAS!

edematous	1	5033 (0.011)	2293 (0.034)	#SAKNAS!
edentate	1	5033 (0.011)	#SAKNAS!	#SAKNAS!
ediacaran	1	5033 (0.011)	#SAKNAS!	#SAKNAS!
edition	1	5033 (0.011)	2293 (0.034)	#SAKNAS!
educate	1	5033 (0.011)	2293 (0.034)	#SAKNAS!
educators	1	5033 (0.011)	#SAKNAS!	#SAKNAS!
edwards	1	5033 (0.011)	2293 (0.034)	#SAKNAS!
edwardsi	1	5033 (0.011)	2293 (0.034)	#SAKNAS!
eel	1	5033 (0.011)	2293 (0.034)	#SAKNAS!
effectively	1	5033 (0.011)	#SAKNAS!	2492 (0.033)
efferents	1	5033 (0.011)	#SAKNAS!	#SAKNAS!
efficient	1	5033 (0.011)	2293 (0.034)	#SAKNAS!
efflux	1	5033 (0.011)	2293 (0.034)	#SAKNAS!
effort	1	5033 (0.011)	#SAKNAS!	2492 (0.033)
efforts	1	5033 (0.011)	2293 (0.034)	#SAKNAS!
egcg	1	5033 (0.011)	2293 (0.034)	#SAKNAS!
egestion	1	5033 (0.011)	2293 (0.034)	#SAKNAS!
eggs	1	5033 (0.011)	2293 (0.034)	#SAKNAS!
egjoo	1	5033 (0.011)	#SAKNAS!	#SAKNAS!
egrets	1	5033 (0.011)	2293 (0.034)	#SAKNAS!
ehealth	1	5033 (0.011)	#SAKNAS!	2492 (0.033)
ehlers-dantos	1	5033 (0.011)	2293 (0.034)	#SAKNAS!
eica-essd-eugms	1	5033 (0.011)	#SAKNAS!	2492 (0.033)
eichner	1	5033 (0.011)	#SAKNAS!	#SAKNAS!
eicosapentaenoic	1	5033 (0.011)	2293 (0.034)	#SAKNAS!
eiffingeri	1	5033 (0.011)	2293 (0.034)	#SAKNAS!
eight	1	5033 (0.011)	#SAKNAS!	2492 (0.033)
einflufl	1	5033 (0.011)	2293 (0.034)	#SAKNAS!
eisenia	1	5033 (0.011)	#SAKNAS!	2492 (0.033)
ejection	1	5033 (0.011)	#SAKNAS!	2492 (0.033)
elaidic	1	5033 (0.011)	#SAKNAS!	#SAKNAS!
elasticity	1	5033 (0.011)	#SAKNAS!	2492 (0.033)
elastography	1	5033 (0.011)	#SAKNAS!	#SAKNAS!
elbasvir	1	5033 (0.011)	#SAKNAS!	2492 (0.033)
e-learning	1	5033 (0.011)	#SAKNAS!	2492 (0.033)
electroacupuncture	1	5033 (0.011)	#SAKNAS!	#SAKNAS!
electrocardiographic	1	5033 (0.011)	2293 (0.034)	#SAKNAS!
electrocautery	1	5033 (0.011)	2293 (0.034)	#SAKNAS!
electrode	1	5033 (0.011)	#SAKNAS!	2492 (0.033)
electrodiagnostic	1	5033 (0.011)	#SAKNAS!	#SAKNAS!
electrolytes	1	5033 (0.011)	#SAKNAS!	#SAKNAS!
electromagnetic	1	5033 (0.011)	2293 (0.034)	#SAKNAS!
electronics	1	5033 (0.011)	#SAKNAS!	#SAKNAS!

electrophysiological	1	5033 (0.011)	#SAKNAS!	#SAKNAS!
elefante	1	5033 (0.011)	#SAKNAS!	2492 (0.033)
elegans	1	5033 (0.011)	#SAKNAS!	2492 (0.033)
elegansnematodes	1	5033 (0.011)	#SAKNAS!	2492 (0.033)
elemental	1	5033 (0.011)	2293 (0.034)	#SAKNAS!
element-binding	1	5033 (0.011)	#SAKNAS!	2492 (0.033)
elephants	1	5033 (0.011)	#SAKNAS!	2492 (0.033)
elephas	1	5033 (0.011)	#SAKNAS!	#SAKNAS!
elevational	1	5033 (0.011)	2293 (0.034)	#SAKNAS!
eleven	1	5033 (0.011)	2293 (0.034)	#SAKNAS!
elicit	1	5033 (0.011)	#SAKNAS!	#SAKNAS!
eligibility	1	5033 (0.011)	#SAKNAS!	#SAKNAS!
elongation	1	5033 (0.011)	#SAKNAS!	#SAKNAS!
elongational	1	5033 (0.011)	#SAKNAS!	2492 (0.033)
elsa-brasil	1	5033 (0.011)	#SAKNAS!	#SAKNAS!
elucidate	1	5033 (0.011)	#SAKNAS!	#SAKNAS!
elucidating	1	5033 (0.011)	#SAKNAS!	#SAKNAS!
elysia	1	5033 (0.011)	2293 (0.034)	#SAKNAS!
embedding	1	5033 (0.011)	#SAKNAS!	#SAKNAS!
embeds	1	5033 (0.011)	#SAKNAS!	2492 (0.033)
emblica	1	5033 (0.011)	#SAKNAS!	2492 (0.033)
emended	1	5033 (0.011)	2293 (0.034)	#SAKNAS!
emergencies	1	5033 (0.011)	#SAKNAS!	2492 (0.033)
emery-dreifuss	1	5033 (0.011)	#SAKNAS!	#SAKNAS!
emissions	1	5033 (0.011)	#SAKNAS!	#SAKNAS!
emitted	1	5033 (0.011)	#SAKNAS!	#SAKNAS!
emotion	1	5033 (0.011)	#SAKNAS!	2492 (0.033)
emperor	1	5033 (0.011)	#SAKNAS!	2492 (0.033)
emphasizing	1	5033 (0.011)	2293 (0.034)	#SAKNAS!
empirically	1	5033 (0.011)	#SAKNAS!	#SAKNAS!
empyema	1	5033 (0.011)	#SAKNAS!	#SAKNAS!
emulgels	1	5033 (0.011)	#SAKNAS!	#SAKNAS!
en	1	5033 (0.011)	#SAKNAS!	2492 (0.033)
enablers	1	5033 (0.011)	#SAKNAS!	#SAKNAS!
enables	1	5033 (0.011)	#SAKNAS!	#SAKNAS!
encephalomyopathy	1	5033 (0.011)	#SAKNAS!	#SAKNAS!
enchytraeid	1	5033 (0.011)	2293 (0.034)	#SAKNAS!
enchytraeus	1	5033 (0.011)	2293 (0.034)	#SAKNAS!
encounter	1	5033 (0.011)	2293 (0.034)	#SAKNAS!
encouragement	1	5033 (0.011)	#SAKNAS!	2492 (0.033)
encuesta	1	5033 (0.011)	#SAKNAS!	2492 (0.033)
endarterectomy	1	5033 (0.011)	2293 (0.034)	#SAKNAS!
endemic	1	5033 (0.011)	2293 (0.034)	#SAKNAS!

endocannabinoids	1	5033 (0.011)	#SAKNAS!	#SAKNAS!
endocycling	1	5033 (0.011)	#SAKNAS!	2492 (0.033)
endoflip	1	5033 (0.011)	#SAKNAS!	#SAKNAS!
endografting	1	5033 (0.011)	#SAKNAS!	2492 (0.033)
endoleak	1	5033 (0.011)	#SAKNAS!	2492 (0.033)
endometriosis	1	5033 (0.011)	2293 (0.034)	#SAKNAS!
endonasal	1	5033 (0.011)	#SAKNAS!	2492 (0.033)
endoparasite	1	5033 (0.011)	#SAKNAS!	#SAKNAS!
endoplasmic	1	5033 (0.011)	#SAKNAS!	2492 (0.033)
endoprostheses	1	5033 (0.011)	#SAKNAS!	#SAKNAS!
endoreplication	1	5033 (0.011)	#SAKNAS!	2492 (0.033)
endoscope	1	5033 (0.011)	2293 (0.034)	#SAKNAS!
endosteal	1	5033 (0.011)	#SAKNAS!	2492 (0.033)
endpoints	1	5033 (0.011)	#SAKNAS!	2492 (0.033)
endproducts	1	5033 (0.011)	#SAKNAS!	2492 (0.033)
end-products	1	5033 (0.011)	2293 (0.034)	#SAKNAS!
energetical	1	5033 (0.011)	2293 (0.034)	#SAKNAS!
energetics	1	5033 (0.011)	2293 (0.034)	#SAKNAS!
energy-cost	1	5033 (0.011)	2293 (0.034)	#SAKNAS!
energy-dense	1	5033 (0.011)	2293 (0.034)	#SAKNAS!
energy-efficient	1	5033 (0.011)	2293 (0.034)	#SAKNAS!
engage	1	5033 (0.011)	#SAKNAS!	2492 (0.033)
enge	1	5033 (0.011)	#SAKNAS!	#SAKNAS!
engineering	1	5033 (0.011)	2293 (0.034)	#SAKNAS!
enhance	1	5033 (0.011)	#SAKNAS!	2492 (0.033)
enhancement	1	5033 (0.011)	#SAKNAS!	#SAKNAS!
enhydra	1	5033 (0.011)	2293 (0.034)	#SAKNAS!
enlargement	1	5033 (0.011)	2293 (0.034)	#SAKNAS!
enrichments	1	5033 (0.011)	2293 (0.034)	#SAKNAS!
enrollment	1	5033 (0.011)	#SAKNAS!	#SAKNAS!
ensifera	1	5033 (0.011)	#SAKNAS!	#SAKNAS!
ensiled	1	5033 (0.011)	2293 (0.034)	#SAKNAS!
enso	1	5033 (0.011)	#SAKNAS!	#SAKNAS!
enteric	1	5033 (0.011)	#SAKNAS!	#SAKNAS!
enterocolitis	1	5033 (0.011)	#SAKNAS!	#SAKNAS!
enteroendocrine	1	5033 (0.011)	#SAKNAS!	#SAKNAS!
enterosalivary	1	5033 (0.011)	#SAKNAS!	2492 (0.033)
enterostomy	1	5033 (0.011)	#SAKNAS!	#SAKNAS!
entropy	1	5033 (0.011)	#SAKNAS!	#SAKNAS!
entropy-balanced	1	5033 (0.011)	#SAKNAS!	#SAKNAS!
entwicklung	1	5033 (0.011)	2293 (0.034)	#SAKNAS!
environmental-conditions	1	5033 (0.011)	2293 (0.034)	#SAKNAS!
en-y	1	5033 (0.011)	#SAKNAS!	2492 (0.033)

enzymatic	1	5033 (0.011)	#SAKNAS!	#SAKNAS!
eortc-qlq-h&n35	1	5033 (0.011)	#SAKNAS!	#SAKNAS!
eosinophilia	1	5033 (0.011)	#SAKNAS!	#SAKNAS!
eotaxin	1	5033 (0.011)	#SAKNAS!	2492 (0.033)
epa	1	5033 (0.011)	#SAKNAS!	2492 (0.033)
epg	1	5033 (0.011)	#SAKNAS!	2492 (0.033)
epibiotic	1	5033 (0.011)	#SAKNAS!	2492 (0.033)
epicardial	1	5033 (0.011)	#SAKNAS!	2492 (0.033)
epic-potsdam	1	5033 (0.011)	#SAKNAS!	2492 (0.033)
epiglottic	1	5033 (0.011)	#SAKNAS!	#SAKNAS!
epilepsy	1	5033 (0.011)	#SAKNAS!	#SAKNAS!
epilithic	1	5033 (0.011)	#SAKNAS!	2492 (0.033)
epimastigotes	1	5033 (0.011)	#SAKNAS!	#SAKNAS!
epipap	1	5033 (0.011)	2293 (0.034)	#SAKNAS!
episodes	1	5033 (0.011)	#SAKNAS!	2492 (0.033)
episodic	1	5033 (0.011)	#SAKNAS!	2492 (0.033)
epithelial	1	5033 (0.011)	#SAKNAS!	2492 (0.033)
epithelium	1	5033 (0.011)	2293 (0.034)	#SAKNAS!
epos	1	5033 (0.011)	#SAKNAS!	#SAKNAS!
epstein-barr	1	5033 (0.011)	#SAKNAS!	#SAKNAS!
eptatretus	1	5033 (0.011)	#SAKNAS!	2492 (0.033)
equal	1	5033 (0.011)	#SAKNAS!	#SAKNAS!
equicaloric	1	5033 (0.011)	#SAKNAS!	2492 (0.033)
equids	1	5033 (0.011)	#SAKNAS!	2492 (0.033)
equine	1	5033 (0.011)	2293 (0.034)	#SAKNAS!
eras	1	5033 (0.011)	#SAKNAS!	#SAKNAS!
erato	1	5033 (0.011)	2293 (0.034)	#SAKNAS!
erbb4	1	5033 (0.011)	#SAKNAS!	#SAKNAS!
erectile	1	5033 (0.011)	2293 (0.034)	#SAKNAS!
eretmochelys	1	5033 (0.011)	2293 (0.034)	#SAKNAS!
ergogenic	1	5033 (0.011)	#SAKNAS!	2492 (0.033)
eridania	1	5033 (0.011)	#SAKNAS!	2492 (0.033)
erosions	1	5033 (0.011)	2293 (0.034)	#SAKNAS!
error	1	5033 (0.011)	#SAKNAS!	2492 (0.033)
errors	1	5033 (0.011)	#SAKNAS!	2492 (0.033)
erupted	1	5033 (0.011)	#SAKNAS!	#SAKNAS!
ervi	1	5033 (0.011)	2293 (0.034)	#SAKNAS!
erythritoland	1	5033 (0.011)	#SAKNAS!	2492 (0.033)
es	1	5033 (0.011)	2293 (0.034)	#SAKNAS!
escape	1	5033 (0.011)	#SAKNAS!	2492 (0.033)
esian	1	5033 (0.011)	#SAKNAS!	2492 (0.033)
esophageal-carcinoma	1	5033 (0.011)	2293 (0.034)	#SAKNAS!
esophageal-perforation	1	5033 (0.011)	2293 (0.034)	#SAKNAS!

esophagocardial	1	5033 (0.011)	2293 (0.034)	#SAKNAS!
esophagojejunal	1	5033 (0.011)	2293 (0.034)	#SAKNAS!
esophagojejunostomy	1	5033 (0.011)	#SAKNAS!	2492 (0.033)
esophagomyotomy	1	5033 (0.011)	2293 (0.034)	#SAKNAS!
esophagoplasty	1	5033 (0.011)	#SAKNAS!	2492 (0.033)
esophagoscope	1	5033 (0.011)	#SAKNAS!	2492 (0.033)
esophagoscopic	1	5033 (0.011)	2293 (0.034)	#SAKNAS!
esophagoscopy	1	5033 (0.011)	#SAKNAS!	2492 (0.033)
esophagostomy	1	5033 (0.011)	#SAKNAS!	#SAKNAS!
esophagram	1	5033 (0.011)	#SAKNAS!	2492 (0.033)
esox	1	5033 (0.011)	#SAKNAS!	2492 (0.033)
espanola	1	5033 (0.011)	#SAKNAS!	2492 (0.033)
espghan-naspghan	1	5033 (0.011)	#SAKNAS!	2492 (0.033)
establishments	1	5033 (0.011)	2293 (0.034)	#SAKNAS!
esteem	1	5033 (0.011)	#SAKNAS!	#SAKNAS!
ester	1	5033 (0.011)	2293 (0.034)	#SAKNAS!
esterase	1	5033 (0.011)	#SAKNAS!	2492 (0.033)
estimators	1	5033 (0.011)	#SAKNAS!	#SAKNAS!
estonia	1	5033 (0.011)	2293 (0.034)	#SAKNAS!
estuarine	1	5033 (0.011)	2293 (0.034)	#SAKNAS!
estuary	1	5033 (0.011)	#SAKNAS!	2492 (0.033)
et-22	1	5033 (0.011)	#SAKNAS!	#SAKNAS!
ethanol	1	5033 (0.011)	#SAKNAS!	2492 (0.033)
ether	1	5033 (0.011)	#SAKNAS!	#SAKNAS!
ethinyl	1	5033 (0.011)	#SAKNAS!	#SAKNAS!
ethiopian	1	5033 (0.011)	2293 (0.034)	#SAKNAS!
ethnically	1	5033 (0.011)	#SAKNAS!	#SAKNAS!
ethnicity-based	1	5033 (0.011)	#SAKNAS!	#SAKNAS!
ethnodentistry	1	5033 (0.011)	#SAKNAS!	#SAKNAS!
ethylicin	1	5033 (0.011)	#SAKNAS!	#SAKNAS!
etruscan	1	5033 (0.011)	2293 (0.034)	#SAKNAS!
eucalypt-feeding	1	5033 (0.011)	2293 (0.034)	#SAKNAS!
eucalyptus	1	5033 (0.011)	#SAKNAS!	2492 (0.033)
eugenii	1	5033 (0.011)	2293 (0.034)	#SAKNAS!
eulophus	1	5033 (0.011)	2293 (0.034)	#SAKNAS!
euproctidis	1	5033 (0.011)	2293 (0.034)	#SAKNAS!
eur	1	5033 (0.011)	2293 (0.034)	#SAKNAS!
eurasian	1	5033 (0.011)	2293 (0.034)	#SAKNAS!
euryhaline	1	5033 (0.011)	#SAKNAS!	#SAKNAS!
eurytemora	1	5033 (0.011)	2293 (0.034)	#SAKNAS!
eustachian	1	5033 (0.011)	#SAKNAS!	#SAKNAS!
euterpe	1	5033 (0.011)	#SAKNAS!	#SAKNAS!
euterpina-acutifrons	1	5033 (0.011)	2293 (0.034)	#SAKNAS!

evacuation	1	5033 (0.011)	#SAKNAS!	#SAKNAS!
eva-jf	1	5033 (0.011)	#SAKNAS!	#SAKNAS!
ever	1	5033 (0.011)	#SAKNAS!	2492 (0.033)
everyday	1	5033 (0.011)	#SAKNAS!	2492 (0.033)
evident	1	5033 (0.011)	#SAKNAS!	#SAKNAS!
exacerbates	1	5033 (0.011)	#SAKNAS!	2492 (0.033)
examinations	1	5033 (0.011)	#SAKNAS!	2492 (0.033)
examinee	1	5033 (0.011)	#SAKNAS!	2492 (0.033)
examiner	1	5033 (0.011)	2293 (0.034)	#SAKNAS!
excellent	1	5033 (0.011)	#SAKNAS!	2492 (0.033)
exception	1	5033 (0.011)	#SAKNAS!	#SAKNAS!
excesses	1	5033 (0.011)	2293 (0.034)	#SAKNAS!
excise	1	5033 (0.011)	2293 (0.034)	#SAKNAS!
excitability	1	5033 (0.011)	2293 (0.034)	#SAKNAS!
excitation	1	5033 (0.011)	#SAKNAS!	2492 (0.033)
excitatory	1	5033 (0.011)	#SAKNAS!	2492 (0.033)
exclusively	1	5033 (0.011)	2293 (0.034)	#SAKNAS!
exert	1	5033 (0.011)	#SAKNAS!	2492 (0.033)
exfoliation	1	5033 (0.011)	#SAKNAS!	2492 (0.033)
exhibit	1	5033 (0.011)	#SAKNAS!	2492 (0.033)
exhibits	1	5033 (0.011)	#SAKNAS!	#SAKNAS!
existence	1	5033 (0.011)	#SAKNAS!	#SAKNAS!
exocrine	1	5033 (0.011)	#SAKNAS!	2492 (0.033)
exogenous	1	5033 (0.011)	#SAKNAS!	2492 (0.033)
exome	1	5033 (0.011)	#SAKNAS!	2492 (0.033)
exopeptidase	1	5033 (0.011)	#SAKNAS!	2492 (0.033)
exophiala	1	5033 (0.011)	2293 (0.034)	#SAKNAS!
exophthalmus	1	5033 (0.011)	2293 (0.034)	#SAKNAS!
exosomes	1	5033 (0.011)	#SAKNAS!	2492 (0.033)
expandable	1	5033 (0.011)	2293 (0.034)	#SAKNAS!
expectancy	1	5033 (0.011)	#SAKNAS!	2492 (0.033)
expenses	1	5033 (0.011)	#SAKNAS!	2492 (0.033)
experimentally	1	5033 (0.011)	#SAKNAS!	#SAKNAS!
experimental-study	1	5033 (0.011)	2293 (0.034)	#SAKNAS!
experts	1	5033 (0.011)	2293 (0.034)	#SAKNAS!
explainable	1	5033 (0.011)	#SAKNAS!	#SAKNAS!
explanations	1	5033 (0.011)	2293 (0.034)	#SAKNAS!
explantation	1	5033 (0.011)	#SAKNAS!	2492 (0.033)
explantion	1	5033 (0.011)	2293 (0.034)	#SAKNAS!
exploit	1	5033 (0.011)	#SAKNAS!	2492 (0.033)
explore	1	5033 (0.011)	#SAKNAS!	2492 (0.033)
expressions	1	5033 (0.011)	#SAKNAS!	2492 (0.033)
ex-q	1	5033 (0.011)	#SAKNAS!	2492 (0.033)

extant	1	5033 (0.011)	#SAKNAS!	2492 (0.033)
extended-care	1	5033 (0.011)	2293 (0.034)	#SAKNAS!
extended-release	1	5033 (0.011)	#SAKNAS!	2492 (0.033)
extends	1	5033 (0.011)	#SAKNAS!	2492 (0.033)
extensive	1	5033 (0.011)	2293 (0.034)	#SAKNAS!
extracorporeal	1	5033 (0.011)	#SAKNAS!	#SAKNAS!
extractions	1	5033 (0.011)	#SAKNAS!	#SAKNAS!
extraordinary	1	5033 (0.011)	2293 (0.034)	#SAKNAS!
extrapontine	1	5033 (0.011)	2293 (0.034)	#SAKNAS!
extremely	1	5033 (0.011)	#SAKNAS!	#SAKNAS!
extremis	1	5033 (0.011)	#SAKNAS!	2492 (0.033)
extrinsic	1	5033 (0.011)	2293 (0.034)	#SAKNAS!
extrused	1	5033 (0.011)	#SAKNAS!	2492 (0.033)
ex-vivo	1	5033 (0.011)	#SAKNAS!	#SAKNAS!
eye	1	5033 (0.011)	2293 (0.034)	#SAKNAS!
fabrication	1	5033 (0.011)	#SAKNAS!	2492 (0.033)
face	1	5033 (0.011)	#SAKNAS!	2492 (0.033)
face-to-face	1	5033 (0.011)	#SAKNAS!	#SAKNAS!
facilitation	1	5033 (0.011)	#SAKNAS!	#SAKNAS!
facilitatory	1	5033 (0.011)	#SAKNAS!	#SAKNAS!
factor-alpha	1	5033 (0.011)	#SAKNAS!	2492 (0.033)
factorial	1	5033 (0.011)	#SAKNAS!	#SAKNAS!
factors-a	1	5033 (0.011)	#SAKNAS!	#SAKNAS!
factory	1	5033 (0.011)	2293 (0.034)	#SAKNAS!
fadoi	1	5033 (0.011)	#SAKNAS!	#SAKNAS!
fads	1	5033 (0.011)	2293 (0.034)	#SAKNAS!
faecal-ft-nirs	1	5033 (0.011)	#SAKNAS!	2492 (0.033)
faeces	1	5033 (0.011)	#SAKNAS!	2492 (0.033)
faers	1	5033 (0.011)	#SAKNAS!	#SAKNAS!
fail	1	5033 (0.011)	2293 (0.034)	#SAKNAS!
fails	1	5033 (0.011)	#SAKNAS!	2492 (0.033)
failure-results	1	5033 (0.011)	#SAKNAS!	#SAKNAS!
failures	1	5033 (0.011)	#SAKNAS!	2492 (0.033)
failure-to-thrive	1	5033 (0.011)	2293 (0.034)	#SAKNAS!
faim2	1	5033 (0.011)	2293 (0.034)	#SAKNAS!
fairule	1	5033 (0.011)	2293 (0.034)	#SAKNAS!
faisalabad	1	5033 (0.011)	#SAKNAS!	#SAKNAS!
falcon	1	5033 (0.011)	#SAKNAS!	#SAKNAS!
faltering	1	5033 (0.011)	2293 (0.034)	#SAKNAS!
familiar	1	5033 (0.011)	2293 (0.034)	#SAKNAS!
familiaris	1	5033 (0.011)	2293 (0.034)	#SAKNAS!
familiarity	1	5033 (0.011)	#SAKNAS!	2492 (0.033)
family-	1	5033 (0.011)	#SAKNAS!	#SAKNAS!

family-centered	1	5033 (0.011)	#SAKNAS!	2492 (0.033)
family-style	1	5033 (0.011)	#SAKNAS!	#SAKNAS!
fangsiao	1	5033 (0.011)	#SAKNAS!	#SAKNAS!
farmland	1	5033 (0.011)	#SAKNAS!	2492 (0.033)
farnese	1	5033 (0.011)	#SAKNAS!	#SAKNAS!
farrowing	1	5033 (0.011)	#SAKNAS!	#SAKNAS!
fars	1	5033 (0.011)	#SAKNAS!	2492 (0.033)
fars-iran	1	5033 (0.011)	#SAKNAS!	#SAKNAS!
fascia	1	5033 (0.011)	#SAKNAS!	#SAKNAS!
fasciitis	1	5033 (0.011)	2293 (0.034)	#SAKNAS!
faso	1	5033 (0.011)	#SAKNAS!	2492 (0.033)
fast-changing	1	5033 (0.011)	#SAKNAS!	2492 (0.033)
fasting-induced	1	5033 (0.011)	#SAKNAS!	2492 (0.033)
fate	1	5033 (0.011)	#SAKNAS!	2492 (0.033)
fat-free	1	5033 (0.011)	#SAKNAS!	2492 (0.033)
fatty-acids	1	5033 (0.011)	2293 (0.034)	#SAKNAS!
favorite	1	5033 (0.011)	#SAKNAS!	#SAKNAS!
fawns	1	5033 (0.011)	2293 (0.034)	#SAKNAS!
fctors	1	5033 (0.011)	#SAKNAS!	#SAKNAS!
fda	1	5033 (0.011)	#SAKNAS!	#SAKNAS!
feather	1	5033 (0.011)	#SAKNAS!	2492 (0.033)
feathers	1	5033 (0.011)	2293 (0.034)	#SAKNAS!
federal	1	5033 (0.011)	2293 (0.034)	#SAKNAS!
feed-deprivation	1	5033 (0.011)	#SAKNAS!	2492 (0.033)
feed-driven	1	5033 (0.011)	2293 (0.034)	#SAKNAS!
feeding-an	1	5033 (0.011)	#SAKNAS!	#SAKNAS!
feeding-habits	1	5033 (0.011)	2293 (0.034)	#SAKNAS!
feeding-response	1	5033 (0.011)	#SAKNAS!	#SAKNAS!
feeding-shifts	1	5033 (0.011)	#SAKNAS!	2492 (0.033)
feedstuffs	1	5033 (0.011)	#SAKNAS!	2492 (0.033)
feeling	1	5033 (0.011)	#SAKNAS!	2492 (0.033)
feelings	1	5033 (0.011)	2293 (0.034)	#SAKNAS!
feira	1	5033 (0.011)	#SAKNAS!	2492 (0.033)
felis	1	5033 (0.011)	2293 (0.034)	#SAKNAS!
femoral	1	5033 (0.011)	#SAKNAS!	#SAKNAS!
fenfluramine	1	5033 (0.011)	2293 (0.034)	#SAKNAS!
fenland	1	5033 (0.011)	#SAKNAS!	2492 (0.033)
fenoxycarb	1	5033 (0.011)	2293 (0.034)	#SAKNAS!
fentanyl	1	5033 (0.011)	#SAKNAS!	2492 (0.033)
ferritin	1	5033 (0.011)	#SAKNAS!	2492 (0.033)
fertiliser	1	5033 (0.011)	#SAKNAS!	#SAKNAS!
fertilitat	1	5033 (0.011)	2293 (0.034)	#SAKNAS!
fescue	1	5033 (0.011)	2293 (0.034)	#SAKNAS!

fever	1	5033 (0.011)	#SAKNAS!	2492 (0.033)
fi	1	5033 (0.011)	#SAKNAS!	#SAKNAS!
fibers	1	5033 (0.011)	#SAKNAS!	#SAKNAS!
fibre-optic	1	5033 (0.011)	#SAKNAS!	2492 (0.033)
fibrillary	1	5033 (0.011)	#SAKNAS!	#SAKNAS!
fibroblast	1	5033 (0.011)	#SAKNAS!	#SAKNAS!
fibroblasts	1	5033 (0.011)	#SAKNAS!	2492 (0.033)
fibrolytic	1	5033 (0.011)	#SAKNAS!	#SAKNAS!
fibrosis-related	1	5033 (0.011)	#SAKNAS!	#SAKNAS!
fibula	1	5033 (0.011)	#SAKNAS!	#SAKNAS!
fiddler-crabs	1	5033 (0.011)	2293 (0.034)	#SAKNAS!
fidelity	1	5033 (0.011)	2293 (0.034)	#SAKNAS!
fields	1	5033 (0.011)	2293 (0.034)	#SAKNAS!
fifteen-year-old	1	5033 (0.011)	#SAKNAS!	2492 (0.033)
fifth-instar	1	5033 (0.011)	#SAKNAS!	2492 (0.033)
fight	1	5033 (0.011)	#SAKNAS!	2492 (0.033)
fiji	1	5033 (0.011)	#SAKNAS!	#SAKNAS!
filefish	1	5033 (0.011)	#SAKNAS!	#SAKNAS!
filiformis	1	5033 (0.011)	2293 (0.034)	#SAKNAS!
fill	1	5033 (0.011)	2293 (0.034)	#SAKNAS!
films	1	5033 (0.011)	#SAKNAS!	#SAKNAS!
final	1	5033 (0.011)	#SAKNAS!	#SAKNAS!
financial	1	5033 (0.011)	#SAKNAS!	#SAKNAS!
fine-mapping	1	5033 (0.011)	#SAKNAS!	2492 (0.033)
finger	1	5033 (0.011)	#SAKNAS!	#SAKNAS!
fingerprint	1	5033 (0.011)	#SAKNAS!	2492 (0.033)
finoral	1	5033 (0.011)	#SAKNAS!	#SAKNAS!
finut	1	5033 (0.011)	#SAKNAS!	2492 (0.033)
fire-induced	1	5033 (0.011)	2293 (0.034)	#SAKNAS!
firmicutes	1	5033 (0.011)	#SAKNAS!	#SAKNAS!
firmus	1	5033 (0.011)	#SAKNAS!	2492 (0.033)
first-ever	1	5033 (0.011)	#SAKNAS!	2492 (0.033)
first-line	1	5033 (0.011)	#SAKNAS!	2492 (0.033)
first-wave	1	5033 (0.011)	#SAKNAS!	#SAKNAS!
first-year	1	5033 (0.011)	#SAKNAS!	2492 (0.033)
fisher-hunter-gatherers	1	5033 (0.011)	#SAKNAS!	2492 (0.033)
fishery	1	5033 (0.011)	#SAKNAS!	2492 (0.033)
fishing	1	5033 (0.011)	#SAKNAS!	#SAKNAS!
fishmeal-free	1	5033 (0.011)	#SAKNAS!	#SAKNAS!
fissure	1	5033 (0.011)	#SAKNAS!	2492 (0.033)
fistulas	1	5033 (0.011)	#SAKNAS!	#SAKNAS!
fit4treatment	1	5033 (0.011)	#SAKNAS!	#SAKNAS!
fitting	1	5033 (0.011)	2293 (0.034)	#SAKNAS!

five-item	1	5033 (0.011)	#SAKNAS!	#SAKNAS!
five-year-old	1	5033 (0.011)	2293 (0.034)	#SAKNAS!
fjord	1	5033 (0.011)	#SAKNAS!	2492 (0.033)
flaccid	1	5033 (0.011)	#SAKNAS!	#SAKNAS!
flagellate	1	5033 (0.011)	2293 (0.034)	#SAKNAS!
flagellates	1	5033 (0.011)	2293 (0.034)	#SAKNAS!
flags	1	5033 (0.011)	#SAKNAS!	2492 (0.033)
flamingo	1	5033 (0.011)	#SAKNAS!	#SAKNAS!
flat	1	5033 (0.011)	#SAKNAS!	#SAKNAS!
flatfish	1	5033 (0.011)	2293 (0.034)	#SAKNAS!
flavescens	1	5033 (0.011)	#SAKNAS!	2492 (0.033)
flavonoid	1	5033 (0.011)	#SAKNAS!	2492 (0.033)
flavored	1	5033 (0.011)	#SAKNAS!	#SAKNAS!
flavour	1	5033 (0.011)	#SAKNAS!	2492 (0.033)
flesh	1	5033 (0.011)	#SAKNAS!	2492 (0.033)
flipbook	1	5033 (0.011)	#SAKNAS!	2492 (0.033)
floc	1	5033 (0.011)	2293 (0.034)	#SAKNAS!
flood	1	5033 (0.011)	#SAKNAS!	#SAKNAS!
floppy	1	5033 (0.011)	2293 (0.034)	#SAKNAS!
florence	1	5033 (0.011)	#SAKNAS!	2492 (0.033)
floss	1	5033 (0.011)	#SAKNAS!	2492 (0.033)
flowchart	1	5033 (0.011)	#SAKNAS!	#SAKNAS!
flower-visiting	1	5033 (0.011)	2293 (0.034)	#SAKNAS!
fluctuate	1	5033 (0.011)	2293 (0.034)	#SAKNAS!
fluctuating	1	5033 (0.011)	2293 (0.034)	#SAKNAS!
flume	1	5033 (0.011)	2293 (0.034)	#SAKNAS!
fluorescens	1	5033 (0.011)	2293 (0.034)	#SAKNAS!
fluorides	1	5033 (0.011)	#SAKNAS!	#SAKNAS!
fluoriferous	1	5033 (0.011)	2293 (0.034)	#SAKNAS!
fluoroscopic-guided	1	5033 (0.011)	#SAKNAS!	2492 (0.033)
fluorouracil	1	5033 (0.011)	#SAKNAS!	2492 (0.033)
flushes-a	1	5033 (0.011)	#SAKNAS!	#SAKNAS!
flying	1	5033 (0.011)	#SAKNAS!	#SAKNAS!
foals	1	5033 (0.011)	#SAKNAS!	2492 (0.033)
foam	1	5033 (0.011)	#SAKNAS!	#SAKNAS!
foams	1	5033 (0.011)	#SAKNAS!	#SAKNAS!
focusing	1	5033 (0.011)	2293 (0.034)	#SAKNAS!
fois	1	5033 (0.011)	#SAKNAS!	2492 (0.033)
fois-it	1	5033 (0.011)	#SAKNAS!	#SAKNAS!
foliar	1	5033 (0.011)	#SAKNAS!	#SAKNAS!
folk	1	5033 (0.011)	#SAKNAS!	2492 (0.033)
follows	1	5033 (0.011)	#SAKNAS!	2492 (0.033)
follow-ups	1	5033 (0.011)	#SAKNAS!	2492 (0.033)

food4toddlers	1	5033 (0.011)	#SAKNAS!	2492 (0.033)
food-carbohydrate	1	5033 (0.011)	2293 (0.034)	#SAKNAS!
food-industry	1	5033 (0.011)	2293 (0.034)	#SAKNAS!
food-intake	1	5033 (0.011)	2293 (0.034)	#SAKNAS!
food-restricted	1	5033 (0.011)	2293 (0.034)	#SAKNAS!
foodscape	1	5033 (0.011)	#SAKNAS!	2492 (0.033)
food-web	1	5033 (0.011)	2293 (0.034)	#SAKNAS!
footballers	1	5033 (0.011)	#SAKNAS!	#SAKNAS!
foothills	1	5033 (0.011)	#SAKNAS!	#SAKNAS!
footprint	1	5033 (0.011)	2293 (0.034)	#SAKNAS!
fora	1	5033 (0.011)	#SAKNAS!	2492 (0.033)
forage-based	1	5033 (0.011)	2293 (0.034)	#SAKNAS!
forager-farmers	1	5033 (0.011)	#SAKNAS!	#SAKNAS!
foramen	1	5033 (0.011)	#SAKNAS!	2492 (0.033)
foraminatus	1	5033 (0.011)	#SAKNAS!	#SAKNAS!
foraminifera	1	5033 (0.011)	#SAKNAS!	#SAKNAS!
forest-dwelling	1	5033 (0.011)	2293 (0.034)	#SAKNAS!
forestiers	1	5033 (0.011)	2293 (0.034)	#SAKNAS!
forest-savanna	1	5033 (0.011)	#SAKNAS!	#SAKNAS!
forget	1	5033 (0.011)	2293 (0.034)	#SAKNAS!
formal	1	5033 (0.011)	#SAKNAS!	#SAKNAS!
formats	1	5033 (0.011)	#SAKNAS!	2492 (0.033)
former	1	5033 (0.011)	#SAKNAS!	2492 (0.033)
formicidae	1	5033 (0.011)	#SAKNAS!	#SAKNAS!
formosan	1	5033 (0.011)	2293 (0.034)	#SAKNAS!
formulae	1	5033 (0.011)	#SAKNAS!	#SAKNAS!
formula-fed	1	5033 (0.011)	2293 (0.034)	#SAKNAS!
formula-feeding	1	5033 (0.011)	2293 (0.034)	#SAKNAS!
formulating	1	5033 (0.011)	#SAKNAS!	#SAKNAS!
forpus	1	5033 (0.011)	2293 (0.034)	#SAKNAS!
fortification	1	5033 (0.011)	#SAKNAS!	#SAKNAS!
fortifying	1	5033 (0.011)	2293 (0.034)	#SAKNAS!
forty-year	1	5033 (0.011)	#SAKNAS!	2492 (0.033)
forward	1	5033 (0.011)	#SAKNAS!	#SAKNAS!
fos-cells	1	5033 (0.011)	#SAKNAS!	2492 (0.033)
fossa	1	5033 (0.011)	#SAKNAS!	#SAKNAS!
foster	1	5033 (0.011)	#SAKNAS!	2492 (0.033)
foundation	1	5033 (0.011)	2293 (0.034)	#SAKNAS!
four-to-six	1	5033 (0.011)	#SAKNAS!	2492 (0.033)
fowls	1	5033 (0.011)	2293 (0.034)	#SAKNAS!
fq	1	5033 (0.011)	#SAKNAS!	#SAKNAS!
fractal	1	5033 (0.011)	#SAKNAS!	#SAKNAS!
fraction	1	5033 (0.011)	#SAKNAS!	2492 (0.033)

fractional	1	5033 (0.011)	2293 (0.034)	#SAKNAS!
fractions	1	5033 (0.011)	2293 (0.034)	#SAKNAS!
fragiless	1	5033 (0.011)	#SAKNAS!	#SAKNAS!
fragment	1	5033 (0.011)	2293 (0.034)	#SAKNAS!
fragmentation	1	5033 (0.011)	2293 (0.034)	#SAKNAS!
franc	1	5033 (0.011)	2293 (0.034)	#SAKNAS!
franciscan	1	5033 (0.011)	#SAKNAS!	2492 (0.033)
fratercula	1	5033 (0.011)	2293 (0.034)	#SAKNAS!
frax	1	5033 (0.011)	#SAKNAS!	2492 (0.033)
fraxinus	1	5033 (0.011)	2293 (0.034)	#SAKNAS!
free-end	1	5033 (0.011)	#SAKNAS!	2492 (0.033)
free-flap	1	5033 (0.011)	#SAKNAS!	#SAKNAS!
free-range	1	5033 (0.011)	#SAKNAS!	2492 (0.033)
freezing	1	5033 (0.011)	#SAKNAS!	#SAKNAS!
frequencies	1	5033 (0.011)	2293 (0.034)	#SAKNAS!
frequency-dependent	1	5033 (0.011)	2293 (0.034)	#SAKNAS!
frequently	1	5033 (0.011)	2293 (0.034)	#SAKNAS!
freshly	1	5033 (0.011)	#SAKNAS!	#SAKNAS!
freshman	1	5033 (0.011)	2293 (0.034)	#SAKNAS!
friderici	1	5033 (0.011)	2293 (0.034)	#SAKNAS!
frondosa	1	5033 (0.011)	#SAKNAS!	2492 (0.033)
frontier	1	5033 (0.011)	#SAKNAS!	2492 (0.033)
front-of-pack	1	5033 (0.011)	#SAKNAS!	#SAKNAS!
front-of-package	1	5033 (0.011)	#SAKNAS!	#SAKNAS!
frozen	1	5033 (0.011)	#SAKNAS!	#SAKNAS!
fructosyltransferase	1	5033 (0.011)	2293 (0.034)	#SAKNAS!
frugiperda	1	5033 (0.011)	#SAKNAS!	2492 (0.033)
frugivore	1	5033 (0.011)	#SAKNAS!	2492 (0.033)
frugivores	1	5033 (0.011)	#SAKNAS!	2492 (0.033)
frugivory	1	5033 (0.011)	2293 (0.034)	#SAKNAS!
fruiting	1	5033 (0.011)	2293 (0.034)	#SAKNAS!
frustrations	1	5033 (0.011)	#SAKNAS!	#SAKNAS!
fry	1	5033 (0.011)	2293 (0.034)	#SAKNAS!
fsmps	1	5033 (0.011)	#SAKNAS!	#SAKNAS!
fucata	1	5033 (0.011)	#SAKNAS!	2492 (0.033)
fuel	1	5033 (0.011)	2293 (0.034)	#SAKNAS!
fueling	1	5033 (0.011)	#SAKNAS!	#SAKNAS!
fuels	1	5033 (0.011)	#SAKNAS!	2492 (0.033)
fukui	1	5033 (0.011)	#SAKNAS!	#SAKNAS!
full-day	1	5033 (0.011)	#SAKNAS!	#SAKNAS!
full-thickness	1	5033 (0.011)	#SAKNAS!	2492 (0.033)
fumiferana	1	5033 (0.011)	2293 (0.034)	#SAKNAS!
function-driven	1	5033 (0.011)	#SAKNAS!	2492 (0.033)

fundamentals	1	5033 (0.011)	2293 (0.034)	#SAKNAS!
fundic	1	5033 (0.011)	#SAKNAS!	#SAKNAS!
fundus	1	5033 (0.011)	2293 (0.034)	#SAKNAS!
funfte	1	5033 (0.011)	#SAKNAS!	2492 (0.033)
funnels	1	5033 (0.011)	#SAKNAS!	2492 (0.033)
furanone	1	5033 (0.011)	#SAKNAS!	2492 (0.033)
furnarius	1	5033 (0.011)	2293 (0.034)	#SAKNAS!
fussy	1	5033 (0.011)	#SAKNAS!	2492 (0.033)
g1359a	1	5033 (0.011)	#SAKNAS!	2492 (0.033)
gaba	1	5033 (0.011)	#SAKNAS!	#SAKNAS!
gabab-induced	1	5033 (0.011)	2293 (0.034)	#SAKNAS!
gad2	1	5033 (0.011)	2293 (0.034)	#SAKNAS!
gadus	1	5033 (0.011)	#SAKNAS!	2492 (0.033)
gadus-morhua	1	5033 (0.011)	2293 (0.034)	#SAKNAS!
gahi	1	5033 (0.011)	#SAKNAS!	#SAKNAS!
gaining	1	5033 (0.011)	#SAKNAS!	2492 (0.033)
galactose	1	5033 (0.011)	#SAKNAS!	#SAKNAS!
galicia	1	5033 (0.011)	2293 (0.034)	#SAKNAS!
galician	1	5033 (0.011)	#SAKNAS!	2492 (0.033)
gallinarum	1	5033 (0.011)	2293 (0.034)	#SAKNAS!
galloprovincialis	1	5033 (0.011)	#SAKNAS!	2492 (0.033)
gallstones	1	5033 (0.011)	#SAKNAS!	#SAKNAS!
game	1	5033 (0.011)	#SAKNAS!	2492 (0.033)
gamma	1	5033 (0.011)	#SAKNAS!	2492 (0.033)
gammarus	1	5033 (0.011)	2293 (0.034)	#SAKNAS!
ganglia	1	5033 (0.011)	#SAKNAS!	#SAKNAS!
gannets	1	5033 (0.011)	#SAKNAS!	2492 (0.033)
ganzi	1	5033 (0.011)	#SAKNAS!	2492 (0.033)
gaoligong	1	5033 (0.011)	#SAKNAS!	2492 (0.033)
gap	1	5033 (0.011)	#SAKNAS!	#SAKNAS!
garden	1	5033 (0.011)	#SAKNAS!	2492 (0.033)
gardening	1	5033 (0.011)	#SAKNAS!	2492 (0.033)
garment	1	5033 (0.011)	2293 (0.034)	#SAKNAS!
garthi	1	5033 (0.011)	#SAKNAS!	2492 (0.033)
gasseri	1	5033 (0.011)	#SAKNAS!	#SAKNAS!
gasterosteus	1	5033 (0.011)	2293 (0.034)	#SAKNAS!
gastric-emptying	1	5033 (0.011)	2293 (0.034)	#SAKNAS!
gastric-tube	1	5033 (0.011)	2293 (0.034)	#SAKNAS!
gastritis	1	5033 (0.011)	#SAKNAS!	2492 (0.033)
gastroenteric	1	5033 (0.011)	#SAKNAS!	2492 (0.033)
gastroenterol	1	5033 (0.011)	2293 (0.034)	#SAKNAS!
gastroenterological	1	5033 (0.011)	2293 (0.034)	#SAKNAS!
gastroenterologist	1	5033 (0.011)	2293 (0.034)	#SAKNAS!

gastronomic	1 5033 (0.011)	#SAKNAS!	#SAKNAS!
gastronomy	1 5033 (0.011)	#SAKNAS!	#SAKNAS!
gastrophysics	1 5033 (0.011)	#SAKNAS!	2492 (0.033)
gastropod	1 5033 (0.011)	2293 (0.034)	#SAKNAS!
gastropoda	1 5033 (0.011)	2293 (0.034)	#SAKNAS!
gastrotomy	1 5033 (0.011)	#SAKNAS!	#SAKNAS!
gastrula	1 5033 (0.011)	#SAKNAS!	#SAKNAS!
gatherings	1 5033 (0.011)	#SAKNAS!	2492 (0.033)
gauge-raised	1 5033 (0.011)	#SAKNAS!	#SAKNAS!
gcn1	1 5033 (0.011)	2293 (0.034)	#SAKNAS!
gcn2	1 5033 (0.011)	2293 (0.034)	#SAKNAS!
gej	1 5033 (0.011)	#SAKNAS!	#SAKNAS!
gelatine	1 5033 (0.011)	2293 (0.034)	#SAKNAS!
gel-based	1 5033 (0.011)	#SAKNAS!	#SAKNAS!
gel-biopolymer	1 5033 (0.011)	#SAKNAS!	#SAKNAS!
gel-forming	1 5033 (0.011)	2293 (0.034)	#SAKNAS!
gelukspan	1 5033 (0.011)	2293 (0.034)	#SAKNAS!
gender-dependent	1 5033 (0.011)	2293 (0.034)	#SAKNAS!
genders	1 5033 (0.011)	#SAKNAS!	2492 (0.033)
gene-expression	1 5033 (0.011)	2293 (0.034)	#SAKNAS!
gene-lifestyle	1 5033 (0.011)	#SAKNAS!	2492 (0.033)
general-hospital	1 5033 (0.011)	2293 (0.034)	#SAKNAS!
generalis	1 5033 (0.011)	#SAKNAS!	2492 (0.033)
generalists	1 5033 (0.011)	2293 (0.034)	#SAKNAS!
generalization	1 5033 (0.011)	#SAKNAS!	2492 (0.033)
general-population	1 5033 (0.011)	#SAKNAS!	#SAKNAS!
generate	1 5033 (0.011)	2293 (0.034)	#SAKNAS!
genesis	1 5033 (0.011)	2293 (0.034)	#SAKNAS!
genioglossus	1 5033 (0.011)	2293 (0.034)	#SAKNAS!
geniohyoid	1 5033 (0.011)	#SAKNAS!	#SAKNAS!
genistein	1 5033 (0.011)	2293 (0.034)	#SAKNAS!
genome	1 5033 (0.011)	#SAKNAS!	2492 (0.033)
genomic	1 5033 (0.011)	#SAKNAS!	2492 (0.033)
genotype-by-sex-by-diet	1 5033 (0.011)	#SAKNAS!	2492 (0.033)
genotypic	1 5033 (0.011)	#SAKNAS!	2492 (0.033)
gentilis	1 5033 (0.011)	2293 (0.034)	#SAKNAS!
geochemistry	1 5033 (0.011)	2293 (0.034)	#SAKNAS!
geographical	1 5033 (0.011)	2293 (0.034)	#SAKNAS!
geometrical	1 5033 (0.011)	2293 (0.034)	#SAKNAS!
george	1 5033 (0.011)	2293 (0.034)	#SAKNAS!
georgia	1 5033 (0.011)	2293 (0.034)	#SAKNAS!
geranium	1 5033 (0.011)	#SAKNAS!	#SAKNAS!
geriatric-neurologic	1 5033 (0.011)	2293 (0.034)	#SAKNAS!

geriatric-patients	1	5033 (0.011)	2293 (0.034)	#SAKNAS!
geriatrics	1	5033 (0.011)	#SAKNAS!	2492 (0.033)
germanica	1	5033 (0.011)	2293 (0.034)	#SAKNAS!
german-speaking	1	5033 (0.011)	#SAKNAS!	#SAKNAS!
germany-the	1	5033 (0.011)	#SAKNAS!	#SAKNAS!
germinated-brown-rice-based	1	5033 (0.011)	#SAKNAS!	#SAKNAS!
gestations	1	5033 (0.011)	#SAKNAS!	#SAKNAS!
getting	1	5033 (0.011)	#SAKNAS!	2492 (0.033)
g-formula	1	5033 (0.011)	#SAKNAS!	2492 (0.033)
gg	1	5033 (0.011)	#SAKNAS!	2492 (0.033)
ghrelin-induced	1	5033 (0.011)	2293 (0.034)	#SAKNAS!
gibbon	1	5033 (0.011)	#SAKNAS!	2492 (0.033)
gibel	1	5033 (0.011)	#SAKNAS!	2492 (0.033)
gibelio	1	5033 (0.011)	#SAKNAS!	2492 (0.033)
gibelion	1	5033 (0.011)	#SAKNAS!	2492 (0.033)
gilts	1	5033 (0.011)	2293 (0.034)	#SAKNAS!
gingipains	1	5033 (0.011)	#SAKNAS!	#SAKNAS!
giniplus	1	5033 (0.011)	2293 (0.034)	#SAKNAS!
ginseng	1	5033 (0.011)	#SAKNAS!	#SAKNAS!
giraffa	1	5033 (0.011)	#SAKNAS!	2492 (0.033)
giraffa-camelopardalis	1	5033 (0.011)	2293 (0.034)	#SAKNAS!
girl	1	5033 (0.011)	#SAKNAS!	2492 (0.033)
girolando	1	5033 (0.011)	#SAKNAS!	#SAKNAS!
give	1	5033 (0.011)	#SAKNAS!	2492 (0.033)
givers	1	5033 (0.011)	2293 (0.034)	#SAKNAS!
giving	1	5033 (0.011)	#SAKNAS!	2492 (0.033)
gla	1	5033 (0.011)	2293 (0.034)	#SAKNAS!
glacier	1	5033 (0.011)	#SAKNAS!	#SAKNAS!
gladii	1	5033 (0.011)	#SAKNAS!	#SAKNAS!
gladius	1	5033 (0.011)	#SAKNAS!	#SAKNAS!
glial	1	5033 (0.011)	#SAKNAS!	#SAKNAS!
glide	1	5033 (0.011)	#SAKNAS!	2492 (0.033)
glider	1	5033 (0.011)	#SAKNAS!	2492 (0.033)
gliders	1	5033 (0.011)	#SAKNAS!	2492 (0.033)
gliptins	1	5033 (0.011)	#SAKNAS!	2492 (0.033)
globus	1	5033 (0.011)	#SAKNAS!	2492 (0.033)
glomerida	1	5033 (0.011)	#SAKNAS!	#SAKNAS!
glomeridae	1	5033 (0.011)	#SAKNAS!	#SAKNAS!
glomeris	1	5033 (0.011)	#SAKNAS!	#SAKNAS!
glottic	1	5033 (0.011)	2293 (0.034)	#SAKNAS!
glp1	1	5033 (0.011)	#SAKNAS!	#SAKNAS!
glp-1	1	5033 (0.011)	#SAKNAS!	#SAKNAS!
glucocorticoids	1	5033 (0.011)	#SAKNAS!	#SAKNAS!

glucomannan-mediated	1	5033 (0.011)	#SAKNAS!	#SAKNAS!
glucomannans	1	5033 (0.011)	#SAKNAS!	2492 (0.033)
glucosides	1	5033 (0.011)	2293 (0.034)	#SAKNAS!
glucosinolate	1	5033 (0.011)	2293 (0.034)	#SAKNAS!
glue	1	5033 (0.011)	2293 (0.034)	#SAKNAS!
glut2	1	5033 (0.011)	#SAKNAS!	#SAKNAS!
glutamine	1	5033 (0.011)	#SAKNAS!	2492 (0.033)
glutaric	1	5033 (0.011)	2293 (0.034)	#SAKNAS!
gluten	1	5033 (0.011)	#SAKNAS!	2492 (0.033)
gluten-free	1	5033 (0.011)	#SAKNAS!	#SAKNAS!
glycemia	1	5033 (0.011)	#SAKNAS!	#SAKNAS!
glycemia-sensitive	1	5033 (0.011)	2293 (0.034)	#SAKNAS!
glycogen	1	5033 (0.011)	#SAKNAS!	2492 (0.033)
glycohemoglobin	1	5033 (0.011)	2293 (0.034)	#SAKNAS!
glycolytic	1	5033 (0.011)	#SAKNAS!	2492 (0.033)
gmelin	1	5033 (0.011)	2293 (0.034)	#SAKNAS!
gnotobiotic	1	5033 (0.011)	#SAKNAS!	#SAKNAS!
gnri	1	5033 (0.011)	#SAKNAS!	#SAKNAS!
gntr	1	5033 (0.011)	#SAKNAS!	#SAKNAS!
goal-directed	1	5033 (0.011)	#SAKNAS!	2492 (0.033)
goats*	1	693 (0.18)	402 (0.28)	1180 (0.1)
goblet	1	5033 (0.011)	#SAKNAS!	#SAKNAS!
gohai	1	5033 (0.011)	#SAKNAS!	#SAKNAS!
goiter	1	5033 (0.011)	#SAKNAS!	#SAKNAS!
goiters	1	5033 (0.011)	2293 (0.034)	#SAKNAS!
gojjam	1	5033 (0.011)	#SAKNAS!	2492 (0.033)
gokceada	1	5033 (0.011)	#SAKNAS!	#SAKNAS!
golden	1	5033 (0.011)	#SAKNAS!	2492 (0.033)
golden-headed	1	5033 (0.011)	#SAKNAS!	#SAKNAS!
gompertz-makeham	1	5033 (0.011)	2293 (0.034)	#SAKNAS!
gorge	1	5033 (0.011)	2293 (0.034)	#SAKNAS!
goteborg	1	5033 (0.011)	2293 (0.034)	#SAKNAS!
governmental	1	5033 (0.011)	#SAKNAS!	2492 (0.033)
gower	1	5033 (0.011)	#SAKNAS!	#SAKNAS!
gpr120	1	5033 (0.011)	#SAKNAS!	2492 (0.033)
gps	1	5033 (0.011)	2293 (0.034)	#SAKNAS!
gradients	1	5033 (0.011)	#SAKNAS!	2492 (0.033)
grafts	1	5033 (0.011)	2293 (0.034)	#SAKNAS!
grain-pellet	1	5033 (0.011)	2293 (0.034)	#SAKNAS!
gramine	1	5033 (0.011)	2293 (0.034)	#SAKNAS!
graminivorous	1	5033 (0.011)	2293 (0.034)	#SAKNAS!
gran	1	5033 (0.011)	2293 (0.034)	#SAKNAS!
granarius	1	5033 (0.011)	#SAKNAS!	#SAKNAS!

grande	1	5033 (0.011)	2293 (0.034)	#SAKNAS!
grandmothers	1	5033 (0.011)	#SAKNAS!	2492 (0.033)
grandparents	1	5033 (0.011)	#SAKNAS!	2492 (0.033)
granivorous	1	5033 (0.011)	#SAKNAS!	2492 (0.033)
granular	1	5033 (0.011)	2293 (0.034)	#SAKNAS!
granulicatella	1	5033 (0.011)	#SAKNAS!	2492 (0.033)
granuloma	1	5033 (0.011)	#SAKNAS!	2492 (0.033)
granulomatosis	1	5033 (0.011)	2293 (0.034)	#SAKNAS!
grape	1	5033 (0.011)	2293 (0.034)	#SAKNAS!
grapes	1	5033 (0.011)	#SAKNAS!	#SAKNAS!
grapevine	1	5033 (0.011)	2293 (0.034)	#SAKNAS!
graphical	1	5033 (0.011)	#SAKNAS!	#SAKNAS!
grassland	1	5033 (0.011)	2293 (0.034)	#SAKNAS!
grasslands	1	5033 (0.011)	2293 (0.034)	#SAKNAS!
grass-silage	1	5033 (0.011)	2293 (0.034)	#SAKNAS!
gravo	1	5033 (0.011)	#SAKNAS!	#SAKNAS!
grayling	1	5033 (0.011)	#SAKNAS!	2492 (0.033)
graz	1	5033 (0.011)	2293 (0.034)	#SAKNAS!
grazed	1	5033 (0.011)	#SAKNAS!	2492 (0.033)
grazoprevir	1	5033 (0.011)	#SAKNAS!	2492 (0.033)
greece	1	5033 (0.011)	#SAKNAS!	#SAKNAS!
greenhouse	1	5033 (0.011)	#SAKNAS!	2492 (0.033)
greenlip	1	5033 (0.011)	#SAKNAS!	2492 (0.033)
green-lipped	1	5033 (0.011)	2293 (0.034)	#SAKNAS!
green-morph	1	5033 (0.011)	#SAKNAS!	#SAKNAS!
gregarious	1	5033 (0.011)	2293 (0.034)	#SAKNAS!
grey	1	5033 (0.011)	2293 (0.034)	#SAKNAS!
grizzled	1	5033 (0.011)	#SAKNAS!	#SAKNAS!
grooming	1	5033 (0.011)	2293 (0.034)	#SAKNAS!
groundwaters	1	5033 (0.011)	2293 (0.034)	#SAKNAS!
grouse	1	5033 (0.011)	2293 (0.034)	#SAKNAS!
grow	1	5033 (0.011)	#SAKNAS!	#SAKNAS!
grow2gether	1	5033 (0.011)	#SAKNAS!	2492 (0.033)
grower-finisher	1	5033 (0.011)	#SAKNAS!	#SAKNAS!
growers	1	5033 (0.011)	2293 (0.034)	#SAKNAS!
growing-finishing	1	5033 (0.011)	#SAKNAS!	2492 (0.033)
growth-development	1	5033 (0.011)	#SAKNAS!	#SAKNAS!
growth-restricted	1	5033 (0.011)	#SAKNAS!	2492 (0.033)
guam	1	5033 (0.011)	#SAKNAS!	#SAKNAS!
guanacos	1	5033 (0.011)	2293 (0.034)	#SAKNAS!
guangdong	1	5033 (0.011)	#SAKNAS!	2492 (0.033)
guanicoe	1	5033 (0.011)	2293 (0.034)	#SAKNAS!
guanidine	1	5033 (0.011)	#SAKNAS!	#SAKNAS!

guaraquecaba	1	5033 (0.011)	2293 (0.034)	#SAKNAS!
guardians	1	5033 (0.011)	#SAKNAS!	#SAKNAS!
guarding	1	5033 (0.011)	#SAKNAS!	#SAKNAS!
guideline-based	1	5033 (0.011)	#SAKNAS!	#SAKNAS!
guides	1	5033 (0.011)	#SAKNAS!	2492 (0.033)
guilan	1	5033 (0.011)	#SAKNAS!	#SAKNAS!
guillemot	1	5033 (0.011)	2293 (0.034)	#SAKNAS!
guinea-pig	1	5033 (0.011)	2293 (0.034)	#SAKNAS!
gum-biopolymers	1	5033 (0.011)	#SAKNAS!	#SAKNAS!
gum-modified	1	5033 (0.011)	#SAKNAS!	#SAKNAS!
gunshot	1	5033 (0.011)	#SAKNAS!	#SAKNAS!
gustation	1	5033 (0.011)	#SAKNAS!	2492 (0.033)
gut-derived	1	5033 (0.011)	#SAKNAS!	2492 (0.033)
guts	1	5033 (0.011)	#SAKNAS!	#SAKNAS!
gvhd	1	5033 (0.011)	#SAKNAS!	2492 (0.033)
gymnodiniales	1	5033 (0.011)	#SAKNAS!	2492 (0.033)
gynecologic	1	5033 (0.011)	#SAKNAS!	2492 (0.033)
gynes	1	5033 (0.011)	#SAKNAS!	#SAKNAS!
gypsy-moth	1	5033 (0.011)	2293 (0.034)	#SAKNAS!
gyrus	1	5033 (0.011)	2293 (0.034)	#SAKNAS!
h2s	1	5033 (0.011)	#SAKNAS!	#SAKNAS!
h70	1	5033 (0.011)	2293 (0.034)	#SAKNAS!
ha	1	5033 (0.011)	2293 (0.034)	#SAKNAS!
h-abc	1	5033 (0.011)	#SAKNAS!	#SAKNAS!
haematopus	1	5033 (0.011)	2293 (0.034)	#SAKNAS!
haemolymph	1	5033 (0.011)	2293 (0.034)	#SAKNAS!
haemonchus	1	5033 (0.011)	#SAKNAS!	2492 (0.033)
haemophilia	1	5033 (0.011)	#SAKNAS!	2492 (0.033)
haemorrhage	1	5033 (0.011)	#SAKNAS!	2492 (0.033)
hague	1	5033 (0.011)	2293 (0.034)	#SAKNAS!
hake	1	5033 (0.011)	#SAKNAS!	2492 (0.033)
half-day	1	5033 (0.011)	#SAKNAS!	#SAKNAS!
half-solidification	1	5033 (0.011)	2293 (0.034)	#SAKNAS!
haliday	1	5033 (0.011)	2293 (0.034)	#SAKNAS!
halo	1	5033 (0.011)	#SAKNAS!	#SAKNAS!
halo-vest	1	5033 (0.011)	#SAKNAS!	2492 (0.033)
hamilton	1	5033 (0.011)	2293 (0.034)	#SAKNAS!
hamsters	1	5033 (0.011)	#SAKNAS!	2492 (0.033)
handfeeding	1	5033 (0.011)	#SAKNAS!	2492 (0.033)
handle	1	5033 (0.011)	2293 (0.034)	#SAKNAS!
handling	1	5033 (0.011)	#SAKNAS!	#SAKNAS!
hands	1	5033 (0.011)	#SAKNAS!	#SAKNAS!
hannai	1	5033 (0.011)	2293 (0.034)	#SAKNAS!

hap1	1	5033 (0.011)	2293 (0.034)	#SAKNAS!
haplochromis	1	5033 (0.011)	2293 (0.034)	#SAKNAS!
happiness	1	5033 (0.011)	#SAKNAS!	#SAKNAS!
haptophyceae	1	5033 (0.011)	2293 (0.034)	#SAKNAS!
harassment	1	5033 (0.011)	#SAKNAS!	2492 (0.033)
harboring	1	5033 (0.011)	#SAKNAS!	2492 (0.033)
hardship	1	5033 (0.011)	#SAKNAS!	#SAKNAS!
hargeisa	1	5033 (0.011)	#SAKNAS!	2492 (0.033)
harlequin	1	5033 (0.011)	2293 (0.034)	#SAKNAS!
harm	1	5033 (0.011)	#SAKNAS!	#SAKNAS!
harsh	1	5033 (0.011)	2293 (0.034)	#SAKNAS!
harvest	1	5033 (0.011)	2293 (0.034)	#SAKNAS!
harvester	1	5033 (0.011)	#SAKNAS!	#SAKNAS!
hasankeyf	1	5033 (0.011)	#SAKNAS!	#SAKNAS!
hashimoto	1	5033 (0.011)	#SAKNAS!	2492 (0.033)
hashish	1	5033 (0.011)	#SAKNAS!	2492 (0.033)
hausa	1	5033 (0.011)	#SAKNAS!	#SAKNAS!
hawkmoth	1	5033 (0.011)	2293 (0.034)	#SAKNAS!
hawksbill	1	5033 (0.011)	2293 (0.034)	#SAKNAS!
hays	1	5033 (0.011)	#SAKNAS!	2492 (0.033)
hay-to-grain	1	5033 (0.011)	#SAKNAS!	2492 (0.033)
hchs	1	5033 (0.011)	#SAKNAS!	2492 (0.033)
hdl	1	5033 (0.011)	#SAKNAS!	2492 (0.033)
hdl-c	1	5033 (0.011)	#SAKNAS!	#SAKNAS!
hdl-cholesterol	1	5033 (0.011)	#SAKNAS!	#SAKNAS!
he	1	5033 (0.011)	#SAKNAS!	2492 (0.033)
he4	1	5033 (0.011)	#SAKNAS!	#SAKNAS!
headed	1	5033 (0.011)	#SAKNAS!	2492 (0.033)
health-enhancing	1	5033 (0.011)	2293 (0.034)	#SAKNAS!
health-problems	1	5033 (0.011)	2293 (0.034)	#SAKNAS!
healthy-eater	1	5033 (0.011)	#SAKNAS!	2492 (0.033)
heat-shock	1	5033 (0.011)	2293 (0.034)	#SAKNAS!
hedgehog	1	5033 (0.011)	#SAKNAS!	2492 (0.033)
heifer	1	5033 (0.011)	#SAKNAS!	#SAKNAS!
helgolandicus	1	5033 (0.011)	2293 (0.034)	#SAKNAS!
helicobacter?□ylori	1	5033 (0.011)	#SAKNAS!	#SAKNAS!
heliconius	1	5033 (0.011)	2293 (0.034)	#SAKNAS!
heliothis-zea	1	5033 (0.011)	2293 (0.034)	#SAKNAS!
helium-neon	1	5033 (0.011)	2293 (0.034)	#SAKNAS!
heller-dor	1	5033 (0.011)	2293 (0.034)	#SAKNAS!
help	1	5033 (0.011)	#SAKNAS!	2492 (0.033)
helping	1	5033 (0.011)	2293 (0.034)	#SAKNAS!
helps	1	5033 (0.011)	#SAKNAS!	#SAKNAS!

hemagglutinins	1	5033 (0.011)	#SAKNAS!	2492 (0.033)
hematological	1	5033 (0.011)	2293 (0.034)	#SAKNAS!
heme-peroxidase	1	5033 (0.011)	#SAKNAS!	2492 (0.033)
hemiglossectomy	1	5033 (0.011)	#SAKNAS!	2492 (0.033)
hemionus	1	5033 (0.011)	2293 (0.034)	#SAKNAS!
hemiparetic	1	5033 (0.011)	#SAKNAS!	2492 (0.033)
hemochromatosis	1	5033 (0.011)	#SAKNAS!	#SAKNAS!
hemocyte	1	5033 (0.011)	#SAKNAS!	2492 (0.033)
hemogram	1	5033 (0.011)	#SAKNAS!	#SAKNAS!
hemorrhagic	1	5033 (0.011)	2293 (0.034)	#SAKNAS!
hemp	1	5033 (0.011)	#SAKNAS!	2492 (0.033)
hepatocellular	1	5033 (0.011)	2293 (0.034)	#SAKNAS!
hepatocyte	1	5033 (0.011)	2293 (0.034)	#SAKNAS!
hepatol	1	5033 (0.011)	2293 (0.034)	#SAKNAS!
herat	1	5033 (0.011)	#SAKNAS!	2492 (0.033)
herb	1	5033 (0.011)	#SAKNAS!	2492 (0.033)
herbaceous	1	5033 (0.011)	2293 (0.034)	#SAKNAS!
herbage	1	5033 (0.011)	2293 (0.034)	#SAKNAS!
herbs	1	5033 (0.011)	#SAKNAS!	2492 (0.033)
herders	1	5033 (0.011)	#SAKNAS!	2492 (0.033)
herniae	1	5033 (0.011)	#SAKNAS!	2492 (0.033)
hernia-related	1	5033 (0.011)	#SAKNAS!	2492 (0.033)
herpesviridae	1	5033 (0.011)	2293 (0.034)	#SAKNAS!
herring	1	5033 (0.011)	#SAKNAS!	2492 (0.033)
herring-feeding	1	5033 (0.011)	#SAKNAS!	#SAKNAS!
herzegovina	1	5033 (0.011)	2293 (0.034)	#SAKNAS!
heterogeneous	1	5033 (0.011)	#SAKNAS!	#SAKNAS!
heterozygous	1	5033 (0.011)	2293 (0.034)	#SAKNAS!
hexacoral	1	5033 (0.011)	#SAKNAS!	#SAKNAS!
hexasticha	1	5033 (0.011)	#SAKNAS!	#SAKNAS!
hf	1	5033 (0.011)	#SAKNAS!	#SAKNAS!
hg	1	5033 (0.011)	#SAKNAS!	2492 (0.033)
hiatoplasty	1	5033 (0.011)	2293 (0.034)	#SAKNAS!
hiatus	1	5033 (0.011)	2293 (0.034)	#SAKNAS!
hidden	1	5033 (0.011)	#SAKNAS!	2492 (0.033)
hidradenitis	1	5033 (0.011)	#SAKNAS!	#SAKNAS!
hierarchical	1	5033 (0.011)	#SAKNAS!	#SAKNAS!
hierarchy	1	5033 (0.011)	2293 (0.034)	#SAKNAS!
high-caloric	1	5033 (0.011)	2293 (0.034)	#SAKNAS!
high-carotid	1	5033 (0.011)	#SAKNAS!	#SAKNAS!
high-concentrate	1	5033 (0.011)	#SAKNAS!	#SAKNAS!
high-dose	1	5033 (0.011)	2293 (0.034)	#SAKNAS!
higher-level	1	5033 (0.011)	#SAKNAS!	#SAKNAS!

high-fat-diet-induced	1 5033 (0.011)	#SAKNAS!	2492 (0.033)
high-frequency	1 5033 (0.011)	#SAKNAS!	#SAKNAS!
high-grade	1 5033 (0.011)	#SAKNAS!	2492 (0.033)
high-incidence	1 5033 (0.011)	#SAKNAS!	#SAKNAS!
high-income	1 5033 (0.011)	#SAKNAS!	#SAKNAS!
high-intensity	1 5033 (0.011)	2293 (0.034)	#SAKNAS!
highlight	1 5033 (0.011)	#SAKNAS!	2492 (0.033)
high-phase	1 5033 (0.011)	#SAKNAS!	#SAKNAS!
high-protein	1 5033 (0.011)	#SAKNAS!	#SAKNAS!
high-quality	1 5033 (0.011)	2293 (0.034)	#SAKNAS!
hindbrain	1 5033 (0.011)	#SAKNAS!	#SAKNAS!
hinders	1 5033 (0.011)	#SAKNAS!	2492 (0.033)
hindi	1 5033 (0.011)	#SAKNAS!	2492 (0.033)
hint-8	1 5033 (0.011)	#SAKNAS!	#SAKNAS!
hippocamelus	1 5033 (0.011)	2293 (0.034)	#SAKNAS!
hippocampus	1 5033 (0.011)	#SAKNAS!	2492 (0.033)
hippocampus-dependent	1 5033 (0.011)	#SAKNAS!	2492 (0.033)
hippodamia	1 5033 (0.011)	#SAKNAS!	2492 (0.033)
hippophae	1 5033 (0.011)	#SAKNAS!	#SAKNAS!
hippurus	1 5033 (0.011)	#SAKNAS!	2492 (0.033)
histamine	1 5033 (0.011)	2293 (0.034)	#SAKNAS!
histogram	1 5033 (0.011)	#SAKNAS!	#SAKNAS!
histomorphometry	1 5033 (0.011)	2293 (0.034)	#SAKNAS!
histopathological	1 5033 (0.011)	#SAKNAS!	#SAKNAS!
hitachi	1 5033 (0.011)	#SAKNAS!	#SAKNAS!
hive	1 5033 (0.011)	#SAKNAS!	#SAKNAS!
hiv-positive	1 5033 (0.011)	2293 (0.034)	#SAKNAS!
hmgcr	1 5033 (0.011)	#SAKNAS!	#SAKNAS!
hoarseness	1 5033 (0.011)	#SAKNAS!	#SAKNAS!
hodgkin	1 5033 (0.011)	2293 (0.034)	#SAKNAS!
holarctic	1 5033 (0.011)	2293 (0.034)	#SAKNAS!
hold	1 5033 (0.011)	#SAKNAS!	#SAKNAS!
holes	1 5033 (0.011)	#SAKNAS!	2492 (0.033)
holidays	1 5033 (0.011)	#SAKNAS!	#SAKNAS!
holistic	1 5033 (0.011)	#SAKNAS!	#SAKNAS!
holobenthic	1 5033 (0.011)	#SAKNAS!	#SAKNAS!
holstein-zebu	1 5033 (0.011)	#SAKNAS!	2492 (0.033)
homa	1 5033 (0.011)	#SAKNAS!	#SAKNAS!
homecare	1 5033 (0.011)	#SAKNAS!	2492 (0.033)
home-delivered	1 5033 (0.011)	#SAKNAS!	#SAKNAS!
home-dwelling	1 5033 (0.011)	#SAKNAS!	2492 (0.033)
homeless	1 5033 (0.011)	2293 (0.034)	#SAKNAS!
home-prepared	1 5033 (0.011)	#SAKNAS!	2492 (0.033)

homes-incidence	1	5033 (0.011)	#SAKNAS!	2492 (0.033)
homes-results	1	5033 (0.011)	#SAKNAS!	2492 (0.033)
homes-the	1	5033 (0.011)	#SAKNAS!	2492 (0.033)
home-visit	1	5033 (0.011)	#SAKNAS!	#SAKNAS!
homim	1	5033 (0.011)	#SAKNAS!	2492 (0.033)
hominids	1	5033 (0.011)	#SAKNAS!	2492 (0.033)
homo	1	5033 (0.011)	2293 (0.034)	#SAKNAS!
homocystein	1	5033 (0.011)	#SAKNAS!	#SAKNAS!
homo-exopolysaccharide	1	5033 (0.011)	#SAKNAS!	2492 (0.033)
homogeneity	1	5033 (0.011)	2293 (0.034)	#SAKNAS!
homogenization	1	5033 (0.011)	#SAKNAS!	#SAKNAS!
honeyed	1	5033 (0.011)	#SAKNAS!	#SAKNAS!
hood	1	5033 (0.011)	2293 (0.034)	#SAKNAS!
hook	1	5033 (0.011)	2293 (0.034)	#SAKNAS!
hope	1	5033 (0.011)	2293 (0.034)	#SAKNAS!
horizons	1	5033 (0.011)	#SAKNAS!	#SAKNAS!
horribilis	1	5033 (0.011)	#SAKNAS!	#SAKNAS!
horseshoe-crab	1	5033 (0.011)	2293 (0.034)	#SAKNAS!
hospital-acquired	1	5033 (0.011)	#SAKNAS!	#SAKNAS!
hospitalizations	1	5033 (0.011)	#SAKNAS!	2492 (0.033)
hospiton	1	5033 (0.011)	#SAKNAS!	2492 (0.033)
host-environment	1	5033 (0.011)	#SAKNAS!	#SAKNAS!
host-locate	1	5033 (0.011)	#SAKNAS!	2492 (0.033)
host-plant	1	5033 (0.011)	2293 (0.034)	#SAKNAS!
host-seeking	1	5033 (0.011)	2293 (0.034)	#SAKNAS!
house	1	5033 (0.011)	#SAKNAS!	2492 (0.033)
housebound	1	5033 (0.011)	2293 (0.034)	#SAKNAS!
hoyuk	1	5033 (0.011)	#SAKNAS!	#SAKNAS!
hpp	1	5033 (0.011)	#SAKNAS!	#SAKNAS!
hrept	1	5033 (0.011)	#SAKNAS!	2492 (0.033)
hscrp	1	5033 (0.011)	#SAKNAS!	#SAKNAS!
hs-crp	1	5033 (0.011)	2293 (0.034)	#SAKNAS!
htr1b	1	5033 (0.011)	2293 (0.034)	#SAKNAS!
hua	1	5033 (0.011)	#SAKNAS!	#SAKNAS!
hualien	1	5033 (0.011)	2293 (0.034)	#SAKNAS!
hudson	1	5033 (0.011)	#SAKNAS!	2492 (0.033)
huge	1	5033 (0.011)	#SAKNAS!	2492 (0.033)
huizhou	1	5033 (0.011)	#SAKNAS!	#SAKNAS!
human-influenced	1	5033 (0.011)	2293 (0.034)	#SAKNAS!
humanitarian	1	5033 (0.011)	#SAKNAS!	2492 (0.033)
human-modified	1	5033 (0.011)	#SAKNAS!	#SAKNAS!
human-natural	1	5033 (0.011)	#SAKNAS!	2492 (0.033)
human-occupied	1	5033 (0.011)	#SAKNAS!	2492 (0.033)

humpback	1	5033 (0.011)	2293 (0.034)	#SAKNAS!
hungary	1	5033 (0.011)	2293 (0.034)	#SAKNAS!
hunter-gatherer	1	5033 (0.011)	#SAKNAS!	#SAKNAS!
huntingtin-associated	1	5033 (0.011)	2293 (0.034)	#SAKNAS!
huso	1	5033 (0.011)	2293 (0.034)	#SAKNAS!
hyaluronate	1	5033 (0.011)	#SAKNAS!	#SAKNAS!
hybrids	1	5033 (0.011)	#SAKNAS!	#SAKNAS!
hydrobia	1	5033 (0.011)	2293 (0.034)	#SAKNAS!
hydrochloride	1	5033 (0.011)	2293 (0.034)	#SAKNAS!
hydrocorals	1	5033 (0.011)	#SAKNAS!	2492 (0.033)
hydrogel	1	5033 (0.011)	#SAKNAS!	2492 (0.033)
hydrogel-based	1	5033 (0.011)	#SAKNAS!	#SAKNAS!
hydrophilic	1	5033 (0.011)	#SAKNAS!	#SAKNAS!
hydrophobic	1	5033 (0.011)	#SAKNAS!	#SAKNAS!
hydroxide	1	5033 (0.011)	#SAKNAS!	2492 (0.033)
hydroxyapatite	1	5033 (0.011)	2293 (0.034)	#SAKNAS!
hydroxycitrate	1	5033 (0.011)	2293 (0.034)	#SAKNAS!
hygienic-dietary	1	5033 (0.011)	#SAKNAS!	#SAKNAS!
hygienist	1	5033 (0.011)	#SAKNAS!	#SAKNAS!
hylastes	1	5033 (0.011)	2293 (0.034)	#SAKNAS!
hymenolepis-diminuta	1	5033 (0.011)	2293 (0.034)	#SAKNAS!
hymenopteran	1	5033 (0.011)	2293 (0.034)	#SAKNAS!
hyoid-complex	1	5033 (0.011)	#SAKNAS!	2492 (0.033)
hyoid-larynx	1	5033 (0.011)	#SAKNAS!	2492 (0.033)
hyolaryngeal	1	5033 (0.011)	#SAKNAS!	2492 (0.033)
hyperandrogenism	1	5033 (0.011)	2293 (0.034)	#SAKNAS!
hypercholesterolemia	1	5033 (0.011)	2293 (0.034)	#SAKNAS!
hypercholesterolemic	1	5033 (0.011)	#SAKNAS!	2492 (0.033)
hyperhomocysteinemia	1	5033 (0.011)	#SAKNAS!	#SAKNAS!
hypericum	1	5033 (0.011)	#SAKNAS!	2492 (0.033)
hyperintensity	1	5033 (0.011)	#SAKNAS!	#SAKNAS!
hyperleptinemia	1	5033 (0.011)	2293 (0.034)	#SAKNAS!
hyperlipemia	1	5033 (0.011)	2293 (0.034)	#SAKNAS!
hyperlipidemia	1	5033 (0.011)	#SAKNAS!	2492 (0.033)
hypermobility	1	5033 (0.011)	#SAKNAS!	2492 (0.033)
hypernatremia	1	5033 (0.011)	#SAKNAS!	2492 (0.033)
hyperostosis	1	5033 (0.011)	2293 (0.034)	#SAKNAS!
hyperparasitoid	1	5033 (0.011)	2293 (0.034)	#SAKNAS!
hyperprolactinemia	1	5033 (0.011)	#SAKNAS!	2492 (0.033)
hypersensitivity	1	5033 (0.011)	#SAKNAS!	2492 (0.033)
hypertense	1	5033 (0.011)	#SAKNAS!	#SAKNAS!
hypertension-associated	1	5033 (0.011)	#SAKNAS!	2492 (0.033)
hypertensives	1	5033 (0.011)	2293 (0.034)	#SAKNAS!

hypertrophy	1	5033 (0.011)	#SAKNAS!	#SAKNAS!
hypnodiet	1	5033 (0.011)	#SAKNAS!	#SAKNAS!
hypnosis	1	5033 (0.011)	#SAKNAS!	#SAKNAS!
hypoalbuminemia	1	5033 (0.011)	#SAKNAS!	2492 (0.033)
hypoallergenic	1	5033 (0.011)	#SAKNAS!	#SAKNAS!
hypocretin-1	1	5033 (0.011)	2293 (0.034)	#SAKNAS!
hypoglycemia	1	5033 (0.011)	#SAKNAS!	#SAKNAS!
hypoglycemic	1	5033 (0.011)	#SAKNAS!	#SAKNAS!
hypokalemia	1	5033 (0.011)	#SAKNAS!	#SAKNAS!
hypomineralisation	1	5033 (0.011)	#SAKNAS!	#SAKNAS!
hypomineralized	1	5033 (0.011)	#SAKNAS!	#SAKNAS!
hypomyelination	1	5033 (0.011)	#SAKNAS!	#SAKNAS!
hypophagia	1	5033 (0.011)	2293 (0.034)	#SAKNAS!
hypophosphatemic	1	5033 (0.011)	2293 (0.034)	#SAKNAS!
hypothalamus-pituitary	1	5033 (0.011)	#SAKNAS!	#SAKNAS!
hypotheses	1	5033 (0.011)	#SAKNAS!	2492 (0.033)
hypouricemic	1	5033 (0.011)	#SAKNAS!	#SAKNAS!
hysterophorus	1	5033 (0.011)	2293 (0.034)	#SAKNAS!
hystricognathi	1	5033 (0.011)	2293 (0.034)	#SAKNAS!
i-1079	1	5033 (0.011)	#SAKNAS!	#SAKNAS!
i-3856	1	5033 (0.011)	#SAKNAS!	2492 (0.033)
iana	1	5033 (0.011)	2293 (0.034)	#SAKNAS!
iasi	1	5033 (0.011)	#SAKNAS!	2492 (0.033)
ib	1	5033 (0.011)	#SAKNAS!	2492 (0.033)
ibadan	1	5033 (0.011)	2293 (0.034)	#SAKNAS!
ibex	1	5033 (0.011)	#SAKNAS!	2492 (0.033)
ice-cream	1	5033 (0.011)	2293 (0.034)	#SAKNAS!
icelandic	1	5033 (0.011)	2293 (0.034)	#SAKNAS!
iceman	1	5033 (0.011)	#SAKNAS!	#SAKNAS!
icm	1	5033 (0.011)	#SAKNAS!	2492 (0.033)
id	1	5033 (0.011)	#SAKNAS!	#SAKNAS!
idaho	1	5033 (0.011)	2293 (0.034)	#SAKNAS!
ideation	1	5033 (0.011)	#SAKNAS!	2492 (0.033)
idefics	1	5033 (0.011)	#SAKNAS!	2492 (0.033)
idella	1	5033 (0.011)	#SAKNAS!	2492 (0.033)
idf	1	5033 (0.011)	#SAKNAS!	2492 (0.033)
iga	1	5033 (0.011)	2293 (0.034)	#SAKNAS!
igf	1	5033 (0.011)	#SAKNAS!	2492 (0.033)
igf1	1	5033 (0.011)	2293 (0.034)	#SAKNAS!
igf-1	1	5033 (0.011)	2293 (0.034)	#SAKNAS!
igfbp-2	1	5033 (0.011)	2293 (0.034)	#SAKNAS!
igf-i	1	5033 (0.011)	2293 (0.034)	#SAKNAS!
igf-ii	1	5033 (0.011)	2293 (0.034)	#SAKNAS!

igr	1	5033 (0.011)	2293 (0.034)	#SAKNAS!
igy	1	5033 (0.011)	#SAKNAS!	2492 (0.033)
iii-iv	1	5033 (0.011)	2293 (0.034)	#SAKNAS!
iii-phase	1	5033 (0.011)	2293 (0.034)	#SAKNAS!
il-10	1	5033 (0.011)	#SAKNAS!	2492 (0.033)
il10-1082	1	5033 (0.011)	2293 (0.034)	#SAKNAS!
il6	1	5033 (0.011)	#SAKNAS!	2492 (0.033)
il-6-174	1	5033 (0.011)	2293 (0.034)	#SAKNAS!
il-8	1	5033 (0.011)	#SAKNAS!	2492 (0.033)
ileal	1	5033 (0.011)	#SAKNAS!	#SAKNAS!
illicit	1	5033 (0.011)	#SAKNAS!	#SAKNAS!
illuminate	1	5033 (0.011)	#SAKNAS!	2492 (0.033)
imam	1	5033 (0.011)	#SAKNAS!	2492 (0.033)
imbricata	1	5033 (0.011)	2293 (0.034)	#SAKNAS!
imidazole-containing	1	5033 (0.011)	2293 (0.034)	#SAKNAS!
immediately	1	5033 (0.011)	#SAKNAS!	2492 (0.033)
immigrants	1	5033 (0.011)	#SAKNAS!	2492 (0.033)
immobility	1	5033 (0.011)	#SAKNAS!	2492 (0.033)
immortalization	1	5033 (0.011)	2293 (0.034)	#SAKNAS!
immunization	1	5033 (0.011)	#SAKNAS!	2492 (0.033)
immuno	1	5033 (0.011)	#SAKNAS!	2492 (0.033)
immunoglobulins	1	5033 (0.011)	#SAKNAS!	#SAKNAS!
immunohistochemical	1	5033 (0.011)	2293 (0.034)	#SAKNAS!
immunoinflammatory	1	5033 (0.011)	#SAKNAS!	#SAKNAS!
immunologic	1	5033 (0.011)	#SAKNAS!	2492 (0.033)
immunometabolic	1	5033 (0.011)	#SAKNAS!	#SAKNAS!
immunopathogeneses	1	5033 (0.011)	2293 (0.034)	#SAKNAS!
immunotest	1	5033 (0.011)	#SAKNAS!	2492 (0.033)
immunotherapy-related	1	5033 (0.011)	#SAKNAS!	#SAKNAS!
impacto	1	5033 (0.011)	#SAKNAS!	#SAKNAS!
impairing	1	5033 (0.011)	#SAKNAS!	#SAKNAS!
impairment-mbsimp	1	5033 (0.011)	2293 (0.034)	#SAKNAS!
impenetrable	1	5033 (0.011)	2293 (0.034)	#SAKNAS!
implant-assisted	1	5033 (0.011)	2293 (0.034)	#SAKNAS!
implantation	1	5033 (0.011)	#SAKNAS!	2492 (0.033)
implantology	1	5033 (0.011)	#SAKNAS!	#SAKNAS!
implementing	1	5033 (0.011)	2293 (0.034)	#SAKNAS!
import	1	5033 (0.011)	#SAKNAS!	#SAKNAS!
impulse	1	5033 (0.011)	#SAKNAS!	2492 (0.033)
imrt-sib	1	5033 (0.011)	#SAKNAS!	2492 (0.033)
imu	1	5033 (0.011)	#SAKNAS!	#SAKNAS!
inability	1	5033 (0.011)	#SAKNAS!	#SAKNAS!
inaccurate	1	5033 (0.011)	#SAKNAS!	2492 (0.033)

inactivation	1	5033 (0.011)	2293 (0.034)	#SAKNAS!
inactive	1	5033 (0.011)	2293 (0.034)	#SAKNAS!
inactivity	1	5033 (0.011)	#SAKNAS!	#SAKNAS!
inadequacies	1	5033 (0.011)	#SAKNAS!	2492 (0.033)
inanopheles	1	5033 (0.011)	#SAKNAS!	2492 (0.033)
inattention	1	5033 (0.011)	2293 (0.034)	#SAKNAS!
inauris	1	5033 (0.011)	2293 (0.034)	#SAKNAS!
incerta	1	5033 (0.011)	#SAKNAS!	2492 (0.033)
incidences	1	5033 (0.011)	#SAKNAS!	2492 (0.033)
incidental	1	5033 (0.011)	#SAKNAS!	#SAKNAS!
incidentaloma	1	5033 (0.011)	#SAKNAS!	#SAKNAS!
incilius	1	5033 (0.011)	#SAKNAS!	#SAKNAS!
incision	1	5033 (0.011)	#SAKNAS!	2492 (0.033)
include	1	5033 (0.011)	#SAKNAS!	2492 (0.033)
includes	1	5033 (0.011)	2293 (0.034)	#SAKNAS!
income-disparities	1	5033 (0.011)	#SAKNAS!	2492 (0.033)
income-related	1	5033 (0.011)	#SAKNAS!	2492 (0.033)
incontinence	1	5033 (0.011)	#SAKNAS!	#SAKNAS!
incorporation	1	5033 (0.011)	2293 (0.034)	#SAKNAS!
incretin	1	5033 (0.011)	#SAKNAS!	#SAKNAS!
incubation	1	5033 (0.011)	2293 (0.034)	#SAKNAS!
incurable	1	5033 (0.011)	#SAKNAS!	2492 (0.033)
independent-living	1	5033 (0.011)	2293 (0.034)	#SAKNAS!
index-2005	1	5033 (0.011)	#SAKNAS!	2492 (0.033)
index-a	1	5033 (0.011)	#SAKNAS!	#SAKNAS!
index-for-age	1	5033 (0.011)	2293 (0.034)	#SAKNAS!
index-related	1	5033 (0.011)	#SAKNAS!	#SAKNAS!
indians	1	5033 (0.011)	2293 (0.034)	#SAKNAS!
indication	1	5033 (0.011)	#SAKNAS!	2492 (0.033)
indication-dependent	1	5033 (0.011)	#SAKNAS!	2492 (0.033)
indicus	1	5033 (0.011)	#SAKNAS!	#SAKNAS!
indies	1	5033 (0.011)	#SAKNAS!	2492 (0.033)
indispensable	1	5033 (0.011)	#SAKNAS!	2492 (0.033)
individuality	1	5033 (0.011)	#SAKNAS!	2492 (0.033)
indolent	1	5033 (0.011)	2293 (0.034)	#SAKNAS!
indomethacin	1	5033 (0.011)	#SAKNAS!	#SAKNAS!
indonesian	1	5033 (0.011)	2293 (0.034)	#SAKNAS!
indoor-fed	1	5033 (0.011)	#SAKNAS!	#SAKNAS!
indoors	1	5033 (0.011)	#SAKNAS!	#SAKNAS!
indo-pacific	1	5033 (0.011)	#SAKNAS!	#SAKNAS!
indri	1	5033 (0.011)	2293 (0.034)	#SAKNAS!
induce	1	5033 (0.011)	#SAKNAS!	#SAKNAS!
inducer	1	5033 (0.011)	#SAKNAS!	2492 (0.033)

inducing	1	5033 (0.011)	#SAKNAS!	2492 (0.033)
industrial	1	5033 (0.011)	#SAKNAS!	2492 (0.033)
industrial-populations	1	5033 (0.011)	2293 (0.034)	#SAKNAS!
in-dwelling	1	5033 (0.011)	#SAKNAS!	#SAKNAS!
inedible	1	5033 (0.011)	#SAKNAS!	2492 (0.033)
inequitable-a	1	5033 (0.011)	#SAKNAS!	#SAKNAS!
inequities	1	5033 (0.011)	#SAKNAS!	#SAKNAS!
inerme	1	5033 (0.011)	#SAKNAS!	2492 (0.033)
inertial	1	5033 (0.011)	#SAKNAS!	#SAKNAS!
ines	1	5033 (0.011)	2293 (0.034)	#SAKNAS!
inexpensive	1	5033 (0.011)	2293 (0.034)	#SAKNAS!
infantile	1	5033 (0.011)	2293 (0.034)	#SAKNAS!
infant-oriented	1	5033 (0.011)	2293 (0.034)	#SAKNAS!
infarct	1	5033 (0.011)	#SAKNAS!	#SAKNAS!
infective	1	5033 (0.011)	#SAKNAS!	2492 (0.033)
in-feed	1	5033 (0.011)	2293 (0.034)	#SAKNAS!
inference	1	5033 (0.011)	#SAKNAS!	#SAKNAS!
inferior	1	5033 (0.011)	#SAKNAS!	2492 (0.033)
inferring	1	5033 (0.011)	2293 (0.034)	#SAKNAS!
infertility	1	5033 (0.011)	#SAKNAS!	2492 (0.033)
infiltrants	1	5033 (0.011)	#SAKNAS!	#SAKNAS!
infiltration	1	5033 (0.011)	#SAKNAS!	2492 (0.033)
infirmary	1	5033 (0.011)	2293 (0.034)	#SAKNAS!
inflammaging	1	5033 (0.011)	#SAKNAS!	#SAKNAS!
inflammasome	1	5033 (0.011)	#SAKNAS!	#SAKNAS!
infliximab	1	5033 (0.011)	#SAKNAS!	2492 (0.033)
influencers	1	5033 (0.011)	#SAKNAS!	2492 (0.033)
influenza	1	5033 (0.011)	#SAKNAS!	#SAKNAS!
influx	1	5033 (0.011)	2293 (0.034)	#SAKNAS!
informatics	1	5033 (0.011)	#SAKNAS!	2492 (0.033)
information-motivation-behavioural	1	5033 (0.011)	#SAKNAS!	2492 (0.033)
informative	1	5033 (0.011)	2293 (0.034)	#SAKNAS!
infusional	1	5033 (0.011)	2293 (0.034)	#SAKNAS!
ingestion-related	1	5033 (0.011)	2293 (0.034)	#SAKNAS!
ingredient-based	1	5033 (0.011)	#SAKNAS!	2492 (0.033)
inhabitant	1	5033 (0.011)	#SAKNAS!	#SAKNAS!
inhabitants	1	5033 (0.011)	#SAKNAS!	2492 (0.033)
inhibitor-1	1	5033 (0.011)	2293 (0.034)	#SAKNAS!
inhibitors	1	5033 (0.011)	#SAKNAS!	#SAKNAS!
ini-0602	1	5033 (0.011)	#SAKNAS!	2492 (0.033)
initiate	1	5033 (0.011)	#SAKNAS!	2492 (0.033)
injecting	1	5033 (0.011)	2293 (0.034)	#SAKNAS!
inmates	1	5033 (0.011)	#SAKNAS!	#SAKNAS!

innate	1	5033 (0.011)	2293 (0.034)	#SAKNAS!
inner-city-dwelling	1	5033 (0.011)	2293 (0.034)	#SAKNAS!
innervation	1	5033 (0.011)	#SAKNAS!	2492 (0.033)
inoculation	1	5033 (0.011)	#SAKNAS!	#SAKNAS!
inoculum	1	5033 (0.011)	#SAKNAS!	2492 (0.033)
in-patients	1	5033 (0.011)	#SAKNAS!	2492 (0.033)
insect-dwelling	1	5033 (0.011)	#SAKNAS!	#SAKNAS!
insectivore	1	5033 (0.011)	#SAKNAS!	2492 (0.033)
insecure	1	5033 (0.011)	#SAKNAS!	#SAKNAS!
insemap	1	5033 (0.011)	#SAKNAS!	#SAKNAS!
insensitive	1	5033 (0.011)	#SAKNAS!	#SAKNAS!
insight	1	5033 (0.011)	#SAKNAS!	#SAKNAS!
insomnia	1	5033 (0.011)	#SAKNAS!	2492 (0.033)
inspirations	1	5033 (0.011)	#SAKNAS!	#SAKNAS!
instability	1	5033 (0.011)	#SAKNAS!	2492 (0.033)
instantaneous	1	5033 (0.011)	#SAKNAS!	#SAKNAS!
institutes	1	5033 (0.011)	#SAKNAS!	2492 (0.033)
institutions	1	5033 (0.011)	2293 (0.034)	#SAKNAS!
instituto	1	5033 (0.011)	#SAKNAS!	2492 (0.033)
in-store	1	5033 (0.011)	#SAKNAS!	2492 (0.033)
instream	1	5033 (0.011)	#SAKNAS!	#SAKNAS!
instreptococcus	1	5033 (0.011)	#SAKNAS!	2492 (0.033)
instruction	1	5033 (0.011)	#SAKNAS!	2492 (0.033)
instrumentation-a	1	5033 (0.011)	#SAKNAS!	2492 (0.033)
instruments	1	5033 (0.011)	#SAKNAS!	#SAKNAS!
insulation	1	5033 (0.011)	2293 (0.034)	#SAKNAS!
insulin-like	1	5033 (0.011)	#SAKNAS!	#SAKNAS!
integral	1	5033 (0.011)	#SAKNAS!	2492 (0.033)
integrate	1	5033 (0.011)	#SAKNAS!	#SAKNAS!
integrator	1	5033 (0.011)	2293 (0.034)	#SAKNAS!
intellectually	1	5033 (0.011)	#SAKNAS!	2492 (0.033)
intelligence	1	5033 (0.011)	#SAKNAS!	#SAKNAS!
interactivity	1	5033 (0.011)	#SAKNAS!	#SAKNAS!
inter-arch	1	5033 (0.011)	#SAKNAS!	2492 (0.033)
interbody	1	5033 (0.011)	#SAKNAS!	#SAKNAS!
intercollegiate	1	5033 (0.011)	#SAKNAS!	2492 (0.033)
interconnectedness	1	5033 (0.011)	#SAKNAS!	#SAKNAS!
inter-dental	1	5033 (0.011)	#SAKNAS!	2492 (0.033)
interdepartmental	1	5033 (0.011)	2293 (0.034)	#SAKNAS!
interdependency	1	5033 (0.011)	2293 (0.034)	#SAKNAS!
interface	1	5033 (0.011)	2293 (0.034)	#SAKNAS!
interface-feeding	1	5033 (0.011)	2293 (0.034)	#SAKNAS!
interfere	1	5033 (0.011)	#SAKNAS!	2492 (0.033)

interim	1	5033 (0.011)	2293 (0.034)	#SAKNAS!
inter-individual	1	5033 (0.011)	#SAKNAS!	2492 (0.033)
interleukin-1	1	5033 (0.011)	#SAKNAS!	2492 (0.033)
interleukin-10	1	5033 (0.011)	2293 (0.034)	#SAKNAS!
interleukin-17	1	5033 (0.011)	2293 (0.034)	#SAKNAS!
interleukin-1耂	1	5033 (0.011)	2293 (0.034)	#SAKNAS!
intermedia	1	5033 (0.011)	#SAKNAS!	2492 (0.033)
intermediate	1	5033 (0.011)	2293 (0.034)	#SAKNAS!
inter-patch	1	5033 (0.011)	2293 (0.034)	#SAKNAS!
interpopulation	1	5033 (0.011)	#SAKNAS!	#SAKNAS!
interpretive	1	5033 (0.011)	#SAKNAS!	2492 (0.033)
inter-professional	1	5033 (0.011)	#SAKNAS!	#SAKNAS!
interrai	1	5033 (0.011)	#SAKNAS!	2492 (0.033)
interrater	1	5033 (0.011)	#SAKNAS!	#SAKNAS!
inter-rater	1	5033 (0.011)	2293 (0.034)	#SAKNAS!
interrogate	1	5033 (0.011)	#SAKNAS!	#SAKNAS!
intersections	1	5033 (0.011)	#SAKNAS!	#SAKNAS!
interspecific	1	5033 (0.011)	2293 (0.034)	#SAKNAS!
intersucking	1	5033 (0.011)	2293 (0.034)	#SAKNAS!
intervene	1	5033 (0.011)	#SAKNAS!	2492 (0.033)
intestinal-transplant	1	5033 (0.011)	#SAKNAS!	2492 (0.033)
intestines	1	5033 (0.011)	2293 (0.034)	#SAKNAS!
intoxicated	1	5033 (0.011)	#SAKNAS!	#SAKNAS!
intoxication	1	5033 (0.011)	2293 (0.034)	#SAKNAS!
intra	1	5033 (0.011)	#SAKNAS!	#SAKNAS!
intracellular	1	5033 (0.011)	#SAKNAS!	#SAKNAS!
intragastric	1	5033 (0.011)	2293 (0.034)	#SAKNAS!
intranasal	1	5033 (0.011)	#SAKNAS!	2492 (0.033)
intraoperative	1	5033 (0.011)	#SAKNAS!	#SAKNAS!
intraperitoneal	1	5033 (0.011)	#SAKNAS!	2492 (0.033)
intra-plant	1	5033 (0.011)	2293 (0.034)	#SAKNAS!
intraruminal	1	5033 (0.011)	2293 (0.034)	#SAKNAS!
intra-sexual	1	5033 (0.011)	#SAKNAS!	2492 (0.033)
intraspecies	1	5033 (0.011)	2293 (0.034)	#SAKNAS!
intraspecific	1	5033 (0.011)	2293 (0.034)	#SAKNAS!
intrathecal	1	5033 (0.011)	2293 (0.034)	#SAKNAS!
intrusive	1	5033 (0.011)	2293 (0.034)	#SAKNAS!
inuit	1	5033 (0.011)	2293 (0.034)	#SAKNAS!
inulin	1	5033 (0.011)	#SAKNAS!	#SAKNAS!
invagination	1	5033 (0.011)	#SAKNAS!	2492 (0.033)
inventory	1	5033 (0.011)	#SAKNAS!	#SAKNAS!
inversion	1	5033 (0.011)	#SAKNAS!	#SAKNAS!
invertebrates	1	5033 (0.011)	#SAKNAS!	#SAKNAS!

| | | | | |
[truncated: 274,118 more chars]
